# Geographical Variation in the Response of Visceral Leishmaniasis to Paromomycin in East Africa: A Multicentre, Open-Label, Randomized Trial

**DOI:** 10.1371/journal.pntd.0000709

**Published:** 2010-10-26

**Authors:** Asrat Hailu, Ahmed Musa, Monique Wasunna, Manica Balasegaram, Sisay Yifru, Getahun Mengistu, Zewdu Hurissa, Workagegnehu Hailu, Teklu Weldegebreal, Samson Tesfaye, Eyasu Makonnen, Eltahir Khalil, Osama Ahmed, Ahmed Fadlalla, Ahmed El-Hassan, Muzamil Raheem, Marius Mueller, Yousif Koummuki, Juma Rashid, Jane Mbui, Geoffrey Mucee, Simon Njoroge, Veronica Manduku, Alice Musibi, Geoffrey Mutuma, Fredrick Kirui, Hudson Lodenyo, Dedan Mutea, George Kirigi, Tansy Edwards, Peter Smith, Lawrence Muthami, Catherine Royce, Sally Ellis, Moses Alobo, Raymond Omollo, Josephine Kesusu, Rhoda Owiti, John Kinuthia, for the Leishmaniasis East Africa Platform (LEAP) group

**Affiliations:** 1 Addis Ababa University, Addis Ababa, Ethiopia; 2 Institute of Endemic Diseases, University of Khartoum, Khartoum, Sudan; 3 Centre for Clinical Research, Kenya Medical Research Institute, Nairobi, Kenya; 4 Medecins Sans Frontieres-Holland, Amsterdam, The Netherlands; 5 Drugs for Neglected Diseases initiative (DNDi), Geneva, Switzerland; 6 Gondar University, Gondar, Ethiopia; 7 Arba Minch Hospital, Regional Health Bureau of SNNP state, Arba Minch, Ethiopia; 8 Faculty of Medicine, Gedaref University, Gedaref, Sudan; 9 London School of Hygiene and Tropical Medicine, London, United Kingdom; London School of Hygiene and Tropical Medicine, United Kingdom

## Abstract

**Background:**

Visceral leishmaniasis (VL) is a major health problem in developing countries. The untreated disease is fatal, available treatment is expensive and often toxic, and drug resistance is increasing. Improved treatment options are needed. Paromomycin was shown to be an efficacious first-line treatment with low toxicity in India.

**Methods:**

This was a 3-arm multicentre, open-label, randomized, controlled clinical trial to compare three treatment regimens for VL in East Africa: paromomycin sulphate (PM) at 15 mg/kg/day for 21 days versus sodium stibogluconate (SSG) at 20 mg/kg/day for 30 days; and the combination of both dose regimens for 17 days. The primary efficacy endpoint was cure based on parasite-free tissue aspirates taken 6 months after treatment.

**Findings:**

Overall, 135 patients per arm were enrolled at five centres in Sudan (2 sites), Kenya (1) and Ethiopia (2), when the PM arm had to be discontinued due to poor efficacy. The trial has continued with the higher dose of PM as well as the combination of PM and SSG arms. These results will be reported later. Baseline patient characteristics were similar among treatment arms. The overall cure with PM was significantly inferior to that with SSG (63.8% versus 92.2%; difference 28.5%, 95%CI 18.8% to 38.8%, p<0.001). The efficacy of PM varied among centres and was significantly lower in Sudan (14.3% and 46.7%) than in Kenya (80.0%) and Ethiopia (75.0% and 96.6%). No major safety issues with PM were identified.

**Conclusion:**

The efficacy of PM at 15 mg/kg/day for 21 days was inadequate, particularly in Sudan. The efficacy of higher doses and the combination treatment warrant further studies.

## Author Summary

Visceral leishmaniasis (VL) is a fatal parasitic disease with 500,000 new cases each year according to WHO estimates. New and better treatment options are urgently needed in disease endemic areas due to the long courses, toxicity and development of resistance to current treatments. Recently, the antibiotic paromomycin was tested and registered in India to treat this disease. The current study describes a clinical trial to test the effectiveness of injectable paromomycin, either alone or in combination with the standard drug sodium stibogluconate in three East African countries—Sudan, Kenya and Ethiopia. The study showed that at the same paromomycin dose that was successfully used and registered in India, a far poorer outcome was obtained, particularly in Sudan, suggesting that there are either differences in the patients ability to respond to the drug or in the susceptibility of parasites in East Africa compared with those in India. However, no major safety concerns were noted with the treatment. Further research was initiated to see if a higher dose of paromomycin would perform better, especially in Sudan. The results of this and the performance of the combination arm will be reported later. Our study highlights the importance of considering geographical differences to treatment responses.

## Introduction

Visceral leishmaniasis (VL) is among the most neglected and serious tropical diseases [1], [2]. The disease is caused by protozoal parasites of the genus *Leishmania* transmitted to humans by bites of female sandflies. The incubation period varies from 2 to 6 months. The most prominent clinical symptoms are recurring fever, hepatosplenomegaly, lymphadenopathy, anemia, weight loss, and weakness. Not all those infected develop symptoms, but factors such as malnutrition and immune suppression including HIV coinfection increase the likelihood that the infection will manifest clinically. VL is usually fatal if not treated.

The WHO estimates that worldwide about 500,000 new cases of VL occur every year. 90% of cases occur in five countries: India (especially Bihar State), Bangladesh, Nepal, Sudan and north eastern Brazil [3]. In East Africa, the annual number of cases of VL is estimated at 30,000 [2] and related deaths at 4000 [4]. Especially in Sudan, Ethiopia and Kenya, VL is associated with high mortality and morbidity, exacerbated by poor nutritional status and the remote location of VL endemic areas.

For decades, antimony-based treatments, such as sodium stibogluconate (SSG), have been the mainstay of treatment, enshrined in the national treatment guidelines of Sudan, Ethiopia, and Kenya. However, the drug has unpredictable toxicity [5], [6] and the requisite 4-week hospitalization imposes a huge economic burden on poor families [7]. In addition, high treatment failure to SSG has been identified in some endemic areas with an incidence of up to 60% in some regions of Bihar, India [8]. However, no resistance to SSG has been documented in Africa. SSG has also been shown to be less effective and more toxic in HIV-infected patients [9]. Other drugs available for VL include paramomycin, miltefosine and AmBisome. However, none of these drugs is widely available or registered in Africa, and all have limitations due to cost, treatment duration, toxicity or heat stability. Therefore, new and better treatment options are urgently needed to replace or complement the few currently available drugs.

Paromomycin (PM), also known as aminosidine, is a broad-spectrum aminoglycoside antibiotic [10], of which an injectable sulphate formulation has been marketed for the treatment of bacterial and parasitic infections in several countries for over 35 years. In the 1990s, PM was shown to be efficacious against VL in pilot and phase II studies in India [11]–[13], Kenya [14] and Sudan [15], [16], both as monotherapy and in combination with SSG. In Sudan, use of PM has been in combination, so its efficacy in monotherapy had not been established before this trial. Recently, in a large randomized, controlled phase III trial enrolling 667 patients with acute symptomatic VL in India, definitive cure rates with PM were comparable to those with amphotericin B, and its safety profile was excellent [17]. Therefore, the aim of this trial was to assess the efficacy and safety of PM alone and in combination with SSG in comparison to the standard reference treatment for VL in East Africa. However, because the 15 mg/kg/day PM arm was prematurely discontinued due to poor efficacy, only the safety and efficacy findings of PM at this dose in comparison to SSG are given here. For the combination treatment, the study has continued at a higher dose of PM, 20 mg/kg/day, and results will be published as complete data become available.

## Methods

The protocol for this trial and supporting CONSORT checklist are available as supporting information; see Checklist S1 and Protocol S1.

### Study design and patients

This prospective, randomized, open-label, three-arm trial was carried out at five centres in three countries of East Africa: MSF Holland treatment centre, Um el Kher, Gedaref State, Sudan; Ministry of Health Hospital, Kassab, Gedaref State, Sudan; Leishmania Research and Treatment Centre, Gondar University Hospital, Amhara Regional State, northern Ethiopia; Leishmania Research and Treatment Centre, Arba Minch Hospital, Gama Gofa, Southern Nations, Nationalities and Peoples (SNNP) Regional State, southern Ethiopia; and Centre for Clinical Research (CCR), Kenya Medical Research Institute (KEMRI), Nairobi, Kenya.

Eligible patients were 4–60 years old and had to have clinical symptoms (fever and splenomegaly) and a diagnosis of VL, confirmed by microscopic verification of parasites in spleen, lymph node or bone marrow tissue aspirates. Patients were excluded if they: (1) had taken any antileishmanial drug in the preceding 6 months; (2) showed severe protein or caloric malnutrition (Kwashiokor or marasmus); (3) had previous hypersensitivity reaction to SSG or aminoglycosides; (4) had a concomitant severe infection (except HIV) or any other serious underlying (cardiac, renal, hepatic) disease; (5) had conditions associated with splenomegaly, such as schistosomiasis; (6) had a history of cardiac arrhythmia or an abnormal electrocardiogram (ECG); (7) were pregnant or lactating; (8) had any relevant outliers of safety laboratory parameters (hemoglobin <5 g/dL, white blood cells <1×10^3^/mm^3^, platelets <40,000/mm^3^, liver function test values >3 times higher than upper limit of normal, serum creatinine values above upper limit of normal); or (9) had clinical hearing loss.

Eligible patients were identified during regular field trips to villages in the endemic areas, or presented spontaneously at the hospital trial sites. At presentation, the trial and its purpose were described to patients in their local language. If patients agreed, they were transported to the trial sites for further investigation of eligibility. If eligible, patients were enrolled after written informed consent was signed either by themselves or, in case of minors, by their parent(s) or guardian. The study protocol was approved in each country by the relevant Institutional National Scientific Ethics Committees and the Ethics Committee of the London School of Hygiene and Tropical Medicine. The trial was registered with ClinicalTrials.gov (number NCT00255567).

### Ethics Statement

The trial was conducted in accordance with the Declaration of Helsinki (2002 version) relating to the conduct of research on human subjects and followed the International Committee on Harmonisation (ICH) guidelines for the conduct of clinical trials. All trial site personnel received relevant training in Good Clinical Practice (GCP). The WHO-TDR handbooks were used for training and for reference before and during the trial.

A Data Safety Monitoring Board (DSMB) was appointed at trial start and met regularly throughout the trial. Members were drawn from each of the participating countries. The trial was regularly monitored at all sites by GCP-trained monitors recruited from Sudan, Ethiopia, Kenya and Uganda. Monitors were allocated to trial sites not within their own country to ensure independence from investigators and to ease communications when dealing with issues arising at the trial sites. Wherever possible during monitoring visits, data entered into the trial case report forms (CRFs) were checked against source data (e.g. laboratory log books, analyzer printouts, nursing records) for verification.

### Randomization

Allocation to treatment was by means of sequentially numbered, sealed envelopes, generated from a computerized randomization list. Each centre received a box of uniquely numbered sealed envelopes from the LEAP Trial Coordination Centre in Nairobi, where centralized randomization and envelope preparation were carried out in blocks of 15 to maintain randomization balance within centers. For each enrolled patient, the investigator took the next lowest numbered envelope and allocated the patient to the treatment given on the card inside.

### Treatment

Patients received either PM alone (manufactured by Gland Pharma, India) at a daily dose of 15 mg/kg body weight given intramuscularly for 21 days, or SSG alone (manufactured by Albert David, India) at a daily dose of 20 mg/kg body weight given intramuscularly or intravenously (KEMRI trial site) for 30 days, or the combination of both at the same daily doses for 17 days. For the duration of treatment, patients were treated as inpatients, thus ensuring high compliance. Patients were followed up at 3 (optional) and 6 months after treatment. If patients did not attend follow-up, health workers visited the patient's village to try to establish if he/she was alive and well, had died or had moved away. Patients for whom trial medication had to be stopped due to lack of response or a serious adverse event (SAE) were given rescue medication (liposomal amphotericin B, manufactured as Ambisome® by Gilead, USA) according to national VL guidelines from participating countries.

### Baseline, efficacy and safety measurements

Baseline information was obtained from all patients through a standard clinical assessment. Patients were offered counseling and testing for HIV in accordance to national guidelines, though this was not a prerequisite to inclusion. Formal assessments were carried out at baseline, days 7, 14, 21, end of treatment, and 3 and 6 months after treatment. This included a clinical assessment (clinical symptoms, vital signs, weight, height, spleen and liver size), hemoglobin, white cell count (not performed in Um el Kher site), platelets, urea, creatinine, liver function tests (bilirubin, aspartate aminotransferase, alanine aminotransferase, alkaline phosphatase), urinalysis, ECG and audiometry (not performed in Um le Kher site). Amylase test was not routinely done in all sites but was performed by investigators if clinically indicated. ECG printouts with corrected QT intervals were interpreted by the site physician and were stored with source documents. Audiometry was performed using a standardized procedure by site investigators who were trained by a qualified audiometrist and recorded as hearing levels in dB at 0.25, 0.5, 1, 2, 4 and 8 kHz frequencies [18]. All reported abnormal audiometric readings were reviewed by the audiometrist. Abnormal audiometry was defined as any patient with audiometric readings in either of the following categories: mild 26–40 dBHL (decibel hearing level); moderate 41–55 dBHL; moderately severe 56–70 dBHL; severe 71–90 dBHL; or profound >90 dBHL. Clinically significant hearing loss or clinically abnormal hearing was defined as any patient who reports to have a hearing loss.

Parasitology was done at baseline, end of treatment and 6 months after treatment. A spleen, lymph node or bone marrow aspirate was performed at diagnosis and for assessment of cure. This varied between sites according to local guidelines. During follow-up, bone marrow aspirates were done in the event of spleen or lymph node regression.

### Outcome measures

The primary endpoint was cure (ie, efficacy) at 6 months after treatment, based on the absence of leishmania parasites in tissue aspirates. Any patient who died from VL, received rescue medication during the treatment period or had parasites visualized on microscopy at the 6-month assessment was regarded as a treatment failure in primary efficacy analyses.

The secondary endpoint was initial cure at the end of treatment (i.e. at day 22 for PM, at day 31 for SSG, and at day 18 for the combination). Any patient who died from VL, received rescue medication during the period of the trial or had parasites visualized on microscopy at the end of treatment was regarded as a treatment failure in the secondary efficacy analyses. Clinical and biological parameters such as temperature, weight, spleen size and hemoglobin were used by clinicians to decide if rescue medication was indicated.

A slow responder was defined as a patient who was parasite positive at end of treatment but was deemed not to require rescue medication due to improvements in clinical and biological parameters and who went on to clear parasites at the primary endpoint (6-month assessment). Therefore, a treatment failure at initial cure could be a treatment success at 6-month follow-up as a slow responder to treatment, provided that no rescue medication was administered.

The evaluation of safety was based on adverse event (AE) monitoring, ECGs, safety laboratory analyses (hematology and biochemistry) and audiometry. AEs were classified according to MedDRA, version 10, and defined as treatment emergent (TEAE) if onset was between the first day of treatment and 30 days after treatment.

### Statistical analysis

Data were analyzed with Stata software, version 9. At baseline, continuous data were summarized using mean and standard deviation (SD) and binary data using proportions. Primary and secondary efficacy analyses were by intention to treat (ITT). Where missing parasitological data at the primary endpoint (6-month follow-up) occurred due to loss to follow-up or death unrelated to VL in patients not receiving rescue medication before loss, two analysis approaches were taken within the ITT framework: complete-case analysis, where patients with missing data were excluded, and worst-case analysis, where the missing outcome was assumed to be treatment failure.

The number and percentage cured is presented by arm and centre. Fisher's exact test was used to assess evidence of significant differences in cure.

TEAE rate per arm was calculated as the number of events per arm divided by the person-time at risk. For the rate analysis, patient-time at risk was calculated as the number of patients per arm multiplied by the maximum patient-time at risk for TEAE (treatment period plus 30 days, i.e. 60 days for SSG and 51 days for PM). Poisson regression was used to obtain a rate ratio comparing PM to SSG, adjusted for centre.

At the start of the trial, it was estimated that 217 patients would be required per arm to detect a 10% difference in efficacy among HIV-uninfected patients at the 5% level, assuming 90% power, 95% efficacy in the reference (SSG) arm, and a 20% drop-out rate from the analysis (10% due to HIV co-infection and 10% due to loss to follow-up between end of treatment and 6-month assessment). HIV infection was not an exclusion criterion. Whilst co-infection was expected to be low, efficacy and toxicity may vary in those with and without HIV. A comparison of PM to SSG is presented here following recruitment of 135 patients per arm after which point recruitment into the 15 mg/kg/day PM arm was stopped.

## Results

**Figure 1:**
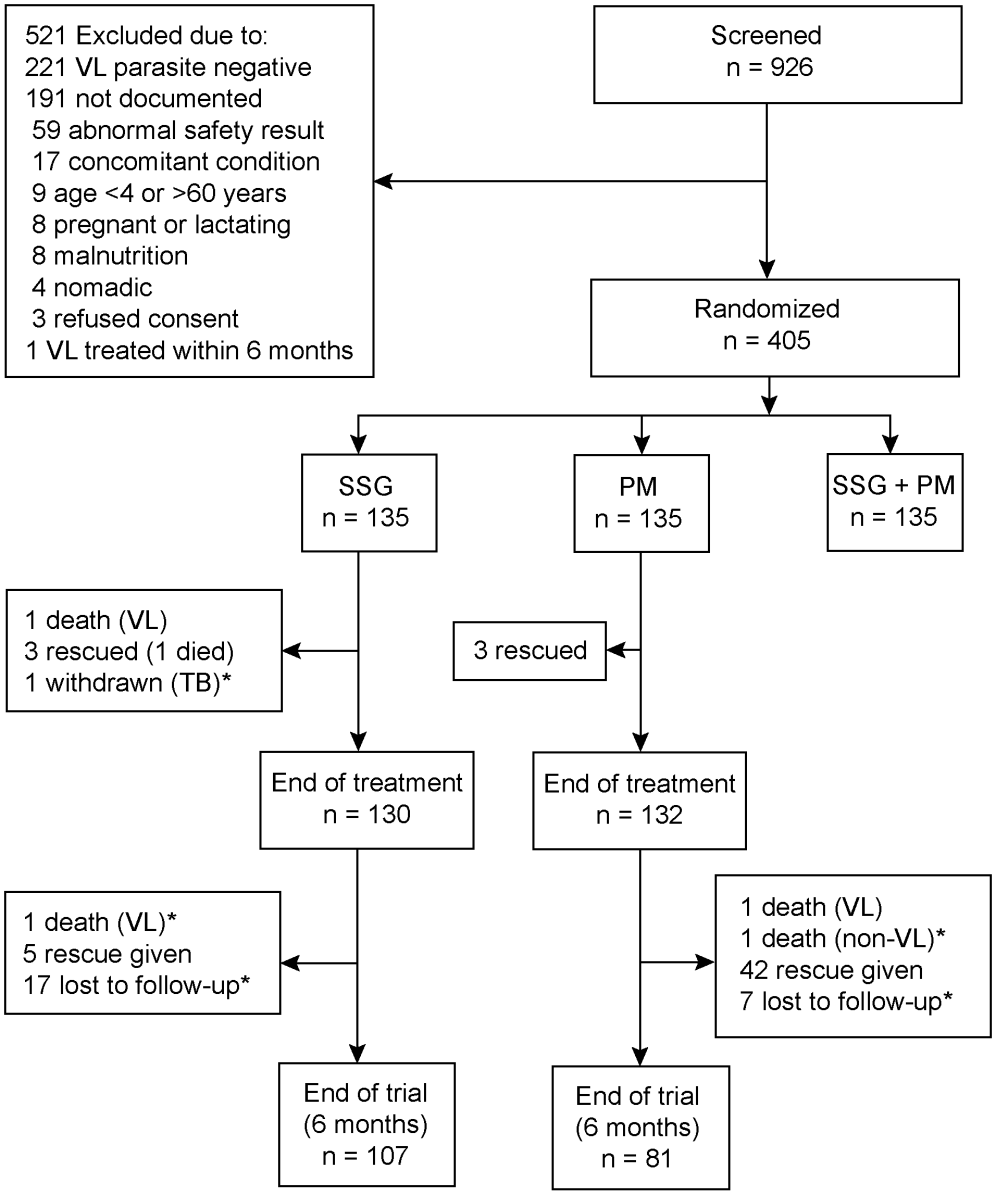
Patient flow chart. *Excluded from the efficacy analysis: 24 patients were lost to follow-up, and three died (n = 2 deaths unrelated to VL and n = 1 death related to VL). Patients who were given rescue medication or who had died from VL were considered treatment failures; i.e. efficacy complete-case analysis set for SSG, n = 116 and for PM, n = 127.

Of 926 patients screened for eligibility, 405 were randomly allocated to one of the three treatment arms (135 per arm). Of these, 135 patients were recruited in Gondar, 90 each in Arba Minch and Um el Kher and 45 each in Kassab and Kenya (Figure 1). The first patient was recruited in November 2004, and the last follow-up was done in April 2008.

### Baseline characteristics

Demographic and clinical characteristics were similar across treatment arms (Table 1). About 50% of patients were children, and the ratio of males to females in both the SSG and PM arms was about 3∶1. Common disease symptoms were fever, weight loss, fatigue, headache and loss of appetite, each affecting more than two thirds of patients across treatment arms (Table 1). Mean hemoglobin concentration was about 8.0 g/dL. Neither baseline laboratory parameters nor vital signs or parasite counts showed significant differences between treatment arms (data not shown). In all arms, most patients had significant splenomegaly and were malnourished, whereas the rate of HIV co-infection was low (3.7% overall) (Table 1). A third of all patients in each arm did not undergo HIV testing; however, of those tested, they were mainly concentrated in Gondar, northern Ethiopia: seven (7.8%) in Gondar, two (3.3%) in Arba Minch and one (1.1%) in Um el Kher.

**Table 1:** Baseline demographic and clinical characteristics.

		SSGn = 135	PMn = 135
Age [years]		16.7 (10.4)	17.8 (11.1)
	Children (4–14 years)	69 (51.1%)	67 (49.6%)
	Adults (≥15 years)	66 (48.9%)	68 (50.4%)
Sex	Female	34 (25.2%)	31 (23.0%)
	Male	101 (74.8%)	104 (77.0%)
Symptoms	Fever	133 (98.5%)	133 (98.5%)
	Weight loss	114 (84.4%)	111 (82.2%)
	Fatigue	109 (80.7%)	102 (75.6%)
	Headache	94 (69.6%)	98 (72.6%)
	Loss of appetite	93 (68.9%)	91 (67.4%)
	Cough	91 (67.4%)	83 (61.5%)
	Abdominal pain	89 (65.9%)	88 (65.2%)
	Night sweats	89 (65.9%)	85 (62.9%)
	Abdominal swelling	77 (57.0%)	84 (62.2%)
	Breathlessness	63 (46.7%)	59 (43.7%)
	Epistaxis	46 (34.1%)	51 (37.8%)
	Diarrhea	34 (25.2%)	31 (23.0%)
	Swelling of legs	27 (20.0%)	20 (14.8%)
Organ size [cm]	Spleen	8.2 (4.3)	8.3 (4.9)
	Liver	2.9 (2.4)	2.9 (2.3)
Nutritional status*	Severe underweight	17 (12.6%)	17 (12.6%)
	Underweight	61 (45.2%)	67 (49.7%)
	Normal weight	57 (42.2%)	50 (37.0%)
	Overweight	0 (0%)	0 (0%)
Co-infections	Malaria	7 (5.2%)	5 (3.7%)
	Pneumonia	6 (4.4%)	4 (3.0%)
	HIV-positive	4 (3.0%)	6 (4.4%)
	HIV-negative	87 (64.4%)	86 (63.7%)
	HIV-not tested	44 (32.6%)	43 (31.9%)

Data are n (%) or mean (SD). Differences between groups were not statistically significant (all p>0.1).

*Children: Weight for age defined as severely underweight if <60%, underweight if between 60% and 80%, normal if >80%. Adults: BMI defined as severely underweight if <16, underweight if between 16.0 and 18.4, normal if between 18.5 and 24.9.

### Efficacy

In the SSG arm (30-day treatment period), two patients received only 14 days of SSG treatment; in the PM arm (21-day treatment period), one patient received 20 days of PM treatment and 1 day of SSG on day 2. In efficacy analyses, these patients were assumed to have received allocated treatment in full, as per ITT.

In the SSG arm, 17 patients were lost between end of treatment and 6 months, and there was one death unrelated to VL during follow-up leading to a denominator of 116 in the complete-case analysis. In the PM arm, seven patients were lost, and there was one unrelated death leading to a complete-case denominator of 127.

At 6 months after treatment, the complete-case analysis shows that the primary cure with PM was significantly inferior to that with SSG standard treatment (63.8% versus 92.2%; difference 28.5%, 95%CI 18.8% to 38.8%, p<0.001). However, efficacy of PM varied substantially across sites (p<0.001; Table 2). It was poorest and significantly inferior to SSG at the Sudanese sites of Um el Kher and Kassab. In Gondar (Ethiopia) and Kenya, the efficacy of PM was between 75% and 80%, but there was less evidence of a difference to SSG in Gondar (p = 0.066) and none in Kenya (p = 0.224). In Arba Minch (Ethiopia), PM and SSG showed similar efficacy with cure around 95%. There was no variation in the efficacy of SSG across centres (p = 0.568).

**Table 2:** Cure (efficacy) at end of treatment and at 6 months after treatment.

	SSG	PM	*P* * †
**End of treatment**			
**Overall**	**123/134 (91.8%)**	**91/135 (67.4%)**	**<0.001**
• Um el Kher, Sudan	26/29 (89.7%)	10/30 (33.3%)	<0.001
• Kassab, Sudan	14/15 (93.3%)	9/15 (60.0%)	0.080
• Kenya	15/15 (100%)	13/15 (86.7%)	0.483
• Gondar, Ethiopia	40/45 (88.9%)	30/45 (66.7%)	0.021
• Arba Minch, Ethiopia	28/30 (93.3%)	29/30 (96.7%)	1.000
***P*** * ‡	0.832	<0.001	
**6-months after treatment**			
**Overall**	**107/116 (92.2%)**	**81/127 (63.8%)** 1	**<0.001**
• Um el Kher, Sudan	14/17 (82.4%)	4/28 (14.3%)	<0.001
• Kassab, Sudan	14/15 (93.3%)	7/15 (46.7%)	0.014
• Kenya	15/15 (100.0%)	12/15 (80.0%)	0.224
• Gondar, Ethiopia	37/40 (92.5%)	30/40 (75.0%)	0.066
• Arba Minch, Ethiopia	27/29 (93.1%)	28/29 (96.6%)	1.000
***P*** * ‡	0.568	<0.001	

1 One patient without parasitology done at 6 months was considered as treatment failure.

*p value from Fisher's exact test.

**†:** Comparison across arms within sites.

**‡:** Comparison across sites within arms.

A worst-case primary efficacy analysis also showed significantly lower efficacy of PM than of SSG (60.0% versus 79.3%; difference 19.3%; 95%CI 8.5% to 30.0%, p<0.001). Effects seen were less marked due to higher loss to follow-up in the SSG arm.

The findings for parasite clearance at the end of treatment (Table 2) and the proportion of slow responders (i.e. patients still having detectable parasites at the end of treatment, without any rescue medication given) were similar in both arms. Poorer efficacy of PM at the end of treatment was also reflected by a significantly lower regression (ANOVA p = 0.001, adjusting for baseline measurements and centre) of spleen size (mean decrease 3.92 cm, 95%CI 4.49 to 3.35) compared with that of patients treated with SSG (mean decrease 4.83 cm, 95%CI 5.38 to 4.28).

The subgroup of HIV-positive patients was small; however, in the SSG arm three out of four patients (75%) were cured at 6 months, whereas in the PM arm only one out of six (17%) was cured.

### Safety

Treatment with both SSG and PM resulted in an increase of liver enzymes, amylase and alkaline phosphatase, which abated spontaneously whilst on treatment (data not shown). On average, vital signs normalized during treatment, although slightly delayed in the PM arm. Less than 1% of patients receiving either SSG or PM developed ECG abnormalities and no clinically significant abnormal audiometry readings were detected by the final assessment.

Fewer patients in the PM arm than in the SSG arm were affected by AEs (Table 3). However, after taking into account the shorter treatment duration with PM than with SSG, there was only weak evidence of a lower rate of TEAEs in the PM arm (adjusted rate ratio 0.78, 95%CI 0.61 to 0.99, p = 0.041). Increases of liver enzymes and injection site pain were the most common AEs in both groups, followed by infections and epistaxis (Table 4).

**Table 3:** Number of patients with adverse events.

	SSGn = 135	PMn = 135
**Deaths**	3 (2.2%)	2 (1.4%)
**Patients with SAE** *	8 (5.9%)	5 (3.7%)
Treatment-emergent†	7 (5.2%)	3 (2.2%)
During follow-up	1 (0.7%)	2 (1.4%)
Unrelated to study drug	3 (2.2%)	2 (1.4%)
Unlikely, possibly or probably related	5 (3.7%)	3 (2.2%)
**Patients with at least one AE at any time**	99 (73.3%)	77 (57.0%)
TEAEs†	90 (66.7%)	65 (48.2%)
AEs during follow-up	35 (25.9%)	33 (24.4%)
**TEAEs**		
Total number of TEAEs	169	112
Total patient-days at risk	8100	6885
TEAE rate	0.021	0.016
TEAE rate ratio (95% CI) comparing PM with SSG, adjusted for centre	0.78 (0.61 to 0.99, p = 0.041)	

Data are n (%) unless otherwise stated. AE = adverse event. SAE = serious adverse event. TEAE = treatment-emergent adverse event.

*No patient experienced more than one SAE.

**†:** TEAEs defined as onset being between day 1 of treatment and 30 days after end of treatment inclusive. AEs occurring during follow-up had onset recorded as between day 31 of trial and end of study.

**Table 4:** Number of patients experiencing non-serious treatment-emergent adverse events (TEAEs).

	SSGn = 135	PMn = 135
**Gastrointestinal disorders**	**6 (4.4%)**	**6 (4.4%)**
Abdominal pain	1 (0.7%)	2 (1.5%)
**General disorders and administration site conditions**	**13 (9.6%)**	**19 (14.1%)**
Injection site pain	12 (8.9%)	16 (11.9%)
**Infections and infestations**	**14 (10.4%)**	**8 (5.9%)**
Gastroenteritis	1 (0.7%)	2 (1.5%)
Pneumonia	2 (1.5%)	0 (0.0%)
Urinary tract infection	4 (3.0%)	1 (0.7%)
PKDL	5 (3.7%)	1 (0.7%)
**Investigations**	**42 (31.1%)**	**24 (17.8%)**
Hepatic enzymes increased	27 (20.0%)	14 (10.4%)
AP increased	7 (5.2%)	5 (3.7%)
Blood amylase increased	4 (3.0%)	0 (0.0%)
Blood creatinine increased	1 (0.7%)	3 (2.2%)
**Nervous system disorders**	**2 (1.5%)**	**5 (3.7%)**
Headache	1 (0.7%)	4 (3.0%)
**Respiratory, thoracic and mediastinal disorders**	**6 (4.4%)**	**2 (1.5%)**
Epistaxis	5 (3.7%)	2 (1.5%)
**Skin and subcutaneous tissue disorders**	**6 (4.4%)**	**1 (0.7%)**
Rash	2 (1.5%)	1 (0.7%)

Data are n (%). AP = alkaline phosphatase. PKDL = post-kala-azar dermal leishmaniasis. TEAEs listed above are related to the study drug with an incidence of at least 1% in any group.

SAEs were reported in eight (5.9%) SSG patients and five (3.7%) PM patients (Table 3). Two patients in each arm prematurely stopped treatment due to SAEs and received rescue medication, and overall five patients died (Figure 1). Two deaths occurred during hospitalization, both due to renal failure in patients receiving SSG, and three after discharge due to other infections, one in the SSG and two in the PM arm. Among the non-fatal SAEs that were considered unlikely, possibly or probably related to study drug, there was one case of clinical pancreatitis and one of seriously elevated serum amylase with SSG and single cases of anemia, cardiac failure, and thrombocytopenia with PM. There were no marked differences in reported SAE rates among sites.

## Discussion

Clinical research and development of drugs against leishmaniasis in Africa has generally to overcome significant logistical hurdles in terms of remote rural settings and lack of resources. Through the establishment of the Leishmaniasis East Africa Platform (LEAP), a network of researchers supported by DND*i* that implements and coordinates clinical trial activities in Sudan, Ethiopia, Kenya, and Uganda, these hurdles were overcome. This report emanates from the largest international study on VL and the first phase III study on PM in Africa.

The PM dose regimen of 15 mg/kg/day was previously shown to be highly efficacious with ∼95% cure rates in the treatment of VL in India [17]; however, in this study its efficacy varied considerably by region. It was particularly low in eastern Sudan (northern endemic zone), while being somewhat better in southern Ethiopia and Kenya (southern endemic zone). However, overall cure was only 63.8% and hence significantly and unacceptably lower than that with SSG. This finding was pronounced and robust as confirmed in both the complete-case and worst-case analyses. Hence, even though the study was not designed to detect differences among sites and despite some variable loss to follow-up among sites, the significant regional variation in efficacy is unlikely to be due to an individual site effect. A phase II trial testing liposomal amphotericin B against primary VL showed differences in site efficacy [19]. In this study, lower doses of amphotericin B were needed to cure patients in India than to cure patients in Kenya or Brazil.

Consequently, there have been three important outcomes following these initial results. The first is that the evaluation of PM at a dose of 15 mg/kg for 21 days was discontinued in the East African countries. Second, because it is difficult to conclude what the underlying causes of PM failure at a dose of 15 mg/kg/day were, based on these data alone, a pharmacokinetic investigation and regional drug sensitivity testing are currently underway to assess whether patients or parasite factors may account for this failure. The third outcome following a dose evaluation study was a decision to continue evaluation of PM at a dose of 20 mg/kg for 21 days in comparison to both SSG and the combination (at the doses previously used).

The reason to continue evaluation of PM is strengthened by the fact that very few treatment options are available for the management of VL in Africa [1]. Antimonials (either SSG or meglumine antimoniate) remain the current first-line treatment in all VL endemic countries. Miltefosine is neither yet registered nor available in East Africa, and has only been evaluated in one trial in Ethiopia [9]. Use of liposomal amphotericin B at its currently recommended dose (20–30 mg/kg) remains limited by cost, even with the recent cost reduction announced in 2007. Furthermore, the most appropriate and practical dose for the region has not been established; however, a clinical trial is underway to assess this. PM is still the cheapest available VL treatment, and its ease of use in resource-limited settings makes it an attractive option [20]. As the drug seems efficacious and relatively well-tolerated in India and in parts of East Africa, a risk–benefit evaluation performed by LEAP favored the assessment of an increased dose.

The intention remains that PM will be used in combination with SSG, as this combination has already been used in the field setting in south Sudan [16], [21]. As shown with other diseases such as malaria, combination therapy remains highly desirable as it may maintain a high efficacy, reduce length of treatment, and protect both drugs from development of resistance. Evaluation of PM monotherapy at a dose of 20 mg/kg for 21 days and the combination of SSG and PM at 15 mg/kg for 17 days has been continued, and is expected to be completed in 2010. After results are analyzed, a definitive decision can be made whether to proceed with registration of PM and recommendation for use of PM in combination with other anti-VL drugs in the VL endemic countries in Africa. Although this study provides negative efficacy results of PM monotherapy in some regions in East Africa, it has strengthened regional clinical research capacity through LEAP and has served as an essential cornerstone upon which further prospective treatments will be evaluated.

## Supporting Information

Protocol S1 Trial protocol details and amendments.
(6.45 MB PDF)

Checklist S1 CONSORT checklist.
(0.19 MB DOC)
