# Supplementary material for: Geographical Variation in the Response of Visceral Leishmaniasis to Paromomycin in East Africa: A Multicentre, Open-Label, Randomized Trial
Source: PLoS Negl Trop Dis. 2010 Oct 26;4(10):e709. doi: 10.1371/journal.pntd.0000709 (PMC2964287; doi:10.1371/journal.pntd.0000709)
Supplement: Protocol S1 — Trial protocol details and amendments. (6.45 MB PDF) [file pntd.0000709.s001.pdf]

16. APPENDICES

Appendix 1	Final Protocol and amendments
Appendix 2	Sample CRF
Appendix 3	List of IECs
Appendix 4	Samples of patient information sheets and consent forms
Appendix 5	List of principal and co-investigators, affiliations and roles
Appendix 6	CVs of principal and co-investigators
Appendix 7	List of DSMB members and their affiliation
Appendix 8	List of monitors
Appendix 9	Signatures
Appendix 10	Listing of patients per allocated treatment and drug batch
Appendix 11	Randomisation master list
Appendix 12:	Clinical Audit
Appendix 13:	Laboratory Audit
Appendix 14:	Parasitology Quality Control
Appendix 15	Drug re-analysis report from IDA
Appendix 16	Statistical Analysis Plan
Appendix 17	Statistical Analysis Report
Appendix 18	Lab normal ranges
Appendix 19	List of trial site monitoring visits
Appendix 20	List for GCP trainings and number of attendees
Appendix 21	Sundar et al. 2007
Appendix 22	CP Thakur et al 2000
Appendix 23	Patient visit dates
Appendix 24	Listing of discontinued patients
Appendix 25	Listing of protocol deviations
Appendix 26	Individual patient demographic data
Appendix 27	Individual Baseline VL symptoms
Appendix 28	Individual Medical History
Appendix 29	Individual Height and Weight
Appendix 30	Individual Heart rate and Axiliary temperature
Appendix 31	Individual Systolic and Diastolic Blood pressure
Appendix 32:	Listing of Daily Paromomycin Treatment
Appendix 33:	Listing of Daily SSG Treatment
Appendix 34:	Listing of Daily Combination Treatment (Paromomycin)
Appendix 35:	Listing of Daily Combination Treatment (SSG)
Appendix 36:	Listing of Rescue medication
Appendix 37:	Listing of concomitant medications
Appendix 38:	Listing of individual efficacy data – Clinical Response and Parasitology
Appendix 39:	Listing of individual efficacy data – Spleen size and liver size

Appendix 40:	Listing of individual efficacy data – Clinical Characteristics, cervical lymphadenopathy and axiliary lymphadenopathy
Appendix 41:	Listing of individual efficacy data – Clinical Characteristics, inguinal lymphadenopathy and muscle wasting
Appendix 42:	Listing of individual efficacy data – Clinical Characteristics, mucosal pallor and jaundice
Appendix 43:	Listing of individual efficacy data – Clinical Characteristics, Petchial haemorrhage
Appendix 44	Listing of adverse events per patient
Appendix 45	Listing of ECG Findings per patient
Appendix 46	Listing of chest x-ray finding per patient
Appendix 47	Listing of audiometry x-ray finding per patient
Appendix 48	SAE reports
Appendix 49:	Listing of Haematology measurements per patient – Haemaglobin
Appendix 50:	Listing of Haematology measurements per patient – White blood cells
Appendix 51:	Listing of Haematology measurements per patient – Platelets
Appendix 52:	Listing of Haematology measurements per patient – Prothrombin time
Appendix 53:	Listing of clinical chemistry measurements per patient – ALT and AST
Appendix 54:	Listing of clinical chemistry measurements per patient – Alkaline Phosphatase
Appendix 55:	Listing of clinical chemistry measurements per patient – Amylase
Appendix 56:	Listing of clinical chemistry measurements per patient – Bilirubin
Appendix 57:	Listing of clinical chemistry measurements per patient – Creatinine and BUN
Appendix 58:	Listing of clinical chemistry measurements per patient – Globulin
Appendix 59:	Listing of clinical chemistry measurements per patient - Albumin
Appendix 60:	Listing of clinical chemistry measurements per patient – Total Protein
Appendix 61:	Listing of urinalysis measurements per patient – Urinary Protein
Appendix 62:	Listing of urinalysis measurements per patient – Urinary blood
Appendix 63:	Listing of urinalysis measurements per patient – pH and Specific Gravity

16.1 STUDY INFORMATION

16.2 Protocol and protocol amendments

Appendix 1 Final Protocol and amendments

TITLE

A MULTICENTRE COMPARATIVE TRIAL OF EFFICACY AND SAFETY OF SODIUM STIBOGLUCONATE (SSG) VERSUS PAROMOMYCIN (PM) VERSUS COMBINATION OF SSG AND PM AS THE FIRST LINE TREATMENT FOR VISCERAL LEISHMANIASIS IN ETHIOPIA, KENYA AND SUDAN

Principal Investigators
Dr Monique Wasunna[1]
Dr. Asrat Hailu [2]
Dr. Getahun Mengistu [2]
Dr. Musa Amudawi[3]
Dr. Manica Balasegaram[4]

Project Manager
Dr Catherine Royce[5]

Statistician
Mr Lawrence Muthami[1]

[1]Kenya Medical Research Institute, Kenya
[2]University of Addis Ababa, Ethiopia
[3]Institute of Endemic Diseases, University of Khartoum, Sudan
[4]Médecins Sans Frontières Holland, Sudan
[5]Drugs for Neglected Diseases Initiative, Switzerland
[6]University of Nairobi, Kenya
[7]Gedarif University, Sudan
[8] National Ribat University, Sudan

Co- investigators: KENYA
Dr. J R Rashid[1]
Dr. J. Mbui[1]
Dr. P. Nyakundi[1]
Dr. G. Mucee[1]
Dr. V. Manduku[1]
Dr. A. Musibi[1]
Dr. Z. Mutuma[1]
Dr. F. Kirui[1]

Dr. H. Lodenyo[1]
Mr. D. Kinoti[1]
Prof. K. Bhatt[6]

Co-investigators: SUDAN

Professor A.M. El-Hassan[3]
Professor Eltahir Awad Gasim Khalil[3]
Dr M.E. Ibrahim[3]
Dr I.M. Elhassan[3]
Dr Ahmed Abdalla[7]
Dr Fawzi Abdeirahim Mahjoub[8]

Co- investigators: ETHIOPIA

Prof Eyasu Makonnen[2]
Dr Yalemtsehay Mekonnen[2]
Dr Shibru Berhanu [Gondar]
Dr Asfawesen Gebre-Yohannes [Gondar]
Dr Sisay Yifru [Gondar]
Dr Abiye Tesfaye [Gondar]
Dr Nurelign Gashu [Gondar]
Dr Samson Tesfaye [Arba-Minch]
Dr Yewubnesh Hailu [Arba-Minch]
Dr Degu Jerene [Arba-Minch]

SIGNATURE PAGE

**Principal Investigators**	**SIGNATURE**	**DATE**
Dr Monique Wasunna	……………………	……………….
Dr. Asrat Hailu	……………………	……………….
Dr. Getahun Mengistu	……………………	……………….
Dr. Musa Amudawi	……………………	……………….
Dr. Manica Balasegaram	……………………	……………….
**Project Manager**		
Dr Catherine Royce	……………………	……………….
**Statistician**		
Mr. Lawrence Muthami	……………………	……………….

TABLE OF CONTENTS

LEAP DRAFT PROPOSAL……1
TITLE……1
INVESTIGATORS……1
SIGNATURE PAGE……3
SUMMARY……6
LITERATURE REVIEW……6
DISTRIBUTION OF VISCERAL LEISHMANIASIS IN EAST AFRICA……7
CLINICAL ASPECTS OF LEISHMANIASIS IN EASTERN AFRICA……8
MAIN TREATMENT OPTIONS FOR VISCERAL LEISHMANIASIS……9
NEED FOR NEW TREATMENT OPTIONS……10
SSG……10
PAROMOMYCIN……11
HISTORICAL PRODUCT PROFILE – FARMITALIA DOSSIER……11
PRE-CLINICALTOXICOLOGY……12
ANIMALTOXICOLOGY……13
CLINICAL PHARMACOLOGY……15
PHARMACOKINETICS……15
DOSE-FINDING STUDIES……15
CLINICAL EXPERIENCE WITH INJECTABLE PAROMOMYCIN IN THE TREATMENT OF VL……16
TRIAL OBJECTIVES AND PURPOSE……17
HYPOTHESIS……17
OBJECTIVES OF THE TRIAL……17
METHODOLOGY……18
STUDY DESIGN……18
STUDY SITES……18
INCLUSION CRITERIA……18
EXCLUSION CRITERIA……19
HIV-STATUS AND VCT……19
CRITERIA FOR PATIENT WITHDRAWAL……20
SAMPLE SIZE……21
TREATMENT……23

DRUG ADMINISTATION……………………………………………………………………………………23

RESCUE MEDICATION……………………………………………………………………………………24

PRIOR AND CONCOMITANT MEDICATIONS……………………………………………………24

EFFICACY ASSESSMENT…………………………………………………………………………………24

PRIMARY EFFICACY ENDPOINT………………………………………………………………………25

SECONDARY EFFICACY ENDPOINT……………………………………………………………………25

SAFETY ASSESSMENTS……………………………………………………………………………………26

ADVERSE EVENTS……………………………………………………………………………………………26

ASSESSMENT OF INTENSITY/SEVERITY…………………………………………………………… 26

ASSESSMENT OF CASUALITY……………………………………………………………………………26

SERIOUS ADVERSE EVENTS………………………………………………………………………………27

DATA COLLECTION, STORAGE AND ANALYSIS…………………………………………………28

QUALITY CONTROL AND QUALITY ASSURANCE…………………………………………………29

ETHICAL CONSIDERATIONS………………………………………………………………………………29

INSURANCE AND LIABILITY………………………………………………………………………………30

TIME FRAME……………………………………………………………………………………………………31

REFERENCES……………………………………………………………………………………………………32

PATIENT INFORMATION AND CONSENT………………………………………………………………35

SUMMARY

Visceral leishmaniasis (VL) or Kala-azar is the most severe form of leishmaniasis. It is estimated that 500,000 new cases world wide of VL are diagnosed annually. 90% of VL cases occur in developing countries: India (especially Bihar), Bangladesh, Nepal, North Eastern Brazil and Sudan. For the past 100 years, antimony has been the first line of treatment for VL cases despite considerable toxicity and the requirement for 4 weeks hospitalization.

Resistance to antimony coupled with emergence of HIV associated with VL is on the increase. New and improved treatment options are urgently needed to replace or complement the few currently available drugs. The wide variety of epidemiological situations and clinical presentations of this disease further warrant a series of treatment options instead of one single treatment or control strategy for the affected populations.

During 2003, experts in VL together with representatives of regulatory authorities and health ministries from Kenya, Ethiopia and Sudan met (Nairobi, May 2003, Khartoum, August 2003) to discuss the development of new treatment options for this fatal but neglected disease.

This research proposal will be a multicentre, prospective, open label, parallel group, comparative trial to determine the efficacy and safety of sodium stibogluconate (SSG) 20mg/kg/day given for 30 days, Paromomycin (PM) 15mg/kg/day for 21 days, and a combination of SSG and PM, 20mg/kg/day, 15mg/kg/day respectively, given for 17days in the treatment of patients suffering from VL in Ethiopia, Kenya and Sudan. Primary endpoint will be cure rate at 6 months.

LITERATURE REVIEW

The leishmaniases are a group of diseases caused by Leishmania parasites, of which at least 20 different species can cause human disease. Leishmania infection is transmitted by the bite of female sandflies. The disease occurs in three forms: self-healing cutaneous leishmaniasis (CL), mutilating mucosal leishmaniasis (ML or MCL) and life-threatening visceral leishmaniasis (VL). Each form varies in degree of severity, with visceral leishmaniasis being by far the most devastating.

Today, of the estimated 350 million people at risk in 88 countries, 12 million people are thought to be affected by leishmaniasis in its different forms, with an estimated 1.5 -2 million new cases occurring annually (1-1.5 million cases of CL/MCL and 500,000 cases of VL) (WHO 2000). In the past decade, the number of leishmaniasis cases has risen (Desjeux 2001) due to increased human exposure to the sandfly vector as well as the spread of AIDS and other immunosuppressive conditions that have increased the risk of *Leishmania*-infected people developing the disease.

Visceral leishmaniasis (VL) or kala-azar is the most severe form of the disease. If untreated, VL has a mortality rate of almost 100%. In 1999, there were 57,000 (reported) deaths due to kala-azar. Ninety per cent of VL cases occur in five developing countries: India (especially Bihar), Bangladesh, Nepal, North Eastern Brazil, and Sudan.

Distribution of Visceral Leishmaniasis in Eastern Africa

In Eastern Africa, especially Sudan, Ethiopia and Kenya, visceral leishmaniasis is by far the most common form of the disease and is the cause of much death and disease.

VL in Ethiopia has been reported from over 40 localities in different parts of the country. The infection is either due to L. donovani, L. infantum or L. archbaldi. Most infections are acquired in north-west Ethiopia in the lowlands of Metema and Humera, south-west Ethiopia in the Segen, Woitu and Omo river basins, and in other isolated foci in the rift valley. The north-western Metema-Humera focus (which extends northwards to Eritrea and westwards into eastern Sudan) is a major VL focus which presently accounts for approximately 60% of the total disease burden in Ethiopia. This focus extends over a huge land mass in two regions, Region 1 (Tigray) and Region 3 (Amhara). In this focus MSF-H is actively involved in treatment of cases, with at least 2000 cases benefiting from treatment every year. The patients in this focus are mostly migrant laborers, and one would expect up to 40% of the cases to be HIV co-infected. VL foci in Segen, Woitu and Omo river basins represent typical endemicty. The VL cases from these foci, account for approximately 20% of the total burden in the country, and HIV co-infection is less than 2%. These foci are located in the Southern Nations, Nationalities and Peoples Regional Government (SNNPRG). Other foci are in Region 4 (Oromia), Region 5 (Somali), and Region 2 (Afar). Sporadic case reports are known from other smaller localities. For instance, in Moyale, at the borders

with Kenya and in areas northeast of Lake Abaya. Members of the Ethiopian Army and Police Forces who acquire VL in the endemic areas are admitted in Addis Ababa referral hospitals. This is a special risk group and HIV co-infection could be expected to be more than 50%. (Hailu 2004, Ayele 2004)

In Eritrea, the Red Sea littoral (localities like Nakfa, Afabet, Algena, Keren) and the district of Teseney also in Eritrea (North of Humera) are endemic.

Eastern Sudan (Gedarif State), Upper Nile and Western-Upper Nile are known endemic areas for visceral leishmaniasis in the Sudan. VL is among the most important health problems in the Sudan with more than 24,660 cases and 1193 deaths that has been reported during 1996-2001. The number of reported cases is mainly a reflection of reporting rather than the actual disease transmission. Reports and published work from Sudan showed that the disease affects mainly children with few adult cases. The disease is reported to be more prevalent among poor people, malnourished, vagrant, farmers, laborers, water carrier, and those out of country, who have a very limited capacity to assume the costs of the disease (Sudan Manual 2004).

In Kenya, the endemic foci of VL include Baringo, Turkana, West Pokot, Kitui, Meru, and Machakos districts. The first 3 districts are in Rift Valley province while the latter are in Eastern province. Numerous outbreaks of VL were reported from these areas in the late nineteen seventies with over 2000 cases reported from Meru and Kitui districts only (WHO 1990). All these areas are generally semi-arid, sparsely populated with low rainfall and high temperatures. Low agricultural and economic productivity has resulted in poor social economic status (SES) of the population in these areas.

Population displacements as a result of war, drought, famine, or rural-urban migration have exacerbated the spread of the disease. For instance, the epidemic in western Upper Nile, an area where VL was previously not endemic, caused an estimated 100,000 deaths between 1984 and 1992, or a population mortality of up to 36% (Seaman et al. 1996)

Clinical Aspects of Leishmaniasis in Eastern Africa

Visceral leishmaniasis, is a devastating illness, fatal if left untreated. Patients with VL present with fever, malaise, cough, abdominal pain, diarrhoea, epistaxis, splenomegaly, hepatomegaly, cachexia, anaemia, pancytopenia, lymhadenopathy and malnutrition.

Not all infected people develop clinical kala azar; some have a sub clinical infection that spontaneously resolves. The ratio of those with clinical disease to those with sub clinical disease varies remarkably from place to place, and during periods of epidemics. In eastern Sudan, the ratio of clinical cases to mild or sub clinical diseases was 1.6:1, and increased to 3.3:1 during a recent outbreak. The scenario is less critical in Brazil where, during an outbreak, the ratio was 1:8 or 1:16 (showing much less disease per infection); in Iran, it is 1:12 (MSFH 2003) Malnutrition, anaemia and immune depression increase the likelihood that infection will progress to the disease. In Ethiopia, Kenya, and Sudan the problems of infected children are compounded by these very reasons, as well as opportunistic infections such as tuberculosis and pneumonia. Infected adults also bear the brunt of these problems – in Ethiopia HIV is found in association with kala-azar in approximately 35-50% of cases (Dr Asrat Hailu – personal communication).

The incubation period for VL varies widely and it is estimated to be between 2 – 6 months. Malnutrition, anaemia and immune depression increase the likelihood that infection will progress to the disease.

A complication of visceral leishmaniasis, especially prevalent in Sudan (and to a lesser extent Ethiopia, and Kenya) is post-kala-azar dermal leishmaniasis (PKDL) (Zijlstra et al 2003) occurring in people who have recovered from VL following treatment.

Main treatment options for visceral leishmaniasis.

Treatment of VL cases in Eastern Africa always presents with challenges such as patients coming late when they are extremely ill and may die during treatment due to the illness as well as toxicity of the drugs used. The other challenges include availability of drugs, drug resistance, and cost of treatment (drugs and hospitalization). In VL endemic areas, facilities may not be available for accurate diagnosis and follow up, and the increasing prevalence of HIV co-infection is an additional challenge, particularly in Ethiopia.

Table 1: Current treatment options for patients with visceral leishmaniasis

Drugs available for use	Associated problems
**Pentavalent antimonials**	Toxic, parasite resistance growing<br>30 day IV/IM treatment in hospital
**Amphotericin B**	Used in case of antimonial resistance but dose-limiting toxicity, 15-20 day IV treatment in hospital
**Liposomal Amphotericin B**	Less toxic but prohibitively expensive
**Miltefosine**	Teratogenic, only registered in India, and expensive

Need for new treatment options

In eastern Africa, the first line treatment today in most endemic areas is antimonial therapy for 4 weeks sodium stibugluconate,(Pentostam® from GSK in Kenya or generic SSG from Albert David in Sudan and Ethiopia) used at 20 mg/kg/day for 28-30 days). Although the efficacy of this treatment is not yet compromised by resistance in this region (in contrast to Bihar – India), the painful daily injections, the need for four weeks of hospitalisation, the toxicity when using longer treatments, the low efficacy in HIV co-infected patients and the risk of inevitable drug resistance, as observed in India, make alternative options a necessity. Second line treatments are either toxic or prohibitively expensive.

In 2002-3, a combination of SSG and PM given for 17 days was used in an epidemic situation in Southern Sudan, with an initial cure rate of 97% (personal communication from Koert Ritmeijer, Médecins Sans Frontières). These findings were in line with previous published experience in the same area (Seaman et al, 1993) and experience in Kenya (Chunge et al, 1990). The proposed study aims to confirm these results in a randomized prospective comparative study.

SSG

Despite the shortcomings listed in table 1, sodium stibogluconate (SSG) is still the most widely used drug for VL in Eastern Africa. SSG is known to cause cardiac, muscle, joint and renal problems. Emergence of resistance as has occurred in the Indian subcontinent (Bihar state) make investigating combination schedules a priority.

Paromomycin

Paromomycin (PM) is a broad-spectrum aminoglycoside antibiotic produced from culture filtrates of Streptomyces krestomyceticus and is identical to aminosidine (Shilling & Shaffner, 1961). PM is very poorly absorbed from the gut, an oral formulation is available for the treatment of infections caused by bacteria, protozoa and worms from the intestinal lumen. For the treatment of systemic infections, for example VL, a parenteral formulation is required.
An injectable formulation of 500 mg of PM sulphate has been marketed in several countries for over 35 years for the treatment of bacterial and parasitic infections, however it has not been licensed specifically for the treatment of VL.

The anti-leishmanial activity of injectable paromomycin was first demonstrated in the 1960s and subsequently confirmed in vitro and in vivo. Since then, it has also been shown to be effective against visceral leishmaniasis (Chunge 1990, and others) and is affordable and well tolerated.
Efficacy of PM has also been shown in Bihar, India, the region with the greatest incidence of kala azar and the highest rates of antimony resistance (Thakur 2000)

Historical Product Profile – Farmitalia dossier

Summary data are available on a total of 2,397 patients treated with injectable paromomycin for various infectious diseases. Patient population ranged from newborn infants to the elderly. In most cases, adults received up to 2g/d for 30 days, although patients with skin infections were given up to 1.5g/d for 49 days.

Summary of results:
Paromomycin was well tolerated. Adverse events (AEs) involving hearing function were reported in 10 (0.4%); two patients had renal function decrease and one albuminuria; 21 additional patients had other AEs. The occurrence of AEs was not related to the age of patients. AEs involving hearing tended to occur in patients administered large dose of PM and/or multiple-drug regimens.

Safety Data - Historical Japanese Post Marketing Data:
Pre and post marketing safety surveillance safety data is available from 2220 patients. The incidence of adverse reactions is as follows: Pain at injection site 94 (4.2%), local rash 30 (1.4%), tinnitus 8 (0.4%), malaise 9 (0.4%), skin rash 5 (0.2%), nausea/vomiting 4 (0.2%), diarrhea 2 (0.1%). The major dose limiting toxicities of injectable paromomycin are the same as other drug in the

aminoglycoside class (e.g. streptomycin, gentamycin) being oto- and renal toxicity. These toxicities are related total dose of the drug given and duration of therapy.

Pre-clinical toxicology

Mutagenicity/Genotoxicity: GLP Institute Pasteur Lille

- Mutagenicity test on bacteria using Ames technique
- Genotoxic activity using the micronucleus test
- Mutation assay at the TK locus in L5178Y Mouse lymphoma cells using a microtitre cloning technique
- Test for chromosomal aberrations by in vitro human lymphocyte metaphase analysis

Results: All tests were negative for mutagenicity/genotoxicity

Animal toxicology

Table 2: Animal toxicology studies – part 1

Study	Species	Route	Dose/Duration	Sponsor	GLP	Main results
Acute	Mouse, Rat	IV, IM, IP, IC, SC, PO	Mice- LD_{50} g/kg<br>- IV 0.106- 0.110<br>- IP 0.750<br>- SC 0.70 - 1.06<br>- IC 0.023<br>- PO 15.0 –17.8<br>Rat – LD_{50} g/kg<br>- IM 1.20<br>- SC 0.87<br>- PO 21.62	FCE	No	The LD_{50} is 8-10x greater than the therapeutic dose in humans
Chronic	Mice, Rats, Cats	IM	Mice mg/kg/day<br>- 400 for 60 days<br>Rats mg/kg/day<br>- 264 for 82 days<br>Cats mg/kg/day<br>- 50 for 37 days	FCE	No	No mortality or vestibular damage seen
Nephrotoxicity	Mice, Rats, Cats	IM	Mice mg/kg/day<br>- 400 for 60 days<br>Rats mg/kg/day<br>- 264 for 82 days<br>Cats mg/kg/day<br>- 50 for 37 days	FCE	No	Mice moderate renal damage<br>Rats slight renal damage<br>Cats moderate renal damage

Table 3: Animal toxicology studies – part 2

Study	Species	Route	Dose/Duration	Sponsor	GLP	Main results
Cochleo Vestibular	Rats, Guinea Pigs,	SC	Rats mg/kg/day<br>- 200, 264 for 60 days<br>G. Pigs mg/kg/day<br>- 50, 100, 200, 400 for 30 days<br>- 20 for 60 days<br>- 200 for 28 days comparative trial with KM and DHSM	FCE	No	Rat dose related cumulative effect on acoustic sensitivity<br>Guinea Pig dose related cumulative effect for oto-toxicity. In the comparative trial AM was less ototoxic than KM or DHSM
Reprotox	Mice, Rats, Rabbits	IM, SC	Teratogenesis<br>Mice mg/kg/day<br>- 100, 200, 300 IM for 7 days<br>Rats mg/kg/day<br>- 100, 200, 300 IM for 7 days<br>Embryo-fetal<br>Rats mg/kg/day<br>- 100, 200 SC for 19 days<br>Rabbits mg/kg/day<br>- 12.5, 25 SC for 28 days	FCE	No	No teratogenic effect detected.<br>No statistically significant embryo-fetal toxicity
Thirteen Week Chronic Toxicity	Dogs	IM	Dogs mg/kg/day<br>30, 100 for 13 weeks	SoloPak	Yes	Low dose dogs slight to minimal renal damage, and a frequency dependent hearing loss at high tones. High doses dogs severe chronic nephropathy, and renal tubular degeneration. Unable to detect audiometric hearing frequencies Swelling and chronic inflammation at injection site

Clinical Pharmacology

Pharmacokinetics

An HPLC assay was developed at the University of Illinois at Chicago under GLP conditions in order to be able to determine the concentration of paromomycin in biological fluids (e.g. urine and plasma)

Single Dose Intramuscular Pharmacokinetics in Healthy Normal Volunteers

Sixteen HNVs were given a single IM dose of paromomycin base either 12 or 15 mg /kg (8 per group)

Table 4: Pharmacokinetic parameters

Dose mg/kg /day	C_{max} (µg/ml)	T_{max} (h)	K_a (h^{-1})	T_{lag} (h)	AUC (µg h/ml)	CL/F (ml/min/1.73M^2)	V_β/F (l/kg)	$t_{1/2}$ (h)
12	21.6	1.19	6.27	0.23	86.3	117.7	0.35	2.21
15	23.4	1.51	2.65	0.20	104.5	126.0	0.41	2.64

Dose finding Studies

1) Randomized phase II clinical study: Kala-azar Research Centre, Muzaffarpur, Bihar, India; T.K. Jha (Jha et al., 1998)

Group (mg/kg/d)	Enrolled	Treatment Completed	Treatment Failures	Relapses	Defaulters	Definitive Cure 180d (%)
PM 12 x 21d	30	30	2	5	0	23/30 (76.7)
PM 16 x 21d	30	30	0	1	1	28/29 (96.5)
PM 20 x 21d	30	30	0	1	0	29/30 (96.7)
SB 20 x 28d	30	30	8	3	0	19/30 (63.3)

2) Randomized phase II clinical study: Patna Medical College, Patna, Bihar, India; (Thakur *et al*, 2000: 94:)

Group (mg/kg/d)	Enrolled	Treatment Completed	Treatment Failures	Relapses	Defaulters	Definitive Cure 180d (%)
PM 12 x 21d	30	30	0	3	0	27/30 (90.0)
PM 16 x 21d	30	30	0	3	3	24/27 (88.9)
PM 20 x 21d	30	30	0	4	1	25/29 (86.2)
SB 20 x 28d	30	30	8	1	1	20/29 (69.0)

3) Randomized, comparative, open-label trial of the safety and efficacy of Paromomycin (PM) + sodium stibogluconate (SB) versus sodium stibogluconate alone for the treatment of visceral leishmaniasis: Patna Medical College, Patna, Bihar, India; (Thakur *et al*, 2000)

Group (mg/kg/d)	Enrolled	Treatment Completed	Treatment Failures	Relapses	Defaulters	Definitive Cure 180d (%)
PM12+SBx 21d	52	51	3	1	0	48/52 (92.3)
PM18+SBx 21d	48	46	2	1	0	45/48 (93.8)
SB20 x 28d	50	46	21	1	1	27/50 (54.0)

Clinical Experience with injectable paromomycin in the Treatment of VL

Previously, clinical trials with injectable PM either alone or in combination with SB for the treatment of VL have been conducted in Africa (Kenya and Sudan), India (Bihar), and in cases imported into the United Kingdom (Jha, et al., 1998, Hassan M, et al., 1995 Thakur et al., 1995; Seaman et al, 1993, Thakur et al, 1992, Scott et al, 1992, Chunge et al., 1990). In all the studies the investigators reported that PM, used as a single agent or combined with SB was highly efficacious and well tolerated in the treatment of VL caused by L. donovani or infantum.

Table 5: Summary of clinical studies using PM

Dose mg/kg/day	Single Agent No. Patients	Place	Combination Therapy with SSG No. Patients	Place
6			40	India (Thakur)
12	60	India (30 Jha, 30 Thakur)	120	India (96 Thakur, 24 Thakur)
14-16	19	Kenya (Chunge)	124	Kenya and Sudan (23 Chunge, 101 Seaman)
16	60	India (30 Jha, 30 Thakur)		
18			50	India (Thakur)
20	60	India (30 Jha, 30 Thakur)		
**Total Patients**	**199**		**384**	

TRIAL OBJECTIVES AND PURPOSE

Currently in the three countries, Sudan, Kenya and Ethiopia many of the patients present themselves in remote areas and need to be treated in relative resource poor settings. It is for this reason that standardised treatment with proven efficacy is much needed. A shorter course of treatment is not only advantageous for the patient but also reduces the overall case load in the clinics thus reducing the risk of disease outbreaks in already immuno-compromised kala-azar patients. Paromomycin, either alone or in combination with SSG would decrease the treatment duration substantially. An additional added value of combination therapy is that it is likely to reduce the chances of development of parasite resistance against the individual drugs.

Leishmaniasis experts in the three countries are in agreement that there are potential benefits of the combination treatment of SSG and PM and that its efficacy should be evaluated with the view to introduce this protocol if proven efficacious and safe. There is ample circumstantial evidence of the use of this combination therapy and its efficacy and tolerability as a standardized protocol. This can only be confirmed through a randomised controlled study with 6 months follow up.

HYPOTHESIS

That a combination course of SSG and PM (17 days) is similar in efficacy to either PM alone(21 days) or SSG alone (30 days) and that the shorter course combination (17 days) is not more toxic than PM or SSG alone.

OBJECTIVES OF THE TRIAL

1) To assess the efficacy and safety of SSG 30 days alone in the treatment of patients with VL.

2) To assess the efficacy and safety of PM 21 days alone in the treatment of patients with VL.

3) To assess the efficacy and safety of SSG and PM as a combination course of 17 days in the treatment of patients with VL.

METHODOLOGY

STUDY DESIGN

This will be a multi-centre, prospective, open, parallel group, comparative trial of efficacy and safety of SSG alone given IM/IV (according to usual hospital practice) for 30 days versus paromomycin alone given IM for 21 days versus a combination of SSG and PM given for 17 days, in the treatment of patients suffering from VL in Ethiopia, Kenya and Sudan.
Patients who have clinical symptoms and a confirmed parasitological diagnosis of VL by splenic aspirate, lymph nodes aspirate or bone marrow aspirate (to be specified for each hospital site) and who have fulfilled the inclusion/exclusion criteria will be enrolled.
The primary endpoint will be cure rate at 6 months post treatment. Secondary endpoints will be cure rate at end of treatment (Day 31 for SSG, Day 22 for PM, Day 18 for PM + SSG) and at three months post treatment.

STUDY SITES

The study will be conducted at the following sites;

Ethiopia:

- Arba Minch hospital
- Gondar hospital

Kenya:

- Centre for Clinical Research (CCR), Kenya Medical Research Institute (KEMRI), Nairobi.

Sudan:

- Kassab Hospital
- Um-El-Kher (MSFH treatment centre)

INCLUSION CRITERIA

Patients who fulfill the following inclusion criteria will be enrolled into the study:-

1) Patients for whom written informed consent has been signed by the patients themselves (if aged 18 years and over) or by parents(s) or legal guardian for patients under 18 years of age.

2) Patients aged between 4 and 60 years (inclusive) who are able to comply with the protocol. It is justified to include children because they represent more than 50% of VL cases.
3) Patients with clinical signs and symptoms of VL and diagnosis confirmed by visualization of parasites in tissue samples (spleen, lymph node or bone marrow) on microscopy.

EXCLUSION CRITERIA

Patients with the following will be excluded from the study:

1) Patients who have received any anti-leishmanial drug in the last 6 months.
2) Patients with a negative splenic / lymph node / bone marrow smears.
3) Patients with a clinical contraindication to splenic/lymph node/ bone marrow aspirates.
4) Patients with severe protein and or caloric malnutrition (Kwashiokor or marasmus)
5) Patients with previous hypersensitivity reaction to SSG or aminoglycosides.
6) Patients suffering from a concomitant severe infection such as TB or any other serious underlying disease (cardiac, renal, hepatic) which would preclude evaluation of the patients response to study medication.
7) Patients suffering from other conditions associated with splenomegaly such as schistosomiasis.
8) Patients with previous history of cardiac arrhythmia or an abnormal ECG
9) Patients who are pregnant or lactating.
10) Patients with haemoglobin < 5gm/dl.
11) Patients with WBC $< 1 \times 10^3/mm^3$.
12) Patients with platelets $< 40{,}000/mm^3$.
13) Patients with liver function tests more than three times the normal range
14) Patients with serum creatinine outside the normal range for age and gender
15) Patients with pre-existing clinical hearing loss.

NB

Relevant tests will be done to exclude the above listed conditions.

HIV-status and VCT

All patients will be offered counseling and screening for HIV (voluntary counseling and testing programme (VCT). This may either be done at the same

time as consent is obtained for inclusion in the trial or at a later date according to hospital practice. HIV positive patients will not be excluded from the clinical trial. Subset analysis will be performed to assess any differences in response.

CRITERIA FOR PATIENT WITHDRAWAL

Patients will be considered to have completed the study if they satisfy all entry criteria, complete the course of treatment and attend the 6 month follow-up visit.

Patients will be considered to have withdrawn from the study if they had entered into the study (i.e. gave informed consent and received at least one day's treatment) but did not complete the treatment period and follow up period.

Treatment failure will be defined as no change or an increase in the patient's disease severity i.e. in signs and symptoms of VL, and parasitology, such that the patient is withdrawn from the study and alternative therapy given.

A patient may be withdrawn from the study at any stage if the investigator or the DSMB considers there is a serious risk to the patient from continuation in the protocol. Alternative therapy will be provided to the patient if needed, upon withdrawal from the study.

A patient may withdraw, or be withdrawn, from the study for one of the following reasons:

- Serious adverse events (drug related or not)
- Deviation from protocol (including non-compliance)
- Lost to follow-up
- Termination by the sponsor
- Withdrawal of consent

The reason for termination will be recorded on the CRF. Patients withdrawn from the study will be followed-up at 3 and 6 months for monitoring of adverse events wherever possible. Every effort will be made to follow up withdrawn patients in order to determine the final outcome. This information will be recorded in the CRF and these patients data will be analysed as those who failed to respond to treatment.

Randomization in the Clinical Trial (RCT)

The multi-country study adopted restricted randomization in a three-arm study per country. This approach prevents the potential pitfall/imbalance in study numbers that could have resulted if one used simple random sampling. In order to avoid manipulation of blocks of small sizes, blocks of size 15 will be used in randomization. This approach ensures that the study balances after the 15th patient. In the allocation of the drugs to the patients concealment will be used in order to minimize selection bias. Opaque envelopes will be numbered sequentially and then sealed. This process will be carried out at the coordinating centre at KEMRI.

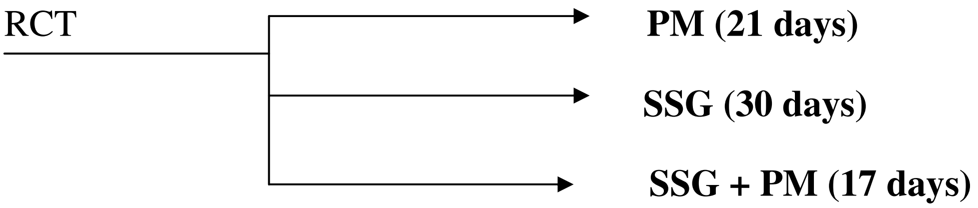

Fig 1: The structure of the RCT

Sample size Determination

The sample size will be used to test the following statistical hypothesis as stipulated earlier:

Hypothesis: The three therapies differ by clinically relevant amounts
Alternative Hypothesis: The three therapies do not differ by clinically relevant amounts

A cure rate of 85% to 95% was used in the sample size calculation, where 85% represents the worst-case scenario and 95% the best outcome. The difference of these two proportions gives the size of the treatment effect sought in the randomized clinical trials.
This means that we are dealing with a dichotomous variable where the patients will be categorized as cured or not cured. A power of 90% and 5% level of significance are also required. The calculated sample size was adjusted for attrition and other covariates such as HIV/AIDS which was observed to be high in some of the participating countries.

Allocation adopted – uniform and equal allocation where $\lambda=n_t/n_c=1$
The sample size $n=r*n_t$ [r is the number of active groups] which is calculated as follows:-

n_t **[per arm]=k* [(p_1(1-p_1) + p_2(1-p_2))/(p_1-p_2)^2]**

Where:
n_t = sample size
$z_{a/2}$ = the corresponding value to the 95% CI
z_β = The corresponding value to power of 90%
p_1 = anticipated cure rate = 95%
p_2= worst case scenario = 85%
$q_1 = 1- p_1$
$q_2 = 1- p_2$
where k= 10.5 for power of 90%

<u>For 90%</u>

n_t >= 10.5 [(0.85(0.15) + 0.95(0.05))/0.01 = 184

The sample size was adjusted to account for attrition rate due to loss to follow up, which is anticipated to be 15%. Thus the minimum sample size becomes n = 217.

HIV/AIDS was likely to be a covariate that could affect the primary variable-cure rate, especially in Ethiopia where the co-infection rate is estimated to be above 20%. This necessitated a further adjustment of the sample size for Ethiopia by 20% in order to be able to attribute the true treatment effect of the test drugs after controlling for HIV/AIDS. Thus the total for the three arms is 695.

The participating teams from Kenya, Ethiopia, Sudan and MSF were asked to specify the number of cases they could conveniently handle given the existing capacity and available infrastructure. This approach was used in absence of any other criteria. On the other hand some of the countries have very high case-load.

Table 1: Sample size per group

Teams	Expected Sample per Group(rounded to complete block of 15)
Kenya	60
Sudan MSF	150
Sudan MOH	225
Ethiopia – Gondar Province	150
Ethiopia-Southern Province	120
Total	705

For further reading on sample size estimation consult (Chan 2003)

TREATMENT

Eligible patients for whom informed consent has been obtained will be randomized to either of the three treatment regimens using a computer generated randomization code provided.

Drug Administration:

SSG will be given IM or IV at a dosage of 20mg/kg/day* for 30 days.
PM will be given IM at a dosage of 15 mg/kg/day for 21 days.
SSG + PM combination: SSG will be given IM or IV at a dosage of 20mg/kg/day* for 17 days and PM at a dosage of 15 mg/kg/day IV/IM for 17days
***The maximum dosage of SSG per day for any patient is 850mg (8.5ml)**

Treatment will be given by the clinicians/nurse at the same time each day and a treatment sheet indicating time of dosing bearing the signature of the attending clinician/nurse will be kept.

Rescue medication

In the event of failure to respond to treatment, clinical deterioration or relapse at any time during the study, rescue treatment consisting of IV Ambisome® (a liposomal formulation of amphotericin B) at a dosage of 3 mg/kg/day for 5 days or according to local Ambisome® rescue protocol, the exact regimen used at each trial site to be documented in the case report form..

Prior and Concomitant Medications

No additional anti-leishmanial therapy will be permitted during the course of the study. If such therapy becomes necessary, the patient will be withdrawn from the study and considered a treatment failure.

Concomitant medication necessary for the health of the patient will be permitted during the course of the study. This will include the concomitant use of drugs such as paracetamol as an analgesic/antipyretic. Details of all concomitant medication taken during the study will be recorded in the CRF with indication, daily dose, route and dates of administration.

In the case of a patient presenting with co-infection, eg. pneumonia or malaria, these infections should be treated first. The patient should be re-assessed for suitability for inclusion in the trial after one week.

EFFICACY ASSESSMENT

Efficacy will be assessed using clinical, haematological, biochemical and parasitological responses.

- Clinical Assessment

The clinical evaluation will involve measuring the spleen size by palpation below the left coastal margin, temperature, blood pressure, body weight on days 0, 7, 14, 21 and end of treatment (18, 22, 31), 3 months and 6 months post treatment.
ECG and audiometry will be done at baseline, day 14, end of treatment and 6 months follow-up, in selected sites.

- Haematological and biochemical assessment

Blood will be analyzed for haemoglobin, WBC, platelets, urea, creatinine, and liver function tests on days 0, 7, 14, 21 and end of treatment (18, 22, 31), 3 months 6 months post-treatment.

- Uninalysis

Urinalysis will be performed on days 0, 7, 14, 21 and end of treatment (18,22,31) 3 months and 6 months post-treatment.

- Parasitological assessment

Parasitological assessment involves aspirating the spleen, lymph node or bone marrow at baseline, end of treatment (18, 22, 31 days depending on treatment arm) on, and at 3 months and 6 months follow up visits for all study patients. In addition, at selected sites aspirates will be cultured. Each patient will have a maximum of four aspirates. Patients who are clinically well with no signs or symptoms of VL and no palpable lymph nodes / spleen at the three months visit do not to have any aspirate at this visit. All patients will have an aspirate at 6 months post treatment.

The source of parasitological specimens should remain unchanged throughout the treatment and follow up periods, unless the spleen / lymph node initially chosen as the source of parasitological specimens becomes unpalpable, in which bone marrow aspirate should be performed.

Since bone marrow, lymph node and splenic aspirates are invasive procedures, these should only be performed at other times if clinically indicated.

PRIMARY EFFICACY ENDPOINT

The primary efficacy variable is parasitological clearance at 6 months post treatment by splenic, lymph node, or bone marrow smear.

SECONDARY EFFICACY ENDPOINT

The secondary efficacy endpoint will be parasitological clearance at the end of treatment (18, 22, 31 days depending on treatment arm – Test of cure (TOC)) and at 3 months post treatment.

SAFETY ASSESSMENTS

During treatment and at follow up, safety will be assessed by means of haematological, urinalysis and biochemical monitoring as above, and by ECG and audiometry at selected sites. In addition, patients will be asked at each visit if they have suffered any side-effects or other unexpected adverse events.

Adverse events

An adverse event will be defined as any noxious, pathological or unintended change in anatomical, physiological or metabolic functions as indicated by physical signs, symptoms and/or laboratory changes occurring in any phase of the clinical study, whether or not they are considered to be associated with the study drug. This includes an exacerbation of pre-existing conditions or events, intercurrent illnesses, drug interaction, or the significant worsening of the disease under investigation that is not recorded elsewhere in the CRF under specific efficacy assessments. Anticipated day to day fluctuations of pre-existing conditions, including the disease under study, that does not represent a clinically significant exacerbation or worsening of the condition, will not to be considered adverse events.

All adverse events occurring after the start of the study (defined as when informed consent was obtained) are to be reported. This is regardless of whether or not they are considered to be drug related. Adverse event (AEs) elicited by the investigator asking the patient or the patient's parent or guardian a non-leading question such as "Do you/has your child felt different in any way since starting the new treatment/the last assessment?" If the response was "Yes", the nature of the event, the date and time (where appropriate) of onset, the duration, maximum intensity (see below) and relationship to treatment are to be established (see below). Details of any changes to the dosage schedule or any corrective treatment are to be recorded on the appropriate pages of the CRF.

Assessment of Intensity/Severity

The assessment of intensity/severity will be based on the investigator's clinical judgment. Maximum intensity/severity will be assigned to one of the following categories.

Mild: An adverse event, which is easily tolerated by the patient, causing minimal discomfort and not interfering with every day activities.

Moderate: An adverse event, which is sufficiently discomforting to interfere with normal everyday activities.

Severe: An adverse event, which prevents normal everyday activities.

Assessment of Causality

The investigator will use clinical judgment to determine the degree of certainty with which adverse event is attributed to drug treatment. Alternative causes, such as natural history of the underlying diseases, concomitant therapy, etc are to be considered taking into account the known pharmacology of the drug, any previous reactions, literature reports and relationship to time of drug ingestion or recurrence on re challenge. Causality will be assessed using the following categories; not related, unlikely, suspected (reasonable possibility) or probable. Patients with adverse events will be followed-up until the event disappears or the condition stabilizes.

Serious Adverse Events

A serious adverse event will be defined as any event which is fatal, life threatening, disabling or incapacitating or results in hospitalization, prolonged hospital stay or is associated with congenital abnormality, cancer or overdose (either accidental or intentional). In addition, any experience which the investigator regards as serious or which suggests any significant hazard, contraindication, side effect or precaution that might be associated with the use of the drug will be reported as a serious event. Any serious adverse event occurring either during the study or within 30 days, or 5 half lives (whichever is longer), of receiving the last dose of study medication, is to be reported by telephone to the study monitor within 24 hours. This will be followed by a full written summary containing relevant hospital case records and autopsy reports where applicable.

As treatment is by parenteral injection, over dosage is not anticipated. However, in the event of over dosage (error of dosage calculation or administration) will be communicated to the study coordinator, Dr. Monique Wasunna, within 24 hours or as soon as possible thereafter. Details of any signs or symptoms and their management will be recorded in the CRF including details of any antidote(s)

administered. As there are no specific antidotes available for the medications to be used in this study, patients will receive all supportive care needed at discretion of the treating physician and after consultation with the study coordinator above.

DATA COLLECTION, STORAGE AND ANALYSIS

Data Management

In order to ensure data quality, a uniform hard copy i.e case report form (CRF) will be designed for use at all the sites. Data will then be sent to the coordinating sitefor data entry. It will be the responsibility of the investigator to ensure that the CRF is correctly completed to avoid unnecessary delays.

The software of choice will be EpiInfo 2003 which has an adequate electronic data capture (EDC) module especially for double entry. The data will be entered using pre-designed screens matching the data collection tool for ease of entry and validation. The entry program will also have in-built checks to minimize entry errors such as minimum and maximum, allowable values, legal values, jumps and values one must fill.

This exercise will be carried out by well-trained data entry personnel who will manage the data under the guidance of the biostatistician at the Centre for Clinical Research Centre of KEMRI.

Analysis

In the analytical approach intention to treat will be used. The aim is to estimate the difference in treatment outcomes for the three arms. The intention to treat analytical method will take care of events such as patent withdrawal from the trial, failure to comply with treatment, change in treatment and lost to follow-up.

There are two basic options:

Analyse final outcome only for those who complied perfectly with each treatment

Analyse data for all subjects in the groups to which they were randomized. This is referred to as 'Intention to treat' analysis or the 'pragmatic approach'.

The second option will be adopted, analysis will be carried out according to the original treatment assignment regardless of adherence to treatment or protocol.

There will be no exclusion of patients or events. Drop-outs will be checked to establish whether they were systematic or non-random. The analysis will, as much as possible stick to the protocol to avoid data dredging as part of post ad hoc analysis.

Note: Specific country analysis will be of little value because it lacks power. The data will have to be pooled for it to achieve the necessary power in the efficacy analysis.

Summary statistical measures to be computed

The summary statistics will include the χ^2 and risk ratios per arm for the overall efficacy comparison. The analysis and interpretation will heavily rely on confidence intervals of the cure rates among the three arms of the RCT. Other estimations to be made will be the calculation of the power finally achieved by the trial. This will be followed by the presentation of the results in terms of basic descriptive statistics and statistical diagrams. Finally multivariate analysis will be carried out to establish the socio-demographic factors associated with the main parameters.

QUALITY CONTROL AND QUALITY ASSURANCE

All study sites and data generated during the study will be regularly monitored by GCP trained clinical monitors. Wherever possible, CRF data will be verified against hospital source data, for example patient notes or laboratory reports, etc.

ETHICAL CONSIDERATIONS

The study protocol together with patient information and consent forms will be submitted to the local scientific and ethics committee and any other regional or national regulatory authorities as required in the three countries, Kenya, Sudan and Ethiopia, before the study starts and any patient receives study medication.

The patients who participate in this study will be hospitalized and under close monitoring. The invasive diagnostic methods used in the study are those used in

normal clinical practice when treating patients with VL. However, the frequency of testing might be increased depending on the patient's response to treatment.

Children will be included in this study because they represent more than 50% of VL cases in this region.

The effective treatment of VL benefits not only the individual patient but also the community by reducing the reservoir of infection for onward transmission by the sandfly vector. The evaluation of new and better treatments for VL, including shorter courses and combinations is anticipated to have a positive effect on development of parasite resistance and will reduce hospitalisation costs. If paromomycin is found to be efficacious and safe, it will be registered for the treatment of VL, providing a new alternative to treatments already available.

Patients will experience some pain while blood is drawn during venepuncture. The amount of blood to be drawn will be 10 mls before treatment and 7 mls at each subsequent visit, with a total of 42 mls (PM alone and a combination of PM and SSG), 49 mls (SSG alone) over the 6 months study period.

SSG has been extensively used in Sudan, Ethiopia and Kenya. Known adverse events include cardiac, muscle, joint and renal toxicity. PM has been used in clinical trials in Sudan and Kenya during the 1990's, and more recently in humanitarian emergency setting in Sudan. Known adverse events of the aminoglycosides include ototoxicity and renal toxicity.

Patients who are found to be HIV positive will be offered anti retroviral treatment at no cost in accordance with national guidelines for treatment.

A Data Safety and Monitoring Board (DSMB) will be set up to regularly review safety data.

Patients will be reimbursed for travel to and from the study site and will not receive any payment for trial participation. Any medication that is required during the trial period will be provided free of charge to the patient.

INSURANCE AND LIABILITY

DNDi is insured to indemnify the collaborating investigator for any injury or harm which occurs during the performance of the trial according to the protocol signed

by the investigator. Furthermore, DNDi will in accordance with the declaration of Helsinki on Ethical principles for medical research involving human subjects, make all reasonable efforts to protect patients from any harm which may occur during the trial, and will wherever possible ensure that any patient that does suffer harm will receive the best possible treatment available in that country to alleviate their suffering.

TIME FRAME

The study is expected to start in October 2004 and will last 12 to 18 months (6-12 months recruitment period plus 6 months follow up)

REFERENCES

Ayele T and Ali A (2004) The distribution of visceral leishmaniasis in Ethiopia. Am. J. Trop Med Hyg., 33, 4, 548 - 552.

Chan Y.H. (2003). Randomised Contolled Trials (RCTs)- Sample size: The magic number? Singapore Med J 44(4): 172-174

Chunge C.N, Owate J, Pamba H. and Donno H.O. (1990). Treatment of visceral Leishmaniasis in Kenya by aminosidine alone or combined with sodium stibogluconate. Trans. Roy. Soc. Trop. Med. & Hyg. 84: 221-225

Desjeux P. 2001 The increase in risk factors for leishmaniasis world wide. *Trans R Soc Trop Med Hyg*. **95**:239-43

Hailu A., Gebre-Michael T., Berhe N and Balkew M. (2004). Leishmaniasis in Ethiopia. In: *The Ecology and Epidemiology of Health and Disease in Ethiopia*. New Edition; Eds, H. Kloos, Berhane Y and Hailemariam D. (in press)

Hassan M, Baat D.B and Hassan K.A (1995).New breakthrough in treatment of visceral leishmaniasis in children.
Journal of the Pakistan Medical Assocaition 45: 155 – 157

Kanyok T.P. Killian A.D., Rodvold K. A and Danziger L.H (1997).
Pharmacokinetics of intramuscularly administered Aminosidine in Healthy Subjects.
Antimicrobial Agents and Chemotherapy 41:982-986

Manual For The Diagnosis and Treatment of Leishmaniasis, Book, PP: 5, (2004) By *Experts in leishmaniasis in Sudan.)*
MSFH Kala azar manual, version March 2003

Schilling R. T and Schaffner C.P. (1961). Differentiation of catenulin-neomycin antibiotics: Identity of catenulin, paromomycin, hydroxymycin and aminosidin.

Antimicrobial Agents and Chemotherapy 4: 275 – 285

Scott J.A.G., Davidson R.N., Moody A.H, Grant H.R., Felmingham D, Scott G.M.S., Olliaro P and Bryceson A.D.M. (1992) .Aminosidine (Paromomycin) in the treatment of leishmaniasis imported into the United Kingdom. Trans. Roy. Soc. Trop. Med. & Hygiene 86: 617-619

Seaman J, Pryce D, Sondorp H.E, Moody A, Bryceson A.D.M. and Davidson R.N. 1993). Epidemic Visceral Leishmaniasis in Sudan: Randomised Trial of aminosidine plus sodium stibogluconate versus sodium stibogluconate alone.
The Journal of Infectious Diseases 168:715 – 20.

Seaman J. Mercer AJ, Sondrop E. (1996) The epidemic of visceral leishmaniasis in western Upper Nile, Southern Sudan: course and impact from 1984 t0 1994.
Int J. Epidemiol, **25**:862-71.

Thakur C.P, Bhowmick S, Dolfi L and Olliaro P. (1995). Aminosidine plus Sodium stibogluconate for the treatment of Indian Kala-azar: a randomized dose-finding clinical trial.
Trans. Roy. Soc. Trop. Med & Hyg 89: 219-233

Thakur C.P., Kanyok T.P., Pandey A.K., Sinha G.P., Zaniewski A.E, Houlihan H.H and Olliaro P. (2000). A prospective randomized, comparative, open label trial of the safety and efficacy of paromomycin (aminosidine) plus Sodium Stibogluconate versus Sodium stibogluconate alone for the treatment of visceral leishmanisis.
Trans.Roy.Sox.Trop.Med & Hygy 94: 429 -431

Thakur C.P., Olliaro P, Gothos Kar S, Bhowmich S, Choudlhury B.K., Prasad S, Kumar M, Verma B.B. (1992) Treatment of Visceral leishmaniasis (Kala-Azar) with Aminosidine (paromomycin) antimonial combinations, a pilot study in Bihar, India.
Trans. Roy. Soc. Trop. Med & Hyg. 386: 615 – 616.

Thakur C.P., Sinha G.P., Pandey A.K, Kumar N, Kumar P, Hassan, Narain S.M. and Roy R. (1998). Do the diminishing efficacy and increasing toxicity of Sodium Stibogluconate in the treatment of Visceral leishmaniasis in

Bihar, India, justify its continued use as a first line drug? An observational study of 80 cases.
Annals of Tropical Medicine and Parasitology 92:561-569

WHO Expert Committee Report (1990). Control of the leishmaniasis. *WHO Tech Rep Ser* 993

WHO Fact Sheet, revised May 2000

Zijlstra E.E, Musa A.M, Khalil EAG, EI Hassan IM, EI- HssanAM(2003). Post Kala-Azar Dermal Leishmaniasis.
Lancet Infect Dis, 3:87-98.

PATIENT INFORMATION AND CONSENT

TITLE: A MULTICENTRE COMPARATIVE TRIAL OF EFFICACY AND SAFETY OF SODIUM STIBOGLUCONATE (SSG) VERSUS PAROMOMYCIN (PM) VERSUS COMBINATION OF SSG AND PM AS THE FIRST LINE TREATMENT FOR VISCERAL LEISHMANIASIS IN ETHIOPIA, KENYA AND SUDAN

**PRINCIPAL INVESTIGATOR(S):**	**ETHIOPIA**	**Dr. Asrat Hailu**
	**KENYA**	**Dr. K.M. Wasunna**
	**SUDAN**	**Dr. Musa Amudawi**
	**SUDAN**	**Dr. Getahun Mengistu**
	**MSFH**	**Dr. Manica Balasegaram**

SPONSOR: Drugs for Neglected Diseases Initiative, Geneva, Switzerland

Introduction

We are studying kala-azar disease, which is common in our countries. The test you have had performed indicates that you have been infected by a parasite called *Leishmania* that causes the illness kala-azar. We are studying new drug treatments for this disease, and would like you to participate in this trial to test a new drug called paromomycin. We wish to compare it with the usual treatment called SSG, and a combination of both drugs used together.

Paromomycin alone and the combination with SSG have been shown to be useful in small studies in some countries but we do not have sufficient evidence of safety and efficacy to enable us to get paromomycin registered in this country.

The study is expected to start in 2004 and will last for 12-18 months. A total of 705 patients will participate.

We would like you to be included in this number, but your participation is voluntary.

Procedures during the trial

Because we do not know which treatment is most effective, you will be allocated to one of the three treatment choices by a process called randomization, which means that the chances of you getting any of the three treatments is the same.

You will be admitted to the ward for the duration of the treatment. This is given as a daily injection into a vein or muscle for up to 30 days, depending on which treatment choice you are allocated.

Known side effects of these drugs include, pain at the injection site, skin rash, nausea and vomiting, diarrhea, feeling tired, ringing in the ears and rarely damage to the heart, kidneys or hearing. During the trial we will regularly assess your progress by means of blood tests, urine tests, heart tracings and hearing tests. A total of 10 mls of blood (two tea spoons) will be drawn at the beginning of the trial and 7 mls (one and a half tea spoons) at each subsequent weekly assessment during treatment and at follow up. We shall also need to repeat the test on your lymph nodes/spleen/bone marrow to make sure that the drugs are killing the kala- azar parasites. These tests are necessary but not without risk.

Occasionally splenic aspiration may result in internal bleeding. This is very unlikely but may occur as a complication in approximately 1 in 1,000 patients. The risks can be minimized in a number of ways. For instance, we will determine any bleeding problem you may have by a blood test. If this test indicates you are at risk of bleeding, we will use lymph node (LN) aspiration or bone marrow (BM) aspiration instead. If it is necessary to do a bone marrow test we will perform it under local anesthesia, to reduce the pain of this procedure. Special precautions will be taken in cases of children. The child must be calm and still during the procedure, so will be held gently onto the bed by a nurse to avoid movement.

Local anaesthetic and mild sedation will be given. In case bleeding occurs we will look after you until you are fully recovered.

On rare occasions there might be failure of treatment using the study drugs. In this case, you will receive treatment with another drug called liposomal amphotericin-B (AmBisome). This drug is known to be very effective and safe. It is not available in Ethiopia, Kenya and Sudan because it is very expensive, however, we shall make this drug available to you, at no cost, to make sure we can cure your disease.

We need to assess the long term effects of the drugs and therefore we shall need you to attend two follow up appointments at 3 months and 6 months after your treatment has finished. For school children, this will mean absence from school on those days.

Benefits

The main benefit of participation in the study is that you will be cured of the disease called kala azar. If the study is successful it means that an alternative shorter treatment will be available for this disease which will benefit your community and may reduce the likelihood of other people getting the disease.

Confidentiality

At the end of the study, we plan to write a report about the results of the study. The reports will not bear any information relating to you personally e.g. your name or identity. We assure you of the confidentiality of such information. Thus, we also need your permission to use the test results for writing a report.

In addition, clinical monitors of the sponsor (DNDi) or the regulatory authorities may wish to inspect your records.

Right to refuse or withdraw

You do not have to take part in this research, your participation is voluntary. If you do not wish to do so and this will not affect your treatment at this centre in any way. You will still have the benefit of treatment for your disease at this centre.

If you do decide to participate and then change your mind later, you may do so, at any time, without losing any of your rights as a patient.

It is also possible that we may decide to withdraw you from the study if we believe it is in your best interests, in which case you will continue to receive the usual treatment for kala-azar until you are better..

The sponsor (DNDi) may also decide to terminate the study. In this event we will continue to treat you until you are better.

In the event that you suffer an injury or illness related to participating in this trial, DNDi will pay all costs relating to treatment of the injury or illness.

You will not receive any money for your participation in the study, however, we will pay your travel expenses to attend the hospital for treatment and hospital follow up visits at 3 and 6 months. In the rare event that you suffer complications due to study treatment, we will do everything possible to ensure you receive the necessary medical care and treatment for this complication.

If you agree to participate in the study, we will ask you to read and sign the consent form.

Do you have any questions?

Patient information for HIV testing

As we have explained to you, you have kala-azar infection and we are treating you with one of the three trial drug treatments. We are now asking you to be tested for another infection. It is a test for HIV infection. We have very important reasons to test you for HIV, which we would like you to understand.

If you are HIV positive you may not respond to treatment and we may need to give you additional treatment. In case you are HIV positive, it will be beneficial for you to know, both for your own well being, and also for your family, friends and other persons living with you.

We advise you to consider being tested. If you wish to be tested, a counselor will hold confidential discussions with you before and after the test. We will inform you of the test results. If you are HIV positive we will treat you for kala-azar first and then treat you for the HIV infection afterwards.

If you fulfill the national criteria for anti-retroviral therapy, we will provide you with anti-retroviral therapy for the duration of the project (18 months) or as required by national guidelines, at no cost to you.

If you do not wish to be tested for HIV, you will still benefit from the treatment for your kala-azar.

There is no obligation for you to accept the HIV test within this study, and if you refuse it, or do not wish to be informed of the results of your test, you will not be deprived of any other medical care that we offer you. You may wish to take time to think about being tested. If you change your mind later, and would like to be tested, we will do this for you at any time during this trial.

Consent Form:

I, the undersigned, confirm that, as I give consent to participate in the study, it is with a clear understanding of the objectives and conditions of the study and with the recognition of my right to withdraw from the study if I change my mind.

I ……………………….. do hereby give consent to Dr …………………………… to include me in the proposed research and the treatment. I have been given the necessary information and understand that there might be some risks involved in the treatment procedures. I have also been assured that I can withdraw my consent at any time without penalty or a loss of benefits. The proposal has been explained to me in the language I understand.

Name of patient : _____________________

Patient's Signature: ____________

Name of Doctor: _________________________

Doctor's Signature:___________________

Date : _________________

Witness: ________________________Date:__________________

CONSENT FOR MINORS (UNDER 18 YRS)

I Mr/Ms ________________________________ being a person aged 18 years and over and being the Parent/Lawful guardian of master/miss______________________

hereby consent to Dr_____________________
to include Master/Miss _____________________________ in the intended research as explained and understood by me.

I have understood the implications, risks and immediate benefits of the tests and treatment to Master/Miss ________________________________.
I accept the tests and treatment to be carried out and the risks attached .

I understand that I have the right to withdraw
Master/Miss ________________________ from the research at any time, for any reason without penalty or harm. In case of withdrawal, I understand that the Physicians will continue to take care of Master/Miss
_________________________________ like any other patient.

All the above conditions have been explained to me in
____
language which I understand

___________________________________ Guardian's full name

___________________________________ Guardian's signature

Date:_____________

___________________________________ Child's full name

___________________________________ Person obtaining consent

___________________________________ Witness

Date:_____________

Consent Form for HIV testing:

I, the undersigned, confirm that, as I give consent to HIV testing, it is with a clear understanding of the objectives of HIV testing in this study,

the availability of counseling services,

the confidentiality of the test results

and in the case that I am positive for HIV, the possibility of receiving anti-retroviral therapy for the duration of the trial (18 months) should I fulfil the criteria set by the national guidelines

I ……………………….. hereby give consent to Dr ___________________

To perform this test.

I have been given the necessary information in a language that I understand.

Name of patient : ______________________

Patient's Signature: ____________

Name of Doctor: __________________________

Doctor's Signature:____________________

Date : __________________

Witness: _________________________Date:___________________

<u>Contact persons (to be customized for each study site)</u>

1. **Name and address of study site investigator**
2. **Name and address of next of kin of study patient**
3. **Name and address of Ethics Committee Chair**

Protocol Amendment 01 for Trial LEAP 0104
Study site; MSF-H treatment centre, Um El Kher, Gedaref State, South Sudan
Date: 29th June 2004

Title:
A multicentre, comparative trial of efficacy and safety of sodium stibogluconate (SSG) versus paromomycin (PM) versus a combination of SSG and PM as first line treatment for visceral leishmaniasis in Ethiopia, Kenya and Sudan.

Reason for amendment:
Due to the rural location of this treatment centre, lack of transport infrastructure, impassability of roads during the rainy season and limited facilities and staff at the study site the following amendments apply to the protocol LEAP 0104:

1. Recruitment period will be limited to November and December only (and January if insufficient suitable patients have been recruited during November and December) to ensure all patients can be followed up for the primary endpoint (six month cure rate) before the onset of the next rainy season.
2. Follow up at 3 months as a secondary efficacy endpoint will be omitted, except in patients where it is anticipated that it will not be possible to obtain follow up at six months eg because of population movements, approaching rainy season or other local factors, when these can be foreseen.
3. Diagnostic and follow up parasitology will be by lymph node only or, in very exceptional circumstances by bone marrow aspirate, if clinically indicated. Splenic aspirates will not be performed due to lack of facilities and surgically trained staff able to deal with the very small risk of uncontrollable haemorrhage occurring following splenic aspirate.
4. Blood testing will be limited to a haemoglobin measurement using a Lovibond or Haemacue haemoglobinometer and VCT for HIV in those patients who consent to this. No renal or liver function blood biochemistry testing will be performed at this study site. Patients with clinical signs of renal or liver failure eg jaundice, will be excluded.
5. No audiometry or ECG testing will be performed at this study site. Patients will be asked if they have suffered any deterioration in hearing ability compared with pre-treatment at the end of treatment and at the follow up visit.

Signatures:

-LEAP 0104 Medical Coordinator, Dr Monique Wasunna, KEMRI

……………………………………………

-LEAP 0104 Biostatistician, Mr Lawrence Muthami, KEMRI

……………………………………………

-LEAP 0104 Principal Investigator for Um El Kher, Dr Manica Balasegaram

……………………………………………

KENYA MEDICAL RESEARCH INSTITUTE

P.O. Box 54840, NAIROBI 00200, Kenya,
Tel: (02) 722541, Fax: (02) 720030, E-mail: kemri-hq@nairobi.mimcom.net Website: www.kemri.org

10th January 2004

CCR/DNDi/45

The Chairman
KEMRI National Ethical Review Committee

RE: 2ND AMMENDMENTS TO PROTOCOL SSC NO. 899 A MULITICENTRE COMPARATIVE TRIAL OF EFFICACY AND SAFETY OF SODIUM SITBOGLUCONATE (SSG) VERSUS PAROMOMYCIN (PM) VERSUS COMBINATION OF SSG AND PM AS THE FIRST LINE TREATMENT FOR VISCERAL LEISHMANIASIS IN ETHIOPIA, KENYA AND SUDAN.

The above protocol was approved by ERC on 14th August 2004 and the first Ammendment received a provisional approval on 5th January 2005.

I would like to make the following amendment.
This protocol is a multicentre Clinical trial in 3 countries Ethiopia, Sudan and Kenya. According to the approved protocol, ECG and Audiometry will be done at baseline, day 14 and at the end of treatment and at 6 months follow up. All countries will do these investigations during those days. We in Kenya would like to amend the frequency of monitoring ECG and Audiometry in order to increase assessment of safety or drugs used as follows:-

ECG and Audiometry to be done at baseline, weekly while on treatment, at the end of treatment, 3 months and at 6 months follow up.

Both investigations are non-invasive. We cannot reflect these amendments on the master protocol but will reflect them on the patient Information Sheet and consent. Both the English and the Kalenjin amended versions are attached and the changes have been bolded.

~~DR. K.M.~~ WASUNNA
ASSISTANT DIRECTOR, KEMRI &
DIRECTOR, CCR

KENYA MEDICAL RESEARCH INSTITUTE

P.O. Box 54840-00200 NAIROBI, Kenya
Tel: (254) (020) 2722541, 2713349, 0722-205901, 0733-400003, Fax (254) (020) 2720030,
E-mail: kemri-hq@nairobi.mimcom.net; director@kemri.org Website: www.kemri.org

November 6th 2006

The Chairman
KEMRI/National Ethical Review Committee

Dear Sir,

RE: SSC Protocol 899 (Amended) – **A multicentre comparative trial of efficacy and safety of Sodium stibogluconate (SSG) versus Paramomycin (PM) versus combination of SSG and PM as the first line treatment for visceral Leishmaniasis in Ethiopia, Kenya and Sudan,** by KM Wasunna *et al*

Reference to the above protocol, I would like to make the following amendments:

- Include Dr. George Kirigi and Dr. Lilian Apadet as Investigator's.
- Delete the phrase and will last 12 to 18 months (6 – 12 months recruitment period plus 6 months follow up) and insert and will last until the expected sample size is attained. (Page 31)
- Delete the phrase the test you have had performed indicates that you have been and insert the signs and symptoms you have suggest that you may be infected (Page 35).
- Change the contact details for the Principal Investigator, Dr. Monique Wasunna, to include the following telephone number (+254 20 2730076), fax number (+254 20 2733031) and new email address: africa@dndi.org
- To change the email address for the KEMRI/National Ethical Review Committee Chairperson to: lawtechs@rachieradvs.co.ke

The changes are bolded in the attached protocol.

Sincerely

Dr. KM Wasunna
Project Principal Investigator

DNDi
Drugs for Neglected Diseases *initiative*

4th May 2005

CCR/DNDI/45

The Chairman,
KEMRI/National Ethical Review Committee

RE: SSC PROTOCOL NO. 899 A MULTICENTRE COMPARATIVE TRIAL OF EFFICACY AND SAFETY OF SODIUM STIBOGLUCONATE (SSG) VERSUS PAROMOMYCIN (PM) VERSUS COMBINATION OF SSG AND PM AS THE FIRST LINE TREATMENT FOR VISCERAL LEISHMANIASIS IN ETHIOPIA, KENYA AND SUDAN

The above named protocol was approved by ERC on 14th August 2004 and subsequent amendments 1st and 2nd were approved on 15th February 2005 respectively. We are submitting the 3rd amendment which is a consent form for adults who will undergo HIV testing during the study period. When we initially submitted the protocol we did not realize that this page was missing and was therefore not covered in the initial approval. We are requesting you to look at this form (**BOLDED**) and approve it before we can use it in our study.

Thanks.

Sincerely,

DR. K.M. WASUNNA
P.I. LEAP PROJECT

Encls. 2

AFRICA LIAISON OFFICE
P. O. Box 20778-00202 . Nairobi. Kenya
Tel: +254 (0)20 273 00 76. Fax: +254 (0)20 273 30 31
E-mail: AfricaDNDi@nairobi.mimcom.net

HEADQUARTERS
1 Place St Gervais. 1201 Geneva. Switzerland
Tel: +41 (0) 22 906 92 30. Fax: +41 (0) 22 906 92 31
www.dndi.org

FINAL VERSION LEAP 0104 31 JULY 2004 CONFIDENTIAL

PATIENT INFORMATION AND CONSENT (KENYA)

TITLE: A MULTICENTRE COMPARATIVE TRIAL OF EFFICACY AND SAFETY OF SODIUM STIBOGLUCONATE (SSG) VERSUS PAROMOMYCIN (PM) VERSUS COMBINATION OF SSG AND PM AS THE FIRST LINE TREATMENT FOR VISCERAL LEISHMANIASIS IN ETHIOPIA, KENYA AND SUDAN

PRINCIPAL INVESTIGATOR(S):

**ETHIOPIA**	**Dr. Asrat Hailu**
**KENYA**	**Dr. K.M. Wasunna**
**SUDAN**	**Dr. Musa Amudawi**
**ETHIOPIA**	**Dr. Getahun Mengistu**
**MSFH**	**Dr. Manica Balasegaram**

SPONSOR: Drugs for Neglected Diseases Initiative, Geneva, Switzerland

Introduction

We are studying kala-azar disease, which is common in our countries. The test you have had performed indicates that you have been infected by a parasite called *Leishmania* that causes the illness kala-azar. We are studying new drug treatments for this disease, and would like you to participate in this trial to test a new drug called paromomycin. We wish to compare it with the usual treatment called SSG, and a combination of both drugs used together.

Paromomycin alone and the combination with SSG have been shown to be useful in small studies in some countries but we do not have sufficient evidence of safety and efficacy to enable us to get paromomycin registered in this country.

The study is expected to start in 2004 and will last for 12-18 months. A total of 705 patients will participate.

We would like you to be included in this number, but your participation is voluntary.

Procedures during the trial

Because we do not know which treatment is most effective, you will be allocated to one of the three treatment choices by a process called randomization, which means that the chances of you getting any of the three treatments is the same.

You will be admitted to the ward for the duration of the treatment. This is given as a daily injection into a vein or muscle for up to 30 days, depending on which treatment choice you are allocated.

Known side effects of these drugs include, pain at the injection site, skin rash, nausea and vomiting, diarrhea, feeling tired, ringing in the ears and rarely damage to the heart, kidneys or hearing. During the trial we will regularly assess your progress by means of blood tests, urine tests, heart tracings and hearing tests. We shall do the heart tracings and hearing tests at recruitment, weekly while on treatment, at the end of treatment, 3 months and at 6 months follow up. The additional testing times do not carry any risks to you. A total of 10 mls of blood (two tea spoons) will be drawn at the beginning of the trial and 7 mls (one and a half tea spoons) at each subsequent weekly assessment during treatment and at follow up. We shall also need to repeat the test on your lymph nodes/spleen/bone marrow to make sure that the drugs are killing the kala- azar parasites. These tests are necessary but not without risk.

If any blood samples are left after performing the investigations listed above, we request your permission to store these samples for future research evaluating newer diagnostic tests or any other relevant research work. The samples will not have your name only the study number and date. We will seek permission from KEMRI Scientific Steering Committee and Ethics Committee before any of your blood sample can be used. Do you agree?

Occasionally splenic aspiration may result in internal bleeding. This is very unlikely but may occur as a complication in approximately 1 in 1,000 patients. The risks can be minimized in a number of ways. For instance, we will determine any bleeding problem you may have by a blood test. If this test indicates you are at risk of bleeding, we will use lymph node (LN) aspiration or bone marrow (BM) aspiration instead. If it is necessary to do a bone marrow test we will perform it under local anesthesia, to reduce the pain of this procedure. Special precautions will be taken in cases of children. The child must be calm and still during the procedure, so will be held gently onto the bed by a nurse to avoid movement. Local anaesthetic and mild sedation will be given. In case bleeding occurs we will look after you until you are fully recovered.

On rare occasions there might be failure of treatment using the study drugs. In this case, you will receive treatment with another drug called liposomal amphotericin-B (AmBisome). This drug is known to be very effective and safe. It is not available in Ethiopia, Kenya and Sudan because it is very expensive, however, we shall make this drug available to you, at no cost, to make sure we can cure your disease.

We need to assess the long term effects of the drugs and therefore we shall need you to attend two follow up appointments at 3 months and 6 months after your treatment has finished. For school children, this will mean absence from school on those days.

Benefits

The main benefit of participation in the study is that you will be cured of the disease called kala azar. If the study is successful it means that an alternative shorter treatment will be available for this disease which will benefit your community and may reduce the likelihood of other people getting the disease.

Confidentiality

At the end of the study, we plan to write a report about the results of the study. The reports will not bear any information relating to you personally e.g. your name or identity. We assure you of the confidentiality of such information. Thus, we also need your permission to use the test results for writing a report.

In addition, clinical monitors of the sponsor (DNDi) or the regulatory authorities may wish to inspect your records.

Right to refuse or withdraw

You do not have to take part in this research, your participation is voluntary. If you do not wish to do so and this will not affect your treatment at this centre in any way. You will still have the benefit of treatment for your disease at this centre.

If you do decide to participate and then change your mind later, you may do so, at any time, without losing any of your rights as a patient.

It is also possible that we may decide to withdraw you from the study if we believe it is in your best interests, in which case you will continue to receive the usual treatment for kala-azar until you are better.

The sponsor (DNDi) may also decide to terminate the study. In this event we will continue to treat you until you are better.

If you fulfill the national criteria for anti-retroviral therapy, we will provide you with anti-retroviral therapy for the duration of the project (18 months) or as required by national guidelines, at no cost to you.

If you do not wish to be tested for HIV, you will still benefit from the treatment for your kala-azar.

There is no obligation for you to accept the HIV test within this study, and if you refuse it, or do not wish to be informed of the results of your test, you will not be deprived of any other medical care that we offer you. You may wish to take time to think about being tested. If you change your mind later, and would like to be tested, we will do this for you at any time during this trial.

Adults Consent Form for HIV testing

I ....give consent to HIV testing, with a clear understanding of the objectives of HIV testing in this study is for data analysis and that HIV results will not disqualify me from participating in the study. Counseling services are available and the test results are confidential and in case I am HIV positive, the possibility of receiving anti-retroviral therapy for the duration of the trial (18 months) should I fulfill the criteria set by the national guidelines. I also understand that all the above conditions have been explained to me in ______________________ language, which I understand.

Name of patient : ____________________

Patient's Signature: ____________________

Doctor's Signature: ____________________

Name of Doctor: ________________________

Date : ________________

Witness: ______________________**Date:**_________________

Contact persons

1. Dr. Monique K. Wasunna
Principal Investigator
Kenya Medical Research Institute
Centre for Clinical Research
P.O. Box 20778 00202
Nairobi, Kenya
Tel: Direct line 254-20-2726781 or 254-20-2726460
Fax: 254-20-2720030
email: AfricaDNDi@nairobi.mimcom.net

2. **Name and address of next of kin of study patient**

3. PROF. S.K. SINEI, CHAIRMAN
KEMRI/NATIONAL ETHICAL REVIEW COMMITTEE
P.O. Box 54840, NAIROBI.
Telephone 722541 Ext. 3218
email: JAndere@NAIROBI.MIMCOM.NET

Protocol Amendment on dosage and pharmacokinetic evaluation (PK) for paromomycin monotherapy arm of trial LEAP 0104 following preliminary analysis of efficacy data presented to a meeting of the Principal Investigators held at the Kenyan Medical Research Institute (KEMRI) Nairobi, Kenya on 20th and 21st June 2005

Title: A multicentre, randomised, comparative trial of efficacy and safety of sodium stibogluconate (SSG) versus paromomycin (PM) versus a combination of SSG and PM as first line treatment for visceral leishmaniasis (VL) in Ethiopia, Kenya and Sudan.

Reason for amendment

Initial results from the trial LEAP 0104 which commenced in November 2004, from 2 trial sites in Sudan (n = 90 Um el Kher, and n = 45 Kassab), indicate that paromomycin at a dosage of 15mg/kg/day for 21 days is less effective, for the treatment of acute, symptomatic treatment of visceral leishmaniasis, based on parasitological test of cure (TOC) at the end of treatment (day 22), by lymph node aspiration, in accordance with national VL policy in Sudan. **Table. 1.** This dosage may, however, be effective in Kenya (based on a small sample size, n=32)

Explanation for table 1:

Table 1: Complete Parasite Clearance by site and treatment

	Combination	PM	SSG	P-Value
**Um El Kher**	25 / 30 (83.3)	10 / 30 (33.3)	26 / 30 (86.7)	< 0.001
**Kassab**	12 / 15 (80.0)	9 / 15 (60.0)	14 / 15 (93.3)	0.113
**Kenya**	10 / 11 (90.9)	11 / 11 (100)	10 / 10 (100)	1.00
**P-Value***	.981	< 0.001	0. 571	

P-value from Fisher's exact test

* Column one shows that there were no significant inter-site differences in response to combination (p=0.981) and SSG (p=0.571)
Column two shows that there were significant differences in response to PM at the different sites (p < 0.001).

The majority of cases of VL in the disease endemic area of the Horn of Africa occur in Sudan, therefore it is considered essential to find a dose which is effective in Sudan if paromomycin is to be a useful alternative therapy for visceral leishmaniasis in this region.

No efficacy data are yet available from Ethiopia as final approval was received from the relevant Ethics and Regulatory agencies in that country only in May 2005 and recruitment only commenced in mid-June '05.

Safety and tolerability of the test drug paromomycin appears to be acceptable based on the data available from adverse and serious adverse event reports **Tables 2a and 2b.**

Table 2a: Adverse and serious adverse event reports

	1 GIT	2 Skin	3 RTI	4 Musculo skeletal	5 ENT	6 EYES	7 Systemic	8 Infection	9 Kidney	10 Liver	11 Heart
**Combined** **1 Not Related**	2	2	1		1	1	1	2	4		
**2 Unlikely**	1	2	1		2	1	2				
**3 Possible**			2	2			1			1	
**4 Probable**				5	1					3	
**Total**	**3**	**4**	**4**	**7**	**4**	**2**	**4**	**2**	**4**	**4**	
**PM** **1 Not Related**	6	3	3		4		2	8	4		
**2 Unlikely**	1	1		1	1		3				
**3 Possible**		1		4			1		2		
**4 Probable**							2			1	
**Total**	**7**	**5**	**3**	**5**	**5**	**0**	**8**	**8**	**6**	**1**	
**SSG** **1 Not Related**	2	3	9	2	9	3	2	10	2		
**2 Unlikely**	1	1	1	1	2				2		
**3 Possible**	1	1		1	3		1				
**4 Probable**		2		1			2		1		2
**Total**	**4**	**7**	**10**	**5**	**14**	**3**	**5**	**10**	**5**	**0**	**2**

Table 2b: SAEs

	1 GIT	2 Skin	3 RTI	4 Musculo skeletal	5 ENT	6 EYES	7 Systemic	8 Infection	9 Kidney	10 Liver	11 Heart
**Combined** **1 Not related**											
**2 Unlikely**											
**3 Possible**										1	
**4 Probable**											1
**Total**											
**PM** **1 Not Related**					1						
**2 Unlikely**											
**3 Possible**											
**4 Probable**											
**Total**											
**SSG** **1 Not Related**					1						
**2 Unlikely**											
**3 Possible**											
**4 Probable**									1		
**Total**					**2**				**1**	**1**	**1**

These results are in stark contrast to those obtained in a recent study of an apparently similar patient population with VL in India. (Shyam Sundar personal communication).

Table 3. Efficacy Results of Trial VLPM01
A randomised controlled trial of Paromomycin (PM) versus amphothericin B in patients with visceral leishmaniasis in Bihar, India. *(Ref. 8)*

	**PM** N=500	**Amphotericin B** N=116
**Initial Cure rate TOC at 4 weeks**	99%	98.8%
**Final Cure at 6 Months**	94.6%	98.8%

Only limited PK data in healthy volunteers are available, Kanyok et al 1997,**Table 4**.

Table 4. Pharmacokinetics of Paromomycin in Health Volunteers (Kanyok et al 1997.)

**RESULTS**		
	**12 mg / kg (N = 8)**	**15 mg / kg (N = 7)**
Cmax (µg/mL)	21.6	23.4
Tmax (h)	1.19	1.51
T ½ (h)	2.21	2.64
AUC	86.3	104.5
Renal Clearance	79.6	76.1
Recovery in time (24h)	67.8%	60.1%
Analytical technique	HPLC	

No data are yet available in patients from the study in India. It is therefore considered to be essential to include PK assessment in a limited number of patients, during the assessment of two further PM dosing schedules in Sudan. See appendix 1.

Previous studies in patients with VL over the last 15 years in both Africa (Kenya and Sudan) and in India have indicated that a dosage of 15mg/kg can be expected to be both effective and well tolerated. **Table 5** (-redrawn and with additional detail from Table 5 of the protocol of 31st July 2004.)

Table 5. Summary of clinical studies using PM

Drug and dosage schedules	Treatment Outcome TOC	Treatment Outcome DC	Statistic	References / Comments
**Combination therapy versus PM or SSG as a single-agent Tx**				Ref: [1]
G1 = PM14-16 mg./kg/day 20days SSG20mg./kg/day 20days; (n = 23)	100%	87.0%	P<0.05 [G1 – G2] P<0.01 [G1 – G3]	Clinical cure rate 100% in each group at end of treatment. Epistaxis associated with SSG
G2= PM14-16 mg./kg/day 20days; (n = 19)	84.2%	79.0%	P<0.01 [G2 – G3]	
G3 =SSG20mg./kg/day 20days; [Standard Tx] (n = 11)	90.9%	54.5%	-	
**Combination therapy versus Standard SSG Tx**				Ref: [2]
G1 = PM 15 mg./kg/day 17days, and SSG 20mg./kg/day 17days; (n = 61)	95.0% at 17 days	n.d.	P=0.039	Clinical cure rate 100% in both groups
G2 = SSG 20mg./kg/day; 30 days; (n = 60)	81.0% at 17 days	n.d.		
**Combination therapy versus Standard SSG Tx**				Ref: [2]
G1 = PM 15 mg./kg/day 17days, and SSG 20mg./kg/day 17days; (n = 61)	95.0% at 30 days	n.d.	n.s.	
G2 = SSG 20mg./kg/day; 30 days; [Standard Tx] (n = 60)	93.4% at 30 days	n.d		

Table 5. Summary of clinical studies using PM (table cont'd)

Drug and dosage schedules	Treatment Outcome TOC	DC	Statistic	References / Comments
PM 12mg + SSG 20mg<br>20 days<br>(n=24)	82%	82%	n.a.	Ref: [3]<br>2 deaths on Tx<br>- splenic rupture<br>- renal failure
G1a = PM 12mg + SSG 20mg<br>(n=32) 20 days<br>G1b = PM 12mg + SSG 10mg<br>(n= 32) 20 days<br>G1c = PM 12mg + SSG 5mg<br>(n=32) 20 days<br><br>G2a = PM 6mg + SSG 20mg<br>(n=13) 20 days<br>G2b = PM 6mg + SSG 10mg<br>(n=12) 20 days<br>G2c = PM 6mg + SSG 5mg<br>(n=13) 20 days	88%<br><br>71%<br><br>72%<br><br><br>69%<br><br>50%<br><br>46%	-<br><br>[80%]<br><br>-<br><br><br>-<br><br>-<br><br>-	n.a.	Ref: [4]<br><br>Only 50% attended FU.<br>5 relapses<br>8 slow responders<br><br>PM 6mg dosage groups stopped prematurely due to inefficacy<br><br>Safety:<br>- No renal toxicity<br>- Increase in liver enzymes common<br>- ECG normal<br>- Audiometry: data inadequate
**PM single agent therapy versus Standard SSG Tx**<br>G1 = PM 12 mg./kg/day<br>21days; (n = 30)<br><br>G2 = PM 16 mg./kg/day<br>21days; (n =30)<br><br>G3 = PM 20 mg./kg/day<br>21days; (n = 30)<br><br>G4 = SSG 20mg./kg/day<br>28days[Standard Tx]<br>(n = 30)	93.3%<br><br><br>100%<br><br><br>100%<br><br><br>73.3%	77%<br><br><br>93%<br><br><br>97%<br><br><br>63%	P = 0.26 [G1 – G4]<br><br><br>P<0.005 [G2 – G4]<br><br><br>P<0.005 [G3 – G4]<br><br><br>-	Ref: [5]<br><br>All treatments well tolerated<br>- No renal toxicity<br>- No ototoxicity<br>- No clinically relevant differences in laboratory values

Table 5. Summary of clinical studies using PM (table cont'd)

Drug and dosage schedules	Treatment Outcome TOC	DC	Statistic	References / Comments
**Combination therapy, and PM Dose finding**				
G1 = PM12 mg./kg/day 21days SSG20mg./kg/day 21days; (n = 52)	94.2%	92.3%	P<0.001 [G1 – G3]	Ref: [6]<br>1 case of myocarditis on SSG.<br>Audiometry data inadequate for analysis.<br>Lab values – no significant changes from baseline values.
G2 = PM18 mg./kg/day 21days SSG20mg./kg/day 21days; (n = 48)	95.8%	93.8%	P<0.001 [G2 – G3]	
G3 = SSG20mg./kg/day 30days; [Standard Tx] (n = 50)	55.1%	53.1%	-	
**PM Single-agent Tx (Dose finding) versus standard Tx**				
G1 = PM 12 mg./kg/day 21days; (n = 30)	100%	90.0%	P< 0.05 for G1 – G4 G2 – G4 G3 – G4	Ref: [7]<br>No important AEs reported.<br>No renal toxicity observed.<br>ECG and audiometry data inadequate for analysis.
G2 = PM 16 mg./kg/day 21days; (n = 30)	100%	88.9%		
G3 = PM 20 mg./kg/day 21days; (n = 30)	100%	86.2%		
G4 = SSG 20mg./kg/day 28days [Standard Tx] (n = 30)	73.3%	69.0%		

Abbreviations
TOC = Test of Cure at end of treatment
DC = Definitive Cure at 6 month follow up
n.s. = Not Significant
n.a. = Not Available
n.d. = Not Done

The only study from Africa of paromomycin monotherapy; Chunge et al which reported a TOC of 100% and a definitive cure (DC) at six months of 87% was performed 15 years ago in Kenya. Seaman et al, in Sudan in 1993 studied a combination of paromomycin and SSG and obtained a 95% TOC at day 30.
Studies in India from Thakur et al and Jha et al have consistently reported high test of cure with paromomycin monotherapy at doses ranging from 12 -20mg/kg/day but doses of 16-20mg/kg have been required to deliver definitive cure rates at six months of 90%.

Figure 1. Dose response for paromomycin – Phase II studies.

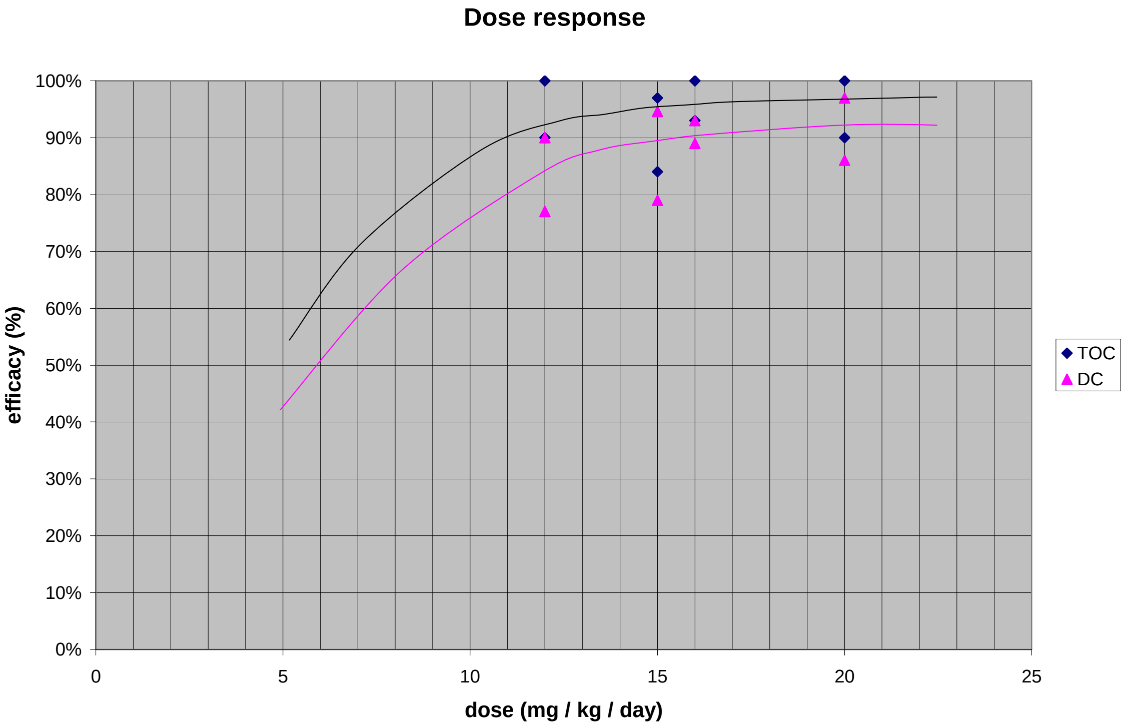

Tolerability and safety have been acceptable in all studies performed so far, using intramuscular injection as the mode of drug administration, with no significant renal toxicity. Only 3 cases of ototoxicity, (<1%) and all reversible, were seen in the large phase III trial from India of 500 patients (Sundar et al personal communication)
In the 20mg/kg studies of Jha et al 1995 and Thakur et al 2000, Jha reported 2 cases of ototoxicity, 1 reversible and one irreversible and Thakur had inadequate data for assessment of ototoxicity.
In contrast administration by **intravenous injection** in the 14-16mg dose range Scott et al 1992 in UK, triggered autotoxicity in 3 of 7 patients in a small case series of imported cases of VL in returned travellers (Table 5).

Based on the evidence above the following protocol amendment is proposed to further elucidate the failure of paromomycin in Sudan.

Group A: Paromomycin 20 mg/kg/day for a total treatment duration of 21 days
Group B: Paromomycin 15mg/kg/day for a treatment duration of 28 days

A total of 42 patients (21 in each treatment arm) will be allocated to receive one of two treatment regimens using a computer-generated randomisation list
The first six patients in each treatment group above, with a body weight of 30kg or more, will have additional venous blood and urine sampling for pharmacokinetic evaluation as outlined in **Appendix 1**.
Inclusion and exclusion criteria are otherwise unchanged from those in the original protocol. (pages 18-19). An amended consent form will be used **Appendix 2.**

Patients who are symptomatic and HIV positive *will be excluded from participation in this protocol amendment but will be offered treatment with SSG and rescue with Ambisome in the event of treatment failure.*

Efficacy The two treatment regimens A and B above will be assessed for efficacy in the 42 patients on the basis of parasitological test of cure following **bone marrow aspiration on day 29 or 30**, this test being acknowledged to be more sensitive but also more invasive and potentially more painful than lymph node aspiration.
Note: Splenic aspiration for parasitological assessment of VL is not allowed in Sudan.

Rescue. Patients who fail to respond to treatment will be given rescue therapy with AmBisome as in the original protocol (Page 24)

Safety will be assessed using the same weekly assessment tests as in the original protocol LEAP 0104 of 31st July 2004 (page 26) -all patients will have ECG and audiometry assessments!

It is expected that following completion of this 42 patient amendment an effective dosing regimen for paromomycin monotherapy in Sudan will have been identified.
This regimen will then be substituted for the current paromomycin only treatment arm, in the 3 arm comparison of the original protocol. Sample size may need to be re-calculated in the light of this new information when it becomes available. A further amendment will be submitted to Ethics Committees and Regulatory Authorities.

Other trial sites
During the period of recruitment and treatment for the 42 patients above at Kassab, Sudan, the trial will be put on hold at KEMRI, Nairobi, Kenya, but recruitment will continue in Ethiopia to n=45 at each trial site (Gondar and Arba Minch hospitals) to obtain contemporaneous information of efficacy and safety of paromomycin at a dosage of 15mg/kg/day for 21 days, as it cannot be assumed that patient response in Ethiopia will match either that in Sudan or Kenya.
The trial site at Um el Kher has been closed by Medecins sans Frontieres (MSF) for operational reasons unrelated to this trial, and will not be available for future patient recruitment to the trial.

Trial site key personnel and monitor update as at June 2005 (can be found in Appendix 3):

References

1. Chunge, C.N., et al., *Treatment of visceral leishmaniasis in Kenya by aminosidine alone or combined with sodium stibogluconate.* Trans R Soc Trop Med Hyg, 1990. 84(2): p. 221-5.
2. Seaman, J., et al., *Epidemic visceral leishmaniasis in Sudan: a randomized trial of aminosidine plus sodium stibogluconate versus sodium stibogluconate alone.* J Infect Dis, 1993. 168(3): p. 715-20.
3. Thakur, C.P., et al., *Treatment of visceral leishmaniasis (kala-azar) with aminosidine (= paromomycin)-antimonial combinations, a pilot study in Bihar, India.* Trans R Soc Trop Med Hyg, 1992. 86(6): p. 615-6.
4. Thakur, C.P., et al., *Aminosidine plus sodium stibogluconate for the treatment of Indian kala-azar: a randomized dose-finding clinical trial.* Trans R Soc Trop Med Hyg, 1995. 89(2): p. 219-23.
5. Jha, T.K., et al., *Randomised controlled trial of aminosidine (paromomycin) v sodium stibogluconate for treating visceral leishmaniasis in North Bihar, India.* Bmj, 1998. 316(7139): p. 1200-5.
6. Thakur, C.P., et al., *A prospective randomized, comparative, open-label trial of the safety and efficacy of paromomycin (aminosidine) plus sodium stibogluconate versus sodium stibogluconate alone for the treatment of visceral leishmaniasis.* Trans R Soc Trop Med Hyg, 2000. 94(4): p. 429-31.
7. Thakur, C.P., et al., *Treatment of visceral leishmaniasis with injectable paromomycin (aminosidine). An open-label randomized phase-II clinical study.* Trans R Soc Trop Med Hyg, 2000. 94(4): p. 432-3.
8. Sundar, S., *Randomised, comparative trial of paromomycin versus amphotericin B in Bihar, India.* Personal communication, 2005.
9. Scott, J.A., et al., *Aminosidine (paromomycin) in the treatment of leishmaniasis imported into the United Kingdom.* Trans R Soc Trop Med Hyg, 1992. 86(6): p. 617-9.
10. Kanyok, T.P., et al., *Pharmacokinetics of intramuscularly administered aminosidine in healthy subjects.* Antimicrob Agents Chemother, 1997. 41(5): p. 982-6.

**Principal Investigators**	**SIGNATURE**	**DATE**
Dr Monique Wasunna<br>(Trial Medical Coordinator)	……………………	
Prof. Asrat Hailu	……………………	
Dr. Getahun Mengistu	……………………	
Dr. Musa A Mudawi	……………………	
Dr. Manica Balasegaram	……………………	
**Trial Manager**<br>Dr Juma Rashid	……………………	
**Trial Statistician**<br>Mr. Lawrence Muthami	……………………	
**Project Manager**<br>Dr Catherine Royce	……………………	

Appendix 1

Pharmacokinetic sampling schedule for patients with a body weight of 30kg or more who consent to participate in the PK part of the study.

Venous sampling for PK patients in group A (n=12)

Day 1 of treatment:
At time 0, 0.25, 0.5, 1.0, 2, 4, 6, 8, 12, and 24 hours after dosing

Day 14 of treatment;
At time 0 before the day 14 dose 0.25, 0.5, 1.0, 2, 4, 6, 8, 12, and 24 hours post dosing.

Venous sampling for patients in group B (n=12)

Day 1 of treatment:
At time 0, 0.25, 0.5, 1.0, 2, 4, 6, 8, 12, and 24 hours after dosing

Day 26 of treatment;

At time 0 before the day 26 dose 0.25, 0.5, 1.0, 2, 4, 6, 8, 12, and 24 hours post dosing.

5 ml of blood at each sampling point will be taken to ensure sufficient plasma is obtained for duplicate test analysis.
Total blood volume taken for PK on each sampling day is 50ml.

Urine sampling

Both groups A and B will also have 24 hour urine collections on PK venous sampling days, collected in aliquots 0-2h, 2-4h, 4-6h, 6-8h, 8-12 and 12-24h.

Safety assessments

Safety assessments (blood biochemistry, haematology, urinalysis, ECG and audiometry,) will be performed as in the original LEAP 0104 protocol before treatment, on day 7, 14 for both groups and additionally on day 22 for group A and day 26 for group B so that direct correlations can be made with the PK data.

Appendix 2

Consent form for Patients participating in PK sampling.

Appendix 3

Site Investigators:

Ethiopia:

Gondar:
Dr Nurelign Gashu (Site PI)
Dr Asfaweseen
Dr Sisay Yifru

Abar Minch:
Dr Samson Tesfaye (Site PI)
Dr Gabriell Teklu

Kenya:
Dr M Wasunna
Dr J R Rashid
Dr Jane Mbui
Dr F Kirui

Sudan:
Dr. Ahmed Musa Mudawi
Prof. E A G Khalil
Dr. Osama M ahmed

Monitors:
Dr Shibru Berhanu
Dr Hilda O’hara

Protocol Amendment- 30th June 2006.

Increased dosage for paromomycin monotherapy arm of trial LEAP 0104 following analysis of efficacy, safety and PK data presented to a meeting of the Principal Investigators held at the Kenyan Medical Research Institute (KEMRI) Nairobi, Kenya on 8th and 9th June 2006

Protocol Title: A multicentre, randomised, comparative trial of efficacy and safety of sodium stibogluconate (SSG) versus paromomycin (PM) versus a combination of SSG and PM as first line treatment for visceral leishmaniasis (VL) in Ethiopia, Kenya and Sudan.

Reason for amendment

Initial results from the trial LEAP 0104 which commenced in November 2004, from 2 trial sites in Sudan (n = 90 Um el Kher, and n = 45 Kassab), indicated that paromomycin at a dosage of 15mg/kg/day for 21 days was inadequate for the treatment of acute, symptomatic treatment of visceral leishmaniasis, based on parasitological test of cure (TOC) at the end of treatment (day 22), by lymph node aspiration, in accordance with national VL policy in Sudan. **Table. 1.** at that time, it was thought to be effective in Kenya (based on a small sample size, n=32)

Table 1: Complete Parasite Clearance by site and treatment

	**Combination**	**PM**	**SSG**	**P-Value**
**Um El Kher**	25 / 30 (83.3)	10 / 30 (33.3)	26 / 30 (86.7)	< 0.001
**Kassab**	12 / 15 (80.0)	9 / 15 (60.0)	14 / 15 (93.3)	0.113
**Kenya**	10 / 11 (90.9)	11 / 11 (100)	10 / 10 (100)	1.00
**P-Value***	.981	< 0.001	0. 571	

P-value from Fisher's exact test

Explanation for table 1:

Column one shows that there were no significant inter-site differences in response to combination (p=0.981) and SSG (p=0.571)

Column two shows that there were significant differences in response to PM at the different sites (p < 0.001).

The majority of cases of VL in the disease endemic area of the Horn of Africa occur in Sudan, therefore it was considered essential to find a dose which would be effective in Sudan if paromomycin was to be a useful alternative therapy for visceral leishmaniasis in this region.

Based on the evidence above the following protocol amendment was proposed to further elucidate the failure of paromomycin in Sudan.

Group A: Paromomycin 20 mg/kg/day for a total treatment duration of 21 days
Group B: Paromomycin 15mg/kg/day for a treatment duration of 28 days

Between September 2005 and March 2006 a total of 42 patients (21 in each treatment arm) were allocated to receive one of two treatment regimens using a computer-generated randomisation list. Inclusion and exclusion criteria were otherwise unchanged from those in the original protocol. (pages 18-19) Results are shown in Table 2.

Table 2

Results

		End of Treatment (TOC)		3 months			
**Regimen**	**Rec-ruited**	**Parasite – ve**	**Parasite + ve**	**Parasite – ve & CRF received**	**No clinical symptoms**	**Relapse**	**Failure + Relapse**
**15 mg for 28 days**	**21**	**19**	**2**	**12**	**9**	**3**	**5 / 21 (23.8%)**
**20 mgs for 21 days**	**21**	**18**	**3**	**12**	**10**	**2**	**5 / 21 (23.8%)**
**Total**			**5**			**5**	

There was no difference in efficacy either at end of treatment or at 3 month follow up, with an overall efficacy rate of 76% for both groups.

No serous adverse events were reported and there were no other signs of increased toxicity with either the increased daily dose or duration of treatment.

In particular there was no evidence of renal toxicity or ototoxicity. No patients experienced clinical hearing loss.

It was planned that the first six patients in each treatment group above, with a body weight of 30kg or more, would have additional venous blood and urine sampling for pharmacokinetic evaluation.
A total of 10 patients consented to participate in the PK sampling, 6 in the 15mg/kg/day arm and 4 in the 20mg/kg/day arm. **Figure 1**

Figure 1 Mean aminosidine concentrations

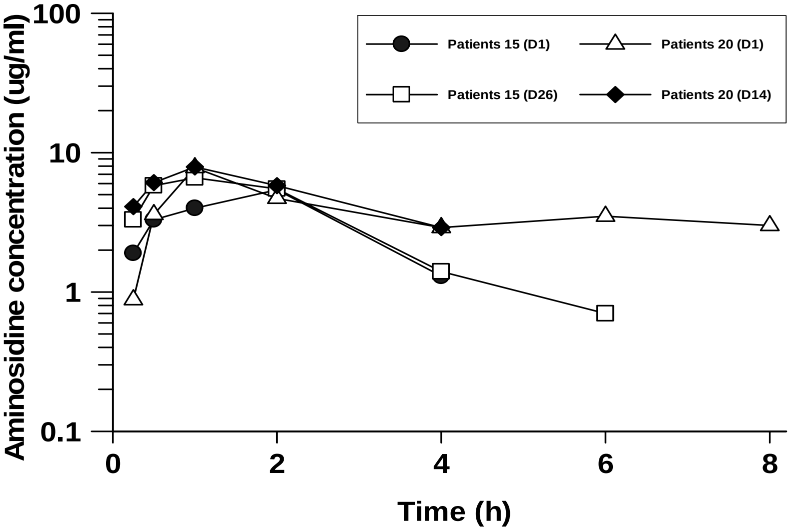

Footnote: Aminosidine is the name by which paromomycin was formerly known

Time to peak plasma concentration was similar for both doses (1.5-2h) Figure 1 indicates that somewhat better concentrations were achieved with the 20mg dose, however patient numbers are small. Blood levels were undetectable after the 8 hour sampling point. Excretion in urine was rapid and complete by 24h.

Discussion of these results by the Principal Investigators of trial LEAP 0104 concluded that both dosing regimens appeared equally effective and were well tolerated.
Whilst the currently available efficacy results at end of treatment and 3 month follow up, indicate only moderate efficacy for paromomycin, all 5 patients who were still positive for parasites at TOC had improved clinically and had achieved a significant reduction in

parasite load of more than 2 log. ie more than 99% clearance in comparison with parasite load at entry.

The unanimous decision of the trial investigators was to adopt the 20mg/kg/day dose given for 21 days, into the original protocol and to re-commence the 3 arm comparative randomised trial at all sites, with no other amendments to the original protocol.

Trial site key personnel and monitor update as at June 2006 can be found in Appendix 1

**Principal Investigators**	**SIGNATURE**	**DATE**
Dr Monique Wasunna<br>(Trial Medical Coordinator)	……………………	
Prof. Asrat Hailu	……………………	
Dr. Getahun Mengistu	……………………	
Dr. Musa A Mudawi	……………………	
Dr. Manica Balasegaram	……………………	

Trial Manager

Dr Juma Rashid ……………………

Trial Statistician

Mr. Lawrence Muthami ……………………

Project Manager

Dr Catherine Royce ……………………

Appendix 1

Site Investigators:

Ethiopia:
Gondar:
Dr Sisay Yifru (Site PI)
Dr Zewdu Hurissa

Abar Minch:
Dr Samson Tesfaye (Site PI)
Dr Teklu Wolde-Gabriel

Kenya:
Dr M Wasunna (PI)
Dr J R Rashid
Dr Jane Mbui
Dr F Kirui

Sudan:
Dr. Ahmed Musa Mudawi (PI)
Prof. E A G Khalil

Monitors:
Dr Hilda O'Hara
Dr. Robert Balikuddembe
Dr. Sarah Nanzigu

Protocol Title: A multicentre, randomised, comparative trial of efficacy and safety of sodium stibogluconate (SSG) versus paromomycin (PM) versus a combination of SSG and PM as first line treatment for visceral leishmaniasis (VL) in Ethiopia, Kenya and Sudan.

This protocol amendment outlines a revision to the sample size and analysis plan for the study including the addition of interim analyses

Reason for Amendment
The following replaces the statistical section of the LEAP 0104 Protocol dated 31st Jul 2004 (Page 21 and 22)

Design:

- The trial will initially run with 3 arms: SSG, PM and Combination.
- The null hypothesis is that there is no difference across treatment arms / no difference between SSG and PM and no difference between SSG and Combination regimens
- A statistically significant p-value would provide evidence of a statistically significant difference between regimens.
- It is thought from LEAP0104 that the standard SSG regimen will clear parasites in approximately 85% of patients at 6 months
- It was considered that a case could be made for licensing PM (for use in combination therapy) if the difference in efficacy of SSG and PM was no more than 15%
- The trial will be powered to test for a statistically significant difference between SSG and PM if the efficacy of SSG is 85% and the efficacy of PM is 70% or less.
- This will require approximately 195 patients per arm for 90% power.

1a) Interim Analysis PM: After recruitment into the trial of 100 patients per arm, an interim analysis will be carried out to compare the *end of treatment* efficacy (TOC) of SSG and PM.

- If the lower bound of the 95% CI of the difference in efficacy is *greater* than the maximum acceptable difference of 15% then there will be evidence to support decision to stop PM and question continuation of the trial. A decision will have to be made in such circumstances if it is worthwhile to continue with SSG and Combination arms only.
- If the lower bound of the 95% CI of the difference in efficacy is *less* than the maximum acceptable difference of 15% then continue recruitment to 195 patients per arm

1b) Efficacy Analysis PM: After recruitment of 195 patients per arm, stop recruiting into the PM arm and when all patients in that arm have reached 6 months follow-up compare the *6 months* efficacy (DC) of SSG and PM and test for a difference. If there is no evidence of significant difference, then conclude that there is no evidence to suggest that the efficacy of PM is more than 15% lower than the efficacy of SSG.

- Continue the trial with just 2 arms (SSG and Combination) and continue to recruit patients until there are 404 patients per arm

2a) Interim Analysis Combination: Compare the efficacy of SSG and Combination regimens *at 6 months* (DC) with 195 patients per arm followed-up for 6 months.

- Consider stopping the trial if the lower 95% confidence bound on the difference is greater than 10%.
- Combine the efficacy data from the SSG and Combination arms from 0104(A) and 0104(B) to include the historic data and repeat the interim analysis.
- Otherwise, continue until there are 404 patients per arm.

2b) Efficacy Analysis Combination: 404 patients per arm will provide 90% power to test for a statistically significant difference if the efficacy of SSG is 85% and the efficacy of the Combination regimen is 75% or less.

- Combine the efficacy data from the SSG and Combination arms from 0104(A) and 0104(B) to include the historic data and repeat the efficacy analysis.

Flow diagram of Recruitment and Analysis:
Following the end of the 1st phase 0104(A), patients will be randomised to receive one of 3 treatment regimens in the 2nd phase 0104(B)

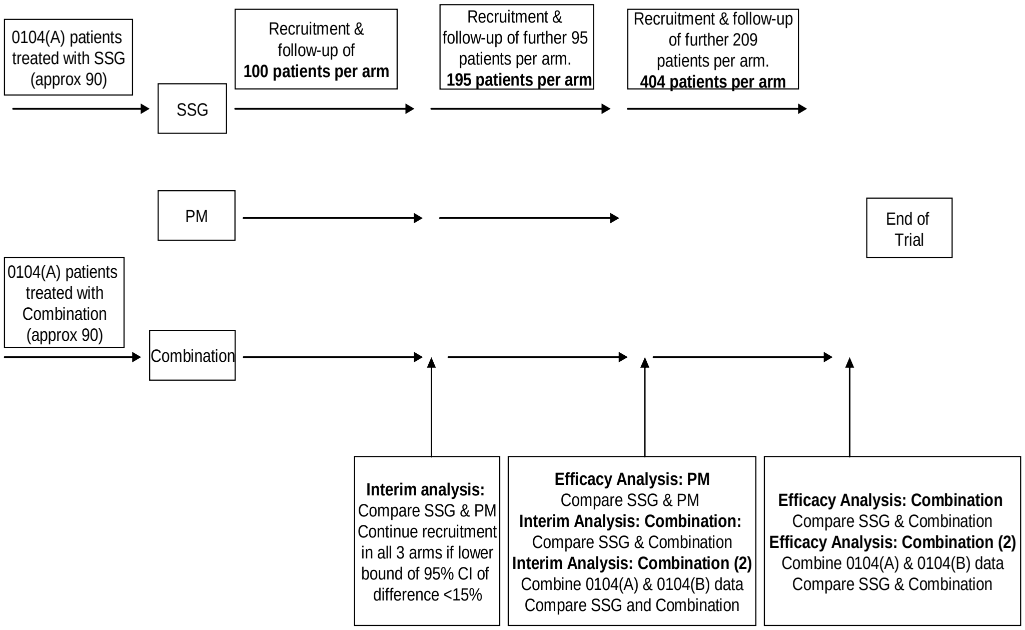

Primary Endpoint and Efficacy Estimation
The definitive cure is measured at 6 months follow-up and the denominators for the efficacy analyses are the number of patients randomised to each treatment arm and entered 6 or more months previously. The numerators for the efficacy estimates are the number of patients who received no other VL treatment other than the randomised regimen and who were found to be

- Parasite free at end of treatment *or* parasite positive at end of treatment with at least 2 log reduction in parasite count (slow responder)

and

- parasite free at 6 months follow-up

Treatment Effect Estimation
The treatment effect will be the treatment difference plus the 95% confidence interval.

Assumptions

- The efficacy data will be analysed overall, not by site
- The trial is not powered for by site analyses
- Interim and final analyses will be carried out only as specified
- Adjustments to the sample size have been made to power the study for a sub-group analysis in HIV negative patients if 10% of patients overall are known to be infected and also to allow for loss-to-follow-up (LTFU) of 10% at 6 months.
- Table 1 gives the numbers needed per arm after adjusting for HIV (10%) and LTFU at 6 months (10%) based on two-sided probabilities with 90% power.

Table 1. Number of patients required per regimen to detect a statistically significant difference, at the 5% level with **90% power**, between the efficacies shown based on a two-sided test for ITT. Estimates are adjusted for 10% to allow for an adequate number of HIV negative patients in sub-group analyses and also for 10% loss-to-follow-up at 6 months.

	**% cured in comparative regimen**								
**% cured SSG**	**0.5**	**0.6**	**0.65**	**0.7**	**0.75**	**0.8**	**0.85**	**0.9**	**0.95**
**0.6**	627								
**0.65**	274	2381							
**0.7**	150	576	2229						
**0.75**	93	245	531	2025					
**0.8**	62	131	223	474	1771				
**0.85**	43	79	117	195	404	1466			
**0.9**	31	50	68	99	161	322	1110		
**0.95**	22	34	43	56	79	121	226	703	
**0.98**	19	27	33	41	54	74	114	222	952

	SIGNATURE	DATE
Dr Monique Wasunna<br>Trial Medical Coordinator	……………………………….	………………….
Mr. Lawrence Muthami<br>Trial Statistician	……………………………….	………………….

Protocol Title: A multicentre, randomised, comparative trial of efficacy and safety of sodium stibogluconate (SSG) versus paromomycin (PM) versus a combination of SSG and PM as first line treatment for visceral leishmaniasis (VL) in Ethiopia, Kenya and Sudan.

This protocol amendment outlines the Further pharmacokinetic (PK) evaluation of Paromomycin (PM), Sodium Stibogluconate (SSG), and the combination of PM and SSG in consenting patients of LEAP 0104 trial following preliminary analysis of initial Pharmacokinetic data presented to a meeting of the Principal and Site Investigators held in Nairobi, Kenya on 22nd Sep 2006.
This amendment applies to sites in Kenya and Sudan only

Addition of Intensive Pharmacokinetic evaluation in Kenya and Sudan

Reason for amendment
Initial results from 2 trial sites in Sudan (n = 90 Um el Kher, and n = 45 Kassab) in the LEAP 0104 trial indicated that paromomycin at a dosage of 15mg/kg/day for 21 days was less effective, for the treatment of acute, symptomatic visceral leishmaniasis.
The majority of cases of VL in the disease endemic area of the Horn of Africa occur in Sudan, therefore it was considered essential to find a dose which is effective in Sudan if paromomycin is to be a useful alternative therapy for VL in this region. Therefore, between September 2005 and March 2006, a total of 42 patients (21 in each treatment arm) were allocated to receive one of two Paromomycin treatment regimens. Results are shown in Table 1.

Table 1:

Regimen	Test of Cure (End of treatment)			3months	Definitive cure (6 month follow-up)
	Number of patients	Parasite -ve	Parasite +ve	Relapse	
15mg for 28 days	21	19	2	3	16/21 (76.2%)
20mg for 21 days	21	18	3	2	16/21 (76.2%)
Total	42	37	5	5	

There was no difference in efficacy either at end of treatment or at 3 and 6 month follow up, with an overall efficacy rate of 76% for both groups.

It was planned that the first six patients in each treatment group, with a body weight of 30kg or more, would have additional venous blood and urine sampling for pharmacokinetic evaluation. A total of 10 patients consented to participate in the PK sampling, 6 in the 15mg/kg/day arm and 4 in the 20mg/kg/day arm.

Figure 1 Mean aminosidine concentrations

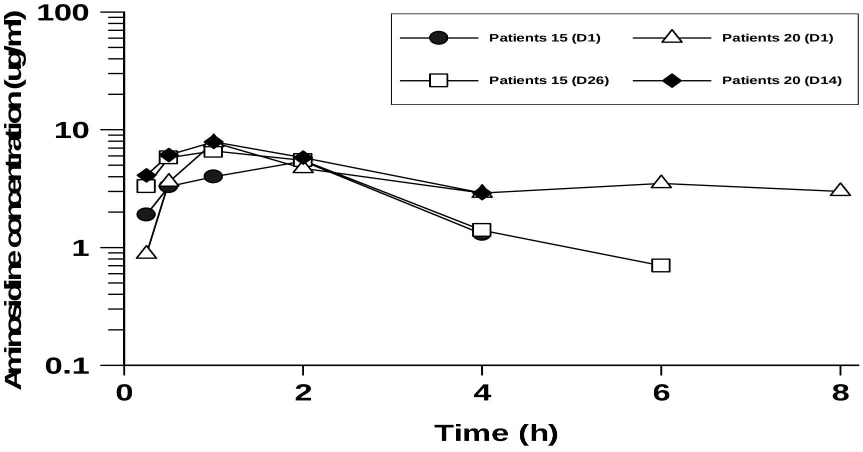

Footnote: Aminosidine is the name by which paromomycin was formerly known

The time to peak plasma concentration (t_{max}) was similar for both doses (1.5-2h). Fig 1 indicates that somewhat better concentrations were achieved with the 20mg dose, however patient numbers are small. Blood levels were undetectable after the 8 hour sampling point. Excretion in urine was rapid and complete at 24h.

Only limited published PK data for paromomycin in healthy volunteers are available, Kanyok et al 1997. These data indicate that American healthy volunteers achieved a C_{max} of 23.4ug/ml.

A small pharmacokinetic study (unpublished: Fig 2) was carried out in healthy Sudanese volunteers which demonstrated that they achieved similar maximum concentration to that of the published data and higher than that of the Sudanese VL patients.

Figure 2

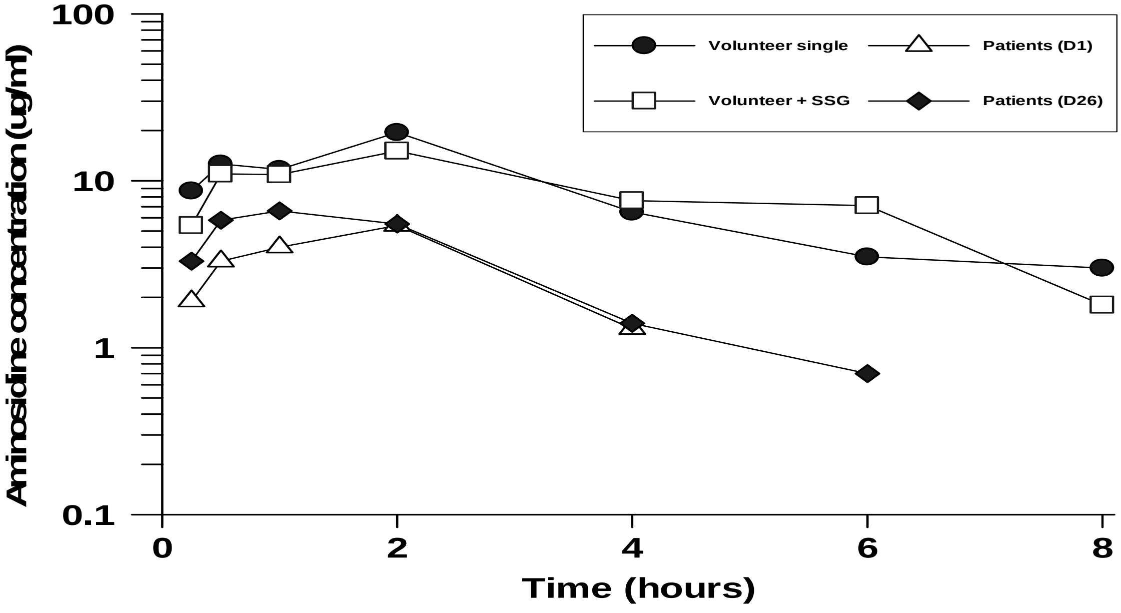

Initial data from Indian VL patients treated with PM 15mg/kg/day demonstrated results (Mordenti et al 2006) similar to those previously published, Kanok et al.

There is also very limited published data on the pharmacokinetics of the combination of SSG and PM. P Ormas et al 1995 investigated the pharmacokinetics of Paromomycin and SSG alone and in combination in dogs.

Based on the evidence above the following protocol amendment is proposed to further elucidate the pharmacokinetics of PM, SSG and Combination of SSG and PM in a greater number of Kenyan, and Sudanese patients.

Amendment
All potentially eligible patients participating in the LEAP 0104 study in Kenya and Sudan will be asked to participate in this pharmacokinetic sub-study. A total of 72 adult patients, 36 in KEMRI and 36 from the Sudanese site in Khasab (12 in each treatment arm) with a body weight of 30kg or more will participate in the intensive pharmacokinetic sampling.

The dosing schedule will remain the same as that described in LEAP 0104 Protocol amendment dated 30th June 2006

The first twelve consenting patients in each treatment group, will have additional venous blood and urine sampling for pharmacokinetic evaluation as outlined in **Appendix 1**.

Inclusion and exclusion criteria are otherwise unchanged from those in the original protocol. (pages 18-19). An additional pharmacokinetic consent form will be used **Appendix 2.**

Patients who are HIV positive *will be excluded from participation in the Pharmacokinetic sub-study.*

Efficacy, Safety and rescue medication All Patients will undergo the same efficacy and safety evaluations and, if required, the same rescue therapy as in the original protocol LEAP 0104 of 31st July 2004 (page 26) and all relevant protocol amendments that have previously been approved by local ethics committees.

References

1. Kanyok, T.P., et al., *Pharmacokinetics of intramuscularly administered aminosidine in healthy subjects.* Antimicrob Agents Chemother, 1997. 41(5): p. 982-6.
2. Mordenti, J., et al, *Paromomycin: An old drug gives birth to a new treatment for Visceral Leishmaniasis* ICOPA 2006

	SIGNATURE	DATE
Dr Monique Wasunna<br>Trial Medical Coordinator	……………………………….	………………….

Principal Investigators

Dr. Musa A Mudawi	……………………………….	………………….
KENYA PI	……………………………….	………………….
SUDAN PI	……………………………….	………………….

Appendix 1

Pharmacokinetic sampling schedule for patients with a body weight of 30kg or more who consent to participate in the PK part of the study.

Plasma Sampling
Venous sampling for PK patients receiving all treatments (n=12 per treatment arm)

Day 1 and End of treatment (Day 21 for PM arm, Day 30 for SSG arm and Day 17 for SSG and PM combination arm):
At time 0, 0.5, 1.0, 3, 6, 10, and 24 hours after dosing

10 ml of blood at each sampling point will be taken to ensure sufficient plasma is obtained for duplicate test analysis.
Total blood volume taken for PK on each sampling day is 70ml.
Total blood volume for the Pharmacokinetic sampling during the treatment period will be 140ml.

Each sample will be collected in a heparinised tube. Samples will be centrifuged at 3000rpm for 10 minutes and plasma will be decanted off and stored in 3 aliquots at - 20°C.

All tubes will be labelled with the Protocol Number LEAP 0104, Centre and Patient number, patient initials, date of sample and time point.

Urine sampling
All patients participating in the sub-study will also have 24 hour urine collections on PK venous sampling days, collected in aliquots 0-2h, 2-4h, 4-6h, 6-8h, 8-10 and 10-24h.

All tubes will be labelled with the Protocol Number LEAP 0104, Centre and Patient number, patient initials, date of sample and time point.

Safety assessments
Safety assessments (blood biochemistry, haematology, urinalysis, ECG and audiometry) will be performed as for other patients participating in the study.

Sample analysis

All samples will be shipped, with prior notice, to Prof Gilbert Kokwaro for analysis

Prof Gilbert Kokwaro,
KEMRI/Wellcome Trust Programme
Dept. of Clinical Pharmacology,
Next to National Public Health Laboratories,
On the Grounds of Kenyatta National Hospital,
PO Box 43640-00100, Nairobi, Kenya

Appendix 2
Consent form for Patients participating in PK sampling.

PATIENT INFORMATION AND CONSENT - Pharmacokinetic sub-study

TITLE: A MULTICENTRE COMPARATIVE TRIAL OF EFFICACY AND SAFETY OF SODIUM STIBOGLUCONATE (SSG) VERSUS PAROMOMYCIN (PM) VERSUS COMBINATION OF SSG AND PM AS THE FIRST LINE TREATMENT FOR VISCERAL LEISHMANIASIS IN ETHIOPIA, KENYA AND SUDAN

PRINCIPAL INVESTIGATOR(S): **ETHIOPIA**
KENYA
SUDAN

SPONSOR: Drugs for Neglected Diseases Initiative, Geneva, Switzerland

Introduction

We are studying the disease, Kala-azar and new drug treatments for this disease. As well as the trial that you have agreed to take part in comparing paromomycin with, SSG, and a combination of both drugs used together, we are conducting a pharmacokinetic (PK) sub-study which looks at the way your body responds to the study drugs. We would like to ask you to participate in this research study.

Procedures during the sub-study

If you agree and you weigh more than 30Kg additional blood samples will be collected on the first day of treatment and again on the last day of treatment. A total of 7 samples will be taken at these times: before study drug, 0.5, 1.0, 3, 6, 10, and 24 hours after study drug.
Approximately 10 ml of blood (about 2 teaspoons) will be taken at each sampling point. The blood volume taken on one day will be approximately 70ml (about 5 tablespoons). A further 70ml of blood will be taken on the last day of treatment.

On the same day as the blood sampling, you will also have 24 hour urine collection. It is planned that this will be collected during the following times 0-2h, 2-4h, 4-6h, 6-8h, 8-10 and 10-24h.

At the site where blood was taken you may have some bruising and it may be painful for a short while.

Benefits

Your participation in this sub-study helps us to know more about your condition which could result in an improved treatment. There is no direct benefit to you but this new knowledge will benefit your community and may reduce the likelihood of other people getting the disease.

Confidentiality

At the end of the study, we plan to write a report about the results of the study. The reports will not bear any information relating to you personally e.g. your name or identity. We assure you of the confidentiality of such information. Thus, we also need your permission to use the test results for writing a report.
We also seek your permission to store any left over samples (blood, tissues) for future studies. We will seek permission from a relevant ethical review committee before any analysis not described in this document is carried out.

In addition, clinical monitors of the sponsor (DNDi) or the regulatory authorities may wish to inspect your records.

Right to refuse or withdraw

You do not have to take part in this research, if you do not wish to do so. This will not affect your treatment at this centre in any way. You will still have the benefit of treatment for your disease at this centre.
If you do decide to participate and then change your mind later, you may do so, at any time, without losing any of your rights as a patient.

In the event that you suffer an injury or illness related to participating in this trial, DNDi will pay all costs relating to treatment of the injury or illness.

If you agree to participate in the study, we will ask you to read and sign the consent form.
Do you have any questions?

Consent Form:

I, the undersigned, confirm that, I give consent to participate in the study, it is with a clear understanding of the objectives and conditions of the study and with the recognition of my right to withdraw from the study if I change my mind.

I ……………………….. do hereby give consent to Dr …………………………… to include me in the proposed research. I have been given the necessary information and understand that there might be some risks involved in the treatment procedures. I have also been assured that I can withdraw my consent at any time without penalty or a loss of benefits. The proposal has been explained to me in the language I understand.

Name of patient: ____________________

Patient's Signature: ____________

Name of Doctor: ________________________

Doctor's Signature: __________________

Date: ________________

Witness: _______________________Date:_________________

Protocol Title: A multicentre, randomised, comparative trial of efficacy and safety of sodium stibogluconate (SSG) versus paromomycin (PM) versus a combination of SSG and PM as first line treatment for visceral leishmaniasis (VL) in Ethiopia, Kenya and Sudan.

This protocol amendment outlines the following changes to the LEAP 0104 protocol:

1. Further pharmacokinetic (PK) evaluation (sparse sampling) of Paromomycin (PM), Sodium Stibogluconate (SSG), and the combination of PM and SSG in consenting patients of LEAP 0104 trial following preliminary analysis of initial Pharmacokinetic data presented to a meeting of the LEAP Investigators held in Nairobi, Kenya on 22nd Sep 2006. This amendment applies to sites in Ethiopia only

Addition of Sparse Pharmacokinetic evaluation in Ethiopia

Reason for amendment

Currently there is limited pharmacokinetic data available on SSG and Paromomycin and no pharmacokinetic data in man on the combination of Paromomycin and SSG.

Preliminary intensive pharmacokinetic evaluation of 2 treatment regimens of paromomycin, 15mg/kg/day for 28 days or 20mg/kg/day for 21 days was carried out in 10 Sudanese patients with a body weight of 30kg or more. The time to peak plasma concentration (t_{max}) was similar for both doses (1.5-2h). Fig 1 indicates that somewhat better concentrations were achieved with the 20mg dose, however patient numbers are small. Blood levels were undetectable after the 8 hour sampling point. Excretion in urine was rapid and complete at 24h.

Figure 1 Mean aminosidine concentrations

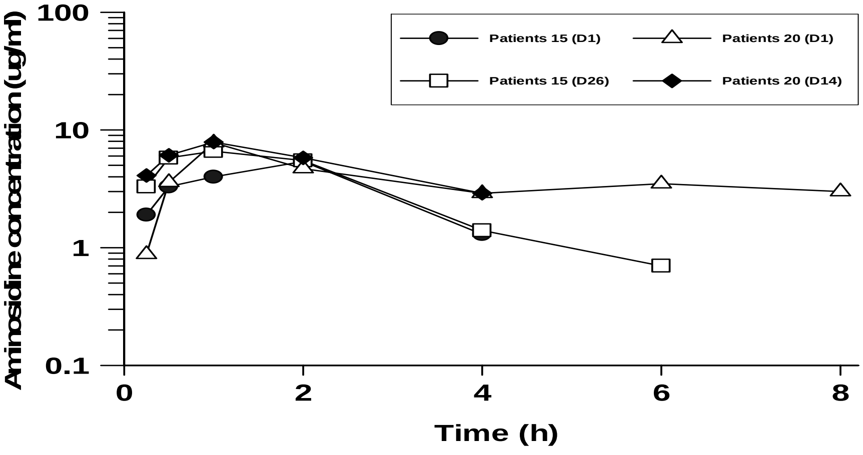

Footnote: Aminosidine is the name by which paromomycin was formerly known

Only limited published PK data for paromomycin in healthy volunteers are available, Kanyok et al 1997. These data indicate that American healthy volunteers achieved a C_{max} of 23.4ug/ml.

A small pharmacokinetic study (unpublished: Fig 2) was carried out in healthy Sudanese volunteers which demonstrated that they achieved similar maximum concentration to that of the published data and higher than that of the Sudanese VL patients.

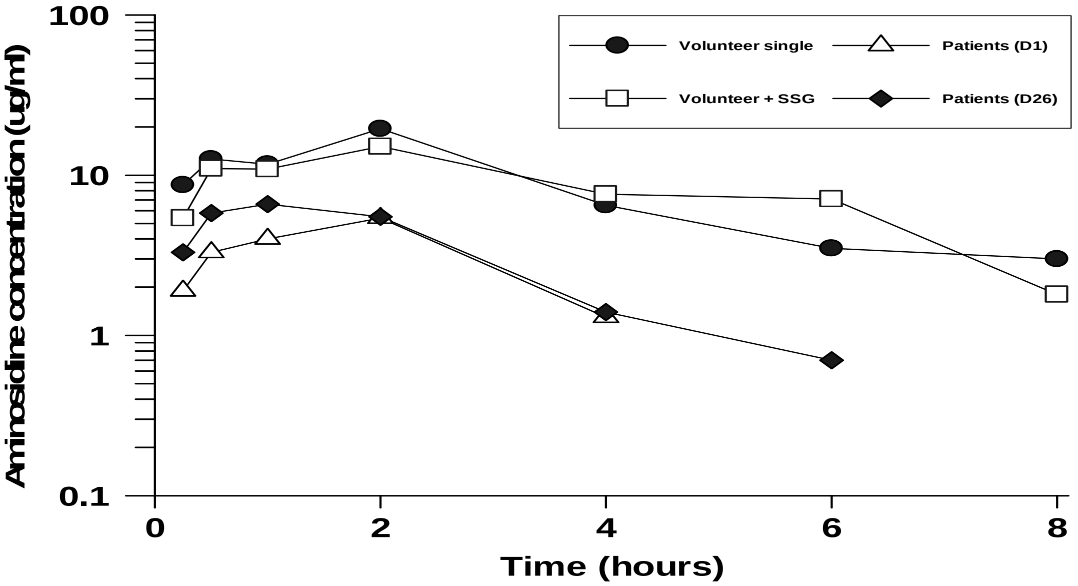

Initial data from Indian VL patients treated with PM 15mg/kg/day demonstrated results (Mordenti et al 2006) similar to those previously published, Kanok et al.

There is also very limited published data on the pharmacokinetics of the combination of SSG and PM. P Ormas et al 1995 investigated the pharmacokinetics of Paromomycin and SSG alone and in combination in dogs.

Based on the evidence above and recent experience from the ongoing trial which indicates that more than 50% of patients may be children the following protocol amendment is proposed to further elucidate the population pharmacokinetics of PM, SSG and Combination of SSG and PM in a greater number of Ethiopian patients including children.

Amendment

All patients aged 7 years and above participating in the LEAP 0104 study will be asked to participate in this sparse sampling pharmacokinetic sub-study.

***Patients who are HIV positive** will be excluded from participation in the Pharmacokinetic sub-study.*

Inclusion and exclusion criteria are otherwise unchanged from those in the original protocol (pages 18-19). An additional pharmacokinetic consent form will be used **Appendix 1.**

All consenting patients will be randomly allocated to one of 6 sample schedules. Each schedule will specify 3 timepoints at which 3 additional venous blood

samples will be taken for pharmacokinetic evaluation. The procedure and sample scheduled is outlined in **Appendix 2**. A randomisation list will be prepared for each treatment arm.

The dosing schedule will remain the same as that described in LEAP 0104 Protocol amendment dated 30th June 2006

Efficacy, Safety and rescue medication All Patients will undergo the same efficacy and safety evaluations and, if required, the same rescue therapy as in the original protocol LEAP 0104 of 31st July 2004 (page 26) and all relevant protocol amendments that have previously been approved by ethics committees.

References

1. Kanyok, T.P., et al., *Pharmacokinetics of intramuscularly administered aminosidine in healthy subjects.* Antimicrob Agents Chemother, 1997. 41(5): p. 982-6.
2. Mordenti, J., et al, *Paromomycin: An old drug gives birth to a new treatment for Visceral Leishmaniasis* ICOPA 2006

	SIGNATURE	DATE
Dr Monique Wasunna<br>Trial Medical Coordinator	………………………………	…………………

Principal Investigators

Prof. Asrat Hailu	………………………………	…………………
Dr. Sisay Yifru	………………………………	…………………
Dr Samson Tesafye	………………………………	…………………

Appendix 2

Consent form for Patients participating in PK sampling.

PATIENT INFORMATION AND CONSENT - Pharmacokinetic sub-study

TITLE: A MULTICENTRE COMPARATIVE TRIAL OF EFFICACY AND SAFETY OF SODIUM STIBOGLUCONATE (SSG) VERSUS PAROMOMYCIN (PM) VERSUS COMBINATION OF SSG AND PM AS THE FIRST LINE TREATMENT FOR VISCERAL LEISHMANIASIS IN ETHIOPIA, KENYA AND SUDAN

PRINCIPAL INVESTIGATOR(S): **ETHIOPIA**
KENYA
SUDAN

SPONSOR: Drugs for Neglected Diseases Initiative, Geneva, Switzerland

Introduction

We are studying the disease, Kala-azar and new drug treatments for this disease. As well as the trial that you have agreed to take part in comparing paromomycin with, SSG, and a combination of both drugs used together, we are conducting a pharmacokinetic (PK) sub-study which looks at the way your body absorbs, distributes and gets rid of the study drugs. We would like to ask you to participate in this research study.

Procedures during the sub-study

If you agree 3 additional blood samples will be collected on one treatment day. Approximately 5 or 10 ml of blood (about 1 or 2 teaspoons) will be taken at each sampling point. The blood volume taken will be approximately 15 or 30ml (about 3 to 6 teaspoons). The volume of blood taken will depend on which treatment you are receiving.

At the site where blood was taken you may have some bruising and it may be painful.

Benefits

There are no benefits to your participation in this sub-study. If the study is successful it means that more will be known about the treatments for kala-azar which could result in improved or shorter treatment periods. This will benefit your community and may reduce the likelihood of other people getting the disease.

Confidentiality

At the end of the study, we plan to write a report about the results of the study. The reports will not bear any information relating to you personally e.g. your name or identity. We assure you of the confidentiality of such information. Thus, we also need your permission to use the test results for writing a report.

In addition, clinical monitors of the sponsor (DNDi) or the regulatory authorities may wish to inspect your records.

Right to refuse or withdraw

You do not have to take part in this research, if you do not wish to do so. This will not affect your treatment at this centre in any way. You will still have the benefit of treatment for your disease at this centre.

If you do decide to participate and then change your mind later, you may do so, at any time, without losing any of your rights as a patient.

In the event that you suffer an injury or illness related to participating in this trial, DNDi will pay all costs relating to treatment of the injury or illness.

If you agree to participate in the study, we will ask you to read and sign the consent form.

Do you have any questions?

Consent Form:

I, the undersigned, confirm that, I give consent to participate in the study, it is with a clear understanding of the objectives and conditions of the study and with the recognition of my right to withdraw from the study if I change my mind.

I ……………………….. do hereby give consent to Dr ……………………………
to include me in the proposed research. I have been given the necessary information and understand that there might be some risks involved in the treatment procedures. I have also been assured that I can withdraw my consent at any time without penalty or a loss of benefits. The proposal has been explained to me in the language I understand.

Name of patient: ______________________

Patient's Signature: _____________

Name of Doctor: ___________________________

Doctor's Signature: ____________________

Date: __________________

Witness: __________________________Date:___________________

CONSENT FOR MINORS (UNDER 18 YRS)

I Mr/Ms ________________________ being a person aged 18 years or over and

being the Parent/Lawful guardian of Master/Miss _________________________

consent to Dr________________________ to include

Master/Miss___________________ in the intended research as explained and

understood by me.

I have understood the implications, risks and immediate benefits of the tests to him / her. I accept the tests to be carried out and the risks attached.

I understand that I have the right to withdraw him / her from the research at any time, for any reason without penalty or harm. In case of withdrawal, I understand that the Physicians will continue to take care of him/her like any other patient.

All the above conditions have been explained to me in a language, which I understand.

Guardian's full name ___________________________________

Guardian's signature ___________________________________

Date:_____________

Child's full name ___________________________________

Person obtaining consent ___________________________________

Witness ___________________________________

Date: _____________

MINORS ASSENT FORM FOR INCLUSION IN THE TRIAL (12-17 years old) (for signatures)

I, the undersigned, confirm that, as I give my assent to participate in the study, it is with a clear understanding of the objectives and conditions of the study and with the recognition of my right to withdraw from the study if I change my mind. I

……………………….. do hereby give my assent to Dr ……………………………to include me in the proposed research. I have been given the necessary information and understand that there might be some risks involved in the treatment procedures.

I have also been assured that I can withdraw my assent at any time without penalty or loss of the benefit of treatment. The study has been explained to me in the language I understand.

I agree to participate

Name of Minor: _____________________

Minor's Signature: __________________

Date:__________________

Appendix 2

Sparse Pharmacokinetic sampling procedure

Plasma Sampling

Each eligible patient will be randomly allocated to one of the following sample schedules.

**Sampling Schedule**	**Day of sampling**	Timepoint post drug administration
1	7	0.5, 1, 3 h
2	7	6,10, 24 h
3	14	0.5, 1, 3 h
4	14	6,10, 24 h
5	End of treatment	0.5, 1, 3 h
6	End of treatment	6,10, 24 h

5ml blood for PM and SSG alone and 10 ml of blood for combination at each sampling point will be taken to ensure sufficient plasma is obtained for duplicate test analysis.
Total blood volume taken for pharmacokinetic analysis is 15 ml for PM or SSG alone and 30ml for SSG and PM combination.

Each sample will be collected in a heparinised tube. Samples will be centrifuged at 3000rpm for 10 minutes and plasma will be decanted off and stored in 3 aliquots at -20°C.

All tubes will be labelled with the Protocol Number LEAP 0104, Centre and Patient number, patient initials, date of sample and timepoint.

Safety assessments

Safety assessments (blood biochemistry, haematology, urinalysis, ECG and audiometry) will be performed as for other patients participating in the study.

Sample analysis

Prior to shipping a Material transfer agreement with ESTA will be put in place.

All samples will be shipped, with prior notice, to Prof Gilbert Kokwaro for analysis

Prof Gilbert Kokwaro,
KEMRI/Wellcome Trust Programme
Dept. of Clinical Pharmacology,
Next to National Public Health Laboratories,
On the Grounds of Kenyatta National Hospital,
PO Box 43640-00100, Nairobi, Kenya

LEAP 0104 Final Protocol amendment Number 11: dated 20th March 2008

Protocol Title: A multicentre, randomised, comparative trial of efficacy and safety of sodium stibogluconate (SSG) versus paromomycin (PM) versus a combination of SSG and PM as first line treatment for visceral leishmaniasis (VL) in Ethiopia, Kenya, Sudan and Uganda.

This protocol amendment outlines a revision to the recruitment strategy of the protocol. The overall sample size remains unchanged and is as described in Protocol amendment number 8 dated 14 Dec 2006. The change will be to the number of patients that are recruited by each site.

Reason for Amendment
Due to the addition of a new site in Uganda and the length of the ethical review process in Ethiopia and in order to minimize the time taken to recruit the remaining patients into the clinical trial the recruitment strategy for the study will be revised. As of 17 March 2008 440 patients have been recruited under the LEAP 0104B protocol amendment

Details of the Protocol Amendment
The recruitment strategy will be revised and recruitment will continue in all countries, with the required ethical and regulatory approvals until the required sample size is achieved. The total sample size remains and is as described in Protocol amendment number 8 dated 14 Dec 2006.

SSG vs PM

- 195 Patients per arm for SSG (20mg/kg/day for 30 days) vs Paromomycin (20mg/kg/day for 21 days)
- Total patients to be recruited for SSG vs PM Analysis = 390 patients (195 per treatment arm)

SSG vs Combination

- 404 Patients for SSG arm vs 404 patients for Combination of SSG and Paromomycin arm
- Total patients to be recruited for SSG vs Combination of SSG + PM Analysis = 808 patients
- 808 patients comprise the following:
    - 538 out of 808 (269 per arm) are to be recruited in the current LEAP 0104B amendment no. 8 dated 14 December 2006.
    - 270 patients so far out of 808 (135 per arm (SSG and Combination only) have been recruited under the original protocol dated 31 July 2004)

The total number of patients to be recruited to complete both analyses (SSG vs PM and SSG vs Combination) is 1003. As of 17 March 2008, there have been 710 patients recruited (440 from 0104B and 270 from 0104A). There are 293 patients still to be recruited to complete all analyses.

The current recruitment as of 17 mar 2008 and Amended maximum number of patients to be recruited into LEAP 0104B at each site will be:

Country	Patient recruited in 0104a & 0104b to 17 Mar 2008	Maximum number of patients recruited per site (maximum patients recruited in 0104B)
Sudan	440	up to 590 patients (500)
Kenya	120	up to 205 patients (175)
Ethiopia	150	up to 240 patients (90)
Uganda		up to 60 patients (60)
**Total**	710	1095 (825)

	SIGNATURE	DATE
Dr Monique Wasunna<br>Trial Medical Coordinator	………………………………	…………………
Dr Musa A. Mudawi<br>Principal Investigator	………………………………	…………………

16.2.1 Sample case report form

Appendix 2 Sample CRF

LEAP 0104	Centre Number			Subject Number				Subject Initials		

CASE REPORT FORM: BOOK ONE (ON DIAGNOSIS AND TREATMENT: PRE-TREATMENT TO DAY 18)

(COMBINATION OF PAROMOMYCIN & SSG)

LEAP 0104

A MULTICENTRE COMPARATIVE TRIAL OF EFFICACY AND SAFETY OF SODIUM STIBO-GLUCONATE (SSG) VERSUS PAROMOMYCIN (PM) VERSUS COMBINATION OF SSG AND PM AS THE FIRST LINE TREATMENT FOR VISCERAL LEISHMANIASIS IN ETHIOPIA, KENYA AND SUDAN

LEAP 0104	Centre Number	Subject Number	Subject Initials

Patient's Hospital Number _____________

Country _____________ Centre No_____________

Patient Study No. _________________Date of Admission _________

Patient's initials____________________________

Patient's Occupation _______________________

Date of Birth (DoB) ___________

Age of the patient (in years): ________________ If DoB is not given provide an estimated age of the patient in years.

Gender: Male Female

Level of Education: 0: None 1: Primary 2: Secondary 3: Post secondary

Complete Address of the patient

Province/Region: _______________________

District: _______________________

Location: ________________Name of the Chief ______________

Sub-Location: _____________Name of Assistant Chief _________

Village: _________________ Name of the Village Head ____________

Name of the Head of Household: ____________________

Contact Address

____

____

____

Randomized to receive Drug Regimen: ___________________

Other Current Diagnoses: ________________________

LEAP 0104	Centre Number	Subject Number	Subject Initials

Baseline

PATIENTS' SYMPTOMS

**Complaints**	Yes	No	Duration
Fever			
Headache			
Fatigue			
Body Weakness			
Malaise			
Epistaxis			
Abdominal pains			
Abdominal swellings			
Swelling of legs			
Cough			
Breathlessness			
Night Sweats			
Loss of appetite			
Weight loss			
Diarrhoea			
Skin lesions			
Others (specify)			

LEAP 0104	Centre Number		Subject Number			Subject Initials		

Baseline

HISTORY OF PAST ILLNESSES

**Illnesses**	Yes	No	Details/Date if known
Previous Treatment for Leishmaniasis			
Previous Treatment for Tuberculosis			
Other causes of significant previous hospital admissions			

LEAP 0104	Centre Number	Subject Number	Subject Initials

Baseline

Clinical Examination:

Weight: ☐☐☐ Kg
(Light clothes, rounded up to the nearest Kg)

Height ☐☐☐ cm

Blood Pressure:
(After 5mins,sitting) ☐☐☐ / ☐☐☐ mmHg

Heart Rate: ☐☐☐ bpm

Axillary Temperature: ☐☐ • ☐ °C

Indicate **present or absent** for each of the characteristics below

**Characteristics**	Present	Absent
Mucosal pallor		
Jaundice		
Cervical lymphadenopathy		
Axillary lymphadenopathy		
Inguinal lymphadenopathy		
Muscle Wasting		
Petechial haemorrhages		
Other (specify)		

Abdominal palpation

Abdominal palpation for spleen size (cm) (by palpation below left costal margin in the anterior axillary line) ___________cm

Abdominal palpation for liver size (cm) (by palpation below right costal margin in the mid-clavicular line) ___________cm

LEAP 0104	Centre Number	Subject Number	Subject Initials

Baseline

Haematologic Examination

Date the sample Analysed	Day Mth Yr	
Laboratory Test	Units	Value
Haemoglobin		
WBC (total)		
Platelets		

HIV STATUS

1. Positive ☐
2. Negative ☐
9. Not Tested ☐

Note: Please note that being HIV positive is not an inclusion /exclusion criteria.

Clinical Chemistry Examination

Date sample Analysed	Day Mth Yr	
Laboratory Test	Units	Value
SGOT/AST		
SGPT/ALT		
Bilirubin		
Alkaline Phosphatase		
Albumin		
Globulin		
Prothrombin Time		
Creatinine		
BUN		
Sodium		
Potassium		
Amylase		
Others		

LEAP 0104	Centre Number	Subject Number	Subject Initials

Baseline

Parasitologic Examination

Date sample Analysed ____________________

Spleen/bone Marrow /lymphnode Aspirate and enter the appropriate value in the box provided:

6+ 100,000/1000 oil emersion x 100
5+ 10,000-100,000/1000 oil emersion x 100
4+ 1,000-10,000/1000 oil emersion x 100 ☐
3+ 100-1,000/1000 oil emersion x 100
2+ 10-100/1000 oil emersion x 100
1+ 1-10/1000 oil emersion x 100
0 0/1000 oil emersion x 100

Urinalysis (Dipstick Test and Microscopy)

**Dipstick Tests**	
Colour	
Specific Gravity	
pH	
Blood	
Glucose	
Ketones	
Protein	
**Microscopy**	**PHF**
White Blood cell count (Pus cell)	
Red blood cell count	
Casts (Specify)	
Bacteria	
Others	

LEAP 0104	Centre Number	Subject Number	Subject Initials

Baseline

Chest X-Ray (CXR)/Posterior –Anterior (if indicated)

Date performed: [|] [|] [|]
Day Mth Yr

Please describe the subject's x-ray by ticking one the boxes below:

☐ Normal (no clinical significant abnormality)

☐ Clinically significant abnormality → Record a summary of the significant radiological findings in the space provided below: -

____

____

____

LEAP 0104	Centre Number	Subject Number	Subject Initials

Baseline

12-Lead Electrocardiographic Examination

Date performed: [ ] [ ] [ ]
Day Mth Yr

Please describe the subject's12-lead electrocardiogram by ticking one of the boxes below:

☐ Normal / no clinically significant abnormality

☐ Clinically significant abnormality → Record a summary of the significant electrocardiographic findings in the space provided below:-

____

____

____

Audiometric Examination

Date performed: [ ] [ ] [ ]
Day Mth Yr

Please describe the subjects audiometry by ticking one of the following boxes below

☐ Normal/no clinically significant abnormality.

☐ Clinically significant abnormality → Record a summary of the significant audiometric findings in the space provided below :-

____

____

____

LEAP 0104	Centre Number	Subject Number	Subject Initials

Baseline

Baseline concomitant diseases

Are there any concomitant Diseases ?Yes / No
If yes, Specify

	Start date	Recovery date
1.________________________	__________	____________
2.________________________	__________	____________
3. _______________________	__________	____________
4.________________________	__________	____________
5.________________________	__________	____________
6.________________________	__________	____________
7.________________________	__________	____________
8.________________________	__________	____________
9.________________________	__________	____________
10.______________________	__________	____________

For every mentioned condition requiring treatment, please fill in the “Concomitant medication Section.

LEAP 0104	Centre Number	Subject Number	Subject Initials

DAY 7:
VISCERAL LEISHMANIASIS SEVERITY ASSESSMENTS
Clinical Examination

Weight: (Light clothes, rounded up to the nearest Kg) ☐☐☐ Kg

Blood Pressure: (After 5mins,sitting) ☐☐☐ / ☐☐☐ mmHg

Heart Rate: ☐☐☐ bpm

Axillary Temperature: ☐☐ • ☐ °C

Indicate **present or absent** for each of the characteristics below

**Characteristics**	Present	Absent
Mucosal pallor		
Jaundice		
Cervical lymphadenopathy		
Axillary lymphadenopathy		
Inguinal lymphadenopathy		
Muscle Wasting		
Petechial haemorrhages		
Other (specify)		

Abdominal palpation

Abdominal palpation for spleen size (cm) (by palpation below left costal margin in the anterior axillary line) ____________________cm

Abdominal palpation for liver size (cm) (by palpation below right costal margin in the mid-clavicular line) ___________cm

LEAP 0104	Centre Number	Subject Number	Subject Initials

Day 7

12-Lead Electrocardiographic Examination

Date performed: ___ | ___ | ___
Day Mth Yr

Please describe the patient's12-lead electrocardiogram by ticking one of the boxes below:

☐ Normal / no clinically significant abnormality

☐ Clinically significant abnormality → Record a summary of significant findings in the space provided below:

____

Audiometric Examination

Date performed: ___ | ___ | ___
Day Mth Yr

Please describe the patients' audiometry by ticking one of the following boxes below

☐ Normal/no clinically significant abnormality.

☐ Clinically significant abnormality → Record a summary of significant findings in the space provided below:

____

____

____

LEAP 0104	Centre Number	Subject Number	Subject Initials

Day 7

Haematologic Examination

Date the sample Analysed	Day Mth Yr	
Laboratory Test	Units	Value
Haemoglobin		
WBC (total)		
Platelets		

Clinical Chemistry Examination

Date sample Analysed	Day Mth Yr	
Laboratory Test	Units	Value
SGOT/AST		
SGPT/ALT		
Bilirubin		
Alkaline Phosphatase		
Albumin		
Globulin		
Prothrombin Time		
Creatinine		
BUN		
Sodium		
Potassium		
Amylase		
Others		

LEAP 0104	Centre Number	Subject Number	Subject Initials

Day 7

Urinalysis (Dipstick Test and Microscopy)

**Dipstick Tests**	
Colour	
Specific Gravity	
pH	
Blood	
Glucose	
Ketones	
Protein	
**Microscopy**	**HPF**
White Blood cell count	
Red blood cell count	
Casts (Specify)	
Bacteria	
Others	

Adverse Experiences

Has this patient experienced any adverse event since the last assessment?

No ☐ YES ☐

If YES Please record the details in the report form in the adverse events section.

Please note, for every adverse event requiring treatment, fill in the “Concomitant medication section.

LEAP 0104	Centre Number	Subject Number	Subject Initials

Day 7

Assessment of clinical response:

Based on the clinical assessment at the screening visit, mark the boxes which best describe the patient's clinical outcome so far:

Improving: ☐ Achievement of at least one of the following changes:
i) Subject becomes afebrile.
ii) Increase in Haemoglobin by 2.0g/dl.
iii) White blood cell count value increased 5 x $10^3/mm^3$.
Continue with study medication.

Stable: ☐ No change yet in the values of the above parameters (i) to (iii) from baseline.
Continue with study medication.

Deteriorating: ☐ Worsening of the values of the above parameters
(i) to (iii) above from baseline.

If the patient's condition is **deteriorating,** please withdraw the patient from the study and start Ambisome treatment, complete Ambisome medication section; early study conclusion section; and clinical response assessment section.

Investigator's name ________________ **Date** _________________

Signature ________________________

Drugs for Neglected Diseases *initiative*

LEAP 0104	Centre Number	Subject Number	Subject Initials

DAY 14:
VISCERAL LEISHMANIASIS SERVERITY ASSESSMENTS
Clinical Examination

Weight: (Light clothes, rounded up to the nearest Kg) ☐☐☐ Kg

Blood Pressure: (After 5mins,sitting) ☐☐☐ / ☐☐☐ mmHg

Heart Rate: ☐☐☐ bpm

Axillary Temperature: ☐☐ • ☐ °C

Indicate **present or absent** for each of the characteristics below

**Characteristics**	Present	Absent
Mucosal pallor		
Jaundice		
Cervical lymphadenopathy		
Axillary lymphadenopathy		
Inguinal lymphadenopathy		
Muscle Wasting		
Petechial haemorrhages		
Other (specify)		

Abdominal palpation

Abdominal palpation for spleen size (cm) (by palpation below left costal margin in the anterior axillary line) ____________________cm

Abdominal palpation for liver size (cm) (by palpation below right costal margin in the mid-clavicular line) ___________cm

LEAP 0104	Centre Number	Subject Number	Subject Initials

Day 14

12-Lead Electrocardiographic Examination

Date performed: ___ ___ ___
Day Mth Yr

Please describe the patient's12-lead electrocardiogram by ticking one of the boxes below:

☐ Normal / no clinically significant abnormality

☐ Clinically significant abnormality → Record a summary of significant findings in the space provided below:

____

Audiometric Examination

Date performed: ___ ___ ___
Day Mth Yr

Please describe the patients' audiometry by ticking one of the following boxes below

☐ Normal/no clinically significant abnormality.

☐ Clinically significant abnormality → Record a summary of significant findings in the space provided below:

____

____

____

____

Haematologic Examination

Date the sample Analysed	Day Mth Yr	
Laboratory Test	Units	Value
Haemoglobin		
WBC (total)		
Platelets		

LEAP 0104	Centre Number	Subject Number	Subject Initials

Day 14

Clinical Chemistry Examination

Date sample Analysed	Day Mth Yr	
Laboratory Test	Units	Value
SGOT/AST		
SGPT/ALT		
Bilirubin		
Alkaline Phosphatase		
Albumin		
Globulin		
Prothrombin Time		
Creatinine		
BUN		
Sodium		
Potassium		
Amylase		
Others		

LEAP 0104	Centre Number		Subject Number			Subject Initials		

Day 14

Urinalysis (Dipstick Test and Microscopy)

**Dipstick Tests**	
Colour	
Specific Gravity	
pH	
Blood	
Glucose	
Ketones	
Protein	
**Microscopy**	**HPF**
White Blood cell count	
Red blood cell count	
Casts (Specify)	
Bacteria	
Others	

Adverse Experiences

Has this patient experienced any adverse event since the last assessment?

No ☐ YES ☐

If YES Please record the details in the appropriate report form in adverse events section.

Please note, for every adverse event requiring treatment, fill in the "Concomitant medication section.

**LEAP 0104**	Centre Number	Subject Number	Subject Initials

Day 14

Assessment of clinical response:

Based on the clinical assessment at the screening visit, mark the boxes which best describe the patient's clinical outcome so far:

**Improving:**	☐	Achievement of at least one of the following changes:<br>i) Subject becomes afebrile.<br>ii) Increase in Haemoglobin by 2.0g/dl.<br>iii) White blood cell count value increased 5 x $10^3/mm^3$.<br>**Continue with study medication.**
**Stable:**	☐	No change yet in the values of the above parameters (i) to (iii) above from baseline. **Continue with study medication**.
**Deteriorating:**	☐	Worsening of the values of the above parameters<br>(i) to (iii) above from baseline.

If the patient's condition is **deteriorating,** please withdraw the patient from the study and start Ambisome treatment complete Ambisome medication section; early study conclusion section; and clinical response assessment section.

Investigator's name ________________ **Date** _________________

Signature ________________________

LEAP 0104	Centre Number	Subject Number	Subject Initials

Day 18

VISCERAL LEISHMANIASIS SEVERITY ASSESSMENTS
Clinical Examination

Weight: ☐☐☐ Kg
(Light clothes, rounded up to the nearest Kg)
Blood Pressure:
(After 5mins,sitting) ☐☐☐ / ☐☐☐ mmHg

Heart Rate: ☐☐☐ bpm

Axillary Temperature: ☐☐ • ☐ °C

Indicate **present or absent** for each of the characteristics below

**Characteristics**	Present	Absent
Mucosal pallor		
Jaundice		
Cervical lymphadenopathy		
Axillary lymphadenopathy		
Inguinal lymphadenopathy		
Muscle Wasting		
Petechial haemorrhages		
Other (specify)		

Abdominal palpation

Abdominal palpation for spleen size (cm) (by palpation below left costal margin in the anterior axillary line) ____________________cm

Abdominal palpation for liver size (cm) (by palpation below right costal margin in the mid-clavicular line) ___________cm

LEAP 0104	Centre Number	Subject Number	Subject Initials

Day 18

12-Lead Electrocardiographic Examination

Date performed: [ ] [ ] [ ]
Day Mth Yr

Please describe the patient's12-lead electrocardiogram by ticking one of the boxes below:

☐ Normal / no clinically significant abnormality

☐ Clinically significant abnormality → Record a summary of significant findings in the space provided below:

____

____

____

____

Audiometric Examination

Date performed: [ ] [ ] [ ]
Day Mth Yr

Please describe the patients' audiometry by ticking one of the following boxes below

☐ Normal/no clinically significant abnormality.

☐ Clinically significant abnormality → Record a summary of significant findings in the space provided below:

____

____

____

____

LEAP 0104	Centre Number	Subject Number	Subject Initials

Day 18

Haematologic Examination

Date the sample Analysed	Day Mth Yr
Laboratory Test	Units	Value
Haemoglobin		
WBC (total)		
Platelets		

Parasitologic Examination

Date sample Analysed _____________________

Spleen/bone Marrow /lymphnode aspirate (Leishmania Index).Enter the appropriate index in the box provided.

6+
5+
4+ ☐
3+
2+
1+
0

LEAP 0104	Centre Number	Subject Number	Subject Initials

Day 18

Clinical Chemistry Examination

Date sample Analysed	Day Mth Yr	
Laboratory Test	Units	Value
SGOT/AST		
SGPT/ALT		
Alkaline Phosphatase		
Albumin		
Globulin		
Prothrombin Time		
Creatinine		
BUN		
Sodium		
Potassium		
Amylase		
Others		

LEAP 0104	Centre Number		Subject Number			Subject Initials		

Day 18

Urinalysis (Dipstick Test and Microscopy)

**Dipstick Tests**	
Colour	
Specific Gravity	
pH	
Blood	
Glucose	
Ketones	
Protein	
**Microscopy**	**HPF**
White Blood cell count	
Red blood cell count	
Casts (Specify)	
Bacteria	
Others	

LEAP 0104	Centre Number	Subject Number	Subject Initials

Day 18

Adverse Experiences

Has this patient experienced any adverse event since the last assessment?

No ☐ YES ☐

If YES Please record the details in the report form.

Please note, for every adverse event requiring treatment, fill in the "Concomitant medication section.

Assessment of Treatment Response

Based on the clinical/physical assessment at the screening visit, mark the box or boxes which best describe the subject's clinical outcome so far:

CLINICAL RESPONSE:

COMPLETE	☐	Resolution of all signs and symptoms of VL
PARTIAL	☐	Some signs or symptoms of VL persist

PARASITOLOGICAL RESPONSE:

**COMPLETE PARASITE CLEARANCE**	☐	-Smear negative after counting 1000 fields<br>-Oil immersion (x100)
**PARTIAL PARASITE CLEARANCE (SLOW RESPONDER)**	☐	Smear positive but ≥99%parasite reduction<br>[≥2 log reduction] compared with pre-treatment slide.
**PERSISTANT PARASITAEMIA (TREATMENT FAILURE)**	☐	Smear positive and <99% parasite reduction<br>[<2 log reduction] compared with pre-treatment slide.

Investigator's name ________________ **Date** _________________

Signature __________________________

LEAP 0104	Centre Number	Subject Number	Subject Initials

TRIAL MEDICATION

**Study Day**	**Medication**	**Dose**	**Time Given** 24hr:min	**Comments (if any)**
1				
2				
3				
4				
5				
6				
7				
8				
9				
10				

Note;
If patient misses a dose for any reason write "not given" in medication column and specify reason in comments column. Continue with the correct dose next day.

LEAP 0104	Centre Number	Subject Number	Subject Initials

TRIAL MEDICATION

Study Day	Medication	Dose	Time Given 24hr:min	Comments
11				
12				
13				
14				
15				
16				
17				

LEAP 0104	Centre Number	Subject Number	Subject Initials

CONCOMITANT MEDICATION (Excluding Ambisome medication)

Please record all baseline medications and all concomitant medication taken during the study.
Mark box below or complete page with concomitant medication details.

☐ Please mark box if no concomitant medication is being used.

Any new medical illness/diagnosis (or symptoms in the absence of a diagnosis) should be recorded on the adverse experiences form utility the same terminology.

Drug Name	Unit Dose (e.g. 500mg)	Freq. (e.g. bid, prn)	Route	Medical illness/ diagnosis (or symptom in absence of diagnosis	Start date(be precise as possible) Day Mth Yr	End date Day Mth Yr

LEAP 0104	Centre Number	Subject Number	Subject Initials

CONCOMITANT MEDICATION (Excluding Ambisome medication)

Please record all baseline medications and all concomitant medication taken during the study.
Mark box below or complete page with concomitant medication details.

☐ Please mark box if no concomitant medication is being used.

Any new medical illness/diagnosis (or symptoms in the absence of a diagnosis) should be recorded on the adverse experiences form utility the same terminology.

Drug Name	Unit Dose (e.g. 500mg)	Freq. (e.g. bid, prn)	Route	Medical illness/ diagnosis (or symptom in absence of diagnosis	Start date(be precise as possible) Day Mth Yr	End date Day Mth Yr

LEAP 0104	Centre Number	Subject Number	Subject Initials

FORM D

Cause of Death:______________________________________

Date of Death: [ ] [ ] [ ] → Complete adverse experience form with regard to death:
Day Mth Yr

Was a post-mortem examination carried out?

☐ No

☐ Yes → If 'Yes', please summarise findings (including diagnosis):

____

____

____

____

____

____

____

____

Physician's Name ___________________________ Date [ ] [ ] [ ]

Signature ___________________________

LEAP 0104	Centre Number	Subject Number	Subject Initials

STUDY CONCLUSION- Early withdrawal ONLY

Please give the reason for withdrawal by marking the one most appropriate category below: Then complete the date of last dose, date of last visit and assessment of clinical response.

1 ☐ Adverse experience, (complete adverse experience section)

2 ☐ Lack of response

3 ☐ Deviation from protocol (including non-compliance)

4 ☐ Lost to follow-up

5 ☐ Termination by sponsor

6 ☐ Other (give details)____

Date of last visit: ☐☐☐ Day Mth Yr   Date of last dose: ☐☐☐ Day Mth Yr

LEAP 0104	Centre Number	Subject Number	Subject Initials

TREATMENT WITH AMBISOME

Did this patient require rescue medication? Yes/No If yes please fill in the table below details of Ambisome medication.

Please record all other Anti-Leishmanial drugs taken during the study.

☐ Please mark box if Ambisome medication was not given.

Ambisome	Unit Dose (e.g. 2mg)	Freq.(e.g. od, bid)	Route	Reason	Start date(be precise as possible) Day Mth Yr	End date Day Mth Yr

LEAP 0104	Centre Number	Subject Number	Subject Initials

ADVERSE EXPERIENCE REPORT FORM (NON SERIOUS)

Record any adverse experiences (using standard medical terminology) Please provide the diagnosis not symptoms where possible. One adverse experience per column.
If no adverse experiences during study, please mark this box ☐ and sign form below

**Adverse Experience**		
	Day Mth Yr	Day Mth Yr
Onset date		
End Date		
Outcome	☐ Resolved<br>☐ Ongoing	☐ Resolved<br>☐ Ongoing
Intensity (maximum)	☐ Mild<br>☐ Moderate<br>☐ Severe	☐ Mild<br>☐ Moderate<br>☐ Severe
Action taken with respect to investigational drug	☐ None<br>☐ Dose reduced<br>☐ Drug interrupted/reduced<br>☐ Drug stopped	☐ None<br>☐ Dose reduced<br>☐ Drug interrupted/restarted<br>☐ Drug stopped.

LEAP 0104	Centre Number	Subject Number	Subject Initials

ADVERSE EXPERIENCE REPORT FORM (NON SERIOUS) Cont.

Relationship to investigational drug	☐ Not related<br>☐ Unlikely<br>☐ possible<br>☐ Probable	☐ Not related<br>☐ Unlikely<br>☐ Possible<br>☐ Probable
Corrective Therapy<br>If 'yes', please record in Concomitant Medication section.	☐ Yes ☐ No	☐ Yes ☐ No
Was the subject withdrawn due to this specific AE?	☐ Yes ☐ No	☐ Yes ☐ No

Investigator's Name ______________________________

Signature:______________________________

LEAP 0104	Centre Number	Subject Number	Subject Initials

ADVERSE EXPERIENCE REPORT FORM (NON SERIOUS)

Record any adverse experiences (using standard medical terminology) Please provide the diagnosis not symptoms where possible. One adverse experience per column.<br>If no adverse experiences during study, please mark this box ☐ and sign form below		
**Adverse Experience**		
	Day Mth Yr	Day Mth Yr
Onset date	☐☐☐	☐☐☐
End Date	☐☐☐	☐☐☐
Outcome	☐ Resolved<br>☐ Ongoing	☐ Resolved<br>☐ Ongoing
Intensity (maximum)	☐ Mild<br>☐ Moderate<br>☐ Severe	☐ Mild<br>☐ Moderate<br>☐ Severe
Action taken with respect to investigational drug	☐ None<br>☐ Dose reduced<br>☐ Drug interrupted/reduced<br>☐ Drug stopped	☐ None<br>☐ Dose reduced<br>☐ Drug interrupted/restarted<br>☐ Drug stopped.

LEAP 0104	Centre Number	Subject Number	Subject Initials

ADVERSE EXPERIENCE REPORT FORM (NON SERIOUS) Cont.

Relationship to investigational drug	☐ Not related<br>☐ Unlikely<br>☐ possible<br>☐ Probable	☐ Not related<br>☐ Unlikely<br>☐ Possible<br>☐ Probable
Corrective Therapy<br>If 'yes', please record in Concomitant Medication section.	☐ Yes ☐ No	☐ Yes ☐ No
Was the subject withdrawn due to this specific AE?	☐ Yes ☐ No	☐ Yes ☐ No

Investigator's Name ______________________________

Signature:______________________________

DNDi
Drugs for Neglected Diseases *initiative*

**LEAP 0104**	Centre Number	Subject Number	Subject Initials

ADVERSE EXPERIENCE REPORT FORM (NON SERIOUS)

Record any adverse experiences (using standard medical terminology) Please provide the diagnosis not symptoms where possible. One adverse experience per column.<br>If no adverse experiences during study, please mark this box ☐ and sign form below		
**Adverse Experience**		
	Day Mth Yr	Day Mth Yr
Onset date	☐☐☐	☐☐☐
End Date	☐☐☐	☐☐☐
Outcome	☐ Resolved<br>☐ Ongoing	☐ Resolved<br>☐ Ongoing
Intensity (maximum)	☐ Mild<br>☐ Moderate<br>☐ Severe	☐ Mild<br>☐ Moderate<br>☐ Severe
Action taken with respect to investigational drug	☐ None<br>☐ Dose reduced<br>☐ Drug interrupted/reduced<br>☐ Drug stopped	☐ None<br>☐ Dose reduced<br>☐ Drug interrupted/restarted<br>☐ Drug stopped.

LEAP 0104	Centre Number	Subject Number	Subject Initials

ADVERSE EXPERIENCE REPORT FORM (NON SERIOUS) Cont.

Relationship to investigational drug	☐ Not related<br>☐ Unlikely<br>☐ possible<br>☐ Probable	☐ Not related<br>☐ Unlikely<br>☐ Possible<br>☐ Probable
Corrective Therapy<br>If 'yes', please record in Concomitant Medication section.	☐ Yes ☐ No	☐ Yes ☐ No
Was the subject withdrawn due to this specific AE?	☐ Yes ☐ No	☐ Yes ☐ No

Investigator's Name ____

Signature:____

LEAP 0104	Centre Number	Subject Number	Subject Initials

ADVERSE EXPERIENCE REPORT FORM (NON SERIOUS)

Record any adverse experiences (using standard medical terminology) Please provide the diagnosis not symptoms where possible. One adverse experience per column.
If no adverse experiences during study, please mark this box ☐ and sign form below

**Adverse Experience**		
	Day Mth Yr	Day Mth Yr
Onset date	☐☐☐	☐☐☐
End Date	☐☐☐	☐☐☐
Outcome	☐ Resolved<br>☐ Ongoing	☐ Resolved<br>☐ Ongoing
Intensity (maximum)	☐ Mild<br>☐ Moderate<br>☐ Severe	☐ Mild<br>☐ Moderate<br>☐ Severe
Action taken with respect to investigational drug	☐ None<br>☐ Dose reduced<br>☐ Drug interrupted/reduced<br>☐ Drug stopped	☐ None<br>☐ Dose reduced<br>☐ Drug interrupted/restarted<br>☐ Drug stopped.

LEAP 0104	Centre Number	Subject Number	Subject Initials

ADVERSE EXPERIENCE REPORT FORM (NON-SERIOUS) Cont.

Relationship to investigational drug	☐ Not related<br>☐ Unlikely<br>☐ possible<br>☐ Probable	☐ Not related<br>☐ Unlikely<br>☐ Possible<br>☐ Probable
Corrective Therapy<br>If 'yes', please record in Concomitant Medication section.	☐ Yes ☐ No	☐ Yes ☐ No
Was the subject withdrawn due to this specific AE?	☐ Yes ☐ No	☐ Yes ☐ No

Investigator's Name ______________________________

Signature:______________________________

LEAP 0104	Centre Number	Subject Number	Subject Initials

ADVERSE EXPERIENCE REPORT FORM (NON-SERIOUS)

Record any adverse experiences (using standard medical terminology) Please provide the diagnosis not symptoms where possible. One adverse experience per column.
If no adverse experiences during study, please mark this box ☐ and sign form below

**Adverse Experience**		
	Day Mth Yr	Day Mth Yr
Onset date	☐ ☐ ☐	☐ ☐ ☐
End Date	☐ ☐ ☐	☐ ☐ ☐
Outcome	☐ Resolved<br>☐ Ongoing	☐ Resolved<br>☐ Ongoing
Intensity (maximum)	☐ Mild<br>☐ Moderate<br>☐ Severe	☐ Mild<br>☐ Moderate<br>☐ Severe
Action taken with respect to investigational drug	☐ None<br>☐ Dose reduced<br>☐ Drug interrupted/reduced<br>☐ Drug stopped	☐ None<br>☐ Dose reduced<br>☐ Drug interrupted/restarted<br>☐ Drug stopped.

LEAP 0104	Centre Number	Subject Number	Subject Initials

ADVERSE EXPERIENCE REPORT FORM (NON-SERIOUS) Cont.

Relationship to investigational drug	☐ Not related<br>☐ Unlikely<br>☐ possible<br>☐ Probable	☐ Not related<br>☐ Unlikely<br>☐ Possible<br>☐ Probable
Corrective Therapy<br>If 'yes', please record in Concomitant Medication section.	☐ Yes ☐ No	☐ Yes ☐ No
Was the subject withdrawn due to this specific AE?	☐ Yes ☐ No	☐ Yes ☐ No

Investigator's Name ____

Signature:____

LEAP 0104	Centre Number	Subject Number	Subject Initials

ADVERSE EXPERIENCE REPORT FORM (NON SERIOUS)

Record any adverse experiences (using standard medical terminology) Please provide the diagnosis not symptoms where possible. One adverse experience per column.
If no adverse experiences during study, please mark this box ☐ and sign form below

**Adverse Experience**		
	Day Mth Yr	Day Mth Yr
Onset date	☐☐☐	☐☐☐
End Date	☐☐☐	☐☐☐
Outcome	☐ Resolved<br>☐ Ongoing	☐ Resolved<br>☐ Ongoing
Intensity (maximum)	☐ Mild<br>☐ Moderate<br>☐ Severe	☐ Mild<br>☐ Moderate<br>☐ Severe
Action taken with respect to investigational drug	☐ None<br>☐ Dose reduced<br>☐ Drug interrupted/reduced<br>☐ Drug stopped	☐ None<br>☐ Dose reduced<br>☐ Drug interrupted/restarted<br>☐ Drug stopped.

LEAP 0104	Centre Number	Subject Number	Subject Initials

ADVERSE EXPERIENCE REPORT FORM (NON SERIOUS) Cont.

Relationship to investigational drug	☐ Not related<br>☐ Unlikely<br>☐ possible<br>☐ Probable	☐ Not related<br>☐ Unlikely<br>☐ Possible<br>☐ Probable
Corrective Therapy<br>If 'yes', please record in Concomitant Medication section.	☐ Yes ☐ No	☐ Yes ☐ No
Was the subject withdrawn due to this specific AE?	☐ Yes ☐ No	☐ Yes ☐ No

Investigator's Name ______________________________

Signature:______________________________

LEAP 0104	Centre Number	Subject Number	Subject Initials

ADVERSE EXPERIENCE REPORT FORM (NON SERIOUS)

Record any adverse experiences (using standard medical terminology) Please provide the diagnosis not symptoms where possible. One adverse experience per column.
If no adverse experiences during study, please mark this box ☐ and sign form below

**Adverse Experience**		
	Day Mth Yr	Day Mth Yr
Onset date	☐☐☐	☐☐☐
End Date	☐☐☐	☐☐☐
Outcome	☐ Resolved<br>☐ Ongoing	☐ Resolved<br>☐ Ongoing
Intensity (maximum)	☐ Mild<br>☐ Moderate<br>☐ Severe	☐ Mild<br>☐ Moderate<br>☐ Severe
Action taken with respect to investigational drug	☐ None<br>☐ Dose reduced<br>☐ Drug interrupted/reduced<br>☐ Drug stopped	☐ None<br>☐ Dose reduced<br>☐ Drug interrupted/restarted<br>☐ Drug stopped.

LEAP 0104	Centre Number	Subject Number	Subject Initials

ADVERSE EXPERIENCE REPORT FORM (NON-SERIOUS) Cont.

Relationship to investigational drug	☐ Not related<br>☐ Unlikely<br>☐ possible<br>☐ Probable	☐ Not related<br>☐ Unlikely<br>☐ Possible<br>☐ Probable
Corrective Therapy<br>If 'yes', please record in Concomitant Medication section.	☐ Yes ☐ No	☐ Yes ☐ No
Was the subject withdrawn due to this specific AE?	☐ Yes ☐ No	☐ Yes ☐ No

Investigator's Name ____________________

Signature:____________________

DNDi
Drugs for Neglected Diseases *initiative*

LEAP 0104	Centre Number	Subject Number	Subject Initials

6 months follow-up

VISCERAL LEISHMANIASIS SEVERITY ASSESSMENTS
Clinical Examination

Weight: ☐☐☐ Kg
(Light clothes, rounded up to the nearest Kg)
Blood Pressure:
(After 5mins,sitting) ☐☐☐ / ☐☐☐ mmHg

Heart Rate: ☐☐☐ bpm

Axillary Temperature: ☐☐ • ☐ °C

Indicate **present or absent** for each of the characteristics below

**Characteristics**	Present	Absent
Mucosal pallor		
Jaundice		
Cervical lymphadenopathy		
Axillary lymphadenopathy		
Inguinal lymphadenopathy		
Muscle Wasting		
Petechial haemorrhages		
Other (specify)		

Abdominal palpation

Abdominal palpation for spleen size (cm) (by palpation below left costal margin in the anterior axillary line) ____________________cm

Abdominal palpation for liver size (cm) (by palpation below right costal margin in the mid-clavicular line) ___________cm

LEAP 0104	Centre Number	Subject Number	Subject Initials

6 months follow-up

12-Lead Electrocardiographic Examination

Date performed: [ ][ ][ ]
Day Mth Yr

Please describe the patient's12-lead electrocardiogram by ticking one of the boxes below:

☐ Normal / no clinically significant abnormality

☐ Clinically significant abnormality → Record a summary of significant findings in the space provided below:

____

____

____

____

Audiometric Examination

Date performed: [ ][ ][ ]
Day Mth Yr

Please describe the patients' audiometry by ticking one of the following boxes below

☐ Normal/no clinically significant abnormality.

☐ Clinically significant abnormality → Record a summary of significant findings in the space provided below:

____

____

____

____

Drugs for Neglected Diseases *initiative*

LEAP 0104	Centre Number	Subject Number	Subject Initials

6 months follow-up

Haematologic Examination

Date the sample Analysed	Day Mth Yr	
Laboratory Test	Units	Value
Haemoglobin		
WBC (total)		
Platelets		

Parasitologic Examination

Date sample was taken ____________________

Spleen/bone Marrow /lymphnode aspirate (Leishmania Index).Enter the appropriate index in the box provided.

6+ ☐

5+ ☐

4+ ☐

3+ ☐

2+ ☐

1+ ☐

0 ☐

***Bone marrow to be done in cases where lymph node or spleen is not palpable**.

LEAP 0104	Centre Number	Subject Number	Subject Initials

6 months follow-up
Clinical Chemistry Examination

Date sample Analysed	Day Mth Yr	
Laboratory Test	Units	Value
SGOT/AST		
SGPT/ALT		
Alkaline Phosphatase		
Albumin		
Globulin		
Prothrombin Time		
Creatinine		
BUN		
Sodium		
Potassium		
Amylase		
Others		

LEAP 0104	Centre Number	Subject Number	Subject Initials

6 months follow-up

Urinalysis (Dipstick Test and Microscopy)

**Dipstick Tests**	**Values/Units**
Colour	
Specific Gravity	
pH	
Blood	
Glucose	
Ketones	
Protein	
**Microscopy**	**PER HIGH POWER FIELD**
White Blood cell count	
Red blood cell count	
Casts (Specify)	
Bacteria	
Others	

LEAP 0104	Centre Number	Subject Number	Subject Initials

6 months follow-up

Adverse Experiences

Are there any adverse events from the previous follow up to be carried forward?

No ☐ YES ☐

If YES Please record the details in the adverse events section.

Has this patient experienced any new adverse events since the last assessment

NO ☐ YES ☐

If YES Please record the details in the adverse events section.

Please note, for every adverse event requiring treatment, fill in the "Concomitant medication" section.

Assessment of Treatment Response

Based on the clinical/physical assessment at the screening visit, mark the box that best describes or boxes that best describe the subject's clinical outcome so far:

CLINICAL RESPONSE:

COMPLETE	☐	Resolution of all signs and symptoms of VL
PARTIAL	☐	Some signs or symptoms of VL persist

PARASITOLOGICAL RESPONSE:

**COMPLETE PARASITE CLEARANCE**	☐	-Smear negative after counting 1000 fields<br>-Oil immersion (x100)
**PARTIAL PARASITE CLEARANCE (SLOW RESPONDER)**	☐	Smear positive but ≥99%parasite reduction<br>[≥2 log reduction] compared with pre-treatment slide.
**PERSISTANT PARASITAEMIA (TREATMENT FAILURE)**	☐	Smear positive and <99% parasite reduction<br>[<2 log reduction] compared with pre-treatment slide.

Investigator's name ________________ **Date** _________________

Signature ________________________

LEAP 0104	Centre Number	Subject Number	Subject Initials

6 months follow-up

CONCOMITANT MEDICATION (Excluding Ambisome medication)

☐ Please mark box if no new concomitant medications are being used during this follow-up.

Please record all concomitant medications carried forward if any from the last follow-up and all other new concomitant medications taken since the last follow-up in the spaces provided below.

Any new medical illness/diagnosis (or symptoms in the absence of a diagnosis) should be recorded on the adverse experiences form utility the same terminology.

Drug Name	Unit Dose (e.g. 500 mg)	Freq. (e.g. bid, prn)	Route	Medical illness/ diagnosis (or symptom in absence of diagnosis	Start date(be precise as possible) Day Mth Yr	End date Day Mth Yr

LEAP 0104	Centre Number	Subject Number	Subject Initials

6 months follow-up

CONCOMITANT MEDICATION (Excluding Ambisome medication)

☐ Please mark box if no new concomitant medication are being used during this follow-up.

Please record all concomitant medications carried forward if any from the last follow-up and all other new concomitant medications taken since the last follow-up in the spaces provided below.

Any new medical illness/diagnosis (or symptoms in the absence of a diagnosis) should be recorded on the adverse experiences form utility the same terminology.

Drug Name	Unit Dose (e.g. 500 mg)	Freq. (e.g. bid, prn)	Route	Medical illness/ diagnosis (or symptom in absence of diagnosis	Start date(be precise as possible) Day Mth Yr	End date Day Mth Yr

DNDi
Drugs for Neglected Diseases *initiative*

LEAP 0104	Centre Number	Subject Number	Subject Initials

6 months follow-up

FORM D

Cause of Death:________________________________

Date of Death: [ ][ ][ ] Day Mth Yr → Complete serious adverse experience form with regard to death:

Was a post-mortem examination carried out?

☐ No

☐ Yes → If 'Yes', please summarise findings (including diagnosis):

____

____

____

____

____

____

____

____

Physician's Name ______________________ Date [ ][ ][ ]

Signature ______________________

LEAP 0104	Centre Number	Subject Number	Subject Initials

6 months follow-up

STUDY CONCLUSION- Early withdrawal ONLY

Please give the reason for withdrawal by marking the one most appropriate category below: Then complete the date of last dose, date of last visit and assessment of clinical response.

1 ☐ Adverse experience, (complete adverse experience section)

2 ☐ Lack of response

3 ☐ Deviation from protocol (including non-compliance)

4 ☐ Lost to follow-up

5 ☐ Termination by sponsor

6 ☐ Other (give details)____

Date of last visit: ☐☐☐
Day Mth Yr

Drugs for Neglected Diseases *initiative*

LEAP 0104	Centre Number	Subject Number	Subject Initials

6 months follow-up

TREATMENT WITH AMBISOME

Did this patient require rescue medication? Yes/No If yes please fill in the table below details of Ambisome medication.

☐ Please mark box if Ambisome medication was not given.

Please record in the spaces provided below if AmBisome was given.

Ambisome	Unit Dose (e.g. 2mg)	Freq. (e.g. od, bid)	Route	Reason	Start date(be precise as possible)<br>Day Mth Yr	End date<br>Day Mth Yr

DNDi
Drugs for Neglected Diseases *initiative*

LEAP 0104	Centre Number	Subject Number	Subject Initials

6 months follow-up

ADVERSE EXPERIENCE REPORT FORM (NON SERIOUS)

☐ If no new adverse experiences during this follow-up, please mark this box and sign form below.

Record any adverse experiences (both carried forward if any and/or new ones),using standard medical terminology. Please provide the diagnosis not symptoms where possible. One adverse experience per column.

**Adverse Experience**		
	Day Mth Yr	Day Mth Yr
Onset date	☐☐☐	☐☐☐
End Date	☐☐☐	☐☐☐
Outcome	☐ Resolved<br>☐ Ongoing	☐ Resolved<br>☐ Ongoing
Intensity (maximum)	☐ Mild<br>☐ Moderate<br>☐ Severe	☐ Mild<br>☐ Moderate<br>☐ Severe
Action taken with respect to investigational drug	☐ None<br>☐ Dose reduced<br>☐ Drug interrupted/reduced<br>☐ Drug stopped	☐ None<br>☐ Dose reduced<br>☐ Drug interrupted/restarted<br>☐ Drug stopped.

DNDi
Drugs for Neglected Diseases *initiative*

LEAP 0104	Centre Number	Subject Number	Subject Initials

6 months follow-up

ADVERSE EXPERIENCES (NON_SERIOUS) Cont.

Relationship to investigational drug	☐ Not related<br>☐ Unlikely<br>☐ Suspected (reasonably possible)<br>☐ Probable	☐ Not related<br>☐ Unlikely<br>☐ Suspected (reasonably possible)<br>☐ Probable
Corrective Therapy<br>If 'yes', please record Concomittant Medication section and /or Resource utilization section if appropiriate.	☐ Yes ☐ No	☐ Yes ☐ No
Was the subject withdrawn due to this specific AE?	☐ Yes ☐ No	☐ Yes ☐ No

Investigator's Name:____

Signature:____

Drugs for Neglected Diseases *initiative*

LEAP 0104	Centre Number	Subject Number	Subject Initials

6 months follow-up

ADVERSE EXPERIENCE REPORT FORM (NON SERIOUS)

☐ If no new adverse experiences during this follow-up, please mark this box and sign form below.

Record any adverse experiences (both carried forward if any and/or new ones),using standard medical terminology. Please provide the diagnosis not symptoms where possible. One adverse experience per column.

**Adverse Experience**		
	Day Mth Yr	Day Mth Yr
Onset date		
End Date		
Outcome	☐ Resolved<br>☐ Ongoing	☐ Resolved<br>☐ Ongoing
Intensity (maximum)	☐ Mild<br>☐ Moderate<br>☐ Severe	☐ Mild<br>☐ Moderate<br>☐ Severe
Action taken with respect to investigational drug	☐ None<br>☐ Dose reduced<br>☐ Drug interrupted/reduced<br>☐ Drug stopped	☐ None<br>☐ Dose reduced<br>☐ Drug interrupted/restarted<br>☐ Drug stopped.

LEAP 0104	Centre Number	Subject Number	Subject Initials

6 months follow-up

ADVERSE EXPERIENCES (NON_SERIOUS) Cont.

Relationship to investigational drug	☐ Not related<br>☐ Unlikely<br>☐ Suspected (reasonably possible)<br>☐ Probable	☐ Not related<br>☐ Unlikely<br>☐ Suspected (reasonably possible)<br>☐ Probable
Corrective Therapy<br>If 'yes', please record Concomittant Medication section and /or Resource utilization section if appropiriate.	☐ Yes ☐ No	☐ Yes ☐ No
Was the subject withdrawn due to this specific AE?	☐ Yes ☐ No	☐ Yes ☐ No

Investigator's Name:____

Signature:____

16.1.2 List of IECs and patient information and consent forms

Appendix 3 List of IECs

LIST OF ETHICS COMMITTEES FOR LEAP 0104 A

SUDAN

1. Ethics Committee for the Federal Ministry of Health
2. Institute of Endemic Diseases, University of Khartoum
3. MSF Ethics Review Board Ethics Committee,

ETHIOPIA

1. University of Addis Ababa Ethics Committee
2. Gondar Ollege of Medical Sciences Institutional Ethics Committee
3. Awasa Regional Ethics Committee
4. Bahir-Dar Regional Ethics Committee
5. National Ethical Clearance Committee

KENYA

1. Scientific Review Committee, KEMRI
2. Kenya Medical Research Institute Ethics Committee

Appendix 4 Samples of patient information sheets and consent forms

See Appendix 1

16.1.3 List of investigators and other study personnel

Appendix 5 List of principal and co-investigators, affiliations and roles#

Centre 11 (Gondar) and 12 (Arba Minch)

NAME	INSTITUTION	RESPONSIBILITY
Asrat Hailu	University of Addis Ababa	Principle Investigator
Eyasu Makonnen	University of Addis Ababa	Investigator
Yalemtsehay Mekonnen	University of Addis Ababa	Investigator
Afewesen Gebre-Yohannes	Gondar University Hospital	Investigator
Sisay Yifru	Gondar University Hospital	Investigator
Abiye Tesfaye	Gondar University Hospital	Investigator
Nurelign Gashu	Gondar University Hospital	Investigator
Samson Tesfaye	Arba Minch Hospital	Site Investigator
Yewubnesh Hailu	Arba Minch Hospital	Investigator
Degu Jerene	Arba Minch Hospital	Investigator

Centre 23: Kenya

NAME	INSTITUTION	RESPONSIBILITY
Monique Wasunna	KEMRI	Principle Investigator
Juma Rashid	KEMRI	Investigator
J. Mbui	KEMRI	Investigator
G. Mucee	KEMRI	Investigator
V. Manduku	KEMRI	Investigator
A. Musibi	KEMRI	Investigator
Z. Mutuma	KEMRI	Investigator
F. Kirui	KEMRI	Investigator
H. Lodenyo	KEMRI	Investigator
K. Bhatt	University of Nairobi	Investigator

Centre 34: Um el Kher

NAME	INSTITUTION	RESPONSIBILITY
Manica Balasegaram	MSF	Principle Investigator
Marius Mueller	MSF	Investigator
Peter Young	MSF	Investigator
Yousif Koummuki	MSF	Investigator
Thomas Allam	MSF	Investigator
Omer Elamin Hassan	MSF	Investigator
Koert Ritmeijer	MSF	Investigator

Centre 35: Kassab

NAME	INSTITUTION	RESPONSIBILITY
Musa Amudawi	IEND	Principle Investigator
A. M. ElHassan	IEND	Investigator
M. E. Ebrahim	IEND	Investigator
I.M. ElHassan	IEND	Investigator
Ahmed Abdalla	Gedarif University Sudan	Investigator
Fawzi Abdeirahim Mahjoub	National Ribat University	Investigator

Appendix 6 CVs of principal and co-investigators

See Appendix 5 for list of Investigators and Trial Master file for signed and dated CVs

Appendix 7 List of DSMB members and their affiliation

Dr. Faiza Osman Mohammed, Institute of Endemic Diseases, University of Khartoum, SUDAN

Dr. Phelgona Otieno, Centre for Clinical Research, Kenya Medical Research Institute, KENYA

Dr. Khalid Abd ElMutalab ElMardi, National Malaria Control Programme, Federal Ministry of Health, SUDAN

Dr. Nuha Hamid Mahamoud, World Health Organization (WHO), SUDAN

Appendix 8 List of monitors

LEAP 0104A

Dr. Robert Balikuddembe, Makerere University, UGANDA
Dr. Sarah Nanzigu, Makerere University, UGANDA
Dr. Shibru Berhum, ETHIOPIA
Dr. Hildah O'hara, KEMRI, KENYA
Dr. Lydia Kivihya, KEMRI, KENYA
Dr. Isaiah Mwangi, Centre for Respiratory Diseases, Kenya Medical Research Institute, KENYA
Dr. Mona Elfakii Eltahir, Institute of Endemic Diseases, University of Khartoum, SUDAN

16.2.4 Signatures of principal or coordinating investigator(s) or sponsor's responsible medical officer

Appendix 9 Signatures

Sponsor (DNDi)	Name	
Sponsor<br>Senior Project Manager	Country	Switzerland
	Phone	+41 22 906 9230
	E-mail	
	Fax	
Signature	Date of Signature (ddmmyyyy)	Time (24-hour clock, place)

Kenya Medical Research Research Institute	Name	Dr. Monique Wasunna
Medical Coordinator / Head of DNDi Africa /LEAP Data centre	Country	Kenya
	Phone	+254 20 273 0076
	E-mail	africa@dndi.org
	Fax	
Signature	Date of Signature: (ddmmmyyy)	Time (24-hour clock, place)

Faculty of Medicine<br>Addis Ababa University	Name	Prof. Asrat Hailu
Principal investigator	Country	Ethiopia
	Phone	+251-911-480993 (mobile)<br>+251-115-533197 (office)
	E-mail	hailu_a2004@yahoo.com
	Fax	+251-115-517701
Signature	Date of Signature (ddmmyyyy)	Time (24-hour clock, place)

Institute of Endemic Diseases, University of Khartoum, Sudan	Name	Prof. Eltahir Khalil
Principal investigator	Country	Sudan
	Phone	+249-9-123-75740 (office)
	E-mail	eltahirgasim@yahoo.ca
	Fax	
Signature	Date of Signature (ddmmyyyy)	Time (24-hour clock, place)

Institute of Endemic Diseases, University of Khartoum, Sudan	Name	Dr Ahmed Musa
Site Lead Investigator (Kassab site)	Country	Sudan
	Phone	+249-9-123-75740 (office)
	E-mail	
	Fax	
Signature	Date of Signature (ddmmyyyy)	Time (24-hour clock, place)

MSF-Holland	Name	Dr Manica Balasegaram
Site Lead Investigator (Um el Kher site)	Country	Switzerland
	Phone	+41 22 906 9230
	E-mail	mbalasegaram@dndi.org
	Fax	
Signature	Date of Signature (ddmmyyyy)	Time (24-hour clock, place)

London School of Hygiene and Medicine	Name	Tansy Edwards (Statistician)
Statistician	Country	United Kingdom
	Phone	+44 (20) 7927 2240
	E-mail	tansy.edwards@lshtm.ac.uk
	Fax	
Signature	Date of Signature (ddmmyyyy)	Time (24-hour clock, place)

16.2.5 Listing of patients receiving test drugs from specific batches, where more than one batch was used

Appendix 10 Listing of patients per allocated treatment and drug batch

Paromomycin

Paromomycin		Centre	Patient numbers
Batch Number	Expiry date		
FB301X	1 Sep 2006	Gondar (11)<br>Arba Minch (12)<br>KEMRI (23)<br>Um el Kher (34)<br>Kassab (35)	001- 090<br>241 - 330<br>361 - 405<br>451 - 540<br>646 - 690
FB501X	1 Jan 2008	Gondar (11)<br>Arba Minch (12)	091-135<br>241 - 330

Sodium Stibogluconate

Sodium Stibogluconate		Centre	Patient numbers
Batch Number	Expiry date		
4P12004	Feb 2007	Gondar (11)<br>Arba Minch (12)<br>KEMRI (23)<br>Um el Kher (34)<br>Kassab (35)	001- 135<br>241 – 330<br>361 – 405<br>451 – 540<br>646 - 690
4P12010	Mar 2007	Gondar (11)	001- 135
5P12036	Aug 2007	Gondar (11)<br>Arba Minch (12)	001- 135<br>241 – 330
5P12037	Aug 2007	Gondar (11)<br>Arba Minch (12)	001- 135<br>241 – 330
6P12001	Feb 2008	Arba Minch (12)	241 - 330

16.2.6 Randomisation scheme and codes

Appendix 11 Randomisation master list

Randomization List for Gondar- Ethiopia

COUNTRY	SITE	PATIENT NUMBER	DRUG	COUNTRY	SITE	PATIENT NUMBER	DRUG
1	1	1	1	1	1	55	1
1	1	2	3	1	1	56	2
1	1	3	3	1	1	57	3
1	1	4	1	1	1	58	3
1	1	5	1	1	1	59	1
1	1	6	3	1	1	60	3
1	1	7	1	1	1	61	1
1	1	8	2	1	1	62	2
1	1	9	2	1	1	63	2
1	1	10	2	1	1	64	2
1	1	11	3	1	1	65	1
1	1	12	3	1	1	66	3
1	1	13	2	1	1	67	1
1	1	14	2	1	1	68	2
1	1	15	1	1	1	69	3
1	1	16	3	1	1	70	3
1	1	17	3	1	1	71	3
1	1	18	2	1	1	72	2
1	1	19	3	1	1	73	1
1	1	20	2	1	1	74	1
1	1	21	2	1	1	75	3
1	1	22	2	1	1	76	1
1	1	23	1	1	1	77	1
1	1	24	1	1	1	78	2
1	1	25	2	1	1	79	1
1	1	26	3	1	1	80	1
1	1	27	1	1	1	81	3
1	1	28	3	1	1	82	2
1	1	29	1	1	1	83	2
1	1	30	1	1	1	84	2
1	1	31	2	1	1	85	2
1	1	32	3	1	1	86	3
1	1	33	2	1	1	87	3
1	1	34	1	1	1	88	1
1	1	35	2	1	1	89	3
1	1	36	2	1	1	90	3

COUNTRY	SITE	PATIENT NUMBER	DRUG	COUNTRY	SITE	PATIENT NUMBER	DRUG
1	1	37	3	1	1	91	1
1	1	38	1	1	1	92	3
1	1	39	1	1	1	93	2
1	1	40	1	1	1	94	2
1	1	41	3	1	1	95	2
1	1	42	3	1	1	96	3
1	1	43	2	1	1	97	3
1	1	44	1	1	1	98	1
1	1	45	3	1	1	99	2
1	1	46	2	1	1	100	1
1	1	47	2	1	1	101	1
1	1	48	3	1	1	102	3
1	1	49	2	1	1	103	3
1	1	50	1	1	1	104	1
1	1	51	3	1	1	105	2
1	1	52	1	1	1	106	3
1	1	53	2	1	1	107	1
1	1	54	1	1	1	108	1
1	1	109	2	1	1	165	3
1	1	110	1	1	1	166	1
1	1	111	3	1	1	167	3
1	1	112	3	1	1	168	2
1	1	113	2	1	1	169	3
1	1	114	2	1	1	170	2
1	1	115	3	1	1	171	1
1	1	116	2	1	1	172	3
1	1	117	1	1	1	173	1
1	1	118	1	1	1	174	3
1	1	119	2	1	1	175	1
1	1	120	3	1	1	176	2
1	1	121	3	1	1	177	3
1	1	122	2	1	1	178	1
1	1	123	2	1	1	179	2
1	1	124	2	1	1	180	2
1	1	125	2	1	1	181	1
1	1	126	3	1	1	182	2
1	1	127	1	1	1	183	2
1	1	128	3	1	1	184	3
1	1	129	1	1	1	185	1
1	1	130	3	1	1	186	1
1	1	131	1	1	1	187	1

COUNTRY	SITE	PATIENT NUMBER	DRUG	COUNTRY	SITE	PATIENT NUMBER	DRUG
1	1	132	2	1	1	188	3
1	1	133	3	1	1	189	3
1	1	134	1	1	1	190	2
1	1	135	1	1	1	191	2
1	1	136	1	1	1	192	1
1	1	137	2	1	1	193	3
1	1	138	2	1	1	194	3
1	1	139	1	1	1	195	2
1	1	140	3	1	1	196	1
1	1	141	3	1	1	197	3
1	1	142	2	1	1	198	3
1	1	143	3	1	1	199	2
1	1	144	1	1	1	200	3
1	1	145	2	1	1	201	2
1	1	146	1	1	1	202	1
1	1	147	1	1	1	203	1
1	1	148	3	1	1	204	3
1	1	149	3	1	1	205	1
1	1	150	2	1	1	206	2
1	1	151	3	1	1	207	3
1	1	152	2	1	1	208	1
1	1	153	1	1	1	209	2
1	1	154	2	1	1	210	2
1	1	155	2	1	1	211	3
1	1	156	3	1	1	212	2
1	1	157	3	1	1	213	1
1	1	158	1	1	1	214	1
1	1	159	2	1	1	215	2
1	1	160	1	1	1	216	3
1	1	161	2	1	1	217	2
1	1	162	1	1	1	218	2
1	1	163	3	1	1	219	2
1	1	164	1	1	1	220	1
1	1	221	3	1	1	231	2
1	1	222	3	1	1	232	2
1	1	223	1	1	1	233	1
1	1	224	3	1	1	234	1
1	1	225	1	1	1	235	3
1	1	226	3	1	1	236	1
1	1	227	2	1	1	237	2
1	1	228	3	1	1	238	2

COUNTRY	SITE	PATIENT NUMBER	DRUG	COUNTRY	SITE	PATIENT NUMBER	DRUG
1	1	229	3	1	1	239	1
1	1	230	3	1	1	240	1

Number of cases read: 240 Number of cases listed: 240

		Frequency	Percent	Valid Percent	Cumulative Percent
Valid	1 PM	80	33.3	33.3	33.3
	2 SSG	80	33.3	33.3	66.7
	3 SSG+PM	80	33.3	33.3	100.0
	Total	240	100.0	100.0	

Randomization List for Arba Minch – Ethiopia

COUNTRY	SITE	PATIENT NUMBER	DRUG	COUNTRY	SITE	PATIENT NUMBER	DRUG
1	2	241	3	1	2	295	3
1	2	242	2	1	2	296	1
1	2	243	1	1	2	297	2
1	2	244	2	1	2	298	1
1	2	245	1	1	2	299	3
1	2	246	1	1	2	300	1
1	2	247	1	1	2	301	2
1	2	248	1	1	2	302	2
1	2	24 9	3	1	2	303	1
1	2	250	2	1	2	304	3
1	2	251	3	1	2	305	1
1	2	252	2	1	2	306	3
1	2	253	2	1	2	307	3
1	2	254	3	1	2	308	1
1	2	255	3	1	2	309	1
1	2	256	3	1	2	310	2
1	2	257	2	1	2	311	2
1	2	258	2	1	2	312	3
1	2	259	1	1	2	313	1
1	2	260	1	1	2	314	2
1	2	261	3	1	2	315	3
1	2	262	1	1	2	316	2
1	2	263	2	1	2	317	2
1	2	264	3	1	2	318	3
1	2	265	2	1	2	319	3
1	2	266	2	1	2	320	3
1	2	267	2	1	2	321	3
1	2	268	1	1	2	322	1
1	2	269	3	1	2	323	2
1	2	270	3	1	2	324	2
1	2	271	2	1	2	325	1
1	2	272	1	1	2	326	1
1	2	273	2	1	2	327	3
1	2	274	2	1	2	328	1
1	2	275	3	1	2	329	2
1	2	276	1	1	2	330	1
1	2	277	3	1	2	331	1
1	2	278	2	1	2	332	2
1	2	279	1	1	2	333	3

COUNTRY	SITE	PATIENT NUMBER	DRUG	COUNTRY	SITE	PATIENT NUMBER	DRUG
1	2	280	2	1	2	334	3
1	2	281	3	1	2	335	1
1	2	282	1	1	2	336	2
1	2	283	1	1	2	337	3
1	2	284	3	1	2	338	2
1	2	285	3	1	2	339	1
1	2	286	1	1	2	340	2
1	2	287	2	1	2	341	1
1	2	288	3	1	2	342	2
1	2	289	3	1	2	343	1
1	2	290	2	1	2	344	3
1	2	291	2	1	2	345	3
1	2	292	1	1	2	346	1
1	2	293	2	1	2	347	3
1	2	294	3	1	2	348	2
1	2	349	2	1	2	355	2
1	2	350	3	1	2	356	2
1	2	351	1	1	2	357	1
1	2	352	3	1	2	358	1
1	2	353	3	1	2	359	3
1	2	354	2	1	2	360	1

Number of cases read: 120 Number of cases listed: 120

		Frequency	Percent	Valid Percent	Cumulative Percent
Valid	1 PM	40	33.3	33.3	33.3
	2 SSG	40	33.3	33.3	66.7
	3 SSG+PM	40	33.3	33.3	100.0
	Total	120	100.0	100.0	

Randomization list for Kenya

COUNTRY	SITE	PATIENT NUMBER	DRUG	COUNTRY	SITE	PATIENT NUMBER	DRUG
2	3	361	3	2	3	406	2
2	3	362	1	2	3	407	3
2	3	363	1	2	3	408	1
2	3	364	3	2	3	409	2
2	3	365	2	2	3	410	1
2	3	366	3	2	3	411	1
2	3	367	2	2	3	412	3
2	3	368	1	2	3	413	1
2	3	369	3	2	3	414	2
2	3	370	2	2	3	415	2
2	3	371	1	2	3	416	3
2	3	372	1	2	3	417	3
2	3	373	2	2	3	418	2
2	3	374	2	2	3	419	3
2	3	375	3	2	3	420	1
2	3	376	2	2	3	421	1
2	3	377	3	2	3	422	3
2	3	378	3	2	3	423	2
2	3	379	1	2	3	424	3
2	3	380	2	2	3	425	2
2	3	381	1	2	3	426	1
2	3	382	3	2	3	427	1
2	3	383	1	2	3	428	1
2	3	384	2	2	3	429	3
2	3	385	2	2	3	430	2
2	3	386	3	2	3	431	3
2	3	387	3	2	3	432	1
2	3	388	1	2	3	433	2
2	3	389	2	2	3	434	3
2	3	390	1	2	3	435	2
2	3	391	3	2	3	436	3
2	3	392	3	2	3	437	2
2	3	393	2	2	3	438	1
2	3	394	1	2	3	439	2
2	3	395	1	2	3	440	1
2	3	396	2	2	3	441	1
2	3	397	2	2	3	442	3
2	3	398	3	2	3	443	3
2	3	399	1	2	3	444	2

COUNTRY	SITE	PATIENT NUMBER	DRUG	COUNTRY	SITE	PATIENT NUMBER	DRUG
2	3	400	1	2	3	445	2
2	3	401	3	2	3	446	3
2	3	402	3	2	3	447	3
2	3	403	2	2	3	448	1
2	3	404	2	2	3	449	2
2	3	405	1	2	3	450	1

Number of cases read: 90 Number of cases listed: 90

	Frequency	Percent	Valid Percent	Cumulative Percent
Valid PM	30	33.3	33.3	33.3
SSG	30	33.3	33.3	66.7
SSG+PM	30	33.3	33.3	100.0
Total	90	100.0	100.0	

Randomization List for Um el Kher - Sudan

COUNTRY	SITE	PATIENT NUMBER	DRUG	COUNTRY	SITE	PATIENT NUMBER	DRUG
3	4	451	3	3	4	505	2
3	4	452	1	3	4	506	2
3	4	453	1	3	4	507	2
3	4	454	3	3	4	508	1
3	4	455	1	3	4	509	1
3	4	456	2	3	4	510	3
3	4	457	3	3	4	511	3
3	4	458	1	3	4	512	1
3	4	459	3	3	4	513	3
3	4	460	1	3	4	514	2
3	4	461	2	3	4	515	2
3	4	462	2	3	4	516	1
3	4	463	3	3	4	517	1
3	4	464	2	3	4	518	2
3	4	465	2	3	4	519	1
3	4	466	3	3	4	520	2
3	4	467	1	3	4	521	1
3	4	468	3	3	4	522	3
3	4	469	2	3	4	523	2
3	4	470	3	3	4	524	3
3	4	471	1	3	4	525	3
3	4	472	2	3	4	526	2

COUNTRY	SITE	PATIENT NUMBER	DRUG	COUNTRY	SITE	PATIENT NUMBER	DRUG
3	4	473	1	3	4	527	3
3	4	474	3	3	4	528	1
3	4	475	1	3	4	529	3
3	4	476	1	3	4	530	3
3	4	477	2	3	4	531	2
3	4	478	3	3	4	532	3
3	4	479	2	3	4	533	2
3	4	480	2	3	4	534	1
3	4	481	3	3	4	535	1
3	4	482	3	3	4	536	1
3	4	483	1	3	4	537	2
3	4	484	1	3	4	538	1
3	4	485	3	3	4	539	3
3	4	486	3	3	4	540	2
3	4	487	2	3	4	541	1
3	4	488	2	3	4	542	2
3	4	489	2	3	4	543	1
3	4	490	1	3	4	544	1
3	4	491	1	3	4	545	2
3	4	492	1	3	4	546	3
3	4	493	2	3	4	547	3
3	4	494	3	3	4	548	2
3	4	495	2	3	4	549	2
3	4	496	2	3	4	550	3
3	4	497	1	3	4	551	2
3	4	498	2	3	4	552	1
3	4	499	3	3	4	553	1
3	4	500	1	3	4	554	3
3	4	501	3	3	4	555	3
3	4	502	3	3	4	556	2
3	4	503	1	3	4	557	2
3	4	504	3	3	4	558	3
3	4	559	2	3	4	603	2
3	4	560	1	3	4	604	1
3	4	561	2	3	4	605	1
3	4	562	1	3	4	606	1
3	4	563	1	3	4	607	2
3	4	564	2	3	4	608	3
3	4	565	1	3	4	609	1
3	4	566	3	3	4	610	2
3	4	567	3	3	4	611	2

COUNTRY	SITE	PATIENT NUMBER	DRUG	COUNTRY	SITE	PATIENT NUMBER	DRUG
3	4	568	3	3	4	612	3
3	4	569	3	3	4	613	1
3	4	570	1	3	4	614	3
3	4	571	1	3	4	615	3
3	4	572	3	3	4	616	2
3	4	573	1	3	4	617	2
3	4	574	3	3	4	618	1
3	4	575	1	3	4	619	1
3	4	576	2	3	4	620	3
3	4	577	3	3	4	621	3
3	4	578	1	3	4	622	2
3	4	579	2	3	4	623	1
3	4	580	2	3	4	624	3
3	4	581	3	3	4	625	2
3	4	582	2	3	4	626	3
3	4	583	3	3	4	627	1
3	4	584	1	3	4	628	2
3	4	585	2	3	4	629	3
3	4	586	1	3	4	630	1
3	4	587	1	3	4	631	3
3	4	588	2	3	4	632	2
3	4	589	2	3	4	633	1
3	4	590	3	3	4	634	3
3	4	591	3	3	4	635	2
3	4	592	3	3	4	636	1
3	4	593	2	3	4	637	2
3	4	594	2	3	4	638	2
3	4	595	1	3	4	639	1
3	4	596	1	3	4	640	2
3	4	597	2	3	4	641	3
3	4	598	1	3	4	642	3
3	4	599	3	3	4	643	1
3	4	600	3	3	4	644	1
3	4	601	2	3	4	645	3
3	4	602	3				

Number of cases read: 195 Number of cases listed: 195

	Frequency	Percent	Valid Percent	Cumulative Percent
Valid PM	65	33.3	33.3	33.3
SSG	65	33.3	33.3	66.7

SSG+PM	65	33.3	33.3	100.0
Total	195	100.0	100.0	

Randomization list for Kassab – Sudan

COUNTRY	SITE	PATIENT NUMBER	DRUG	COUNTRY	SITE	PATIENT NUMBER	DRUG
3	35	646	1	3	35	701	1
3	35	647	1	3	35	702	3
3	35	648	3	3	35	703	2
3	35	649	3	3	35	704	1
3	35	650	2	3	35	705	1
3	35	651	2	3	35	706	1
3	35	652	1	3	35	707	3
3	35	653	3	3	35	708	3
3	35	654	2	3	35	709	2
3	35	655	3	3	35	710	1
3	35	656	2	3	35	711	3
3	35	657	1	3	35	712	1
3	35	658	3	3	35	713	3
3	35	659	2	3	35	714	2
3	35	660	1	3	35	715	2
3	35	661	3	3	35	716	1
3	35	662	1	3	35	717	3
3	35	663	2	3	35	718	2
3	35	664	3	3	35	719	1
3	35	665	2	3	35	720	2
3	35	666	2	3	35	721	2
3	35	667	3	3	35	722	3
3	35	668	1	3	35	723	3
3	35	669	1	3	35	724	3
3	35	670	2	3	35	725	2
3	35	671	1	3	35	726	2
3	35	672	3	3	35	727	1
3	35	673	2	3	35	728	1
3	35	674	3	3	35	729	2
3	35	675	1	3	35	730	2
3	35	676	1	3	35	731	1
3	35	677	2	3	35	732	1
3	35	678	3	3	35	733	1
3	35	679	2	3	35	734	3
3	35	680	3	3	35	735	3
3	35	681	3	3	35	736	3
3	35	682	1	3	35	737	2
3	35	683	3	3	35	738	3
3	35	684	2	3	35	739	2

COUNTRY	SITE	PATIENT NUMBER	DRUG	COUNTRY	SITE	PATIENT NUMBER	DRUG
3	35	685	1	3	35	740	3
3	35	686	1	3	35	741	3
3	35	687	2	3	35	742	3
3	35	688	1	3	35	743	1
3	35	689	3	3	35	744	2
3	35	690	2	3	35	745	1
3	35	691	2	3	35	746	1
3	35	692	1	3	35	747	2
3	35	693	3	3	35	748	1
3	35	694	2	3	35	749	2
3	35	695	2	3	35	750	1
3	35	696	3	3	35	751	3
3	35	697	3	3	35	752	3
3	35	698	3	3	35	753	2
3	35	699	2	3	35	754	2
3	35	700	1	3	35	755	1
3	35	756	2	3	35	812	2
3	35	757	1	3	35	813	3
3	35	758	2	3	35	814	3
3	35	759	1	3	35	815	2
3	35	760	3	3	35	816	2
3	35	761	2	3	35	817	1
3	35	762	3	3	35	818	1
3	35	763	1	3	35	819	3
3	35	764	1	3	35	820	1
3	35	765	3	3	35	821	2
3	35	766	3	3	35	822	2
3	35	767	3	3	35	823	1
3	35	768	1	3	35	824	3
3	35	769	1	3	35	825	1
3	35	770	3	3	35	826	3
3	35	771	2	3	35	827	2
3	35	772	2	3	35	828	3
3	35	773	2	3	35	829	2
3	35	774	1	3	35	830	1
3	35	775	3	3	35	831	2
3	35	776	1	3	35	832	1
3	35	777	2	3	35	833	1
3	35	778	2	3	35	834	2
3	35	779	1	3	35	835	3
3	35	780	3	3	35	836	1

COUNTRY	SITE	PATIENT NUMBER	DRUG	COUNTRY	SITE	PATIENT NUMBER	DRUG
3	35	781	1	3	35	837	2
3	35	782	3	3	35	838	3
3	35	783	3	3	35	839	2
3	35	784	1	3	35	840	3
3	35	785	1	3	35	841	1
3	35	786	3	3	35	842	2
3	35	787	1	3	35	843	3
3	35	788	2	3	35	844	3
3	35	789	3	3	35	845	2
3	35	790	2	3	35	846	2
3	35	791	1	3	35	847	1
3	35	792	2	3	35	848	3
3	35	793	2	3	35	849	2
3	35	794	2	3	35	850	1
3	35	795	3	3	35	851	1
3	35	796	1	3	35	852	2
3	35	797	1	3	35	853	1
3	35	798	2	3	35	854	3
3	35	799	3	3	35	855	3
3	35	800	3	3	35	856	3
3	35	801	3	3	35	857	3
3	35	802	1	3	35	858	2
3	35	803	2	3	35	859	2
3	35	804	2	3	35	860	2
3	35	805	2	3	35	861	1
3	35	806	1	3	35	862	1
3	35	807	2	3	35	863	1
3	35	808	1	3	35	864	3
3	35	809	3	3	35	865	1
3	35	810	3	3	35	866	2
3	35	811	3	3	35	867	3
3	35	868	1	3	35	892	2
3	35	869	2	3	35	893	3
3	35	870	3	3	35	894	1
3	35	871	2	3	35	895	3
3	35	872	2	3	35	896	1
3	35	873	1	3	35	897	1
3	35	874	3	3	35	898	2
3	35	875	1	3	35	899	3
3	35	876	1	3	35	900	1
3	35	877	2	3	35	901	2

COUNTRY	SITE	PATIENT NUMBER	DRUG	COUNTRY	SITE	PATIENT NUMBER	DRUG
3	35	878	3	3	35	902	2
3	35	879	1	3	35	903	3
3	35	880	3	3	35	904	1
3	35	881	2	3	35	905	3
3	35	882	1	3	35	906	1
3	35	883	3	3	35	907	3
3	35	884	2	3	35	908	3
3	35	885	3	3	35	909	2
3	35	886	3	3	35	910	2
3	35	887	3	3	35	911	1
3	35	888	2	3	35	912	1
3	35	889	1	3	35	913	2
3	35	890	2	3	35	914	3
3	35	891	2	3	35	915	1

Number of cases read: 270 Number of cases listed: 270

		Frequency	Percent	Valid Percent	Cumulative Percent
Valid	1 PM	90	33.3	33.3	33.3
	2 SSG	90	33.3	33.3	66.7
	3 SSG+PM	90	33.3	33.3	100.0
	Total	270	100.0	100.0	

16.2.7 Audit certificates

Appendix 12: Clinical Audit

The following GCP audit were conducted on the Trial Master Files held at KEMRI

1. Date of audit 27th-29th March 2006

The following GCP audit site audits were conducted

2. KEMRI, Kenya	25th November – 1st December 2006
3. Kassab, Sudan	5th -10th March 2007
4. Arba Minch Ethiopia	8th-12th December 2006
5. Gondar, Ethiopia	7th – 9th March 2007

Appendix 13: Laboratory Audit

The following Clinical audit focussing on Laboratory were conducted

6. KEMRI, Kenya	22nd April 2007
7. Kassab, Sudan	24th – 25th April 2007
8. Arba Minch Ethiopia	13th – 14th May 2007
9. Gondar, Ethiopia	9th – 11th May 2007

Appendix 14: Parasitology Quality Control

LEAP 0104A – Parasitology Quality Control conducted be Tony Moody

Slide Review - UM EL KHER Q1 2005

Slide preparation
The slides were un-mounted but well labelled with a diamond marker.
There was some confusion on the labelling as the DAT number was used on the slide and both the DAT and Study number was present in the box chart. The statistician required the study number but this was easily corrected. The boxes were 100 spaces and the slides were easily identified by their position.
The majority of the slides were well prepared with the aspirate adequately distributed and having good cellularity. In most cases the staining with Giemsa stain was excellent and the parasites clearly seen at 1000x.
A few slides did not stain well or were poor aspirates and these are indicated in the report. Some dates were obscured

Um El Kher

LN DIAGNOSTIC

No. of slides examined:	90
No. found positive:	85
No. found negative:	5 (5.5%)

FIRST FOLLOW UP

No. found positive:	6
No. found negative:	9
No. found unreadable:	1 (no cells)

TOC AFTER AMBISONE TREATMENT

No of slides examined:	65
No found positive:	15
No found negative:	49
No. unsuitable for examination:	1

TOC AFTER INITIAL TREATMENT

No of slides examined:	92
No. found positive:	14
No found negative:	78
No. unsuitable for examination:	0

STUDY FOLLOW UP NEG

No. of slides examined:	62
No. found positive:	2
No. found negative:	60 (1 peripheral blood)

Slide number	Patient id	Date	Result	Comment
93	3447G	19/1/05	Neg	
52	4452	6/6/2005	Neg	
87	4452	15/2/05	Neg	
9	4452	19/1/05	Neg	
86	4452	24/1/05	Neg	
95	12564	15/11/04	Neg	
88	17974	5/2/2005	Neg	
94	19256		Neg	
89	20490	5/2/2005	Neg	
38	24777	3/6/2005	Neg	
39	24777	15/12/04	3+	
39	24977	10/1/2005	1+	
8	26876	11/12/2004	1+	
10	26876	9/2/2005	Neg	
8	26876	19/11/04	4+	
9	26876	19/2/05	Neg	
3	26912	9/12/2004	Neg	
3	26912	2/1/2005	3+	
4	26912	7/1/2005	Neg	
62	26912	12/1/2005	1+	
61	26912	17/1/05	Neg	
3	26912	17/11/04	4+	
1	26932	4/12/2004	Neg	
36	26932	3/6/2005	Neg	
1	26932	16/11/04	2+	

Slide number	Patient id	Date	Result	Comment
2	26937	6/12/2004	Neg	Also 15/12/04 Neg
2	26937	6/1/2005	Neg	
2	26937	16/11/04	2+	
4	26964	8/12/2004	Neg	
54	26964	7/6/2005	Neg	
4	26964	18/11/04	4+	
5	26968	10/12/2004	Neg	
6	26968	3/1/2005	Neg	
5	26968	18/11/04	3+	
5	26968	24/12/04	Neg	
6	26969	18/11/04	3+	
6	26969	19/12/04	Neg	
7	26969	20/2/05	1+	
2	26969	25/1/05	3+	
8	26969	25/2/05	Neg	
7	26976	6/12/2004	1+	
7	26976	18/11/04	3+	
9	27013	7/12/2004	Neg	
37	27013	3/6/2005	Neg	
4	27013	7/1/2005	Neg	
9	27013	20/11/04	3+	
11	27059	24/11/04	3+	
11	27059	26/12/04	Neg	
11	27081	17/1/05	Neg	
12	27081	17/1/05	Neg	
10	27081	22/11/04	2+	
19	27081	31/5/05	Neg	
10	27087	15/12/04	Neg	
57	27132	7/6/2005	Neg	
12	27132	25/11/04	4+	
12	27132	25/12/04	Neg	
13	27143	4/12/2004	Neg	
1	27143	16/6/05	1+	
13	27143	27/11/04	1+	
30	27159	2/6/2005	Neg	
14	27159	26/12/04	Neg	
14	27159	27/11/04	2+	
31	27194	3/6/2005	Neg	
15	27194	27/11/04	1+	
15	27194	29/12/04	Neg	
24	27203	1/6/2005	Neg	
14	27203	16/1/05	Neg	
16	27203	17/12/04	Neg	
16	27203	27/11/04	1+	
14	27220	5/2/2005	Neg	
15	27229	1/3/2005	Neg	
16	27229	6/3/2005	NegVPS	
17	27229	20/12/04	Neg	
17	27229	28/11/04	2+	
39	27236	4/6/2005	Neg	
18	27236	28/11/04	1+	
20	27238	9/12/2004	Neg	
9	27238	10/6/2005	Neg	
19	27238	28/11/04	2+	

Slide number	Patient id	Date	Result	Comment
55	27239	7/6/2005	Neg	
18	27239	16/12/04	Neg	
20	27239	29/11/04	3+	
17	27241	3/1/2005	Neg	
21	27241	29/11/04	2+	
22	27269	1/12/2004	2+	
22	27269	3/1/2005	Neg	
11	27269	9/6/2005	Neg	
23	27273	1/12/2004	1+	
19	27273	15/1/05	Neg	
20	27273	23/1/05	Neg	
23	27273	24/12/05	Neg	
26	27301	4/12/2004	5+	
23	27301	3/2/2005	Neg	
5	27301	9/1/2005	3+	
26	27301	26/12/04	4+	
24	27301		Neg	
24	27302	3/12/2004	3+	
25	27302	1/6/2005	Neg	
24	27302	21/12/04	Neg	
25	27330	4/12/2004	6+	
60	27330	8/6/2005	Neg	
21	27330	19/1/05	1+	
25	27330	20/12/04	5+	
22	27330	23/2/05	unsuitable	v.poor slide
67	27330	29/1/05	3+	
21	27341	20/12/04	Neg	
27	27360	6/12/2004	1+	
27	27360	5/1/2005	Neg	
28	27361	6/12/2004	4+	
40	27361	5/6/2005	Neg	
49	27361	7/1/2005	Neg	
28	27361	24/12/04	Neg	
29	27393	8/12/2004	1+	
23	27393	1/6/2005	Neg	
29	27393	7/1/2005	Neg	
32	27405	9/12/2004	2+	
41	27405	5/6/2005	Neg	
32	27405	29/12/05	Neg	
30	27415	8/12/2004	2+	
30	27415	8/1/2005	Neg	
7	27415	13/6/05	Neg	
31	27429	9/12/2004	2+	
34	27429	3/6/2005	Neg	
31	27429	27/12/04	1+	
33	27430	9/12/2004	Neg	
12	27430	10/4/2005	Neg	
33	27430	29/12/04	Neg	
16	27430	31/5/06	Neg	
34	27454	10/12/2004	2+	
25	27454	2/1/2005	Neg	
47	27454	6/6/2005	Neg	
34	27454	17/1/05	Neg	
35	27458	10/12/2004	4+	

Slide number	Patient id	Date	Result	Comment
50	27458	6/6/2005	Neg	
35	27458	30/12/04	Neg	
36	27473	11/12/2004	1+	
58	27473	7/6/2005	Neg	
36	27473	30/12/04	Neg	
37	27482	12/12/2004	2+	
26	27482	1/6/2005	Neg	
37	27482	12/1/2005	Neg	
3	27498	7/2/2005	Neg	
53	27498	7/6/2005	Neg	
38	27498	13/1/05	Neg	
38	27498	13/12/04	5+	
66	27516	27/1/05	Neg	
40	27543	6/1/2005	Neg	
3	27543	15/6/05	Neg	
40	27543	16/12/04	4+	
11	27548	2/11/2004	Neg	
29	27548	2/6/2005	Neg	(peripheral blood)
42	27548	9/1/2005	Neg	
42	27548	16/12/04	1+	
28	27555	3/1/2005	Neg	
27	27555	5/2/2005	Neg	
41	27555	7/1/2005	Neg	
41	27555	16/12/04	3+	
44	27561	17/12/04	1+	
46	27564	6/6/2005	Neg	
15	27564	12/4/2005	Neg	
43	27564	17/12/04	5+	
1	27564	19/5/05	Neg	Peripheral blood
44	27567	3/1/2005	Neg	
32	27567	3/6/2005	Neg	
65	27584	5/1/2005	1+	
27	27586	2/6/2005	Neg	
45	27586	12/1/2005	Neg	
45	27586	17/12/04	1+	
28	27601	2/6/2005	Neg	
46	27601	18/1/05	Neg	
46	27602	18/12/04	2+	
29	27612	9/3/2005	Neg	
10	27612	11/7/2005	Neg	
30	27612	13/3/05	Neg	
47	27612	20/12/04	Neg	
59	27645	7/6/2005	Neg	
50	27645	12/1/2005	Neg	
50	27645	20/12/04	1+	
49	27654	6/6/2005	Neg	
48	27654	20/12/04	2+	
48	27654	21/1/05	Neg	
51	27655	6/6/2005	Neg	
49	27655	20/12/04	2+	
51	27662	8/1/2005	Neg	
61	27662	8/6/2005	Neg	
51	27662	21/12/04	5+	
62	27687	8/6/2005	Neg	

Slide number	Patient id	Date	Result	Comment
52	27687	9/1/2005	Neg	
52	27687	22/12/04	Neg	
31	27716	9/2/2005	Neg	
32	27716	14/2/05	Neg	
53	27716	15/1/05	Neg	
53	27716	24/12/04	Neg	
33	27756	6/2/2005	2+	
68	27756	11/2/2005	Neg	
54	27756	13/1/05	2+	
34	27756	16/2/05	Neg	
54	27756	26/12/04	3+	
22	27768	1/6/2005	Neg	
55	27768	27/1/05	Neg	
55	27768	27/12/04	4+	
57	27770	29/12/04	5+	
57	27770	31/1/05	1+	
14	27770	31/5/05	Neg	
56	27786	30/1/05	Neg	
56	27786	30/12/04	1+	
36	27790	8/2/2005	Neg	
35	27790	14/2/05	Neg	
58	27790	21/1/05	Neg	
58	27790	31/12/04	2+	
69	27795	16/2/05	Neg	
37	27795	21/2/05	Neg	
59	27795	23/1/05	2+	
38	27795	26/2/05	Neg	
59	27795	31/12/04	4+	
6	27797	13/6/05	Neg	
60	27797	18/1/05	Neg	
60	27797	31/12/04	2+	
61	27808	1/1/2005	2+	
10	27808	10/6/2005	Neg	
61	27808	18/1/05	Neg	
62	27817	1/1/2005	4+	
39	27817	17/2/05	Neg	
40	27817	22/1/05	1+	
62	27817	23/1/05	4+	
63	27828	2/1/2005	6+	
63	27828	20/1/05	Neg	
16	27828	28/2/05	1+	
17	27828	31/5/06	Neg	
64	27835	3/1/2005	1+	
56	27835	7/6/2005	Neg	
91	27835	11/1/2005	Neg	
64	27835	3/2/05 ?	Neg	
66	27842	5/1/2005	Neg	
48	27842	6/6/2005	Neg	
96	27849	5/2/2005	Neg	
65	27854	4/2/2005	Neg	
6	27854	13/5/05	Neg	
67	27871	6/1/2005	4+	
42	27877	1/3/2005	1+	
41	27877	21/2/05	1+	

Slide number	Patient id	Date	Result	Comment
92	27877	26/12/04	Neg	
97	27878	27/1/05	3+	
35	27881	3/6/2005	Neg	
68	27881	6/1/2005	4+	
68	27881	8/2/2005	Neg	
7	27884	2/4/2005	1+	Degenerate parasites
43	27884	2/5/2005	Neg	
69	27884	6/1/2005	1+	
71	27884	8/5/2005	Neg	
8	27884	22/3 /05	1+	Degenerate parasites
44	27884	25/3/05	1+	
70	27884	27/4/05	Neg	
69	27884	28/1/05	Neg	
67	27887	27/1/05	4+	
42	27935	5/6/2005	Neg	
70	27935	10/1/2005	1+	
70	27935	10/2/2005	Neg	
71	27962	1/2/2005	Neg	
46	27962	3/3/2005	Neg	
71	27962	10/1/2005	1+	
45	27962	26/2/05	Neg	
72	27969	11/1/2005	1+	
98	27969	11/1/2005	2+	
72	27969	28/1/05	Neg	
44	27973	5/6/2005	Neg	
74	27973	12/1/2005	2+	
74	27973	30/1/05	Neg	
21	27983	1/6/2005	Neg	
73	27983	12/1/2005	2+	
73	27983	13/2/05	Neg	
13	27994	9/6/2005	Neg	
75	27994	12/1/2005	1+	
75	27994	30/1/05	Neg	
90	27997	5/2/2005	Neg	
2	28007	4/6/2005	1+	
76	28007	13/1/05	2+	
48	28007	13/7/05	Neg	
77	28013	1/2/2005	Neg	
77	28013	14/1/05	1+	
20	28013	31/5/05	Neg	
49	28041	4/4/2005	Neg	
79	28041	7/2/2005	3+	
50	28041	9/4/2005	Neg	
78	28041	14/1/05	3+	
80	28049	4/2/2005	Neg	
5	28049	14/6/05	Neg	
79	28049	15/1/05	3+	
52	28059	6/3/2005	Neg	
81	28059	20/1/05	2+	
82	28059	20/2/05	1+	
51	28059	21/3/05	Neg	
33	28061	3/6/2005	Neg	
82	28061	20/1/05	4+	
81	28062	6/2/2005	2+	

Slide number	Patient id	Date	Result	Comment
4	28062	14/6/05	Neg	
80	28062	19/1/05	4+	
53	28074	11/3/2005	Neg	
54	28074	3-Jun	Neg	
85	28074	15/2/05	2+	28074 14/2/05 3+
84	28074	23/1/05	2+	
12	28080	9/6/2005	Neg	
83	28080	22/1/05	1+	
84	28080	22/2/05	Neg	
86	28087	14/2/05	Neg	
56	28087	18/4/05	Neg	
85	28087	23/1/05	2+	
72	28087	23/4/05	Neg	
13	28087	24/3/05	Unsuitable	No cells
55	28087	Missing		
43	28090	5/6/2005	Neg	
90	28090	12/2/2005	Neg	
89	28090	25/1/05	1+	
87	28100	24/1/05	1+	
88	28100	24/2/05	? 1+	
15	28100	31/5/05	Neg	
59	28105	12/3/2005	1+	
89	28105	16/2/05	3+	
58	28105	17/3/05	2+	
57	28105	22/3/05	2+	
88	28105	24/1/05	3+	
90	28119	26/1/05	2+	
91	28119	26/2/05	Neg	
18	28119	31/5/05	Neg	
66	26876 (1)	5/1/2005	2+	
65	26876 (2)	10/2/05	Neg	
64	26876 (3)	2/2/2005	Neg	
45	27360/1	5/6/2005	Neg	(slide labeled 27360)
8	27786 (27772)	10/6/2005	Neg	

KASSAB-SUDAN – Q1 2005

**INITIAL DIAGNOSTIC SLIDES**		**TOC SLIDES**	
No. of initial slides:	56	No. of TOC slides:	51
No. found positive:	54	No. found positive:	8
No. found negative:	2	No. found negative:	42
		No. found unreadable:	1

Slide number	Screening Number	Date	Result	Comment
1	1001 ln	26/1/05	1+	Parasites lysed
2	1001 ln toc	17/2/05	Neg	
3	1001 ln fu	6/7/2005	Neg	?lysed cytoplasm
4	1001 bm fu	6/7/2005	3+	?lysed cytoplasm
5	1003	27/1/05	2+	
6	1003 toc	19/2/05	Neg	
7	1004	27/1/05	4+	
8	1004 toc	16/2/05	Neg	
9	1005	29/1/05	4+	
10	1005 toc	No date	2+	
11	1006	28/1/05	1+	
12	1006 toc	1/3/2005	Neg	
13	1007	28/1/05	1+	
14	1007 toc	1/3/2005	Neg	
15	1008	30/1/05	3+	
16	1008 toc	22/2/05	neg	Poor slide
17	1009	1/2/2005	5+	
18	1009 toc	19/2/05	Neg	
19	1010			missing
20	1011 bm	1/2/2005	2+	
21	1011 bm toc	5/3/2005	Neg	
22	1012			missing
23	1013			missing
24	1014	1/2/2005	1+	
25	1014 toc	18/2/05	Neg	
26	1015	5/2/2005	1+	
27	1015 toc	3/3/2005	Neg	
28	1016			missing
29	1017	1/2/2005	1+	
30	1017 toc	5/3/2005	Neg	
31	1018			missing
32	1019			missing
33	1020			missing
34	1021			missing
35	1022			missing
36	1023			missing
37	1024			missing
38	1025 bm	7/2/2005	Neg	
39	1025 bm toc	1/3/2005	Neg	
40	1026			missing
41	1027	6/2/2005	1+	
42	1027 toc	25/2/05	Neg	
43	1028			missing
44	1029	7/2/2005	3+	
45	1029 toc	10/3/2005		Unreadable
46	1030			missing
47	1031			missing
48	1032	8/2/2005	4+	

Slide number	Screening Number	Date	Result	Comment
49	1032 toc	30/3/05	2+	Degenerated
50	1033			missing
51	1034			missing
52	1035			missing
53	1036			missing
54	1037			missing
55	1038	14/2/05	3+	
56	1038 toc	15/3/05	Neg	
57	1039			missing
58	1040			missing
59	1041 ln	19/2/05	5+	
60	1041 ln toc	12/3/2005	4+	
61	1041 bm	No date	4+	
62	1041 bm toc	14/3/05	2+	
63	1042	No date	4+	
64	1042 toc	No date	Neg	
65	1042 bm	No date	3+	
66	1043 bm	No date	2+	
67	1043 bm toc	No date	Neg	
68	1044	21/2/05	1+	
69	1044 toc	26/3/05	Neg	
70	1045			missing
71	1046	24/2/05	3+	
72	1046 toc	28/3/05	Neg	
73	1047			missing
74	1048			missing
75	1049			missing
76	1050			missing
77	1051	No date	3+	
78	1051	No date	1+	Poor slide
79	1051 toc	17/3/05	Neg	
80	1052	No date	1+	
81	1052	No date	Neg	
82	1052 toc	No date	Neg	
83	1053	No date	4+	
84	1053	No date	5+	
85	1053 bm toc	No date	Neg	
86	1053 ln toc	No date	4+	
87	1054	No date		missing
88	1055	No date	3+	
89	1055 toc	No date	Neg	
90	1056	No date		missing
91	1057	No date		missing
92	1058	No date	4+	
93	1058 ln toc	No date	5+	Some degenerate
94	1058 bm toc	No date	3+	
95	1059	No date		missing
96	1060	9/3/2005	1+	
97	1060 toc	28/3/05	Neg	
98	1061	No date		missing
99	1062	No date		missing
100	1063	No date	4+	
101	1063 toc	No date	Neg	
102	1064	No date	3+	

Slide number	Screening Number	Date	Result	Comment
103	1064 toc	No date	Neg	
104	1065	No date		missing
105	1066	No date		missing
106	1067	No date		missing
107	1068	No date	4+	
108	1068 toc	No date	? 1+	
109	1069	No date		missing
110	1070	No date	1+	
111	1070 toc	No date	Neg	
112	1071m	No date		missing
113	1072	No date	1+	
114	1072 toc	No date	Neg	
115	1073m	No date		missing
116	1074	No date	2+	
117	1074 toc	No date	Neg	
118	1075	No date	2+	
119	1075 toc	No date	Neg	
120	1076	No date	2+	
121	1076 toc	No date	Neg	
123	1077	No date	2+	
124	1077 toc	No date	Neg	
125	1078	No date	1+	Very poor slide
126	1078 toc	No date	Neg	
127	1079			missing
128	1079			missing
129	1080	No date	1+	
130	1080 toc	No date	Neg	
131	1081	No date		missing
132	1082	No date		missing
133	1083	No date		missing
134	1084	No date		missing
135	1085	No date		missing
136	1086	No date		missing
137	1087	No date		missing
138	1088 bm	No date	Neg	
139	1088 bm	No date	Neg	
140	1089	No date		missing
141	1090	No date		missing
142	1091	No date		missing
143	1092	6/4/2005	4+	
144	1092 toc	No date	Neg	
145	1093	No date		missing
146	1094	No date	1+	
147	1094 toc	No date	Neg	
148	1095	No date		missing
149	1096 bm	No date	3+	
150	1096 bm	No date	Neg	
151	1097	No date	1+	
152	1097	No date	Neg	
153	1098	No date		missing
154	1099	No date		missing
155	1100 bm	No date	3+	
156	1100 bm toc	No date	Neg	
157	1100 ln	No date	1+	Very scanty

Slide number	Screening Number	Date	Result	Comment
158	1100 ln toc	No date	Neg	
159	1101	No date		missing
160	1102 ln	No date	3+	
161	1102 bm	14/4/05	2+	
162	1102 bm toc	No date	Neg	
163	1102 ln toc	No date	Neg	
164	11073 bm	No date	Neg	
165	11073 ln	No date	Neg	

Study Slides for Leishmania from KEMRI
Examined by A Moody October 2005

General comment: many slides had very poor staining with little red stain or differentiation, water bubbles in the mounting media was considerable and obscured the field. 13 Diagnostic slides and 20 TOC slides were poorly or very poorly stained or presented

**Initial diagnostic slides**		**TOC slides**	
No. of initial slides	45	No. of TOC slides	45
No. found positive	43	No. found positive	1
No. found negative	1	No. found negative	39
No. found unreadable	1	No. found unreadable	5

Slide Box number	CCR NO.	Study No.	Date of smear	Day	Microscopic	Comment
1	2408	1	17.01.05	D	4+	Very poor stain
2	2408	1	06.02.05	TOC	Negative	Very poor stain
3	2410	2	17.01.05	D	4+	
4	2410	2	10.02.05	TOC	Negative	Very poor stain
5	2411	3	17.01.05	D	4+	
6	2411	3	10.02.05	TOC	Unreadable	Very poor stain
7	2412	4	17.01.05	D	5+	
8	2412	4	06.02.05	TOC	Negative	
9	2413	5	17.01.05	D	4+	
10	2413	5	19.02.05	TOC	Negative	
11	2414	6	27.01.05	D	6+	
12	2414	6	21.02.05	TOC	Negative	Very poor stain
13	2415	7	27.01.05	D	3+	
14	2415	7	18.02.05	TOC	Negative	
15	2416	8	27.01.05	D	4+	
16	2416	8	03.03.05	TOC	Negative	Very poor stain
17	2417	9	27.01.05	D	4+	
18	2417	9	22.02.05	TOC	Negative	
19	2418	10	27.01.05	D	4+	
20	2418	10	06.03.05	TOC	Negative	
21	2419	11	03.02.05	D	1+	Very poor stain
22	2419	11	25.02.05	TOC	Negative	
23	2422	12	10.02.05	D	3+	poor stain
24	2422	12	04.03.05	TOC	Negative	
25	2423	13	16.02.05	D	3+	poor stain
26	2423	13	20.03.05	TOC	Negative	
27	2424	14	17.02.05	D	4+	
28	2424	14	26.03.05	TOC	Negative	
29	2449	28	14.04.05	D	Negative	
30	2449	28	10.05.05	TOC	Negative	
31	2428	15	23.02.05	D	?2+	Very poor stain
32	2428	15	14.03.05	TOC	Negative	
33	2429	19	23.02.05	D	5+	Very poor stain
34	2429	19	31.03.05	TOC	Negative	poor stain
35	2430	16	25.02.05	D	4+	
36	2430	16	31.03.05	TOC	Negative	
37	2433	20	08.03.05	D	4+	Very poor stain
38	2433	20	14.04.05	TOC	Negative	Very poor stain
39	2434	17	08.03.05	D	3+	
40	2434	17	23.03.05	TOC	Negative	
41	2435	21	08.03.05	D	1+	Very poor stain
42	2435	21	05.04.05	TOC	Negative	Very poor stain
43	2436	18	08.03.05	D	5+	
44	2436	18	26.03.06	TOC	Negative	Very poor stain
45	2438	22	17.03.05	D	4+	Very poor stain
46	2438	22	04.04.05	TOC	Negative	Very poor stain

Slide Box number	CCR NO.	Study No.	Date of smear	Day	Microscopic	Comment
47	2440	24	24.03.05	D	3+	poor stain
48	2440	24	05.05.05	TOC	Negative	
49	2441	23	24.03.05	D	4+	Very poor stain
50	2441	23	26.04.05	TOC	Negative	Very poor stain
51	2442	25	31.03.05	D	3+	
52	2442	25	17.05.05	TOC	Unreadable	
53	2446	26	14.04.05	D	4+	
54	2446	26	02.05.05	TOC	Negative	
55	2447	27	14.04.05	D	4+	
56	2447	27	06.05.05	TOC	Negative	
57	2448	34	14.04.05	D	2+	
58	2448	34	17.05.05	TOC	Negative	Very poor stain
59	2450	29	14.04.05	D	4+	
60	2450	29	20.05.05	TOC	Negative	Lymphocytes ++
61	2451	30	14.04.05	D	2+	
62	2451	30	11.05.05	TOC	Negative	Plasma cells +, lymphocytes ++
63	2453	31	20.04.05	D	1+	Plasma cells +, lymphocytes ++
64	2453	31	09.05.05	TOC	Negative	
65	2454	32	20.04.05	D	4+	
66	2454	32	05.05.05	TOC	Negative	Very poor stain
67	2455	33	20.04.05	D	3+	
68	2455	33	23.05.05	TOC	Negative	
69	2456	35	11.05.05	D	4+	
70	2456	35	12.06.05	TOC	1+	
71	2457	36	11.05.05	D	5+	
72	2457	36	12.06.05	TOC	Negative	Very poor stain
73	2458	37	13.05.05	D	3+	
74	2458	37	24.06.05	TOC	Negative	Very poor stain
75	2461	40	13.05.05	D	3+	Very poor stain
76	2461	40	24.06.05	TOC	Negative	poor stain
77	2462	39	13.05.05	D	Unreadable	
78	2462	39	08.06.05	TOC	Unreadable	
79	2465	45	01.06,05	D	3+	
80	2465	45	01.07.05	TOC	Negative	Very poor stain
81	2466	41	01.06.05	D	4+	
82	2466	41	20.06.05	TOC	Unreadable	Very poor stain
83	2467	42	01.06.05	D	6+	
84	2467	42	20.06.05	TOC	Unreadable	Very poor stain
85	2468	43	01.06.05	D	5+	
86	2468	43	04.07.05	TOC	Negative	
87	2469	44	01.06.05	D	3+	
88	2469	44	04.07.05	TOC	Negative	
89	2459	38	13.05.05	D	4+	Very poor stain
90	2459	38	04.06.05	TOC	Negative	Very poor stain

Key: D = Diagnostic screening TOC = Test of cure

Review of slides from Gondar site 13th- 17th Feb 2006

**Initial diagnostic slides**		**TOC slides**	
No. of initial slides:	45	No. of TOC slides:	45
No. found positive:	9	No. found positive:	0
No. found negative:	25 (many inadequate)	No. found negative:	29 (many inadequate)
No. found unreadable:	11	No. found unreadable:	16

Problems associated with set of slides from Gondar:

- Cover slips applied prior to staining leaving tiny viewable area
- Poor staining or over differentiation of stain, inadequate material on some
- ? Wrights' stain used instead of Giemsa
- Most slides that were readable had adequate suitable material
- All Day 0 slides that were negative were reviewed twice
- Frosted slides obscured diamond labelling, lab number on slides were visible but no date or indication of day taken was available

General comment

This centred mostly on ability to provide adequate information to the statisticians to collate the given information from the slide reports with the patient Data forms.
Suggested conformity of slide labelling was the most useful approach.
Training of laboratory staff is also an important future consideration and will be a necessary part of the future programme.

Follow up visit to Gondar hospital 13-17th February 2006

1. During the visit I initially met and toured the laboratories and familiarised myself with the capabilities and conditions of the hospital laboratories and technicians. The staff are very friendly and Yegnesew has been able to establish his section in an area of the blood bank. This has sufficient space and light to make an adequate laboratory facility for the project.
2. I was introduced to Dr Zewdu and to Tegist the study nurse, they were very welcoming and showed me the ward facility.
3. Yegnesew and I then worked together to re-plan the laboratory space to accommodate a staining bench which was accomplished. Obtaining Giemsa stain, calibrating water pH and stain dilutions and organising stain troughs now means that patient slides can now be prepared, stained and examined in one area.
4. Investigation into possible problems with mounting media for the slides was conducted. Polystyrene mounting media prepared and Canada Balsam obtained from the Histopathology laboratory.
   Neither had a deleterious effect on the stained slides up to the time I left and so the possible mounting media effect on the preparation of the earlier set of slides can be discounted. The problem still remains a mystery.
5. Three 6 month follow up patients attended during my visit and were a test for the new set up which worked well. Both Yegnesew and Dr Zewdu were able to stain and spend time searching spleen and bone marrow aspirate slides.
6. The Biochemical tests that were being performed were well done and I was particularly pleased to hear about a quality control sample obtained from Addis had been used for calibration/QC and was well within the expected limits for the tests performed. I recommended that a QC sample with results outside the normal range be obtained next to further stretch the QC system.
7. I attended a meeting with the Hospital Director, senior doctors and the project manager to meet with and discuss how we could help the Pathologist but unfortunately he did not attend the meeting. Subsequent decisions on this matter can be seen in the Project managers report.

I left Gondar with high hopes that there is now an efficient team in place who will be able to produce good slide data and who are capable of knowing when to contact Asrat for help if needed.

ID	Day	Microscopy	comment	ID	Day	Microscopy	comment
001	Day 0	Negative	Main body unstained, tiny edge only viewable	024	19/8/05	Unsuitable for searching	No stain differentiation
001	Day 22	Unsuitable for searching	Not stained	024	10/9/05	Negative	
002	Day 0	Unsuitable for searching	Not stained	025	18/8/05	3+	Tiny edge area only, main slide unstained
002	Day 28	Unsuitable for	Not stained	025	19/9/05	Negative	

ID	Day	Microscopy	comment	ID	Day	Microscopy	comment
		searching					
003	No day	3+	Tiny end only<br>P malariae present	026	3/8/05	Unsuitable for searching	unstained
003	No day	Negative	Tiny end only<br>P malariae present	026	7/9/05	Unsuitable for searching	unstained
004	No day	Negative	Very poor slide	027	16/9/05	5+	Very small area on edge, rest unstained cover slipped
004	No day	Negative		027	No date	Unsuitable for searching	unstained
005	No day	Negative	Very poor slide<br>P falciparum	028	Day 0	Unsuitable for searching	VPS
005	No day	Negative	Very poor slide<br>P falciparum	028	Day 18	Negative	
006	No day	Negative		029	Day 0	1+	
006	No day	Negative		029	Day 22	Negative	
007	No day	Negative	Tiny edge only viewable<br>P falciparum	030	20/9/05	Unsuitable for searching	
007	2/7/05	Negative		030	13/10/05	Negative	
008	No day	Negative		031	Day 0	Negative	
008	1/7/05	Negative		031	Day 31	Negative	P falciparum
009	1/7/05	Negative	Very poor slide<br>P malariae	032	Day 0	Unsuitable for searching	Very poor stain
009	No date	Negative		032	Day18	Negative	
010	1/9/05	Negative	Very poor slide<br>P malariae	033	No date	VPS Unsuitable for searching	
010	No date	Unsuitable for searching		033	No slide		
011	6/7/05	Negative	Very poor slide<br>Main body unstained, tiny edge only viewable<br>P malariae, Pigment ++	034	Day 0	4+	
011	Day 18	Negative		034	Day 22	Negative	P falciparum
012	1/7/05	Negative	Malaria+	035	Day 0	3+	
012	25/7/05	Negative	Malaria Pf	035	Day 22	Negative	
013	Day 0	Negative		036	Day 0	1+	
013	10/8/05	Unsuitable for searching	Small amount material, very poor stain	036	Day 21	Negative	
014	Day 0	Negative	Small edge only viewable	037	No date	Peripheral blood Negative	P falciparum
014	No date	Unsuitable for searching	Pink stain??	037	No slide		
015	11/7/05	Negative	Unstained<br>Very small area viewable	038	No date	Peripheral blood Negative	
015	No date	Unsuitable for searching	Unstained	038	No slide		
016	8/7/05	Negative	Unstained<br>Very small area viewable	039	Day 0	Negative	
016	1/8/05	Negative		039	Day 22	Negative	
017	Day 0	Negative	Edge only	040	Day 0	2+	
017	Day 18	Unsuitable for searching	Unstained	040	Day 22	Negative	

Gondar DNDi site audited slides (A Moody external slide reviewer) - April 2007

Pt Initials	S.D No	Day 0	End of Rx	3 month FU	6 month FU	Reviewer Result Day 0	Reviewer Result End of Rx	Reviewer Result 3 month FU	Reviewer Result 6 month FU
AT	46	3+	0	ND	0	3+	0	ND	0
TA	47	4+	0	ND		4+	0	ND	
TG	48	4+	0	ND	0	4+	0	ND	0
WM	49	1+	0	ND	0	1+	0	ND	0
DG	50	3+	2+	0	0	3+	2+	0	0
OY	51	3+	0	ND	0	3+	0	ND	0
WA	52	3+	0	0		3+	0	0	
AW	53	5+	0	ND	0	5+	0	ND	0
DM	54	6+	5+	5+	5+	6+	5+	5+	5+
GS	55	2+	1+	ND	0	2+	1+	ND	0
MG	56	6+	0	0	0	6+	0	0	0
KW	57	4+	0	0	0	4+	0	0	0
AA	58	4+	0	ND	0	4+	0	ND	0
SM	59	4+	0	ND	0	4+	0	ND	0
DS	60	3+	0	0	0	3+	0	0	0
ET	61	2+	0	ND	0	2+	0	ND	0
AM	62	2+	0	ND	0	2+	0	ND	0
GG	63	6+	2+	0	0	6+	2+	0	0
GA	64	2+	0	ND	0	2+	0	ND	0
FM	65	2+	0	ND		2+	0	ND	
MB	66	3+	0	ND	0	3+	0	ND	0<br>NB. Eos ++
ME	67	4+	0	ND	0	4+	0	ND	0
EB	68	4+	0	0	0	4+	0	0	0
TF	69	1+	0	0	0	1+	0	0	0
AG	70	4+	0	ND	0	4+	0	ND	0
GT	71	6+	5+	5+		6+	5+	5+	
AT	72	6+	ND	ND	0	6+	ND	ND	0
GT	73	1+	0	0		1+	0	0	
AY	74	2+	0	ND	0	2+	0	ND	0
AS	75	6+	3+	3+		6+	3+	3+	
YA	76	2+	0	ND	0	2+	0	ND	0
AS	77	3+	0	0	0	3+	0	0	0
AG	78	2+	0	0	0	2+	0	0	0
AT	79	4+	0	0		4+	0	0	
FA	80	4+	0	ND		4+	0	ND	
AM	81	6+	0	0	0	6+	0	0	0
AY	82	3+	0	ND	0	3+	0	ND	0
SA	83	5+	0	ND	0	5+	0	ND	0
GA	84	4+	0	ND	0	4+	0	ND	0
BT	85	5+	0	ND		5+	0	ND	
DY	86	3+	0	ND	0	3+	0	ND	0
AT	87	2+	0	ND	0	2+	0	ND	0
AG	88	1+	0	ND	0	1+	0	ND	0
SD	89	2+	0	0		2+	0	0	
HT	90	2+	0	ND		3+	0	ND	

Code: ND = Not done

Reviewers comment: Slides were prepared and stained to a high quality and the results were excellent

Report of visit by Tony Moody as external reviewer to Arba Minch Leishmania Treatment and Research Centre 8-13th April 2006

Purpose of visit:
This visit was made on behalf of DNDi to to review the laboratory performance of this centre and provide audit of patient slides currently available that had not previously been seen by the external reviewer.

ArbaMinch Leishmania treatment and Research Department is a recently opened facility dedicated to the treatment and care of patients from surrounding villages suffering with Visceral Leishmaniasis. I had the pleasure of spending two days at this centre and was warmly welcomed and offered full co-operation during my visit.

Dr Samson Tesfaye the responsible physician for this unit and Negussu Abebe the laboratory BioMedical Scientist provided all the facilities and information I needed and also organised the other laboratory staff and some of the Medical staff to attend some lectures I was able to give in addition.

The laboratory.
The laboratory is well designed for its purpose but as yet is still incomplete. There are no working sink drains which means the patient slides are still stained in the main hospital laboratory and all haematological and Biochemical tests are still performed in the hospital laboratory (see comments on hospital laboratory). The space and patient base does mean that both patient sample examination and potential research opportunities will eventually be performed in the unit.
There are other laboratory personnel involved in the project who provide help and cover for Negussu and the laboratory enthusiasm is excellent and should be encouraged with additional training opportunities.

Slide Examination
Together with Negussu I examined all 33 archived positive slides and 30% of the 52 negative slides. The slides were a mixture of splenic aspirates, bone marrow aspirates and one LN aspirate. Preparation and staining by Giemsa stain was excellent and apart from very few slight adjustments from 3+ to 4+ or 4+ to 3+ there was complete correlation and agreement on the readings (results attached). Negussu had a very efficient and satisfactory system of slide labelling and archiving and his record keeping was very satisfactory.
I have no adverse comments to make about the laboratory work and look forward to its further development as equipment becomes available.

Hospital main Laboratory
Dr Samson had arranged for me to visit the main hospital laboratory and I was shown round by the Senior BioMedical Scientist.
The laboratory is not large and does not have a medical consultant. There is a limited amount of automation for the haematology and Clinical Chemistry sections but no Microbiology beyond basic staining for TB and gram stain is available. A limited blood bank is present using tile grouping and xmatching. Parasitology is basic and limited but potential is enormous given the presence of endemic malaria and HIV.

The Biochemisry available to the Leishmania unit is adequate using an automated system with a good backup facility. Quality control is performed with preci-normal sera but a range of abnormal sera would enhance this. There did not seem to be QC available for any of the haematology parameters.
The laboratory is a busy department and the visit was interesting and again emphasis the need for additional training opportunities for staff.

Summary
The laboratory department of the Leishmaniasis unit is functioning efficiently and the standard of work is satisfactory. There is great potential for further interesting development within the laboratory once the deficient structural work is completed and the involvement of Negussu and his colleagues in this work will be a rewarding stimulus.
I thank Dr Samson for his hospitality and care which helped to make my visit successful.

Number	Slide No / Hospital card No	Procedure / Period	Collection Date	Result
8	4018	Day 0	22-10-05	4
24	4018	Day 18	06-03-98 E.C	NO LD SEEN
12	4018	3 month	3/3/2006	NO LD SEEN
3	4019	Day 0	25-10-05	4
22	4019	Day 31	16-03-98 E.C	NO LD SEEN
2	4362	Day 0	28-10-05	5
23	4362	Day 18	16-11-05	NO LD SEEN
29	4364	Day 31	29-111-05	NO LD SEEN
5	4364	Day 0	28-10-05	3
28	4365	Day 31	29-11-05	NO LD SEEN
6	4365	Day 0	28-10-05	2
21	4366	Day 0	28-10-05	NO LD SEEN
20	4367	Day 0	28-10-05	NO LD SEEN
7	4433	Day 0	28-10-05	3
26	4433	Day 18	16-11-05	NO LD SEEN
32	4615	Day 31	31-11-05	NO LD SEEN
10	4615	Day 0	31-10-05	3
19	4616	Day 0	31-10-05	NO LD SEEN
9	5752	Day 0	14-11-05	4
25	5752	Day 18	05-12-98 E.C	NO LD SEEN
41	5753	Day 31	18-12-05	NO LD SEEN
13	5753	Day 0	14-11-05	3
29	7282	Day 0	21-03-98	3
34	7282	Day 31	15-01-06	NO LD SEEN
31	7283	Day 0		NO LD SEEN
27	7284	Day 0		NO LD SEEN
30	7285	Day 0		NO LD SEEN
21	9265	Day 0	28 -12-05	4
36	9265	Day 18	20-01-06	NO LD SEEN
12	9266	Day 0	29-12-05	5
33	9267	Day 0	29-11-05	NO LD SEEN
11	9268	Day 0	29-12-05	5
37	9268	Day 22	22-01-06	NO LD SEEN
14	9270	Day 0	28-12-05	2
38	9270	Day 22	22-01-06	NO LD SEEN
16	9271	Day 0	28-12-05	2
40	9271	Day 22	22-01-06	NO LD SEEN
49	9272	Day 0		NO LD SEEN
35	9282	Day 0	28-12-05	NO LD SEEN
9	11065	Day 31	25-02-06	NO LD SEEN
22	11065	Day 0	23-01-06	3
39	11067	Day 0	23-01-06	NO LD SEEN
17	13340*	Day 0	11/2/2006	4
15	13141	Day 0	11/2/2006	4
3	13141	Day 18	4/3/2006	NO LD SEEN
5	13142	Day 0	11/2/2006	NO LD SEEN
6	13338	Day 0	11/2/2006	NO LD SEEN
7	13339	Day 0	11/2/2006	NO LD SEEN

Number	Slide No / Hospital card No	Procedure / Period	Collection Date	Result
19	13341	Day 0	9/2/2006	4
2	13341	Day 22	8/3/2006	NO LD SEEN
46	13342	Day 31	17-03-06	NO LD SEEN
18	13342	Day 0	11/2/2006	4
8	13343	Day 0	11/2/2006	NO LD SEEN
4<br>20	13378<br>15151	Day 0<br>Day 0	11/2/2006<br>8/3/2006	NO LD SEEN<br>3
48	15151	Day 22	3/4/2006	NO LD SEEN
1	15152	Day 0	8/3/2006	NO LD SEEN
44	15153	Day 0	8/3/2006	NO LD SEEN
18	15301	Day 0	31-10-05	NO LD SEEN
24	15793	Day 0	15-03-06	5
45	15793	Day 18	3/4/2006	NO LD SEEN
26	15794	Day 0	15-03-06	6
43	15794	Day 18	3/4/2006	NO LD SEEN
27	16355	Day 0	23-03-06	5
25	16681	Day 0	3/4/2006	4
23	16814	Day 0	3/4/2006	3
52	17419	Day 0	7/4/2006	NO LD SEEN
51	17422	Day 0	7/4/2006	NO LD SEEN
33	17423	Day 0	7/4/2006	4
31	17425	Day 0	7/4/2006	3
32	17426	Day 0	7/4/2006	4
11	24641	6 month	11/2/2006	NO LD SEEN
42	24642	6 month	5/4/2006	NO LD SEEN
47	24646	3 month	26-10-05	NO LD SEEN
10	24646	6 month	11/2/2006	NO LD SEEN
4	27952	3 month	16-11-05	5
1	29636	Day 31	19-11-97 E.C	4
50	29636	6 month	3/1/2006	NO LD SEEN
30	29636	Day 0	2/8/2005	5
17	29944	Day 0	28-09-05	NO LD SEEN
14	29945	Day 0	28-09-05	NO LD SEEN
13	33376	Day 0	28-09-05	NO LD SEEN
16	33378	Day 0	28-09-05	NO LD SEEN
28	33380	Day 0	4/1/1998	4
15	33381	Day 22	14-10-05	NO LD SEEN

Appendix 15 Drug re-analysis report from IDA

Received form HH 20 07 2005
Quality control and samples from DNDi study in Sudan.

Paromomycin sulphate 500mg/ml equivalent to 365mg/ml paromomycin base

	FB301X (Gland)			5032:0012 (PPL)		
	mfd 1-10-03 exp 01-09-06			mfd 1-04-02 exp 01-04-05		
	date	assay mg/ml	%	date	assay mg/ml	%
HPLC by Parmamed PPL	12-10-03	380	104,1%	18-6-02	378	103,5%
HPLC by Gland	7-4-05					
Micro assay by Gland	16-10-03	363,1	99,5%			
Repeat micro-biological assay by Gland	8-6-05	342,6	93,9%			
Repeat micro- biological assay by Gland during validation study	7-6-05	344,7	94,4%			
Independent laboratory #1 micro-biological assay	11-2-04	343	93,9%	11-2-04	372	102,0%
Independent laboratory #2 micro-biological assay<br>confidence interval	13-4-05	307<br>294-320	84,1%<br>81-88%	13-4-05	332<br>317-348	91,0%<br>87-95%
Independent laboratory #2 micro-biological assay<br>confidence interval	31-5-05	345<br>324-366	94,5%<br>89-100%			
All values recalculated to paromomycin base were applicable						

blue = sample received back from field from DNDi study
precision micro assay reported RSD 4.2%
original Gland analysis micro based on potency 717ug/g for batch FB301X

16.2.8 Documentation of statistical methods

Appendix 16 Statistical Analysis Plan

LEAP 0104

A MULTICENTRE INDIVIDUALLY RANDOMISED TRIAL OF EFFICACY AND SAFETY OF SODIUM STIBO-GLUCONATE (SSG) VERSUS PAROMOMYCIN (PM) AND VERSUS A COMBINATION OF SSG AND PM FOR THE TREATMENT OF VISCERAL LEISHMANIASIS IN ETHIOPIA, KENYA AND SUDAN

STATISTICAL ANALYSIS PLAN

Table of Contents

1. TRIAL OBJECTIVES 5

2. STUDY DESIGN 5

2.1 Study Sites 5
2.2 Inclusion Criteria 5
2.3 Exclusion Criteria 5
2.4 HIV-status and VCT 6
2.5 Dose Schedule 6

3. RANDOMISATION 6

4. PRIMARY ENDPOINT 6

5. SECONDARY ENDPOINTS 6

5.1 Efficacy 6
5.1.1 End of Treatment Parasitology 6
5.1.2 Parasitology at 3 months follow-up 6
5.2 Safety 7
5.2.1. Adverse events (AEs) and Serious adverse events (SAEs) 7
5.2.2. ECG and Audiometry 7
5.3. Biological Parameters 7

6. PATIENT ASSESSMENT SCHEDULE 7

7. ANALYSIS DEFINITIONS 8

7.1 Primary Efficacy Analysis 8
7.2 Secondary Efficacy Analysis 9
7.3 Analysis of ECG, Audiometry Biological Marker and AE Data 9

8. LEAP 0104A ANALYSIS 10

8.1 Participant Flow 10
8.2 Baseline Characteristics 11
8.3 Deviations from Protocol: Exclusion criteria at baseline 13
8.4 Primary Efficacy Analysis: Overall 14
8.4.1Primary Efficacy Analysis: By centre 15
8.5 Secondary Efficacy Parasitology Analysis 16
8.5.1 Secondary Efficacy Parasitology Analysis 16
8.6 Safety ECG and Audiometry Analysis 17
8.6.1 Safety Analysis: ECG 17
8.6.2 Safety Analysis: Audiometry 18
8.6.3 Urinalysis: Protein 18
8.6.4 Urinalysis: Blood 19
8.7 Secondary Efficacy Analysis: Biological Markers during Treatment 19
8.8 Adverse Events 21
8.8.1 Summary Results during treatment 22
8.8.2 Summary Results at 3 months follow-up 23
8.8.3 Summary Results at 6 months follow-up 23
8.8.4 Summary of All Adverse Events during Treatment Period and Follow-up by treatment 24
8.8.5 Summary of All Adverse Events during Treatment Period and Follow-up by severity 29

9. SUBGROUP ANALYSES 34

9.1 Primary Efficacy Analysis: HIV negative patients 34
9.2 Efficacy Analysis: HIV positive patients 35
9.3 Efficacy Analysis: Age 35

10. STATISTICAL METHODS 35

10.1 DATA SUMMARY AND COMPARISON........35
10.2 PARASITOLOGICAL EFFICACY........36
10.3 BIOLOGICAL MARKERS, ECG AND AUDIOMETRY........36
10.4 SERIOUS AND NON-SERIOUS ADVERSE EVENTS........36

11. POWER: EFFICACY ANALYSIS........37

12. REFERENCES........37

Tables

TABLE 1 PATIENT ASSESSMENT SCHEDULE........7
TABLE 2 IMPUTING VALUES FOR MISSING SIX MONTH PARASITOLOGICAL DATA........8
TABLE 3 BASELINE DEMOGRAPHIC CHARACTERISTICS........11
TABLE 4 BASELINE BIOLOGICAL MARKERS........11
TABLE 5 BASELINE LABORATORY PARAMETERS........12
TABLE 6 BASELINE CLINICAL CHARACTERISTICS........12
TABLE 7 BASELINE SYMPTOMS........13
TABLE 8 SUMMARY OF PATIENTS THAT DID NOT MEET PROTOCOL ENTRY CRITERIA........13
TABLE 9 DEFINITIVE CURE ANALYSIS 1: COMPLETE CASE ANALYSIS........14
TABLE 10 DEFINITIVE CURE ANALYSIS 2: LAST PARASITOLOGY CARRIED FORWARD........14
TABLE 11 DEFINITIVE CURE ANALYSIS 3 – WORST-CASE ANALYSIS........14
TABLE 12 PRIMARY EFFICACY ANALYSIS: BY CENTRE........15
TABLE 13 TEST OF CURE ANALYSIS 1: COMPLETE CASE ANALYSIS........16
TABLE 14 TEST OF CURE ANALYSIS 2: WORST CASE ANALYSIS........16
TABLE 15 SECONDARY EFFICACY ANALYSIS: BY CENTRE........16
TABLE 16 ECG........17
TABLE 17 AUDIOMETRY........18
TABLE 18 URINALYSIS: PROTEIN........18
TABLE 19 URINALYSIS: BLOOD........19
TABLE 20 PREDICTED CHANGES FOR BIOLOGICAL MARKERS DURING TREATMENT.... **ERROR! BOOKMARK NOT DEFINED.**
TABLE 21 DIFFERENCE IN BIOLOGICAL MARKERS BETWEEN BASELINE AND DAY 7........20
TABLE 22 DIFFERENCE IN BIOLOGICAL MARKERS BETWEEN DAY 7 AND DAY 14........20
TABLE 23 DIFFERENCE IN BIOLOGICAL MARKERS BETWEEN DAY 14 AND END OF TREATMENT........21
TABLE 24 ADVERSE EVENT RATES DURING TREATMENT.........22
TABLE 25 ADVERSE EVENTS AT 3 MONTHS FOLLOW-UP........23
TABLE 26 ADVERSE EVENTS AT 6 MONTHS FOLLOW-UP........23
TABLE 27 ALL ADVERSE EVENTS SUMMARISED BY TREATMENT........24
TABLE 28 ALL ADVERSE EVENTS SUMMARISED BY SEVERITY........29
TABLE 29 DEFINITIVE CURE ANALYSIS 1: COMPLETE CASE ANALYSIS........34
TABLE 30 DEFINITIVE CURE ANALYSIS 2: LAST PARASITOLOGY CARRIED FORWARD........34
TABLE 31 DEFINITIVE CURE ANALYSIS 3 – WORST-CASE ANALYSIS........34
TABLE 32 DEFINITIVE CURE SUMMARY FOR HIV POSITIVE PATIENTS........35

Figures

FIGURE 1 LEAP 0104A SCREENING AND ENROLMENT: ALL SITES........10
FIGURE 2 LEAP 0104A TREATMENT: ALL SITES........10
FIGURE 3 LEAP 0104A FOLLOW-UP: ALL SITES........10

Abbreviations

AE	Adverse event
ALT	Alanine aminotransferase (SGPT)
AP	Alkaline Phosphatase
AST	Aspartate aminotransferase (SGOT)
CBC	Complete blood count
CRF	Case report form
DNDi	Drugs for neglected diseases initiative
IEC	Independent ethics committee
FDA	Food and Drug Administration
GCP	Good clinical practice
ICH	International Conferences on Harmonization
IV	Intravenous
PI	Principal investigator (see note Section 13)
SAE	Serious adverse event
ULN	Upper limit of normal
WBC	White blood cell
WHO	World Health Organization
WNL	Within normal limits
SSG	Sodium Stibogluconate
PM	Paromomycin
Comb	SSG and PM Combination treatment
CI	Confidence Interval
VCT	Voluntary counselling and testing
TOC	Test of Cure
SD	Standard Deviation
IQR	Interquartile range
DC	Definitive Cure, parasite free 6 months post treatment, no rescue or additional VL medication required

1. Trial Objectives

To compare the efficacy and safety of:

- o Sodium Stibogluconate (SSG) monotherapy
- o Paromomycin (PM) monotherapy
- o SSG and PM in a combination therapy (Comb)

2. Study Design

Multi-centre individually randomised controlled trial.

2.1 Study Sites

- Kenya:
    - o KEMRI, Nairobi (Co-ordinating Centre)
- Sudan:
    - o Um El Kher (UeK)
    - o Kassab
- Ethiopia:
    - o Gondar
    - o Arba Minch

2.2 Inclusion Criteria

- Patients for whom written informed consent has been signed by the patients themselves (if aged 18 years and over) or by parents(s) or legal guardian for patients under 18 years of age.
- Patients aged between 4 and 60 years (inclusive).
- Patients with clinical signs and symptoms of VL and diagnosis confirmed by visualization of parasites in tissue samples (spleen, lymph node or bone marrow) on microscopy.

2.3 Exclusion Criteria

- Patients who have received any anti-leishmanial drug in the last 6 months.
- Patients with a clinical contraindication to splenic/lymph node/ bone marrow aspirates.
- Patients with severe protein and or caloric malnutrition (Kwashiokor or marasmus)
- Patients with previous hypersensitivity reaction to SSG or aminoglycosides.
- Patients suffering from a concomitant severe infection such as TB or any other serious underlying disease (cardiac, renal, hepatic) which would preclude evaluation of the patient's response to study medication.
- Patients suffering from other conditions associated with splenomegaly such as schistosomiasis.
- Patients with previous history of cardiac arrhythmia or an abnormal ECG
- Patients who are pregnant or lactating.
- Patients with haemoglobin < 5gm/dl.
- Patients with WBC < 1 x 10^3/mm
- Patients with platelets < 40,000/mm
- Patients with liver function tests more than three times the normal range
- Patients with serum creatinine outside the normal range for age and gender.
- Patients with pre-existing clinical hearing loss

2.4 HIV-status and VCT

All patients were offered counselling and screening for HIV under a voluntary counselling and testing programme (VCT). This was either to be done at the same time as consent was obtained for inclusion in the trial or at a later date according to hospital practice. **A HIV positive result is not an exclusion criterion**.

Subset analysis will be performed to assess any differences in response within the strata

- HIV negative
- HIV positive
- HIV status unknown

2.5 Dose Schedule

Treatment was administered by IM or IV at the same time each day according to the following schedule for each regimen;

- SSG monotherapy: 20mg/kg/day for 30 days, with a maximum daily dose of 850mg (8.5ml) per patient except in Sudan where there was no maximum dose
- PM monotherapy: 15 mg/kg/day for 21 days.
- Combination SSG 20mg/kg/day and PM 15 mg/kg/day for 17 days

3. Randomisation

Restricted block randomization was performed for the three arms per site within each country. Block sizes of 15 were used. Opaque envelopes were numbered sequentially and then sealed. The process was carried out at the DNDi Trial Co-ordinating centre at KEMRI, Nairobi where a copy of the randomisation schedule is kept securely.

4. Primary Endpoint

Parasitology at 6 months follow up: measured by visualization of parasites in tissue samples (spleen, lymph node or bone marrow) on microscopy.

5. Secondary Endpoints

5.1 Efficacy

5.1.1 End of Treatment Parasitology

Parasitology at end of treatment: measured by visualization of parasites in tissue samples (spleen, lymph node or bone marrow) on microscopy.

The tissue sample is taken on the day following the last day of treatment:

- SSG: Day 31
- PM: Day 22
- Combination: Day 18

5.1.2 Parasitology at 3 months follow-up

Follow up at 3 months is optional, dependent on investigator concerns following discharge and seasonal access to remote communities.

Patients who attend for follow up are examined for clinical symptoms of VL and classed as clinically well or clinically unwell. Parasitology, measured by visualization of parasites in tissue samples (spleen, lymph node or bone marrow) on microscopy, is performed in patients who are clinically unwell.

5.2 Safety

5.2.1. Adverse events (AEs) and Serious adverse events (SAEs)

Recorded by spontaneous recording and active examination and questioning.
AEs will be coded according to MEDRA version 10.0

5.2.2. ECG and Audiometry

Recorded at specified assessment times (see Section 10) and categorised as Normal or Abnormal by site investigators at assessment times.

5.3. Biological Parameters

The following parameters were measured at specified assessment times, according to schedule specified in the next section.

- Temperature: (˚Celsius)
- Spleen size: (centimetres (cm) by palpation below left costal margin in the line of growth)
- Liver size: (cm by palpation below right costal margin in the mid-clavicular line)
- Weight: (kg)
- Haemoglobin: (g/dl)
- Platelet Count: (x 10^3/µl)
- White Cell Count: (x 10^3/µl)
- Heart Rate: (beats per minute)
- Systolic Blood Pressure: (mm Hg)
- Diastolic Blood Pressure: (mm Hg)
- Total Bilirubin: (mg/dl)
- BUN: (mg/dl)
- Creatinine: (mg/dl)
- AST: (U/L)
- ALT: (U/L)
- Albumin: (gm/dl)
- Amylase: (micromol/L)

6. Patient Assessment Schedule

Table 1 below shows patient assessment schedule for efficacy, ECG and Audiometry and biological markers parameters by day of treatment and follow-up at three and six months.

Table 1 Patient Assessment Schedule

Assessments	Day of treatment					Follow-up	
	0	7	14	21*	End of Treatment †	3 Months	6 Months
Efficacy: Parasitology ‡	✓				✓	✓	✓
ECG and Audiometry‖	✓		✓		✓	✓	✓
Biological Markers §	✓	✓	✓	✓	✓	✓	✓

* SSG only
† End of treatment is on Day 31 for SSG, Day 22 for Paromomycin and day 18 for Combination
‡ Only measured at 3 months if patient clinically unwell
‖ ECG and audiometry also carried out on Day 7 and Day 21 at KEMRI and Kassab, Audiometry not done at Um el Kehr
§ Temperature, Spleen size, Liver Size, Weight, Haemoglobin, Platelets,White cell count, heart Rate, Systolic and Diastolic blood pressure, bilirubin, BUN, Creatinine, ALT, AST

7. Analysis Definitions

7.1 Primary Efficacy Analysis

Treatment success, or Definitive Cure (DC), is defined as

- Absence of parasites on microscopy slide at 6 months, provided no rescue medication was given during treatment or follow up period.

Treatment failure is defined as

- receipt of rescue medication at any point in the trial
- parasites visualized on a microscopy slide at 6 months in patients who have *not* received rescue medication during treatment or follow up period

The efficacy in each arm will be the proportion of treatment successes. The treatment effect will be calculated as the difference in efficacy between SSG and PM arms and SSG and Combination at 6 months follow-up.

If any of 6 months parasitology data are missing (only likely in the case of death, loss to follow up or withdrawal of consent) efficacy analyses will be carried out in three ways;

- Complete case analysis: excluding patients with missing data from estimation of efficacy in each arm
- Last parasitology carried forward: Patients with missing efficacy data will have their last parasitology results carried forward (Table 2).
- Worst case analysis: Patients with missing efficacy data will be allocated efficacy results based on their ‘worst-case’ scenario i.e. treatment failure.

Table 2 shows possible scenarios at 6 month parasitology result based on last parasitological measurements carried forward.

Table 2 Imputing values for missing six month parasitological data

**Scenario***		**Assumed parasitology result at 6 months**	**Patient Treatment Outcome**
1	Parasitological measurements taken at 3 months: parasites visualised	Positive	Failure
2	Parasitological measurements taken at 3 months: parasites not visualised	Negative	Success
3	Patient attended for 3 month visit: Parasitological measurements not taken but parasites visualised at end of treatment	Positive	Failure
4	Patient attended for 3 month visit: Parasitological measurements not taken but parasites not visualised at end of treatment	Negative	Success
5	Last seen at end of treatment: parasites visualised	Positive	Failure
6	Last seen at end of treatment: parasites not visualised	Negative	Success

* receipt of rescue medication prior to 6 months follow-up visit is considered a treatment failure

If a patient withdraws full consent at any time in the trial period, data collection, for the purposes of the trial, ceases and any primary or secondary outcome data beyond this point will be missing.

Patients whose baseline characteristics meet exclusion criteria (as additional protocol violations) will be included in the analyses.

7.2 Secondary Efficacy Analysis

Treatment success, or Test of Cure (TOC), is defined as:

- Absence of parasites on microscopy slide at end of treatment provided no rescue medication was given during treatment period.

Slow response is defined as:

- Presence of parasites on microscopy slide at end of treatment, with a value of at least 2 log values lower than the baseline measurement and no rescue medication given during treatment period.

Treatment failure is defined as

- receipt of rescue medication during treatment period or following cessation of study treatment

The treatment effect will be calculated as the difference in efficacy between SSG and PM arms and SSG and Combination at end of treatment.

If any end of treatment parasitology data are missing (only likely to be due to withdrawal of consent) efficacy analyses will be carried out in two ways;

- complete case analysis: excluding patients with missing data from estimation of efficacy in each arm
- worst case analysis: patients with missing efficacy data will be allocated efficacy results based on their ‘worst-case’ scenario i.e. treatment failure.

If a patient withdraws full consent at any time in the trial period, data collection, for the purposes of the trial, ceases and any primary or secondary outcome data beyond this point will be missing.

Patients whose baseline characteristics meet exclusion criteria (as additional protocol violations) will be included in the analyses.

Analyses assume that end of treatment time points are comparable, by definition of the treatment regimens.

7.3 Analysis of ECG, Audiometry Biological Marker and AE Data

Data collected during treatment will be analysed according to treatment allocation, using data from two groups of patients;

- Patients completing treatment period and patients withdrawn from treatment up until the point of withdrawal
- Patients completing treatment period and patients withdrawn from treatment up until the end of treatment

8. LEAP 0104A Analysis

8.1 Participant Flow

Figure 1 LEAP 0104A Screening and Enrolment: all sites

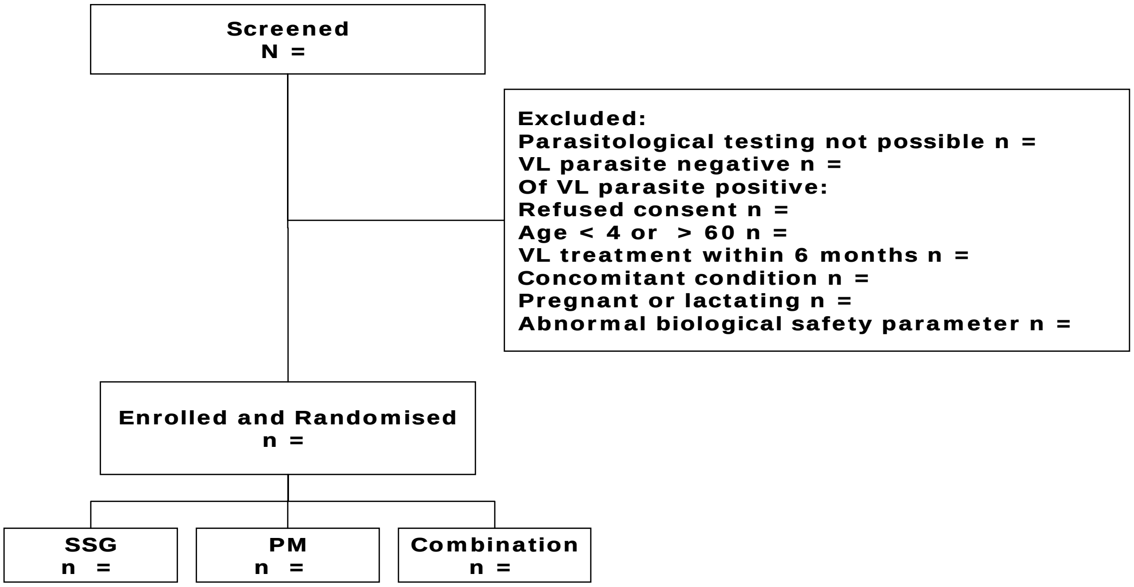

Figure 2 LEAP 0104A Treatment: All sites

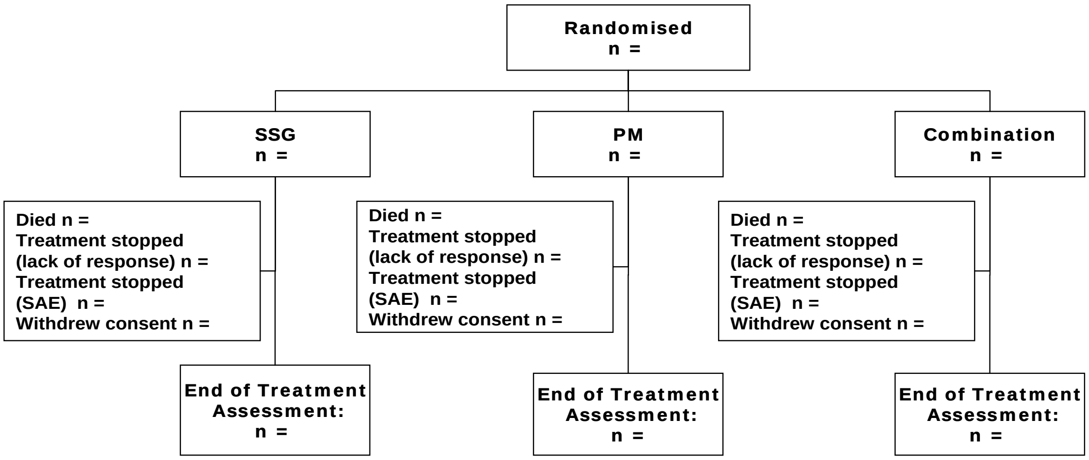

Figure 3 LEAP 0104A Follow-up: All sites

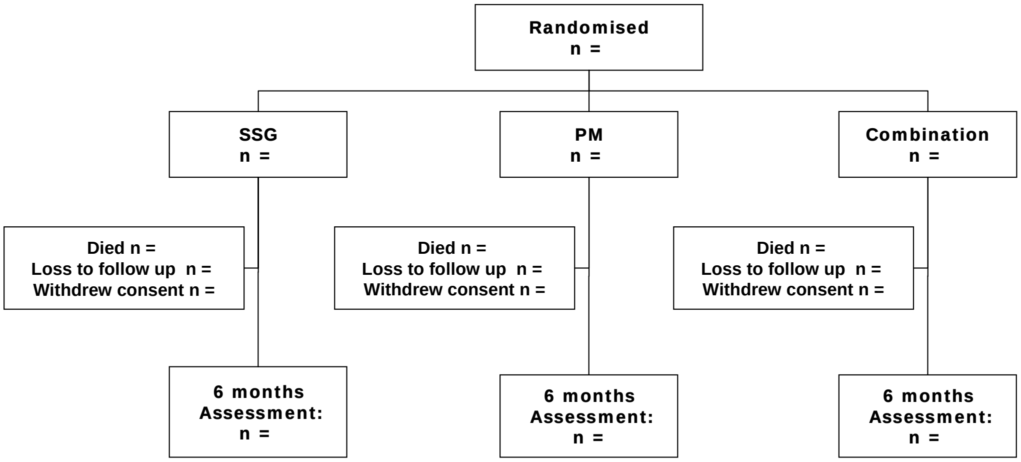

8.2 Baseline Characteristics

Table 3 Baseline Demographic characteristics

**Demographics, n (%)**		**SSG N =**	**PM N =**	**Comb N =**	**p-value***	**p-value†**
Age (years) ‡	mean (SD)					
	4 – 14					
	≥ 15					
Sex	Female					
	Male					
Randomised at Centre	Kenya					
	Um El Kher					
	Kassab					
	Gondar					
	Arba Minch					

* p-value from ANOVA for age comparison and chi-squared test for sex and categorical age comparisons, across all arms

† p-value from t-test for age comparison and chi-squared test for sex and categorical age comparisons, between SSG and PM

‡ Children classified as aged 4 to 14 years and adults, 15 years and above.

Table 4 Baseline Biological Markers

		**SSG N =**	**PM N =**	**Comb N =**	**p-value***	**p-value†**
Temperature (°C)	mean (SD)					
	median (IQR)					
Heart Rate, (beats/min)	mean (SD)					
	median (IQR)					
Spleen Size (cm)	mean (SD)					
	median (IQR)					
Liver Size (cm)	mean (SD)					
	median (IQR)					
Weight for age (children)‡	Severely underweight: <60%					
	Underweight:60-80%					
	Normal: >80%					
BMI (adults)‡	Severely underweight: <16					
	Underweight: 16.0-18.4					
	Normal weight:18.5-24.9					

* p-value from ANOVA for comparison of mean values, Kruskall-Wallis where median values are given and chi-squared test for categorical data comparisons.

† p-value from t-test for comparison of means and chi-squared test for sex and categorical age comparisons, between SSG and PM

‡ Children classified as aged 4 to 14 years and adults, 15 years and above.

Table 5 Baseline Laboratory parameters

Laboratory parameters*		SSG N =	PM N =	Comb N =	p-value†	p-value‡
Parasite Count (log scale)	6+					
	5+					
	4+					
	3+					
	2+					
	1+					
Haemoglobin (g/dl)	mean (SD)					
	median (IQR)					
White-cell Count ($x10^3/\mu L$)	mean (SD)					
	median (IQR)					
Platelets ($x10^3/\mu L$)	mean (SD)					
	median (IQR)					
AST, (U/L)	mean (SD)					
	median (IQR)					
ALT, (U/L)	mean (SD)					
	median (IQR)					
Bilirubin, (mg/dl)	mean (SD)					
	median (IQR)					
BUN, (mg/dl)	mean (SD)					
	median (IQR)					
Creatinine (mg/dl)	mean (SD)					
	median (IQR)					
Amylase, (micromol/L)	mean (SD)					
	median (IQR)					
Alkaline Phosphatase, (U/L)	mean (SD)					
	median (IQR)					
HIV	Positive, n (%)					
	Negative, n (%)					
	Not tested n (%)					

* Measurements were not done at all the sites; WBC not measured at Um El Kher, Alkaline Phosphatase not measured at Kassab, Albumin only measured at Kenya and Kassab, Serum amylase only measured at Arba Minch.

† p-value from ANOVA for comparison of mean values, Kruskall-Wallis where median values are given and chi-squared test for categorical data comparisons.

‡ p-value from t-test for comparison of means and chi-squared test for sex and categorical age comparisons, between SSG and PM

Table 6 Baseline clinical characteristics

Clinical characteristics, n (%)		SSG N =	PM N =	Comb N =	p-value*	p-value†
Audiometry‡	Normal					
	Abnormal					
	Not done					
ECG‡	Normal					
	Abnormal					
	Not done					
Malaria prior to treatment						
Pneumonia						
Otitis Media						

* p-value from chi-squared test across all arms

† p-value from chi-squared test between SSG and PM

‡ Abnormal readings considered to be clinically significant

Table 7 Baseline Symptoms

Symptom, n (%)	SSG N =	PM N =	Comb N =	p-value*	p-value†
Fever					
Headache					
Fatigue					
Epistaxis					
Abdominal pains					
Abdominal swellings					
Swelling of legs					
Cough					
Breathlessness					
Night Sweats					
Loss of appetite					
Weight loss					
Diarrhoea					
Skin lesions					

* p-value from chi-squared test across all arms
† p-value from chi-squared test between SSG and PM

8.3 Deviations from Protocol: Exclusion criteria at baseline

If any patients are found not to have met inclusion and/or exclusion criteria at recruitment, a summary of the criteria will be given here.

Table 8 Summary of patients that did not meet protocol entry criteria

Entry Criteria, n	SSG N =	PM N =	Comb N =
Patients aged < 4 or > 60 years			
Patients not diagnosed with VL			
Patients that received anti-leishmanial drug in the last 6 months			
Patients with malnutrition			
Patients with haemoglobin < 5gm/dl			
Patients with WBC < 1 x 10^3/mm			
Patients with platelets < 40,000/mm			
Patients with liver function tests more than 3 times ULN			
Patients with creatinine outside the normal range for age & gender			
Patients with pre-existing clinical hearing loss			
Patients with history of cardiac arrhythmia or an abnormal ECG			
Patients suffering from a concomitant severe infection			

8.4 Primary Efficacy Analysis: Overall

Table 9 Definitive Cure Analysis 1: Complete Case Analysis

**Estimation**	**SSG N =**	**PM N =**	**Comb N =**
Treatment Efficacy at 6 months, n (%)			
Test of difference across arms: p-value*			
Difference between SSG & PM (95% CI)			
Difference between SSG & Combination (95% CI)			
Test of difference across centres, after adjustment for treatment : p-value*			

* p-value from likelihood ratio test, comparing models with and without variable being tested.

Table 10 Definitive Cure Analysis 2: Last parasitology carried forward

**Estimation**	**SSG N =**	**PM N =**	**Comb N =**
Treatment efficacy at 6 months, n (%)			
Test of difference across arms: p-value*			
Difference between SSG & PM (95% CI)			
Difference between SSG & Combination (95% CI)			
Test of difference across centres, after adjustment for treatment: p-value*			

* p-value from likelihood ratio test, comparing models with and without variable being tested.

Table 11 Definitive Cure Analysis 3 – Worst-case analysis

**Estimation**	**SSG N =**	**PM N =**	**Comb N =**
Parasite free at 6 months, n (%)			
Test of difference across arms: p-value*			
Difference between SSG & PM (95% CI)			
Difference between SSG & Combination (95% CI)			
Test of difference across centres, after adjustment for treatment: p-value*			

* p-value from likelihood ratio test, comparing models with and without variable being tested.

8.4.1 Primary Efficacy Analysis: By centre

If the LRT p-value following comparison of models with and without centre ≤ 0.05, by-centre efficacy analyses will be presented.

Table 12 Primary Efficacy Analysis: By Centre

Site	Numbers randomised and treatment successes	SSG N =	PM N =	Comb N =	p-value*†	p-value*‖
Um El Kher	Randomised					
	DC: complete case					
	DC: parasitology carried forward					
	DC: worst case					
Kassab	Randomised					
	DC: complete case					
	DC: parasitology carried forward					
	DC: worst case					
Kenya	Randomised					
	DC: complete case					
	DC: parasitology carried forward					
	DC: worst case					
Gondar	Randomised					
	DC: complete case					
	DC: parasitology carried forward					
	DC: worst case					
Arba Minch	Randomised					
	DC: complete case					
	DC: parasitology carried forward					
	DC: worst case					
p-value*‡	DC: complete case					
	DC: parasitology carried forward					
	DC: worst case					

* p-value from chi-squared test or Fisher's exact test if justified
† comparison across arms by site
‖ comparison between SSG and PM by site
‡ comparison across sites by arm

8.5 Secondary Efficacy Parasitology Analysis

Table 13 Test of Cure Analysis 1: Complete Case Analysis

Estimation	SSG<br>N =	PM<br>N =	Comb<br>N =
Treatment Success at end of treatment, n (%)			
Test of difference across arms: p-value*			
Difference between SSG & PM (95% CI)			
Difference between SSG & Combination (95% CI)			
Test of difference across centres, after adjustment for treatment: p-value*			

* p-value from likelihood ratio test, comparing models with and without variable being tested.

Table 14 Test of Cure Analysis 2: Worst Case Analysis

Estimation	SSG<br>N =	PM<br>N =	Comb<br>N =
Treatment Success at end of treatment, n (%)			
Test of difference across arms: p-value*			
Difference between SSG & PM (95% CI)			
Difference between SSG & Combination (95% CI)			
Test of difference across centres, after adjustment for treatment: p-value*			

* p-value from likelihood ratio test, comparing models with and without variable being tested.

8.5.1 Secondary Efficacy Parasitology Analysis

If the LRT p-value following comparison of models with and without centre ≤ 0.05, by-centre efficacy analyses will be presented.

Table 15 Secondary Efficacy Analysis: By Centre

Site	Numbers randomised and treatment successes	SSG<br>N =	PM<br>N =	Comb<br>N =	p-value*
Um El Kher	Randomised				
	TOC: complete case				
	TOC: worst case				
Kassab	Randomised				
	TOC: complete case				
	TOC: worst case				
Kenya	Randomised				
	TOC: complete case				
	TOC: worst case				
Gondar	Randomised				
	TOC: complete case				
	TOC: worst case				
Arba Minch	Randomised				
	TOC: complete case				
	TOC: worst case				
p-value*	TOC: complete case				
	TOC: worst case				

* p-value from chi-squared test or Fisher's exact test if justified

8.6 Safety ECG and Audiometry Analysis

Data coded as normal or abnormal on treatment assessment days will be summarised using the following proportions;

- proportion of patients who have an abnormal reading *on* day 7
- proportion of patients who have an abnormal reading *on* day 14
- proportion of patients who have had an abnormal reading *by* day 14 (abnormal reading on day 7, 14 or both)
- proportion of patients who have had an abnormal reading *by* end of treatment (abnormal reading on day 7, 14, end of treatment or all)
- proportion of patients who have an abnormal reading at end of treatment
- proportion of patients who have an abnormal reading at end of treatment that has not returned to normal at 6 months

The denominator for all proportion will the number of patients randomised to treatment who recorded normal at baseline.

8.6.1 Safety Analysis: ECG

Table 16 ECG

**Estimation**	**SSG N =**	**PM N =**	**Comb N =**
Number of patients randomised			
Abnormal *on* day 7, n (%)			
Abnormal *on* day 14, n (%)			
Abnormal *by* day 14, n (%)			
Abnormal *by* end of treatment, n (%)			
Abnormal result *at* end of treatment, n (%)			
Abnormal result remaining at 6 months†, n (%)			
Test of Difference across all arms *at* end of treatment* If p ≤ 0.05, perform pairwise tests:			
Between SSG & PM (95% CI):			
Test of difference*			
Between SSG & Combination (95% CI):			
Test of difference*			
Test of Difference across all arms at 6 months * If p ≤ 0.01, perform pairwise tests:			
Between SSG & PM (95% CI):			
Test of difference*			
Between SSG & Combination (95% CI):			
Test of difference*			

* p-value from chi-squared test
† abnormal result at end of treatment, not returning to normal by end of follow-up

8.6.2 Safety Analysis: Audiometry

Table 17 Audiometry

**Estimation**	**SSG N =**	**PM N =**	**Comb N =**
Abnormal *on* day 7, n (%)			
Abnormal *on* day 14, n (%)			
Abnormal *by* day 14, n (%)			
Abnormal *by* end of treatment, n (%)			
Abnormal result *at* end of treatment, n (%)			
Abnormal result remaining at 6 months†, n (%)			
Test of Difference across all arms in result *at* end of treatment*<br>If p ≤ 0.05, perform pairwise tests:			
Between SSG & PM (95% CI):			
Test of difference*			
Between SSG & Combination (95% CI):			
Test of difference*			
Test of Difference across all arms at 6 months†*<br>If p ≤ 0.01, perform pairwise tests:			
Between SSG & PM (95% CI):			
Test of difference*			
Between SSG & Combination (95% CI):			
Test of difference*			

* p-value from chi-squared test
† abnormal result at end of treatment, not returning to normal by end of follow-up

8.6.3 Urinalysis: Protein

Table 18 Urinalysis: Protein

**Estimation**	**SSG N =**	**PM N =**	**Comb N =**
Number of patients randomised			
Abnormal *on* day 7, n (%)			
Abnormal *on* day 14, n (%)			
Abnormal *by* day 14, n (%)			
Abnormal *by* end of treatment, n (%)			
Abnormal result *at* end of treatment, n (%)			
Test of Difference across all arms in result *at* end of treatment*<br>If p ≤ 0.05, perform pairwise tests:			
Between SSG & PM (95% CI):			
Test of difference*			
Between SSG & Combination (95% CI):			
Test of difference*			

* p-value from chi-squared test

8.6.4 Urinalysis: Blood

Table 19 Urinalysis: Blood

**Estimation**	**SSG N =**	**PM N =**	**Comb N =**
Number of patients randomised			
Abnormal *on* day 7, n (%)			
Abnormal *on* day 14, n (%)			
Abnormal *by* day 14, n (%)			
Abnormal *by* end of treatment, n (%)			
Abnormal result *at* end of treatment, n (%)			
Test of Difference across all arms in result *at* end of treatment*			
If p ≤ 0.05, perform pairwise tests:			
Between SSG & PM (95% CI):			
Test of difference*			
Between SSG & Combination (95% CI):			
Test of difference*			

* p-value from chi-squared test

8.7 Secondary Efficacy Analysis: Biological Markers during Treatment

Predicted changes during treatment are presented with corresponding 95% CIs along with results of overall statistical comparisons across all arms. Pairwise results are only performed where there is some evidence of an overall difference.

Mean differences in parameters between time points are also presented with corresponding 95% CIs. ANCOVA tests for evidence of a difference between treatment arms also allow for adjustment for centre.

All available data will be used in safety analyses.

**Biological Parameter**		**SSG**	**PM**	**Comb**	**p-value***		
					**LRT Overall**	**SSG v PM†‖**	**SSG v Comb†**
Weight gain (Kg)	n						
	Predicted change						
	95% CI						
Temperature (°C)	n						
	Predicted change						
	95% CI						
Heart Rate, (beats/min)	n						
	Predicted change						
	95% CI						
Spleen Size (cm)	n						
	Predicted change						
	95% CI						
Liver Size (cm)	n						
	Predicted change						
	95% CI						

§ p-value from random effects regression modelling
‖ Wald test p-value if overall LRT comparison p-value ≤ 0.05

Table 20 Difference in biological markers between baseline and Day 7

Efficacy Marker	Mean Difference (95% CI)			p-value*		
	SSG	PM	Comb	Overall	SSG v PM	SSG v Comb
Weight gain (Kg)						
Temperature (°C)						
Heart Rate, (beats/min)						
Spleen Size (cm)						
Liver Size (cm)						
Haemoglobin (g/dl)						
White-cell Count (x10^3/µL)						
Platelets (x10^3/µL)						
AST, (U/L)						
ALT, (U/L)						
Bilirubin, (mg/dl)						
BUN, (mg/dl)						
Creatinine (mg/dl)						
Amylase, (micromol/L)						
Alkaline Phosphatase (U/L)						

* p-value from ANCOVA between arms in day 7 measurements, adjusting for baseline values

Table 21 Difference in biological markers between Day 7 and Day 14

Efficacy Marker	Mean Difference (95% CI)			p-value*		
	SSG	PM	Comb	Overall	SSG v PM	SSG v Comb
Weight gain (Kg)						
Temperature (°C)						
Heart Rate, (beats/min)						
Spleen Size (cm)						
Liver Size (cm)						
Haemoglobin (g/dl)						
White-cell Count (x10^3/µL)						
Platelets (x10^3/µL)						
AST, (U/L)						
ALT, (U/L)						
Bilirubin, (mg/dl)						
BUN, (mg/dl)						
Creatinine (mg/dl)						
Amylase, (micromol/L)						
Alkaline Phosphatase (U/L)						

* p-value from ANCOVA between arms in day 14 measurements, adjusting for day 7 values

Table 22 Difference in biological markers between Day 14 and End of treatment

Efficacy Marker	Mean Difference (95% CI)			p-value*		
	SSG	PM	Comb	Overall	SSG v PM	SSG v Comb
Weight gain (Kg)						
Temperature (°C)						
Heart Rate, (beats/min)						
Spleen Size (cm)						
Liver Size (cm)						
Haemoglobin (g/dl)						
White-cell Count ($x10^3/\mu L$)						
Platelets ($x10^3/\mu L$)						
AST, (U/L)						
ALT, (U/L)						
Bilirubin, (mg/dl)						
BUN, (mg/dl)						
Creatinine (mg/dl)						
Amylase, (micromol/L)						
Alkaline Phosphatase (U/L)						

* P-value from ANCOVA between arms in end of treatment measurements (day 31 for SSG, day 22 for PM, day 18 for Combination), adjusting for day 14 values

8.8 Adverse Events

Adverse events will be summarised separately during treatment and follow-up. Severity and relation to study drug will be considered and summaries of numbers of AEs experienced will be given according to Medra, version 10.0.

8.8.1 Summary Results during treatment

Table 23 Adverse Event Rates during Treatment.

	Total person-days on treatment (T) & rate per arm*			SSG vs PM			SSG vs Comb		
	SSG T =	PM T =	Comb T =	Rate Ratio (95% CI)	Correlation p-value†	Adjusted Rate Ratio‡ (95% CI)	Rate Ratio (95% CI)	Correlation p-value†	Adjusted Rate Ratio‡ (95% CI)
**Serious adverse event**									
Any									
Adverse drug reaction§									
Unrelated to study drug‖									
**Non-Serious adverse event**									
Any									
Adverse drug reaction§									
Unrelated to study drug‖									
Mild									
Moderate									
Severe									

* multiple AEs per patient treated as separate AEs so contributes to the person-days on treatment per arm.
† p-value from random effects poisson regression to show strength of evidence of correlation within patients
‡ rate ratio adjusted for correlation between patients where there are multiple AEs per patient and evidence of correlation
§ Recorded as possible, probable or unlikely relation to study drug
|| Recorded as unrelated to study drug

8.8.2 Summary Results at 3 months follow-up

Table 24 Adverse Events at 3 months follow-up

**Patients experiencing at least one AE**	Absolute risk*			SSG vs PM		SSG vs Comb	
	SSG N =	PM N =	Comb N =	Relative Risk (95% CI)	Risk Difference†	Relative Risk (95% CI)	Risk Difference†
Any							
Adverse drug reaction§							
Unrelated to study drug||							
Mild							
Moderate							
Severe							

* data are n (%)
† negative difference implies an absolute risk decrease for experimental treatment compared to SSG
§ a non-serious adverse event recorded as probably, possibly or unlikely to be related to study drug
|| a non-serious adverse event recorded as unrelated to study drug

8.8.3 Summary Results at 6 months follow-up

Table 25 Adverse Events at 6 months follow-up

**Patients experiencing at least one AE**	Absolute risk*			SSG vs PM		SSG vs Comb	
	SSG N =	PM N =	Comb N =	Relative Risk (95% CI)	Risk Difference†	Relative Risk (95% CI)	Risk Difference†
Any							
Adverse drug reaction§							
Unrelated to study drug||							
Mild							
Moderate							
Severe							

* data are n (%)
† negative difference implies an absolute risk decrease for experimental treatment compared to SSG
§ a non-serious adverse event recorded as probably, possibly or unlikely to be related to study drug
|| a non-serious adverse event recorded as unrelated to study drug

8.8.4 Summary of All Adverse Events during Treatment Period and Follow-up by treatment

Table 26 All adverse events summarised by treatment

Adverse Event	SSG		PM		Comb	
Body system (Preferred term)	Related*	Not related†	Related*	Not related†	Related*	Not related†
**BLOOD AND LYMPHATIC DISORDERS**<br>ANAEMIA<br>EOSINOPHILIA<br>LEUKOCYTOSIS<br>THROMBOCYTOPENIA						
**CARDIAC DISORDERS**<br>BRADYCARDIA<br>CARDIAC FAILURE HIGH OUTPUT<br>SINUS ARRHYTHMIA<br>SINUS BRADYCARDIA						
**EAR AND LABYRINTH DISORDERS**<br>EAR PAIN						
**ENDOCRINE DISORDERS**						
**EYE DISORDERS**<br>CONJUCTIVITIS ALLERGIC<br>CONJUNCTIVITIS<br>EYE PAIN						
**GASTROINTESTINAL DISORDERS**<br>ABDOMINAL DISTENSION<br>ABDOMINAL PAIN<br>ABDOMINAL PAIN LEFT<br>ABDOMINAL PAIN UPPER<br>ABDOMINAL SEPSIS<br>ASCITES<br>DENTAL CARIES<br>DUODENAL ULCER<br>DYSPEPSIA<br>GASTRITIS<br>GINGIVAL BLEEDING						

Adverse Event	SSG		PM		Comb	
**Body system (Preferred term)**	**Related***	**Not related†**	**Related***	**Not related†**	**Related***	**Not related†**
GINGIVITIS<br>MOUTH ULCERATION<br>MUCOUS STOOLS<br>NAUSEA<br>ORAL SOFT TISSUE DISORDER<br>OESOPHAGEAL VARICES<br>PANCREATITIS<br>PERITONEAL HAEMORRHAGE<br>PERITONITIS<br>VOMITING						
**GENERAL DISORDERS AND ADMINISTRATION SITE CONDITIONS**<br>CHILLS<br>INJECTION SITE PAIN<br>INJECTION SITE SWELLING<br>PYREXIA<br>TENDERNESS						
**HEPATOBILIARY DISORDERS**						
**IMMUNE SYSTEM DISORDERS**<br>ALLERGY TO ARTHROPOD BITE						
**INFECTIONS AND INFESTATIONS**<br>AMOEBIC DYSENTERY<br>BODY TINEA<br>CONJUNCTIVITIS INFECTIVE<br>CROUP INFECTIOUS<br>ASCARIASIS<br>CELLULITIS<br>DYSENTERY<br>EAR INFECTION<br>FUNGAL SKIN INFECTION<br>GASTROENTERITIS<br>GIARDIASIS<br>HOOKWORM INFECTION<br>INJECTION SITE CELLULITIS						

**Adverse Event**	**SSG**		**PM**		**Comb**	
**Body system (Preferred term)**	**Related***	**Not related†**	**Related***	**Not related†**	**Related***	**Not related†**
LARYNGITIS<br>LYMPHADENITIS BACTERIAL<br>MALARIA<br>MOLLUSCUM CONTAGIOSUM<br>NASOPHARYNGITIS<br>OTITIS MEDIA<br>OTITIS MEDIA ACUTE<br>PARASITIC INFECTION INTESTINAL<br>PERICARDITIS MYCOPLASMAL<br>PNEUMONIA<br>PNEUMONIA PRIMARY ATYPICAL<br>STRONGYLOIDIASIS<br>TINEA CAPITIS<br>TUBERCULOSIS<br>UPPER RESPIRATORY TRACT INFECTION<br>URINARY TRACT INFECTION<br>VARICELLA<br>VISCERAL LEISHMANIASIS<br>WOUND SEPSIS						
**INJURY, POISONING AND PROCEDURAL COMPLICATIONS**<br>CONTUSION<br>SCRATCH<br>SKIN LACERATION<br>WOUND						
**INVESTIGATIONS**<br>ALANINE AMINOTRANSFERASE INCREASED<br>ASPARTATE AMINOTRANSFERASE INCREASED<br>BLOOD AMYLASE INCREASED<br>BLOOD ALKALINE PHOSPHATASE INCREASED<br>BLOOD CREATININE INCREASED<br>ELECTROCARDIOGRAM CHANGE<br>ELECTROCARDIOGRAM QT PROLONGED<br>HEPATIC ENZYMES INCREASED						

Adverse Event<br>Body system (Preferred term)	SSG		PM		Comb	
	Related*	Not related†	Related*	Not related†	Related*	Not related†
HAEMAGLOBIN DECREASED<br>LIVER FUNCTION TEST ABNORMAL<br>PLATELET COUNT DECREASED<br>PROTHROMBIN TIME PROLONGED<br>TRANSAMINASES INCREASED<br>WEIGHT DECREASED<br>WHITE BLOOD CELLS URINE POSITIVE						
**METABOLISM AND NUTRITION DISORDERS**<br>HYPOGLYCAEMIA						
**MUSCULOSKELETAL AND CONNECTIVE TISSUE DISORDERS**<br>ARTHRALGIA<br>BACK PAIN<br>NECK PAIN						
**NEOPLASMS BENIIGN, MALIGNANT AND UNSPECIFIED (INCL CYSTS AND POLYPS)**						
**NERVOUS SYSTEM DISORDERS**<br>HEADACHE<br>INSOMINIA<br>NEUROPATHY PERIPHERAL						
**PREGNANCY, PUERPERIUM AND PERINATAL CONDITIONS**						
**PSYCHIATRIC DISORDERS**						
**RENAL AND URINARY DISORDERS**<br>ALBUMINURIA<br>HAEMATURIA<br>RENAL FAILURE<br>RENAL FAILURE ACUTE						
**REPRODUCTIVE SYSTEM AND BREAST DISORDERS**						

Adverse Event<br>Body system (Preferred term)	SSG		PM		Comb	
	Related*	Not related†	Related*	Not related†	Related*	Not related†
**RESPIRATORY, THORACIC AND MEDIASTINAL DISORDERS**<br>ASTHMA<br>ALLERGIC BRONCHITIS<br>COUGH<br>EPISTAXIS						
**SKIN AND SUBCUTANEOUS TISSUE DISORDERS**<br>ACARODERMATITIS<br>ACNE<br>PRURITUS<br>RASH<br>RASH MACULO-PAPULAR<br>RASH PAPULAR<br>SKIN LESION<br>SKIN ULCER<br>SWELLING FACE						
**SURGICAL AND MEDICAL PROCEDURES**						
**VASCULAR DISORDERS**<br>HYPERTENSION						

Data are n (%) of total patients.
* Recorded as probably, possibly or unlikely to be related to study drug
† Recorded as unrelated to study drug

8.8.5 Summary of All Adverse Events during Treatment Period and Follow-up by severity

Table 27 All adverse events summarised by Severity

Adverse Event	Mild		Moderate		Severe	
**Body system (Preferred term)**	**Related***	**Not related†**	**Related***	**Not related†**	**Related***	**Not related†**
**BLOOD AND LYMPHATIC DISORDERS**						
ANAEMIA						
EOSINOPHILIA						
LEUKOCYTOSIS						
THROMBOCYTOPENIA						
**CARDIAC DISORDERS**						
BRADYCARDIA						
CARDIAC FAILURE HIGH OUTPUT						
SINUS ARRHYTHMIA						
SINUS BRADYCARDIA						
**EAR AND LABYRINTH DISORDERS**						
EAR PAIN						
**ENDOCRINE DISORDERS**						
**EYE DISORDERS**						
CONJUCTIVITIS ALLERGIC						
CONJUNCTIVITIS						
EYE PAIN						
**GASTROINTESTINAL DISORDERS**						
ABDOMINAL DISTENSION						
ABDOMINAL PAIN						
ABDOMINAL PAIN LEFT						
ABDOMINAL PAIN UPPER						
ABDOMINAL SEPSIS						
ASCITES						
DENTAL CARIES						
DUODENAL ULCER						
DYSPEPSIA						
GASTRITIS						
GINGIVAL BLEEDING						

Adverse Event	Mild		Moderate		Severe	
**Body system (Preferred term)**	**Related***	**Not related†**	**Related***	**Not related†**	**Related***	**Not related†**
GINGIVITIS<br>MOUTH ULCERATION<br>MUCOUS STOOLS<br>NAUSEA<br>ORAL SOFT TISSUE DISORDER<br>OESOPHAGEAL VARICES<br>PANCREATITIS<br>PERITONEAL HAEMORRHAGE<br>PERITONITIS<br>VOMITING						
**GENERAL DISORDERS AND ADMINISTRATION SITE CONDITIONS**<br>CHILLS<br>INJECTION SITE PAIN<br>INJECTION SITE SWELLING<br>PYREXIA<br>TENDERNESS						
**HEPATOBILIARY DISORDERS**						
**IMMUNE SYSTEM DISORDERS**<br>ALLERGY TO ARTHROPOD BITE						
**INFECTIONS AND INFESTATIONS**<br>AMOEBIC DYSENTERY<br>BODY TINEA<br>CONJUNCTIVITIS INFECTIVE<br>CROUP INFECTIOUS<br>ASCARIASIS<br>CELLULITIS<br>DYSENTERY<br>EAR INFECTION<br>FUNGAL SKIN INFECTION<br>GASTROENTERITIS<br>GIARDIASIS<br>HOOKWORM INFECTION<br>INJECTION SITE CELLULITIS						

Adverse Event<br>Body system (Preferred term)	Mild		Moderate		Severe	
	Related*	Not related†	Related*	Not related†	Related*	Not related†
LARYNGITIS<br>LYMPHADENITIS BACTERIAL<br>MALARIA<br>MOLLUSCUM CONTAGIOSUM<br>NASOPHARYNGITIS<br>OTITIS MEDIA<br>OTITIS MEDIA ACUTE<br>PARASITIC INFECTION INTESTINAL<br>PERICARDITIS MYCOPLASMAL<br>PNEUMONIA<br>PNEUMONIA PRIMARY ATYPICAL<br>STRONGYLOIDIASIS<br>TINEA CAPITIS<br>TUBERCULOSIS<br>UPPER RESPIRATORY TRACT INFECTION<br>URINARY TRACT INFECTION<br>VARICELLA<br>VISCERAL LEISHMANIASIS<br>WOUND SEPSIS						
**INJURY, POISONING AND PROCEDURAL COMPLICATIONS**<br>CONTUSION<br>SCRATCH<br>SKIN LACERATION<br>WOUND						
**INVESTIGATIONS**<br>ALANINE AMINOTRANSFERASE INCREASED<br>ASPARTATE AMINOTRANSFERASE INCREASED<br>BLOOD AMYLASE INCREASED<br>BLOOD ALKALINE PHOSPHATASE INCREASED<br>BLOOD CREATININE INCREASED<br>ELECTROCARDIOGRAM CHANGE<br>ELECTROCARDIOGRAM QT PROLONGED<br>HEPATIC ENZYMES INCREASED						

**Adverse Event**<br>**Body system (Preferred term)**	**Mild**		**Moderate**		**Severe**	
	**Related***	**Not related†**	**Related***	**Not related†**	**Related***	**Not related†**
HAEMAGLOBIN DECREASED<br>LIVER FUNCTION TEST ABNORMAL<br>PLATELET COUNT DECREASED<br>PROTHROMBIN TIME PROLONGED<br>TRANSAMINASES INCREASED<br>WEIGHT DECREASED<br>WHITE BLOOD CELLS URINE POSITIVE						
**METABOLISM AND NUTRITION DISORDERS**<br>HYPOGLYCAEMIA						
**MUSCULOSKELETAL AND CONNECTIVE TISSUE DISORDERS**<br>ARTHRALGIA<br>BACK PAIN<br>NECK PAIN						
**NEOPLASMS BENIIGN, MALIGNANT AND UNSPECIFIED (INCL CYSTS AND POLYPS)**						
**NERVOUS SYSTEM DISORDERS**<br>HEADACHE<br>INSOMINIA<br>NEUROPATHY PERIPHERAL						
**PREGNANCY, PUERPERIUM AND PERINATAL CONDITIONS**						
**PSYCHIATRIC DISORDERS**						
**RENAL AND URINARY DISORDERS**<br>ALBUMINURIA<br>HAEMATURIA<br>RENAL FAILURE<br>RENAL FAILURE ACUTE						
**REPRODUCTIVE SYSTEM AND BREAST DISORDERS**						
**RESPIRATORY, THORACIC AND MEDIASTINAL DISORDERS**<br>ASTHMA						

Adverse Event<br>Body system (Preferred term)	Mild		Moderate		Severe	
	Related*	Not related†	Related*	Not related†	Related*	Not related†
ALLERGIC BRONCHITIS<br>COUGH<br>EPISTAXIS						
**SKIN AND SUBCUTANEOUS TISSUE DISORDERS**<br>ACARODERMATITIS<br>ACNE<br>PRURITUS<br>RASH<br>RASH MACULO-PAPULAR<br>RASH PAPULAR<br>SKIN LESION<br>SKIN ULCER<br>SWELLING FACE						
**SURGICAL AND MEDICAL PROCEDURES**						
**VASCULAR DISORDERS**<br>HYPERTENSION						

Data are n (%) of total patients.
* Recorded as probably, possibly or unlikely to be related to study drug
† Recorded as unrelated to study drug

9. Subgroup Analyses

9.1 Primary Efficacy Analysis: HIV negative patients

Few HIV positive patients are expected in the trial population. However, if the overall prevalence of HIV in randomised patients is 10% or greater, the primary efficacy analysis will be repeated in HIV negative patients.

Table 28 Definitive Cure Analysis 1: Complete Case Analysis

Estimation	SSG<br>N =	PM<br>N =	Comb<br>N =
Treatment Efficacy at 6 months, n (%)			
Test of difference across arms: p-value*			
Difference between SSG & PM (95% CI)			
Difference between SSG & Combination (95% CI)			
Test of difference across centres, after adjustment for treatment : p-value*			

* p-value from likelihood ratio test, comparing models with and without variable being tested.

Table 29 Definitive Cure Analysis 2: Last parasitology carried forward

Estimation	SSG<br>N =	PM<br>N =	Comb<br>N =
Treatment efficacy at 6 months, n (%)			
Test of difference across arms: p-value*			
Difference between SSG & PM (95% CI)			
Difference between SSG & Combination (95% CI)			
Test of difference across centres, after adjustment for treatment: p-value*			

* p-value from likelihood ratio test, comparing models with and without variable being tested.

Table 30 Definitive Cure Analysis 3 – Worst-case analysis

Estimation	SSG<br>N =	PM<br>N =	Comb<br>N =
Treatment efficacy at 6 months, n (%)			
Test of difference across arms: p-value*			
Difference between SSG & PM (95% CI)			
Difference between SSG & Combination (95% CI)			
Test of difference across centres, after adjustment for treatment: p-value*			

* p-value from likelihood ratio test, comparing models with and without variable being tested.

If the LRT p-value following comparison of models with and without centre ≤ 0.05, by-centre efficacy analyses will be presented.

9.2 Efficacy Analysis: HIV positive patients

For patients testing HIV positive a summary of the response to treatment at 6 Months follow-up will be completed, depending on the number of patients and their distribution between the groups. If insufficient numbers or uneven distribution then the treatment allocation, parasitology results at end of treatment and follow up will be presented here grouped by treatment, along with indication of receipt of rescue medication and timing of receipt.

Table 31 Definitive Cure Summary for HIV positive patients

**Treatment efficacy at 6 months, n (%)**	**SSG N =**	**PM N =**	**Comb N =**
Definitive Cure 1: Complete Case Analysis			
Definitive Cure Analysis 2: Last parasitology carried forward			
Definitive Cure Analysis 3: Worst-case analysis			

Parasitology Data for HIV positive patients by Treatment

**Patient Number**	**Treatment arm**	**Parasitology result**			**Rescue administered: Yes/No**
		**End of Treatment**	**3 month follow-up**	**6 month follow-up**	
	SSG	Positive	Not Done	Negative	Yes

9.3 Efficacy Analysis: Age

Summary of

**Treatment efficacy at 6 months, n (%)**	**SSG N =**	**PM N =**	**Comb N =**
**Adults**			
Definitive Cure 1: Complete Case Analysis			
Definitive Cure Analysis 2: Last parasitology carried forward			
Definitive Cure Analysis 3: Worst-case analysis			
**Children**			
Definitive Cure 1: Complete Case Analysis			
Definitive Cure Analysis 2: Last parasitology carried forward			
Definitive Cure Analysis 3: Worst-case analysis			

10. Statistical Methods

10.1 Data Summary and Comparison

Age will be summarised as a continuous variable and with the categories paediatric (4 to 14 years) and adult (15 and above).

To classify nutritional status, weight-for-age values will be used in children and BMI in adults.

For each child, the weight-for-age is calculated as observed weight ÷ expected median weight-for-age x 100, where the expected median weight-for-age is calculated as (2 x age in years) + 8

Children are classified as normal if weight-for-age is > 80%; underweight if 60% ≤ weight-for-age ≤ 80% and severely underweight if weight-for-age < 60%.

For each adult, body mass index (BMI) is calculated as weight in kg ÷ height in metres squared. Adults are classified as normal if 18.5 ≤ BMI ≤ 24.9; underweight if 16.0 ≤ BMI ≤ 18.4 and severely underweight if BMI < 16.

Continuous data are to be summarised using mean and standard deviation (SD) if normally distributed and tested using t-tests or ANOVA where appropriate or using median and inter-quartile range (IQR) and non-parametric testing if not normally distributed. Binary and categorical data will be summarised using proportions and compared using chi-squared tests.

10.2 Parasitological Efficacy

The unadjusted treatment difference will be calculated for all sites, comparing each treatment to SSG using a binomial regression model with an identity link to provide difference estimates for equivalence with two-sided 95% confidence intervals.

Centre will then be added to the model as a covariate and the models with and without centre compared using the likelihood ratio test (LRT). If the LRT p-value following comparison of models with and without centre, after adjustment for treatment, is ≤0.05 treatment effectiveness by centre will be tabulated (Federov 2005).

If deemed necessary, adjustments will be made for multiple comparisons using Bonferroni adjustment where the significance level would be divided by the number of comparisons, c. Evidence of a difference between two groups will be identified if p-value estimated ≤ specified cut-off (usually 0.05)/c.

10.3 Biological Markers, ECG and Audiometry

For repeated measurements made during treatment, change during treatment will be modelled including random intercept terms to allow for correlation within patient's measurements, assuming an appropriate distribution for each marker. Models with and without random slope terms will be compared using the likelihood ratio test to test for variation in changes within treatment arms. For pairwise comparisons, SSG will be the reference treatment.

Where there is evidence of a difference during treatment and it is of interest to know between which time point changes occurred, a mean difference in parameter values between consecutive time points will be summarised for each parameter and tests carried out across arms using ANCOVA, adjusting for values at the earlier time point. Evidence of variability in mean differences by centre will also be investigated this way.

10.4 Serious and Non-serious Adverse Events

In addition to data listings provided in the full trial report, SAEs and AEs are tabulated by treatment arm according to relation to study drug and severity of event.

Relation to study drug is classified as not related where original recording indicates unrelated and classified as related where recording indicates a possible or probable or unlikely relation.

For overall analysis of AEs *during treatment*, the AE rate will be calculated as the number of events divided by the person-days on treatment, for each arm and comparisons made across arms. Rate ratios and corresponding 95% CIs will be estimated for PM versus SSG and Combination treatment versus SSG. For AEs occurring at 3 months and 6 months follow-up, the absolute risk of an AE or SAE will be calculated for each treatment arm as the number of patients experiencing at least one AE divided by the number of patients randomised, per arm. Relative risks with corresponding 95% CIs will be estimated for PM versus SSG and Combination treatment versus SSG. The absolute risk difference with corresponding 95% confidence intervals will also be provided for these two comparisons. These measures will be repeated within categories of relation to study drug and severity of event.

When considering the occurrence of specific AEs, according to the MEDRA coding system, multiple AEs per patient will be considered as separate individual AEs. Evidence of correlation between individual patients will be assessed and if found, account will be taken of multiple AEs per patient in comparisons.

11. Power: Efficacy Analysis

If treatment efficacy in the reference arm, SSG, is expected to be 95%, 135 patients per arm would provide around 80% power to show evidence of a difference in efficacy at the 5% level, if the efficacy of PM or Combination treatment is 85% or lower, based on a two-sided test.

If the efficacy of SSG is lower at say 85%, 135 patients per arm would provide 80% power to show evidence of a difference at the 5% level, if the efficacy of PM or Combination treatment is 70% or lower, based on a two-sided test.

With 135 patients per arm, it will be possible to show, with around 80% power, that the efficacy of PM or Combination treatment is no more than 7% lower than the efficacy of SSG, if the efficacy of SSG is assumed to be 95%.

Note that these estimations assume there are no missing data for definitive cure.

Number needed *per regimen* for a two-sample, two-sided test between two proportions (Kirkwood & Sterne):

$$\frac{\left\{u\sqrt{\pi_1(1-\pi_1)+\pi_0(1-\pi_0)}+v\sqrt{2\bar{\pi}(1-\bar{\pi})}\right\}^2}{(\pi_0-\pi_1)^2}$$

$$\bar{\pi}=\frac{\pi_1+\pi_0}{2}$$

u = one-sided percentage point of the normal distribution corresponding to 100% - power, so, for power of 80%, u = 0.84 and for power of 90%, u = 1.28
v = percentage point of the normal distribution corresponding to the significance, so, for significance of 5% and a two-sided percentage point v = 1.96.

12. References

Valerii Fedorov and Byron Jones. The design of multicentre trials. Statistical Methods in Medical Research 2005; 14: 205-248

John P.A. Ioannidis, MD; Stephen J.W. Evans, MSc; Peter C. Gøtzsche, MD, DrMedSci; Robert T. O'Neill, PhD; Douglas G. Altman, DSc; Kenneth Schulz, PhD; and David Moher, PhD, for the CONSORT Group. Better Reporting of Harms in Randomized Trials: An Extension of the CONSORT Statement. Ann Intern Med. 2004; 141:781-788.

Kirkwood & Sterne. Essential Medical Statistics.

Report CIOMS Working Group IV. Management of Safety Information from Clinical trials

Appendix 17 Statistical Analysis Report

LEAP 0104A

A MULTICENTRE INDIVIDUALLY RANDOMISED TRIAL OF EFFICACY AND SAFETY OF SODIUM STIBO-GLUCONATE (SSG) VERSUS PAROMOMYCIN (PM) AND VERSUS A COMBINATION OF SSG AND PM FOR THE TREATMENT OF VISCERAL LEISHMANIASIS IN ETHIOPIA, KENYA AND SUDAN

STATISTICAL ANALYSIS REPORT

Table of Contents

Abbreviations **5**

1. TRIAL OBJECTIVES **6**

2. STUDY DESIGN **6**

2.1 Study Sites **6**

2.2 Inclusion Criteria **6**

2.3 Exclusion Criteria **6**

2.4 HIV-status and VCT **7**

2.5 Dose Schedule **7**

3. RANDOMISATION **7**

4. PRIMARY ENDPOINT **7**

5. SECONDARY ENDPOINTS **7**

5.1 Efficacy **7**
5.1.1 End of Treatment Parasitology 7
5.1.2 Parasitology at 3 months follow-up 8

5.2 Safety **8**
5.2.1 Serious adverse events (SAE) and non-serious Adverse events (AE) 8
5.2.2 ECG and Audiometry 8
5.2.3 Urinalysis: Blood and Protein 8

5.3 Biological Parameters **8**

6. PATIENT ASSESSMENT SCHEDULE **9**

7. ANALYSIS DEFINITIONS **10**

7.1 Primary Efficacy Analysis **10**

7.2 Secondary Efficacy Analysis **11**
7.2.1 Test of Cure 11
7.2.2 Slow Response to Treatment 11

7.3 Safety Analysis **11**
7.3.1 ECG and Audiometry 11
7.3.2 Urinalysis: Blood and Protein 12
7.3.3 Serious and Non-Serious Adverse Events 12

7.4 Biological Marker Data **12**

8. STATISTICAL METHODS **12**

8.1 Baseline Data **12**

8.2 Timing of Follow-up Data Collection **12**

8.3 Parasitological Efficacy **13**

8.4 Biological Markers, ECG, Audiometry and Urinalysis **13**

8.5 Serious and Non-serious Adverse Events **14**

9. POWER AND SAMPLE SIZE **15**

10. RESULTS **16**

10.1 Participant Flow **16**

10.2 Baseline Characteristics **17**

10.3 Deviations from Protocol: **21**
10.3.1 Exclusion criteria at baseline 21
10.3.2 Non-Compliance to Treatment Regimen 21
10.3.3 Timing of Final Assessment 21

10.4 Primary Efficacy Analysis: Overall **22**
10.4.1 Data handling of 6 months data 22
10.4.2 Primary Efficacy Analysis 23
10.4.3 Primary Efficacy Analysis: By centre 25

10.5 Secondary Efficacy Parasitology Analysis **27**
10.5.1 Data Handling at Test of Cure 27
10.5.2 Secondary Efficacy Parasitology Analysis: All sites 27
10.5.3 Secondary Efficacy Parasitology Analysis: By Centre 28
10.5.4 Slow response to Treatment 29
10.5.5 Rescue Medication Outcomes 30
10.5.6 Follow-up at 3 months 30

10.6 Safety ECG and Audiometry Analysis **31**
10.6.1 Safety Analysis: ECG 31
10.6.2 Safety Analysis: Audiometry 32
10.6.3 Urinalysis: Protein and Blood 32

10.7 Secondary Efficacy Analysis: Biological Parameters during Treatment **33**

10.8 Serious Adverse Events (SAEs) and Non-Serious Adverse Events (AEs) **42**
10.8.1 Treatment Emergent AEs: Summary Results 44
10.8.2 Serious Adverse Events: Listing 45
10.8.3 Non-Serious Treatment Emergent Adverse Events: Listing 46
10.8.4 All Non-Serious Adverse Events: Listing 50

10.9 Subgroup Analyses **54**
10.9.1 HIV positive patients 54
10.9.2 Post Kala-azar Dermal Leishmaniasis 56

References **56**

Tables

Table 1 Patient Assessment Schedule 9
Table 2 Imputed values for missing six month parasitological data 10
Table 3 Baseline Demographic characteristics 17
Table 4 Baseline Biological Markers 18
Table 5 Baseline Laboratory parameters 19
Table 6 Baseline clinical characteristics 20
Table 7 Baseline Symptoms 20
Table 8 Minor Protocol Violations at Baseline 21
Table 9 Timing of final assessment 22
Table 10 Definitive Cure Analysis 1: Complete Case Analysis 23
Table 11 Definitive Cure Analysis 2: Last parasitology carried forward 24
Table 12 Definitive Cure Analysis 3: Worst-case analysis 24
Table 13 DC: Complete Case Analysis: By Centre 25
Table 14 DC: LPCF Analysis: By Centre 26
Table 15 DC: Worst Case Analysis: By Centre 26
Table 16 Test of Cure Analysis 1: Complete Case Analysis 27
Table 17 Test of Cure Analysis 2: Worst Case Analysis 28
Table 18 TOC: Complete Case Analysis By-Centre 28
Table 19 TOC: LPCF Analysis By-Centre 29
Table 20 Slow Response by treatment arm 29
Table 21 Rescue Medication and Study Outcome by Treatment 30
Table 22 Overall Results 3 months Post End of Treatment 31
Table 23 Number of ECG Tests performed per centre 31
Table 24 ECG Analysis during treatment and follow-up 31
Table 25 Number of Audiometry examinations performed per centre 32
Table 26 Urinalysis: Protein 33
Table 27 Urinalysis: Blood 33
Table 28 Difference in biological parameters between baseline and Day 7 35
Table 29 Difference in biological parameters between Day 7 and Day 14 36
Table 30 Difference in biological parameters between Baseline and Day 14 37
Table 31 Difference in biological parameters between Day 14 and End of treatment. 38
Table 32 Difference in biological parameters between Baseline and End of treatment39
Table 33 Summary: Comparison of mean changes in biological parameters 40
Table 34 Summary: Change from Baseline to End of Treatment 41
Table 35 Number of patients experiencing adverse events 42
Table 36 Number of adverse events 42
Table 37 Treatment Emergent Adverse Event Rate Ratios 44
Table 38 Serious Adverse Events, by treatment and relation to study drug 45
Table 39 Deaths during Treatment and Follow-up 45
Table 40 Treatment Emergent Non- Serious AEs 46
Table 41 All Non- Serious AEs, by treatment and relation to study drug 50
Table 42 Number of adults and children tested for HIV at each centre 55
Table 43 Parasitology Data for HIV positive patients by Treatment 55

Figures

Figure 1 Data Collection during Follow-up: Data Handling 13
Figure 2 LEAP 0104A Screening and Enrolment: all sites 16
Figure 3 LEAP 0104A Treatment and Follow-up: all sites 16

Abbreviations

AE	Adverse event
ALT	Alanine aminotransferase (SGPT)
AP	Alkaline Phosphatase
AST	Aspartate aminotransferase (SGOT)
CBC	Complete blood count
CRF	Case report form
DNDi	Drugs for neglected diseases initiative
ECG	Electrocardiogram
FDA	Food and Drug Administration
GCP	Good clinical practice
ICH	International Conferences on Harmonization
IEC	Independent ethics committee
IV	Intravenous
PI	Principal investigator (see note Section 13)
SAE	Serious adverse event
ULN	Upper limit of normal
WBC	White blood cell
WHO	World Health Organization
WNL	Within normal limits
SSG	Sodium Stibogluconate
PM	Paromomycin
Comb	SSG and PM Combination treatment
CI	Confidence Interval
VCT	Voluntary counselling and testing
TOC	Test of Cure
SD	Standard Deviation
IQR	Interquartile range
DC	Definitive Cure
NR	Not related to study drug
ADR	Adverse drug reaction
KEMRI	Kenya Medical Research Institute
LTFU	Loss to follow up
LPCF	Last parasitology carried forward
RR	Rate ratio
TB	Tuberculosis

1. Trial Objectives

To compare the efficacy and safety of:

- Sodium Stibogluconate (SSG) monotherapy
- Paromomycin (PM) monotherapy
- SSG and PM in a combination therapy (Comb)

2. Study Design

Multi-centre individually randomised controlled trial.

2.1 Study Sites

- Kenya:
  - KEMRI, Nairobi (Co-ordinating Centre)
- Sudan:
  - Um El Kher (UeK)
  - Kassab
- Ethiopia:
  - Gondar
  - Arba Minch

2.2 Inclusion Criteria

- Patients for whom written informed consent has been signed by the patients themselves (if aged 18 years and over) or by parent(s) or legal guardian for patients under 18 years of age.
- Patients aged between 4 and 60 years (inclusive).
- Patients with clinical signs and symptoms of VL and diagnosis confirmed by visualization of parasites in tissue samples (spleen, lymph node or bone marrow) on microscopy.

2.3 Exclusion Criteria

- Patients who have received any anti-leishmanial drug in the last 6 months.
- Patients with a clinical contraindication to splenic/lymph node/ bone marrow aspirates.
- Patients with severe protein and or caloric malnutrition (Kwashiokor or marasmus)
- Patients with previous hypersensitivity reaction to SSG or aminoglycosides.
- Patients suffering from a concomitant severe infection such as TB or any other serious underlying disease (cardiac, renal, hepatic) which would preclude evaluation of the patient’s response to study medication.

- Patients suffering from other conditions associated with splenomegaly such as schistosomiasis.
- Patients with previous history of cardiac arrhythmia or an abnormal ECG
- Patients who are pregnant or lactating.
- Patients with haemoglobin < 5gm/dl.
- Patients with WBC < 1 x 10^3/mm
- Patients with platelets < 40,000/mm
- Patients with liver function tests more than three times the normal range
- Patients with serum creatinine outside the normal range for age and gender.
- Patients with pre-existing clinical hearing loss

2.4 HIV-status and VCT

All patients were offered counselling and screening for HIV under a voluntary counselling and testing programme (VCT). This was either to be done at the same time as consent was obtained for inclusion in the trial or at a later date, according to hospital practice. A HIV positive result was **not** an exclusion criterion.

2.5 Dose Schedule

Treatment was administered by IM or IV at the same time each day according to the following schedule for each regimen;

- SSG monotherapy: 20mg/kg/day for 30 days, with a maximum daily dose of 850mg (8.5ml) per patient except in Sudan where there was no maximum dose
- PM monotherapy: 15 mg/kg/day for 21 days.
- Combination SSG 20mg/kg/day and PM 15 mg/kg/day for 17 days

3. Randomisation

Restricted block randomization was performed for the three arms per site within each country. Block sizes of 15 were used. Opaque envelopes were numbered sequentially and then sealed. The process was carried out at the DNDi Trial Co-ordination Centre, Nairobi, where a copy of the randomisation schedule is kept securely.

4. Primary Endpoint

Parasitology at 6 months follow up: measured by visualization of parasites in tissue samples (spleen, lymph node or bone marrow) on microscopy.

5. Secondary Endpoints

5.1 Efficacy

5.1.1 End of Treatment Parasitology

Parasitology at end of treatment: measured by visualization of parasites in tissue samples (spleen, lymph node or bone marrow) on microscopy.

The tissue sample was taken on the day following the last day of treatment:

- SSG: Day 31
- PM: Day 22
- Combination: Day 18

5.1.2 Parasitology at 3 months follow-up

Follow up at 3 months was optional, dependent on investigator concerns following discharge post treatment and seasonal access to remote communities.

Patients who attended follow up were examined for clinical symptoms of VL and classified as clinically well or clinically unwell. Parasitology, measured by visualization of parasites in tissue samples (spleen, lymph node or bone marrow) on microscopy, was performed in patients who were deemed to be clinically unwell.

5.2 Safety

5.2.1 Serious adverse events (SAE) and non-serious Adverse events (AE)

Data were collected through spontaneous recording and active examination and questioning. AEs were coded according to Medical Dictionary for Regulatory Activities, version 10.0.

5.2.2 ECG and Audiometry

Recorded at specified assessment times and categorised as Normal, Clinically Insignificant Abnormality or Clinically Significant Abnormality.

5.2.3 Urinalysis: Blood and Protein

Recorded at each assessment during treatment and at follow up. Data values were categorised as Negative (indicating a Normal result) or Positive (indicating an Abnormality).

5.3 Biological Parameters

The following parameters were measured at specified assessment times, according to schedule specified in the next section.

- Temperature: ($^{\circ}$Celsius)
- Heart Rate: (beats per minute)
- Spleen size: (centimetres, cm by palpation below left costal margin in the line of growth)
- Liver size: (cm by palpation below right costal margin in the mid-clavicular line)
- Weight: (kg)
- Haemoglobin: (g/dl)
- White Cell Count: (x 10^3/µl)
- Platelet Count: (x 10^3/µl)

- Systolic Blood Pressure: (mm Hg)
- Diastolic Blood Pressure: (mm Hg)
- Total Bilirubin: (umol/L)
- BUN: (mmol/L)
- Creatinine: (umol/L)
- AST: (U/L)
- ALT: (U/L)
- Amylase: (micromol/L)
- Alkaline Phosphatase (U/L)

6. Patient Assessment Schedule

There was a pre-specified patient assessment schedule for efficacy, ECG and Audiometry and biological markers parameters by day of treatment and follow-up at three and six months (Table 1).

Table 1 Patient Assessment Schedule

Assessments	Day of treatment					Follow-up	
	0	7	14	21*	End of Treatment †	3 Months	6 Months
Efficacy: Parasitology ‡	✓				✓	✓	✓
ECG and Audiometry ‖	✓		✓		✓	✓	✓
Biological Markers §	✓	✓	✓	✓	✓	✓	✓

* SSG only

† End of treatment is Day 31 for SSG, Day 22 for Paromomycin and day 18 for Combination, assuming Day 1 is first day of treatment for all regimens

‡ Measured at 3 months if patient clinically unwell

‖ ECG and audiometry also carried out on Day 7 and Day 21 in Kenya and Kassab, Audiometry not done in Um el Kher

§ Temperature, Spleen size, Liver Size, Weight, Haemoglobin, Platelets, White cell count, Heart Rate, Systolic and Diastolic blood pressure, Bilirubin, BUN, Creatinine, ALT, AST, Amylase (Arba Minch only), Alkaline Phosphatase (excluding Kassab)

7. Analysis Definitions

7.1 Primary Efficacy Analysis

Treatment success, or Definitive Cure (DC), was defined as

- Absence of parasites on microscopy slide at 6 months, provided no rescue medication was given during treatment or follow up period.

Treatment failure was defined as

- receipt of rescue medication at any point in the trial
- parasites visualized on a microscopy slide at 6 months in patients who have *not* received rescue medication during treatment or follow up period

The efficacy in each arm is the proportion, or percentage, of treatment successes. The treatment effect is the difference in efficacy between SSG and PM arms and SSG and Combination arms at 6 months follow-up.

Where 6 months parasitology data were missing, efficacy analyses were carried out, within an Intention-to Treat framework, in three ways;

- Complete case analysis (full analysis set): excluding patients with missing efficacy data from estimation of efficacy in each arm
- Last parasitology carried forward: Patients with missing efficacy data will have their last parasitology results carried forward (Table 2).
- Worst case analysis: Patients with missing efficacy data will be allocated efficacy results based on their 'worst-case' scenario i.e. treatment failure.

Table 2 Imputed values for missing six month parasitological data

**Scenario***		**Assumed parasitology result at 6 months**	**Patient Treatment Outcome**
1	Parasitological measurements taken at 3 months: parasites visualised	Positive	Failure
2	Parasitological measurements taken at 3 months: parasites not visualised	Negative	Success
3	Patient attended for 3 month visit: Parasitological measurements not taken but parasites visualised at end of treatment	Positive	Failure
4	Patient attended for 3 month visit: Parasitological measurements not taken but parasites not visualised at end of treatment	Negative	Success
5	Last seen at end of treatment: parasites visualised	Positive	Failure
6	Last seen at end of treatment: parasites not visualised	Negative	Success

* receipt of rescue medication prior to 6 months follow-up visit is considered a treatment failure

If a patient withdrew full consent at any time in the trial period, data collection for the purposes of the trial ceased and any primary or secondary outcome data beyond this point were treated as missing.

Patients with confirmed VL whose baseline characteristics met exclusion criteria (minor protocol violations) were included in all efficacy analyses.

7.2 Secondary Efficacy Analysis

7.2.1 Test of Cure

Treatment success, or Test of Cure (TOC), was defined as:

- absence of parasites on microscopy slide at end of treatment

Treatment failure was defined as

- receipt of rescue medication during treatment period or initial hospitalisation period

The treatment effect is the difference in efficacy between SSG and PM arms and SSG and Combination arms, at the end of treatment.

Missing parasitology data at the end of treatment arose if

- a patient died prior to the end of the treatment period
- a patient was withdrawn from study treatment
- a parasitology exam should have been done but was not (investigator error)

To take account of missing data, efficacy analyses were carried out, also within an Intention-to Treat framework, in two ways;

- complete case analysis: excluding patients with missing data from estimation of efficacy in each arm
- worst case analysis: patients with missing efficacy data will be allocated efficacy results based on their 'worst-case' scenario i.e. treatment failure.

Analyses assume that end of treatment time points are comparable, by definition of the treatment regimens.

7.2.2 Slow Response to Treatment

Slow response to treatment was described as the number and percentage of patients, randomised per arm, in whom parasites were visualised at the end of treatment but not visualised at 6 months post end of treatment, with no administration of rescue medication.

7.3 Safety Analysis

7.3.1 ECG and Audiometry

Recorded categories of 'normal' and 'clinically insignificant abnormality' were combined in the analysis to one category of normal. A clinically significant abnormality indicated an abnormal result.

7.3.2 Urinalysis: Blood and Protein

Recorded categories were positive and negative. A positive reading was assumed to indicate an abnormal result and a negative reading a normal result.

7.3.3 Serious and Non-Serious Adverse Events

Treatment emergent adverse events were defined as adverse events with onset between day 1 of treatment and 30 days post end of treatment, inclusive.
Serious adverse events were summarised and compared using proportions per arm.
Non-serious adverse events were summarised and compared using rates, to take account of different time at risk due to different length of treatment periods.

7.4 Biological Marker Data

Each biological marker was treated as a separate outcome. Data for each treatment assessment day were summarised and compared between subsequent time points.

8. Statistical Methods

8.1 Baseline Data

At baseline, age was summarised as a continuous variable and within the categories; paediatric (4 to 14 years) and adult (15 and above).
To classify nutritional status, weight-for-age values were used in children and BMI in adults. For each child, the weight-for-age was calculated as (observed weight ÷ expected median weight-for-age) x 100, where the expected median weight-for-age is calculated as (2 x age in years) + 8. Children were classified as normal if weight-for-age was > 80%; underweight if 60% ≤ weight-for-age ≤ 80% and severely underweight if weight-for-age < 60%. For each adult, body mass index (BMI) was calculated as weight in kg ÷ height in metres squared. Adults were classified as normal if 18.5 ≤ BMI ≤ 24.9; underweight if 16.0 ≤ BMI ≤ 18.4 and severely underweight if BMI < 16.
To present and compare baseline data, continuous data were summarised using mean and standard deviation (SD) if normally distributed and tested using t-tests or ANOVA where appropriate or using median and inter-quartile range (IQR) and non-parametric testing if not normally distributed. Binary and categorical data were summarised using proportions and compared using chi-squared or Fishers test.

8.2 Timing of Follow-up Data Collection

Every effort was made to follow-up patients as close as possible to 3 months or 6 months post end of treatment. Where

- the date of a patient follow-up falls between 1.5 and 4.5 months post end of treatment assessment (expected 3 months follow-up date ±45 days), data were treated as 3 months follow-up data (Figure 1).

- the date of a patient follow-up falls anywhere beyond 4.5 months post end of treatment assessment, data were treated as 6 months follow-up data (TOC date plus 135 days or more).

Figure 1 Data Collection during Follow-up: Data Handling

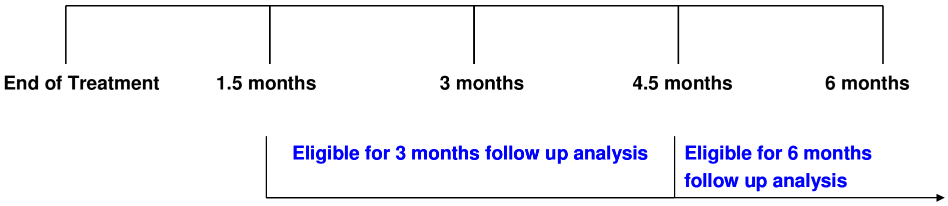

8.3 Parasitological Efficacy

The unadjusted treatment difference was calculated for all sites, comparing each of the test regimens (PM and Combination) to SSG, using a binomial regression model with an identity link to provide difference estimates for equivalence with two-sided 95% confidence intervals.

Centre was added to the model as a covariate and the models with and without centre compared using the likelihood ratio test (LRT). If the LRT p-value following comparison of models with and without centre, after adjustment for treatment was ≤0.05, treatment effectiveness by centre was tabulated (Federov 2005).

8.4 Biological Markers, ECG, Audiometry and Urinalysis

For biological measurements measured repeatedly during treatment and follow-up, the mean difference in parameter values between consecutive time points was calculated, within each randomisation arm, for each parameter. Tests for differences in the mean difference were carried out across arms using ANCOVA, adjusting for values at the earlier time point and centre.

ECG data were analysed as binary data, where categories were normal (normal or clinically insignificant abnormality) and abnormal (clinically significant abnormality).

Data were summarised using the following proportions, where the denominator in all cases in the number of patients randomised to treatment with normal result at baseline.

- proportion of patients who had an abnormal reading *on* day 7
- proportion of patients who had an abnormal reading *on* day 14
- proportion of patients who had had an abnormal reading *by* day 14 (abnormal reading on day 7, 14 or both)
- proportion of patients who had had an abnormal reading *by* end of treatment (abnormal reading on day 7, 14, end of treatment or all)
- proportion of patients who had an abnormal reading at end of treatment

- proportion of patients who had an abnormal reading at end of treatment that had not returned to normal at 6 months

Chi-squared tests were used to compare proportions across arms.

Patients were not expected to have negative (normal) values for the urinalysis parameters, protein and blood, at baseline. Patients who had urinalysis results at baseline were categorised based on recorded changes between baseline and end of treatment, into one of the following distinct groups,

- Baseline negative, end of treatment negative
- Baseline negative, end of treatment positive
- Baseline positive, end of treatment positive
- Baseline positive, end of treatment negative
- Baseline negative, end of treatment missing
- Baseline positive, end of treatment missing

Comparisons were made across all treatment arms and between SSG and PM separately. Note that this analysis assumes that a negative reading indicates a normal result and positive reading indicates an abnormal result.

8.5 Serious and Non-serious Adverse Events

SAEs and AEs, classified according to Medra, version 10, were tabulated by treatment arm according to their corresponding System Organ Class and preferred terms.

Relation to study drug was classified as not related where original recording indicated unrelated and classified as related where a recording indicated a possible or probable or unlikely relation.

Treatment emergent adverse events were defined as those beginning at any time between the first day of treatment and 30 days after the expected end of treatment, as specified in the protocol.

For the analysis of treatment emergent AEs, the AE rate was calculated as the number of events divided by the person-days at risk, for each arm and comparisons made across arms. The person-time at risk for a single patient in each arm was defined as follows,

- SSG: 30 days treatment + 30 days = 60 days,
- PM: : 21 days treatment + 30 days = 51 days,
- Combination: 17 days treatment + 30 days = 47 days.

The total person-time at risk per arm is the product of the total number of patients and the person-time at risk corresponding to that arm. The treatment emergent AE rate is the total number of AE per arm divided by the total person-time at risk per arm.

Rate ratios and corresponding 95% CIs were estimated for PM versus SSG and Combination treatment versus SSG, using poisson regression, which also accounts for multiple AEs within patients. After adjustment for treatment, models with and without centre were compared using the LRT to assess evidence of heterogeneity between study

centres and evidence for a treatment-centre interaction. Where evidence of heterogeneity or interaction was found, by-centre effects were reported.

9. Power and Sample Size

If treatment efficacy in the reference arm, SSG, was expected to be 95%, 135 patients per arm would provide around 80% power to show evidence of a difference in efficacy at a 5% significance level, if the efficacy of PM or Combination treatment was 85% or lower, based on a two-sided test.

If the efficacy of SSG was lower at say 85%, 135 patients per arm would provide 80% power to show evidence of a difference at a 5% significance level, if the efficacy of PM or Combination treatment was 70% or lower, based on a two-sided test.

With 135 patients per arm, it would be possible to show, with around 80% power, that the efficacy of PM or Combination treatment was no more than 7% lower than the efficacy of SSG, if the efficacy of SSG was assumed to be 95%.

Note that these estimations assume no missing data for definitive cure.

Number needed *per regimen* for a two-sample, two-sided test between two proportions (Kirkwood & Sterne, 2003):

$$\frac{\left\{u\sqrt{\pi_1(1-\pi_1)+\pi_0(1-\pi_0)}+v\sqrt{2\overline{\pi}(1-\overline{\pi})}\right\}^2}{(\pi_0-\pi_1)^2}$$

$$\overline{\pi}=\frac{\pi_1+\pi_0}{2}$$

u = one-sided percentage point of the normal distribution corresponding to 100% - power, so, for power of 80%, *u* = 0.84 and for power of 90%, *u* = 1.28

v = percentage point of the normal distribution corresponding to the significance, so, for significance of 5% and a two-sided percentage point *v* = 1.96.

10. Results

10.1 Participant Flow

Figure 2 LEAP 0104A Screening and Enrolment: all sites

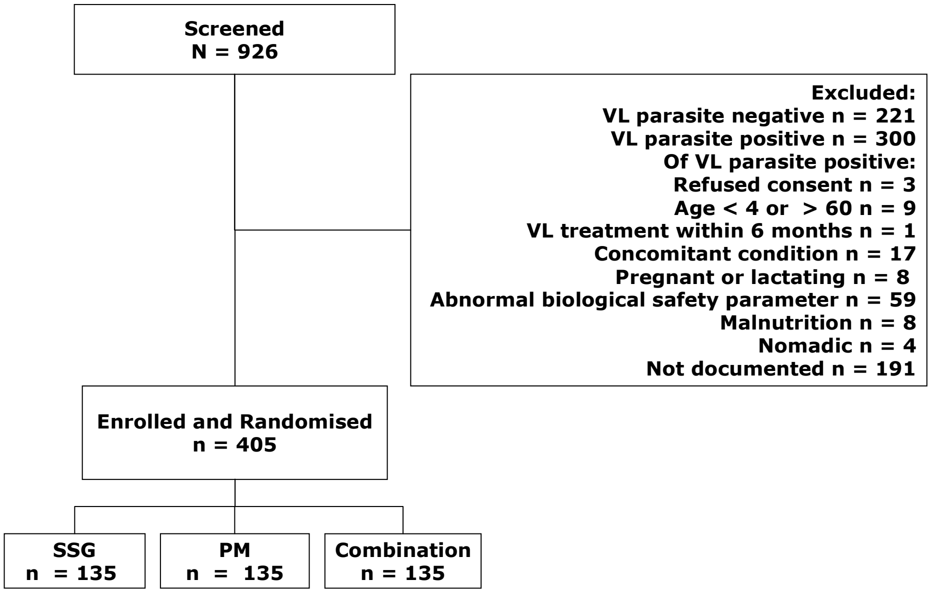

Figure 3 LEAP 0104A Treatment and Follow-up: all sites

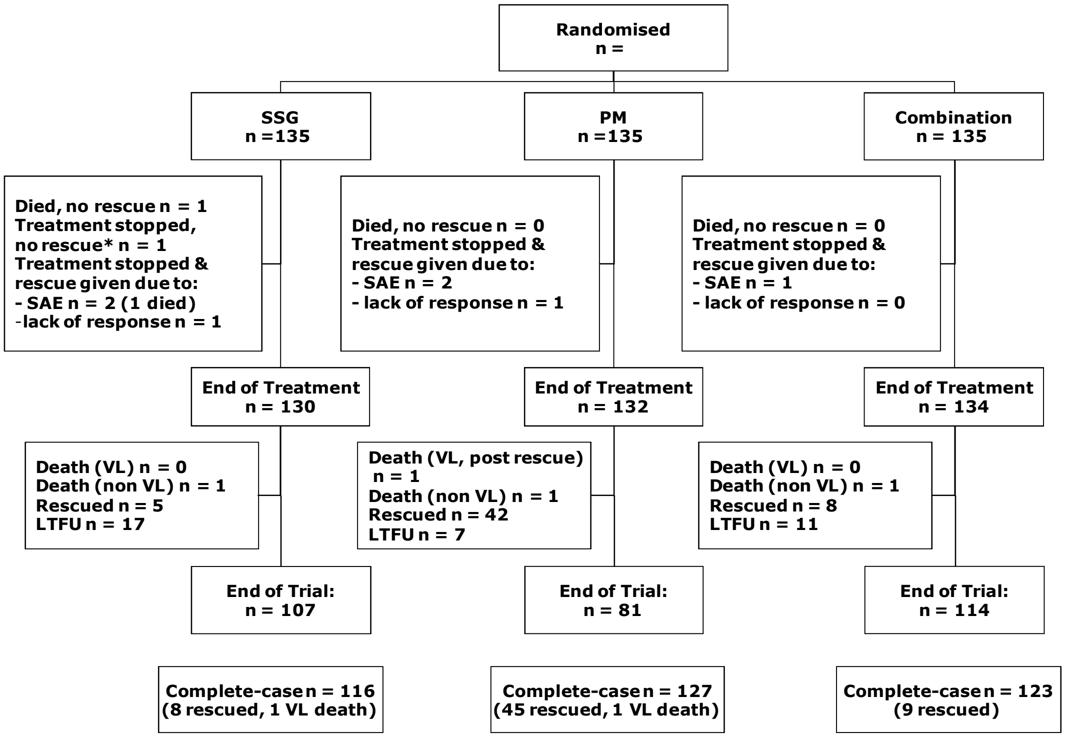

LTFU = loss to follow-up
* Tuberculosis diagnosis day 14 of treatment, no rescue medication given, loss to follow-up

10.2 Baseline Characteristics

Table 3 Baseline Demographic characteristics

**Demographics, n (%)**		**SSG N = 135**	**PM N = 135**	**Comb N =135**	**p-value***	**p-value†**
Age (years) ‡	mean (SD)	16.7 (10.4)	17.8 (11.1)	16.1 (9.4)	0.385	0.397
	4 – 14	69 (51.1)	67 (49.6)	68 (50.4)	0.992	0.903
	≥ 15	66 (48.9)	68 (50.4)	67 (49.6)		
Sex	Female	34 (25.2)	31 (23.0)	34 (25.2)	0.896	0.776
	Male	101 (74.8)	104 (77.0)	101 (74.8)		
Randomised at Centre	Kenya	15 (11.1)	15 (11.1)	15 (11.1)		
	Um El Kher	30 (22.2)	30 (22.2)	30 (22.2)		
	Kassab	15 (11.1)	15 (11.1)	15 (11.1)		
	Gondar	45 (33.3)	45 (33.3)	45 (33.3)		
	Arba Minch	30 (22.2)	30 (22.2)	30 (22.2)		

* p-value from ANOVA for age comparison and chi-squared or Fishers test for sex and categorical age comparisons, across all arms

† p-value from t-test for age comparison and chi-squared or Fisher's test for sex and categorical age comparisons, between SSG and PM

‡ Children classified as aged 4 to 14 years and adults, 15 years and above.

There was little or no evidence of variation in baseline parameters across all three treatment arms or between SSG and PM arms (Tables 3 to 7).

Table 4 Baseline Biological Markers

		SSG	PM	Comb	p-value*	p-value†
Temperature (°C)	mean (SD)	37.9 (1.1)	38.0 (1.2)	38.0 (1.1)	0.727	0.579
	median (IQR)	37.7 (1.7)	38.0 (1.9)	38.0 (1.8)	0.626	0.443
Heart Rate, (beats/min)	mean (SD)	104.7 (16.8)	102.7 (17.8)	103.8 (15.5)	0.613	0.341
	median (IQR)	108 (20)	102 (24)	104 (23)	0.518	0.262
Spleen Size (cm)	mean (SD)	8.2 (4.3)	8.3 (4.9)	8.2 (4.5)	0.976	0.828
	median (IQR)	7 (5)	8 (6)	7 (7)	0.994	0.919
Liver Size (cm)	mean (SD)	2.9 (2.4)	2.9 (2.3)	3.0 (2.4)	0.921	0.872
	median (IQR)	3 (4)	3 (4)	3 (4.5)	0.914	0.698
Systolic BP (mm Hg)	mean (SD)	97.6 (11.3)	100.2 (10.9)	97.3 (11.2)	0.066	0.057
	median (IQR)	100 (10)	100 (20)	100 (10)	0.080	0.063
Diastolic BP (mm Hg)	mean (SD)	63.3 (9.3)	64.7 (9.5)	62.8 (8.9)	0.225	0.207
	median (IQR)	60 (10)	60 (10)	60 (10)	0.191	0.172
Nutritional Status‡	Severely underweight	17 (12.6)	17 (12.6)	28 (20.7)	0.262	0.685
	Underweight	61 (45.2)	67 (49.7)	56 (41.6)		
	Normal	57 (42.2)	50 (37.0)	50 (37.0)		
	Obese/overweight	0 (0)	0 (0)	1 (0.7)		

BP = Blood pressure

* p-value from ANOVA for comparison of mean values, Kruskall-Wallis where median values are given and chi-squared or Fishers test for categorical data comparisons.

† p-value from t-test for comparison of means and chi-squared or Fishers test for categorical data comparisons, between SSG and PM

‡ Based on weight for age in children (4-14 years), defined as severely underweight if <60%, underweight: 60 - 80%, normal: >80%. Based on BMI in adults (15-60 years) defined as severely underweight if < 16, underweight: 16.0 – 18.4, normal: 18.5 – 24.9. Missing BMI value for 1 patient in the PM arm.

Table 5 Baseline Laboratory parameters

Laboratory parameters*		SSG	PM	Comb	p-value†	p-value‡
Parasite Count (log scale)	6+	7 (5.2%)	11 (8.2%)	14 (10.4%)	0.524	0.440
	5+	18 (13.3%)	21 (15.6%)	24 (17.8%)		
	4+	28 (20.7%)	27 (20.0%)	24 (17.8%)		
	3+	30 (22.2%)	19 (14.1%)	29 (21.5%)		
	2+	34 (25.2%)	32 (23.7%)	26 (19.3%)		
	1+	18 (13.3%)	25 (18.5%)	18 (13.3%)		
Haemoglobin (g/dl)	mean (SD)	8.0 (1.8)	8.1 (1.9)	7.8 (2.0)	0.446	0.686
	median (IQR)	7.8 (2.3)	7.9 (2.4)	7.5 (2.5)	0.236	0.598
White-cell Count ($x10^3/\mu L$)	mean (SD)	2.7 (1.7)	2.6 (1.6)	2.4 (1.5)	0.443	0.714
	median (IQR)	2.3 (1.7)	2.1 (1.5)	2 (1.3)	0.368	0.939
Platelets§ ($x10^3/\mu L$)	mean (SD)	109.2 (54.7)	108 (88.4)	105.1 (49.9)	0.900	0.901
	median (IQR)	94 (61.0)	95.5 (59.5)	95.5 (55)	0.896	0.672
AST||, (U/L)	mean (SD)	50.5 (28.2)	50.5 (36.0)	49.9 (32.9)	0.986	0.992
	median (IQR)	46 (45.4)	42.3 (40.7)	44 (40)	0.823	0.576
ALT, (U/L)	mean (SD)	28.1 (17.7)	26.5 (19.9)	27.1 (19.7)	0.786	0.488
	median (IQR)	23.2 (18)	20.5 (16.3)	22 (19)	0.239	0.102
Bilirubin¶, (umol/L)	mean (SD)	11.2 (6.7)	11.2 (5.5)	11.4 (5.4)	0.953	0.957
	median (IQR)	9.6 (5.1)	9.9 (5.4)	10.3 (6.8)	0.732	0.771
BUN**, (mmol/L)	mean (SD)	7.2 (3.3)	7.3 (3.4)	7.6 (3.7)	0.712	0.998
	median (IQR)	6.2 (5.1)	6.3 (4.8)	6.5 (6.0)	0.851	0.912
Creatinine (umol/L)	mean (SD)	75.9 (24.9)	74.5 (25.1)	75.0 (25.5)	0.894	0.639
	median (IQR)	74.2 (44.7)	70.7 (42.7)	73.7 (47.6)	0.937	0.733
Amylase††, (micromol/L)	mean (SD)	82.1 (35.4)	87.8 (50.1)	69.6 (37.6)	0.313	0.668
	median (IQR)	76 (45.3)	82.1 (53.7)	66 (40.4)	0.324	0.934
Alkaline Phosphatase, (U/L)	mean (SD)	325.1 (291.6)	265.1 (178.8)	272.2 (228.5)	0.103	0.056
	median (IQR)	244 (205)	223 (172.5)	206 (173)	0.150	0.221

Data are n (%) of number randomised to each arm for parasite count, other data summaries are mean (SD) of number of values indicated as being available for each measure

* Measurements were not done at all the sites; WBC not measured at Um El Kher, Alkaline Phosphatase not measured at Kassab, Serum amylase only measured at Arba Minch.

† p-value from ANOVA for comparison of mean values, Kruskall-Wallis where median values are given and chi-squared or Fishers test for categorical data comparisons.

‡ p-value from t-test for comparison of means and chi-squared or Fishers test for sex and categorical age comparisons, between SSG and PM

§ Missing platelet values: SSG 2, PM 1, Comb 1

|| Missing AST values: PM 1

¶ Missing Bilirubin values: SSG 3, PM 3, Comb 2

** Missing BUN values: SSG 22, PM 22, Comb 26

†† Missing Amylase values: SSG 9, PM 6, Comb 6

Table 6 Baseline clinical characteristics

Clinical characteristics, n (%)		SSG N =135	PM N =135	Comb N =135	p-value*	p-value†
Audiometry	Normal	101 (74.8)	101 (74.8)	101 (74.8)		
	Abnormal‡	0	0	1 (0.7)		
	Not done	34 (25.2)	34 (25.2)	33 (24.5)		
ECG	Normal	134 (99.3)	135 (100)	134 (99.3)		
	Abnormal‡	0	0	1 (0.7)		
	Not done	1 (0.7)	0	0		
Malaria prior to treatment		7 (5.2)	5 (3.7)	4 (3.0)	0.634	0.769
Pneumonia		6 (4.4)	4 (3.0)	3 (2.2)	0.573	0.749
Otitis Media		0	0	0		
HIV	Positive	4 (3.0)	6 (4.4)	6 (4.4)	0.959	0.812
	Negative	87 (64.4)	86 (63.7)	88 (65.2)		
	Not tested	44 (32.6)	43 (31.9)	41 (30.4)		

Data are n (%)

* p-value from chi-squared or Fishers test across all arms

† p-value from chi-squared of Fishers test between SSG and PM

‡ Abnormal readings considered to be clinically significant; normal and clinically insignificant recordings grouped together. A clinically significant abnormality in Audiometry data corresponds to clinical hearing loss.

Table 7 Baseline Symptoms

Symptom, n (%)	SSG N =135	PM N =135	Comb N = 135	p-value*	p-value†
Fever	133 (98.5)	133 (98.5)	131 (97.0)	0.600	1.000
Headache	94 (69.6)	98 (72.6)	92 (68.2)	0.656	0.525
Fatigue	109 (80.7)	102 (75.6)	108 (80)	0.530	0.303
Epistaxis	46 (34.1)	51 (37.8)	47 (34.8)	0.797	0.526
Abdominal pains	89 (65.9)	88 (65.2)	79 (58.5)	0.381	0.898
Abdominal swellings	77 (57.0)	84 (62.2)	72 (53.3)	0.332	0.385
Swelling of legs	27 (20.0)	20 (14.8)	18 (13.3)	0.293	0.261
Cough	91 (67.4)	83 (61.5)	84 (62.2)	0.544	0.309
Breathlessness	63 (46.7)	59 (43.7)	65 (48.2)	0.757	0.625
Night Sweats	89 (65.9)	85 (62.9)	80 (59.3)	0.525	0.611
Loss of appetite	93 (68.9)	91 (67.4)	94 (69.6)	0.923	0.794
Weight loss	114 (84.4)	111 (82.2)	108 (80.0)	0.634	0.624
Diarrhoea	34 (25.2)	31 (23.0)	40 (29.6)	0.445	0.669
Skin lesions	6 (4.4)	7 (5.19)	5 (3.7)	0.840	0.776

* p-value from chi-squared test across all arms

† p-value from chi-squared test between SSG and PM

10.3 Deviations from Protocol:

10.3.1 Exclusion criteria at baseline

Table 8 Minor Protocol Violations at Baseline

Entry Criteria, n	SSG<br>N = 135	PM<br>N = 135	Comb<br>N = 135
Aged < 4 or > 60 years	0	0	0
Not diagnosed with VL	0	0	0
Received anti-leishmanial drug in the last 6 months	0	0	0
Malnutrition*	17 (12.6)	17 (12.6)	28 (20.7)
Haemoglobin < 5g/dl	0	1 (0.7)	3 (2.1)
WBC < 1 x 10^3/mm	0	0	0
Platelets < 40,000/mm	0	1 (0.7)	0
Liver function tests more than 3 times ULN	0	1 (0.7)	1 (0.7)
Creatinine outside the normal range for age & gender	4 (3.0)	5 (3.7)	5 (3.7)
Pre-existing clinical hearing loss†	0	0	1 (0.7)
History of cardiac arrhythmia or an abnormal ECG‡	0	0	1 (0.7)
Concomitant severe infection	0	0	0

Data are n (%)

* assessed as severely underweight using weight-for-age in 4-14 year olds and BMI in patients 15 years and above

† 1 patient had moderate to moderately severe hearing loss in the right ear at baseline

‡ clinically significant abnormality

10.3.2 Non-Compliance to Treatment Regimen

SSG Arm (30 day treatment period):

- 1 patient received 14 days of treatment only
- 1 patient received full 30 days but a gap of 1 day on day 18 of treatment
- 1 patient received 14 days of treatment: once daily for 4 days, twice daily (half dose) for 10 days

PM Arm (21 day treatment period):

- 1 patient received 20 days of PM treatment and one day of SSG on day 2

Combination Arm (17 days treatment period):

- 1 patient received 11 days of Combination treatment: days 1 – 8, 14 – 16 inclusive

10.3.3 Timing of Final Assessment

Outcome data were missing for 25 patients at 6 months as a result of early follow up. Of those, 20 patients were followed up once within the first 4.5 months after the end of treatment. Follow-up data collected for these patients were handled as 3 months data. A further 2 patients were seen within 1.5 months of TOC and again within 4.5 months of TOC. For these patients, data from the later visit was handled as 3 month follow-up data in analyses. For the remaining 3 patients, data were collected within 1.5 months of

the expected 3 month follow-up data and again within 4.5 months after TOC. Since the final data collection took place prior to 4.5 months post end of treatment, the 6 months outcome data were handled as missing data.

For 18 patients, primary endpoint data were collected more than 1.5 months (45 days) after the expected date of 6 months follow-up (Table 9).

Table 9 Timing of final assessment

**Final evaluation**	**SSG**<br>**N = 110**	**PM**<br>**N =99**	**Comb**<br>**N =121**
Within 2 weeks of expected date	51 (46.4)	52 (52.5)	71 (58.7)
>2 weeks to <1.5 months	53 (48.2)	39 (39.4)	46 (38.0)
1.5 to 3 months after expected date	5 (4.5)	6 (6.1)	3 (2.5)
>3 to 4 months after expected date	1 (0.9)	1 (1.0)	0
>4 to 5 months after expected date	0	0	1 (0.8)
>7 to 8 months after expected date	0	1 (1.0)	0

Data are n (%)

10.4 Primary Efficacy Analysis: Overall

10.4.1 Data handling of 6 months data

As described in the methods, in the complete-case analysis missing values were **replaced as treatment failures** if

- Death occurred during initial hospitalisation period, regardless of whether rescue medication was administered
- Death occurred during follow-up and rescue was administered previously
- Patients were not seen at 6 months (LTFU) but rescue was administered previously
- Any other scenario where parasitology data were not available at 6 months but rescue was administered previously

Data remain missing

- Where patients were LTFU with no record of receipt of rescue medication prior to loss,
- In the case of three deaths that occurred during follow up, confirmed to be independent of VL and the study drug by the medical co-ordinator (one death per arm);
    1. Unknown cause
    2. Sepsis and Immune Reconstitution
    3. Pericarditis due to TB

10.4.2 Primary Efficacy Analysis

Table 10 Definitive Cure Analysis 1: Complete Case Analysis

**Estimation**	**SSG**<br>**N = 116**	**PM**<br>**N = 127**	**Comb**<br>**N = 123**
Treatment Efficacy at 6 months, n (%)	107 (92.2)	81 (63.8)	110 (89.4)
Difference between SSG & PM (95% CI)	28.5% (18.8 to 38.1)		
Difference between SSG & Combination (95% CI)	2.8% (-4.5 to 10.1)		
Test of difference across arms: p-value*	< 0.001		
Test of difference across centres, after adjustment for treatment : p-value*	0.001		
Test of difference between adult & paediatric† patients, after adjustment for treatment : p-value*	0.454		

* p-value from likelihood ratio test, comparing models with and without variable being tested.

† paediatric: 4 to 14 years, adult 15 – 60 years

There was strong evidence of a difference in efficacy between PM and SSG arms; the efficacy of PM was approximately 30% lower than the efficacy of SSG overall, although the efficacy could be somewhere between 20% and 40% lower than SSG (Table 10). There was no evidence of a difference in overall efficacy between the Combination and SSG arms. The efficacy of the Combination treatment was around 3% lower than SSG but 95% confidence bounds around the difference in efficacy show that results regarding inferiority of Combination treatment, compared to SSG, were inconclusive.

There was strong evidence of an overall difference in efficacy by treatment arm ($p < 0.001$), due to the much lower efficacy in the PM arm.

After taking into account treatment allocation, there was strong evidence of a difference in efficacy by centre ($p = 0.001$) but no evidence of a difference in efficacy by age ($p = 0.454$) when considering adult patients compared to paediatric patients.

In the LPCF analysis (Table 11), missing values for parasitology at 6 months were replaced by the parasitology result obtained during the previous parasitological examination. One patient in the SSG arm was excluded from this analysis because parasitological data were not available for this patients at the end of treatment or during follow-up.

Differences in results of the LPCF analysis and complete-case analysis were negligible.

Table 11 Definitive Cure Analysis 2: Last parasitology carried forward

Estimation	SSG† N = 134	PM N = 135	Comb N = 135
Treatment efficacy at 6 months, n (%)	124 (92.5)	88 (65.2)	122 (90.4)
Difference between SSG & PM (95% CI)	27.4% (18.2 to 36.5)		
Difference between SSG & Combination (95% CI)	2.2% (-4.5 to 8.8)		
Test of difference across arms: p-value*	< 0.001		
Test of difference across centres, after adjustment for treatment: p-value*	0.018		
Test of difference between adult & paediatric‡ patients, after adjustment for treatment : p-value*	0.379		

* p-value from likelihood ratio test, comparing models with and without variable being tested.

† Parasitology examination not performed at end of treatment, 3 or 6 months follow-up for one patient in the SSG arm.

‡ paediatric: 4 to 14 years, adult 15 – 60 years

Loss to follow up was substantially higher in the SSG arm so, in the worst case analysis, the efficacy of SSG is calculated as 79% rather than 93% (Table 12).

In the worst case analysis, the efficacy of the combination treatment is estimated to be higher than in the SSG arm, although the absolute difference and corresponding confidence bounds around the difference in efficacy are very similar to results of the previous two analyses. The lower estimate of efficacy in the SSG arm leads to a smaller estimated difference in efficacy between SSG and PM; around 20% overall, compared to 30% in the previous two analyses.

The worst case analysis also provides strong evidence of variability by centre, after adjustment for treatment received ($p < 0.001$) and no evidence of variability between age groups ($p = 0.346$).

Table 12 Definitive Cure Analysis 3: Worst-case analysis

Estimation	SSG N = 135	PM N =135	Comb N = 135
Parasite free at 6 months, n (%)	107 (79.3)	81 (60.0)	110 (81.5)
Difference between SSG & PM (95% CI)	19.3% (8.5 to 30.0)		
Difference between SSG & Combination (95% CI)	-2.2% (-11.7 to 7.2)		
Test of difference across arms: p-value*	< 0.001		
Test of difference across centres, after adjustment for treatment: p-value*	< 0.001		
Test of difference between adult & paediatric† patients, after adjustment for treatment : p-value*	0.346		

* p-value from likelihood ratio test, comparing models with and without variable being tested.

† paediatric: 4 to 14 years, adult 15 – 60 years

10.4.3 Primary Efficacy Analysis: By centre

Table 13 DC: Complete Case Analysis: By Centre

Site	SSG	PM	Comb	p-value*†
Um el Kher	14 / 17 (82.3%)	4 / 28 (14.3%)	18 / 20 (90.0%)	< 0.001
Kassab	14 / 15 (93.3%)	7 / 15 (46.7%)	14 / 15 (93.3%)	0.003
Kenya	15 / 15 (100.0)	12 / 15 (80.0%)	11 / 15 (73.3%)	0.134
Gondar	37 / 40 (92.5%)	30 / 40 (75.0%)	39 / 43 (90.7%)	0.064
Arba Minch	27 / 29 (93.1%)	28 / 29 (96.6%)	28 / 30 (93.3%)	1.000
p-value*‡	0.568	< 0.001	0.373	

* p-value from Fisher's exact test

† comparison across arms within sites

‡ comparison across sites within arms

In the complete-case analysis, there was strong evidence of a difference in PM efficacy across sites ($p < 0.001$) and also between all treatment regimens in Sudanese sites ($p < 0.01$), due to much lower efficacy in the PM arm (Table 13).

In Gondar, the efficacy of SSG and Combination treatments was around 90% but approximately 15% lower in PM arm with weak statistical evidence of a difference across all arms ($p = 0.064$).

In Kenya, the efficacy of PM was around 20% lower than SSG and the efficacy of the combination treatment was almost 25% lower. The small sample size in Kenya does not provide adequate power to detect statistical evidence of a difference and it is impossible to predict whether the results based on increased numbers of patients in Kenya would be more balanced.

Similar efficacy was observed in all three arms in Arba Minch.

LTFU at 6m was higher in Um el Kher with evidence of a difference in LTFU by arm (Fisher's exact test $p = 0.001$).

The LPCF analysis by-centre produced very similar results to the complete-case analysis (Table 14).

Table 14 DC: LPCF Analysis: By Centre

Site	SSG	PM	Comb	p-value*†
Um el Kher	25 / 29 (89.2%)	6 / 30 (20.0)	28 / 30 (93.3%)	< 0.001
Kassab	14 / 15 (93.3%)	7 / 15 (46.7%)	14 / 15 (93.3%)	0.003
Kenya	15 / 15 (100%)	12 / 15 (80.0%)	11 / 15 (73.3%)	0.134
Gondar	42 / 45 (93.3%)	34 / 45 (75.6%)	41 / 45 (91.1%)	0.037
Arba Minch	28 / 29 (96.6%)	29 / 30 (96.7%)	28 / 30 (93.3%)	1.000
p-value*‡	0.636	< 0.001	0.302	

* p-value from Fisher's exact test

† comparison across arms within sites

‡ comparison across sites within arms

In the worst-case analysis assuming treatment failure for all patients with missing 6 months efficacy data in Um el Kher, substantially reduced efficacy estimates in the SSG and Combination arms. Based on these reduced estimates, there was strong evidence of a difference in efficacy in SSG and Combination arms, across treatment centres, results not seen in previous by-centre analyses.

Results of the worst case analysis, comparing efficacy across arms within each site were similar to complete-case and LPCF analyses (Table 15).

Table 15 DC: Worst Case Analysis: By Centre

Site	SSG	PM	Comb	p-value*†
Um el Kher	14 / 30 (46.7%)	4 / 30 (13.3%)	18 / 30 (60.0%)	0.001
Kassab	14 / 15 (93.3%)	7 / 15 (46.7%)	14 / 15 (93.3%)	0.003
Kenya	15 / 15 (100%)	12 / 15 (80.0%)	11 / 15 (73.3%)	0.134
Gondar	37 / 45 (82.2%)	30 / 45 (66.7%)	39 / 45 (86.7%)	0.059
Arba Minch	27 / 30 (90.0%)	28 / 30 (93.3%)	28 / 30 (93.3%)	1.000
p-value*‡	< 0.001	< 0.001	0.007	

* p-value from Fisher's exact test

† comparison across arms within sites

‡ comparison across sites within arms

10.5 Secondary Efficacy Parasitology Analysis

10.5.1 Data Handling at Test of Cure

In the complete-case analysis, missing efficacy data were replaced as treatment failures in the following cases, since these events could not be assumed to be independent of VL or the study drug;

- Death during initial hospitalization period, before or after receipt of rescue
- Stopping of study treatment due to SAE or lack of response to treatment

Stopping treatment due to a protocol violation or, investigator error in not conducting parasitological exam were not assumed to indicate treatment failure.

10.5.2 Secondary Efficacy Parasitology Analysis: All sites

There was too little power to test for a difference by centre, after adjustment for treatment arm. Given the strong evidence of a difference between centres in the primary analysis, a by-centre analysis was also carried out for the secondary end point.

Table 16 Test of Cure Analysis 1: Complete Case Analysis

Estimation	SSG N = 134	PM N =135	Comb‡ N = 133
Treatment Efficacy at end of treatment, n (%)	123 (91.8)	91 (67.4)	121 (91.0)
Difference between SSG & PM (95% CI)	24.4% (15.2 to 33.6%)		
Difference between SSG & Combination (95% CI)	0.8% (-5.9 to 7.5)		
Test of difference across arms: p-value*	< 0.001		
Test of difference between adult & paediatric† patients, after adjustment for treatment : p-value*	0.793		

* p-value from likelihood ratio test, comparing models with and without variable being tested.

† paediatric: 4 to 14 years, adult 15 – 60 years

‡ Treatment was stopped prematurely for 2 patients who did not subsequently receive rescue medication or undergo parasitological testing at end of treatment

Comparing complete-case results at DC and TOC, efficacy in the SSG and Combination arms only differed by 1% (Tables 10 & 16). In the PM arm, efficacy differed by 3%, so overall differences in efficacy and corresponding 95% CIs showed similar results for primary and secondary end points.

Results from both the worst-case and complete-case analyses at TOC were very similar due to there being only 3 missing values (Tables 16 & 17).

Table 17 Test of Cure Analysis 2: Worst Case Analysis

Estimation	SSG<br>N = 135	PM<br>N =135	Comb<br>N = 135
Treatment Efficacy at end of treatment, n (%)	123 (91.1)	91 (67.4)	121 (89.6)
Difference between SSG & PM (95% CI)	23.7% (14.5 to 33.0%)		
Difference between SSG & Combination (95% CI)	1.5% (-5.5 to 8.5)		
Test of difference across arms: p-value*	< 0.001		
Test of difference between adult & paediatric† patients, after adjustment for treatment : p-value*	0.678		

* p-value from likelihood ratio test, comparing models with and without variable being tested.

† paediatric: 4 to 14 years, adult 15 – 60 years

10.5.3 Secondary Efficacy Parasitology Analysis: By Centre

The overall efficacy of each treatment regimen remained almost the same between TOC and DC in complete-case analyses. In Kenya and Sudan there appeared to be more failures during follow-up in the PM arm and some variability in Combination treatment efficacy across sites. Patient numbers by-centre were too small however to consider statistical evidence of potential differences.

Table 18 TOC: Complete Case Analysis By-Centre

Site	SSG	PM	Comb	p-value*†
Um el Kher	26 / 29 (89.7%)	10 / 30 (33.3%)	26 / 30 (86.7%)	< 0.001
Kassab	14 / 15 (93.3%)	9 / 15 (60.0%)	12 / 15 (80.0%)	0.113
Kenya	15 / 15 (100%)	13 / 15 (86.7%)	13 / 13 (100%)	0.319
Gondar	40 / 45 (88.9%)	30 / 45 (66.7%)	40 / 45 (88.9%)	0.014
Arba Minch	28 / 30 (93.3%)	29 / 30 (96.7%)	30 / 30 (100%)	0.770
p-value*‡	0.832	< 0.001	0.127	

* p-value from chi-squared test or Fisher's exact test if justified

† comparison across arms by site

‡ comparison across sites by arm

Table 19 TOC: Worst Case Analysis By-Centre

Site	SSG	PM	Comb	p-value*†
Um el Kher	26 / 30 (86.7%)	10 / 30 (33.3%)	26 / 30 (86.7%)	< 0.001
Kassab	14 / 15 (93.3%)	9 / 15 (60.0%)	12 / 15 (80.0%)	0.113
Kenya	15 / 15 (100%)	13 / 15 (86.7%)	13 / 15 (86.7%)	0.524
Gondar	40 / 45 (88.9%)	30 / 45 (66.7%)	40 / 45 (88.9%)	0.014
Arba Minch	28 / 30 (93.3%)	29 / 30 (96.7%)	30 / 30 (100%)	0.770
p-value*‡	0.713	< 0.001	0.122	

* p-value from chi-squared test or Fisher's exact test if justified

† comparison across arms by site

‖ comparison between SSG and PM by site

‡ comparison across sites by arm

10.5.4 Slow response to Treatment

The proportion of slow responders was less than 5% in all three arms (Table 20).

Table 20 Slow Response by treatment arm

Efficacy Outcome		SSG N = 116	PM N = 127	Comb* N = 121
Treatment Responder†		104 (90.4)	75 (59.1)	105 (85.4)
Slow Responder‡		3 (2.6)	6 (4.7)	5 (4.1)
Log drop in parasite load from baseline to end of treatment in slow responders	1	0	2	1
	2	0	2	2
	>2	3	2	2

Data are n (%)

* Two patients had missing values for parasitology at end of treatment

† parasites not detected at end of treatment or at 6 months follow-up, no rescue administered

‡ parasites detected at end of treatment, not detected at 6 months follow-up, no rescue administered

10.5.5 Rescue Medication Outcomes

Table 21 Rescue Medication and Study Outcome by Treatment

	SSG	PM	Comb	Total
Total number of patients rescued prior to end of trial*	8	46	9	63
Timing of 1st rescue treatment:				
During treatment period	3	3	1	7
During follow-up, prior to 6m assessment	5	43	8	56
Outcome Post Rescue:				
Died*	1	1	0	2
No VL at end of trial†	3	15	5	23
VL at end of trial†	0	3	2	5
Loss to follow up at 6 months	4	26	2	32
Parasitology not done at 6 months‡	0	1	0	1
VL detected at end of trial, first rescue treatment administered at end of trial.	0	0	4	4

EOT = end of treatment: SSG day 31, PM day 22, Combination day 18

* PM arm only: 46 rescued patients presented as 45 rescued patients and 1 VL death in participant flow diagram

† VL measured as presence of parasites in tissue samples on microscopy

‡ patient was seen at 6 months

10.5.6 Follow-up at 3 months

Overall, 278 (83.2%) of 333 patients alive and parasite negative at TOC were followed up at 3 months along with 34 (50.0%) of 65 surviving treatment failures at TOC. Relapses had occurred in approximately 9% of PM patients and less than 5% of each of the SSG and Combination patients (Table 22).

Table 22 Overall Results 3 months Post End of Treatment

Follow up at 3 months	SSG N = 135	PM N = 135	Comb N = 135
Deaths	3 (2.2)	1 (0.7)	1 (0.7)
Patients not seen	23 (17.0)	33 (24.4)	31 (23.0)
Patients seen: assumed to be clinically well*	78 (57.8)	61 (45.2)	72 (53.3)
Patients seen: parasites not detected	27 (20.0)	24 (17.8)	25 (18.5)
Patients seen: parasites detected	4 (3.0)	16 (11.9)	6 (4.4)
Patients seen: parasites not detected at TOC	102	82	94
Relapses†	2	7	4

EOT = end of treatment

* clinically well patients did not have parasitological examination.

† Relapse: parasites not detected at TOC, parasites detected at 3 months

10.6 Safety ECG and Audiometry Analysis

10.6.1 Safety Analysis: ECG

Table 23 Number of ECG Tests performed per centre

Day	Centre				
	Gondar N = 135	Arba Minch N = 90	Kenya N = 45	Um el Kher N = 90	Kassab N = 45
0	135	89	45	90	45
7	91	46	45	1	45
14	134	90	44	88	43
21	30	16	15	0	14
EOT	132	88	43	87	43
90	84	45	42	11	0
180	121	85	43	35	33

EOT = End of Treatment

Table 24 ECG Analysis during treatment and follow-up

Estimation	SSG	PM	Comb
Number of patients randomised & normal on day 0	134	135	134
Abnormal *on* day 7, n (%)	1 (0.8)	1 (0.7)	1 (0.8)
Abnormal *on* day 14, n (%)	2 (1.5)	1 (0.7)	0
Abnormal *by* day 14, n (%)	3 (2.2)	2 (1.5)	1 (0.8)
Abnormal *by* end of treatment, n (%)	4 (3.0)	3 (2.2)	1 (0.8)
Abnormal result *at* end of treatment, n (%)	1 (0.8)	1 (0.7)	0
Abnormal result remaining at 6 months*, n (%)	0	0	0

* abnormal result at end of treatment, not returning to normal by end of follow-up

Less than 1% of patients in each of the SSG and PM arms with normal ECG results at baseline had an abnormal result at the end of treatment (Table 24). No patients with a normal baseline reading in the Combination arm had an abnormal result at end of treatment. Tests were not conducted across arms due to the small numbers of abnormal results at end of treatment.

10.6.2 Safety Analysis: Audiometry

Table 25 Number of Audiometry examinations performed per centre

Day	Centre				
	**Gondar N = 135**	**Arba Minch N = 90**	**Kenya N = 45**	**Um el Kher N = 90**	**Kassab N = 45**
**0**	135	79	45	0	45
**7**	91	35	45	0	45
**14**	134	78	43	0	41
**21**	30	14	15	0	14
**End of Treatment**	132	77	43	0	43
**90**	86	38	40	0	0
**180**	121	78	44	0	33

One patient allocated to Combination treatment had moderate to moderately severe hearing loss in their right ear at baseline and throughout the study. This was recorded earlier as a minor protocol violation.

There were no clinically significant audiometric readings recorded during treatment or follow-up

10.6.3 Urinalysis: Protein and Blood

Comparisons were made across all treatment arms and between SSG and PM separately. In addition to comparisons across all categorisations, comparisons were made for patients who completed treatment and hence had urinalysis results at both baseline and end of treatment.

Note that this analysis assumes that a negative reading indicates a normal result and positive, abnormal.

There was no evidence of a difference in how urinalysis parameters changed during treatment, across arms, or between SSG and PM arms.

Table 26 Urinalysis: Protein

Estimation	SSG<br>N = 135	PM<br>N =135	Comb<br>N =135
Baseline negative, end of treatment negative	73 (54.1)	78 (57.8)	72 (53.3)
Baseline negative, end of treatment positive	4 (3.0)	5 (3.7)	9 (6.7)
Baseline positive, end of treatment positive	6 (4.4)	5 (3.7)	6 (4.4)
Baseline positive, end of treatment negative	45 (33.3)	41 (30.4)	44 (32.6)
Baseline negative, end of treatment missing	4 (3.0)	2 (1.5)	3 (2.2)
Baseline positive, end of treatment missing	2 (1.5)	1 (0.7)	0 (0)
Test of Difference across all arms *	0.892		
Test of Difference across all arms †	0.848		
Test of Difference between SSG & PM *	0.911		
Test of Difference between SSG & PM †	0.906		

Data are n (%)

Data unavailable at both baseline and end of treatment for 5 patients; SSG 1, PM 3, Comb 1

* p-value from chi-squared or Fishers test across all categories

† p-value from chi-squared or Fishers test across patients with values at both baseline and end of treatment (1st four categories)

Table 27 Urinalysis: Blood

Estimation	SSG<br>N = 135	PM<br>N =135	Comb<br>N =135
Baseline negative, end of treatment negative	102 (75.6)	96 (71.1)	102 (75.6)
Baseline negative, end of treatment positive	4 (3.0)	5 (3.7)	6 (4.4)
Baseline positive, end of treatment positive	10 (7.4)	16 (11.9)	8 (5.9)
Baseline positive, end of treatment negative	11 (8.2)	12 (8.9)	13 (9.6)
Baseline negative, end of treatment missing	6 (4.4)	1 (0.7)	3 (2.2)
Baseline positive, end of treatment missing	0 (0)	2 (1.5)	0 (0)
Test of Difference across all arms *	0.411		
Test of Difference across all arms †	0.718		
Test of Difference between SSG & PM *	0.209		
Test of Difference between SSG & PM †	0.622		

Data are n (%)

Data unavailable at both baseline and end of treatment for 8 patients; SSG 2, PM 3, Comb 3

* p-value from chi-squared or Fishers test across all categories

† p-value from chi-squared or Fishers test across patients with values at both baseline and end of treatment (1st four categories)

10.7 Secondary Efficacy Analysis: Biological Parameters during Treatment

Mean differences in parameters between time points are also presented with corresponding 95% CIs. ANCOVA tests for evidence of a difference between treatment arms adjust for baseline values and treatment centre.

All available data were used in this analysis. Data collection ceased if a patient was withdrawn from treatment due to a serious adverse event. It is important to note that other patients may have experienced an adverse event that led to a large change in one of the parameters included in this analysis, such values were not excluded.

Table 28 Difference in biological parameters between baseline and Day 7

Efficacy Marker	SSG		PM		Comb		p-value*	p-value†
	N	Mean Change (95% CI)	N	Mean Change (95% CI)	N	Mean Change (95% CI)		
Weight gain (Kg)	135	0.76 (0.51 to 1.01)	135	0.52 (0.27 to 0.77)	135	0.53 (0.27 to 0.79)	0.345	0.186
Temperature (°C)	135	-1.30 (-1.53 to -1.08)	135	-0.91 (-1.11 to -0.70)	135	-1.42 (-1.64 to -1.20)	<0.001	<0.001
Heart Rate, (beats/min)	135	-7.71 (-10.29 to -5.13)	135	-3.67 (-6.26 to -1.09)	135	-5.17 (-7.53 to -2.81)	0.118	0.053
Spleen Size (cm)	135	-1.55 (-1.86 to -1.25)	135	-0.97 (-1.28 to -0.66)	135	-1.51 (-1.81 to -1.20)	0.006	0.003
Liver Size (cm)	135	-0.42 (-0.64 to -0.21)	135	-0.26 (-0.47 to -0.05)	135	-0.41 (-0.64 to -0.19)	0.450	0.214
Systolic BP (mm Hg)	135	-1.41 (-3.99 to 0.23)	135	-2.33 (-3.99 to -0.68)	135	-1.07 (-2.64 to 0.49)	0.965	0.973
Diastolic BP (mm Hg)	135	-0.22 (-1.74 to 1.30	135	-1.00 (-2.51 to 0.51)	135	-0.37 (-1.71 to 0.97)	0.902	0.958
Haemoglobin (g/dl)	135	0.12 (-0.08 to 0.32)	135	0.03 (-0.18 to 0.23)	135	0.39 (0.22 to 0.57)	0.042	0.572
White-cell Count ($x10^3/\mu L$)	105	0.24 (-0.04 to 0.53)	105	0.44 (-0.14 to 1.02)	105	0.30 (0.05 to 0.56)	0.746	0.601
Platelets ($x10^3/\mu L$)	102	41.5 (28.8 to 54.2)	104	5.95 (-4.28 to 16.2)	104	38.4 (25.3 to 51.5)	<0.001	<0.001
AST, (U/L)	135	24.9 (16.9 to 33.0)	134	10.4 (4.9 to 15.9)	135	30.8 (21.7 to 39.8)	0.001	0.004
ALT, (U/L)	135	23.7 (16.9 to 30.5)	135	8.8 (5.4 to 12.3)	135	24.5 (17.1 to 31.8)	<0.001	<0.001
Bilirubin, (umol/L)	132	-0.81 (-1.69 to 0.07)	131	0.09 (-0.93 to 1.11)	131	-1.48 (-2.30 to -0.65)	0.020	0.111
BUN, (mmol/L)	104	-0.14 (-1.28 to 1.01)	107	0.05 (-0.30 to 0.41)	101	-0.54 (-1.24 to 0.16)	0.639	0.773
Creatinine (umol/L)	135	-2.90 (-7.17 to 1.36)	135	0.19 (-2.97 to 3.36)	135	-1.82 (-6.29 to 2.64)	0.610	0.298
Amylase, (micromol/L)	21	83.9 (30.97 to 136.83)	22	7.61 (-15.74 to 30.97)	23	71.87 (22.83 to 120.93)	0.002	0.009
Alkaline Phosphatase (U/L)	120	193.18 (110.24 to 276.12)	120	44.7 (14.34 to 75.06)	120	156.13 (117.65 to 194.61)	0.002	0.004

SD = standard deviation, BP = blood pressure

A negative value indicates a decrease in day 7 values, on average, compared to baseline

* p-value from ANCOVA between arms in day 7 measurements, adjusting for baseline values and centre

† p-value from ANCOVA between SSG and PM in day 7 measurements, adjusting for baseline values and centre

Table 29 Difference in biological parameters between Day 7 and Day 14

Efficacy Marker	SSG		PM		Comb		p-value*	p-value†
	N	Mean Change (95% CI)	N	Mean Change (95% CI)	N	Mean Change (95% CI)		
Weight gain (Kg)	134	0.35 (0.13 to 0.57)	133	0.47 (0.28 to 0.65)	133	0.34 (0.13 to 0.55)	0.637	0.424
Temperature (°C)	134	-0.03 (-0.15 to -0.10)	133	-0.43 (-0.60 to -0.26)	133	0 (-0.10 to 0.10)	0.583	0.714
Heart Rate, (beats/min)	134	-2.22 (-4.32 to -0.13)	133	-2.03 (-3.86 to -0.20)	133	-2.90 (-4.68 to -1.12)	0.731	0.515
Spleen Size (cm)	134	-1.46 (-1.72 to -1.02)	133	-1.32 (-1.61 to -1.02)	133	-2.05 (-2.34 to -1.75)	<0.001	0.121
Liver Size (cm)	132	-0.42 (-0.65 to -0.19)	132	-0.47 (-0.66 to -0.29)	133	-0.82 (-1.08 to -0.57)	0.010	0.935
Systolic BP (mm Hg)	134	1.68 (0.11 to 3.24)	133	-0.19 (-1.77 to 1.40)	133	2.86 (1.28 to 4.44)	0.235	0.238
Diastolic BP (mm Hg)	134	0.93 (-0.60 to 2.47)	133	0.60 (-1.02 to 2.22)	133	2.56 (1.12 to 3.99)	0.265	0.911
Haemoglobin (g/dl)	134	1.06 (0.88 to 1.23)	133	1.10 (0.89 to 1.32)	131	1.23 (1.04 to 1.42)	0.324	0.714
White-cell Count (x10^3/µL)	104	0.49 (0.28 to 0.70)	104	0.62 (0.03 to 1.22)	101	0.85 (0.57 to 1.12)	0.386	0.213
Platelets (x10^3/µL)	103	37.8 (22.3 to 53.4)	104	54.1 (43.6 to 64.6)	101	57.2 (45.2 to 69.1)	0.154	0.376
AST, (U/L)	134	-14.1 (-23.1 to -5.13)	133	0.38 (-7.30 to 8.06)	131	-19.0 (-29.3 to -8.75)	0.459	0.322
ALT, (U/L)	134	-5.75 (-12.8 to 1.29)	133	10.1 (5.16 to 15.0)	131	-5.05 (-12.9 to 2.82)	0.211	0.053
Bilirubin, (umol/L)	134	-0.03 (-0.75 to 0.69)	131	-0.82 (-1.86 to 0.23)	129	0.23 (-0.56 to 1.02)	0.704	0.562
BUN, (mmol/L)	106	0.25 (-0.18 to 0.68)	106	0.47 (0.10 to 0.83)	99	0.05 (-0.41 to 0.51)	0.239	0.300
Creatinine (umol/L)	134	-0.50 (-2.81 to 1.80)	133	-1.08 (-3.24 to 1.08)	131	0.22 (-2.56 to 3.01)	0.924	0.991
Amylase, (micromol/L)	22	45.8 (-13.1 to 104.6)	21	3.42 (-9.55 to 16.4)	21	-19.6 (-53.2 to 13.9)	0.092	0.771
Alkaline Phosphatase (U/L)	120	-85.6 (-140.7 to -30.5)	118	50.1 (26.7 to 73.4)	119	-41.9 (-84.4 to 0.67)	0.024	0.005

SD = standard deviation, BP = blood pressure

A negative value indicates a decrease in day 14 values, on average, compared to day 7

* p-value from ANCOVA between arms in day 14 measurements, adjusting for day 7 values and centre

† p-value from ANCOVA between SSG and PM in day 14 measurements, adjusting for day 7 values and centre

Table 30 Difference in biological parameters between Baseline and Day 14

Efficacy Marker	SSG		PM		Comb		p-value*	p-value†
	N	Mean Change (95% CI)	N	Mean Change (95% CI)	N	Mean Change (95% CI)		
Weight gain (Kg)	134	1.14 (0.85 to 1.42)	133	1.02 (0.75 to 1.30)	133	0.88 (0.56 to 1.21)	0.487	0.518
Temperature (°C)	134	-1.34 (-1.56 to -1.11)	133	-1.37 (-1.58 to -1.16)	133	-1.41 (-1.62 to-1.20)	0.729	0.463
Heart Rate, (beats/min)	134	-10.0 (-12.7 to -7.34)	133	-5.97 (-8.54 to -3.39)	133	-8.06 (-10.5 to -5.58)	0.161	0.063
Spleen Size (cm)	134	-3.00 (-3.42 to -2.58)	133	-2.28 (-2.69 to -1.87)	133	-3.56 (-3.97 to -3.15)	< 0.001	0.002
Liver Size (cm)	133	-0.84 (-1.17 to -0.51)	132	-0.77 (-1.05 to -0.48)	133	-1.26 (-1.56 to 0.89)	0.033	0.537
Systolic BP (mm Hg)	134	0.15 (-1.57 to 1.87)	133	-2.48 (-4.35 to -0.61)	133	1.92 (0.38 to 3.46)	0.019	0.208
Diastolic BP (mm Hg)	134	0.56 (-1.18 to 2.30)	133	-0.41 (-2.22 to 1.40)	133	2.33 (0.58 to 4.09)	0.299	0.999
Haemoglobin (g/dl)	134	1.18 (0.94 to 1.42)	133	1.16 (0.89 to 1.42)	131	1.61 (1.36 to 1.86)	0.034	0.958
White-cell Count (x10^3/µL)	104	0.72 (0.40 to 1.05)	104	1.07 (0.81 to 1.32)	101	1.14 (0.81 to 1.47)	0.180	0.084
Platelets (x10^3/µL)	102	78.5 (63.4 to 93.6)	103	59.9 (48.3 to 71.5)	100	94.5 (80.3 to 108.8)	0.002	0.047
AST, (U/L)	134	11.0 (4.50 to 17.5)	132	10.3 (2.95 to 17.7)	131	10.5 (3.85 to 17.2)	0.985	0.879
ALT, (U/L)	134	18.0 (12.3 to 23.7)	133	19.0 (14.1 to 23.9)	131	18.0 (13.0 to 22.9)	0.968	0.832
Bilirubin, (umol/L)	131	-0.83 (-1.79 to 0.13)	129	-0.67 (-1.63 to 0.28)	129	-1.01 (-2.01 to -0.01)	0.942	0.935
BUN, (mmol/L)	103	-0.38 (-0.89 to 0.13)	105	0.43 (-0.03 to 0.88)	100	-0.82 (-1.38 to -0.26)	0.002	0.012
Creatinine (umol/L)	134	-4.95 (-8.05 to -1.85)	133	-0.88 (-4.17 to 2.41)	131	-2.79 (-6.29 to 0.70)	0.208	0.071
Amylase, (micromol/L)	19	146.8 (32.3 to 261.4)	18	10.1 (-12.0 to 32.3)	19	52.7 (8.73 to 96.8)	0.025	0.201
Alkaline Phosphatase (U/L)	120	107.5 (62.5 to 152.6)	118	97.0 (57.1 to 136.9)	119	111.4 (78.4 to 144.5)	0.719	0.569

SD = standard deviation, BP = blood pressure

A negative value indicates a decrease in day 14 values, on average, compared to baseline

* p-value from ANCOVA between arms in day 14 measurements, adjusting for baseline values and centre

† p-value from ANCOVA between SSG and PM in day 14 measurements, adjusting for baseline values and centre

Table 31 Difference in biological parameters between Day 14 and End of treatment

Efficacy Marker	SSG		PM		Comb		p-value*	p-value†
	N	Mean Change (95% CI)	N	Mean Change (95% CI)	N	Mean Change (95% CI)		
Weight gain (Kg)	131	0.99 (0.71 to 1.26)	132	0.72 (0.52 to 0.92)	132	0.36 (0.21 to 0.51)	<0.001	0.118
Temperature (°C)	131	-0.02 (-0.13 to 0.09)	132	-0.10 (-0.21 to 0.01)	132	0.03 (-0.05 to 0.11)	0.111	0.570
Heart Rate, (beats/min)	130	-1.08 (-3.30 to 1.12)	132	-4.0 (-5.89 to -2.11)	132	-1.70 (-3.12 to -0.28)	0.112	0.099
Spleen Size (cm)	131	-1.81 (-2.14 to -1.47)	132	-1.64 (-1.95 to -1.32)	132	-0.86 (-1.07 to -0.65)	<0.001	0.059
Liver Size (cm)	131	-0.63 (-0.87 to -0.39)	131	-0.65 (-0.87 to -0.44)	132	-0.30 (-0.46 to -0.15)	0.107	0.659
Systolic BP (mm Hg)	131	1.64 (0.08 to 3.20)	132	3.26 (1.76 to 4.75)	132	0.72 (-0.88 to 2.32)	0.099	0.115
Diastolic BP (mm Hg)	131	1.22 (-0.31 to 3.83)	132	2.12 (0.41 to 3.83)	132	-0.34 (-1.80 to 1.11)	0.103	0.185
Haemoglobin (g/dl)	130	1.12 (0.89 to 1.35)	132	0.92 (0.68 to 1.15)	129	0.35 (0.20 to 0.50)	<0.001	0.237
White-cell Count (x10^3/µL)	101	1.46 (1.09 to 1.82)	103	0.88 (0.63 to 1.12)	100	1.29 (-0.61 to 3.19)	0.807	0.021
Platelets (x10^3/µL)	101	23.87 (11.07 to 36.68)	103	31.31 (19.50 to 43.13)	99	21.51 (10.38 to 32.63)	0.858	0.614
AST, (U/L)	130	-5.59 (-12.46 to 1.28)	132	-2.14 (-8.74 to 4.45)	130	-1.78 (-5.95 to 2.39)	0.471	0.367
ALT, (U/L)	130	-10.1 (-15.4 to -4.78)	132	0.80 (-4.06 to 5.65)	130	-3.22 (-6.89 to 0.45)	0.001	<0.001
Bilirubin, (umol/L)	129	0.15 (-0.52 to 0.83)	128	0.73 (0.05 to 1.42)	128	-0.34 (-1.02 to 0.34)	0.030	0.103
BUN, (mmol/L)	101	0.91 (-0.68 to 2.50)	104	0.06 (-0.42 to 0.54)	101	-0.23 (-0.58 to 0.12)	0.256	0.321
Creatinine (umol/L)	130	4.11 (-1.30 to 9.51)	132	1.36 (-1.59 to 4.31)	130	0.30 (-2.82 to 3.41)	0.361	0.618
Amylase, (micromol/L)	24	-29.87 (-107.52 to 47.77)	22	1.33 (-11.36 to 14.02)	24	4.63 (-22.47 to 31.74)	0.297	0.055
Alkaline Phosphatase (U/L)	116	-30.63 (-74.94 to 13.67)	117	-8.09 (-46.14 to 29.95)	118	-7.17 (-30.06 to 15.73)	0.914	0.824

SD = standard deviation, BP = blood pressure

A negative value indicates a decrease in end of treatment values, on average, compared to day 14

* p-value from ANCOVA between arms in end of treatment measurements, adjusting for day 14 values and centre

† p-value from ANCOVA between SSG and PM in end of treatment measurements, adjusting for day 14 values and centre

Table 32 Difference in biological parameters between Baseline and End of treatment

Efficacy Marker	SSG		PM		Comb		p-value*	p-value†
	N	Mean Change (95% CI)	N	Mean Change (95% CI)	N	Mean Change (95% CI)		
Weight gain (Kg)	131	2.13 (1.79 to 2.47)	132	1.74 (1.42 to 2.07)	132	1.25 (0.92 to 1.58)	0.001	0.085
Temperature (°C)	131	-1.37 (-1.58 to -1.17)	132	-1.45 (-1.66 to -1.25)	132	-1.39 (-1.60 to -1.18)	0.166	0.705
Heart Rate, (beats/min)	130	-11.8 (-14.3 to -9.25)	132	-10.1 (-12.7 to -7.46)	132	-9.70 (-12.2 to -7.24)	0.547	0.995
Spleen Size (cm)	131	-4.83 (-5.38 to -4.28)	132	-3.92 (-4.49 to -3.35)	132	-4.41 (-4.88 to -3.95)	0.001	0.001
Liver Size (cm)	130	-1.52 (-1.90 to -1.15)	131	-1.43 (-1.80 to -1.05)	132	-1.53 (-1.91 to -1.15)	0.607	0.343
Systolic BP (mm Hg)	131	1.56 (-0.31 to 3.44)	132	0.68 (-1.36 to 2.73)	132	2.58 (0.74 to 4.41)	0.784	0.810
Diastolic BP (mm Hg)	131	1.64 (-0.18 to 3.46)	132	1.78 (0.01 to 3.55)	132	2.01 (0.41 to 3.61)	0.497	0.258
Haemoglobin (g/dl)	130	2.33 (2.03 to 2.63)	132	2.10 (1.80 to 2.39)	131	1.96 (1.72 to 2.21)	0.042	0.339
White-cell Count (x10^3/µL)	101	2.17 (1.67 to 2.66)	103	1.97 (1.65 to 2.29)	102	2.49 (0.61 to 4.37)	0.874	0.226
Platelets (x10^3/µL)	99	103.3 (84.1 to 122.4)	102	92.6 (77.6 to 107.5)	100	118.4 (102.9 to 134.0)	0.084	0.360
AST, (U/L)	130	4.19 (-2.96 to 11.3)	131	7.99 (0.63 to 15.3)	132	8.75 (2.12 to 15.4)	0.532	0.383
ALT, (U/L)	130	7.82 (3.87 to 11.8)	132	19.9 (14.2 to 25.6)	132	14.6 (9.07 to 20.2)	0.004	0.001
Bilirubin, (umol/L)	126	-0.80 (-1.93 to 0.33)	126	-0.18 (-1.11 to 0.76)	128	-1.42 (-2.33 to -0.52)	0.095	0.233
BUN, (mmol/L)	98	0.55 (-1.18 to 2.28)	102	0.50 (-0.10 to 1.10)	101	-1.06 (-1.65 to -0.47)	0.103	0.974
Creatinine (umol/L)	130	-1.24 (-6.44 to 3.95)	132	0.54 (-3.46 to 4.54)	132	-3.19 (-6.59 to 0.22)	0.360	0.712
Amylase, (micromol/L)	20	133.5(22.3 to 244.6)	21	8.42 (-13.1 to 30.0)	19	58.7 (27.4 to 90.0)	0.026	0.027
Alkaline Phosphatase (U/L)	116	75.5 (23.5 to 127.4)	117	90.2 (44.7 to 135.7)	118	106.4 (76.9 to 135.8)	0.793	0.578

SD = standard deviation, BP = blood pressure

A negative value indicates a decrease in end of treatment values, on average, compared to baseline

* p-value from ANCOVA between arms in end of treatment measurements, adjusting for baseline values and centre

† p-value from ANCOVA between SSG and PM in end of treatment measurements, adjusting for baseline values and centre

Table 33 Summary: Comparison of mean changes in biological parameters

Results for mean changes within and between arms	Baseline to Day 7	Day 7 to 14	Baseline to Day 14	Day 14 to End of Treatment
Significant reduction in all arms, on average<br>Evidence of difference in reduction between arms	Temperature<br>Spleen Size	Spleen Size<br>Liver Size	Spleen Size<br>(smaller for PM)	Spleen Size
Significant reduction in all arms, on average<br>No or little evidence of difference in reduction between arms	Heart Rate<br>Liver Size	Temperature<br>Heart Rate	Temperature<br>Heart Rate, Liver Size	Liver Size
Significant increase in all arms, on average<br>Evidence of difference in increase between arms	AST, ALT<br>Alkaline Phosphatase		HB, Platelets	Weight, HB
Significant increase in all arms, on average<br>No or little evidence of difference in increase between arms	Weight	Weight, Platelets<br>HB, WBC	Weight, WBC, AST,<br>ALT, Alk. Phosphatase	Platelets
Significant increase in parameter at least one arm*<br>Evidence of a difference in average change between arms	Platelets, Amylase<br>(SSG Comb)	Alk. Phosphatase (PM)<br>(between PM & SSG)		
Significant increase in parameter at least one arm*<br>No or little evidence of a difference between arms	HB (Comb)<br>WBC (Comb)	ALT (PM)<br>BUN (PM)	Amylase (SSG)	WBC (SSG PM)<br>Bilirubin (PM)
Significant reduction in parameter at least one arm*<br>Evidence of a difference in average change between arms		AST (SSG & Comb)	Bilirubin (Comb)	ALT (SSG)
Significant reduction in parameter at least one arm*<br>No or little evidence of a difference between arms	Bilirubin (Comb)		Bilirubin (Comb)<br>Creatinine (SSG)	Heart Rate (PM<br>Comb)
No significant change in parameter in any arm<br>No evidence of a difference between arms	BUN<br>Creatinine	Bilirubin<br>Creatinine, Amylase		Temperature, AST,<br>BUN, Creatinine,<br>Alk. Phosphatase

HB = Haemoglobin, WBC = White Blood Cell Count

Significant changes (reduction or increase) in parameter, on average, within arms assumed where 95% CI around mean difference excludes zero.

Due to amount of comparisons made, evidence of statistical significance of variation in mean change across arms was assumed where $p < 0.01$

* arm in which increase or reductions occurs is given in parentheses

Table 34 Summary: Change from Baseline to End of Treatment

Results for mean changes within and between arms	Change from Baseline to End of Treatment
Significant reduction in all arms, on average<br>Evidence of difference in reduction between arms	Spleen Size (smaller reduction PM)
Significant reduction in all arms, on average<br>No or little evidence of difference in reduction between arms	Temperature, liver size
Significant increase in all arms, on average<br>Evidence of difference in increase between arms	Weight, heart rate
Significant increase in all arms, on average<br>No or little evidence of difference in increase between arms	Platelets, AST, ALT, Alkaline Phosphatase
Significant increase in parameter at least one arm*<br>Evidence of a difference in average change between arms	
Significant increase in parameter at least one arm*<br>No or little evidence of a difference between arms	HB (Comb), Bilirubin (PM, Comb), Amylase (SSG, Comb), WBC (Comb)
Significant reduction in parameter at least one arm*<br>Evidence of a difference in average change between arms	
Significant reduction in parameter at least one arm*<br>No or little evidence of a difference between arms	BUN (Comb)
No significant change in parameter in any arm<br>No evidence of a difference between arms	Creatinine

HB = Haemoglobin, WBC = White Blood Cell Count

Significant changes (reduction or increase) in parameter, on average, within arms assumed where 95% CI around mean difference excludes zero.

Due to amount of comparisons made, evidence of statistical significance from ANCOVA of variation in mean change across arms was assumed where $p < 0.01$

* arm in which increase or reductions occurs is given in parentheses

10.8 Serious Adverse Events (SAEs) and Non-Serious Adverse Events (AEs)

Table 35 Number of patients experiencing adverse events

		SSG	PM	Comb	Total
Number Randomised		135	135	135	405
Number of patients with SAE*:					
	Total	8 (5.9%)	5 (3.7%)	3 (2.2%)	16
	Treatment Emergent†	7 (5.2%)	3 (2.2%)	3 (2.2%)	13
	During Follow-up	1 (0.7%)	2 (1.4%)	0 (0)	3
	Adverse drug reaction‡	5 (3.7%)	3 (2.2%)	2 (1.4%)	10
	Unrelated to study drug§	3 (2.2%)	2 (1.4%)	1 (0.7%)	3
Patients with at least one AE:					
	At any time	99 (73.3%)	77 (57.0%)	93 (68.9%)	269
	Treatment Emergent†	90 (66.7%)	65 (48.2%)	75 (55.6%)	230
	During Follow-up	35 (25.9%)	33 (24.4%)	42 (31.1%)	110

SAE = Serious Adverse Event, AE = Non-serious adverse event

* no patients experienced more than one SAE

† Treatment emergent defined as onset being between day 1 of treatment and 30 days post end of treatment, inclusive. AEs occurring during follow-up had onset recorded as between day 31 of trial and end of study.

‡ Adverse drug reaction recorded as unlikely, possible or probable relation to study drug.

§ Adverse drug reaction recorded as Not Related to study drug

Table 36 Number of adverse events

		Total	SSG	PM	Comb
Number of AE*:					
	Total	566	217 (38.3%)	168 (29.7%)	181 (32.0%)
	Treatment Emergent	411	169 (41.1%)	112 (27.3%)	130 (31.6%)
	During Follow-up	155	48 (31.0%)	56 (36.1%)	51 (32.9%)
Number of AEs per patient:					
Median			1	1	1
Range			0 - 8	0 - 8	0 – 6

SAE = Serious Adverse Event, AE = Non-serious adverse event, IQR = Inter-quartile range

* not all patients experienced an AE whereas some patients experienced more than one AE

After adjustment for centre using Poisson regression (Table 36), there was weak evidence of a lower rate of treatment emergent AE between PM and SSG (p=0.041), but no evidence of a difference in rates between Combination and SSG arms (p=0.876). Tere was strong evidence (p<0.001) of heterogeneity in the rate of treatment emergent AE between study centres, after adjustment for treatment allocation, and strong evidence of a treatment-centre interaction (p<0.001). Rate ratios (RR) comparing PM to SSG and also Combination to SSG are presented by centre to demonstrate this (Table 37).

Across centres, the rate of treatment emergent AE in the PM arm, compared to SSG, was approximately equivalent in Arba Minch. In all other sites, the rate appeared to be lower but there was only weak statistical evidence to support this in Gondar.

In all centres except Gondar, the rate of treatment emergent AE in the Combination arm, was also estimated to be lower compared to SSG, with strong statistical evidence of a reduced rate in Um el Kher. In Gondar, the treatment emergent AE rate in the Combination arm was significantly higher than in the SSG arm (p=0.007), an opposite effect to that observed at all other sites.

10.8.1 Treatment Emergent AEs: Summary Results

Table 37 Treatment Emergent Adverse Event Rate Ratios

	Total person-days on treatment (T) *			Number of events*			PM vs SSG		Comb vs SSG	
	SSG	PM	Comb	SSG	PM	Comb	Adjusted Rate Ratio (95% CI)	p-value‡	Adjusted Rate Ratio† (95% CI)	p-value‡
**Non-Serious adverse event: Treatment Emergent**										
All	8100	6885	6345	169	112	130	0.78 (0.61 to 0.99)†	0.041	0.98 (0.78 to 1.23)†	0.876
Gondar	2700	2295	2115	62	35	77	0.66 (0.44 to 1.00)	0.053	1.59 (1.13 to 2.22)	0.007
Arba Minch	1800	1530	1410	25	22	14	1.03 (0.58 to 1.84)	0.906	0.71 (0.37 to 1.38)	0.315
Kenya	900	765	705	29	23	15	0.93 (0.54 to 1.61)	0.804	0.66 (0.35 to 1.23)	0.192
Um el Kher	1800	1530	1410	36	24	11	0.78 (0.47 to 1.31)	0.357	0.39 (0.20 to 0.77)	0.006
Kassab	900	765	705	17	8	13	0.55 (0.24 to 1.28)	0.168	0.98 (0.47 to 2.01)	0.948

SSG is the reference arm used in all analyses. Rate Ratio (RR) < 1 suggests a reduced rate compared to SSG; RR > 1 suggests an increased rate.

Treatment emergent defined as beginning between day 1 of treatment and 30 days post end of treatment, inclusive.

Patients recruited: Gondar 135, Arba Minch 90, Kenya 45, Um el Kher 90, Kassab 45

* some patients experienced more than 1 AE

† rate ratio adjusted for centre

‡ Wald p-value from Poisson regression model

10.8.2 Serious Adverse Events: Listing

Table 38 Serious Adverse Events, by treatment and relation to study drug

System Organ Class and Preferred MedDRA Term	SSG NR	SSG ADR	PM NR	PM ADR	Comb NR	Comb ADR	Total
**BLOOD AND LYMPHATIC DISORDERS**	**0**	**0**	**0**	**1**	**0**	**0**	**1**
ANAEMIA	0	0	0	1	0	0	1
**CARDIAC DISORDERS**	**0**	**0**	**0**	**1**	**0**	**0**	**1**
CARDIAC FAILURE HIGH OUTPUT	0	0	0	1	0	0	1
**GASTROINTESTINAL DISORDERS**	**1**	**1**	**0**	**0**	**0**	**0**	**2**
PANCREATITIS	0	1	0	0	0	0	1
PERITONEAL HAEMORRHAGE	1	0	0	0	0	0	1
**GENERAL DISORDERS AND ADMINISTRATION SITE CONDITIONS**	**1**	**0**	**1**	**0**	**0**	**0**	**2**
DEATH	1	0	1	0	0	0	2
**INFECTIONS AND INFESTATIONS**	**0**	**1**	**1**	**0**	**1**	**0**	**3**
ABDOMINAL SEPSIS	0	1	1	0	0	0	2
PERICARDITIS MYCOPLASMAL	0	0	0	0	1	0	1
**INVESTIGATIONS**	**0**	**1**	**0**	**1**	**0**	**1**	**3**
BLOOD AMYLASE INCREASED	0	1	0	0	0	0	1
HEPATIC ENZYME INCREASED	0	0	0	0	0	1	1
PLATELET COUNT DECREASED	0	0	0	1	0	0	1
**RENAL AND URINARY DISORDERS**	**0**	**2**	**0**	**0**	**0**	**1**	**3**
RENAL FAILURE	0	0	0	0	0	1	1
RENAL FAILURE ACUTE	0	2	0	0	0	0	2
**RESPIRATORY, THORACIC AND MEDIASTINAL DISORDERS**	**1**	**0**	**0**	**0**	**0**	**0**	**1**
EPISTAXIS	1	0	0	0	0	0	1

NR = Not Related to study drug, as recorded by investigator

ADR = Adverse drug reaction, recorded as Probable, Possible or Unlikely relation to study drug by investigator

Total number of AE, by System Organ Class, highlighted in bold type

Table 39 Deaths during Treatment and Follow-up

Arm	Time of death*	Cause of death	System Organ Class†	Relation to Study Drug
SSG	87	Not clearly known	General disorders‡	Not related
SSG	34	Acute renal failure	Renal & urinary disorders	Possible
SSG	12	Acute renal failure	Renal & urinary disorders	Probable
PM	154	Sepsis secondary to gastroenteritis	General disorders‡	Not related
PM	183	Sepsis & immune reconstitution	Infections & infestations	Not related
Comb	88	Pericarditis due to TB	Infections & infestations	Not related

* time: trial day that death occurred, calculated assuming day 1 of trial is day 1 of treatment

† classified according to MedDRA, version 10.0

‡ full description: General disorders and administrative site conditions

10.8.3 Non-Serious Treatment Emergent Adverse Events: Listing

In the tables below,

- NR = Not Related to study drug, as recorded by investigator, ADR = Adverse drug reaction, recorded as Probable, Possible or Unlikely relation to study drug by investigator
- Data are number of AE, by relation to study drug and treatment [number of patients experiencing AE, by relation to study drug and treatment]
- Totals are for each system organ class and preferred MedDRA term. Totals in bold type correspond to overall totals for each system organ class

Table 40 Treatment Emergent Non- Serious AEs

Non-Serious Adverse Events	SSG		PM		Comb		Total
	NR	ADR	NR	ADR	NR	ADR	
**BLOOD AND LYMPHATIC DISORDERS**	**1**	**2**	**0**	**0**	**0**	**0**	**3**
ANAEMIA	0	1	0	0	0	0	1
LYMPHADENITIS	1	0	0	0	0	0	1
THROMBOCYTOPENIA	0	1	0	0	0	0	1
**CARDIAC DISORDERS**	**0**	**0**	**0**	**0**	**0**	**0**	**0**
**EAR AND LABYRINTH DISORDERS**	**0**	**0**	**1**	**0**	**0**	**0**	**1**
DEAFNESS	0	0	0	0	0	0	0
EAR PAIN	0	0	1	0	0	0	1
**ENDOCRINE DISORDERS**	**1**	**0**	**0**	**0**	**0**	**0**	**1**
HYPOGLYCAEMIA	1	0	0	0	0	0	1
**EYE DISORDERS**	**5**	**0**	**0**	**0**	**1**	**1**	**7**
CONJUNCTIVITIS	2	0	0	0	1	1	4
CONJUNCTIVITIS ALLERGIC	1	0	0	0	0	0	1
CONJUNCTIVITIS INFECTIVE	1	0	0	0	0	0	1
EYE PAIN	1	0	0	0	0	0	1
**GASTROINTESTINAL DISORDERS**	**7**	**6**	**7**	**7 [6]**	**4**	**11**	**42**
ABDOMINAL DISTENSION	0	1	0	0	0	0	1
ABDOMINAL PAIN	1	1	1	2	0	1	6
ABDOMINAL PAIN UPPER	0	1	0	0	1	0	2
ASCITES	0	0	1	0	0	0	1
CHEILOSIS	0	1	0	0	0	0	1
DIARRHOEA	1	0	3	1	1	1	7
DYSPEPSIA	1	0	0	1	1	3	6
GASTRITIS	0	0	0	1	0	1	2
GINGIVAL BLEEDING	0	1	0	0	0	0	1
GINGIVITIS	1	0	0	0	0	0	1
MOUTH ULCERATION	1	0	0	0	0	0	1
MUCOUS STOOLS	1	0	0	0	0	0	1
NAUSEA	0	1	0	0	0	0	1
ORAL SOFT TISSUE DISORDER	0	0	0	0	0	1	1
PERITONITIS	0	0	1	0	0	0	1
VOMITING	1	0	1	2 [1]	1	4	9

Non-Serious Adverse Events (cont.)	SSG		PM		Comb		Total
	NR	ADR	NR	ADR	NR	ADR	
**GENERAL DISORDERS AND ADMINISTRATION SITE CONDITIONS**	**4**	**13**	**3**	**20 [19]**	**0**	**35**	**75 [74]**
CHEST PAIN	1	0	0	0	0	0	1
CHILLS	1	0	0	1	0	0	2
INJECTION SITE PAIN	0	12	1	16	0	34	63
INJECTION SITE SWELLING	0	0	0	0	0	1	1
PERIPHERAL OEDEMA	1	0	0	0	0	0	1
PYREXIA	0	1	2	2 [1]	0	0	5
SWELLING	1	0	0	0	0	0	1
TENDERNESS	0	0	0	1	0	0	1
**IMMUNE SYSTEM DISORDERS**	**1**	**0**	**0**	**0**	**0**	**0**	**1**
ALLERGY TO ARTHROPOD BITE	1	0	0	0	0	0	1
**INFECTIONS AND INFESTATIONS**	**46 [44]**	**14**	**25**	**8**	**20**	**8**	**121 [119]**
AMOEBIASIS	1	0	1	1	1	0	4
AMOEBIC DYSENTERY	0	0	0	0	1	0	1
ASCARIASIS	0	0	2	0	0	0	2
CELLULITIS	1	0	0	0	0	1	2
CROUP INFECTIOUS	1	0	0	0	0	0	1
DYSENTRY	0	1	0	0	0	0	1
FUNGAL SKIN INFECTION	0	0	0	1	0	0	1
GASTROENTERITIS	0	1	1	2	1	0	5
GIARDIASIS	3	0	0	1	1	0	5
HERPES ZOSTER	1	0	0	0	0	0	1
HOOKWORM INFECTION	1	0	0	0	1	1	3
INJECTION SITE CELLULITIS	0	0	0	0	0	3	3
LARYNGITIS	1	0	0	0	0	0	1
MALARIA	11 [10]	0	10	0	5	0	26
MOLLUSCUM CONTAGIOSUM	0	0	0	0	1	0	1
NASOPHARYNGITIS	6	0	1	0	1	0	8
OTITIS MEDIA	2	0	1	1	0	1	5
OTITIS MEDIA ACUTE	1	0	1	0	0	0	2
PNEUMONIA	9 [8]	2	4	0	1	1	17
PNEUMONIA PRIMARY ATYPICAL	1	1	0	0	1	0	3
STRONGYLOIDIASIS	0	0	0	0	1	0	1
TAENIASIS	1	0	0	0	0	0	1
UPPER RESPIRATORY TRACT INFECTION	2	0	1	0	4	0	7
URINARY TRACT INFECTION	3	4	3	1	1	0	12
VARICELLA	1	0	0	0	0	0	1
VISCERAL LEISHMANIASIS	0	5	0	1	0	1	7

Non-Serious Adverse Events (cont.)	SSG		PM		Comb		Total
	NR	ADR	NR	ADR	NR	ADR	
**INJURY, POISONING AND PROCEDURAL**	**1**	**0**	**2**	**0**	**0**	**0**	**3**
CONTUSION	0	0	1	0	0	0	1
RADIUS FRACTURE	1	0	0	0	0	0	1
SCRATCH	0	0	1	0	0	0	1
**INVESTIGATIONS**	**0**	**43 [42]**	**0**	**24**	**1**	**37**	**105 [104]**
ALANINE AMINOTRANSFERASE INCREASED	0	7	0	3	0	3	13
ASPARTATE AMINOTRANSFERASE INCREASED	0	13	0	7	0	15	35
AUDIOGRAM ABNORMAL	0	0	0	1	0	1	2
BLOOD ALKALINE PHOSPHATASE INCREASED	0	7	0	5	0	6	18
BLOOD AMYLASE INCREASED	0	4	0	0	0	2	6
BLOOD CREATININE INCREASED	0	1	0	3	0	1	5
ELECTROCARDIOGRAM CHANGE	0	1	0	0	0	0	1
ELECTROCARDIOGRAM QT PROLONGED	0	2 [1]	0	0	0	0	2
HEPATIC ENZYMES INCREASED	0	2	0	0	0	2	4
PLATELET COUNT DECREASED	0	1	0	0	0	1	2
TRANSAMINASES INCREASED	0	5	0	4	0	6	15
WEIGHT DECREASED	0	0	0	1	0	0	1
WHITE BLOOD CELLS URINE POSITIVE	0	0	0	0	1	0	1
**METABOLISM AND NUTRITION DISORDERS**	**0**	**0**	**0**	**0**	**0**	**1**	**1**
ANOREXIA	0	0	0	0	0	1	1
**MUSCULOSKELETAL AND CONNECTIVE TISSUE DISORDERS**	**2**	**2**	**1**	**2**	**0**	**0**	**7**
ARTHRALGIA	0	1	0	1	0	0	2
BACK PAIN	1	1	1	1	0	0	4
NECK PAIN	1	0	0	0	0	0	1
**NERVOUS SYSTEM DISORDORS**	**3**	**2**	**0**	**5**	**1**	**0**	**11**
BURNING SENSATION	0	1	0	0	0	0	1
HEADACHE	3	1	0	4	1	0	9
NEUROPATHY PERIPHERAL	0	0	0	1	0	0	1
**PSYCHIATRIC DISORDERS**	**0**	**0**	**0**	**0**	**1**	**0**	**1**
INSOMINIA	0	0	0	0	1	0	1
**RENAL AND URINARY DISORDERS**	**0**	**0**	**0**	**1**	**1**	**0**	**2**
ALBUMINURIA	0	0	0	1	0	0	1
HAEMATURIA	0	0	0	0	1	0	1

Non-Serious Adverse Events (cont.)	SSG		PM		Comb		Total
	NR	ADR	NR	ADR	NR	ADR	
**RESPIRATORY, THORACIC AND MEDIASTINAL DISORDERS**	**3 [1]**	**7 [6]**	**1**	**2**	**0**	**6 [5]**	**19 [12]**
ALLERGIC BRONCHITIS	0	1	0	0	0	0	1
ASTHMA	0	0	0	0	0	3 [2]	3
COUGH	0	0	1	0	0	1	2
EPISTAXIS	3 [1]	6 [5]	0	2	0	2	13
**SKIN AND SUBCUTANEOUS TISSUE DISORDERS**	**0**	**6**	**1**	**1**	**2**	**0**	**10**
ACNE	0	1	0	0	0	0	1
PRURITUS	0	1	0	0	0	0	1
RASH	0	2	0	1	0	0	3
RASH MACULO-PAPULAR	0	1	0	0	0	0	1
RASH PAPULAR	0	1	0	0	0	0	1
RASH VESICULAR	0	0	0	0	1	0	1
SKIN LESION	0	0	0	0	1	0	1
SWELLING FACE	0	0	1	0	0	0	1
**VASCULAR DISORDERS**	**0**	**0**	**0**	**1**	**0**	**0**	**1**
HYPERTENSION	0	0	0	1	0	0	1

10.8.4 All Non-Serious Adverse Events: Listing

Table 41 All Non- Serious AEs, by treatment and relation to study drug

Non-Serious Adverse Events	SSG		PM		Comb		Total
	NR	ADR	NR	ADR	NR	ADR	
**BLOOD AND LYMPHATIC DISORDERS**	**2**	**2**	**1**	**1**	**0**	**0**	**6**
ANAEMIA	0	1	0	0	0	0	1
EOSINOPHILIA	0	0	1	0	0	0	1
LEUKOCYTOSIS	0	0	0	1	0	0	1
LYMPHADENITIS	1	0	0	0	0	0	1
THROMBOCYTOPENIA	1	1	0	0	0	0	2
**CARDIAC DISORDERS**	**0**	**2**	**0**	**2**	**0**	**0**	**4**
BRADYCARDIA	0	2	0	1	0	0	3
SINUS ARRHYTHMIA	0	0	0	1	0	0	1
**EAR AND LABYRINTH DISORDERS**	**0**	**0**	**1**	**0**	**0**	**1**	**2**
DEAFNESS	0	0	0	0	0	1	0
EAR PAIN	0	0	1	0	0	0	0
**ENDOCRINE DISORDERS**	**1**	**0**	**0**	**0**	**0**	**0**	**1**
HYPOGLYCAEMIA	1	0	0	0	0	0	1
**EYE DISORDERS**	**7**	**0**	**2**	**0**	**3**	**1**	**13**
CONJUNCTIVITIS	4	0	2	0	3	1	10
CONJUNCTIVITIS ALLERGIC	1	0	0	0	0	0	1
CONJUNCTIVITIS INFECTIVE	1	0	0	0	0	0	1
EYE PAIN	1	0	0	0	0	0	1

Non-Serious Adverse Events (cont.)	SSG		PM		Comb		Total
	NR	ADR	NR	ADR	NR	ADR	
**GASTROINTESTINAL DISORDERS**	**9**	**7**	**10**	**8**	**6**	**11**	**51**
ABDOMINAL DISTENSION	0	1	0	0	0	0	1
ABDOMINAL PAIN	1	1	1	2	0	1	6
ABDOMINAL PAIN UPPER	2	1	0	0	1	0	4
ASCITES	0	0	1	0	0	0	1
CHEILOSIS	0	1	0	0	0	0	1
DENTAL CARIES	0	0	1	0	0	0	1
DIARRHOEA	1	0	3	1	1	1	7
DOUDENAL ULCER	0	0	1	0	0	0	1
DYSPEPSIA	1	1	0	2	3	3	10
GASTRITIS	0	0	0	1	0	1	2
GINGIVAL BLEEDING	0	1	0	0	0	0	1
GINGIVITIS	1	0	0	0	0	0	1
MOUTH ULCERATION	1	0	0	0	0	0	1
MUCOUS STOOLS	1	0	0	0	0	0	1
NAUSEA	0	1	0	0	0	0	1
ORAL SOFT TISSUE DISORDER	0	0	0	0	0	1	1
PERITONITIS	0	0	1	0	0	0	1
VARICES OESOPHAGEAL	0	0	1	0	0	0	1
VOMITING	1	0	1	2	1	4	9
**GENERAL DISORDERS AND ADMINISTRATION SITE CONDITIONS**	**5**	**13**	**3**	**21**	**1**	**37**	**80**
CHEST PAIN	1	0	0	0	0	0	1
CHILLS	1	0	0	1	0	0	2
INJECTION SITE PAIN	0	12	1	16	0	34	63
INJECTION SITE SWELLING	0	0	0	0	0	1	1
MALAISE	0	0	0	0	0	1	1
PERIPHERAL OEDEMA	1	0	0	0	0	0	1
PYREXIA	1	1	2	3	1	1	9
SWELLING	1	0	0	0	0	0	1
TENDERNESS	0	0	0	1	0	0	1
**IMMUNE SYSTEM DISORDERS**	**1**	**0**	**0**	**0**	**0**	**0**	**1**
ALLERGY TO ARTHROPOD BITE	1	0	0	0	0	0	1

**Non-Serious Adverse Events (cont.)**	**SSG**		**PM**		**Comb**		**Total**
	**NR**	**ADR**	**NR**	**ADR**	**NR**	**ADR**	
**INFECTIONS AND INFESTATIONS**	**58**	**29**	**40**	**10**	**32**	**15**	**184**
ACARODERMATITIS	0	0	0	0	1	0	1
AMOEBIASIS	1	0	1	1	2	0	5
AMOEBIC DYSENTERY	0	0	1	0	1	0	2
ASCARIASIS	0	0	2	0	0	0	2
BODY TINEA	0	0	1	0	0	0	1
CELLULITIS	1	0	0	0	0	1	2
CHRONIC SINUSITIS	0	0	1	0	0	0	1
CROUP INFECTIOUS	1	0	0	0	0	0	1
DYSENTRY	0	1	2	0	0	0	3
EAR INFECTION	0	0	0	0	1	0	1
FUNGAL SKIN INFECTION	0	0	0	1	0	0	1
GASTROENTERITIS	0	1	1	2	1	0	5
GIARDIASIS	3	0	0	1	2	0	6
HERPES ZOSTER	1	0	0	0	0	0	1
HOOKWORM INFECTION	1	0	0	0	1	1	3
INFECTION PARASITIC	1	0	0	0	1	0	2
INJECTION SITE CELLULITIS	0	0	0	0	0	3	3
LARYNGITIS	1	0	0	0	0	0	1
MALARIA	15	0	13	0	8	0	36
MOLLUSCUM CONTAGIOSUM	0	0	0	0	1	0	1
NASOPHARYNGITIS	7	0	1	0	3	0	11
OTITIS MEDIA	3	0	2	1	0	1	7
OTITIS MEDIA ACUTE	1	0	1	0	0	0	2
PNEUMONIA	11	2	5	1	2	2	23
PNEUMONIA PRIMARY ATYPICAL	1	1	1	0	1	0	4
SEPSIS	0	0	1	0	0	0	1
STRONGYLOIDIASIS	0	0	0	0	1	0	1
TAENIASIS	1	0	0	0	0	0	1
TUBERCULOSIS	1	0	0	0	0	0	1
UPPER RESPIRATORY TRACT INFECTION	4	0	2	0	5	0	11
URINARY TRACT INFECTION	3	4	4	2	1	0	14
VARICELLA	1	0	0	0	0	0	1
VISCERAL LEISHMANIASIS	0	20	0	1	0	7	28
WOUND SEPSIS	0	0	1	0	0	0	1

Non-Serious Adverse Events (cont.)	SSG		PM		Comb		Total
	NR	ADR	NR	ADR	NR	ADR	
**INJURY, POISONING AND PROCEDURAL**	**2**	**0**	**3**	**0**	**2**	**0**	**7**
CONTUSION	1	0	1	0	1	0	3
LIMB INJURY	0	0	1	0	0	0	1
RADIUS FRACTURE	1	0	0	0	0	0	1
SCRATCH	0	0	1	0	0	0	1
SKIN LACERATION	0	0	0	0	1	0	1
**INVESTIGATIONS**	**2**	**48**	**4**	**40**	**5**	**47**	**146**
ALANINE AMINOTRANSFERASE INCREASED	1	7	1	8	1	4	22
ASPARTATE AMINOTRANSFERASE INCREASED	0	14	2	11	1	17	45
AUDIOGRAM ABNORMAL	0	0	0	3	0	3	6
BLOOD ALKALINE PHOSPHATASE INCREASED	0	7	1	6	0	8	22
BLOOD AMYLASE INCREASED	1	4	0	0	0	2	7
BLOOD CREATININE INCREASED	0	2	0	4	0	2	8
ELECTROCARDIOGRAM CHANGE	0	1	0	0	0	0	1
ELECTROCARDIOGRAM QT PROLONGED	0	2	0	0	0	0	2
HAEMAGLOBIN DECREASED	0	1	0	0	1	0	2
HEPATIC ENZYMES INCREASED	0	2	0	0	0	2	4
PLATELET COUNT DECREASED	0	2	0	0	1	2	5
TRANSAMINASES INCREASED	0	5	0	7	0	6	18
WEIGHT DECREASED	0	0	0	1	0	0	1
WHITE BLOOD CELL DECREASED	0	1	0	0	0	1	2
WHITE BLOOD CELLS URINE POSITIVE	0	0	0	0	1	0	1
**METABOLISM AND NUTRITION DISORDERS**	**1**	**0**	**0**	**0**	**0**	**1**	**2**
ANOREXIA	1	0	0	0	0	1	2
**MUSCULOSKELETAL AND CONNECTIVE TISSUE DISORDERS**	**2**	**2**	**1**	**2**	**1**	**1**	**9**
ARTHRALGIA	0	1	0	1	0	0	2
BACK PAIN	1	1	1	1	1	0	5
MUSCULOSKELETAL PAIN	0	0	0	0	0	1	1
NECK PAIN	1	0	0	0	0	0	1

Non-Serious Adverse Events (cont.)	SSG		PM		Comb		Total
	NR	ADR	NR	ADR	NR	ADR	
**NERVOUS SYSTEM DISORDORS**	**4**	**2**	**1**	**5**	**2**	**0**	**14**
BURNING SENSATION	0	1	0	0	0	0	1
HEADACHE	4	1	1	4	2	0	12
NEUROPATHY PERIPHERAL	0	0	0	1	0	0	1
**PSYCHIATRIC DISORDERS**	**0**	**0**	**0**	**0**	**1**	**0**	**1**
INSOMINIA	0	0	0	0	1	0	1
**RENAL AND URINARY DISORDERS**	**0**	**0**	**1**	**4**	**1**	**0**	**6**
ALBUMINURIA	0	0	0	1	0	0	1
HAEMATURIA	0	0	1	3	1	0	5
**RESPIRATORY, THORACIC AND MEDIASTINAL DISORDERS**	**3**	**7**	**3**	**2**	**1**	**7**	**23**
ALLERGIC BRONCHITIS	0	1	0	0	0	0	1
ASTHMA	0	0	1	0	0	3	4
COUGH	0	0	2	0	0	1	3
EPISTAXIS	3	6	0	2	1	3	15
**SKIN AND SUBCUTANEOUS TISSUE DISORDERS**	**1**	**7**	**1**	**1**	**3**	**0**	**13**
ACNE	0	1	0	0	1	0	2
PRURITUS	0	1	0	0	0	0	1
RASH	0	2	0	1	0	0	3
RASH MACULO-PAPULAR	0	1	0	0	0	0	1
RASH PAPULAR	0	1	0	0	0	0	1
RASH VESICULAR	0	0	0	0	1	0	1
SKIN LESION	0	1	0	0	1	0	2
SKIN ULCER	1	0	0	0	0	0	1
SWELLING FACE	0	0	1	0	0	0	1
**VASCULAR DISORDERS**	**0**	**0**	**0**	**1**	**1**	**0**	**2**
HYPERTENSION	0	0	0	1	1	0	2

10.9 Subgroup Analyses

10.9.1 HIV positive patients

The overall prevalence of HIV was 4.0% (16 / 405). In adults, the prevalence was 6.5% (13 / 201) and in children, 1.5% (3 / 204). Given the number of patients not tested, prevalence could be underestimated.

Table 42 Number of adults and children tested for HIV at each centre

Centre	Adults: 15 - 60 years		Children: 4 to 14 years		Total	
	**N**	**Tested**	**N**	**Tested**	**N**	**Tested**
**Gondar**	94	91	41	39	135	130
**Arba Minch**	47	47	43	43	90	90
**Kenya**	20	0	25	0	45	0
**Um el Kher**	22	12	68	0	90	12
**Kassab**	18	18	27	27	45	45
**Total**	201	168 (83.6%)	204	109 (53.4%)	405	277 (68.4%)

Table 43 Parasitology Data for HIV positive patients by Treatment

Parasitology result			Rescue given: trial day
**End of Treatment: TOC Result**	**3 month follow-up**	**6 month follow-up: DC Result**	
**SSG**			
Negative: TOC Success	Not indicated	Negative: DC Success	No
Negative: TOC Success	Negative	Negative: DC Success	No
Negative: TOC Success	Negative	Negative: DC Success	No
Missing: TOC Failure	Not indicated	Negative: DC Failure	Yes: day 26
**PM**			
Positive: TOC Failure	Positive	Died*: DC Failure	Yes: day 138
Positive: TOC Failure	Not seen	LTFU: DC Missing	No
Positive: TOC Failure	Negative	Died†: DC Missing	No
Negative: TOC Success	Negative	Negative: DC Success	No
Positive: TOC Failure	Not seen	LTFU: DC Failure	Yes: day 22
Positive: TOC Failure	Positive	Positive: DC Failure	Yes: day 25
**Combination**			
Negative: TOC Success	Not indicated	Negative: DC Success	No
Negative: TOC Success	Not indicated	Positive: DC Failure	Yes: day 190
Negative: TOC Success	Negative	Negative: DC Success	No
Positive: TOC Failure	Positive	Positive: DC Failure	Yes: day 21
Positive: TOC Failure	Not seen	Positive: DC Failure	Yes: day 178‡
Negative: TOC Success	Not indicated	Negative: DC Success	No

Not indicated at 3 months = deemed clinically well by investigator

Note that approximate trial day of 6m follow-up would be day 211 for SSG patients, day 202 for PM patients & day 198 for Combination patients; calculated as days on treatment plus one plus 180 days.

TOC = Test of Cure, DC = Definitive Cure

* Cause of death: Sepsis secondary to gastroenteritis

† Cause of death: Sepsis & immune reconstitution

‡ Rescue medication given outside the trial by MSF Holland: 15 days of SSG plus 6 doses of Ambisome.

10.9.2 Post Kala-azar Dermal Leishmaniasis

In total, 28 patients developed PKDL during the trial; 20 patients allocated to SSG, 1 allocated to PM and 7 allocated to Combination treatment.

In the SSG patients who developed PKDL, Ambisome rescue medication was given to 2 patients for the VL infection and SSG was administered during follow-up to treat the PKDL for an additional 2 patients. The primary outcome at 6 months for the 2 patients receiving additional treatment with SSG was missing due to LTFU. Both patients have parasitological data for 3 months follow-up and parasites were detected in one of the two patients.

The PM patient who developed PKDL was given Ambisome during follow-up.

References

Valerii Fedorov and Byron Jones. The design of multicentre trials. Statistical Methods in Medical Research 2005; 14: 205-248

David Moher, Kenneth F Schulz, Douglas G Altman, for the CONSORT Group. The CONSORT statement: revised recommendations for improving the quality of reports of parallel-group randomised trials. Lancet 2001; 357: 1191–94

WHO. Management of Safety Information from Clinical trials: Report CIOMS Working Group IV. 2005. World Health Organisation

John P.A. Ioannidis, MD; Stephen J.W. Evans, MSc; Peter C. Gøtzsche, MD, DrMedSci; Robert T. O'Neill, PhD; Douglas G. Altman, DSc; Kenneth Schulz, PhD; and David Moher, PhD, for the CONSORT Group. Better Reporting of Harms in Randomized Trials: An Extension of the CONSORT Statement. Ann Intern Med. 2004; 141:781-788.

ICH E9: Statistical Principles for Clinical Trials. 1998

Kirkwood & Sterne. Essential Medical Statistics 2nd Edition. 2003. Blackwell Science Ltd.

Needlman RD: Part II Growth and Development. In *Nelson Textbook of Pediatrics*. *Volume 10-13*. 17th edition. Edited by: Behrman RE, Kliegman RM and Jenson HB. Philadelphia, Pennsylvania, Saunders; 2004:31-53.

Medical Dictionary for Regulatory Activities (MedDRA) and Maintenance and Support Services Organization (MSSO) http://meddramsso.com/MSSOWeb/index.htm

16.2.9 Documentation of laboratory methods and quality assurance procedures

Appendix 18 Lab normal ranges

Lab Parameter	Standard units			
	KEMRI	Khassb	Gondar	Arba Minch
Haemoglobin	11-18g/dL	12-18g/dL	12-18g/dL	9.9-15.7g/dL
WBC	4.5-10.5x10^3 /µL	4.6-10.2x10^9 /L	4.6-10.2x10^9 /L	2.6-10.3x10^9 /L
Platelets	150-450x10^3 /µL	140-400x10^9 /L	140-400x10^9 /L	128-434x10^9 /L
ALT / SGPT	0-43U/L	<38U/L	<41U/L	<40U/L
AST / SGOT	0-37U/L	<40U/L	<38U/L	<37U/L
Bilirubin (Total)	0-20µmol/L		<1mg/dl	<1mg/dl
Alkaline Phosphatase	<270		<270U/L	<645U/L
Albumin	32-52g/L	3.5-5g/dL	2.8-4.8g/dL	
Total Protein	60-80g/L	6-8g/dL	4.6-8.7g/dL	6.7-8.7g/dL
Prothrombin time		12-17s		
Creatinine	60-130µmol/L		0.5-1.2mg/dL	0.6-1.1mg/dl
BUN	1.7-9.1mmol/L		4.7-23mg/dL	
Amylase				
Urea			15-45mg/dL	15-45 mg/dL

Appendix 19 List of trial site monitoring visits

UM-EL-KHER

Date of visit	Type of visit	Visited by	Designation
13th -20th November 2004	Initiation visit	Shibru Berhanu	Clinical Monitor
13th -14th December 2004	Monitoring visit	Shibru Berhanu	Clinical Monitor
23rd – 26th January 2005	Monitoring visit	Shibru Berhanu	Clinical Monitor
15th – 17th February 2005	Monitoring visit	Shibru Berhanu	Clinical Monitor
15th -16th March 2005	Monitoring visit	Shibru Berhanu	Clinical Monitor
11th -13th June 2005	Close out visits	Shibru Berhanu	Clinical Monitor
28th -30th June 2005	Close out visits	Shibru Berhanu	Clinical Monitor

KASSAB

Date of visit	Type of visit	Visited by	Designation
22nd November 2004	Site Assessment visit	Shibru Berhanu	Clinical Monitor
16th December 2004	Initiation visit	Shibru Berhanu	Clinical Monitor
27th -28th January 2005	Initiation visit	Shibru Berhanu	Clinical Monitor
18th February 2005	Monitoring visit	Shibru Berhanu	Clinical Monitor
18th March 2005	Monitoring visit	Shibru Berhanu	Clinical Monitor
17th -21st April 2005	Monitoring visit	Shibru Berhanu	Clinical Monitor
15th -16th May 2005	Monitoring visit	Shibru Berhanu	Clinical Monitor
9th -11th July 2005	Monitoring visit	Shibru Berhanu	Clinical Monitor
15th -18th September 2005	Monitoring visit	Shibru Berhanu	Clinical Monitor
18th -21st December 2005	Monitoring visit	Rashid Juma	Clinical Trial Manager
11th -15th April 2006	Monitoring visit	Rashid Juma	Clinical Trial Manager
19th-22 May 2006	Monitoring visit	Robert Balikuddembe and Rashid Juma	Clinical Monitor and Clinical Trial Manager
12th -15th August 2006	Monitoring visit	Robert Balikuddembe	Clinical Monitor
31st Oct -3rd Nov 2006	Monitoring visit	Robert Balikuddembe and Dedan Kinoti	Clinical Monitors

KEMRI

Date of visit	Type of visit	Visited by	Designation
29th -31st November 2004	Initiation visit	Shibru Berhanu	Clinical Monitor
17th - 19th January 2005	Initiation visit Follow up	Shibru Berhanu	Clinical Monitor
23rd -26th February 2005	Monitoring visit	Shibru Berhanu	Clinical Monitor
4th -9th April 2005	Monitoring visit	Shibru Berhanu	Clinical Monitor
5th -11th May 2005	Monitoring visit	Shibru Berhanu	Clinical Monitor
2nd-7th June 2005	Monitoring visit	Shibru Berhanu	Clinical Monitor
18th -21st July 2005	Monitoring visit	Shibru Berhanu	Clinical Monitor
31st August – 1st Sept 2005	Monitoring visit	Shibru Berhanu	Clinical Monitor
11th -13th October 2005	Monitoring visit	Shibru Berhanu	Clinical Monitor
26th -28th May 2006	Monitoring visit	Robert Balikuddembe	Clinical Monitor
17th -19th August 2006	Monitoring visit	Robert Balikuddembe	Clinical Monitor
24th – 26th October 2006	Initiation visit LEAP B	Ahmed Bedru	Clinical Monitor
27th -30th November 2006	Monitoring visit	Ahmed Bedru	Clinical Monitor
5th -9th February 2007	Monitoring visit	Ahmed Bedru	Clinical Monitor
21st -24th March 2007	Monitoring visit	Ahmed Bedru	Clinical Monitor
22nd -25th April 2007	Monitoring visit	Ahmed Bedru	Clinical Monitor

ARBA MINCH

Date of visit	Type of visit	Visited by	Designation
9th -10th December 2004	Initiation visit/ Monitoring visit	Hilda O'Hara	Clinical Monitor
25th -28th January 2005	Monitoring visit	Hilda O'Hara	Clinical Monitor
1st -3rd June 2005	Monitoring visit	Hilda O'Hara	Clinical Monitor
4th -8th July 2005	Monitoring visit	Hilda O'Hara	Clinical Monitor
29th -31st August 2005	Monitoring visit	Hilda O'Hara	Clinical Monitor
10th -14th October 2005	Monitoring visit	Hilda O'Hara	Clinical Monitor
26th Feb to 1st March 2006	Monitoring visit	Hilda O'Hara	Clinical Monitor
18th -21st June 2006	Monitoring visit	Hilda O'Hara	Clinical Monitor
13th -16th August 2006	Monitoring visit	Hilda O'Hara	Clinical Monitor
22nd -26th January 2007	Monitoring visit	Isaiah Mwangi	Clinical Monitor
26th Feb -2nd March 2007	Monitoring visit	Isaiah Mwangi	Clinical Monitor
28th -30th March 2007	Monitoring visit	Isaiah Mwangi and Moses Alobo	Clinical Monitor and Clinical Trial Manager
13th-15th May 2007	Monitoring visit	Isaiah Mwangi	Clinical Monitor
10th -13th July2007	Monitoring visit	Isaiah Mwangi	Clinical Monitor
18th -23rd September 2007	Monitoring visit	Isaiah Mwangi	Clinical Monitor
3rd -5th December 2007	Monitoring visit LEAP A and Initiation visit for LEAP B	Isaiah Mwangi and Sally Ellis	Clinical Monitor and Project Coordinator
19th -21st Mach 2008	Monitoring visit	Isaiah Mwangi	Clinical Monitor

GONDAR

Date of visit	Type of visit	Visited by	Designation
14th -16th December 2004	Initiation/ Monitoring visit	Lydia Kivihya-Ndugga	Clinical Monitor
31st Jan – 4th Feb 2005	Initiation/ Monitoring visit	Lydia Kivihya-Ndugga	Clinical Monitor
16th -19th May 2005	Initiation/ Monitoring visit	Lydia Kivihya-Ndugga	Clinical Monitor
3rd-6th July 2005	Monitoring visit	Lydia Kivihya-Ndugga	Clinical Monitor
1st -3rd August 2005	Monitoring visit	Rashid Juma,	Clinical Trial Manager
26th September – 5th October 2005	Monitoring visit	Sarah Nanzigu and Rashid Juma	Clinical Monitor and Clinical Trial Manager
20th -23rd December 2005	Monitoring visit	Sarah Nanzigu	Clinical Monitor
24th -27th January 2006	Monitoring visit	Sarah Nanzigu	Clinical Monitor
28th March -1st April 2006	Monitoring visit	Sarah Nanzigu	Clinical Monitor
2nd -7th July 2006	Monitoring visit	Sarah Nanzigu and Rashid Juma	Clinical Monitor and Clinical Trial Manager
9th -11th of October 2006	Monitoring visit	Rashid Juma	Clinical Monitor
7th -9th of February 2007	Monitoring visit	Sarah Nanzigu	Clinical Monitor
5th -9th March 2007	Monitoring visit	Sarah Nanzigu	Clinical Monitor
8th -11th May 2007	Monitoring visit	Isaiah Mwangi, Moses Alobo and Mona Elfakii	Clinical Monitor, Clinical Trial Manager and Trainee Clinical Monitor
2nd-5th July 2007	Monitoring visit	Sarah Nanzigu	Clinical Monitor
5th -9th October 2007	Monitoring visit	Isaiah Mwangi	Clinical Monitor
4th -5th December 2007	Monitoring visit	Sarah Nanzigu and Moses Alobo	Clinical Monitor and Clinical Trial Manager
17th -19th March 2008	Monitoring visit	Sarah Nanzigu	Clinical Monitor
1st -3rd May 2008	Monitoring visit	Isaiah Mwangi and Mona Elfakii	Clinical Monitor and Trainee Clinical Monitor

Appendix 20 List for GCP trainings and number of attendees

TRAINING

Course	Date	No of attendees	Site
GCP Introductory Course	28–29 Jul 04	5	Addis Ababa, Ethiopia
GCP Training	28–29 Jul 04	17	Addis Ababa, Ethiopia
GCP Introductory Course	30–31 Jul 04	47	Nairobi, Kenya
GCP Training	26–27 Sep 04	23	Khartoum, Sudan
Audiometry Training	6–7 Dec 04	8	Addis Ababa, Ethiopia
Audiometry Training	Dec 04	6	Kassab, Sudan
Audiometry Training	11–12 Jan 05	10	Nairobi, Kenya
GCP Introductory Course	1–2 June 05	13	Arba Minch, Ethiopia
GCP Refresher Course	25–26 Jan 06	15	Gondar, Ethiopia
Laboratory Safety and Refresher Parasitology Course	20 Sep 06	14	Nairobi, Kenya
GCP & Trial Site Audit Preparation Training Course	20 Sep 06	40	Nairobi, Kenya
Audiometry Training	2–3 Nov 06		Khassab, Sudan
Audiometry Training	9–10 Nov 06	2	KEMRI, Kenya
Workshop on Ethics and Good Clinical Practice in Research in Africa	16 March 2007	35	Hotel Africana, Kampala Uganda
GCP Training Course	29 – 30 March 2007	22	Arba Minch Hospital, Ethiopia
From Molecule to Medicine	24 – 25 May 2007	4	CTC Basel, Switzerland
Pharmacovigilance Training Course	11 September 2007	27	The Royal Society of Medicine, London UK

16.1.10 Important publications referenced in the report

Appendix 21 Sundar et al. 2007

LEAP 0104a Appendices

The NEW ENGLAND JOURNAL of MEDICINE

ESTABLISHED IN 1812 JUNE 21, 2007 VOL. 356 NO. 25

Injectable Paromomycin for Visceral Leishmaniasis in India

Shyam Sundar, M.D., T.K. Jha, M.D., Chandreshwar P. Thakur, M.D., Prabhat K. Sinha, M.D., and Sujit K. Bhattacharya, M.D.*

ABSTRACT

BACKGROUND

Visceral leishmaniasis (kala-azar) affects large, rural, resource-poor populations in South Asia, Africa, and Brazil. Safe, effective, and affordable new therapies are needed. We conducted a randomized, controlled, phase 3 open-label study comparing paromomycin, an aminoglycoside, with amphotericin B, the present standard of care in Bihar, India.

METHODS

In four treatment centers for visceral leishmaniasis, 667 patients between 5 and 55 years of age who were negative for the human immunodeficiency virus and had parasitologically confirmed visceral leishmaniasis were randomly assigned in a 3:1 ratio to receive paromomycin (502 patients) at a dose of 11 mg per kilogram of body weight intramuscularly daily for 21 days or amphotericin B (165 patients) at a dose of 1 mg per kilogram intravenously every other day for 30 days. Final cure was assessed 6 months after the end of treatment; safety assessments included daily clinical evaluations and weekly laboratory and audiometric evaluations. Noninferiority testing was used to compare 6-month cure rates, with a chosen margin of noninferiority of 10 percentage points.

RESULTS

Paromomycin was shown to be noninferior to amphotericin B (final cure rate, 94.6% vs. 98.8%; difference, 4.2 percentage points; upper bound of the 97.5% confidence interval, 6.9; P<0.001). Mortality rates in the two groups were less than 1%. Adverse events, which were more common among patients receiving paromomycin than among those receiving amphotericin B (6% vs. 2%, P=0.02), included transient elevation of aspartate aminotransferase levels (>3 times the upper limit of the normal range); transient reversible ototoxicity (2% vs. 0, P=0.20); and injection-site pain (55% vs. 0, P<0.001); and in patients receiving amphotericin B, as compared with those receiving paromomycin, nephrotoxicity (4% vs. 0, P<0.001), fevers (57% vs. 3%), rigors (24% vs. 0, P<0.001), and vomiting (10% vs. <1%, P<0.001).

CONCLUSIONS

Paromomycin was shown to be noninferior to amphotericin B for the treatment of visceral leishmaniasis in India. (ClinicalTrials.gov number, NCT00216346.)

From the Institute of Medical Sciences, Banaras Hindu University, Varanasi, Uttar Pradesh (S.S.); the Kala-azar Research Centre, Brahmpura, Muzaffarpur, Bihar (T.K.J.); Balaji Utthan Sansthan, Patna, Bihar (C.P.T.); and the Rajendra Memorial Research Institute of Medical Sciences, Patna, Bihar (P.K.S., S.K.B.) — all in India. Address reprint requests to Dr. Sundar at the Institute of Medical Sciences, Banaras Hindu University, Varanasi 221005, India, or at drshyamsundar@hotmail.com.

*Additional members of the Paromomycin for Visceral Leishmaniasis Study Teams are listed in the Appendix.

N Engl J Med 2007;356:2571-81.
Copyright © 2007 Massachusetts Medical Society.

Downloaded from www.nejm.org on June 21, 2007 . Copyright © 2007 Massachusetts Medical Society. All rights reserved.

Visceral leishmaniasis (kala-azar) is primarily a fatal vectorborne parasitic disease characterized by fever, hepatosplenomegaly, and pancytopenia. Most of the approximately 500,000 cases of visceral leishmaniasis reported worldwide affect the rural poor in India, Nepal, Bangladesh, Sudan, and Brazil.[1] Treatment options for visceral leishmaniasis are limited. Sodium stibogluconate, a historically effective and affordable pentavalent antimonial compound, is associated with fatal toxic effects,[2-4] and in some regions its use has led to the development of resistant strains of *Leishmania donovani*,[5] with the result that fewer than 50% of treated patients are cured.[2,6-8] In regions where antimony resistance is prevalent, intravenous amphotericin B (desoxycholate) (Fungizone, Sarabhai Piramel Pharmaceuticals) is used, but it is expensive and may require weeks of hospitalization with intensive clinical and laboratory monitoring. Liposomal formulations of amphotericin B (AmBisome, Gilead Sciences), which require a shorter treatment course (5 days) and have fewer side effects, remain unaffordable at nearly 30 times the cost of conventional formulations.[9,10] Miltefosine (Impavido, Aeterna Zentaris), the first effective oral therapy for visceral leishmaniasis,[11] is expensive,[12] is potentially teratogenic, and has significant gastrointestinal side effects.[11] Safe, effective, and affordable treatments for visceral leishmaniasis in regions where the disease is endemic are urgently needed, particularly in formulations that are compatible with rural settings.

Because humans are the reservoir for visceral leishmaniasis in the Indian subcontinent, the infection could be eliminated with widespread treatment of patients and rigorous vector control[1] (although elimination may not be possible in regions where zoonotic visceral leishmaniasis is prevalent, such as Brazil). Paromomycin, an aminoglycoside antibiotic, has been shown to have a dose–response efficacy in the treatment of visceral leishmaniasis when administered intramuscularly at a dose of 12, 16, or 20 mg of sulfate per kilogram of body weight daily for 21 days.[13,14] We present the results of a phase 3, multicenter, noninferiority clinical trial comparing the safety and efficacy of paromomycin and of amphotericin B for the treatment of visceral leishmaniasis in Bihar, India.

Methods

Study Design

This study was an open-label, prospective, randomized trial comparing paromomycin with amphotericin B (Sarabhai Piramal Pharmaceuticals) (hereafter referred to as amphotericin) in which the primary end point was safety and the secondary end point was efficacy. The study was conducted between June 2003 and November 2004 in Bihar, India; all patients provided written informed consent. The protocol was approved by the independent ethics committee at each of the four participating centers, the Drug Controller General of India, and the Steering Committee on Research Involving Human Subjects of the World Health Organization. AmBisome was donated by Gilead Sciences for use as rescue medication in the study, but the company had no role in the design of the study, the accrual or analysis of the data, or the preparation of the manuscript.

Study Medications

Paromomycin solution, 375 mg per milliliter (500 mg per milliliter as paromomycin sulfate) (Pharmamed Parenterals), was administered by deep gluteal intramuscular injection at a dose of 11 mg per kilogram (15 mg per kilogram as the sulfate) daily for 21 days. Amphotericin was diluted in water and 5% dextrose and, after an initial dose (to test for an allergic response), was infused intravenously for 6 hours at a dose of 1 mg per kilogram every other day for 30 days (a total of 15 infusions). Liposomal amphotericin, infused intravenously at a dose of 3 mg per kilogram daily for 5 days, was used as rescue medication in patients in whom the study treatment failed or relapse occurred.

Study Patients

Eligible subjects were between 5 and 55 years of age and had clinically suspected visceral leishmaniasis. Inclusion criteria were parasitologically positive splenic or bone marrow smear; negative serologic testing for the human immunodeficiency virus (HIV); hemoglobin level of at least 5.0 g per deciliter; white blood count greater than or equal to 1×10^9 per liter; platelet count greater than or equal to 50×10^9 per liter; levels of aspartate aminotransferase, alanine aminotransferase, and alkaline phosphatase less than or equal to three times the

Downloaded from www.nejm.org on June 21, 2007 . Copyright © 2007 Massachusetts Medical Society. All rights reserved.

upper limit of the normal range; prothrombin time less than or equal to 5 seconds greater than that among control subjects; and serum creatinine and potassium levels within the normal limits. Exclusion criteria were treatment for visceral leishmaniasis during the 2 weeks before enrollment, a hearing loss of 75 dB in frequencies 1 through 8 kHz, a history of vestibular or auditory dysfunction, prior treatment with amphotericin without response, allergy or hypersensitivity to aminoglycosides, significant proteinuria (≥2+ on strip testing), significant coexisting diseases possibly affecting the response to the study treatment response, and pregnancy or lactation.

STUDY PROCEDURES

Enrolled patients were randomly assigned to treatment with paromomycin or amphotericin in a 3:1 ratio in permuted blocks of four. A fraction of the patients in the paromomycin group were also randomly assigned to a substudy in which pharmacokinetic sampling was performed. All patients were hospitalized for the duration of the study treatment; vital signs were assessed daily and adverse events were reported according to the Common Toxicity Criteria (CTC) of the National Cancer Institute.[15] Patients were monitored for hematologic variables, serum chemistry, body weight, and the size of the spleen and liver every week during treatment, at the end of treatment, and at 6 months after the treatment ended. Audiometric testing was performed every week during treatment in all patients and repeated every 2 weeks and then monthly for up to 6 months after treatment ended in patients with ototoxicity. A sparse sampling design was used for the collection of pharmacokinetic samples. Splenic or bone marrow aspiration was performed at the end of treatment, at the 4-week follow-up in patients with few residual parasites at end of treatment, and in those who had a relapse of visceral leishmaniasis, during the 6-month follow-up period. Patients who were not cured or who had a parasitologically confirmed relapse received rescue medication.

END POINTS

Safety

The safety end points were reported adverse events, protocol-defined nephrotoxicity (defined as an increase in serum creatinine that was either double the baseline levels and more than 2.0 mg per deciliter [177 μmol per liter], or more than 2.5 mg per deciliter[16,17]) and ototoxicity (defined as a confirmed shift from baseline in audiometric thresholds by either 25+ dB at one or more of the tested frequencies [1 to 12 kHz], or 20+ dB at two or more adjacent frequencies), laboratory evaluations, and vital signs. Patients with potential ototoxicity were reviewed by an audiometry expert who was unaware of the treatment assignments.

Efficacy

Parasite density was graded on a log scale by pathologists who were also unaware of the treatment assignments. Final cure was defined as an initial cure (clinical improvement with no parasites at the end of treatment or parasite density of 1 at the end of treatment with no parasites on repeated smear 1 month after the end of treatment) and no relapse during follow-up. Relapse was defined as suspected visceral leishmaniasis after an initial cure, followed by a positive result on analysis of a specimen obtained by splenic or bone marrow aspiration. Treatment failure was defined as lack of an initial cure or occurrence of relapse. For pharmacokinetic sampling, plasma paromomycin levels were measured with the use of an assay validated by liquid chromatography–tandem mass spectrometry.

STATISTICAL ANALYSIS

Statistical analyses were performed with the use of SAS software, version 8.2, unless otherwise noted. Statistical tests included Student's t-test, Fisher's exact test, the chi-square test or the Cochran–Mantel–Haenszel test, and general linear equations, generalized estimation equations, or linear mixed-effect models. All tests were two-sided. Efficacy calculations were performed with the use of StatXact, version 7.0 (Cytel). Assuming a 99% cure rate for amphotericin, 666 patients were needed in a 3:1 ratio to support a one-sided, noninferiority analysis without stratification and with 80% power to detect a type I error rate of 5%. Primary efficacy was calculated as the proportion of patients achieving a final cure; an exact confidence interval for that proportion was computed; the exact, one-sided, upper bound of the 97.5% confidence interval for the difference in success probabilities was compared with the use of $\delta = 0.10$ (the chosen margin for noninferiority). All P values except the value for noninferiority were two-tailed. Pharmacokinetic analyses were performed with NONMEM software, version V, level 1.1 (GloboMax).

Downloaded from www.nejm.org on June 21, 2007 . Copyright © 2007 Massachusetts Medical Society. All rights reserved.

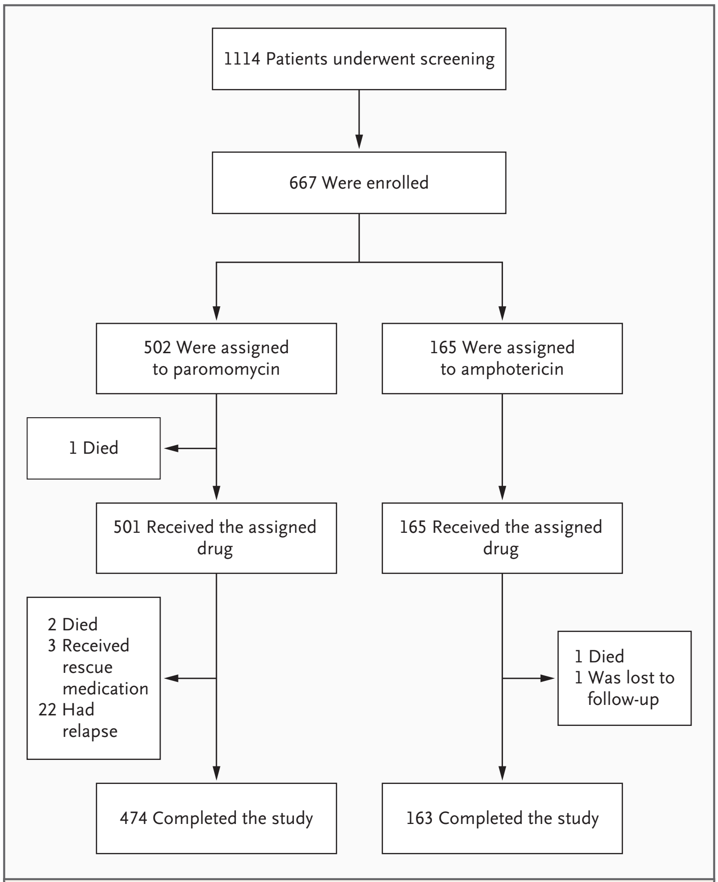

Figure 1. Disposition of the Patients.

One patient in the paromomycin group received a full course of amphotericin. Three patients in the paromomycin group who were not cured at the end of treatment received rescue medication. Relapse was defined as parasitologically confirmed visceral leishmaniasis at any time after an initial cure; the 22 patients who had a relapse were treated with rescue medication. The one patient lost to follow-up could not be located for the 6-month visit. One patient in the amphotericin group discontinued the study drug after receiving eight doses because pulmonary tuberculosis developed, but this patient was followed for the full 6 months.

RESULTS

Of 1114 patients who underwent screening, 667 (60%) were enrolled (Fig. 1). One patient assigned to paromomycin died before administration of the study drug. Ninety-six percent of the patients received the first dose of a study drug within 1 day after randomization. One patient assigned to paromomycin received a full course of amphotericin. Of the enrolled patients, 252 (38%) were pediatric patients, defined as 5 to 14 years of age, and 415 were adult patients, defined as 15 to 55 years of age. In 10% of the patients, either visceral leishmaniasis did not respond to previous treatment or relapses of visceral leishmaniasis had occurred (Table 1). The two study groups were balanced with respect to baseline characteristics.

SAFETY

Serious Adverse Events

Seven serious adverse events occurred, including four deaths (0.6%) and three events (0.4%) requiring discontinuation of the study drug (Table 2). Of the four deaths, one occurred in a patient before the administration of paromomycin; a second death, deemed by the investigator as possibly related to paromomycin, occurred in a patient with suspected alcoholism who received only two doses of the drug, after which aspartate aminotransferase levels increased to more than six times the upper limit of the normal range; a third death, deemed by the investigator to be unrelated to paromomycin, resulted from septicemia secondary to a thigh abscess after the patient had received 11 doses of the drug (no obvious connection between the injection site and the abscess was found); and a fourth death resulted from gastroenteritis and diarrhea and was considered to be probably related to amphotericin. The three nonfatal serious adverse events included two in patients who had elevated levels of hepatic enzymes after 8 doses of paromomycin (both patients required rescue medication), and one patient with bacterial pneumonia was treated with gatifloxacin after 10 doses of amphotericin.

Five patients (1%) in the paromomycin group had other adverse events requiring drug discontinuation: one patient had reversible ototoxicity and one had elevated levels of hepatic enzymes, and both required rescue therapy; three patients (one with elevated levels of hepatic enzymes, one with transient reversible ototoxicity, and one with injection-site pain) received eight or more doses of paromomycin and did not require rescue therapy. One patient (0.6%) in the amphotericin group discontinued the study drug after receiving eight doses because pulmonary tuberculosis developed.

Audiometric Testing

Audiometry data were available for a total of 589 patients (442 in the paromomycin group and 147 in the amphotericin group). After review by an expert audiologist who was unaware of the treatment as-

Downloaded from www.nejm.org on June 21, 2007 . Copyright © 2007 Massachusetts Medical Society. All rights reserved.

Table 1. Baseline Characteristics of the Patients.*

Characteristic	Paromomycin Group (N=501)	Amphotericin Group (N=165)
Age — yr	22.1±12.3	20.8±11.7
Age category — no. (%)†		
Pediatric	188 (38)	64 (39)
Adult	313 (63)	101 (61)
Sex — no. (%)		
Male	321 (64)	95 (58)
Female	180 (36)	70 (42)
Weight — kg	35.5±11.8	33.6±11.0
Height — cm	146.9±18.5	145.9±16.7
Body-mass index‡	15.9±2.9	15.3±2.8
Pediatric	13.8±2.0	13.1±2.2
Adult	17.2±2.5	16.7±2.2
Visceral leishmaniasis — no. (%)		
Newly diagnosed	449 (90)	150 (91)
Previously diagnosed without response to treatment§	52 (10)	15 (9)
Parasite density¶		
Median	2	2
Interquartile range	1–3	1–3
Palpable spleen size — cm below costal margin	6.6±3.8	6.8±4.0
Palpable liver size — cm below costal margin	2.1±1.2	2.1±1.3
Vital signs		
Body temperature — °F	99.9±1.3	100.0±1.4
Heart rate — beats/min‖	98.1±9.9	98.1±11.3
Respiratory rate — breaths/min‖	21.9±2.6	22.0±2.9
Systolic blood pressure — mm Hg‖	105.4±9.8	104.5±10.8
Laboratory values		
White-cell count — $\times10^{-3}/mm^3$	3.1±1.4	3.2±1.5
Platelet count — $\times10^{-3}/mm^3$	120.4±60.8	117.1±54.6
Hemoglobin — g/dl	7.8±1.7	7.7±1.6
Serum alanine aminotransferase — U/liter‖	40.5±25.6	41.1±26.2
Serum aspartate aminotransferase — U/liter‖	44.1±22.0	45.1±22.5
Alkaline phosphatase — U/liter‖	216.4±114	228.2±130
Total bilirubin — mg/dl‖	0.6±0.2	0.6±0.2
Serum creatinine — mg/dl‖	0.8±0.2	0.8±0.2
Blood urea nitrogen — mg/dl‖	11.7±4.2	11.9±3.8
Albumin — g/dl	3.1±0.5	3.1±0.7

* Plus–minus values are means ±SD. To convert the values for bilirubin to micromoles per liter, multiply by 17.1 To convert the values for creatinine to micromoles per liter, multiply by 88.4. To convert the values for urea nitrogen to millimoles per liter, multiply by 0.357.

† The pediatric age group was defined as patients 5 to 14 years of age, and the adult age group was defined as those 15 to 55 years of age.

‡ Body-mass index is the weight in kilograms divided by the square of the height in meters. The two groups were similar for all variables other than body-mass index (P=0.02); the difference was not statistically significant after adjustment for age and study center (P=0.06 for the comparison between the two groups among pediatric patients; P=0.09 for the comparison between the two groups among adult patients). Values are adjusted for study center.

§ Patients in this category had received prior treatment for visceral leishmaniasis but remained symptomatic or had been considered cured but had become symptomatic. Among the patients in the paromomycin group who had received previous treatment, 47 received sodium stibogluconate, 4 received miltefosine, and 1 patient received both these drugs. Among the patients in the amphotericin group who had received previous treatment, 14 received sodium stibogluconate and 1 received miltefosine.

¶ Density ranges from 0 to 6, with higher values indicating greater splenic parasite load.

‖ The safety substudy included 500 patients in the paromomycin group and 166 in the amphotericin group.

Downloaded from www.nejm.org on June 21, 2007 . Copyright © 2007 Massachusetts Medical Society. All rights reserved.

Table 2. Summary of Adverse Events Occurring during the Study.*

Variable	Paromomycin Group (N=500)	Amphotericin Group (N=166)	P Value†
	no. of patients (%)		
Deaths‡	2 (<1)	1 (1)	1.00
Nonfatal serious events§	2 (<1)	1 (1)	1.00
Reported events			
Any	299 (60)	111 (67)	0.12
CTC grade 3 or 4	11 (2)	11 (7)	0.01
Specific events¶			
Injection-site pain	276 (55)	0	<0.001
Pyrexia	13 (3)	94 (57)	<0.001
Rigors	0	39 (24)	<0.001
Vomiting	3 (1)	16 (10)	<0.001
Nephrotoxicity‖			
Protocol-defined	0	7 (4)	<0.001
Defined post hoc	4 (1)	42 (25)	<0.001
Ototoxicity**	7 (1)	0	0.20
Liver-function values††			
Aspartate aminotransferase >3× ULN	31 (6)	3 (2)	0.02
Aspartate aminotransferase >5× ULN	9 (2)	0	0.12
Alanine aminotransferase >3× ULN	10 (2)	1 (1)	0.31
Alanine aminotransferase >5× ULN‡‡	4 (1)	0	0.58

* Plus–minus values are means ±SD. Patients may have had more than one adverse event. CTC denotes Common Toxicity Criteria, and ULN upper limit of the normal range.

† P values for the comparison between the two groups were calculated with the use of Fisher's exact test.

‡ Of the deaths reported during the study, one occurred before the patient received a study drug, one was considered to be possibly related to paromomycin, one was considered to be probably related to amphotericin, and one was deemed by the investigator to be unrelated to paromomycin and resulted from septicemia secondary to a thigh abscess (no obvious connection between the injection site and the abscess was found).

§ Of the patients who had elevated levels of hepatic enzymes, two were in the paromomycin group and one with bacterial pneumonia was in the amphotericin group.

¶ Specific events were those with an incidence ≥5%.

‖ Protocol-defined nephrotoxicity included events for which the value was double the baseline value and greater than 2.0 mg per deciliter, or greater than 2.5 mg per deciliter; nephrotoxic events defined post hoc were those with values greater than or equal to 1.4 mg per deciliter and represented an increase during the treatment period of 50% or more above the baseline serum creatinine level.

** Ototoxicity was defined as a confirmed shift from baseline audiometric thresholds ≥25 dB at one or more of the tested frequencies (1 to 12 kHz), or as a confirmed threshold shift ≥20 dB at two or more adjacent frequencies.

†† Events with a CTC grade of 3 or 4 in the paromomycin group included increased aspartate aminotransferase levels (seven patients), increased alanine aminotransferase levels (three patients), diarrhea (one patient), abnormal audiogram (one patient), neutropenia (one patient), and decreased white-cell count (one patient). Events with a CTC grade of 3 or 4 in the amphotericin group included pyrexia (five patients), diarrhea (one patient), rigors (two patients), increased levels of alkaline phosphatase (one patient), jaundice (one patient), and toxic neuropathy (one patient).

‡‡ All four patients also had aspartate aminotransferase levels greater than five times the upper limit of normal.

signments, seven patients (2%) in the paromomycin group and none in the amphotericin group were found to have confirmed threshold shifts for protocol-specified ototoxicity (P=0.20) (Table 2). In six patients, the threshold shifts were at high frequency above the hearing range, and in all seven patients the shift was transient, with levels returning to near baseline values during follow-up. No patient reported hearing loss or vestibular dysfunction.

Renal Evaluation

None of the patients in the paromomycin group and seven patients (4%) in the amphotericin group had protocol-defined nephrotoxicity (P<0.001) (Ta-

Downloaded from www.nejm.org on June 21, 2007 . Copyright © 2007 Massachusetts Medical Society. All rights reserved.

ble 2). To ascertain potential renal dysfunction, we performed a post hoc analysis of creatinine elevation greater than or equal to 50% of the baseline levels and greater than or equal to 1.4 mg per deciliter during treatment. According to this definition, some renal dysfunction developed in 4 patients (1%) in the paromomycin group and in 42 patients (25%) in the amphotericin group (P<0.001). Mean changes from baseline to the end of treatment in levels of blood urea nitrogen and serum creatinine were significantly higher in the amphotericin group than in the paromomycin group (P<0.001 for both comparisons) (Table 3).

Other Adverse Events

Injection-site pain was the most frequently reported adverse event among patients receiving paromomycin (55%); adverse events that were most frequently reported among patients receiving amphotericin were infusion reactions of fever, rigors, and vomiting (57%, 24%, and 10%, respectively) (P<0.001 for all comparisons) (Table 2). Injection-site pain in those receiving paromomycin was rarely associated with swelling (0.4%) and was generally reported as CTC grade 1 (grade 2, <2%); only one patient (<1%) treated with paromomycin discontinued the study drug because of injection-site pain and swelling. CTC grades 3 and 4 events occurred less frequently in the paromomycin group than in the amphotericin group. In addition, use of concomitant medication was less common in the paromomycin group than in the amphotericin group (14% vs. 77%, P<0.001).

Other Laboratory Tests

Almost half the patients enrolled had elevated levels of alanine aminotransferase or aspartate aminotransferase, consistent with hepatic involvement in 25 to 40% of patients with visceral leishmaniasis.[18-21] Aspartate aminotransferase levels higher than three times the upper limit of the normal range developed in 31 patients (6%) in the paromomycin group, as compared with 3 patients (2%) in the amphotericin group (P=0.02). In nine (2%) of these patients in the paromomycin group, as compared with none in the amphotericin group, the increases in aspartate aminotransferase levels were greater than five times the upper limit of the normal range (P=0.12) (Table 2); and five patients in the paromomycin group discontinued the study drug because of adverse events (reversible ototoxicity, elevated levels of hepatic enzymes, and injection-site pain). Liver-function testing showed that levels returned to near baseline for all surviving patients.

EFFICACY

During the treatment period, 12 patients discontinued the study treatment. Of these, five remained cured at the 6-month follow-up visit. A total of 493 patients in the paromomycin group and 164 in the amphotericin group had an initial cure at the end of treatment. One patient in the amphotericin group was lost to follow-up; all 22 patients who had relapses were in the paromomycin group (Fig. 1).

Final cure rates 6 months after the end of treatment were 95% (474 of 501) in the paromomycin group and 99% (163 of 165) in the amphotericin group; the difference in rates was 4.2 percentage points with an upper bound of the 97.5% confidence interval of 6.9 percentage points, demonstrating the noninferiority of paromomycin (Table 4). This finding was consistent across all tested subgroups. Of 449 patients with newly diagnosed visceral leishmaniasis in the paromomycin group, 423 (94%) were cured, and of the 52 patients with prior visceral leishmaniasis, 51 (98%) were cured.

PHARMACOKINETIC ANALYSES

Paromomycin was absorbed quickly after intramuscular injection, reaching peak plasma levels within 1 hour. During the 21 days of treatment, the mean (±SD) peak plasma levels of paromomycin at 1 hour after injection ranged from 18.3 μg per milliliter (±8.86) to 20.5 μg per milliliter (±7.01) and the trough plasma levels at 24 hours after injection ranged from 1.31 μg per milliliter (±4.16) to 4.53 μg per milliliter (±6.71). The plasma levels on days 1, 8, 15, 21, and 22 were similar, and there was no evidence of drug accumulation or the induction of metabolism. No significant differences in the mean peak or trough plasma levels at day 21 were observed between pediatric and adult patients.

DISCUSSION

Paromomycin (administered intramuscularly at a dose of 11 mg per kilogram daily for 21 days) was shown to be noninferior to and to have an adverse-event profile similar to that of amphotericin (administered intravenously at a dose of 1 mg per kilogram every other day for 30 days) in the treatment of visceral leishmaniasis. On the basis of this study, paromomycin was approved by the Indian government in August 2006 for the treatment of patients

Downloaded from www.nejm.org on June 21, 2007 . Copyright © 2007 Massachusetts Medical Society. All rights reserved.

Table 3. Mean (±SD) End-of-Treatment Values and Changes in Clinical Measures and in Liver-Function and Renal-Function Values.*

Variable	At End of Treatment		Change from Baseline to End of Treatment†		P Value‡
	Paromomycin Group	Amphotericin Group	Paromomycin Group	Amphotericin Group	
**Safety analysis§**					
Heart rate (beats/min)	84.4±5.5	84.8±7.7	−13.7±12.2	−13.2±14.7	0.48¶
Respiratory rate (breaths/min)	20.4±2.8	20.6±4.3	−1.4±3.6	−1.4±5.1	0.55¶
Systolic blood pressure (mm Hg)	109.4±9.1	108.5±9.6	4.0±8.6	4.0±8.9	0.55¶
Alanine aminotransferase (U/liter)	48.3±30.7	32.2±18.5	7.8±35.6	−8.9±30.1	<0.001
Aspartate aminotransferase (U/liter)	52.2±35.9	33.0±16.8	8.2±39.2	−12.1±26.4	<0.001
Alkaline phosphatase (U/liter)	227.5±110	249.9±191	12.5±123	21.8±158	0.15
Total bilirubin (mg/dl)	0.6±0.3	0.6±0.2	−0.0±0.4	0.0±0.3	0.58
Serum creatinine (mg/dl)	0.7±0.2	1.0±0.4	−0.1±0.3	0.2±0.4	<0.001
Blood urea nitrogen (U/liter)	12.0±3.4	17.0±7.4	0.3±4.8	5.1±8.0	<0.001
**Efficacy analysis‖**					
Temperature (°F)	97.5±0.8	97.6±1.0	−2.4±1.5	−2.4±1.7	0.10
Spleen size (cm below costal margin)	1.7±2.2	1.3±1.8	−4.8±2.6	−5.5±3.2	<0.001
Weight (kg)	37.0±12.1	35.1±11.1	1.5±2.6	1.5±1.5	0.94
Hemoglobin (g/dl)	9.6±1.5	8.4±1.3	1.7±1.3	0.7±1.5	<0.001
Platelet count ($\times 10^{-3}$/mm^3)	254.5±122	253.9±120	133.9±111	136.8±111	0.81
White-cell count ($\times 10^{-3}$/mm^3)	6.7±2.9	6.9±2.2	3.6±2.7	3.7±2.2	0.65
Albumin (g/dl)	3.5±0.6	3.5±0.7	0.4±0.7	0.4±0.8	0.94

* To convert the values for bilirubin to micromoles per liter, multiply by 17.1. To convert the values for creatinine to micromoles per liter, multiply by 88.4. To convert the values for urea nitrogen to millimoles per liter, multiply by 0.357.
† For baseline values, see Table 1.
‡ Analysis of variance was used to correlate laboratory values at the end of treatment with the baseline value in the model.
§ Patients in the safety analysis included 500 in the paromomycin group and 166 in the amphotericin group.
¶ P values were calculated with the use of a mixed model in which heterogeneous variance and a first-order autocorrelation structure are assumed.
‖ Patients in the efficacy analysis included 501 in the paromomycin group and 165 in the amphotericin group.

with visceral leishmaniasis and is now available as a public health tool in a nationwide program to eliminate visceral leishmaniasis.

The overall cure rate of 95% with the use of paromomycin was similar in pediatric patients (96%), female patients (95%), and male patients (94%), and the difference in cure rates between patients treated with paromomycin or amphotericin was consistently less than 10 percentage points. The cure rate among those whose disease had not responded to previous treatment with sodium stibogluconate or miltefosine or who had had a relapse was high (98%). This finding is important in Bihar, where the failure of sodium stibogluconate therapy is due primarily to drug resistance.[2,6,7] Although experience with the use of paromomycin during pregnancy is limited, this drug may be used, when clinically indicated, in women of childbearing potential.[22] Furthermore, paromomycin can be administered intramuscularly according to body weight (milligrams per kilogram) to patients with visceral leishmaniasis who have normal renal function, including children, without the need for therapeutic monitoring or dose adjustment.

The duration of treatment with paromomycin (daily for 21 days) is shorter than with amphotericin (every other day for 30 days), sodium stibogluconate (daily for 30 days), or miltefosine (daily for 28 days), though the visit burden may be higher. In this study, protocol-defined nephrotoxicity did not develop in any of the patients treated with paromomycin. When more stringent definitions of potential renal dysfunction were ap-

Downloaded from www.nejm.org on June 21, 2007 . Copyright © 2007 Massachusetts Medical Society. All rights reserved.

Table 4. Efficacy (Cure Rate at 6 Months) of Paromomycin versus Amphotericin.

Patients	Paromomycin Group	Amphotericin Group	Difference in Rate	Upper Bound of 97.5% CI for Difference*
	no. cured/total no. (%)		%	%
Overall†	474/501 (94.6)	163/165 (98.8)	4.2	6.9
Age category — no. (%)‡				
Pediatric	181/188 (96.3)	63/64 (98.4)	2.1	6.5
Adult	293/313 (93.6)	100/101 (99.0)	5.4	9.1
Sex				
Male	303/321 (94.4)	95/95 (100)	5.6	8.9
Female	171/180 (95.0)	68/70 (97.1)	2.1	7.2

* The value was determined by a test of noninferiority (margin of noninferiority, 0.10). CI denotes confidence interval.
† This category includes all patients who underwent randomization and received at least one dose of a study drug.
‡ The pediatric age group was defined as patients 5 to 14 years of age, and the adult age group was defined as those 15 to 55 years of age.

plied, only 1% of the patients in the paromomycin group had renal dysfunction, as compared with 25% of those in the amphotericin group. This relative absence of nephrotoxicity among patients with visceral leishmaniasis who were treated with paromomycin at a dose of 11 mg per kilogram is not surprising, since the patients included in the study were generally young and had normal renal function and since leishmania parasites do not typically invade the kidneys. Audiometric data showed transient reversible ototoxicity during treatment in seven patients in the paromomycin group (2%); no long-term clinical hearing loss or vestibular effects were reported. These findings may help to guide the safety monitoring required for large-scale medication use in India.

All the medications used to treat visceral leishmaniasis (pentavalent antimonial compounds, pentamidine, amphotericin, liposomal amphotericin, sodium stibogluconate, and miltefosine) may be associated with a significant increase in levels of liver enzymes during treatment,[11,19,23,24] which some think may be due to the killing of the parasites in the liver, rather than to direct medication-induced hepatic toxic effects. Aminoglycosides used as parenteral antibiotics are rarely associated with increased levels of liver enzymes.[25] Although the exact cause of the transient significant increase in levels of hepatic enzymes, which affected patients in the paromomycin group but not those in the amphotericin group in this study, is difficult to ascertain, the possibilities include faster destruction of the parasites in liver tissue in patients treated with paromomycin or an emerging toxicity of paromomycin treatment in this setting. Monitoring of aspartate aminotransferase or alanine aminotransferase levels, or both, in a program to control visceral leishmaniasis will be an important consideration, especially in patients with preexisting liver disease.

Limitations of the study include high variation at the study sites in reporting injection-site pain, ranging from 2% to 97%. The data collection was not standardized for this specific outcome. Moreover, the patients were followed for only 6 months, which is the standard for visceral leishmaniasis trials, because most relapses occur during this period.[26] Although rare, post–kala-azar dermal leishmaniasis (PKDL) can occur years after treatment[27]; long-term follow up for PKDL was beyond the scope of this study. Though the overall relapse rate among patients treated with paromomycin was acceptable (4%), for a program to eliminate visceral leishmaniasis to succeed, early detection and prompt treatment of relapses, as well as mechanisms to identify and treat patients with PKDL, are imperative. Combination chemotherapy must also be explored to reduce the risk of drug resistance over time.

It is currently critical to address the elimination of visceral leishmaniasis in the Indian subcontinent, where the rates of HIV infection and HIV–visceral leishmaniasis coinfection are rising.[28] Since patients with HIV who are coinfected with visceral leishmaniasis will probably have relapse without lifelong antiretroviral therapy, they

Downloaded from www.nejm.org on June 21, 2007 . Copyright © 2007 Massachusetts Medical Society. All rights reserved.

may remain an infectious reservoir until proper HIV therapy can be deployed in a sustainable manner. Furthermore, a significant proportion of the at-risk population may have subclinical leishmania infection, contributing to transmission of visceral leishmaniasis.[29] For widespread public health use, the intramuscular administration of paromomycin is challenging; however, primary health-center personnel have experience with intramuscular administration of sodium stibogluconate, and supervised dosing can limit the resistance due to noncompliance with the regimen.

In conclusion, paromomycin was shown to be noninferior to amphotericin, and, with the exception of mild injection-site pain and a transient increase in values on liver-function testing, it has a reasonable safety profile. Paromomycin may be advantageous because of the shorter duration of its administration and its demonstrated safety and efficacy in pediatric patients and in patients in whom visceral leishmaniasis did not respond to previous treatment. The health care delivery system in India is well suited to the intramuscular administration of paromomycin under directly observed therapy, and the local manufacture of paromomycin in India, potentially at a very low cost, makes this an approachable therapy in the setting of limited resources.

Supported by grants from the Bill and Melinda Gates Foundation to the Institute for OneWorld Health; and by the Institute for OneWorld Health and the Special Program for Research and Training in Tropical Diseases (TDR) of the United Nations Development Program, the World Bank, and the World Health Organization (WHO).

Dr. Sundar reports receiving travel support from Asta Medica, Zentaris, Liposome, GlaxoSmithKline, DiaMed, Institute for OneWorld Health and grant support from GlaxoSmithKline, Asta Medica, Nexstar, Gilead Sciences, and the WHO; and Dr. Jha, grant support from GlaxoSmithKline. No other potential conflict of interest relevant to this article was reported.

We thank our patients and the staff and administration of the four Kala-Azar Centers of Excellence: Drs. I. Singh, D. Verma, M. Kumar Singh, A. Kumar, K. Pandey, N. Kumar, S.M. Hassan, C.P.N. Thakur, N. Verma, C.S. Lal, S.K. Jaiswal, S.K. Verma, and S. Sharma; many colleagues at the WHO TDR for developing paromomycin for the treatment of visceral leishmaniasis in prior clinical trials in Bihar; Médecins sans Frontières and the International Dispensary Association for providing access to paromomycin; Dr. T. Brewer, of the Bill and Melinda Gates Foundation, and Dr. C. Ley, for reviewing an earlier draft of the manuscript; and A. Herskowitz, V. Hale, C. Rask, G. Crean, A. Llosa, K. Oliver, E. Cooper, D. Tranowski, J. Mordenti, and M. McGuffey, of the Institute for OneWorld Health, and J. Berman, W. Gutteridge, and A. Bryceson, of the Product Development Team, WHO TDR, Geneva.

APPENDIX

Other members of the Paromomycin for VL Study Teams are as follows: **Data Coordinating Centers**: *Institute for OneWorld Health, San Francisco* (lead center): B. Nguyen, E. Kwan, A. Oudin, K. Valcke, S. Mathie, C. Ley; *Majaro InfoSystems, Santa Clara, CA*: M. Rosenberg; *DIEM Computing Services, Newark, CA*: E.L. Gaithersburg, L. Muenz, D. He; **Data Safety Monitoring Board:** *Harvard School of Public Health, Boston*: L.J. Wei (chair); *University of New Mexico, Albuquerque*: B. Ballanchanda; *Diablo Nephrology Medical Group, Walnut Creek, CA*: E. Wrone; *Touro University College of Osteopathic Medicine, Vallejo, CA*: E. Mahmoud; *Northwick Park Hospital, Middlesex, United Kingdom*: R. Davidson; **Audiology Monitoring Committee**: *University of California San Francisco Medical Center, San Francisco*: R. Sweetow (chair); *University of New Mexico, Albuquerque*: B. Ballanchanda; *Washington University Medical Center, St. Louis*: M. Valente; **Pharmacokinetic Analysis Group**: *University of California San Francisco Medical Center, San Francisco*: L. Sheiner (deceased), S. Beal (deceased); *University of California San Francisco Drug Studies Unit, San Francisco*: E. Lin, W. Gee, Y. Huang, H. Chang, X. Li.

REFERENCES

1. Desjeux P. Leishmaniasis: current situation and new perspectives. Comp Immunol Microbiol Infect Dis 2004;27:305-18.
2. Sundar S, More DK, Singh MK, et al. Failure of pentavalent antimony in visceral leishmaniasis in India: report from the center of the Indian epidemic. Clin Infect Dis 2000;31:1104-7.
3. Thakur CP, Sinha GP, Pandey AK, et al. Do the diminishing efficacy and increasing toxicity of sodium stibogluconate in the treatment of visceral leishmaniasis in Bihar, India, justify its continued use as a first-line drug? An observational study of 80 cases. Ann Trop Med Parasitol 1998;92:561-9.
4. Ahasan HA, Chowdhury MA, Azhar MA, Rafiqueuddin AK, Azad KA. Deaths in visceral leishmaniasis (Kala-azar) during treatment. Med J Malaysia 1996;51:29-32.
5. Lira R, Sundar S, Makharia A, et al. Evidence that the high incidence of treatment failures in Indian kala-azar is due to the emergence of antimony-resistant strains of Leishmania donovani. J Infect Dis 1999;180:564-7.
6. Sundar S, Singh VP, Sharma S, Makharia MK, Murray HW. Response to interferon-gamma plus pentavalent antimony in Indian visceral leishmaniasis. J Infect Dis 1997;176:1117-9.
7. Thakur CP, Narayan S, Ranjan A. Epidemiological, clinical and pharmacological study of antimony-resistant visceral leishmaniasis in Bihar, India. Indian J Med Res 2004;120:166-72.
8. Das VN, Ranjan A, Bimal S, et al. Magnitude of unresponsiveness to sodium stibogluconate in the treatment of visceral leishmaniasis in Bihar. Natl Med J India 2005;18:131-3.
9. Sundar S, Rai M. Advances in the treatment of leishmaniasis. Curr Opin Infect Dis 2002;15:593-8.
10. Rosenthal E, Marty P. Recent understanding in the treatment of visceral leishmaniasis. J Postgrad Med 2003;49:61-8.
11. Sundar S, Jha TK, Thakur CP, et al. Oral miltefosine for Indian visceral leishmaniasis. N Engl J Med 2002;347:1739-46.
12. Sundar S, Murray HW. Availability of miltefosine for the treatment of kala-azar in India. Bull World Health Organ 2005;83:394-5.
13. Thakur CP, Kanyok TP, Pandey AK, Sinha GP, Messick C, Olliaro P. Treatment of visceral leishmaniasis with injectable paromomycin (aminosidine): an open-label randomized phase-II clinical study. Trans R Soc Trop Med Hyg 2000;94:432-3.
14. Jha TK, Olliaro P, Thakur CP, et al. Randomised controlled trial of aminosidine (paromomycin) v sodium stibogluconate for treating visceral leishmaniasis in North Bihar, India. BMJ 1998;316:1200-5.
15. Cancer Therapy Evaluation Program.

Downloaded from www.nejm.org on June 21, 2007 . Copyright © 2007 Massachusetts Medical Society. All rights reserved.

Common toxicity criteria. Bethesda, MD: National Cancer Institute, 1998. (Accessed May 25, 2007, at http://ctep.cancer.gov/forms/CTCv20_4-30-992.pdf.)
16. Wingard JR, Kublis P, Lee L, et al. Clinical significance of nephrotoxicity in patients treated with amphotericin B for suspected or proven aspergillosis. Clin Infect Dis 1999;29:1402-7.
17. Walsh TJ, Pappas P, Winston DJ, et al. Voriconazole compared with liposomal amphotericin B for empirical antifungal therapy in patients with neutropenia and persistent fever. N Engl J Med 2002;346:225-34. [Erratum, N Engl J Med 2007;356:760.]
18. Singh UK, Sinha RK, Sharma VK. Fulminant hepatitis in Kala-azar. Indian J Pediatr 1995;62:571-4.
19. Jha TK, Sundar S, Thakur CP, et al. Miltefosine, an oral agent, for the treatment of Indian visceral leishmaniasis. N Engl J Med 1999;341:1795-800.
20. el Hag IA, Hashim FA, el Toum IA, Homeida M, el Kalifa M, el Hassan AM. Liver morphology and function in visceral leishmaniasis (Kala-azar). J Clin Pathol 1994;47:547-51.
21. Aggarwal P, Wali JP, Chopra P. Liver in kala-azar. Indian J Gastroenterol 1990; 9:135-6.
22. Czeizel AE, Rockenbauer M, Olsen J, Sorensen HT. A teratological study of aminoglycoside antibiotic treatment during pregnancy. Scand J Infect Dis 2000;32:309-13.
23. Thakur CP, Kanyok TP, Pandey AK, et al. A prospective randomized, comparative, open-label trial of the safety and efficacy of paromomycin (aminosidine) plus sodium stibogluconate versus sodium stibogluconate alone for the treatment of visceral leishmaniasis. Trans R Soc Trop Med Hyg 2000;94:429-31.
24. Chunge CN, Owate J, Pamba HO, Donno L. Treatment of visceral leishmaniasis in Kenya by aminosidine alone or combined with sodium stibogluconate. Trans R Soc Trop Med Hyg 1990;84:221-5.
25. Chambers HF. The aminoglycosides. In: Brunton LB, Lazo JS, Parker KL, eds. Goodman & Gilman's The pharmacological basis of therapeutics. 11th ed. New York: McGraw-Hill, 2006:1155-71.
26. Collin S, Davidson R, Ritmeijer K, et al. Conflict and kala-azar: determinants of adverse outcomes of kala-azar among patients in southern Sudan. Clin Infect Dis 2004;38:612-9.
27. Zijlstra EE, Musa AM, Khalil EA, el-Hassan IM, el-Hassan AM. Post-kala-azar dermal leishmaniasis. Lancet Infect Dis 2003;3:87-98.
28. Sinha PK, Bimal S, Singh SK, Pandey K, Gangopadhyay DN, Bhattacharya SK. Pre- and post-treatment evaluation of immunological features in Indian visceral leishmaniasis (VL) patients with HIV co-infection. Indian J Med Res 2006;123:197-202.
29. Sundar S, Maurya R, Singh RK, et al. Rapid, noninvasive diagnosis of visceral leishmaniasis in India: comparison of two immunochromatographic strip tests for detection of anti-K39 antibody. J Clin Microbiol 2006;44:251-3.

Copyright © 2007 Massachusetts Medical Society.

CLINICAL TRIAL REGISTRATION

The *Journal* requires investigators to register their clinical trials in a public trials registry. The members of the International Committee of Medical Journal Editors (ICMJE) will consider most clinical trials for publication only if they have been registered (see N Engl J Med 2004;351:1250-1). Current information on requirements and appropriate registries is available at www.icmje.org/faq.pdf.

Downloaded from www.nejm.org on June 21, 2007 . Copyright © 2007 Massachusetts Medical Society. All rights reserved.

Appendix 222 CP Thakur et al 2000

Short Report

Treatment of visceral leishmaniasis with injectable paromomycin (aminosidine). An open-label randomized phase-II clinical study

C. P. Thakur[1], T. P. Kanyok[2], A. K. Pandey[1], G. P. Sinha[1], C. Messick[2] and P. Olliaro[2] [1]*Kala-azar Research Centre, Patna, Bihar, India;* [2]*UNDP/World Bank/WHO Special Programme for Research and Training in Tropical Diseases, World Health Organization, Geneva, Switzerland*

Keywords: visceral leishmaniasis, chemotherapy, paromomycin, sodium stibogluconate, clinical trial, India

Paromomycin (PM), in a series of clinical trials alone or in combination with sodium stibogluconate (SB), has been shown to be highly efficacious and well tolerated for the treatment of visceral leishmaniasis (VL) (CHUNGE *et al.*, 1990; SCOTT *et al.*, 1992; THAKUR *et al.*, 1992, 1995; SEAMAN *et al.*, 1993; HASSAN *et al.*, 1995; JHA *et al.*, 1998). This study, a phase-II randomized, open-label, dose-finding trial, performed in 1996, was designed to determine the maximal safe and effective dose of PM (given at 12, 16 or 20 mg/kg daily for 21 days)(Gabbromicina®; Farmitalia–Carlo Erba, Milan, Italy [now Pharmacia & Upjohn]) compared to sodium stibogluconate (20 mg/kg daily, maximum 8·5 mL/day, for 28 days)(Albert-Davis Ltd, Calcutta, India) in 120 patients (30 per treatment arm) aged 6–50 years with VL diagnosed by a Giemsa-stained spleen or bone-marrow aspirate. Refer to JHA *et al.* (1998) for a complete description of materials and methods employed in this study.

Results of the study are as follows. The 4 groups did not differ in any pre-treatment patient demographics. The majority of patients were male, and ages ranged from 6 to 50 (mean ± SD, 22·41 ± 11·37) years. Table 1 shows that a final cure, defined as clinical improvement and parasitological cure at 180 days after the end of treatment, was achieved in 27 of 30, 24 of 27 and 25 of 29 patients given PM 12, 16, and 20 mg/kg daily, respectively, compared to 20 of 29 treated with SB. Of the 3 PM dose regimens only the PM12 treatment was significantly more effective than SB (χ^2 $P = 0·04$).

At the end of treatment, spleen or bone-marrow aspirates were negative for *Leishmania* amastigotes in 30, 30, and 30 patients given PM 12, 16, and 20 mg/kg daily, respectively, compared to 22 patients treated with SB. All 3 PM regimens proved significantly better than SB (χ^2 $P < 0·05$) in curing patients at the end of treatment. During the drug-free follow-up: 3, 3, and 4 patients who had received PM 12, 16, and 20 mg/kg daily, respectively, compared to 1 patient who had received SB, relapsed after initial parasite clearance. In PM16, 3 patients did not report for the 6-month follow-up; in PM20, 1 patient did not report fot the 6-month follow-up; and in SB, 1 patient did not report for the 6-month follow-up. Table 2 summarizes the end of treatment changes from baseline.

The results of this phase-II study build upon the work of previously published studies conducted with single-agent PM (CHUNGE *et al.*, 1990; SCOTT *et al.*, 1992; HASSAN *et al.*,1995; JHA *et al.*, 1998). This study along with the results of a sister study (JHA *et al.*, 1998) confirm that in Bihar, India, single-agent PM is a safe and effective therapy for the treatment of VL. The study by JHA *et al.* (1998) produced results similar to those obtained in this study with final cure rates of 23 of 30 (77%), 28 of 30 (93%), and 29 of 30 (97%) patients in the groups taking PM 12, 16, and 20 mg/kg daily for 21 days compared with 19 of 30 (63%) patients in the group taking SB 20 mg/kg daily for 28 days. In the study by JHA *et al.* (1998) PM dosed at 16 and 20 mg/kg daily was significantly more active than the standard dose of SB employed in the trial (χ^2 $P < 0·05$). In our study, only PM dosed at 12 mg/kg daily was significantly more active than a standard dose of SB, but not significantly different from PM 16 and 20 mg/kg daily. However, if the results of the PM-treated patients in both studies are compared to the results of SB therapy using Peto's fixed odds ratio (OR), PM at all 3 treatment levels is significantly more effective than SB. The odds ratio versus SB was 2·58 (1·14–5·84) for PM 12, 3·21 (1·40–7·37) for PM 16, and 4·12 (1·76–9·64) for PM 20.

The adverse events were also similar with no major differences reported in the incidence or severity of reactions between either study. One major limitation of this study was the incomplete recording of audiometric data in all patients receiving PM or SB treatment, as well as incomplete ECG data on all patients enrolled in the study. Since oto-toxicity is 1 of the 2 major historical toxicities of PM, all future studies that are being planned with PM will need to plan and record rigorously baseline, post-treatment and 6-month follow-up audiometric data in order to quantify and characterize better the incidence and severity of oto-toxicity in patients after a 21-day course of injectable PM. The other major adverse event reported with injectable paromomycin is renal toxicity. However, renal toxicity was not observed in this or in the other sister study conducted in Bihar (JHA *et al.*, 1998).

The results of this study and the study by JHA *et al.* (1998) lead the authors to recommend PM as a replace-

Table 1. Overall assessment of treatment of visceral leishmaniasis with injectable paromomycin or sodium stibogluconate in Bihar (India)

Treatment group	Final cure	Failure	Relapse	Defaulters	Total
PM12	27/30 (90·0%)	0	3	0	30
PM16	24/27 (88·9%)	0	3	3	30
PM20	25/29 (86·2%)	0	4	1	30
SB20	20/29 (69·0%)	8	1	1	30

PM12, PM16, and PM20, paromomycin given daily for 21 days at 12, 16 or 20 mg/kg, respectively; SB, sodium stibogluconate at 20 mg/kg daily for 28 days (maximum 8·5 mL/day). In PM16, PM20 and SB groups, 3, 1 and 1 patients, respectively, did not report for 6-month follow-up. Therefore, all could not be re-assessed for final cure.

Address for correspondence: Dr T. P. Kanyok, Manager, Product Development, UNDP/World Bank/WHO TDR, World Health Organization, 20 Avenue Appia, CH-1211, Geneva 27, Switzerland; fax +41 22 7913111.
The authors alone are responsible for the views expressed in this article.

Table 2. Summary of changes in efficacy parameters at end of treatment (EoT) compared with baseline

Parameter	PM12	PM16	PM20	SB20	*P*-value EoT
Weight (kg)	2·3 ± 2·32✓	1·07 ± 1·43	1·40 ± 3·14	0·45 ± 1·83✓	0·019✓
Fever (°C)	(2·01) ± 0·68✓	(1·85) ± 0·88✓	(2·53) ± 0·95✓	(2·56) ± 0·84✓	0·001✓
Spleen size (cm)	(4·20) ± 2·47	(4·96) ± 3·58	(5·65) ± 2·65✓	(3·32) ± 3·00✓	0·016✓
Hb% (g/dL)	1·60 ± 1·39✓	2·00 ± 1·38✓	1·27 ± 1·26	0·48 ± 1·73✓	0·001✓
WBC ($\times 10^9$/L)	2·60 ± 2·38	2·47 ± 6·71	2·68 ± 2·66	1·33 ± 2·99	0·54
Platelet count ($\times 10^9$/L)	22·83 ± 31·78	18·67 ± 54·94	35·83 ± 33·06	29·80 ± 38·46	0·45
Albumin (g/dL)	0·18 ± 0·57✓	0·60 ± 0·44✓	0·46 ± 0·61	0·19 ± 0·44✓	0·008✓
Parasite grading	(1·38) ± 0·56	(1·33) ± 0·48	(1·57) ± 0·82	(1·27) ± 1·14	0·68

PM12, PM16, and PM20, paromomycin given daily for 21 days at 12, 16 or 20 mg/kg, respectively; SB20, sodium stibogluconate at 20 mg/kg daily for 28 days (maximum 8·5 mL/day). Thirty patients in each group were assessed for parameter changes at 21 days (PM groups), or 30 days (SB group), after treatment by 1-way ANOVA with post-hoc Tukey's HSD. ✓, statistically significant difference at EoT; values in parentheses express a decrease from baseline.

ment for antimony for the treatment for VL in Bihar, India, and to recommend the conduct of a pivotal phase-III randomized, comparative control trial of PM at 16 mg/kg daily for 21 days compared to standard therapy.

References

Chunge, C. N., Owate, J., Pamba, H. O. & Donno, L. (1990). Treatment of visceral leishmaniasis in Kenya by aminosidine alone or combined with sodium stibogluconate. *Transactions of the Royal Society of Tropical Medicine and Hygiene*, **84**, 221–225.

Hassan, M., Baat, D. B. & Hassan, K. A. (1995). New breakthrough in treatment of visceral leishmaniasis in children. *Journal of the Pakistan Medical Association*, **45**, 155–157.

Jha, T. K., Olliaro, P., Thakur, C. P., Kanyok, T. P., Singhania, B. L., Singh, I. J., Singh, N. K., Akhoury, S. & Jha, S. (1998). Randomised controlled trial of aminosidine (paromomycin) v sodium stibogluconate for treating visceral leishmaniasis in North Bihar, India. *British Medical Journal*, **316**, 1200–1205.

Seaman, J., Pryce, D., Sondorp, H. E., Moody, A., Bryceson, A. D. & Davidson, R. N. (1993). Epidemic visceral leishmaniasis in Sudan: a randomised trial of aminosidine plus sodium stibogluconate versus sodium stibogluconate alone. *Journal of Infectious Diseases*, **168**, 715–720.

Scott, J. A. G., Davidson, R. N., Moody, A. H., Grant, H. R., Felmingham, D., Scott, G. M. S., Olliaro, P. & Bryceson, A. D. M. (1992). Aminosidine (paromomycin) in the treatment of leishmaniasis imported into the United Kingdom. *Transactions of the Royal Society of Tropical Medicine and Hygiene*, **86**, 617–619.

Thakur, C. P., Olliaro, P., Gothoskar, S., Bhowmick, S., Choudhury, B. K., Prasad, S., Kumar, M. & Verma, B. B. (1992). Treatment of visceral leishmaniasis (kala-azar) with aminosidine (=paromomycin)-antimonial compounds, a pilot study in Bihar, India. *Transactions of the Royal Society of Tropical Medicine and Hygiene*, **86**, 615–616.

Thaker, C. P., Bhowmick, S., Dolfi, L. & Olliaro, P. (1995). Aminosidine plus sodium stibogluconate for the treatment of Indian kala-azar: a randomized dose-finding clinical trial. *Transactions of the Royal Society of Tropical Medicine and Hygiene*, **89**, 219–223.

Received 8 September 1999; revised 16 December 1999; accepted for publication 6 January 2000

Announcements

Nineteenth European Course in Tropical Epidemiology (ECTE 2000)

Verona, Italy
4–15 September 2000

For further details please contact Dr Gloria Castellani, Centro per le Malattie Tropicali, Hospital 'S. Cuore-Don G. Calabria', 37024 Negrar, Verona, Italy; phone +39 045 6013324, fax +39 045 7500480, e-mail castelglo@tropicalmed.org, website www.tropicalmed.org

VIIIth European Multicolloquium of Parasitology

Poznań, Poland
10–14 September 2000

The Multicolloquium will be organized by the Polish Parasitological Society. Further information can be obtained from: Prof. Krystyna Boczoń, VIIIth European Multicolloquium of Parasitology, Department of Biology and Medical Parasitology, Karol Marcinkowski University of Medical Sciences, Fredry 10, 60-701 Poznań, Poland; phone +48 61 8521161, fax +48 61 8527192.

16.2 PATIENT DATA LISTINGS

16.2.1 Patient Visit Dates

Appendix 233 Patient visit dates

Centre	Patient	Treatment	Assessment Date							
Number	Number		Baseline	Day 1	Day 7	Day 14	Day 21	EOT	3 Mon FU	6 Mon FU
11	1	PM	17-Jun-05	18-Jun-05	24-Jun-05	1-Jul-05		9-Jul-05	5-Oct-05	4-Jan-06
11	2	Combination	17-Jun-05	18-Jun-05	24-Jun-05	1-Jul-05		5-Jul-05	29-Sep-05	5-Jan-06
11	3	Combination	17-Jun-05	19-Jun-05	25-Jun-05	2-Jul-05		6-Jul-05	30-Sep-05	29-Dec-05
11	4	PM	27-Jun-05	30-Jun-05	6-Jul-05	13-Jul-05		21-Jul-05	31-Dec-05	27-Jan-06
11	5	PM	27-Jun-05	30-Jun-05	6-Jul-05	13-Jul-05		21-Jul-05	18-Oct-05	14-Feb-06
11	6	Combination	27-Jun-05	2-Jul-05	8-Jul-05	15-Jul-05		19-Jul-05	13-Oct-05	11-Jan-06
11	7	PM	29-Jun-05	2-Jul-05	8-Jul-05	15-Jul-05		23-Jul-05	20-Oct-05	18-Jan-06
11	8	SSG	29-Jun-05	2-Jul-05	8-Jul-05	15-Jul-05	25-Jul-05	1-Aug-05	30-Oct-05	30-Jan-06
11	9	SSG	29-Jun-05	3-Jul-05	9-Jul-05	16-Jul-05	25-Jul-05	2-Aug-05	1-Nov-05	30-Jan-06
11	10	SSG	29-Jun-05	3-Jul-05	9-Jul-05	16-Jul-05	25-Jul-05	2-Aug-05	1-Nov-05	30-Jan-06
11	11	Combination	1-Jul-05	5-Jul-05	11-Jul-05	18-Jul-05		22-Jul-05	20-Oct-05	20-Jan-06
11	12	Combination	29-Jun-05	8-Jul-05	14-Jul-05	21-Jul-05		27-Jul-05	25-Oct-05	6-Feb-06
11	13	SSG	7-Jul-05	12-Jul-05	18-Jul-05	25-Jul-05	1-Aug-05	11-Aug-05	9-Nov-05	6-Feb-06
11	14	SSG	7-Jul-05	12-Jul-05	18-Jul-05	25-Jul-05	1-Aug-05	11-Aug-05	11-Nov-05	16-Apr-06
11	15	PM	13-Jul-05	14-Jul-05	20-Jul-05				7-Nov-05	5-Apr-06
11	16	Combination	12-Jul-05	15-Jul-05	21-Jul-05	28-Jul-05		1-Aug-05	30-Oct-05	6-Feb-06
11	17	Combination	12-Jul-05	19-Jul-05	25-Jul-05	1-Aug-05		5-Aug-05	7-Nov-05	6-Feb-06
11	18	SSG	13-Aug-05	16-Aug-05	22-Aug-05	29-Aug-05	5-Sep-05	15-Sep-05	22-Dec-05	15-Mar-06
11	19	Combination	14-Aug-05	16-Aug-05	22-Aug-05	29-Aug-05		2-Sep-05	29-Nov-05	27-Feb-06
11	20	SSG	14-Aug-05	16-Aug-05	22-Aug-05	29-Aug-05	5-Sep-05	15-Sep-05	14-Dec-05	14-Mar-06
11	21	SSG	14-Aug-05	16-Aug-05	22-Aug-05	29-Aug-05	5-Sep-05	15-Sep-05	22-Dec-05	15-Mar-06
11	22	SSG	15-Aug-05	17-Aug-05	23-Aug-05	30-Aug-05	6-Sep-05	16-Sep-05	15-Dec-05	14-Apr-06
11	23	PM	18-Aug-05	20-Aug-05	26-Aug-05	2-Sep-05		10-Sep-05	11-Jan-06	31-May-06
11	24	PM	18-Aug-05	20-Aug-05	26-Aug-05	2-Sep-05		10-Sep-05	28-Nov-05	10-Feb-06
11	25	SSG	18-Aug-05	20-Aug-05	26-Aug-05	2-Sep-05	9-Sep-05	19-Sep-05	2-Jan-06	14-Apr-06
11	26	Combination	15-Aug-05	25-Aug-05	31-Aug-05	7-Sep-05		12-Sep-05	10-Nov-05	16-Apr-06
11	27	PM	16-Sep-05	19-Sep-05	25-Sep-05	2-Oct-05		10-Oct-05	23-Jan-06	
11	28	Combination	16-Sep-05	21-Sep-05	27-Sep-05	4-Oct-05		8-Oct-05	4-Jan-06	4-Apr-06
11	29	PM	21-Sep-05	22-Sep-05	28-Sep-05	5-Oct-05		13-Oct-05	9-Jan-06	18-Apr-06
11	30	PM	20-Sep-05	22-Sep-05	28-Sep-05	5-Oct-05		13-Oct-05	2-Jan-06	14-Apr-06
11	31	SSG	20-Sep-05	23-Sep-05	29-Sep-05	6-Oct-05	13-Oct-05	23-Oct-05	2-Feb-06	5-Apr-06
11	32	Combination	20-Sep-05	24-Sep-05	30-Sep-05	7-Oct-05		11-Oct-05	9-Jan-06	3-Apr-06
11	33	SSG	23-Sep-05	25-Sep-05	1-Oct-05	8-Oct-05	15-Oct-05	25-Oct-05	23-Jan-06	16-Apr-06
11	34	PM	23-Sep-05	27-Sep-05	3-Oct-05	10-Oct-05		18-Oct-05	17-Jan-06	16-Apr-06
11	35	SSG	24-Sep-05	27-Sep-05	3-Oct-05	10-Oct-05	17-Oct-05	27-Oct-05	11-Jan-06	16-Apr-06
11	36	SSG	21-Sep-05	28-Sep-05	4-Oct-05	11-Oct-05	18-Oct-05	28-Oct-05	24-Jan-06	10-Jun-06
11	37	Combination	26-Sep-05	28-Sep-05	4-Oct-05	11-Oct-05		15-Oct-05	17-Jan-06	14-Apr-06
11	38	PM	13-Oct-05	19-Oct-05	25-Oct-05	1-Nov-05		9-Nov-05	15-Feb-06	30-May-06
11	39	PM	13-Oct-05	19-Oct-05	25-Oct-05	1-Nov-05		9-Nov-05	6-Mar-06	22-May-06
11	40	PM	13-Oct-05	20-Oct-05	26-Oct-05	2-Nov-05			14-Apr-06	23-May-06
11	41	Combination	13-Oct-05	20-Oct-05	26-Oct-05	2-Nov-05		6-Nov-05	13-Feb-06	31-May-06
11	42	Combination	13-Oct-05	21-Oct-05	27-Oct-05	3-Nov-05		7-Nov-05	6-Feb-06	7-Jun-06
11	43	SSG	24-Oct-05	28-Oct-05	3-Nov-05	10-Nov-05	17-Nov-05	27-Nov-05	22-Feb-06	23-May-06
11	44	PM	24-Oct-05	28-Oct-05	3-Nov-05	10-Nov-05		18-Nov-05	14-Feb-06	19-May-06
11	45	Combination	24-Oct-05	28-Oct-05	3-Nov-05	10-Nov-05		14-Nov-05	14-Feb-06	19-May-06

Centre	Patient	Treatment	Assessment Date							
Number	Number		Baseline	Day 1	Day 7	Day 14	Day 21	EOT	3 Mon FU	6 Mon FU
11	46	SSG	2-May-06	5-May-06	11-May-06	18-May-06	25-May-06	4-Jun-06	24-Oct-06	5-Dec-06
11	47	SSG	2-May-06	5-May-06	11-May-06	18-May-06	25-May-06	4-Jun-06		
11	48	Combination	3-May-06	5-May-06	11-May-06	18-May-06		22-May-06		2-Jan-07
11	49	SSG	7-May-06	9-May-06	15-May-06	22-May-06	29-May-06	8-Jun-06	9-Oct-06	27-Feb-07
11	50	PM	16-May-06	18-May-06	24-May-06	31-May-06		8-Jun-06	6-Sep-06	11-Dec-06
11	51	Combination	18-May-06	20-May-06	26-May-06	2-Jun-06		6-Jun-06	7-Sep-06	2-Jan-07
11	52	PM	19-May-06	20-May-06	26-May-06	2-Jun-06		10-Jun-06	26-Sep-06	11-Jan-07
11	53	SSG	25-May-06	26-May-06	1-Jun-06	8-Jun-06	15-Jun-06	25-Jun-06	26-Sep-06	11-Dec-06
11	54	PM	25-May-06	27-May-06	2-Jun-06	9-Jun-06		17-Jun-06	28-Aug-06	1-Jan-07
11	55	PM	29-May-06	30-May-06	5-Jun-06	12-Jun-06		20-Jun-06	26-Sep-06	11-Dec-06
11	56	SSG	29-May-06	31-May-06	6-Jun-06	13-Jun-06	20-Jun-06	30-Jun-06	28-Sep-06	14-Dec-06
11	57	Combination	3-Jun-06	4-Jun-06	10-Jun-06	17-Jun-06		21-Jun-06	12-Sep-06	12-Dec-06
11	58	Combination	3-Jun-06	6-Jun-06	12-Jun-06	19-Jun-06		23-Jun-06	26-Sep-06	13-Dec-06
11	59	PM	12-Jun-06	13-Jun-06	19-Jun-06	26-Jun-06		4-Jul-06	3-Oct-06	2-Jan-07
11	60	Combination	12-Jun-06	13-Jun-06	19-Jun-06	26-Jun-06		30-Jun-06	28-Sep-06	18-Dec-06
11	61	PM	12-Jun-06	13-Jun-06	19-Jun-06	26-Jun-06		4-Jul-06	3-Oct-06	2-Jan-07
11	62	SSG	15-Jun-06	16-Jun-06	22-Jun-06	29-Jun-06	6-Jul-06	16-Jul-06	25-Oct-06	11-Jan-07
11	63	SSG	19-Jun-06	20-Jun-06	26-Jun-06	3-Jul-06	10-Jul-06	20-Jul-06	3-Oct-06	28-Dec-06
11	64	SSG	21-Jun-06	22-Jun-06	28-Jun-06	5-Jul-06	12-Jul-06	22-Jul-06	29-Sep-06	14-Dec-06
11	65	PM	22-Jun-06	23-Jun-06	29-Jun-06	6-Jul-06		14-Jul-06		
11	66	Combination	19-Jun-06	23-Jun-06	29-Jun-06	6-Jul-06		10-Jul-06	26-Sep-06	11-Dec-06
11	67	PM	30-Jun-06	1-Jul-06	7-Jul-06	14-Jul-06		22-Jul-06	3-Oct-06	2-Jan-07
11	68	SSG	7-Jul-06	8-Jul-06	14-Jul-06	21-Jul-06	28-Jul-06	7-Aug-06	11-Oct-06	9-Jan-07
11	69	Combination	14-Jul-06	15-Jul-06	21-Jul-06	28-Jul-06		1-Aug-06	9-Oct-06	13-Dec-06
11	70	Combination	14-Jul-06	15-Jul-06	21-Jul-06	28-Jul-06		1-Aug-06	9-Oct-06	2-Jan-07
11	71	Combination	21-Jul-06	22-Jul-06	28-Jul-06	4-Aug-06		8-Aug-06	12-Oct-06	9-Jan-07
11	72	SSG	21-Jul-06	22-Jul-06	28-Jul-06	4-Aug-06			9-Nov-06	7-Feb-07
11	73	PM	24-Jul-06	25-Jul-06	31-Jul-06	7-Aug-06		15-Aug-06	24-Oct-06	2-Apr-07
11	74	PM	27-Jul-06	28-Jul-06	3-Aug-06	10-Aug-06		18-Aug-06	25-Oct-06	23-Jan-07
11	75	Combination	27-Jul-06	28-Jul-06	3-Aug-06	10-Aug-06		14-Aug-06	31-Oct-06	9-Jan-07
11	76	PM	30-Jul-06	31-Jul-06	6-Aug-06	13-Aug-06		21-Aug-06	30-Oct-06	29-Jan-07
11	77	PM	29-Jul-06	31-Jul-06	6-Aug-06	13-Aug-06		21-Aug-06	30-Oct-06	29-Jan-07
11	78	SSG	30-Jul-06	31-Jul-06	6-Aug-06	13-Aug-06	20-Aug-06	30-Aug-06	30-Oct-06	29-Jan-07
11	79	PM	30-Jul-06	31-Jul-06	6-Aug-06	13-Aug-06		21-Aug-06	30-Oct-06	30-Jan-07
11	80	PM	2-Aug-06	3-Aug-06	9-Aug-06	16-Aug-06		24-Aug-06	23-Nov-06	17-Mar-07
11	81	Combination	5-Aug-06	6-Aug-06	12-Aug-06	19-Aug-06		23-Aug-06	7-Nov-06	6-Feb-07
11	82	SSG	13-Aug-06	15-Aug-06	21-Aug-06	28-Aug-06	4-Sep-06	14-Sep-06	13-Nov-06	5-Feb-07
11	83	SSG	14-Aug-06	16-Aug-06	22-Aug-06	29-Aug-06	5-Sep-06	15-Sep-06	13-Nov-06	13-Feb-07
11	84	SSG	17-Aug-06	18-Aug-06	24-Aug-06	31-Aug-06	7-Sep-06	17-Sep-06	15-Nov-06	14-Feb-07
11	85	SSG	24-Aug-06	25-Aug-06	31-Aug-06	7-Sep-06	14-Sep-06	24-Sep-06	21-Nov-06	
11	86	Combination	28-Aug-06	29-Aug-06	4-Sep-06	11-Sep-06		15-Sep-06	13-Nov-06	5-Feb-07
11	87	Combination	28-Aug-06	29-Aug-06	4-Sep-06	11-Sep-06		15-Sep-06	14-Nov-06	5-Feb-07
11	88	PM	28-Aug-06	29-Aug-06	4-Sep-06	11-Sep-06		19-Sep-06	21-Nov-06	27-Feb-07
11	89	Combination	31-Aug-06	2-Sep-06	8-Sep-06	15-Sep-06		19-Sep-06	17-Nov-06	15-Mar-07
11	90	Combination	1-Sep-06	2-Sep-06	8-Sep-06	15-Sep-06		19-Sep-06		

Centre	Patient	Treatment	Assessment Date							
Number	Number		Baseline	Day 1	Day 7	Day 14	Day 21	EOT	3 Mon FU	6 Mon FU
11	91	PM	16-Dec-06	18-Dec-06	24-Dec-06	31-Dec-06		8-Jan-07	5-Mar-07	18-Jun-07
11	92	Combination	30-Dec-06	31-Dec-06	6-Jan-07	13-Jan-07		17-Jan-07	30-Mar-07	27-Jun-07
11	93	SSG	1-Jan-07	2-Jan-07	8-Jan-07	15-Jan-07	22-Jan-07	1-Feb-07	9-May-07	7-Aug-07
11	94	SSG	3-Jan-07	4-Jan-07	10-Jan-07	17-Jan-07	24-Jan-07	3-Feb-07	2-Apr-07	25-Jun-07
11	95	SSG	3-Jan-07	4-Jan-07	10-Jan-07	17-Jan-07	24-Jan-07	3-Feb-07	3-Apr-07	23-Jun-07
11	96	Combination	19-Jan-07	20-Jan-07	26-Jan-07	2-Feb-07		6-Feb-07	23-Apr-07	6-Sep-07
11	97	Combination	19-Jan-07	20-Jan-07	26-Jan-07	2-Feb-07		6-Feb-07	23-Apr-07	25-Jul-07
11	98	PM	23-Jan-07	24-Jan-07	30-Jan-07	6-Feb-07		14-Feb-07	25-Apr-07	26-Jul-07
11	99	SSG	25-Jan-07	26-Jan-07	1-Feb-07	8-Feb-07	15-Feb-07	25-Feb-07	30-Apr-07	30-Jul-07
11	100	PM	25-Jan-07	26-Jan-07	1-Feb-07	8-Feb-07		16-Feb-07		
11	101	PM	2-Feb-07	3-Feb-07	9-Feb-07	16-Feb-07		24-Feb-07	24-May-07	6-Nov-07
11	102	Combination	2-Feb-07	3-Feb-07	9-Feb-07	16-Feb-07		20-Feb-07	1-May-07	30-Jul-07
11	103	Combination	6-Feb-07	7-Feb-07	13-Feb-07	20-Feb-07		24-Feb-07	7-May-07	10-Aug-07
11	104	PM	9-Feb-07	10-Feb-07	16-Feb-07	23-Feb-07		3-Mar-07	16-May-07	27-Aug-07
11	105	SSG	23-Feb-07	24-Feb-07	2-Mar-07	9-Mar-07	16-Mar-07	26-Mar-07	29-May-07	27-Aug-07
11	106	Combination	24-Feb-07	25-Feb-07	3-Mar-07	10-Mar-07		14-Mar-07	25-May-07	19-Aug-07
11	107	PM	24-Feb-07	25-Feb-07	3-Mar-07	10-Mar-07		18-Mar-07	29-May-07	28-Aug-07
11	108	PM	28-Feb-07	2-Mar-07	8-Mar-07	15-Mar-07		23-Mar-07	21-May-07	27-Aug-07
11	109	SSG	27-Feb-07	3-Mar-07	9-Mar-07	16-Mar-07	23-Mar-07	2-Apr-07	8-May-07	6-Aug-07
11	110	PM	2-Mar-07	3-Mar-07	9-Mar-07	16-Mar-07		24-Mar-07	29-May-07	27-Aug-07
11	111	Combination	23-Mar-07	24-Mar-07	30-Mar-07	6-Apr-07		10-Apr-07	22-Jun-07	24-Sep-07
11	112	Combination	23-Mar-07	24-Mar-07	30-Mar-07	6-Apr-07		10-Apr-07	30-Jul-07	24-Sep-07
11	113	SSG	23-Mar-07	28-Mar-07	3-Apr-07	10-Apr-07	17-Apr-07	27-Apr-07	29-Jun-07	
11	114	SSG	28-Mar-07	30-Mar-07	5-Apr-07	12-Apr-07	19-Apr-07	29-Apr-07	2-Jul-07	5-Oct-07
11	115	Combination	30-Mar-07	31-Mar-07	6-Apr-07	13-Apr-07		17-Apr-07	7-Jul-07	21-Sep-07
11	116	SSG	30-Mar-07	31-Mar-07	6-Apr-07	13-Apr-07	20-Apr-07	30-Apr-07	31-Jul-07	1-Nov-07
11	117	PM	3-Apr-07	4-Apr-07	10-Apr-07	17-Apr-07		25-Apr-07	26-Jun-07	
11	118	PM	18-Apr-07	19-Apr-07	25-Apr-07	2-May-07		10-May-07	6-Jul-07	19-Sep-07
11	119	SSG	20-Apr-07	21-Apr-07	27-Apr-07	4-May-07	11-May-07	21-May-07	24-Jul-07	29-Oct-07
11	120	Combination	20-Apr-07	21-Apr-07	27-Apr-07	4-May-07		8-May-07	23-Jul-07	29-Oct-07
11	121	Combination	20-Apr-07	21-Apr-07	27-Apr-07	4-May-07		8-May-07	23-Jul-07	29-Oct-07
11	122	SSG	20-Apr-07	21-Apr-07	27-Apr-07	4-May-07	11-May-07	21-May-07		
11	123	SSG	27-Apr-07	28-Apr-07	4-May-07	11-May-07	18-May-07	28-May-07	30-Jul-07	19-Oct-07
11	124	SSG	20-May-07	21-May-07	27-May-07	3-Jun-07	10-Jun-07	20-Jun-07		7-Apr-08
11	125	SSG	23-May-07	24-May-07	30-May-07	6-Jun-07	13-Jun-07	23-Jun-07	20-Aug-07	19-Nov-07
11	126	Combination	26-May-07	28-May-07	3-Jun-07	10-Jun-07		14-Jun-07		10-Dec-07
11	127	PM	26-May-07	28-May-07	3-Jun-07	10-Jun-07		18-Jun-07	27-Aug-07	30-Oct-07
11	128	Combination	1-Jun-07	2-Jun-07	8-Jun-07	15-Jun-07		19-Jun-07	15-Aug-07	15-Nov-07
11	129	PM	6-Jun-07	7-Jun-07	13-Jun-07	20-Jun-07		28-Jun-07	17-Sep-07	21-Nov-07
11	130	Combination	7-Jun-07	9-Jun-07	15-Jun-07	22-Jun-07		26-Jun-07	17-Sep-07	19-Nov-07
11	131	PM	10-Jun-07	11-Jun-07	17-Jun-07	24-Jun-07		2-Jul-07	8-Oct-07	12-Dec-07
11	132	SSG	15-Jun-07	16-Jun-07	22-Jun-07	29-Jun-07	6-Jul-07	16-Jul-07	5-Sep-07	19-Dec-07
11	133	Combination	1-Jul-07	4-Jul-07	10-Jul-07	17-Jul-07		21-Jul-07	17-Oct-07	2-Jan-08
11	134	PM	10-Jul-07	11-Jul-07	17-Jul-07	24-Jul-07		1-Aug-07	9-Nov-07	8-Jan-08
11	135	PM	12-Jul-07	13-Jul-07	19-Jul-07	26-Jul-07		3-Aug-07	3-Oct-07	31-Dec-07

Centre	Patient	Treatment	Assessment Date							
Number	Number		Baseline	Day 1	Day 7	Day 14	Day 21	EOT	3 Mon FU	6 Mon FU
12	241	Combination	28-Jun-05	29-Jun-05	5-Jul-05	12-Jul-05		16-Jul-05		9-Feb-06
12	242	SSG	28-Jun-05	29-Jun-05	5-Jul-05	12-Jul-05	19-Jul-05	29-Jul-05	24-Oct-05	9-Feb-06
12	243	PM	28-Jun-05	29-Jun-05	5-Jul-05	12-Jul-05		20-Jul-05	4-Nov-05	7-Aug-06
12	244	SSG	7-Jun-05	29-Jun-05	5-Jul-05	12-Jul-05	19-Jul-05	29-Jul-05	29-Oct-05	4-Apr-06
12	245	PM	16-Jul-05	20-Jul-05	25-Jul-05	1-Aug-05		9-Aug-05	18-Nov-05	
12	246	PM	2-Aug-05	4-Aug-05	10-Aug-05	17-Aug-05		25-Aug-05	25-Nov-05	
12	247	PM	12-Sep-05	28-Sep-05	4-Oct-05	11-Oct-05		19-Oct-05	18-Jan-06	17-Apr-06
12	248	PM	12-Sep-05	23-Sep-05	29-Sep-05	6-Oct-05		14-Oct-05	23-Jan-06	17-Apr-06
12	249	Combination	24-Oct-05	25-Oct-05	31-Oct-05	7-Nov-05		11-Nov-05	3-Mar-06	15-May-06
12	250	SSG	24-Oct-05	25-Oct-05	31-Oct-05	7-Nov-05	14-Nov-05	24-Nov-05	22-Feb-06	8-Jul-06
12	251	Combination	27-Oct-05	29-Oct-05	4-Nov-05	11-Nov-05		15-Nov-05	12-Feb-06	15-May-06
12	252	SSG	27-Oct-05	29-Oct-05	4-Nov-05	11-Nov-05	18-Nov-05	28-Nov-05	4-Apr-06	21-Jun-06
12	253	SSG	27-Oct-05	29-Oct-05	4-Nov-05	11-Nov-05	18-Nov-05	28-Nov-05	4-Apr-06	21-Jun-06
12	254	Combination	27-Oct-05	29-Oct-05	4-Nov-05	11-Nov-05		15-Nov-05	12-Feb-06	24-May-06
12	255	Combination	29-Oct-05	2-Nov-05	7-Nov-05	14-Nov-05		18-Nov-05	9-Mar-06	17-May-06
12	256	Combination	14-Nov-05	17-Nov-05	23-Nov-05	30-Nov-05		5-Dec-05	4-Mar-06	6-Jun-06
12	257	SSG	14-Nov-05	17-Nov-05	23-Nov-05	30-Nov-05	7-Dec-05	17-Dec-05	17-Apr-06	14-Jun-06
12	258	SSG	30-Nov-05	6-Dec-05	12-Dec-05	19-Dec-05	26-Dec-05	5-Jan-06		13-Jul-06
12	259	PM	26-Dec-05	31-Dec-05	6-Jan-06	13-Jan-06		23-Jan-06	8-May-06	27-Jul-06
12	260	PM	26-Dec-05	3-Jan-06	9-Jan-06	16-Jan-06		24-Jan-06		11-Jul-06
12	261	Combination	26-Dec-05	3-Jan-06	9-Jan-06	16-Jan-06		20-Jan-06	17-Apr-06	21-Jul-06
12	262	PM	26-Dec-05	3-Jan-06	9-Jan-06	16-Jan-06		23-Jan-06	22-Jun-06	27-Jul-06
12	263	SSG	20-Jan-06	26-Jan-06	1-Feb-06	9-Feb-06	15-Feb-06	25-Feb-06	29-May-06	25-Aug-06
12	264	Combination	7-Feb-06	15-Feb-06	21-Feb-06	28-Feb-06		4-Mar-06	16-Jun-06	1-Sep-06
12	265	SSG	8-Feb-06	15-Feb-06	21-Feb-06	28-Feb-06	7-Mar-06	15-Mar-06		
12	266	PM	8-Feb-06	15-Feb-06	21-Feb-06	28-Feb-06		8-Mar-06	2-Jun-06	4-Oct-06
12	267	SSG	8-Feb-06	15-Feb-06	21-Feb-06	28-Feb-06	7-Mar-06	17-Mar-06	15-Jun-06	14-Sep-06
12	268	PM	6-Mar-06	10-Mar-06	16-Mar-06	23-Mar-06		31-Mar-06	29-Jun-06	26-Sep-06
12	269	Combination	13-Mar-06	16-Mar-06	22-Mar-06	29-Mar-06		2-Apr-06	6-Jul-06	13-Nov-06
12	270	Combination	13-Mar-06	16-Mar-06	22-Mar-06	29-Mar-06		4-Apr-06		9-Oct-06
12	271	SSG	27-Mar-06	31-Mar-06	6-Apr-06	13-Apr-06	20-Apr-06	30-Apr-06	31-Jul-06	31-Oct-06
12	272	PM	4-Apr-06	8-Apr-06	14-Apr-06	21-Apr-06		29-Apr-06	27-Jun-06	13-Nov-06
12	273	SSG	4-Apr-06	8-Apr-06	14-Apr-06	21-Apr-06	28-Apr-06	8-May-06	8-Aug-06	13-Nov-06
12	274	SSG	4-Apr-06	8-Apr-06	14-Apr-06	21-Apr-06	28-Apr-06	8-May-06	7-Aug-06	13-Nov-06
12	275	Combination	4-Apr-06	8-Apr-06	14-Apr-06	21-Apr-06		25-Apr-06	24-Jul-06	24-Oct-06
12	276	PM	4-Apr-06	8-Apr-06	14-Apr-06	21-Apr-06		29-Apr-06	19-Jul-06	29-Oct-06
12	277	Combination	17-Apr-06	21-Apr-06	27-Apr-06	4-May-06		8-May-06	4-Aug-06	13-Nov-06
12	278	SSG	17-Apr-06	21-Apr-06	27-Apr-06	4-May-06	11-May-06	21-May-06	17-Aug-06	
12	279	PM	17-Apr-06	21-Apr-06	27-Apr-06	4-May-06		12-May-06	9-Aug-06	13-Nov-06
12	280	SSG	17-Apr-06	21-Apr-06	27-Apr-06	4-May-06	21-May-06	21-May-06	25-Aug-06	21-Nov-06
12	281	Combination	17-Apr-06	21-Apr-06	27-Apr-06	4-May-06		8-May-06	7-Aug-06	21-Dec-06
12	282	PM	27-Apr-06	28-Apr-06	4-May-06	11-May-06		19-May-06	25-Aug-06	18-Dec-06
12	283	PM	10-May-06	11-May-06	17-May-06	24-May-06		1-Jun-06	25-Aug-06	29-Dec-06
12	284	Combination	18-May-06	20-May-06	26-May-06	2-Jun-06		6-Jun-06	4-Sep-06	5-Dec-06
12	285	Combination	18-May-06	20-May-06	26-May-06	2-Jun-06		6-Jun-06	4-Sep-06	5-Dec-06

Centre	Patient	Treatment	Assessment Date							
Number	Number		Baseline	Day 1	Day 7	Day 14	Day 21	EOT	3 Mon FU	6 Mon FU
12	286	PM	21-Dec-06	2-Jan-07	8-Jan-07	15-Jan-07		23-Jan-07	30-Apr-07	4-Sep-07
12	287	SSG	21-Dec-06	2-Jan-07	8-Jan-07	15-Jan-07	22-Jan-07	1-Feb-07	16-Jun-07	7-Aug-07
12	288	Combination	26-Dec-06	2-Jan-07	8-Jan-07	15-Jan-07		19-Jan-07	20-Apr-07	24-Jul-07
12	289	Combination	26-Dec-06	2-Jan-07	8-Jan-07	15-Jan-07		19-Jan-07	30-Apr-07	3-Sep-07
12	290	SSG	4-Jan-07	6-Jan-07	12-Jan-07	19-Jan-07	26-Jan-07	5-Feb-07		5-Sep-07
12	291	SSG	8-Jan-07	11-Jan-07	17-Jan-07	24-Jan-07	31-Jan-07	10-Feb-07	30-Apr-07	3-Sep-07
12	292	PM	15-Jan-07	18-Jan-07	24-Jan-07	31-Jan-07		7-Feb-07		21-Aug-07
12	293	SSG	22-Jan-07	24-Jan-07	30-Jan-07	6-Feb-07	13-Feb-07	23-Feb-07		5-Sep-07
12	294	Combination	19-Feb-07	22-Feb-07	28-Feb-07	7-Mar-07		11-Mar-07	11-Jun-07	7-Sep-07
12	295	Combination	11-Mar-07	16-Mar-07	22-Mar-07	29-Mar-07		2-Apr-07		20-Sep-07
12	296	PM	4-Apr-07	12-Apr-07	18-Apr-07	25-Apr-07		3-May-07	31-Jul-07	30-Oct-07
12	297	SSG	4-Apr-07	12-Apr-07	18-Apr-07	25-Apr-07				
12	298	PM	16-Apr-07	21-Apr-07	27-Apr-07	4-May-07		14-May-07	8-Aug-07	9-Nov-07
12	299	Combination	1-Jun-07	5-Jun-07	11-Jun-07	18-Jun-07		22-Jun-07		31-Dec-07
12	300	PM	1-Jun-07	5-Jun-07	11-Jun-07	18-Jun-07		26-Jun-07	25-Sep-07	24-Dec-07
12	301	SSG	2-Jun-07	5-Jun-07	11-Jun-07	18-Jun-07	25-Jun-07	5-Jul-07	8-Oct-07	4-Feb-08
12	302	SSG	2-Jun-07	5-Jun-07	11-Jun-07	18-Jun-07	25-Jun-07	5-Jul-07	1-Oct-07	26-Dec-07
12	303	PM	2-Jun-07	5-Jun-07	11-Jun-07	18-Jun-07		26-Jun-07	1-Oct-07	26-Dec-07
12	304	Combination	2-Jun-07	5-Jun-07	11-Jun-07	18-Jun-07		22-Jun-07	1-Oct-07	26-Dec-07
12	305	PM	2-Jun-07	5-Jun-07	11-Jun-07	18-Jun-07		26-Jun-07	1-Oct-07	26-Dec-07
12	306	Combination	2-Jun-07	5-Jun-07	11-Jun-07	18-Jun-07		22-Jun-07	1-Oct-07	26-Dec-07
12	307	Combination	13-Jun-07	15-Jun-07	21-Jun-07	28-Jun-07		2-Jul-07	1-Oct-07	26-Dec-07
12	308	PM	13-Jun-07	15-Jun-07	21-Jun-07	28-Jun-07		6-Jul-07	8-Oct-07	26-Dec-07
12	309	PM	16-Jun-07	16-Jun-07	22-Jun-07	29-Jun-07		7-Jul-07	1-Oct-07	26-Dec-07
12	310	SSG	16-Jun-07	16-Jun-07	22-Jun-07	29-Jun-07	6-Jul-07	16-Jul-07	18-Oct-07	4-Feb-08
12	311	SSG	4-Jul-07	5-Jul-07	11-Jul-07	18-Jul-07	25-Jul-07	6-Aug-07	1-Nov-07	11-Mar-08
12	312	Combination	10-Jul-07	12-Jul-07	18-Jul-07	25-Jul-07		30-Jul-07	27-Oct-07	19-Mar-08
12	313	PM	31-Jul-07	2-Aug-07	8-Aug-07	15-Aug-07		23-Aug-07	22-Nov-07	21-Feb-08
12	314	SSG	31-Jul-07	7-Aug-07	13-Aug-07	20-Aug-07	27-Aug-07	6-Sep-07	4-Dec-07	4-Mar-08
12	315	Combination	20-Aug-07	29-Aug-07	4-Sep-07	11-Sep-07		17-Sep-07	8-Jan-08	11-Aug-08
12	316	SSG	20-Aug-07	29-Aug-07	4-Sep-07	11-Sep-07		25-Sep-07	28-Dec-07	1-Apr-08
12	317	SSG	21-Aug-07	29-Aug-07	4-Sep-07	11-Sep-07	18-Sep-07	28-Sep-07		22-Apr-08
12	318	Combination	27-Aug-07	28-Aug-07	4-Sep-07	11-Sep-07		17-Sep-07	17-Dec-07	15-Mar-08
12	319	Combination	27-Aug-07	28-Aug-07	4-Sep-07	11-Sep-07		17-Sep-07	20-Dec-07	14-Mar-08
12	320	Combination	4-Sep-07	6-Sep-07	12-Sep-07	19-Sep-07		23-Sep-07	24-Dec-07	19-Mar-08
12	321	Combination	4-Sep-07	6-Sep-07	12-Sep-07	19-Sep-07		23-Sep-07	24-Dec-07	24-Mar-08
12	322	PM	11-Sep-07	13-Sep-07	19-Sep-07	26-Sep-07		4-Oct-07	16-Jan-08	19-Apr-08
12	323	SSG	18-Sep-07	20-Sep-07	26-Sep-07	3-Oct-07	10-Oct-07	20-Oct-07	18-Jan-08	16-Apr-08
12	324	SSG	18-Sep-07	20-Sep-07	26-Sep-07	3-Oct-07	10-Oct-07	20-Oct-07	18-Jan-08	16-Apr-08
12	325	PM	18-Sep-07	27-Sep-07	3-Oct-07	10-Oct-07		18-Oct-07	15-Jan-08	16-Apr-08
12	326	PM	2-Oct-07	3-Oct-07	9-Oct-07	16-Oct-07		24-Oct-07	23-Jan-08	19-Apr-08
12	327	Combination	2-Oct-07	3-Oct-07	9-Oct-07	16-Oct-07		20-Oct-07	18-Jan-08	16-Apr-08
12	328	PM	3-Oct-07	5-Oct-07	11-Oct-07	18-Oct-07		26-Oct-07	24-Jan-08	14-Apr-08
12	329	SSG	8-Oct-07	10-Oct-07	16-Oct-07	23-Oct-07	30-Oct-07	9-Nov-07	5-Feb-08	6-May-08
12	330	PM	8-Oct-07	10-Oct-07	16-Oct-07	23-Oct-07		31-Oct-07	4-Feb-08	6-May-08

Centre	Patient	Treatment	Assessment Date							
Number	Number		Baseline	Day 1	Day 7	Day 14	Day 21	EOT	3 Mon FU	6 Mon FU
23	361	Combination	19-Jan-05	20-Jan-05	26-Jan-05	2-Feb-05		6-Feb-05	5-May-05	10-Aug-05
23	362	PM	18-Jan-05	20-Jan-05	26-Jan-05	2-Feb-05		10-Feb-05	10-May-05	16-Aug-05
23	363	PM	18-Jan-05	20-Jan-05	27-Jan-05	2-Feb-05		10-Feb-05	5-May-05	10-Aug-05
23	364	Combination	18-Jan-05	20-Jan-05	26-Jan-05	2-Feb-05		8-Feb-05	27-Apr-05	19-Aug-05
23	365	SSG	18-Jan-05	20-Jan-05	27-Jan-05	2-Feb-05	10-Feb-05	10-Feb-05	20-May-05	30-Aug-05
23	366	Combination	27-Jan-05	1-Feb-05	7-Feb-05	14-Feb-05		18-Feb-05	20-May-05	19-Aug-05
23	367	SSG	28-Jan-05	1-Feb-05	8-Feb-05	14-Feb-05	22-Feb-05	3-Mar-05	8-Jun-05	23-Sep-05
23	368	PM	27-Jan-05	1-Feb-05	7-Feb-05	14-Feb-05		22-Feb-05	26-May-05	5-Sep-05
23	369	Combination	3-Feb-05	4-Feb-05	10-Feb-05	17-Feb-05		21-Feb-05	26-May-05	30-Aug-05
23	370	SSG	27-Jan-05	4-Feb-05	10-Feb-05	17-Feb-05	24-Feb-05	6-Mar-05	8-Jun-05	23-Sep-05
23	371	PM	2-Feb-05	4-Feb-05	10-Feb-05	17-Feb-05		25-Feb-05	26-May-05	6-Sep-05
23	372	PM	9-Feb-05	11-Feb-05	17-Feb-05	24-Feb-05		4-Mar-05	6-Jun-05	9-Sep-05
23	373	SSG	16-Feb-05	18-Feb-05	24-Feb-05	3-Mar-05	10-Mar-05	21-Mar-05	6-Jul-05	24-Oct-05
23	374	SSG	17-Feb-05	24-Feb-05	2-Mar-05	9-Mar-05	16-Mar-05	28-Mar-05	27-Jun-05	29-Sep-05
23	375	Combination	23-Feb-05	25-Feb-05	3-Mar-05	10-Mar-05		14-Mar-05	15-Jun-05	23-Sep-05
23	376	SSG	24-Feb-05	1-Mar-05	7-Mar-05	14-Mar-05	21-Mar-05	31-Mar-05	29-Jun-05	28-Sep-05
23	377	Combination	7-Mar-05	9-Mar-05	17-Mar-05					23-Sep-05
23	378	Combination	7-Mar-05	9-Mar-05	15-Mar-05	22-Mar-05		26-Mar-05	6-Jul-05	23-Sep-05
23	379	PM	23-Feb-05	10-Mar-05	17-Mar-05	23-Mar-05		31-Mar-05		30-Sep-05
23	380	SSG	8-Mar-05	15-Mar-05	21-Mar-05	28-Mar-05	4-Apr-05	14-Apr-05	19-Jul-05	13-Oct-05
23	381	PM	14-Mar-05	15-Mar-05	21-Mar-05	28-Mar-05		5-Apr-05	12-Jul-05	16-Nov-05
23	382	Combination	17-Mar-05	18-Mar-05	24-Mar-05	31-Mar-05		4-Apr-05	12-Jul-05	29-Sep-05
23	383	PM	23-Mar-05	5-Apr-05	11-Apr-05	18-Apr-05		26-Apr-05	29-Sep-05	24-Oct-05
23	384	SSG	23-Mar-05	5-Apr-05	11-Apr-05	18-Apr-05	25-Apr-05	5-May-05	28-Sep-05	16-Nov-05
23	385	SSG	23-Mar-05	7-Apr-05	13-Apr-05	20-Apr-05	27-Apr-05	7-May-05	10-Aug-05	11-Nov-05
23	386	Combination	14-Apr-05	19-Apr-05	25-Apr-05	11-May-05		16-May-05	19-Aug-05	11-Nov-05
23	387	Combination	14-Apr-05	19-Apr-05	25-Apr-05	2-May-05		6-May-05	10-Aug-05	11-Nov-05
23	388	PM	14-Apr-05	19-Apr-05	25-Apr-05	4-May-05		10-May-05	28-Sep-05	2-Dec-05
23	389	SSG	14-Apr-05	20-Apr-05	26-Apr-05	4-May-05	10-May-05	20-May-05	6-Sep-05	9-Dec-05
23	390	PM	14-Apr-05	20-Apr-05	26-Apr-05	3-May-05		11-May-05	10-Aug-05	2-Dec-05
23	391	Combination	21-Apr-05	22-Apr-05	28-Apr-05	5-May-05		9-May-05	19-Aug-05	16-Nov-05
23	392	Combination	21-Apr-05	22-Apr-05	28-Apr-05	5-May-05			22-Jul-05	29-Sep-05
23	393	SSG	20-Apr-05	22-Apr-05	28-Apr-05	5-May-05	12-May-05	22-May-05	19-Aug-05	16-Nov-05
23	394	PM	14-Apr-05	26-Apr-05	2-May-05	9-May-05		17-May-05	19-Aug-05	16-Nov-05
23	395	PM	28-Apr-05	29-Apr-05	5-May-05	13-May-05		20-May-05	1-Jul-05	14-Dec-05
23	396	SSG	10-May-05	13-May-05	19-May-05	26-May-05	2-Jun-05	12-Jun-05	16-Sep-05	14-Dec-05
23	397	SSG	10-May-05	18-May-05	19-May-05	26-May-05	2-Jun-05	12-Jun-05	16-Sep-05	14-Dec-05
23	398	Combination	13-May-05	18-May-05	24-May-05	31-May-05		6-Jun-05	30-Sep-05	14-Dec-05
23	399	PM	12-May-05	18-May-05	24-May-05	31-May-05		8-Jun-05	14-Oct-05	14-Dec-05
23	400	PM	2-Jun-05	3-Jun-05	9-Jun-05	16-Jun-05		24-Jun-05	14-Oct-05	12-Jan-06
23	401	Combination	30-May-05	3-Jun-05	9-Jun-05	16-Jun-05		20-Jun-05	19-Sep-05	20-Dec-05
23	402	Combination	30-May-05	3-Jun-05	9-Jun-05	16-Jun-05		20-Jun-05	23-Sep-05	20-Dec-05
23	403	SSG	30-May-05	4-Jun-05	10-Jun-05	17-Jun-05	24-Jun-05	4-Jul-05	30-Sep-05	12-Jan-06
23	404	SSG	30-May-05	4-Jun-05	10-Jun-05	17-Jun-05	24-Jun-05	4-Jul-05		12-Jan-06
23	405	PM	9-Jun-05	10-Jun-05	16-Jun-05	24-Jun-05		1-Jul-05	29-Sep-05	5-Jan-06

Centre Number	Patient Number	Treatment	Assessment Date Baseline	Day 1	Day 7	Day 14	Day 21	EOT	3 Mon FU	6 Mon FU
34	451	Combination	16-Nov-04	17-Nov-04	23-Nov-04	30-Nov-04		4-Dec-04		3-Jun-05
34	452	PM	16-Nov-04	17-Nov-04	23-Nov-04	30-Nov-04		8-Dec-04		
34	453	PM	17-Nov-04	18-Nov-04	24-Nov-04	1-Dec-04		9-Dec-04		
34	454	Combination	18-Nov-04	21-Nov-04	27-Nov-04	4-Dec-04		8-Dec-04		7-Jun-05
34	455	PM	18-Nov-04	19-Nov-04	25-Nov-04	2-Dec-04		10-Dec-04		
34	456	SSG	18-Nov-04	19-Nov-04	25-Nov-04	2-Dec-04	9-Dec-04	19-Dec-04	25-Feb-05	
34	457	Combination	18-Nov-04	19-Nov-04	25-Nov-04	2-Dec-04		6-Dec-04		
34	458	PM	19-Nov-04	20-Nov-04	26-Nov-04	3-Dec-04		11-Dec-04	16-Jan-05	
34	459	Combination	20-Nov-04	20-Nov-04	26-Nov-04	3-Dec-04		7-Dec-04	7-Jan-05	3-Jun-05
34	460	PM	22-Nov-04	24-Nov-04	30-Nov-04	7-Dec-04		15-Dec-04		
34	461	SSG	24-Nov-04	26-Nov-04	2-Dec-04	9-Dec-04				
34	462	SSG	24-Nov-04	25-Nov-04	1-Dec-04	8-Dec-04	15-Dec-04	25-Dec-04		7-Jun-05
34	463	Combination	25-Nov-04	27-Nov-04	3-Dec-04	10-Dec-04		14-Dec-04		16-Jun-05
34	464	SSG	26-Nov-04	27-Nov-04	3-Dec-04	10-Dec-04	17-Dec-04	27-Dec-04		2-Jun-05
34	465	SSG	27-Nov-04	29-Nov-04	5-Dec-04	12-Dec-04	19-Dec-04	29-Dec-04		3-Jun-05
34	466	Combination	27-Nov-04	30-Nov-04	6-Dec-04	13-Dec-04		17-Dec-04		
34	467	PM	28-Nov-04	29-Nov-04	5-Dec-04	12-Dec-04		20-Dec-04	5-Feb-05	
34	468	Combination	28-Nov-04	29-Nov-04	5-Dec-04	12-Dec-04		16-Dec-04		4-Jun-05
34	469	SSG	28-Nov-04	29-Nov-04	5-Dec-04	12-Dec-04	19-Dec-04	29-Dec-04		10-Jun-05
34	470	Combination	29-Nov-04	29-Nov-04	5-Dec-04	12-Dec-04		16-Dec-04		7-Jun-05
34	471	PM	29-Nov-04	29-Nov-04	5-Dec-04	12-Dec-04		20-Dec-04		
34	472	SSG	1-Dec-04	1-Dec-04	7-Dec-04	14-Dec-04	21-Dec-04	31-Dec-04		9-Jun-05
34	473	PM	1-Dec-04	3-Dec-04	9-Dec-04	16-Dec-04		24-Dec-04		
34	474	Combination	3-Dec-04	4-Dec-04	10-Dec-04	17-Dec-04		21-Dec-04		1-Jun-05
34	475	PM	4-Dec-04	5-Dec-04	11-Dec-04	18-Dec-04		26-Dec-04		
34	476	PM	4-Dec-04	5-Dec-04	11-Dec-04	18-Dec-04		26-Dec-04	10-Jan-05	
34	477	SSG	6-Dec-04	6-Dec-04	12-Dec-04	19-Dec-04	26-Dec-04	5-Jan-05		5-Jun-05
34	478	Combination	6-Dec-04	7-Dec-04	13-Dec-04	20-Dec-04		24-Dec-04		5-Jun-05
34	479	SSG	8-Dec-04	8-Dec-04	14-Dec-04	21-Dec-04	28-Dec-04	7-Jan-05		1-Jun-05
34	480	SSG	8-Dec-04	9-Dec-04	15-Dec-04	22-Dec-04	29-Dec-04	8-Jan-05		13-Jun-05
34	481	Combination	9-Dec-04	9-Dec-04	15-Dec-04	22-Dec-04		26-Dec-04		3-Jun-05
34	482	Combination	9-Dec-04	10-Dec-04	16-Dec-04	23-Dec-04		27-Dec-04		5-Jun-05
34	483	PM	9-Dec-04	10-Dec-04	16-Dec-04	23-Dec-04		31-Dec-04		31-May-05
34	484	PM	10-Dec-04	12-Dec-04	18-Dec-04					
34	485	Combination	10-Dec-04	12-Dec-04	18-Dec-04	25-Dec-04		29-Dec-04		6-Jun-05
34	486	Combination	11-Dec-04	13-Dec-04	19-Dec-04	26-Dec-04		30-Dec-04		7-Jun-05
34	487	SSG	12-Dec-04	13-Dec-04	19-Dec-04	26-Dec-04	2-Jan-05	12-Jan-05		2-Jun-05
34	488	SSG	13-Dec-04	14-Dec-04	20-Dec-04	27-Dec-04	3-Jan-05	13-Jan-05	17-Feb-05	7-Jun-05
34	489	SSG	15-Dec-04	16-Dec-04	22-Dec-04	29-Dec-04	5-Jan-05	15-Jan-05		3-Jun-05
34	490	PM	16-Dec-04	16-Dec-04	22-Dec-04	29-Dec-04		6-Jan-05		15-Jun-05
34	491	PM	16-Dec-04	17-Dec-04	23-Dec-04	30-Dec-04		7-Jan-05		
34	492	PM	16-Dec-04	19-Dec-04	25-Dec-04	1-Jan-05		9-Jan-05		2-Jun-05
34	493	SSG	16-Dec-04	17-Dec-04	23-Dec-04	30-Dec-04	6-Jan-05	16-Jan-05		
34	494	Combination	17-Dec-04	17-Dec-04	23-Dec-04	30-Dec-04		3-Jan-05		3-Jun-05
34	495	SSG	17-Dec-04	18-Dec-04	24-Dec-04	31-Dec-04	7-Jan-05	17-Jan-05		2-Jun-05

Centre	Patient	Treatment	Assessment Date							
Number	Number		Baseline	Day 1	Day 7	Day 14	Day 21	EOT	3 Mon FU	6 Mon FU
34	496	SSG	18-Dec-04	19-Dec-04	25-Dec-04	1-Jan-05	8-Jan-05	18-Jan-05		2-Jun-05
34	497	PM	20-Dec-04	21-Dec-04	27-Dec-04	3-Jan-05		11-Jan-05	12-Feb-05	
34	498	SSG	20-Dec-04	21-Dec-04	27-Dec-04	3-Jan-05	10-Jan-05	20-Jan-05		6-Jun-05
34	499	Combination	20-Dec-04	21-Dec-04	27-Dec-04	3-Jan-05		7-Jan-05		6-Jun-05
34	500	PM	20-Dec-04	22-Dec-04	28-Dec-04	4-Jan-05		12-Jan-05		7-Jun-05
34	501	Combination	21-Dec-04	22-Dec-04	28-Dec-04	4-Jan-05		8-Jan-05		8-Jun-05
34	502	Combination	22-Dec-04	23-Dec-04	29-Dec-04	5-Jan-05		9-Jan-05		8-Jun-05
34	503	PM	24-Dec-04	25-Dec-04	31-Dec-04	7-Jan-05		15-Jan-05		
34	504	Combination	26-Dec-04	27-Dec-04	2-Jan-05	9-Jan-05		13-Jan-05		
34	505	SSG	27-Dec-04	28-Dec-04	3-Jan-05	10-Jan-05	17-Jan-05	27-Jan-05		1-Jun-05
34	506	SSG	30-Dec-04	31-Dec-04	6-Jan-05	13-Jan-05	20-Jan-05	30-Jan-05		10-Jun-05
34	507	SSG	30-Dec-04	31-Dec-04	6-Jan-05	13-Jan-05	20-Jan-05	30-Jan-05		31-May-05
34	508	PM	30-Dec-04	31-Dec-04	6-Jan-05	13-Jan-05		21-Jan-05		
34	509	PM	31-Dec-04	2-Jan-05	8-Jan-05	15-Jan-05		23-Jan-05		
34	510	Combination	31-Dec-04	1-Jan-05	7-Jan-05	14-Jan-05		18-Jan-05		13-Jun-05
34	511	Combination	31-Dec-04	1-Jan-05	7-Jan-05	14-Jan-05		18-Jan-05		10-Jun-05
34	512	PM	1-Jan-05	2-Jan-05	8-Jan-05	15-Jan-05		23-Jan-05		
34	513	Combination	2-Jan-05	3-Jan-05	9-Jan-05	16-Jan-05		20-Jan-05	28-Feb-05	31-May-05
34	514	SSG	3-Jan-05	4-Jan-05	10-Jan-05	17-Jan-05	24-Jan-05	3-Feb-05		7-Jun-05
34	515	SSG	4-Jan-05	5-Jan-05	11-Jan-05	18-Jan-05	25-Jan-05	4-Feb-05	13-May-05	
34	516	PM	5-Jan-05	6-Jan-05	12-Jan-05	19-Jan-05		27-Jan-05		6-Jun-05
34	517	PM	5-Jan-05	6-Jan-05	12-Jan-05	19-Jan-05		27-Jan-05		
34	518	SSG	6-Jan-05	9-Jan-05	14-Jan-05	21-Jan-05	29-Jan-05	8-Feb-05		3-Jun-05
34	519	PM	6-Jan-05	7-Jan-05	13-Jan-05	20-Jan-05		28-Jan-05	3-Apr-05	
34	520	SSG	10-Jan-05	11-Jan-05	17-Jan-05	24-Jan-05	31-Jan-05	10-Feb-05		5-Jun-05
34	521	PM	10-Jan-05	11-Jan-05	17-Jan-05	24-Jan-05		1-Feb-05		
34	522	Combination	10-Jan-05	11-Jan-05	17-Jan-05	24-Jan-05		28-Jan-05		
34	523	SSG	12-Jan-05	14-Jan-05	20-Jan-05	27-Jan-05	3-Feb-05	13-Feb-05		1-Jun-05
34	524	Combination	12-Jan-05	13-Jan-05	19-Jan-05	26-Jan-05		30-Jan-05		5-Jun-05
34	525	Combination	12-Jan-05	13-Jan-05	19-Jan-05	26-Jan-05		30-Jan-05		9-Jun-05
34	526	SSG	13-Jan-05	14-Jan-05	20-Jan-05	27-Jan-05	3-Feb-05	13-Feb-05		4-Jun-05
34	527	Combination	14-Jan-05	15-Jan-05	21-Jan-05	28-Jan-05		1-Feb-05		31-May-05
34	528	PM	16-Jan-05	17-Jan-05	23-Jan-05	30-Jan-05		7-Feb-05		
34	529	Combination	17-Jan-05	18-Jan-05	24-Jan-05	31-Jan-05		4-Feb-05		14-Jun-05
34	530	Combination	19-Jan-05	20-Jan-05	26-Jan-05	2-Feb-05		6-Feb-05		14-Jun-05
34	531	SSG	20-Jan-05	21-Jan-05	27-Jan-05	3-Feb-05	10-Feb-05	20-Feb-05		
34	532	Combination	20-Jan-05	21-Jan-05	27-Jan-05	3-Feb-05		7-Feb-05		3-Jun-05
34	533	SSG	22-Jan-05	23-Jan-05	29-Jan-05	5-Feb-05	12-Feb-05	22-Feb-05		9-Jun-05
34	534	PM	23-Jan-05	24-Jan-05	30-Jan-05	6-Feb-05		14-Feb-05		
34	535	PM	23-Jan-05	24-Jan-05	30-Jan-05	6-Feb-05		14-Feb-05	25-Mar-05	
34	536	PM	24-Jan-05	25-Jan-05	31-Jan-05	7-Feb-05		15-Feb-05		6-May-06
34	537	SSG	24-Jan-05	25-Jan-05	31-Jan-05	7-Feb-05	15-Feb-05	24-Feb-05		31-May-05
34	538	PM	24-Jan-05	26-Jan-05	1-Feb-05	8-Feb-05		16-Feb-05		
34	539	Combination	25-Jan-05	26-Jan-05	1-Feb-05	8-Feb-05		12-Feb-05		5-Jun-05
34	540	SSG	26-Jan-05	27-Jan-05	2-Feb-05	9-Feb-05	16-Feb-05	26-Feb-05		31-May-05

Centre	Patient	Treatment	Assessment Date							
Number	Number		Baseline	Day 1	Day 7	Day 14	Day 21	EOT	3 Mon FU	6 Mon FU
35	646	PM	26-Jan-05	28-Jan-05	3-Feb-05	10-Feb-05		18-Feb-05		5-Dec-05
35	647	PM	29-Jan-05	30-Jan-05	5-Feb-05	12-Feb-05		20-Feb-05	21-May-05	15-Aug-05
35	648	Combination	30-Jan-05	30-Jan-05	5-Feb-05			16-Feb-05	21-May-05	25-Aug-05
35	649	Combination	29-Jan-05	30-Jan-05	5-Feb-05			16-Feb-05	18-May-05	12-Sep-05
35	650	SSG	30-Jan-05	30-Jan-05	5-Feb-05	12-Feb-05	19-Feb-05	1-Mar-05	6-Jun-05	12-Sep-05
35	651	SSG	30-Jan-05	30-Jan-05	5-Feb-05	12-Feb-05	19-Feb-05	1-Mar-05	9-Jun-05	14-Sep-05
35	652	PM	31-Jan-05	1-Feb-05	7-Feb-05	14-Feb-05		22-Feb-05		15-Aug-05
35	653	Combination	2-Feb-05	3-Feb-05	9-Feb-05	16-Feb-05		20-Feb-05	24-May-05	25-Aug-05
35	654	SSG	2-Feb-05	3-Feb-05	9-Feb-05	16-Feb-05	23-Feb-05	5-Mar-05	9-Jun-05	19-Sep-05
35	655	Combination	2-Feb-05	3-Feb-05	9-Feb-05	16-Feb-05		20-Feb-05	23-May-05	25-Aug-05
35	656	SSG	1-Feb-05	3-Feb-05	9-Feb-05	16-Feb-05	23-Feb-05	5-Mar-05	6-Jun-05	6-Oct-05
35	657	PM	7-Feb-05	8-Feb-05	14-Feb-05	21-Feb-05		1-Mar-05	9-Jun-05	14-Sep-05
35	658	Combination	7-Feb-05	8-Feb-05	14-Feb-05	21-Feb-05		25-Feb-05	21-May-05	25-Aug-05
35	659	SSG	7-Feb-05	8-Feb-05	14-Feb-05	21-Feb-05	28-Feb-05	10-Mar-05	5-Jun-05	25-Oct-05
35	660	PM	5-Feb-05	10-Feb-05	16-Feb-05	23-Feb-05		3-Mar-05	9-Jun-05	15-Sep-05
35	661	Combination	15-Feb-05	16-Feb-05	22-Feb-05	1-Mar-05		5-Mar-05	9-Jun-05	24-Sep-05
35	662	PM	19-Feb-05	20-Feb-05	26-Feb-05	5-Mar-05		12-Mar-05	9-Jun-05	17-Sep-05
35	663	SSG	19-Feb-05	20-Feb-05	26-Feb-05	5-Mar-05	12-Mar-05	22-Mar-05	22-Jun-05	22-Sep-05
35	664	Combination	19-Feb-05	20-Feb-05	26-Feb-05					10-Oct-05
35	665	SSG	22-Feb-05	23-Feb-05	1-Mar-05	8-Mar-05	15-Mar-05	25-Mar-05	28-Jun-05	25-Oct-05
35	666	SSG	25-Feb-05	26-Feb-05	4-Mar-05	11-Mar-05	18-Mar-05	28-Mar-05	27-Jun-05	25-Sep-05
35	667	Combination	27-Feb-05	28-Feb-05	6-Mar-05	13-Mar-05		17-Mar-05	5-Jun-05	20-Oct-05
35	668	PM	27-Feb-05	28-Feb-05	6-Mar-05	13-Mar-05		21-Mar-05		25-Sep-05
35	669	PM	28-Feb-05	28-Feb-05	6-Mar-05	13-Mar-05		21-Mar-05		
35	670	SSG	2-Mar-05	3-Mar-05	9-Mar-05	16-Mar-05	23-Mar-05	2-Apr-05	4-Jul-05	12-Oct-05
35	671	PM	10-Mar-05	11-Mar-05	17-Mar-05	24-Mar-05		1-Apr-05		
35	672	Combination	10-Mar-05	11-Mar-05	17-Mar-05	24-Mar-05		28-Mar-05		20-Oct-05
35	673	SSG	10-Mar-05	11-Mar-05	17-Mar-05	24-Mar-05	31-Mar-05	10-Apr-05	15-Jul-05	16-Oct-05
35	674	Combination	12-Mar-05	13-Mar-05	19-Mar-05	26-Mar-05		30-Mar-05	10-Jul-05	20-Oct-05
35	675	PM	13-Mar-05	14-Mar-05	20-Mar-05	27-Mar-05		4-Apr-05		7-Dec-05
35	676	PM	19-Mar-05	20-Mar-05	26-Mar-05	2-Apr-05		10-Apr-05	18-Jul-05	20-Oct-05
35	677	SSG	19-Mar-05	20-Mar-05	26-Mar-05					
35	678	Combination	20-Mar-05	22-Mar-05	28-Mar-05	4-Apr-05		8-Apr-05	7-Jul-05	25-Oct-05
35	679	SSG	20-Mar-05	22-Mar-05	28-Mar-05	4-Apr-05	11-Apr-05	21-Apr-05	24-Jul-05	25-Oct-05
35	680	Combination	20-Mar-05	22-Mar-05	28-Mar-05	4-Apr-05		8-Apr-05	10-Jul-05	12-Oct-05
35	681	Combination	21-Mar-05	22-Mar-05	28-Mar-05	4-Apr-05		8-Apr-05	7-Jul-05	20-Oct-05
35	682	PM	21-Mar-05	22-Mar-05	28-Mar-05	4-Apr-05		12-Apr-05		19-Oct-05
35	683	Combination	21-Mar-05	22-Mar-05	28-Mar-05	4-Apr-05		8-Apr-05		29-Oct-05
35	684	SSG	8-Apr-05	9-Apr-05	15-Apr-05	22-Apr-05	29-Apr-05	9-May-05	20-Aug-05	29-Oct-05
35	685	PM	8-Apr-05	9-Apr-05	15-Apr-05	22-Apr-05		30-Apr-05	2-Aug-05	29-Oct-05
35	686	PM	8-Apr-05	13-Apr-05	19-Apr-05	26-Apr-05		4-May-05	7-Aug-05	26-Oct-05
35	687	SSG	12-Apr-05	13-Apr-05	19-Apr-05	26-Apr-05	3-May-05	13-May-05	20-Aug-05	29-Oct-05
35	688	PM	15-Apr-05	16-Apr-05	22-Apr-05	29-Apr-05		7-May-05	18-Aug-05	25-Oct-05
35	689	Combination	15-Apr-05	16-Apr-05	22-Apr-05	29-Apr-05		3-May-05	3-Aug-05	26-Oct-05
35	690	SSG	15-Apr-05	16-Apr-05	22-Apr-05	29-Apr-05	6-May-05	16-May-05	22-Aug-05	29-Oct-05

16.3 PATIENT DATA LISTINGS

16.3.1 Discontinued patients

Appendix 24 Listing of discontinued patients

Centre Number	Patient Number	Treatment	Treatment Start date	End date	Date of last visit	Days on Treatment	Reason for no 6 month follow-up	Serious adverse event / Comment
11	27	PM	19-Sep-05			21	Other	DEATH
11	47	SSG	5-May-06			30	Other	DEATH
11	65	PM	23-Jun-06			21	Lost to follow-up	
11	85	SSG	25-Aug-06			30	Lost to follow-up	
11	90	Combination	2-Sep-06			17	Lost to follow-up	
11	100	PM	26-Jan-07			21	Lost to follow-up	
11	113	SSG	28-Mar-07			30	Lost to follow-up	
11	117	PM	4-Apr-07			21	Lost to follow-up	
11	122	SSG	21-Apr-07			30	Lost to follow-up	
12	245	PM	20-Jul-05			21	Lost to follow-up	
12	265	SSG	15-Feb-06	13-Mar-06		27		ACUTE RENAL FAILURE
12	278	SSG	21-Apr-06			30	Lost to follow-up	
12	297	SSG	12-Apr-07	25-Apr-07		14	Lost to follow-up	PANCREATITIS
34	452	PM	17-Nov-04	7-Dec-04	7-Dec-04	21		Comment: Treatment failure, received rescue
34	453	PM	18-Nov-04	8-Dec-04	8-Dec-04	21		Comment: Treatment failure, received rescue
34	455	PM	19-Nov-04	9-Dec-04	9-Dec-04	21		Comment: Treatment failure, received rescue
34	456	SSG	19-Nov-04			30		Comment: Treatment failure, received rescue
34	457	Combination	19-Nov-04			17	Lost to follow-up	
34	458	PM	20-Nov-04			21		Comment: Treatment failure, received rescue
34	460	PM	24-Nov-04	14-Dec-04	14-Dec-04	21		Comment: Treatment failure, received rescue
34	461	SSG	26-Nov-04	10-Dec-04	10-Dec-04	4		EXTENSIVE EPISTAXIS
34	466	Combination	30-Nov-04	17-Dec-04	17-Dec-04	17		Comment: Treatment failure, received rescue
34	467	PM	29-Nov-04			21		Comment: Treatment failure, received rescue
34	471	PM	29-Nov-04	19-Dec-04	19-Dec-04	21		Comment: Treatment failure, received rescue
34	473	PM	3-Dec-04	23-Dec-04	24-Dec-04	20		Comment: Treatment failure, received rescue
34	475	PM	5-Dec-04	25-Dec-04	25-Dec-04	21		Comment: Treatment failure, received rescue
34	476	PM	5-Dec-04	26-Dec-04	26-Dec-04	21		Comment: Treatment failure, received rescue
34	484	PM	12-Dec-04	18-Dec-04	18-Dec-04	7		Comment: Treatment failure, received rescue
34	491	PM	17-Dec-04	6-Jan-05	7-Jan-05	21		Comment: Treatment failure, received rescue
34	493	SSG	17-Dec-04			30		Comment: lost to follow-up
34	497	PM	21-Dec-04			21		Comment: Treatment failure, received rescue
34	503	PM	25-Dec-04	14-Jan-05	14-Jan-05	21		Comment: Treatment failure, received rescue
34	504	Combination	27-Dec-04	12-Jan-05	13-Jan-05	17		Comment: Treatment failure, received rescue
34	508	PM	31-Dec-04	20-Jan-05	21-Jan-05	21		Comment: Treatment failure, received rescue
34	509	PM	2-Jan-05	22-Jan-05	23-Jan-05	21		Comment: Treatment failure, received rescue
34	512	PM	2-Jan-05	23-Jan-05	24-Jan-05	21		Comment: Treatment failure, received rescue
34	515	SSG	5-Jan-05			30		Comment: lost to follow-up
34	517	PM	6-Jan-05	26-Jan-05	27-Jan-05	21		Comment: Treatment failure, received rescue
34	519	PM	7-Jan-05			21		Comment: Treatment failure, received rescue
34	521	PM	11-Jan-05	31-Jan-05	31-Jan-05	21		Comment: Treatment failure, received rescue
34	522	Combination	11-Jan-05			17		SUSPECTEDTB PERICARDITIS
34	528	PM	17-Jan-05	6-Feb-05	7-Feb-05	21		Comment: Treatment failure, received rescue
34	531	SSG	21-Jan-05	19-Feb-05	20-Feb-05	30		Comment: Treatment failure, received rescue
34	534	PM	24-Jan-05	14-Feb-05	14-Feb-05	21		THREATENED ANAEMIC HEART FAILURE
34	535	PM	24-Jan-05			21		Comment: Treatment failure, received rescue
34	538	PM	26-Jan-05	15-Feb-05	16-Feb-05	21		Comment: Treatment failure, received rescue
35	669	PM	28-Feb-05	20-Mar-05	21-Mar-05	21	Lost to follow-up	
35	677	SSG	20-Mar-05	26-Mar-05	31-Mar-05	7		ACUTE RENAL FAILURE

16.3.2 Protocol deviations

Appendix 25 Listing of protocol deviations

Centre Number	Patients Number	Treatment	Day of Assessment	Assessment Missing
11	1	PM	Day 7	BUN
11	1	PM	Day 7	Bilirubin
11	2	Combination	Day 7	BUN
11	2	Combination	Day 7	Bilirubin
11	3	Combination	Day 7	BUN
11	3	Combination	Day 7	Bilirubin
11	3	Combination	EOT	Platelet count
11	3	Combination	EOT	Haemaglobin
11	4	PM	Baseline	BUN
11	4	PM	Baseline	Bilirubin
11	5	PM	Baseline	BUN
11	5	PM	Baseline	Bilirubin
11	6	Combination	Baseline	BUN
11	6	Combination	Baseline	Bilirubin
11	7	PM	Baseline	BUN
11	7	PM	Baseline	Bilirubin
11	8	SSG	Baseline	BUN
11	8	SSG	Baseline	Bilirubin
11	9	SSG	Baseline	BUN
11	9	SSG	Baseline	Bilirubin
11	10	SSG	Baseline	BUN
11	10	SSG	Baseline	Bilirubin
11	12	Combination	Baseline	BUN
11	12	Combination	Baseline	Bilirubin
11	15	PM	Day 14	Creatinine
11	15	PM	Day 14	BUN
11	15	PM	Day 14	Bilirubin
11	15	PM	Day 14	Alkaline Phosphatase
11	15	PM	Day 14	Aspartate Aminotransferase
11	15	PM	Day 14	Alanine Aminotransferase
11	15	PM	Day 14	Haemaglobin
11	15	PM	Day 14	White Blood Cell Count
11	15	PM	Day 14	Platelet count
11	15	PM	EOT	Creatinine
11	15	PM	EOT	BUN
11	15	PM	EOT	Bilirubin
11	15	PM	EOT	Alkaline Phosphatase
11	15	PM	EOT	Aspartate Aminotransferase
11	15	PM	EOT	Alanine Aminotransferase
11	15	PM	EOT	Platelet count
11	15	PM	EOT	Haemaglobin
11	15	PM	EOT	White Blood Cell Count
11	18	SSG	Day 7	Platelet count
11	40	PM	EOT	Creatinine
11	40	PM	EOT	BUN
11	40	PM	EOT	Bilirubin
11	40	PM	EOT	Alkaline Phosphatase
11	40	PM	EOT	Aspartate Aminotransferase
11	40	PM	EOT	Alanine Aminotransferase
11	40	PM	EOT	Platelet count

Centre Number	Patients Number	Treatment	Day of Assessment	Assessment Missing
11	40	PM	EOT	Haemaglobin
11	40	PM	EOT	White Blood Cell Count
11	65	PM	6 mon FU	Creatinine
11	65	PM	6 mon FU	BUN
11	65	PM	6 mon FU	Bilirubin
11	65	PM	6 mon FU	Alkaline Phosphatase
11	65	PM	6 mon FU	Aspartate Aminotransferase
11	65	PM	6 mon FU	Alanine Aminotransferase
11	65	PM	6 mon FU	Platelet count
11	65	PM	6 mon FU	Haemaglobin
11	65	PM	6 mon FU	White Blood Cell Count
11	72	SSG	Day 7	BUN
11	72	SSG	EOT	Creatinine
11	72	SSG	EOT	BUN
11	72	SSG	EOT	Bilirubin
11	72	SSG	EOT	Alkaline Phosphatase
11	72	SSG	EOT	Aspartate Aminotransferase
11	72	SSG	EOT	Alanine Aminotransferase
11	72	SSG	EOT	Platelet count
11	72	SSG	EOT	Haemaglobin
11	72	SSG	EOT	White Blood Cell Count
11	85	SSG	6 mon FU	Creatinine
11	85	SSG	6 mon FU	BUN
11	85	SSG	6 mon FU	Bilirubin
11	85	SSG	6 mon FU	Alkaline Phosphatase
11	85	SSG	6 mon FU	Aspartate Aminotransferase
11	85	SSG	6 mon FU	Alanine Aminotransferase
11	85	SSG	6 mon FU	Platelet count
11	85	SSG	6 mon FU	Haemaglobin
11	85	SSG	6 mon FU	White Blood Cell Count
11	90	Combination	6 mon FU	Creatinine
11	90	Combination	6 mon FU	BUN
11	90	Combination	6 mon FU	Bilirubin
11	90	Combination	6 mon FU	Alkaline Phosphatase
11	90	Combination	6 mon FU	Aspartate Aminotransferase
11	90	Combination	6 mon FU	Alanine Aminotransferase
11	90	Combination	6 mon FU	Platelet count
11	90	Combination	6 mon FU	Haemaglobin
11	90	Combination	6 mon FU	White Blood Cell Count
11	96	Combination	6 mon FU	Bilirubin
11	100	PM	6 mon FU	Creatinine
11	100	PM	6 mon FU	BUN
11	100	PM	6 mon FU	Bilirubin
11	100	PM	6 mon FU	Alkaline Phosphatase
11	100	PM	6 mon FU	Aspartate Aminotransferase
11	100	PM	6 mon FU	Alanine Aminotransferase
11	100	PM	6 mon FU	Platelet count
11	100	PM	6 mon FU	Haemaglobin
11	100	PM	6 mon FU	White Blood Cell Count
11	113	SSG	6 mon FU	Creatinine

Centre Number	Patients Number	Treatment	Day of Assessment	Assessment Missing
11	113	SSG	6 mon FU	BUN
11	113	SSG	6 mon FU	Bilirubin
11	113	SSG	6 mon FU	Alkaline Phosphatase
11	113	SSG	6 mon FU	Aspartate Aminotransferase
11	113	SSG	6 mon FU	Alanine Aminotransferase
11	113	SSG	6 mon FU	Platelet count
11	113	SSG	6 mon FU	Haemaglobin
11	113	SSG	6 mon FU	White Blood Cell Count
11	117	PM	6 mon FU	Creatinine
11	117	PM	6 mon FU	BUN
11	117	PM	6 mon FU	Bilirubin
11	117	PM	6 mon FU	Alkaline Phosphatase
11	117	PM	6 mon FU	Aspartate Aminotransferase
11	117	PM	6 mon FU	Alanine Aminotransferase
11	117	PM	6 mon FU	Platelet count
11	117	PM	6 mon FU	Haemaglobin
11	117	PM	6 mon FU	White Blood Cell Count
11	122	SSG	6 mon FU	Creatinine
11	122	SSG	6 mon FU	BUN
11	122	SSG	6 mon FU	Bilirubin
11	122	SSG	6 mon FU	Alkaline Phosphatase
11	122	SSG	6 mon FU	Aspartate Aminotransferase
11	122	SSG	6 mon FU	Alanine Aminotransferase
11	122	SSG	6 mon FU	Platelet count
11	122	SSG	6 mon FU	Haemaglobin
11	122	SSG	6 mon FU	White Blood Cell Count
12	241	Combination	Baseline	Platelet count
12	241	Combination	Baseline	Amylase
12	242	SSG	Baseline	Platelet count
12	242	SSG	Baseline	Amylase
12	243	PM	6 mon FU	Amylase
12	243	PM	Baseline	Platelet count
12	243	PM	Baseline	Amylase
12	243	PM	EOT	Amylase
12	244	SSG	Baseline	Platelet count
12	244	SSG	Baseline	Amylase
12	245	PM	6 mon FU	Creatinine
12	245	PM	6 mon FU	BUN
12	245	PM	6 mon FU	Bilirubin
12	245	PM	6 mon FU	Alkaline Phosphatase
12	245	PM	6 mon FU	Aspartate Aminotransferase
12	245	PM	6 mon FU	Alanine Aminotransferase
12	245	PM	6 mon FU	Platelet count
12	245	PM	6 mon FU	Amylase
12	245	PM	6 mon FU	Total Protein
12	245	PM	6 mon FU	Haemaglobin
12	245	PM	6 mon FU	White Blood Cell Count
12	246	PM	Day 14	Total Protein
12	247	PM	6 mon FU	Amylase
12	247	PM	Day 7	Total Protein

Centre Number	Patients Number	Treatment	Day of Assessment	Assessment Missing
12	248	PM	6 mon FU	Amylase
12	248	PM	Day 14	Total Protein
12	248	PM	Day 7	Total Protein
12	248	PM	EOT	Total Protein
12	250	SSG	6 mon FU	Amylase
12	252	SSG	6 mon FU	Amylase
12	253	SSG	6 mon FU	Amylase
12	256	Combination	6 mon FU	Amylase
12	257	SSG	6 mon FU	Amylase
12	258	SSG	6 mon FU	Amylase
12	259	PM	6 mon FU	Amylase
12	259	PM	EOT	Total Protein
12	260	PM	6 mon FU	Amylase
12	260	PM	Day 14	Amylase
12	261	Combination	6 mon FU	Amylase
12	262	PM	6 mon FU	Amylase
12	262	PM	Baseline	Total Protein
12	263	SSG	6 mon FU	Amylase
12	264	Combination	6 mon FU	Amylase
12	264	Combination	Baseline	Amylase
12	264	Combination	Day 7	Total Protein
12	265	SSG	Day 7	Total Protein
12	265	SSG	EOT	Total Protein
12	266	PM	Day 7	Total Protein
12	267	SSG	6 mon FU	Amylase
12	267	SSG	6 mon FU	Total Protein
12	267	SSG	Day 7	Total Protein
12	270	Combination	Baseline	Total Protein
12	271	SSG	6 mon FU	Amylase
12	271	SSG	6 mon FU	Total Protein
12	271	SSG	Baseline	Amylase
12	271	SSG	Day 14	Amylase
12	271	SSG	Day 7	Total Protein
12	271	SSG	Day 7	Amylase
12	271	SSG	EOT	Amylase
12	272	PM	Baseline	Amylase
12	272	PM	Day 14	Amylase
12	272	PM	Day 7	Amylase
12	272	PM	EOT	Amylase
12	272	PM	EOT	Total Protein
12	273	SSG	Baseline	Amylase
12	273	SSG	Day 14	Amylase
12	273	SSG	Day 7	Amylase
12	274	SSG	Baseline	Amylase
12	274	SSG	Day 14	Amylase
12	274	SSG	Day 7	Amylase
12	275	Combination	6 mon FU	Amylase
12	275	Combination	Baseline	Amylase
12	275	Combination	Day 14	Amylase
12	275	Combination	Day 7	Amylase

Centre Number	Patients Number	Treatment	Day of Assessment	Assessment Missing
12	275	Combination	EOT	Amylase
12	276	PM	6 mon FU	Amylase
12	276	PM	Day 14	Amylase
12	276	PM	Day 7	Amylase
12	276	PM	EOT	Amylase
12	277	Combination	Baseline	Amylase
12	277	Combination	Day 7	Amylase
12	278	SSG	6 mon FU	Creatinine
12	278	SSG	6 mon FU	BUN
12	278	SSG	6 mon FU	Bilirubin
12	278	SSG	6 mon FU	Alkaline Phosphatase
12	278	SSG	6 mon FU	Aspartate Aminotransferase
12	278	SSG	6 mon FU	Alanine Aminotransferase
12	278	SSG	6 mon FU	Platelet count
12	278	SSG	6 mon FU	Amylase
12	278	SSG	6 mon FU	Total Protein
12	278	SSG	6 mon FU	Haemaglobin
12	278	SSG	6 mon FU	White Blood Cell Count
12	278	SSG	Baseline	Amylase
12	278	SSG	Day 7	Amylase
12	279	PM	Baseline	Amylase
12	279	PM	Day 14	Total Protein
12	279	PM	Day 7	Amylase
12	280	SSG	6 mon FU	Creatinine
12	280	SSG	6 mon FU	BUN
12	280	SSG	6 mon FU	Amylase
12	280	SSG	6 mon FU	Total Protein
12	280	SSG	Baseline	Amylase
12	280	SSG	Day 7	Amylase
12	281	Combination	6 mon FU	Creatinine
12	281	Combination	6 mon FU	BUN
12	281	Combination	6 mon FU	Amylase
12	281	Combination	6 mon FU	Total Protein
12	281	Combination	Baseline	Amylase
12	281	Combination	Day 7	Amylase
12	282	PM	6 mon FU	Creatinine
12	282	PM	6 mon FU	BUN
12	282	PM	6 mon FU	Amylase
12	282	PM	6 mon FU	Total Protein
12	282	PM	Baseline	Amylase
12	282	PM	Day 14	Bilirubin
12	283	PM	6 mon FU	Amylase
12	283	PM	6 mon FU	Total Protein
12	283	PM	EOT	Amylase
12	284	Combination	6 mon FU	Creatinine
12	284	Combination	6 mon FU	BUN
12	284	Combination	6 mon FU	Alkaline Phosphatase
12	284	Combination	6 mon FU	Amylase
12	284	Combination	6 mon FU	Total Protein
12	284	Combination	Day 14	Amylase

Centre Number	Patients Number	Treatment	Day of Assessment	Assessment Missing
12	284	Combination	Day 7	Amylase
12	284	Combination	EOT	Amylase
12	285	Combination	6 mon FU	Creatinine
12	285	Combination	6 mon FU	BUN
12	285	Combination	6 mon FU	Amylase
12	285	Combination	6 mon FU	Total Protein
12	285	Combination	Day 14	Amylase
12	285	Combination	EOT	Amylase
12	286	PM	6 mon FU	Amylase
12	286	PM	Baseline	Amylase
12	287	SSG	Baseline	Amylase
12	294	Combination	6 mon FU	Amylase
12	297	SSG	6 mon FU	Creatinine
12	297	SSG	6 mon FU	BUN
12	297	SSG	6 mon FU	Bilirubin
12	297	SSG	6 mon FU	Alkaline Phosphatase
12	297	SSG	6 mon FU	Aspartate Aminotransferase
12	297	SSG	6 mon FU	Alanine Aminotransferase
12	297	SSG	6 mon FU	Platelet count
12	297	SSG	6 mon FU	Amylase
12	297	SSG	6 mon FU	Haemaglobin
12	297	SSG	6 mon FU	White Blood Cell Count
12	297	SSG	EOT	Creatinine
12	297	SSG	EOT	BUN
12	297	SSG	EOT	Bilirubin
12	297	SSG	EOT	Alkaline Phosphatase
12	297	SSG	EOT	Aspartate Aminotransferase
12	297	SSG	EOT	Alanine Aminotransferase
12	297	SSG	EOT	Platelet count
12	297	SSG	EOT	Amylase
12	297	SSG	EOT	Haemaglobin
12	297	SSG	EOT	White Blood Cell Count
12	299	Combination	Day 14	Amylase
12	299	Combination	EOT	Amylase
12	300	PM	Day 14	Amylase
12	300	PM	EOT	Bilirubin
12	301	SSG	Day 14	Amylase
12	302	SSG	Day 14	Amylase
12	303	PM	Day 14	Amylase
12	304	Combination	Day 14	Amylase
12	304	Combination	EOT	Amylase
12	305	PM	Day 14	Amylase
12	306	Combination	Day 14	Amylase
12	306	Combination	EOT	Amylase
12	307	Combination	Baseline	Amylase
12	307	Combination	Day 7	Amylase
12	308	PM	Baseline	Amylase
12	308	PM	Day 7	Amylase
12	309	PM	Baseline	Amylase
12	309	PM	Day 7	Amylase

Centre Number	Patients Number	Treatment	Day of Assessment	Assessment Missing
12	310	SSG	Baseline	Amylase
12	310	SSG	Day 7	Amylase
12	316	SSG	EOT	Creatinine
12	316	SSG	EOT	BUN
12	316	SSG	EOT	Bilirubin
12	316	SSG	EOT	Alkaline Phosphatase
12	316	SSG	EOT	Aspartate Aminotransferase
12	316	SSG	EOT	Alanine Aminotransferase
12	316	SSG	EOT	Platelet count
12	316	SSG	EOT	Haemaglobin
12	316	SSG	EOT	White Blood Cell Count
23	377	Combination	Day 14	Creatinine
23	377	Combination	Day 14	BUN
23	377	Combination	Day 14	Albumin
23	377	Combination	Day 14	Globulin
23	377	Combination	Day 14	Bilirubin
23	377	Combination	Day 14	Alkaline Phosphatase
23	377	Combination	Day 14	Aspartate Aminotransferase
23	377	Combination	Day 14	Alanine Aminotransferase
23	377	Combination	Day 14	Haemaglobin
23	377	Combination	Day 14	White Blood Cell Count
23	377	Combination	Day 14	Platelet count
23	377	Combination	EOT	Creatinine
23	377	Combination	EOT	BUN
23	377	Combination	EOT	Albumin
23	377	Combination	EOT	Globulin
23	377	Combination	EOT	Bilirubin
23	377	Combination	EOT	Alkaline Phosphatase
23	377	Combination	EOT	Aspartate Aminotransferase
23	377	Combination	EOT	Alanine Aminotransferase
23	377	Combination	EOT	Platelet count
23	377	Combination	EOT	Haemaglobin
23	377	Combination	EOT	White Blood Cell Count
23	379	PM	Baseline	Aspartate Aminotransferase
23	392	Combination	EOT	Creatinine
23	392	Combination	EOT	BUN
23	392	Combination	EOT	Albumin
23	392	Combination	EOT	Globulin
23	392	Combination	EOT	Bilirubin
23	392	Combination	EOT	Alkaline Phosphatase
23	392	Combination	EOT	Aspartate Aminotransferase
23	392	Combination	EOT	Alanine Aminotransferase
23	392	Combination	EOT	Platelet count
23	392	Combination	EOT	Haemaglobin
23	392	Combination	EOT	White Blood Cell Count
34	461	SSG	EOT	Creatinine
34	461	SSG	EOT	Bilirubin
34	461	SSG	EOT	Alkaline Phosphatase
34	461	SSG	EOT	Aspartate Aminotransferase
34	461	SSG	EOT	Alanine Aminotransferase

Centre Number	Patients Number	Treatment	Day of Assessment	Assessment Missing
34	461	SSG	EOT	Haemaglobin
34	484	PM	Day 14	Creatinine
34	484	PM	Day 14	Bilirubin
34	484	PM	Day 14	Alkaline Phosphatase
34	484	PM	Day 14	Aspartate Aminotransferase
34	484	PM	Day 14	Alanine Aminotransferase
34	484	PM	Day 14	Haemaglobin
34	484	PM	EOT	Creatinine
34	484	PM	EOT	Bilirubin
34	484	PM	EOT	Alkaline Phosphatase
34	484	PM	EOT	Aspartate Aminotransferase
34	484	PM	EOT	Alanine Aminotransferase
34	484	PM	EOT	Haemaglobin
34	492	PM	EOT	Bilirubin
34	502	Combination	EOT	Bilirubin
34	506	SSG	EOT	Bilirubin
34	512	PM	EOT	Bilirubin
34	525	Combination	EOT	Bilirubin
35	646	PM	6 mon FU	Prothrombin time
35	647	PM	6 mon FU	Prothrombin time
35	648	Combination	Day 14	Creatinine
35	648	Combination	Day 14	BUN
35	648	Combination	Day 14	Albumin
35	648	Combination	Day 14	Bilirubin
35	648	Combination	Day 14	Aspartate Aminotransferase
35	648	Combination	Day 14	Alanine Aminotransferase
35	648	Combination	Day 14	Prothrombin time
35	648	Combination	Day 14	Platelet count
35	648	Combination	Day 14	Total Protein
35	648	Combination	Day 14	Haemaglobin
35	648	Combination	Day 14	White Blood Cell Count
35	649	Combination	Day 14	Creatinine
35	649	Combination	Day 14	BUN
35	649	Combination	Day 14	Albumin
35	649	Combination	Day 14	Bilirubin
35	649	Combination	Day 14	Aspartate Aminotransferase
35	649	Combination	Day 14	Alanine Aminotransferase
35	649	Combination	Day 14	Prothrombin time
35	649	Combination	Day 14	Platelet count
35	649	Combination	Day 14	Total Protein
35	649	Combination	Day 14	Haemaglobin
35	649	Combination	Day 14	White Blood Cell Count
35	652	PM	6 mon FU	Prothrombin time
35	654	SSG	Day 7	Total Protein
35	661	Combination	Day 14	Albumin
35	662	PM	6 mon FU	Prothrombin time
35	662	PM	Day 7	Albumin
35	664	Combination	Day 14	Creatinine
35	664	Combination	6 mon FU	Prothrombin time
35	664	Combination	Day 14	BUN

Centre Number	Patients Number	Treatment	Day of Assessment	Assessment Missing
35	664	Combination	Day 14	Albumin
35	664	Combination	Day 14	Bilirubin
35	664	Combination	Day 14	Aspartate Aminotransferase
35	664	Combination	Day 14	Alanine Aminotransferase
35	664	Combination	Day 14	Prothrombin time
35	664	Combination	Day 14	Platelet count
35	664	Combination	Day 14	Total Protein
35	664	Combination	Day 14	Haemaglobin
35	664	Combination	Day 14	White Blood Cell Count
35	664	Combination	EOT	Creatinine
35	664	Combination	EOT	BUN
35	664	Combination	EOT	Albumin
35	664	Combination	EOT	Bilirubin
35	664	Combination	EOT	Aspartate Aminotransferase
35	664	Combination	EOT	Alanine Aminotransferase
35	664	Combination	EOT	Prothrombin time
35	664	Combination	EOT	Platelet count
35	664	Combination	EOT	Total Protein
35	664	Combination	EOT	Haemaglobin
35	664	Combination	EOT	White Blood Cell Count
35	665	SSG	Baseline	Prothrombin time
35	669	PM	6 mon FU	Creatinine
35	669	PM	6 mon FU	BUN
35	669	PM	6 mon FU	Albumin
35	669	PM	6 mon FU	Bilirubin
35	669	PM	6 mon FU	Aspartate Aminotransferase
35	669	PM	6 mon FU	Alanine Aminotransferase
35	669	PM	6 mon FU	Prothrombin time
35	669	PM	6 mon FU	Platelet count
35	669	PM	6 mon FU	Total Protein
35	669	PM	6 mon FU	Haemaglobin
35	669	PM	6 mon FU	White Blood Cell Count
35	671	PM	6 mon FU	Prothrombin time
35	675	PM	6 mon FU	Prothrombin time
35	679	SSG	EOT	Albumin
35	682	PM	6 mon FU	Prothrombin time
35	684	SSG	Day 7	Total Protein
35	686	PM	6 mon FU	Prothrombin time
35	689	Combination	6 mon FU	Prothrombin time

16.3.3 Patients excluded from the efficacy analysis

Not applicable.

16.3.4 Demographic data

Appendix 26 Individual patient demographic data

Appendix 27 Individual Baseline VL symptoms

Appendix 28 Individual Height and Weight

Appendix 29 Individual Heart rate and Axiliary temperature

Appendix 30 Individual Systolic and Diastolic Blood pressure

Centre Number	Patient Number	Date of Bith	Age	Sex	HIV status	Date of Admission	Treatment
11	1		30	Male	negative	17-Jun-05	PM
11	2		19	Male	negative	17-Jun-05	Combination
11	3		20	Male	negative	18-Jun-05	Combination
11	4		19	Male	negative	27-Jun-05	PM
11	5		10	Female	negative	27-Jun-05	PM
11	6		10	Female	negative	27-Jun-05	Combination
11	7		45	Female	negative	28-Jun-05	PM
11	8		28	Male	negative	28-Jun-05	SSG
11	9		20	Male	negative	2-Jul-05	SSG
11	10		17	Male	negative	2-Jul-05	SSG
11	11		12	Male	not tested	30-Jun-05	Combination
11	12		18	Male	negative	29-Jun-05	Combination
11	13		40	Female	negative	5-Jul-05	SSG
11	14		7	Male	negative	6-Jul-05	SSG
11	15		20	Male	negative	8-Jul-05	PM
11	16		35	Female	negative	6-Jul-05	Combination
11	17		40	Male	negative	6-Jul-05	Combination
11	18		34	Male	negative	12-Aug-05	SSG
11	19		33	Male	negative	13-Aug-05	Combination
11	20		7	Male	negative	13-Aug-05	SSG
11	21		28	Male	negative	13-Aug-05	SSG
11	22		11	Male	negative	14-Aug-05	SSG
11	23		10	Male	negative	17-Aug-05	PM
11	24		7	Female	negative	17-Aug-05	PM
11	25		10	Female	negative	17-Aug-05	SSG
11	26		8	Female	negative	14-Aug-05	Combination
11	27		31	Male	positive	16-Sep-05	PM
11	28		13	Male	positive	16-Sep-05	Combination
11	29		25	Male	not tested	17-Sep-05	PM
11	30		6	Female	negative	19-Sep-05	PM
11	31		40	Female	negative	19-Sep-05	SSG
11	32		22	Female	negative	19-Sep-05	Combination
11	33		25	Male	negative	22-Sep-05	SSG
11	34		55	Male	negative	23-Sep-05	PM
11	35		18	Male	negative	23-Sep-05	SSG
11	36		20	Male	negative	19-Sep-05	SSG
11	37		40	Male	not tested	24-Sep-05	Combination
11	38		47	Male	negative	12-Oct-05	PM
11	39		6	Male	negative	12-Oct-05	PM
11	40		8	Female	negative	12-Oct-05	PM
11	41		5	Male	negative	12-Oct-05	Combination
11	42		30	Male	negative	12-Oct-05	Combination
11	43		15	Male	negative	23-Oct-05	SSG
11	44		16	Male	negative	23-Oct-05	PM
11	45		16	Male	negative	23-Oct-05	Combination
11	46		6	Female	negative	2-May-06	SSG
11	47		20	Male	negative	2-May-06	SSG
11	48		15	Male	negative	3-May-06	Combination
11	49		7	Male	negative	6-May-06	SSG
11	50		20	Male	negative	16-May-06	PM
11	51		15	Male	negative	17-May-06	Combination
11	52		11	Male	negative	17-May-06	PM
11	53		26	Male	negative	24-May-06	SSG
11	54		24	Male	positive	25-May-06	PM

Centre Number	Patient Number	Date of Bith	Age	Sex	HIV status	Date of Admission	Treatment
11	55		27	Male	negative	26-May-06	PM
11	56		13	Female	negative	26-May-06	SSG
11	57		27	Male	negative	2-Jun-06	Combination
11	58		12	Male	negative	3-Jun-06	Combination
11	59		12	Male	negative	9-Jun-06	PM
11	60		32	Male	negative	9-Jun-06	Combination
11	61		27	Male	negative	9-Jun-06	PM
11	62		6	Male	negative	12-Jun-06	SSG
11	63		32	Male	negative	16-Jun-06	SSG
11	64		26	Male	negative	17-Jun-06	SSG
11	65		5	Male	negative	19-Jun-06	PM
11	66		17	Female	negative	16-Jun-06	Combination
11	67		11	Male	negative	28-Jun-06	PM
11	68		9	Male	negative	5-Jul-06	SSG
11	69		18	Male	negative	12-Jul-06	Combination
11	70		20	Male	negative	12-Jul-06	Combination
11	71		33	Male	positive	19-Jul-06	Combination
11	72		23	Male	positive	20-Jul-06	SSG
11	73		23	Male	positive	21-Jul-06	PM
11	74		34	Male	negative	26-Jul-06	PM
11	75		25	Male	negative	26-Jul-06	Combination
11	76		23	Male	negative	26-Jul-06	PM
11	77		10	Male	negative	28-Jul-06	PM
11	78		30	Male	positive	28-Jul-06	SSG
11	79		5	Male	negative	28-Jul-06	PM
11	80		9	Female	negative	31-Jul-06	PM
11	81		25	Male	negative	3-Aug-06	Combination
11	82		15	Male	negative	11-Aug-06	SSG
11	83		22	Male	negative	14-Aug-06	SSG
11	84		15	Male	negative	11-Aug-06	SSG
11	85		20	Male	negative	23-Aug-06	SSG
11	86		5	Male	negative	24-Aug-06	Combination
11	87		18	Male	positive	24-Aug-06	Combination
11	88		25	Male	negative	24-Aug-06	PM
11	89		24	Male	negative	23-Aug-06	Combination
11	90		10	Male	negative	29-Aug-06	Combination
11	91		35	Male	negative	14-Dec-06	PM
11	92		7	Male	negative	21-Dec-06	Combination
11	93		26	Male	negative	29-Dec-06	SSG
11	94		5	Female	negative	1-Jan-07	SSG
11	95		6	Male	negative	1-Jan-07	SSG
11	96		12	Male	positive	17-Jan-07	Combination
11	97		19	Male	negative	18-Jan-07	Combination
11	98		20	Male	negative	22-Jan-07	PM
11	99		19	Male	negative	24-Jan-07	SSG
11	100		36	Male	positive	25-Jan-07	PM
11	101		22	Male	negative	1-Feb-07	PM
11	102		22	Male	negative	1-Feb-07	Combination
11	103		28	Male	negative	31-Jan-07	Combination
11	104		13	Female	negative	6-Feb-07	PM
11	105		18	Male	positive	19-Feb-07	SSG
11	106		18	Male	negative	23-Feb-07	Combination
11	107		21	Male	negative	23-Feb-07	PM
11	108		25	Male	negative	28-Feb-07	PM

Centre Number	Patient Number	Date of Bith	Age	Sex	HIV status	Date of Admission	Treatment
11	109		19	Male	negative	26-Feb-07	SSG
11	110		6	Female	negative	1-Mar-07	PM
11	111		19	Male	negative	15-Mar-07	Combination
11	112		28	Male	negative	22-Mar-07	Combination
11	113		18	Male	negative	22-Mar-07	SSG
11	114		25	Male	negative	20-Mar-07	SSG
11	115		12	Male	negative	28-Mar-07	Combination
11	116		23	Male	negative	28-Mar-07	SSG
11	117		22	Male	negative	28-Mar-07	PM
11	118		25	Male	negative	11-Apr-07	PM
11	119		26	Male	negative	17-Apr-07	SSG
11	120		17	Male	negative	18-Apr-07	Combination
11	121		6	Male	negative	18-Apr-07	Combination
11	122		20	Male	negative	18-Apr-07	SSG
11	123		14	Male	not tested	25-Apr-07	SSG
11	124		30	Male	not tested	18-May-07	SSG
11	125		22	Male	negative	22-May-07	SSG
11	126		23	Male	positive	23-May-07	Combination
11	127		14	Female	negative	25-May-07	PM
11	128		30	Male	positive	30-May-07	Combination
11	129		31	Male	negative	4-Jun-07	PM
11	130		25	Male	negative	6-Jun-07	Combination
11	131		6	Male	negative	8-Jun-07	PM
11	132		20	Male	negative	11-Jun-07	SSG
11	133		20	Male	negative	29-Jun-07	Combination
11	134		23	Male	negative	9-Jul-07	PM
11	135		17	Male	negative	11-Jul-07	PM
12	241		20	Male	negative	7-Jun-05	Combination
12	242		7	Male	negative	7-Jun-05	SSG
12	243		10	Male	negative	7-Jun-05	PM
12	244		20	Male	negative	7-Jun-05	SSG
12	245		36	Male	negative	15-Jul-05	PM
12	246		40	Male	positive	3-Aug-05	PM
12	247		11	Male	negative	9-Sep-05	PM
12	248		28	Male	negative	9-Sep-05	PM
12	249		16	Male	negative	22-Oct-05	Combination
12	250		15	Male	negative	22-Oct-05	SSG
12	251		18	Male	negative	26-Oct-05	Combination
12	252		14	Male	positive	26-Oct-05	SSG
12	253		9	Male	negative	26-Oct-05	SSG
12	254		8	Male	negative	27-Oct-05	Combination
12	255		14	Male	negative	29-Oct-05	Combination
12	256		35	Female	negative	14-Nov-05	Combination
12	257		15	Male	negative	14-Nov-05	SSG
12	258		10	Male	negative	29-Nov-05	SSG
12	259		20	Male	negative	23-Dec-05	PM
12	260		16	Male	negative	23-Dec-05	PM
12	261		13	Male	negative	24-Dec-05	Combination
12	262		10	Male	negative	23-Dec-05	PM
12	263		17	Female	negative	22-Jan-06	SSG
12	264		9	Male	negative	8-Feb-06	Combination
12	265		5	Female	negative	8-Feb-06	SSG
12	266		32	Male	negative	8-Feb-06	PM
12	267		30	Female	negative	8-Feb-06	SSG

Centre Number	Patient Number	Date of Bith	Age	Sex	HIV status	Date of Admission	Treatment
12	268		28	Male	negative	4-Mar-06	PM
12	269		18	Male	negative	13-Mar-06	Combination
12	270		10	Male	negative	13-Mar-06	Combination
12	271		28	Male	negative	27-Mar-06	SSG
12	272		44	Male	negative	24-Mar-06	PM
12	273		13	Female	negative	27-Mar-06	SSG
12	274		10	Female	negative	4-Apr-06	SSG
12	275		16	Female	negative	4-Apr-06	Combination
12	276		10	Male	negative	4-Apr-06	PM
12	277		18	Male	negative	17-Apr-06	Combination
12	278		25	Male	negative	17-Apr-06	SSG
12	279		41	Male	negative	17-Apr-06	PM
12	280		5	Male	negative	17-Apr-06	SSG
12	281		16	Male	negative	17-Apr-06	Combination
12	282		8	Male	negative	26-Apr-06	PM
12	283		25	Male	negative	10-May-06	PM
12	284		9	Female	negative	17-May-06	Combination
12	285		17	Male	negative	17-May-06	Combination
12	286		12	Male	negative	21-Dec-06	PM
12	287		10	Male	negative	21-Dec-06	SSG
12	288		6	Male	negative	26-Dec-06	Combination
12	289		40	Male	negative	26-Dec-06	Combination
12	290		10	Female	negative	4-Jan-07	SSG
12	291		45	Male	negative	8-Jan-07	SSG
12	292		30	Male	negative	14-Jan-07	PM
12	293		13	Male	negative	21-Jan-07	SSG
12	294		5	Female	negative	13-Feb-07	Combination
12	295		10	Male	negative	9-Mar-07	Combination
12	296		12	Male	negative	3-Apr-07	PM
12	297		29	Male	negative	3-Apr-07	SSG
12	298		12	Male	negative	16-Apr-07	PM
12	299		30	Male	negative	1-Jun-07	Combination
12	300		20	Female	negative	1-Jun-07	PM
12	301		25	Male	negative	2-Jun-07	SSG
12	302		4	Male	negative	2-Jun-07	SSG
12	303		25	Female	negative	2-Jun-07	PM
12	304		17	Male	negative	2-Jun-07	Combination
12	305		6	Male	negative	2-Jun-07	PM
12	306		5	Male	negative	2-Jun-07	Combination
12	307		39	Male	negative	12-Jun-07	Combination
12	308		31	Male	negative	12-Jun-07	PM
12	309		18	Male	negative	15-Jun-07	PM
12	310		13	Female	negative	15-Jun-07	SSG
12	311		12	Male	negative	3-Jul-07	SSG
12	312		20	Female	negative	9-Jul-07	Combination
12	313		25	Male	negative	31-Jul-07	PM
12	314		18	Male	negative	31-Jul-07	SSG
12	315		10	Male	negative	19-Aug-07	Combination
12	316		36	Male	negative	19-Aug-07	SSG
12	317		4	Male	negative	21-Aug-07	SSG
12	318		8	Male	negative	26-Aug-07	Combination
12	319		8	Male	negative	26-Aug-07	Combination
12	320		8	Male	negative	3-Sep-07	Combination
12	321		8	Male	negative	3-Sep-07	Combination

Centre Number	Patient Number	Date of Bith	Age	Sex	HIV status	Date of Admission	Treatment
12	322		16	Female	negative	10-Sep-07	PM
12	323		32	Male	negative	18-Sep-07	SSG
12	324		30	Male	negative	18-Sep-07	SSG
12	325		22	Male	negative	18-Sep-07	PM
12	326		25	Male	negative	2-Oct-07	PM
12	327		14	Male	negative	2-Oct-07	Combination
12	328		14	Male	negative	3-Oct-07	PM
12	329		6	Male	negative	8-Oct-07	SSG
12	330		11	Male	negative	8-Oct-07	PM
23	361	15-Jun-97	8	Male	not tested	17-Jan-05	Combination
23	362	15-Jun-91	14	Male	not tested	17-Jan-05	PM
23	363	15-Jun-00	5	Female	not tested	16-Jan-05	PM
23	364	15-Jun-93	12	Female	not tested	16-Jan-05	Combination
23	365	15-Jun-90	15	Male	not tested	16-Jan-05	SSG
23	366	15-Jun-93	12	Male	not tested	26-Jan-05	Combination
23	367	15-Jun-85	20	Male	not tested	26-Jan-05	SSG
23	368	15-Jun-89	16	Female	not tested	26-Jan-05	PM
23	369	15-Jun-93	12	Male	not tested	26-Jan-05	Combination
23	370	15-Jun-97	8	Male	not tested	26-Jan-05	SSG
23	371	15-Jun-91	14	Female	not tested	1-Feb-05	PM
23	372	15-Jun-80	25	Male	not tested	9-Feb-05	PM
23	373		21	Male	not tested	16-Feb-05	SSG
23	374	15-Jun-81	24	Female	not tested	16-Feb-05	SSG
23	375	15-Jun-93	12	Male	not tested	23-Feb-05	Combination
23	376	15-Jun-89	16	Female	not tested	22-Feb-05	SSG
23	377	15-Jun-65	40	Female	not tested	6-Mar-05	Combination
23	378	15-Jun-98	7	Male	not tested	6-Mar-05	Combination
23	379	15-Jun-00	5	Male	not tested	23-Feb-05	PM
23	380	15-Jun-98	7	Female	not tested	6-Mar-05	SSG
23	381	15-Jun-92	13	Male	not tested	7-Mar-05	PM
23	382	15-Jun-90	15	Male	not tested	15-Mar-05	Combination
23	383	15-Jun-75	30	Male	not tested	22-Mar-05	PM
23	384	15-Jun-92	13	Male	not tested	22-Mar-05	SSG
23	385	15-Jun-91	14	Male	not tested	23-Mar-05	SSG
23	386	15-Jun-92	13	Female	not tested	13-Apr-05	Combination
23	387	15-Jun-90	15	Female	not tested	13-Apr-05	Combination
23	388	15-Jun-90	15	Male	not tested	13-Apr-05	PM
23	389	15-Jun-93	12	Male	not tested	13-Apr-05	SSG
23	390	15-Jun-85	20	Male	not tested	13-Apr-05	PM
23	391	15-Jun-93	12	Female	not tested	19-Apr-05	Combination
23	392	15-Jun-98	7	Male	not tested	19-Apr-05	Combination
23	393	15-Jun-93	12	Male	not tested	19-Apr-05	SSG
23	394	15-Jun-96	9	Male	not tested	13-Apr-05	PM
23	395	15-Jun-63	42	Male	not tested	27-Apr-05	PM
23	396	15-Jun-89	16	Male	not tested	9-May-05	SSG
23	397	15-Jun-91	14	Female	not tested	9-May-05	SSG
23	398	15-Jun-97	8	Male	not tested	12-May-05	Combination
23	399	15-Jun-99	6	Male	not tested	12-May-05	PM
23	400	15-Jun-99	6	Female	not tested	12-May-05	PM
23	401	15-Jun-65	40	Male	not tested	29-May-05	Combination
23	402	15-Jun-90	15	Male	not tested	29-May-05	Combination
23	403	15-Jun-83	22	Male	not tested	29-May-05	SSG
23	404	15-Jun-89	16	Male	not tested	29-May-05	SSG
23	405	15-Jun-89	16	Male	not tested	29-May-05	PM

Centre Number	Patient Number	Date of Bith	Age	Sex	HIV status	Date of Admission	Treatment
34	451		6	Female	not tested	16-Nov-04	Combination
34	452		15	Male	not tested	16-Nov-04	PM
34	453		4	Male	not tested	17-Nov-04	PM
34	454		5	Female	not tested	18-Nov-04	Combination
34	455		11	Male	not tested	18-Nov-04	PM
34	456		8	Female	not tested	18-Nov-04	SSG
34	457		25	Male	negative	18-Nov-04	Combination
34	458		12	Female	not tested	19-Nov-04	PM
34	459		9	Female	not tested	20-Nov-04	Combination
34	460		4	Male	not tested	22-Nov-04	PM
34	461		50	Male	not tested	24-Nov-04	SSG
34	462		4	Female	not tested	24-Nov-04	SSG
34	463		4	Male	not tested	25-Nov-04	Combination
34	464		22	Male	not tested	26-Nov-04	SSG
34	465		25	Female	negative	27-Nov-04	SSG
34	466		10	Female	not tested	27-Nov-04	Combination
34	467		14	Male	not tested	28-Nov-04	PM
34	468		25	Female	negative	28-Nov-04	Combination
34	469		11	Female	not tested	28-Nov-04	SSG
34	470		4	Female	not tested	29-Nov-04	Combination
34	471		13	Male	not tested	29-Nov-04	PM
34	472		4	Male	not tested	1-Dec-04	SSG
34	473		5	Male	not tested	1-Dec-04	PM
34	474		10	Male	not tested	3-Dec-04	Combination
34	475		9	Male	not tested	4-Dec-04	PM
34	476		10	Female	not tested	4-Dec-04	PM
34	477		8	Female	not tested	6-Dec-04	SSG
34	478		5	Female	not tested	6-Dec-04	Combination
34	479		4	Male	not tested	8-Dec-04	SSG
34	480		12	Male	not tested	8-Dec-04	SSG
34	481		15	Male	not tested	9-Dec-04	Combination
34	482		18	Male	negative	9-Dec-04	Combination
34	483		24	Male	not tested	9-Dec-04	PM
34	484		5	Male	not tested	10-Dec-04	PM
34	485		5	Female	not tested	10-Dec-04	Combination
34	486		12	Male	not tested	11-Dec-04	Combination
34	487		12	Male	not tested	12-Dec-04	SSG
34	488		7	Male	not tested	13-Dec-04	SSG
34	489		11	Male	not tested	15-Dec-04	SSG
34	490		9	Female	not tested	16-Dec-04	PM
34	491		50	Male	positive	16-Dec-04	PM
34	492		39	Male	negative	16-Dec-04	PM
34	493		12	Female	not tested	17-Dec-04	SSG
34	494		30	Male	negative	17-Dec-04	Combination
34	495		6	Female	not tested	17-Dec-04	SSG
34	496		35	Male	not tested	18-Dec-04	SSG
34	497		22	Male	not tested	20-Dec-04	PM
34	498		10	Female	not tested	20-Dec-04	SSG
34	499		12	Male	not tested	20-Dec-04	Combination
34	500		14	Male	not tested	20-Dec-04	PM
34	501		9	Male	not tested	21-Dec-04	Combination
34	502		12	Female	not tested	22-Dec-04	Combination
34	503		11	Male	not tested	24-Dec-04	PM
34	504		8	Female	not tested	26-Dec-04	Combination

Centre Number	Patient Number	Date of Bith	Age	Sex	HIV status	Date of Admission	Treatment
34	505		40	Male	negative	27-Dec-04	SSG
34	506		8	Male	not tested	30-Dec-04	SSG
34	507		6	Male	not tested	30-Dec-04	SSG
34	508		4	Male	not tested	31-Dec-04	PM
34	509		20	Female	negative	31-Dec-04	PM
34	510		8	Male	not tested	31-Dec-04	Combination
34	511		6	Male	not tested	1-Jan-05	Combination
34	512		6	Female	not tested	1-Jan-05	PM
34	513		10	Male	not tested	2-Jan-05	Combination
34	514		7	Male	not tested	3-Jan-05	SSG
34	515		10	Male	not tested	5-Jan-05	SSG
34	516		15	Male	not tested	6-Jan-05	PM
34	517		12	Male	not tested	6-Jan-05	PM
34	518		4	Male	not tested	6-Jan-05	SSG
34	519		8	Female	not tested	6-Jan-05	PM
34	520		19	Male	negative	10-Jan-05	SSG
34	521		5	Male	not tested	10-Jan-05	PM
34	522		36	Male	negative	11-Jan-05	Combination
34	523		11	Male	not tested	12-Jan-05	SSG
34	524		15	Male	not tested	12-Jan-05	Combination
34	525		8	Male	not tested	12-Jan-05	Combination
34	526		5	Male	not tested	13-Jan-05	SSG
34	527		19	Female	not tested	14-Jan-05	Combination
34	528		6	Male	not tested	16-Jan-05	PM
34	529		5	Female	not tested	18-Jan-05	Combination
34	530		22	Male	negative	19-Jan-05	Combination
34	531		5	Male	not tested	20-Jan-05	SSG
34	532		4	Male	not tested	20-Jan-05	Combination
34	533		10	Male	not tested	22-Jan-05	SSG
34	534		7	Male	not tested	23-Jan-05	PM
34	535		11	Female	not tested	23-Jan-05	PM
34	536		12	Male	not tested	25-Jan-05	PM
34	537		4	Female	not tested	25-Jan-05	SSG
34	538		6	Male	not tested	25-Jan-05	PM
34	539		6	Male	not tested	25-Jan-05	Combination
34	540		11	Female	not tested	26-Jan-05	SSG
35	646		16	Male	negative	26-Jan-05	PM
35	647		23	Male	negative	29-Jan-05	PM
35	648		5	Male	negative	29-Jan-05	Combination
35	649		13	Female	negative	29-Jan-05	Combination
35	650		6	Male	negative	29-Jan-05	SSG
35	651		5	Male	negative	29-Jan-05	SSG
35	652		8	Female	negative	31-Jan-05	PM
35	653		6	Female	negative	1-Feb-05	Combination
35	654		5	Female	negative	31-Jan-05	SSG
35	655		11	Male	negative	1-Feb-05	Combination
35	656		8	Female	negative	3-Feb-05	SSG
35	657		10	Female	negative	7-Feb-05	PM
35	658		15	Male	negative	6-Feb-05	Combination
35	659		6	Male	negative	7-Feb-05	SSG
35	660		32	Male	negative	6-Feb-05	PM
35	661		27	Male	negative	14-Feb-05	Combination
35	662		25	Male	negative	19-Feb-05	PM
35	663		48	Male	negative	19-Feb-05	SSG

Centre Number	Patient Number	Date of Bith	Age	Sex	HIV status	Date of Admission	Treatment
35	664		37	Female	negative	19-Feb-05	Combination
35	665		13	Male	negative	22-Feb-05	SSG
35	666		12	Female	negative	25-Feb-05	SSG
35	667		13	Female	negative	26-Feb-05	Combination
35	668		17	Female	negative	26-Feb-05	PM
35	669		9	Female	negative	27-Feb-05	PM
35	670		10	Male	negative	1-Mar-05	SSG
35	671		9	Female	negative	10-Mar-05	PM
35	672		8	Male	negative	10-Mar-05	Combination
35	673		11	Female	negative	10-Mar-05	SSG
35	674		9	Female	negative	11-Mar-05	Combination
35	675		8	Female	negative	13-Mar-05	PM
35	676		8	Female	negative	19-Mar-05	PM
35	677		37	Male	negative	19-Mar-05	SSG
35	678		15	Male	negative	20-Mar-05	Combination
35	679		45	Female	negative	20-Mar-05	SSG
35	680		11	Male	negative	21-Mar-05	Combination
35	681		16	Male	negative	21-Mar-05	Combination
35	682		8	Female	negative	21-Mar-05	PM
35	683		12	Female	negative	21-Mar-05	Combination
35	684		15	Female	negative	8-Apr-05	SSG
35	685		14	Male	negative	8-Apr-05	PM
35	686		28	Male	negative	8-Apr-05	PM
35	687		21	Female	negative	13-Apr-05	SSG
35	688		17	Male	negative	14-Apr-05	PM
35	689		18	Female	negative	14-Apr-05	Combination
35	690		10	Male	negative	14-Mar-05	SSG

Centre Number	Patient Number	VL Symptom Fever	Headache	Fatigue	Epistaxis	Abdiminal pain	Abdominal swelling	Swollen Legs	Cough
11	1	Yes	No	Yes	Yes	No	No	No	Yes
11	2	Yes	Yes	Yes	No	No	No	No	No
11	3	Yes	Yes	Yes	No	No	No	No	Yes
11	4	Yes	Yes	Yes	No	No	Yes	No	Yes
11	5	Yes	Yes	Yes	No	No	Yes	No	No
11	6	Yes	Yes	No	Yes	Yes	Yes	No	Yes
11	7	Yes	No	Yes	No	Yes	Yes	No	Yes
11	8	Yes	No	Yes	Yes	Yes	Yes	No	Yes
11	9	Yes	Yes	Yes	No	Yes	No	No	No
11	10	Yes	Yes	No	No	Yes	No	No	Yes
11	11	Yes	No	Yes	No	Yes	No	No	No
11	12	Yes	Yes	Yes	No	No	Yes	No	Yes
11	13	Yes	Yes	Yes	No	Yes	No	No	Yes
11	14	Yes	Yes	Yes	No	No	Yes	No	Yes
11	15	Yes		Yes	No	No	Yes	No	No
11	16	Yes	No	Yes	Yes	No	No	No	Yes
11	17	Yes	No	No	Yes	No	No	No	Yes
11	18	Yes	No	Yes	Yes	Yes	Yes	No	No
11	19	Yes	Yes	Yes	Yes	No	Yes	No	No
11	20	Yes	Yes	Yes	No	Yes	No	No	No
11	21	Yes	Yes	Yes	No	No	Yes	No	No
11	22	Yes	Yes	Yes	No	No	Yes	No	Yes
11	23	Yes	Yes	Yes	No	Yes	Yes	No	No
11	24	Yes	No	Yes	No	Yes	Yes	No	No
11	25	Yes	Yes	Yes	No	Yes	Yes	No	No
11	26	Yes	No	Yes	No	Yes	Yes	No	No
11	27	Yes	No	Yes	Yes	No	No	Yes	No
11	28	Yes	Yes	Yes	No	Yes	Yes	No	No
11	29	Yes	Yes	Yes	No	Yes	Yes	No	No
11	30	Yes	Yes	Yes	No	Yes	Yes	No	No
11	31	Yes	Yes	Yes	No	Yes	Yes	Yes	Yes
11	32	Yes	Yes	Yes	No	Yes	No	No	No
11	33	Yes	Yes	Yes	No	No	Yes	No	No
11	34	Yes	No	Yes	No	Yes	Yes	No	No
11	35	No	No	Yes	No	Yes	Yes	No	No
11	36	Yes	Yes	Yes	No	No	No	Yes	No
11	37	Yes	No	Yes	Yes	No	No	No	No
11	38	Yes	Yes	Yes	No	No	Yes	No	No
11	39	Yes	No	No	No	Yes	Yes	No	No
11	40	Yes	Yes	Yes	No	Yes	Yes	No	No
11	41	Yes	Yes	Yes	No	Yes	Yes	No	Yes
11	42	Yes	Yes	Yes	No	Yes	Yes	No	Yes
11	43	Yes	Yes	Yes	Yes	Yes	No	No	No
11	44	Yes	Yes	Yes	Yes	Yes	Yes	No	No
11	45	Yes	Yes	Yes	No	Yes	Yes	No	No
11	46	Yes	Yes	No	Yes	Yes	No	No	Yes
11	47	Yes	Yes	Yes	Yes	No	No	No	Yes
11	48	Yes	Yes	Yes	No	Yes	Yes	No	Yes
11	49	Yes	Yes	Yes	No	Yes	Yes	Yes	Yes
11	50	Yes	No	Yes	Yes	No	Yes	Yes	Yes
11	51	Yes	Yes	Yes	Yes	No	No	Yes	No
11	52	Yes	Yes	Yes	Yes	Yes	Yes	No	Yes

Centre Number	Patient Number	VL Symptom Fever	Headache	Fatigue	Epistaxis	Abdiminal pain	Abdominal swelling	Swollen Legs	Cough
11	53	Yes	No	Yes	No	Yes	Yes	No	Yes
11	54	Yes	Yes	Yes	Yes	Yes	Yes	No	No
11	55	Yes	Yes	Yes	Yes	Yes	Yes	Yes	No
11	56	Yes	Yes	Yes	Yes	Yes	Yes	Yes	Yes
11	57	Yes	No	Yes	No	No	Yes	No	Yes
11	58	Yes	Yes	Yes	No	Yes	Yes	No	No
11	59	Yes	Yes	Yes	No	No	Yes	Yes	Yes
11	60	Yes	Yes	Yes	Yes	Yes	Yes	Yes	Yes
11	61	Yes	Yes	Yes	Yes	Yes	Yes	Yes	Yes
11	62	Yes	No	Yes	No	No	Yes	No	Yes
11	63	Yes	No	Yes	No	Yes	Yes	No	No
11	64	Yes	Yes	Yes	No	Yes	Yes	No	Yes
11	65	Yes	Yes	Yes	No	Yes	Yes	No	Yes
11	66	Yes	Yes	Yes	No	No	Yes	Yes	Yes
11	67	Yes	Yes	Yes	No	Yes	Yes	No	Yes
11	68	Yes	Yes	Yes	Yes	No	Yes	Yes	Yes
11	69	Yes	Yes	Yes	Yes	Yes	Yes	Yes	Yes
11	70	Yes	No	Yes	Yes	Yes	Yes	Yes	Yes
11	71	Yes	Yes	Yes	Yes	Yes	Yes	No	No
11	72	Yes	Yes	Yes	Yes	No	No	No	Yes
11	73	Yes	Yes	Yes	Yes	No	Yes	No	No
11	74	Yes	Yes	Yes	No	Yes	Yes	No	Yes
11	75	Yes	No	Yes	No	Yes	No	No	Yes
11	76	Yes	Yes	Yes	No	Yes	Yes	No	Yes
11	77	Yes	Yes	Yes	Yes	Yes	Yes	No	Yes
11	78	Yes	No	Yes	Yes	No	Yes	No	Yes
11	79	Yes	Yes	Yes	No	Yes	Yes	No	Yes
11	80	Yes	Yes	Yes	No	Yes	Yes	No	Yes
11	81	Yes	No	Yes	Yes	Yes	Yes	Yes	Yes
11	82	Yes	No	Yes	No	Yes	Yes	Yes	Yes
11	83	Yes	No	Yes	No	Yes	Yes	Yes	Yes
11	84	Yes	Yes	Yes	Yes	Yes	Yes	Yes	Yes
11	85	Yes	Yes	Yes	Yes	Yes	Yes	No	Yes
11	86	Yes	Yes	Yes	Yes	Yes	Yes	Yes	Yes
11	87	Yes	No	Yes	No	Yes	Yes	No	Yes
11	88	Yes	No	Yes	Yes	No	No	No	Yes
11	89	Yes	Yes	Yes	Yes	Yes	Yes	Yes	Yes
11	90	Yes	No	Yes	No	No	No	No	Yes
11	91	Yes	Yes	Yes	Yes	No	Yes	Yes	Yes
11	92	Yes	No	Yes	No	Yes	No	Yes	Yes
11	93	Yes	Yes	Yes	No	Yes	Yes	No	No
11	94	Yes	Yes	Yes	No	Yes	Yes	No	Yes
11	95	Yes	Yes	Yes	No	Yes	No	No	Yes
11	96	Yes	No	Yes	No	Yes	Yes	Yes	Yes
11	97	Yes	Yes	Yes	No	Yes	Yes	Yes	Yes
11	98	Yes	Yes	Yes	Yes	Yes	Yes	Yes	Yes
11	99	Yes	Yes	Yes	No	No	Yes	No	No
11	100	Yes	No	Yes	No	No	No	No	No
11	101	Yes	Yes	Yes	Yes	Yes	Yes	No	Yes
11	102	Yes	Yes	Yes	Yes	No	Yes	Yes	Yes
11	103	Yes	No	Yes	No	Yes	Yes	No	Yes
11	104	Yes	Yes	Yes	Yes	Yes	Yes	Yes	Yes

Centre Number	Patient Number	VL Symptom							
		Fever	Headache	Fatigue	Epistaxis	Abdiminal pain	Abdominal swelling	Swollen Legs	Cough
11	105	Yes	No	Yes	No	No	Yes	Yes	Yes
11	106	Yes	Yes	Yes	No	No	Yes	No	No
11	107	Yes	Yes	No	Yes	No	Yes	No	Yes
11	108	Yes	No	Yes	Yes	Yes	Yes	Yes	Yes
11	109	Yes	No	Yes	No	No	Yes	Yes	Yes
11	110	Yes	Yes	Yes	No	Yes	Yes	No	No
11	111	Yes	No	Yes	Yes	Yes	No	Yes	Yes
11	112	Yes	No	Yes	No	No	Yes	No	Yes
11	113	Yes	Yes	Yes	Yes	Yes	Yes	Yes	Yes
11	114	Yes	Yes	Yes	No	Yes	Yes	Yes	Yes
11	115	Yes	Yes	Yes	Yes	No	Yes	No	Yes
11	116	Yes	No	Yes	Yes	No	Yes	No	Yes
11	117	Yes	Yes	Yes	Yes	Yes	Yes	No	Yes
11	118	No	No	Yes	No	Yes	Yes	No	Yes
11	119	Yes	No	Yes	No	No	No	No	Yes
11	120	Yes	Yes	Yes	No	No	No	No	No
11	121	Yes	Yes	Yes	No	Yes	Yes	No	Yes
11	122	Yes	Yes	Yes	No	No	Yes	Yes	Yes
11	123	Yes	No	Yes	Yes	Yes	No	Yes	Yes
11	124	Yes	Yes	Yes	No	Yes	No	No	Yes
11	125	Yes	Yes	Yes	Yes	Yes	Yes	Yes	Yes
11	126	Yes	Yes	Yes	Yes	Yes	Yes	Yes	Yes
11	127	Yes	Yes	Yes	No	Yes	No	No	Yes
11	128	Yes	Yes	Yes	Yes	Yes	Yes	No	No
11	129	Yes	No	Yes	Yes	No	No	No	Yes
11	130	Yes	Yes	Yes	Yes	No	No	Yes	Yes
11	131	Yes	No	Yes	No	No	No	No	No
11	132	Yes	No	Yes	No	No	No	Yes	Yes
11	133	Yes	Yes	Yes	Yes	Yes	Yes	No	Yes
11	134	Yes	No	Yes	Yes	No	No	Yes	Yes
11	135	Yes	Yes	Yes	Yes	Yes	No	No	Yes
12	241	Yes	Yes	Yes	No	Yes	Yes	No	Yes
12	242	Yes	Yes	Yes	No	No	Yes	No	No
12	243	Yes	Yes	Yes	Yes	Yes	Yes	No	Yes
12	244	Yes	Yes	Yes	No	Yes	Yes	No	Yes
12	245	Yes	Yes	Yes	Yes	Yes	Yes	No	Yes
12	246	Yes	Yes	Yes	No	Yes	Yes	Yes	No
12	247	Yes	Yes	Yes	Yes	Yes	Yes	No	Yes
12	248	Yes	Yes	Yes	Yes	Yes	Yes	Yes	No
12	249	Yes	Yes	Yes	Yes	Yes	Yes	No	Yes
12	250	Yes	Yes	Yes	No	No	No	No	No
12	251	No	Yes	Yes	No	No	No	No	Yes
12	252	Yes	No	Yes	Yes	Yes	No	No	Yes
12	253	Yes	Yes	Yes	No	Yes	Yes	No	Yes
12	254	Yes	Yes	Yes	No	No	No	No	No
12	255	Yes	Yes	Yes	No	No	No	No	No
12	256	Yes	Yes	Yes	Yes	No	No	No	Yes
12	257	Yes	Yes	Yes	Yes	No	Yes	No	Yes
12	258	Yes	Yes	Yes	Yes	No	Yes	Yes	No
12	259	Yes	Yes	Yes	No	Yes	Yes	No	Yes
12	260	Yes	No	Yes	Yes	Yes	Yes	No	No
12	261	Yes	No	Yes	No	Yes	Yes	No	Yes

Centre Number	Patient Number	VL Symptom Fever	Headache	Fatigue	Epistaxis	Abdiminal pain	Abdominal swelling	Swollen Legs	Cough
12	262	Yes	No	Yes	No	Yes	Yes	No	No
12	263	Yes	Yes	Yes	Yes	Yes	Yes	No	Yes
12	264	Yes	Yes	Yes	No	Yes	Yes	Yes	Yes
12	265	Yes	Yes	Yes	No	Yes	Yes	No	No
12	266	Yes	Yes	Yes	No	Yes	Yes	No	Yes
12	267	Yes	Yes	Yes	No	No	Yes	No	Yes
12	268	Yes	Yes	Yes	Yes	No	No	No	Yes
12	269	Yes	No	Yes	No	Yes	Yes	No	No
12	270	No	No	Yes	No	Yes	Yes	No	No
12	271	Yes	Yes	Yes	No	Yes	Yes	No	Yes
12	272	Yes	No	Yes	No	Yes	Yes	No	Yes
12	273	Yes	Yes	Yes	Yes	Yes	Yes	No	Yes
12	274	Yes	Yes	Yes	No	No	No	No	Yes
12	275	Yes	Yes	Yes	Yes	Yes	Yes	No	No
12	276	Yes	Yes	Yes	No	Yes	Yes	No	Yes
12	277	Yes	Yes	Yes	Yes	Yes	Yes	No	Yes
12	278	Yes	Yes	Yes	Yes	Yes	Yes	No	Yes
12	279	Yes	Yes	Yes	No	Yes	Yes	No	No
12	280	Yes	No	Yes	No	Yes	Yes	Yes	Yes
12	281	Yes	Yes	Yes	No	No	No	No	Yes
12	282	Yes	Yes	Yes	No	Yes	Yes	No	Yes
12	283	Yes	Yes	Yes	No	Yes	Yes	No	Yes
12	284	Yes	Yes	Yes	No	Yes	No	No	Yes
12	285	Yes	Yes	Yes	Yes	Yes	No	No	Yes
12	286	Yes	Yes	Yes	No	No	Yes	Yes	Yes
12	287	Yes	Yes	Yes	Yes	Yes	Yes	Yes	Yes
12	288	Yes	Yes	Yes	No	Yes	Yes	No	No
12	289	Yes	Yes	Yes	No	Yes	Yes	No	Yes
12	290	Yes	Yes	Yes	No	Yes	Yes	No	Yes
12	291	No	No	Yes	No	Yes	Yes	No	No
12	292	Yes	Yes	Yes	Yes	Yes	Yes	No	Yes
12	293	Yes	Yes	Yes	No	Yes	Yes	No	Yes
12	294	Yes	Yes	Yes	No	Yes	Yes	No	Yes
12	295	Yes	Yes	Yes	No	Yes	Yes	No	No
12	296	Yes	Yes	Yes	No	Yes	Yes	Yes	No
12	297	Yes	Yes	Yes	No	Yes	Yes	No	No
12	298	Yes	Yes	Yes	No	Yes	No	No	No
12	299	Yes	Yes	Yes	Yes	Yes	Yes	No	Yes
12	300	Yes	Yes	Yes	No	Yes	Yes	No	Yes
12	301	Yes	No	Yes	No	No	Yes	Yes	No
12	302	Yes	No	Yes	No	No	Yes	No	Yes
12	303	Yes	No	Yes	No	No	Yes	No	Yes
12	304	Yes	No	Yes	No	Yes	Yes	No	Yes
12	305	Yes	No	Yes	No	No	Yes	No	Yes
12	306	Yes	No	Yes	No	Yes	Yes	No	Yes
12	307	Yes	Yes	Yes	No	Yes	Yes	No	Yes
12	308	Yes	Yes	Yes	Yes	Yes	Yes	Yes	Yes
12	309	Yes	Yes	Yes	Yes	Yes	Yes	No	No
12	310	Yes	Yes	Yes	No	Yes	Yes	Yes	Yes
12	311	Yes	Yes	Yes	Yes	Yes	Yes	Yes	Yes
12	312	Yes	Yes	Yes	Yes	Yes	Yes	No	Yes
12	313	Yes	Yes	Yes	No	Yes	Yes	No	Yes

Centre Number	Patient Number	VL Symptom Fever	Headache	Fatigue	Epistaxis	Abdiminal pain	Abdominal swelling	Swollen Legs	Cough
12	314	Yes	Yes	Yes	No	Yes	Yes	No	Yes
12	315	Yes	Yes	Yes	Yes	Yes	Yes	Yes	Yes
12	316	Yes	Yes	Yes	Yes	Yes	Yes	No	Yes
12	317	Yes	Yes	Yes	No	Yes	Yes	Yes	Yes
12	318	Yes	Yes	Yes	No	Yes	Yes	No	Yes
12	319	Yes	Yes	Yes	Yes	Yes	Yes	No	Yes
12	320	Yes	Yes	Yes	Yes	No	No	No	No
12	321	Yes	Yes	Yes	Yes	Yes	Yes	No	Yes
12	322	Yes	Yes	Yes	No	Yes	Yes	Yes	No
12	323	Yes	Yes	Yes	Yes	Yes	Yes	No	No
12	324	Yes	Yes	Yes	No	Yes	Yes	No	Yes
12	325	Yes	Yes	Yes	No	Yes	Yes	No	Yes
12	326	Yes	Yes	Yes	Yes	Yes	Yes	No	Yes
12	327	Yes	No	Yes	No	Yes	Yes	No	Yes
12	328	Yes	Yes	Yes	Yes	Yes	Yes	No	Yes
12	329	Yes	Yes	Yes	No	Yes	Yes	No	Yes
12	330	Yes	Yes	Yes	Yes	Yes	Yes	No	Yes
23	361	No	No	No	Yes	No	Yes	No	Yes
23	362	No	No	No	Yes	No	Yes	No	Yes
23	363	Yes	Yes	No	No	Yes	Yes	Yes	Yes
23	364	No	Yes	No	Yes	Yes	Yes	No	No
23	365	Yes	Yes	Yes	No	Yes	Yes	No	No
23	366	Yes	Yes	Yes	Yes	Yes	Yes	No	Yes
23	367	Yes	Yes	Yes	Yes	Yes	Yes	Yes	Yes
23	368	Yes	Yes	No	No	Yes	Yes	No	Yes
23	369	Yes	Yes	Yes	Yes	No	Yes	No	Yes
23	370	Yes	Yes	Yes	No	Yes	Yes	No	Yes
23	371	Yes	Yes	No	Yes	Yes	Yes	Yes	Yes
23	372	Yes	Yes	Yes	No	Yes	Yes	No	No
23	373	Yes	No	No	No	Yes	Yes	No	No
23	374	Yes	Yes	Yes	Yes	Yes	Yes	Yes	No
23	375	Yes	Yes	Yes	No	Yes	Yes	No	Yes
23	376	Yes	Yes	No	Yes	Yes	No	No	Yes
23	377	Yes	Yes	No	No	Yes	Yes	No	No
23	378	Yes	No	No	No	Yes	Yes	No	No
23	379	Yes	Yes	Yes	No	Yes	Yes	No	No
23	380	Yes	Yes	No	Yes	Yes	Yes	Yes	No
23	381	Yes	No	No	No	Yes	Yes	No	No
23	382	Yes	No	No	No	No	Yes	No	Yes
23	383	Yes	Yes	No	Yes	Yes	Yes	Yes	Yes
23	384	Yes	Yes	No	Yes	Yes	Yes	No	No
23	385	Yes	Yes	No	Yes	Yes	Yes	No	Yes
23	386	Yes	Yes	Yes	Yes	Yes	Yes	No	Yes
23	387	Yes	Yes	Yes	No	Yes	Yes	No	Yes
23	388	Yes	Yes	Yes	No	Yes	No	No	Yes
23	389	Yes	Yes	Yes	No	Yes	Yes	No	Yes
23	390	Yes	Yes	Yes	No	No	Yes	No	Yes
23	391	Yes	Yes	Yes	Yes	No	Yes	No	Yes
23	392	Yes	Yes	Yes	No	Yes	Yes	No	Yes
23	393	Yes	Yes	Yes	No	No	No	No	No
23	394	Yes	Yes	No	Yes	Yes	Yes	No	Yes
23	395	Yes	Yes	Yes	No	Yes	Yes	No	No

Centre Number	Patient Number	VL Symptom Fever	Headache	Fatigue	Epistaxis	Abdiminal pain	Abdominal swelling	Swollen Legs	Cough
23	396	Yes	Yes	Yes	Yes	Yes	Yes	No	Yes
23	397	Yes	Yes	Yes	Yes	Yes	Yes	No	Yes
23	398	Yes	Yes	No	Yes	Yes	Yes	Yes	Yes
23	399	Yes	Yes	No	Yes	Yes	Yes	No	No
23	400	Yes	Yes	No	Yes	Yes	Yes	Yes	Yes
23	401	Yes	Yes	Yes	No	Yes	Yes	No	Yes
23	402	Yes	Yes	Yes	Yes	Yes	Yes	No	No
23	403	Yes	Yes	Yes	Yes	No	Yes	No	Yes
23	404	Yes	Yes	Yes	No	Yes	Yes	Yes	Yes
23	405	Yes	Yes	Yes	Yes	Yes	Yes	No	Yes
34	451	Yes	No	No	No	No	No	No	No
34	452	Yes	No	No	Yes	No	No	No	No
34	453	Yes	No	No	No	No	No	No	Yes
34	454	Yes	No	No	No	No	No	No	Yes
34	455	Yes	Yes	No	No	No	No	No	No
34	456	Yes	Yes	No	No	No	No	No	No
34	457	Yes	No	No	No	No	No	No	No
34	458	Yes	Yes	Yes	No	No	No	No	No
34	459	Yes	Yes	No	No	Yes	No	No	Yes
34	460	Yes	Yes	Yes	No	Yes	No	No	Yes
34	461	Yes	No	Yes	Yes	No	No	No	Yes
34	462	Yes	Yes	No	Yes	Yes	No	No	Yes
34	463	Yes	No	Yes	No	Yes	No	No	Yes
34	464	Yes	Yes	Yes	No	Yes	No	No	Yes
34	465	Yes	Yes	Yes	No	Yes	No	No	Yes
34	466	Yes	No	No	Yes	Yes	No	No	No
34	467	Yes	Yes	Yes	Yes	Yes	No	No	Yes
34	468	Yes	Yes	Yes	No	No	No	No	No
34	469	Yes	No	Yes	No	No	No	No	Yes
34	470	Yes	No	No	No	No	No	No	Yes
34	471	Yes	Yes	Yes	No	Yes	No	No	Yes
34	472	Yes	Yes	No	No	No	No	No	Yes
34	473	Yes	Yes	No	No	No	No	No	Yes
34	474	Yes	Yes	No	Yes	Yes	No	No	No
34	475	Yes	Yes	No	Yes	Yes	No	No	No
34	476	Yes	Yes	No	Yes	Yes	No	No	No
34	477	Yes	Yes	No	No	Yes	No	No	Yes
34	478	Yes	Yes	No	No	No	No	No	Yes
34	479	Yes	No	No	No	Yes	No	No	No
34	480	Yes	Yes	No	No	Yes	No	No	Yes
34	481	Yes	No	Yes	No	No	No	No	No
34	482	Yes	Yes	No	No	Yes	No	No	Yes
34	483	Yes	Yes	No	No	No	No	No	No
34	484	Yes	No	No	No	No	No	No	No
34	485	Yes	No	No	Yes	Yes	No	No	No
34	486	Yes	Yes	Yes	No	Yes	No	No	Yes
34	487	Yes	Yes	Yes	Yes	Yes	No	No	Yes
34	488	Yes	Yes	No	No	Yes	No	No	No
34	489	Yes	No	No	No	Yes	No	No	No
34	490	Yes	Yes	No	No	No	No	No	Yes
34	491	Yes	No	No	No	Yes	Yes	No	Yes
34	492	Yes	Yes	No	No	Yes	No	No	No

Centre Number	Patient Number	VL Symptom Fever	Headache	Fatigue	Epistaxis	Abdiminal pain	Abdominal swelling	Swollen Legs	Cough
34	493	Yes	No	Yes	No	Yes	No	No	No
34	494	Yes	Yes	Yes	No	No	No	No	Yes
34	495	Yes	Yes	No	No	No	No	No	Yes
34	496	Yes	Yes	No	No	No	No	No	No
34	497	Yes	Yes	Yes	No	No	No	No	Yes
34	498	Yes	Yes	Yes	Yes	No	No	No	Yes
34	499	Yes	Yes	Yes	No	No	No	No	No
34	500	Yes	Yes	Yes	Yes	No	No	No	No
34	501	Yes	No	No	No	No	No	No	No
34	502	Yes	Yes	No	No	No	No	No	No
34	503	Yes	Yes	Yes	No	No	No	No	No
34	504	Yes	Yes	No	No	Yes	No	No	No
34	505	Yes	Yes	Yes	No	No	No	No	Yes
34	506	Yes	Yes	No	Yes	Yes	No	No	No
34	507	Yes	No	No	No	No	No	No	No
34	508	Yes	No	No	No	Yes	No	No	No
34	509	Yes	No	No	No	Yes	No	No	Yes
34	510	Yes	Yes	Yes	No	No	No	No	No
34	511	Yes	No	Yes	No	No	No	No	Yes
34	512	Yes	Yes	No	No	Yes	No	No	No
34	513	Yes	Yes	Yes	No	No	No	No	Yes
34	514	Yes	No	No	No	No	No	No	Yes
34	515	Yes	Yes	No	No	Yes	No	No	No
34	516	Yes	Yes	Yes	No	No	No	No	No
34	517	Yes	No	No	No	No	No	No	Yes
34	518	Yes	No	Yes	No	No	No	No	Yes
34	519	Yes	No	No	No	Yes	No	No	Yes
34	520	Yes	Yes	Yes	No	No	No	No	Yes
34	521	Yes	Yes	No	No	No	No	No	Yes
34	522	Yes	No	No	No	Yes	No	No	Yes
34	523	Yes	No	Yes	No	No	No	No	Yes
34	524	Yes	Yes	Yes	No	No	No	No	No
34	525	Yes	Yes	Yes	No	No	No	No	Yes
34	526	Yes	No	No	No	Yes	No	No	Yes
34	527	Yes	No	Yes	No	No	No	No	No
34	528	Yes	Yes	Yes	No	No	No	No	Yes
34	529	Yes	No	Yes	No	Yes	No	No	Yes
34	530	Yes	Yes	Yes	Yes	No	No	No	Yes
34	531	Yes	No	Yes	No	Yes	No	No	Yes
34	532	Yes	Yes	No	No	No	No	No	No
34	533	Yes	Yes	Yes	No	No	No	No	No
34	534	Yes	Yes	No	No	No	No	No	Yes
34	535	Yes	No	No	No	No	No	No	Yes
34	536	Yes	Yes	No	No	Yes	No	No	No
34	537	Yes	No	No	No	Yes	No	No	No
34	538	Yes	No	No	No	No	No	No	Yes
34	539	Yes	Yes	No	No	No	No	No	No
34	540	Yes	Yes	Yes	No	Yes	No	No	No
35	646	Yes	Yes	Yes	Yes	Yes	Yes	No	Yes
35	647	Yes	Yes	Yes	No	Yes	No	No	Yes
35	648	Yes	Yes	No	No	No	Yes	No	Yes
35	649	Yes	Yes	No	No	No	Yes	No	No

Centre Number	Patient Number	VL Symptom Fever	Headache	Fatigue	Epistaxis	Abdiminal pain	Abdominal swelling	Swollen Legs	Cough
35	650	Yes	No	No	No	No	Yes	No	No
35	651	Yes	No	Yes	No	Yes	Yes	No	No
35	652	Yes	Yes	Yes	No	Yes	No	No	No
35	653	Yes	No	Yes	No	Yes	No	No	No
35	654	Yes	Yes	Yes	Yes	Yes	Yes	No	No
35	655	Yes	Yes	Yes	No	Yes	No	No	No
35	656	Yes	No	Yes	No	No	No	No	No
35	657	Yes	Yes	Yes	Yes	Yes	Yes	No	Yes
35	658	Yes	Yes	Yes	Yes	Yes	No	No	Yes
35	659	Yes	No	Yes	Yes	Yes	No	No	Yes
35	660	Yes	No	Yes	No	No	No	No	Yes
35	661	Yes	No	Yes	No	Yes	No	No	No
35	662	Yes	Yes	Yes	Yes	Yes	No	No	Yes
35	663	Yes	Yes	Yes	No	Yes	No	No	Yes
35	664	Yes	Yes	Yes	No	No	No	No	Yes
35	665	Yes	No	Yes	No	No	No	No	No
35	666	Yes	No	No	Yes	No	No	No	Yes
35	667	Yes	Yes	Yes	Yes	Yes	No	No	Yes
35	668	Yes	Yes	Yes	No	Yes	Yes	No	Yes
35	669	Yes	Yes	Yes	No	No	No	No	No
35	670	Yes	Yes	Yes	Yes	No	No	No	Yes
35	671	Yes	Yes	Yes	Yes	No	Yes	No	Yes
35	672	Yes	No	Yes	Yes	No	Yes	No	No
35	673	Yes	Yes	Yes	Yes	Yes	No	No	Yes
35	674	Yes	Yes	Yes	No	No	No	No	No
35	675	Yes	No	No	No	No	No	No	No
35	676	Yes	Yes	Yes	No	Yes	No	No	No
35	677	Yes	Yes	Yes	Yes	Yes	Yes	No	Yes
35	678	Yes	No	No	No	No	No	No	No
35	679	Yes	Yes	Yes	No	Yes	No	No	Yes
35	680	Yes	Yes	Yes	Yes	Yes	Yes	No	Yes
35	681	Yes	Yes	Yes	No	No	No	No	No
35	682	Yes	No	Yes	No	No	Yes	No	Yes
35	683	Yes	Yes	Yes	No	No	No	No	Yes
35	684	Yes	No	No	No	Yes	Yes	No	No
35	685	Yes	No	Yes	No	No	No	No	No
35	686	Yes	No	Yes	Yes	Yes	No	No	Yes
35	687	Yes	Yes	Yes	No	Yes	Yes	No	Yes
35	688	Yes	Yes	Yes	Yes	No	No	No	No
35	689	Yes	No	Yes	No	No	No	No	No
35	690	Yes	No	Yes	Yes	Yes	No	No	Yes

Centre Number	Patient Number	Medical History
11	7	URINARY TRACT INFECTION
11	14	BR. ASTHMA
11	15	T. VERSICOLOR
11	16	BRONCHIAL ASTHMA
11	18	NON - ULCER DYSPEPSIA
11	25	HOOK WORM
11	31	TYPE - 2DM
11	40	P.FALCIPARNE MALARIA
11	43	TINEA VERSICOLOR
11	46	T.FACIES
11	49	STRONGLOIDIASIS
11	55	HYPOPIGMENTED SKIN (T. VERSICOLOR)
11	55	DYSPEPSIA
11	57	COMMUNITY ACQUIRED PNEUMONIA
11	68	SCLERITIES
11	71	STAGE IV HIV/AIDS
11	72	DYSPEPSIA
11	76	DYSPEPSIA
11	83	HEMORRHOID
11	97	ACUTE GASTROENTERITIS
11	98	T. VERSICOLOR
11	100	DYSPEPSIA
11	105	DYSPEPSIA
11	109	DYSPEPSIA
11	113	DYSPEPSIA
11	114	T.VERSICOLOR
11	124	IP (AMOEBIASIS)
11	124	CELLULITIS
11	126	HERPES ZOSTER
11	127	DYSPEPSIA
11	135	T.VERSICOLOR
12	264	URINARY TRACT INFE
12	264	PLASMODIUM FALCIPARUM
12	264	GIARDIASIS
12	276	GIARDIASIS
12	283	AMEBIASIS
12	295	GIARDIASIS
12	295	HOOK WORM
12	297	GIARDIASIS
12	314	PNEUMONIA
12	325	GIARDIASIS
12	325	TINIA ALBA
23	369	ABDOMINAL WOUND
23	369	BRONCH PNEUMONIA
23	370	HEMATURIA
23	370	BIL. B/PNEUMONIA
23	371	ASCARIS LUMBRICOIDES
23	371	DENTAL CARIES
23	374	B. PNEUMONIA
23	376	PROLONGED PROTHROMBIN TIME
23	376	TINEA CAPITIS

Centre Number	Patient Number	Medical History
23	379	PNEUMONIA
23	380	ENTAMEBA HISTOLYTICA
23	380	ENTAMEBA COLI
23	380	GIADIA LAMBLIA
23	381	TINEA CAPITIS
23	381	A. HISTOLYTICA CYSTS
23	383	B.PNEUMONIA
23	384	PNEUMONIA
23	385	PROLONGED PROTHROMBIN TIME
23	385	PROTEINURIA
23	385	PNEUMONIA
23	394	MICROSCOPIC HAEMATURIA
23	394	PNEUMONIA
23	394	SEVERE ANAEMIA
23	397	BASAL PNEUMONITIS
23	400	ANAEMIA
23	400	PNEUMONIA
23	403	DENTAL CARIES
23	405	ATYPICAL PNEUMONIA
34	454	PNEUMONIA
34	456	HEPATITIS B (DISCOVERED 25.11.04)
34	460	OTITIS MEDIA
34	461	SUSPECTED TB
34	461	CONFIRMED TB (SPUTUM)
34	461	PNEUMONIA
34	465	PNEUMONIA
34	466	MALARIA
34	474	FIRST DEGREE AV BLOCK - BINIGN CONDITION
34	486	PNEUMONIA
34	490	FIRST DEGREE AV BLOCK - BINIGN CONDITION
34	492	MUCOID DIARREA
34	492	SUPERFICIAL FUNGEAL SKIN INFECTION (ABDOMINAL+ EPIGASTRIC)
34	492	SUSPECTED DYSENTRY
34	496	RIGHT VENTRICULAR CONDUCTION DELAY - BINIGN CONDITION
34	500	MALARIA
34	509	URINARY TRACT INFECTION
34	518	MALARIA
34	523	MALARIA (P. FALCIPARUM)
34	534	MALARIA
34	536	RBBB-RIGHT BUNDLE BRANCH BLOCK - BIGIGN CONDITION
34	537	RIGHT VENTRICULAR CONDUCTION DELAY - BINIGN CONDITION
34	538	WATERY DIARRHOEA
34	539	MALARIA
35	659	BRONCHOPNEUMONIA
35	660	CYSTIC CHANGES IN THE RIGHT LUNG
35	660	TINEA VERSICOLOR
35	686	BRONCHO-PNEUMONIA

Centre Number	Patient Number	Treatment	Height Baseline	Weight Baseline	Weight Day 7	Weight Day 14	Weight Day 21	Weight EOT	Weight 3 Mon FU	Weight 6 Mon FU
11	1	PM	1.72	48	51	52		52	56	58
11	2	Combination	1.68	52	52	52		54	61	58
11	3	Combination	1.72	42	44	41		41	54	54
11	4	PM	1.65	45	49	48		49	49	49
11	5	PM	1.24	20	21	22		21	23	22
11	6	Combination	1.3	25	28	28		28	30	27
11	7	PM	1.58	47	48	46		46	50	51
11	8	SSG	1.72	50	53	52	52	53	53	54
11	9	SSG	1.69	52	52	53	51	51	52	52
11	10	SSG	1.52	37	37	38	36	36	38	39
11	11	Combination	1.26	21	21	20		20	20	20
11	12	Combination	1.7	56	55	52		52	53	55
11	13	SSG	1.59	47	45	46	46	46	49	46
11	14	SSG	1.17	20	20	20	21	20	20	21
11	15	PM	1.65	47	45				49	50
11	16	Combination	1.49	45	45	46		46	50	46
11	17	Combination	1.71	58	57	59		59	58	56
11	18	SSG	1.72	52	52	52	52	52	50	55
11	19	Combination	1.71	53	53	52		51	56	53
11	20	SSG	1.21	22	24	23	23	22	24	25
11	21	SSG	1.65	48	50	50	50	50	50	50
11	22	SSG	1.29	24	23	23	23	25	25	25
11	23	PM	1.29	26	25	25		26	26	26
11	24	PM	1.1	18	16	16		17	17	18
11	25	SSG	1.2	20	17	18	19	20	20	21
11	26	Combination	1.21	20	20	20		20	20	20
11	27	PM	1.64	46	46	44		42	41	
11	28	Combination	1.4	29	29	28		29	31	32
11	29	PM	1.6	42	42	43		43	45	45
11	30	PM	1.03	15	14	15		14	15	17
11	31	SSG	1.56	39	40	38	40	42	50	48
11	32	Combination	1.54	38	38	38		39	50	50
11	33	SSG	1.74	55	56	58	59	59	59	56
11	34	PM	1.59	49	49	50		49	51	49
11	35	SSG	1.5	47	47	47	49	48	50	46
11	36	SSG	1.69	43	45	45	49	47	51	56
11	37	Combination	1.7	59	57	57		57	68	69
11	38	PM	1.76	48	48	49		51	53	54
11	39	PM	1.12	18	18	18		19	18	19
11	40	PM	1.18	16	18	17			21	21
11	41	Combination	1.01	17	17	17		17	18	19
11	42	Combination	1.71	53	54	55		56	58	58
11	43	SSG	1.51	44	44	44	45	46	45	45
11	44	PM	1.61	45	46	47		49	48	47
11	45	Combination	1.57	50	51	52		52	52	52

Centre Number	Patient Number	Treatment	Height Baseline	Weight Baseline	Weight Day 7	Weight Day 14	Weight Day 21	Weight EOT	Weight 3 Mon FU	Weight 6 Mon FU
11	46	SSG	0.95	16	15	16	16	16	16	16
11	47	SSG	1.67	44	44	45	47	49		
11	48	Combination	1.6	40	41	42		42		50
11	49	SSG	1.1	16	17	18	20	20	21	21
11	50	PM	1.73	55	55	56		55	62	64
11	51	Combination	1.43	32	33	32		33	38	38
11	52	PM	1.3	24	25	25		26	26	25
11	53	SSG	1.73	46	47	47	47	48	50	51
11	54	PM	1.64	46	47	48		48	54	52
11	55	PM	1.6	46	46	48		47	55	56
11	56	SSG	1.44	33	31	32	33	34	36	35
11	57	Combination	1.2	47	45	46		48	56	57
11	58	Combination	1.22	22	24	24		24	24	25
11	59	PM	1.27	23	22	23		24	25	26
11	60	Combination	1.72	50	49	49		49	53	51
11	61	PM	1.66	47	48	48		49	52	52
11	62	SSG	1.09	16	17	16	16	17	18	20
11	63	SSG	1.69	59	60	60	62	62	61	61
11	64	SSG	1.62	49	50	47	48	48	52	59
11	65	PM	0.93	14	15	15		15		
11	66	Combination	1.5	34	32	31		31	47	51
11	67	PM	1.31	22	24	23		24	24	25
11	68	SSG	1.17	21	19	19	19	21	21	22
11	69	Combination	1.65	41	43	43		42	42	42
11	70	Combination	1.65	49	48	48		48	55	55
11	71	Combination	1.78	46	48	48		47	50	49
11	72	SSG	1.72	50	48	49			51	50
11	73	PM	1.6	48	48	49		48	55	54
11	74	PM	1.68	49	48	49		49	56	54
11	75	Combination	1.7	52	53	53		53	54	57
11	76	PM	1.62	45	45	45		46	50	50
11	77	PM	1.27	20	20	22		23	23	24
11	78	SSG	1.63	46	48	50	50	52	49	53
11	79	PM	0.99	12	13	13		15	14	15
11	80	PM	1.19	22	21	22		23	23	25
11	81	Combination	1.74	53	53	55		54	55	62
11	82	SSG	1.32	25	28	27	26	28	29	29
11	83	SSG	1.67	40	39	40	39	40	47	54
11	84	SSG	1.28	19	23	23	24	24	23	25
11	85	SSG	1.52	42	42	43	43	44	50	
11	86	Combination	0.9	11	13	13		13	14	16
11	87	Combination	1.53	35	37	38		38	40	43
11	88	PM	1.6	53	52	53		55	57	58
11	89	Combination	1.76	59	59	55		57	65	68
11	90	Combination	1	14	17	16		17		

Centre Number	Patient Number	Treatment	Height Baseline	Weight Baseline	Weight Day 7	Weight Day 14	Weight Day 21	Weight EOT	Weight 3 Mon FU	Weight 6 Mon FU
11	91	PM	1.62	52	53	53		54	55	55
11	92	Combination	1.19	16	17	17		18	20	20
11	93	SSG	1.7	46	45	45	46	50	52	51
11	94	SSG	0.9	14	15	14	15	14	15	16
11	95	SSG	0.98	15	15	16	17	17	16	16
11	96	Combination	1.14	17	19	20		20	21	21
11	97	Combination	1.62	37	38	41		43	49	50
11	98	PM	1.7	35	35	34		33	45	48
11	99	SSG	1.56	38	38	39	38	39	42	44
11	100	PM	1.68	44	45	45		46		
11	101	PM	1.68	44	43	43		45	50	46
11	102	Combination	1.7	51	51	51		51	60	57
11	103	Combination	1.8	51	50	51		51	57	61
11	104	PM	1.4	30	29	29		30	33	32
11	105	SSG	1.6	36	36	38	39	40	46	51
11	106	Combination	1.74	50	49	50		49	58	60
11	107	PM	1.64	47	48	49		50	59	55
11	108	PM	1.65	46	47	48		50	55	57
11	109	SSG	1.63	47	47	48	49	48	56	55
11	110	PM	1.06	15	15	14		15	17	19
11	111	Combination	1.68	47	49	51		52	58	59
11	112	Combination	1.6	46	44	47		47	50	50
11	113	SSG	1.62	46	44	46	47	47	54	
11	114	SSG	1.62	51	51	51	52	51	56	56
11	115	Combination	1.36	26	28	28		28	34	32
11	116	SSG	1.64	48	47	47	48	51	54	54
11	117	PM	1.8	54	52	53		52	58	
11	118	PM	1.59	47	49	50		52	54	57
11	119	SSG	1.66	45	47	49	50	52	54	51
11	120	Combination	1.71	46	47	48		48	51	50
11	121	Combination	1.07	15	15	15		15	16	15
11	122	SSG	1.68	49	52	50	52	53		
11	123	SSG	1.6	45	45	46	48	49	48	48
11	124	SSG	1.74	55	58	63	61	56		64
11	125	SSG	1.81	51	52	50	52	54	56	63
11	126	Combination	1.69	52	52	52		52		57
11	127	PM	1.51	27	28	29		30	33	32
11	128	Combination	1.8	52	53	51		50	57	54
11	129	PM	1.58	40	39	40		42	48	46
11	130	Combination	1.72	47	44	45		45	54	52
11	131	PM	1.08	17	17	18		18	17	18
11	132	SSG	1.68	50	49	51	50	51	55	54
11	133	Combination	1.8	48	48	48		49	59	54
11	134	PM	1.7	53	51	52		52	57	63
11	135	PM	1.62	47	48	48		50	51	51

Centre Number	Patient Number	Treatment	Height Baseline	Weight Baseline	Weight Day 7	Weight Day 14	Weight Day 21	Weight EOT	Weight 3 Mon FU	Weight 6 Mon FU
12	241	Combination	1.55	42	44.5	44		45		55
12	242	SSG	1.2	20	21	22	21	21	23	23
12	243	PM	1.18	22	22	25		26	28.5	29
12	244	SSG	1.67	53	56	55	56	56	57	57
12	245	PM		43	43	43		44	45	
12	246	PM	1.87	47	49	49		49	48	
12	247	PM	1.33	24	26	25		26.3	30	30
12	248	PM	1.87	53.5	55	55		55	60	38
12	249	Combination	1.54	35	35	34		34	38	39
12	250	SSG	1.56	37	35.5	38	36	37	41.5	43
12	251	Combination	1.71	46	46	46		46	52	52
12	252	SSG	1.63	34	38	40	41	40	43	46
12	253	SSG	1.4	25	27	27	27	29	30	31
12	254	Combination	1.22	20	20	20		22	24	27
12	255	Combination	1.54	37	38	37		37	40	40
12	256	Combination	1.5	40	40	40		40	46	48
12	257	SSG	1.58	42	43	42	43	45	47	48
12	258	SSG	1.31	24	25	25	25	26		28
12	259	PM	1.8	58	60	60		65	64	64
12	260	PM	1.48	37	39	39		39		40
12	261	Combination	1.4	35	35	36		36	38	39
12	262	PM	1.26	22	25	26		26	27	27
12	263	SSG	1.62	45	47	48	48	50	54	51
12	264	Combination	1.29	24	25	24		24	25	26
12	265	SSG	1.02	15	15	15	16	15		
12	266	PM	1.64	59	60	62		64	60	60
12	267	SSG	1.68	57	57	58	57	56	52	61
12	268	PM	1.71	60	60	62		64	66	67
12	269	Combination	1.55	41	40	42		43	50	50
12	270	Combination	1.24	22	23	22		23		25
12	271	SSG	1.72	59	57	57	55	54	66	65
12	272	PM	0.88	11	12	12		12.5	13	14.5
12	273	SSG	1.32	26	26.5	26	28	28	28	29
12	274	SSG	1.27	23	24	24	25	25	25	27
12	275	Combination	1.56	38	39	38		38	41	43
12	276	PM	1.25	20	21.5	22		24	24	26
12	277	Combination	1.64	44	45	45		46	48	52
12	278	SSG	1.64	45	46	45	47	49	57	
12	279	PM	1.72	44	46	48		49	56	61
12	280	SSG	0.9	12	13	14	15	16	16.5	17.5
12	281	Combination	1.66	45	46	45		45	48	52
12	282	PM	1.2	18	20	20		21	22	24
12	283	PM	1.64	54	55	56		56	57	56
12	284	Combination	1.12	14	16	16		16	18	20
12	285	Combination	1.64	38	38	41		42	44	52

Centre Number	Patient Number	Treatment	Height Baseline	Weight Baseline	Weight Day 7	Weight Day 14	Weight Day 21	Weight EOT	Weight 3 Mon FU	Weight 6 Mon FU
12	286	PM	1.45	31	32	32		32	35	36
12	287	SSG	1.39	26	28	28	29	29	31	31
12	288	Combination	1.2	22	22	22		22	24	25
12	289	Combination	1.62	50	50	51		51.5	55	57
12	290	SSG	1.385	26	28	28	30	28		32
12	291	SSG	1.6	48	50	50	51	52	55	56
12	292	PM	1.74	50	50	50		50		63
12	293	SSG	1.4	30	30	30	30	30		35
12	294	Combination	1.08	19	18	18		19	20	22
12	295	Combination	1.4	25	25	27		27		32
12	296	PM	1.26	23	24	24		24	24	25
12	297	SSG	1.56	47	47	47				
12	298	PM	1.33	27	26	27		27	28	28
12	299	Combination	1.86	56	56	55		57		65
12	300	PM	1.62	45	44	44		46	49	48
12	301	SSG	1.7	45	45	44.5	45	46	52	52
12	302	SSG	0.92	13	13	14	15	15	18	19
12	303	PM	1.6	40	40	40		41	46	48
12	304	Combination	1.63	42	44	42		43	51	50
12	305	PM	0.99	14	14	14		15	16	17
12	306	Combination	1	15	14	14.5		14.5	16	16
12	307	Combination	1.76	61	62	65		66	68	74
12	308	PM	1.85	57	56	57.5		58	62	63
12	309	PM	1.75	53	53	53		53	58	59
12	310	SSG	1.41	30	30	30	31	32	34	36
12	311	SSG	1.27	27	28	27	28	28	33	31
12	312	Combination	1.62	48	48	50		50	56	62
12	313	PM	1.75	58	58	59		60	66	67
12	314	SSG	1.78	46	48	46	47	49	51	55
12	315	Combination	1.14	18	20	22		21	21	21
12	316	SSG	1.73	54	56	53		54	61	60
12	317	SSG	0.95	13	14	14	14	14		14
12	318	Combination	1.15	16	17	16		17	19	20
12	319	Combination	1.28	23	24	24		24	26	26
12	320	Combination	1.23	22	22	22		22	23	24
12	321	Combination	1.16	21	21	21		21	22	24
12	322	PM	1.55	40	39	39		41	45	44
12	323	SSG	1.8	58	59	59	61	61	62	61
12	324	SSG	1.84	61	63	62	65	65	68	70
12	325	PM	1.74	55	58	60		60	62	60
12	326	PM	1.74	45	46	45		48	58	62
12	327	Combination	1.42	30	33	33		32	34	34
12	328	PM	1.43	29	28	30		28	30	30
12	329	SSG	1.02	14	15	15	15	15	16	16
12	330	PM	1.3	26	29	29		29	33	27

Centre Number	Patient Number	Treatment	Height Baseline	Weight Baseline	Weight Day 7	Weight Day 14	Weight Day 21	Weight EOT	Weight 3 Mon FU	Weight 6 Mon FU
23	361	Combination	1.2	17	18	19		20	19	20
23	362	PM	1.2	39	39	38		40	38	40
23	363	PM	1.1	18	17	17		18	18	18
23	364	Combination	1.31	23	23	23		24	24	25
23	365	SSG	1.6	41	42	42	42	45	41	46
23	366	Combination	1.41	28	30	31		31	26	30
23	367	SSG	1.64	44	43	44	44	45	58	50
23	368	PM	1.54	48	51	50		51	57	61
23	369	Combination	1.47	26	26	27		28	31	32
23	370	SSG	1.18	18	19	19	20	20	18	20
23	371	PM	1.43	38	37	37		38	38	41
23	372	PM	1.72	55	55	57		59	58	58
23	373	SSG	1.64	40	41	43	44	46	45	46
23	374	SSG	1.51	39	40	39	40	41	43	42
23	375	Combination	1.37	23	25	26		27	26	26
23	376	SSG	1.56	36	37	38	38	39	41	42
23	377	Combination	1.58	46	48					54
23	378	Combination	1.16	17	17	18		18	17	20
23	379	PM	1.17	18	20	20		20	19	21
23	380	SSG	1.15	16	17	17	18	18	18	18
23	381	PM	1.37	29	29	30		33	31	32
23	382	Combination	1.67	40	40	41		42	45	45
23	383	PM	1.68	42	44	46		49	49	50
23	384	SSG	1.47	32	34	35	35	35	34	34
23	385	SSG	1.5	28	31	32	31	31	31	32
23	386	Combination	1.55	30	32	33		33	34	35
23	387	Combination	1.69	43	44	45		45	45	48
23	388	PM	1.47	26	28	29		30	30	30
23	389	SSG	1.38	29	30	31	32	33	33	31
23	390	PM	1.72	45	45	47		48	49	50
23	391	Combination	1.45	30	30	31		32	30	30
23	392	Combination	1.15	18	18	18			18	19
23	393	SSG	1.34	23	24	26	26	28	25	26
23	394	PM	1.24	19	20	22		22	22	22
23	395	PM	1.69	54	54	56		58	59	56
23	396	SSG	1.67	51	52	53	53	55	55	55
23	397	SSG	1.56	30	32	34	35	37	36	35
23	398	Combination	1.24	20	21	22		22	24	23
23	399	PM	1.17	20	21	21		23	21	22
23	400	PM	1.21	19	19	18		20	19	21
23	401	Combination	1.72	55	57	56		57	56	55
23	402	Combination	1.6	43	44	43		44	47	47
23	403	SSG	1.7	45	46	43	45	46	50	52
23	404	SSG	1.64	45	47	48	50	52		50
23	405	PM	1.52	39	39	41		42	41	43

Centre Number	Patient Number	Treatment	Height Baseline	Weight Baseline	Weight Day 7	Weight Day 14	Weight Day 21	Weight EOT	Weight 3 Mon FU	Weight 6 Mon FU
34	451	Combination	1.16	16	18	18		18		21
34	452	PM	1.44	28	28	29		29		
34	453	PM	1	15	14	16		16		
34	454	Combination	0.96	12	12	12		12		14
34	455	PM	1.31	21	24	23		23		
34	456	SSG	1.2	20	22	22	23	24	23	
34	457	Combination	1.72	48	55	55		56		
34	458	PM	1.36	24	26	25		27	27	
34	459	Combination	1.38	24	25	24		26	27	27
34	460	PM	0.96	12	12	13		13		
34	461	SSG	1.74	55	60	59				
34	462	SSG	0.95	11	12	12	12	13		13
34	463	Combination	0.89	10	11	11		12		13
34	464	SSG	1.77	52	54	54	54	54		58
34	465	SSG	1.73	51	56	56	56	56		55
34	466	Combination	1.19	20	21	21		22		
34	467	PM	1.52	30	32	32		32	30	
34	468	Combination	1.61	46	48	49		49		51
34	469	SSG	1.4	25	26	27	26	27		28
34	470	Combination	0.98	14	14	16		16		15
34	471	PM	1.42	29	30	31		32		
34	472	SSG	0.94	13	14	14	15	14		14
34	473	PM	1.03	16	16	15		15		
34	474	Combination	1.31	24	26	26		27		
34	475	PM	1.19	19	19	19		19		
34	476	PM	1.31	26	27	27		28	27	
34	477	SSG	1.24	18	18	19	19	18		17
34	478	Combination	0.9	10	11	12		12		12
34	479	SSG	0.91	10	11	12	12	11		12
34	480	SSG	1.46	28	29	30	30	30		31
34	481	Combination	1.34	23	23	25		25		29
34	482	Combination	1.68	55	57	56		56		63
34	483	PM	1.87	57	57	55		56		55
34	484	PM	1.07	15	13					
34	485	Combination	1.12	15	15	16		19		17
34	486	Combination	1.44	32	33	34		33		37
34	487	SSG	1.65	37	41	40	41	41		47
34	488	SSG	1.29	20	22	21	23	23	23	23
34	489	SSG	1.48	26	27	27	26	27		29
34	490	PM	1.28	19	20	20		20		23
34	491	PM	1.71	50	53	51		51		
34	492	PM	1.91	66	76	75		72		74
34	493	SSG	1.52	33	36	33	33	36	36	
34	494	Combination	1.76	58	49	49		51		62
34	495	SSG	1.12	15	17	16	16	17		17

Centre Number	Patient Number	Treatment	Height Baseline	Weight Baseline	Weight Day 7	Weight Day 14	Weight Day 21	Weight EOT	Weight 3 Mon FU	Weight 6 Mon FU
34	496	SSG	1.73	63	63	63	62	65		66
34	497	PM	1.66	49	49	50		50	52	
34	498	SSG	1.47	32	31	34	34	34		35
34	499	Combination	1.31	26	26	28		27		27
34	500	PM	1.55	34	34	36		39		37
34	501	Combination	1.32	22	23	24		24		26
34	502	Combination	1.47	35	34	34		35		39
34	503	PM	1.37	25	23	23		25		
34	504	Combination	1.1	16	15	17		17		
34	505	SSG	1.56	52	54	57	58	57		59
34	506	SSG	1.25	18	19	19	19	20		21
34	507	SSG	1.09	15	17	18	17	18		18
34	508	PM	0.98	14	13	14		14		
34	509	PM	1.89	55	58	61		61		
34	510	Combination	1.22	19	20	22		23		22
34	511	Combination	1.08	16	16	18		17		17
34	512	PM	1.06	14	15	16		16		
34	513	Combination	1.22	21	23	24		21	25	26
34	514	SSG	1.21	21	22	24	24	23		25
34	515	SSG	1.28	24	24	26	26	26	26	
34	516	PM	1.63	44	44	45		44		46
34	517	PM	1.39	27	28	27		28		
34	518	SSG	0.96	14	16	14	15	15		16
34	519	PM	1.31	20	22	22		22	20	
34	520	SSG	1.67	53	52	51	52	52		65
34	521	PM	1.1	16	17	16		17		
34	522	Combination	1.64	48	51	53		53		
34	523	SSG	1.31	23	22	24	24	24		27
34	524	Combination	1.47	33	34	33		34		35
34	525	Combination	1.22	22	22	22		23		22
34	526	SSG	1.02	13	13	13	14	13		13
34	527	Combination	1.65	47	46	44		45		56
34	528	PM	1.16	19	19	19		19		
34	529	Combination	1.05	13	15	15		15		15
34	530	Combination	1.76	53	55	57		57		62
34	531	SSG	0.98	13	14	15	16	15		
34	532	Combination	0.95	12	13	13		13		14
34	533	SSG	1.45	28	29	29	29	31		33
34	534	PM	1.09	14	14	13		14		
34	535	PM	1.36	28	27	28		29	28	
34	536	PM	1.34	25	25	26		27		30
34	537	SSG	0.97	11	12	12	13	13		13
34	538	PM	1.15	17	18	18		18		
34	539	Combination	1.3	21	21	22		21		19
34	540	SSG	1.43	29	30	30	32	31		35

Centre Number	Patient Number	Treatment	Height Baseline	Weight Baseline	Weight Day 7	Weight Day 14	Weight Day 21	Weight EOT	Weight 3 Mon FU	Weight 6 Mon FU
35	646	PM	1.58	32	32	34		36		46
35	647	PM	1.83	61	61	65		65	63	65
35	648	Combination	1	15	15	15		15	16	18
35	649	Combination	1.45	29	32	32		32	35	37
35	650	SSG	1.04	15	15	16	16	16	16	17
35	651	SSG	1.09	16	16	16	17	17	20	22
35	652	PM	1.25	20	20	20		21		21
35	653	Combination	1.12	15	15	15		15	17	17
35	654	SSG	1.01	13	13	13	13	14	13	15
35	655	Combination	1.43	29	29	29		29	31	35
35	656	SSG	1.36	22	22	22	22	23	25	28
35	657	PM	1.28	22	22	23		26	30	31
35	658	Combination	1.61	36	37	39		39	43	46
35	659	SSG	1.08	16	16	17	16	16	20	22
35	660	PM	1.76	52	52	53		53	55	56
35	661	Combination	1.75	50	50	50		53	55	59
35	662	PM	1.82	61	61	62		61	58	63
35	663	SSG	1.75	57	57	58	58	58	60	60
35	664	Combination	1.71	73	70					70
35	665	SSG	1.41	24	25	27	28	28	30	30
35	666	SSG	1.47	33	33	35	36	37	36	39
35	667	Combination	1.38	23	25	25		26	28	30
35	668	PM	1.5	42	40	40		42		49
35	669	PM	1.47	29	29	30		31		
35	670	SSG	1.34	22	22	25	25	26	27	28
35	671	PM	1.22	18	18	19		17		20
35	672	Combination	1.28	23	23	23		24		26
35	673	SSG	1.64	26	28	27	28	28	30	30
35	674	Combination	1.25	20	20	20		21	25	30
35	675	PM	1.43	24	24	25		26		24
35	676	PM	1.24	18	18	17		17	18	21
35	677	SSG	1.8	61	57					
35	678	Combination	1.54	31	30	32		33	35	37
35	679	SSG	1.47	32	32	32	32	34	38	38
35	680	Combination	1.33	22	22	24		25	28	30
35	681	Combination	1.62	46	46	48		48	47	47
35	682	PM	1.27	15	16	15		16		16
35	683	Combination	1.32	30	31	30		31		32
35	684	SSG	1.58	49	47	47	47	45	50	51
35	685	PM	1.59	37	37	38		40	40	42
35	686	PM	1.65	47	47	48		48	50	53
35	687	SSG	1.6	49	49	50	51	51	51	53
35	688	PM	1.65	39	41	41		43	47	46
35	689	Combination	1.64	42	42	43		43	55	58
35	690	SSG	1.43	21	22	25	26	26	28	30

Centre Number	Patient Number	Treatment	Heart rate (Beats per minute)						
			Baseline	Day 7	Day 14	Day 21	EOT	3Mon FU	6Mon FU
11	1	PM	84	100	104		100	100	84
11	2	Combination	94	88	88		84	84	82
11	3	Combination	88	80	88		90	78	78
11	4	PM	91	80	96		80	64	64
11	5	PM	83	116	84		80	90	82
11	6	Combination	104	116	108		108	88	78
11	7	PM	78	100	90		88	80	80
11	8	SSG	82	80	60	80	72	68	58
11	9	SSG	70	68	76	68	84	96	64
11	10	SSG	80	74	86	76	78	80	64
11	11	Combination	88	124	130		112	112	108
11	12	Combination	75	104	84		76	74	65
11	13	SSG	80	100	84	76	80	65	67
11	14	SSG	96	112	108	116	112	108	112
11	15	PM	100	124				80	76
11	16	Combination	88	74	90		88	80	82
11	17	Combination	80	80	68		84	78	64
11	18	SSG	72	68	80	92	86	70	68
11	19	Combination	74	88	94		102	80	74
11	20	SSG	90	94	76	104	80	80	82
11	21	SSG	74	74	64	104	70	54	60
11	22	SSG	140	104	98	90	90	92	74
11	23	PM	74	86	92		96	67	66
11	24	PM	120	123	120		104	96	100
11	25	SSG	110	70	84	96	90	96	88
11	26	Combination	100	94	104		108	78	94
11	27	PM	96	90	88		94	116	
11	28	Combination	80	88	84		86	84	60
11	29	PM	92	84	90		84	78	80
11	30	PM	120	120	108		100	110	94
11	31	SSG	98	90	76	94	90	80	84
11	32	Combination	100	98	94		78	88	90
11	33	SSG	80	88	64	62	70	66	66
11	34	PM	68	74	72		72	60	54
11	35	SSG	88	96	94	94	100	78	78
11	36	SSG	108	100	88	84	102	68	60
11	37	Combination	100	116	100		94	67	70
11	38	PM	84	80	90		80	64	88
11	39	PM	100	110	96		96	96	94
11	40	PM	110	120	120			102	84
11	41	Combination	86	100	96		98	100	110
11	42	Combination	88	80	96		96	68	57
11	43	SSG	100	100	90	108	84	100	54
11	44	PM	80	80	70		70	70	78
11	45	Combination	88	84	94		84	80	76
11	46	SSG	100	80	70	88	88	92	80
11	47	SSG	120	96	84	94	80		
11	48	Combination	100	86	92		95		80
11	49	SSG	108	93	80	78	86	90	88
11	50	PM	94	104	102		84	80	74
11	51	Combination	106	104	108		98	88	94

Centre	Patient	Treatment	Heart rate (Beats per minute)						
Number	Number		Baseline	Day 7	Day 14	Day 21	EOT	3Mon FU	6Mon FU
11	52	PM	102	92	88		84	80	74
11	53	SSG	120	100	80	88	86	72	84
11	54	PM	102	84	86		80	80	80
11	55	PM	96	109	80		90	64	68
11	56	SSG	120	100	96	94	92	63	74
11	57	Combination	120	100	94		100	78	82
11	58	Combination	106	108	90		100	102	94
11	59	PM	112	100	116		112	100	88
11	60	Combination	104	90	100		86	60	84
11	61	PM	116	99	100		96	96	88
11	62	SSG	108	100	120	100	84	94	80
11	63	SSG	94	94	80	80	76	80	72
11	64	SSG	96	120	90	80	94	64	68
11	65	PM	120	100	105		92		
11	66	Combination	120	108	98		96	98	80
11	67	PM	112	68	80		98	84	80
11	68	SSG	140	100	98	80	82	85	90
11	69	Combination	80	84	80		78	86	80
11	70	Combination	100	94	84		70	80	74
11	71	Combination	80	82	80		86	80	100
11	72	SSG	104	100	110			80	68
11	73	PM	100	82	82		68	84	72
11	74	PM	69	80	76		76	72	80
11	75	Combination	114	100	100		84	84	80
11	76	PM	96	98	84		86	64	72
11	77	PM	88	82	92		94	80	84
11	78	SSG	96	78	80	84	80	78	82
11	79	PM	108	98	96		86	96	92
11	80	PM	120	84	88		100	80	76
11	81	Combination	94	96	90		80	96	76
11	82	SSG	108	84	100	96	106	84	100
11	83	SSG	108	100	100	100	96	102	80
11	84	SSG	88	80	98	86	100	90	72
11	85	SSG	120	114	90	98	96	80	
11	86	Combination	110	102	106		98	90	100
11	87	Combination	108	86	90		98	88	92
11	88	PM	124	96	83		90	70	64
11	89	Combination	104	96	90		98	100	90
11	90	Combination	120	98	88		97		
11	91	PM	102	92	84		82	82	78
11	92	Combination	80	96	84		80	90	84
11	93	SSG	110	100	104	92	96	82	82
11	94	SSG	112	88	80	100	94	108	98
11	95	SSG	94	82	84	94	100	80	84
11	96	Combination	108	98	80		86	90	60
11	97	Combination	110	92	88		96	80	62
11	98	PM	120	116	110		100	104	72
11	99	SSG	104	100	96	80	80	92	78
11	100	PM	106	96	96		92		
11	101	PM	94	80	80		98	84	76
11	102	Combination	104	100	80		94	74	82

Centre Number	Patient Number	Treatment	Heart rate (Beats per minute) Baseline	Day 7	Day 14	Day 21	EOT	3Mon FU	6Mon FU
11	103	Combination	106	100	84		96	78	64
11	104	PM	112	108	104		100	82	78
11	105	SSG	120	100	112	104	112	78	70
11	106	Combination	100	84	84		84	68	64
11	107	PM	70	84	104		92	72	66
11	108	PM	96	94	90		88	82	80
11	109	SSG	112	98	88	96	112	92	68
11	110	PM	112	126	108		124	80	86
11	111	Combination	100	82	86		82	72	68
11	112	Combination	80	120	98		86	60	64
11	113	SSG	106	82	80	92	88	80	
11	114	SSG	110	82	62	88	98	76	82
11	115	Combination	126	100	92		86	80	86
11	116	SSG	120	92	94	80	100	72	86
11	117	PM	88	80	84		98	70	
11	118	PM	96	110	100		98	100	78
11	119	SSG	112	120	124	100	96	80	70
11	120	Combination	102	92	100		96	88	88
11	121	Combination	116	106	100		102	112	90
11	122	SSG	72	80	86	90	90		
11	123	SSG	108	98	84	104	96	90	86
11	124	SSG	126	96	82	102	104		80
11	125	SSG	116	80	76	94	112	64	68
11	126	Combination	68	88	86		80		92
11	127	PM	106	118	116		96	102	82
11	128	Combination	100	108	90		86	88	96
11	129	PM	110	108	88		92	80	76
11	130	Combination	92	104	110		96	100	76
11	131	PM	126	120	86		120	100	86
11	132	SSG	120	80	76	80	96	78	80
11	133	Combination	130	120	112		98	78	64
11	134	PM	100	104	106		94	100	80
11	135	PM	104	86	86		86	86	70
12	241	Combination	84	92	80		76		78
12	242	SSG	90	96	88	84	88	88	86
12	243	PM	104	102	92		88	76	76
12	244	SSG	84	92	76	80	84	86	76
12	245	PM	100	80	80		80	96	
12	246	PM	88	82	88		80	90	
12	247	PM	104	96	80		100	89	88
12	248	PM	80	64	68		68	89	64
12	249	Combination	76	80	92		104	100	72
12	250	SSG	96	80	80	88	80	74	87
12	251	Combination	104	80	84		92	78	80
12	252	SSG	84	76	84	80	100	80	82
12	253	SSG	100	96	96	90	80	90	90
12	254	Combination	96	100	100		100	92	80
12	255	Combination	76	80	88		88	88	88
12	256	Combination	104	90	94		100	76	76
12	257	SSG	90	100	94	90	96	70	86
12	258	SSG	82	94	88	84	80		82

Centre Number	Patient Number	Treatment	Heart rate (Beats per minute) Baseline	Day 7	Day 14	Day 21	EOT	3Mon FU	6Mon FU
12	259	PM	80	87	92		74	72	68
12	260	PM	84	88	92		80		76
12	261	Combination	100	80	92		80	84	82
12	262	PM	104	94	96		96	84	96
12	263	SSG	88	84	96	90	92	80	74
12	264	Combination	84	98	90		96	92	80
12	265	SSG	100	100	100	90	88		
12	266	PM	76	72	80		80	72	68
12	267	SSG	80	80	76	76	60	76	84
12	268	PM	80	82	80		82	68	78
12	269	Combination	92	84	84		80	68	82
12	270	Combination	100	108	80		80		68
12	271	SSG	96	80	72	92	68	48	70
12	272	PM	96	100	100		90	104	98
12	273	SSG	88	76	88	76	90	66	80
12	274	SSG	80	96	96	80	90	68	72
12	275	Combination	84	92	84		80	64	70
12	276	PM	84	92	88		100	88	92
12	277	Combination	100	92	82		80	82	60
12	278	SSG	84	98	94	92	90	68	
12	279	PM	84	90	90		96	72	68
12	280	SSG	96	110	110	100	100	108	104
12	281	Combination	92	96	92		88	80	76
12	282	PM	92	92	86		86	92	84
12	283	PM	92	60	76		64	64	60
12	284	Combination	112	120	100		120	100	96
12	285	Combination	96	90	84		80	74	89
12	286	PM	88	100	108		100	88	80
12	287	SSG	96	128	100	100	120	89	84
12	288	Combination	120	108	96		100	88	80
12	289	Combination	88	96	80		80	76	72
12	290	SSG	72	106	104	104	92		88
12	291	SSG	108	88	82	82	82	84	80
12	292	PM	100	100	92		84		82
12	293	SSG	120	100	96	100	100		88
12	294	Combination	112	100	92		90	104	90
12	295	Combination	104	76	108		100		72
12	296	PM	120	90	92		84	84	90
12	297	SSG	92	96	92				
12	298	PM	104	96	96		82	88	80
12	299	Combination	92	80	100		86		60
12	300	PM	76	84	60		64	68	88
12	301	SSG	84	84	100	82	86	72	76
12	302	SSG	116	112	100	100	100	100	104
12	303	PM	112	100	100		98	64	80
12	304	Combination	92	88	80		80	80	72
12	305	PM	112	108	100		80	104	108
12	306	Combination	100	112	100		80	108	104
12	307	Combination	88	86	82		76	68	72
12	308	PM	100	80	80		76	72	64
12	309	PM	108	88	82		80	80	84

Centre Number	Patient Number	Treatment	Heart rate (Beats per minute) Baseline	Day 7	Day 14	Day 21	EOT	3Mon FU	6Mon FU
12	310	SSG	112	108	96	86	86	86	76
12	311	SSG	96	96	96	88	92	90	92
12	312	Combination	112	88	90		88	64	76
12	313	PM	100	80	80		80	68	64
12	314	SSG	92	80	72	80	68	76	60
12	315	Combination	112	112	108		108	86	92
12	316	SSG	64	64	84			60	52
12	317	SSG	108	104	120	108	104		92
12	318	Combination	132	108	116		112	108	108
12	319	Combination	128	108	100		100	100	80
12	320	Combination	104	92	100		90	96	80
12	321	Combination	116	100	108		100	72	68
12	322	PM	88	88	80		84	82	84
12	323	SSG	72	60	60	68	76	60	60
12	324	SSG	76	76	78	72	76	68	80
12	325	PM	68	60	60		76	76	66
12	326	PM	100	96	88		76	100	76
12	327	Combination	104	92	92		88	84	82
12	328	PM	120	104	100		88	88	76
12	329	SSG	128	118	100	108	104	106	94
12	330	PM	112	84	84		88	86	80
23	361	Combination	112	108	96		96	102	80
23	362	PM	74	112	96		84	100	100
23	363	PM	144	107	96		100	100	104
23	364	Combination	120	104	96		100	112	100
23	365	SSG	104	92	84	84	98	88	84
23	366	Combination	106	102	106		92	84	86
23	367	SSG	100	100	112	84	96	80	78
23	368	PM	80	100	96		86	86	84
23	369	Combination	80	84	104		108	94	80
23	370	SSG	104	100	104	112	104	100	96
23	371	PM	100	104	100		96	108	80
23	372	PM	76	80	80		76	96	86
23	373	SSG	88	98	78	88	84	84	52
23	374	SSG	108	108	102	100	84	102	100
23	375	Combination	116	100	96		96	100	92
23	376	SSG	94	140	80	88	96	94	78
23	377	Combination	92	98					76
23	378	Combination	128	112	104		104	96	104
23	379	PM	132	108	102		102	105	94
23	380	SSG	120	112	100	104	104	96	114
23	381	PM	104	100	104		94	108	100
23	382	Combination	120	100	86		80	104	96
23	383	PM	88	120	102		88	92	88
23	384	SSG	100	96	96	100	86	98	80
23	385	SSG	98	104	96	102	98	100	100
23	386	Combination	100	100	100		98	100	96
23	387	Combination	98	98	96		100	104	96
23	388	PM	80	98	110		100	88	100
23	389	SSG	120	102	100	100	96	80	88
23	390	PM	100	92	88		98	96	92

Centre Number	Patient Number	Treatment	Heart rate (Beats per minute)						
			Baseline	Day 7	Day 14	Day 21	EOT	3Mon FU	6Mon FU
23	391	Combination	100	104	96		104	108	80
23	392	Combination	120	120	105			100	110
23	393	SSG	110	122	100	100	94	104	78
23	394	PM	126	100	118		104	100	104
23	395	PM	105	100	100		84	80	84
23	396	SSG	98	96	94	88	86	80	84
23	397	SSG	98	104	112	96	94	80	90
23	398	Combination	104	120	108		98	88	88
23	399	PM	120	120	118		100	112	96
23	400	PM	120	104	104		96	120	102
23	401	Combination	84	84	84		96	82	80
23	402	Combination	108	96	100		98	88	88
23	403	SSG	116	96	90	90	96	64	84
23	404	SSG	80	76	76	96	94		80
23	405	PM	80	98	96		98	92	80
34	451	Combination	120	120	104		108		92
34	452	PM	104	120	112		96		
34	453	PM	128	120	120		104		
34	454	Combination	136	120	124		108		128
34	455	PM	120	112	120		96		
34	456	SSG	128	124	120	120	120	122	
34	457	Combination	112	104	104		92		
34	458	PM	140	136	124		116	128	
34	459	Combination	100	100	104		104	71	80
34	460	PM	112	108	104		104		
34	461	SSG	96	108	120				
34	462	SSG	140	124	128	120	120		120
34	463	Combination	140	88	108		108		132
34	464	SSG	100	112	104	96	89		61
34	465	SSG	112	100	88	96	124		88
34	466	Combination	112	96	92		77		
34	467	PM	100	100	112		96	139	
34	468	Combination	92	88	88		88		80
34	469	SSG	120	100	120	120	100		88
34	470	Combination	140	128	120		120		130
34	471	PM	104	120	120		100		
34	472	SSG	120	112	112	120	108		96
34	473	PM	124	108	112		104		
34	474	Combination	112	104	92		88		96
34	475	PM	128	116	114		116		
34	476	PM	88	92	95		96	101	
34	477	SSG	112	96	96	120	108		104
34	478	Combination	124	120	120		100		88
34	479	SSG	132	108	100	108	96		88
34	480	SSG	104	104	108	100	92		92
34	481	Combination	100	96	100		100		82
34	482	Combination	96	92	72		71		55
34	483	PM	92	92	88		92		88
34	484	PM	120	124					
34	485	Combination	116	116	116		108		81
34	486	Combination	112	92	96		100		110

Centre Number	Patient Number	Treatment	Heart rate (Beats per minute) Baseline	Day 7	Day 14	Day 21	EOT	3Mon FU	6Mon FU
34	487	SSG	112	96	88	120	76		79
34	488	SSG	108	100	94	100	104	100	96
34	489	SSG	100	100	112	92	80		80
34	490	PM	120	100	124		108		90
34	491	PM	112	96	96		96		
34	492	PM	88	76	76		88		75
34	493	SSG	108	96	100	96	96	88	
34	494	Combination	98	92	88		88		70
34	495	SSG	142	100	120	108	104		78
34	496	SSG	104	82	100	100	96		79
34	497	PM	92	88	88		72	78	
34	498	SSG	112	124	116	92	92		99
34	499	Combination	112	108	106		111		88
34	500	PM	92	88	78		82		72
34	501	Combination	120	112	86		86		70
34	502	Combination	120	92	89		96		82
34	503	PM	112	104	105		94		
34	504	Combination	92	92	100		89		
34	505	SSG	128	108	89	92	83		72
34	506	SSG	108	92	109	96	96		82
34	507	SSG	120	116	112	104	100		80
34	508	PM	128	132	112		120		
34	509	PM	148	124	117		102		
34	510	Combination	108	108	108		105		88
34	511	Combination	100	104	102		92		92
34	512	PM	112	136	145		109		
34	513	Combination	120	104	112		108	100	96
34	514	SSG	128	108	115	104	89		91
34	515	SSG	112	96	92	92	83	80	
34	516	PM	76	88	80		76		70
34	517	PM	112	92	96		75		
34	518	SSG	128	120	112	96	97		120
34	519	PM	112	124	96		92	100	
34	520	SSG	114	96	104	88	85		78
34	521	PM	136	124	117		115		
34	522	Combination	120	120	106		106		
34	523	SSG	118	120	106	100	93		96
34	524	Combination	96	96	107		96		78
34	525	Combination	137	128	125		112		96
34	526	SSG	120	112	125	100	109		111
34	527	Combination	140	112	114		120		96
34	528	PM	120	120	112		89		
34	529	Combination	112	96	91		101		100
34	530	Combination	88	100	77		80		67
34	531	SSG	128	112	112	96	104		
34	532	Combination	120	120	115		115		100
34	533	SSG	96	88	87	88	82		72
34	534	PM	112	128	117		149		
34	535	PM	131	112	141		113	146	
34	536	PM	104	92	74		66		75
34	537	SSG	158	142	150	140	147		100

Centre	Patient	Treatment	Heart rate (Beats per minute)						
Number	Number		Baseline	Day 7	Day 14	Day 21	EOT	3Mon FU	6Mon FU
34	538	PM	157	132	130		121		
34	539	Combination	87	92	101		94		92
34	540	SSG	125	92	103	108	92		80
35	646	PM	104	84	92		94		72
35	647	PM	98	110	106		94	82	86
35	648	Combination	80	104	108		110	94	82
35	649	Combination	110	88	92		84	90	84
35	650	SSG	120	100	108	105	104	106	92
35	651	SSG	108	110	100	110	98	104	98
35	652	PM	112	104	100		106		114
35	653	Combination	90	106	100		90	88	104
35	654	SSG	115	100	112	118	120	110	96
35	655	Combination	115	118	104		112	92	88
35	656	SSG	118	92	100	94	112	74	80
35	657	PM	140	96	104		100	82	84
35	658	Combination	88	90	78		100	108	100
35	659	SSG	115	118	104	98	110	104	100
35	660	PM	76	78	92		90	78	70
35	661	Combination	116	90	92		98	74	78
35	662	PM	95	84	90		96	86	82
35	663	SSG	96	76	88	68	88	76	76
35	664	Combination	102	84					92
35	665	SSG	104	96	94	96	94	78	92
35	666	SSG	114	102	86	80	90	84	92
35	667	Combination	110	92	104		108	82	78
35	668	PM	94	102	80		80		78
35	669	PM	112	100	100		104		
35	670	SSG	112	92	90	94	98	80	74
35	671	PM	122	106	108		104		112
35	672	Combination	120	108	88		84		82
35	673	SSG	104	100	108	106	98	90	80
35	674	Combination	118	106	100		100	76	80
35	675	PM	100	118	96		108		116
35	676	PM	110	94	118		102	92	102
35	677	SSG	104	108					
35	678	Combination	128	104	88		96	82	74
35	679	SSG	120	85	84	80	88	74	86
35	680	Combination	104	112	108		100	84	78
35	681	Combination	112	88	76		90	84	78
35	682	PM	104	112	118		118		104
35	683	Combination	102	104	114		100		78
35	684	SSG	98	100	104	96	92	78	70
35	685	PM	96	108	98		88	78	84
35	686	PM	88	94	102		100	78	80
35	687	SSG	108	74	92	88	92	72	82
35	688	PM	128	112	106		86	88	72
35	689	Combination	128	90	96		88	76	82
35	690	SSG	120	100	108	104	108	92	88

Centre Number	Patient Number	Treatment	Axiliary Temperature (°C) Baseline	Day 7	Day 14	Day 21	EOT	3Mon FU	6Mon FU
11	1	PM	37	37	37		35.9	37.2	36.4
11	2	Combination	37	36.7	36.5		36.5	37	36.4
11	3	Combination	38	36.8	36.7		35.7	36.8	36.4
11	4	PM	37.4	37	36.6		36.8	36.7	36.9
11	5	PM	36.5	37.8	36.8		36.6	36	36.8
11	6	Combination	37.5	37.5	37.1		37.4	37.5	36.7
11	7	PM	36.9	37.2	37.3		37.1	36.7	36.3
11	8	SSG	37.5	37.2	35.9	37.3	36.4	37.2	37.1
11	9	SSG	37.2	36.5	37	36.6	35.1	36.4	36.7
11	10	SSG	37.2	36.2	37.3	36.1	35.9	36.5	36.7
11	11	Combination	36.9	36.8	37.6		36.9	36.9	37.1
11	12	Combination	36.9	37.8	36		36.6	36.9	36.1
11	13	SSG	36.5	36.7	37.1	37	36.1	36.9	37
11	14	SSG	36.5	36.4	36.7	36.5	36	36.7	36.5
11	15	PM	37.9	37.4				36.3	37.1
11	16	Combination	37.2	36.4	36.8		36.5	36.5	36.4
11	17	Combination	36.8	36.5	36.4		35.8	36.7	36.3
11	18	SSG	36.8	36.5	36.1	36.5	36.1	36.4	36.1
11	19	Combination	36.9	36.6	37.1		37.6	36.7	36.8
11	20	SSG	37.5	37.3	36.7	37	36.8	37.4	37
11	21	SSG	36.9	36.7	36.3	37	36.7	36	36
11	22	SSG	37.1	36.5	36	36	36.3	36.6	36.4
11	23	PM	37.1	36.6	36.3		37	36.8	36
11	24	PM	36.7	36.2	36.3		36	36.8	36.3
11	25	SSG	38.1	35.5	36.5	36.8	36.6	36.5	36.6
11	26	Combination	37.2	36.7	36.5		36.5	36.7	36.5
11	27	PM	37.4	37	35.6		36.4	38.6	
11	28	Combination	36.9	37	36.7		37.5	36.6	36.4
11	29	PM	37.7	36.8	36.8		36.7	36.4	36
11	30	PM	37	36.7	36		36.9	36.4	36.7
11	31	SSG	37.3	37.2	35.7	36.5	36.2	36.6	36.5
11	32	Combination	37.3	35.9	36.4		36.4	36.4	36
11	33	SSG	36.6	36.6	36.8	37	36.9	37.3	37
11	34	PM	37.4	36.7	36.4		36.6	36.5	36.3
11	35	SSG	36.7	36.5	37.2	36.9	36.6	37	36
11	36	SSG	38.4	35.9	36.6	37	37.4	36.8	36.6
11	37	Combination	38.3	36.1	36.1		36.5	36.6	36.3
11	38	PM	36.6	36	36.8		36.3	36.9	36
11	39	PM	36.6	36.2	37.1		36.8	36.9	36.3
11	40	PM	40	40	36.7			37.2	36.7
11	41	Combination	37.5	37.4	36.8		37.2	37.1	36.4
11	42	Combination	38.6	36.5	37.1		37.2	36.9	36.6
11	43	SSG	37.7	36.6	36.4	37.1	36.6	37	36.1
11	44	PM	37.8	36.6	37		36.5	36.4	37
11	45	Combination	37.6	36.8	37		36.9	36.7	37.1
11	46	SSG	37	36.8	36.2	36.4	36.6	36.7	36.2
11	47	SSG	39.3	36.2	36.3	36.9	36.4		
11	48	Combination	37.5	37.2	37.1		37.8		37
11	49	SSG	40.3	36.1	36.8	37.3	36.8	37	36.8
11	50	PM	38.2	38.5	37.5		36.2	36.4	36.4
11	51	Combination	39.3	37.2	37.4		37	36.8	36.5

Centre Number	Patient Number	Treatment	Axiliary Temperature (°C) Baseline	Day 7	Day 14	Day 21	EOT	3Mon FU	6Mon FU
11	52	PM	37.3	37	36.6		36.8	36.1	36
11	53	SSG	38.4	37	36.4	36.9	36.6	36.2	36.6
11	54	PM	37.8	37.3	36.8		36.8	36.7	36.4
11	55	PM	37.4	37.3	36		36.5	36.5	36.4
11	56	SSG	37.8	36.2	36.5	36.7	37	36.2	36.4
11	57	Combination	39.9	36	37.4		36.6	36.8	36.4
11	58	Combination	37	36.8	36.8		37	36.8	37
11	59	PM	38.9	38.3	37		37.3	36.1	36.4
11	60	Combination	40.2	36	36.1		36.6	36.1	36.4
11	61	PM	37.9	37.5	36.7		36.5	36.2	36.8
11	62	SSG	37.9	36.5	37.3	36	36	36.1	36.5
11	63	SSG	37.7	36.6	36.9	36.6	36.5	36.6	36.8
11	64	SSG	39.6	39	36	36.5	36	36.2	36
11	65	PM	37.5	36.3	36.6		36.5		
11	66	Combination	38.9	36.3	36.4		36	36.8	36.1
11	67	PM	39.5	36.8	36		36.5	36.8	36.1
11	68	SSG	39.6	36.6	36.2	36	36	36.4	36.8
11	69	Combination	38.8	36.8	36.5		36.2	37.1	36.9
11	70	Combination	38.3	36	36.2		36.2	37	36.1
11	71	Combination	36.6	36	36		36	35.9	36.4
11	72	SSG	38.1	37.6	39			36.4	36
11	73	PM	39.3	37	36.1		36.5	36.3	36.4
11	74	PM	39.5	36.4	36.5		36	36.8	36.4
11	75	Combination	38.2	36.9	36.9		36.5	36.4	36.7
11	76	PM	38.3	36.8	36.7		36.4	36.4	36.8
11	77	PM	38.9	36.2	36.9		36	36.6	37.1
11	78	SSG	40	37	36.9	36.7	36.5	36.7	36.6
11	79	PM	39.6	38	36.6		36.6	36.4	36
11	80	PM	39.9	36	36.1		36.3	36.2	36
11	81	Combination	38.4	36.3	36.4		36.2	36.7	36.4
11	82	SSG	39.6	36.3	36.8	36.8	37	37.1	37
11	83	SSG	39	36.7	36.1	36	36	37	37
11	84	SSG	36.8	36.9	37	37	36.8	36.2	37
11	85	SSG	38.2	37	36.6	36.7	36.8	37	
11	86	Combination	39.5	37	36.4		36.8	36.4	37.2
11	87	Combination	38.8	36.6	37		36.3	36.1	36.4
11	88	PM	39.2	36.8	36.5		37	36.5	36
11	89	Combination	38.6	36.4	36.8		37.1	36.6	36.6
11	90	Combination	38.8	36.8	36.8		36.7		
11	91	PM	38	36	36		36	37.2	36.1
11	92	Combination	37.5	36.6	36		36.4	37.2	36.8
11	93	SSG	37.1	36	36.3	36.2	36.4	36.6	36.4
11	94	SSG	38.6	36.4	36	36.2	36.6	37.1	36.9
11	95	SSG	38.1	36.1	36.3	36	36.4	36.5	36.8
11	96	Combination	39.8	36.8	36.1		36.8	37	36.5
11	97	Combination	39.6	37.2	36.4		37	36	36.3
11	98	PM	40.3	38.9	38.8		37.5	36.9	36
11	99	SSG	41	36.8	36	36	35.9	35.8	35.4
11	100	PM	38	36.2	36.5		36.9		
11	101	PM	38	37	36.3		36.5	36	36.2
11	102	Combination	40.9	36.8	36.4		36.2	36.3	36.1

Centre	Patient	Treatment	Axiliary Temperature (°C)						
Number	Number		Baseline	Day 7	Day 14	Day 21	EOT	3Mon FU	6Mon FU
11	103	Combination	38.8	36.6	36.8		37	36.2	36.5
11	104	PM	38.2	38.8	36.3		36.7	36.4	36.5
11	105	SSG	38.8	36.5	36.5	35.9	36.6	36.1	36.3
11	106	Combination	39.8	36.6	36.4		36.4	36	36
11	107	PM	39.8	36.2	37		36.8	37.2	36.3
11	108	PM	38.1	36.2	36.4		36.2	36.4	36.8
11	109	SSG	38	35.4	36.2	36	36	36.7	36.4
11	110	PM	39	36.8	36.3		36.1	36.3	36.3
11	111	Combination	38.8	36.5	37		36.8	37.1	36.6
11	112	Combination	36.8	38	36		36.2	36.1	35.1
11	113	SSG	37.8	36	36.8	36.8	36.5	36.4	
11	114	SSG	38.3	36.2	36.1	36.9	36.7	36.4	37.1
11	115	Combination	39	36.5	36		36.3	36.5	36.3
11	116	SSG	39.6	36.4	35.5	36	36	36.8	37
11	117	PM	38.2	36.1	36.4		36.4	36.2	
11	118	PM	37	36.9	37		36.8	37.1	36
11	119	SSG	38	35.8	36.5	36.7	37.1	36.4	36.6
11	120	Combination	39.4	36.1	36.5		36.6	36	36.3
11	121	Combination	39.1	36.4	36.4		36.6	36.2	36.9
11	122	SSG	38.8	35.7	36.4	36.9	36.6		
11	123	SSG	39.4	36	36.1	36.7	36.2	36.3	36.5
11	124	SSG	39.9	36.7	35.6	36.5	37		37
11	125	SSG	39.5	35.6	35.9	36.7	36.5	36.7	36.1
11	126	Combination	36	35.8	36.5		36.5		37
11	127	PM	39	38.3	37.1		36.5	36.7	36.2
11	128	Combination	39.9	37.8	37.6		37.9	36.9	36.8
11	129	PM	39.2	40.1	36.4		36.5	36.6	36.5
11	130	Combination	38.8	36.8	37.1		36.5	36.8	36.3
11	131	PM	40.3	38.8	36.6		37	36.7	36.7
11	132	SSG	39.4	36	36.1	36.6	36.8	36.2	36.9
11	133	Combination	39.2	36.5	36.5		36.8	36.4	36
11	134	PM	37.9	37.5	37.4		36.6	36.6	36.5
11	135	PM	37.8	36.3	36.2		37.8	36.7	36.2
12	241	Combination	37	36	37.3		36.6		37
12	242	SSG	36.8	36.4	36.8	36.5	36.8	36.8	37.3
12	243	PM	38	36.5	37		36.3	36.8	36.2
12	244	SSG	36.8	37	36.8	36.6	36.7	36.4	36
12	245	PM	37	36.1	36		36.2	37.2	
12	246	PM	36	36	36		36.2	36.8	
12	247	PM	37.8	36.5	36.8		38	36.8	36.6
12	248	PM	36.5	36	36.5		36.5	37	37
12	249	Combination	36	36.5	37		37	37.4	36.5
12	250	SSG	36.2	37	36.6	37.2	36.5	36.5	37
12	251	Combination	36.9	36.8	36.8		37.5	36.5	36.7
12	252	SSG	37.8	36.2	37	37	36	36	36.7
12	253	SSG	37.2	37	37	36.5	36.8	36.2	36
12	254	Combination	37	37	37.2		37.1	36.8	36.5
12	255	Combination	37	37.8	37.6		36.8	36.2	36.8
12	256	Combination	36.8	36.5	36		36	36	36.5
12	257	SSG	37.4	36.3	37	36.5	36.8	36.8	36.8
12	258	SSG	36.5	37.2	36	36	36		36

Centre Number	Patient Number	Treatment	Axiliary Temperature (°C)					
			Baseline	Day 7	Day 14	Day 21	EOT	3Mon FU	6Mon FU
12	259	PM	37	37	36.2		36	37	36
12	260	PM	37	36	36.4		36.2		36
12	261	Combination	38	36	36.4		36.8	36.2	36.8
12	262	PM	36.4	36	36		36	36.8	37.1
12	263	SSG	36.6	36.8	37.3	37.1	37.4	37	36.5
12	264	Combination	37	36.3	36.8		36.2	37	36.6
12	265	SSG	37.1	37	36.6	37	36.5		
12	266	PM	37.1	36.3	36.5		36	36.8	36.6
12	267	SSG	36.4	36	36.4	36.4	37	36.5	36.8
12	268	PM	37	37.3	36.8		37	36.2	36.8
12	269	Combination	38	36.5	36		36.7	36.4	36.2
12	270	Combination	36.4	37.2	36.5		36		36.5
12	271	SSG	37.3	37	36.6	39.8	36	36	36
12	272	PM	39	36.2	36.3		36.4	36.8	36.8
12	273	SSG	36.1	36.5	36.1	37	36	36.6	36.6
12	274	SSG	36.8	37.1	37.1	37	36	36.2	36.4
12	275	Combination	36.6	36.6	36.2		36.1	36.5	36.2
12	276	PM	36.2	36.6	36.5		36	37.1	36.8
12	277	Combination	38.4	37	37		36.8	36	36.2
12	278	SSG	36.6	37	36.4	36.5	37.1	36.4	
12	279	PM	36	37	36.8		36.6	36.7	35.8
12	280	SSG	36.4	37.1	37.1	37.1	36.5	36.8	37.4
12	281	Combination	37.4	37.3	36.5		36.5	36.8	36.4
12	282	PM	36.4	37	36.7		37.2	37	36.2
12	283	PM	38	36	36.7		36.1	36.2	36.5
12	284	Combination	37.1	37.5	37.4		36.5	36.6	36.5
12	285	Combination	37	37.2	36.8		37	36.9	37.1
12	286	PM	37	35.5	36.5		36.4	36.7	36.8
12	287	SSG	37.6	36.6	36	37	37	37	36.6
12	288	Combination	37.1	35	37.3		37.6	36.5	36.7
12	289	Combination	37	37	36.5		36.8	36.4	37
12	290	SSG	37.2	37.4	36.8	37	36.8		37
12	291	SSG	37.1	37	36	36.8	37.1	36.6	36.5
12	292	PM	37.8	37	38		36		37
12	293	SSG	37.4	37.2	37.3	37	36.7		36.5
12	294	Combination	37.4	37.2	37.2		37	37	36.8
12	295	Combination	38	36.4	37		37		37
12	296	PM	37	37.3	36.5		37	37	36.5
12	297	SSG	37	36.6	36.2				
12	298	PM	38.4	37.3	37.3		37	37	36.8
12	299	Combination	37	35.8	37		36		35.8
12	300	PM	37.2	37.1	36.3		36.5	36.6	36.8
12	301	SSG	37	36.8	36.3	36	36.7	36.2	36.9
12	302	SSG	37.3	37	38	37	36.8	37	36.3
12	303	PM	38.8	36.6	36		36	36.5	36.3
12	304	Combination	37.1	37	36.2		36.8	36.6	36.2
12	305	PM	37.5	36.5	37		36.2	36.6	36
12	306	Combination	36	36.5	36		36.2	37.2	36.6
12	307	Combination	38.8	36.8	36.2		36.8	36.8	36.2
12	308	PM	37	36.5	37		36.5	37.2	36.5
12	309	PM	38.2	36	36.6		36.8	36.5	36.5

Centre Number	Patient Number	Treatment	Axiliary Temperature (°C) Baseline	Day 7	Day 14	Day 21	EOT	3Mon FU	6Mon FU
12	310	SSG	36.5	36.7	36.8	37	37.3	37	35.5
12	311	SSG	36.9	37	36.4	36.1	37	36.8	37.2
12	312	Combination	37.6	36.5	36.5		37	35.8	36.5
12	313	PM	36.2	36.3	36.5		36.5	36.5	36.3
12	314	SSG	37	36	36.8	36.1	36.5	36.3	36.5
12	315	Combination	37.3	36.8	37.2		36.8	37	37.7
12	316	SSG	36.7	36.5	36.6		36.5	36.5	36.7
12	317	SSG	36.8	36.7	37	36	36.8		36.6
12	318	Combination	38.2	37	36.3		36.2	36.4	36.9
12	319	Combination	38.2	36.2	37		36.4	37	36.2
12	320	Combination	36.5	36.4	36.5		36.8	36.4	36.3
12	321	Combination	36.8	37	37.3		36.8	36.5	36.2
12	322	PM	37	36.5	36		36	36.5	36.8
12	323	SSG	37.2	36.1	36	36.8	36.4	36.5	36.6
12	324	SSG	37	36.7	36.5	36.6	36.5	36.4	36.5
12	325	PM	36.8	36	36.2		36.6	36.8	36.5
12	326	PM	38	38.5	35.4		36	36.9	36.3
12	327	Combination	39.4	36.6	36.6		36.8	36.8	36.7
12	328	PM	40	36.5	37		35.6	37	37
12	329	SSG	38.3	37	37.8	36.7	37	37.3	37
12	330	PM	38.2	36.4	36.3		36.3	36.8	37.6
23	361	Combination	37.6	36.4	36.4		36	37	36.6
23	362	PM	36.8	36.8	36		36	36.4	36.8
23	363	PM	38	37	36.6		36	36.4	36.7
23	364	Combination	37.3	36	36.4		36.4	37.6	36.4
23	365	SSG	36	36	36.2	36.6	36.4	36.6	36.4
23	366	Combination	38.2	36.4	36.4		36.4	36.2	36.6
23	367	SSG	37.4	36.2	36.2	36	36.2	36.4	36.5
23	368	PM	37	36.2	36.4		36.8	36.8	36.4
23	369	Combination	36	36.2	36.2		36.3	36.2	36.8
23	370	SSG	37.2	36.2	36.7	37	36.2	36.6	36.5
23	371	PM	37.8	36	36.4		36.4	36.8	36
23	372	PM	37	37	36.4		36	36.8	36.4
23	373	SSG	36.6	36.6	36.8	36.2	36	36.6	36.6
23	374	SSG	39	36.2	36.6	36.4	36.4	36.8	36.8
23	375	Combination	38	36.2	36.2		36	36	36
23	376	SSG	36.6	38.4	36.2	36.2	36.2	36.4	35.6
23	377	Combination	36.8	36.6					36.4
23	378	Combination	38	36.6	37		36.8	37	37.4
23	379	PM	37.6	36.2	36.2		36.4	36.5	36.4
23	380	SSG	37	36	36.4	36.4	36.8	36.2	36.5
23	381	PM	36.4	36.7	36.4		36.4	37	36.5
23	382	Combination	37	36.4	36.6		36.4	36	36
23	383	PM	36.6	36.6	36.2		36.4	36.6	36.8
23	384	SSG	36.8	36	36	36.4	36.6	36.6	36.5
23	385	SSG	36.8	36.4	36.2	36.6	36	36.8	36.4
23	386	Combination	39	36.6	36.4		37	36.6	36.6
23	387	Combination	36.4	37	36.8		36.4	36.8	36.8
23	388	PM	35.8	36.6	36.4		36.4	36.8	36.8
23	389	SSG	37	37.2	36.8	36.6	36.6	36	36.4
23	390	PM	37.4	36.4	36.4		36	36.8	36.8

Centre Number	Patient Number	Treatment	Axiliary Temperature (°C)						
			Baseline	Day 7	Day 14	Day 21	EOT	3Mon FU	6Mon FU
23	391	Combination	37	37	36.2		37.2	36.4	36.4
23	392	Combination	37	36.6	36.4			36.4	36.8
23	393	SSG	36.2	36.4	36.4	36.6	36.2	36.4	37.2
23	394	PM	39.4	36.4	36.4		36.2	36.4	36.4
23	395	PM	37.8	36	36.4		36	36.4	36.6
23	396	SSG	37.8	36.6	36.6	36.4	37	36.2	36.2
23	397	SSG	38	36.2	36.8	36.8	36.8	36.6	36.6
23	398	Combination	36.8	36.2	36.8		37	36.4	36
23	399	PM	37.8	36.6	36.8		36.4	35.6	36.3
23	400	PM	38.6	36.8	36.6		36.6	36.6	36.8
23	401	Combination	36.7	36.6	37		36.4	36.6	37.2
23	402	Combination	37.5	36.6	36.4		37	36.6	36.6
23	403	SSG	37	36.3	37.2	36.6	36.4	36.2	37
23	404	SSG	36.8	36.2	36.2	36.4	36.2		36.4
23	405	PM	36	36.4	36.4		36	36.4	36.2
34	451	Combination	39.4	36.8	37.1		36.6		37.1
34	452	PM	39.4	38.4	35.7		36.4		
34	453	PM	39.2	38.4	36.7		36.6		
34	454	Combination	39.9	36.6	36.8		36.2		36.8
34	455	PM	39.3	39.7	39.2		36.1		
34	456	SSG	39.9	37.8	36.9	36.1	36.8	37.1	
34	457	Combination	39.9	36.7	36.8		36.7		
34	458	PM	39.8	39.1	37.5		37.5	38.1	
34	459	Combination	38.6	35.8	36.4		37	36.5	
34	460	PM	38.1	36.2	36.1		36.4		
34	461	SSG	37.2	37.9	35				
34	462	SSG	39.3	36.3	34.8	36.8	37.2		37.3
34	463	Combination	39.6	36.2	36.1		36.8		36.8
34	464	SSG	38.3	37.8	35.9	36.7	34.1		36
34	465	SSG	39.4	36.3	36.6	36.8	37.3		37
34	466	Combination	36.6	36.7	36.7		35.8		
34	467	PM	38.2	36.1	37.7		36.4	39.3	
34	468	Combination	38.1	36.4	36.6		36.9		36.7
34	469	SSG	39.7	37.7	38.5	37.1	36.9		36.5
34	470	Combination	40.1	37.1	37		36.6		36.5
34	471	PM	38.4	38.4	37.8		37.2		
34	472	SSG	37.1	36.4	36.8	36.8	36.7		36.5
34	473	PM	40.2	36.8	37		36.7		
34	474	Combination	37.8	36.8	36.2		36.1		37
34	475	PM	38.7	38.1	36.9		37.8		
34	476	PM	38.1	38.2	37.3		37.6	38	
34	477	SSG	39.1	36.5	36.6	38.8	36.7		37.5
34	478	Combination	38.6	36.2	36.9		37		36.9
34	479	SSG	38.9	35.5	36.9	36.8	36.8		36.7
34	480	SSG	36.5	36.4	37	36.7	36.6		36.7
34	481	Combination	37.5	36.4	37.1		37		37.2
34	482	Combination	37.9	35.8	36.2		35		36.1
34	483	PM	38.6	37.4	36.2		36.9		37.9
34	484	PM	36.7	39.4					
34	485	Combination	39.6	37.7	37.4		37.3		36.1
34	486	Combination	38.5	36.6	37.1		36.4		36.4

Centre Number	Patient Number	Treatment	Axiliary Temperature (°C) Baseline	Day 7	Day 14	Day 21	EOT	3Mon FU	6Mon FU
34	487	SSG	39.4	36.3	36.3	38.8	36.7		37.1
34	488	SSG	37.7	36.9	37	36.8	36.7	37.1	37.1
34	489	SSG	37.6	36.1	36.7	36.6	37		36.7
34	490	PM	36.7	35.9	37.8		36.9		36.5
34	491	PM	38.1	38.1	36.6		36.8		
34	492	PM	36.6	36.9	36.7		36.9		36.9
34	493	SSG	38.8	36.2	37.3	36.3	36.6	37.4	
34	494	Combination	38.1	36	36		36.6		36
34	495	SSG	40.4	36.4	37.1	36.2	36.2		37.2
34	496	SSG	39.8	36.2	36.2	36.7	36.7		36.9
34	497	PM	39.5	36.8	36.5		36.1	35.9	
34	498	SSG	38.1	37.1	36.8	36.5	36.9		36.5
34	499	Combination	38.8	36.7	36.7		36.4		37
34	500	PM	38.2	36.5	36.7		36.7		36.9
34	501	Combination	40.6	35.9	36.8		37		36.9
34	502	Combination	39.6	36.2	36.5		36.8		36.7
34	503	PM	40	37.5	37.5		37		
34	504	Combination	36.5	36.6	37.2		36.8		
34	505	SSG	39.3	36.9	36.9	36.9	36.7		36.7
34	506	SSG	38.1	35.8	37	36.4	36.7		36.7
34	507	SSG	39.8	34.7	36	37	36.9		37.3
34	508	PM	39.3	39.4	36		36.7		
34	509	PM	40.2	36.6	36.6		35.5		
34	510	Combination	39.9	36.8	37.2		37.3		36.7
34	511	Combination	36.5	37.3	36.8		36.6		36.8
34	512	PM	37.3	39.3	38		37.7		
34	513	Combination	38.4	37	36.6		36.4	36.5	37.2
34	514	SSG	38.7	37	36.5	36.9	36.6		36.7
34	515	SSG	38.4	36.5	36.7	36.7	36.7	36.2	
34	516	PM	34.9	36.7	36.9		35.6		36.5
34	517	PM	37.3	38.6	37.2		35.5		
34	518	SSG	38.2	36.8	38	36.7	37		37.3
34	519	PM	38.4	39.4	37.5		36.9	39.4	
34	520	SSG	40.2	36.9	36.9	36.3	36.7		37.1
34	521	PM	38.9	37.1	36.7		37.1		
34	522	Combination	37.1	36.1	35.9		36.7		
34	523	SSG	37.7	37.2	36.9	36.8	37		36.8
34	524	Combination	37.8	37	37		36.7		36.7
34	525	Combination	37.4	37.1	36.8		37.3		36.8
34	526	SSG	39.7	36.6	37	37	36.4		36.9
34	527	Combination	38.2	36.3	36.7		36.9		37.5
34	528	PM	37.9	36.5	36.9		36.5		
34	529	Combination	38.1	36.6	37.1		37.2		37.2
34	530	Combination	37.7	36.6	36.6		36.2		35.8
34	531	SSG	38.6	37.6	37.4	36.7	36.9		
34	532	Combination	38.8	37.2	36.8		37.2		37.5
34	533	SSG	37.9	36.8	36.7	36.1	36.1		36.2
34	534	PM	39.8	39.2	36		37.2		
34	535	PM	37.8	36.7	38		36.8	38	
34	536	PM	39.8	36.7	36.9		36.9		36.7
34	537	SSG	39.4	37.2	36.9	37.4	37.2		37.6

Centre	Patient	Treatment			Axiliary Temperature (°C)				
Number	Number		Baseline	Day 7	Day 14	Day 21	EOT	3Mon FU	6Mon FU
34	538	PM	40.3	38.5	37.2		36.9		
34	539	Combination	37	37.2	36.8		36.6		36.5
34	540	SSG	39.1	36.9	36.5	36.8	37.2		36.7
35	646	PM	39	36.5	36		36.2		36.2
35	647	PM	39.1	37.6	36.4		36	35.6	35.6
35	648	Combination	36.8	36	36		37	36.1	36.4
35	649	Combination	38.5	35.5	35.6		36	35.7	37
35	650	SSG	36.7	35.7	35.8	36	36.2	36	36.5
35	651	SSG	37.4	36	35.5	36.2	36.3	36.2	37
35	652	PM	38.8	37.5	36.1		36.5		37.2
35	653	Combination	38.8	37.2	36		36.1	36.2	36.6
35	654	SSG	36	36.1	36	35.7	35.6	36.5	35.2
35	655	Combination	39	35.7	35.8		35.9	36	36.2
35	656	SSG	37	36.7	36.4	35.8	36.5	36	35.5
35	657	PM	38	36.1	36.2		36	35.9	36.5
35	658	Combination	39.2	35.6	35.7		36	35.7	36.7
35	659	SSG	37.5	37.7	37.5	35.5	36.9	36.4	36.8
35	660	PM	36.4	36.5	36		35.8	35	36.5
35	661	Combination	38.5	35.5	36		36.7	35.5	36.4
35	662	PM	38	38.5	35.5		35.8	35.5	37.5
35	663	SSG	38.5	35.4	36	36.5	36.5	36.4	36.8
35	664	Combination	39	35.2					35.2
35	665	SSG	39.4	36.5	36.4	36.7	36.4	36	35.7
35	666	SSG	37.6	35.6	36.1	36	35.9	37	36
35	667	Combination	39	36.3	36.4		36.1	36.5	36.5
35	668	PM	39	36	35.9		36		35.6
35	669	PM	39	36.4	36.8		37.4		
35	670	SSG	38.7	36.4	36.2	35.5	36.6	35.4	36
35	671	PM	38.8	36.5	36.2		36.3		35.6
35	672	Combination	38.3	36	36.2		36.1		35.7
35	673	SSG	37.8	36.2	36.1	36.5	36.3	35.6	36.5
35	674	Combination	38.4	38.3	36.3		36.6	37	36
35	675	PM	38.2	38.4	35.5		36.7		36.5
35	676	PM	38.6	38.6	36.6		36.8	36.2	36.6
35	677	SSG	37.9	37.4					
35	678	Combination	38.1	36.5	35.3		35.8	36.6	37
35	679	SSG	38.5	36.4	35	35.8	36	37	36.7
35	680	Combination	36.3	36.1	35.3		36.1	36.5	36
35	681	Combination	39.9	35.5	36.1		36	36.1	35.8
35	682	PM	38.5	37.1	36		36.5		37.2
35	683	Combination	38.1	35.8	35.6		35.9		36.4
35	684	SSG	38.5	37.6	36.6	35.8	35.9	36.5	36.3
35	685	PM	36.1	37.2	36		36	36.4	35.7
35	686	PM	37.1	36.1	36		36	35.8	36.2
35	687	SSG	38.6	36.1	36.8	37.3	35.7	35.8	36
35	688	PM	37.2	36.2	36.5		36.2	36	36.6
35	689	Combination	38.4	36	36		37	37.1	36
35	690	SSG	38.3	36.7	36	36.5	37.3	36.4	36.1

Centre Number	Patient Number	Treatment	Systolic Blood Pressure (mmHg)						
			Baseline	Day 7	Day 14	Day 21	EOT	3Mon FU	6Mon FU
11	1	PM	100	100	100		100	100	110
11	2	Combination	100	100	100		100	120	110
11	3	Combination	100	100	100		110	110	120
11	4	PM	110	110	100		120	90	120
11	5	PM	100	120	120		125	100	120
11	6	Combination	100	100	110		100	100	100
11	7	PM	100	110	100		100	90	120
11	8	SSG	100	100	100	110	80	100	100
11	9	SSG	120	100	110	100	110	100	110
11	10	SSG	100	90	100	90	90	100	100
11	11	Combination	110	100	100		105	105	100
11	12	Combination	120	120	100		100	100	100
11	13	SSG	100	105	80	80	80	100	90
11	14	SSG	100	95	100	100	100	100	90
11	15	PM	120	110				100	110
11	16	Combination	100	90	90		90	110	100
11	17	Combination	90	80	90		110	100	100
11	18	SSG	100	100	100	100	100	90	90
11	19	Combination	100	100	110		100	100	110
11	20	SSG	80	90	90	90	90	110	90
11	21	SSG	120	110	120	90	120	120	100
11	22	SSG	100	100	90	100	90	100	80
11	23	PM	90	80	90		90	100	100
11	24	PM	90	100	90		100	100	100
11	25	SSG	100	80	80	90	90	100	100
11	26	Combination	110	100	110		100	100	100
11	27	PM	100	100	100		100	80	
11	28	Combination	110	90	90		90	90	100
11	29	PM	110	90	90		110	100	90
11	30	PM	110	90	80		100	100	100
11	31	SSG	100	100	80	90	100	100	80
11	32	Combination	100	90	100		90	110	100
11	33	SSG	110	120	120	110	120	120	120
11	34	PM	110	110	100		100	110	110
11	35	SSG	100	110	90	110	110	100	90
11	36	SSG	100	110	100	100	110	110	120
11	37	Combination	110	110	120		100	140	140
11	38	PM	120	100	110		100	90	80
11	39	PM	100	100	110		110	90	100
11	40	PM	100	100	110			110	100
11	41	Combination	100	100	110		100	100	100
11	42	Combination	110	100	120		120	100	110
11	43	SSG	100	100	100	100	100	100	100
11	44	PM	100	100	100		100	100	90
11	45	Combination	100	110	100		100	100	90
11	46	SSG	100	100	90	100	100	110	90
11	47	SSG	100	80	90	90	100		
11	48	Combination	100	90	80		90		100
11	49	SSG	100	80	90	90	90	90	100
11	50	PM	100	100	100		100	100	100
11	51	Combination	110	90	120		100	100	100
11	52	PM	100	90	100		110	90	100
11	53	SSG	100	90	100	100	110	100	100
11	54	PM	80	90	80		90	90	100

Centre Number	Patient Number	Treatment	Systolic Blood Pressure (mmHg) Baseline	Day 7	Day 14	Day 21	EOT	3Mon FU	6Mon FU
11	55	PM	90	100	90		90	100	110
11	56	SSG	110	90	100	100	100	100	90
11	57	Combination	100	80	90		110	100	110
11	58	Combination	100	110	100		100	90	100
11	59	PM	110	100	110		140	140	110
11	60	Combination	100	90	100		90	100	100
11	61	PM	100	100	90		100	100	110
11	62	SSG	100	90	110	100	110	100	110
11	63	SSG	100	100	100	90	100	110	120
11	64	SSG	80	80	90	80	80	100	100
11	65	PM	90	100	100		90		
11	66	Combination	100	100	80		80	80	90
11	67	PM	110	100	90		100	110	100
11	68	SSG	100	90	90	100	100	90	80
11	69	Combination	90	80	100		100	110	110
11	70	Combination	110	90	100		110	120	100
11	71	Combination	80	90	90		90	100	90
11	72	SSG	110	100	110			110	120
11	73	PM	90	100	100		110	100	120
11	74	PM	90	90	100		100	100	100
11	75	Combination	100	100	110		120	110	110
11	76	PM	80	90	110		90	110	100
11	77	PM	100	80	80		100	100	100
11	78	SSG	80	100	100	110	100	100	100
11	79	PM	100	100	90		80	90	100
11	80	PM	100	100	100		90	90	90
11	81	Combination	100	100	100		110	110	90
11	82	SSG	90	100	110	100	100	100	100
11	83	SSG	90	100	90	100	100	90	110
11	84	SSG	80	90	90	100	100	100	100
11	85	SSG	90	90	90	100	100	90	
11	86	Combination	70	80	80		90	90	100
11	87	Combination	80	80	80		100	90	120
11	88	PM	110	100	90		100	110	100
11	89	Combination	110	110	100		100	120	120
11	90	Combination	90	70	90		80		
11	91	PM	100	100	90		100	100	100
11	92	Combination	80	90	90		100	90	90
11	93	SSG	90	80	100	100	110	90	120
11	94	SSG	100	80	90	100	100	100	100
11	95	SSG	90	100	90	90	100	80	80
11	96	Combination	110	90	100		110	110	100
11	97	Combination	90	80	110		100	110	100
11	98	PM	90	100	80		90	90	80
11	99	SSG	90	80	100	90	90	80	80
11	100	PM	80	80	90		100		
11	101	PM	100	100	120		110	100	100
11	102	Combination	90	80	90		100	100	80
11	103	Combination	100	100	100		120	110	100
11	104	PM	110	120	100		110	100	100
11	105	SSG	100	100	100	100	100	90	100
11	106	Combination	100	100	110		90	120	110
11	107	PM	100	110	100		100	90	100
11	108	PM	100	100	80		100	100	100

Centre Number	Patient Number	Treatment	Systolic Blood Pressure (mmHg) Baseline	Day 7	Day 14	Day 21	EOT	3Mon FU	6Mon FU
11	109	SSG	100	110	110	90	100	120	100
11	110	PM	90	70	90		80	80	80
11	111	Combination	80	100	80		100	110	110
11	112	Combination	80	90	80		110	100	80
11	113	SSG	90	90	90	100	100	100	
11	114	SSG	110	100	110	100	120	110	100
11	115	Combination	100	100	110		110	130	110
11	116	SSG	100	90	100	100	100	100	110
11	117	PM	120	120	100		110	120	
11	118	PM	100	100	110		120	100	110
11	119	SSG	100	100	110	100	100	110	100
11	120	Combination	120	100	120		110	100	110
11	121	Combination	100	90	90		100	80	90
11	122	SSG	90	70	90	90	80		
11	123	SSG	90	90	100	100	110	100	100
11	124	SSG	100	100	100	100	90		100
11	125	SSG	100	90	90	100	120	110	110
11	126	Combination	90	90	90		100		90
11	127	PM	100	80	100		100	100	100
11	128	Combination	100	90	90		80	100	100
11	129	PM	100	100	90		100	100	110
11	130	Combination	90	80	90		100	100	110
11	131	PM	90	90	80		90	90	100
11	132	SSG	90	100	110	90	100	100	100
11	133	Combination	100	100	100		100	110	100
11	134	PM	100	100	100		110	110	100
11	135	PM	100	90	100		100	100	100
12	241	Combination	100	100	110		100		120
12	242	SSG	90	90	90	80	90	90	90
12	243	PM	90	80	90		90	100	90
12	244	SSG	100	100	100	100	120	120	120
12	245	PM	100	90	90		110	100	
12	246	PM	100	110	110		120	120	
12	247	PM	100	90	100		100	100	90
12	248	PM	110	90	100		100	120	110
12	249	Combination	90	90	80		90	100	90
12	250	SSG	90	90	90	90	100	110	100
12	251	Combination	120	110	100		100	100	90
12	252	SSG	100	100	100	110	110	110	110
12	253	SSG	100	90	100	90	100	100	100
12	254	Combination	80	80	80		80	80	90
12	255	Combination	110	100	100		120	110	100
12	256	Combination	80	90	100		100	110	110
12	257	SSG	90	100	100	100	100	100	100
12	258	SSG	80	90	90	90	100		100
12	259	PM	120	100	100		100	110	110
12	260	PM	120	100	100		110		110
12	261	Combination	100	100	100		90	110	110
12	262	PM	100	100	90		90	90	80
12	263	SSG	100	90	100	100	100	100	100
12	264	Combination	90	100	100		100	90	90
12	265	SSG	80	90	80	90	70		
12	266	PM	100	100	105		120	110	100
12	267	SSG	90	90	90	90	90	110	100

Centre Number	Patient Number	Treatment	Systolic Blood Pressure (mmHg)						
			Baseline	Day 7	Day 14	Day 21	EOT	3Mon FU	6Mon FU
12	268	PM	100	100	110		110	100	110
12	269	Combination	90	100	100		110	120	110
12	270	Combination	100	90	80		80		90
12	271	SSG	100	105	110	110	90	110	110
12	272	PM	90	80	90		90	90	80
12	273	SSG	90	80	80	90	90	90	100
12	274	SSG	90	80	80	100	90	90	90
12	275	Combination	90	100	90		90	90	100
12	276	PM	90	90	90		100	90	90
12	277	Combination	100	100	100		100	110	110
12	278	SSG	90	90	100	100	100	110	
12	279	PM	80	80	90		90	110	100
12	280	SSG	70	80	80	90	90	80	90
12	281	Combination	100	100	100		100	100	100
12	282	PM	70	90	90		90	90	90
12	283	PM	110	90	100		100	100	110
12	284	Combination	70	70	80		80	80	80
12	285	Combination	90	90	100		100	100	100
12	286	PM	100	90	100		90	90	90
12	287	SSG	110	100	100	100	90	100	100
12	288	Combination	100	100	100		90	90	90
12	289	Combination	120	110	120		120	100	130
12	290	SSG	100	90	80	90	90		90
12	291	SSG	100	90	110	100	100	100	100
12	292	PM	80	70	80		90		120
12	293	SSG	90	80	100	100	100		90
12	294	Combination	80	80	90		90	90	90
12	295	Combination	80	80	100		80		90
12	296	PM	100	90	90		90	90	100
12	297	SSG	90	100	90				
12	298	PM	100	90	100		90	100	110
12	299	Combination	90	80	90		100		90
12	300	PM	100	100	100		100	110	100
12	301	SSG	100	110	100	100	100	110	110
12	302	SSG	80	80	70	80	80	90	80
12	303	PM	120	110	100		110	120	130
12	304	Combination	90	100	100		80	100	100
12	305	PM	70	70	70		60	80	70
12	306	Combination	80	80	80		80	80	80
12	307	Combination	100	100	110		110	100	120
12	308	PM	120	90	90		90	120	110
12	309	PM	100	90	100		100	100	110
12	310	SSG	100	90	90	90	90	90	100
12	311	SSG	70	80	80	90	80	90	80
12	312	Combination	100	90	90		90	100	110
12	313	PM	100	100	90		100	100	100
12	314	SSG	90	100	90	100	90	120	110
12	315	Combination	80	90	100		100	90	100
12	316	SSG	110	120	100		100	100	90
12	317	SSG	60	80	90	90	90		70
12	318	Combination	100	80	100		80	100	80
12	319	Combination	100	90	90		100	100	80
12	320	Combination	80	80	80		80	100	80
12	321	Combination	90	90	100		90	90	80

Centre Number	Patient Number	Treatment	Systolic Blood Pressure (mmHg) Baseline	Day 7	Day 14	Day 21	EOT	3Mon FU	6Mon FU
12	322	PM	100	90	90		90	100	100
12	323	SSG	110	100	100	100	110	110	110
12	324	SSG	100	100	100	110	100	120	120
12	325	PM	110	90	100		120	100	100
12	326	PM	90	100	100		100	120	120
12	327	Combination	100	100	100		90	90	90
12	328	PM	100	100	100		100	100	100
12	329	SSG	90	90	90	100	90	90	80
12	330	PM	100	90	100		100	100	60
23	361	Combination	90	100	100		90	100	100
23	362	PM	110	110	100		120	110	100
23	363	PM	100	100	100		90	100	100
23	364	Combination	110	110	90		110	100	100
23	365	SSG	110	100	120	110	110	110	100
23	366	Combination	90	100	100		100	100	100
23	367	SSG	90	120	110	110	120	110	100
23	368	PM	100	100	100		100	120	100
23	369	Combination	90	100	100		100	80	90
23	370	SSG	90	90	100	100	90	90	80
23	371	PM	100	100	110		110	100	90
23	372	PM	110	120	110		120	100	120
23	373	SSG	110	110	100	100	110	110	100
23	374	SSG	110	90	100	100	100	110	100
23	375	Combination	100	100	100		100	100	100
23	376	SSG	110	100	100	100	100	100	90
23	377	Combination	100	110					100
23	378	Combination	90	90	100		90	90	100
23	379	PM	100	90	90		100	90	100
23	380	SSG	90	90	100	90	90	90	90
23	381	PM	90	100	100		100	100	100
23	382	Combination	100	100	100		100	90	100
23	383	PM	110	110	100		110	110	100
23	384	SSG	100	100	100	100	100	100	100
23	385	SSG	120	100	100	100	100	90	100
23	386	Combination	110	110	100		90	100	110
23	387	Combination	100	90	100		100	100	100
23	388	PM	90	90	100		110	100	90
23	389	SSG	100	100	100	100	100	90	110
23	390	PM	100	110	110		110	110	110
23	391	Combination	90	100	100		100	110	100
23	392	Combination	90	90	100			90	100
23	393	SSG	100	100	100	90	100	100	100
23	394	PM	100	100	100		100	100	100
23	395	PM	120	110	110		110	105	120
23	396	SSG	110	100	110	110	100	110	100
23	397	SSG	100	100	110	90	110	100	100
23	398	Combination	90	90	90		90	90	90
23	399	PM	90	90	90		100	100	100
23	400	PM	100	90	90		90	100	90
23	401	Combination	100	110	110		110	110	110
23	402	Combination	100	110	100		110	100	100
23	403	SSG	100	100	110	110	100	110	120
23	404	SSG	100	100	110	110	110		110
23	405	PM	100	100	100		100	100	100

Centre Number	Patient Number	Treatment	Systolic Blood Pressure (mmHg) Baseline	Day 7	Day 14	Day 21	EOT	3Mon FU	6Mon FU
34	451	Combination	100	100	110		110		110
34	452	PM	100	100	120		120		
34	453	PM	105	110	110		110		
34	454	Combination	90	90	90		90		90
34	455	PM	110	120	110		90		
34	456	SSG	95	95	95	110	120	90	
34	457	Combination	120	120	120		120		
34	458	PM	100	110	110		110	110	
34	459	Combination	100	110	110		100	100	100
34	460	PM	110	120	110		110		
34	461	SSG	100	130	130				
34	462	SSG	90	90	85	90	90		90
34	463	Combination	90	90	90		90		90
34	464	SSG	120	120	120	120	120		120
34	465	SSG	110	120	120	120	120		120
34	466	Combination	90	90	90		90		
34	467	PM	90	100	110		120	100	
34	468	Combination	120	120	120		120		110
34	469	SSG	110	100	110	100	110		110
34	470	Combination	105	120	120		120		100
34	471	PM	120	120	110		120		
34	472	SSG	100	100	100	100	80		90
34	473	PM	95	90	80		80		
34	474	Combination	100	100	100		110		100
34	475	PM	110	100	100		110		
34	476	PM	110	110	110		120	100	
34	477	SSG	110	100	100	110	100		110
34	478	Combination	90	90	100		110		80
34	479	SSG	90	90	100	90	80		80
34	480	SSG	100	100	110	110	110		110
34	481	Combination	100	100	110		120		110
34	482	Combination	120	120	120		120		110
34	483	PM	120	120	110		120		120
34	484	PM	90	90					
34	485	Combination	100	80	110		100		90
34	486	Combination	110	110	120		110		110
34	487	SSG	110	120	120	120	120		110
34	488	SSG	110	100	95	90	90	90	100
34	489	SSG	110	100	80	80	90		110
34	490	PM	100	100	85		90		110
34	491	PM	100	90	100		110		
34	492	PM	100	110	110		120		120
34	493	SSG	100	100	100	90	95	90	
34	494	Combination	110	120	120		120		120
34	495	SSG	100	100	90	85	85		90
34	496	SSG	120	110	110	120	120		110
34	497	PM	100	100	100		100	100	
34	498	SSG	90	100	100	110	110		100
34	499	Combination	80	80	90		90		100
34	500	PM	100	110	100		100		110
34	501	Combination	100	80	90		90		90
34	502	Combination	110	110	100		110		110
34	503	PM	120	80	90		90		
34	504	Combination	100	100	100		100		

Centre Number	Patient Number	Treatment	Systolic Blood Pressure (mmHg)					
			Baseline	Day 7	Day 14	Day 21	EOT	3Mon FU	6Mon FU
34	505	SSG	120	120	120	120	120		120
34	506	SSG	80	80	80	90	90		90
34	507	SSG	100	90	90	100	100		100
34	508	PM	85	90	90		100		
34	509	PM	120	120	120		120		
34	510	Combination	90	90	90		90		90
34	511	Combination	80	90	90		90		90
34	512	PM	80	90	90		90		
34	513	Combination	100	100	100		90	90	100
34	514	SSG	90	90	90	90	90		110
34	515	SSG	90	90	90	90	90	90	
34	516	PM	120	120	120		110		110
34	517	PM	90	90	100		90		
34	518	SSG	100	90	90	90	90		90
34	519	PM	100	95	90		80	90	
34	520	SSG	120	120	110	120	110		120
34	521	PM	90	80	80		80		
34	522	Combination	110	120	120		120		
34	523	SSG	90	90	90	90	90		100
34	524	Combination	110	110	110		110		100
34	525	Combination	100	110	100		110		100
34	526	SSG	100	90	90	80	80		80
34	527	Combination	110	110	110		110		120
34	528	PM	90	90	90		90		
34	529	Combination	80	80	80		80		80
34	530	Combination	110	110	110		110		110
34	531	SSG	90	90	85	90	90		
34	532	Combination	90	100	90		100		100
34	533	SSG	100	90	100	100	90		100
34	534	PM	90	80	70		70		
34	535	PM	100	100	90		100	100	
34	536	PM	90	80	80		70		85
34	537	SSG	90	90	80	80	90		100
34	538	PM	100	100	90		80		
34	539	Combination	90	90	90		90		90
34	540	SSG	100	110	100	100	110		120
35	646	PM	105	105	100		100		110
35	647	PM	100	100	110		120	110	120
35	648	Combination	80	75	80		80	80	95
35	649	Combination	100	105	95		100	100	100
35	650	SSG	90	90	80	80	80	90	100
35	651	SSG	95	90	90	90	90	80	95
35	652	PM	90	95	90		90		90
35	653	Combination	90	80	90		90	100	90
35	654	SSG	80	80	80	85	85	90	90
35	655	Combination	100	90	95		110	100	100
35	656	SSG	80	80	75	95	90	110	100
35	657	PM	100	90	100		100	90	100
35	658	Combination	90	100	100		110	110	110
35	659	SSG	70	60	90	90	90	90	90
35	660	PM	110	110	110		120	120	120
35	661	Combination	110	110	120		120	110	120
35	662	PM	110	100	115		120	120	110
35	663	SSG	120	110	130	110	120	120	120

Centre Number	Patient Number	Treatment	Systolic Blood Pressure (mmHg) Baseline	Day 7	Day 14	Day 21	EOT	3Mon FU	6Mon FU
35	664	Combination	130	100					120
35	665	SSG	100	100	110	110	100	100	100
35	666	SSG	110	110	110	100	110	100	100
35	667	Combination	90	100	110		110	110	100
35	668	PM	105	100	100		90		110
35	669	PM	90	100	80		100		
35	670	SSG	85	80	80	80	95	110	110
35	671	PM	80	85	85		80		90
35	672	Combination	90	80	85		90		100
35	673	SSG	100	110	115	110	110	105	100
35	674	Combination	80	80	80		90	90	90
35	675	PM	110	110	105		100		90
35	676	PM	100	100	80		90	80	80
35	677	SSG	110	125					
35	678	Combination	110	120	120		110	110	105
35	679	SSG	95	90	90	90	110	120	120
35	680	Combination	90	80	90		80	90	95
35	681	Combination	100	100	100		100	110	110
35	682	PM	80	80	90		90		100
35	683	Combination	100	120	110		110		100
35	684	SSG	120	110	110	110	120	120	120
35	685	PM	90	100	100		110	110	100
35	686	PM	120	110	110		110	120	120
35	687	SSG	105	100	105	100	100	110	120
35	688	PM	120	110	100		100	105	110
35	689	Combination	90	100	105		110	110	110
35	690	SSG	80	90	90	100	100	100	100

Centre Number	Patient Number	Treatment	Diastolic Blood Pressure (mmHg)						
			Baseline	Day 7	Day 14	Day 21	EOT	3Mon FU	6Mon FU
11	1	PM	80	70	70		70	60	70
11	2	Combination	80	70	70		80	80	70
11	3	Combination	70	70	70		80	80	80
11	4	PM	60	60	70		70	60	60
11	5	PM	80	70	70		70	70	80
11	6	Combination	60	60	60		60	70	60
11	7	PM	70	70	70		60	60	60
11	8	SSG	70	70	60	70	60	60	60
11	9	SSG	80	70	80	60	80	70	80
11	10	SSG	70	50	60	60	60	60	60
11	11	Combination	80	70	70		65	65	70
11	12	Combination	70	70	60		60	70	70
11	13	SSG	70	65	50	50	60	70	60
11	14	SSG	80	60	70	70	60	60	60
11	15	PM	70	70				70	70
11	16	Combination	70	60	60		60	70	60
11	17	Combination	60	60	70		70	70	60
11	18	SSG	80	70	70	70	70	50	60
11	19	Combination	60	60	70		60	70	70
11	20	SSG	60	60	60	60	60	80	70
11	21	SSG	60	80	80	60	80	80	80
11	22	SSG	70	70	60	70	50	60	60
11	23	PM	60	70	60		70	70	70
11	24	PM	60	70	70		70	60	70
11	25	SSG	70	60	60	60	60	60	60
11	26	Combination	80	60	70		60	70	80
11	27	PM	70	70	80		70	40	
11	28	Combination	70	60	60		60	60	60
11	29	PM	60	70	60		70	60	70
11	30	PM	70	50	60		60	60	60
11	31	SSG	60	70	70	70	70	60	80
11	32	Combination	60	60	70		60	80	60
11	33	SSG	80	70	80	80	80	80	80
11	34	PM	70	70	60		60	70	60
11	35	SSG	60	70	60	60	70	60	60
11	36	SSG	60	70	60	60	60	70	80
11	37	Combination	80	80	80		90	100	100
11	38	PM	80	70	60		60	50	60
11	39	PM	70	70	60		70	60	60
11	40	PM	80	70	70			80	70
11	41	Combination	70	60	70		60	60	70
11	42	Combination	70	70	80		70	70	70
11	43	SSG	70	70	70	70	70	70	60
11	44	PM	70	60	70		60	60	80
11	45	Combination	60	80	60		70	70	70
11	46	SSG	80	70	50	60	70	70	60
11	47	SSG	70	50	50	60	60		
11	48	Combination	70	60	50		50		70
11	49	SSG	50	50	60	50	60	70	60
11	50	PM	70	60	70		70	70	60
11	51	Combination	60	60	80		50	60	70
11	52	PM	70	60	70		70	70	70
11	53	SSG	60	50	60	60	70	60	60
11	54	PM	60	50	60		50	50	60

Centre Number	Patient Number	Treatment	Diastolic Blood Pressure (mmHg) Baseline	Day 7	Day 14	Day 21	EOT	3Mon FU	6Mon FU
11	55	PM	60	50	60		50	70	60
11	56	SSG	70	50	60	70	60	70	60
11	57	Combination	60	50	50		70	60	60
11	58	Combination	70	70	50		60	60	60
11	59	PM	70	50	70		100	70	70
11	60	Combination	60	50	60		60	60	60
11	61	PM	60	50	60		60	70	70
11	62	SSG	70	50	70	70	70	60	70
11	63	SSG	60	60	60	60	60	70	70
11	64	SSG	60	50	60	60	60	60	70
11	65	PM	60	60	60		60		
11	66	Combination	70	60	40		50	60	60
11	67	PM	70	70	60		80	80	70
11	68	SSG	60	50	50	60	70	70	60
11	69	Combination	60	60	70		60	70	70
11	70	Combination	70	60	70		70	70	70
11	71	Combination	50	70	70		70	60	50
11	72	SSG	70	70	80			70	80
11	73	PM	50	60	60		70	60	80
11	74	PM	60	60	60		70	70	60
11	75	Combination	60	60	70		80	60	60
11	76	PM	40	60	70		60	80	70
11	77	PM	60	60	60		70	70	60
11	78	SSG	40	60	60	70	70	60	70
11	79	PM	70	60	60		60	50	60
11	80	PM	60	60	60		70	60	60
11	81	Combination	70	70	90		80	70	70
11	82	SSG	50	60	75	60	60	70	60
11	83	SSG	50	60	60	60	70	70	70
11	84	SSG	50	60	60	60	60	70	60
11	85	SSG	70	60	60	70	70	60	
11	86	Combination	50	60	60		60	60	60
11	87	Combination	60	60	60		60	60	80
11	88	PM	60	70	60		60	80	70
11	89	Combination	70	60	60		60	80	80
11	90	Combination	60	60	70		50		
11	91	PM	70	60	60		60	70	60
11	92	Combination	40	50	60		60	60	60
11	93	SSG	60	60	60	60	70	60	80
11	94	SSG	60	60	50	70	60	60	60
11	95	SSG	70	60	60	60	60	60	50
11	96	Combination	60	60	60		60	60	60
11	97	Combination	60	60	70		60	80	70
11	98	PM	50	60	60		50	70	60
11	99	SSG	50	60	60	50	60	60	60
11	100	PM	60	60	60		60		
11	101	PM	60	80	80		80	70	80
11	102	Combination	50	50	50		70	70	60
11	103	Combination	70	70	60		90	70	80
11	104	PM	70	70	60		70	70	70
11	105	SSG	60	60	70	70	70	60	60
11	106	Combination	60	50	60		50	70	70
11	107	PM	60	60	80		70	60	70
11	108	PM	70	60	60		70	60	60

Centre Number	Patient Number	Treatment	Diastolic Blood Pressure (mmHg)						
			Baseline	Day 7	Day 14	Day 21	EOT	3Mon FU	6Mon FU
11	109	SSG	60	70	70	60	60	80	60
11	110	PM	50	50	50		60	50	60
11	111	Combination	50	60	60		60	80	70
11	112	Combination	60	50	60		80	60	60
11	113	SSG	60	60	50	60	70	60	
11	114	SSG	70	60	80	60	60	70	80
11	115	Combination	60	80	80		70	80	80
11	116	SSG	60	60	60	60	70	70	70
11	117	PM	60	80	70		70	80	
11	118	PM	60	70	70		80	60	70
11	119	SSG	60	60	70	60	60	80	60
11	120	Combination	60	60	70		60	60	70
11	121	Combination	60	60	60		70	60	50
11	122	SSG	50	50	60	60	50		
11	123	SSG	50	60	70	80	70	60	70
11	124	SSG	60	50	90	70	60		60
11	125	SSG	60	60	60	60	70	70	80
11	126	Combination	60	60	50		60		60
11	127	PM	60	50	60		60	60	70
11	128	Combination	60	60	50		50	70	70
11	129	PM	70	70	60		70	60	80
11	130	Combination	60	60	70		60	80	70
11	131	PM	60	50	60		60	60	60
11	132	SSG	60	60	80	60	80	70	60
11	133	Combination	60	60	70		60	70	60
11	134	PM	40	50	70		70	80	60
11	135	PM	60	60	60		70	80	70
12	241	Combination	60	70	80		70		80
12	242	SSG	60	60	60	60	70	60	60
12	243	PM	60	60	70		70	60	60
12	244	SSG	60	60	60	80	80	70	80
12	245	PM	60	60	70		70	70	
12	246	PM	70	70	70		80	80	
12	247	PM	60	60	60		70	70	60
12	248	PM	60	70	70		70	80	80
12	249	Combination	60	60	60		60	60	60
12	250	SSG	60	70	60	60	60	60	60
12	251	Combination	80	80	70		80	60	60
12	252	SSG	70	60	70	80	70	70	60
12	253	SSG	60	60	60	60	80	60	70
12	254	Combination	50	50	50		50	50	60
12	255	Combination	80	70	60		80	60	60
12	256	Combination	60	60	70		70	60	70
12	257	SSG	60	70	80	80	60	60	60
12	258	SSG	50	60	60	60	80		60
12	259	PM	70	80	60		60	70	70
12	260	PM	80	60	60		60		60
12	261	Combination	70	60	60		60	80	80
12	262	PM	60	60	60		60	60	50
12	263	SSG	70	70	60	60	70	60	60
12	264	Combination	60	60	70		60	60	60
12	265	SSG	50	60	50	50	40		
12	266	PM	70	60	70		70	60	70
12	267	SSG	70	50	60	60	60	70	60

Centre Number	Patient Number	Treatment	Diastolic Blood Pressure (mmHg)						
			Baseline	Day 7	Day 14	Day 21	EOT	3Mon FU	6Mon FU
12	268	PM	60	60	70		70	70	70
12	269	Combination	60	60	70		70	70	70
12	270	Combination	70	50	40		50		60
12	271	SSG	60	60	60	70	60	80	70
12	272	PM	50	50	60		60	50	50
12	273	SSG	60	60	50	60	70	60	60
12	274	SSG	60	50	50	60	60	60	60
12	275	Combination	60	70	60		60	60	60
12	276	PM	60	60	60		70	60	65
12	277	Combination	60	60	70		80	60	80
12	278	SSG	60	60	70	70	70	70	
12	279	PM	50	50	60		60	80	70
12	280	SSG	40	50	50	50	50	40	60
12	281	Combination	70	70	70		70	60	60
12	282	PM	40	60	60		60	50	60
12	283	PM	70	70	60		60	70	70
12	284	Combination	50	50	40		50	50	45
12	285	Combination	60	60	70		70	70	70
12	286	PM	80	60	70		60	60	60
12	287	SSG	70	80	70	70	60	60	60
12	288	Combination	70	60	60		60	60	60
12	289	Combination	70	60	80		80	70	80
12	290	SSG	80	60	60	60	60		60
12	291	SSG	60	60	70	60	60	60	70
12	292	PM	50	50	50		60		70
12	293	SSG	60	60	70	60	60		70
12	294	Combination	50	40	60		50	60	60
12	295	Combination	50	40	60		60		60
12	296	PM	60	60	60		60	60	60
12	297	SSG	60	70	60				
12	298	PM	80	70	70		70	70	70
12	299	Combination	70	60	60		60		70
12	300	PM	60	70	60		60	70	60
12	301	SSG	60	80	70	70	70	80	70
12	302	SSG	40	50	50	50	50	60	40
12	303	PM	80	70	70		70	80	80
12	304	Combination	60	80	80		70	70	70
12	305	PM	30	40	50		50	40	40
12	306	Combination	50	40	60		50	40	50
12	307	Combination	80	70	70		70	70	70
12	308	PM	80	60	60		60	80	80
12	309	PM	70	60	60		60	70	60
12	310	SSG	70	70	60	60	60	60	70
12	311	SSG	50	50	60	60	50	60	50
12	312	Combination	60	60	60		60	60	80
12	313	PM	60	60	60		60	70	70
12	314	SSG	60	60	50	60	60	80	70
12	315	Combination	40	50	70		70	50	70
12	316	SSG	60	80	60		60	50	60
12	317	SSG	30	40	50	60	60		40
12	318	Combination	60	50	70		50	60	40
12	319	Combination	60	60	50		60	60	50
12	320	Combination	50	50	60		50	50	60
12	321	Combination	60	60	60		60	50	50

Centre Number	Patient Number	Treatment	Diastolic Blood Pressure (mmHg)						
			Baseline	Day 7	Day 14	Day 21	EOT	3Mon FU	6Mon FU
12	322	PM	70	60	70		60	60	60
12	323	SSG	60	60	60	60	60	60	70
12	324	SSG	70	60	60	60	60	80	80
12	325	PM	60	60	60		80	70	70
12	326	PM	50	50	60		60	80	70
12	327	Combination	70	70	70		60	60	60
12	328	PM	70	60	60		80	70	60
12	329	SSG	50	50	50	60	60	60	50
12	330	PM	70	50	70		60	60	30
23	361	Combination	50	60	60		60	60	60
23	362	PM	80	70	60		80	70	60
23	363	PM	70	60	60		60	60	60
23	364	Combination	70	70	60		70	60	60
23	365	SSG	80	60	60	70	70	70	60
23	366	Combination	60	60	70		60	70	60
23	367	SSG	60	70	70	60	80	70	60
23	368	PM	60	70	60		60	60	60
23	369	Combination	50	60	60		60	50	50
23	370	SSG	60	60	60	60	60	60	50
23	371	PM	60	60	70		70	60	50
23	372	PM	70	70	70		70	60	70
23	373	SSG	80	70	60	60	70	60	60
23	374	SSG	60	60	60	60	60	60	70
23	375	Combination	60	60	60		60	60	60
23	376	SSG	60	60	60	60	70	60	60
23	377	Combination	70	60					60
23	378	Combination	50	60	60		60	60	70
23	379	PM	60	60	60		60	60	50
23	380	SSG	60	60	60	60	60	60	60
23	381	PM	60	60	60		60	60	60
23	382	Combination	60	60	60		60	60	60
23	383	PM	60	70	60		70	70	60
23	384	SSG	60	60	70	60	60	60	60
23	385	SSG	70	60	60	60	60	60	60
23	386	Combination	70	60	60		60	60	70
23	387	Combination	70	60	60		60	70	70
23	388	PM	60	60	50		60	70	60
23	389	SSG	70	60	70	60	60	50	60
23	390	PM	60	70	70		60	70	70
23	391	Combination	60	60	60		60	60	60
23	392	Combination	60	50	60			60	60
23	393	SSG	60	60	60	60	60	60	70
23	394	PM	60	70	60		60	60	60
23	395	PM	80	60	70		70	70	70
23	396	SSG	70	60	60	70	60	70	70
23	397	SSG	60	60	60	60	60	60	60
23	398	Combination	50	50	60		60	60	70
23	399	PM	60	50	50		60	60	60
23	400	PM	60	60	60		60	60	50
23	401	Combination	60	60	70		60	60	70
23	402	Combination	70	70	70		70	70	70
23	403	SSG	60	60	60	60	60	70	60
23	404	SSG	60	60	60	70	60		70
23	405	PM	60	60	60		60	70	60

Centre Number	Patient Number	Treatment	Diastolic Blood Pressure (mmHg)					
			Baseline	Day 7	Day 14	Day 21	EOT	3Mon FU	6Mon FU
34	451	Combination	60	60	75		70		70
34	452	PM	60	65	80		80		
34	453	PM	60	70	70		70		
34	454	Combination	60	60	60		60		65
34	455	PM	60	80	80		60		
34	456	SSG	55	60	60	70	80	60	
34	457	Combination	70	75	80		80		
34	458	PM	60	75	15		80	70	
34	459	Combination	65	70	65		60	60	70
34	460	PM	70	80	70		70		
34	461	SSG	70	80	80				
34	462	SSG	60	70	60	60	70		60
34	463	Combination	50	60	60		55		70
34	464	SSG	80	70	80	80	80		80
34	465	SSG	70	80	80	80	80		80
34	466	Combination	60	60	60		60		
34	467	PM	70	70	80		80	60	
34	468	Combination	80	80	80		80		80
34	469	SSG	80	80	70	80	80		70
34	470	Combination	70	80	80		80		70
34	471	PM	80	70	80		80		
34	472	SSG	60	60	60	60	50		60
34	473	PM	60	50	50		60		
34	474	Combination	60	60	60		70		70
34	475	PM	70	60	60		80		
34	476	PM	80	80	80		80	70	
34	477	SSG	60	60	70	70	60		70
34	478	Combination	60	60	70		70		60
34	479	SSG	55	60	70	60	60		50
34	480	SSG	60	70	80	80	70		70
34	481	Combination	70	70	70		70		70
34	482	Combination	80	80	80		80		70
34	483	PM	80	80	80		80		70
34	484	PM	60	60					
34	485	Combination	60	50	70		60		60
34	486	Combination	70	80	80		80		70
34	487	SSG	70	70	80	80	80		80
34	488	SSG	65	80	60	60	60	60	70
34	489	SSG	70	80	60	60	60		70
34	490	PM	65	70	60		60		80
34	491	PM	70	70	70		70		
34	492	PM	70	70	70		70		80
34	493	SSG	70	70	70	60	60	60	
34	494	Combination	70	80	80		80		70
34	495	SSG	70	80	60	60	60		60
34	496	SSG	70	70	70	80	80		80
34	497	PM	70	70	70		70	70	
34	498	SSG	70	70	70	70	70		70
34	499	Combination	60	60	60		60		70
34	500	PM	80	80	60		80		70
34	501	Combination	70	60	60		60		60
34	502	Combination	70	80	70		70		70
34	503	PM	70	60	60		60		
34	504	Combination	80	70	70		70		

Centre Number	Patient Number	Treatment	Diastolic Blood Pressure (mmHg) Baseline	Day 7	Day 14	Day 21	EOT	3Mon FU	6Mon FU
34	505	SSG	80	80	80	80	80		80
34	506	SSG	60	60	60	60	60		50
34	507	SSG	60	60	60	60	60		60
34	508	PM	60	60	60		60		
34	509	PM	80	80	80		80		
34	510	Combination	60	60	60		60		60
34	511	Combination	60	60	60		60		60
34	512	PM	60	60	60		60		
34	513	Combination	60	60	60		60	60	60
34	514	SSG	70	70	70	70	70		75
34	515	SSG	60	60	60	60	60	60	
34	516	PM	80	80	80		70		70
34	517	PM	70	60	70		60		
34	518	SSG	70	60	60	60	60		60
34	519	PM	70	70	60		60	60	
34	520	SSG	80	80	70	80	70		80
34	521	PM	60	60	60		60		
34	522	Combination	80	80	80		80		
34	523	SSG	60	60	60	60	60		70
34	524	Combination	80	70	80		80		70
34	525	Combination	70	70	70		70		60
34	526	SSG	60	60	60	60	60		60
34	527	Combination	70	70	70		70		70
34	528	PM	60	60	60		60		
34	529	Combination	60	60	60		60		60
34	530	Combination	70	70	70		70		70
34	531	SSG	60	60	60	60	60		
34	532	Combination	60	60	60		65		60
34	533	SSG	70	60	70	70	60		70
34	534	PM	60	50	50		50		
34	535	PM	70	70	60		70	70	
34	536	PM	60	60	60		50		60
34	537	SSG	70	60	60	60	60		70
34	538	PM	80	70	60		60		
34	539	Combination	60	60	60		60		60
34	540	SSG	80	80	70	70	80		70
35	646	PM	65	55	60		70		75
35	647	PM	50	50	60		80	70	80
35	648	Combination	50	50	50		50	55	50
35	649	Combination	60	55	50		60	65	55
35	650	SSG	60	55	50	50	40	50	50
35	651	SSG	70	70	65	70	65	60	60
35	652	PM	70	65	75		60		65
35	653	Combination	50	60	50		60	65	50
35	654	SSG	60	60	60	65	65	60	60
35	655	Combination	50	50	60		60	60	60
35	656	SSG	60	65	65	70	65	60	60
35	657	PM	70	75	70		70	60	60
35	658	Combination	60	70	80		80	75	80
35	659	SSG	50	55	60	55	60	65	60
35	660	PM	70	80	80		70	80	75
35	661	Combination	60	60	70		65	80	70
35	662	PM	65	65	75		80	70	70
35	663	SSG	70	70	80	60	75	85	85

Centre Number	Patient Number	Treatment	Diastolic Blood Pressure (mmHg) Baseline	Day 7	Day 14	Day 21	EOT	3Mon FU	6Mon FU
35	664	Combination	80	70					70
35	665	SSG	80	70	80	70	65	70	60
35	666	SSG	60	60	80	70	80	70	65
35	667	Combination	50	70	60		60	70	70
35	668	PM	65	70	80		60		70
35	669	PM	60	60	60		60		
35	670	SSG	70	60	60	55	60	60	65
35	671	PM	50	65	60		60		60
35	672	Combination	60	60	60		70		70
35	673	SSG	60	70	70	60	70	70	70
35	674	Combination	50	50	55		70	65	70
35	675	PM	80	70	60		80		60
35	676	PM	60	65	65		60	50	50
35	677	SSG	60	80					
35	678	Combination	70	70	80		70	70	70
35	679	SSG	60	60	60	60	70	70	65
35	680	Combination	60	65	70		60	70	70
35	681	Combination	60	80	80		60	70	75
35	682	PM	55	60	70		60		60
35	683	Combination	70	80	80		80		70
35	684	SSG	70	70	70	60	80	70	80
35	685	PM	70	60	65		70	65	65
35	686	PM	80	80	75		80	80	80
35	687	SSG	65	60	60	70	60	70	80
35	688	PM	60	60	60		70	70	70
35	689	Combination	50	60	60		60	75	80
35	690	SSG	50	50	50	75	70	70	65

16.2.6 Compliance and/or drug concentration data

Appendix 32: Listing of Daily Paromomycin Treatment

Appendix 33: Listing of Daily SSG Treatment

Appendix 34: Listing of Daily Combination Treatment (Paromomycin)

Appendix 35: Listing of Daily Combination Treatment (SSG)

Appendix 36: Listing of Rescue medication

Appendix 37: Listing of concomitant medications

Centre	Patient	Paromomycin mg/day (Dose 15mg/kg/day)																				
Number	Number	D1	D2	D3	D4	D5	D6	D7	D8	D9	D10	D11	D12	D13	D14	D15	D16	D17	D18	D19	D20	D21
11	1	720	720	720	720	720	720	720	720	720	720	720	720	765	765	765	765	765	765	765	765	765
11	4	675	675	675	675	675	675	675	735	735	735	735	735	735	735	720	720	720	720	720	720	720
11	5	300	300	300	300	300	300	300	315	315	315	315	315	315	315	330	330	330	330	330	330	330
11	7	705	705	705	705	705	705	705	720	720	720	720	720	720	720	690	690	690	690	690	690	690
11	15	705	705	705	705	705	705	705														
11	23	390	390	390	390	390	390	390	375	375	375	375	375	375	375	375	375	375	375	375	375	375
11	24	270	270	270	270	270	270	270	240	240	240	240	240	240	240	240	240	240	240	240	240	240
11	27	690	690	690	690	690	690	690	690	690	690	690	690	690	690	690	690	690	690	690	690	690
11	29	630	630	630	630	630	630	630	630	630	630	630	630	630	630	645	645	645	645	645	645	645
11	30	225	225	225	225	225	225	225	210	210	210	210	210	210	210	225	225	225	225	225	225	225
11	34	735	735	735	735	735	735	735	735	735	735	735	735	735	735	735	735	735	735	735	735	735
11	38	720	720	720	720	720	720	720	720	720	720	720	720	720	720	735	735	735	735	735	735	735
11	39	270	270	270	270	270	270	270	270	270	270	270	270	270	270	270	270	270	270	270	270	270
11	40	240	240	240	240	240	240	240	240	270	270	270	270	270	270	255						
11	44	675	675	675	675	675	675	675	690	690	690	690	690	690	690	705	705	705	705	705	705	705
11	50	825	825	825	825	825	825	825	825	825	825	825	825	825	825	840	840	840	840	840	840	840
11	52	360	360	360	360	360	360	360	375	375	375	375	375	375	375	375	375	375	375	375	375	375
11	54	690	690	690	690	690	690	690	705	705	705	705	705	705	705	720	720	720	720	720	720	720
11	55	690	690	690	690	690	690	690	690	690	690	690	690	690	690	720	720	720	720	720	720	720
11	59	345	345	345	345	345	345	345	330	330	330	330	330	330	330	345	345	345	345	345	345	345
11	61	705	705	705	705	705	705	705	720	720	720	720	720	720	720	720	720	720	720	720	720	720
11	65	210	210	210	210	210	210	210	225	225	225	225	225	225	225	225	225	225	225	225	225	225
11	67	330	330	330	330	330	330	330	360	360	360	360	360	360	360	345	345	345	345	345	345	345
11	73	720	720	720	720	720	720	720	720	720	720	720	720	720	720	735	735	735	735	735	735	735
11	74	735	735	735	735	735	735	735	720	720	720	720	720	720	720	735	735	735	735	735	735	735
11	76	675	675	675	675	675	675	675	675	675	675	675	675	675	675	675	675	675	675	675	675	675
11	77	300	300	300	300	300	300	300	300	300	300	300	300	300	300	330	330	330	330	330	330	330
11	79	180	180	180	180	180	180	180	195	195	195	195	195	195	195	195	195	195	195	195	195	195
11	80	330	330	330	330	330	330	330	315	315	315	315	315	315	315	330	330	330	330	330	330	330
11	88	795	795	795	795	795	795	795	780	780	780	780	780	780	780	795	795	795	795	795	795	795
11	91	780	780	780	780	780	780	780	795	795	795	795	795	795	795	795	795	795	795	795	795	795
11	98	525	525	525	525	525	525	525	525	525	525	525	525	525	525	510	510	510	510	510	510	510
11	100	660	660	660	660	660	660	660	675	675	675	675	675	675	675	675	675	675	675	675	675	675
11	101	660	660	660	660	660	660	660	645	645	645	645	645	645	645	645	645	645	645	645	645	645
11	104	450	450	450	450	450	450	450	435	435	435	435	435	435	435	435	435	435	435	435	435	435
11	107	705	705	705	705	705	705	705	720	720	720	720	720	720	720	735	735	735	735	735	735	735
11	108	690	690	690	690	690	690	690	705	705	705	705	705	705	705	720	720	720	720	720	720	720
11	110	225	225	225	225	225	225	225	225	225	225	225	225	225	225	210	210	210	210	210	210	210
11	117	810	810	810	810	810	810	810	810	810	810	780	780	780	780	795	795	795	795	795	795	795
11	118	705	705	705	705	705	705	705	735	735	735	735	735	735	735	750	750	750	750	750	750	750
11	127	405	405	405	405	405	405	405	420	420	420	420	420	420	420	420	420	420	420	420	420	420
11	129	600	600	600	600	600	600	600	585	585	585	585	585	585	585	600	600	600	600	600	600	600
11	131	255	255	255	255	255	255	255	255	255	255	255	255	255	255	270	270	270	270	270	270	270
11	134	795	795	795	795	795	795	795	765	765	765	765	765	765	765	780	780	780	780	780	780	780
11	135	705	705	705	705	705	705	705	720	720	720	720	720	720	720	720	720	720	720	720	720	720
12	243	330	330	330	330	330	330	330	330	330	330	330	330	330	330	375	375	375	375	375	375	375
12	245	645	645	645	645	645	645	645	645	645	645	645	645	645	645	645	645	645	645	645	645	645
12	246	690	690	690	690	690	690	690	735	735	735	735	735	735	735	735	735	735	735	735	735	735
12	247	360	360	360	360	360	360	360	390	390	390	390	390	390	390	375	375	375	375	375	375	375
12	248	803	803	803	803	803	803	825	825	825	825	825	825	825	825	825	825	825	825	825	825	825
12	259	870	870	870	870	870	870	870	900	900	900	900	900	900	900	900	900	900	900	900	900	900
12	260	555	555	555	555	555	555	555	585	585	585	585	585	585	585	585	585	585	585	585	585	585
12	262	345	345	345	345	345	345	375	375	375	375	375	375	375	375	375	375	375	375	375	375	375
12	266	885	885	885	885	885	885	885	900	900	900	900	900	900	900	900	900	900	900	900	900	900

Centre	Patient	Paromomycin mg/day (Dose 15mg/kg/day)																				
Number	Number	D1	D2	D3	D4	D5	D6	D7	D8	D9	D10	D11	D12	D13	D14	D15	D16	D17	D18	D19	D20	D21
12	268	900	900	900	900	900	900	900	900	900	900	900	900	900	900	930	930	930	930	930	930	930
12	272	165	165	165	165	165	165	165	180	180	180	180	180	180	180	180	180	180	180	180	180	180
12	276	300	300	300	300	300	300	300	323	323	323	323	323	323	323	330	330	330	330	330	330	330
12	279	660	660	660	660	660	660	660	690	690	690	690	690	690	690	720	720	720	720	720	720	720
12	282	270	270	270	270	270	270	270	300	300	300	300	300	300	300	300	300	300	300	300	300	300
12	283	810	810	810	810	810	810	810	825	825	825	825	825	825	825	840	840	840	840	840	840	840
12	286	465	465	465	465	465	465	465	480	480	480	480	480	480	480	480	480	480	480	480	480	480
12	292	750	750	750	750	750	750	750	750	750	750	750	750	750	750	750	750	750	750	750	750	750
12	296	345	345	345	345	345	345	345	360	360	360	360	360	360	360	360	360	360	360	360	360	360
12	298	405	405	405	405	405	405	405	390	390	390	390	390	390	390	405	405	405	405	405	405	405
12	300	675	675	675	675	675	675	660	660	660	660	660	660	660	660	660	660	660	660	660	660	660
12	303	600	600	600	600	600	600	600	600	600	600	600	600	600	600	600	600	600	600	600	600	600
12	305	210	210	210	210	210	210	210	210	210	210	210	210	210	210	210	210	210	210	210	210	210
12	308	855	855	855	855	855	855	855	840	840	840	840	840	840	840	863	863	863	863	863	863	863
12	309	795	795	795	795	795	795	795	795	795	795	795	795	795	795	795	795	795	795	795	795	795
12	313	870	870	870	870	870	870	870	870	870	870	870	870	870	870	885	885	885	885	885	885	885
12	322	600	600	600	600	600	600	600	585	585	585	585	585	585	585	585	585	585	585	585	585	855
12	325	825	825	825	825	825	825	825	870	870	870	870	870	870	870	900	900	900	900	900	900	900
12	326	675	675	675	675	675	675	675	690	690	690	690	690	690	690	675	675	675	675	675	675	675
12	328	435	435	435	435	435	435	435	420	420	420	420	420	420	420	420	420	420	420	420	420	420
12	330	390	390	390	390	390	390	390	435	435	435	435	435	435	435	435	435	435	435	435	435	435
23	362	600	600	600	600	600	600	600	600	600	600	600	600	600	600	600	600	600	600	600	600	600
23	363	250	250	250	250	250	250	250	250	250	250	250	250	250	250	250	250	250	250	250	250	250
23	368	750	750	750	750	750	750	750	750	750	750	750	750	750	750	750	750	750	750	750	750	750
23	371	550	550	550	550	550	550	550	550	550	550	550	550	550	550	550	550	550	550	550	550	550
23	372	850	850	850	850	850	850	850	850	850	850	850	850	850	850	850	850	850	850	850	850	850
23	379	300	300	300	300	300	300	300	300	300	300	300	300	300	300	300	300	300	300	300	300	300
23	381	450	450	450	450	450	450	450	450	450	450	450	450	450	450	450	450	450	450	450	450	450
23	383	650	650	650	650	650	650	650	650	650	650	650	650	650	700	700	700	700	700	700	700	700
23	388	400	400	400	400	400	400	400	400	400	400	400	400	400	450	450	450	450	450	450	450	450
23	390	700	700	700	700	700	700	700	700	700	700	700	700	700	700	700	700	700	700	700	700	700
23	394	300	300	300	300	300	300	300	300	300	300	300	300	300	325	325	325	325	325	325	325	325
23	395	800	800	800	800	800	800	800	800	800	800	800	850	850	850	850	850	850	850	850	850	850
23	399	300	300	300	300	300	300	300	300	300	300	300	300	300	300	300	300	300	300	300	300	300
23	400	250	250	250	250	250	250	250	250	250	250	250	250	250	250	250	250	250	250	250	250	250
23	405	600	600	600	600	600	600	600	600	600	600	600	600	600	600	600	600	600	600	600	600	600
34	452	400	400	400	400	400	400	400	400	400	400	400	400	400	400	450	450	450	450	450	450	450
34	453	250	250	250	250	250	250	250	250	250	250	250	250	250	250	250	250	250	250	250	250	250
34	455	300	300	300	300	300	300	300	350	350	350	350	350	350	350	350	350	350	350	350	350	350
34	458	350	350	350	350	350	350	350	400	400	400	400	400	400	400	400	400	400	400	400	400	400
34	460	200	200	200	200	200	200	200	200	200	200	200	200	200	200	200	200	200	200	200	200	200
34	467	450	450	450	450	450	450	450	500	500	500	500	500	500	500	500	500	500	500	500	500	500
34	471	450	450	450	450	450	450	450	450	450	450	450	450	450	450	450	450	450	450	450	450	450
34	473	250		250	250	250	250	250	250	250	250	250	250	250	250	250	250	250	250	250	250	250
34	475	250	250	250	250	250	250	250	250	250	250	250	250	250	250	250	250	250	250	250	250	250
34	476	400	400	400	400	400	400	400	400	400	400	400	400	400	400	400	400	400	400	400	400	400
34	483	850	850	850	850	850	850	850	850	850	850	850	850	850	850	850	850	850	850	850	850	850
34	484	200	200	200	200	200	200	200														
34	490	250	250	250	250	250	250	250	300	300	300	300	300	300	300	300	300	300	300	300	300	300
34	491	750	750	750	750	750	750	750	800	800	800	800	800	800	800	750	750	750	750	750	750	750
34	492	1000	1000	1000	1000	1000	1000	1000	1150	1150	1150	1150	1150	1150	1150	1150	1150	1150	1150	1150	1150	1150
34	497	750	750	750	750	750	750	750	750	750	750	750	750	750	750	750	750	750	750	750	750	750
34	500	500	500	500	500	500	500	500	500	500	500	500	500	500	500	550	550	550	550	550	550	550
34	503	400	400	400	400	400	400	400	350	350	350	350	350	350	350	350	350	350	350	350	350	350

Appendix 32: Individual listing of Paromomycin total daily dose.

Centre	Patient	Paromomycin mg/day (Dose 15mg/kg/day)																				
Number	Number	D1	D2	D3	D4	D5	D6	D7	D8	D9	D10	D11	D12	D13	D14	D15	D16	D17	D18	D19	D20	D21
34	508	200	200	200	200	200	200	200	200	200	200	200	200	200	200	200	200	200	200	200	200	200
34	509	850	850	850	850	850	850	850	850	850	850	850	850	850	850	900	900	900	900	900	900	900
34	512	200	200	200	200	200	200	200	250	250	250	250	250	250	250	250	250	250	250	250	250	250
34	516	650	650	650	650	650	650	650	650	650	650	650	650	650	650	700	700	700	700	700	700	700
34	517	400	400	400	400	400	400	400	400	400	400	400	400	400	400	400	400	400	400	400	400	400
34	519	300	300	300	300	300	300	300	350	350	350	350	350	350	350	350	350	350	350	350	350	350
34	521	250	250	250	250	250	250	250	250	250	250	250	250	250	250	250	250	250	250	250	250	250
34	528	300	300	300	300	300	300	300	300	300	300	300	300	300	300	300	300	300	300	300	300	300
34	534	200	200	200	200	200	200	200	200	200	200	200	200	200	200	200	200	200	200	200	200	200
34	535	400	400	400	400	400	400	400	400	400	400	400	400	400	400	400	400	400	400	400	400	400
34	536	400	400	400	400	400	400	400	400	400	400	400	400	400	400	400	400	400	400	400	400	400
34	538	250	250	250	250	250	250	250	250	250	250	250	250	250	250	250	250	250	250	250	250	250
35	646	480	480	480	480	480	480	480	480	480	480	480	480	480	510	510	510	510	510	510	510	510
35	647	915	915	915	915	915	915	915	915	915	915	915	915	915	975	975	975	975	975	975	975	975
35	652	300	300	300	300	300	300	300	300	300	300	300	300	300	300	300	300	300	300	300	300	300
35	657	330	330	330	330	330	330	330	330	330	330	330	330	330	345	345	345	345	345	345	345	345
35	660	800	800	800	800	800	800	800	800	800	800	800	800	800	800	800	800	800	800	800	800	800
35	662	900	900	900	900	900	900	900	900	900	900	900	900	900	900	930	930	930	930	930	930	930
35	668	630	630	630	630	630	630	600	600	600	600	600	600	600	600	600	600	600	600	600	600	600
35	669	435	435	435	435	435	435	435	435	435	435	435	435	435	450	450	450	450	450	450	450	450
35	671	270	270	270	270	270	270	270	270	270	270	270	270	270	285	285	285	285	285	285	285	285
35	675	360	360	360	360	360	360	360	360	360	360	360	360	360	375	375	375	375	375	375	375	375
35	676	250	250	250	250	250	250	250	250	250	250	250	250	250	250	250	250	250	250	250	250	250
35	682	225	225	225	225	225	225	225	240	240	240	240	240	240	225	225	225	225	225	225	225	225
35	685	555	555	555	555	555	555	555	555	555	555	555	555	555	570	570	570	570	570	570	570	570
35	686	400	400	400	400	400	400	400	400	400	400	400	400	400	520	520	520	520	520	520	520	520
35	688	585	585	585	585	585	585	615	615	615	615	615	615	615	615	615	615	615	615	615	615	615

Centre	Patient	Sodium Stibogluconate mg/day (Dose 20mg/kg/day)														
Number	Number	D1	D2	D3	D4	D5	D6	D7	D8	D9	D10	D11	D12	D13	D14	D15
11	8	850	850	850	850	850	850	850	850	850	850	850	850	850	850	850
11	9	850	850	850	850	850	850	850	850	850	850	850	850	850	850	850
11	10	740	740	740	740	740	740	740	740	740	740	740	740	740	740	760
11	13	850	850	850	850	850	850	850	850	850	850	850	850	850	850	850
11	14	400	400	400	400	400	400	400	400	400	400	400	400	400	400	400
11	18	850	850	850	850	850	850	850	850	850	850	850	850	850	850	850
11	20	440	440	440	440	440	440	440	480	480	480	480	480	480	480	480
11	21	850	850	850	850	850	850	850	850	850	850	850	850	850	850	850
11	22	480	480	480	480	480	480	480	460	460	460	460	460	460	460	460
11	25	400	400	400	400	400	400	400	340	340	340	340	340	340	340	360
11	31	780	780	780	780	780	780	780	800	800	800	800	800	800	800	760
11	33	850	850	850	850	850	850	850	850	850	850	850	850	850	850	850
11	35	850	850	850	850	850	850	850	850	850	850	850	850	850	850	850
11	36	850	850	850	850	850	850	850	850	850	850	850	850	850	850	850
11	43	850	850	850	850	850	850	850	850	850	850	850	850	850	850	850
11	46	320	320	320	320	320	320	320	320	320	320	320	320	320	320	320
11	47	850	850	850	850	850	850	850	850	850	850	850	850	850	850	850
11	49	320	320	320	320	320	320	320	320	320	320	320	320	320	320	320
11	53	850	850	850	850	850	850	850	850	850	850	850	850	850	850	850
11	56	660	660	660	660	660	660	660	620	620	620	620	620	620	620	640
11	62	320	320	320	320	320	320	320	340	340	340	340	340	340	340	320
11	63	850	850	850	850	850	850	850	850	850	850	850	850	850	850	850
11	64	850	850	850	850	850	850	850	850	850	850	850	850	850	850	850
11	68	420	420	420	420	420	420	420	380	380	380	380	380	380	380	380
11	72	850	850	850	850	850	850	850	850	850	850	850	850	850	850	850
11	78	850	850	850	850	850	850	850	850	850	850	850	850	850	850	850
11	82	500	500	500	500	500	500	500	500	500	500	500	500	500	500	540
11	83	800	800	800	800	800	800	800	780	780	780	780	780	780	780	800
11	84	380	380	380	380	380	380	380	460	460	460	460	460	460	460	460
11	85	840	840	840	840	840	840	840	840	840	840	840	840	840	840	850
11	93	850	850	850	850	850	850	850	850	850	850	850	850	850	850	850
11	94	280	280	280	280	280	280	280	300	300	300	300	300	300	300	280
11	95	300	300	300	300	300	300	300	300	300	300	300	300	300	300	300
11	99	760	760	760	760	760	760	760	760	760	760	760	760	760	760	780
11	105	720	720	720	720	720	720	720	720	720	720	720	720	720	720	760
11	109	850	850	850	850	850	850	850	850	850	850	850	850	850	850	850
11	113	850	850	850	850	850	850	850	850	850	850	850	850	850	850	850
11	114	850	850	850	850	850	850	850	850	850	850	850	850	850	850	850
11	116	850	850	850	850	850	850	850	850	850	850	850	850	850	850	850

Centre	Patient	Sodium Stibogluconate mg/day (Dose 20mg/kg/day)														
Number	Number	D16	D17	D18	D19	D20	D21	D22	D23	D24	D25	D26	D27	D28	D29	D30
11	8	850	850	850	850	850	850	850	850	850	850	850	850	850	850	850
11	9	850	850	850	850	850	850	850	850	850	850	850	850	850	850	850
11	10	760	760	760	760	760	760	760	760	720	720	720	720	720	720	720
11	13	850	850	850	850	850	850	850	850	850	850	850	850	850	850	850
11	14	400	400	400	400	400	400	420	420	420	420	420	420	420	420	420
11	18	850	850	850	850	850	850	850	850	850	850	850	850	850	850	850
11	20	460	460	460	460	460	460	460	460	460	460	460	460	460	460	460
11	21	850	850	850	850	850	850	850	850	850	850	850	850	850	850	850
11	22	460	460	460	460	460	460	460	460	460	460	460	460	460	460	460
11	25	360	360	360	360	360	360	380	380	380	380	380	380	380	380	380
11	31	760	760	760	760	760	760	800	800	800	800	800	800	800	800	800
11	33	850	850	850	850	850	850	850	850	850	850	850	850	850	850	850
11	35	850	850	850	850	850	850	850	850	850	850	850	850	850	850	850
11	36	850	850	850	850	850	850	850	850	850	850	850	850	850	850	850
11	43	850	850	850	850	850	850	850	850	850	850	850	850	850	850	850
11	46	320	320	320	320	320	320	320	320	320	320	320	320	320	320	320
11	47	850	850	850	850	850	850	850	850	850	850	850	850	850	850	850
11	49	320	320	320	320	320	320	320	320	320	320	320	320	320	320	320
11	53	850	850	850	850	850	850	850	850	850	850	850	850	850	850	850
11	56	640	640	640	640	640	640	660	660	660	660	660	660	660	660	660
11	62	320	320	320	320	320	320	320	320	320	320	320	320	320	320	320
11	63	850	850	850	850	850	850	850	850	850	850	850	850	850	850	850
11	64	850	850	850	850	850	850	850	850	850	850	850	850	850	850	850
11	68	380	380	380	380	380	380	380	380	380	380	380	380	380	380	380
11	72	850														
11	78	850	850	850	850	850	850	850	850	850	850	850	850	850	850	850
11	82	540	540	540	540	540	540	520	520	520	520	520	520	520	520	520
11	83	800	800	800	800	800	800	780	780	780	780	780	780	780	780	780
11	84	460	460	460	460	460	460	480	480	480	480	480	480	480	480	480
11	85	850	850	850	850	850	850	850	850	850	850	850	850	850	850	850
11	93	850	850	850	850	850	850	850	850	850	850	850	850	850	850	850
11	94	280	280	280	280	280	280	300	300	300	300	300	300	300	300	300
11	95	300	300	300	300	300	300	340	340	340	340	340	340	340	340	340
11	99	780	780	780	780	780	780	760	760	760	760	760	760	760	760	760
11	105	760	760	760	760	760	760	780	780	780	780	780	780	780	780	780
11	109	850	850	850	850	850	850	850	850	850	850	850	850	850	850	850
11	113	850	850	850	850	850	850	850	850	850	850	850	850	850	850	850
11	114	850	850	850	850	850	850	850	850	850	850	850	850	850	850	850
11	116	850	850	850	850	850	850	850	850	850	850	850	850	850	850	850

Centre	Patient	Sodium Stibogluconate mg/day (Dose 20mg/kg/day)														
Number	Number	D1	D2	D3	D4	D5	D6	D7	D8	D9	D10	D11	D12	D13	D14	D15
11	119	850	850	850	850	850	850	850	850	850	850	850	850	850	850	850
11	122	850	850	850	850	850	850	850	850	850	850	850	850	850	850	850
11	123	850	850	850	850	850	850	850	850	850	850	850	850	850	850	850
11	124	850	850	850	850	850	850	850	850	850	850	850	850	850	850	850
11	125	850	850	850	850	850	850	850	850	850	850	850	850	850	850	850
11	132	850	850	850	850	850	850	850	850	850	850	850	850	850	850	850
12	242	400	400	400	400	400	400	400	420	420	420	420	420	420	420	440
12	244	850	850	850	850	850	850	850	850	850	850	850	850	850	850	850
12	250	740	740	740	740	740	740	740	710	710	710	710	710	710	710	760
12	252	680	680	680	680	680	680	680	760	760	760	760	760	760	760	800
12	253	500	500	500	500	500	500	500	540	540	540	540	540	540	540	540
12	257	840	840	840	840	840	840	840	850	850	850	850	850	850	850	850
12	258	480	480	480	480	480	480	480	500	500	500	500	500	500	500	500
12	263	850	850	850	850	850	850	850	850	850	850	850	850	850	850	850
12	265	300	300	300	300	300	300	300	300	300	300	300	300	300	300	300
12	267	850	850	850	850	850	850	850	850	850	850	850	850	850	850	850
12	271	850	850	850	850	850	850	850	850	850	850	850	850	850	850	850
12	273	520	520	520	520	520	520	520	530	530	530	530	530	530	530	520
12	274	460	460	460	460	460	460	460	480	480	480	480	480	480	480	480
12	278	850	850	850	850	850	850	850	850	850	850	850	850	850	850	850
12	280	240	240	240	240	240	240	240	260	260	260	260	260	260	260	280
12	287	520	520	520	520	520	520	520	560	560	560	560	560	560	560	560
12	290	520	520	520	520	520	520	520	560	560	560	560	560	560	560	560
12	291	850	850	850	850	850	850	850	850	850	850	850	850	850	850	850
12	293	600	600	600	600	600	600	600	600	600	600	600	600	600	600	600
12	297	850	850	850	850	850	850	850	850	850	850	850	850	850	850	
12	301	850	850	850	850	850	850	850	850	850	850	850	850	850	850	850
12	302	260	260	260	260	260	260	260	280	280	280	280	280	280	280	280
12	310	600	600	600	600	600	600	600	600	600	600	600	600	600	600	600
12	311	540	540	540	540	540	540	540	560	560	560	560	560	560	560	560
12	314	850	850	850	850	850	850	850	850	850	850	850	850	850	850	850
12	316	850	850	850	850	850	850	850	850	850	850	850	850	850	850	
12	317	260	260	260	260	260	260	260	280	280	280	280	280	280	280	280
12	323	850	850	850	850	850	850	850	850	850	850	850	850	850	850	850
12	324	850	850	850	850	850	850	850	850	850	850	850	850	850	850	850
12	329	280	280	280	280	280	280	280	300	300	300	300	300	300	300	300
23	365	850	850	850	850	850	850	850	850	850	850	850	850	850	850	850
23	367	850	850	850	850	850	850	850	850	850	850	850	850	850	850	850
23	370	370	370	370	370	370	370	370	370	370	370	370	370	370	370	370

Centre	Patient	Sodium Stibogluconate mg/day (Dose 20mg/kg/day)														
Number	Number	D16	D17	D18	D19	D20	D21	D22	D23	D24	D25	D26	D27	D28	D29	D30
11	119	850	850	850	850	850	850	850	850	850	850	850	850	850	850	850
11	122	850	850	850	850	850	850	850	850	850	850	850	850	850	850	850
11	123	850	850	850	850	850	850	850	850	850	850	850	850	850	850	850
11	124	850	850	850	850	850	850	850	850	850	850	850	850	850	850	850
11	125	850	850	850	850	850	850	850	850	850	850	850	850	850	850	850
11	132	850	850	850	850	850	850	850	850	850	850	850	850	850	850	850
12	242	440	440	440	440	440	440	420	420	420	420	420	420	420	420	420
12	244	850	850	850	850	850	850	850	850	850	850	850	850	850	850	850
12	250	760	760	760	760	760	760	720	720	720	720	720	720	720	720	720
12	252	800	800	800	800	800	800	820	820	820	820	820	820	820	820	820
12	253	540	540	540	540	540	540	540	540	540	540	540	540	540	540	540
12	257	850	850	850	850	850	850	850	850	850	850	850	850	850	850	850
12	258	500	500	500	500	500	500	500	500	500	500	500	500	500	500	500
12	263	850	850	850	850	850	850	850	850	850	850	850	850	850	850	850
12	265	300	300	300	300	300	300	320	320	320	320	320	320			
12	267	850	850	850	850	850	850	850	850	850	850	850	850	850	850	850
12	271	850	850	850	850	850	850	850	850	850	850	850	850	850	850	850
12	273	520	520	520	520	520	560	560	560	560	560	560	560	560	560	560
12	274	480	480	480	480	480	480	500	500	500	500	500	500	500	500	500
12	278	850	850	850	850	850	850	850	850	850	850	850	850	850	850	850
12	280	280	280	280	280	280	280	300	300	300	300	300	300	300	300	300
12	287	560	560	560	560	560	560	580	580	580	580	580	580	580	580	580
12	290	560	560	560	560	560	560	600	600	600	600	600	600	600	600	600
12	291	850	850	850	850	850	850	850	850	850	850	850	850	850	850	850
12	293	600	600	600	600	600	600	600	600	600	600	600	600	600	600	600
12	297															
12	301	850	850	850	850	850	850	850	850	850	850	850	850	850	850	850
12	302	280	280	280	280	280	280	280	280	280	280	280	280	280	280	280
12	310	600	600	600	600	600	600	620	620	620	620	620	620	620	620	620
12	311	560	560	560	560	560	560	560	560	560	560	560	560	560	560	560
12	314	850	850	850	850	850	850	850	850	850	850	850	850	850	850	850
12	316															
12	317	280	280	280	280	280	280	280	280	280	280	280	280	280	280	280
12	323	850	850	850	850	850	850	850	850	850	850	850	850	850	850	850
12	324	850	850	850	850	850	850	850	850	850	850	850	850	850	850	850
12	329	300	300	300	300	300	300	300	300	300	300	300	300	300	300	300
23	365	850	850	850	850	850	850	850	850	850	850	850	850	850	850	850
23	367	850	850	850	850	850	850	850	850	850	850	850	850	850	850	850
23	370	370	370	370	370	370	400	400	400	400	400	400	400	400	400	400

Appendix 33: Individual Listing of SSG total daily dose

Centre	Patient	Sodium Stibogluconate mg/day (Dose 20mg/kg/day)														
Number	Number	D1	D2	D3	D4	D5	D6	D7	D8	D9	D10	D11	D12	D13	D14	D15
23	373	840	840	840	840	840	840	840	840	840	840	840	840	840	840	840
23	374	780	780	780	780	780	780	780	780	780	780	780	780	780	780	780
23	376	740	740	740	740	740	740	740	740	740	740	740	740	740	760	760
23	380	320	320	320	320	320	320	320	320	320	320	320	320	320	340	340
23	384	700	700	700	700	700	700	700	700	700	700	700	700	700	700	700
23	385	630	630	630	630	630	630	630	630	630	630	630	630	630	640	640
23	389	600	600	600	600	600	600	600	600	600	600	600	600	600	600	600
23	393	460	460	460	460	460	460	460	460	460	460	460	460	460	510	510
23	396	850	850	850	850	850	850	850	850	850	850	850	850	850	850	850
23	397	540	540	540	540	540	540	640	640	640	640	640	640	640	690	690
23	403	850	850	850	850	850	850	850	850	850	850	850	850	850	850	850
23	404	850	850	850	850	850	850	850	850	850	850	850	850	850	850	850
34	456	400	400	400	400	400	400	400	440	440	440	440	440	440	440	440
34	461	1100	1100	1100	1100											
34	462	230	230	230	230	230	230	230	240	240	240	240	240	240	240	240
34	464	1040	1040	1040	1040	1040	1040	1040	1080	1080	1080	1080	1080	1080	1080	1080
34	465	1020	1020	1020	1020	1020	1020	1020	1120	1120	1120	1120	1120	1120	1120	1120
34	469	500	500	500	500	500	500	500	520	520	520	520	520	520	520	540
34	472	260	260	260	260	260	260	260	260	260	260	260	260	260	260	260
34	477	360	360	360	360	360	360	360	360	360	360	360	360	360	360	380
34	479	200	200	200	200	200	200	200	220	220	220	220	220	220	220	240
34	480	550	550	550	550	550	550	550	570	570	570	570	570	570	570	600
34	487	740	740	740	740	740	740	740	820	820	820	820	820	820	820	800
34	488	400	400	400	400	400	400	400	440	440	440	440	440	440	440	420
34	489	520	520	520	520	520	520	520	540	540	540	540	540	540	540	540
34	493	660	660	660	660	660	660	660	720	720	720	720	720	720	720	660
34	495	300	300	300	300	300	300	300	330	330	330	330	330	330	330	320
34	496	1260	1260	1260	1260	1260	1260	1260	1260	1260	1260	1260	1260	1260	1260	1260
34	498	640	640	640	640	640	640	640	620	620	620	620	620	620	620	680
34	505	1040	1040	1040	1040	1040	1040	1040	1080	1080	1080	1080	1080	1080	1080	1130
34	506	360	360	360	360	360	360	360	380	380	380	380	380	380	380	370
34	507	300	300	300	300	300	300	300	340	340	340	340	340	340	340	360
34	514	420	420	420	420	420	420	420	440	440	440	440	440	440	440	470
34	515	470	470	470	470	470	470	470	480	480	480	480	480	480	480	520
34	518	280	280	280	280	280	280	280	320	320	320	320	320	320	320	280
34	520	1050	1050	1050	1050	1050	1050	1050	1040	1040	1040	1040	1040	1040	1040	1020
34	523	460	460	460	460	460	460	460	440	440	440	440	440	440	440	470
34	526	260	260	260	260	260	260	260	260	260	260	260	260	260	260	260
34	531	260	260	260	260	260	260	260	280	280	280	280	280	280	280	300

Centre	Patient	Sodium Stibogluconate mg/day (Dose 20mg/kg/day)														
Number	Number	D16	D17	D18	D19	D20	D21	D22	D23	D24	D25	D26	D27	D28	D29	D30
23	373	840	840	840	840	840	840	850	850	850	850	850	850	850	850	850
23	374	780	780	780	780	780	800	800	800	800	800	800	800	800	800	800
23	376	760	760	760	760	760	760	760	760	760	760	760	760	760	760	760
23	380	340	340	340	340	340	360	360	360	360	360	360	360	360	360	360
23	384	700	700	700	700	700	700	700	700	700	700	700	700	700	700	700
23	385	640	640	640	640	640	640	640	640	640	640	640	640	640	640	640
23	389	600	600	600	600	600	650	650	650	650	650	650	650	650	650	650
23	393	510	510	510	510	510	520	520	520	520	520	520	520	520	520	520
23	396	850	850	850	850	850	850	850	850	850	850	850	850	850	850	850
23	397	690	690	690	690	690	690	690	690	690	690	690	690	690	690	690
23	403	850	850	850	850	850	850	850	850	850	850	850	850	850	850	850
23	404	850	850	850	850	850	850	850	850	850	850	850	850	850	850	850
34	456	440	440	440	440	440	440	460	460	460	460	460	460	460	460	460
34	461															
34	462	240	240	240	240	240	240	240	240	240	240	240	240	240	240	240
34	464	1080	1080	1080	1080	1080	1080	1080	1080	1080	1080	1080	1080	1080	1080	1080
34	465	1120	1120	1120	1120	1120	1120	1120	1120	1120	1120	1120	1120	1120	1120	1120
34	469	540	540	540	540	540	540	540	540	540	520	520	520	520	520	520
34	472	260	260	260	260	260	260	290	290	290	290	290	290	290	290	290
34	477	380	380	380	380	380	380	380	380	380	380	380	380	380	380	380
34	479	240	240	240	240	240	240	240	240	240	240	240	240	240	240	240
34	480	600	600	600	600	600	600	600	600	600	600	600	600	600	600	600
34	487	800	800	800	800	800	800	820	820	820	820	820	820	820	820	820
34	488	420	420	420	420	420	420	460	460	460	460	460	460	460	460	460
34	489	540	540	540	540	540	540	520	520	520	520	520	520	520	520	520
34	493	660	660	660	660	660	660	660	660	660	660	660	660	660	660	660
34	495	320	320	320	320	320	320	320	320	320	320	320	320	320	320	320
34	496	1260	1260	1260	1260	1260	1260	1240	1240	1240	1240	1240	1240	1240	1240	1240
34	498	680	680	680	680	680	680	680	680	680	680	680	680	680	680	680
34	505	1130	1130	1130	1130	1130	1130	1160	1160	1160	1160	1160	1160	1160	1160	1160
34	506	370	370	370	370	370	370	370	370	370	370	370	370	370	370	370
34	507	360	360	360	360	360	360	340	340	340	340	340	340	340	340	340
34	514	470	470	470	470	470	470	470	470	470	470	470	470	470	470	470
34	515	520	520	520	520	520	520	520	520	520	520	520	520	520	520	520
34	518	280	280	300	280	280	280	280	300	300	300	300	300	300	300	300
34	520	1020	1020	1020	1020	1020	1020	1040	1040	1040	1040	1040	1040	1040	1040	1040
34	523	470	470	470	470	470	470	480	480	480	480	480	480	480	480	480
34	526	260	260	260	260	260	260	270	270	270	270	270	270	270	270	270
34	531	300	300	300	300	300	300	320	320	320	320	320	320	320	320	320

Appendix 33: Individual Listing of SSG total daily dose

Centre	Patient	Sodium Stibogluconate mg/day (Dose 20mg/kg/day)														
Number	Number	D1	D2	D3	D4	D5	D6	D7	D8	D9	D10	D11	D12	D13	D14	D15
34	533	560	560	560	560	560	560	560	570	570	570	570	570	570	570	580
34	537	220	220	220	220	220	220	220	240	240	240	240	240	240	240	240
34	540	580	580	580	580	580	580	580	600	600	600	600	600	600	600	600
35	650	300	300	300	300	300	300	300	300	300	300	300	300	300	320	320
35	651	320	320	320	320	320	320	320	320	320	320	320	320	320	320	320
35	654	260	260	260	260	260	260	260	260	260	260	260	260	260	260	260
35	656	440	440	440	440	440	440	440	440	440	440	440	440	440	440	440
35	659	320	320	320	320	320	320	320	320	320	320	320	320	320	340	340
35	663	1140	1140	1140	1140	1140	1140	1140	1140	1140	1140	1140	1140	1140	1140	1160
35	665	480	480	480	480	480	480	500	500	500	500	500	500	500	500	500
35	666	660	660	660	660	660	660	660	660	660	660	660	660	660	710	710
35	670	440	440	440	440	440	440	440	440	440	440	440	440	440	500	500
35	673	520	520	520	520	520	520	560	560	560	560	560	560	560	540	540
35	677	1220	1220	1220	1220	1220	1220	1140								
35	679	640	640	640	640	640	640	640	640	640	640	640	640	640	640	640
35	684	980	980	980	980	980	980	940	940	940	940	940	940	940	940	940
35	687	980	980	980	980	980	980	980	980	980	980	980	980	980	1000	1000
35	690	420	420	420	420	420	420	440	440	440	440	440	440	440	500	500

Centre	Patient	Sodium Stibogluconate mg/day (Dose 20mg/kg/day)														
Number	Number	D16	D17	D18	D19	D20	D21	D22	D23	D24	D25	D26	D27	D28	D29	D30
34	533	580	580	580	580	580	580	580	580	580	580	580	580	580	580	580
34	537	240	240	240	240	240	240	240	250	250	250	250	250	250	250	250
34	540	600	600	600	600	600	600	630	630	630	630	630	630	630	630	630
35	650	320	320	320	320	320	320	320	320	320	320	320	320	320	320	320
35	651	320	320	320	320	320	340	340	340	340	340	340	340	340	340	340
35	654	260	260	260	260	260	260	260	260	260	260	260	260	260	260	260
35	656	440	440	440	440	440	440	440	440	440	440	440	440	440	440	440
35	659	340	340	340	340	340	320	320	320	320	320	320	320	320	320	320
35	663	1160	1160	1160	1160	1160	1160	1140	1140	1140	1140	1140	1140	1140	1140	1140
35	665	500	500	500	500	500	560	560	560	560	560	560	560	560	560	560
35	666	710	710	710	710	710	720	720	720	720	720	720	720	720	720	720
35	670	500	500	500	500	500	500	500	500	500	500	500	500	500	500	500
35	673	540	540	540	540	540	560	560	560	560	560	560	560	560	560	560
35	677															
35	679	640	640	640	640	640	640	640	640	640	640	640	640	640	640	640
35	684	940	940	940	940	940	940	940	940	940	940	940	940	940	940	940
35	687	1000	1000	1000	1000	1000	1020	1020	1020	1020	1020	1020	1020	1020	1020	1020
35	690	500	500	500	500	500	520	520	520	520	520	520	520	520	520	520

Centre	Patient	Combination, Paromomycin mg/day (Dose 15mg/kg/day)																
Number	Number	D1	D2	D3	D4	D5	D6	D7	D8	D9	D10	D11	D12	D13	D14	D15	D16	D17
11	2	788	788	788	788	788	788	788	788	788	788	788	788	788	788	788	788	788
11	3	630	630	630	630	630	630	630	630	630	630	630	660	660	660	630	630	630
11	6	275	275	275	275	275	375	375	420	420	420	420	420	420	420	420	420	420
11	11	315	315	315	315	315	315	315	315	315	315	315	315	315	315	300	300	300
11	12	840	840	840	840	840	840	840	825	825	825	825	825	825	825	780	780	780
11	16	675	675	675	675	675	675	675	675	675	675	675	675	675	675	690	690	690
11	17	870	870	870	870	870	870	870	855	855	855	855	855	855	855	885	885	885
11	19	795	795	795	795	795	795	795	795	795	795	795	795	795	795	780	780	780
11	26	300	300	300	300	300	300	300	300	300	300	300	300	300	300	300	300	300
11	28	435	435	435	435	435	435	435	435	435	435	435	435	435	435	420	420	420
11	32	570	570	570	570	570	570	570	570	570	570	570	570	570	570	570	570	570
11	37	885	885	885	885	885	885	885	855	855	855	855	855	855	855	855	855	855
11	41	235	235	235	235	235	235	235	235	235	235	235	235	235	235	235	235	235
11	42	795	795	795	795	795	795	795	795	810	810	810	810	810	810	810	810	810
11	45	750	750	750	750	750	750	750	765	765	765	765	765	765	765	780	780	780
11	48	600	600	600	600	600	600	600	600	600	600	600	600	600	600	600	630	630
11	51	480	480	480	480	480	480	480	495	495	495	495	495	495	495	480	480	680
11	57	705	705	705	705	705	705	705	675	675	675	675	675	675	675	675	675	675
11	58	330	330	330	330	330	330	330	360	360	360	360	360	360	360	360	360	360
11	60	750	750	750	750	750	750	750	735	735	735	735	735	735	735	735	735	735
11	66	510	510	510	510	510	510	510	480	480	480	480	480	480	480	465	465	465
11	69	615	615	615	615	615	615	615	645	645	645	645	645	645	645	645	645	645
11	70	735	735	735	735	735	735	735	720	720	720	720	720	720	720	720	720	720
11	71	690	690	690	690	690	690	690	720	720	720	720	720	720	720	720	720	720
11	75	780	780	780	780	780	780	780	795	795	795	795	795	795	795	795	795	795
11	81	795	795	795	795	795	795	795	795	795	795	795	795	795	795	825	825	825
11	86	165	165	165	165	165	165	165	195	195	195	195	195	195	195	195	195	195
11	87	525	525	525	525	525	525	525	555	555	555	555	555	555	555	570	570	570
11	89	885	885	885	885	885	885	885	885	885	885	885	885	885	885	825	825	825
11	90	210	210	210	210	210	210	210	255	255	255	255	355	355	255	240	240	240
11	92	240	240	240	240	240	240	240	255	255	255	255	255	255	255	255	255	255
11	96	255	255	255	255	255	255	255	255	255	255	255	255	255	255	300	300	300
11	97	555	555	555	555	555	555	555	570	570	570	570	570	570	570	615	615	615
11	102	765	765	765	765	765	765	765	765	765	765	765	765	765	765	765	765	765
11	103	765	765	765	765	765	765	765	750	750	750	750	750	750	750	765	765	765
11	106	750	750	750	750	750	750	750	735	735	735	735	735	735	735	750	750	750
11	111	705	705	705	705	705	705	705	705	735	735	735	735	735	735	765	765	765
11	112	690	690	690	690	690	690	690	660	660	660	660	660	660	660	705	705	705
11	115	390	390	390	390	390	390	390	420	420	420	420	420	420	420	420	420	420
11	120	690	690	690	690	690	690	690	705	705	705	705	705	705	705	720	720	720
11	121	225	225	225	225	225	225	225	225	225	225	225	225	225	225	225	225	225
11	126	780	780	780	780	780	780	780	780	780	780	780	780	780	780	780	780	780
11	128	780	780	780	780	780	780	780	795	795	795	795	795	795	795	765	765	765
11	130	705	705	705	705	705	705	705	660	660	660	660	660	660	660	675	675	675
11	133	720	720	720	720	720	720	720	720	720	720	720	720	720	720	720	720	720
12	241	630	630	630	630	630	630	630	660	660	660	660	660	660	660	660	660	660
12	249	525	525	525	525	525	525	525	525	525	525	525	525	525	525	490	490	490
12	251	690	690	690	690	690	690	690	690	690	690	690	690	690	690	690	690	690
12	254	300	300	300	300	300	300	300	285	285	285	285	285	285	285	300	300	300
12	255	570	570	570	570	570	570	570	555	555	555	555	555	555	555	555	555	555
12	256	600	600	600	600	600	600	600	600	600	600	600	600	600	600	600	600	600
12	261	525	525	525	525	525	525	525	525	525	525	525	525	525	525	525	525	525
12	264	360	360	360	360	360	360	360	375	375	375	375	375	375	375	375	375	375
12	269	615	615	615	615	615	615	615	600	600	600	600	600	600	600	630	630	630

Centre	Patient	Combination, Paromomycin mg/day (Dose 15mg/kg/day)																
Number	Number	D1	D2	D3	D4	D5	D6	D7	D8	D9	D10	D11	D12	D13	D14	D15	D16	D17
12	270	330	330	330	330	330	330	330	345	345	345	345	345	345	345	345	345	345
12	275	570	570	570	570	570	570	570	585	585	585	585	585	585	585	570	570	570
12	277	660	660	660	660	660	660	660	675	675	675	675	675	675	675	675	675	675
12	281	675	675	675	675	675	675	675	690	690	690	690	690	690	690	675	675	675
12	284	210	210	210	210	210	210	210	240	240	240	240	240	240	240	240	240	240
12	285	570	570	570	570	570	570	570	570	570	570	570	570	570	570	615	615	615
12	288	330	330	330	330	330	330	330	330	330	330	330	330	330	330	330	330	330
12	289	750	750	750	750	750	750	750	750	750	750	750	750	750	750	750	750	750
12	294	380	285	285	285	285	285	285	270	270	270	270	270	270	270	285	285	285
12	295	375	375	375	375	375	375	375	375	375	375	375	375	375	405	405	405	405
12	299	840	840	840	840	840	840	840	840	840	840	840	840	840	840	840	840	840
12	304	630	630	630	630	630	630	630	645	645	645	645	645	645	645	645	645	645
12	306	225	225	225	225	225	225	225	210	210	260	210	210	210	210	210	210	210
12	307	915	915	915	915	915	915	915	900	900	900	900	900	900	900	975	975	975
12	312	720	720	720	720	720	720	720	720	720	720	720	720	720	720	750	750	750
12	315	270	270	270	270	270	270	270	300	300	300	300	300	300	300	330	330	330
12	318	240	240	240	240	240	240	240	255	255	255	255	255	255	255	240	240	240
12	319	345	345	345	345	345	345	345	360	360	360	360	360	360	360	360	360	360
12	320	330	330	330	330	330	330	330	330	330	330	330	330	330	330	330	330	330
12	321	315	315	315	315	315	315	315	315	315	315	315	315	315	315	315	315	315
12	327	450	450	450	450	450	450	450	480	480	480	480	480	480	480	495	495	495
23	361	250	250	250	250	250	250	250	250	250	250	250	250	250	250	250	250	250
23	364	350	350	350	350	350	350	350	350	350	350	350	350	350	350	350	350	350
23	366	400	400	400	400	400	400	400	400	400	400	400	400	400	450	450	450	450
23	369	400	400	400	400	400	400	400	400	400	400	400	400	400	400	400	400	400
23	375	350	350	350	350	350	350	350	350	350	350	350	350	350	350	350	350	350
23	377	700	700	700	700	700	700	700	700	700								
23	378	250	250	250	250	250	250	250	250	250	250	250	250	250	250	250	250	250
23	382	600	600	600	600	600	600	600	600	600	600	600	600	600	600	600	600	600
23	386	500	500	500	500	500	500	500	500	500	500	500	500	500	500	500	500	500
23	387	650	650	650	650	650	650	650	650	650	650	650	650	650	650	650	650	650
23	391	450	450	450	450	450	450	450	450	450	450	450	450	450	450	450	450	450
23	392	250	250	250	250	250	250	250	250						250	250	250	
23	398	300	300	300	300	300	300	300	300	300	300	300	300	300	300	300	300	300
23	401	850	850	850	850	850	850	850	850	850	850	850	850	850	850	850	850	850
23	402	650	650	650	650	650	650	650	650	650	650	650	650	650	650	650	650	650
34	451	250	250	250	250	250	250	250	250	250	250	250	250	250	250	250	250	250
34	454	150	150	150	150	150	150	150	200	200	200	200	200	200	200	200	200	200
34	457	700	700	700	700	700	700	700	800	800	800	800	800	800	800	800	800	800
34	459	350	350	350	350	350	350	350	400	400	400	400	400	400	400	350	350	350
34	463	150	150	150	150	150	150	150	150	150	150	150	150	150	150	150	150	150
34	466	300	300	300	300	300	300	300	300	300	300	300	300	300	300	300	300	300
34	468	700	700	700	700	700	700	700	700	700	700	700	700	700	700	750	750	750
34	470	200	200	200	200	200	200	200	200	200	200	200	200	200	200	250	250	250
34	474	350	350	350	350	350	350	350	350	350	350	350	350	350	350	400	400	400
34	478	150	150	150	150	150	150	150	150	150	150	150	150	150	150	200	200	200
34	481	350	350	350	350	350	350	350	350	350	350	350	350	350	350	400	400	400
34	482	800	800	800	800	800	800	800	850	850	850	850	850	850	850	850	850	850
34	485	250	250	250	250	250	250	250	250	250	250	250	250	250	250	250	250	250
34	486	450	450	450	450	450	450	450	500	500	500	500	500	500	500	500	500	500
34	494	850	850	850	850	850	850	850	750	750	750	750	750	750	750	750	750	750
34	499	400	400	400	400	400	400	400	400	400	400	400	400	400	400	400	400	400
34	501	350	350	350	350	350	350	350	350	350	350	350	350	350	350	350	350	350
34	502	550	550	550	550	550	550	550	500	500	500	500	500	500	500	500	500	500

Centre Number	Patient Number	Combination, Paromomycin mg/day (Dose 15mg/kg/day)																
		D1	D2	D3	D4	D5	D6	D7	D8	D9	D10	D11	D12	D13	D14	D15	D16	D17
34	504	250	250	250	250	250	250	250	250	250	250	250	250	250	250	250	250	250
34	510	300	300	300	300	300	300	300	300	300	300	300	300	300	300	350	350	350
34	511	250	250	250	250	250	250	250	250	250	250	250	250	250	250	250	250	250
34	513	300	300	300	300	300	300	300	350	350	350	350	350	350	350	350	350	350
34	522	700	700	700	700	700	700	700	750	750	750	750	750	750	750	800	800	800
34	524	500	500	500	500	500	500	500	500	500	500	500	500	500	500	500	500	500
34	525	350	350	350	350	350	350	350	350	350	350	350	350	350	350	350	350	350
34	527	700	700	700	700	700	700	700	700	700	700	700	700	700	700	650	650	650
34	529	200	200	200	200	200	200	200	250	250	250	250	250	250	250	250	250	250
34	530	800	800	800	800	800	800	800	850	850	850	850	850	850	850	850	850	850
34	532	200	200	200	200	200	200	200	200	200	200	200	200	200	200	200	200	200
34	539	300	300	300	300	300	300	300	300	300	300	300	300	300	300	350	350	350
35	648	225	225	225	225	225	225	225	225	225	225	225	225	225	225	225	225	225
35	649	435	435	435	435	435	435	480	480	480	480	480	480	480	480	480	480	480
35	653	225	225	225	225	225	225	225	225	225	225	225	225	225	225	225	225	225
35	655	435	435	435	435	435	435	435	435	435	435	435	435	435	435	435	435	435
35	658	550	550	550	550	550	550	555	555	555	555	555	555	555	585	585	585	585
35	661	750	750	750	750	750	750	750	750	750	750	750	750	750	750	765	765	765
35	664	1095	1095	1095	1095	1095	1095	1050										
35	667	345	345	345	345	345	345	345	375	375	375	375	375	375	375	375	375	375
35	672	345	345	345	345	345	345	345	345	345	345	345	345	345	345	345	345	345
35	674	300	300	300	300	300	300	300	300	300	300	300	300	300	300	300	300	300
35	678	465	465	465	465	465	465	465	465	465	465	465	465	465	480	480	480	480
35	680	330	330	330	330	330	330	330	330	330	330	330	330	330	360	360	360	360
35	681	690	690	690	690	690	690	690	690	690	690	690	690	690	720	720	720	720
35	683	450	450	450	450	450	450	450	450	450	450	450	450	450	450	450	450	450
35	689	630	630	630	630	630	630	630	630	630	630	630	630	630	645	645	645	645

Appendix 35: Individual listing for combination patients for SSG total daily dose

Centre	Patient	Combination, SSG mg/day (Dose 20mg/kg/day)																
Number	Number	D1	D2	D3	D4	D5	D6	D7	D8	D9	D10	D11	D12	D13	D14	D15	D16	D17
11	2	850	850	850	850	850	850	850	850	850	850	850	850	850	850	850	850	850
11	3	840	840	840	840	840	840	840	840	840	840	840	840	840	840	840	840	840
11	6	500	500	500	500	500	500	500	560	560	560	560	560	560	560	560	560	560
11	11	420	420	420	420	420	420	420	420	420	420	420	420	420	420	400	400	400
11	12	850	850	850	850	850	850	850	850	850	850	850	850	850	850	850	850	850
11	16	850	850	850	850	850	850	850	850	850	850	850	850	850	850	850	850	850
11	17	850	850	850	850	850	850	850	850	850	850	850	850	850	850	850	850	850
11	19	850	850	850	850	850	850	850	850	850	850	850	850	850	850	850	850	850
11	26	400	400	400	400	400	400	400	400	400	400	400	400	400	400	400	400	400
11	28	580	580	580	580	580	580	580	580	580	580	580	580	580	580	560	560	560
11	32	760	760	760	760	760	760	760	760	760	760	760	760	760	760	760	760	760
11	37	850	850	850	850	850	850	850	850	850	850	850	850	850	850	850	850	850
11	41	340	340	340	340	340	340	340	340	340	340	340	340	340	340	340	340	340
11	42	850	850	850	850	850	850	850	850	850	850	850	850	850	850	850	850	850
11	45	850	850	850	850	850	850	850	850	850	850	850	850	850	850	850	850	850
11	48	800	800	800	800	800	800	800	800	800	800	800	800	800	800	800	840	840
11	51	640	640	640	640	640	640	640	660	660	660	660	660	660	660	640	640	640
11	57	850	850	850	850	850	850	850	850	850	850	850	850	850	850	850	850	850
11	58	440	440	440	440	440	440	440	480	480	480	480	480	480	480	480	480	480
11	60	850	850	850	850	850	850	850	850	850	850	850	850	850	850	850	850	850
11	66	680	680	680	680	680	680	680	640	640	640	640	640	640	640	620	620	620
11	69	820	820	820	820	820	820	820	850	850	850	850	850	850	850	850	850	850
11	70	850	850	850	850	850	850	850	850	850	850	850	850	850	850	850	850	850
11	71	850	850	850	850	850	850	850	850	850	850	850	850	850	850	850	850	850
11	75	850	850	850	850	850	850	850	850	850	850	850	850	850	850	850	850	850
11	81	850	850	850	850	850	850	850	850	850	850	850	850	850	850	850	850	850
11	86	220	220	220	220	220	220	220	260	260	260	260	260	260	260	260	260	260
11	87	700	700	700	700	700	700	700	740	740	740	740	740	740	740	760	760	760
11	89	850	850	850	850	850	850	850	850	850	850	850	850	850	850	850	850	850
11	90	280	280	280	280	280	280	280	340	340	340	340	340	340	340	320	320	320
11	92	320	320	320	320	320	320	320	340	340	340	340	340	340	340	340	340	340
11	96	340	340	340	340	340	340	340	340	340	340	340	340	340	340	400	400	400
11	97	740	740	740	740	740	740	740	760	760	760	760	760	760	760	820	820	820
11	102	850	850	850	850	850	850	850	850	850	850	850	850	850	850	850	850	850
11	103	850	850	850	850	850	850	850	850	850	850	850	850	850	850	850	850	850
11	106	850	850	850	850	850	850	850	850	850	850	850	850	850	850	850	850	850
11	111	850	850	850	850	850	850	850	850	850	850	850	850	850	850	850	850	850
11	112	850	850	850	850	850	850	850	850	850	850	850	850	850	850	850	850	850
11	115	520	520	520	520	520	520	520	560	560	560	560	560	560	560	560	560	560
11	120	850	850	850	850	850	850	850	850	850	850	850	850	850	850	850	850	850
11	121	300	300	300	300	300	300	300	300	300	300	300	300	300	300	300	300	300
11	126	850	850	850	850	850	850	850	850	850	850	850	850	850	850	850	850	850
11	128	850	850	850	850	850	850	850	850	850	850	850	850	850	850	850	850	850
11	130	850	850	850	850	850	850	850	850	850	850	850	850	850	850	850	850	850
11	133	850	850	850	850	850	850	850	850	850	850	850	850	850	850	850	850	850
12	241	840	840	840	840	840	840	840	850	850	850	850	850	850	850	850	850	850
12	249	700	700	700	700	700	700	700	700	700	700	700	700	700	700	660	660	660
12	251	850	850	850	850	850	850	850	850	850	850	850	850	850	850	850	850	850
12	254	400	400	400	400	400	400	400	380	380	380	380	380	380	380	400	400	400
12	255	760	760	760	760	760	760	760	740	740	740	740	740	740	740	740	740	740
12	256	800	800	800	800	800	800	800	800	800	800	800	800	800	800	800	800	800
12	261	700	700	700	700	700	700	700	700	700	700	700	700	700	700	700	700	700
12	264	480	480	480	480	480	480	480	500	500	500	500	500	500	500	500	500	500
12	269	820	820	820	820	820	820	820	800	800	800	800	800	800	800	840	840	840

Centre	Patient	Combination, SSG mg/day (Dose 20mg/kg/day)																
Number	Number	D1	D2	D3	D4	D5	D6	D7	D8	D9	D10	D11	D12	D13	D14	D15	D16	D17
12	270	440	440	440	440	440	440	440	460	460	460	460	460	460	460	460	460	460
12	275	760	760	760	760	760	760	760	780	780	780	780	780	780	780	760	760	760
12	277	850	850	850	850	850	850	850	850	850	850	850	850	850	850	850	850	850
12	281	850	850	850	850	850	850	850	850	850	850	850	850	850	850	850	850	850
12	284	280	280	280	280	280	280	280	320	320	320	320	320	320	320	320	320	320
12	285	760	760	760	760	760	760	760	760	760	760	760	760	760	760	820	820	820
12	288	440	440	440	440	440	440	440	440	440	440	440	440	440	440	440	440	440
12	289	850	850	850	850	850	850	850	850	850	850	850	850	850	850	850	850	850
12	294	285	380	380	380	380	380	380	360	360	360	360	360	360	360	380	380	380
12	295	500	500	500	500	500	500	500	500	500	500	500	500	500	540	540	540	540
12	299	850	850	850	850	850	850	850	850	850	850	850	850	850	850	850	850	850
12	304	840	840	840	840	840	840	840	850	850	850	850	850	850	850	850	850	850
12	306	300	300	300	300	300	300	300	280	280	280	280	280	280	280	290	290	290
12	307	850	850	850	850	850	850	850	850	850	850	850	850	850	850	850	850	850
12	312	850	850	850	850	850	850	850	850	850	850	85	850	850	850	850	850	850
12	315	360	360	360	360	360	360	360	400	400	400	400	400	400	400	440	440	440
12	318	320	320	320	320	320	320	320	340	340	340	340	340	340	340	320	320	320
12	319	460	460	460	460	460	460	460	480	480	480	480	480	480	480	480	480	480
12	320	440	440	440	440	440	440	440	440	440	440	440	440	440	440	440	440	440
12	321	420	420	420	420	420	420	420	420	420	420	420	420	420	420	420	420	420
12	327	600	600	600	600	600	600	600	640	640	640	640	640	640	640	660	660	660
23	361	350	350	350	350	350	350	350	350	350	350	350	350	350	350	350	350	350
23	364	450	450	450	450	450	450	450	450	450	450	450	450	450	450	450	450	450
23	366	560	560	560	560	560	560	560	560	560	560	560	560	560	600	600	600	600
23	369	540	540	540	540	540	540	540	540	540	540	540	540	540	540	540	540	540
23	375	460	460	460	460	460	460	460	460	460	460	460	460	460	460	460	460	460
23	377	850	850	850	850	850	850	850	850	850								
23	378	340	340	340	340	340	340	340	340	340	340	340	340	340	340	340	340	340
23	382	800	800	800	800	800	800	800	800	800	800	800	800	800	800	800	800	800
23	386	620	620	620	620	620	620	620	620	640	640	640	640	640	660	660	660	660
23	387	850	850	850	850	850	850	850	850	850	850	850	850	850	850	850	850	850
23	391	580	580	580	580	580	580	580	580	580	580	580	580	580	620	620	620	620
23	392	340	340	340	340	340	340	340	340						360	360	360	
23	398	430	430	430	430	430	430	430	430	430	430	430	430	430	430	430	430	430
23	401	850	850	850	850	850	850	850	850	850	850	850	850	850	850	850	850	850
23	402	850	850	850	850	850	850	850	850	850	850	850	850	850	850	850	850	850
34	451	320	320	320	320	320	320	320	360	360	360	360	360	360	360	350	350	350
34	454	230	230	230	230	230	230	230	240	240	240	240	240	240	240	240	240	240
34	457	960	960	960	960	960	960	960	1100	1100	1100	1100	1100	1100	1100	1100	1100	1100
34	459	480	480	480	480	480	480	480	500	500	500	500	500	500	500	480	480	480
34	463	210	210	210	210	210	210	210	210	210	210	210	210	210	210	210	220	220
34	466	400	400	400	400	400	400	400	420	420	420	420	420	420	420	420	420	420
34	468	920	920	920	920	920	920	920	960	960	960	960	960	960	960	980	980	980
34	470	280	280	280	280	280	280	280	280	280	280	280	280	280	280	320	320	320
34	474	480	480	480	480	480	480	480	510	510	510	510	510	510	510	520	520	520
34	478	200	200	200	200	200	200	200	220	220	220	220	220	220	220	240	240	240
34	481	460	460	460	460	460	460	460	460	460	460	460	460	460	460	500	500	500
34	482	1100	1100	1100	1100	1100	1100	1100	1130	1130	1130	1130	1130	1130	1130	1120	1120	1120
34	485	300	300	300	300	300	300	300	300	300	300	300	300	300	300	320	320	320
34	486	640	640	640	640	640	640	640	660	660	660	660	660	660	660	680	680	680
34	494	1160	1160	1160	1160	1160	1160	1160	980	980	980	980	980	980	980	980	980	980
34	499	520	520	520	520	520	520	520	520	520	520	520	520	520	520	560	560	560
34	501	440	440	440	440	440	440	440	460	460	460	460	460	460	460	480	480	480
34	502	700	700	700	700	700	700	700	680	680	680	680	680	680	680	680	680	680

Appendix 35: Individual listing for combination patients for SSG total daily dose

Centre	Patient	Combination, SSG mg/day (Dose 20mg/kg/day)																
Number	Number	D1	D2	D3	D4	D5	D6	D7	D8	D9	D10	D11	D12	D13	D14	D15	D16	D17
34	504	320	320	320	320	320	320	320	300	300	300	300	300	300	300	340	340	340
34	510	380	380	380	380	380	380	380	400	400	400	400	400	400	400	440	440	440
34	511	320	320	320	320	320	320	320	320	320	320	320	320	320	320	360	360	360
34	513	420	420	420	420	420	420	420	460	460	460	460	460	460	460	480	480	480
34	522	960	960	960	960	960	960	960	1020	1020	1020	1020	1020	1020	1020	1050	1050	1050
34	524	660	660	660	660	660	660	660	680	680	680	680	680	680	680	660	660	660
34	525	440	440	440	440	440	440	440	440	440	440	440	440	440	440	440	440	440
34	527	940	940	940	940	940	940	940	920	920	920	920	920	920	920	880	880	880
34	529	260	260	260	260	260	260	260	300	300	300	300	300	300	300	300	300	300
34	530	1060	1060	1060	1060	1060	1060	1060	1100	1100	1100	1100	1100	1100	1100	1130	1130	1130
34	532	240	240	240	240	240	240	240	240	260	260	260	260	260	260	260	260	260
34	539	420	420	420	420	420	420	420	420	420	420	420	420	420	420	440	440	440
35	648	300	300	300	300	300	300	300	300	300	300	300	300	300	300	300	300	300
35	649	580	580	580	580	580	580	640	640	640	640	640	640	640	640	640	640	640
35	653	300	300	300	300	300	300	300	300	300	300	300	300	300	300	300	300	300
35	655	580	580	580	580	580	580	580	580	580	580	580	580	580	580	580	580	580
35	658	720	720	720	720	720	720	740	740	740	740	740	740	740	780	780	780	780
35	661	1000	1000	1000	1000	1000	1000	1000	1000	1000	1000	1000	1000	1000	1000	1020	1020	1020
35	664	1460	1460	1460	1460	1460	1460	1400										
35	667	460	460	460	460	460	460	460	500	500	500	500	500	500	500	500	500	500
35	672	460	460	460	460	460	460	460	460	460	460	460	460	460	460	460	460	460
35	674	400	400	400	400	400	400	400	400	400	400	400	400	400	400	400	400	400
35	678	620	620	620	620	620	620	620	620	620	620	620	620	620	640	640	640	640
35	680	440	440	440	440	440	440	440	440	440	440	440	440	440	480	480	480	480
35	681	920	920	920	920	920	920	920	920	920	920	920	920	920	960	960	960	960
35	683	600	600	600	600	600	600	600	600	600	600	600	600	600	600	600	600	600
35	689	840	840	840	840	840	840	840	840	840	840	840	840	840	860	860	860	860

Centre Number	Patient Number	Treatment	Treatment Start date	Rescue Medication - Ambisome Dose	Frequency	Start date	End Date
11	7	PM	2-Jul-05	150MG		26-Oct-05	31-Oct-05
11	10	SSG	3-Jul-05	110		6-Sep-05	15-Sep-05
11	10	SSG	3-Jul-05	110 MG		6-Sep-05	7-Sep-05
11	10	SSG	3-Jul-05	110 MG		8-Sep-05	11-Sep-05
11	15	PM	14-Jul-05	140MG		29-Jul-05	8-Aug-05
11	21	SSG	16-Aug-05	147MG		15-Oct-05	20-Oct-05
11	21	SSG	16-Aug-05	147MG		15-Oct-05	20-Oct-05
11	27	PM	19-Sep-05	123MG		3-Feb-06	12-Feb-06
11	34	PM	27-Sep-05	150MG		25-Nov-05	11-Dec-05
11	40	PM	20-Oct-05	47.5MG		12-Nov-05	25-Nov-05
11	50	PM	18-May-06	165 MG		17-Jun-06	26-Jun-06
11	50	PM	18-May-06	165 MG		8-Jun-06	18-Jun-06
11	54	PM	27-May-06	147 MG		20-Jun-06	29-Jun-06
11	54	PM	27-May-06	153 MG		27-Sep-06	6-Oct-06
11	54	PM	27-May-06	156 MG		30-Dec-06	8-Jan-07
11	55	PM	30-May-06	141 MG		21-Jun-06	30-Jun-06
11	55	PM	30-May-06	141 MG		21-Jun-06	30-Jun-06
11	71	Combination	22-Jul-06	141		11-Aug-06	20-Aug-06
11	71	Combination	22-Jul-06	150 MG		30-Dec-06	8-Jan-07
11	71	Combination	22-Jul-06	150 MG		30-Dec-06	9-Jan-07
11	72	SSG	22-Jul-06	141 MG		16-Aug-06	25-Aug-06
11	75	Combination	28-Jul-06	180 MG		30-Dec-06	8-Jan-07
11	91	PM	18-Dec-06	165 MG	O.D	5-Mar-07	14-Mar-07
11	91	PM	18-Dec-06	162 MG	O.D	26-Jun-07	5-Jul-07
11	104	PM	10-Feb-07	90 MG	OD	5-Mar-07	14-Mar-07
11	126	Combination	28-May-07	SSG 15MG		21-Nov-07	7-Dec-07
11	126	Combination	28-May-07	6 DOSES		21-Nov-07	26-Nov-07
11	128	Combination	2-Jun-07	AMBISOME		8-Dec-07	17-Dec-07
12	245	PM	20-Jul-05	150MG	OD	25-Nov-05	4-Dec-05
12	284	Combination	20-May-06	60MG	DAILY	7-Dec-06	11-Dec-06
12	284	Combination	20-May-06	60MG	/DAY	16-Dec-06	16-Dec-06
12	297	SSG	12-Apr-07	144MG	DAILY	4-May-07	8-May-07
12	297	SSG	12-Apr-07	144MG	ONCE	13-May-07	
12	315	Combination	29-Aug-07	63MG	DAILY	11-Jan-08	20-Jan-08
23	364	Combination	20-Jan-05	48MG	OD	28-Apr-05	4-May-05
23	378	Combination	9-Mar-05	34.4	OD	7-Jul-05	13-Jul-05
23	379	PM	10-Mar-05	38MG	O.D	30-Jun-05	6-Jul-05
23	395	PM	29-Apr-05	120MG	OD	26-May-05	1-Jun-05
23	398	Combination	18-May-05	48MG	OD	15-Dec-05	21-Dec-05
23	400	PM	3-Jun-05	40 MG	O.D	25-Jun-05	1-Jul-05
23	401	Combination	3-Jun-05	112MG	OD	21-Sep-05	27-Sep-05
34	452	PM	17-Nov-04	145 MG	ONCE	8-Dec-04	
34	452	PM	17-Nov-04	145 MG	ONCE	9-Dec-04	
34	452	PM	17-Nov-04	145 MG		10-Dec-04	
34	452	PM	17-Nov-04	145 MG		12-Dec-04	
34	452	PM	17-Nov-04	145 MG		17-Dec-04	
34	452	PM	17-Nov-04	145 MG		22-Dec-04	
34	453	PM	18-Nov-04	80 MG	ONCE	9-Dec-04	
34	453	PM	18-Nov-04	80 MG	ONCE	10-Dec-04	
34	453	PM	18-Nov-04	80 MG		11-Dec-04	
34	453	PM	18-Nov-04	80 MG		13-Dec-04	
34	453	PM	18-Nov-04	90 MG		18-Dec-04	
34	453	PM	18-Nov-04	90 MG		23-Dec-04	
34	455	PM	19-Nov-04	112.5 MG	ONCE	10-Dec-04	

Centre Number	Patient Number	Treatment	Treatment Start date	Dose	Rescue Medication - Ambisome Frequency	Start date	End Date
34	455	PM	19-Nov-04	112.5 MG	ONCE	11-Dec-04	
34	455	PM	19-Nov-04	112.5 MG		12-Dec-04	
34	455	PM	19-Nov-04	112.5 MG		14-Dec-04	
34	455	PM	19-Nov-04	120 MG		19-Dec-04	
34	456	SSG	19-Nov-04	115 MG	ONCE	27-Jan-05	
34	456	SSG	19-Nov-04	115 MG		28-Jan-05	
34	456	SSG	19-Nov-04	115 MG		29-Jan-05	
34	456	SSG	19-Nov-04	115 MG		31-Jan-05	
34	456	SSG	19-Nov-04	115 MG		5-Feb-05	
34	456	SSG	19-Nov-04	115 MG			
34	458	PM	20-Nov-04	135 MG	ONCE	16-Jan-05	
34	458	PM	20-Nov-04	135 MG		17-Jan-05	
34	458	PM	20-Nov-04	135 MG		18-Jan-05	
34	458	PM	20-Nov-04	135 MG		20-Jan-05	
34	458	PM	20-Nov-04	135 MG		25-Jan-05	
34	458	PM	20-Nov-04	135 MG			
34	460	PM	24-Nov-04	65 MG	ONCE	15-Dec-04	
34	460	PM	24-Nov-04	65 MG	ONCE	16-Dec-04	
34	460	PM	24-Nov-04	65 MG		17-Dec-04	
34	460	PM	24-Nov-04	65 MG		19-Dec-04	
34	460	PM	24-Nov-04	75 MG		24-Dec-04	
34	460	PM	24-Nov-04	75 MG		29-Dec-04	
34	466	Combination	30-Nov-04	110 MG	ONCE	18-Dec-04	
34	466	Combination	30-Nov-04	110 MG	ONCE	19-Dec-04	
34	466	Combination	30-Nov-04	110 MG		20-Dec-04	
34	466	Combination	30-Nov-04	110 MG		22-Dec-04	
34	466	Combination	30-Nov-04	110 MG		27-Dec-04	
34	466	Combination	30-Nov-04	100 MG		1-Jan-05	
34	467	PM	29-Nov-04	150 MG	ONCE	5-Feb-05	
34	467	PM	29-Nov-04	150 MG		6-Feb-05	
34	467	PM	29-Nov-04	150 MG		7-Feb-05	
34	467	PM	29-Nov-04	150 MG		9-Feb-05	
34	467	PM	29-Nov-04	150 MG		14-Feb-05	
34	467	PM	29-Nov-04	150 MG			
34	471	PM	29-Nov-04	160 MG	ONCE	20-Dec-04	
34	471	PM	29-Nov-04	160 MG	ONCE	21-Dec-04	
34	471	PM	29-Nov-04	160 MG		22-Dec-04	
34	471	PM	29-Nov-04	160 MG		24-Dec-04	
34	471	PM	29-Nov-04	160 MG		29-Dec-04	
34	471	PM	29-Nov-04	160 MG		3-Jan-05	
34	473	PM	3-Dec-04	75 MG	ONCE	24-Dec-04	
34	473	PM	3-Dec-04	75 MG	ONCE	25-Dec-04	
34	473	PM	3-Dec-04	75 MG		26-Dec-04	
34	473	PM	3-Dec-04	75 MG		28-Dec-04	
34	473	PM	3-Dec-04	75 MG		2-Jan-05	
34	473	PM	3-Dec-04	75 MG		7-Jan-05	
34	475	PM	5-Dec-04	90 MG	ONCE	26-Dec-04	
34	475	PM	5-Dec-04	90 MG	ONCE	27-Dec-04	
34	475	PM	5-Dec-04	90 MG		28-Dec-04	
34	475	PM	5-Dec-04	90 MG		30-Dec-04	
34	475	PM	5-Dec-04	90 MG		4-Jan-05	
34	475	PM	5-Dec-04	100 MG		9-Jan-05	
34	476	PM	5-Dec-04	135 MG	ONCE	10-Jan-05	
34	476	PM	5-Dec-04	135 MG	ONCE	11-Jan-05	

Centre Number	Patient Number	Treatment	Treatment Start date	Dose	Rescue Medication - Ambisome Frequency	Start date	End Date
34	476	PM	5-Dec-04	135 MG		12-Jan-05	
34	476	PM	5-Dec-04	135 MG		14-Jan-05	
34	476	PM	5-Dec-04	135 MG		19-Jan-05	
34	476	PM	5-Dec-04	135 MG		24-Jan-05	
34	476	PM	5-Dec-04	135 MG	ONCE	10-Jan-05	
34	476	PM	5-Dec-04	135 MG		11-Jan-05	
34	476	PM	5-Dec-04	135 MG		12-Jan-05	
34	476	PM	5-Dec-04	135 MG		14-Jan-05	
34	476	PM	5-Dec-04	135 MG		19-Jan-05	
34	476	PM	5-Dec-04	135 MG			
34	484	PM	12-Dec-04	65 MG	ONCE	19-Dec-04	
34	484	PM	12-Dec-04	65 MG	ONCE	20-Dec-04	
34	484	PM	12-Dec-04	65 MG		21-Dec-04	
34	484	PM	12-Dec-04	70 MG		23-Dec-04	
34	484	PM	12-Dec-04	70 MG		28-Dec-04	
34	484	PM	12-Dec-04	70 MG		2-Jan-05	
34	491	PM	17-Dec-04	255 MG	ONCE	7-Jan-05	
34	491	PM	17-Dec-04	255 MG	ONCE	8-Jan-05	
34	491	PM	17-Dec-04	255 MG		9-Jan-05	
34	491	PM	17-Dec-04	255 MG		11-Jan-05	
34	491	PM	17-Dec-04	255 MG		16-Jan-05	
34	491	PM	17-Dec-04	255 MG		21-Jan-05	
34	497	PM	21-Dec-04	260 MG	ONCE	12-Feb-05	
34	497	PM	21-Dec-04	260 MG		13-Feb-05	
34	497	PM	21-Dec-04	260 MG		14-Feb-05	
34	497	PM	21-Dec-04	260 MG		16-Feb-05	
34	497	PM	21-Dec-04	260 MG		21-Feb-05	
34	497	PM	21-Dec-04	260 MG			
34	503	PM	25-Dec-04	125 MG	ONCE	16-Jan-05	
34	503	PM	25-Dec-04	125 MG	ONCE	17-Jan-05	
34	503	PM	25-Dec-04	125 MG		18-Jan-05	
34	503	PM	25-Dec-04	125 MG		20-Jan-05	
34	503	PM	25-Dec-04	125 MG		25-Jan-05	
34	503	PM	25-Dec-04	125 MG		30-Jan-05	
34	504	Combination	27-Dec-04	85 MG	ONCE	13-Jan-05	
34	504	Combination	27-Dec-04	85 MG	ONCE	14-Jan-05	
34	504	Combination	27-Dec-04	85 MG		15-Jan-05	
34	504	Combination	27-Dec-04	85 MG		17-Jan-05	
34	504	Combination	27-Dec-04	85 MG		22-Jan-05	
34	504	Combination	27-Dec-04	85 MG		27-Jan-05	
34	508	PM	31-Dec-04	70 MG	ONCE	21-Jan-05	
34	508	PM	31-Dec-04	70 MG	ONCE	22-Jan-05	
34	508	PM	31-Dec-04	70 MG		23-Jan-05	
34	508	PM	31-Dec-04	70 MG		25-Jan-05	
34	508	PM	31-Dec-04	70 MG		30-Jan-05	
34	508	PM	31-Dec-04	70 MG		4-Feb-05	
34	509	PM	2-Jan-05	300 MG	ONCE	23-Jan-05	
34	509	PM	2-Jan-05	300 MG	ONCE	24-Jan-05	
34	509	PM	2-Jan-05	300 MG		25-Jan-05	
34	509	PM	2-Jan-05	300 MG		27-Jan-05	
34	509	PM	2-Jan-05	300 MG		1-Feb-05	
34	509	PM	2-Jan-05	300 MG		6-Feb-05	
34	512	PM	2-Jan-05	80 MG	ONCE	24-Jan-05	
34	512	PM	2-Jan-05	80 MG	ONCE	25-Jan-05	

Centre Number	Patient Number	Treatment	Treatment Start date	Rescue Medication - Ambisome Dose	Frequency	Start date	End Date
34	512	PM	2-Jan-05	80 MG		26-Jan-05	
34	512	PM	2-Jan-05	80 MG		28-Jan-05	
34	512	PM	2-Jan-05	80 MG		2-Feb-05	
34	512	PM	2-Jan-05	80 MG		7-Feb-05	
34	517	PM	6-Jan-05	140 MG	ONCE	28-Jan-05	
34	517	PM	6-Jan-05	140 MG	ONCE	29-Jan-05	
34	517	PM	6-Jan-05	140 MG		30-Jan-05	
34	517	PM	6-Jan-05	140 MG		1-Feb-05	
34	517	PM	6-Jan-05	140 MG		6-Feb-05	
34	517	PM	6-Jan-05	140 MG		11-Feb-05	
34	519	PM	7-Jan-05	100 MG	ONCE DAILY	3-Apr-05	
34	519	PM	7-Jan-05	100 MG		4-Apr-05	
34	519	PM	7-Jan-05	100 MG		5-Apr-05	
34	519	PM	7-Jan-05	100 MG		7-Apr-05	
34	519	PM	7-Jan-05	100 MG		12-Apr-05	
34	519	PM	7-Jan-05	100 MG			
34	521	PM	11-Jan-05	85 MG	ONCE	2-Feb-05	
34	521	PM	11-Jan-05	85 MG	ONCE	3-Feb-05	
34	521	PM	11-Jan-05	85 MG		4-Feb-05	
34	521	PM	11-Jan-05	85 MG		6-Feb-05	
34	521	PM	11-Jan-05	85 MG		11-Feb-05	
34	521	PM	11-Jan-05	85 MG		16-Feb-05	
34	528	PM	17-Jan-05	95 MG	ONCE	11-Mar-05	
34	528	PM	17-Jan-05	95 MG	ONCE	12-Mar-05	
34	528	PM	17-Jan-05	95 MG		13-Mar-05	
34	528	PM	17-Jan-05	95 MG		15-Mar-05	
34	531	SSG	21-Jan-05	75 MG	ONCE	20-Feb-05	
34	531	SSG	21-Jan-05	75 MG	ONCE	21-Feb-05	
34	531	SSG	21-Jan-05	75 MG		22-Feb-05	
34	531	SSG	21-Jan-05	75 MG		24-Feb-05	
34	531	SSG	21-Jan-05	75 MG		1-Mar-05	
34	531	SSG	21-Jan-05	80 MG		6-Mar-05	6-Mar-05
34	534	PM	24-Jan-05	67.5 MG	ONCE	15-Feb-05	
34	534	PM	24-Jan-05	67.5 MG	ONCE	16-Feb-05	
34	534	PM	24-Jan-05	67.5 MG		17-Feb-05	
34	534	PM	24-Jan-05	67.5 MG		19-Feb-05	
34	534	PM	24-Jan-05	67.5 MG		24-Feb-05	
34	534	PM	24-Jan-05	75 MG		1-Mar-05	1-Mar-05
34	535	PM	24-Jan-05	140 MG	ONCE DAILY	25-Mar-05	
34	535	PM	24-Jan-05	140 MG		26-Mar-05	
34	535	PM	24-Jan-05	140 MG		27-Mar-05	
34	535	PM	24-Jan-05	140 MG		29-Mar-05	
34	535	PM	24-Jan-05	140 MG		3-Apr-05	
34	535	PM	24-Jan-05	140 MG			
34	538	PM	26-Jan-05	87.5 MG	ONCE	16-Feb-05	
34	538	PM	26-Jan-05	87.5 MG	ONCE	17-Feb-05	
34	538	PM	26-Jan-05	87.5 MG		18-Feb-05	
34	538	PM	26-Jan-05	87.5 MG		20-Feb-05	
34	538	PM	26-Jan-05	87.5 MG		25-Feb-05	
34	538	PM	26-Jan-05	85 MG		2-Mar-05	2-Mar-05
35	646	PM	28-Jan-05	100 MG		6-Jul-05	15-Jul-05
35	647	PM	30-Jan-05	3MG/KG/D		28-Mar-05	6-Apr-05
35	647	PM	30-Jan-05	175 MG		28-Mar-05	6-Apr-05
35	652	PM	1-Feb-05	60 MG		22-Mar-05	31-Mar-05

Centre	Patient	Treatment	Treatment	Rescue Medication - Ambisome			
Number	Number		Start date	Dose	Frequency	Start date	End Date
35	662	PM	20-Feb-05	3 MG/KG/DAY		12-Apr-05	21-Apr-05
35	662	PM	20-Feb-05	150 MG		12-Apr-05	21-Apr-05
35	664	Combination	20-Feb-05	3 MG/KG		15-Mar-05	19-Mar-05
35	669	PM	28-Feb-05	3 MG/KG		22-Mar-05	31-Mar-05
35	671	PM	11-Mar-05	50 MG		1-May-05	9-May-05
35	675	PM	14-Mar-05	75 MG		4-Jun-05	13-Jun-05
35	677	SSG	20-Mar-05	3 MG/KG		28-Mar-05	29-Mar-05
35	682	PM	22-Mar-05	50 MG		5-Jul-05	14-Jul-05

Centre Number	Patient Number	Treatment	Treatment start date	Concomitant Medication	Diagnosis	Start date	End date
11	3	Combination	19-Jun-05	CIMITHIDINE	DYSPEPSIA	2-Jul-05	7-Jul-05
11	3	Combination	19-Jun-05	ORS	ACUTE GASTROENTERITIS	7-Jul-05	8-Jul-05
11	5	PM	30-Jun-05	VIT A	XEROPHTHALMIA	6-Jul-05	19-Jul-05
11	5	PM	30-Jun-05	DIPYRON	FEVER & CHILLINESS	8-Jul-05	8-Jul-05
11	5	PM	30-Jun-05	METOCLOPRAMID	VOMITING	8-Jul-05	8-Jul-05
11	7	PM	2-Jul-05	NORFLOXECILLIN	UTI	29-Jun-05	11-Jul-05
11	7	PM	2-Jul-05	40% GLUCOSE	HYPOGLUCOMIA	8-Jul-05	8-Jul-05
11	7	PM	2-Jul-05	1000CC N\S	HYPOGLYCOMIA FOR DEXTROSE INFUSION	8-Jul-05	8-Jul-05
11	7	PM	2-Jul-05	PARACETAMOL	INJ. SITE PAIN	13-Jul-05	17-Jul-05
11	7	PM	2-Jul-05	DIPYRON	INJ. SITE PAIN	18-Jul-05	18-Jul-05
11	7	PM	2-Jul-05	SALBUTAMOL	BRONCHIAL ATHMA	25-Oct-05	
11	7	PM	2-Jul-05	COUGH SYRUP	BRONCHIAL ATHMA	25-Oct-05	
11	7	PM	2-Jul-05	NORFLOXACLINE	UTI (URINARY TRACT INFECTION)	23-Jan-06	29-Jan-06
11	9	SSG	3-Jul-05	AL(OH)3 OR MALOX	DYSPEPSIA	6-Jul-05	
11	9	SSG	3-Jul-05	MALOX SYRUP	DYSPEPSIA	31-Jan-06	
11	9	SSG	3-Jul-05	CIMITIDIN	DYSPEPSIA	31-Jan-06	11-Feb-06
11	10	SSG	3-Jul-05	QUININ	MALARIA	17-Jul-05	19-Jul-05
11	10	SSG	3-Jul-05	FANSIDAR	MALARIA	19-Jul-05	19-Jul-05
11	10	SSG	3-Jul-05	PARACETAMOL	FEVER	17-Jul-05	19-Jul-05
11	10	SSG	3-Jul-05	PARAC	HEADACHE	29-Jul-05	29-Jul-05
11	12	Combination	8-Jul-05	QUININE	MALARIA (P.F)	14-Jul-05	20-Jul-05
11	12	Combination	8-Jul-05	PARACETAMOL	HEADACHE	6-Feb-06	
11	14	SSG	12-Jul-05	METRONIDAZOLE	GIARDIASIS	3-Aug-05	8-Aug-05
11	14	SSG	12-Jul-05	AMOXACILLINE	PNEUMONIA	10-Aug-05	
11	14	SSG	12-Jul-05	SALBUTAMOL	B. ASTHMA	19-Jul-05	3-Aug-05
11	14	SSG	12-Jul-05	EPHEDRINE	B. ASTHMA	19-Jul-05	3-Aug-05
11	15	PM	14-Jul-05	CLOTTRIMAZOLE CREAM	T.VERSICOLOR	13-Jul-05	
11	15	PM	14-Jul-05	MALOX SYRUP	PUD	20-Jul-05	
11	16	Combination	15-Jul-05	THEOPHEDRIN	BR. ASTHMA	14-Jul-05	28-Jul-05
11	16	Combination	15-Jul-05	SALBUT.	BR. ASTHMA	14-Jul-05	28-Jul-05
11	16	Combination	15-Jul-05	MALOX SYRUP	PUD	12-Jul-05	
11	16	Combination	15-Jul-05	NORFLOX	UTI	29-Jul-05	4-Aug-05
11	16	Combination	15-Jul-05	VERMOX	HOOK WORM	29-Jul-05	31-Jul-05
11	16	Combination	15-Jul-05	ALOH3	DYSPEPSIA	31-Oct-05	4-Nov-05
11	16	Combination	15-Jul-05	CIMITIDIN	DYSPEPSIA	6-Feb-06	20-Feb-06
11	16	Combination	15-Jul-05	NORFLOXACIN	DYSPEPSIA	6-Feb-06	13-Feb-06
11	16	Combination	15-Jul-05	MALOX	DYSPEPSIA	6-Feb-06	
11	17	Combination	19-Jul-05	ALBENDAZOLE	INTESTINAL PARASITOSIS	24-Jul-05	27-Jul-05
11	17	Combination	19-Jul-05	HYOCINE	ABDOMINAL CRAMP	24-Jul-05	
11	18	SSG	16-Aug-05	CIMITEDIN	NUD	20-Aug-05	
11	18	SSG	16-Aug-05	MEBADIZOLE	HOOK WORM INFECTION	2-Sep-05	
11	18	SSG	16-Aug-05	AMOXACILIN	COMMUNITY ACQUIRED PNEUMONIA	23-Dec-05	2-Jan-06
11	19	Combination	16-Aug-05	CIMITIDIN	DSPEPSIA	29-Aug-05	
11	19	Combination	16-Aug-05	CIMITIDIN	DYSPEPSIA	29-Nov-05	13-Dec-05
11	19	Combination	16-Aug-05	CHLOROQUINE	TROPICAL SPELNOMEGALY SYNDROM	29-Nov-05	28-Jan-06
11	19	Combination	16-Aug-05	METRONIDAZOLE	E.HISTOLOTICA	27-Feb-06	8-Mar-06
11	20	SSG	16-Aug-05	AMOXACCLLINE	PNEUMONIA	9-Sep-05	16-Sep-05

Centre Number	Patient Number	Treatment	Treatment start date	Concomitant Medication	Diagnosis	Start date	End date
11	22	SSG	17-Aug-05	DYPRON	CHILLS	18-Aug-05	
11	22	SSG	17-Aug-05	PARACETAMOL	CHILLS	18-Aug-05	
11	25	SSG	20-Aug-05	MEBENDAZLE	HOOK WORM	18-Aug-05	21-Aug-05
11	25	SSG	20-Aug-05	PARACITAMOLE	FEVER	21-Aug-05	23-Aug-05
11	25	SSG	20-Aug-05	MEBENDAZOLE	ABDOMINAL DISTENSION	4-Jan-06	6-Jan-06
11	27	PM	19-Sep-05	NORFLOXACILLIN	ACUTE GASTRO ENTERITIS	22-Sep-05	26-Sep-05
11	27	PM	19-Sep-05	DICLOFENAC	PAIN AT INJECTION SITE	2-Oct-05	
11	27	PM	19-Sep-05	ORS	ACUTE GASTRO ENTERITIS	21-Sep-05	23-Sep-05
11	27	PM	19-Sep-05	CEFTRAXONE	SEPSIS OF GI	20-Jan-06	27-Jan-06
11	27	PM	19-Sep-05	40% DEXTROSE	HYPOGLYCEMIA	20-Jan-06	23-Jan-06
11	27	PM	19-Sep-05	ORS	SHOCK	20-Jan-06	28-Jan-06
11	27	PM	19-Sep-05	NORMAL SALINE	MAINTENANCE	20-Jan-06	24-Jan-06
11	27	PM	19-Sep-05	CO-TRIMOZAXOLE	PCP PROPHOLAXIS	12-Feb-06	18-Feb-06
11	29	PM	22-Sep-05	PARACETAMOL	HEADACHE	27-Sep-05	27-Sep-05
11	31	SSG	23-Sep-05	GLIBEN CLAMIDE	TYPE II DM	3-Feb-06	4-Mar-06
11	31	SSG	23-Sep-05	REGULAR INSULIN	TYPE 1 DM	7-Apr-06	7-Apr-06
11	31	SSG	23-Sep-05	LENTE INSULIN	TYPE 1 DM	8-Apr-06	14-Apr-06
11	31	SSG	23-Sep-05	LENTE INSULIN	TYPE 1 DM	14-Apr-06	19-Apr-06
11	31	SSG	23-Sep-05	LENTE INSULIN	TYPE 1 DM	20-Apr-06	24-Apr-06
11	31	SSG	23-Sep-05	LENTE INSULIN	TYPE 1 DM	24-Apr-06	3-May-06
11	31	SSG	23-Sep-05	LENTE INSULIN	TYPE 1 DM	3-May-06	10-May-06
11	31	SSG	23-Sep-05	LENTE INSULIN	TYPE 1 DM	10-May-06	
11	33	SSG	25-Sep-05	PARACETAMOL	HEADACHE	4-Oct-05	4-Oct-05
11	37	Combination	28-Sep-05	HYDROCHLORTHIAZIDE	HYPERTENSIVE	14-Apr-06	
11	38	PM	19-Oct-05	PARCITAMOLE	HEADACHE	1-Nov-05	5-Nov-05
11	38	PM	19-Oct-05	OMPEROZOLE	PEPTIC ULCER DISEASE	30-May-06	5-Jun-06
11	38	PM	19-Oct-05	CLARITHROMCIN	PEPTIC ULCER DISEASE	30-May-06	5-Jun-06
11	38	PM	19-Oct-05	AMOXACILLIN	PEPTIC ULCER DISEASE	30-May-06	5-Jun-06
11	40	PM	20-Oct-05	COARTEM	P.FALCIPAREN MALARIA	18-Oct-05	21-Oct-05
11	40	PM	20-Oct-05	PARACETAMOL	HEADACHE	23-Oct-05	23-Oct-05
11	40	PM	20-Oct-05	DYPRONE	FEVER	18-Oct-05	18-Oct-05
11	41	Combination	20-Oct-05	COARTEM	MALARIA	22-Oct-05	25-Oct-05
11	41	Combination	20-Oct-05	CHLOROQUINE	MALARIA	22-Oct-05	25-Oct-05
11	41	Combination	20-Oct-05	DIPZLRONE	MALARIA	22-Oct-05	25-Oct-05
11	42	Combination	21-Oct-05	QUININE	MALARIA	7-Nov-05	14-Nov-05
11	43	SSG	28-Oct-05	CLOTRIMAZOLE	TINEA VERSICOLAR	27-Oct-05	
11	43	SSG	28-Oct-05	CO-TRIMOXAZOLE	PYURIA (UTI)	3-Nov-05	7-Nov-05
11	45	Combination	28-Oct-05	ALBENDAZOLE	INTESTINAL PARASITOSIS	19-May-06	21-May-06
11	46	SSG	5-May-06	METRONIDAZOLE	GIADIACYST	10-May-06	16-May-06
11	46	SSG	5-May-06	CLOTRIMAZOLE	T. FACIES	16-May-06	
11	48	Combination	5-May-06	PARACETAMOLE	HEADACHE	11-May-06	11-May-06
11	49	SSG	9-May-06	ALBENDAZOLE	STRONGYIODSIS	10-May-06	12-May-06
11	52	PM	20-May-06	PARACETAMOL	FEVER	22-May-06	22-May-06
11	54	PM	27-May-06	METRONDAZOLE	E-HISTOLOTICA CYST	23-Jun-06	2-Jul-06
11	54	PM	27-May-06	CO-TRIMOXAZOLE	PROFLAXIS	29-Jun-06	
11	54	PM	27-May-06	METRONDAZOLE	AMOEBIASIS	23-Jun-06	2-Jul-06
11	54	PM	27-May-06	STAVUDINE	HIV	7-Jul-06	
11	54	PM	27-May-06	LAMUVIDINE	HIV	7-Jul-06	
11	54	PM	27-May-06	NEVIRAPINE	HIV	7-Jul-06	
11	54	PM	27-May-06	CETRIMOXAZOLE	PCP PROPHYLAXIS	29-Jun-06	
11	54	PM	27-May-06	COARTEM	MALARIA	18-Dec-06	20-Dec-06

Centre Number	Patient Number	Treatment	Treatment start date	Concomitant Medication	Diagnosis	Start date	End date
11	54	PM	27-May-06	CHLOROQUINE	MALARIA	18-Dec-06	20-Dec-06
11	54	PM	27-May-06	AMOXACILLINE	DENTAL CARIES	25-Dec-06	1-Jan-07
11	54	PM	27-May-06	IBUPROFEN	DENTAL CARIES	25-Dec-06	29-Dec-06
11	55	PM	30-May-06	CLOTRIMAZOLE CREAM	T. VERSICOLOR	2-Jun-06	20-Jun-06
11	55	PM	30-May-06	CIMITIDIN	DYSPEPSIA	2-Jun-06	12-Jun-06
11	57	Combination	4-Jun-06	AMOXACELLINE	CAP (COMMUNITY ACQUIRED PNEUMONIA)	6-Jun-06	13-Jun-06
11	57	Combination	4-Jun-06	MALOX SYRUP	DYSPEPSIA	10-Jun-06	
11	57	Combination	4-Jun-06	CLOTRIMAZOLE	T. VERSICOLOR	10-Jun-06	
11	58	Combination	6-Jun-06	PARACETAMOL	PAIN AT INJECTION SITE	20-Jun-06	20-Jun-06
11	59	PM	13-Jun-06	ALDOMET	HYPERTENSION	8-Jul-06	11-Jul-06
11	59	PM	13-Jun-06	ALDOMET	HYPERTENSION	29-Aug-06	
11	59	PM	13-Jun-06	ALDOMET	HYPERTENSION	29-Aug-06	
11	66	Combination	23-Jun-06	NORFLOXACILLIN	URINARY TRACT INFECTION	23-Jun-06	30-Jun-06
11	69	Combination	15-Jul-06	TINIDAZOLE	GIARDIASIS	31-Oct-06	31-Oct-06
11	70	Combination	15-Jul-06	PARACETAMOLE	INJECTION SITE PAIN	28-Jul-06	28-Jul-06
11	70	Combination	15-Jul-06	BENZYL BENZOATE	SCABIES	9-Oct-06	12-Oct-06
11	71	Combination	22-Jul-06	MALLOX	DYSPEPSIA	1-Aug-06	
11	71	Combination	22-Jul-06	CIMETIDINE	DYSPEPSIA	1-Aug-06	10-Aug-06
11	71	Combination	22-Jul-06	ORS	DIARRHEA	29-Jul-06	4-Aug-06
11	71	Combination	22-Jul-06	STAVUDIN	HIV	19-Jul-05	
11	71	Combination	22-Jul-06	LAMVUDIN	HIV	19-Jul-05	
11	71	Combination	22-Jul-06	EFV	HIV	19-Jul-05	
11	71	Combination	22-Jul-06	CO-TRIMOXOZOL	PCP PROPHYLAXIS	19-Jul-05	
11	71	Combination	22-Jul-06	METRONIDAZOLE	AMOEBIASIS	21-Aug-06	31-Aug-06
11	71	Combination	22-Jul-06	MALLOX	PUD	4-Jan-06	
11	71	Combination	22-Jul-06	MULTIVITAMIN	EASYFATIGABILITY	4-Jan-06	14-Jan-06
11	72	SSG	22-Jul-06	MALOX SYRUP	DYSPEPSIA	21-Jul-06	15-Aug-06
11	72	SSG	22-Jul-06	MULTI-VITAMIN	LOSS OF APPITITE	27-Jul-06	5-Aug-06
11	72	SSG	22-Jul-06	DNS	MAINTENANCE	1-Aug-06	5-Aug-06
11	72	SSG	22-Jul-06	DEXTROSE	HEPATITIS	1-Aug-06	1-Aug-06
11	72	SSG	22-Jul-06	VITAMIN B.COMPLEX	HEPATITIS (DRUG INDUCED)	1-Aug-06	5-Aug-06
11	72	SSG	22-Jul-06	CEFTRIAXONE	SEPSIS OF GI FOCUS	5-Aug-06	
11	72	SSG	22-Jul-06	ORS	FOR ONGOING REPLACEMENT	5-Aug-06	7-Aug-06
11	72	SSG	22-Jul-06	GENTAMYCIN	SEPSIS OF GI FOCUS	6-Aug-06	
11	72	SSG	22-Jul-06	STAVUDIN	HIV	31-Jul-06	
11	72	SSG	22-Jul-06	LAMUVIDIN	HIV	31-Jul-06	
11	72	SSG	22-Jul-06	NEVIRAPINE	HIV	31-Jul-06	
11	72	SSG	22-Jul-06	CO-TRIMOXOZOL	PCP PROPHYLAXIS	31-Jul-06	
11	75	Combination	28-Jul-06	CIPROFLOXACLIN	ABDOMINAL CRAMP	10-Aug-06	16-Aug-06
11	76	PM	31-Jul-06	MALOX SYRUP	DYSPEPSIA	30-Jul-06	21-Aug-06
11	79	PM	31-Jul-06	MULTI-VITAMIN	LOSS OF APPETITE	3-Aug-06	13-Aug-06
11	79	PM	31-Jul-06	ORS	ACUTE GASTROENTIRITIS	5-Aug-06	6-Aug-06
11	79	PM	31-Jul-06	NS NORMAL SALINE	ACUTE GASTROENTIRITIS	5-Aug-06	6-Aug-06
11	83	SSG	16-Aug-06	CIPROFLOXACELINE	ACUTE GASTRO INTERITIS	22-Aug-06	29-Aug-06
11	84	SSG	18-Aug-06	DOXYCYCLINE	PNEUMONIA	17-Sep-06	24-Sep-06
11	85	SSG	25-Aug-06	MALLOX	DYSPEPSIA	21-Nov-06	5-Dec-06
11	85	SSG	25-Aug-06	RAMTIDINE	DYSPEPSIA	21-Nov-06	5-Dec-06
11	88	PM	29-Aug-06	DOXYCYCLINE	PNEMONIA	21-Nov-06	30-Nov-06

Centre Number	Patient Number	Treatment	Treatment start date	Concomitant Medication	Diagnosis	Start date	End date
11	89	Combination	2-Sep-06	DOXYCYCLINE	ATYPICAL PNEUMONIA	17-Nov-06	27-Nov-06
11	91	PM	18-Dec-06	MALOX	DYSPEPSIA	24-Apr-07	3-Jul-07
11	91	PM	18-Dec-06	CIMETIDINE	DYSPEPSIA	24-Apr-07	8-May-07
11	97	Combination	20-Jan-07	CIPROFLOXACILIN	ACUTE GASTROENTERITIES	21-Jan-07	27-Jan-07
11	98	PM	24-Jan-07	CLOTRIMAZOLE CREAM	T. VERSICOLOR	23-Jan-07	10-Mar-07
11	98	PM	24-Jan-07	CIPROFLOXACILIN	UTI URINARY TRACT INFE	8-Feb-07	15-Feb-07
11	100	PM	26-Jan-07	MALOX SYRUP	DYSPEPSIA	12-Feb-07	28-Feb-07
11	103	Combination	7-Feb-07	MULTIVITAMIN	LOSS OF APPETITE	2-Feb-07	12-Feb-07
11	106	Combination	25-Feb-07	PARACETAMOLE	FEVER	24-Feb-07	26-Feb-07
11	108	PM	2-Mar-07	CHLOROQUINE	VIVAX MALARIA	23-Mar-07	25-Mar-07
11	109	SSG	3-Mar-07	MALOX	DYSPEPSIA	4-Mar-07	2-Apr-07
11	109	SSG	3-Mar-07	TINIDAZOL	INTESTINAL PARACITE	29-Mar-07	29-Mar-07
11	109	SSG	3-Mar-07	MEBENDAZOL	INTESTINAL PARACITE	29-Mar-07	1-Apr-07
11	109	SSG	3-Mar-07	DOXYCYCLINE	ATYPICAL PNEUMONIA	31-Mar-07	9-Apr-07
11	113	SSG	28-Mar-07	NICLOSAMIDE	INTESTINAL PARACITOSIS	3-Apr-07	3-Apr-07
11	113	SSG	28-Mar-07	AMOXACILLINE	URTI	20-Apr-07	27-Apr-07
11	114	SSG	30-Mar-07	CLOTRIMAZOLE CREAM	T.VERSICOLOR	2-Apr-07	30-Apr-07
11	116	SSG	31-Mar-07	ACYCLOVIR	HERPES ZOSTER	30-Apr-07	5-May-07
11	117	PM	4-Apr-07	CIPROFLOXACIN	UTI	15-Apr-07	22-Apr-07
11	117	PM	4-Apr-07	NEUROBION	PERIPHERAL NEUROPATHY	23-Apr-07	3-May-07
11	122	SSG	21-Apr-07	CLOXACILLINE	PYOGENIC LYMPHADENOITIS	4-May-07	11-May-07
11	122	SSG	21-Apr-07	CHLORAMPHENICOL	PYOGENIC LYMPHADENITIS	8-May-07	19-May-07
11	123	SSG	28-Apr-07	CLOXACILLIN	CELLULITIS	18-May-07	25-May-07
11	124	SSG	21-May-07	M. VITAMIN	LOSS OF APPETITE	20-May-07	31-May-07
11	124	SSG	21-May-07	TINIDAZOLE	AMOEBIASIS	22-May-07	22-May-07
11	124	SSG	21-May-07	CLOXACILLIN	CELLULITIS	25-May-07	4-Jun-07
11	124	SSG	21-May-07	CHLORAMPHENCOL	CELLULITIS	25-May-07	4-Jun-07
11	124	SSG	21-May-07	ACYCLOVIR	CHICKENPOX	18-Jun-07	
11	124	SSG	21-May-07	CLOXACILLIN	IMPETIGENIZED CHICKENPOX	18-Jun-07	
11	126	Combination	28-May-07	ACYCLOVIR	HERPES ZOSTER	29-May-07	5-Jun-07
11	126	Combination	28-May-07	DICLOFENAC	NEUROPATHIC PAIN	29-May-07	5-Jun-07
11	126	Combination	28-May-07	CO-TRIMOXAZOLE	CO-TRIZOXAZOLE PROPHYLAXIS	10-Jun-07	
11	126	Combination	28-May-07	AMITRYPITLIN	NEUROPATHIC PAIN	25-Jun-07	3-Jul-07
11	126	Combination	28-May-07	OMEPEVOZOLE	DYSPEPSIA	13-Dec-07	27-Dec-07
11	126	Combination	28-May-07	STAVUDINE	HIV	23-Jun-07	
11	126	Combination	28-May-07	LAMUVIDINE	HIV	23-Jun-07	
11	126	Combination	28-May-07	EFAVIVENZ	HIV	23-Jun-07	
11	128	Combination	2-Jun-07	CO-TRIMOXAZOLE	CO-TRIMOXAZOLE PROPHYLAXIS	10-Jun-07	
11	132	SSG	16-Jun-07	ANTIMALARIA	MALARIA	19-Nov-07	21-Nov-07
11	135	PM	13-Jul-07	CLOTRIMAZOLE CREAM	T.VERSICOLOR	12-Jul-07	
12	241	Combination	29-Jun-05	AMOXACILLIN	CELLULITIS	13-Jul-05	18-Jul-05
12	241	Combination	29-Jun-05	PARACETAMOL	INJECTION SITE PAIN	13-Jul-05	18-Jul-05
12	242	SSG	29-Jun-05	AMPICILLIN	UTI	18-Jul-05	22-Jul-05

Centre Number	Patient Number	Treatment	Treatment start date	Concomitant Medication	Diagnosis	Start date	End date
12	242	SSG	29-Jun-05	ORS	VOMITING	17-Jul-05	18-Jul-05
12	242	SSG	29-Jun-05	TTC EYE OINTMENT	BACTERIAL CONJECTIVITIS	24-Oct-05	29-Oct-05
12	242	SSG	29-Jun-05	CLOXACILLIN	INFECTED SKIN LESION	9-Feb-06	16-Feb-06
12	244	SSG	29-Jun-05	METRONIDAZOLE	GIARDIASIS	8-Jul-05	12-Jul-05
12	244	SSG	29-Jun-05	ALBENDAZOLE	STROGLOIDIASIS	8-Jul-05	10-Jul-05
12	244	SSG	29-Jun-05	COTRIMOXAZOLE	UTI	20-Jul-05	24-Jul-05
12	244	SSG	29-Jun-05	METRONIDAZOLE	GIARDIASIS	30-Oct-05	4-Nov-05
12	244	SSG	29-Jun-05	AMP	LEG ULCER	4-Apr-06	20-Apr-06
12	244	SSG	29-Jun-05	TTC EYE OINT	CONJUCTIVITIS	4-Apr-06	11-Apr-06
12	245	PM	20-Jul-05	DOXYCYCLINE	PNEUMONIA	28-Nov-05	5-Dec-05
12	245	PM	20-Jul-05	PARACETAMOL	PNEUMONIA	28-Nov-05	5-Dec-05
12	246	PM	4-Aug-05	ORS	AGE	21-Aug-05	23-Aug-05
12	246	PM	4-Aug-05	PARACITAMOL	HEADACHE BACK PAI	24-Aug-05	25-Aug-05
12	246	PM	4-Aug-05	ORS	CHRONIC DIARRHEA	21-Aug-05	25-Aug-05
12	246	PM	4-Aug-05	COTRIMOXAZOLE	DYSENTRY	4-Nov-05	11-Nov-05
12	246	PM	4-Aug-05	KETOCOMAZOLE	TINIA CORPORIS	4-Nov-05	19-Nov-05
12	246	PM	4-Aug-05	WHITE FIELD	TINIA CORPORIS	4-Nov-05	11-Nov-05
12	247	PM	28-Sep-05	PCM	INJECTION SITE PAIN	10-Oct-05	13-Oct-05
12	247	PM	28-Sep-05	AMOX	PNEUMONIA	19-Oct-05	22-Oct-05
12	249	Combination	25-Oct-05	PCM	FEVER	25-Oct-05	25-Oct-05
12	249	Combination	25-Oct-05	DICLOPLEN	FEVER	25-Oct-05	25-Oct-05
12	249	Combination	25-Oct-05	DIPYRON	FEVER	25-Oct-05	25-Oct-05
12	249	Combination	25-Oct-05	AMOX	PNEUMONIA	4-Mar-06	8-Mar-06
12	249	Combination	25-Oct-05	PCM	PNEUMONIA	4-Mar-06	4-Mar-06
12	249	Combination	25-Oct-05	ERYTHROMYCIN	PNEUMONIA	8-Mar-06	22-Mar-06
12	249	Combination	25-Oct-05	COTRIMOX	PNEUMONIA	8-Mar-06	22-Mar-06
12	250	SSG	25-Oct-05	AMOX	OTITIS MEDIA	17-Nov-05	22-Nov-05
12	251	Combination	29-Oct-05	AMOXACILLIN	OTITIS MEDIA	11-Nov-05	15-Nov-05
12	253	SSG	29-Oct-05	PARACITAMOL	COMMON COLD	9-Nov-05	10-Nov-05
12	254	Combination	29-Oct-05	PARACITAMOL	PAIN ON INJECTION SITE	11-Nov-05	12-Nov-05
12	261	Combination	3-Jan-06	PCM	INJECT PAIN	16-Jan-06	20-Jan-06
12	264	Combination	15-Feb-06	COARTEM	P.FALCIPARUM	7-Feb-06	10-Feb-06
12	264	Combination	15-Feb-06	METRONIDA	GIARDIASIS	7-Feb-06	14-Feb-06
12	264	Combination	15-Feb-06	PARACETAMOL	INJECTION SITE PAIN	4-Mar-06	8-Mar-06
12	265	SSG	15-Feb-06	AMOXACILLIN	1 SUPERINFECTED CHILLOSIS	25-Feb-06	4-Mar-06
12	265	SSG	15-Feb-06	AMOXACILLIN	2 UTI	25-Feb-06	4-Mar-06
12	265	SSG	15-Feb-06	PARACITAMOL	FEVER	25-Feb-06	28-Feb-06
12	265	SSG	15-Feb-06	CLOXACILLIN	HOSPITAL ACQUIRED PNEU	13-Mar-06	15-Mar-06
12	265	SSG	15-Feb-06	CEFTRIAXONE	HAP + SEPSIS	14-Mar-06	20-Mar-06
12	265	SSG	15-Feb-06	LASIX	ACUTE RENAL FAILURE	14-Mar-06	
12	265	SSG	15-Feb-06	BLOOD TRANSFUSION	SEVERE ANEEMIA + BLEEDING	18-Mar-06	
12	271	SSG	31-Mar-06	PARACITAMOL	FEVER	21-Apr-06	22-Apr-06
12	276	PM	8-Apr-06	METRONIDAZOL	GIARDIASIS	4-Apr-06	11-Apr-06
12	283	PM	11-May-06	METRINIDAZOLE	AMEBIASIS	10-May-06	17-May-06
12	284	Combination	20-May-06	ADRENALIN	EPISTAXIS	9-Dec-06	10-Dec-06
12	295	Combination	16-Mar-07	MEBANDAZO	HOOKWORM	11-Mar-07	14-Mar-07
12	295	Combination	16-Mar-07	METRINDAZO	GIARDIASIS	19-Mar-07	24-Mar-07
12	303	PM	5-Jun-07	TETRACYCLINE	CONJUCTIVITIS	1-Oct-07	5-Oct-07
12	307	Combination	15-Jun-07	MAGNISIUM TRISILICATE	GASTRITIS	22-Jun-07	26-Jun-07
12	307	Combination	15-Jun-07	PARACITAMOL	SHOULDER PAIN	1-Oct-07	4-Oct-07

Centre Number	Patient Number	Treatment	Treatment start date	Concomitant Medication	Diagnosis	Start date	End date
12	308	PM	15-Jun-07	ANTIACID SUSPENSION	GASTRITIS	18-Jun-07	22-Jun-07
12	308	PM	15-Jun-07	ERYTHROMYCIN	PNEUMONIA	10-Oct-07	20-Oct-07
12	308	PM	15-Jun-07	AMOXACILLIN	CHRONIC SINUSITIS	27-Dec-07	2-Jan-08
12	310	SSG	16-Jun-07	PARACETAMOL	INJECTION SITE PAIN	6-Jul-07	9-Jul-07
12	321	Combination	6-Sep-07	PARACITAMOL	INJECTION SITE PAIN	12-Sep-07	21-Sep-07
12	321	Combination	6-Sep-07	METRONIDAZOL	GIARDIASIS	19-Sep-07	24-Sep-07
12	322	PM	13-Sep-07	METRONIDAZOLE	GIARDIASIS	25-Sep-07	1-Oct-07
12	322	PM	13-Sep-07	METRONIDAZOLE	AMEOBIASIS	25-Sep-07	1-Oct-07
12	324	SSG	20-Sep-07	METRONIDAZOLE	AMOEBIASIS	4-Oct-07	11-Oct-07
12	325	PM	27-Sep-07	TINIDAZOLE	GIARDIASIS	18-Sep-07	18-Sep-07
12	325	PM	27-Sep-07	KETOCOMAZOLE	TINIA ALBA	3-Oct-07	13-Oct-07
12	326	PM	3-Oct-07	MEBENDAZOLE	ASCARIASIS	9-Oct-07	11-Oct-07
12	326	PM	3-Oct-07	AMOXACILIN	OTITIS MEDIA	13-Oct-07	17-Oct-07
12	329	SSG	10-Oct-07	AMOXACILLIN	OTITIS MEDIA	18-Oct-07	23-Oct-07
23	362	PM	20-Jan-05	BENZYL-PENICILLIN	LOBAR PNEUMONIA	26-Jan-05	1-Feb-05
23	364	Combination	20-Jan-05	PARACETAMOL	FEVER	27-Apr-05	1-May-05
23	364	Combination	20-Jan-05	BROZEDEX	COUGH	27-Apr-05	4-May-05
23	366	Combination	1-Feb-05	TETRACYCLINE OINTMENT	CONJUNCTIVITIS (BILATERAL)	19-Aug-05	
23	367	SSG	1-Feb-05	CRYSTALLINE PENICILLIN	PNEUMONIA	26-Feb-05	28-Feb-05
23	367	SSG	1-Feb-05	GENTAMYCIN	PNEUMONIA	26-Feb-05	26-Feb-05
23	367	SSG	1-Feb-05	PARACETAMOL	PNEUMONIA	26-Feb-05	2-Mar-05
23	367	SSG	1-Feb-05	CIPROFLOXACIN	PNEUMONIA	27-Feb-05	5-Mar-05
23	367	SSG	1-Feb-05	GENTIN VIOLET	HERPETIC MOUTH ULCER	3-Mar-05	3-Mar-05
23	367	SSG	1-Feb-05	CRYSTALLINE PENICILLIN	PNEUMONIA	9-Feb-05	14-Feb-05
23	367	SSG	1-Feb-05	MAKDEX EYE DROPS	ALLERGIC CONJUCTIVITIS	8-Jun-05	13-Jun-05
23	367	SSG	1-Feb-05	FLOXAPEN	OTITIS MEDIA	7-Mar-05	12-Mar-05
23	367	SSG	1-Feb-05	CATEFLAM	OTITIS MEDIA	7-Mar-05	12-Mar-05
23	369	Combination	4-Feb-05	CRYSTAL PEN.	BRONCHO PNEUMONIA	28-Jan-05	3-Feb-05
23	369	Combination	4-Feb-05	GENTAMICIN	BRONCHO PNEUMONIA	28-Jan-05	2-Feb-05
23	370	SSG	4-Feb-05	CRYTALLIN PENICILLIN	B/PNEUMONIA	28-Jan-05	2-Feb-05
23	371	PM	4-Feb-05	PARACETAMOL	ABDOMINAL PAIN	4-Feb-05	14-Feb-05
23	371	PM	4-Feb-05	CRYSTALLINE PENICILLIN	PERITONITIS	6-Feb-05	9-Feb-05
23	371	PM	4-Feb-05	MEBENDAZOLE	ASCARIS INFECTION	7-Feb-05	9-Feb-05
23	371	PM	4-Feb-05	VITAMIN K	ASCITES ? BLEEDING	7-Feb-05	9-Feb-05
23	371	PM	4-Feb-05	FLAGYL	PERITONITIS	7-Feb-05	14-Feb-05
23	371	PM	4-Feb-05	AUGUMENTIN	U.T.I (URINARY TRACT INFECTION)	21-Feb-05	28-Feb-05
23	371	PM	4-Feb-05	BROZEDEX	URTI	5-Sep-05	10-Sep-05
23	371	PM	4-Feb-05	CLAVULIN	URTI	7-Sep-05	14-Sep-05
23	371	PM	4-Feb-05	DUCOLAX	?CONSTIPESLIN	14-Sep-05	15-Sep-05
23	374	SSG	24-Feb-05	PARACETAMOL	FEVER	19-Feb-05	23-Feb-05
23	374	SSG	24-Feb-05	BENZYL PENICILLIN	B. PNEUMONIA	19-Feb-05	24-Feb-05
23	374	SSG	24-Feb-05	NITROFUNATON	UTI	7-Mar-05	13-Mar-05
23	374	SSG	24-Feb-05	VIT K	BLEEDING GUMS	12-Mar-05	14-Mar-05
23	374	SSG	24-Feb-05	PARACETAMOL	NECK PAINS	17-Mar-05	20-Mar-05
23	375	Combination	25-Feb-05	TETRACYCLINE OINTMENT	CONJUNCTIVITIS	11-Mar-05	15-Mar-05

Centre Number	Patient Number	Treatment	Treatment start date	Concomitant Medication	Diagnosis	Start date	End date
23	375	Combination	25-Feb-05	PARACETAMOL	EYE PAIN	11-Mar-05	13-Mar-05
23	376	SSG	1-Mar-05	VIT. K.	PROLONGED PROTHROMBIN TIME	23-Feb-05	26-Feb-05
23	376	SSG	1-Mar-05	PARACETAMOL	FEVER HEADACHE JOINT PAINS	7-Mar-05	9-Mar-05
23	376	SSG	1-Mar-05	TETRACYCLINE EYE OINTMENT	CONJUCTIVITIS	28-Mar-05	31-Mar-05
23	377	Combination	9-Mar-05	PARACETAMOL	1 GM	13-Mar-05	14-Mar-05
23	378	Combination	9-Mar-05	CEFTRIAXONE	BIL PNEUMONIA	7-Jul-05	11-Jul-05
23	379	PM	10-Mar-05	ERYTHROMYCIN	PNEUMONIA	1-Mar-05	7-Mar-05
23	379	PM	10-Mar-05	AMPICLOX	SEPTIC WOUND	30-Sep-05	7-Oct-05
23	379	PM	10-Mar-05	PARACETAMOL	SEPTIC WOUND	30-Sep-05	7-Oct-05
23	379	PM	10-Mar-05	BETADINE	SEPTIC WOUND	30-Sep-05	7-Oct-05
23	380	SSG	15-Mar-05	ALBENDAZOLE	DE-WORMING	10-Mar-05	10-Mar-05
23	380	SSG	15-Mar-05	FLAGYL	AMOEBIASIS GIARDIASIS	10-Mar-05	14-Mar-05
23	381	PM	15-Mar-05	ALBENDAZOLE TABS	GENERAL DEWORMING	10-Mar-05	10-Mar-05
23	381	PM	15-Mar-05	MEBENDAZOLE TABS	A HISTOLYTICA CYSTS	10-Mar-05	15-Mar-05
23	381	PM	15-Mar-05	ENHANCIN	PNEUMONIA	13-Jul-05	22-Jul-05
23	381	PM	15-Mar-05	NIZORAL	TINEA CAPITIS	19-Jul-05	22-Jul-05
23	381	PM	15-Mar-05	ENHANCIN	PNEUMONIA	13-Jul-05	21-Jul-05
23	381	PM	15-Mar-05	NIZORAL	TINEA CAPITIS	19-Jul-05	2-Aug-05
23	382	Combination	18-Mar-05	CRYSTALLINE PENICILLIN	U.R.T.I (UPPER RESP. TRACT INFECTION)	21-Mar-04	23-Mar-04
23	382	Combination	18-Mar-05	AMOXIL	URTI	23-Mar-04	29-Mar-04
23	382	Combination	18-Mar-05	ASCORIL	URTI	25-Mar-04	29-Mar-04
23	382	Combination	18-Mar-05	PARACETAMOL	CELLULITIS RIGHT BUTTOCK	3-Apr-05	7-Apr-05
23	382	Combination	18-Mar-05	SUPRAPEN	CELLULITIS RT. BUTTOCK	4-Apr-05	7-Apr-05
23	383	PM	5-Apr-05	XPEN	B. PNEUMONIA	27-Mar-05	31-Mar-05
23	383	PM	5-Apr-05	CLAVULIN	B. PNEUMONIA	1-Apr-05	4-Apr-05
23	383	PM	5-Apr-05	PANADOL	B. PNEUMONIA	29-Mar-05	5-Apr-05
23	383	PM	5-Apr-05	CERUMOL	EAR WAX	1-Apr-05	4-Apr-05
23	383	PM	5-Apr-05	PANADOL	GLUTEAL TENDERNESS	20-Apr-05	25-Apr-05
23	384	SSG	5-Apr-05	AMOXYCILLIN CAPS	PNEUMONIA	28-Mar-05	3-Apr-05
23	384	SSG	5-Apr-05	POP	LT RADIAL	28-Apr-05	
23	384	SSG	5-Apr-05	CLAVULIN	PNEUMONIA	17-Nov-05	24-Nov-05
23	384	SSG	5-Apr-05	ASCORIL	WHEEZING	17-Nov-05	24-Nov-05
23	385	SSG	7-Apr-05	VITAMIN K	PROLONGED PROTHROMBIN TIME	24-Mar-05	26-Mar-05
23	385	SSG	7-Apr-05	VIT K	PROLONGED PROTHROMBIN TIME	29-Mar-05	31-Mar-05
23	385	SSG	7-Apr-05	AMOXIL	PNEUMONIA	1-Apr-05	5-Apr-05
23	385	SSG	7-Apr-05	CEFITRIAXONE	PNEUMONIA	15-Apr-05	20-Apr-05
23	392	Combination	22-Apr-05	XPEN	PNEUMONIA	25-Apr-05	29-Apr-05
23	392	Combination	22-Apr-05	PIRITON	ALLERGIC SKIN REACTION	12-May-05	25-May-05
23	392	Combination	22-Apr-05	BETADINE	ALLERGIC SKIN REACTION	19-May-05	27-May-05
23	392	Combination	22-Apr-05	BETADINE	BRUISES ON BOTH LEGS	22-Jul-05	29-Jul-05
23	394	PM	26-Apr-05	CRYSTALLINE PENICILLIN	PNEUMONIA	19-Apr-05	19-Apr-05
23	394	PM	26-Apr-05	AUGUMENTIN	PNEUMONIA	19-Apr-05	26-Apr-05
23	394	PM	26-Apr-05	ALBENDAZOLE	POSSIBLE HELMINTHS	19-Apr-05	19-Apr-05
23	394	PM	26-Apr-05	BUSCOPAN	ABDOMINAL PAIN	30-Apr-05	1-May-05
23	395	PM	29-Apr-05	PARACETAMOL	HEADACHE & FEVER	15-May-05	17-May-05

Centre Number	Patient Number	Treatment	Treatment start date	Concomitant Medication	Diagnosis	Start date	End date
23	396	SSG	13-May-05	AMOXYCILLIN CAPS	ACUTE OTITIS MEDIA	20-May-05	27-May-05
23	396	SSG	13-May-05	PARACETAMOL TABS	ACUTE OTITIS MEDIA	20-May-05	27-May-05
23	397	SSG	18-May-05	(CEPHOGRAM) CETRIAXONE	BASAL PNEUMONITIS	13-May-05	18-May-05
23	397	SSG	18-May-05	PARACETAMOL	BASAL PNEUMONITIS	13-May-05	15-May-05
23	397	SSG	18-May-05	VIT K	NOT KNOWN	13-May-05	16-May-05
23	399	PM	18-May-05	TETRACYCLINE	CONJUCTIVITIS	14-Oct-05	21-Oct-05
23	399	PM	18-May-05	TETANUS TOXOID	WOUND RT TOE	14-Oct-05	14-Oct-05
23	400	PM	3-Jun-05	CRYSTALLINE PENICILLIN	PNEUMONIA	14-May-05	24-May-05
23	400	PM	3-Jun-05	PANADOL	PNEUMONIA	14-May-05	23-May-05
23	400	PM	3-Jun-05	MUCO SLOVAN	PNEUMONIA	20-May-05	30-May-05
23	400	PM	3-Jun-05	BLOOD	ANAEMIA	25-May-05	26-May-05
23	400	PM	3-Jun-05	CEFTRIOXONE	PNEUMONIA	23-May-05	30-May-05
23	401	Combination	3-Jun-05	PARACETAMOL	FEVER	8-Sep-05	14-Sep-05
23	401	Combination	3-Jun-05	LASIX	TO COVER TRANSFUSION	15-Sep-05	15-Sep-05
23	401	Combination	3-Jun-05	FANSIDAR	TO COVER TRANSFUSION	15-Sep-05	15-Sep-05
23	401	Combination	3-Jun-05	LASIX	TRANSFUSION	15-Sep-05	15-Sep-05
23	401	Combination	3-Jun-05	FANSIDER	TRANSFUSION	15-Sep-05	15-Sep-05
23	403	SSG	4-Jun-05	BETADINE MOUTH WASH	DENTAL CARIES & GINGIVITIS	9-Jun-05	29-Jun-05
23	403	SSG	4-Jun-05	PIRITON	PAPULAR RASH	23-Jun-05	26-Jun-05
23	405	PM	10-Jun-05	ERYTHROMYCIN	ATYPICAL PNEUMONIA	4-Jun-05	8-Jun-05
34	451	Combination	17-Nov-04	MEASLES VACC.	PROPHYLAXIS	17-Nov-04	
34	451	Combination	17-Nov-04	MEASLES VACC.	PROPHYLAXIS	4-Dec-04	
34	451	Combination	17-Nov-04	VITAMIN A	SUPPLEMENTARY	17-Nov-04	
34	451	Combination	17-Nov-04	MULTI VIT	SUPPLEMENTARY	17-Nov-04	3-Dec-04
34	451	Combination	17-Nov-04	FOLIC ACID	ANAEMIA	17-Nov-04	3-Dec-04
34	451	Combination	17-Nov-04	FERROUS SULFATE	ANAEMIA	17-Nov-04	3-Dec-04
34	451	Combination	17-Nov-04	PARACETAMOL	FEVER	17-Nov-04	19-Nov-04
34	452	PM	17-Nov-04	MEASLES VACC	PROPHYLAXIS	17-Nov-04	
34	452	PM	17-Nov-04	MEASLES VACC.	PROPHYLAXIS	7-Dec-04	
34	452	PM	17-Nov-04	VITAMIN A	SUPPLEMENTARY	17-Nov-04	
34	452	PM	17-Nov-04	FOLIC ACID	ANAEMIA	17-Nov-04	7-Dec-04
34	452	PM	17-Nov-04	FERROUS SULFATE	ANAEMIA	17-Nov-04	7-Dec-04
34	452	PM	17-Nov-04	MULTI VITS	SUPPLEMENTARY	17-Nov-04	7-Dec-04
34	452	PM	17-Nov-04	PARACETAMOL	FEVER	16-Nov-04	17-Nov-04
34	453	PM	18-Nov-04	PARACETAMOL	FEVER	18-Nov-04	24-Nov-04
34	453	PM	18-Nov-04	FOLIC ACID	ANAEMIA	18-Nov-04	17-Jan-05
34	453	PM	18-Nov-04	FERROUS SULFATE	ANAEMIA	18-Nov-04	17-Jan-05
34	453	PM	18-Nov-04	MULTI VITS	SUPPLEMENTARY	18-Nov-04	17-Jan-05
34	453	PM	18-Nov-04	SULFADOXINE	MALARIA	21-Nov-04	
34	453	PM	18-Nov-04	PYRIMETHAMINE	MALARIA	21-Nov-04	
34	453	PM	18-Nov-04	ARTEMETHER	MALARIA	22-Nov-04	25-Nov-04
34	453	PM	18-Nov-04	ARTESUNATE	MALARIA	26-Nov-04	27-Nov-04
34	454	Combination	21-Nov-04	MEASLES VACC.	PROPHYLAXIS	19-Nov-04	
34	454	Combination	21-Nov-04	MEASLES VACC.	PROPHYLAXIS	8-Dec-04	
34	454	Combination	21-Nov-04	VITAMIN A	SUPPLEMENTARY	18-Nov-04	
34	454	Combination	21-Nov-04	FOLIC ACID	ANAEMIA	18-Nov-04	8-Dec-04
34	454	Combination	21-Nov-04	FERROUS SULFATE	ANAEMIA	18-Nov-04	8-Dec-04
34	454	Combination	21-Nov-04	MULTI VITS	ANAEMIA	18-Nov-04	8-Dec-04
34	454	Combination	21-Nov-04	PARACETAMOL	FEVER	18-Nov-04	22-Nov-04
34	454	Combination	21-Nov-04	AMOXICILLIN	PNEUMONIA	18-Nov-04	22-Nov-04

Centre Number	Patient Number	Treatment	Treatment start date	Concomitant Medication	Diagnosis	Start date	End date
34	455	PM	19-Nov-04	MEASLES VACC.	PROPHYLAXIS	19-Nov-04	
34	455	PM	19-Nov-04	MEASLES VACC.	PROPHYLAXIS	9-Dec-04	
34	455	PM	19-Nov-04	VITAMIN A	SUPPLEMENTARY	18-Nov-04	
34	455	PM	19-Nov-04	FOLIC ACID	ANAEMIA	18-Nov-04	1-Jan-05
34	455	PM	19-Nov-04	FERROUS SULFATE	ANAEMIA	18-Nov-04	1-Jan-05
34	455	PM	19-Nov-04	MULTI VITS	SUPPLEMENTARY	18-Nov-04	1-Jan-05
34	455	PM	19-Nov-04	PARACETAMOL	FEVER	18-Nov-04	29-Nov-04
34	455	PM	19-Nov-04	ARTESUNATE	MALARIA	25-Nov-04	27-Nov-04
34	455	PM	19-Nov-04	SULFADOXINE		25-Nov-04	
34	455	PM	19-Nov-04	PYRIMETHAMINE		25-Nov-04	
34	455	PM	19-Nov-04	QUININE		28-Nov-04	4-Dec-04
34	456	SSG	19-Nov-04	MEASLES VACC.	PROPHYLAXIS	18-Nov-04	
34	456	SSG	19-Nov-04	MEASLES VACC.	PROPHYLAXIS	16-Dec-04	
34	456	SSG	19-Nov-04	VITAMIN A	SUPPLEMENTARY	18-Nov-04	
34	456	SSG	19-Nov-04	FOLIC ACID	ANAEMIA	18-Nov-04	17-Dec-04
34	456	SSG	19-Nov-04	FERROUS SULFATE	ANAEMIA	18-Nov-04	17-Dec-04
34	456	SSG	19-Nov-04	MULTI VITS	SUPPLEMENTARY	18-Nov-04	17-Dec-04
34	456	SSG	19-Nov-04	PARACETAMOL	FEVER	18-Nov-04	24-Nov-04
34	457	Combination	19-Nov-04	VITAMIN A	SUPPLEMENTARY	18-Nov-04	
34	457	Combination	19-Nov-04	FOLIC ACID	ANAEMIA	18-Nov-04	5-Dec-04
34	457	Combination	19-Nov-04	FERROUS SULPHATE	ANAEMIA	18-Nov-04	5-Dec-04
34	457	Combination	19-Nov-04	PARACETAMOL	FEVER	18-Nov-04	21-Nov-04
34	457	Combination	19-Nov-04	DEXTROSE 5%	DEHYDRATION	18-Nov-04	
34	457	Combination	19-Nov-04	MULTI VIT	SUPPLEMENTARY	18-Nov-04	5-Dec-04
34	458	PM	20-Nov-04	MEASLES VACC.	PROPHYLAXIS	20-Nov-04	
34	458	PM	20-Nov-04	MEASLES VACC.	PROPHYLAXIS	12-Dec-04	
34	458	PM	20-Nov-04	VITAMIN A	SUPPLEMENTARY	19-Nov-04	
34	458	PM	20-Nov-04	FOLIC ACID	ANAEMIA	19-Nov-04	11-Dec-04
34	458	PM	20-Nov-04	FERROUS SULPHATE	ANAEMIA	19-Nov-04	11-Dec-04
34	458	PM	20-Nov-04	PARACETAMOL	FEVER	19-Nov-04	20-Nov-04
34	458	PM	20-Nov-04	MULTI VITAMIN	SUPPLEMENTARY	19-Nov-04	11-Dec-04
34	458	PM	20-Nov-04	ARTESUNATE	MALARIA	26-Nov-04	28-Nov-04
34	458	PM	20-Nov-04	SULFADOXINE		26-Nov-04	
34	458	PM	20-Nov-04	PYRIMETHAMINE		26-Nov-04	
34	459	Combination	20-Nov-04	MEASLES VACC	PROPHYLAXIS	20-Nov-04	
34	459	Combination	20-Nov-04	MEASLES VACC	PROPHYLAXIS	7-Dec-04	
34	459	Combination	20-Nov-04	VITAMIN A	SUPPLEMENTARY	20-Nov-04	
34	459	Combination	20-Nov-04	FOLIC ACID	ANAEMIA	20-Nov-04	6-Dec-04
34	459	Combination	20-Nov-04	FERROUS SULFATE	ANAEMIA	20-Nov-04	6-Dec-04
34	459	Combination	20-Nov-04	MULTI VITS	SUPPLEMENTARY	20-Nov-04	6-Dec-04
34	459	Combination	20-Nov-04	PARACETAMOL	FEVER	20-Nov-04	22-Nov-04
34	460	PM	24-Nov-04	MEASLES VACC.	PROPHYLAXIS	23-Nov-04	
34	460	PM	24-Nov-04	PARACETAMOL	FEVER	22-Nov-04	24-Nov-04
34	460	PM	24-Nov-04	MULTI VITS	SUPPLEMENTARY	22-Nov-04	12-Jan-05
34	460	PM	24-Nov-04	FOLIC ACID	ANAEMIA	22-Nov-04	12-Jan-05
34	460	PM	24-Nov-04	FERROUS SULFATE	ANAEMIA	22-Nov-04	12-Jan-05
34	460	PM	24-Nov-04	MEASLES VACC	PROPHYLAXIS	14-Dec-04	
34	460	PM	24-Nov-04	VITAMIN A	SUPPLEMENTARY	22-Nov-04	
34	460	PM	24-Nov-04	AMOXICILLIN	OTITIS MEDIA	22-Nov-04	26-Nov-04
34	461	SSG	26-Nov-04	VITAMIN A	SUPPLEMENTARY	24-Nov-04	
34	461	SSG	26-Nov-04	FOLIC ACID	ANAEMIA	24-Nov-04	10-Dec-04
34	461	SSG	26-Nov-04	FERROUS SULPHATE	ANAEMIA	24-Nov-04	10-Dec-04
34	461	SSG	26-Nov-04	MULTI VITS	SUPPLEMENTARY	24-Nov-04	10-Dec-04

Centre Number	Patient Number	Treatment	Treatment start date	Concomitant Medication	Diagnosis	Start date	End date
34	461	SSG	26-Nov-04	CEFTRIAXONE	PNEUMONIA	24-Nov-04	2-Dec-04
34	461	SSG	26-Nov-04	PARACETAMOL	CHEST PAIN	25-Nov-04	27-Nov-04
34	461	SSG	26-Nov-04	GELATINE POLYSUCCINATE 4%	EPISTAXIS	28-Nov-04	
34	461	SSG	26-Nov-04	VITAMIN K	EPISTAXIS	28-Nov-04	29-Nov-04
34	461	SSG	26-Nov-04	SALBUTAMOL		29-Nov-04	
34	461	SSG	26-Nov-04	BLOOD TRANSFUSION		30-Nov-04	
34	461	SSG	26-Nov-04	BLOOD TRANSFUSION		10-Dec-04	
34	461	SSG	26-Nov-04	CIPROFLOXACINE		3-Dec-04	9-Dec-04
34	461	SSG	26-Nov-04	SPIRONOLACTON		9-Dec-04	10-Dec-04
34	461	SSG	26-Nov-04	HYDROCORTISON		9-Dec-04	
34	461	SSG	26-Nov-04	ETHAMBUTOL		9-Dec-04	
34	461	SSG	26-Nov-04	RIFANPICINE		9-Dec-04	
34	461	SSG	26-Nov-04	ISOMIAZIOLE		9-Dec-04	
34	461	SSG	26-Nov-04	PYRAZINAMIDE		9-Dec-04	
34	462	SSG	25-Nov-04	MEASLES VACC.	PROPHYLAXIS	25-Nov-04	
34	462	SSG	25-Nov-04	MEASLES VACC.	PROPHYLAXIS	24-Dec-04	
34	462	SSG	25-Nov-04	PARACETAMOL	FEVER	25-Nov-04	27-Nov-04
34	462	SSG	25-Nov-04	VITAMIN A	SUPPLEMENTARY	25-Nov-04	
34	462	SSG	25-Nov-04	FOLIC ACID	ANAEMIA	25-Nov-04	24-Dec-04
34	462	SSG	25-Nov-04	FERROUS SULPHATE	ANAEMIA	25-Nov-04	24-Dec-04
34	462	SSG	25-Nov-04	MULTI VITS	SUPPLEMENTARY	25-Nov-04	24-Dec-04
34	463	Combination	27-Nov-04	VITAMIN A	SUPPLEMENTARY	25-Nov-04	
34	463	Combination	27-Nov-04	FOLIC ACID	ANAEMIA	25-Nov-04	14-Dec-04
34	463	Combination	27-Nov-04	FERROUS SULFATE	ANAEMIA	25-Nov-04	14-Dec-04
34	463	Combination	27-Nov-04	MULTI VITS	SUPPLEMENTARY	25-Nov-04	14-Dec-04
34	463	Combination	27-Nov-04	PARACETAMOL	FEVER	25-Nov-04	28-Nov-04
34	463	Combination	27-Nov-04	TETRACYCLIN EYE OINTMENT	CONJUNCTIVITIS	27-Nov-04	29-Nov-04
34	463	Combination	27-Nov-04	MEASLES VACC.	PROPHYLAXIS	28-Nov-04	
34	463	Combination	27-Nov-04	MEASLES VACC.	PROPHYLAXIS	14-Dec-04	
34	464	SSG	27-Nov-04	VITAMIN A	SUPPLEMENTARY	26-Nov-04	
34	464	SSG	27-Nov-04	FOLIC ACID	ANAEMIA	26-Nov-04	26-Dec-04
34	464	SSG	27-Nov-04	FERROUS SULPHATE	ANAEMIA	26-Nov-04	26-Dec-04
34	464	SSG	27-Nov-04	MULTI VITS	SUPPLEMENTARY	26-Nov-04	26-Dec-04
34	464	SSG	27-Nov-04	PARACETAMOL	FEVER	26-Nov-04	28-Nov-04
34	465	SSG	29-Nov-04	VITAMIN A	SUPPLEMENTARY	27-Nov-04	
34	465	SSG	29-Nov-04	FOLIC ACID	ANAEMIA	27-Nov-04	27-Dec-04
34	465	SSG	29-Nov-04	FERROUS SULFATE	ANAEMIA	27-Nov-04	27-Dec-04
34	465	SSG	29-Nov-04	MULTI VITS	SUPPLEMENTARY	27-Nov-04	27-Dec-04
34	465	SSG	29-Nov-04	PARACETAMOL	FEVER	1-Dec-04	
34	465	SSG	29-Nov-04	CEFTRIAXONE	PNEUMONIA	27-Nov-04	1-Dec-04
34	465	SSG	29-Nov-04	TETRACYCLINE EYE OINTMENT	CONJUNCTIVITIS	25-Dec-04	27-Dec-04
34	466	Combination	30-Nov-04	VITAMIN A	SUPPLEMENTARY	28-Nov-04	
34	466	Combination	30-Nov-04	FOLIC ACID	ANAEMIA	4-Dec-04	15-Jan-05
34	466	Combination	30-Nov-04	FERROUS SULFATE	ANAEMIA	28-Nov-04	15-Jan-05
34	466	Combination	30-Nov-04	MULTI VITS	SUPPLEMENTARY	28-Nov-04	15-Jan-05
34	466	Combination	30-Nov-04	ARTESUNATE	MALARIA	28-Nov-04	30-Nov-04
34	466	Combination	30-Nov-04	SULFADOXINE	MALARIA	28-Nov-04	
34	466	Combination	30-Nov-04	PYRIMETHAMINE	MALARIA	28-Nov-04	
34	466	Combination	30-Nov-04	PARACETAMOL	FEVER	28-Nov-04	29-Nov-04

Centre Number	Patient Number	Treatment	Treatment start date	Concomitant Medication	Diagnosis	Start date	End date
34	467	PM	29-Nov-04	VITAMIN A	SUPPLEMENTARY	29-Nov-04	
34	467	PM	29-Nov-04	FOLIC ACID	ANAEMIA	29-Nov-04	19-Dec-04
34	467	PM	29-Nov-04	FERROUS SULFATE	ANAEMIA	29-Nov-04	19-Dec-04
34	467	PM	29-Nov-04	PARACETAMOL	FEVER	28-Nov-04	19-Dec-04
34	467	PM	29-Nov-04	MEASLES VACCINE	PROPHYLAXIS	29-Nov-04	
34	467	PM	29-Nov-04	ARTESUNATE	MALARIA	8-Dec-04	10-Dec-04
34	467	PM	29-Nov-04	SULFADOXINE	MALARIA	8-Dec-04	
34	467	PM	29-Nov-04	PYRIMETHAMINE	MALARIA	8-Dec-04	
34	467	PM	29-Nov-04	MEASLES VACC.		19-Dec-04	
34	468	Combination	29-Nov-04	VITAMIN A	SUPPLEMENTARY	29-Nov-04	
34	468	Combination	29-Nov-04	FOLIC ACID	ANAEMIA	29-Nov-04	15-Dec-04
34	468	Combination	29-Nov-04	FERROUS SULFATE	ANAEMIA	29-Nov-04	15-Dec-04
34	468	Combination	29-Nov-04	MULTI VITS	SUPPLEMENTARY	29-Nov-04	15-Dec-04
34	468	Combination	29-Nov-04	PARACETAMOL	FEVER	28-Nov-04	30-Nov-04
34	469	SSG	29-Nov-04	VITAMIN A	SUPPLEMENTARY	29-Nov-04	
34	469	SSG	29-Nov-04	FOLIC ACID	ANAEMIA	29-Nov-04	27-Dec-04
34	469	SSG	29-Nov-04	FERROUS SULPHATE	ANAEMIA	29-Nov-04	27-Dec-04
34	469	SSG	29-Nov-04	MULTI VITS	SUPPLEMENTARY	29-Nov-04	27-Dec-04
34	469	SSG	29-Nov-04	PARACETAMOL	FEVER	28-Nov-04	3-Dec-04
34	469	SSG	29-Nov-04	MEASLES VACCINE	PROPHYLAXIS	3-Dec-04	
34	469	SSG	29-Nov-04	ARTESUNATE	MALARIA	9-Dec-04	11-Dec-04
34	469	SSG	29-Nov-04	SULFADOXINE	MALARIA	9-Dec-04	
34	469	SSG	29-Nov-04	PYRIMETHAMINE		9-Dec-04	
34	470	Combination	29-Nov-04	VITAMIN A	SUPPLEMENTARY	29-Nov-04	
34	470	Combination	29-Nov-04	FOLIC ACID	ANAEMIA	29-Nov-04	15-Dec-04
34	470	Combination	29-Nov-04	FERROUS SULPHATE	ANAEMIA	29-Nov-04	15-Dec-04
34	470	Combination	29-Nov-04	MULTI VITS	SUPPLEMENTARY	29-Nov-04	15-Dec-04
34	470	Combination	29-Nov-04	PARACETAMOL	FEVER	29-Nov-04	4-Dec-04
34	470	Combination	29-Nov-04	MEASLES VACCINE	PROPHYLAXIS	29-Nov-04	
34	470	Combination	29-Nov-04	MEASLES VACCINE	PROPHYLAXIS	17-Dec-04	
34	471	PM	29-Nov-04	VITAMIN A	SUPPLEMENTARY	29-Nov-04	
34	471	PM	29-Nov-04	FOLIC ACID	ANAEMIA	29-Nov-04	18-Jan-05
34	471	PM	29-Nov-04	FERROUS SULFATE	ANAEMIA	29-Nov-04	18-Jan-05
34	471	PM	29-Nov-04	MULTI VITS	SUPPLEMENTARY	29-Nov-04	18-Jan-05
34	471	PM	29-Nov-04	PARACETAMOL	FEVER	29-Nov-04	30-Nov-04
34	471	PM	29-Nov-04	MEASLES VACCINE	PROPHYLAXIS	3-Dec-04	
34	471	PM	29-Nov-04	ARTESUNATE	MALARIA	5-Dec-04	7-Dec-04
34	471	PM	29-Nov-04	SULFADOXINE	MALARIA	5-Dec-04	
34	471	PM	29-Nov-04	PYRIMETHAMINE		5-Dec-04	
34	471	PM	29-Nov-04	MEASLES VACC.		19-Dec-04	
34	472	SSG	1-Dec-04	VITAMIN A	SUPPLEMENTARY	1-Dec-04	
34	472	SSG	1-Dec-04	FOLIC ACID	ANAEMIA	1-Dec-04	29-Dec-04
34	472	SSG	1-Dec-04	FERROUS SULPHATE	ANAEMIA	1-Dec-04	29-Dec-04
34	472	SSG	1-Dec-04	MULTI VITS	SUPPLEMENTARY	1-Dec-04	29-Dec-04
34	472	SSG	1-Dec-04	PARACETAMOL	FEVER	1-Dec-04	2-Dec-04
34	472	SSG	1-Dec-04	MEASLES VACCINE	PROPHYLAXIS	4-Dec-04	
34	472	SSG	1-Dec-04	MEASLES VACC.	PROPHYLAXIS	30-Dec-04	
34	473	PM	3-Dec-04	VITAMIN A	SUPPLEMENTARY	1-Dec-04	
34	473	PM	3-Dec-04	FOLIC ACID	ANAEMIA	1-Dec-04	22-Jan-05
34	473	PM	3-Dec-04	FERROUS SULPHATE	ANAEMIA	1-Dec-04	22-Jan-05
34	473	PM	3-Dec-04	MULTI VITS	SUPPLEMENTARY	1-Dec-04	22-Jan-05
34	473	PM	3-Dec-04	PARACETAMOL	FEVER	1-Dec-04	2-Dec-04
34	473	PM	3-Dec-04	MEASLES VACC.	PROPHYLAXIS	5-Dec-04	
34	473	PM	3-Dec-04	MEASLES VACC.	PROPHYLAXIS	24-Dec-04	
34	474	Combination	4-Dec-04	MEASLES VACC.	PROPHYLAXIS	4-Dec-04	
34	474	Combination	4-Dec-04	VITAMIN A	SUPPLEMENTARY	4-Dec-04	
34	474	Combination	4-Dec-04	FOLIC ACID	ANAEMIA	4-Dec-04	21-Dec-04

Centre Number	Patient Number	Treatment	Treatment start date	Concomitant Medication	Diagnosis	Start date	End date
34	474	Combination	4-Dec-04	FERROUS SULFATE	ANAEMIA	4-Dec-04	21-Dec-04
34	474	Combination	4-Dec-04	MULTI VITS	SUPPLEMENTARY	4-Dec-04	21-Dec-04
34	474	Combination	4-Dec-04	MEASLES VACC.	PROPHYLAXIS	21-Dec-04	
34	475	PM	5-Dec-04	VITAMIN A	SUPPLEMENTARY	4-Dec-04	
34	475	PM	5-Dec-04	FOLIC ACID	ANAEMIA	4-Dec-04	25-Dec-04
34	475	PM	5-Dec-04	FERROUS SULFATE	ANAEMIA	4-Dec-04	25-Dec-04
34	475	PM	5-Dec-04	MULTI VITS	SUPPLEMENTARY	4-Dec-04	25-Dec-04
34	475	PM	5-Dec-04	PARACETAMOL	FEVER	4-Dec-04	
34	475	PM	5-Dec-04	MEASLES VACC.	PROPHYLAXIS	5-Dec-04	
34	475	PM	5-Dec-04	MEASLES VACC.	PROPHYLAXIS	25-Dec-04	
34	476	PM	5-Dec-04	VITAMIN A	SUPPLEMENTARY	4-Dec-04	
34	476	PM	5-Dec-04	FOLIC ACID	ANAEMIA	4-Dec-04	25-Dec-04
34	476	PM	5-Dec-04	FERROUS SULFATE	ANAEMIA	4-Dec-04	25-Dec-04
34	476	PM	5-Dec-04	MULTI VITS	SUPPLEMENTARY	4-Dec-04	25-Dec-04
34	476	PM	5-Dec-04	PARACETAMOL	FEVER	4-Dec-04	
34	476	PM	5-Dec-04	MEASLES VACC.	PROPHYLAXIS	5-Dec-04	
34	476	PM	5-Dec-04	MEASLES VACC.	PROPHYLAXIS	25-Dec-04	
34	477	SSG	6-Dec-04	VITAMIN A	SUPPLEMENTARY	6-Dec-04	
34	477	SSG	6-Dec-04	FOLIC ACID	ANAEMIA	6-Dec-04	4-Jan-05
34	477	SSG	6-Dec-04	FERROUS SULPHATE	ANAEMIA	6-Dec-04	4-Jan-05
34	477	SSG	6-Dec-04	MULTI VITS	SUPPLEMENTARY	6-Dec-04	4-Jan-05
34	477	SSG	6-Dec-04	PARACETAMOL	FEVER	6-Dec-04	7-Dec-04
34	477	SSG	6-Dec-04	MEASLES VACC.	PROPHYLAXIS	6-Dec-04	
34	477	SSG	6-Dec-04	ARTESUNATE	MALARIA	26-Dec-04	28-Dec-04
34	477	SSG	6-Dec-04	PYRIMETHAMINE	MALARIA	26-Dec-04	
34	477	SSG	6-Dec-04	SULFADOXINE		26-Dec-04	
34	477	SSG	6-Dec-04	ARTESUNATE	MALARIA	5-Jun-05	7-Jun-05
34	477	SSG	6-Dec-04	SULFADOXINE	MALARIA	5-Jun-05	
34	477	SSG	6-Dec-04	PYRIMETHAMINE	MALARIA	5-Jun-05	
34	477	SSG	6-Dec-04	PARACETAMOL	FEVER	5-Jun-05	
34	478	Combination	7-Dec-04	VITAMIN A	SUPPLEMENTARY	6-Dec-04	
34	478	Combination	7-Dec-04	FERROUS SULFATE	ANAEMIA	6-Dec-04	23-Dec-04
34	478	Combination	7-Dec-04	FOLIC ACID	ANAEMIA	6-Dec-04	23-Dec-04
34	478	Combination	7-Dec-04	MULTI VITS	SUPPLEMENTARY	6-Dec-04	23-Dec-04
34	478	Combination	7-Dec-04	PARACETAMOL	FEVER	6-Dec-04	7-Dec-04
34	478	Combination	7-Dec-04	MEASLES VACC.	PROPHYLAXIS	7-Dec-04	
34	478	Combination	7-Dec-04	MEASLES VACC.	PROPHYLAXIS	23-Dec-04	
34	479	SSG	8-Dec-04	MEASLES VACC.	PROPHYLAXIS	7-Dec-04	
34	479	SSG	8-Dec-04	VITAMIN A	SUPPLEMENTARY	8-Dec-04	
34	479	SSG	8-Dec-04	FOLIC ACID	ANAEMIA	8-Dec-04	6-Jan-05
34	479	SSG	8-Dec-04	FERROUS SULPHATE	ANAEMIA	8-Dec-04	6-Jan-05
34	479	SSG	8-Dec-04	PARACETAMOL	FEVER	8-Dec-04	10-Dec-04
34	479	SSG	8-Dec-04	MULTI VITS	SUPPLEMENTARY	8-Dec-04	6-Jan-05
34	480	SSG	9-Dec-04	VITAMIN A	SUPPLEMENTARY	9-Dec-04	
34	480	SSG	9-Dec-04	MEASLES VACC.	PROPHYLAXIS	9-Dec-04	
34	480	SSG	9-Dec-04	FOLIC ACID	ANAEMIA	9-Dec-04	7-Jan-05
34	480	SSG	9-Dec-04	FERROUS SULPHATE	ANAEMIA	9-Dec-04	7-Jan-05
34	480	SSG	9-Dec-04	MULTI VITS	SUPPLEMENTARY	9-Dec-04	7-Jan-05
34	480	SSG	9-Dec-04	MEASLES VACC.	PROPHYLAXIS	7-Jan-05	
34	481	Combination	9-Dec-04	MEASLES VACC.	PROPHYLAXIS	9-Dec-04	
34	481	Combination	9-Dec-04	FOLIC ACID	ANAEMIA	9-Dec-04	25-Dec-04
34	481	Combination	9-Dec-04	FERROUS SULPHATE	ANAEMIA	9-Dec-04	25-Dec-04
34	481	Combination	9-Dec-04	VITAMIN A	SUPPLEMENTARY	9-Dec-04	
34	481	Combination	9-Dec-04	MULTI VITAMIN	SUPPLEMENTARY	9-Dec-04	25-Dec-04
34	481	Combination	9-Dec-04	PARACETAMOL	FEVER	9-Dec-04	
34	481	Combination	9-Dec-04	MEASLES VACC.	PROPHYLAXIS	25-Dec-04	
34	482	Combination	10-Dec-04	VITAMIN A	SUPPLEMENTARY	10-Dec-04	

Centre Number	Patient Number	Treatment	Treatment start date	Concomitant Medication	Diagnosis	Start date	End date
34	482	Combination	10-Dec-04	FOLIC ACID	SUPPLEMENTARY	10-Dec-04	26-Dec-04
34	482	Combination	10-Dec-04	FERROUS SULPHATE	SUPPLEMENTARY	10-Dec-04	26-Dec-04
34	482	Combination	10-Dec-04	MULTI VITS	SUPPLEMENTARY	10-Dec-04	26-Dec-04
34	482	Combination	10-Dec-04	PARACETAMOL	FEVER	9-Dec-04	10-Dec-04
34	482	Combination	10-Dec-04	ARTESUNATE	MALARIA	14-Mar-05	16-Mar-05
34	482	Combination	10-Dec-04	SULFADOXINE	MALARIA	14-Mar-05	
34	482	Combination	10-Dec-04	PYRIMETHAMINE	MALARIA	14-Mar-05	
34	482	Combination	10-Dec-04	PARACETAMOL	FEVER	14-Mar-05	
34	483	PM	10-Dec-04	VITAMIN A	SUPPLEMENTARY	10-Dec-04	
34	483	PM	10-Dec-04	FOLIC ACID	SUPPLEMENTARY	10-Dec-04	29-Dec-04
34	483	PM	10-Dec-04	FERROUS SULPHATE	SUPPLEMENTARY	10-Dec-04	29-Dec-04
34	483	PM	10-Dec-04	MULTI VITS	SUPPLEMENTARY	10-Dec-04	29-Dec-04
34	483	PM	10-Dec-04	PARACETAMOL	FEVER	9-Dec-04	13-Dec-04
34	483	PM	10-Dec-04	AMOXICILLIN	PNEUMONIA	13-Dec-04	19-Dec-04
34	484	PM	12-Dec-04	VITAMIN A	SUPPLEMENTARY	11-Dec-04	
34	484	PM	12-Dec-04	MULTI VITS	SUPPLEMENTARY	11-Dec-04	16-Jan-05
34	484	PM	12-Dec-04	FOLIC ACID	ANAEMIA	11-Dec-04	16-Jan-05
34	484	PM	12-Dec-04	FERROUS SULPHATE	ANAEMIA	11-Dec-04	16-Jan-05
34	484	PM	12-Dec-04	PARACETAMOL	FEVER	11-Dec-04	18-Dec-04
34	484	PM	12-Dec-04	MEASLES VACC	PROPHYLAXIS	13-Dec-04	
34	485	Combination	12-Dec-04	VITAMIN A	PROPHYLAXIS	11-Dec-04	
34	485	Combination	12-Dec-04	FERROUS SULPHATE	ANAEMIA	11-Dec-04	28-Dec-04
34	485	Combination	12-Dec-04	FOLIC ACID	ANAEMIA	11-Dec-04	28-Dec-04
34	485	Combination	12-Dec-04	MULTI VIT	SUPPLEMENTARY	11-Dec-04	28-Dec-04
34	485	Combination	12-Dec-04	PARACETAMOL	FEVER	11-Dec-04	15-Dec-04
34	485	Combination	12-Dec-04	MEASLES VACC.	PROPHYLAXIS	16-Dec-04	
34	485	Combination	12-Dec-04	MEASLES VACC.	PROPHYLAXIS	28-Dec-04	
34	485	Combination	12-Dec-04	ARTESUNATE	MALARIA	1-May-05	3-May-05
34	485	Combination	12-Dec-04	SULFADOXINE	MALARIA	1-May-05	
34	485	Combination	12-Dec-04	PYRIMETHAMINE	MALARIA	1-May-05	
34	485	Combination	12-Dec-04	PARACETAMOL	FEVER	1-May-05	3-May-05
34	486	Combination	13-Dec-04	VITAMIN A	SUPPLEMENTARY	11-Dec-04	
34	486	Combination	13-Dec-04	AMOXICILLIN	PNEUMONIA	11-Dec-04	15-Dec-04
34	486	Combination	13-Dec-04	FERROUS SULPHATE	ANAEMIA	11-Dec-04	29-Dec-04
34	486	Combination	13-Dec-04	FOLIC ACID	ANAEMIA	11-Dec-04	29-Dec-04
34	486	Combination	13-Dec-04	MULTI VIT	SUPPLEMENTARY	11-Dec-04	29-Dec-04
34	486	Combination	13-Dec-04	PARACETAMOL	FEVER	11-Dec-04	12-Dec-04
34	486	Combination	13-Dec-04	MEASLES VACC.	PROPHYLAXIS	13-Dec-04	
34	486	Combination	13-Dec-04	MEASLES VACC.	PROPHYLAXIS	30-Dec-04	
34	487	SSG	13-Dec-04	PARACETAMOL	FEVER	13-Dec-04	14-Dec-04
34	487	SSG	13-Dec-04	MEASLES VACC.	PROPHYLAXIS	18-Dec-04	
34	487	SSG	13-Dec-04	VITAMIN A	SUPPLEMENTARY	13-Dec-04	
34	487	SSG	13-Dec-04	FOLIC ACID	ANAEMIA	13-Dec-04	12-Jan-05
34	487	SSG	13-Dec-04	FERROUS SULFATE	ANAEMIA	13-Dec-04	12-Jan-05
34	487	SSG	13-Dec-04	MULTI VITS	SUPPLEMENTARY	13-Dec-04	12-Jan-05
34	487	SSG	13-Dec-04	PYRIMETHAMINE	MALARIA	2-Jan-05	
34	487	SSG	13-Dec-04	SULFADOXINE	MALARIA	2-Jan-05	
34	487	SSG	13-Dec-04	ARTESUNATE		2-Jan-05	4-Jan-05
34	487	SSG	13-Dec-04	MEASLES VACC.		12-Jan-05	
34	488	SSG	14-Dec-04	MEASLES VACC.	PROPHYLAXIS	18-Dec-04	
34	488	SSG	14-Dec-04	VITAMIN A	SUPPLEMENTARY	13-Dec-04	
34	488	SSG	14-Dec-04	FOLIC ACID	ANAEMIA	13-Dec-04	12-Jan-05
34	488	SSG	14-Dec-04	FERROUS SULFATE	ANAEMIA	13-Dec-04	12-Jan-05
34	488	SSG	14-Dec-04	MULTI VITS	SUPPLEMENTARY	13-Dec-04	12-Jan-05
34	488	SSG	14-Dec-04	PARACETAMOL	FEVER	13-Dec-04	
34	488	SSG	14-Dec-04	MEASLES VACC	PROPHYLAXIS	13-Jan-05	
34	489	SSG	16-Dec-04	MEASLES VACC.	PROPHYLAXIS	16-Dec-04	

Centre Number	Patient Number	Treatment	Treatment start date	Concomitant Medication	Diagnosis	Start date	End date
34	489	SSG	16-Dec-04	VITAMIN A	SUPPLEMENTARY	16-Dec-04	
34	489	SSG	16-Dec-04	FOLIC ACID	ANAEMIA	16-Dec-04	14-Jan-05
34	489	SSG	16-Dec-04	FERROUS SULPHATE	ANAEMIA	16-Dec-04	14-Jan-05
34	489	SSG	16-Dec-04	PARACETAMOL	FEVER	16-Dec-04	
34	489	SSG	16-Dec-04	MULTI VITS	SUPPLEMENTARY	16-Dec-04	14-Jan-05
34	489	SSG	16-Dec-04	MEASLES VACC	PROPHYLAXIS	13-Jan-05	
34	490	PM	16-Dec-04	VITAMIN A	PROPHYLAXIS	16-Dec-04	
34	490	PM	16-Dec-04	FOLIC ACID	ANAEMIA	16-Dec-04	5-Jan-05
34	490	PM	16-Dec-04	FERROUS SULFATE	ANAEMIA	16-Dec-04	5-Jan-05
34	490	PM	16-Dec-04	MULTI VITS	SUPPLEMENTARY	16-Dec-04	5-Jan-05
34	490	PM	16-Dec-04	PARACETAMOL	FEVER	16-Dec-04	
34	490	PM	16-Dec-04	MEASLES VACC.	PROPHYLAXIS	17-Dec-04	
34	490	PM	16-Dec-04	MEASLES VACC.	PROPHYLAXIS	7-Jan-05	
34	491	PM	17-Dec-04	VITAMIN A	SUPPLEMENTARY	16-Dec-04	
34	491	PM	17-Dec-04	FOLIC ACID	ANAEMIA	16-Dec-04	7-Jan-05
34	491	PM	17-Dec-04	FERROUS SULPHATE	ANAEMIA	16-Dec-04	7-Jan-05
34	491	PM	17-Dec-04	MULTI VITS	SUPPLEMENTARY	16-Dec-04	7-Jan-05
34	491	PM	17-Dec-04	PARACETAMOL	FEVER	16-Dec-04	
34	491	PM	17-Dec-04	SULFAMETHOXAZOLE	PROPHYLAXIS	24-Dec-04	
34	491	PM	17-Dec-04	TRIMETHOPRIM	PROPHYLAXIS	24-Dec-04	
34	492	PM	19-Dec-04	VITAMIN A	PROPHYLAXIS	16-Dec-04	
34	492	PM	19-Dec-04	TINIDAZOL	MUCOID DIARRHEA	16-Dec-04	18-Dec-04
34	492	PM	19-Dec-04	FOLIC ACID	SUPPLEMENTARY	16-Dec-04	9-Jan-05
34	492	PM	19-Dec-04	FERROUS SULFATE	SUPPLEMENTARY	16-Dec-04	9-Jan-05
34	492	PM	19-Dec-04	MULTI VITS	SUPPLEMENTARY	16-Dec-04	9-Jan-05
34	492	PM	19-Dec-04	RINGER LACFELE	DEHYDRATION	16-Dec-04	
34	492	PM	19-Dec-04	HYOSCINE BUTYLBROMIDE	ABDOMINAL PAIN	16-Dec-04	18-Dec-04
34	492	PM	19-Dec-04	CIPROFLOXACIN	SUSPECTED DYSENTRY	17-Dec-04	21-Dec-04
34	492	PM	19-Dec-04	BENZOIC ACID		18-Dec-04	25-Dec-04
34	492	PM	19-Dec-04	SALICYLIC ACID		18-Dec-04	25-Dec-04
34	492	PM	19-Dec-04	METRONIDAZOL	AMOEBIASIS	30-Mar-05	5-Apr-05
34	492	PM	19-Dec-04	CIPROFLOXACIN	DYSENTRY	6-Apr-05	11-Apr-05
34	493	SSG	17-Dec-04	MEASLES VACC.	PROPHYLAXIS	17-Dec-04	
34	493	SSG	17-Dec-04	VITAMIN A	SUPPLEMENTARY	17-Dec-04	
34	493	SSG	17-Dec-04	FOLIC ACID	ANAEMIA	17-Dec-04	15-Jan-05
34	493	SSG	17-Dec-04	FERROUS SULFATE	ANAEMIA	17-Dec-04	15-Jan-05
34	493	SSG	17-Dec-04	MULTI VITS	SUPPLEMENTARY	17-Dec-04	15-Jan-05
34	493	SSG	17-Dec-04	PARACETAMOL	FEVER	17-Dec-04	19-Dec-04
34	493	SSG	17-Dec-04	GLUCOSE	HYPOGLYCEMIA	17-Dec-04	
34	493	SSG	17-Dec-04	ARTESUNATE	MALARIA	1-Jan-05	3-Jan-05
34	493	SSG	17-Dec-04	SULFADOXINE		1-Jan-05	
34	493	SSG	17-Dec-04	PYRIMETHAMINE		1-Jan-05	
34	493	SSG	17-Dec-04	MEASLES VACC.		13-Jan-05	
34	493	SSG	17-Dec-04	SSG	PKDL WITH MUCOSAL INVOLVEMENT	12-Apr-05	18-May-05
34	494	Combination	17-Dec-04	VITAMIN A	PROPHYLAXIS	17-Dec-04	
34	494	Combination	17-Dec-04	FOLIC ACID	ANAEMIA	17-Dec-04	3-Jan-05
34	494	Combination	17-Dec-04	FERROUS SULFATE	ANAEMIA	17-Dec-04	3-Jan-05
34	494	Combination	17-Dec-04	MULTI VITS	SUPPLEMENTARY	17-Dec-04	3-Jan-05
34	494	Combination	17-Dec-04	PARACETAMOL	HEADACHE	17-Dec-04	
34	494	Combination	17-Dec-04	TETRACYCLINE EYE OINTMENT	CONJUNCTIVITIS	3-Jun-05	8-Jun-05

Centre Number	Patient Number	Treatment	Treatment start date	Concomitant Medication	Diagnosis	Start date	End date
34	495	SSG	18-Dec-04	VITAMIN A	SUPPLEMENTARY	17-Dec-04	
34	495	SSG	18-Dec-04	FOLIC ACID	ANAEMIA	17-Dec-04	17-Jan-05
34	495	SSG	18-Dec-04	FERROUS SULFATE	ANAEMIA	17-Dec-04	17-Jan-05
34	495	SSG	18-Dec-04	MULTI VITS	SUPPLEMENTARY	17-Dec-04	17-Jan-05
34	495	SSG	18-Dec-04	PARACETAMOL	FEVER	17-Dec-04	18-Dec-05
34	495	SSG	18-Dec-04	MEASLES VACC.	PROPHYLAXIS	18-Dec-04	
34	495	SSG	18-Dec-04	PROMETHAZINE	ALLERGIC REACTION	24-Dec-04	
34	495	SSG	18-Dec-04	MEASLES VACC	PROPHYLAXIS	13-Jan-05	
34	496	SSG	19-Dec-04	VITAMIN A	SUPPLEMENTARY	18-Dec-04	
34	496	SSG	19-Dec-04	FOLIC ACID	ANAEMIA	18-Dec-04	18-Jan-05
34	496	SSG	19-Dec-04	FERROUS SULFATE	ANAEMIA	18-Dec-04	18-Jan-05
34	496	SSG	19-Dec-04	MULTI VITS	SUPPLEMENTARY	18-Dec-04	18-Jan-05
34	496	SSG	19-Dec-04	PARACETAMOL	FEVER	18-Dec-04	20-Dec-04
34	497	PM	21-Dec-04	VITAMIN A	PROPHYLAXIS	21-Dec-04	
34	497	PM	21-Dec-04	FOLIC ACID	ANAEMIA	21-Dec-04	11-Jan-05
34	497	PM	21-Dec-04	FERROUS SULPHATE	ANAEMIA	21-Dec-04	11-Jan-05
34	497	PM	21-Dec-04	MULTI VITS	SUPPLEMENTARY	21-Dec-04	11-Jan-05
34	497	PM	21-Dec-04	PARACETAMOL	FEVER	20-Dec-04	22-Dec-04
34	498	SSG	21-Dec-04	MEASLES VACC.	PROPHYLAXIS	21-Dec-04	
34	498	SSG	21-Dec-04	PARACETAMOL	FEVER	20-Dec-04	21-Dec-04
34	498	SSG	21-Dec-04	VITAMIN A	SUPPLEMENTARY	21-Dec-04	
34	498	SSG	21-Dec-04	FOLIC ACID	ANAEMIA	21-Dec-04	20-Jan-05
34	498	SSG	21-Dec-04	FERROUS SULPHATE	ANAEMIA	21-Dec-04	20-Jan-05
34	498	SSG	21-Dec-04	MULTI VITS	SUPPLEMENTARY	21-Dec-04	20-Jan-05
34	498	SSG	21-Dec-04	AMOXICILLIN	PNEUMONIA	18-Jan-05	20-Jan-05
34	498	SSG	21-Dec-04	MEASLES VACC	PROPHYLAXIS	20-Jan-05	
34	499	Combination	21-Dec-04	MEASLES VACC	PROPHYLAXIS	21-Dec-04	
34	499	Combination	21-Dec-04	VITAMIN A	SUPPLEMENTARY	21-Dec-04	
34	499	Combination	21-Dec-04	MULTI VITS	SUPPLEMENTARY	21-Dec-04	6-Jan-05
34	499	Combination	21-Dec-04	FOLIC ACID	ANAEMIA	21-Dec-04	6-Jan-05
34	499	Combination	21-Dec-04	FERROUS SULFATE	ANAEMIA	21-Dec-04	6-Jan-05
34	499	Combination	21-Dec-04	PARACETAMOL	FEVER	20-Dec-04	21-Dec-04
34	499	Combination	21-Dec-04	GENTIAN VIOLET	MUCOSAL LESION IN MOUTH	30-Dec-04	3-Jan-05
34	499	Combination	21-Dec-04	MEASLES VACC	PROPHYLAXIS	6-Jan-05	
34	500	PM	22-Dec-04	MEASLES VACC.	PROPHYLAXIS	21-Dec-04	
34	500	PM	22-Dec-04	VITAMIN A	SUPPLEMENTARY	21-Dec-04	
34	500	PM	22-Dec-04	MULTI VITS	SUPPLEMENTARY	21-Dec-04	11-Jan-05
34	500	PM	22-Dec-04	FOLIC ACID	ANAEMIA	22-Dec-04	11-Jan-05
34	500	PM	22-Dec-04	FERROUS SULFATE	ANAEMIA	27-Dec-04	11-Jan-05
34	500	PM	22-Dec-04	PARACETAMOL	FEVER	20-Dec-04	
34	500	PM	22-Dec-04	ARTESUNATE	MALARIA	20-Dec-04	22-Dec-04
34	500	PM	22-Dec-04	PYRIMETHAMINE	MALARIA	20-Dec-04	
34	500	PM	22-Dec-04	SALFADOXINE		20-Dec-04	
34	500	PM	22-Dec-04	MEASLES VACC		11-Jan-05	
34	501	Combination	22-Dec-04	MEASLES VACC.	PROPHYLAXIS	22-Dec-04	
34	501	Combination	22-Dec-04	VITAMIN A	SUPPLEMENTARY	21-Dec-04	
34	501	Combination	22-Dec-04	FOLIC ACID	ANAEMIA	21-Dec-04	7-Jan-05
34	501	Combination	22-Dec-04	FERROUS SULFATE	ANAEMIA	21-Dec-04	7-Jan-05
34	501	Combination	22-Dec-04	MULTI VITS	ANAEMIA	21-Dec-04	7-Jan-05
34	501	Combination	22-Dec-04	PARACETAMOL	FEVER	21-Dec-04	23-Dec-04
34	501	Combination	22-Dec-04	MEASLES VACC	PROPHYLAXIS	8-Jan-05	

Centre Number	Patient Number	Treatment	Treatment start date	Concomitant Medication	Diagnosis	Start date	End date
34	502	Combination	23-Dec-04	MEASLES VACC.	PROPHYLAXIS	22-Dec-04	
34	502	Combination	23-Dec-04	VITAMIN A	SUPPLEMENTARY	22-Dec-04	
34	502	Combination	23-Dec-04	FOLIC ACID	ANAEMIA	22-Dec-04	9-Jan-05
34	502	Combination	23-Dec-04	FERROUS SULFATE	ANAEMIA	22-Dec-04	9-Jan-05
34	502	Combination	23-Dec-04	MULTI VITS	SUPPLEMENTARY	22-Dec-04	9-Jan-05
34	502	Combination	23-Dec-04	PARACETAMOL	FEVER	22-Dec-04	24-Dec-04
34	502	Combination	23-Dec-04	MEASLES VACC	PROPHYLAXIS	8-Jan-05	
34	503	PM	25-Dec-04	MEASLES VACC.	PROPHYLAXIS	25-Dec-04	
34	503	PM	25-Dec-04	VITAMIN A	SUPPLEMENTARY	24-Dec-04	
34	503	PM	25-Dec-04	MULTI VITS	SUPPLEMENTARY	24-Dec-04	15-Jan-05
34	503	PM	25-Dec-04	FOLIC ACID	ANAEMIA	24-Dec-04	15-Jan-05
34	503	PM	25-Dec-04	FERROUS SULFATE	ANAEMIA	24-Dec-04	15-Jan-05
34	503	PM	25-Dec-04	PARACETAMOL	FEVER	24-Dec-04	25-Dec-04
34	503	PM	25-Dec-04	MEASLES VACC	PROPHYLAXIS	15-Jan-05	
34	504	Combination	27-Dec-04	MEASLES VACC.	PROPHYLAXIS	27-Dec-04	
34	504	Combination	27-Dec-04	VITAMIN A	SUPPLEMENTARY	26-Dec-04	
34	504	Combination	27-Dec-04	FOLIC ACID	ANAEMIA	26-Dec-04	
34	504	Combination	27-Dec-04	FERROUS SULFATE	ANAEMIA	26-Dec-04	
34	504	Combination	27-Dec-04	MULTI VITS	SUPPLEMENTARY	26-Dec-04	
34	504	Combination	27-Dec-04	PARACETAMOL	FEVER	27-Dec-04	
34	504	Combination	27-Dec-04	MEASLES VACC	PROPHYLAXIS	12-Jan-05	
34	505	SSG	28-Dec-04	VITAMIN A	SUPPLEMENTARY	27-Dec-04	
34	505	SSG	28-Dec-04	FOLIC ACID	ANAEMIA	27-Dec-04	27-Jan-05
34	505	SSG	28-Dec-04	FERROUS SULFATE	ANAEMIA	27-Dec-04	27-Jan-05
34	505	SSG	28-Dec-04	MULTI VITS	SUPPLEMENTARY	27-Dec-04	27-Jan-05
34	505	SSG	28-Dec-04	CIPROFLOXACIN	UTI	3-Jan-05	10-Jan-05
34	505	SSG	28-Dec-04	PARACETAMOL	FEVER	27-Dec-04	28-Dec-04
34	506	SSG	31-Dec-04	VITAMIN A	SUPPLEMENTARY	30-Dec-04	
34	506	SSG	31-Dec-04	FOLIC ACID	ANAEMIA	30-Dec-04	29-Jan-05
34	506	SSG	31-Dec-04	FERROUS SULFATE	ANAEMIA	30-Dec-04	29-Jan-05
34	506	SSG	31-Dec-04	MULTI VITS	SUPPLEMENTARY	30-Dec-04	29-Jan-05
34	506	SSG	31-Dec-04	PARACETAMOL	FEVER	30-Dec-04	1-Jan-05
34	506	SSG	31-Dec-04	MEASLES VACC.	PROPHYLAXIS	3-Jan-05	
34	506	SSG	31-Dec-04	MEASLES VACC	PROPHYLAXIS	29-Jan-05	
34	507	SSG	31-Dec-04	VITAMIN A	SUPPLEMENTARY	30-Dec-04	
34	507	SSG	31-Dec-04	FOLIC ACID	ANAEMIA	30-Dec-04	30-Jan-05
34	507	SSG	31-Dec-04	FERROUS SULFATE	ANAEMIA	30-Dec-04	30-Jan-05
34	507	SSG	31-Dec-04	MULTI VITS	SUPPLEMENTARY	30-Dec-04	30-Jan-05
34	507	SSG	31-Dec-04	PARACETAMOL	FEVER	30-Dec-04	4-Jan-05
34	507	SSG	31-Dec-04	MEASLES VACC.	PROPHYLAXIS	6-Jan-05	
34	507	SSG	31-Dec-04	SULFADOXINE	MALARIA	15-Jan-05	
34	507	SSG	31-Dec-04	PYRIMETHAMINE	MALARIA	15-Jan-05	
34	507	SSG	31-Dec-04	ARTESUNATE		15-Jan-05	17-Jan-05
34	507	SSG	31-Dec-04	MEASLES VACC.		29-Jan-05	
34	508	PM	31-Dec-04	VITAMIN A	SUPPLEMENTARY	31-Dec-04	
34	508	PM	31-Dec-04	PARACETAMOL	FEVER	31-Dec-04	4-Jan-05
34	508	PM	31-Dec-04	FOLIC ACID	ANAEMIA	31-Dec-04	
34	508	PM	31-Dec-04	FERROUS SULFATE	ANAEMIA	31-Dec-04	
34	508	PM	31-Dec-04	MULTI VITS	SUPPLEMENTARY	31-Dec-04	
34	508	PM	31-Dec-04	MEASLES VACC.	PROPHYLAXIS	31-Dec-04	

Centre Number	Patient Number	Treatment	Treatment start date	Concomitant Medication	Diagnosis	Start date	End date
34	508	PM	31-Dec-04	MEASLES VACC	PROPHYLAXIS	21-Jan-05	
34	509	PM	2-Jan-05	VITAMIN A	SUPPLEMENTARY	31-Dec-04	
34	509	PM	2-Jan-05	FOLIC ACID	ANAEMIA	31-Dec-04	
34	509	PM	2-Jan-05	FERROUS SULFATE	ANAEMIA	1-Jan-05	
34	509	PM	2-Jan-05	MULTI VITS	SUPPLEMENTARY	31-Dec-04	
34	509	PM	2-Jan-05	CIPROFLOXACIN	UTI	31-Dec-04	8-Jan-05
34	509	PM	2-Jan-05	PARACETAMOL	FEVER	31-Dec-04	1-Jan-05
34	510	Combination	1-Jan-05	MEASLES VACC.	PROPHYLAXIS	9-Jan-05	
34	510	Combination	1-Jan-05	VITAMIN A	SUPPLEMENTARY	31-Dec-04	
34	510	Combination	1-Jan-05	FOLIC ACID	ANAEMIA	31-Dec-04	18-Jan-05
34	510	Combination	1-Jan-05	FERROUS SULFATE	ANAEMIA	31-Dec-04	18-Jan-05
34	510	Combination	1-Jan-05	MULTI VITS	SUPPLEMENTARY	31-Dec-04	18-Jan-05
34	510	Combination	1-Jan-05	PARACETAMOL	FEVER	31-Dec-04	1-Jan-05
34	510	Combination	1-Jan-05	ARTESUNATE	MALARIA	3-Jan-05	5-Jan-05
34	510	Combination	1-Jan-05	SULFADOXINE	MALARIA	3-Jan-05	
34	510	Combination	1-Jan-05	PYRIMETHAMINE		3-Jan-05	
34	511	Combination	1-Jan-05	VITAMIN A	SUPPLEMENTARY	1-Jan-05	
34	511	Combination	1-Jan-05	FOLIC ACID	ANAEMIA	1-Jan-05	18-Jan-05
34	511	Combination	1-Jan-05	FERROUS SULFATE	ANAEMIA	1-Jan-05	18-Jan-05
34	511	Combination	1-Jan-05	MULTI VITS	SUPPLEMENTARY	1-Jan-05	18-Jan-05
34	511	Combination	1-Jan-05	PARACETAMOL	FEVER	1-Jan-05	
34	511	Combination	1-Jan-05	MEASLES VACC.	PROPHYLAXIS	3-Jan-05	
34	511	Combination	1-Jan-05	MEASLES VACC.	PROPHYLAXIS	17-Jan-05	
34	511	Combination	1-Jan-05	ARTESUNATE	MALARIA	11-Jan-05	13-Jan-05
34	511	Combination	1-Jan-05	SULFADOXINE		11-Jan-05	
34	511	Combination	1-Jan-05	PYRIMETHAMINE		11-Jan-05	
34	512	PM	2-Jan-05	VITAMIN A	SUPPLEMENTARY	2-Jan-05	
34	512	PM	2-Jan-05	FOLIC ACID	ANAEMIA	2-Jan-05	
34	512	PM	2-Jan-05	FERROUS SULFATE	ANAEMIA	2-Jan-05	
34	512	PM	2-Jan-05	MULTI VITS	SUPPLEMENTARY	2-Jan-05	
34	512	PM	2-Jan-05	PARACETAMOL	FEVER	2-Jan-05	3-Jan-05
34	512	PM	2-Jan-05	MEASLES VACC.	PROPHYLAXIS	1-Jan-05	
34	512	PM	2-Jan-05	ARTESUNATE	MALARIA	8-Jan-05	10-Jan-05
34	512	PM	2-Jan-05	SULFADOXINE	MALARIA	8-Jan-05	
34	512	PM	2-Jan-05	PYRIMETHAMINE		8-Jan-05	
34	512	PM	2-Jan-05	MEASLES VACC.		23-Jan-05	
34	513	Combination	3-Jan-05	MEASLES VACC.	PROPHYLAXIS	6-Jan-05	
34	513	Combination	3-Jan-05	VITAMIN A	SUPPLEMENTARY	2-Jan-05	
34	513	Combination	3-Jan-05	FOLIC ACID	ANAEMIA	2-Jan-05	20-Jan-05
34	513	Combination	3-Jan-05	FERROUS SULFATE	ANAEMIA	2-Jan-05	20-Jan-05
34	513	Combination	3-Jan-05	MULTI VITS	SUPPLEMENTARY	2-Jan-05	20-Jan-05
34	513	Combination	3-Jan-05	PARACETAMOL	FEVER	2-Jan-05	3-Jan-05
34	513	Combination	3-Jan-05	METRONIDAZOL	ACUTE WATERY DIARRHOEA	7-Jan-05	13-Jan-05
34	513	Combination	3-Jan-05	GENTIAN VIOLET	SUPERFICIAL SKIN LESION	15-Jan-05	19-Jan-05
34	513	Combination	3-Jan-05	MEASLES VACC		19-Jan-05	
34	514	SSG	4-Jan-05	MEASLES VACC.	PROPHYLAXIS	6-Jan-05	
34	514	SSG	4-Jan-05	VITAMIN A	SUPPLEMENTARY	3-Jan-05	
34	514	SSG	4-Jan-05	FOLIC ACID	ANAEMIA	3-Jan-05	2-Feb-05
34	514	SSG	4-Jan-05	FERROUS SULFATE	ANAEMIA	3-Jan-05	2-Feb-05

Centre Number	Patient Number	Treatment	Treatment start date	Concomitant Medication	Diagnosis	Start date	End date
34	514	SSG	4-Jan-05	MULTI VITS	SUPPLEMENTARY	3-Jan-05	2-Feb-05
34	514	SSG	4-Jan-05	PARACETAMOL	FEVER	3-Jan-05	4-Jan-05
34	514	SSG	4-Jan-05	MEASLES VACC	PROPHYLAXIS	2-Feb-05	
34	514	SSG	4-Jan-05	AMOXICILLIN	PNEUMONIA	29-Jan-05	2-Feb-05
34	515	SSG	5-Jan-05	MEASLES VACC.	PROPHYLAXIS	5-Jan-05	
34	515	SSG	5-Jan-05	VITAMIN A	SUPPLEMENTARY	5-Jan-05	
34	515	SSG	5-Jan-05	PARACETAMOL	FEVER	5-Jan-05	
34	515	SSG	5-Jan-05	FOLIC ACID	ANAEMIA	5-Jan-05	3-Feb-05
34	515	SSG	5-Jan-05	FERROUS SULFATE	ANAEMIA	5-Jan-05	3-Feb-05
34	515	SSG	5-Jan-05	MULTI VITS	SUPPLEMENTARY	5-Jan-05	3-Feb-05
34	515	SSG	5-Jan-05	MEASLES VACC.	PROPHYLAXIS	3-Feb-05	
34	515	SSG	5-Jan-05	ARTESUNATE	MALARIA	13-May-05	15-May-05
34	515	SSG	5-Jan-05	SULFADOXINE	MALARIA	13-May-05	
34	515	SSG	5-Jan-05	PYRIMETHAMINE	MALARIA	13-May-05	
34	515	SSG	5-Jan-05	PARACETAMOL	FEVER	13-May-05	
34	515	SSG	5-Jan-05	SSG	PKDL II	14-May-05	
34	516	PM	6-Jan-05	VITAMIN A	SUPPLEMENTARY	6-Jan-05	
34	516	PM	6-Jan-05	FOLIC ACID	ANAEMIA	6-Jan-05	26-Jan-05
34	516	PM	6-Jan-05	FERROUS SULFATE	ANAEMIA	6-Jan-05	26-Jan-05
34	516	PM	6-Jan-05	MULTI VITS	SUPPLEMENTARY	6-Jan-05	26-Jan-05
34	516	PM	6-Jan-05	MEASLES VACC.	PROPHYLAXIS	6-Jan-05	
34	516	PM	6-Jan-05	PARACETAMOL	FEVER	10-Jan-05	
34	516	PM	6-Jan-05	BENZOIC ACID	SUPERFICIAL FUNGAL SKIN INFECTION	19-Jan-05	26-Jan-05
34	516	PM	6-Jan-05	SALICYLIC ACID	SUPERFICIAL FUNGAL SKIN INFECTION	19-Jan-05	26-Jan-05
34	516	PM	6-Jan-05	ALUMINIUM HYDROXIDE		24-Jan-05	
34	516	PM	6-Jan-05	MEASLES VACC.		27-Jan-05	
34	517	PM	6-Jan-05	VITAMIN A	SUPPLEMENTARY	6-Jan-05	
34	517	PM	6-Jan-05	FOLIC ACID	ANAEMIA	6-Jan-05	26-Jan-05
34	517	PM	6-Jan-05	FERROUS SULFATE	ANAEMIA	6-Jan-05	26-Jan-05
34	517	PM	6-Jan-05	MULTI VITS	SUPPLEMENTARY	6-Jan-05	26-Jan-05
34	517	PM	6-Jan-05	MEASLES VACC.	PROPHYLAXIS	7-Jan-05	
34	517	PM	6-Jan-05	PARACETAMOL	FEVER	18-Jan-05	
34	518	SSG	9-Jan-05	MEASLES VACC.	PROPHYLAXIS	7-Jan-05	
34	518	SSG	9-Jan-05	VITAMIN A	SUPPLEMENTARY	7-Jan-05	
34	518	SSG	9-Jan-05	MULTI VITS	SUPPLEMENTARY	6-Jan-05	8-Feb-05
34	518	SSG	9-Jan-05	FOLIC ACID	ANAEMIA	15-Jan-05	8-Feb-05
34	518	SSG	9-Jan-05	FERROUS SULFATE	ANAEMIA	15-Jan-05	8-Feb-05
34	518	SSG	9-Jan-05	PARACETAMOL	FEVER	6-Jan-05	8-Jan-05
34	518	SSG	9-Jan-05	ARTESUNATE	MALARIA	6-Jan-05	8-Jan-05
34	518	SSG	9-Jan-05	SULFADOXINE	MALARIA	6-Jan-05	
34	518	SSG	9-Jan-05	PYRIMETHAMINE		6-Jan-05	
34	518	SSG	9-Jan-05	ARTEMETHER		11-Jan-05	13-Jan-05
34	518	SSG	9-Jan-05	LUMEFANTRINE		11-Jan-05	13-Jan-05
34	518	SSG	9-Jan-05	QUININE		19-Jan-05	24-Jan-05
34	518	SSG	9-Jan-05	CEFTRIAXONE		18-Jan-05	22-Jan-05
34	518	SSG	9-Jan-05	MEASLES VACC		7-Feb-05	
34	519	PM	7-Jan-05	MEASLES VACC.	PROPHYLAXIS	8-Jan-05	
34	519	PM	7-Jan-05	VITAMIN A	SUPPLEMENTARY	6-Jan-05	
34	519	PM	7-Jan-05	FOLIC ACID	SUPPLEMENTARY	6-Jan-05	27-Jan-05
34	519	PM	7-Jan-05	FERROUS SULFATE	SUPPLEMENTARY	6-Jan-05	27-Jan-05

Centre Number	Patient Number	Treatment	Treatment start date	Concomitant Medication	Diagnosis	Start date	End date
34	519	PM	7-Jan-05	MULTI VITS	SUPPLEMENTARY	6-Jan-05	27-Jan-05
34	519	PM	7-Jan-05	PARACETAMOL	FEVER	6-Jan-05	13-Jan-05
34	519	PM	7-Jan-05	ARTESUNATE	MALARIA	13-Jan-05	15-Jan-05
34	519	PM	7-Jan-05	SULFADOXINE	MALARIA	13-Jan-05	
34	519	PM	7-Jan-05	PYRIMETHAMINE		13-Jan-05	
34	519	PM	7-Jan-05	ARTESUNATE	MALARIA	22-Mar-05	25-Mar-05
34	519	PM	7-Jan-05	PARACETAMOL	FEVER	22-Mar-05	25-Mar-05
34	519	PM	7-Jan-05	SULFADOXINE	MALARIA	22-Mar-05	
34	519	PM	7-Jan-05	PYRIMETHAMINE	MALARIA	22-Mar-05	
34	520	SSG	11-Jan-05	VITAMIN A	SUPPLEMENTARY	10-Jan-05	
34	520	SSG	11-Jan-05	FOLIC ACID	ANAEMIA	10-Jan-05	8-Feb-05
34	520	SSG	11-Jan-05	FERROUS SULFATE	ANAEMIA	10-Jan-05	8-Feb-05
34	520	SSG	11-Jan-05	MULTI VITS	SUPPLEMENTARY	10-Jan-05	8-Feb-05
34	520	SSG	11-Jan-05	PARACETAMOL	FEVER	10-Jan-05	13-Jan-05
34	520	SSG	11-Jan-05	TINIDAZOLE	ACUTE WATERY DIARRHOEA	17-Jan-05	19-Jan-05
34	520	SSG	11-Jan-05	METRONIDAZOL	MUCOID STOOL	23-Jan-05	29-Jan-05
34	520	SSG	11-Jan-05	SULFADOXINE	MALARIA	15-May-05	
34	520	SSG	11-Jan-05	PYRIMETHAMINE	MALARIA	15-May-05	
34	520	SSG	11-Jan-05	CHLOROQUINE	MALARIA	15-May-05	
34	521	PM	11-Jan-05	MEASLES VACC.	PROPHYLAXIS	11-Jan-05	
34	521	PM	11-Jan-05	VITAMIN A	SUPPLEMENTARY	10-Jan-05	
34	521	PM	11-Jan-05	FOLIC ACID	ANAEMIA	10-Jan-05	31-Jan-05
34	521	PM	11-Jan-05	FERROUS SULFATE	ANAEMIA	10-Jan-05	31-Jan-05
34	521	PM	11-Jan-05	MULTI VITS	SUPPLEMENTARY	10-Jan-05	31-Jan-05
34	521	PM	11-Jan-05	PARACETAMOL	FEVER	10-Jan-05	11-Jan-05
34	521	PM	11-Jan-05	ARTESUNATE	MALARIA	20-Jan-05	22-Jan-05
34	521	PM	11-Jan-05	SULFADOXINE	MALARIA	20-Jan-05	
34	521	PM	11-Jan-05	PYRIMETHAMINE		20-Jan-05	
34	522	Combination	11-Jan-05	FERROUS SULFATE	ANAEMIA	11-Jan-05	27-Jan-05
34	522	Combination	11-Jan-05	FOLIC ACID	ANAEMIA	11-Jan-05	27-Jan-05
34	522	Combination	11-Jan-05	MULTI VITS	SUPPLEMENTARY	11-Jan-05	27-Jan-05
34	522	Combination	11-Jan-05	VITAMIN A	SUPPLEMENTARY	11-Jan-05	
34	522	Combination	11-Jan-05	SALBUTAMOL	ASTHMA	19-Jan-05	21-Jan-05
34	522	Combination	11-Jan-05	SALBUTAMOL	ASTHMA	24-Jan-05	27-Jan-05
34	522	Combination	11-Jan-05	HYDROCORTISON	ASTHMA	18-Jan-05	
34	523	SSG	14-Jan-05	MEASLES VACC.	PROPHYLAXIS	12-Jan-05	
34	523	SSG	14-Jan-05	SULFADOXINE	MALARIA	12-Jan-05	
34	523	SSG	14-Jan-05	PYRIMETHAMINE	MALARIA	12-Jan-05	
34	523	SSG	14-Jan-05	ARTESUNATE	MALARIA	12-Jan-05	14-Jan-05
34	523	SSG	14-Jan-05	PARACETAMOL	FEVER	12-Jan-05	
34	523	SSG	14-Jan-05	VITAMIN A	SUPPLEMENTARY	12-Jan-05	
34	523	SSG	14-Jan-05	MULTI VITS	SUPPLEMENTARY	12-Jan-05	12-Feb-05
34	523	SSG	14-Jan-05	FOLIC ACID	ANAEMIA	16-Jan-05	12-Feb-05
34	523	SSG	14-Jan-05	FERROUS SULFATE		16-Jan-05	12-Feb-05
34	523	SSG	14-Jan-05	MEASLES VACC.		8-Feb-05	
34	524	Combination	13-Jan-05	MEASLES VACC.	PROPHYLAXIS	13-Jan-05	
34	524	Combination	13-Jan-05	VITAMIN A	SUPPLEMENTARY	12-Jan-05	
34	524	Combination	13-Jan-05	PARACETAMOL	FEVER	12-Jan-05	13-Jan-05
34	524	Combination	13-Jan-05	FOLIC ACID	ANAEMIA	12-Jan-05	30-Jan-05
34	524	Combination	13-Jan-05	FERROUS SULFATE	ANAEMIA	12-Jan-05	30-Jan-05
34	524	Combination	13-Jan-05	MULTI VITS	SUPPLEMENTARY	12-Jan-05	30-Jan-05
34	524	Combination	13-Jan-05	MEASLES VACC	PROPHYLAXIS	29-Jan-05	

Centre Number	Patient Number	Treatment	Treatment start date	Concomitant Medication	Diagnosis	Start date	End date
34	525	Combination	13-Jan-05	MEASLES VACC.	PROPHYLAXIS	13-Jan-05	
34	525	Combination	13-Jan-05	VITAMIN A	SUPPLEMENTARY	13-Jan-05	
34	525	Combination	13-Jan-05	FOLIC ACID	ANAEMIA	13-Jan-05	30-Jan-05
34	525	Combination	13-Jan-05	FERROUS SULFATE	ANAEMIA	13-Jan-05	30-Jan-05
34	525	Combination	13-Jan-05	MULTI VITS	SUPPLEMENTARY	13-Jan-05	30-Jan-05
34	525	Combination	13-Jan-05	PARACETAMOL	FEVER	13-Jan-05	
34	525	Combination	13-Jan-05	MEASLES VACC	PROPHYLAXIS	29-Jan-05	
34	526	SSG	14-Jan-05	MEASLES VACC	PROPHYLAXIS	13-Jan-05	
34	526	SSG	14-Jan-05	PARACETAMOL	FEVER	13-Jan-05	17-Jan-05
34	526	SSG	14-Jan-05	VITAMIN A	SUPPLEMENTARY	13-Jan-05	
34	526	SSG	14-Jan-05	MULTI VITS	SUPPLEMENTARY	13-Jan-05	12-Feb-05
34	526	SSG	14-Jan-05	FOLIC ACID	ANAEMIA	13-Jan-05	16-Jan-05
34	526	SSG	14-Jan-05	FERROUS SULFATE	ANAEMIA	13-Jan-05	16-Jan-05
34	526	SSG	14-Jan-05	SULFADOXINE	MALARIA	16-Jan-05	
34	526	SSG	14-Jan-05	PYRIMETHAMINE	MALARIA	16-Jan-05	
34	526	SSG	14-Jan-05	ARTESUNATE		16-Jan-05	18-Jan-05
34	526	SSG	14-Jan-05	MEASLES VACC		12-Feb-05	
34	526	SSG	14-Jan-05	BENZYL PENICILLIN	BACTERIAL INFECTION	15-Apr-05	16-Apr-05
34	526	SSG	14-Jan-05	AMPICILLIN SYRUP	BACTERIAL INFECTION	17-Apr-05	22-Apr-05
34	527	Combination	15-Jan-05	VITAMIN A	SUPPLEMENTARY	14-Jan-05	
34	527	Combination	15-Jan-05	FOLIC ACID	ANAEMIA	14-Jan-05	
34	527	Combination	15-Jan-05	FERROUS SULFATE	ANAEMIA	14-Jan-05	
34	527	Combination	15-Jan-05	MULTI VITS	SUPPLEMENTARY	14-Jan-05	
34	527	Combination	15-Jan-05	PARACETAMOL	FEVER	14-Jan-05	15-Jan-05
34	527	Combination	15-Jan-05	PROMETHAZINE	NAUSEA + VOMITING	17-Jan-05	
34	528	PM	17-Jan-05	MEASLES VACC.	PROPHYLAXIS	17-Jan-05	
34	528	PM	17-Jan-05	VITAMIN A	SUPPLEMENTARY	16-Jan-05	
34	528	PM	17-Jan-05	MULTI VITS	SUPPLEMENTARY	16-Jan-05	6-Feb-05
34	528	PM	17-Jan-05	FOLIC ACID	ANAEMIA	16-Jan-05	6-Feb-05
34	528	PM	17-Jan-05	FERROUS SULFATE	ANAEMIA	16-Jan-05	6-Feb-05
34	528	PM	17-Jan-05	PARACETAMOL	FEVER	16-Jan-05	17-Jan-05
34	529	Combination	18-Jan-05	MEASLES VACC.	PROPHYLAXIS	18-Jan-05	
34	529	Combination	18-Jan-05	PARACETAMOL	FEVER	18-Jan-05	19-Jan-05
34	529	Combination	18-Jan-05	VITAMIN A	SUPPLEMENTARY	18-Jan-05	
34	529	Combination	18-Jan-05	FOLIC ACID	ANAEMIA	18-Jan-05	4-Feb-05
34	529	Combination	18-Jan-05	FERROUS SULFATE	ANAEMIA	18-Jan-05	4-Feb-05
34	529	Combination	18-Jan-05	MULTI VITS	SUPPLEMENTARY	18-Jan-05	4-Feb-05
34	529	Combination	18-Jan-05	MEASLES VACC	PROPHYLAXIS	3-Feb-05	
34	530	Combination	20-Jan-05	VITAMIN A	SUPPLEMENTARY	19-Jan-05	
34	530	Combination	20-Jan-05	MULTI VITS	SUPPLEMENTARY	19-Jan-05	6-Feb-05
34	530	Combination	20-Jan-05	FOLIC ACID	ANAEMIA	19-Jan-05	6-Feb-05
34	530	Combination	20-Jan-05	FERROUS SULFATE	ANAEMIA	19-Jan-05	6-Feb-05
34	530	Combination	20-Jan-05	PARACETAMOL	FEVER	19-Jan-05	20-Jan-05
34	530	Combination	20-Jan-05	AMOXICILLIN	EAR INFECTION	14-Apr-05	19-Apr-05
34	531	SSG	21-Jan-05	MEASLES VACC	PROPHYLAXIS	21-Jan-05	
34	531	SSG	21-Jan-05	VITAMIN A	SUPPLEMENTARY	20-Jan-05	
34	531	SSG	21-Jan-05	FOLIC ACID	ANAEMIA	20-Jan-05	19-Feb-05
34	531	SSG	21-Jan-05	FERROUS SULFATE	ANAEMIA	20-Jan-05	19-Feb-05
34	531	SSG	21-Jan-05	MULTI VITS	SUPPLEMENTARY	20-Jan-05	19-Feb-05
34	531	SSG	21-Jan-05	PARACETAMOL	FEVER	20-Jan-05	21-Jan-05
34	531	SSG	21-Jan-05	MEASLES VACC	PROPHYLAXIS	19-Feb-05	
34	532	Combination	21-Jan-05	MEASLES VACC.	PROPHYLAXIS	21-Jan-05	
34	532	Combination	21-Jan-05	VITAMIN A	SUPPLEMENTARY	20-Jan-05	

Centre Number	Patient Number	Treatment	Treatment start date	Concomitant Medication	Diagnosis	Start date	End date
34	532	Combination	21-Jan-05	MULTI VITS	SUPPLEMENTARY	20-Jan-05	6-Feb-05
34	532	Combination	21-Jan-05	FOLIC ACID	ANAEMIA	20-Jan-05	6-Feb-05
34	532	Combination	21-Jan-05	FERROUS SULFATE	ANAEMIA	20-Jan-05	6-Feb-05
34	532	Combination	21-Jan-05	PARACETAMOL	FEVER	20-Jan-05	21-Jan-05
34	533	SSG	23-Jan-05	MEASLES VACC	PROPHYLAXIS	23-Jan-05	
34	533	SSG	23-Jan-05	VITAMIN A	SUPPLEMENTARY	22-Jan-05	
34	533	SSG	23-Jan-05	FOLIC ACID	ANAEMIA	22-Jan-05	22-Feb-05
34	533	SSG	23-Jan-05	FERROUS SULFATE	ANAEMIA	22-Jan-05	22-Feb-05
34	533	SSG	23-Jan-05	MULTI VITS	SUPPLEMENTARY	22-Jan-05	22-Feb-05
34	533	SSG	23-Jan-05	PARACETAMOL	FEVER	22-Jan-05	23-Jan-05
34	534	PM	24-Jan-05	MEASLES VACC	PROPHYLAXIS	24-Jan-05	
34	534	PM	24-Jan-05	ARTESUNATE	MALARIA	23-Jan-05	
34	534	PM	24-Jan-05	PARACETAMOL	FEVER	23-Jan-05	24-Jan-05
34	534	PM	24-Jan-05	VITAMIN A	SUPPLEMENTARY	23-Jan-05	
34	534	PM	24-Jan-05	FOLIC ACID	ANAEMIA	23-Jan-05	14-Feb-05
34	534	PM	24-Jan-05	FERROUS SULFATE	ANAEMIA	23-Jan-05	14-Feb-05
34	534	PM	24-Jan-05	MULTI VITS	SUPPLEMENTARY	23-Jan-05	14-Feb-05
34	534	PM	24-Jan-05	CEFTRIAXONE	PNEUMONIA	30-Jan-05	3-Feb-05
34	534	PM	24-Jan-05	MEASLES VACC		14-Feb-05	
34	535	PM	24-Jan-05	MEASLES VACC	PROPHYLAXIS	24-Jan-05	
34	535	PM	24-Jan-05	VITAMIN A	SUPPLEMENTARY	23-Jan-05	
34	535	PM	24-Jan-05	FOLIC ACID	ANAEMIA	23-Jan-05	14-Feb-05
34	535	PM	24-Jan-05	FERROUS SULFATE	ANAEMIA	23-Jan-05	14-Feb-05
34	535	PM	24-Jan-05	MULTI VITS	SUPPLEMENTARY	23-Jan-05	14-Feb-05
34	535	PM	24-Jan-05	PARACETAMOL	FEVER	23-Jan-05	24-Jan-05
34	535	PM	24-Jan-05	TINIDAZOLE	ACUTE WATERY DIARRHOE	6-Feb-05	8-Feb-05
34	535	PM	24-Jan-05	MEASLES VACC	PROPHYLAXIS	14-Feb-05	
34	536	PM	25-Jan-05	MEASLES VACC.	PROPHYLAXIS	25-Jan-05	
34	536	PM	25-Jan-05	VITAMIN A	SUPPLEMENTARY	25-Jan-05	
34	536	PM	25-Jan-05	FOLIC ACID	ANAEMIA	25-Jan-05	15-Feb-05
34	536	PM	25-Jan-05	FERROUS SULFATE	ANAEMIA	25-Jan-05	15-Feb-05
34	536	PM	25-Jan-05	MULTI VITS	SUPPLEMENTARY	25-Jan-05	15-Feb-05
34	536	PM	25-Jan-05	PARACETAMOL	FEVER	25-Jan-05	
34	536	PM	25-Jan-05	MEASLES VACC	PROPHYLAXIS	14-Feb-05	
34	536	PM	25-Jan-05	PARACETAMOL	HEADACHE	5-May-05	6-May-05
34	537	SSG	25-Jan-05	MEASLES VACC.	PROPHYLAXIS	25-Jan-05	
34	537	SSG	25-Jan-05	VITAMIN A	SUPPLEMENTARY	25-Jan-05	
34	537	SSG	25-Jan-05	FOLIC ACID	ANAEMIA	25-Jan-05	24-Feb-05
34	537	SSG	25-Jan-05	FERROUS SULFATE	ANAEMIA	25-Jan-05	24-Feb-05
34	537	SSG	25-Jan-05	MULTI VITS	SUPPLEMENTARY	25-Jan-05	24-Feb-05
34	537	SSG	25-Jan-05	PARACETAMOL	FEVER	25-Jan-05	
34	538	PM	26-Jan-05	MEASLES VACC	PROPHYLAXIS	26-Jan-05	
34	538	PM	26-Jan-05	VITAMIN A	SUPPLEMENTARY	25-Jan-05	
34	538	PM	26-Jan-05	FOLIC ACID	ANAEMIA	25-Jan-05	16-Feb-05
34	538	PM	26-Jan-05	FERROUS SULFATE	ANAEMIA	25-Jan-05	16-Feb-05
34	538	PM	26-Jan-05	MULTI VITS	SUPPLEMENTARY	25-Jan-05	16-Feb-05
34	538	PM	26-Jan-05	PARACETAMOL	FEVER	25-Jan-05	26-Jan-05
34	538	PM	26-Jan-05	TINIDAZOL	WATERY DIARRHOEA	25-Jan-05	27-Jan-05
34	538	PM	26-Jan-05	AMOXICILLIN	MUCOSAL INFECTION OF THE NOSE	31-Jan-05	4-Feb-05
34	538	PM	26-Jan-05	MEASLES VACC		15-Feb-05	
34	539	Combination	26-Jan-05	MEASLES VACC.	PROPHYLAXIS	26-Jan-05	
34	539	Combination	26-Jan-05	VITAMIN A	SUPPLEMENTARY	25-Jan-05	
34	539	Combination	26-Jan-05	MULTI VITS	SUPPLEMENTARY	25-Jan-05	12-Feb-05

Centre Number	Patient Number	Treatment	Treatment start date	Concomitant Medication	Diagnosis	Start date	End date
34	539	Combination	26-Jan-05	FOLIC ACID	ANAEMIA	25-Jan-05	12-Feb-05
34	539	Combination	26-Jan-05	FERROUS SULFATE	ANAEMIA	25-Jan-05	12-Feb-05
34	539	Combination	26-Jan-05	PARACETAMOL	FEVER	25-Jan-05	26-Jan-05
34	539	Combination	26-Jan-05	ARTESUNATE	MALARIA	25-Jan-05	
34	539	Combination	26-Jan-05	MEASLES VACC	PROPHYLAXIS	11-Feb-05	
34	539	Combination	26-Jan-05	CHLOROQUINE	MALARIA	2-Jun-05	7-Jun-05
34	539	Combination	26-Jan-05	VITAMIN B COMPL	?	2-Jun-05	7-Jun-05
34	539	Combination	26-Jan-05	CHLORPHENIRAMINE	?	2-Jun-05	7-Jun-05
34	540	SSG	27-Jan-05	MEASLES VACC	PROPHYLAXIS	2-Feb-05	
34	540	SSG	27-Jan-05	VITAMIN A	SUPPLEMENTARY	26-Jan-05	
34	540	SSG	27-Jan-05	MULTI VITS	SUPPLEMENTARY	26-Jan-05	25-Feb-05
34	540	SSG	27-Jan-05	FOLIC ACID	ANAEMIA	26-Jan-05	25-Feb-05
34	540	SSG	27-Jan-05	FERROUS SULFATE	ANAEMIA	26-Jan-05	25-Feb-05
34	540	SSG	27-Jan-05	PARACETAMOL	FEVER	26-Jan-05	28-Jan-05
34	540	SSG	27-Jan-05	ALUMINIUM HYDROXIDE	EPIGASTRIC PAIN	29-Jan-05	30-Jan-05
34	540	SSG	27-Jan-05	AMOXICILLIN	PNEUMONIA	9-Feb-05	15-Feb-05
35	646	PM	28-Jan-05	PARACETAMOL	HIGH-GRADE FEVER	29-Jan-05	29-Jan-05
35	646	PM	28-Jan-05	AMPICLOX CAPS	SUPPURATIVE OTITIS MEDIA	3-Feb-05	12-Feb-05
35	647	PM	30-Jan-05	PARACETAMOL	FEVER	31-Jan-05	1-Feb-05
35	647	PM	30-Jan-05	PARACETAMOL	FEVER	1-Feb-05	2-Feb-05
35	647	PM	30-Jan-05	COTRIMOXAZOLE	UTI	20-Mar-05	24-Mar-05
35	648	Combination	30-Jan-05	SALBUTAMOL	ASTHMATIC ATTACK	8-Feb-05	8-Feb-05
35	648	Combination	30-Jan-05	SALBUTAMOL	ASTHMATIC ATTACK	8-Feb-05	16-Feb-05
35	651	SSG	30-Jan-05	CHLOROQUINE SUSP.	MALARIA	18-Feb-05	21-Feb-05
35	651	SSG	30-Jan-05	PARACETAMOL	FEVER	18-Feb-05	18-Feb-05
35	652	PM	1-Feb-05	PARACETAMOL	FEVER	1-Feb-05	1-Feb-05
35	652	PM	1-Feb-05	PARACETAMOL	FEVER	5-Feb-05	6-Feb-05
35	652	PM	1-Feb-05	AMOXYCILLIN SUSP.	ACUTE OTITIS MEDIA	7-Feb-05	16-Feb-05
35	652	PM	1-Feb-05	PARACETAMOL	FEVER EARACHE	7-Feb-05	8-Feb-05
35	654	SSG	3-Feb-05	TETRACYCLINE OINTMENT	PURULENT CONJUNCTIVITIS	21-Feb-05	25-Feb-05
35	655	Combination	3-Feb-05	PARACETAMOL	FEVER	4-Feb-05	4-Feb-05
35	656	SSG	3-Feb-05	PARACETAMOL	FEVER	4-Feb-05	4-Feb-05
35	656	SSG	3-Feb-05	PARACETAMOL	FEVER	5-Feb-05	5-Feb-05
35	658	Combination	8-Feb-05	TINIDAZOLE	AMOEBIC DYSENTRY	17-Feb-05	17-Feb-05
35	659	SSG	8-Feb-05	BENZYL PENICILLIN	BRONCHO-PNEUMONIA	9-Feb-05	11-Feb-05
35	659	SSG	8-Feb-05	PARACETAMOL	PNEUMONIA WITH HIGH GRADE FEVER	9-Feb-05	10-Feb-05
35	659	SSG	8-Feb-05	AMOXYCILLIN	PNEUMONIA CONTINUATION	11-Feb-05	15-Feb-05
35	662	PM	20-Feb-05	CHLOROQUINE	UNCOMPLICATED MALARIA	21-Feb-05	23-Feb-05
35	662	PM	20-Feb-05	PARACETAMOL	UNCOMPLICATED MALARIA	21-Feb-05	21-Feb-05
35	663	SSG	20-Feb-05	CEPHALEXIN	PNEUMONIA	23-Feb-05	27-Feb-05
35	664	Combination	20-Feb-05	ANTACID	HEART BURN	21-Feb-05	23-Feb-05
35	676	PM	20-Mar-05	PARACETAMOLE	HIGH GRADE FEVER	22-Mar-05	23-Mar-05
35	677	SSG	20-Mar-05	ANTACID	HEART BURN	27-Mar-05	29-Mar-05
35	677	SSG	20-Mar-05	PARACETAMOL	FEVER	27-Mar-05	27-Mar-05
35	681	Combination	22-Mar-05	CRYSTALLINE	PNEUMONIA	27-Mar-05	28-Mar-05
35	681	Combination	22-Mar-05	ERYTHROMYCIN TABS	PNEUMONIA	28-Mar-05	1-Apr-05

Centre Number	Patient Number	Treatment	Treatment start date	Concomitant Medication	Diagnosis	Start date	End date
35	684	SSG	9-Apr-05	PARACETAMOLE	UN-COMPLICATED MALARIA	5-May-05	6-May-05
35	684	SSG	9-Apr-05	PYRIDOXINE	UN-COMPLICATED MALARIA	5-May-05	5-May-05
35	684	SSG	9-Apr-05	CHLOROQUINE	UN-COMPLICATED MALARIA	5-May-05	7-May-05
35	687	SSG	13-Apr-05	CHLORPHENERAMINE MALEATE	ALLERGIC BRONCHITIS	16-Apr-05	19-Apr-05
35	687	SSG	13-Apr-05	CEPHALEXINE	UTI	5-May-05	11-May-05
35	688	PM	16-Apr-05	CHLOROQUINE	MALARIA	15-Sep-05	18-Sep-05

16.3.6 Individual efficacy response data

Appendix 38: Listing of individual efficacy data – Clinical Response and Parasitology

Appendix 39: Listing of individual efficacy data – Spleen size and liver size

Appendix 40: Listing of individual efficacy data – Clinical Characteristics, cervical lymphadenopathy and axiliary lymphadenopathy

Appendix 41: Listing of individual efficacy data – Clinical Characteristics, inguinal lymphadenopathy and muscle wasting

Appendix 42: Listing of individual efficacy data – Clinical Characteristics, mucosal pallor and jaundice

Appendix 43: Listing of individual efficacy data – Clinical Characteristics, Petchial haemorrhage

Centre Number	Patient Number	Treatment	Clinical Response to treatment					
			Day 7	Day 14	Day 21	EOT	3Mon FU	6Mon FU
11	1	PM	Stable	Improving		Partial	Partial	Partial
11	2	Combination	Improving	Improving		Partial	Partial	Partial
11	3	Combination	Improving	Improving		Partial	Partial	Partial
11	4	PM	Improving	Improving		Partial	Partial	Complete
11	5	PM	Stable	Stable		Partial	Partial	Complete
11	6	Combination	Stable	Improving		Partial	Partial	Partial
11	7	PM	Stable	Stable		Partial	Partial	Partial
11	8	SSG	Improving	Improving	Improving	Partial	Partial	Partial
11	9	SSG	Stable	Stable	Stable	Partial	Partial	Partial
11	10	SSG	Improving	Improving	Improving	Partial	Complete	Complete
11	11	Combination	Stable	Stable		Partial	Partial	Partial
11	12	Combination	Stable	Deteriorating		Partial	Complete	Complete
11	13	SSG	Stable	Stable	Stable	Partial	Partial	Complete
11	14	SSG	Stable	Stable	Improving	Partial	Partial	Partial
11	15	PM	Deteriorating				Partial	Partial
11	16	Combination	Stable	Stable		Complete	Complete	Complete
11	17	Combination	Stable	Stable		Partial	Partial	Partial
11	18	SSG	Stable	Improving	Improving	Partial	Partial	Partial
11	19	Combination	Improving	Improving		Partial	Partial	Partial
11	20	SSG	Improving	Improving	Improving	Partial	Partial	Partial
11	21	SSG	Stable	Stable	Stable	Partial	Partial	Partial
11	22	SSG	Improving	Improving	Improving	Partial	Complete	Complete
11	23	PM	Improving	Improving		Partial	Partial	Partial
11	24	PM	Stable	Stable		Partial	Partial	Partial
11	25	SSG	Improving	Improving	Improving	Partial	Partial	Complete
11	26	Combination	Stable	Stable		Partial	Partial	Partial
11	27	PM	Improving	Improving		Partial	Partial	
11	28	Combination	Stable	Stable		Partial	Partial	Partial
11	29	PM	Improving	Stable		Partial	Partial	Partial
11	30	PM	Improving	Improving		Partial	Partial	Partial
11	31	SSG	Stable	Improving	Improving	Partial	Partial	Partial
11	32	Combination	Improving	Improving		Complete	Complete	Complete
11	33	SSG	Stable	Improving	Improving	Complete	Complete	Complete
11	34	PM	Improving	Improving		Partial	Partial	Partial
11	35	SSG	Stable	Stable	Stable	Partial	Partial	Partial
11	36	SSG	Improving	Improving	Improving	Partial	Partial	Complete
11	37	Combination	Improving	Improving		Complete	Complete	Complete
11	38	PM	Stable	Stable		Partial	Partial	Partial
11	39	PM	Improving	Improving		Partial	Partial	Partial
11	40	PM	Stable	Improving			Partial	Partial
11	41	Combination	Stable	Improving		Partial	Partial	Complete
11	42	Combination	Improving	Improving		Complete	Complete	Complete
11	43	SSG	Improving	Improving	Stable	Partial	Partial	Partial
11	44	PM	Improving	Improving		Partial	Partial	Complete
11	45	Combination	Improving	Improving		Partial	Partial	Complete
11	46	SSG	Stable	Improving	Improving	Partial	Partial	Complete
11	47	SSG	Improving	Improving	Improving	Partial		
11	48	Combination	Improving	Improving		Partial		Complete
11	49	SSG	Improving	Improving	Improving	Partial	Complete	Complete
11	50	PM	Stable	Stable		Partial	Partial	Partial
11	51	Combination	Improving	Improving		Partial	Complete	Complete
11	52	PM	Stable	Stable		Partial	Partial	Partial
11	53	SSG	Improving	Improving	Improving	Partial	Complete	Complete

Centre Number	Patient Number	Treatment	Clinical Response to treatment					
			Day 7	Day 14	Day 21	EOT	3Mon FU	6Mon FU
11	54	PM	Improving	Improving		Partial	Partial	Partial
11	55	PM	Stable	Stable		Partial	Complete	Complete
11	56	SSG	Improving	Improving	Improving	Partial	Partial	Partial
11	57	Combination	Improving	Improving		Partial	Partial	Partial
11	58	Combination	Stable	Improving		Partial	Complete	Complete
11	59	PM	Stable	Improving		Partial	Partial	Complete
11	60	Combination	Improving	Improving		Partial	Partial	Partial
11	61	PM	Stable	Improving		Partial	Partial	Partial
11	62	SSG	Improving	Improving	Improving	Partial	Partial	Partial
11	63	SSG	Improving	Improving	Improving	Partial	Partial	Partial
11	64	SSG	Stable	Improving	Improving	Partial	Partial	Partial
11	65	PM	Improving	Improving		Partial		
11	66	Combination	Improving	Improving		Partial	Complete	Complete
11	67	PM	Improving	Improving		Partial	Partial	Complete
11	68	SSG	Improving	Improving	Improving	Partial	Partial	Partial
11	69	Combination	Improving	Improving		Partial	Partial	Partial
11	70	Combination	Improving	Improving		Partial	Complete	Complete
11	71	Combination	Stable	Stable		Partial	Partial	Partial
11	72	SSG	Stable	Stable			Partial	Partial
11	73	PM	Improving	Improving		Partial	Partial	Partial
11	74	PM	Improving	Improving		Complete	Complete	Complete
11	75	Combination	Improving	Improving		Partial	Partial	Partial
11	76	PM	Improving	Improving		Partial	Complete	Complete
11	77	PM	Improving	Improving		Partial	Partial	Partial
11	78	SSG	Improving	Improving	Improving	Partial	Partial	Partial
11	79	PM	Stable	Improving		Partial	Partial	Partial
11	80	PM	Improving	Improving		Partial	Partial	Partial
11	81	Combination	Improving	Improving		Partial	Partial	Partial
11	82	SSG	Improving	Improving	Improving	Partial	Partial	Partial
11	83	SSG	Improving	Improving	Improving	Partial	Complete	Complete
11	84	SSG	Improving	Improving	Improving	Partial	Complete	Complete
11	85	SSG	Improving	Improving	Improving	Partial	Partial	
11	86	Combination	Improving	Improving		Complete	Complete	Complete
11	87	Combination	Improving	Improving		Partial	Partial	Partial
11	88	PM	Improving	Improving		Partial	Complete	Complete
11	89	Combination	Improving	Improving		Partial	Partial	Complete
11	90	Combination	Improving	Improving		Partial		
11	91	PM	Improving	Improving		Partial	Partial	Partial
11	92	Combination	Improving	Improving		Partial	Partial	Complete
11	93	SSG	Improving	Improving	Improving	Partial	Complete	Complete
11	94	SSG	Improving	Improving	Improving	Partial	Partial	Partial
11	95	SSG	Improving	Improving	Improving	Partial	Partial	Complete
11	96	Combination	Improving	Improving		Partial	Complete	Complete
11	97	Combination	Improving	Improving		Partial	Partial	Complete
11	98	PM	Stable	Stable		Partial	Partial	Complete
11	99	SSG	Improving	Improving	Improving	Partial	Partial	Complete
11	100	PM	Improving	Improving		Partial		
11	101	PM	Improving	Improving		Partial	Partial	Partial
11	102	Combination	Improving	Improving		Partial	Complete	Complete
11	103	Combination	Improving	Improving		Partial	Complete	Complete
11	104	PM	Stable	Improving		Partial	Partial	Partial
11	105	SSG	Improving	Improving	Improving	Partial	Partial	Partial
11	106	Combination	Improving	Improving		Complete	Complete	Complete

Centre Number	Patient Number	Treatment	Clinical Response to treatment					
			Day 7	Day 14	Day 21	EOT	3Mon FU	6Mon FU
11	107	PM	Improving	Improving		Partial	Partial	Partial
11	108	PM	Improving	Improving		Complete	Complete	Complete
11	109	SSG	Improving	Improving	Improving	Partial	Partial	Partial
11	110	PM	Improving	Improving		Partial	Partial	Partial
11	111	Combination	Improving	Improving		Complete	Complete	Complete
11	112	Combination	Stable	Improving		Partial	Complete	Complete
11	113	SSG	Improving	Improving	Improving	Partial	Partial	
11	114	SSG	Improving	Improving	Improving	Partial	Partial	Complete
11	115	Combination	Improving	Improving		Partial	Complete	Complete
11	116	SSG	Improving	Improving	Improving	Partial	Complete	Complete
11	117	PM	Improving	Improving		Partial	Partial	
11	118	PM	Stable	Improving		Partial	Partial	Partial
11	119	SSG	Improving	Improving	Improving	Partial	Partial	Partial
11	120	Combination	Improving	Improving		Partial	Complete	Complete
11	121	Combination	Improving	Improving		Partial	Complete	Complete
11	122	SSG	Improving	Improving	Improving	Partial		
11	123	SSG	Improving	Improving	Improving	Partial	Partial	Partial
11	124	SSG	Improving	Improving	Improving	Partial		Complete
11	125	SSG	Improving	Improving	Improving	Complete	Complete	Complete
11	126	Combination	Stable	Stable		Partial		Partial
11	127	PM	Stable	Improving		Partial	Partial	Partial
11	128	Combination	Stable	Stable		Partial	Partial	Partial
11	129	PM	Stable	Improving		Partial	Complete	Complete
11	130	Combination	Improving	Improving		Complete	Complete	Complete
11	131	PM	Stable	Improving		Partial	Complete	Complete
11	132	SSG	Improving	Improving	Improving	Partial	Complete	Partial
11	133	Combination	Improving	Improving		Partial	Complete	Complete
11	134	PM	Stable	Improving		Partial	Partial	Partial
11	135	PM	Improving	Improving		Partial	Partial	Complete
12	241	Combination	Improving	Improving		Complete		Complete
12	242	SSG	Improving	Improving	Improving	Complete	Partial	Complete
12	243	PM	Improving	Improving		Partial	Complete	Complete
12	244	SSG	Improving	Improving	Stable	Partial	Partial	Complete
12	245	PM	Improving	Improving		Partial	Partial	
12	246	PM	Improving	Stable		Complete	Partial	
12	247	PM	Improving	Improving		Partial	Complete	Complete
12	248	PM	Improving	Improving		Complete	Complete	Complete
12	249	Combination	Stable	Stable		Partial	Partial	Complete
12	250	SSG	Improving	Improving	Improving	Complete	Partial	Complete
12	251	Combination	Stable	Improving		Partial	Complete	Complete
12	252	SSG	Improving	Improving	Improving	Complete	Complete	Complete
12	253	SSG	Stable	Improving	Improving	Partial	Complete	Complete
12	254	Combination	Improving	Stable		Partial	Partial	Complete
12	255	Combination	Improving	Improving		Complete	Complete	Complete
12	256	Combination	Improving	Improving		Complete	Complete	Complete
12	257	SSG	Improving	Improving	Improving	Partial	Partial	Complete
12	258	SSG	Improving	Improving	Improving	Partial		Complete
12	259	PM	Improving	Improving		Complete	Complete	Complete
12	260	PM	Improving	Improving		Complete		Complete
12	261	Combination	Improving	Improving		Partial	Complete	Complete
12	262	PM	Improving	Improving		Complete	Complete	Complete
12	263	SSG	Stable	Stable	Stable	Complete	Complete	Complete
12	264	Combination	Improving	Improving		Partial	Partial	Complete

Centre Number	Patient Number	Treatment	Clinical Response to treatment Day 7	Day 14	Day 21	EOT	3Mon FU	6Mon FU
12	265	SSG	Improving	Stable	Improving			
12	266	PM	Improving	Improving		Partial	Partial	Complete
12	267	SSG	Improving	Improving	Improving	Complete	Complete	Complete
12	268	PM	Stable	Stable		Partial	Complete	Partial
12	269	Combination	Improving	Improving		Partial	Complete	Complete
12	270	Combination	Improving	Improving		Partial		Complete
12	271	SSG	Improving	Improving	Improving	Complete	Complete	Complete
12	272	PM	Improving	Improving		Complete	Complete	Complete
12	273	SSG	Improving	Improving	Improving	Complete	Complete	Complete
12	274	SSG	Improving	Improving	Improving	Complete	Complete	Complete
12	275	Combination	Stable	Improving		Partial	Complete	Complete
12	276	PM	Improving	Improving		Complete	Complete	Complete
12	277	Combination	Improving	Improving		Partial	Complete	Complete
12	278	SSG	Stable	Improving	Improving	Partial	Complete	
12	279	PM	Improving	Improving		Complete	Complete	Complete
12	280	SSG	Improving	Improving	Improving	Partial	Complete	Complete
12	281	Combination	Stable	Improving		Partial	Complete	Complete
12	282	PM	Stable	Improving		Partial	Complete	Complete
12	283	PM	Improving	Improving		Complete	Complete	Complete
12	284	Combination	Improving	Improving		Complete	Partial	Partial
12	285	Combination	Stable	Improving		Partial	Complete	Complete
12	286	PM	Stable	Improving		Partial	Complete	Complete
12	287	SSG	Improving	Stable	Improving	Partial	Complete	Complete
12	288	Combination	Improving	Improving		Complete	Complete	Complete
12	289	Combination	Improving	Improving		Complete	Complete	Complete
12	290	SSG	Stable	Improving	Improving	Complete		Complete
12	291	SSG	Stable	Stable	Improving	Partial	Complete	Complete
12	292	PM	Improving	Improving		Complete		Complete
12	293	SSG	Improving	Improving	Improving	Complete		Complete
12	294	Combination	Stable	Improving		Partial	Partial	Complete
12	295	Combination	Improving	Improving		Complete		Complete
12	296	PM	Improving	Improving		Complete	Complete	Complete
12	297	SSG	Stable	Stable				
12	298	PM	Improving	Improving		Complete	Complete	Complete
12	299	Combination	Stable	Improving		Partial		Complete
12	300	PM	Improving	Improving		Complete	Complete	Complete
12	301	SSG	Improving	Improving	Improving	Complete	Complete	Complete
12	302	SSG	Stable	Improving	Improving	Partial	Partial	Complete
12	303	PM	Stable	Improving		Partial	Partial	
12	304	Combination	Improving	Improving		Complete	Complete	Complete
12	305	PM	Improving	Improving		Complete	Partial	Complete
12	306	Combination	Improving	Improving		Partial	Partial	Partial
12	307	Combination	Improving	Improving		Partial	Complete	Complete
12	308	PM	Improving	Improving		Partial	Partial	Complete
12	309	PM	Stable	Improving		Partial	Complete	Complete
12	310	SSG	Improving	Improving	Improving	Partial	Complete	Complete
12	311	SSG	Stable	Stable	Improving	Complete	Partial	Complete
12	312	Combination	Improving	Improving		Partial	Complete	Complete
12	313	PM	Stable	Improving		Complete	Complete	Complete
12	314	SSG	Improving	Improving	Improving	Partial	Complete	Complete
12	315	Combination	Improving	Improving		Partial	Partial	Complete
12	316	SSG	Improving	Improving		Partial	Complete	Complete
12	317	SSG	Improving	Improving	Improving	Partial		Complete

Centre Number	Patient Number	Treatment	Clinical Response to treatment					
			Day 7	Day 14	Day 21	EOT	3Mon FU	6Mon FU
12	318	Combination	Improving	Improving		Partial	Partial	Complete
12	319	Combination	Improving	Improving		Complete	Complete	Complete
12	320	Combination	Improving	Improving		Partial	Partial	Complete
12	321	Combination	Improving	Improving		Partial	Partial	Partial
12	322	PM	Improving	Improving		Partial	Partial	Complete
12	323	SSG	Improving	Improving	Improving	Complete	Complete	Complete
12	324	SSG	Improving	Improving	Improving	Partial	Complete	Complete
12	325	PM	Improving	Improving		Partial	Complete	Complete
12	326	PM	Stable	Improving		Partial	Partial	Complete
12	327	Combination	Improving	Improving		Complete	Complete	Complete
12	328	PM	Stable	Improving		Partial	Complete	Complete
12	329	SSG	Improving	Stable	Improving	Partial	Complete	Partial
12	330	PM	Improving	Improving		Partial	Partial	Partial
23	361	Combination	Improving	Improving		Partial	Partial	Complete
23	362	PM	Stable	Improving		Complete	Complete	Complete
23	363	PM	Improving	Improving		Partial	Complete	Complete
23	364	Combination	Stable	Improving		Complete		Complete
23	365	SSG	Improving	Improving	Improving	Complete	Complete	Complete
23	366	Combination	Improving	Improving		Partial	Complete	Complete
23	367	SSG	Improving	Improving	Improving	Partial	Complete	Complete
23	368	PM	Stable	Improving		Partial	Complete	Complete
23	369	Combination	Stable	Stable		Partial	Complete	Partial
23	370	SSG	Improving	Improving	Improving	Partial	Complete	Complete
23	371	PM	Stable	Stable		Partial	Partial	Partial
23	372	PM	Improving	Stable		Partial	Complete	Complete
23	373	SSG	Improving	Improving	Improving	Partial	Complete	Partial
23	374	SSG	Stable	Improving	Improving	Complete	Complete	Complete
23	375	Combination	Improving	Improving		Partial	Complete	Complete
23	376	SSG	Improving	Stable	Improving	Partial	Complete	Complete
23	377	Combination	Improving					Complete
23	378	Combination	Improving	Improving		Partial	Partial	Complete
23	379	PM	Improving	Improving		Partial		Complete
23	380	SSG	Stable	Improving	Improving	Complete	Complete	Complete
23	381	PM	Improving	Improving		Partial	Partial	Complete
23	382	Combination	Improving	Improving		Partial	Complete	Complete
23	383	PM	Stable	Improving		Complete	Complete	Complete
23	384	SSG	Stable	Stable	Stable	Partial	Complete	Complete
23	385	SSG	Improving	Improving	Improving	Partial	Partial	Complete
23	386	Combination	Stable	Improving		Complete	Complete	Complete
23	387	Combination	Stable	Stable		Partial	Complete	Complete
23	388	PM	Improving	Improving		Partial	Partial	Complete
23	389	SSG	Improving	Improving	Improving	Complete	Complete	Complete
23	390	PM	Improving	Improving		Partial	Complete	Complete
23	391	Combination	Improving	Improving		Partial	Partial	Complete
23	392	Combination	Stable	Improving			Complete	Complete
23	393	SSG	Improving	Improving	Improving	Partial	Complete	Complete
23	394	PM	Improving	Improving		Partial	Complete	Complete
23	395	PM	Improving	Improving		Complete	Complete	Complete
23	396	SSG	Improving	Improving	Improving	Partial	Complete	Complete
23	397	SSG	Improving	Improving	Improving	Partial	Complete	Complete
23	398	Combination	Improving	Improving		Complete	Complete	Partial
23	399	PM	Improving	Improving		Partial	Complete	Complete
23	400	PM	Improving	Improving		Partial	Complete	Partial

Centre Number	Patient Number	Treatment	Clinical Response to treatment Day 7	Day 14	Day 21	EOT	3Mon FU	6Mon FU
23	401	Combination	Stable	Improving		Complete	Partial	Partial
23	402	Combination	Improving	Improving		Partial	Complete	Complete
23	403	SSG	Improving	Improving	Improving	Partial	Complete	Complete
23	404	SSG	Stable	Improving	Improving	Complete		Complete
23	405	PM	Improving	Improving		Partial	Complete	Complete
34	451	Combination	Improving	Improving		Partial		Complete
34	452	PM	Improving	Improving		Partial		
34	453	PM	Stable	Improving		Partial		
34	454	Combination	Improving	Improving		Complete		Complete
34	455	PM	Stable	Stable		Partial		
34	456	SSG	Stable	Improving	Improving	Complete	Partial	
34	457	Combination	Improving	Improving		Partial		
34	458	PM	Stable	Improving		Partial	Partial	
34	459	Combination	Improving	Improving		Partial	Complete	Complete
34	460	PM	Improving	Improving		Complete		
34	461	SSG	Deteriorating	Deteriorating				
34	462	SSG	Improving	Improving	Improving	Complete		Complete
34	463	Combination	Improving	Improving		Complete		Complete
34	464	SSG	Stable	Improving	Improving	Partial		Complete
34	465	SSG	Improving	Improving	Improving	Partial		Complete
34	466	Combination	Improving	Improving		Partial		
34	467	PM	Stable	Improving		Partial	Partial	
34	468	Combination	Improving	Improving		Complete		Complete
34	469	SSG	Improving	Stable	Improving	Partial		Complete
34	470	Combination	Improving	Improving		Partial		Partial
34	471	PM	Stable	Improving		Partial		
34	472	SSG	Improving	Improving	Improving	Partial		Complete
34	473	PM	Improving	Improving		Partial		
34	474	Combination	Improving	Improving		Complete		Complete
34	475	PM	Improving	Improving		Partial		
34	476	PM	Improving	Improving		Partial	Partial	
34	477	SSG	Improving	Improving	Stable	Partial		Partial
34	478	Combination	Improving	Improving		Partial		Complete
34	479	SSG	Improving	Improving	Improving	Partial		Complete
34	480	SSG	Improving	Improving	Improving	Complete		Complete
34	481	Combination	Improving	Improving		Complete		Complete
34	482	Combination	Improving	Improving		Partial		Complete
34	483	PM	Improving	Improving		Complete		Complete
34	484	PM	Deteriorating					
34	485	Combination	Improving	Improving		Partial		Complete
34	486	Combination	Improving	Improving		Partial		Complete
34	487	SSG	Improving	Improving	Improving	Complete		Complete
34	488	SSG	Improving	Improving	Improving	Partial	Partial	Partial
34	489	SSG	Improving	Improving	Improving	Partial		Complete
34	490	PM	Stable	Improving		Partial		Complete
34	491	PM	Stable	Improving		Partial		
34	492	PM	Improving	Improving		Partial		Complete
34	493	SSG	Improving	Improving	Improving	Complete	Partial	
34	494	Combination	Improving	Improving		Partial		Complete
34	495	SSG	Improving	Improving	Improving	Complete		Complete
34	496	SSG	Improving	Improving	Improving	Partial		Complete
34	497	PM	Improving	Improving		Partial	Partial	
34	498	SSG	Improving	Improving	Improving	Complete		Complete

Centre Number	Patient Number	Treatment	Clinical Response to treatment					
			Day 7	Day 14	Day 21	EOT	3Mon FU	6Mon FU
34	499	Combination	Improving	Improving		Partial		Complete
34	500	PM	Improving	Improving		Partial		Complete
34	501	Combination	Improving	Improving		Partial		Complete
34	502	Combination	Improving	Improving		Partial		Complete
34	503	PM	Improving	Improving		Partial		
34	504	Combination	Improving	Improving		Partial		
34	505	SSG	Improving	Improving	Improving	Complete		Complete
34	506	SSG	Improving	Improving	Improving	Partial		Complete
34	507	SSG	Improving	Improving	Improving	Complete		Complete
34	508	PM	Stable	Improving		Complete		
34	509	PM	Improving	Improving		Complete		
34	510	Combination	Improving	Improving		Complete		Complete
34	511	Combination	Improving	Improving		Partial		Partial
34	512	PM	Stable	Improving		Partial		
34	513	Combination	Improving	Improving		Partial	Partial	Complete
34	514	SSG	Improving	Improving	Improving	Partial		Complete
34	515	SSG	Improving	Improving	Improving	Complete	Partial	
34	516	PM	Improving	Improving		Partial		Partial
34	517	PM	Stable	Improving		Partial		
34	518	SSG	Improving	Improving	Improving	Partial		Partial
34	519	PM	Stable	Improving		Partial	Partial	
34	520	SSG	Improving	Improving	Improving	Partial		Complete
34	521	PM	Improving	Improving		Partial		
34	522	Combination	Improving	Improving		Partial		
34	523	SSG	Improving	Improving	Improving	Complete		Complete
34	524	Combination	Improving	Improving		Complete		Complete
34	525	Combination	Improving	Improving		Partial		Complete
34	526	SSG	Improving	Improving	Improving	Complete		Partial
34	527	Combination	Improving	Improving		Complete		Complete
34	528	PM	Improving	Improving		Partial		
34	529	Combination	Improving	Improving		Complete		Complete
34	530	Combination	Improving	Improving		Partial		Partial
34	531	SSG	Improving	Improving	Improving	Partial		
34	532	Combination	Improving	Improving		Partial		Complete
34	533	SSG	Improving	Improving	Improving	Partial		Partial
34	534	PM	Stable	Improving		Partial		
34	535	PM	Improving	Improving		Partial	Partial	
34	536	PM	Improving	Improving		Partial		Complete
34	537	SSG	Improving	Improving	Improving	Partial		Complete
34	538	PM	Stable	Improving		Partial		
34	539	Combination	Improving	Improving		Complete		Partial
34	540	SSG	Improving	Improving	Improving	Partial		Partial
35	646	PM	Improving	Improving		Partial		Complete
35	647	PM	Improving	Improving		Partial	Complete	Complete
35	648	Combination	Improving	Improving		Partial	Complete	Complete
35	649	Combination	Improving	Improving		Partial	Complete	Complete
35	650	SSG	Improving	Improving	Improving	Partial	Complete	Complete
35	651	SSG	Improving	Improving	Improving	Partial	Complete	Complete
35	652	PM	Stable	Improving		Partial		Complete
35	653	Combination	Improving	Improving		Partial	Complete	Complete
35	654	SSG	Improving	Improving	Improving	Partial	Complete	Complete
35	655	Combination	Improving	Improving		Partial	Complete	Complete
35	656	SSG	Improving	Improving	Improving	Partial	Complete	Complete

Centre Number	Patient Number	Treatment	Clinical Response to treatment					
			Day 7	Day 14	Day 21	EOT	3Mon FU	6Mon FU
35	657	PM	Improving	Improving		Partial	Complete	Complete
35	658	Combination	Improving	Improving		Partial	Complete	Complete
35	659	SSG	Stable	Improving	Improving	Partial	Complete	Complete
35	660	PM	Improving	Improving		Partial	Complete	Complete
35	661	Combination	Improving	Improving		Partial	Complete	Complete
35	662	PM	Stable	Improving		Partial	Complete	Complete
35	663	SSG	Improving	Improving	Improving	Partial	Partial	Partial
35	664	Combination	Improving					Complete
35	665	SSG	Improving	Improving	Improving	Partial	Complete	Complete
35	666	SSG	Improving	Improving	Improving	Partial	Complete	Complete
35	667	Combination	Improving	Improving		Partial	Complete	Complete
35	668	PM	Improving	Improving		Partial		Complete
35	669	PM	Improving	Improving		Partial		
35	670	SSG	Improving	Improving	Improving	Partial	Complete	Complete
35	671	PM	Improving	Improving		Partial		Complete
35	672	Combination	Improving	Improving		Partial		Partial
35	673	SSG	Improving	Improving	Improving	Partial	Complete	Complete
35	674	Combination	Stable	Improving		Partial	Complete	Complete
35	675	PM	Stable	Improving		Partial		Complete
35	676	PM	Stable	Improving		Partial	Partial	Complete
35	677	SSG	Stable					
35	678	Combination	Improving	Improving		Partial	Complete	Complete
35	679	SSG	Improving	Improving	Improving	Partial	Complete	Complete
35	680	Combination	Improving	Improving		Partial	Complete	Complete
35	681	Combination	Improving	Improving		Partial	Complete	Complete
35	682	PM	Improving	Improving		Partial		Complete
35	683	Combination	Improving	Improving		Partial		Complete
35	684	SSG	Stable	Improving	Improving	Partial	Complete	Complete
35	685	PM	Stable	Improving		Partial	Complete	Complete
35	686	PM	Improving	Improving		Partial	Complete	Complete
35	687	SSG	Improving	Improving	Improving	Partial	Complete	Complete
35	688	PM	Improving	Improving		Partial	Complete	Complete
35	689	Combination	Improving	Improving		Partial	Complete	Complete
35	690	SSG	Improving	Improving	Improving	Partial	Complete	Complete

Centre Number	Patient Number	Treatment	Parasite Count Baseline	EOT	3Mon FU	6Mon FU
11	1	PM	4	1	0	0
11	2	Combination	5	2	0	0
11	3	Combination	5	0		0
11	4	PM	4	0		0
11	5	PM	3	0		0
11	6	Combination	4	0	0	0
11	7	PM	2	0	1	0
11	8	SSG	4	0		0
11	9	SSG	3	0	0	0
11	10	SSG	5	1		0
11	11	Combination	3	0		0
11	12	Combination	3	0		0
11	13	SSG	4	0		0
11	14	SSG	2	0	0	0
11	15	PM	2		0	0
11	16	Combination	3	0		0
11	17	Combination	3	0	0	0
11	18	SSG	3	0	0	0
11	19	Combination	3	0	0	0
11	20	SSG	3	0	0	0
11	21	SSG	2	1	0	0
11	22	SSG	3	0		0
11	23	PM	2	0		0
11	24	PM	3	0	0	0
11	25	SSG	4	0		0
11	26	Combination	2	0		0
11	27	PM	5	1	6	
11	28	Combination	3	0	0	0
11	29	PM	3	0	0	0
11	30	PM	4	1		0
11	31	SSG	3	0		0
11	32	Combination	3	0		0
11	33	SSG	3	0		0
11	34	PM	5	1	0	0
11	35	SSG	4	1	0	0
11	36	SSG	2	0		0
11	37	Combination	3	0		0
11	38	PM	2	0	0	0
11	39	PM	2	0	0	0
11	40	PM	3			0
11	41	Combination	3	0		0
11	42	Combination	5	0		0
11	43	SSG	3	0	0	0
11	44	PM	3	0	0	0
11	45	Combination	3	0		0
11	46	SSG	3	0		0
11	47	SSG	4	0		
11	48	Combination	4	0		0
11	49	SSG	1	0		0
11	50	PM	3	2	0	0
11	51	Combination	3	0		0
11	52	PM	3	0	0	0
11	53	SSG	5	0		0

Centre Number	Patient Number	Treatment	Parasite Count Baseline	EOT	3Mon FU	6Mon FU
11	54	PM	6	5	5	6
11	55	PM	2	1		0
11	56	SSG	6	0	0	0
11	57	Combination	4	0	0	0
11	58	Combination	4	0		0
11	59	PM	4	0		0
11	60	Combination	3	0	0	0
11	61	PM	2	0		0
11	62	SSG	2	0		0
11	63	SSG	6	2	0	0
11	64	SSG	2	0		0
11	65	PM	2	0		
11	66	Combination	3	0		0
11	67	PM	4	0		0
11	68	SSG	4	0	0	0
11	69	Combination	1	0	0	0
11	70	Combination	4	0		0
11	71	Combination	6	5	5	6
11	72	SSG	6			0
11	73	PM	1	0	0	0
11	74	PM	2	0		0
11	75	Combination	6	3	3	0
11	76	PM	2	0		0
11	77	PM	3	0	0	0
11	78	SSG	2	0	0	0
11	79	PM	4	0	0	0
11	80	PM	4	0		0
11	81	Combination	6	0	0	0
11	82	SSG	3	0		0
11	83	SSG	5	0		0
11	84	SSG	4	0		0
11	85	SSG	5	0		
11	86	Combination	3	0		0
11	87	Combination	2	0		0
11	88	PM	1	0		0
11	89	Combination	2	0	0	0
11	90	Combination	1	0		
11	91	PM	4	2	5	2
11	92	Combination	3	0		0
11	93	SSG	2	0		0
11	94	SSG	2	0		0
11	95	SSG	1	0		0
11	96	Combination	2	0		0
11	97	Combination	4	1	0	0
11	98	PM	2	0		0
11	99	SSG	4	0		0
11	100	PM	6	4		
11	101	PM	2	0		0
11	102	Combination	2	0		0
11	103	Combination	2	0		0
11	104	PM	4	3		0
11	105	SSG	2	0	0	0
11	106	Combination	3	0		0

Centre Number	Patient Number	Treatment	Parasite Count Baseline	EOT	3Mon FU	6Mon FU
11	107	PM	2	0		0
11	108	PM	2	0		0
11	109	SSG	3	0		0
11	110	PM	4	0	0	0
11	111	Combination	2	0		0
11	112	Combination	5	0		0
11	113	SSG	1	0		
11	114	SSG	2	0		0
11	115	Combination	3	0		0
11	116	SSG	2	0		0
11	117	PM	3	0		
11	118	PM	2	0		0
11	119	SSG	1	0		0
11	120	Combination	3	0		0
11	121	Combination	2	0		0
11	122	SSG	2	0		
11	123	SSG	4	0	0	0
11	124	SSG	2	0		0
11	125	SSG	2	0		0
11	126	Combination	6	4		2
11	127	PM	3	0		0
11	128	Combination	5	0		6
11	129	PM	3	1	0	0
11	130	Combination	3	0		0
11	131	PM	3	1		0
11	132	SSG	3	0		0
11	133	Combination	5	0		0
11	134	PM	2	1	0	0
11	135	PM	4	0		0
12	241	Combination	5	0		0
12	242	SSG	5	0	0	0
12	243	PM	4	0		0
12	244	SSG	2	0		0
12	245	PM	5	0	5	
12	246	PM	6	4	0	
12	247	PM	3	0		0
12	248	PM	2	0		0
12	249	Combination	4	0	0	0
12	250	SSG	4	0		0
12	251	Combination	5	0		0
12	252	SSG	3	0		0
12	253	SSG	2	0		0
12	254	Combination	3	0		0
12	255	Combination	3	0		0
12	256	Combination	4	0		0
12	257	SSG	3	0		0
12	258	SSG	3	0		0
12	259	PM	5	0		0
12	260	PM	2	0		0
12	261	Combination	4	0		0
12	262	PM	2	0		0
12	263	SSG	3	0		0
12	264	Combination	4	0		0

Centre Number	Patient Number	Treatment	Parasite Count Baseline	EOT	3Mon FU	6Mon FU
12	265	SSG	4			
12	266	PM	4	0	0	0
12	267	SSG	4	0		0
12	268	PM	3	0		0
12	269	Combination	5	0		0
12	270	Combination	6	0		0
12	271	SSG	5	0		0
12	272	PM	4	0		0
12	273	SSG	3	0		0
12	274	SSG	4	0		0
12	275	Combination	4	0		0
12	276	PM	3	0		0
12	277	Combination	5	0		0
12	278	SSG	3	0		
12	279	PM	6	0		0
12	280	SSG	3	0		0
12	281	Combination	4	0		0
12	282	PM	5	0		0
12	283	PM	2	0		0
12	284	Combination	5	0		5
12	285	Combination	5	0		0
12	286	PM	5	0		0
12	287	SSG	2	0		0
12	288	Combination	5	0		0
12	289	Combination	3	0		0
12	290	SSG	2	0		0
12	291	SSG	5	0		0
12	292	PM	5	0		0
12	293	SSG	5	0		0
12	294	Combination	5	0		0
12	295	Combination	5	0		0
12	296	PM	5	0		0
12	297	SSG	5			
12	298	PM	5	0		0
12	299	Combination	4	0		0
12	300	PM	5	0		0
12	301	SSG	4	0		0
12	302	SSG	5	0		0
12	303	PM	5	0		0
12	304	Combination	5	0		0
12	305	PM	5	0		0
12	306	Combination	5	0		0
12	307	Combination	5	0		0
12	308	PM	5	0	0	0
12	309	PM	3	0		0
12	310	SSG	2	0		0
12	311	SSG	3	0		0
12	312	Combination	5	0		0
12	313	PM	5	0		0
12	314	SSG	5	0		0
12	315	Combination	6	0	3	0
12	316	SSG	2	0	0	0
12	317	SSG	3	0		0

Centre Number	Patient Number	Treatment	Parasite Count Baseline	EOT	3Mon FU	6Mon FU
12	318	Combination	5	0		0
12	319	Combination	5	0		0
12	320	Combination	4	0		0
12	321	Combination	4	0		0
12	322	PM	6	0		0
12	323	SSG	4	0		0
12	324	SSG	6	0		0
12	325	PM	4	0		0
12	326	PM	5	0	0	0
12	327	Combination	2	0		0
12	328	PM	5	0		0
12	329	SSG	3	0		0
12	330	PM	4	0	0	0
23	361	Combination	6	0		0
23	362	PM	4	0		0
23	363	PM	6	0		0
23	364	Combination	6	0	6	0
23	365	SSG	5	0		0
23	366	Combination	4	0		0
23	367	SSG	5	0		0
23	368	PM	5	0		0
23	369	Combination	6	0		0
23	370	SSG	6	0		0
23	371	PM	5	0	0	0
23	372	PM	4	0		0
23	373	SSG	4	0		0
23	374	SSG	4	0		0
23	375	Combination	2	0		0
23	376	SSG	5	0		0
23	377	Combination	5			0
23	378	Combination	6	0	5	0
23	379	PM	6	0	6	
23	380	SSG	3	0		0
23	381	PM	2	0	0	0
23	382	Combination	4	0	0	0
23	383	PM	5	0	0	0
23	384	SSG	4	0		0
23	385	SSG	4	0		0
23	386	Combination	4	0		0
23	387	Combination	4	0		0
23	388	PM	4	0		0
23	389	SSG	4	0		0
23	390	PM	2	0		0
23	391	Combination	3	0		0
23	392	Combination	4			0
23	393	SSG	4	0		0
23	394	PM	4	0		0
23	395	PM	6	3	0	0
23	396	SSG	6	0		0
23	397	SSG	3	0		0
23	398	Combination	6	0	0	4
23	399	PM	4	0	0	0
23	400	PM	5	2	0	1

Centre	Patient	Treatment	Parasite Count			
Number	Number		Baseline	EOT	3Mon FU	6Mon FU
23	401	Combination	5	0	6	6
23	402	Combination	6	0		0
23	403	SSG	5	0		0
23	404	SSG	4	0		0
23	405	PM	4	0		0
34	451	Combination	2	0		0
34	452	PM	1	2		
34	453	PM	3	3		
34	454	Combination	3	0		0
34	455	PM	2	3		
34	456	SSG	3	1	3	
34	457	Combination	2	0		
34	458	PM	4	1	2	
34	459	Combination	2	1	0	0
34	460	PM	1	1		
34	461	SSG	3			
34	462	SSG	6	0		0
34	463	Combination	2	0		0
34	464	SSG	2	0		0
34	465	SSG	1	0		0
34	466	Combination	1	1		
34	467	PM	2	0	1	
34	468	Combination	1	0		0
34	469	SSG	2	0		0
34	470	Combination	4	0		0
34	471	PM	2	2		
34	472	SSG	1	0		0
34	473	PM	1	1		
34	474	Combination	2	0		0
34	475	PM	6	5		
34	476	PM	5	3	2	
34	477	SSG	2	0		0
34	478	Combination	1	0		0
34	479	SSG	1	0		0
34	480	SSG	4	0		0
34	481	Combination	2	0		0
34	482	Combination	1	0		0
34	483	PM	1	0		0
34	484	PM	1			
34	485	Combination	2	0		0
34	486	Combination	1	0		0
34	487	SSG	2	0		0
34	488	SSG	5	1	0	0
34	489	SSG	4	0		0
34	490	PM	4	0		0
34	491	PM	1	1		
34	492	PM	1	0		0
34	493	SSG	4	0	0	
34	494	Combination	4	0		0
34	495	SSG	2	0		0
34	496	SSG	4	0		0
34	497	PM	1	0	2	
34	498	SSG	1	0		0

Centre Number	Patient Number	Treatment	Parasite Count Baseline	EOT	3Mon FU	6Mon FU
34	499	Combination	2	0		0
34	500	PM	1	0		0
34	501	Combination	5	0		0
34	502	Combination	1	0		0
34	503	PM	2	1		
34	504	Combination	2	1		
34	505	SSG	2	0		0
34	506	SSG	2	0		0
34	507	SSG	5	0		0
34	508	PM	2	1		
34	509	PM	3	2		
34	510	Combination	2	0		0
34	511	Combination	2	0		0
34	512	PM	4	2		
34	513	Combination	6	1	0	0
34	514	SSG	2	0		0
34	515	SSG	1	0	1	
34	516	PM	1	0		0
34	517	PM	1	3		
34	518	SSG	3	0		0
34	519	PM	1	0	1	
34	520	SSG	3	0		0
34	521	PM	1	1		
34	522	Combination	1	0		
34	523	SSG	3	0		0
34	524	Combination	3	0		0
34	525	Combination	1	0		0
34	526	SSG	2	0		2
34	527	Combination	2	0		0
34	528	PM	2	1		
34	529	Combination	3	0		0
34	530	Combination	4	0		0
34	531	SSG	2	1		
34	532	Combination	3	0		0
34	533	SSG	1	0		0
34	534	PM	2	1		
34	535	PM	3	0	2	
34	536	PM	1	0		0
34	537	SSG	2	0		0
34	538	PM	4	3		
34	539	Combination	1	0		0
34	540	SSG	3	0		0
35	646	PM	1	1		0
35	647	PM	1	0		0
35	648	Combination	1	0		0
35	649	Combination	3	1	0	0
35	650	SSG	1	0		0
35	651	SSG	1	0		0
35	652	PM	2	1		0
35	653	Combination	4	2	0	0
35	654	SSG	1	0		0
35	655	Combination	1	0		0
35	656	SSG	1	0		0

Centre Number	Patient Number	Treatment	Parasite Count Baseline	EOT	3Mon FU	6Mon FU
35	657	PM	1	0		0
35	658	Combination	2	0		0
35	659	SSG	2	0		0
35	660	PM	1	0		0
35	661	Combination	1	0		0
35	662	PM	6	4		0
35	663	SSG	4	0		0
35	664	Combination	2			0
35	665	SSG	2	0		0
35	666	SSG	5	0		0
35	667	Combination	1	0		0
35	668	PM	2	1		0
35	669	PM	6	6		
35	670	SSG	1	0		0
35	671	PM	4	3		0
35	672	Combination	3	0		0
35	673	SSG	2	0		0
35	674	Combination	6	0		0
35	675	PM	1	0		0
35	676	PM	1	0		0
35	677	SSG	1			
35	678	Combination	2	0		0
35	679	SSG	1	0		0
35	680	Combination	2	0		0
35	681	Combination	1	0		0
35	682	PM	1	0		0
35	683	Combination	1	0		0
35	684	SSG	4	0		0
35	685	PM	1	0		0
35	686	PM	2	0		0
35	687	SSG	1	0		0
35	688	PM	1	0		0
35	689	Combination	1	0		0
35	690	SSG	3	0		0

Centre Number	Patient Number	Treatment	Spleen size (cm) Baseline	Day7	Day 14	Day 21	EOT	3 mon FU	6 mon FU
11	1	PM	5	5	5		5	4.5	5
11	2	Combination	9	9	6		5	4	7
11	3	Combination	10	8	4		4	0.5	0.5
11	4	PM	5	5	5		3.5	2	0
11	5	PM	3	3	4		3.5	2.5	0
11	6	Combination	14	13	10		6	5	2.5
11	7	PM	9	8	7		5	7	6
11	8	SSG	4	5	4	2	1.5	1.5	2
11	9	SSG	5	5	5	5	4.5	3	4
11	10	SSG	5	5	4	3	2.5	0	0
11	11	Combination	4	3.5	3		3	0	0
11	12	Combination	5	3.5	4		3	0	0
11	13	SSG	8	7.5	6	6	6	2	0
11	14	SSG	9	5.5	4	7	6	4	3
11	15	PM	8	5.5				5.5	5
11	16	Combination	0	0	0		0	0	0
11	17	Combination	5.5	5.5	4		4	7	5
11	18	SSG	9	9	8	7.5	6	5.5	5
11	19	Combination	15	14	12		11.5	14	11
11	20	SSG	9	7	7	7	7	5.5	5
11	21	SSG	11	9	8	9.5	7	3	3
11	22	SSG	6	6	3	2	2	0	0
11	23	PM	10	10	7		5	2	3
11	24	PM	8	7	6		5	5	5
11	25	SSG	9	6.5	6	5	3.5	2.5	0
11	26	Combination	6	3	3		3	2	2
11	27	PM	7	7	5.5		5.5	6	
11	28	Combination	10	9	7		6	4	3
11	29	PM	6	5	4		4	4	2
11	30	PM	6	5.5	5		4	2.5	1
11	31	SSG	6.5	5	5	3	5	2.5	1
11	32	Combination	6	0.5	0		0	0	0
11	33	SSG	7	4	2	2	0	0	0
11	34	PM	10	10	9		8	5	4.5
11	35	SSG	13	13	10	10	10	9	8
11	36	SSG	3	3	4	5	5	2	0
11	37	Combination	4.5	3	0		0	0	0
11	38	PM	5	5	6		6	3	3
11	39	PM	13	11	10		6	5	1
11	40	PM	8	6	5.5			2	1
11	41	Combination	6	4	3		3	1	0
11	42	Combination	3	0.5	0		0	0	0
11	43	SSG	6	6	5.5	5.5	5.5	4	1
11	44	PM	10	6	5		3	2	0
11	45	Combination	4	2.5	2		2	2	0

Centre Number	Patient Number	Treatment	Spleen size (cm) Baseline	Day7	Day 14	Day 21	EOT	3 mon FU	6 mon FU
11	46	SSG	6	6	4	3	2.5	0	0
11	47	SSG	7	5	3	3	3		
11	48	Combination	6.5	6.5	4		3		2
11	49	SSG	4	3	3	2	2.5	0	0
11	50	PM	14	12	10		4	3	2
11	51	Combination	6	6	4		4.5	0	0
11	52	PM	11	10	7.5		6.5	4	5
11	53	SSG	6	6	5	3	3	0	0
11	54	PM	6	5	4		1	3	5
11	55	PM	5	5	4		3	0	0
11	56	SSG	7	8	6	6	5	3	2
11	57	Combination	8	7	4		4	4.5	4
11	58	Combination	7	6	5		3.5	0	0
11	59	PM	4	4	3.5		1	1	0
11	60	Combination	7	5	4		3.5	3	2
11	61	PM	7	7	4		3	2.5	2
11	62	SSG	8	6	5	4	4	2.5	1.5
11	63	SSG	6	5	4	3.5	3	3	3
11	64	SSG	6.5	5	5	4	4	2.5	2
11	65	PM	6	8	8		4		
11	66	Combination	4.5	4	2.5		2.5	0	0
11	67	PM	4	4	4		2.5	2	0
11	68	SSG	5	5	4	3	3	3	2
11	69	Combination	8	7.5	5		5	5	4
11	70	Combination	6	5	3		3	0	1
11	71	Combination	8	8	8		8	6	13
11	72	SSG	7	6	6			2.5	3
11	73	PM	9	7	7		5	4	2
11	74	PM	3	2	0		0	0	0
11	75	Combination	7	8	6		5	4	5
11	76	PM	4	4	3		3	0	0
11	77	PM	5.5	5	3.5		3	3.5	2.5
11	78	SSG	6	5	5	3	4	3.5	3
11	79	PM	4	4	3.5		3	3.5	3
11	80	PM	9.5	7	6		5	2	2
11	81	Combination	8	7	5		5	5	3
11	82	SSG	8	4.5	6	5	3.5	1.5	1
11	83	SSG	4	4	3.5	4	3.5	0	0
11	84	SSG	4	3	2	3	2	0	0
11	85	SSG	7	5	5	6	3.5	2	
11	86	Combination	3	2	0		0	0	0
11	87	Combination	5	5	5		4	2.5	1
11	88	PM	2.5	1	1		1	0	0
11	89	Combination	6	5	5		4.5	4	0
11	90	Combination	7	5	3.5		3.5		

Centre Number	Patient Number	Treatment	Spleen size (cm) Baseline	Day7	Day 14	Day 21	EOT	3 mon FU	6 mon FU
11	91	PM	6	5	4		2.5	3	4
11	92	Combination	5	5	5		5	2	0
11	93	SSG	4.5	3	2	3	1.5	0	0
11	94	SSG	9	8	5	4	3	2.5	2.5
11	95	SSG	3.5	1	2	2	1	0	0
11	96	Combination	4	3.5	3.5		3.5	0	0
11	97	Combination	6.5	5	4		3	3	0
11	98	PM	4	3.5	3		2	2.5	0
11	99	SSG	6.5	5.5	4.5	3.5	3	1.5	0
11	100	PM	6.5	5	4.5		4.5		
11	101	PM	5	5	3.5		3.5	2.5	2
11	102	Combination	7.5	6	3.5		3.5	0	0
11	103	Combination	5	3.5	2.5		2	0	0
11	104	PM	5	4	4		4	2	2
11	105	SSG	8	6	6	6	6	4	2.5
11	106	Combination	4	2	0		0	0	0
11	107	PM	8	4	3		3	2.5	2
11	108	PM	7	5	3		0	0	0
11	109	SSG	6.5	5	4	4	4	2	1.5
11	110	PM	6	6	6		5	3.5	2
11	111	Combination	4	4	0		0	0	0
11	112	Combination	7	4.5	3		2.5	0	0
11	113	SSG	6.5	6.5	4.5	4	4	2.5	
11	114	SSG	4	3.5	3	3	3	2	0
11	115	Combination	5.5	5	4		3	0	0
11	116	SSG	5	4.5	3	3	2.5	0	0
11	117	PM	7	4.5	4		2.5	2	
11	118	PM	4	4	3.5		3	2.5	2
11	119	SSG	6	4.5	4	4	4	2.5	2.5
11	120	Combination	7	3	2		2	0	0
11	121	Combination	3.5	4.5	3		2.5	0	0
11	122	SSG	4.5	4	4	3.5	3		
11	123	SSG	4.5	4.5	4.5	4	3.5	3	2
11	124	SSG	4	3.5	3	2.5	2.5		0
11	125	SSG	4.5	3	3	2.5	0	0	0
11	126	Combination	4	3	2.5		2.5		5
11	127	PM	6	3.5	3		3	1.5	1.5
11	128	Combination	4.5	3	2.5		2.5	2	6
11	129	PM	3.5	3.5	3.5		2.5	0	0
11	130	Combination	3.5	3	1		0	0	0
11	131	PM	4	4	3		3	0	0
11	132	SSG	5	3	3	2.5	2	0	4
11	133	Combination	7.5	7.5	5		4	0	0
11	134	PM	6.5	6.5	5		5	5	5
11	135	PM	4	3	3		2	1	0

Centre Number	Patient Number	Treatment	Spleen size (cm) Baseline	Day7	Day 14	Day 21	EOT	3 mon FU	6 mon FU
12	241	Combination	8	4	3		3		0
12	242	SSG	8	9	4	1.5	0.5	0	0
12	243	PM	7	7	7		5.5	0	0
12	244	SSG	11	7	7	6	6	3	2
12	245	PM	12	8	8		5.5	8	
12	246	PM	3	0.5	0		0	0	
12	247	PM	12.5	10	10		10	1	0
12	248	PM	8.5	7	6		4	0	0
12	249	Combination	15	15	15		12	5	11
12	250	SSG	12	10	9	8	6	4	2
12	251	Combination	12	8	4		3	0	0
12	252	SSG	8	3	1.5	0	0	0	0
12	253	SSG	8	5	4	3	2	0	0
12	254	Combination	9	8	8		6	3	0
12	255	Combination	10	7	5		3	0	0
12	256	Combination	6	5	3		3	0	0
12	257	SSG	16	10	8	8	7	1	0
12	258	SSG	9	7	6	5	4		0
12	259	PM	8	7	5		0	0	0
12	260	PM	4	4	2		0		0
12	261	Combination	9	9	7		6	0	0
12	262	PM	7	6	5		3	0	0
12	263	SSG	7	5	3	1	0	0	0
12	264	Combination	10	8	9		9	3	2
12	265	SSG	14	13	11	9	13		
12	266	PM	12	10	10		9	5	4
12	267	SSG	2	1	0	0	0	0	0
12	268	PM	11	10	8		4	0	4
12	269	Combination	12	10	9		7	1	0
12	270	Combination	7	6	4		2.5		0
12	271	SSG	9	7	4	3	3	0	0
12	272	PM	5	4.5	3.5		3	0.5	0
12	273	SSG	9	7	5	4	3	0	0
12	274	SSG	4	3	2	2	1	0	0
12	275	Combination	12	11	10		10	3	3
12	276	PM	12	10	7		5	4	2
12	277	Combination	12	8	6		4	0	0
12	278	SSG	8	5	3	2	1	0	
12	279	PM	13	11	8		6	2	0
12	280	SSG	15	12	8	6	4	0	2
12	281	Combination	6	5	4		3	0	0
12	282	PM	13	10	6		6	2	2
12	283	PM	4	3	2		0	0	0
12	284	Combination	10	8	7		6	3	6
12	285	Combination	8	5	0		0	0	0

Centre Number	Patient Number	Treatment	Spleen size (cm) Baseline	Day7	Day 14	Day 21	EOT	3 mon FU	6 mon FU
12	286	PM	18	17.5	10		8	2	1
12	287	SSG	10	8.5	8	6	3	0	0
12	288	Combination	13	12	8		6	0	0
12	289	Combination	12.5	12	8		4	0	3
12	290	SSG	14	13	10	6	2		0
12	291	SSG	9	7	5	5	4	0	3
12	292	PM	16	15	14		13		0
12	293	SSG	8	7	6	5	3		0
12	294	Combination	8	8	6		5	4	0
12	295	Combination	8	0	0		0		0
12	296	PM	8	4	2		0	0	0
12	297	SSG	4	1	0				
12	298	PM	7	6	5		3	0	0
12	299	Combination	14	13.5	10		7		0
12	300	PM	10	9.5	7		1	5	0
12	301	SSG	10	3	1	1	0	0	0
12	302	SSG	15	15	14	10	6	2	0
12	303	PM	25	25	18		15	1	3
12	304	Combination	13	6	5		3	0	0
12	305	PM	13	13	10		8	3	0
12	306	Combination	20	18	15		10	2	4
12	307	Combination	17	10	10		8	0	0
12	308	PM	20	13	10		6	3	0
12	309	PM	11.5	10.5	6		3	0	0
12	310	SSG	13	12	8	6	4	0	0
12	311	SSG	16	10	10	8	5	2	0
12	312	Combination	22	18	10		7	0	0
12	313	PM	18	14	10		0	0	0
12	314	SSG	14	12	4	5	3	0	0
12	315	Combination	16	18	17		11	11	0
12	316	SSG	18	10	6		3	0	0
12	317	SSG	12	11	10	7	6		0
12	318	Combination	10	5	3		3	0	0
12	319	Combination	7	7	2		0	0	0
12	320	Combination	13	6	4		3	0	0
12	321	Combination	20	15	15		10	3	7
12	322	PM	18	8	8		4	2	0
12	323	SSG	6	5	4	3	0	0	0
12	324	SSG	10	12	10	8	6	2	0
12	325	PM	9	10	8		3	0	0
12	326	PM	10	10	4		4	7	0
12	327	Combination	2	0	0		0	0	0
12	328	PM	8	11	6		3	0	0
12	329	SSG	9	7	8	6	3	0	2
12	330	PM	9	6	5		4	4	3

Centre Number	Patient Number	Treatment	Spleen size (cm) Baseline	Day7	Day 14	Day 21	EOT	3 mon FU	6 mon FU
23	361	Combination	17	14.5	12.5		12	1.5	0
23	362	PM	15.5	13	12		7	0	0
23	363	PM	8	9.5	7		3.5	0	0
23	364	Combination	18	17	11.5		11	16	3
23	365	SSG	11.5	9.5	6.5	3	0	0	0
23	366	Combination	15.5	15	9		9	0	0
23	367	SSG	18	18	17	12	11	0	0
23	368	PM	19	16	10.5		9	0	0
23	369	Combination	15	15	10		9	0	5
23	370	SSG	10.5	10	3.5	3	3	0	0
23	371	PM	22.5	22	22.5		16	6.5	4
23	372	PM	8	6	5.5		3	0	0
23	373	SSG	13.5	14	9.5	8	6	0	6
23	374	SSG	8.5	8.5	5.5	4.5	0	0	0
23	375	Combination	8	6.5	3.5		1	0	0
23	376	SSG	13	9	5	4.5	1	0	0
23	377	Combination	13	11					0
23	378	Combination	6	6	6		6.5	9.5	1
23	379	PM	12	11	9		8	10	0
23	380	SSG	8	7.5	7.5	4	4	0	0
23	381	PM	14.5	14	10.5		4	6	0
23	382	Combination	15	14	12		9	5	0
23	383	PM	11.5	11	8		5	0	0
23	384	SSG	13	13	10	10	9	0	0
23	385	SSG	16	15	13	13	12.5	1.5	0
23	386	Combination	15	15	8		5	0	0
23	387	Combination	15	16	16		14	1	0
23	388	PM	9	9	6		5	5	0
23	389	SSG	20	16	14	11.5	10	0	0
23	390	PM	13.5	12	10		9	1	0
23	391	Combination	16	13	8		9	0.5	0
23	392	Combination	11	8	6			0	0
23	393	SSG	7	7	4.5	4.5	4.5	0	0
23	394	PM	11	11	10.5		7	0	0
23	395	PM	10	7	3.5		0	0	0
23	396	SSG	23	20	17	15	13	1	0
23	397	SSG	18	13	11	10	9	0	0
23	398	Combination	16	14.5	12		10	5.5	16
23	399	PM	18	21	17		14	12.5	9
23	400	PM	19	20	16.5		16	7	4
23	401	Combination	10.5	11	7.5		7	20	19
23	402	Combination	15	14.5	10		10	0	0
23	403	SSG	14	9.5	9	7	6	0	0
23	404	SSG	16	8	7	5	1		0
23	405	PM	20	13	13		13	3	0

Centre Number	Patient Number	Treatment	Spleen size (cm) Baseline	Day7	Day 14	Day 21	EOT	3 mon FU	6 mon FU
34	451	Combination	6	4	2.5		2		0
34	452	PM	5	5	5		5		
34	453	PM	6.5	6	5		4		
34	454	Combination	3	3	1		0		0
34	455	PM	8	5	5		5		
34	456	SSG	4.5	2.5	1	0	0	3	
34	457	Combination	6	4	1		0		
34	458	PM	6.5	6.5	6		6	10	
34	459	Combination	3.5	3.5	1		0	0	0
34	460	PM	3	2	1		0		
34	461	SSG	10	8.5	8.5				
34	462	SSG	2	1	1	0	0		0
34	463	Combination	3	2	0		0		0
34	464	SSG	13	13	12	11	7		0
34	465	SSG	10	8	5	5	5		0
34	466	Combination	3	1	0		0		
34	467	PM	10	9	7		4	10	
34	468	Combination	0	0	0		0		0
34	469	SSG	9	9	6	5	3		0
34	470	Combination	9	6	4		3		4
34	471	PM	4	2	0		4		
34	472	SSG	1	1	0	0	0		0
34	473	PM	4.5	3	3		3		
34	474	Combination	4	3	1		0		0
34	475	PM	14.5	14	13		10		
34	476	PM	14	13	13		12	12.5	
34	477	SSG	7	4	2	3	4		4
34	478	Combination	11	8	3		2		0
34	479	SSG	2	2	0	0	0		0
34	480	SSG	2	2	0	0	0		0
34	481	Combination	3	2	0		0		0
34	482	Combination	10	6	0		0		0
34	483	PM	0	0	0		0		0
34	484	PM	5	5					
34	485	Combination	0	0	0		0		0
34	486	Combination	14	10	6		5		0
34	487	SSG	3	3	0	0	0		0
34	488	SSG	14	10	6	6	6	3	3
34	489	SSG	6.5	6	6	5	2		0
34	490	PM	14	8	13		11		0
34	491	PM	17	15	13.5		11		
34	492	PM	0.5	0	0		0		0
34	493	SSG	7	5	3	3	0	4	
34	494	Combination	6	2	0		0		0
34	495	SSG	2	0	0	0	0		0

Centre Number	Patient Number	Treatment	Spleen size (cm) Baseline	Day7	Day 14	Day 21	EOT	3 mon FU	6 mon FU
34	496	SSG	6	4	2	0	0		0
34	497	PM	8	13	13		12	18	
34	498	SSG	0	0	0	0	0		0
34	499	Combination	9	7	4		4		0
34	500	PM	0	2	3		1		0
34	501	Combination	4	3.5	1		0		0
34	502	Combination	9	8	6		2		0
34	503	PM	3	5	2		0		
34	504	Combination	5	2	0		0		
34	505	SSG	2	1	0	0	0		0
34	506	SSG	3	2	1	0	0		0
34	507	SSG	3	3	3	0	0		0
34	508	PM	3	2.5	2.5		0		
34	509	PM	6	5	2		0		
34	510	Combination	0	0	0		0		0
34	511	Combination	5	4.5	4.5		4		3
34	512	PM	6	5	5		3		
34	513	Combination	7	6	3		2.5	0.5	0
34	514	SSG	7	7	3	2.5	2.5		0
34	515	SSG	6	2	2	2	0	4	
34	516	PM	6	5	6		5		4
34	517	PM	8	7	7		5		
34	518	SSG	11	12	14	11	8		4
34	519	PM	2	0	0		2	3	
34	520	SSG	5	5	3	2	2		0
34	521	PM	4	4	3		2		
34	522	Combination	7	7	5		3		
34	523	SSG	8	3.5	1	0	0		0
34	524	Combination	2	0	0		0		0
34	525	Combination	5	0	1		0		0
34	526	SSG	2	2	2	0	0		0
34	527	Combination	4	0	0		0		0
34	528	PM	9	10	11		8		
34	529	Combination	5	3	2		0		0
34	530	Combination	8	8	7		5		1
34	531	SSG	6	7	6	6	3.5		
34	532	Combination	4	6	4		3		0
34	533	SSG	7	7	5	5	4		2
34	534	PM	6	5	3		0		
34	535	PM	4	3	3		2	6	
34	536	PM	0	0	0		0		0
34	537	SSG	2	1	0	0	0		0
34	538	PM	8	9	8		8		
34	539	Combination	4	2	0		0		4
34	540	SSG	6	5	5	5	3		3

Centre Number	Patient Number	Treatment	Spleen size (cm) Baseline	Day7	Day 14	Day 21	EOT	3 mon FU	6 mon FU
35	646	PM	4	4	2		0		0
35	647	PM	6	6	4		4	0	0
35	648	Combination	8	6	1		1	0	0
35	649	Combination	12	10	7		7	0	0
35	650	SSG	6	4	2	2	0	0	0
35	651	SSG	5	4	3	3	3	0	0
35	652	PM	6	5	5		5		0
35	653	Combination	8	8	6		4	0	0
35	654	SSG	8	4	4	4	2	0	0
35	655	Combination	7	3	0		0	0	0
35	656	SSG	9	6	5	4	0	0	0
35	657	PM	12	12	12		8	2	0
35	658	Combination	7	5	2		2	0	0
35	659	SSG	10	10	10	7	6	0	0
35	660	PM	0	0	0		0	0	0
35	661	Combination	4	4	1		0	0	0
35	662	PM	8	8	2		0	0	0
35	663	SSG	13	13	8	8	6	2	2
35	664	Combination	6	6					0
35	665	SSG	9	6	6	2	0	0	0
35	666	SSG	7	4	2	0	0	0	0
35	667	Combination	2	2	0		0	0	0
35	668	PM	8	8	6		1		0
35	669	PM	9	9	9		9		
35	670	SSG	4	4	2	2	0	0	0
35	671	PM	13	13	9		9		0
35	672	Combination	12	12	10		7		2
35	673	SSG	10	8	4	4	4	0	0
35	674	Combination	9	9	6		6	0	0
35	675	PM	0	0	0		0		0
35	676	PM	8	7	7		7	2	0
35	677	SSG	14	12					
35	678	Combination	12	12	8		8	0	0
35	679	SSG	6	4	4	2	3	0	0
35	680	Combination	13	13	7		6	0	0
35	681	Combination	4	4	0		0	0	0
35	682	PM	4	4	3		0		0
35	683	Combination	9	9	5		4		0
35	684	SSG	8	8	8	4	4	0	0
35	685	PM	4	4	4		2	0	0
35	686	PM	10	8	4		4	0	0
35	687	SSG	18	14	8	8	8	0	0
35	688	PM	8	5	2		0	0	0
35	689	Combination	12	6	4		4	0	0
35	690	SSG	7	2	0	0	0	0	0

Centre Number	Patient Number	Treatment	Liver size (cm)						
			Baseline	Day7	Day 14	Day 21	EOT	3 mon FU	6 mon FU
11	1	PM	6	4	3		0	0	0
11	2	Combination	0	0	5		5	3	3
11	3	Combination	0	0	0		0	0	0
11	4	PM	3	3	0		0	0	0
11	5	PM	3	2	3		2	0	0
11	6	Combination	3.5	4	3		2	0	0
11	7	PM	10	8	5		0	0	0
11	8	SSG	3.5	0	0	0	0	0	0
11	9	SSG	5	0	0	0	0	0	0
11	10	SSG	4	4	0	0	0	0	0
11	11	Combination	6.5	5.5	4.5		5	3	4
11	12	Combination	0	0	0		0	0	0
11	13	SSG	3	2	0	0	0	0	0
11	14	SSG	6	3.5	3	3	2	3	2
11	15	PM	0	1.5				0	0
11	16	Combination	0	0	0		0	0	0
11	17	Combination	3	3	3		3	4.5	4
11	18	SSG	4	4	3	3	3	0	0
11	19	Combination	0	0	0		0	4	3
11	20	SSG	0	0	0	0	0	0	0
11	21	SSG	0	0	0	0	0	0	0
11	22	SSG	2	2	2	2	0	0	0
11	23	PM	7	6	7		6	0	0
11	24	PM	3	2	2		0	0	0
11	25	SSG	0	0	0	0	0	0	0
11	26	Combination	5	4	2		2	0	0
11	27	PM	6	4.5	5		5	4	
11	28	Combination	0	0	0		0	0	0
11	29	PM	0	0	0		0	0	0
11	30	PM	4	3	3		3	0	0
11	31	SSG	3	4	4	2	3	2	0
11	32	Combination	6	3.5	0		0	0	0
11	33	SSG	0	0	0	0	0	0	0
11	34	PM	5	5	4		3	4	3
11	35	SSG	0	0	3	4	3	0	0
11	36	SSG	0	0	4	4	3.5	2	0
11	37	Combination	4	4	0		0	0	0
11	38	PM	3	3	4		3	0	4
11	39	PM	6	6	4.5		3	1	0
11	40	PM	0	4	0			0	0
11	41	Combination	0	0	0		0	0	0
11	42	Combination	4	2	0		0	0	0
11	43	SSG	5	2	0	0	0	0	0
11	44	PM	0	0	0		0	0	0
11	45	Combination	0	0	0		0	0	0

Centre Number	Patient Number	Treatment	Liver size (cm) Baseline	Day7	Day 14	Day 21	EOT	3 mon FU	6 mon FU
11	46	SSG	6	3	3	3	2	1.5	0
11	47	SSG	0	0	0	0	0		
11	48	Combination	0	0	0		0		0
11	49	SSG	6	3	0	0	0	0	0
11	50	PM	3	3	3		0	0	0
11	51	Combination	4	5	2		2	0	0
11	52	PM	5	5	6		4	3	3
11	53	SSG	0	0	0	0	0	0	0
11	54	PM	4	3	2		0	0	4
11	55	PM	0	0	0		0	0	0
11	56	SSG	6	5	2	2	2	0	0
11	57	Combination	3	3	3		3	1.5	0
11	58	Combination	0	0	0		0	0	0
11	59	PM	4	3.5	3		2	3	3
11	60	Combination	2	0	0		0	0	0
11	61	PM	3	4	3		2	0	0
11	62	SSG	8	7	7	7	6	4	0
11	63	SSG	0	0	0	0	0	0	0
11	64	SSG	4.5	3	3	3	0	0	0
11	65	PM	8	6	6		3		
11	66	Combination	0	0	0		0	0	0
11	67	PM	3.5	3	2		0	0	0
11	68	SSG	3	4	2	0	0	0	0
11	69	Combination	4	4	3		3	2	2
11	70	Combination	4	4	2		2	0	0
11	71	Combination	5	3	3		4	3	5
11	72	SSG	4	3	3			0	0
11	73	PM	4	2	0		0	0	0
11	74	PM	0	0	0		0	0	0
11	75	Combination	6	4	3		1	2	0
11	76	PM	2	2	0		0	0	0
11	77	PM	4	4	2		0	0	0
11	78	SSG	4	2	0	0	0	0	0
11	79	PM	0	0	0		0	0	0
11	80	PM	5	7	5		2.5	3	0
11	81	Combination	0	0	0		0	0	0
11	82	SSG	3	3	3	3	4	3	0
11	83	SSG	2	3	2	2	1.5	0	0
11	84	SSG	0	0	0	3	3	0	0
11	85	SSG	6	7	7	9	6	4	
11	86	Combination	2	3	0		0	0	0
11	87	Combination	2	3	3		2	0	0
11	88	PM	0	0	0		0	0	0
11	89	Combination	5	5	5		5	5	0
11	90	Combination	0	0	0		0		

Centre Number	Patient Number	Treatment	Baseline	Day7	Day 14	Liver size (cm) Day 21	EOT	3 mon FU	6 mon FU
11	91	PM	2	3	2		2	0	0
11	92	Combination	3	3	3		2.5	0	0
11	93	SSG	2	2	0	0	0	0	0
11	94	SSG	4	5	3	3	2	0	0
11	95	SSG	3.5	3	3	3	3	2	0
11	96	Combination	6	3	3		3	0	0
11	97	Combination	3	3	3		3	0	0
11	98	PM	4	2.5	2		0	0	0
11	99	SSG	3	3	3	2.5	1.5	0	0
11	100	PM	4	3	3		3		
11	101	PM	4	4	3		3.5	2	0
11	102	Combination	4.5	3	0		0	0	0
11	103	Combination	4	3	2		0	0	0
11	104	PM	6	6	5		4	1	0
11	105	SSG	4	4	4	4	4	2	1
11	106	Combination	0	0	0		0	0	0
11	107	PM	4	3	0		0	0	0
11	108	PM	4	3	0		0	0	0
11	109	SSG	3	3	3	3	3	0	0
11	110	PM	4.5	4.5	4		4	2	2
11	111	Combination	2	3	0		0	0	0
11	112	Combination	4.5	4.5	2		0	0	0
11	113	SSG	7	6	4.5	3	3	3	
11	114	SSG	2	2	0	0	0	0	0
11	115	Combination	6	4	2		2	0	0
11	116	SSG	2.5	0	0	0	0	0	0
11	117	PM	4	2	0		0	0	
11	118	PM	3	1	0		0	0	0
11	119	SSG	4	3	2	2	2	0	0
11	120	Combination	4.5	2.5	0		0	0	0
11	121	Combination	2	2.5	2.5		2	0	0
11	122	SSG	4	1.5	1	0	0		
11	123	SSG	4	4	3	3	3	2	0
11	124	SSG	2	2	0	0	0		0
11	125	SSG	4	2.5	2	1.5	0	0	0
11	126	Combination	2	2	2		1.5		4
11	127	PM	3	3	2		2	1	0
11	128	Combination	2	2.5	1		0	0	2
11	129	PM	2	2	2		2	0	0
11	130	Combination	0	0	0		0	0	0
11	131	PM	3	3	2		2	0	0
11	132	SSG	4.5	2.5	2	1.5	0	0	0
11	133	Combination	2	2	0		0	0	0
11	134	PM	3.5	3.5	2		2	2	2
11	135	PM	3.5	2	2		0	0	0

Centre Number	Patient Number	Treatment	Liver size (cm) Baseline	Day7	Day 14	Day 21	EOT	3 mon FU	6 mon FU
12	241	Combination	2	2	0.5		0.5		0
12	242	SSG	4	3	3	0.5	0.5	0	0
12	243	PM	0.5	3	3		3	0	0
12	244	SSG	4	6	4	8	7	4.5	0
12	245	PM	4	7	7		6	8	
12	246	PM	3	0.5	0		0	0	
12	247	PM	3	2.5	5		5	0	0
12	248	PM	1	0.5	0.5		0	0	0
12	249	Combination	10	10	9		7	3	0
12	250	SSG	8	8	9	9	6	7	2
12	251	Combination	3	3	0		0	0	0
12	252	SSG	0	0	0	0	0	0	0
12	253	SSG	3	3	4	0	1	0	2
12	254	Combination	1	2	2		3	0	0
12	255	Combination	0	0	0		0	0	0
12	256	Combination	0	0	0		0	0	0
12	257	SSG	4	2	2	1	1	0	0
12	258	SSG	10	10	6	6	3		0
12	259	PM	2	2	0		0	0	0
12	260	PM	2	2	0		0		0
12	261	Combination	8	7	4		3	0	0
12	262	PM	5	5	4		0	0	0
12	263	SSG	2	2	0	0	0	0	0
12	264	Combination	0	0	3		5	2	0
12	265	SSG	0	2	2	3	6		
12	266	PM	2	1	3		6	2	0
12	267	SSG	0	0	0	0	0	0	0
12	268	PM	0	0	0		0	0	0
12	269	Combination	0	0	0		0	0	0
12	270	Combination	3	0	0		0		0
12	271	SSG			0	0	0	0	0
12	272	PM	0	0	0		0	0	0
12	273	SSG	3	2	2	2	3	0	0
12	274	SSG	0		0	0	0	0	0
12	275	Combination	5	3	0		0	0	0
12	276	PM	5	4	3		2	2	0
12	277	Combination	5	3	2		1	0	0
12	278	SSG	4	2	0	0	0	0	
12	279	PM	2	0	0		0	3	0
12	280	SSG	5	3	0	0	0	0	0
12	281	Combination	3	0	0		0	0	0
12	282	PM	2	2	2		2	0	0
12	283	PM	0	0	0		0	0	0
12	284	Combination	3	2	0		0	2	1
12	285	Combination	3	1	0		0	0	0

Centre	Patient					Liver size (cm)			
Number	Number	Treatment	Baseline	Day7	Day 14	Day 21	EOT	3 mon FU	6 mon FU
12	286	PM	4	2	2		1	0	0
12	287	SSG	7.5	6	4	2	0	0	0
12	288	Combination	0	0	0		0.5	0	0
12	289	Combination	0	2	1		1	0	2
12	290	SSG	0	0	0	0	0		0
12	291	SSG	0	0	0	0	0	0	0
12	292	PM	0	0	0		0		0
12	293	SSG	0.5	0	0	0	0		0
12	294	Combination	0	4	2		2	3	0
12	295	Combination	2	0	0		0		0
12	296	PM	3	1	0		0	0	0
12	297	SSG	5	0	2				
12	298	PM	0	0	0		0	2	0
12	299	Combination	0	0	0		0		0
12	300	PM	0	0	2		0	4	0
12	301	SSG	0	0	0	0	0	0	0
12	302	SSG	6	7	7	5	3	2	0
12	303	PM	3	2	3		3	0	0
12	304	Combination	8	7	1		0	0	0
12	305	PM	2	3	4		4	3	0
12	306	Combination	6	5	4		4	1	0
12	307	Combination	5	10	7		4	0	0
12	308	PM	8.5	4	2		0	0	0
12	309	PM	0	0	0		0	0	0
12	310	SSG	2	2	0	0	0	0	0
12	311	SSG	3	2	1	0	0	1	0
12	312	Combination	0	0	0		0	0	0
12	313	PM	0	0	0		0	0	0
12	314	SSG	0	0	0	0	0	0	0
12	315	Combination	2	6	2		0	0	0
12	316	SSG	0	0	0		0	0	0
12	317	SSG	8	8	6	5	4		0
12	318	Combination	0	0	0		0	0	0
12	319	Combination	0	0	0		0	0	0
12	320	Combination	4	4	2		0	0	0
12	321	Combination	4	2	1		0	0	0
12	322	PM	0	0	3		0	0	0
12	323	SSG	1	0	0	0	0	0	0
12	324	SSG	0	0	0	4	4	0	0
12	325	PM	4	2	0		1	0	0
12	326	PM	5	4	4		4	4	0
12	327	Combination	4	4	0		0	0	0
12	328	PM	0	0	0		0	0	0
12	329	SSG	3	3	2	0	0	0	0
12	330	PM	5	3	3		1	2	0

Centre Number	Patient Number	Treatment	Liver size (cm) Baseline	Day7	Day 14	Day 21	EOT	3 mon FU	6 mon FU
23	361	Combination	0	0	0		0	0	0
23	362	PM	0	0	0		0	0	0
23	363	PM	1	1				0	0
23	364	Combination	4	4	3.5		3	0	4
23	365	SSG	0	0	0	0	0	0	0
23	366	Combination	11	11.5	4		4	0	0
23	367	SSG	3	3	3	3	2	0	0
23	368	PM	0	0	0		0	0	0
23	369	Combination	2	2	3		3	0	0
23	370	SSG	0	0	0	0	0	0	0
23	371	PM	8	7	7		3	0	0
23	372	PM	3	1	0		0	0	0
23	373	SSG	0	0	0	0	0	0	1
23	374	SSG	6.5	6	3.5	1.5	0	0	0
23	375	Combination	4	4	4		3.5	0	0
23	376	SSG	2	2	1	1	0	0	0
23	377	Combination	7.5	5					0
23	378	Combination	2	2	2		2	3	0
23	379	PM	3	1.5	0		0	1.5	0
23	380	SSG	1	1	0	0	0	0	0
23	381	PM	4	4	2		0	2	0
23	382	Combination	3	3	3		0	0	0
23	383	PM	0	0	0		0	0	0
23	384	SSG	3.5	2	1.5	1	1	0	0
23	385	SSG	5	6	3.5	2	2	0	0
23	386	Combination	3	1.5	0		0	0	0
23	387	Combination	0	0	0		0	0	0
23	388	PM	0	0	0		0	0	0
23	389	SSG	10.5	8	5	4	4	1.5	0
23	390	PM	4.5	4	4		4	0	0
23	391	Combination	4	4	4		4	0	0
23	392	Combination	5	5	4			0	0
23	393	SSG	2.5	2.5	2.5	2.5	2.5	0	0
23	394	PM	5	5	5		2	0	0
23	395	PM	5	4	2		2	0	0
23	396	SSG	5	3	3	3	2	0	0
23	397	SSG	3	3	3	2	2	0	0
23	398	Combination	2	1	1		1	1	2
23	399	PM	5	5	5		5	0	0
23	400	PM	3	3	2.5		1	0	0
23	401	Combination	2	1	1		0	2	0
23	402	Combination	3	3	2		2	0	0
23	403	SSG	0	0	0	0	0	0	0
23	404	SSG	0	0	0	0	0		0
23	405	PM	0	0	0		0	0	0

Centre Number	Patient Number	Treatment	Liver size (cm) Baseline	Day7	Day 14	Day 21	EOT	3 mon FU	6 mon FU
34	451	Combination	5	5	4		3		0
34	452	PM	0.5	0	0		0		
34	453	PM	2	1	0		0		
34	454	Combination	4	4	3		3		0
34	455	PM	0	0	0		0		
34	456	SSG	4	5	5	3	2	4	
34	457	Combination	0	0	0		0		
34	458	PM	2	2	2		3	3	
34	459	Combination	0	0	0		0	0	0
34	460	PM	0	0	0		0		
34	461	SSG	1	4	9				
34	462	SSG	5	5	3.5	3.5	2		1
34	463	Combination	0	0	0		0		0
34	464	SSG	0	0	0	0	0		0
34	465	SSG	0	0	0	0	0		0
34	466	Combination	1	0	0		0		
34	467	PM	0	0	0		0	5	
34	468	Combination	0	0	0		0		0
34	469	SSG	2	0	0	0	0		0
34	470	Combination	6	5	5		2		4
34	471	PM	5	4	4		3		
34	472	SSG	4	2	0	0	0		0
34	473	PM	5	3	3		3		
34	474	Combination	0	0	0		0		0
34	475	PM	4	4	4		5		
34	476	PM	5	5	4.5		7	8	
34	477	SSG	0	0	0	0	0		0
34	478	Combination	4	3	2		0		2
34	479	SSG	3	3	2	3	3		0
34	480	SSG	0	0	0	0	0		0
34	481	Combination	5	3	3		1		0
34	482	Combination	8	5	0		0		0
34	483	PM	0	0	0		0		0
34	484	PM	0	2					
34	485	Combination	7	5	4		2		4
34	486	Combination	4	2	2		0		0
34	487	SSG	0	0	0	0	0		0
34	488	SSG	3	3	3	3	3	2	0
34	489	SSG	3	2	5.5	3.5	1		0
34	490	PM	8	5	5		5		0
34	491	PM	5	5	4		4		
34	492	PM	0	0	0		0		0
34	493	SSG	0	0	0	0	0	0	
34	494	Combination	0	0	0		0		0
34	495	SSG	4	2	4	2.5	1		1

Centre Number	Patient Number	Treatment	Liver size (cm) Baseline	Day7	Day 14	Day 21	EOT	3 mon FU	6 mon FU
34	496	SSG	0	0	0	0	0		0
34	497	PM	3	3	3		1	5	
34	498	SSG	0	0	0	0	0		0
34	499	Combination	5	4.5	4		4		0
34	500	PM	0	4	4		2.5		0
34	501	Combination	3	3	3		3		0
34	502	Combination	6	4	2		1.5		0
34	503	PM	2.5	4	3		3		
34	504	Combination	3	2	2		2		
34	505	SSG	2	2	2	1	0		0
34	506	SSG	5	4	3	2	1.5		0
34	507	SSG	2	5	5	2	3.5		4
34	508	PM	4	4	4		2		
34	509	PM	0	3	2		0		
34	510	Combination	3	3	3		2		0
34	511	Combination	0	0	0		0		0
34	512	PM	5	6	6		4		
34	513	Combination	3	3	2.5		2.5	2.5	2.5
34	514	SSG	4	4	4	4	4		2
34	515	SSG	2.5	1	0	2	2	0	
34	516	PM	0	0	0		0		0
34	517	PM	5	4	3.5		2		
34	518	SSG	5	5	6	6	5		5
34	519	PM	0	2	2		2	2	
34	520	SSG	0	0	0	0	0		0
34	521	PM	4	3	3		3		
34	522	Combination	5	5	4		9		
34	523	SSG	4	2.5	2.5	2	1		0
34	524	Combination	2	1	0		0		0
34	525	Combination	6	1.5	2		0		0
34	526	SSG	0	0	3	2	0		0
34	527	Combination	5	4	3		2		0
34	528	PM	3	6	5.5		5		
34	529	Combination	4	4	3		3		1
34	530	Combination	2	3	3		2		0
34	531	SSG	2.5	6	6	4	2		
34	532	Combination	3	5	3		2		3
34	533	SSG	3	3	2	2	1		0
34	534	PM	2	2	2		4		
34	535	PM	3	2	2		2	4	
34	536	PM	4	3	2		0		0
34	537	SSG	4	3	3	3	1		0
34	538	PM	5	5	4		3		
34	539	Combination	3	1	0		0		0
34	540	SSG	4	4	2	2	0		0

Centre Number	Patient Number	Treatment	Liver size (cm) Baseline	Day7	Day 14	Day 21	EOT	3 mon FU	6 mon FU
35	646	PM	4	4	4		4		0
35	647	PM	4	4	4		4	0	0
35	648	Combination	4	4	2		2	0	0
35	649	Combination	6	4	3		3	0	0
35	650	SSG	4	4	4	2	2	0	0
35	651	SSG	3	3	2	2	2	0	0
35	652	PM	4	4	3		3		0
35	653	Combination	5	5	4		4	0	0
35	654	SSG	5	4	4	4	4	0	0
35	655	Combination	2	2	2		2	0	0
35	656	SSG	4	4	4	2	2	0	0
35	657	PM	5	5	5		3	0	0
35	658	Combination	4	4	4		2	0	0
35	659	SSG	5	4	4	2	2	0	0
35	660	PM	0	0	0		0	0	0
35	661	Combination	2	2	2		2	0	0
35	662	PM	4	4	2		0	0	0
35	663	SSG	4	4	2	2	2	0	0
35	664	Combination	2	2					0
35	665	SSG	2	2	2	2	0	0	0
35	666	SSG	2	2	2	0	0	0	0
35	667	Combination	2	2	2		2	0	0
35	668	PM	2	2	2		0		0
35	669	PM	1	1	1		1		
35	670	SSG	0	0	0	0	0	0	0
35	671	PM	4	4	3		2		0
35	672	Combination	6	6	6		6		0
35	673	SSG	3	2	2	2	0	0	0
35	674	Combination	3	3	3		3	0	0
35	675	PM	2	2	2		0		0
35	676	PM	6	7	6		6	2	0
35	677	SSG	4	4					
35	678	Combination	4	4	3		3	0	0
35	679	SSG	2	2	0	0	0	0	0
35	680	Combination	5	5	5		4	0	0
35	681	Combination	0	0	0		0	0	0
35	682	PM	5	4	4		0		0
35	683	Combination	4	3	0		0		0
35	684	SSG	0	0	0	0	0	0	0
35	685	PM	0	0	0		0	0	0
35	686	PM	7	6	2		0	0	0
35	687	SSG	7	6	6	6	6	0	0
35	688	PM	4	2	0		0	0	0
35	689	Combination	8	2	2		2	0	0
35	690	SSG	5	5	4	4	4	0	0

Centre	Patient				Cervical lymphadenopathy				
Number	Number	Treatment	Baseline	Day7	Day 14	Day 21	EOT	3 mon FU	6 mon FU
11	1	PM	Absent	Absent	Absent		Absent	Absent	Absent
11	2	Combination	Absent	Absent	Absent		Absent	Absent	Absent
11	3	Combination	Absent	Absent	Absent		Absent	Absent	Absent
11	4	PM	Absent	Absent	Absent		Absent	Absent	Absent
11	5	PM	Absent	Absent	Absent		Absent	Absent	Absent
11	6	Combination	Absent	Absent	Absent		Absent	Absent	Absent
11	7	PM	Absent	Absent	Absent		Absent	Absent	Absent
11	8	SSG	Absent	Absent	Absent	Absent	Absent	Absent	Absent
11	9	SSG	Absent	Absent	Absent	Absent	Absent	Absent	Absent
11	10	SSG	Absent	Absent	Absent	Absent	Absent	Absent	Absent
11	11	Combination	Absent	Absent	Absent		Absent	Absent	Absent
11	12	Combination	Absent	Absent	Absent		Absent	Absent	Absent
11	13	SSG	Absent	Absent	Absent	Absent	Absent	Absent	Absent
11	14	SSG	Absent	Absent	Absent	Absent	Absent	Absent	Absent
11	15	PM	Absent	Absent				Absent	Absent
11	16	Combination	Absent	Absent	Absent		Absent	Absent	Absent
11	17	Combination	Absent	Absent	Absent		Absent	Absent	Absent
11	18	SSG	Absent	Absent	Absent	Absent	Absent	Absent	Absent
11	19	Combination	Absent	Absent	Absent		Absent	Absent	Absent
11	20	SSG	Absent	Absent	Absent	Absent	Absent	Absent	Absent
11	21	SSG	Absent	Absent	Absent	Absent	Absent	Absent	Absent
11	22	SSG	Absent	Absent	Absent	Absent	Absent	Absent	Absent
11	23	PM	Absent	Absent	Absent		Absent	Absent	Absent
11	24	PM	Absent	Absent	Absent		Absent	Absent	Absent
11	25	SSG	Absent	Absent	Absent	Absent	Absent	Absent	Absent
11	26	Combination	Absent	Absent	Absent		Absent	Absent	Absent
11	27	PM	Absent	Absent	Absent		Absent	Absent	
11	28	Combination	Absent	Absent	Absent		Absent	Absent	Absent
11	29	PM	Absent	Absent	Absent		Absent	Absent	Absent
11	30	PM	Absent	Absent	Absent		Absent	Absent	Absent
11	31	SSG	Absent	Absent	Absent	Absent	Absent	Absent	Absent
11	32	Combination	Absent	Absent	Absent		Absent	Absent	Absent
11	33	SSG	Absent	Absent	Absent	Absent	Absent	Absent	Absent
11	34	PM	Absent	Absent	Absent		Absent	Absent	Absent
11	35	SSG	Absent	Absent	Absent	Absent	Absent	Absent	Absent
11	36	SSG	Absent	Absent	Absent	Absent	Absent	Absent	Absent
11	37	Combination	Absent	Absent	Absent		Absent	Absent	Absent
11	38	PM	Absent	Absent	Absent		Absent	Absent	Absent
11	39	PM	Absent	Absent	Absent		Absent	Absent	Absent
11	40	PM	Absent	Absent	Absent			Absent	Absent
11	41	Combination	Absent	Absent	Absent		Absent	Absent	Absent
11	42	Combination	Absent	Absent	Absent		Absent	Absent	Absent
11	43	SSG	Absent	Absent	Absent	Absent	Absent	Absent	Absent
11	44	PM	Absent	Absent	Absent		Absent	Absent	Absent
11	45	Combination	Absent	Absent	Absent		Absent	Absent	Absent
11	46	SSG	Absent	Absent	Absent	Absent	Absent	Absent	Absent
11	47	SSG	Absent	Absent	Absent	Absent	Absent		
11	48	Combination	Absent	Absent	Absent		Absent		Absent
11	49	SSG	Absent	Absent	Absent	Absent	Absent	Absent	Absent
11	50	PM	Absent	Absent	Absent		Absent	Absent	Absent
11	51	Combination	Absent	Absent	Absent		Absent	Absent	Absent
11	52	PM	Present	Absent	Absent		Absent	Absent	Absent
11	53	SSG	Absent	Absent	Absent	Absent	Absent	Absent	Absent
11	54	PM	Absent	Absent	Absent		Absent	Absent	Absent

Centre Number	Patient Number	Treatment	Cervical lymphadenopathy Baseline	Day7	Day 14	Day 21	EOT	3 mon FU	6 mon FU
11	55	PM	Absent	Absent	Absent		Absent	Absent	Absent
11	56	SSG	Absent	Absent	Absent	Absent	Absent	Absent	Absent
11	57	Combination	Absent	Absent	Absent		Absent	Absent	Absent
11	58	Combination	Absent	Absent	Absent		Absent	Absent	Absent
11	59	PM	Absent	Absent	Absent		Absent	Absent	Absent
11	60	Combination	Absent	Absent	Absent		Absent	Absent	Absent
11	61	PM	Absent	Absent	Absent		Absent	Absent	Absent
11	62	SSG	Absent	Absent	Absent	Absent	Absent	Absent	Absent
11	63	SSG	Absent	Absent	Absent	Absent	Absent	Absent	Absent
11	64	SSG	Absent	Absent	Absent	Absent	Absent	Absent	Absent
11	65	PM	Absent	Absent	Absent		Absent		
11	66	Combination	Absent	Absent	Absent		Absent	Absent	Absent
11	67	PM	Absent	Absent	Absent		Absent	Absent	Absent
11	68	SSG	Present	Absent	Absent	Absent	Absent	Absent	Absent
11	69	Combination	Present	Absent	Absent		Absent	Absent	Absent
11	70	Combination	Absent	Absent	Absent		Absent	Absent	Absent
11	71	Combination	Absent	Absent	Absent		Absent	Absent	Absent
11	72	SSG	Absent	Absent	Absent			Absent	Absent
11	73	PM	Absent	Absent	Absent		Absent	Absent	Absent
11	74	PM	Absent	Absent	Absent		Absent	Absent	Absent
11	75	Combination	Absent	Absent	Absent		Absent	Absent	Absent
11	76	PM	Absent	Absent	Absent		Absent	Absent	Absent
11	77	PM	Absent	Absent	Absent		Absent	Absent	Absent
11	78	SSG	Absent	Absent	Absent	Absent	Absent	Absent	Absent
11	79	PM	Absent	Absent	Absent		Absent	Absent	Absent
11	80	PM	Absent	Absent	Absent		Absent	Absent	Absent
11	81	Combination	Absent	Absent	Absent		Absent	Absent	Absent
11	82	SSG	Absent	Absent	Absent	Absent	Absent	Absent	Absent
11	83	SSG	Absent	Absent	Absent	Absent	Absent	Absent	Absent
11	84	SSG	Absent	Absent	Absent	Absent	Absent	Absent	Absent
11	85	SSG	Absent	Absent	Absent	Absent	Absent	Absent	
11	86	Combination	Absent	Absent	Absent		Absent	Absent	Absent
11	87	Combination	Absent	Absent	Absent		Absent	Absent	Absent
11	88	PM	Absent	Absent	Absent		Absent	Absent	Absent
11	89	Combination	Absent	Absent	Absent		Absent	Absent	Absent
11	90	Combination	Absent	Absent	Absent		Absent		
11	91	PM	Absent	Absent	Absent		Absent	Absent	Absent
11	92	Combination	Absent	Absent	Absent		Absent	Absent	Absent
11	93	SSG	Absent	Absent	Absent	Absent	Absent	Absent	Absent
11	94	SSG	Absent	Absent	Absent	Absent	Absent	Absent	Absent
11	95	SSG	Absent	Absent	Absent	Absent	Absent	Absent	Absent
11	96	Combination	Absent	Absent	Absent		Absent	Absent	Absent
11	97	Combination	Absent	Absent	Absent		Absent	Absent	Absent
11	98	PM	Absent	Absent	Absent		Absent	Absent	Absent
11	99	SSG	Absent	Absent	Absent	Absent	Absent	Absent	Absent
11	100	PM	Absent	Absent	Absent		Absent		
11	101	PM	Absent	Absent	Absent		Absent	Absent	Absent
11	102	Combination	Absent	Absent	Absent		Absent	Absent	Absent
11	103	Combination	Absent	Absent	Absent		Absent	Absent	Absent
11	104	PM	Absent	Absent	Absent		Absent	Absent	Absent
11	105	SSG	Absent	Absent	Absent	Absent	Absent	Absent	Absent
11	106	Combination	Absent	Absent	Absent		Absent	Absent	Absent
11	107	PM	Absent	Absent	Absent		Absent	Absent	Absent
11	108	PM	Absent	Absent	Absent		Absent	Absent	Absent

Centre	Patient		Cervical lymphadenopathy						
Number	Number	Treatment	Baseline	Day7	Day 14	Day 21	EOT	3 mon FU	6 mon FU
11	109	SSG	Absent	Absent	Absent	Absent	Absent	Absent	Absent
11	110	PM	Absent	Absent	Absent		Absent	Absent	Absent
11	111	Combination	Absent	Absent	Absent		Absent	Absent	Absent
11	112	Combination	Absent	Absent	Absent		Absent	Absent	Absent
11	113	SSG	Absent	Absent	Absent	Absent	Absent	Absent	
11	114	SSG	Absent	Absent	Absent	Absent	Absent	Absent	Absent
11	115	Combination	Absent	Absent	Absent		Absent	Absent	Absent
11	116	SSG	Absent	Absent	Absent	Absent	Absent	Absent	Absent
11	117	PM	Absent	Absent	Absent		Absent	Absent	
11	118	PM	Absent	Absent	Absent		Absent	Absent	Absent
11	119	SSG	Absent	Absent	Absent	Absent	Absent	Absent	Absent
11	120	Combination	Absent	Absent	Absent		Absent	Absent	Absent
11	121	Combination	Absent	Absent	Absent		Absent	Absent	Absent
11	122	SSG	Absent	Absent	Absent	Absent	Absent		
11	123	SSG	Absent	Absent	Absent	Absent	Absent	Absent	Absent
11	124	SSG	Absent	Absent	Absent	Absent	Absent		Absent
11	125	SSG	Absent	Absent	Absent	Absent	Absent	Absent	Absent
11	126	Combination	Absent	Absent	Absent		Absent		Absent
11	127	PM	Absent	Absent	Absent		Absent	Absent	Absent
11	128	Combination	Absent	Absent	Absent		Absent	Absent	Absent
11	129	PM	Absent	Absent	Absent		Absent	Absent	Absent
11	130	Combination	Absent	Absent	Absent		Absent	Absent	Absent
11	131	PM	Absent	Absent	Absent		Absent	Absent	Absent
11	132	SSG	Absent	Absent	Absent	Absent	Absent	Absent	Absent
11	133	Combination	Absent	Absent	Absent		Absent	Absent	Absent
11	134	PM	Absent	Absent	Absent		Absent	Absent	Absent
11	135	PM	Absent	Absent	Absent		Absent	Absent	Absent
12	241	Combination	Absent	Absent	Absent		Absent		Absent
12	242	SSG	Absent	Absent	Absent	Absent	Absent	Absent	Absent
12	243	PM	Absent	Absent	Absent		Absent	Absent	Absent
12	244	SSG	Absent	Absent	Absent	Absent	Absent	Absent	Absent
12	245	PM	Absent	Absent	Absent		Absent	Absent	
12	246	PM	Absent	Absent	Absent		Absent	Absent	
12	247	PM	Present	Present	Present		Present	Absent	Absent
12	248	PM	Absent	Absent	Absent		Absent	Absent	Absent
12	249	Combination	Present	Absent	Absent		Absent	Absent	Absent
12	250	SSG	Absent	Present	Present	Absent	Absent	Absent	Absent
12	251	Combination	Present	Absent	Absent		Absent	Absent	Absent
12	252	SSG	Present	Absent	Absent	Absent	Absent	Absent	Absent
12	253	SSG	Present	Absent	Absent	Absent	Absent	Absent	Absent
12	254	Combination	Absent	Absent	Absent		Absent	Absent	Absent
12	255	Combination	Absent	Absent	Absent		Absent	Absent	Absent
12	256	Combination	Absent	Absent	Absent		Absent	Absent	Absent
12	257	SSG	Absent	Absent	Absent	Absent	Absent	Absent	Absent
12	258	SSG	Absent	Absent	Absent	Absent	Absent		Absent
12	259	PM	Absent	Absent	Absent		Absent	Absent	Absent
12	260	PM	Absent	Absent	Absent		Absent		Absent
12	261	Combination	Present	Absent	Absent		Absent	Absent	Absent
12	262	PM	Absent	Absent	Absent		Absent	Absent	Absent
12	263	SSG	Absent	Absent	Absent	Absent	Absent	Absent	Absent
12	264	Combination	Absent	Absent	Absent		Absent	Absent	Absent
12	265	SSG	Absent	Absent	Absent	Absent	Absent		
12	266	PM	Absent	Absent	Absent		Absent	Absent	Absent
12	267	SSG	Absent	Absent	Absent	Absent	Absent	Absent	Absent

Centre Number	Patient Number	Treatment	Cervical lymphadenopathy Baseline	Day7	Day 14	Day 21	EOT	3 mon FU	6 mon FU
12	268	PM	Absent	Absent	Absent		Absent	Absent	Absent
12	269	Combination	Absent	Absent	Absent		Absent	Absent	Absent
12	270	Combination	Absent	Absent	Absent		Absent		Absent
12	271	SSG	Absent	Absent	Absent	Absent	Absent	Absent	Absent
12	272	PM	Absent	Absent	Absent		Absent	Present	Absent
12	273	SSG	Absent	Absent	Absent	Absent	Absent	Absent	Absent
12	274	SSG	Present	Absent	Absent	Absent	Absent	Absent	Absent
12	275	Combination	Absent	Absent	Absent		Absent	Absent	Absent
12	276	PM	Absent	Absent	Absent		Absent	Absent	Absent
12	277	Combination	Absent	Absent	Absent		Absent	Absent	Absent
12	278	SSG	Absent	Absent	Absent	Absent	Absent	Absent	
12	279	PM	Absent	Absent	Absent		Absent	Absent	Absent
12	280	SSG	Absent	Absent	Absent	Absent	Absent	Absent	Absent
12	281	Combination	Absent	Absent	Absent		Absent	Absent	Absent
12	282	PM	Absent	Absent	Absent		Absent	Absent	Absent
12	283	PM	Absent	Absent	Absent		Absent	Absent	Absent
12	284	Combination	Absent	Absent	Absent		Absent	Absent	Absent
12	285	Combination	Absent	Absent	Absent		Absent	Absent	Absent
12	286	PM	Absent	Absent	Absent		Absent	Absent	Absent
12	287	SSG	Absent	Absent	Absent	Absent	Absent	Absent	Absent
12	288	Combination	Absent	Absent	Absent		Absent	Absent	Absent
12	289	Combination	Absent	Absent	Absent		Absent	Absent	Absent
12	290	SSG	Absent	Absent	Absent	Absent	Absent		Absent
12	291	SSG	Absent	Absent	Absent	Absent	Absent	Absent	Absent
12	292	PM	Absent	Absent	Absent		Absent		Absent
12	293	SSG	Absent	Absent	Absent	Absent	Absent		Absent
12	294	Combination	Absent	Absent	Absent		Absent	Absent	Absent
12	295	Combination	Absent	Absent	Absent		Absent		Absent
12	296	PM	Absent	Absent	Absent		Absent	Absent	Absent
12	297	SSG	Present	Absent	Absent				
12	298	PM	Absent	Absent	Absent		Absent	Absent	Absent
12	299	Combination	Absent	Absent	Absent		Absent		Absent
12	300	PM	Absent	Absent	Absent		Absent	Absent	Absent
12	301	SSG	Absent	Absent	Absent	Absent	Absent	Absent	Absent
12	302	SSG	Absent	Absent	Absent	Absent	Absent	Absent	Absent
12	303	PM	Absent	Absent	Absent		Absent	Absent	Absent
12	304	Combination	Absent	Absent	Absent		Absent	Absent	Absent
12	305	PM	Present	Present	Absent		Absent	Absent	Absent
12	306	Combination	Absent	Absent	Absent		Absent	Absent	Absent
12	307	Combination	Absent	Absent	Absent		Absent	Absent	Absent
12	308	PM	Absent	Absent	Absent		Absent	Absent	Absent
12	309	PM	Absent	Absent	Absent		Absent	Absent	Absent
12	310	SSG	Absent	Absent	Absent	Absent	Absent	Absent	Absent
12	311	SSG	Absent	Absent	Absent	Absent	Absent	Absent	Absent
12	312	Combination	Absent	Absent	Absent		Absent	Absent	Absent
12	313	PM	Absent	Absent	Absent		Absent	Absent	Absent
12	314	SSG	Absent	Absent	Absent	Absent	Absent	Absent	Absent
12	315	Combination	Absent	Absent	Absent		Absent	Absent	Absent
12	316	SSG	Absent	Absent	Absent		Absent	Absent	Absent
12	317	SSG	Absent	Absent	Absent	Absent	Absent		Absent
12	318	Combination	Absent	Absent	Absent		Absent	Absent	Absent
12	319	Combination	Absent	Absent	Absent		Absent	Absent	Absent
12	320	Combination	Absent	Absent	Absent		Absent	Absent	Absent
12	321	Combination	Absent	Absent	Absent		Absent	Absent	Absent

Centre	Patient				Cervical lymphadenopathy				
Number	Number	Treatment	Baseline	Day7	Day 14	Day 21	EOT	3 mon FU	6 mon FU
12	322	PM	Absent	Absent	Absent		Absent	Absent	Absent
12	323	SSG	Absent	Absent	Absent	Absent	Absent	Absent	Absent
12	324	SSG	Absent	Absent	Absent	Absent	Absent	Absent	Absent
12	325	PM	Absent	Absent	Absent		Absent	Absent	Absent
12	326	PM	Absent	Absent	Absent		Absent	Absent	Absent
12	327	Combination	Absent	Absent	Absent		Absent	Absent	Absent
12	328	PM	Absent	Absent	Absent		Absent	Absent	Absent
12	329	SSG	Absent	Absent	Absent	Absent	Absent	Absent	Absent
12	330	PM	Absent	Absent	Absent		Absent	Absent	Absent
23	361	Combination	Absent	Absent	Absent		Absent	Absent	Absent
23	362	PM	Absent	Absent	Absent		Absent	Absent	Absent
23	363	PM	Absent	Absent	Absent		Absent	Absent	Absent
23	364	Combination	Absent	Absent	Absent		Absent	Absent	Absent
23	365	SSG	Absent	Absent	Absent	Absent	Absent	Absent	Absent
23	366	Combination	Absent	Absent	Absent		Absent	Absent	Absent
23	367	SSG	Absent	Absent	Absent	Absent	Absent	Absent	Absent
23	368	PM	Absent	Absent	Absent		Absent	Absent	Absent
23	369	Combination	Absent	Absent	Absent		Absent	Absent	Absent
23	370	SSG	Absent	Absent	Absent	Absent	Absent	Absent	Absent
23	371	PM	Absent	Absent	Absent		Absent	Present	Absent
23	372	PM	Absent	Absent	Absent		Absent	Absent	Absent
23	373	SSG	Absent	Absent	Absent	Absent	Absent	Absent	Absent
23	374	SSG	Absent	Absent	Absent	Absent	Absent	Absent	Absent
23	375	Combination	Absent	Absent	Absent		Absent	Absent	Absent
23	376	SSG	Absent	Absent	Absent	Absent	Absent	Absent	Absent
23	377	Combination	Absent	Absent					Absent
23	378	Combination	Present	Present	Present		Present	Absent	Absent
23	379	PM	Absent	Absent	Absent		Absent	Absent	Absent
23	380	SSG	Absent	Absent	Absent	Absent	Absent	Absent	Absent
23	381	PM	Absent	Absent	Absent		Absent	Absent	Absent
23	382	Combination	Absent	Absent	Absent		Absent	Absent	Absent
23	383	PM	Absent	Absent	Absent		Absent	Absent	Absent
23	384	SSG	Absent	Absent	Absent	Absent	Absent	Absent	Absent
23	385	SSG	Present	Absent	Absent	Present	Present	Present	Absent
23	386	Combination	Absent	Absent	Absent		Absent	Absent	Absent
23	387	Combination	Absent	Absent	Absent		Absent	Absent	Absent
23	388	PM	Absent	Absent	Absent		Absent	Absent	Absent
23	389	SSG	Present	Present	Present	Absent	Absent	Absent	Absent
23	390	PM	Absent	Absent	Absent		Absent	Absent	Absent
23	391	Combination	Absent	Absent	Absent		Absent	Present	Absent
23	392	Combination	Present	Absent	Present			Absent	Absent
23	393	SSG	Absent	Absent	Absent	Absent	Absent	Absent	Absent
23	394	PM	Absent	Absent	Absent		Absent	Absent	Absent
23	395	PM	Absent	Absent	Absent		Absent	Absent	Absent
23	396	SSG	Absent	Absent	Absent	Absent	Absent	Absent	Absent
23	397	SSG	Absent	Absent	Absent	Absent	Absent	Absent	Absent
23	398	Combination	Absent	Absent	Absent		Absent	Absent	Absent
23	399	PM	Absent	Absent	Absent		Absent	Absent	Absent
23	400	PM	Absent	Absent	Absent		Absent	Absent	Absent
23	401	Combination	Absent	Absent	Absent		Absent	Absent	Absent
23	402	Combination	Absent	Absent	Absent		Absent	Absent	Absent
23	403	SSG	Absent	Absent	Absent	Absent	Absent	Absent	Absent
23	404	SSG	Absent	Absent	Absent	Absent	Absent		Absent
23	405	PM	Absent	Absent	Absent		Absent	Absent	Absent

Centre	Patient		Cervical lymphadenopathy						
Number	Number	Treatment	Baseline	Day7	Day 14	Day 21	EOT	3 mon FU	6 mon FU
34	451	Combination	Absent	Absent	Absent		Absent		Absent
34	452	PM	Absent	Absent	Absent		Absent		
34	453	PM	Absent	Absent	Absent		Absent		
34	454	Combination	Absent	Absent	Absent		Absent		Absent
34	455	PM	Absent	Absent	Absent		Absent		
34	456	SSG	Absent	Absent	Absent	Absent	Absent	Absent	
34	457	Combination	Absent	Absent	Absent		Absent		
34	458	PM	Absent	Absent	Absent		Absent	Absent	
34	459	Combination	Absent	Absent	Absent		Absent	Absent	Absent
34	460	PM	Absent	Absent	Absent		Absent		
34	461	SSG	Absent	Absent	Absent				
34	462	SSG	Absent	Absent	Absent	Absent	Absent		Present
34	463	Combination	Present	Present	Present		Absent		Absent
34	464	SSG	Absent	Absent	Absent	Absent	Absent		Absent
34	465	SSG	Absent	Absent	Absent	Absent	Absent		Absent
34	466	Combination	Absent	Absent	Absent		Absent		
34	467	PM	Absent	Absent	Absent		Absent	Absent	
34	468	Combination	Absent	Absent	Absent		Absent		Absent
34	469	SSG	Absent	Absent	Absent	Absent	Absent		Absent
34	470	Combination	Absent	Absent	Absent		Absent		Absent
34	471	PM	Absent	Absent	Absent		Absent		
34	472	SSG	Absent	Absent	Absent	Absent	Absent		Absent
34	473	PM	Absent	Absent	Absent		Absent		
34	474	Combination	Absent	Absent	Absent		Absent		Absent
34	475	PM	Absent	Absent	Absent		Absent		
34	476	PM	Absent	Absent	Absent		Absent	Present	
34	477	SSG	Absent	Absent	Absent	Absent	Absent		Absent
34	478	Combination	Absent	Absent	Absent		Absent		Absent
34	479	SSG	Absent	Absent	Absent	Absent	Absent		Absent
34	480	SSG	Absent	Absent	Absent	Absent	Absent		Absent
34	481	Combination	Absent	Absent	Absent		Absent		Present
34	482	Combination	Absent	Absent	Absent		Absent		Absent
34	483	PM	Absent	Absent	Absent		Absent		Absent
34	484	PM	Absent	Absent					
34	485	Combination	Absent	Absent	Absent		Absent		Absent
34	486	Combination	Absent	Absent	Absent		Absent		Absent
34	487	SSG	Absent	Absent	Absent	Absent	Absent		Absent
34	488	SSG	Absent	Absent	Absent	Absent	Absent	Absent	Present
34	489	SSG	Absent	Absent	Absent	Absent	Absent		Absent
34	490	PM	Absent	Absent	Absent		Absent		Absent
34	491	PM	Absent	Absent	Absent		Absent		
34	492	PM	Absent	Absent	Absent		Absent		Absent
34	493	SSG	Absent	Absent	Absent	Absent	Absent	Absent	
34	494	Combination	Absent	Absent	Absent		Absent		Absent
34	495	SSG	Absent	Absent	Absent	Absent	Absent		Present
34	496	SSG	Absent	Absent	Absent	Absent	Absent		Absent
34	497	PM	Absent	Absent	Absent		Absent	Absent	
34	498	SSG	Absent	Absent	Absent	Absent	Absent		Present
34	499	Combination	Absent	Absent	Absent		Absent		Absent
34	500	PM	Absent	Absent	Absent		Absent		Absent
34	501	Combination	Absent	Absent	Absent		Absent		Absent
34	502	Combination	Absent	Absent	Absent		Absent		Absent
34	503	PM	Absent	Absent	Absent		Absent		
34	504	Combination	Absent	Absent	Absent		Absent		

Centre	Patient				Cervical lymphadenopathy				
Number	Number	Treatment	Baseline	Day7	Day 14	Day 21	EOT	3 mon FU	6 mon FU
34	505	SSG	Absent	Absent	Absent	Absent	Absent		Absent
34	506	SSG	Absent	Absent	Absent	Absent	Absent		Present
34	507	SSG	Absent	Absent	Present	Absent	Absent		Absent
34	508	PM	Absent	Absent	Absent		Absent		
34	509	PM	Absent	Absent	Absent		Absent		
34	510	Combination	Absent	Absent	Absent		Absent		Absent
34	511	Combination	Absent	Absent	Absent		Absent		Present
34	512	PM	Absent	Absent	Absent		Absent		
34	513	Combination	Absent	Absent	Absent		Absent	Absent	Absent
34	514	SSG	Absent	Absent	Absent	Absent	Absent		Absent
34	515	SSG	Absent	Absent	Absent	Absent	Absent	Absent	
34	516	PM	Absent	Absent	Absent		Absent		Absent
34	517	PM	Absent	Absent	Absent		Absent		
34	518	SSG	Absent	Absent	Absent	Absent	Absent		Absent
34	519	PM	Absent	Absent	Absent		Absent	Absent	
34	520	SSG	Absent	Absent	Absent	Absent	Absent		Absent
34	521	PM	Absent	Absent	Absent		Absent		
34	522	Combination	Absent	Absent	Absent		Absent		
34	523	SSG	Absent	Absent	Absent	Absent	Absent		Absent
34	524	Combination	Absent	Absent	Absent		Absent		Absent
34	525	Combination	Absent	Absent	Absent		Absent		Present
34	526	SSG	Absent	Absent	Absent	Absent	Absent		Present
34	527	Combination	Absent	Absent	Absent		Absent		Absent
34	528	PM	Absent	Absent	Absent		Absent		
34	529	Combination	Absent	Absent	Absent		Absent		Absent
34	530	Combination	Absent	Absent	Absent		Absent		Absent
34	531	SSG	Absent	Absent	Absent	Absent	Absent		
34	532	Combination	Absent	Absent	Absent		Absent		Absent
34	533	SSG	Absent	Absent	Absent	Absent	Absent		Absent
34	534	PM	Absent	Absent	Absent		Absent		
34	535	PM	Absent	Absent	Absent		Absent	Absent	
34	536	PM	Absent	Absent	Absent		Absent		Absent
34	537	SSG	Absent	Absent	Absent	Absent	Absent		Absent
34	538	PM	Absent	Absent	Absent		Absent		
34	539	Combination	Absent	Absent	Absent		Absent		Absent
34	540	SSG	Absent	Absent	Absent	Absent	Absent		Absent
35	646	PM	Absent	Absent	Absent		Absent		Absent
35	647	PM	Absent	Absent	Absent		Absent	Absent	Absent
35	648	Combination	Present	Present	Absent		Absent	Absent	Absent
35	649	Combination	Absent	Absent	Absent		Absent	Absent	Absent
35	650	SSG	Absent	Absent	Absent	Absent	Absent	Absent	Absent
35	651	SSG	Absent	Absent	Absent	Absent	Absent	Absent	Absent
35	652	PM	Present	Present	Present		Present		Absent
35	653	Combination	Present	Present	Present		Present	Absent	Absent
35	654	SSG	Absent	Absent	Absent	Absent	Absent	Absent	Absent
35	655	Combination	Present	Present	Present		Present	Absent	Absent
35	656	SSG	Present	Absent	Absent	Absent	Absent	Absent	Absent
35	657	PM	Present	Present	Present		Present	Absent	Absent
35	658	Combination	Absent	Absent	Absent		Absent	Absent	Absent
35	659	SSG	Present	Present	Present	Present	Present	Absent	Absent
35	660	PM	Absent	Absent	Absent		Absent	Absent	Absent
35	661	Combination	Absent	Absent	Absent		Absent	Absent	Absent
35	662	PM	Absent	Absent	Absent		Absent	Absent	Absent
35	663	SSG	Absent	Absent	Absent	Absent	Absent	Absent	Absent

Centre Number	Patient Number	Treatment	Cervical lymphadenopathy Baseline	Day7	Day 14	Day 21	EOT	3 mon FU	6 mon FU
35	664	Combination	Absent	Absent					Absent
35	665	SSG	Present	Present	Present	Present	Absent	Absent	Absent
35	666	SSG	Absent	Absent	Absent	Absent	Absent	Absent	Absent
35	667	Combination	Absent	Absent	Absent		Absent	Absent	Absent
35	668	PM	Absent	Absent	Absent		Absent		Absent
35	669	PM	Absent	Absent	Absent		Absent		
35	670	SSG	Absent	Absent	Absent	Absent	Absent	Absent	Absent
35	671	PM	Absent	Absent	Absent		Absent		Absent
35	672	Combination	Absent	Absent	Absent		Absent		Absent
35	673	SSG	Absent	Absent	Absent	Absent	Absent	Absent	Absent
35	674	Combination	Absent	Absent	Absent		Absent	Absent	Absent
35	675	PM	Absent	Absent	Absent		Absent		Absent
35	676	PM	Absent	Absent	Absent		Absent	Absent	Absent
35	677	SSG	Absent	Absent					
35	678	Combination	Absent	Absent	Absent		Absent	Absent	Absent
35	679	SSG	Absent	Absent	Absent	Absent	Absent	Absent	Absent
35	680	Combination	Absent	Absent	Absent		Absent	Absent	Absent
35	681	Combination	Absent	Absent	Absent		Absent	Absent	Absent
35	682	PM	Absent	Absent	Absent		Absent		Absent
35	683	Combination	Absent	Absent	Absent		Absent		Absent
35	684	SSG	Absent	Absent	Absent	Absent	Absent	Absent	Absent
35	685	PM	Absent	Absent	Absent		Absent	Absent	Absent
35	686	PM	Absent	Absent	Absent		Absent	Absent	Absent
35	687	SSG	Absent	Absent	Absent	Absent	Absent	Absent	Absent
35	688	PM	Absent	Absent	Absent		Absent	Absent	Absent
35	689	Combination	Absent	Absent	Absent		Absent	Absent	Absent
35	690	SSG	Absent	Absent	Absent	Absent	Absent	Absent	Absent

Centre	Patient				Axiliary lymphadenopathy				
Number	Number	Treatment	Baseline	Day7	Day 14	Day 21	EOT	3 mon FU	6 mon FU
11	1	PM	Absent	Absent	Absent		Absent	Absent	Absent
11	2	Combination	Absent	Absent	Absent		Absent	Absent	Absent
11	3	Combination	Absent	Absent	Absent		Absent	Absent	Absent
11	4	PM	Present	Absent	Absent		Absent	Absent	Absent
11	5	PM	Absent	Absent	Absent		Absent	Absent	Absent
11	6	Combination	Absent	Absent	Absent		Absent	Present	Absent
11	7	PM	Absent	Absent	Absent		Absent	Absent	Absent
11	8	SSG	Absent	Absent	Absent	Absent	Absent	Absent	Absent
11	9	SSG	Absent	Absent	Absent	Absent	Absent	Absent	Absent
11	10	SSG	Present	Present	Present	Present	Present	Absent	Absent
11	11	Combination	Absent	Absent	Absent		Absent	Absent	Absent
11	12	Combination	Present	Present	Present		Present	Present	Absent
11	13	SSG	Absent	Absent	Absent	Absent	Absent	Absent	Absent
11	14	SSG	Absent	Present	Absent	Absent	Absent	Absent	Absent
11	15	PM	Absent	Absent				Absent	Absent
11	16	Combination	Absent	Absent	Absent		Absent	Absent	Absent
11	17	Combination	Absent	Absent	Absent		Absent	Absent	Absent
11	18	SSG	Present	Present	Present	Absent	Present	Absent	Absent
11	19	Combination	Absent	Absent	Absent		Absent	Absent	Absent
11	20	SSG	Absent	Absent	Absent	Absent	Absent	Absent	Absent
11	21	SSG	Present	Present	Present	Absent	Absent	Absent	Absent
11	22	SSG	Absent	Absent	Absent	Absent	Absent	Absent	Absent
11	23	PM	Absent	Absent	Absent		Absent	Absent	Absent
11	24	PM	Absent	Absent	Absent		Absent	Absent	Absent
11	25	SSG	Absent	Absent	Absent	Absent	Absent	Absent	Absent
11	26	Combination	Present	Present	Present		Present	Absent	Absent
11	27	PM	Absent	Absent	Absent		Absent	Absent	
11	28	Combination	Absent	Absent	Absent		Absent	Absent	Absent
11	29	PM	Absent	Absent	Absent		Absent	Absent	Absent
11	30	PM	Absent	Absent	Absent		Absent	Absent	Absent
11	31	SSG	Absent	Absent	Absent	Absent	Absent	Absent	Absent
11	32	Combination	Absent	Absent	Absent		Absent	Absent	Absent
11	33	SSG	Absent	Absent	Absent	Absent	Absent	Absent	Absent
11	34	PM	Absent	Absent	Absent		Absent	Absent	Absent
11	35	SSG	Present	Absent	Absent	Present	Absent	Absent	Absent
11	36	SSG	Absent	Absent	Absent	Absent	Absent	Absent	Absent
11	37	Combination	Absent	Absent	Absent		Absent	Absent	Absent
11	38	PM	Absent	Absent	Absent		Absent	Absent	Absent
11	39	PM	Absent	Absent	Absent		Absent	Absent	Absent
11	40	PM	Absent	Absent	Absent			Absent	Absent
11	41	Combination	Absent	Absent	Absent		Absent	Absent	Absent
11	42	Combination	Absent	Absent	Absent		Absent	Absent	Absent
11	43	SSG	Absent	Absent	Absent	Absent	Absent	Absent	Absent
11	44	PM	Absent	Absent	Absent		Absent	Absent	Absent
11	45	Combination	Absent	Absent	Absent		Absent	Absent	Absent
11	46	SSG	Absent	Absent	Absent	Absent	Absent	Absent	Absent
11	47	SSG	Present	Absent	Absent	Absent	Absent		
11	48	Combination	Absent	Absent	Absent		Absent		Absent
11	49	SSG	Absent	Absent	Absent	Absent	Absent	Absent	Absent
11	50	PM	Absent	Absent	Absent		Absent	Absent	Absent
11	51	Combination	Absent	Absent	Absent		Absent	Absent	Absent
11	52	PM	Absent	Absent	Absent		Absent	Absent	Absent
11	53	SSG	Absent	Absent	Absent	Absent	Absent	Absent	Absent
11	54	PM	Absent	Absent	Absent		Absent	Absent	Absent

Centre	Patient					Axiliary lymphadenopathy			
Number	Number	Treatment	Baseline	Day7	Day 14	Day 21	EOT	3 mon FU	6 mon FU
11	55	PM	Absent	Absent	Absent		Absent	Absent	Absent
11	56	SSG	Absent	Absent	Absent	Absent	Absent	Absent	Absent
11	57	Combination	Absent	Absent	Absent		Absent	Absent	Absent
11	58	Combination	Absent	Absent	Absent		Absent	Absent	Absent
11	59	PM	Absent	Absent	Absent		Absent	Absent	Absent
11	60	Combination	Absent	Absent	Absent		Absent	Absent	Absent
11	61	PM	Absent	Absent	Absent		Absent	Absent	Absent
11	62	SSG	Absent	Absent	Absent	Absent	Absent	Absent	Absent
11	63	SSG	Absent	Absent	Absent	Absent	Absent	Absent	Absent
11	64	SSG	Absent	Absent	Absent	Absent	Absent	Absent	Absent
11	65	PM	Absent	Absent	Absent		Absent		
11	66	Combination	Absent	Absent	Absent		Absent	Absent	Absent
11	67	PM	Absent	Absent	Absent		Absent	Absent	Absent
11	68	SSG	Absent	Absent	Absent	Absent	Absent	Absent	Absent
11	69	Combination	Absent	Absent	Absent		Absent	Absent	Absent
11	70	Combination	Absent	Absent	Absent		Absent	Absent	Absent
11	71	Combination	Absent	Absent	Absent		Absent	Absent	Absent
11	72	SSG	Present	Present	Present			Absent	Absent
11	73	PM	Absent	Absent	Absent		Absent	Absent	Absent
11	74	PM	Absent	Absent	Absent		Absent	Absent	Absent
11	75	Combination	Absent	Absent	Absent		Absent	Absent	Absent
11	76	PM	Absent	Absent	Absent		Absent	Absent	Absent
11	77	PM	Absent	Absent	Absent		Absent	Absent	Absent
11	78	SSG	Absent	Absent	Absent	Absent	Absent	Absent	Absent
11	79	PM	Absent	Absent	Absent		Absent	Absent	Absent
11	80	PM	Absent	Absent	Absent		Absent	Absent	Absent
11	81	Combination	Absent	Absent	Absent		Absent	Absent	Absent
11	82	SSG	Absent	Absent	Absent	Absent	Absent	Absent	Absent
11	83	SSG	Absent	Absent	Absent	Absent	Absent	Absent	Absent
11	84	SSG	Absent	Absent	Absent	Absent	Absent	Absent	Absent
11	85	SSG	Absent	Absent	Absent	Absent	Absent	Absent	
11	86	Combination	Absent	Absent	Absent		Absent	Absent	Absent
11	87	Combination	Absent	Absent	Absent		Absent	Absent	Absent
11	88	PM	Absent	Absent	Absent		Absent	Absent	Absent
11	89	Combination	Absent	Absent	Absent		Absent	Absent	Absent
11	90	Combination	Absent	Absent	Absent		Absent		
11	91	PM	Absent	Absent	Absent		Absent	Absent	Absent
11	92	Combination	Absent	Absent	Absent		Absent	Absent	Absent
11	93	SSG	Absent	Absent	Absent	Absent	Absent	Absent	Absent
11	94	SSG	Absent	Absent	Absent	Absent	Absent	Absent	Absent
11	95	SSG	Absent	Absent	Absent	Absent	Absent	Absent	Absent
11	96	Combination	Absent	Absent	Absent		Absent	Absent	Absent
11	97	Combination	Absent	Absent	Absent		Absent	Absent	Absent
11	98	PM	Absent	Absent	Absent		Absent	Absent	Absent
11	99	SSG	Absent	Absent	Absent	Absent	Absent	Absent	Absent
11	100	PM	Absent	Absent	Absent		Absent		
11	101	PM	Absent	Absent	Absent		Absent	Absent	Absent
11	102	Combination	Absent	Absent	Absent		Absent	Absent	Absent
11	103	Combination	Absent	Absent	Absent		Absent	Absent	Absent
11	104	PM	Absent	Absent	Absent		Absent	Absent	Absent
11	105	SSG	Absent	Absent	Absent	Absent	Absent	Absent	Absent
11	106	Combination	Absent	Absent	Absent		Absent	Absent	Absent
11	107	PM	Present	Absent	Absent		Absent	Absent	Absent
11	108	PM	Absent	Absent	Absent		Absent	Absent	Absent

Centre Number	Patient Number	Treatment	Axiliary lymphadenopathy Baseline	Day7	Day 14	Day 21	EOT	3 mon FU	6 mon FU
11	109	SSG	Absent	Absent	Absent	Absent	Absent	Absent	Absent
11	110	PM	Absent	Absent	Absent		Absent	Absent	Absent
11	111	Combination	Absent	Absent	Absent		Absent	Absent	Absent
11	112	Combination	Absent	Absent	Absent		Absent	Absent	Absent
11	113	SSG	Absent	Absent	Absent	Absent	Absent	Absent	
11	114	SSG	Absent	Absent	Absent	Absent	Absent	Absent	Absent
11	115	Combination	Absent	Absent	Absent		Absent	Absent	Absent
11	116	SSG	Absent	Absent	Absent	Absent	Absent	Absent	Absent
11	117	PM	Absent	Absent	Absent		Absent	Absent	
11	118	PM	Absent	Absent	Absent		Absent	Absent	Absent
11	119	SSG	Absent	Absent	Absent	Absent	Absent	Absent	Absent
11	120	Combination	Absent	Absent	Absent		Absent	Absent	Absent
11	121	Combination	Absent	Absent	Absent		Absent	Absent	Absent
11	122	SSG	Absent	Absent	Absent	Absent	Absent		
11	123	SSG	Absent	Absent	Absent	Absent	Absent	Absent	Absent
11	124	SSG	Absent	Absent	Absent	Absent	Absent		Absent
11	125	SSG	Absent	Absent	Absent	Absent	Absent	Absent	Absent
11	126	Combination	Absent	Absent	Absent		Absent		Absent
11	127	PM	Absent	Absent	Absent		Absent	Absent	Absent
11	128	Combination	Present	Present	Absent		Absent	Absent	Absent
11	129	PM	Absent	Absent	Absent		Absent	Absent	Absent
11	130	Combination	Absent	Absent	Absent		Absent	Absent	Absent
11	131	PM	Absent	Absent	Absent		Absent	Absent	Absent
11	132	SSG	Absent	Absent	Absent	Absent	Absent	Absent	Absent
11	133	Combination	Absent	Absent	Absent		Absent	Absent	Absent
11	134	PM	Absent	Absent	Absent		Absent	Absent	Absent
11	135	PM	Absent	Absent	Absent		Absent	Absent	Absent
12	241	Combination	Absent	Absent	Absent		Absent		Absent
12	242	SSG	Present	Present	Present	Present	Present	Absent	Absent
12	243	PM	Absent	Absent	Absent		Absent	Absent	Absent
12	244	SSG	Absent	Absent	Absent	Absent	Absent	Absent	Absent
12	245	PM	Present	Present	Present		Present	Absent	
12	246	PM	Absent	Absent	Absent		Absent	Absent	
12	247	PM	Absent	Absent	Absent		Absent	Absent	Absent
12	248	PM	Absent	Absent	Absent		Absent	Absent	Absent
12	249	Combination	Absent	Absent	Absent		Absent	Absent	Absent
12	250	SSG	Absent	Absent	Present	Absent	Absent	Absent	Absent
12	251	Combination	Absent	Present	Present		Present	Absent	Absent
12	252	SSG	Absent	Absent	Absent	Absent	Absent	Absent	Absent
12	253	SSG	Absent	Absent	Present	Present	Present	Absent	Absent
12	254	Combination	Present	Present	Absent		Absent	Absent	Absent
12	255	Combination	Present	Absent	Absent		Absent	Absent	Absent
12	256	Combination	Absent	Absent	Absent		Absent	Absent	Absent
12	257	SSG	Present	Present	Absent	Absent	Absent	Absent	Absent
12	258	SSG	Absent	Absent	Absent	Absent	Absent		Absent
12	259	PM	Absent	Absent	Absent		Absent	Absent	Absent
12	260	PM	Absent	Absent	Absent		Absent		Absent
12	261	Combination	Absent	Absent	Absent		Absent	Absent	Absent
12	262	PM	Absent	Absent	Absent		Absent	Absent	Absent
12	263	SSG	Absent	Absent	Absent	Absent	Absent	Absent	Absent
12	264	Combination	Present	Absent	Absent		Absent	Absent	Absent
12	265	SSG	Absent	Absent	Absent	Absent	Absent		
12	266	PM	Absent	Absent	Absent		Absent	Absent	Absent
12	267	SSG	Absent	Absent	Absent	Absent	Absent	Absent	Absent

Centre Number	Patient Number	Treatment	Axiliary lymphadenopathy Baseline	Day7	Day 14	Day 21	EOT	3 mon FU	6 mon FU
12	268	PM	Absent	Absent	Absent		Absent	Absent	Absent
12	269	Combination	Absent	Absent	Absent		Absent	Absent	Absent
12	270	Combination	Absent	Absent	Absent		Absent		Absent
12	271	SSG	Absent	Absent	Absent	Absent	Absent	Absent	Absent
12	272	PM	Absent	Absent	Absent		Absent	Absent	Absent
12	273	SSG	Absent	Absent	Absent	Absent	Absent	Absent	Absent
12	274	SSG	Absent	Absent	Absent	Absent	Absent	Absent	Absent
12	275	Combination	Absent	Absent	Absent		Absent	Absent	Absent
12	276	PM	Present	Present	Absent		Absent	Absent	Absent
12	277	Combination	Absent	Absent	Absent		Absent	Absent	Absent
12	278	SSG	Absent	Absent	Absent	Absent	Absent	Absent	
12	279	PM	Absent	Absent	Absent		Absent	Absent	Absent
12	280	SSG	Absent	Absent	Absent	Absent	Absent	Absent	Absent
12	281	Combination	Absent	Absent	Absent		Absent	Absent	Absent
12	282	PM	Absent	Absent	Absent		Absent	Absent	Absent
12	283	PM	Absent	Absent	Absent		Absent	Absent	Absent
12	284	Combination	Absent	Absent	Absent		Absent	Present	Present
12	285	Combination	Present	Present	Present		Absent	Present	Absent
12	286	PM	Absent	Absent	Absent		Absent	Absent	Present
12	287	SSG	Absent	Absent	Absent	Absent	Absent	Absent	Absent
12	288	Combination	Absent	Absent	Absent		Absent	Absent	Absent
12	289	Combination	Absent	Absent	Absent		Absent	Absent	Absent
12	290	SSG	Absent	Absent	Absent	Absent	Absent		Absent
12	291	SSG	Absent	Absent	Absent	Absent	Absent	Absent	Absent
12	292	PM	Absent	Absent	Absent		Absent		Absent
12	293	SSG	Absent	Absent	Absent	Absent	Absent		Absent
12	294	Combination	Absent	Absent	Absent		Absent	Absent	Absent
12	295	Combination	Absent	Absent	Absent		Absent		Absent
12	296	PM	Absent	Absent	Absent		Absent	Absent	Absent
12	297	SSG	Present	Absent	Absent				
12	298	PM	Absent	Absent	Absent		Absent	Present	Absent
12	299	Combination	Absent	Absent	Absent		Absent		Absent
12	300	PM	Absent	Absent	Absent		Absent	Absent	Absent
12	301	SSG	Absent	Absent	Absent	Absent	Absent	Absent	Absent
12	302	SSG	Absent	Absent	Absent	Absent	Absent	Absent	Absent
12	303	PM	Absent	Absent	Absent		Absent	Absent	Absent
12	304	Combination	Absent	Absent	Absent		Absent	Absent	Present
12	305	PM	Absent	Absent	Absent		Absent	Absent	Absent
12	306	Combination	Absent	Absent	Absent		Absent	Absent	Absent
12	307	Combination	Absent	Absent	Absent		Absent	Absent	Absent
12	308	PM	Absent	Absent	Absent		Absent	Absent	Absent
12	309	PM	Absent	Absent	Absent		Absent	Absent	Absent
12	310	SSG	Absent	Absent	Absent	Absent	Absent	Absent	Absent
12	311	SSG	Absent	Absent	Absent	Absent	Absent	Absent	Absent
12	312	Combination	Absent	Absent	Absent		Absent	Absent	Absent
12	313	PM	Absent	Absent	Absent		Absent	Absent	Absent
12	314	SSG	Absent	Absent	Absent	Absent	Absent	Absent	Absent
12	315	Combination	Absent	Absent	Absent		Absent	Absent	Absent
12	316	SSG	Absent	Absent	Absent		Absent	Absent	Absent
12	317	SSG	Absent	Absent	Absent	Absent	Absent		Absent
12	318	Combination	Absent	Absent	Absent		Absent	Present	Absent
12	319	Combination	Absent	Absent	Absent		Absent	Absent	Absent
12	320	Combination	Absent	Absent	Absent		Absent	Present	Absent
12	321	Combination	Absent	Present	Absent		Absent	Present	Present

Centre Number	Patient Number	Treatment	Axiliary lymphadenopathy						
			Baseline	Day7	Day 14	Day 21	EOT	3 mon FU	6 mon FU
12	322	PM	Absent	Absent	Absent		Absent	Present	Absent
12	323	SSG	Present	Present	Absent	Absent	Absent	Absent	Absent
12	324	SSG	Absent	Absent	Absent	Absent	Absent	Absent	Absent
12	325	PM	Absent	Absent	Absent		Absent	Absent	Absent
12	326	PM	Absent	Absent	Absent		Absent	Absent	Absent
12	327	Combination	Absent	Absent	Absent		Absent	Absent	Absent
12	328	PM	Absent	Absent	Absent		Absent	Absent	Absent
12	329	SSG	Absent	Absent	Absent	Absent	Absent	Absent	Absent
12	330	PM	Absent	Absent	Absent		Absent	Absent	Absent
23	361	Combination	Absent	Absent	Absent		Absent	Absent	Absent
23	362	PM	Present	Absent	Absent		Absent	Absent	Absent
23	363	PM	Present	Absent	Absent		Absent	Present	Absent
23	364	Combination	Absent	Absent	Absent		Absent	Absent	Absent
23	365	SSG	Absent	Absent	Absent	Absent	Absent	Absent	Absent
23	366	Combination	Absent	Absent	Absent		Absent	Absent	Absent
23	367	SSG	Absent	Absent	Absent	Absent	Absent	Absent	Absent
23	368	PM	Absent	Absent	Absent		Absent	Absent	Absent
23	369	Combination	Absent	Absent	Absent		Absent	Absent	Absent
23	370	SSG	Absent	Absent	Absent	Absent	Absent	Absent	Absent
23	371	PM	Absent	Absent	Absent		Absent	Absent	Absent
23	372	PM	Absent	Absent	Absent		Absent	Absent	Absent
23	373	SSG	Absent	Absent	Absent	Absent	Absent	Absent	Absent
23	374	SSG	Absent	Absent	Absent	Absent	Absent	Absent	Absent
23	375	Combination	Absent	Absent	Absent		Absent	Absent	Absent
23	376	SSG	Absent	Absent	Absent	Absent	Absent	Absent	Absent
23	377	Combination	Absent	Absent					Absent
23	378	Combination	Absent	Absent	Absent		Absent	Absent	Absent
23	379	PM	Absent	Absent	Absent		Absent	Absent	Absent
23	380	SSG	Absent	Absent	Present	Absent	Absent	Absent	Absent
23	381	PM	Absent	Absent	Absent		Absent	Absent	Absent
23	382	Combination	Absent	Absent	Absent		Absent	Absent	Absent
23	383	PM	Absent	Absent	Absent		Absent	Absent	Absent
23	384	SSG	Absent	Absent	Absent	Absent	Absent	Absent	Absent
23	385	SSG	Present	Absent	Absent	Present	Present	Present	Absent
23	386	Combination	Absent	Absent	Absent		Absent	Absent	Absent
23	387	Combination	Absent	Absent	Absent		Absent	Absent	Absent
23	388	PM	Absent	Absent	Absent		Absent	Absent	Absent
23	389	SSG	Present	Present	Present	Absent	Absent	Absent	Absent
23	390	PM	Absent	Absent	Absent		Absent	Absent	Absent
23	391	Combination	Absent	Absent	Absent		Absent	Present	Absent
23	392	Combination	Present	Present	Present			Absent	Absent
23	393	SSG	Absent	Absent	Absent	Absent	Absent	Absent	Absent
23	394	PM	Absent	Absent	Absent		Absent	Absent	Absent
23	395	PM	Present	Present	Present		Absent	Absent	Absent
23	396	SSG	Absent	Absent	Absent	Absent	Absent	Absent	Absent
23	397	SSG	Absent	Absent	Absent	Absent	Absent	Absent	Absent
23	398	Combination	Present	Absent	Absent		Absent	Absent	Absent
23	399	PM	Absent	Absent	Absent		Absent	Absent	Absent
23	400	PM	Absent	Absent	Absent		Absent	Absent	Absent
23	401	Combination	Absent	Absent	Absent		Absent	Absent	Absent
23	402	Combination	Absent	Absent	Absent		Absent	Absent	Absent
23	403	SSG	Absent	Absent	Absent	Absent	Absent	Absent	Absent
23	404	SSG	Absent	Absent	Absent	Absent	Absent		Absent
23	405	PM	Absent	Absent	Absent		Absent	Absent	Absent

Centre Number	Patient Number	Treatment	Axiliary lymphadenopathy Baseline	Day7	Day 14	Day 21	EOT	3 mon FU	6 mon FU
34	451	Combination	Absent	Absent	Absent		Absent		Absent
34	452	PM	Absent	Absent	Absent		Absent		
34	453	PM	Absent	Absent	Absent		Absent		
34	454	Combination	Absent	Absent	Absent		Absent		Absent
34	455	PM	Absent	Absent	Absent		Absent		
34	456	SSG	Absent	Absent	Absent	Absent	Absent	Absent	
34	457	Combination	Absent	Absent	Absent		Absent		
34	458	PM	Absent	Absent	Absent		Absent	Absent	
34	459	Combination	Absent	Absent	Absent		Absent	Absent	Absent
34	460	PM	Absent	Absent	Absent		Absent		
34	461	SSG	Absent	Absent	Absent				
34	462	SSG	Absent	Absent	Absent	Absent	Absent		Absent
34	463	Combination	Absent	Absent	Absent		Absent		Absent
34	464	SSG	Absent	Absent	Absent	Absent	Absent		Absent
34	465	SSG	Absent	Absent	Absent	Absent	Absent		Absent
34	466	Combination	Absent	Absent	Absent		Absent		
34	467	PM	Absent	Absent	Absent		Absent	Absent	
34	468	Combination	Absent	Absent	Absent		Absent		Absent
34	469	SSG	Absent	Absent	Absent	Absent	Absent		Absent
34	470	Combination	Absent	Absent	Absent		Absent		Absent
34	471	PM	Absent	Absent	Absent		Absent		
34	472	SSG	Absent	Absent	Absent	Absent	Absent		Absent
34	473	PM	Absent	Absent	Absent		Absent		
34	474	Combination	Absent	Absent	Absent		Absent		Absent
34	475	PM	Absent	Absent	Absent		Absent		
34	476	PM	Absent	Absent	Absent		Absent	Present	
34	477	SSG	Absent	Absent	Absent	Absent	Absent		Absent
34	478	Combination	Absent	Absent	Absent		Absent		Absent
34	479	SSG	Absent	Absent	Absent	Absent	Absent		Absent
34	480	SSG	Absent	Absent	Absent	Absent	Absent		Absent
34	481	Combination	Absent	Absent	Absent		Absent		Absent
34	482	Combination	Absent	Absent	Absent		Absent		Absent
34	483	PM	Absent	Absent	Absent		Absent		Absent
34	484	PM	Absent	Absent					
34	485	Combination	Absent	Absent	Absent		Absent		Absent
34	486	Combination	Absent	Absent	Absent		Absent		Absent
34	487	SSG	Absent	Absent	Absent	Absent	Absent		Absent
34	488	SSG	Absent	Absent	Absent	Absent	Absent	Absent	Absent
34	489	SSG	Absent	Absent	Absent	Absent	Absent		Absent
34	490	PM	Absent	Absent	Absent		Absent		Absent
34	491	PM	Absent	Absent	Absent		Absent		
34	492	PM	Absent	Absent	Absent		Absent		Absent
34	493	SSG	Absent	Absent	Absent	Absent	Absent	Absent	
34	494	Combination	Absent	Absent	Absent		Absent		Absent
34	495	SSG	Absent	Absent	Absent	Absent	Absent		Absent
34	496	SSG	Absent	Absent	Absent	Absent	Absent		Absent
34	497	PM	Absent	Absent	Absent		Absent	Absent	
34	498	SSG	Absent	Absent	Absent	Absent	Absent		Absent
34	499	Combination	Absent	Absent	Absent		Absent		Absent
34	500	PM	Absent	Absent	Absent		Absent		Absent
34	501	Combination	Absent	Absent	Absent		Absent		Absent
34	502	Combination	Absent	Absent	Absent		Absent		Absent
34	503	PM	Absent	Absent	Absent		Absent		
34	504	Combination	Absent	Absent	Absent		Absent		

Centre Number	Patient Number	Treatment	Axiliary lymphadenopathy						
			Baseline	Day7	Day 14	Day 21	EOT	3 mon FU	6 mon FU
34	505	SSG	Absent	Absent	Absent	Absent	Absent		Absent
34	506	SSG	Absent	Absent	Absent	Absent	Absent		Present
34	507	SSG	Absent	Absent	Absent	Absent	Absent		Absent
34	508	PM	Absent	Absent	Absent		Absent		
34	509	PM	Absent	Absent	Absent		Absent		
34	510	Combination	Absent	Absent	Absent		Absent		Absent
34	511	Combination	Absent	Absent	Absent		Absent		Absent
34	512	PM	Absent	Absent	Absent		Absent		
34	513	Combination	Absent	Absent	Absent		Absent	Absent	Absent
34	514	SSG	Absent	Absent	Absent	Absent	Absent		Absent
34	515	SSG	Absent	Absent	Absent	Absent	Absent	Absent	
34	516	PM	Absent	Absent	Absent		Absent		Absent
34	517	PM	Absent	Absent	Absent		Absent		
34	518	SSG	Absent	Absent	Absent	Absent	Absent		Absent
34	519	PM	Absent	Absent	Absent		Absent	Absent	
34	520	SSG	Absent	Absent	Absent	Absent	Absent		Absent
34	521	PM	Absent	Absent	Absent		Absent		
34	522	Combination	Absent	Absent	Absent		Absent		
34	523	SSG	Absent	Absent	Absent	Absent	Absent		Absent
34	524	Combination	Absent	Absent	Absent		Absent		Absent
34	525	Combination	Absent	Absent	Absent		Absent		Absent
34	526	SSG	Absent	Absent	Absent	Absent	Absent		Present
34	527	Combination	Absent	Absent	Absent		Absent		Absent
34	528	PM	Absent	Absent	Absent		Absent		
34	529	Combination	Absent	Absent	Absent		Absent		Absent
34	530	Combination	Absent	Absent	Absent		Absent		Absent
34	531	SSG	Absent	Absent	Absent	Absent	Absent		
34	532	Combination	Absent	Absent	Absent		Absent		Absent
34	533	SSG	Absent	Absent	Absent	Absent	Absent		Absent
34	534	PM	Absent	Absent	Absent		Absent		
34	535	PM	Absent	Absent	Absent		Absent	Absent	
34	536	PM	Absent	Absent	Absent		Absent		Absent
34	537	SSG	Absent	Absent	Absent	Absent	Absent		Absent
34	538	PM	Absent	Absent	Absent		Absent		
34	539	Combination	Absent	Absent	Absent		Absent		Absent
34	540	SSG	Absent	Absent	Absent	Absent	Absent		Absent
35	646	PM	Absent	Absent	Absent		Absent		Absent
35	647	PM	Absent	Absent	Absent		Absent	Absent	Absent
35	648	Combination	Absent	Absent	Absent		Absent	Absent	Absent
35	649	Combination	Absent	Absent	Absent		Absent	Absent	Absent
35	650	SSG	Absent	Absent	Absent	Absent	Absent	Absent	Absent
35	651	SSG	Absent	Absent	Absent	Absent	Absent	Absent	Absent
35	652	PM	Absent	Absent	Absent		Absent		Absent
35	653	Combination	Absent	Absent	Absent		Absent	Absent	Absent
35	654	SSG	Absent	Absent	Absent	Absent	Absent	Absent	Absent
35	655	Combination	Absent	Absent	Absent		Absent	Absent	Absent
35	656	SSG	Absent	Absent	Absent	Absent	Absent	Absent	Absent
35	657	PM	Absent	Absent	Absent		Absent	Absent	Absent
35	658	Combination	Absent	Absent	Absent		Absent	Absent	Absent
35	659	SSG	Absent	Absent	Absent	Absent	Absent	Absent	Absent
35	660	PM	Absent	Absent	Absent		Absent	Absent	Absent
35	661	Combination	Absent	Absent	Absent		Absent	Absent	Absent
35	662	PM	Absent	Absent	Absent		Absent	Absent	Absent
35	663	SSG	Absent	Absent	Absent	Absent	Absent	Absent	Absent

Centre Number	Patient Number	Treatment	Axiliary lymphadenopathy						
			Baseline	Day7	Day 14	Day 21	EOT	3 mon FU	6 mon FU
35	664	Combination	Absent	Absent					Absent
35	665	SSG	Absent	Absent	Absent	Absent	Absent	Absent	Absent
35	666	SSG	Absent	Absent	Absent	Absent	Absent	Absent	Absent
35	667	Combination	Absent	Absent	Absent		Absent	Absent	Absent
35	668	PM	Absent	Absent	Absent		Absent		Absent
35	669	PM	Absent	Absent	Absent		Absent		
35	670	SSG	Absent	Absent	Absent	Absent	Absent	Absent	Absent
35	671	PM	Absent	Absent	Absent		Absent		Absent
35	672	Combination	Absent	Absent	Absent		Absent		Absent
35	673	SSG	Absent	Absent	Absent	Absent	Absent	Absent	Absent
35	674	Combination	Absent	Absent	Absent		Absent	Absent	Absent
35	675	PM	Absent	Absent	Absent		Absent		Absent
35	676	PM	Absent	Absent	Absent		Absent	Absent	Absent
35	677	SSG	Absent	Absent					
35	678	Combination	Absent	Absent	Absent		Absent	Absent	Absent
35	679	SSG	Absent	Absent	Absent	Absent	Absent	Absent	Absent
35	680	Combination	Absent	Absent	Absent		Absent	Absent	Absent
35	681	Combination	Absent	Absent	Absent		Absent	Absent	Absent
35	682	PM	Absent	Absent	Absent		Absent		Absent
35	683	Combination	Absent	Absent	Absent		Absent		Absent
35	684	SSG	Absent	Absent	Absent	Absent	Absent	Absent	Absent
35	685	PM	Absent	Absent	Absent		Absent	Absent	Absent
35	686	PM	Absent	Absent	Absent		Absent	Absent	Absent
35	687	SSG	Absent	Absent	Absent	Absent	Absent	Absent	Absent
35	688	PM	Absent	Absent	Absent		Absent	Absent	Absent
35	689	Combination	Absent	Absent	Absent		Absent	Absent	Absent
35	690	SSG	Absent	Absent	Absent	Absent	Absent	Absent	Absent

Appendix 41: Individual listing of clinical characteristics, inguinal lyphadenopathy and muscle wasting

Centre Number	Patient Number	Treatment	Inguinal lymphadenopathy Baseline	Day7	Day 14	Day 21	EOT	3 mon FU	6 mon FU
11	1	PM	Absent	Absent	Absent		Absent	Absent	Absent
11	2	Combination	Absent	Absent	Absent		Absent	Absent	Absent
11	3	Combination	Absent	Absent	Absent		Absent	Absent	Absent
11	4	PM	Present	Absent	Absent		Absent	Absent	Absent
11	5	PM	Absent	Absent	Absent		Absent	Absent	Absent
11	6	Combination	Absent	Absent	Absent		Absent	Absent	Absent
11	7	PM	Absent	Absent	Absent		Absent	Absent	Absent
11	8	SSG	Absent	Absent	Absent	Absent	Absent	Absent	Absent
11	9	SSG	Absent	Absent	Absent	Absent	Absent	Absent	Absent
11	10	SSG	Absent	Absent	Absent	Absent	Absent	Absent	Absent
11	11	Combination	Absent	Absent	Absent		Absent	Absent	Absent
11	12	Combination	Present	Present	Present		Present	Absent	Absent
11	13	SSG	Absent	Absent	Absent	Present	Absent	Absent	Absent
11	14	SSG	Absent	Absent	Absent	Absent	Absent	Absent	Absent
11	15	PM	Absent	Absent				Absent	Absent
11	16	Combination	Absent	Absent	Absent		Present	Absent	Absent
11	17	Combination	Absent	Absent	Absent		Absent	Absent	Absent
11	18	SSG	Absent	Absent	Absent	Absent	Absent	Absent	Absent
11	19	Combination	Absent	Absent	Absent		Absent	Absent	Absent
11	20	SSG	Absent	Absent	Absent	Absent	Absent	Absent	Absent
11	21	SSG	Absent	Absent	Absent	Absent	Absent	Absent	Absent
11	22	SSG	Absent	Absent	Absent	Absent	Absent	Absent	Absent
11	23	PM	Absent	Absent	Absent		Absent	Absent	Absent
11	24	PM	Absent	Absent	Absent		Absent	Absent	Absent
11	25	SSG	Absent	Absent	Absent	Absent	Absent	Absent	Absent
11	26	Combination	Absent	Absent	Absent		Absent	Absent	Absent
11	27	PM	Present	Absent	Absent		Absent	Absent	
11	28	Combination	Absent	Absent	Absent		Absent	Absent	Absent
11	29	PM	Absent	Absent	Absent		Absent	Absent	Absent
11	30	PM	Absent	Absent	Absent		Absent	Absent	Absent
11	31	SSG	Absent	Absent	Absent	Absent	Absent	Absent	Absent
11	32	Combination	Absent	Absent	Absent		Absent	Absent	Absent
11	33	SSG	Absent	Absent	Absent	Absent	Absent	Absent	Absent
11	34	PM	Absent	Absent	Absent		Absent	Absent	Absent
11	35	SSG	Absent	Absent	Absent	Absent	Absent	Absent	Absent
11	36	SSG	Absent	Absent	Absent	Absent	Absent	Absent	Absent
11	37	Combination	Absent	Absent	Absent		Absent	Absent	Absent
11	38	PM	Present	Absent	Absent		Absent	Absent	Absent
11	39	PM	Absent	Absent	Absent		Absent	Absent	Absent
11	40	PM	Absent	Absent	Absent			Absent	Absent
11	41	Combination	Absent	Absent	Absent		Absent	Absent	Absent
11	42	Combination	Present	Absent	Absent		Absent	Absent	Absent
11	43	SSG	Absent	Absent	Absent	Absent	Absent	Absent	Absent
11	44	PM	Absent	Absent	Absent		Absent	Absent	Absent
11	45	Combination	Absent	Absent	Absent		Absent	Absent	Absent
11	46	SSG	Absent	Absent	Absent	Absent	Absent	Absent	Absent
11	47	SSG	Absent	Absent	Absent	Absent	Absent		
11	48	Combination	Absent	Absent	Absent		Absent		Absent
11	49	SSG	Absent	Absent	Absent	Absent	Absent	Absent	Absent
11	50	PM	Absent	Absent	Absent		Absent	Absent	Absent
11	51	Combination	Absent	Absent	Absent		Absent	Absent	Absent
11	52	PM	Absent	Absent	Absent		Absent	Absent	Absent
11	53	SSG	Absent	Absent	Absent	Absent	Absent	Absent	Absent
11	54	PM	Absent	Absent	Absent		Absent	Absent	Absent

Centre Number	Patient Number	Treatment	Inguinal lymphadenopathy						
			Baseline	Day7	Day 14	Day 21	EOT	3 mon FU	6 mon FU
11	55	PM	Absent	Absent	Absent		Absent	Absent	Absent
11	56	SSG	Absent	Absent	Absent	Absent	Absent	Absent	Absent
11	57	Combination	Absent	Absent	Absent		Absent	Absent	Absent
11	58	Combination	Absent	Absent	Absent		Absent	Absent	Absent
11	59	PM	Absent	Absent	Absent		Absent	Absent	Absent
11	60	Combination	Absent	Absent	Absent		Absent	Absent	Absent
11	61	PM	Absent	Absent	Absent		Absent	Absent	Absent
11	62	SSG	Absent	Absent	Absent	Absent	Absent	Absent	Absent
11	63	SSG	Absent	Absent	Absent	Absent	Absent	Absent	Absent
11	64	SSG	Absent	Absent	Absent	Absent	Absent	Absent	Absent
11	65	PM	Absent	Absent	Absent		Absent		
11	66	Combination	Absent	Absent	Absent		Absent	Absent	Absent
11	67	PM	Absent	Absent	Absent		Absent	Absent	Absent
11	68	SSG	Absent	Absent	Absent	Absent	Absent	Absent	Absent
11	69	Combination	Absent	Absent	Absent		Absent	Absent	Absent
11	70	Combination	Absent	Absent	Absent		Absent	Absent	Absent
11	71	Combination	Absent	Absent	Absent		Absent	Absent	Absent
11	72	SSG	Absent	Absent	Absent			Absent	Absent
11	73	PM	Absent	Absent	Absent		Absent	Absent	Absent
11	74	PM	Absent	Absent	Absent		Absent	Absent	Absent
11	75	Combination	Absent	Absent	Absent		Absent	Absent	Absent
11	76	PM	Absent	Absent	Absent		Absent	Absent	Absent
11	77	PM	Absent	Absent	Absent		Absent	Absent	Absent
11	78	SSG	Absent	Absent	Absent	Absent	Absent	Absent	Absent
11	79	PM	Absent	Absent	Absent		Absent	Absent	Absent
11	80	PM	Absent	Absent	Absent		Absent	Absent	Absent
11	81	Combination	Absent	Absent	Absent		Absent	Absent	Absent
11	82	SSG	Absent	Absent	Absent	Absent	Absent	Absent	Absent
11	83	SSG	Absent	Absent	Absent	Absent	Absent	Absent	Absent
11	84	SSG	Absent	Absent	Absent	Absent	Absent	Absent	Absent
11	85	SSG	Absent	Absent	Absent	Absent	Absent	Absent	
11	86	Combination	Absent	Absent	Absent		Absent	Absent	Absent
11	87	Combination	Absent	Absent	Absent		Absent	Absent	Absent
11	88	PM	Absent	Absent	Absent		Absent	Absent	Absent
11	89	Combination	Absent	Absent	Absent		Absent	Absent	Absent
11	90	Combination	Absent	Absent	Absent		Absent		
11	91	PM	Absent	Absent	Absent		Absent	Absent	Absent
11	92	Combination	Absent	Absent	Absent		Absent	Absent	Absent
11	93	SSG	Absent	Absent	Absent	Absent	Absent	Absent	Absent
11	94	SSG	Absent	Absent	Absent	Absent	Absent	Absent	Absent
11	95	SSG	Absent	Absent	Absent	Absent	Absent	Absent	Absent
11	96	Combination	Absent	Absent	Absent		Absent	Absent	Absent
11	97	Combination	Present	Absent	Absent		Absent	Absent	Absent
11	98	PM	Absent	Absent	Absent		Absent	Absent	Absent
11	99	SSG	Present	Absent	Absent	Absent	Absent	Absent	Absent
11	100	PM	Absent	Absent	Absent		Absent		
11	101	PM	Absent	Absent	Absent		Absent	Absent	Absent
11	102	Combination	Absent	Absent	Absent		Absent	Absent	Absent
11	103	Combination	Absent	Absent	Absent		Absent	Absent	Absent
11	104	PM	Absent	Absent	Absent		Absent	Absent	Absent
11	105	SSG	Absent	Absent	Absent	Absent	Absent	Absent	Absent
11	106	Combination	Absent	Absent	Absent		Absent	Absent	Absent
11	107	PM	Absent	Absent	Absent		Absent	Absent	Absent
11	108	PM	Absent	Absent	Absent		Absent	Absent	Absent

Centre Number	Patient Number	Treatment	Inguinal lymphadenopathy Baseline	Day7	Day 14	Day 21	EOT	3 mon FU	6 mon FU
11	109	SSG	Absent	Absent	Absent	Absent	Absent	Absent	Absent
11	110	PM	Absent	Absent	Absent		Absent	Absent	Absent
11	111	Combination	Absent	Absent	Absent		Absent	Absent	Absent
11	112	Combination	Absent	Absent	Absent		Absent	Absent	Absent
11	113	SSG	Absent	Absent	Absent	Absent	Absent	Absent	
11	114	SSG	Absent	Absent	Absent	Absent	Absent	Absent	Absent
11	115	Combination	Absent	Absent	Absent		Absent	Absent	Absent
11	116	SSG	Absent	Absent	Absent	Absent	Absent	Absent	Absent
11	117	PM	Absent	Absent	Absent		Absent	Absent	
11	118	PM	Absent	Absent	Absent		Absent	Absent	Absent
11	119	SSG	Present	Absent	Absent	Absent	Absent	Absent	Absent
11	120	Combination	Absent	Absent	Absent		Absent	Absent	Absent
11	121	Combination	Absent	Absent	Absent		Absent	Absent	Absent
11	122	SSG	Present	Absent	Absent	Absent	Absent		
11	123	SSG	Absent	Absent	Absent	Absent	Absent	Absent	Absent
11	124	SSG	Absent	Absent	Absent	Absent	Absent		Absent
11	125	SSG	Absent	Absent	Absent	Absent	Absent	Absent	Absent
11	126	Combination	Absent	Absent	Absent		Absent		Absent
11	127	PM	Absent	Absent	Absent		Absent	Absent	Absent
11	128	Combination	Absent	Absent	Absent		Absent	Absent	Absent
11	129	PM	Absent	Absent	Absent		Absent	Absent	Absent
11	130	Combination	Absent	Absent	Absent		Absent	Absent	Absent
11	131	PM	Absent	Absent	Absent		Absent	Absent	Absent
11	132	SSG	Absent	Absent	Absent	Absent	Absent	Absent	Absent
11	133	Combination	Absent	Absent	Absent		Absent	Absent	Absent
11	134	PM	Absent	Absent	Absent		Absent	Absent	Absent
11	135	PM	Absent	Absent	Absent		Absent	Absent	Absent
12	241	Combination	Absent	Absent	Absent		Absent		Absent
12	242	SSG	Present	Present	Absent	Absent	Absent	Absent	Present
12	243	PM	Absent	Absent	Absent		Absent	Absent	Absent
12	244	SSG	Present	Absent	Absent	Absent	Absent	Absent	Absent
12	245	PM	Present	Present	Present		Present	Absent	
12	246	PM	Absent	Absent	Absent		Absent	Absent	
12	247	PM	Present	Present	Present		Present	Absent	Absent
12	248	PM	Absent	Absent	Absent		Absent	Absent	Absent
12	249	Combination	Present	Absent	Absent		Absent	Absent	Absent
12	250	SSG	Absent	Present	Absent	Absent	Absent	Absent	Absent
12	251	Combination	Present	Present	Present		Present	Absent	Absent
12	252	SSG	Present	Present	Absent	Absent	Absent	Absent	Absent
12	253	SSG	Absent	Absent	Absent	Absent	Absent	Absent	Absent
12	254	Combination	Present	Present	Present		Present	Absent	Absent
12	255	Combination	Absent	Absent	Absent		Absent	Absent	Absent
12	256	Combination	Absent	Absent	Absent		Absent	Absent	Absent
12	257	SSG	Present	Present	Absent	Absent	Absent	Absent	Absent
12	258	SSG	Absent	Absent	Absent	Absent	Absent		Absent
12	259	PM	Absent	Absent	Absent		Absent	Absent	Absent
12	260	PM	Absent	Absent	Absent		Absent		Absent
12	261	Combination	Absent	Absent	Absent		Absent	Absent	Present
12	262	PM	Absent	Absent	Absent		Absent	Absent	Absent
12	263	SSG	Absent	Absent	Absent	Absent	Absent	Absent	Absent
12	264	Combination	Absent	Absent	Absent		Absent	Absent	Absent
12	265	SSG	Absent	Absent	Absent	Absent	Absent		
12	266	PM	Absent	Absent	Absent		Absent	Absent	Absent
12	267	SSG	Absent	Absent	Absent	Absent	Absent	Absent	Absent

Centre Number	Patient Number	Treatment	Inguinal lymphadenopathy						
			Baseline	Day7	Day 14	Day 21	EOT	3 mon FU	6 mon FU
12	268	PM	Absent	Absent	Absent		Absent	Absent	Absent
12	269	Combination	Absent	Absent	Absent		Absent	Absent	Absent
12	270	Combination	Absent	Absent	Absent		Absent		Absent
12	271	SSG	Absent	Absent	Absent	Absent	Absent	Absent	Absent
12	272	PM	Absent	Absent	Absent		Absent	Absent	Absent
12	273	SSG	Present	Present	Absent	Absent	Absent	Absent	Absent
12	274	SSG	Absent	Absent	Absent	Absent	Absent	Absent	Absent
12	275	Combination	Present	Absent	Absent		Absent	Absent	Absent
12	276	PM	Absent	Absent	Absent		Absent	Absent	Absent
12	277	Combination	Present	Present	Present		Present	Absent	Absent
12	278	SSG	Absent	Absent	Absent	Absent	Absent	Absent	
12	279	PM	Absent	Absent	Absent		Absent	Absent	Absent
12	280	SSG	Absent	Absent	Absent	Absent	Absent	Absent	Absent
12	281	Combination	Absent	Absent	Absent		Absent	Absent	Absent
12	282	PM	Absent	Absent	Absent		Absent	Absent	Absent
12	283	PM	Absent	Absent	Absent		Absent	Absent	Absent
12	284	Combination	Absent	Absent	Absent		Absent	Absent	Absent
12	285	Combination	Present	Present	Present		Present	Absent	Absent
12	286	PM	Absent	Absent	Absent		Absent	Absent	Present
12	287	SSG	Absent	Absent	Absent	Absent	Absent	Absent	Absent
12	288	Combination	Absent	Absent	Absent		Absent	Absent	Absent
12	289	Combination	Absent	Absent	Absent		Absent	Absent	Absent
12	290	SSG	Absent	Absent	Absent	Absent	Absent		Absent
12	291	SSG	Absent	Absent	Absent	Absent	Absent	Absent	Absent
12	292	PM	Absent	Absent	Absent		Absent		Absent
12	293	SSG	Absent	Absent	Absent	Absent	Absent		Absent
12	294	Combination	Absent	Absent	Absent		Absent	Absent	Absent
12	295	Combination	Absent	Absent	Absent		Absent		Absent
12	296	PM	Absent	Present	Present		Present	Absent	Absent
12	297	SSG	Present	Absent	Absent				
12	298	PM	Absent	Absent	Absent		Absent	Present	Absent
12	299	Combination	Absent	Absent	Absent		Absent		Absent
12	300	PM	Absent	Absent	Absent		Absent	Absent	Absent
12	301	SSG	Absent	Absent	Absent	Absent	Absent	Absent	Absent
12	302	SSG	Absent	Absent	Absent	Absent	Absent	Absent	Absent
12	303	PM	Absent	Absent	Absent		Absent	Absent	Absent
12	304	Combination	Absent	Absent	Absent		Absent	Absent	Present
12	305	PM	Absent	Absent	Absent		Absent	Absent	Absent
12	306	Combination	Absent	Absent	Absent		Absent	Absent	Present
12	307	Combination	Absent	Absent	Absent		Absent	Absent	Absent
12	308	PM	Absent	Absent	Absent		Absent	Absent	Absent
12	309	PM	Absent	Absent	Absent		Absent	Absent	Absent
12	310	SSG	Absent	Absent	Absent	Absent	Absent	Absent	Absent
12	311	SSG	Absent	Absent	Absent	Absent	Absent	Absent	Absent
12	312	Combination	Absent	Absent	Absent		Absent	Absent	Absent
12	313	PM	Absent	Absent	Absent		Absent	Absent	Absent
12	314	SSG	Absent	Absent	Absent	Absent	Absent	Absent	Absent
12	315	Combination	Absent	Absent	Absent		Absent	Absent	Absent
12	316	SSG	Absent	Absent	Absent		Absent	Absent	Absent
12	317	SSG	Absent	Absent	Absent	Absent	Absent		Absent
12	318	Combination	Absent	Absent	Absent		Present	Present	Absent
12	319	Combination	Present	Absent	Absent		Absent	Absent	Absent
12	320	Combination	Absent	Absent	Absent		Absent	Present	Absent
12	321	Combination	Absent	Absent	Absent		Absent	Present	Present

Centre Number	Patient Number	Treatment	Inguinal lymphadenopathy Baseline	Day7	Day 14	Day 21	EOT	3 mon FU	6 mon FU
12	322	PM	Absent	Absent	Absent		Absent	Absent	Absent
12	323	SSG	Absent	Absent	Present	Absent	Absent	Absent	Absent
12	324	SSG	Present	Present	Present	Present	Present	Absent	Absent
12	325	PM	Present	Present	Present		Absent	Absent	Absent
12	326	PM	Present	Present	Present		Present	Absent	Absent
12	327	Combination	Absent	Absent	Present		Absent	Absent	Absent
12	328	PM	Absent	Absent	Absent		Absent	Absent	Absent
12	329	SSG	Absent	Absent	Absent	Absent	Absent	Absent	Absent
12	330	PM	Absent	Absent	Absent		Absent	Absent	Absent
23	361	Combination	Absent	Absent	Absent		Absent	Present	Absent
23	362	PM	Absent	Absent	Absent		Absent	Absent	Absent
23	363	PM	Absent	Absent	Absent		Absent	Present	Absent
23	364	Combination	Absent	Absent	Absent		Absent	Absent	Absent
23	365	SSG	Absent	Absent	Absent	Absent	Absent	Absent	Absent
23	366	Combination	Absent	Absent	Absent		Absent	Absent	Absent
23	367	SSG	Absent	Absent	Absent	Absent	Absent	Absent	Absent
23	368	PM	Absent	Absent	Absent		Absent	Absent	Absent
23	369	Combination	Absent	Absent	Absent		Absent	Absent	Absent
23	370	SSG	Absent	Absent	Absent	Absent	Absent	Absent	Absent
23	371	PM	Absent	Absent	Absent		Absent	Absent	Absent
23	372	PM	Present	Absent	Absent		Absent	Absent	Absent
23	373	SSG	Absent	Absent	Absent	Absent	Absent	Absent	Absent
23	374	SSG	Absent	Absent	Absent	Absent	Absent	Absent	Absent
23	375	Combination	Absent	Absent	Absent		Absent	Absent	Absent
23	376	SSG	Absent	Absent	Absent	Absent	Absent	Absent	Absent
23	377	Combination	Absent	Absent					Absent
23	378	Combination	Absent	Absent	Absent		Absent	Absent	Absent
23	379	PM	Absent	Absent	Absent		Absent	Absent	Absent
23	380	SSG	Absent	Absent	Absent	Absent	Absent	Absent	Absent
23	381	PM	Absent	Absent	Absent		Absent	Absent	Absent
23	382	Combination	Present	Present	Absent		Absent	Absent	Absent
23	383	PM	Absent	Absent	Absent		Absent	Absent	Absent
23	384	SSG	Absent	Absent	Absent	Absent	Absent	Absent	Absent
23	385	SSG	Present	Absent	Absent	Present	Present	Present	Absent
23	386	Combination	Absent	Absent	Absent		Absent	Absent	Absent
23	387	Combination	Absent	Absent	Absent		Absent	Absent	Absent
23	388	PM	Absent	Absent	Absent		Absent	Absent	Absent
23	389	SSG	Present	Present	Present	Present	Present	Absent	Absent
23	390	PM	Absent	Absent	Absent		Absent	Absent	Absent
23	391	Combination	Absent	Absent	Absent		Absent	Absent	Absent
23	392	Combination	Present	Present	Absent			Absent	Absent
23	393	SSG	Present	Present	Present	Present	Absent	Absent	Absent
23	394	PM	Absent	Absent	Absent		Absent	Absent	Absent
23	395	PM	Absent	Present	Absent		Absent	Absent	Absent
23	396	SSG	Absent	Absent	Absent	Absent	Absent	Absent	Absent
23	397	SSG	Absent	Absent	Absent	Absent	Absent	Absent	Absent
23	398	Combination	Absent	Absent	Absent		Absent	Absent	Absent
23	399	PM	Absent	Absent	Absent		Absent	Absent	Absent
23	400	PM	Absent	Absent	Absent		Absent	Absent	Absent
23	401	Combination	Absent	Absent	Absent		Absent	Absent	Absent
23	402	Combination	Absent	Absent	Absent		Absent	Absent	Absent
23	403	SSG	Absent	Absent	Absent	Absent	Absent	Absent	Absent
23	404	SSG	Absent	Absent	Absent	Absent	Absent		Absent
23	405	PM	Absent	Absent	Absent		Absent	Absent	Absent

Centre Number	Patient Number	Treatment	Inguinal lymphadenopathy Baseline	Day7	Day 14	Day 21	EOT	3 mon FU	6 mon FU
34	451	Combination	Present	Present	Present		Present		Present
34	452	PM	Present	Present	Present		Present		
34	453	PM	Present	Present	Present		Present		
34	454	Combination	Present	Present	Present		Present		Present
34	455	PM	Present	Present	Present		Present		
34	456	SSG	Present	Present	Present	Present	Present	Present	
34	457	Combination	Present	Present	Present		Present		
34	458	PM	Present	Present	Present		Present	Present	
34	459	Combination	Present	Present	Present		Present	Present	Present
34	460	PM	Present	Present	Present		Present		
34	461	SSG	Present	Present	Present				
34	462	SSG	Present	Present	Present	Present	Present		Present
34	463	Combination	Present	Present	Present		Present		Present
34	464	SSG	Present	Present	Present	Present	Present		Present
34	465	SSG	Present	Present	Present	Present	Present		Present
34	466	Combination	Present	Present	Present		Present		
34	467	PM	Present	Present	Present		Present	Present	
34	468	Combination	Present	Present	Present		Present		Present
34	469	SSG	Present	Present	Present	Present	Present		Present
34	470	Combination	Present	Present	Present		Present		Present
34	471	PM	Present	Present	Present		Present		
34	472	SSG	Present	Present	Present	Present	Present		Present
34	473	PM	Present	Present	Present		Present		
34	474	Combination	Present	Present	Present		Present		Present
34	475	PM	Present	Present	Present		Present		
34	476	PM	Present	Present	Present		Present	Present	
34	477	SSG	Present	Present	Present	Present	Present		Present
34	478	Combination	Present	Present	Present		Present		Present
34	479	SSG	Present	Present	Absent	Absent	Absent		Present
34	480	SSG	Present	Present	Present	Present	Present		Present
34	481	Combination	Present	Present	Present		Present		Present
34	482	Combination	Present	Present	Present		Present		Present
34	483	PM	Present	Present	Present		Present		Present
34	484	PM	Present	Present					
34	485	Combination	Present	Present	Present		Present		Present
34	486	Combination	Present	Present	Present		Present		Present
34	487	SSG	Present	Present	Present	Present	Present		Present
34	488	SSG	Present	Present	Present	Present	Present	Present	Present
34	489	SSG	Present	Present	Present	Present	Present		Present
34	490	PM	Present	Present	Present		Present		Present
34	491	PM	Present	Present	Present		Present		
34	492	PM	Present	Present	Present		Present		Present
34	493	SSG	Present	Present	Present	Present	Present	Present	
34	494	Combination	Present	Present	Present		Present		Present
34	495	SSG	Present	Present	Present	Present	Present		Present
34	496	SSG	Present	Present	Present	Present	Present		Present
34	497	PM	Present	Present	Present		Present	Present	
34	498	SSG	Present	Present	Present	Present	Present		Present
34	499	Combination	Present	Present	Present		Present		Present
34	500	PM	Present	Present	Present		Present		Present
34	501	Combination	Present	Present	Present		Present		Present
34	502	Combination	Present	Present	Present		Present		Present
34	503	PM	Present	Present	Present		Present		
34	504	Combination	Present	Present	Present		Present		

Centre Number	Patient Number	Treatment	Inguinal lymphadenopathy						
			Baseline	Day7	Day 14	Day 21	EOT	3 mon FU	6 mon FU
34	505	SSG	Present	Present	Present	Present	Present		Present
34	506	SSG	Present	Present	Present	Present	Present		Present
34	507	SSG	Present	Present	Present	Present	Present		Present
34	508	PM	Present	Present	Present		Present		
34	509	PM	Present	Present	Present		Present		
34	510	Combination	Present	Present	Present		Present		Present
34	511	Combination	Present	Present	Present		Present		Present
34	512	PM	Present	Present	Present		Present		
34	513	Combination	Present	Present	Present		Present	Present	Present
34	514	SSG	Present	Present	Present	Present	Present		Present
34	515	SSG	Present	Present	Present	Present	Present	Present	
34	516	PM	Present	Present	Present		Present		Present
34	517	PM	Present	Present	Present		Present		
34	518	SSG	Present	Present	Present	Present	Present		Present
34	519	PM	Present	Present	Present		Present	Present	
34	520	SSG	Present	Present	Present	Present	Present		Present
34	521	PM	Present	Present	Present		Present		
34	522	Combination	Present	Present	Present		Present		
34	523	SSG	Present	Present	Present	Present	Present		Present
34	524	Combination	Present	Present	Present		Present		Present
34	525	Combination	Present	Present	Present		Present		Present
34	526	SSG	Present	Present	Present	Present	Present		Present
34	527	Combination	Present	Present	Present		Present		Absent
34	528	PM	Present	Present	Present		Present		
34	529	Combination	Present	Present	Present		Present		Present
34	530	Combination	Present	Present	Present		Present		Present
34	531	SSG	Present	Present	Present	Present	Present		
34	532	Combination	Present	Present	Present		Present		Present
34	533	SSG	Present	Present	Present	Present	Present		Present
34	534	PM	Present	Present	Present		Present		
34	535	PM	Present	Present	Present		Present	Present	
34	536	PM	Present	Present	Present		Present		Present
34	537	SSG	Present	Present	Present	Present	Present		Present
34	538	PM	Present	Present	Present		Present		
34	539	Combination	Present	Present	Present		Present		Present
34	540	SSG	Present	Present	Present	Present	Present		Present
35	646	PM	Present	Present	Present		Present		Absent
35	647	PM	Present	Present	Present		Present	Absent	Absent
35	648	Combination	Present	Present	Present		Present	Present	Absent
35	649	Combination	Present	Present	Present		Present	Present	Absent
35	650	SSG	Present	Present	Present	Present	Present	Absent	Absent
35	651	SSG	Present	Present	Present	Present	Present	Absent	Absent
35	652	PM	Present	Present	Present		Present		Absent
35	653	Combination	Present	Present	Present		Present	Absent	Absent
35	654	SSG	Present	Present	Present	Present	Present	Present	Absent
35	655	Combination	Present	Present	Present		Present	Present	Absent
35	656	SSG	Absent	Present	Present	Present	Present	Absent	Absent
35	657	PM	Present	Present	Present		Present	Present	Absent
35	658	Combination	Present	Present	Present		Present	Present	Absent
35	659	SSG	Present	Present	Present	Present	Present	Absent	Absent
35	660	PM	Present	Present	Present		Present	Absent	Absent
35	661	Combination	Present	Present	Present		Present	Absent	Absent
35	662	PM	Present	Present	Present		Present	Present	Absent
35	663	SSG	Absent	Absent	Absent	Present	Present	Absent	Absent

Centre Number	Patient Number	Treatment	Inguinal lymphadenopathy Baseline	Day7	Day 14	Day 21	EOT	3 mon FU	6 mon FU
35	664	Combination	Absent	Absent					Absent
35	665	SSG	Present	Present	Present	Present	Present	Absent	Absent
35	666	SSG	Present	Present	Present	Present	Present	Absent	Absent
35	667	Combination	Present	Present	Present		Present	Absent	Absent
35	668	PM	Present	Present	Present		Present		Absent
35	669	PM	Present	Present	Present		Present		
35	670	SSG	Present	Present	Present	Present	Present	Absent	Absent
35	671	PM	Present	Present	Present		Present		Absent
35	672	Combination	Present	Present	Present		Present		Absent
35	673	SSG	Present	Present	Present	Present	Present	Absent	Absent
35	674	Combination	Present	Present	Present		Present	Absent	Absent
35	675	PM	Present	Present	Present		Present		Absent
35	676	PM	Present	Present	Present		Present	Absent	Absent
35	677	SSG	Present	Present					
35	678	Combination	Present	Present	Present		Present	Absent	Absent
35	679	SSG	Present	Present	Present	Present	Present	Absent	Absent
35	680	Combination	Present	Present	Present		Present	Absent	Absent
35	681	Combination	Present	Present	Present		Present	Absent	Absent
35	682	PM	Present	Present	Present		Present		Absent
35	683	Combination	Present	Present	Present		Present		Absent
35	684	SSG	Present	Present	Present	Present	Present	Absent	Absent
35	685	PM	Present	Present	Present		Present	Absent	Absent
35	686	PM	Present	Present	Present		Present	Absent	Absent
35	687	SSG	Present	Present	Present	Present	Present	Absent	Absent
35	688	PM	Present	Present	Present		Present	Absent	Absent
35	689	Combination	Present	Present	Present		Present	Absent	Absent
35	690	SSG	Present	Present	Present	Present	Present	Absent	Absent

Centre Number	Patient Number	Treatment	Baseline	Day7	Day 14	Muscle Wasting<br>Day 21	EOT	3 mon FU	6 mon FU
11	1	PM	Absent	Absent	Absent		Absent	Absent	Absent
11	2	Combination	Absent	Absent	Absent		Absent	Absent	Absent
11	3	Combination	Absent	Absent	Absent		Absent	Absent	Absent
11	4	PM	Absent	Absent	Absent		Absent	Absent	Absent
11	5	PM	Absent	Absent	Absent		Absent	Absent	Absent
11	6	Combination	Absent	Absent	Absent		Absent	Absent	Absent
11	7	PM	Absent	Absent	Absent		Absent	Absent	Absent
11	8	SSG	Absent	Absent	Absent	Absent	Absent	Absent	Absent
11	9	SSG	Absent	Absent	Absent	Absent	Absent	Absent	Absent
11	10	SSG	Absent	Absent	Absent	Absent	Absent	Absent	Absent
11	11	Combination	Absent	Absent	Absent		Absent	Absent	Absent
11	12	Combination	Absent	Absent	Absent		Absent	Absent	Absent
11	13	SSG	Absent	Absent	Absent	Absent	Absent	Absent	Absent
11	14	SSG	Absent	Absent	Absent	Absent	Absent	Absent	Absent
11	15	PM	Absent	Absent				Absent	Absent
11	16	Combination	Absent	Absent	Absent		Absent	Absent	Absent
11	17	Combination	Absent	Absent	Absent		Absent	Absent	Absent
11	18	SSG	Absent	Absent	Absent	Absent	Absent	Absent	Absent
11	19	Combination	Absent	Absent	Absent		Absent	Absent	Absent
11	20	SSG	Absent	Absent	Absent	Absent	Absent	Absent	Absent
11	21	SSG	Absent	Absent	Absent	Absent	Absent	Absent	Absent
11	22	SSG	Absent	Absent	Absent	Absent	Absent	Absent	Absent
11	23	PM	Absent	Absent	Absent		Absent	Absent	Absent
11	24	PM	Absent	Absent	Absent		Absent	Absent	Absent
11	25	SSG	Absent	Absent	Absent	Absent	Absent	Absent	Absent
11	26	Combination	Absent	Absent	Absent		Absent	Absent	Absent
11	27	PM	Present	Present	Present		Present	Absent	
11	28	Combination	Absent	Absent	Absent		Absent	Absent	Absent
11	29	PM	Present	Absent	Absent		Absent	Absent	Absent
11	30	PM	Absent	Absent	Absent		Absent	Absent	Absent
11	31	SSG	Absent	Absent	Present	Absent	Absent	Absent	Absent
11	32	Combination	Absent	Absent	Absent		Absent	Absent	Absent
11	33	SSG	Present	Absent	Absent	Absent	Absent	Absent	Absent
11	34	PM	Absent	Absent	Absent		Absent	Absent	Absent
11	35	SSG	Absent	Absent	Absent	Absent	Absent	Absent	Absent
11	36	SSG	Present	Present	Absent	Absent	Absent	Absent	Absent
11	37	Combination	Absent	Absent	Absent		Absent	Absent	Absent
11	38	PM	Present	Present	Present		Absent	Absent	Present
11	39	PM	Present	Present	Absent		Absent	Absent	Absent
11	40	PM	Absent	Absent	Absent			Absent	Absent
11	41	Combination	Absent	Absent	Absent		Absent	Absent	Absent
11	42	Combination	Absent	Absent	Absent		Absent	Absent	Absent
11	43	SSG	Absent	Absent	Absent	Absent	Absent	Absent	Absent
11	44	PM	Absent	Absent	Absent		Absent	Absent	Absent
11	45	Combination	Absent	Absent	Absent		Absent	Absent	Absent
11	46	SSG	Absent	Absent	Absent	Absent	Absent	Absent	Absent
11	47	SSG	Present	Present	Absent	Absent	Absent		
11	48	Combination	Absent	Absent	Absent		Absent		Absent
11	49	SSG	Present	Absent	Absent	Absent	Absent	Absent	Absent
11	50	PM	Absent	Absent	Absent		Absent	Absent	Absent
11	51	Combination	Present	Present	Absent		Absent	Absent	Absent
11	52	PM	Absent	Absent	Absent		Absent	Absent	Absent
11	53	SSG	Present	Present	Absent	Absent	Absent	Absent	Absent
11	54	PM	Present	Absent	Absent		Absent	Absent	Absent

Centre Number	Patient Number	Treatment	Baseline	Day7	Day 14	Muscle Wasting Day 21	EOT	3 mon FU	6 mon FU
11	55	PM	Absent	Absent	Absent		Absent	Absent	Absent
11	56	SSG	Present	Present	Present	Absent	Absent	Absent	Absent
11	57	Combination	Present	Present	Absent		Absent	Absent	Absent
11	58	Combination	Absent	Absent	Absent		Absent	Absent	Absent
11	59	PM	Present	Absent	Absent		Absent	Absent	Absent
11	60	Combination	Present	Present	Present		Present	Absent	Absent
11	61	PM	Absent	Absent	Absent		Absent	Absent	Absent
11	62	SSG	Present	Present	Present	Present	Absent	Absent	Absent
11	63	SSG	Absent	Absent	Absent	Absent	Absent	Absent	Absent
11	64	SSG	Absent	Absent	Absent	Absent	Absent	Absent	Absent
11	65	PM	Absent	Absent	Absent		Absent		
11	66	Combination	Present	Present	Present		Present	Absent	Absent
11	67	PM	Present	Present	Present		Absent	Absent	Absent
11	68	SSG	Absent	Absent	Absent	Absent	Absent	Absent	Absent
11	69	Combination	Absent	Absent	Absent		Absent	Absent	Absent
11	70	Combination	Absent	Absent	Absent		Absent	Absent	Absent
11	71	Combination	Absent	Absent	Absent		Absent	Absent	Present
11	72	SSG	Absent	Absent	Absent			Absent	Absent
11	73	PM	Absent	Absent	Absent		Absent	Absent	Absent
11	74	PM	Absent	Absent	Absent		Absent	Absent	Absent
11	75	Combination	Absent	Absent	Absent		Absent	Absent	Absent
11	76	PM	Absent	Absent	Absent		Absent	Absent	Absent
11	77	PM	Absent	Absent	Absent		Absent	Absent	Absent
11	78	SSG	Present	Present	Absent	Absent	Absent	Absent	Absent
11	79	PM	Present	Present	Present		Absent	Absent	Absent
11	80	PM	Absent	Absent	Absent		Absent	Absent	Absent
11	81	Combination	Absent	Absent	Absent		Absent	Absent	Absent
11	82	SSG	Absent	Absent	Absent	Absent	Absent	Absent	Absent
11	83	SSG	Present	Present	Present	Present	Present	Absent	Absent
11	84	SSG	Present	Present	Present	Present	Present	Absent	Absent
11	85	SSG	Present	Present	Present	Present	Present	Absent	
11	86	Combination	Present	Present	Present		Present	Absent	Absent
11	87	Combination	Absent	Absent	Absent		Absent	Absent	Absent
11	88	PM	Absent	Absent	Absent		Absent	Absent	Absent
11	89	Combination	Absent	Absent	Absent		Absent	Absent	Absent
11	90	Combination	Present	Present	Present		Present		
11	91	PM	Absent	Absent	Absent		Absent	Absent	Absent
11	92	Combination	Present	Absent	Absent		Absent	Absent	Absent
11	93	SSG	Absent	Absent	Absent	Absent	Absent	Absent	Absent
11	94	SSG	Absent	Absent	Absent	Absent	Absent	Absent	Absent
11	95	SSG	Absent	Absent	Absent	Absent	Absent	Absent	Absent
11	96	Combination	Absent	Absent	Absent		Absent	Absent	Absent
11	97	Combination	Present	Present	Absent		Absent	Absent	Absent
11	98	PM	Present	Present	Present		Present	Absent	Absent
11	99	SSG	Absent	Absent	Absent	Absent	Absent	Absent	Absent
11	100	PM	Absent	Absent	Absent		Absent		
11	101	PM	Absent	Absent	Absent		Absent	Absent	Absent
11	102	Combination	Absent	Present	Present		Absent	Absent	Absent
11	103	Combination	Absent	Absent	Absent		Absent	Absent	Absent
11	104	PM	Absent	Absent	Absent		Absent	Absent	Absent
11	105	SSG	Present	Present	Present	Present	Absent	Absent	Absent
11	106	Combination	Present	Present	Present		Absent	Absent	Absent
11	107	PM	Present	Absent	Absent		Absent	Absent	Absent
11	108	PM	Absent	Absent	Absent		Absent	Absent	Absent

Centre Number	Patient Number	Treatment	Muscle Wasting Baseline	Day7	Day 14	Day 21	EOT	3 mon FU	6 mon FU
11	109	SSG	Absent	Absent	Absent	Absent	Absent	Absent	Absent
11	110	PM	Absent	Absent	Absent		Absent	Absent	Absent
11	111	Combination	Absent	Absent	Absent		Absent	Absent	Absent
11	112	Combination	Absent	Absent	Absent		Absent	Absent	Absent
11	113	SSG	Absent	Absent	Absent	Absent	Absent	Absent	
11	114	SSG	Absent	Absent	Absent	Absent	Absent	Absent	Absent
11	115	Combination	Absent	Absent	Absent		Absent	Absent	Absent
11	116	SSG	Absent	Absent	Absent	Absent	Absent	Absent	Absent
11	117	PM	Absent	Absent	Absent		Absent	Absent	
11	118	PM	Absent	Absent	Absent		Absent	Absent	Absent
11	119	SSG	Absent	Absent	Absent	Absent	Absent	Absent	Absent
11	120	Combination	Absent	Absent	Absent		Absent	Absent	Absent
11	121	Combination	Absent	Absent	Absent		Absent	Absent	Absent
11	122	SSG	Absent	Absent	Absent	Absent	Absent		
11	123	SSG	Absent	Absent	Absent	Absent	Absent	Absent	Absent
11	124	SSG	Absent	Absent	Absent	Absent	Absent		Absent
11	125	SSG	Present	Absent	Absent	Absent	Absent	Absent	Absent
11	126	Combination	Absent	Absent	Absent		Absent		Absent
11	127	PM	Present	Present	Absent		Absent	Absent	Absent
11	128	Combination	Absent	Absent	Absent		Absent	Absent	Absent
11	129	PM	Absent	Absent	Absent		Absent	Absent	Absent
11	130	Combination	Present	Absent	Absent		Absent	Absent	Absent
11	131	PM	Absent	Absent	Absent		Absent	Absent	Absent
11	132	SSG	Absent	Absent	Absent	Absent	Absent	Absent	Absent
11	133	Combination	Absent	Absent	Absent		Absent	Absent	Absent
11	134	PM	Absent	Absent	Absent		Absent	Absent	Absent
11	135	PM	Absent	Absent	Absent		Absent	Absent	Absent
12	241	Combination	Present	Present	Absent		Absent		Absent
12	242	SSG	Present	Present	Absent	Absent	Absent	Absent	Absent
12	243	PM	Present	Present	Present		Present	Absent	Absent
12	244	SSG	Absent	Absent	Absent	Absent	Absent	Absent	Absent
12	245	PM	Present	Present	Present		Present	Present	
12	246	PM	Present	Present	Present		Present	Absent	
12	247	PM	Present	Present	Absent		Absent	Absent	Absent
12	248	PM	Absent	Absent	Absent		Absent	Absent	Absent
12	249	Combination	Present	Present	Present		Present	Present	Absent
12	250	SSG	Present	Present	Present	Present	Present	Absent	Absent
12	251	Combination	Absent	Absent	Absent		Absent	Absent	Absent
12	252	SSG	Present	Absent	Absent	Absent	Absent	Absent	Absent
12	253	SSG	Present	Present	Absent	Absent	Absent	Absent	Absent
12	254	Combination	Present	Absent	Present		Absent	Absent	Absent
12	255	Combination	Present	Present	Present		Present	Absent	Absent
12	256	Combination	Present	Present	Present		Present	Absent	Absent
12	257	SSG	Absent	Absent	Absent	Absent	Absent	Absent	Absent
12	258	SSG	Absent	Absent	Absent	Absent	Absent		Absent
12	259	PM	Absent	Absent	Absent		Absent	Absent	Absent
12	260	PM	Absent	Absent	Absent		Absent		Absent
12	261	Combination	Absent	Present	Present		Absent	Absent	Absent
12	262	PM	Absent	Absent	Absent		Absent	Absent	Absent
12	263	SSG	Absent	Absent	Absent	Absent	Absent	Absent	Absent
12	264	Combination	Absent	Absent	Absent		Absent	Absent	Absent
12	265	SSG	Absent	Absent	Absent	Absent	Absent		
12	266	PM	Present	Absent	Absent		Absent	Absent	Absent
12	267	SSG	Absent	Absent	Absent	Absent	Absent	Absent	Absent

Centre Number	Patient Number	Treatment	Baseline	Day7	Day 14	Muscle Wasting Day 21	EOT	3 mon FU	6 mon FU
12	268	PM	Absent	Absent	Absent		Absent	Absent	Absent
12	269	Combination	Absent	Absent	Absent		Absent	Absent	Absent
12	270	Combination	Absent	Absent	Absent		Absent		Absent
12	271	SSG	Absent	Absent	Absent	Absent	Absent	Absent	Absent
12	272	PM	Absent	Absent	Absent		Absent	Absent	Absent
12	273	SSG	Absent	Absent	Absent	Absent	Absent	Absent	Absent
12	274	SSG	Present	Present	Absent	Absent	Absent	Absent	Absent
12	275	Combination	Absent	Absent	Absent		Absent	Absent	Absent
12	276	PM	Present	Present	Present		Absent	Absent	Absent
12	277	Combination	Absent	Absent	Absent		Absent	Absent	Absent
12	278	SSG	Absent	Absent	Absent	Absent	Absent	Absent	
12	279	PM	Absent	Absent	Absent		Absent	Absent	Absent
12	280	SSG	Absent	Absent	Absent	Absent	Absent	Absent	Absent
12	281	Combination	Absent	Absent	Absent		Absent	Absent	Absent
12	282	PM	Absent	Absent	Absent		Absent	Absent	Absent
12	283	PM	Absent	Absent	Absent		Absent	Absent	Absent
12	284	Combination	Absent	Absent	Absent		Absent	Absent	Absent
12	285	Combination	Absent	Absent	Absent		Absent	Absent	Absent
12	286	PM	Present	Present	Present		Absent	Absent	Absent
12	287	SSG	Absent	Absent	Absent	Absent	Absent	Absent	Absent
12	288	Combination	Absent	Absent	Absent		Absent	Absent	Absent
12	289	Combination	Absent	Absent	Absent		Absent	Absent	Absent
12	290	SSG	Absent	Absent	Absent	Absent	Absent		Absent
12	291	SSG	Absent	Absent	Absent	Absent	Absent	Absent	Absent
12	292	PM	Absent	Absent	Absent		Absent		Absent
12	293	SSG	Absent	Absent	Absent	Absent	Absent		Absent
12	294	Combination	Present	Present	Absent		Absent	Absent	Absent
12	295	Combination	Absent	Absent	Absent		Absent		Absent
12	296	PM	Absent	Absent	Absent		Absent	Absent	Absent
12	297	SSG	Absent	Absent	Absent				
12	298	PM	Absent	Absent	Absent		Absent	Absent	Absent
12	299	Combination	Absent	Absent	Absent		Absent		Absent
12	300	PM	Absent	Absent	Absent		Absent	Absent	Absent
12	301	SSG	Absent	Absent	Absent	Absent	Absent	Absent	Absent
12	302	SSG	Absent	Absent	Absent	Absent	Absent	Absent	Absent
12	303	PM	Absent	Absent	Absent		Absent	Absent	Absent
12	304	Combination	Absent	Absent	Absent		Absent	Absent	Absent
12	305	PM	Absent	Absent	Absent		Absent	Absent	Absent
12	306	Combination	Absent	Absent	Absent		Absent	Absent	Absent
12	307	Combination	Absent	Absent	Absent		Absent	Absent	Absent
12	308	PM	Absent	Absent	Absent		Absent	Absent	Absent
12	309	PM	Absent	Absent	Absent		Absent	Absent	Absent
12	310	SSG	Absent	Absent	Absent	Absent	Absent	Absent	Absent
12	311	SSG	Absent	Absent	Absent	Absent	Absent	Absent	Absent
12	312	Combination	Absent	Absent	Absent		Absent	Absent	Absent
12	313	PM	Absent	Absent	Absent		Absent	Absent	Absent
12	314	SSG	Absent	Absent	Absent	Absent	Absent	Absent	Absent
12	315	Combination	Absent	Absent	Absent		Absent	Absent	Absent
12	316	SSG	Absent	Absent	Absent		Absent	Absent	Absent
12	317	SSG	Absent	Absent	Absent	Absent	Absent		Absent
12	318	Combination	Absent	Absent	Absent		Absent	Absent	Absent
12	319	Combination	Absent	Absent	Absent		Absent	Absent	Absent
12	320	Combination	Absent	Absent	Absent		Absent	Absent	Absent
12	321	Combination	Absent	Absent	Absent		Absent	Absent	Absent

Centre	Patient					Muscle Wasting			
Number	Number	Treatment	Baseline	Day7	Day 14	Day 21	EOT	3 mon FU	6 mon FU
12	322	PM	Absent	Absent	Absent		Absent	Absent	Absent
12	323	SSG	Absent	Absent	Absent	Absent	Absent	Absent	Absent
12	324	SSG	Absent	Absent	Absent	Absent	Absent	Absent	Absent
12	325	PM	Absent	Absent	Absent		Absent	Absent	Absent
12	326	PM	Absent	Absent	Absent		Absent	Absent	Absent
12	327	Combination	Absent	Absent	Absent		Absent	Absent	Absent
12	328	PM	Absent	Absent	Absent		Absent	Absent	Absent
12	329	SSG	Absent	Absent	Absent	Absent	Absent	Absent	Absent
12	330	PM	Absent	Absent	Absent		Absent	Absent	Absent
23	361	Combination	Absent	Absent	Absent		Absent	Absent	Absent
23	362	PM	Absent	Absent	Absent		Absent	Absent	Absent
23	363	PM	Absent	Absent	Absent		Absent	Absent	Absent
23	364	Combination	Absent	Absent	Absent		Absent	Absent	Absent
23	365	SSG	Absent	Absent	Absent	Absent	Absent	Absent	Absent
23	366	Combination	Absent	Absent	Absent		Absent	Absent	Absent
23	367	SSG	Absent	Absent	Absent	Absent	Absent	Absent	Absent
23	368	PM	Absent	Absent	Absent		Absent	Absent	Absent
23	369	Combination	Absent	Absent	Absent		Absent	Absent	Absent
23	370	SSG	Absent	Absent	Absent	Absent	Absent	Absent	Absent
23	371	PM	Absent	Absent	Absent		Absent	Absent	Absent
23	372	PM	Absent	Absent	Absent		Absent	Absent	Absent
23	373	SSG	Absent	Absent	Absent	Absent	Absent	Absent	Absent
23	374	SSG	Absent	Absent	Absent	Absent	Absent	Absent	Absent
23	375	Combination	Absent	Absent	Absent		Absent	Absent	Absent
23	376	SSG	Absent	Absent	Absent	Absent	Absent	Absent	Absent
23	377	Combination	Absent	Absent					Absent
23	378	Combination	Present	Present	Present		Present	Absent	Absent
23	379	PM	Absent	Absent	Absent		Absent	Absent	Absent
23	380	SSG	Absent	Absent	Absent	Absent	Absent	Absent	Absent
23	381	PM	Absent	Absent	Absent		Absent	Absent	Absent
23	382	Combination	Absent	Absent	Absent		Absent	Absent	Absent
23	383	PM	Absent	Absent	Absent		Absent	Absent	Absent
23	384	SSG	Absent	Absent	Absent	Absent	Absent	Absent	Absent
23	385	SSG	Absent	Absent	Absent	Absent	Absent	Absent	Absent
23	386	Combination	Absent	Absent	Absent		Absent	Absent	Absent
23	387	Combination	Absent	Absent	Absent		Absent	Absent	Absent
23	388	PM	Absent	Absent	Absent		Absent	Absent	Absent
23	389	SSG	Present	Absent	Absent	Absent	Absent	Absent	Absent
23	390	PM	Present	Present	Present		Absent	Absent	Absent
23	391	Combination	Absent	Absent	Absent		Absent	Absent	Absent
23	392	Combination	Absent	Absent	Absent			Absent	Absent
23	393	SSG	Absent	Absent	Absent	Absent	Absent	Absent	Absent
23	394	PM	Absent	Absent	Absent		Absent	Absent	Absent
23	395	PM	Present	Absent	Absent		Absent	Absent	Absent
23	396	SSG	Present	Present	Absent	Absent	Absent	Absent	Absent
23	397	SSG	Present	Absent	Absent	Absent	Absent	Absent	Absent
23	398	Combination	Present	Absent	Absent		Absent	Absent	Absent
23	399	PM	Present	Present	Present		Absent	Absent	Absent
23	400	PM	Present	Absent	Absent		Absent	Absent	Absent
23	401	Combination	Absent	Absent	Absent		Absent	Absent	Absent
23	402	Combination	Present	Present	Present		Absent	Absent	Absent
23	403	SSG	Absent	Absent	Absent	Absent	Absent	Absent	Absent
23	404	SSG	Absent	Absent	Absent	Absent	Absent		Absent
23	405	PM	Absent	Absent	Absent		Absent	Absent	Absent

Centre Number	Patient Number	Treatment	Baseline	Day7	Day 14	Muscle Wasting Day 21	EOT	3 mon FU	6 mon FU
34	451	Combination	Absent	Absent	Absent		Absent		Absent
34	452	PM	Present	Present	Absent		Absent		
34	453	PM	Absent	Absent	Absent		Absent		
34	454	Combination	Absent	Absent	Absent		Absent		Absent
34	455	PM	Absent	Absent	Absent		Absent		
34	456	SSG	Absent	Absent	Absent	Absent	Absent	Absent	
34	457	Combination	Absent	Absent	Absent		Absent		
34	458	PM	Present	Absent	Absent		Absent	Absent	
34	459	Combination	Present	Absent	Absent		Absent	Absent	Absent
34	460	PM	Present	Absent	Absent		Absent		
34	461	SSG	Present	Present	Present				
34	462	SSG	Present	Absent	Absent	Absent	Absent		Absent
34	463	Combination	Present	Present	Present		Absent		Absent
34	464	SSG	Present	Present	Present	Present	Present		Absent
34	465	SSG	Present	Absent	Absent	Absent	Absent		Absent
34	466	Combination	Absent	Absent	Absent		Absent		
34	467	PM	Absent	Absent	Absent		Absent	Absent	
34	468	Combination	Absent	Absent	Absent		Absent		Absent
34	469	SSG	Absent	Absent	Absent	Absent	Absent		Absent
34	470	Combination	Absent	Absent	Absent		Absent		Absent
34	471	PM	Present	Absent	Absent		Absent		
34	472	SSG	Absent	Absent	Absent	Absent	Absent		Absent
34	473	PM	Absent	Absent	Absent		Absent		
34	474	Combination	Absent	Absent	Absent		Absent		Absent
34	475	PM	Present	Present	Present		Present		
34	476	PM	Absent	Absent	Absent		Absent	Absent	
34	477	SSG	Absent	Absent	Absent	Absent	Absent		Absent
34	478	Combination	Absent	Absent	Absent		Absent		Absent
34	479	SSG	Present	Present	Present	Absent	Absent		Absent
34	480	SSG	Present	Present	Absent	Absent	Absent		Absent
34	481	Combination	Present	Present	Absent		Absent		Absent
34	482	Combination	Absent	Absent	Absent		Absent		Absent
34	483	PM	Absent	Absent	Absent		Absent		Absent
34	484	PM	Absent	Absent					
34	485	Combination	Absent	Absent	Absent		Absent		Absent
34	486	Combination	Present	Absent	Absent		Absent		Absent
34	487	SSG	Absent	Absent	Absent	Absent	Absent		Absent
34	488	SSG	Present	Absent	Absent	Absent	Absent	Absent	Absent
34	489	SSG	Absent	Absent	Absent	Absent	Absent		Absent
34	490	PM	Present	Present	Present		Present		Absent
34	491	PM	Absent	Absent	Absent		Absent		
34	492	PM	Absent	Absent	Absent		Absent		Absent
34	493	SSG	Present	Absent	Absent	Absent	Absent	Absent	
34	494	Combination	Present	Present	Present		Present		Absent
34	495	SSG	Present	Absent	Absent	Absent	Absent		Absent
34	496	SSG	Absent	Absent	Absent	Absent	Absent		Absent
34	497	PM	Absent	Absent	Absent		Absent	Absent	
34	498	SSG	Absent	Absent	Absent	Absent	Absent		Absent
34	499	Combination	Absent	Absent	Absent		Absent		Absent
34	500	PM	Absent	Absent	Absent		Absent		Absent
34	501	Combination	Absent	Absent	Absent		Absent		Absent
34	502	Combination	Absent	Absent	Absent		Absent		Absent
34	503	PM	Absent	Absent	Absent		Absent		
34	504	Combination	Absent	Absent	Absent		Absent		

Centre Number	Patient Number	Treatment	Muscle Wasting Baseline	Day7	Day 14	Day 21	EOT	3 mon FU	6 mon FU
34	505	SSG	Absent	Absent	Absent	Absent	Absent		Absent
34	506	SSG	Absent	Absent	Absent	Absent	Absent		Absent
34	507	SSG	Absent	Absent	Absent	Absent	Absent		Absent
34	508	PM	Absent	Absent	Absent		Absent		
34	509	PM	Present	Absent	Absent		Absent		
34	510	Combination	Absent	Absent	Absent		Absent		Absent
34	511	Combination	Present	Present	Absent		Absent		Absent
34	512	PM	Present	Present	Present		Absent		
34	513	Combination	Absent	Absent	Absent		Absent	Absent	Absent
34	514	SSG	Absent	Absent	Absent	Absent	Absent		Absent
34	515	SSG	Present	Absent	Absent	Absent	Absent	Absent	
34	516	PM	Absent	Absent	Absent		Absent		Absent
34	517	PM	Absent	Absent	Absent		Absent		
34	518	SSG	Absent	Absent	Absent	Absent	Absent		Absent
34	519	PM	Absent	Absent	Absent		Absent	Absent	
34	520	SSG	Absent	Absent	Absent	Absent	Absent		Absent
34	521	PM	Present	Present	Present		Absent		
34	522	Combination	Present	Absent	Absent		Absent		
34	523	SSG	Absent	Absent	Absent	Absent	Absent		Absent
34	524	Combination	Absent	Absent	Absent		Absent		Absent
34	525	Combination	Absent	Absent	Absent		Absent		Absent
34	526	SSG	Absent	Absent	Absent	Absent	Absent		Present
34	527	Combination	Present	Absent	Absent		Absent		Absent
34	528	PM	Absent	Absent	Absent		Absent		
34	529	Combination	Present	Absent	Absent		Absent		Absent
34	530	Combination	Absent	Absent	Absent		Absent		Absent
34	531	SSG	Absent	Absent	Absent	Absent	Absent		
34	532	Combination	Absent	Absent	Absent		Absent		Absent
34	533	SSG	Absent	Absent	Absent	Absent	Absent		Absent
34	534	PM	Absent	Absent	Absent		Present		
34	535	PM	Absent	Absent	Absent		Absent	Absent	
34	536	PM	Absent	Absent	Absent		Absent		Absent
34	537	SSG	Present	Present	Absent	Absent	Absent		Absent
34	538	PM	Absent	Absent	Absent		Absent		
34	539	Combination	Absent	Absent	Absent		Absent		Absent
34	540	SSG	Absent	Absent	Absent	Absent	Absent		Absent
35	646	PM	Present	Present	Present		Present		Absent
35	647	PM	Absent	Absent	Absent		Absent	Absent	Absent
35	648	Combination	Absent	Absent	Absent		Absent	Absent	Absent
35	649	Combination	Present	Present	Absent		Absent	Absent	Absent
35	650	SSG	Present	Present	Present	Absent	Absent	Absent	Absent
35	651	SSG	Present	Present	Absent	Absent	Absent	Absent	Absent
35	652	PM	Present	Present	Present		Present		Absent
35	653	Combination	Present	Present	Present		Present	Absent	Absent
35	654	SSG	Present	Present	Present	Present	Absent	Absent	Absent
35	655	Combination	Present	Present	Present		Present	Absent	Absent
35	656	SSG	Absent	Absent	Absent	Absent	Absent	Absent	Absent
35	657	PM	Present	Present	Present		Present	Absent	Absent
35	658	Combination	Absent	Absent	Absent		Absent	Absent	Absent
35	659	SSG	Present	Present	Present	Absent	Absent	Absent	Absent
35	660	PM	Present	Present	Present		Present	Absent	Absent
35	661	Combination	Present	Present	Absent		Absent	Absent	Absent
35	662	PM	Absent	Absent	Absent		Absent	Absent	Absent
35	663	SSG	Present	Present	Present	Absent	Absent	Absent	Absent

Centre	Patient					Muscle Wasting			
Number	Number	Treatment	Baseline	Day7	Day 14	Day 21	EOT	3 mon FU	6 mon FU
35	664	Combination	Absent	Absent					Absent
35	665	SSG	Present	Present	Present	Absent	Absent	Absent	Absent
35	666	SSG	Absent	Absent	Absent	Absent	Absent	Absent	Absent
35	667	Combination	Absent	Absent	Absent		Absent	Absent	Absent
35	668	PM	Absent	Absent	Absent		Absent		Absent
35	669	PM	Absent	Absent	Absent		Absent		
35	670	SSG	Present	Present	Present	Absent	Absent	Absent	Absent
35	671	PM	Present	Present	Absent		Absent		Absent
35	672	Combination	Present	Present	Present		Present		Absent
35	673	SSG	Present	Present	Absent	Absent	Absent	Absent	Absent
35	674	Combination	Present	Present	Absent		Absent	Absent	Absent
35	675	PM	Absent	Absent	Absent		Absent		Absent
35	676	PM	Present	Present	Present		Present	Absent	Absent
35	677	SSG	Absent	Present					
35	678	Combination	Present	Present	Present		Present	Absent	Absent
35	679	SSG	Present	Present	Present	Present	Present	Absent	Absent
35	680	Combination	Present	Present	Present		Present	Absent	Absent
35	681	Combination	Present	Present	Present		Present	Absent	Absent
35	682	PM	Present	Present	Present		Present		Absent
35	683	Combination	Absent	Absent	Absent		Absent		Absent
35	684	SSG	Absent	Absent	Absent	Absent	Absent	Absent	Absent
35	685	PM	Present	Present	Present		Absent	Absent	Absent
35	686	PM	Present	Present	Present		Absent	Absent	Absent
35	687	SSG	Absent	Absent	Absent	Absent	Absent	Absent	Absent
35	688	PM	Present	Present	Present		Present	Absent	Absent
35	689	Combination	Present	Present	Present		Present	Absent	Absent
35	690	SSG	Present	Present	Present	Absent	Absent	Absent	Absent

Appendix 42: Individual Listing of clinical characteristic, Mucosal pallor and Jaundice

Centre number	Patient number	Treatment	Mucosal Pallor Baseline	Day7	Day 14	Day 21	EOT	3 mon FU	6 mon FU
11	1	PM	Present	Absent	Absent		Absent	Absent	Absent
11	2	Combination	Absent	Absent	Absent		Absent	Absent	Absent
11	3	Combination	Present	Present	Absent		Absent	Absent	Absent
11	4	PM	Present	Present	Absent		Absent	Absent	Absent
11	5	PM	Absent	Absent	Absent		Absent	Absent	Absent
11	6	Combination	Present	Present	Present		Present	Present	Absent
11	7	PM	Absent	Absent	Absent		Absent	Absent	Absent
11	8	SSG	Present	Present	Present	Absent	Absent	Absent	Absent
11	9	SSG	Absent	Present	Absent	Absent	Absent	Absent	Absent
11	10	SSG	Absent	Absent	Absent	Absent	Absent	Present	Absent
11	11	Combination	Absent	Absent	Absent		Absent	Absent	Absent
11	12	Combination	Absent	Absent	Absent		Absent	Absent	Absent
11	13	SSG	Present	Present	Present	Present	Absent	Present	Absent
11	14	SSG	Absent	Absent	Absent	Absent	Absent	Absent	Absent
11	15	PM	Present	Present				Absent	Absent
11	16	Combination	Absent	Absent	Absent		Absent	Absent	Absent
11	17	Combination	Present	Absent	Absent		Absent	Absent	Absent
11	18	SSG	Absent	Present	Present	Absent	Absent	Absent	Absent
11	19	Combination	Present	Present	Present		Present	Absent	Absent
11	20	SSG	Present	Present	Present	Present	Present	Absent	Absent
11	21	SSG	Present	Present	Present	Present	Present	Absent	Absent
11	22	SSG	Present	Present	Present	Present	Present	Absent	Absent
11	23	PM	Absent	Absent	Absent		Absent	Absent	Absent
11	24	PM	Absent	Absent	Absent		Absent	Absent	Absent
11	25	SSG	Absent	Present	Present	Absent	Absent	Absent	Absent
11	26	Combination	Absent	Absent	Absent		Absent	Absent	Absent
11	27	PM	Present	Present	Present		Present	Present	
11	28	Combination	Present	Present	Absent		Absent	Absent	Absent
11	29	PM	Present	Present	Present		Absent	Absent	Absent
11	30	PM	Present	Present	Absent		Absent	Absent	Absent
11	31	SSG	Present	Present	Present	Absent	Absent	Absent	Absent
11	32	Combination	Absent	Present	Absent		Absent	Absent	Absent
11	33	SSG	Present	Present	Absent	Absent	Absent	Absent	Absent
11	34	PM	Present	Present	Absent		Absent	Absent	Absent
11	35	SSG	Absent	Absent	Absent	Absent	Absent	Absent	Absent
11	36	SSG	Present	Present	Present	Present	Absent	Absent	Absent
11	37	Combination	Present	Present	Absent		Absent	Absent	Absent
11	38	PM	Absent	Absent	Present		Absent	Absent	Absent
11	39	PM	Present	Present	Absent		Absent	Absent	Absent
11	40	PM	Present	Present	Present			Absent	Absent
11	41	Combination	Present	Absent	Absent		Absent	Absent	Absent
11	42	Combination	Absent	Absent	Absent		Absent	Absent	Absent
11	43	SSG	Present	Present	Present	Present	Present	Absent	Absent
11	44	PM	Absent	Absent	Absent		Absent	Absent	Absent
11	45	Combination	Absent	Absent	Absent		Absent	Absent	Absent
11	46	SSG	Absent	Absent	Absent	Absent	Absent	Absent	Absent
11	47	SSG	Present	Present	Present	Absent	Absent		
11	48	Combination	Present	Present	Absent		Absent		Absent
11	49	SSG	Present	Present	Absent	Absent	Absent	Absent	Absent
11	50	PM	Present	Absent	Absent		Absent	Absent	Absent
11	51	Combination	Present	Present	Absent		Absent	Absent	Absent
11	52	PM	Present	Present	Present		Present	Absent	Absent
11	53	SSG	Present	Present	Present	Present	Absent	Absent	Absent

Centre number	Patient number	Treatment	Mucosal Pallor Baseline	Day7	Day 14	Day 21	EOT	3 mon FU	6 mon FU
11	54	PM	Absent	Absent	Absent		Absent	Absent	Absent
11	55	PM	Present	Present	Present		Present	Absent	Absent
11	56	SSG	Present	Present	Absent	Present	Present	Absent	Absent
11	57	Combination	Present	Present	Present		Present	Absent	Absent
11	58	Combination	Present	Present	Present		Present	Absent	Absent
11	59	PM	Present	Present	Present		Absent	Absent	Absent
11	60	Combination	Present	Present	Present		Present	Absent	Absent
11	61	PM	Present	Present	Present		Present	Absent	Absent
11	62	SSG	Present	Present	Present	Present	Present	Absent	Absent
11	63	SSG	Present	Absent	Absent	Absent	Absent	Absent	Absent
11	64	SSG	Present	Present	Present	Present	Present	Absent	Absent
11	65	PM	Present	Present	Present		Present		
11	66	Combination	Present	Present	Present		Present	Absent	Absent
11	67	PM	Present	Present	Present		Absent	Absent	Absent
11	68	SSG	Present	Present	Present	Present	Absent	Absent	Absent
11	69	Combination	Present	Present	Present		Present	Absent	Absent
11	70	Combination	Present	Present	Present		Present	Absent	Absent
11	71	Combination	Present	Present	Present		Present	Present	Absent
11	72	SSG	Present	Present	Present			Absent	Absent
11	73	PM	Present	Present	Absent		Absent	Absent	Absent
11	74	PM	Absent	Absent	Absent		Absent	Absent	Absent
11	75	Combination	Present	Present	Present		Present	Absent	Absent
11	76	PM	Present	Present	Absent		Absent	Absent	Absent
11	77	PM	Present	Present	Present		Absent	Absent	Absent
11	78	SSG	Present	Present	Present	Absent	Absent	Absent	Absent
11	79	PM	Present	Present	Present		Present	Absent	Absent
11	80	PM	Present	Present	Absent		Absent	Absent	Absent
11	81	Combination	Present	Present	Present		Present	Absent	Absent
11	82	SSG	Present	Present	Present	Present	Absent	Absent	Absent
11	83	SSG	Present	Present	Present	Present	Absent	Absent	Absent
11	84	SSG	Present	Present	Present	Absent	Absent	Absent	Absent
11	85	SSG	Present	Present	Present	Absent	Absent	Absent	
11	86	Combination	Present	Present	Absent		Absent	Absent	Absent
11	87	Combination	Present	Present	Absent		Absent	Absent	Absent
11	88	PM	Present	Absent	Absent		Absent	Absent	Absent
11	89	Combination	Present	Present	Present		Absent	Absent	Absent
11	90	Combination	Present	Absent	Absent		Absent		
11	91	PM	Present	Present	Present		Absent	Absent	Present
11	92	Combination	Present	Present	Present		Absent	Absent	Absent
11	93	SSG	Present	Present	Present	Absent	Absent	Absent	Absent
11	94	SSG	Present	Present	Absent	Absent	Absent	Absent	Absent
11	95	SSG	Absent	Absent	Absent	Absent	Absent	Absent	Absent
11	96	Combination	Present	Present	Present		Present	Absent	Absent
11	97	Combination	Present	Present	Present		Absent	Absent	Absent
11	98	PM	Present	Present	Present		Present	Absent	Absent
11	99	SSG	Present	Present	Present	Present	Absent	Absent	Absent
11	100	PM	Present	Present	Present		Present		
11	101	PM	Present	Present	Present		Absent	Absent	Absent
11	102	Combination	Present	Present	Present		Present	Absent	Absent
11	103	Combination	Present	Present	Absent		Absent	Absent	Absent
11	104	PM	Present	Present	Present		Present	Absent	Absent
11	105	SSG	Present	Present	Present	Present	Absent	Absent	Absent
11	106	Combination	Present	Present	Present		Present	Absent	Absent

Centre	Patient		Mucosal Pallor						
number	number	Treatment	Baseline	Day7	Day 14	Day 21	EOT	3 mon FU	6 mon FU
11	107	PM	Present	Present	Present		Absent	Absent	Absent
11	108	PM	Present	Present	Present		Absent	Absent	Absent
11	109	SSG	Present	Present	Present	Absent	Absent	Absent	Absent
11	110	PM	Present	Present	Present		Present	Absent	Absent
11	111	Combination	Present	Present	Absent		Absent	Absent	Absent
11	112	Combination	Present	Present	Present		Absent	Absent	Absent
11	113	SSG	Present	Present	Absent	Present	Absent	Absent	
11	114	SSG	Present	Present	Absent	Absent	Absent	Absent	Absent
11	115	Combination	Present	Present	Absent		Absent	Absent	Absent
11	116	SSG	Present	Present	Absent	Absent	Absent	Absent	Absent
11	117	PM	Present	Present	Absent		Absent	Absent	
11	118	PM	Present	Present	Absent		Absent	Absent	Absent
11	119	SSG	Present	Present	Present	Absent	Absent	Absent	Absent
11	120	Combination	Present	Present	Present		Absent	Absent	Absent
11	121	Combination	Present	Present	Present		Absent	Absent	Absent
11	122	SSG	Present	Present	Present	Absent	Absent		
11	123	SSG	Present	Present	Absent	Absent	Absent	Absent	Absent
11	124	SSG	Present	Present	Present	Present	Present		Absent
11	125	SSG	Present	Present	Absent	Absent	Absent	Absent	Absent
11	126	Combination	Present	Present	Present		Present		Absent
11	127	PM	Present	Present	Absent		Absent	Absent	Absent
11	128	Combination	Present	Present	Present		Present	Absent	Absent
11	129	PM	Present	Present	Present		Absent	Absent	Absent
11	130	Combination	Present	Present	Absent		Absent	Absent	Absent
11	131	PM	Present	Present	Absent		Absent	Absent	Absent
11	132	SSG	Present	Present	Absent	Absent	Absent	Absent	Absent
11	133	Combination	Present	Present	Present		Absent	Absent	Absent
11	134	PM	Present	Present	Present		Present	Absent	Absent
11	135	PM	Present	Present	Present		Present	Absent	Absent
12	241	Combination	Present	Present	Present		Present		Absent
12	242	SSG	Present	Present	Absent	Absent	Absent	Absent	Absent
12	243	PM	Present	Present	Absent		Absent	Absent	Absent
12	244	SSG	Absent	Absent	Absent	Absent	Absent	Absent	Absent
12	245	PM	Present	Present	Present		Present	Present	
12	246	PM	Present	Absent	Absent		Absent	Absent	
12	247	PM	Present	Present	Absent		Absent	Absent	Absent
12	248	PM	Present	Present	Present		Absent	Absent	Absent
12	249	Combination	Present	Present	Present		Present	Present	Absent
12	250	SSG	Present	Present	Present	Present	Absent	Absent	Absent
12	251	Combination	Present	Absent	Absent		Absent	Absent	Absent
12	252	SSG	Present	Absent	Absent	Absent	Absent	Absent	Absent
12	253	SSG	Present	Absent	Absent	Absent	Absent	Absent	Absent
12	254	Combination	Present	Present	Absent		Absent	Absent	Absent
12	255	Combination	Present	Present	Present		Absent	Absent	Absent
12	256	Combination	Present	Present	Present		Absent	Absent	Absent
12	257	SSG	Present	Absent	Absent	Absent	Absent	Absent	Absent
12	258	SSG	Present	Present	Present	Absent	Absent		Absent
12	259	PM	Absent	Absent	Absent		Absent	Absent	Absent
12	260	PM	Present	Present	Present		Absent		Absent
12	261	Combination	Present	Present	Present		Present	Absent	Absent
12	262	PM	Absent	Absent	Absent		Absent	Absent	Absent
12	263	SSG	Absent	Absent	Absent	Absent	Absent	Absent	Absent
12	264	Combination	Absent	Absent	Absent		Absent	Absent	Absent

Centre number	Patient number	Treatment	Mucosal Pallor Baseline	Day7	Day 14	Day 21	EOT	3 mon FU	6 mon FU
12	265	SSG	Absent	Absent	Absent	Present	Present		
12	266	PM	Absent	Absent	Present		Present	Absent	Absent
12	267	SSG	Absent	Absent	Absent	Absent	Absent	Absent	Absent
12	268	PM	Present	Absent	Absent		Absent	Absent	Absent
12	269	Combination	Absent	Present	Present		Absent	Absent	Absent
12	270	Combination	Absent	Present	Present		Present		Absent
12	271	SSG	Present	Present	Present	Present	Absent	Absent	Absent
12	272	PM	Absent	Present	Present		Present	Absent	Absent
12	273	SSG	Present	Present	Absent	Absent	Absent	Absent	Absent
12	274	SSG	Present	Present	Present	Absent	Absent	Absent	Absent
12	275	Combination	Present	Present	Present		Present	Absent	Absent
12	276	PM	Present	Present	Present		Absent	Absent	Absent
12	277	Combination	Present	Present	Absent		Absent	Absent	Absent
12	278	SSG	Present	Present	Absent	Absent	Absent	Absent	
12	279	PM	Present	Present	Absent		Absent	Absent	Absent
12	280	SSG	Present	Present	Absent	Absent	Absent	Absent	Absent
12	281	Combination	Absent	Absent	Absent		Absent	Absent	Absent
12	282	PM	Present	Absent	Absent		Absent	Absent	Absent
12	283	PM	Absent	Absent	Absent		Absent	Absent	Absent
12	284	Combination	Present	Present	Absent		Absent	Absent	Present
12	285	Combination	Present	Present	Absent		Absent	Absent	Absent
12	286	PM	Present	Present	Present		Absent	Absent	Absent
12	287	SSG	Present	Present	Absent	Absent	Absent	Absent	Absent
12	288	Combination	Present	Present	Present		Present	Absent	Absent
12	289	Combination	Absent	Absent	Absent		Absent	Absent	Absent
12	290	SSG	Present	Present	Present	Present	Present		Absent
12	291	SSG	Present	Absent	Absent	Absent	Absent	Absent	Absent
12	292	PM	Present	Present	Absent		Absent		Absent
12	293	SSG	Present	Present	Present	Absent	Absent		Absent
12	294	Combination	Present	Present	Present		Absent	Absent	Absent
12	295	Combination	Present	Absent	Absent		Absent		Absent
12	296	PM	Present	Absent	Absent		Absent	Absent	Absent
12	297	SSG	Present	Absent	Absent				
12	298	PM	Present	Present	Absent		Absent	Absent	Absent
12	299	Combination	Present	Present	Present		Absent		Absent
12	300	PM	Present	Present	Present		Absent	Absent	Absent
12	301	SSG	Present	Present	Present	Absent	Absent	Absent	Absent
12	302	SSG	Present	Present	Present	Absent	Absent	Absent	Absent
12	303	PM	Present	Present	Present		Present	Absent	Absent
12	304	Combination	Present	Present	Present		Absent	Absent	Absent
12	305	PM	Present	Present	Present		Present	Absent	Absent
12	306	Combination	Present	Present	Present		Absent	Absent	Absent
12	307	Combination	Absent	Absent	Absent		Absent	Absent	Absent
12	308	PM	Present	Present	Absent		Absent	Absent	Absent
12	309	PM	Present	Present	Absent		Absent	Absent	Absent
12	310	SSG	Present	Present	Absent	Absent	Absent	Absent	Absent
12	311	SSG	Present	Absent	Absent	Absent	Absent	Absent	Absent
12	312	Combination	Absent	Absent	Absent		Absent	Absent	Absent
12	313	PM	Present	Present	Absent		Absent	Absent	Absent
12	314	SSG	Present	Absent	Absent	Absent	Absent	Absent	Absent
12	315	Combination	Present	Present	Present		Absent	Absent	Absent
12	316	SSG	Absent	Present	Absent		Absent	Absent	Absent
12	317	SSG	Absent	Absent	Absent	Absent	Absent		Absent

Centre number	Patient number	Treatment	Mucosal Pallor Baseline	Day7	Day 14	Day 21	EOT	3 mon FU	6 mon FU
12	318	Combination	Present	Present	Absent		Absent	Absent	Absent
12	319	Combination	Present	Present	Absent		Absent	Absent	Absent
12	320	Combination	Present	Present	Absent		Absent	Absent	Absent
12	321	Combination	Present	Present	Absent		Absent	Absent	Absent
12	322	PM	Present	Absent	Absent		Absent	Absent	Absent
12	323	SSG	Present	Present	Absent	Absent	Absent	Absent	Absent
12	324	SSG	Present	Present	Present	Absent	Absent	Absent	Absent
12	325	PM	Absent	Absent	Absent		Absent	Absent	Absent
12	326	PM	Present	Absent	Absent		Absent	Absent	Absent
12	327	Combination	Absent	Absent	Absent		Absent	Absent	Absent
12	328	PM	Present	Present	Absent		Absent	Absent	Absent
12	329	SSG	Present	Absent	Present	Absent	Absent	Absent	Absent
12	330	PM	Absent	Absent	Absent		Absent	Absent	Absent
23	361	Combination	Present	Absent	Absent		Absent	Absent	Absent
23	362	PM	Present	Present	Absent		Absent	Absent	Absent
23	363	PM	Absent	Absent	Absent		Absent	Present	Absent
23	364	Combination	Present	Present	Absent		Absent	Present	Absent
23	365	SSG	Present	Present	Absent	Absent	Absent	Absent	Absent
23	366	Combination	Present	Present	Absent		Absent	Absent	Absent
23	367	SSG	Present	Present	Absent	Absent	Absent	Absent	Absent
23	368	PM	Present	Present	Present		Present	Absent	Absent
23	369	Combination	Absent	Absent	Absent		Absent	Absent	Absent
23	370	SSG	Present	Present	Present	Present	Absent	Absent	Absent
23	371	PM	Present	Present	Absent		Absent	Present	Absent
23	372	PM	Absent	Absent	Absent		Absent	Absent	Absent
23	373	SSG	Present	Present	Present	Absent	Absent	Absent	Absent
23	374	SSG	Present	Present	Present	Present	Absent	Absent	Absent
23	375	Combination	Present	Present	Present		Absent	Absent	Absent
23	376	SSG	Absent	Present	Present	Present	Absent	Absent	Absent
23	377	Combination	Present	Absent					Absent
23	378	Combination	Present	Present	Present		Absent	Present	Absent
23	379	PM	Present	Absent	Absent		Absent	Present	Absent
23	380	SSG	Present	Present	Present	Absent	Absent	Absent	Absent
23	381	PM	Present	Present	Present		Absent	Absent	Absent
23	382	Combination	Present	Absent	Absent		Absent	Absent	Absent
23	383	PM	Present	Present	Absent		Absent	Absent	Absent
23	384	SSG	Present	Present	Absent	Absent	Absent	Absent	Absent
23	385	SSG	Present	Present	Absent	Absent	Present	Present	Absent
23	386	Combination	Present	Present	Present		Absent	Absent	Absent
23	387	Combination	Present	Present	Present		Present	Absent	Absent
23	388	PM	Present	Present	Present		Present	Present	Absent
23	389	SSG	Present	Present	Present	Present	Present	Absent	Absent
23	390	PM	Present	Present	Present		Present	Absent	Absent
23	391	Combination	Present	Absent	Present		Present	Present	Absent
23	392	Combination	Present	Present	Present			Absent	Absent
23	393	SSG	Present	Present	Present	Absent	Present	Absent	Absent
23	394	PM	Present	Present	Present		Present	Absent	Absent
23	395	PM	Absent	Present	Absent		Absent	Absent	Absent
23	396	SSG	Present	Present	Absent	Absent	Absent	Absent	Absent
23	397	SSG	Present	Present	Present	Absent	Present	Absent	Absent
23	398	Combination	Present	Present	Present		Present	Absent	Absent
23	399	PM	Present	Present	Present		Present	Present	Absent
23	400	PM	Present	Absent	Absent		Present	Absent	Absent

Centre number	Patient number	Treatment	Mucosal Pallor Baseline	Day7	Day 14	Day 21	EOT	3 mon FU	6 mon FU
23	401	Combination	Present	Absent	Present		Absent	Present	Present
23	402	Combination	Present	Present	Absent		Present	Absent	Absent
23	403	SSG	Present	Present	Present	Present	Present	Absent	Absent
23	404	SSG	Present	Absent	Absent	Absent	Absent		Absent
23	405	PM	Present	Present	Present		Absent	Absent	Absent
34	451	Combination	Present	Present	Absent		Absent		Absent
34	452	PM	Present	Present	Present		Absent		
34	453	PM	Present	Present	Absent		Absent		
34	454	Combination	Present	Present	Present		Absent		Absent
34	455	PM	Absent	Absent	Absent		Absent		
34	456	SSG	Present	Present	Present	Absent	Absent	Absent	
34	457	Combination	Absent	Absent	Absent		Absent		
34	458	PM	Present	Present	Absent		Absent	Absent	
34	459	Combination	Present	Absent	Absent		Absent	Absent	Absent
34	460	PM	Absent	Absent	Absent		Absent		
34	461	SSG	Present	Present	Present				
34	462	SSG	Absent	Absent	Absent	Absent	Absent		Absent
34	463	Combination	Present	Present	Present		Absent		Absent
34	464	SSG	Present	Present	Present	Present	Absent		Absent
34	465	SSG	Absent	Absent	Absent	Absent	Absent		Absent
34	466	Combination	Absent	Absent	Absent		Absent		
34	467	PM	Absent	Absent	Absent		Absent	Absent	
34	468	Combination	Absent	Absent	Absent		Absent		Absent
34	469	SSG	Absent	Absent	Absent	Absent	Absent		Absent
34	470	Combination	Absent	Absent	Absent		Absent		Absent
34	471	PM	Absent	Absent	Absent		Absent		
34	472	SSG	Present	Present	Present	Absent	Absent		Absent
34	473	PM	Absent	Absent	Absent		Absent		
34	474	Combination	Absent	Absent	Absent		Absent		Absent
34	475	PM	Present	Present	Absent		Absent		
34	476	PM	Absent	Absent	Absent		Absent	Absent	
34	477	SSG	Absent	Absent	Absent	Absent	Absent		Absent
34	478	Combination	Present	Present	Absent		Absent		Absent
34	479	SSG	Present	Present	Absent	Absent	Absent		Absent
34	480	SSG	Present	Present	Absent	Absent	Absent		Absent
34	481	Combination	Present	Present	Absent		Absent		Absent
34	482	Combination	Absent	Absent	Absent		Absent		Absent
34	483	PM	Absent	Absent	Absent		Absent		Absent
34	484	PM	Absent	Present					
34	485	Combination	Present	Present	Present		Absent		Absent
34	486	Combination	Present	Absent	Absent		Absent		Absent
34	487	SSG	Absent	Absent	Absent	Absent	Absent		Absent
34	488	SSG	Present	Absent	Absent	Absent	Absent	Absent	Absent
34	489	SSG	Present	Present	Present	Absent	Absent		Absent
34	490	PM	Present	Present	Absent		Absent		Absent
34	491	PM	Present	Absent	Absent		Absent		
34	492	PM	Absent	Absent	Absent		Absent		Absent
34	493	SSG	Present	Absent	Absent	Absent	Absent	Absent	
34	494	Combination	Present	Present	Absent		Absent		Absent
34	495	SSG	Present	Absent	Absent	Absent	Absent		Absent
34	496	SSG	Absent	Absent	Absent	Absent	Absent		Absent
34	497	PM	Absent	Absent	Absent		Absent	Absent	
34	498	SSG	Absent	Absent	Absent	Absent	Absent		Absent

Centre number	Patient number	Treatment	Mucosal Pallor						
			Baseline	Day7	Day 14	Day 21	EOT	3 mon FU	6 mon FU
34	499	Combination	Absent	Absent	Absent		Absent		Absent
34	500	PM	Absent	Absent	Absent		Absent		Absent
34	501	Combination	Absent	Present	Absent		Absent		Absent
34	502	Combination	Absent	Absent	Absent		Absent		Absent
34	503	PM	Absent	Absent	Absent		Absent		
34	504	Combination	Absent	Absent	Absent		Absent		
34	505	SSG	Absent	Absent	Absent	Absent	Absent		Absent
34	506	SSG	Absent	Absent	Absent	Absent	Absent		Absent
34	507	SSG	Absent	Present	Present	Absent	Absent		Absent
34	508	PM	Present	Present	Absent		Absent		
34	509	PM	Absent	Absent	Absent		Absent		
34	510	Combination	Absent	Absent	Absent		Absent		Absent
34	511	Combination	Present	Present	Absent		Absent		Absent
34	512	PM	Present	Present	Absent		Absent		
34	513	Combination	Absent	Absent	Absent		Absent	Absent	Absent
34	514	SSG	Present	Absent	Absent	Absent	Absent		Absent
34	515	SSG	Absent	Absent	Absent	Absent	Absent	Absent	
34	516	PM	Absent	Absent	Absent		Absent		Absent
34	517	PM	Absent	Absent	Absent		Absent		
34	518	SSG	Absent	Absent	Absent	Absent	Absent		Absent
34	519	PM	Absent	Absent	Absent		Absent	Absent	
34	520	SSG	Absent	Present	Absent	Absent	Absent		Absent
34	521	PM	Present	Present	Present		Absent		
34	522	Combination	Present	Absent	Absent		Absent		
34	523	SSG	Present	Absent	Absent	Absent	Absent		Absent
34	524	Combination	Absent	Absent	Absent		Absent		Absent
34	525	Combination	Absent	Absent	Absent		Absent		Absent
34	526	SSG	Absent	Absent	Absent	Absent	Absent		Absent
34	527	Combination	Present	Present	Present		Absent		Absent
34	528	PM	Absent	Absent	Absent		Absent		
34	529	Combination	Present	Present	Absent		Absent		Absent
34	530	Combination	Absent	Present	Absent		Absent		Absent
34	531	SSG	Absent	Absent	Absent	Absent	Absent		
34	532	Combination	Absent	Absent	Absent		Absent		Absent
34	533	SSG	Absent	Absent	Absent	Absent	Absent		Absent
34	534	PM	Absent	Absent	Absent		Present		
34	535	PM	Absent	Absent	Absent		Absent	Absent	
34	536	PM	Absent	Absent	Absent		Absent		Absent
34	537	SSG	Absent	Absent	Absent	Absent	Absent		Absent
34	538	PM	Absent	Present	Absent		Absent		
34	539	Combination	Absent	Absent	Absent		Absent		Absent
34	540	SSG	Present	Absent	Absent	Absent	Absent		Absent
35	646	PM	Present	Present	Present		Present		Absent
35	647	PM	Absent	Absent	Absent		Absent	Absent	Absent
35	648	Combination	Absent	Absent	Absent		Absent	Absent	Absent
35	649	Combination	Present	Present	Absent		Absent	Absent	Absent
35	650	SSG	Present	Present	Absent	Absent	Absent	Absent	Absent
35	651	SSG	Present	Present	Absent	Absent	Absent	Absent	Absent
35	652	PM	Present	Present	Present		Present		Absent
35	653	Combination	Present	Present	Present		Present	Absent	Absent
35	654	SSG	Present	Present	Present	Present	Absent	Absent	Absent
35	655	Combination	Absent	Absent	Absent		Absent	Absent	Absent
35	656	SSG	Present	Present	Present	Present	Absent	Absent	Absent

Centre	Patient					Mucosal Pallor			
number	number	Treatment	Baseline	Day7	Day 14	Day 21	EOT	3 mon FU	6 mon FU
35	657	PM	Present	Present	Present		Absent	Absent	Absent
35	658	Combination	Absent	Absent	Absent		Absent	Absent	Absent
35	659	SSG	Present	Present	Present	Absent	Absent	Absent	Absent
35	660	PM	Absent	Absent	Absent		Absent	Absent	Absent
35	661	Combination	Absent	Absent	Absent		Absent	Absent	Absent
35	662	PM	Absent	Absent	Absent		Absent	Absent	Absent
35	663	SSG	Absent	Absent	Absent	Absent	Absent	Absent	Absent
35	664	Combination	Present	Present					Absent
35	665	SSG	Present	Present	Present	Absent	Absent	Absent	Absent
35	666	SSG	Present	Present	Present	Absent	Absent	Absent	Absent
35	667	Combination	Absent	Absent	Absent		Absent	Absent	Absent
35	668	PM	Present	Present	Present		Present		Absent
35	669	PM	Present	Present	Present		Absent		
35	670	SSG	Absent	Absent	Absent	Absent	Absent	Absent	Absent
35	671	PM	Present	Present	Present		Present		Absent
35	672	Combination	Present	Present	Absent		Absent		Absent
35	673	SSG	Present	Present	Absent	Absent	Absent	Absent	Absent
35	674	Combination	Present	Present	Present		Present	Absent	Absent
35	675	PM	Present	Present	Absent		Absent		Absent
35	676	PM	Present	Present	Present		Present	Absent	Absent
35	677	SSG	Present	Present					
35	678	Combination	Present	Present	Present		Present	Absent	Absent
35	679	SSG	Present	Present	Present	Present	Absent	Absent	Absent
35	680	Combination	Present	Present	Present		Present	Absent	Absent
35	681	Combination	Present	Present	Present		Present	Absent	Absent
35	682	PM	Present	Present	Present		Present		Absent
35	683	Combination	Absent	Absent	Absent		Absent		Absent
35	684	SSG	Present	Present	Present	Absent	Absent	Absent	Absent
35	685	PM	Present	Present	Present		Absent	Absent	Absent
35	686	PM	Present	Present	Present		Absent	Absent	Absent
35	687	SSG	Absent	Absent	Present	Absent	Absent	Absent	Absent
35	688	PM	Present	Present	Present		Present	Absent	Absent
35	689	Combination	Present	Present	Present		Absent	Absent	Absent
35	690	SSG	Present	Present	Present	Absent	Absent	Absent	Absent

Centre number	Patient number	Treatment	Baseline	Day7	Day 14	Jaundice<br>Day 21	EOT	3 mon FU	6 mon FU
11	1	PM	Absent	Absent	Absent		Absent	Absent	Absent
11	2	Combination	Absent	Absent	Absent		Absent	Absent	Absent
11	3	Combination	Absent	Absent	Absent		Absent	Absent	Absent
11	4	PM	Absent	Absent	Absent		Absent	Absent	Absent
11	5	PM	Absent	Absent	Absent		Absent	Absent	Absent
11	6	Combination	Absent	Absent	Absent		Absent	Absent	Absent
11	7	PM	Absent	Absent	Absent		Absent	Absent	Absent
11	8	SSG	Absent	Absent	Absent	Absent	Absent	Absent	Absent
11	9	SSG	Absent	Absent	Absent	Absent	Absent	Absent	Absent
11	10	SSG	Absent	Absent	Absent	Absent	Absent	Absent	Absent
11	11	Combination	Absent	Absent	Absent		Absent	Absent	Absent
11	12	Combination	Absent	Absent	Absent		Absent	Absent	Absent
11	13	SSG	Absent	Absent	Absent	Absent	Absent	Absent	Absent
11	14	SSG	Absent	Absent	Absent	Absent	Absent	Absent	Absent
11	15	PM	Absent	Present				Absent	Absent
11	16	Combination	Absent	Absent	Absent		Absent	Absent	Absent
11	17	Combination	Absent	Absent	Absent		Absent	Absent	Absent
11	18	SSG	Absent	Absent	Absent	Absent	Absent	Absent	Absent
11	19	Combination	Absent	Absent	Absent		Absent	Absent	Absent
11	20	SSG	Absent	Absent	Absent	Absent	Absent	Absent	Absent
11	21	SSG	Absent	Absent	Absent	Absent	Absent	Absent	Absent
11	22	SSG	Absent	Absent	Absent	Absent	Absent	Absent	Absent
11	23	PM	Absent	Absent	Absent		Absent	Absent	Absent
11	24	PM	Absent	Absent	Absent		Absent	Absent	Absent
11	25	SSG	Absent	Absent	Absent	Absent	Absent	Absent	Absent
11	26	Combination	Absent	Absent	Absent		Absent	Absent	Absent
11	27	PM	Absent	Absent	Absent		Absent	Absent	
11	28	Combination	Absent	Absent	Absent		Absent	Absent	Absent
11	29	PM	Absent	Absent	Absent		Absent	Absent	Absent
11	30	PM	Absent	Absent	Absent		Absent	Absent	Absent
11	31	SSG	Absent	Absent	Absent	Absent	Absent	Absent	Absent
11	32	Combination	Absent	Absent	Absent		Absent	Absent	Absent
11	33	SSG	Absent	Absent	Absent	Absent	Absent	Absent	Absent
11	34	PM	Absent	Absent	Absent		Absent	Absent	Absent
11	35	SSG	Absent	Absent	Absent	Absent	Absent	Absent	Absent
11	36	SSG	Absent	Absent	Absent	Absent	Absent	Absent	Absent
11	37	Combination	Absent	Absent	Absent		Absent	Absent	Absent
11	38	PM	Absent	Absent	Absent		Absent	Absent	Absent
11	39	PM	Absent	Absent	Absent		Absent	Absent	Absent
11	40	PM	Absent	Absent	Absent			Absent	Absent
11	41	Combination	Absent	Absent	Absent		Absent	Absent	Absent
11	42	Combination	Absent	Absent	Absent		Absent	Absent	Absent
11	43	SSG	Absent	Absent	Absent	Absent	Absent	Absent	Absent
11	44	PM	Absent	Absent	Absent		Absent	Absent	Absent
11	45	Combination	Absent	Absent	Absent		Absent	Absent	Absent
11	46	SSG	Absent	Absent	Absent	Absent	Absent	Absent	Absent
11	47	SSG	Absent	Absent	Absent	Absent	Absent		
11	48	Combination	Absent	Absent	Absent		Absent		Absent
11	49	SSG	Absent	Absent	Absent	Absent	Absent	Absent	Absent
11	50	PM	Absent	Absent	Absent		Absent	Absent	Absent
11	51	Combination	Absent	Absent	Absent		Absent	Absent	Absent
11	52	PM	Absent	Absent	Absent		Absent	Absent	Absent
11	53	SSG	Absent	Absent	Absent	Absent	Absent	Absent	Absent

Centre	Patient					Jaundice			
number	number	Treatment	Baseline	Day7	Day 14	Day 21	EOT	3 mon FU	6 mon FU
11	54	PM	Absent	Absent	Absent		Absent	Absent	Absent
11	55	PM	Absent	Absent	Absent		Absent	Absent	Absent
11	56	SSG	Absent	Absent	Absent	Absent	Absent	Absent	Absent
11	57	Combination	Absent	Absent	Absent		Absent	Absent	Absent
11	58	Combination	Absent	Absent	Absent		Absent	Absent	Absent
11	59	PM	Absent	Absent	Absent		Absent	Absent	Absent
11	60	Combination	Absent	Absent	Absent		Absent	Absent	Absent
11	61	PM	Absent	Absent	Absent		Absent	Absent	Absent
11	62	SSG	Absent	Absent	Absent	Absent	Absent	Absent	Absent
11	63	SSG	Absent	Absent	Absent	Absent	Absent	Absent	Absent
11	64	SSG	Absent	Absent	Absent	Absent	Absent	Absent	Absent
11	65	PM	Absent	Absent	Absent		Absent		
11	66	Combination	Absent	Absent	Absent		Absent	Absent	Absent
11	67	PM	Absent	Absent	Absent		Absent	Absent	Absent
11	68	SSG	Absent	Absent	Absent	Absent	Absent	Absent	Absent
11	69	Combination	Absent	Absent	Absent		Absent	Absent	Absent
11	70	Combination	Absent	Absent	Absent		Absent	Absent	Absent
11	71	Combination	Absent	Absent	Absent		Absent	Absent	Absent
11	72	SSG	Absent	Absent	Absent			Absent	Absent
11	73	PM	Absent	Absent	Absent		Absent	Absent	Absent
11	74	PM	Absent	Absent	Absent		Absent	Absent	Absent
11	75	Combination	Absent	Absent	Absent		Absent	Absent	Absent
11	76	PM	Absent	Absent	Absent		Absent	Absent	Absent
11	77	PM	Absent	Absent	Absent		Absent	Absent	Absent
11	78	SSG	Absent	Absent	Absent	Absent	Absent	Absent	Absent
11	79	PM	Absent	Absent	Absent		Absent	Absent	Absent
11	80	PM	Absent	Absent	Absent		Absent	Absent	Absent
11	81	Combination	Absent	Absent	Absent		Absent	Absent	Absent
11	82	SSG	Absent	Absent	Absent	Absent	Absent	Absent	Absent
11	83	SSG	Absent	Absent	Absent	Absent	Absent	Absent	Absent
11	84	SSG	Absent	Absent	Absent	Absent	Absent	Absent	Absent
11	85	SSG	Absent	Absent	Absent	Absent	Absent	Absent	
11	86	Combination	Absent	Absent	Absent		Absent	Absent	Absent
11	87	Combination	Absent	Absent	Absent		Absent	Absent	Absent
11	88	PM	Absent	Absent	Absent		Absent	Absent	Absent
11	89	Combination	Absent	Absent	Absent		Absent	Absent	Absent
11	90	Combination	Absent	Absent	Absent		Absent		
11	91	PM	Absent	Absent	Absent		Absent	Absent	Absent
11	92	Combination	Absent	Absent	Absent		Absent	Absent	Absent
11	93	SSG	Absent	Absent	Absent	Absent	Absent	Absent	Absent
11	94	SSG	Absent	Absent	Absent	Absent	Absent	Absent	Absent
11	95	SSG	Absent	Absent	Absent	Absent	Absent	Absent	Absent
11	96	Combination	Absent	Absent	Absent		Absent	Absent	Absent
11	97	Combination	Absent	Absent	Absent		Absent	Absent	Absent
11	98	PM	Absent	Absent	Absent		Absent	Absent	Absent
11	99	SSG	Absent	Absent	Absent	Absent	Absent	Absent	Absent
11	100	PM	Absent	Absent	Absent		Absent		
11	101	PM	Absent	Absent	Absent		Absent	Absent	Absent
11	102	Combination	Absent	Absent	Absent		Absent	Absent	Absent
11	103	Combination	Absent	Absent	Absent		Absent	Absent	Absent
11	104	PM	Absent	Absent	Absent		Absent	Absent	Absent
11	105	SSG	Absent	Absent	Absent	Absent	Absent	Absent	Absent
11	106	Combination	Absent	Absent	Absent		Absent	Absent	Absent

Centre number	Patient number	Treatment	Baseline	Day7	Day 14	Jaundice Day 21	EOT	3 mon FU	6 mon FU
11	107	PM	Absent	Absent	Absent		Absent	Absent	Absent
11	108	PM	Absent	Absent	Absent		Absent	Absent	Absent
11	109	SSG	Absent	Absent	Absent	Absent	Absent	Absent	Absent
11	110	PM	Absent	Absent	Absent		Absent	Absent	Absent
11	111	Combination	Absent	Absent	Absent		Absent	Absent	Absent
11	112	Combination	Absent	Absent	Absent		Absent	Absent	Absent
11	113	SSG	Absent	Absent	Absent	Absent	Absent	Absent	
11	114	SSG	Absent	Absent	Absent	Absent	Absent	Absent	Absent
11	115	Combination	Absent	Absent	Absent		Absent	Absent	Absent
11	116	SSG	Absent	Absent	Absent	Absent	Absent	Absent	Absent
11	117	PM	Absent	Absent	Absent		Absent	Absent	
11	118	PM	Absent	Absent	Absent		Absent	Absent	Absent
11	119	SSG	Absent	Absent	Absent	Absent	Absent	Absent	Absent
11	120	Combination	Absent	Absent	Absent		Absent	Absent	Absent
11	121	Combination	Absent	Absent	Absent		Absent	Absent	Absent
11	122	SSG	Absent	Absent	Absent	Absent	Absent		
11	123	SSG	Absent	Absent	Absent	Absent	Absent	Absent	Absent
11	124	SSG	Absent	Absent	Absent	Absent	Absent		Absent
11	125	SSG	Absent	Absent	Absent	Absent	Absent	Absent	Absent
11	126	Combination	Absent	Absent	Absent		Absent		Absent
11	127	PM	Absent	Absent	Absent		Absent	Absent	Absent
11	128	Combination	Absent	Absent	Absent		Absent	Absent	Absent
11	129	PM	Absent	Absent	Absent		Absent	Absent	Absent
11	130	Combination	Absent	Absent	Absent		Absent	Absent	Absent
11	131	PM	Absent	Absent	Absent		Absent	Absent	Absent
11	132	SSG	Absent	Absent	Absent	Absent	Absent	Absent	Absent
11	133	Combination	Absent	Absent	Absent		Absent	Absent	Absent
11	134	PM	Absent	Absent	Absent		Absent	Absent	Absent
11	135	PM	Absent	Absent	Absent		Absent	Absent	Absent
12	241	Combination	Absent	Absent	Absent		Absent		Absent
12	242	SSG	Absent	Absent	Absent	Absent	Absent	Absent	Absent
12	243	PM	Absent	Absent	Absent		Absent	Absent	Absent
12	244	SSG	Absent	Absent	Absent	Absent	Absent	Absent	Absent
12	245	PM	Absent	Absent	Absent		Absent	Absent	
12	246	PM	Absent	Absent	Absent		Absent	Absent	
12	247	PM	Absent	Absent	Absent		Absent	Absent	Absent
12	248	PM	Absent	Absent	Absent		Absent	Absent	Absent
12	249	Combination	Absent	Absent	Absent		Absent	Absent	Absent
12	250	SSG	Absent	Absent	Absent	Absent	Absent	Absent	Absent
12	251	Combination	Absent	Absent	Absent		Absent	Absent	Absent
12	252	SSG	Absent	Absent	Absent	Absent	Absent	Absent	Absent
12	253	SSG	Absent	Absent	Absent	Absent	Absent	Absent	Absent
12	254	Combination	Absent	Absent	Absent		Absent	Absent	Absent
12	255	Combination	Absent	Absent	Absent		Absent	Absent	Absent
12	256	Combination	Absent	Absent	Absent		Absent	Absent	Absent
12	257	SSG	Absent	Absent	Absent	Absent	Absent	Absent	Absent
12	258	SSG	Absent	Absent	Absent	Absent	Absent		Absent
12	259	PM	Absent	Absent	Absent		Absent	Absent	Absent
12	260	PM	Absent	Absent	Absent		Absent		Absent
12	261	Combination	Absent	Absent	Absent		Absent	Absent	Absent
12	262	PM	Absent	Absent	Absent		Absent	Absent	Absent
12	263	SSG	Absent	Absent	Absent	Absent	Absent	Absent	Absent
12	264	Combination	Absent	Absent	Absent		Absent	Absent	Absent

Centre number	Patient number	Treatment	Jaundice Baseline	Day7	Day 14	Day 21	EOT	3 mon FU	6 mon FU
12	265	SSG	Absent	Absent	Absent	Absent	Absent		
12	266	PM	Absent	Absent	Absent		Absent	Absent	Absent
12	267	SSG	Absent	Absent	Absent	Absent	Absent	Absent	Absent
12	268	PM	Absent	Absent	Absent		Absent	Absent	Absent
12	269	Combination	Absent	Absent	Absent		Absent	Absent	Absent
12	270	Combination	Absent	Absent	Absent		Absent		Absent
12	271	SSG	Absent	Absent	Absent	Absent	Absent	Absent	Absent
12	272	PM	Absent	Absent	Absent		Absent	Absent	Absent
12	273	SSG	Absent	Absent	Absent	Absent	Absent	Absent	Absent
12	274	SSG	Absent	Absent	Absent	Absent	Absent	Absent	Absent
12	275	Combination	Absent	Absent	Absent		Absent	Absent	Absent
12	276	PM	Absent	Absent	Absent		Absent	Absent	Absent
12	277	Combination	Absent	Absent	Absent		Absent	Absent	Absent
12	278	SSG	Absent	Absent	Absent	Absent	Absent	Absent	
12	279	PM	Absent	Absent	Absent		Absent	Absent	Absent
12	280	SSG	Absent	Absent	Absent	Absent	Absent	Absent	Absent
12	281	Combination	Absent	Absent	Absent		Absent	Absent	Absent
12	282	PM	Absent	Absent	Absent		Absent	Absent	Absent
12	283	PM	Absent	Absent	Absent		Absent	Absent	Absent
12	284	Combination	Absent	Absent	Absent		Absent	Absent	Absent
12	285	Combination	Absent	Absent	Absent		Absent	Absent	Absent
12	286	PM	Absent	Absent	Absent		Absent	Absent	Absent
12	287	SSG	Absent	Absent	Absent	Absent	Absent	Absent	Absent
12	288	Combination	Absent	Absent	Absent		Absent	Absent	Absent
12	289	Combination	Absent	Absent	Absent		Absent	Absent	Absent
12	290	SSG	Absent	Absent	Absent	Absent	Absent		Absent
12	291	SSG	Absent	Absent	Absent	Absent	Absent	Absent	Absent
12	292	PM	Absent	Absent	Absent		Absent		Absent
12	293	SSG	Absent	Absent	Absent	Absent	Absent		Absent
12	294	Combination	Absent	Absent	Absent		Absent	Absent	Absent
12	295	Combination	Absent	Absent	Absent		Absent		Absent
12	296	PM	Absent	Absent	Absent		Absent	Absent	Absent
12	297	SSG	Absent	Absent	Absent				
12	298	PM	Absent	Absent	Absent		Absent	Absent	Absent
12	299	Combination	Absent	Absent	Absent		Absent		Absent
12	300	PM	Absent	Absent	Absent		Absent	Absent	Absent
12	301	SSG	Absent	Absent	Absent	Absent	Absent	Absent	Absent
12	302	SSG	Absent	Absent	Absent	Absent	Absent	Absent	Absent
12	303	PM	Absent	Absent	Absent		Absent	Absent	Absent
12	304	Combination	Absent	Absent	Absent		Absent	Absent	Absent
12	305	PM	Absent	Absent	Absent		Absent	Absent	Absent
12	306	Combination	Absent	Absent	Absent		Absent	Absent	Absent
12	307	Combination	Absent	Absent	Absent		Absent	Absent	Absent
12	308	PM	Absent	Absent	Absent		Absent	Absent	Absent
12	309	PM	Absent	Absent	Absent		Absent	Absent	Absent
12	310	SSG	Absent	Absent	Absent	Absent	Absent	Absent	Absent
12	311	SSG	Absent	Absent	Absent	Absent	Absent	Absent	Absent
12	312	Combination	Absent	Absent	Absent		Absent	Absent	Absent
12	313	PM	Absent	Absent	Absent		Absent	Absent	Absent
12	314	SSG	Absent	Absent	Absent	Absent	Absent	Absent	Absent
12	315	Combination	Absent	Absent	Absent		Absent	Absent	Absent
12	316	SSG	Absent	Absent	Absent		Absent	Absent	Absent
12	317	SSG	Absent	Absent	Absent	Absent	Absent		Absent

Centre	Patient					Jaundice			
number	number	Treatment	Baseline	Day7	Day 14	Day 21	EOT	3 mon FU	6 mon FU
12	318	Combination	Absent	Absent	Absent		Absent	Absent	Absent
12	319	Combination	Absent	Absent	Absent		Absent	Absent	Absent
12	320	Combination	Absent	Absent	Absent		Absent	Absent	Absent
12	321	Combination	Absent	Absent	Absent		Absent	Absent	Absent
12	322	PM	Absent	Absent	Absent		Absent	Absent	Absent
12	323	SSG	Absent	Absent	Absent	Absent	Absent	Absent	Absent
12	324	SSG	Absent	Absent	Absent	Absent	Absent	Absent	Absent
12	325	PM	Absent	Absent	Absent		Absent	Absent	Absent
12	326	PM	Absent	Absent	Absent		Absent	Absent	Absent
12	327	Combination	Absent	Absent	Absent		Absent	Absent	Absent
12	328	PM	Absent	Absent	Absent		Absent	Absent	Absent
12	329	SSG	Absent	Absent	Absent	Absent	Absent	Absent	Absent
12	330	PM	Absent	Absent	Absent		Absent	Absent	Absent
23	361	Combination	Absent	Absent	Absent		Absent	Absent	Absent
23	362	PM	Absent	Absent	Absent		Absent	Absent	Absent
23	363	PM	Absent	Absent	Absent		Absent	Absent	Absent
23	364	Combination	Absent	Absent	Absent		Absent	Absent	Absent
23	365	SSG	Absent	Absent	Absent	Absent	Absent	Absent	Absent
23	366	Combination	Absent	Absent	Absent		Absent	Absent	Absent
23	367	SSG	Absent	Absent	Absent	Absent	Absent	Absent	Absent
23	368	PM	Absent	Absent	Absent		Absent	Absent	Absent
23	369	Combination	Absent	Absent	Absent		Absent	Absent	Absent
23	370	SSG	Absent	Absent	Absent	Absent	Absent	Absent	Absent
23	371	PM	Absent	Absent	Absent		Absent	Absent	Absent
23	372	PM	Absent	Absent	Absent		Absent	Absent	Absent
23	373	SSG	Absent	Absent	Absent	Absent	Absent	Absent	Absent
23	374	SSG	Absent	Absent	Absent	Absent	Absent	Absent	Absent
23	375	Combination	Absent	Absent	Absent		Absent	Absent	Absent
23	376	SSG	Absent	Absent	Absent	Absent	Absent	Absent	Absent
23	377	Combination	Absent	Absent					Absent
23	378	Combination	Absent	Absent	Absent		Absent	Absent	Absent
23	379	PM	Absent	Absent	Absent		Absent	Absent	Absent
23	380	SSG	Absent	Absent	Absent	Absent	Absent	Absent	Absent
23	381	PM	Absent	Absent	Absent		Absent	Absent	Absent
23	382	Combination	Absent	Absent	Absent		Absent	Absent	Absent
23	383	PM	Absent	Absent	Absent		Absent	Absent	Absent
23	384	SSG	Absent	Absent	Absent	Absent	Absent	Absent	Absent
23	385	SSG	Absent	Absent	Absent	Absent	Absent	Absent	Absent
23	386	Combination	Absent	Absent	Absent		Absent	Absent	Absent
23	387	Combination	Absent	Absent	Absent		Absent	Absent	Absent
23	388	PM	Absent	Absent	Absent		Absent	Absent	Absent
23	389	SSG	Absent	Absent	Absent	Absent	Absent	Absent	Absent
23	390	PM	Absent	Absent	Absent		Absent	Absent	Absent
23	391	Combination	Absent	Absent	Absent		Absent	Absent	Absent
23	392	Combination	Absent	Absent	Absent			Absent	Absent
23	393	SSG	Absent	Absent	Absent	Absent	Absent	Absent	Absent
23	394	PM	Absent	Absent	Absent		Absent	Absent	Absent
23	395	PM	Absent	Absent	Absent		Absent	Absent	Absent
23	396	SSG	Absent	Absent	Absent	Absent	Absent	Absent	Absent
23	397	SSG	Absent	Absent	Absent	Absent	Absent	Absent	Absent
23	398	Combination	Absent	Absent	Absent		Absent	Absent	Absent
23	399	PM	Absent	Absent	Absent		Absent	Absent	Absent
23	400	PM	Absent	Absent	Absent		Absent	Absent	Absent

Centre number	Patient number	Treatment	Baseline	Day7	Day 14	Jaundice<br>Day 21	EOT	3 mon FU	6 mon FU
23	401	Combination	Absent	Absent	Absent		Absent	Absent	Absent
23	402	Combination	Absent	Absent	Absent		Absent	Absent	Absent
23	403	SSG	Absent	Absent	Absent	Absent	Absent	Absent	Absent
23	404	SSG	Absent	Absent	Absent	Absent	Absent		Absent
23	405	PM	Absent	Absent	Absent		Absent	Absent	Absent
34	451	Combination	Absent	Absent	Absent		Absent		Absent
34	452	PM	Absent	Absent	Absent		Absent		
34	453	PM	Absent	Absent	Absent		Absent		
34	454	Combination	Absent	Absent	Absent		Absent		Absent
34	455	PM	Absent	Absent	Absent		Absent		
34	456	SSG	Absent	Absent	Absent	Absent	Absent	Absent	
34	457	Combination	Absent	Absent	Absent		Absent		
34	458	PM	Absent	Absent	Absent		Absent	Absent	
34	459	Combination	Absent	Absent	Absent		Absent	Absent	Absent
34	460	PM	Absent	Absent	Absent		Absent		
34	461	SSG	Absent	Absent	Absent				
34	462	SSG	Absent	Absent	Absent	Absent	Absent		Absent
34	463	Combination	Absent	Absent	Absent		Absent		Absent
34	464	SSG	Absent	Absent	Absent	Absent	Absent		Absent
34	465	SSG	Absent	Absent	Absent	Absent	Absent		Absent
34	466	Combination	Absent	Absent	Absent		Absent		
34	467	PM	Absent	Absent	Absent		Absent	Absent	
34	468	Combination	Absent	Absent	Absent		Absent		Absent
34	469	SSG	Absent	Absent	Absent	Absent	Absent		Absent
34	470	Combination	Absent	Absent	Absent		Absent		Absent
34	471	PM	Absent	Absent	Absent		Absent		
34	472	SSG	Absent	Absent	Absent	Absent	Absent		Absent
34	473	PM	Absent	Absent	Absent		Absent		
34	474	Combination	Absent	Absent	Absent		Absent		Absent
34	475	PM	Absent	Absent	Absent		Absent		
34	476	PM	Absent	Absent	Absent		Absent	Absent	
34	477	SSG	Absent	Absent	Absent	Absent	Absent		Absent
34	478	Combination	Absent	Absent	Absent		Absent		Absent
34	479	SSG	Absent	Absent	Absent	Absent	Absent		Absent
34	480	SSG	Absent	Absent	Absent	Absent	Absent		Absent
34	481	Combination	Absent	Absent	Absent		Absent		Absent
34	482	Combination	Absent	Absent	Absent		Absent		Absent
34	483	PM	Absent	Absent	Absent		Absent		Absent
34	484	PM	Absent	Absent					
34	485	Combination	Present	Absent	Absent		Absent		Absent
34	486	Combination	Absent	Absent	Absent		Absent		Absent
34	487	SSG	Absent	Absent	Absent	Absent	Absent		Absent
34	488	SSG	Absent	Absent	Absent	Absent	Absent	Absent	Absent
34	489	SSG	Absent	Absent	Absent	Absent	Absent		Absent
34	490	PM	Absent	Absent	Absent		Absent		Absent
34	491	PM	Absent	Absent	Absent		Absent		
34	492	PM	Absent	Absent	Absent		Absent		Absent
34	493	SSG	Absent	Absent	Absent	Absent	Absent	Absent	
34	494	Combination	Absent	Absent	Absent		Absent		Absent
34	495	SSG	Absent	Absent	Absent	Absent	Absent		Absent
34	496	SSG	Absent	Absent	Absent	Absent	Absent		Absent
34	497	PM	Absent	Absent	Absent		Absent	Absent	
34	498	SSG	Absent	Absent	Absent	Absent	Absent		Absent

Centre number	Patient number	Treatment	Jaundice Baseline	Day7	Day 14	Day 21	EOT	3 mon FU	6 mon FU
34	499	Combination	Absent	Absent	Absent		Absent		Absent
34	500	PM	Absent	Absent	Absent		Absent		Absent
34	501	Combination	Absent	Absent	Absent		Absent		Absent
34	502	Combination	Absent	Absent	Absent		Absent		Absent
34	503	PM	Absent	Absent	Absent		Absent		
34	504	Combination	Absent	Absent	Absent		Absent		
34	505	SSG	Absent	Absent	Absent	Absent	Absent		Absent
34	506	SSG	Absent	Absent	Absent	Absent	Absent		Absent
34	507	SSG	Absent	Absent	Absent	Absent	Absent		Absent
34	508	PM	Absent	Absent	Absent		Absent		
34	509	PM	Absent	Absent	Absent		Absent		
34	510	Combination	Absent	Absent	Absent		Absent		Absent
34	511	Combination	Absent	Absent	Absent		Absent		Absent
34	512	PM	Absent	Absent	Absent		Absent		
34	513	Combination	Absent	Absent	Absent		Absent	Absent	Absent
34	514	SSG	Absent	Absent	Absent	Absent	Absent		Absent
34	515	SSG	Absent	Absent	Absent	Absent	Absent	Absent	
34	516	PM	Absent	Absent	Absent		Absent		Absent
34	517	PM	Absent	Absent	Absent		Absent		
34	518	SSG	Absent	Absent	Absent	Absent	Absent		Absent
34	519	PM	Absent	Absent	Absent		Absent	Absent	
34	520	SSG	Absent	Absent	Absent	Absent	Absent		Absent
34	521	PM	Absent	Absent	Absent		Absent		
34	522	Combination	Absent	Absent	Absent		Absent		
34	523	SSG	Absent	Absent	Absent	Absent	Absent		Absent
34	524	Combination	Absent	Absent	Absent		Absent		Absent
34	525	Combination	Absent	Absent	Absent		Absent		Absent
34	526	SSG	Absent	Absent	Absent	Absent	Absent		Absent
34	527	Combination	Absent	Absent	Absent		Absent		Absent
34	528	PM	Absent	Absent	Absent		Absent		
34	529	Combination	Absent	Absent	Absent		Absent		Absent
34	530	Combination	Absent	Absent	Absent		Absent		Absent
34	531	SSG	Absent	Absent	Absent	Absent	Absent		
34	532	Combination	Absent	Absent	Absent		Absent		Absent
34	533	SSG	Absent	Absent	Absent	Absent	Absent		Absent
34	534	PM	Absent	Absent	Absent		Absent		
34	535	PM	Absent	Absent	Absent		Absent	Absent	
34	536	PM	Absent	Absent	Absent		Absent		Absent
34	537	SSG	Absent	Absent	Absent	Absent	Absent		Absent
34	538	PM	Absent	Absent	Absent		Absent		
34	539	Combination	Absent	Absent	Absent		Absent		Absent
34	540	SSG	Absent	Absent	Absent	Absent	Absent		Absent
35	646	PM	Absent	Absent	Absent		Absent		Absent
35	647	PM	Absent	Absent	Absent		Absent	Absent	Absent
35	648	Combination	Absent	Absent	Absent		Absent	Absent	Absent
35	649	Combination	Absent	Absent	Absent		Absent	Absent	Absent
35	650	SSG	Absent	Absent	Absent	Absent	Absent	Absent	Absent
35	651	SSG	Absent	Absent	Absent	Absent	Absent	Absent	Absent
35	652	PM	Absent	Absent	Absent		Absent		Absent
35	653	Combination	Absent	Absent	Absent		Absent	Absent	Absent
35	654	SSG	Absent	Absent	Absent	Absent	Absent	Absent	Absent
35	655	Combination	Absent	Absent	Absent		Absent	Absent	Absent
35	656	SSG	Absent	Absent	Absent	Absent	Absent	Absent	Absent

Centre	Patient					Jaundice			
number	number	Treatment	Baseline	Day7	Day 14	Day 21	EOT	3 mon FU	6 mon FU
35	657	PM	Absent	Absent	Absent		Absent	Absent	Absent
35	658	Combination	Absent	Absent	Absent		Absent	Absent	Absent
35	659	SSG	Absent	Absent	Absent	Absent	Absent	Absent	Absent
35	660	PM	Absent	Absent	Absent		Absent	Absent	Absent
35	661	Combination	Absent	Absent	Absent		Absent	Absent	Absent
35	662	PM	Absent	Absent	Absent		Absent	Absent	Absent
35	663	SSG	Absent	Absent	Absent	Absent	Absent	Absent	Absent
35	664	Combination	Absent	Absent					Absent
35	665	SSG	Absent	Absent	Absent	Absent	Absent	Absent	Absent
35	666	SSG	Absent	Absent	Absent	Absent	Absent	Absent	Absent
35	667	Combination	Absent	Absent	Absent		Absent	Absent	Absent
35	668	PM	Absent	Absent	Absent		Absent		Absent
35	669	PM	Absent	Absent	Absent		Absent		
35	670	SSG	Absent	Absent	Absent	Absent	Absent	Absent	Absent
35	671	PM	Absent	Absent	Absent		Absent		Absent
35	672	Combination	Absent	Absent	Absent		Absent		Absent
35	673	SSG	Absent	Absent	Absent	Absent	Absent	Absent	Absent
35	674	Combination	Absent	Absent	Absent		Absent	Absent	Absent
35	675	PM	Absent	Absent	Absent		Absent		Absent
35	676	PM	Absent	Absent	Absent		Absent	Absent	Absent
35	677	SSG	Absent	Absent					
35	678	Combination	Absent	Absent	Absent		Absent	Absent	Absent
35	679	SSG	Absent	Absent	Absent	Absent	Absent	Absent	Absent
35	680	Combination	Absent	Absent	Absent		Absent	Absent	Absent
35	681	Combination	Absent	Absent	Absent		Absent	Absent	Absent
35	682	PM	Absent	Absent	Absent		Absent		Absent
35	683	Combination	Absent	Absent	Absent		Absent		Absent
35	684	SSG	Absent	Absent	Absent	Absent	Absent	Absent	Absent
35	685	PM	Absent	Absent	Absent		Absent	Absent	Absent
35	686	PM	Absent	Absent	Absent		Absent	Absent	Absent
35	687	SSG	Absent	Absent	Absent	Absent	Absent	Absent	Absent
35	688	PM	Absent	Absent	Absent		Absent	Absent	Absent
35	689	Combination	Absent	Absent	Absent		Absent	Absent	Absent
35	690	SSG	Absent	Absent	Absent	Absent	Absent	Absent	Absent

Centre Number	Patient Number	Treatment	Petchial haemorrhage						
			Baseline	Day7	Day 14	Day 21	EOT	3 mon FU	6 mon FU
11	1	PM	Absent	Absent	Absent		Absent	Absent	Absent
11	2	Combination	Absent	Absent	Absent		Absent	Absent	Absent
11	3	Combination	Absent	Absent	Absent		Absent	Absent	Absent
11	4	PM	Absent	Absent	Absent		Absent	Absent	Absent
11	5	PM	Absent	Absent	Absent		Absent	Absent	Absent
11	6	Combination	Absent	Absent	Absent		Absent	Absent	Absent
11	7	PM	Absent	Absent	Absent		Absent	Absent	Absent
11	8	SSG	Absent	Absent	Absent	Absent	Absent	Absent	Absent
11	9	SSG	Absent	Absent	Absent	Absent	Absent	Absent	Absent
11	10	SSG	Absent	Absent	Absent	Absent	Absent	Absent	Absent
11	11	Combination	Absent	Absent	Absent		Absent	Absent	Absent
11	12	Combination	Absent	Absent	Absent		Absent	Absent	Absent
11	13	SSG	Absent	Absent	Absent	Absent	Absent	Absent	Absent
11	14	SSG	Absent	Absent	Absent	Absent	Absent	Absent	Absent
11	15	PM	Absent	Absent				Absent	Absent
11	16	Combination	Absent	Absent	Absent		Absent	Absent	Absent
11	17	Combination	Absent	Absent	Absent		Absent	Absent	Absent
11	18	SSG	Absent	Absent	Absent	Absent	Absent	Absent	Absent
11	19	Combination	Absent	Absent	Absent		Absent	Absent	Absent
11	20	SSG	Absent	Absent	Absent	Absent	Absent	Absent	Absent
11	21	SSG	Absent	Absent	Absent	Absent	Absent	Absent	Absent
11	22	SSG	Absent	Absent	Absent	Absent	Absent	Absent	Absent
11	23	PM	Absent	Absent	Absent		Absent	Absent	Absent
11	24	PM	Absent	Absent	Absent		Absent	Absent	Absent
11	25	SSG	Absent	Absent	Absent	Absent	Absent	Absent	Absent
11	26	Combination	Absent	Absent	Absent		Absent	Absent	Absent
11	27	PM	Absent	Absent	Absent		Absent	Absent	
11	28	Combination	Absent	Absent	Absent		Absent	Absent	Absent
11	29	PM	Absent	Absent	Absent		Absent	Absent	Absent
11	30	PM	Absent	Absent	Absent		Absent	Absent	Absent
11	31	SSG	Absent	Absent	Absent	Absent	Absent	Absent	Absent
11	32	Combination	Absent	Absent	Absent		Absent	Absent	Absent
11	33	SSG	Absent	Absent	Absent	Absent	Absent	Absent	Absent
11	34	PM	Absent	Absent	Absent		Absent	Absent	Absent
11	35	SSG	Absent	Absent	Absent	Absent	Absent	Absent	Absent
11	36	SSG	Absent	Absent	Absent	Absent	Absent	Absent	Absent
11	37	Combination	Absent	Absent	Absent		Absent	Absent	Absent
11	38	PM	Absent	Absent	Absent		Absent	Absent	Present
11	39	PM	Absent	Absent	Absent		Absent	Absent	Absent
11	40	PM	Absent	Absent	Present			Absent	Absent
11	41	Combination	Absent	Absent	Absent		Absent	Absent	Absent
11	42	Combination	Absent	Absent	Absent		Absent	Absent	Absent
11	43	SSG	Absent	Absent	Absent	Absent	Absent	Absent	Absent
11	44	PM	Absent	Absent	Absent		Absent	Absent	Absent
11	45	Combination	Absent	Absent	Absent		Absent	Absent	Absent
11	46	SSG	Absent	Absent	Absent	Absent	Absent	Absent	Absent
11	47	SSG	Absent	Absent	Absent	Absent	Absent		
11	48	Combination	Absent	Absent	Absent		Absent		Absent
11	49	SSG	Absent	Absent	Absent	Absent	Absent	Absent	Absent
11	50	PM	Absent	Absent	Absent		Absent	Absent	Absent
11	51	Combination	Absent	Absent	Absent		Absent	Absent	Absent
11	52	PM	Absent	Absent	Absent		Absent	Absent	Absent
11	53	SSG	Absent	Absent	Absent	Absent	Absent	Absent	Absent
11	54	PM	Absent	Absent	Absent		Absent	Absent	Absent

Centre	Patient		Petchial haemorrhage						
Number	Number	Treatment	Baseline	Day7	Day 14	Day 21	EOT	3 mon FU	6 mon FU
11	55	PM	Absent	Absent	Absent		Absent	Absent	Absent
11	56	SSG	Absent	Absent	Absent	Absent	Absent	Absent	Absent
11	57	Combination	Absent	Absent	Absent		Absent	Absent	Absent
11	58	Combination	Absent	Absent	Absent		Absent	Absent	Absent
11	59	PM	Absent	Absent	Absent		Absent	Absent	Absent
11	60	Combination	Absent	Absent	Absent		Absent	Absent	Absent
11	61	PM	Absent	Absent	Absent		Absent	Absent	Absent
11	62	SSG	Absent	Absent	Absent	Absent	Absent	Absent	Absent
11	63	SSG	Absent	Absent	Absent	Absent	Absent	Absent	Absent
11	64	SSG	Absent	Absent	Absent	Absent	Absent	Absent	Absent
11	65	PM	Absent	Absent	Absent		Absent		
11	66	Combination	Absent	Absent	Absent		Absent	Absent	Absent
11	67	PM	Absent	Absent	Absent		Absent	Absent	Absent
11	68	SSG	Absent	Absent	Absent	Absent	Absent	Absent	Absent
11	69	Combination	Absent	Absent	Absent		Absent	Absent	Absent
11	70	Combination	Absent	Absent	Absent		Absent	Absent	Absent
11	71	Combination	Absent	Absent	Absent		Absent	Absent	Absent
11	72	SSG	Absent	Absent	Absent			Absent	Absent
11	73	PM	Absent	Absent	Absent		Absent	Absent	Absent
11	74	PM	Absent	Absent	Absent		Absent	Absent	Absent
11	75	Combination	Absent	Absent	Absent		Absent	Absent	Absent
11	76	PM	Absent	Absent	Absent		Absent	Absent	Absent
11	77	PM	Absent	Absent	Absent		Absent	Absent	Absent
11	78	SSG	Absent	Absent	Absent	Absent	Absent	Absent	Absent
11	79	PM	Absent	Absent	Absent		Absent	Absent	Absent
11	80	PM	Absent	Absent	Absent		Absent	Absent	Absent
11	81	Combination	Absent	Absent	Absent		Absent	Absent	Absent
11	82	SSG	Absent	Absent	Absent	Absent	Absent	Absent	Absent
11	83	SSG	Absent	Absent	Absent	Absent	Absent	Absent	Absent
11	84	SSG	Absent	Absent	Absent	Absent	Absent	Absent	Absent
11	85	SSG	Absent	Absent	Absent	Absent	Absent	Absent	
11	86	Combination	Absent	Absent	Absent		Absent	Absent	Absent
11	87	Combination	Absent	Absent	Absent		Absent	Absent	Absent
11	88	PM	Absent	Absent	Absent		Absent	Absent	Absent
11	89	Combination	Absent	Absent	Absent		Absent	Absent	Absent
11	90	Combination	Absent	Absent	Absent		Absent		
11	91	PM	Absent	Absent	Absent		Absent	Absent	Absent
11	92	Combination	Absent	Absent	Absent		Absent	Absent	Absent
11	93	SSG	Absent	Absent	Absent	Absent	Absent	Absent	Absent
11	94	SSG	Absent	Absent	Absent	Absent	Absent	Absent	Absent
11	95	SSG	Absent	Absent	Absent	Absent	Absent	Absent	Absent
11	96	Combination	Absent	Absent	Absent		Absent	Absent	Absent
11	97	Combination	Absent	Absent	Absent		Absent	Absent	Absent
11	98	PM	Absent	Absent	Absent		Absent	Absent	Absent
11	99	SSG	Absent	Absent	Absent	Absent	Absent	Absent	Absent
11	100	PM	Absent	Absent	Absent		Absent		
11	101	PM	Absent	Absent	Absent		Absent	Absent	Absent
11	102	Combination	Absent	Absent	Absent		Absent	Absent	Absent
11	103	Combination	Absent	Absent	Absent		Absent	Absent	Absent
11	104	PM	Absent	Absent	Absent		Absent	Absent	Absent
11	105	SSG	Absent	Absent	Absent	Absent	Absent	Absent	Absent
11	106	Combination	Absent	Absent	Absent		Absent	Absent	Absent
11	107	PM	Absent	Absent	Absent		Absent	Absent	Absent
11	108	PM	Absent	Absent	Absent		Absent	Absent	Absent

Centre Number	Patient Number	Treatment	Petchial haemorrhage Baseline	Day7	Day 14	Day 21	EOT	3 mon FU	6 mon FU
11	109	SSG	Absent	Absent	Absent	Absent	Absent	Absent	Absent
11	110	PM	Absent	Absent	Absent		Absent	Absent	Absent
11	111	Combination	Absent	Absent	Absent		Absent	Absent	Absent
11	112	Combination	Absent	Absent	Absent		Absent	Absent	Absent
11	113	SSG	Absent	Absent	Absent	Absent	Absent	Absent	
11	114	SSG	Absent	Absent	Absent	Absent	Absent	Absent	Absent
11	115	Combination	Absent	Absent	Absent		Absent	Absent	Absent
11	116	SSG	Absent	Absent	Absent	Absent	Absent	Absent	Absent
11	117	PM	Absent	Absent	Absent		Absent	Absent	
11	118	PM	Absent	Absent	Absent		Absent	Absent	Absent
11	119	SSG	Absent	Absent	Absent	Absent	Absent	Absent	Absent
11	120	Combination	Absent	Absent	Absent		Absent	Absent	Absent
11	121	Combination	Absent	Absent	Absent		Absent	Absent	Absent
11	122	SSG	Absent	Absent	Absent	Absent	Absent		
11	123	SSG	Absent	Absent	Absent	Absent	Absent	Absent	Absent
11	124	SSG	Absent	Absent	Absent	Absent	Absent		Absent
11	125	SSG	Absent	Absent	Absent	Absent	Absent	Absent	Absent
11	126	Combination	Absent	Absent	Absent		Absent		Absent
11	127	PM	Absent	Absent	Absent		Absent	Absent	Absent
11	128	Combination	Absent	Absent	Absent		Absent	Absent	Absent
11	129	PM	Absent	Absent	Absent		Absent	Absent	Absent
11	130	Combination	Absent	Absent	Absent		Absent	Absent	Absent
11	131	PM	Absent	Absent	Absent		Absent	Absent	Absent
11	132	SSG	Absent	Absent	Absent	Absent	Absent	Absent	Absent
11	133	Combination	Absent	Absent	Absent		Absent	Absent	Absent
11	134	PM	Absent	Absent	Absent		Absent	Absent	Absent
11	135	PM	Absent	Absent	Absent		Absent	Absent	Absent
12	241	Combination	Absent	Absent	Absent		Absent		Absent
12	242	SSG	Absent	Absent	Absent	Absent	Absent	Absent	Absent
12	243	PM	Absent	Absent	Absent		Absent	Absent	Absent
12	244	SSG	Absent	Absent	Absent	Absent	Absent	Absent	Absent
12	245	PM	Absent	Absent	Absent		Absent	Absent	
12	246	PM	Absent	Absent	Absent		Absent	Absent	
12	247	PM	Absent	Absent	Absent		Absent	Absent	Absent
12	248	PM	Absent	Absent	Absent		Absent	Absent	Absent
12	249	Combination	Absent	Absent	Absent		Absent	Absent	Absent
12	250	SSG	Absent	Absent	Absent	Absent	Absent	Absent	Absent
12	251	Combination	Absent	Absent	Absent		Absent	Absent	Absent
12	252	SSG	Absent	Absent	Absent	Absent	Absent	Absent	Absent
12	253	SSG	Absent	Absent	Absent	Absent	Absent	Absent	Absent
12	254	Combination	Absent	Absent	Absent		Absent	Absent	Absent
12	255	Combination	Absent	Absent	Absent		Absent	Absent	Absent
12	256	Combination	Absent	Absent	Absent		Absent	Absent	Absent
12	257	SSG	Absent	Absent	Absent	Absent	Absent	Absent	Absent
12	258	SSG	Absent	Absent	Absent	Absent	Absent		Absent
12	259	PM	Absent	Absent	Absent		Absent	Absent	Absent
12	260	PM	Absent	Absent	Absent		Absent		Absent
12	261	Combination	Absent	Absent	Absent		Absent	Absent	Absent
12	262	PM	Absent	Absent	Absent		Absent	Absent	Absent
12	263	SSG	Absent	Absent	Absent	Absent	Absent	Absent	Absent
12	264	Combination	Absent	Absent	Absent		Absent	Absent	Absent
12	265	SSG	Absent	Absent	Absent	Absent	Absent		
12	266	PM	Absent	Absent	Absent		Absent	Absent	Absent
12	267	SSG	Absent	Absent	Absent	Absent	Absent	Absent	Absent

Centre Number	Patient Number	Treatment	Petchial haemorrhage Baseline	Day7	Day 14	Day 21	EOT	3 mon FU	6 mon FU
12	268	PM	Absent	Absent	Absent		Absent	Absent	Absent
12	269	Combination	Absent	Absent	Absent		Absent	Absent	Absent
12	270	Combination	Absent	Absent	Absent		Absent		Absent
12	271	SSG	Absent	Absent	Absent	Absent	Absent	Absent	Absent
12	272	PM	Absent	Absent	Absent		Absent	Absent	Absent
12	273	SSG	Absent	Absent	Absent	Absent	Absent	Absent	Absent
12	274	SSG	Absent	Absent	Absent	Absent	Absent	Absent	Absent
12	275	Combination	Absent	Absent	Absent		Absent	Absent	Absent
12	276	PM	Absent	Absent	Absent		Absent	Absent	Absent
12	277	Combination	Absent	Absent	Absent		Absent	Absent	Absent
12	278	SSG	Absent	Absent	Absent	Absent	Absent	Absent	
12	279	PM	Absent	Absent	Absent		Absent	Absent	Absent
12	280	SSG	Absent	Absent	Absent	Absent	Absent	Absent	Absent
12	281	Combination	Absent	Absent	Absent		Absent	Absent	Absent
12	282	PM	Absent	Absent	Absent		Absent	Absent	Absent
12	283	PM	Absent	Absent	Absent		Absent	Absent	Absent
12	284	Combination	Absent	Absent	Absent		Absent	Absent	Absent
12	285	Combination	Absent	Absent	Absent		Absent	Absent	Absent
12	286	PM	Absent	Absent	Absent		Absent	Absent	Absent
12	287	SSG	Absent	Absent	Absent	Absent	Absent	Absent	Absent
12	288	Combination	Absent	Absent	Absent		Absent	Absent	Absent
12	289	Combination	Absent	Absent	Absent		Absent	Absent	Absent
12	290	SSG	Absent	Absent	Absent	Absent	Absent		Absent
12	291	SSG	Absent	Absent	Absent	Absent	Absent	Absent	Absent
12	292	PM	Absent	Absent	Absent		Absent		Absent
12	293	SSG	Absent	Absent	Absent	Absent	Absent		Absent
12	294	Combination	Absent	Absent	Absent		Absent	Absent	Absent
12	295	Combination	Absent	Absent	Absent		Absent		Absent
12	296	PM	Absent	Absent	Absent		Absent	Absent	Absent
12	297	SSG	Absent	Absent	Absent				
12	298	PM	Absent	Absent	Absent		Absent	Absent	Absent
12	299	Combination	Absent	Absent	Absent		Absent		Absent
12	300	PM	Absent	Absent	Absent		Absent	Absent	Absent
12	301	SSG	Absent	Absent	Absent	Absent	Absent	Absent	Absent
12	302	SSG	Absent	Absent	Absent	Absent	Absent	Absent	Absent
12	303	PM	Absent	Absent	Absent		Absent	Absent	Absent
12	304	Combination	Absent	Absent	Absent		Absent	Absent	Absent
12	305	PM	Absent	Absent	Absent		Absent	Absent	Absent
12	306	Combination	Absent	Absent	Absent		Absent	Absent	Absent
12	307	Combination	Absent	Absent	Absent		Absent	Absent	Absent
12	308	PM	Absent	Absent	Absent		Absent	Absent	Absent
12	309	PM	Absent	Absent	Absent		Absent	Absent	Absent
12	310	SSG	Absent	Absent	Absent	Absent	Absent	Absent	Absent
12	311	SSG	Absent	Absent	Absent	Absent	Absent	Absent	Absent
12	312	Combination	Absent	Absent	Absent		Absent	Absent	Absent
12	313	PM	Absent	Absent	Absent		Absent	Absent	Absent
12	314	SSG	Absent	Absent	Absent	Absent	Absent	Absent	Absent
12	315	Combination	Absent	Absent	Absent		Absent	Absent	Absent
12	316	SSG	Absent	Absent	Absent		Absent	Absent	Absent
12	317	SSG	Absent	Absent	Absent	Absent	Absent		Absent
12	318	Combination	Absent	Absent	Absent		Absent	Absent	Absent
12	319	Combination	Absent	Absent	Absent		Absent	Absent	Absent
12	320	Combination	Absent	Absent	Absent		Absent	Absent	Absent
12	321	Combination	Absent	Absent	Absent		Absent	Absent	Absent

Centre Number	Patient Number	Treatment	Petchial haemorrhage Baseline	Day7	Day 14	Day 21	EOT	3 mon FU	6 mon FU
12	322	PM	Absent	Absent	Absent		Absent	Absent	Absent
12	323	SSG	Absent	Absent	Absent	Absent	Absent	Absent	Absent
12	324	SSG	Absent	Absent	Absent	Absent	Absent	Absent	Absent
12	325	PM	Absent	Absent	Absent		Absent	Absent	Absent
12	326	PM	Absent	Absent	Absent		Absent	Absent	Absent
12	327	Combination	Absent	Absent	Absent		Absent	Absent	Absent
12	328	PM	Absent	Absent	Absent		Absent	Absent	Absent
12	329	SSG	Absent	Absent	Absent	Absent	Absent	Absent	Absent
12	330	PM	Absent	Absent	Absent		Absent	Absent	Absent
23	361	Combination	Absent	Absent	Absent		Absent	Absent	Absent
23	362	PM	Absent	Absent	Absent		Absent	Absent	Absent
23	363	PM	Absent	Absent	Absent		Absent	Absent	Absent
23	364	Combination	Absent	Absent	Absent		Absent	Absent	Absent
23	365	SSG	Absent	Absent	Absent	Absent	Absent	Absent	Absent
23	366	Combination	Absent	Absent	Absent		Absent	Absent	Absent
23	367	SSG	Absent	Absent	Absent	Absent	Absent	Absent	Absent
23	368	PM	Absent	Absent	Absent		Absent	Absent	Absent
23	369	Combination	Absent	Absent	Absent		Absent	Absent	Absent
23	370	SSG	Absent	Absent	Absent	Absent	Absent	Absent	Absent
23	371	PM	Absent	Absent	Absent		Absent	Absent	Absent
23	372	PM	Absent	Absent	Absent		Absent	Absent	Absent
23	373	SSG	Absent	Absent	Absent	Absent	Absent	Absent	Absent
23	374	SSG	Absent	Absent	Absent	Absent	Absent	Absent	Absent
23	375	Combination	Absent	Absent	Absent		Absent	Absent	Absent
23	376	SSG	Absent	Absent	Absent	Absent	Absent	Absent	Absent
23	377	Combination	Absent	Absent					Absent
23	378	Combination	Absent	Absent	Absent		Absent	Absent	Absent
23	379	PM	Absent	Absent	Absent		Absent	Absent	Absent
23	380	SSG	Absent	Absent	Absent	Absent	Absent	Absent	Absent
23	381	PM	Absent	Absent	Absent		Absent	Absent	Absent
23	382	Combination	Absent	Absent	Absent		Absent	Absent	Absent
23	383	PM	Absent	Absent	Absent		Absent	Absent	Absent
23	384	SSG	Absent	Absent	Absent	Absent	Absent	Absent	Absent
23	385	SSG	Absent	Absent	Absent	Absent	Absent	Absent	Absent
23	386	Combination	Absent	Absent	Absent		Absent	Absent	Absent
23	387	Combination	Absent	Absent	Absent		Absent	Absent	Absent
23	388	PM	Absent	Absent	Absent		Absent	Absent	Absent
23	389	SSG	Absent	Absent	Absent	Absent	Absent	Absent	Absent
23	390	PM	Absent	Absent	Absent		Absent	Absent	Absent
23	391	Combination	Absent	Absent	Absent		Absent	Absent	Absent
23	392	Combination	Absent	Absent	Absent			Absent	Absent
23	393	SSG	Absent	Absent	Absent	Absent	Absent	Absent	Absent
23	394	PM	Absent	Absent	Absent		Absent	Absent	Absent
23	395	PM	Absent	Absent	Absent		Absent	Absent	Absent
23	396	SSG	Absent	Absent	Absent	Absent	Absent	Absent	Absent
23	397	SSG	Absent	Absent	Absent	Absent	Absent	Absent	Absent
23	398	Combination	Absent	Absent	Absent		Absent	Absent	Absent
23	399	PM	Absent	Absent	Absent		Absent	Absent	Absent
23	400	PM	Absent	Absent	Absent		Absent	Absent	Absent
23	401	Combination	Absent	Absent	Absent		Absent	Absent	Absent
23	402	Combination	Absent	Absent	Absent		Absent	Absent	Absent
23	403	SSG	Absent	Absent	Absent	Absent	Absent	Absent	Absent
23	404	SSG	Absent	Absent	Absent	Absent	Absent		Absent
23	405	PM	Absent	Absent	Absent		Absent	Absent	Absent

Centre Number	Patient Number	Treatment	Petchial haemorrhage Baseline	Day7	Day 14	Day 21	EOT	3 mon FU	6 mon FU
34	451	Combination	Absent	Absent	Absent		Absent		Absent
34	452	PM	Absent	Absent	Absent		Absent		
34	453	PM	Absent	Absent	Absent		Absent		
34	454	Combination	Absent	Absent	Absent		Absent		Absent
34	455	PM	Absent	Absent	Absent		Absent		
34	456	SSG	Absent	Absent	Absent	Absent	Absent	Absent	
34	457	Combination	Absent	Absent	Absent		Absent		
34	458	PM	Absent	Absent	Absent		Absent	Absent	
34	459	Combination	Absent	Absent	Absent		Absent	Absent	Absent
34	460	PM	Absent	Absent	Absent		Absent		
34	461	SSG	Absent	Absent	Absent				
34	462	SSG	Absent	Absent	Absent	Absent	Absent		Absent
34	463	Combination	Absent	Absent	Absent		Absent		Absent
34	464	SSG	Absent	Absent	Absent	Absent	Absent		Absent
34	465	SSG	Absent	Absent	Absent	Absent	Absent		Absent
34	466	Combination	Absent	Absent	Absent		Absent		
34	467	PM	Absent	Absent	Absent		Absent	Absent	
34	468	Combination	Absent	Absent	Absent		Absent		Absent
34	469	SSG	Absent	Absent	Absent	Absent	Absent		Absent
34	470	Combination	Absent	Absent	Absent		Absent		Absent
34	471	PM	Absent	Absent	Absent		Absent		
34	472	SSG	Absent	Absent	Absent	Absent	Absent		Absent
34	473	PM	Absent	Absent	Absent		Absent		
34	474	Combination	Absent	Absent	Absent		Absent		Absent
34	475	PM	Absent	Absent	Absent		Absent		
34	476	PM	Absent	Absent	Absent		Absent	Absent	
34	477	SSG	Absent	Absent	Absent	Absent	Absent		Absent
34	478	Combination	Absent	Absent	Absent		Absent		Absent
34	479	SSG	Absent	Absent	Absent	Absent	Absent		Absent
34	480	SSG	Absent	Absent	Absent	Absent	Absent		Absent
34	481	Combination	Absent	Absent	Absent		Absent		Absent
34	482	Combination	Absent	Absent	Absent		Absent		Absent
34	483	PM	Absent	Absent	Absent		Absent		Absent
34	484	PM	Absent	Absent					
34	485	Combination	Absent	Absent	Absent		Absent		Absent
34	486	Combination	Absent	Absent	Absent		Absent		Absent
34	487	SSG	Absent	Absent	Absent	Absent	Absent		Absent
34	488	SSG	Absent	Absent	Absent	Absent	Absent	Absent	Absent
34	489	SSG	Absent	Absent	Absent	Absent	Absent		Absent
34	490	PM	Absent	Absent	Absent		Absent		Absent
34	491	PM	Absent	Absent	Absent		Absent		
34	492	PM	Absent	Absent	Absent		Absent		Absent
34	493	SSG	Absent	Absent	Absent	Absent	Absent	Absent	
34	494	Combination	Absent	Absent	Absent		Absent		Absent
34	495	SSG	Absent	Absent	Absent	Absent	Absent		Absent
34	496	SSG	Absent	Absent	Absent	Absent	Absent		Absent
34	497	PM	Absent	Absent	Absent		Absent	Absent	
34	498	SSG	Absent	Absent	Absent	Absent	Absent		Absent
34	499	Combination	Absent	Absent	Absent		Absent		Absent
34	500	PM	Absent	Absent	Absent		Absent		Absent
34	501	Combination	Absent	Absent	Absent		Absent		Absent
34	502	Combination	Absent	Absent	Absent		Absent		Absent
34	503	PM	Absent	Absent	Absent		Absent		
34	504	Combination	Absent	Absent	Absent		Absent		

Centre Number	Patient Number	Treatment	Petchial haemorrhage Baseline	Day7	Day 14	Day 21	EOT	3 mon FU	6 mon FU
34	505	SSG	Absent	Absent	Absent	Absent	Absent		Absent
34	506	SSG	Absent	Absent	Absent	Absent	Absent		Absent
34	507	SSG	Absent	Absent	Absent	Absent	Absent		Absent
34	508	PM	Absent	Absent	Absent		Absent		
34	509	PM	Absent	Absent	Absent		Absent		
34	510	Combination	Absent	Absent	Absent		Absent		Absent
34	511	Combination	Absent	Absent	Absent		Absent		Absent
34	512	PM	Absent	Absent	Absent		Absent		
34	513	Combination	Absent	Absent	Absent		Absent	Absent	Absent
34	514	SSG	Absent	Absent	Absent	Absent	Absent		Absent
34	515	SSG	Absent	Absent	Absent	Absent	Absent	Absent	
34	516	PM	Absent	Absent	Absent		Absent		Absent
34	517	PM	Absent	Absent	Absent		Absent		
34	518	SSG	Absent	Absent	Absent	Absent	Absent		Absent
34	519	PM	Absent	Absent	Absent		Absent	Absent	
34	520	SSG	Absent	Absent	Absent	Absent	Absent		Absent
34	521	PM	Absent	Absent	Absent		Absent		
34	522	Combination	Absent	Absent	Absent		Absent		
34	523	SSG	Absent	Absent	Absent	Absent	Absent		Absent
34	524	Combination	Absent	Absent	Absent		Absent		Absent
34	525	Combination	Absent	Absent	Absent		Absent		Absent
34	526	SSG	Absent	Absent	Absent	Absent	Absent		Absent
34	527	Combination	Absent	Absent	Absent		Absent		Absent
34	528	PM	Absent	Absent	Absent		Absent		
34	529	Combination	Absent	Absent	Absent		Absent		Absent
34	530	Combination	Absent	Absent	Absent		Absent		Absent
34	531	SSG	Absent	Absent	Absent	Absent	Absent		
34	532	Combination	Absent	Absent	Absent		Absent		Absent
34	533	SSG	Absent	Absent	Absent	Absent	Absent		Absent
34	534	PM	Absent	Absent	Absent		Absent		
34	535	PM	Absent	Absent	Absent		Absent	Absent	
34	536	PM	Absent	Absent	Absent		Absent		Absent
34	537	SSG	Absent	Absent	Absent	Absent	Absent		Absent
34	538	PM	Absent	Absent	Absent		Absent		
34	539	Combination	Absent	Absent	Absent		Absent		Absent
34	540	SSG	Absent	Absent	Absent	Absent	Absent		Absent
35	646	PM	Absent	Absent	Absent		Absent		Absent
35	647	PM	Absent	Absent	Absent		Absent	Absent	Absent
35	648	Combination	Absent	Absent	Absent		Absent	Absent	Absent
35	649	Combination	Absent	Absent	Absent		Absent	Absent	Absent
35	650	SSG	Absent	Absent	Absent	Absent	Absent	Absent	Absent
35	651	SSG	Absent	Absent	Absent	Absent	Absent	Absent	Absent
35	652	PM	Absent	Absent	Absent		Absent		Absent
35	653	Combination	Absent	Absent	Absent		Absent	Absent	Absent
35	654	SSG	Absent	Absent	Absent	Absent	Absent	Absent	Absent
35	655	Combination	Absent	Absent	Absent		Absent	Absent	Absent
35	656	SSG	Absent	Absent	Absent	Absent	Absent	Absent	Absent
35	657	PM	Absent	Absent	Absent		Absent	Absent	Absent
35	658	Combination	Absent	Absent	Absent		Absent	Absent	Absent
35	659	SSG	Absent	Absent	Absent	Absent	Absent	Absent	Absent
35	660	PM	Absent	Absent	Absent		Absent	Absent	Absent
35	661	Combination	Absent	Absent	Absent		Absent	Absent	Absent
35	662	PM	Absent	Absent	Absent		Absent	Absent	Absent
35	663	SSG	Absent	Absent	Absent	Absent	Absent	Absent	Absent

Centre	Patient		Petchial haemorrhage						
Number	Number	Treatment	Baseline	Day7	Day 14	Day 21	EOT	3 mon FU	6 mon FU
35	664	Combination	Absent	Absent					Absent
35	665	SSG	Absent	Absent	Absent	Absent	Absent	Absent	Absent
35	666	SSG	Absent	Absent	Absent	Absent	Absent	Absent	Absent
35	667	Combination	Absent	Absent	Absent		Absent	Absent	Absent
35	668	PM	Absent	Absent	Absent		Absent		Absent
35	669	PM	Absent	Absent	Absent		Absent		
35	670	SSG	Absent	Absent	Absent	Absent	Absent	Absent	Absent
35	671	PM	Absent	Absent	Absent		Absent		Absent
35	672	Combination	Absent	Absent	Absent		Absent		Absent
35	673	SSG	Absent	Absent	Absent	Absent	Absent	Absent	Absent
35	674	Combination	Absent	Absent	Absent		Absent	Absent	Absent
35	675	PM	Absent	Absent	Absent		Absent		Absent
35	676	PM	Absent	Absent	Absent		Absent	Absent	Absent
35	677	SSG	Absent	Absent					
35	678	Combination	Absent	Absent	Absent		Absent	Absent	Absent
35	679	SSG	Absent	Absent	Absent	Absent	Absent	Absent	Absent
35	680	Combination	Absent	Absent	Absent		Absent	Absent	Absent
35	681	Combination	Absent	Absent	Absent		Absent	Absent	Absent
35	682	PM	Absent	Absent	Absent		Absent		Absent
35	683	Combination	Absent	Absent	Absent		Absent		Absent
35	684	SSG	Absent	Absent	Absent	Absent	Absent	Absent	Absent
35	685	PM	Absent	Absent	Absent		Absent	Absent	Absent
35	686	PM	Absent	Absent	Absent		Absent	Absent	Absent
35	687	SSG	Absent	Absent	Absent	Absent	Absent	Absent	Absent
35	688	PM	Absent	Absent	Absent		Absent	Absent	Absent
35	689	Combination	Absent	Absent	Absent		Absent	Absent	Absent
35	690	SSG	Absent	Absent	Absent	Absent	Absent	Absent	Absent

16.3.7 Adverse event listings (each patient)

Appendix 44 Listing of adverse events per patient

Appendix 45 Listing of ECG Findings per patient

Appendix 46 Listing of chest x-ray finding per patient

Appendix 47 Listing of audiometry x-ray finding per patient

Appendix 44: Listing of adverse events per patient

Cent No	Pat No	Age	Sex	Treatment	Treatment start date	Adverse Event	Preferred term	AE start date	AE end date	Outcome	Intensity	Action taken	Related
11	2	19	Male	Comb	18-Jun-05	DROP OF HEMOGLOBIN	HAEMOGLOBIN DECREASED	5-Jan-06		Ongoing	Moderate	None	Not related
11	2	19	Male	Comb	18-Jun-05	DROP OF PLATLATE	PLATELET COUNT DECREASED	5-Jan-06		Ongoing	Moderate	None	Not related
11	3	20	Male	Comb	19-Jun-05	EPIGASTRIC PAIN	ABDOMINAL PAIN UPPER	19-Jun-05	7-Jul-05	Resolved	Mild	None	Not related
11	3	20	Male	Comb	19-Jun-05	ACUTE GASTROENTERITIS	DIARRHOEA	6-Jul-05	7-Jul-05	Resolved	Mild	None	Unlikely
11	3	20	Male	Comb	19-Jun-05	RAISED SGOT	ASPARTATE AMINOTRANSFERASE	25-Jun-05	6-Jul-05	Resolved	Mild	None	Possible
11	3	20	Male	Comb	19-Jun-05	RAISED ALP	BLOOD ALKALINE PHOSPHATASE	25-Jun-05	6-Jul-05	Resolved	Mild	None	Possible
11	5	10	Female	PM	30-Jun-05	CHILS & RIGER	CHILLS	8-Jul-05	8-Jul-05	Resolved	Mild	None	Unlikely
11	5	10	Female	PM	30-Jun-05	VOMITING	VOMITING	8-Jul-05	8-Jul-05	Resolved	Moderate	None	Unlikely
11	7	45	Female	PM	2-Jul-05	INJECTION SITE PAIN	INJECTION SITE PAIN	13-Jul-05		Ongoing	Moderate	None	Possible
11	7	45	Female	PM	2-Jul-05	AIRWAY DISEASE (BRONCHIAL ATHMA	ASTHMA	11-Oct-05	8-Nov-05	Resolved	Mild	None	Not related
11	7	45	Female	PM	2-Jul-05	UTI (URINARY TRACT INFECTION)	URINARY TRACT INFECTION	18-Jan-06	30-Jan-06	Resolved	Mild	None	Not related
11	9	20	Male	SSG	3-Jul-05	DYSPEPSIA	DYSPEPSIA	8-Jul-05		Ongoing	Mild	None	Not related
11	9	20	Male	SSG	3-Jul-05	LEFT LOWER CHEST PAIN	CHEST PAIN	8-Jul-05		Ongoing	Mild	None	Not related
11	10	17	Male	SSG	3-Jul-05	MALARIA	MALARIA	17-Jul-05	20-Jul-05	Resolved	Mild	None	Not related
11	10	17	Male	SSG	3-Jul-05	INCREASED SGOT	ASPARTATE AMINOTRANSFERASE INCREASED	27-Jul-05	3-Aug-05	Resolved	Moderate	None	Possible
11	10	17	Male	SSG	3-Jul-05	INCREASED SGPT	ALANINE AMINOTRANSFERASE INCREASED	27-Jul-05	3-Aug-05	Resolved	Moderate	None	Possible
11	11	12	Male	Comb	5-Jul-05	INJECTION SITE PAIN	INJECTION SITE PAIN	18-Jul-05		Ongoing	Mild	None	Probable
11	11	12	Male	Comb	5-Jul-05	ELEVATED LIVER ENZYME(SGOT)	ASPARTATE AMINOTRANSFERASE INCREASED	20-Jan-06		Ongoing	Moderate	None	Probable
11	12	18	Male	Comb	8-Jul-05	MALARIA	MALARIA	14-Jul-05	20-Jul-05	Resolved	Mild	None	Not related
11	12	18	Male	Comb	8-Jul-05	LEFT EAR AUDIOMETRIC SHIFT	AUDIOGRAM ABNORMAL	14-Jul-05	25-Jul-05	Resolved	Moderate	None	Possible
11	12	18	Male	Comb	8-Jul-05	HEADACHE	HEADACHE	20-Nov-05		Ongoing	Mild	None	Not related
11	13	40	Female	SSG	12-Jul-05	RIGHT EYE PAIN	EYE PAIN	1-Aug-05		Ongoing	Mild	None	Not related
11	14	7	Male	SSG	12-Jul-05	GIARDIASIS	GIARDIASIS	3-Aug-05	8-Aug-05	Resolved	Mild	None	Not related
11	14	7	Male	SSG	12-Jul-05	PNEUMONIA	PNEUMONIA	10-Aug-05	25-Aug-05	Resolved	Moderate	None	Unlikely
11	16	35	Female	Comb	15-Jul-05	UTI	URINARY TRACT INFECTION	28-Jul-05	4-Aug-05	Resolved	Mild	None	Not related
11	16	35	Female	Comb	15-Jul-05	HOOK WORM	HOOKWORM INFECTION	28-Jul-05	31-Jul-05	Resolved	Mild	None	Not related
11	16	35	Female	Comb	15-Jul-05	HEADACHE	HEADACHE	29-Jul-05		Ongoing	Mild	None	Not related
11	16	35	Female	Comb	15-Jul-05	RIGHT AUDIOMETRIC SHIFT	AUDIOGRAM ABNORMAL	31-Oct-05		Ongoing	Moderate	None	Probable
11	17	40	Male	Comb	19-Jul-05	OVA OF HOOK WORM 1/5	HOOKWORM INFECTION	25-Jul-05	28-Jul-05	Resolved	Moderate	None	Possible
11	17	40	Male	Comb	19-Jul-05	LARVEA OF S. STERCORALIS 1/5	STRONGYLOIDIASIS	25-Jul-05	28-Jul-05	Resolved	Moderate	None	Not related
11	17	40	Male	Comb	19-Jul-05	COUGH	COUGH	1-Aug-05		Ongoing	Mild	None	Unlikely
11	17	40	Male	Comb	19-Jul-05	DECREASE IN WBC	WHITE BLOOD CELL DECREASED	7-Nov-05		Ongoing	Moderate	None	Possible
11	17	40	Male	Comb	19-Jul-05	DECREASE IN PLATELET	PLATELET COUNT DECREASED	7-Nov-05	6-Feb-06	Resolved	Moderate	None	Possible
11	18	34	Male	SSG	16-Aug-05	HEADACHE	HEADACHE	18-Aug-05	19-Aug-05	Resolved	Mild	None	Not related
11	18	34	Male	SSG	16-Aug-05	NAUSEA	NAUSEA	18-Aug-05	19-Aug-05	Resolved	Mild	None	Unlikely

Cent No	Pat No	Age	Sex	Treatment	Treatment start date	Adverse Event	Preferred term	AE start date	AE end date	Outcome	Intensity	Action taken	Related
11	18	34	Male	SSG	16-Aug-05	COMMUNITY ACQUIRED PNEUMONIA	PNEUMONIA	15-Dec-05	2-Jan-06	Resolved	Mild	None	Not related
11	19	33	Male	Comb	16-Aug-05	PLATLATE(DROP OF PLATLATE)	PLATELET COUNT DECREASED	22-Aug-05		Ongoing	Severe	None	Possible
11	19	33	Male	Comb	16-Aug-05	INSOMINIA	INSOMINIA	18-Aug-05	22-Aug-05	Resolved	Mild	None	Not related
11	19	33	Male	Comb	16-Aug-05	ENTAMOEBA HISTOLYTICA DYSENTRY	AMOEBIASIS	27-Feb-06		Ongoing	Mild	None	Not related
11	20	7	Male	SSG	16-Aug-05	PNEUMONIA	PNEUMONIA	9-Sep-05	16-Sep-05	Resolved	Moderate	None	Not related
11	22	11	Male	SSG	17-Aug-05	CHILS	CHILLS	18-Aug-05		Resolved	Mild	None	Not related
11	22	11	Male	SSG	17-Aug-05	VOMITING	VOMITING	18-Aug-05		Resolved	Mild	None	Not related
11	23	10	Male	PM	20-Aug-05	PAIN AT INJECTION SITE	INJECTION SITE PAIN	26-Aug-05		Resolved	Mild	None	Not related
11	24	7	Female	PM	20-Aug-05	DIARRHEA	DIARRHOEA	20-Aug-05	27-Aug-05	Resolved	Mild	None	Not related
11	25	10	Female	SSG	20-Aug-05	INCREASED SGOT	ASPARTATE AMINOTRANSFERASE INCREASED	26-Aug-05	19-Sep-05	Resolved	Moderate	None	Probable
11	25	10	Female	SSG	20-Aug-05	INCREASED SGPT	ALANINE AMINOTRANSFERASE INCREASED	26-Aug-05	19-Sep-05	Resolved	Moderate	None	Probable
11	25	10	Female	SSG	20-Aug-05	INTESTINAL PARASITOSIS	INFECTION PARASITIC	4-Jan-06	14-Apr-06	Resolved	Mild	None	Not related
11	26	8	Female	Comb	25-Aug-05	INJECTION SITE PAIN	INJECTION SITE PAIN	31-Aug-05	7-Sep-05	Resolved	Mild	None	Probable
11	27	31	Male	PM	19-Sep-05	ACUTE GASTRO ENTERITIS	GASTROENTERITIS	20-Sep-05	23-Sep-05	Resolved	Mild	None	Not related
11	27	31	Male	PM	19-Sep-05	PAIN AT INJECTION SITE	INJECTION SITE PAIN	29-Sep-05		Ongoing	Moderate	None	Probable
11	27	31	Male	PM	19-Sep-05	HEADACHE	HEADACHE	23-Sep-05	25-Oct-05	Resolved	Mild	None	Unlikely
11	27	31	Male	PM	19-Sep-05	SEPSIS	SEPSIS	20-Jan-06	27-Jan-06	Resolved	Severe	None	Not related
11	28	13	Male	Comb	21-Sep-05	ACUTE GASTROENTERITIS	GASTROENTERITIS	24-Sep-05	24-Sep-05	Resolved	Mild	None	Not related
11	28	13	Male	Comb	21-Sep-05	PAIN AT INJECTION SITE	INJECTION SITE PAIN	27-Sep-05	8-Oct-05	Resolved	Mild	None	Possible
11	31	40	Female	SSG	23-Sep-05	ELEVATED LIVER ENZYMES (ASYMPTOMATIC)	HEPATIC ENZYME INCREASED	13-Oct-05		Ongoing	Mild	None	Possible
11	33	25	Male	SSG	25-Sep-05	ELEVATED LIVER ENZYMES	HEPATIC ENZYME INCREASED	1-Oct-05	15-Oct-05	Resolved	Mild	None	Possible
11	33	25	Male	SSG	25-Sep-05	REDUCED PLATELATE COUNT	PLATELET COUNT DECREASED	8-Oct-05		Ongoing	Mild	None	Possible
11	35	18	Male	SSG	27-Sep-05	EPISTAXIS	EPISTAXIS	19-Oct-05	21-Oct-05	Resolved	Mild	None	Probable
11	35	18	Male	SSG	27-Sep-05	DECREASE IN HGB LEVEL	HAEMOGLOBIN DECREASED	11-Jan-06	16-Apr-06	Resolved	Moderate	None	Possible
11	35	18	Male	SSG	27-Sep-05	DECREASE IN PLATELET COUNT	PLATELET COUNT DECREASED	11-Jan-06	16-Apr-06	Resolved	Moderate	None	Possible
11	35	18	Male	SSG	27-Sep-05	DECREASE IN WBC	WHITE BLOOD CELL DECREASED	11-Jan-06	16-Apr-06	Resolved	Moderate	None	Possible
11	36	20	Male	SSG	28-Sep-05	SEVERE ANEMIA	ANAEMIA	4-Oct-05	11-Oct-05	Resolved	Moderate	None	Unlikely
11	37	40	Male	Comb	28-Sep-05	VOMITING	VOMITING	3-Oct-05	4-Oct-05	Resolved	Mild	None	Unlikely
11	37	40	Male	Comb	28-Sep-05	ANOROXIA	ANOREXIA	3-Oct-05	4-Oct-05	Resolved	Mild	None	Unlikely
11	37	40	Male	Comb	28-Sep-05	VESICULAR SKIN LESION	RASH VESICULAR	7-Oct-05	15-Oct-05	Resolved	Mild	None	Not related
11	37	40	Male	Comb	28-Sep-05	INJECTION SITE PAIN	INJECTION SITE PAIN	7-Oct-05	15-Oct-05	Resolved	Mild	None	Probable
11	37	40	Male	Comb	28-Sep-05	STAGE II HYPERTENSION	HYPERTENSION	17-Jan-06		Ongoing	Moderate	None	Not related
11	38	47	Male	PM	19-Oct-05	ESOPHAGEAL VARICES	VARICES OESOPHAGEAL	29-May-06		Ongoing	Moderate	None	Not related
11	38	47	Male	PM	19-Oct-05	DUODENAL ULCER	DOUDENAL ULCER	29-May-06		Ongoing	Moderate	None	Not related

Cent No	Pat No	Age	Sex	Treatment	Treatment start date	Adverse Event	Preferred term	AE start date	AE end date	Outcome	Intensity	Action taken	Related
11	39	6	Male	PM	19-Oct-05	AUDIOMETRIC SHIFT	AUDIOGRAM ABNORMAL	7-Mar-06	22-May-06	Resolved	Moderate	None	Possible
11	39	6	Male	PM	19-Oct-05	SINUS ARRYTHMIA	SINUS ARRHYTHMIA	6-Mar-06		Ongoing	Mild	None	Possible
11	41	5	Male	Comb	20-Oct-05	MALARIA	MALARIA	22-Oct-05	24-Oct-05	Resolved	Mild	None	Not related
11	42	30	Male	Comb	21-Oct-05	ASYMPTOMATIC ELEVATION OF LIVER ENZYMES	HEPATIC ENZYME INCREASED	27-Oct-05	3-Nov-05	Resolved	Mild	None	Possible
11	42	30	Male	Comb	21-Oct-05	MALARIA (AMA	MALARIA	5-Nov-05	14-Nov-05	Resolved	Mild	None	Not related
11	42	30	Male	Comb	21-Oct-05	LOW BACK PAIN	BACK PAIN	26-Jan-06	4-Feb-06	Resolved	Mild	None	Not related
11	43	15	Male	SSG	28-Oct-05	UTI (PYURIA)	URINARY TRACT INFECTION	3-Nov-05	10-Nov-05	Resolved	Mild	None	Not related
11	45	16	Male	Comb	28-Oct-05	PAIN OVER INJECTION SITE	INJECTION SITE PAIN	10-Nov-05		Ongoing	Mild	None	Possible
11	45	16	Male	Comb	28-Oct-05	INTESTINAL PARASITOSIS	INFECTION PARASITIC	19-May-06	21-May-06	Resolved	Mild	None	Not related
11	46	6	Female	SSG	5-May-06	GIARDIASIS	GIARDIASIS	8-May-06	11-May-06	Resolved	Mild	None	Not related
11	47	20	Male	SSG	5-May-06	ELEVATED LIVER TRANSAMINASE	TRANSAMINASES INCREASED	11-May-06	18-May-06	Resolved	Mild	None	Possible
11	49	7	Male	SSG	9-May-06	ELEVATED LIVER TRANSAMINASE LEVEL	TRANSAMINASES INCREASED	15-May-06		Ongoing	Moderate	None	Possible
11	51	15	Male	Comb	20-May-06	INJECTION SITE PAIN	INJECTION SITE PAIN	30-May-06		Ongoing	Mild	None	Possible
11	52	11	Male	PM	20-May-06	RAISED TRANSAMINASE LEVEL (SGOT & SGPT)	TRANSAMINASES INCREASED	11-Jan-07	22-Jan-07	Resolved	Severe	None	Unlikely
11	53	26	Male	SSG	26-May-06	RAISED ALK. PHOSPHATASE LEVEL	BLOOD ALKALINE PHOSPHATASE INCREASED	8-Jun-06	12-Dec-06	Resolved	Moderate	None	Possible
11	54	24	Male	PM	27-May-06	AMOEBIASIS	AMOEBIASIS	23-Jun-06	2-Jul-06	Resolved	Moderate	None	Not related
11	54	24	Male	PM	27-May-06	MALARIA	MALARIA	18-Dec-06	21-Dec-06	Resolved	Moderate	None	Not related
11	54	24	Male	PM	27-May-06	DENTAL CARRIES	DENTAL CARIES	25-Dec-06	1-Jan-07	Resolved	Moderate	None	Not related
11	57	27	Male	Comb	4-Jun-06	RAISED TRANSAMINASE LEVEL	TRANSAMINASES INCREASED	14-Jun-06	21-Jun-06	Resolved	Moderate	None	Possible
11	57	27	Male	Comb	4-Jun-06	DYSPEPSIA	DYSPEPSIA	10-Jun-06	17-Jun-06	Resolved	Mild	None	Probable
11	58	12	Male	Comb	6-Jun-06	PAIN AT INJECTION SITE	INJECTION SITE PAIN	15-Jun-06	26-Sep-06	Resolved	Moderate	None	Possible
11	58	12	Male	Comb	6-Jun-06	RAISED TRANSAMINATE LEVEL	TRANSAMINASES INCREASED	19-Jun-06		Ongoing	Moderate	None	Possible
11	58	12	Male	Comb	6-Jun-06	RAISED SGOT	ASPARTATE AMINOTRANSFERASE INCREASED	19-Jun-06	13-Dec-06	Resolved	Moderate	None	Possible
11	58	12	Male	Comb	6-Jun-06	RAISED SGPT	ALANINE AMINOTRANSFERASE INCREASED	19-Jun-06	13-Dec-06	Resolved	Moderate	None	Possible
11	59	12	Male	PM	13-Jun-06	INJECTION SITE PAIN	INJECTION SITE PAIN	25-Jun-06	26-Aug-06	Resolved	Mild	None	Possible
11	59	12	Male	PM	13-Jun-06	INCREASED ALK PHOSPHATASE	BLOOD ALKALINE PHOSPHATASE INCREASED	4-Jul-06		Ongoing	Moderate	None	Possible
11	59	12	Male	PM	13-Jun-06	HYPERTENSION	HYPERTENSION	4-Jul-06		Ongoing	Moderate	None	Probable

Cent No	Pat No	Age	Sex	Treatment	Treatment start date	Adverse Event	Preferred term	AE start date	AE end date	Outcome	Intensity	Action taken	Related
11	59	12	Male	PM	13-Jun-06	RAISED SGOT	ASPARTATE AMINOTRANSFERASE INCREASED	26-Aug-06		Ongoing	Moderate	None	Probable
11	59	12	Male	PM	13-Jun-06	RAISED SGPT	ALANINE AMINOTRANSFERASE INCREASED	26-Aug-06		Ongoing	Moderate	None	Probable
11	59	12	Male	PM	13-Jun-06	RAISED TRANSAMINASE LEVEL (SGOT & SGPT)	TRANSAMINASES INCREASED	26-Aug-06		Ongoing	Moderate	None	Probable
11	60	32	Male	Comb	13-Jun-06	RAISED TRANSAMINASE LEVEL (SGOT & SGPT)	TRANSAMINASES INCREASED	19-Jun-06	28-Sep-06	Resolved	Mild	None	Possible
11	60	32	Male	Comb	13-Jun-06	RAISED ALK. PHOSPHATASE	BLOOD ALKALINE PHOSPHATASE INCREASED	19-Jun-06	18-Dec-06	Resolved	Moderate	None	Possible
11	60	32	Male	Comb	13-Jun-06	INJECTION SITE PAIN	INJECTION SITE PAIN	22-Jun-06	28-Sep-06	Resolved	Mild	None	Possible
11	61	27	Male	PM	13-Jun-06	INJECTION SITE PAIN	INJECTION SITE PAIN	24-Jun-06		Ongoing	Mild	None	Possible
11	62	6	Male	SSG	16-Jun-06	INJECTION SITE PAIN	INJECTION SITE PAIN	22-Jun-06		Ongoing	Mild	None	Possible
11	64	26	Male	SSG	22-Jun-06	INJECTION SITE PAIN	INJECTION SITE PAIN	6-Jul-06	29-Sep-06	Resolved	Mild	None	Possible
11	67	11	Male	PM	1-Jul-06	INJECTION SITE POIN	INJECTION SITE PAIN	14-Jul-06	3-Oct-06	Resolved	Mild	None	Possible
11	69	18	Male	Comb	15-Jul-06	INJECTION SITE PAIN	INJECTION SITE PAIN	27-Jul-06	1-Aug-06	Resolved	Mild	None	Possible
11	69	18	Male	Comb	15-Jul-06	GIARDIASIS	GIARDIASIS	31-Oct-06	13-Dec-06	Resolved	Moderate	None	Not related
11	70	20	Male	Comb	15-Jul-06	RAISED TRANSAMINASE LEVEL	TRANSAMINASES INCREASED	21-Jul-06		Ongoing	Mild	None	Possible
11	70	20	Male	Comb	15-Jul-06	INJECTION SITE PAIN	INJECTION SITE PAIN	25-Jul-06		Ongoing	Moderate	None	Possible
11	70	20	Male	Comb	15-Jul-06	SCABIES	ACARODERMATITIS	9-Oct-06	2-Jan-07	Resolved	Moderate	None	Not related
11	71	33	Male	Comb	22-Jul-06	INJECTION SITE PAIN	INJECTION SITE PAIN	4-Aug-06	20-Aug-06	Resolved	Mild	None	Possible
11	71	33	Male	Comb	22-Jul-06	DYSPEPSIA	DYSPEPSIA	30-Jul-06	8-Aug-06	Resolved	Moderate	None	Probable
11	71	33	Male	Comb	22-Jul-06	AMOEBIASIS	AMOEBIASIS	21-Aug-06	12-Oct-06	Resolved	Moderate	None	Not related
11	71	33	Male	Comb	22-Jul-06	DYSPEPSIE	DYSPEPSIA	1-Jan-07		Ongoing	Mild	None	Not related
11	71	33	Male	Comb	22-Jul-06	EPISTAXIS	EPISTAXIS	12-Feb-07		Ongoing	Mild	None	Not related
11	72	23	Male	SSG	22-Jul-06	RAISED TRANSAMINASE LEVEL	TRANSAMINASES INCREASED	28-Jul-06		Ongoing	Moderate	None	Possible
11	72	23	Male	SSG	22-Jul-06	RAISED ALK. PHOSPHATASE	BLOOD ALKALINE PHOSPHATASE INCREASED	28-Jul-06	9-Nov-06	Resolved	Moderate	None	Possible
11	72	23	Male	SSG	22-Jul-06	RAISED TRANSAMINASE LEVEL (SGOT)	ASPARTATE AMINOTRANSFERASE INCREASED	28-Jul-06	9-Nov-06	Resolved	Moderate	None	Possible
11	72	23	Male	SSG	22-Jul-06	SKIN RASH WHICH IS PAPULONODULAR OVER THE FACE (PKDL)	VISCERAL LEISHMANIASIS	9-Oct-06		Ongoing	Mild	None	Possible
11	74	34	Male	PM	28-Jul-06	RAISED TRANSAMINASE LEVEL (SGOT & SGPT)	TRANSAMINASES INCREASED	3-Aug-06	18-Aug-06	Resolved	Moderate	None	Possible
11	75	25	Male	Comb	28-Jul-06	INJECTION SITE PAIN	INJECTION SITE PAIN	3-Aug-06	29-Aug-06	Resolved	Mild	None	Possible
11	76	23	Male	PM	31-Jul-06	RAISED TRANSAMINASE LEVEL SGOT	ASPARTATE AMINOTRANSFERASE INCREASED	2-Aug-06	21-Aug-06	Resolved	Mild	None	Possible
11	78	30	Male	SSG	31-Jul-06	INJECTION SITE PAIN	INJECTION SITE PAIN	12-Aug-06	30-Oct-06	Resolved	Mild	None	Possible
11	79	5	Male	PM	31-Jul-06	ACUTE GESTROINTERITS	GASTROENTERITIS	5-Aug-06	6-Aug-06	Resolved	Mild	None	Possible

Cent No	Pat No	Age	Sex	Treatment	Treatment start date	Adverse Event	Preferred term	AE start date	AE end date	Outcome	Intensity	Action taken	Related
11	79	5	Male	PM	31-Jul-06	RAISED TRANSAMINASE LEVEL (SGOT)	ASPARTATE AMINOTRANSFERASE INCREASED	30-Oct-06	30-Jan-07	Resolved	Mild	None	Probable
11	81	25	Male	Comb	6-Aug-06	PAIN AT INJECTION SITE	INJECTION SITE PAIN	16-Aug-06	7-Nov-06	Resolved	Moderate	None	Possible
11	82	15	Male	SSG	15-Aug-06	RAISED TRANSAMINASE (SGOT)	ASPARTATE AMINOTRANSFERASE INCREASED	21-Aug-06	13-Nov-06	Resolved	Moderate	Dose Reduced	Possible
11	82	15	Male	SSG	15-Aug-06	INJECTION SITE PAIN	INJECTION SITE PAIN	4-Sep-06	13-Nov-06	Resolved	Mild	None	Possible
11	83	22	Male	SSG	16-Aug-06	PAPULAR RASH W/C ARE DISTRIBUTED OVER THE FACE [PKDL GRADE I]	VISCERAL LEISHMANIASIS	13-Dec-06		Ongoing	Mild	None	Possible
11	84	15	Male	SSG	18-Aug-06	ATYPICAL PNEUMONIA	PNEUMONIA ATYPICAL PRIMARY	10-Sep-06	15-Nov-06	Resolved	Moderate	None	Unlikely
11	85	20	Male	SSG	25-Aug-06	RAISED TRANSAMINASE LEVEL (SGOT)	ASPARTATE AMINOTRANSFERASE INCREASED	31-Aug-06	21-Nov-06	Resolved	Moderate	None	Possible
11	85	20	Male	SSG	25-Aug-06	RAISED ALK PHOSPHATASE	BLOOD ALKALINE PHOSPHATASE INCREASED	31-Aug-06	21-Nov-06	Resolved	Moderate	None	Possible
11	85	20	Male	SSG	25-Aug-06	INJECTION SITE PAIN	INJECTION SITE PAIN	7-Sep-06	21-Nov-06	Resolved	Moderate	None	Possible
11	85	20	Male	SSG	25-Aug-06	DYSPEPSIA	DYSPEPSIA	8-Nov-06		Ongoing	Moderate	None	Probable
11	86	5	Male	Comb	29-Aug-06	PAIN AT INJECTION SITE	INJECTION SITE PAIN	8-Sep-06	13-Nov-06	Resolved	Moderate	None	Possible
11	87	18	Male	Comb	29-Aug-06	RAISED TRANSAMINASE LEVEL (SGOT)	ASPARTATE AMINOTRANSFERASE INCREASED	4-Sep-06	14-Nov-06	Resolved	Moderate	None	Possible
11	88	25	Male	PM	29-Aug-06	ATYPICAL PNEUMONIA	PNEUMONIA ATYPICAL PRIMARY	14-Nov-06	28-Feb-07	Resolved	Mild	None	Not related
11	89	24	Male	Comb	2-Sep-06	RAISED TRANSAMINASE LEVEL (SGOT)	ASPARTATE AMINOTRANSFERASE INCREASED	5-Sep-06	17-Nov-06	Resolved	Moderate	None	Possible
11	89	24	Male	Comb	2-Sep-06	INJECTION SITE PAIN	INJECTION SITE PAIN	8-Sep-06	17-Nov-06	Resolved	Moderate	None	Possible
11	89	24	Male	Comb	2-Sep-06	VOMITING	VOMITING	5-Sep-06	5-Sep-06	Resolved	Mild	None	Possible
11	89	24	Male	Comb	2-Sep-06	ATYPICAL PNEUMONIA	PNEUMONIA ATYPICAL PRIMARY	17-Oct-06	15-Jan-07	Resolved	Moderate	None	Not related
11	91	35	Male	PM	18-Dec-06	PAIN AT INJECTION SITE	INJECTION SITE PAIN	29-Dec-06	5-Mar-07	Resolved	Mild	None	Possible
11	91	35	Male	PM	18-Dec-06	DYSPEPSIA HO PUD	DYSPEPSIA	9-Apr-07	3-Jul-07	Resolved	Moderate	None	Unlikely
11	91	35	Male	PM	18-Dec-06	INCREASED CREATINEN	BLOOD CREATININE INCREASED	15-Mar-07	6-Sep-07	Resolved	Moderate	None	Possible
11	92	7	Male	Comb	31-Dec-06	INJECTION SITE SWEALING	INJECTION SITE SWELLING	13-Jan-07		Ongoing	Mild	None	Possible
11	92	7	Male	Comb	31-Dec-06	INJECTION SITE PAIN	INJECTION SITE PAIN	13-Jan-07	30-Mar-07	Resolved	Mild	None	Possible
11	93	26	Male	SSG	2-Jan-07	INJECTION SITE PAIN	INJECTION SITE PAIN	15-Jan-07	1-Feb-07	Resolved	Mild	None	Possible
11	95	6	Male	SSG	4-Jan-07	RAISED TRANSAMINASE LEVEL (SGOT&SGPT)	TRANSAMINASES INCREASED	10-Jan-07	3-Feb-07	Resolved	Moderate	None	Possible

Cent No	Pat No	Age	Sex	Treatment	Treatment start date	Adverse Event	Preferred term	AE start date	AE end date	Outcome	Intensity	Action taken	Related
11	95	6	Male	SSG	4-Jan-07	RAISED ALK P	BLOOD ALKALINE PHOSPHATASE INCREASED	10-Jan-07	3-Feb-07	Resolved	Moderate	None	Possible
11	96	12	Male	Comb	20-Jan-07	RAISED LIVER ENZYME (SGOT)	ASPARTATE AMINOTRANSFERASE INCREASED	26-Jan-07	23-Apr-07	Resolved	Moderate	None	Possible
11	96	12	Male	Comb	20-Jan-07	INJECTION SITE PAIN	INJECTION SITE PAIN	26-Jan-07	23-Apr-07	Resolved	Moderate	None	Possible
11	96	12	Male	Comb	20-Jan-07	RAISED LIVER ENZYME (SGPT)	ALANINE AMINOTRANSFERASE INCREASED	29-Jan-07	23-Apr-07	Resolved	Moderate	None	Possible
11	97	19	Male	Comb	20-Jan-07	RAISED ALK. PHOSPHATASE	BLOOD ALKALINE PHOSPHATASE INCREASED	26-Jan-07		Ongoing	Moderate	None	Possible
11	98	20	Male	PM	24-Jan-07	RAISED TRANSAMINASE LEVEL (SGOT)	ASPARTATE AMINOTRANSFERASE INCREASED	6-Feb-07		Ongoing	Mild	None	Possible
11	98	20	Male	PM	24-Jan-07	URINARY TRACT INFECTION	URINARY TRACT INFECTION	8-Feb-07	15-Feb-07	Resolved	Moderate	None	Not related
11	98	20	Male	PM	24-Jan-07	RAISED TRANSAMINE LEVEL (SGPT)	ALANINE AMINOTRANSFERASE INCREASED	25-Apr-07	26-Jul-07	Resolved	Mild	None	Unlikely
11	100	36	Male	PM	26-Jan-07	INJECTION SITE PAIN	INJECTION SITE PAIN	5-Feb-07		Ongoing	Mild	None	Possible
11	101	22	Male	PM	3-Feb-07	RAISED ALK. PHOSPHATASE	BLOOD ALKALINE PHOSPHATASE INCREASED	9-Feb-07	6-Nov-07	Resolved	Mild	None	Possible
11	101	22	Male	PM	3-Feb-07	INJECTION SITE PAIN	INJECTION SITE PAIN	13-Feb-07	24-May-07	Resolved	Mild	None	Possible
11	103	28	Male	Comb	7-Feb-07	RAISED TRANSAMINASE LEVEL (SGOT SGPT)	TRANSAMINASES INCREASED	13-Feb-07	7-May-07	Resolved	Mild	None	Possible
11	103	28	Male	Comb	7-Feb-07	INJECTION SITE PAIN	INJECTION SITE PAIN	16-Feb-07	7-May-07	Resolved	Mild	None	Possible
11	104	13	Female	PM	10-Feb-07	RAISED TRANSAMINASE LEVEL (SGOT)	ASPARTATE AMINOTRANSFERASE INCREASED	16-Feb-07	20-Feb-07	Resolved	Moderate	None	Possible
11	104	13	Female	PM	10-Feb-07	RAISED ALK P	BLOOD ALKALINE PHOSPHATASE INCREASED	16-Feb-07	3-Mar-07	Resolved	Moderate	None	Possible
11	105	18	Male	SSG	24-Feb-07	GRADE I PKDL	VISCERAL LEISHMANIASIS	30-Apr-07		Ongoing	Moderate	None	Possible
11	106	18	Male	Comb	25-Feb-07	INJECTION SITE PAIN	INJECTION SITE PAIN	6-Mar-07	29-Mar-07	Resolved	Moderate	None	Possible
11	108	25	Male	PM	2-Mar-07	VIVAX MALARIA	MALARIA	23-Mar-07	25-Mar-07	Resolved	Mild	None	Not related
11	109	19	Male	SSG	3-Mar-07	ACUTE GASTRO ENTERITES	GASTROENTERITIS	9-Mar-07	10-Mar-07	Resolved	Mild	None	Unlikely
11	109	19	Male	SSG	3-Mar-07	GIARDIASIS	GIARDIASIS	28-Mar-07	2-Apr-07	Resolved	Mild	None	Not related
11	109	19	Male	SSG	3-Mar-07	ATYPICAL PNEUMONIA	PNEUMONIA ATYPICAL PRIMARY	26-Mar-07	2-Apr-07	Resolved	Mild	None	Not related
11	109	19	Male	SSG	3-Mar-07	HOOKWORM	HOOKWORM INFECTION	28-Mar-07	2-Apr-07	Resolved	Mild	None	Not related

Cent No	Pat No	Age	Sex	Treatment	Treatment start date	Adverse Event	Preferred term	AE start date	AE end date	Outcome	Intensity	Action taken	Related
11	110	6	Female	PM	3-Mar-07	RAISED TRANSAMINE (SGOT, SGPT)	TRANSAMINASES INCREASED	29-May-07		Ongoing	Moderate	None	Probable
11	110	6	Female	PM	3-Mar-07	RAISED ALK P	BLOOD ALKALINE PHOSPHATASE INCREASED	24-Mar-07	27-Aug-07	Resolved	Mild	None	Possible
11	110	6	Female	PM	3-Mar-07	RAISED SGOT	ASPARTATE AMINOTRANSFERASE INCREASED	29-May-07	27-Aug-07	Resolved	Moderate	None	Probable
11	110	6	Female	PM	3-Mar-07	RAISED SGPT	ALANINE AMINOTRANSFERASE INCREASED	29-May-07	27-Aug-07	Resolved	Moderate	None	Probable
11	111	19	Male	Comb	24-Mar-07	RAISED TRANSAMINASE LEVEL (SGOT/SGPT	TRANSAMINASES INCREASED	30-Mar-07	22-Jun-07	Resolved	Mild	None	Possible
11	111	19	Male	Comb	24-Mar-07	RAISED ALK P	BLOOD ALKALINE PHOSPHATASE INCREASED	30-Mar-07	22-Jun-07	Resolved	Moderate	None	Possible
11	112	28	Male	Comb	24-Mar-07	RAISED ALK. PHO.	BLOOD ALKALINE PHOSPHATASE INCREASED	30-Mar-07	30-Jul-07	Resolved	Moderate	None	Possible
11	112	28	Male	Comb	24-Mar-07	INJECTION SITE PAIN	INJECTION SITE PAIN	30-Mar-07	2-May-07	Resolved	Moderate	None	Possible
11	113	18	Male	SSG	28-Mar-07	RAISED TRANSAMIN (SGOT)	ASPARTATE AMINOTRANSFERASE INCREASED	3-Apr-07	29-Jun-07	Resolved	Moderate	None	Possible
11	113	18	Male	SSG	28-Mar-07	RAISED ALK. PHOS.	BLOOD ALKALINE PHOSPHATASE INCREASED	3-Apr-07		Ongoing	Moderate	None	Possible
11	113	18	Male	SSG	28-Mar-07	TAENIASIS	TAENIASIS	3-Apr-07	8-Apr-07	Resolved	Mild	None	Not related
11	113	18	Male	SSG	28-Mar-07	URTI	UPPER RESPIRATORY TRACT INFECTION	16-Apr-07	1-Jun-07	Resolved	Moderate	None	Not related
11	113	18	Male	SSG	28-Mar-07	INJECTION SITE PAIN	INJECTION SITE PAIN	20-Apr-07		Ongoing	Moderate	None	Possible
11	114	25	Male	SSG	30-Mar-07	RAISED TRANSAMINE (SGOT, SGPT)	TRANSAMINASES INCREASED	5-Apr-07		Ongoing	Mild	None	Possible
11	114	25	Male	SSG	30-Mar-07	RAISED ALK.P	BLOOD ALKALINE PHOSPHATASE INCREASED	5-Apr-07	5-Oct-07	Resolved	Mild	None	Possible
11	114	25	Male	SSG	30-Mar-07	RAISED TRANSAMINASE SGPT	ALANINE AMINOTRANSFERASE INCREASED	5-Apr-07	2-Jul-07	Resolved	Mild	None	Possible
11	114	25	Male	SSG	30-Mar-07	RAISED TRANSAMINE SGOT - LEVEL	ASPARTATE AMINOTRANSFERASE INCREASED	5-Apr-07		Ongoing	Mild	None	Possible
11	115	12	Male	Comb	31-Mar-07	ABDOMINAL CRAMP	ABDOMINAL PAIN	2-Apr-07	6-Apr-07	Resolved	Mild	None	Unlikely
11	115	12	Male	Comb	31-Mar-07	INJECTION SITE PAIN	INJECTION SITE PAIN	6-Apr-07	1-May-07	Resolved	Moderate	None	Possible

Cent No	Pat No	Age	Sex	Treatment	Treatment start date	Adverse Event	Preferred term	AE start date	AE end date	Outcome	Intensity	Action taken	Related
11	116	23	Male	SSG	31-Mar-07	HERPES ZOSTER	HERPES ZOSTER	26-Apr-07	8-May-07	Resolved	Moderate	None	Not related
11	117	22	Male	PM	4-Apr-07	URINARY TRACT INFECTION (UTI)	URINARY TRACT INFECTION	14-Apr-07	23-Apr-07	Resolved	Moderate	None	Not related
11	117	22	Male	PM	4-Apr-07	PERIPHERAL NEUROPATHY	NEUROPATHY PERIPHERAL	22-Apr-07	25-May-07	Resolved	Mild	None	Probable
11	118	25	Male	PM	19-Apr-07	RAISED TRANSAMINASE LEVEL SGOT/SGPT	TRANSAMINASES INCREASED	2-May-07	6-Jul-07	Resolved	Mild	None	Possible
11	118	25	Male	PM	19-Apr-07	INJECTION SITE PAIN	INJECTION SITE PAIN	25-Apr-07	12-Jun-07	Resolved	Mild	None	Possible
11	119	26	Male	SSG	21-Apr-07	PAIN AT INJECTION SITE	INJECTION SITE PAIN	3-May-07	15-May-07	Resolved	Mild	None	Possible
11	120	17	Male	Comb	21-Apr-07	INJECTION SITE PAIN	INJECTION SITE PAIN	26-Apr-07	23-Jun-07	Resolved	Moderate	None	Possible
11	122	20	Male	SSG	21-Apr-07	RAISED ALK P	BLOOD ALKALINE PHOSPHATASE INCREASED	27-Apr-07		Ongoing	Moderate	None	Possible
11	122	20	Male	SSG	21-Apr-07	PYOGENIC LYMPHADENITIS	LYMPHADENITIS	4-May-07	20-May-07	Resolved	Moderate	None	Not related
11	123	14	Male	SSG	28-Apr-07	SWELLING OF LEFT FOREARM [CELLULITIS]	CELLULITIS	15-May-07	27-May-07	Resolved	Moderate	None	Not related
11	124	30	Male	SSG	21-May-07	RAISED TRANSAMINASE LEVEL [SGOT]	ASPARTATE AMINOTRANSFERASE INCREASED	27-May-07	10-Jun-07	Resolved	Mild	None	Possible
11	124	30	Male	SSG	21-May-07	TRACE PEDAL AND PRETIBIA EDEMA (NUTRITIONAL EDEMA)	PERIPHERAL OEDEMA	1-Jun-07	10-Jun-07	Resolved	Mild	None	Not related
11	124	30	Male	SSG	21-May-07	CHICKENPOX	VARICELLA	13-Jun-07	24-Jun-07	Resolved	Moderate	None	Not related
11	126	23	Male	Comb	28-May-07	RAISED TRANSAMINASE (SGOT)	ASPARTATE AMINOTRANSFERASE INCREASED	6-Jun-07	10-Dec-07	Resolved	Mild	None	Possible
11	126	23	Male	Comb	28-May-07	INJECTION SITE PAIN	INJECTION SITE PAIN	5-Jun-07	20-Jun-07	Resolved	Moderate	None	Possible
11	126	23	Male	Comb	28-May-07	URTI	UPPER RESPIRATORY TRACT INFECTION	9-Jul-07	6-Aug-07	Resolved	Mild	None	Not related
11	126	23	Male	Comb	28-May-07	SGPT (RAISED)	ALANINE AMINOTRANSFERASE INCREASED	9-Jul-07	10-Dec-07	Resolved	Mild	None	Probable
11	126	23	Male	Comb	28-May-07	DYSPEPSIA	DYSPEPSIA	10-Nov-07	13-Mar-08	Resolved	Mild	None	Not related
11	127	14	Female	PM	28-May-07	RAISED TRANSAMINASE (SGOT & SGPT)	TRANSAMINASES INCREASED	10-Jun-07	27-Aug-07	Resolved	Mild	None	Possible
11	128	30	Male	Comb	2-Jun-07	INJECTION SITE PAIN	INJECTION SITE PAIN	7-Jun-07	25-Jun-07	Resolved	Mild	None	Possible
11	128	30	Male	Comb	2-Jun-07	RAISED (SGOT)	ASPARTATE AMINOTRANSFERASE INCREASED	8-Jun-07		Ongoing	Mild	None	Possible

Cent No	Pat No	Age	Sex	Treatment	Treatment start date	Adverse Event	Preferred term	AE start date	AE end date	Outcome	Intensity	Action taken	Related
11	128	30	Male	Comb	2-Jun-07	URTI	UPPER RESPIRATORY TRACT INFECTION	8-Jun-07	14-Aug-07	Resolved	Mild	None	Not related
11	128	30	Male	Comb	2-Jun-07	RAISED CREATININE	BLOOD CREATININE INCREASED	15-Jun-07	19-Jul-07	Resolved	Mild	None	Possible
11	129	31	Male	PM	7-Jun-07	ALBUMINURIA (2+)	ALBUMINURIA	13-Jun-07	20-Jun-07	Resolved	Moderate	None	Possible
11	129	31	Male	PM	7-Jun-07	INJECTION SITE PAIN	INJECTION SITE PAIN	24-Jun-07	28-Jun-07	Resolved	Mild	None	Possible
11	130	25	Male	Comb	9-Jun-07	RAISED TRANSAMINASE [SGOT]	ASPARTATE AMINOTRANSFERASE INCREASED	15-Jun-07	19-Nov-07	Resolved	Mild	None	Possible
11	130	25	Male	Comb	9-Jun-07	DYSPEPSIA	DYSPEPSIA	14-Jun-07	17-Sep-07	Resolved	Mild	None	Possible
11	132	20	Male	SSG	16-Jun-07	ABDOMINAL CRAMP	ABDOMINAL PAIN	22-Jun-07	27-Jun-07	Resolved	Mild	None	Probable
11	132	20	Male	SSG	16-Jun-07	MALARIA	MALARIA	19-Nov-07	21-Nov-07	Resolved	Moderate	None	Not related
11	133	20	Male	Comb	4-Jul-07	INJECTION SITE PAIN	INJECTION SITE PAIN	12-Jul-07	21-Jul-07	Resolved	Mild	None	Possible
11	135	17	Male	PM	13-Jul-07	INJECTION SITE PAIN	INJECTION SITE PAIN	21-Jul-07	17-Aug-07	Resolved	Mild	None	Possible
12	241	20	Male	Comb	29-Jun-05	INJECTION SITE CELLULITIS	INJECTION SITE CELLULITIS	12-Jul-05	16-Jul-05	Resolved	Mild	None	Possible
12	242	7	Male	SSG	29-Jun-05	URINARY TRACT INFECTION	URINARY TRACT INFECTION	17-Jul-05	22-Jul-05	Resolved	Mild	None	Not related
12	242	7	Male	SSG	29-Jun-05	CONJECTIVITIS	CONJUNCTIVITIS	23-Oct-05	29-Oct-05	Resolved	Mild	None	Not related
12	242	7	Male	SSG	29-Jun-05	SKIN LESION	SKIN LESION	9-Feb-06	16-Feb-06	Resolved	Mild	None	Unlikely
12	244	20	Male	SSG	29-Jun-05	DYSENTRY	DYSENTRY	8-Jul-05	12-Jul-05	Resolved	Moderate	None	Unlikely
12	244	20	Male	SSG	29-Jun-05	URINARY TRACT INFECTION	URINARY TRACT INFECTION	19-Jul-05	24-Jul-05	Resolved	Mild	None	Unlikely
12	244	20	Male	SSG	29-Jun-05	FOOT ULCER	SKIN ULCER	29-Oct-05	4-Nov-05	Resolved	Mild	None	Not related
12	244	20	Male	SSG	29-Jun-05	CONJUCTIVITIS	CONJUNCTIVITIS	2-Apr-06	7-Apr-06	Resolved	Mild	None	Not related
12	245	36	Male	PM	20-Jul-05	FEVER	PYREXIA	18-Nov-05	5-Dec-05	Resolved	Moderate	None	Possible
12	245	36	Male	PM	20-Jul-05	PNEUMONIA	COUGH	28-Nov-05	4-Dec-05	Resolved	Moderate	None	Not related
12	246	40	Male	PM	4-Aug-05	ACUTE GASTRO ENTERITIES	GASTROENTERITIS	21-Aug-05	23-Aug-05	Resolved	Mild	None	Unlikely
12	246	40	Male	PM	4-Aug-05	HEADACHE	HEADACHE	24-Aug-05	25-Aug-05	Resolved	Mild	None	Unlikely
12	246	40	Male	PM	4-Aug-05	BACK PAIN	BACK PAIN	24-Aug-05	25-Aug-05	Resolved	Mild	None	Unlikely
12	246	40	Male	PM	4-Aug-05	CHRONIC DIARRHEA	DIARRHOEA	21-Aug-05	25-Nov-05	Resolved	Moderate	None	Unlikely
12	246	40	Male	PM	4-Aug-05	DYSENTRY	DYSENTRY	4-Nov-05	11-Nov-05	Resolved	Moderate	None	Not related
12	246	40	Male	PM	4-Aug-05	TENIA CORPORIS	BODY TINEA	4-Nov-05	22-Nov-05	Resolved	Mild	None	Not related
12	247	11	Male	PM	28-Sep-05	INJECTION SITE PAIN	INJECTION SITE PAIN	10-Oct-05	13-Oct-05	Resolved	Mild	None	Possible
12	247	11	Male	PM	28-Sep-05	PNEUMONIA	PNEUMONIA	19-Oct-05	22-Oct-05	Resolved	Mild	None	Not related
12	249	16	Male	Comb	25-Oct-05	PNEUMONIA	PNEUMONIA	1-Mar-06		Ongoing	Moderate	None	Not related
12	249	16	Male	Comb	25-Oct-05	FEVER	PYREXIA	1-Mar-06	8-Mar-06	Resolved	Moderate	None	Not related
12	250	15	Male	SSG	25-Oct-05	OTITIS MEDIA	OTITIS MEDIA	17-Nov-05	22-Nov-05	Resolved	Mild	None	Not related
12	251	18	Male	Comb	29-Oct-05	OTITIS MEDIA	OTITIS MEDIA	11-Nov-05	13-Nov-05	Resolved	Moderate	None	Unlikely
12	251	18	Male	Comb	29-Oct-05	PAIN ON INJECTION SITE	INJECTION SITE PAIN	10-Nov-05	13-Nov-05	Resolved	Mild	None	Possible
12	253	9	Male	SSG	29-Oct-05	COMMON COLD	NASOPHARYNGITIS	8-Nov-05	11-Nov-05	Resolved	Mild	None	Not related
12	254	8	Male	Comb	29-Oct-05	PAIN ON INJECTION SITE	INJECTION SITE PAIN	11-Nov-05	12-Nov-05	Resolved	Mild	None	Possible
12	261	13	Male	Comb	3-Jan-06	INJECTION SITE CELLULITIS	INJECTION SITE CELLULITIS	16-Jan-06	20-Jan-06	Resolved	Moderate	None	Possible
12	261	13	Male	Comb	3-Jan-06	AN AUDIOMETRIC SHIFT	AUDIOGRAM ABNORMAL	22-Jul-06		Ongoing	Moderate	None	Unlikely

Cent No	Pat No	Age	Sex	Treatment	Treatment start date	Adverse Event	Preferred term	AE start date	AE end date	Outcome	Intensity	Action taken	Related
12	261	13	Male	Comb	3-Jan-06	AUDIOMETRIC SHIFT FROM MODERATE TO SEVERE HEARING LOSS	DEAFNESS	19-Apr-06		Ongoing	Moderate	None	Unlikely
12	264	9	Male	Comb	15-Feb-06	INJECTION SITE CELLULITIS	INJECTION SITE CELLULITIS	4-Mar-06	8-Mar-06	Resolved	Mild	None	Probable
12	265	5	Female	SSG	15-Feb-06	CHEILOSIS	CHEILOSIS	25-Feb-06	2-Mar-06	Resolved	Moderate	None	Unlikely
12	265	5	Female	SSG	15-Feb-06	URINARY TRACT INFECTION	URINARY TRACT INFECTION	28-Feb-06	4-Mar-06	Resolved	Mild	None	Unlikely
12	265	5	Female	SSG	15-Feb-06	HOSPITAL ACQUIRED PNEUMONIA	PNEUMONIA	12-Mar-06	18-Mar-06	Resolved	Moderate	Drug interupt	Probable
12	265	5	Female	SSG	15-Feb-06	THROMBOCYTOPENIA	THROMBOCYTOPENIA	14-Mar-06		Ongoing	Severe	Drug	Possible
12	268	28	Male	PM	10-Mar-06	PAIN AT THE INJECTION SITE	INJECTION SITE PAIN	23-Mar-06	23-Mar-06	Resolved	Mild	None	Possible
12	280	5	Male	SSG	21-Apr-06	RAISED LIVER FUNCTION TEST (SG0T)	ASPARTATE AMINOTRANSFERASE INCREASED	27-Apr-06	25-Aug-06	Resolved	Mild	None	Possible
12	280	5	Male	SSG	21-Apr-06	RAISED LIVER FUNCTION TEST (SGPT)	ALANINE AMINOTRANSFERASE INCREASED	27-Apr-06		Ongoing	Mild	None	Possible
12	284	9	Female	Comb	20-May-06	EPISTAXIS	EPISTAXIS	9-Dec-06	10-Dec-06	Resolved	Mild	None	Unlikely
12	297	29	Male	SSG	12-Apr-07	RAISED SERUM AMYLASE LEVEL	BLOOD AMYLASE INCREASED	18-Apr-07		Ongoing	Moderate	None	Possible
12	301	25	Male	SSG	5-Jun-07	RAISED SERUM AMYLASE	BLOOD AMYLASE INCREASED	5-Jul-07		Ongoing	Mild	None	Possible
12	303	25	Female	PM	5-Jun-07	CONJUCTIVITIS	CONJUNCTIVITIS	1-Oct-07	5-Oct-07	Resolved	Mild	None	Not related
12	307	39	Male	Comb	15-Jun-07	GASTRITIS	GASTRITIS	22-Jun-07	26-Jun-07	Resolved	Mild	None	Unlikely
12	307	39	Male	Comb	15-Jun-07	RAISED SERUM AMYLASE TO 245	BLOOD AMYLASE INCREASED	28-Jun-07		Ongoing	Mild	None	Possible
12	307	39	Male	Comb	15-Jun-07	RIGHT SHOULDER PAIN	MUSCULOSKELETAL PAIN	1-Oct-07	4-Oct-07	Resolved	Mild	None	Unlikely
12	307	39	Male	Comb	15-Jun-07	RAISED SGOT	ASPARTATE AMINOTRANSFERASE INCREASED	1-Oct-07		Ongoing	Mild	None	Unlikely
12	307	39	Male	Comb	15-Jun-07	RAISED SGPT	ALANINE AMINOTRANSFERASE INCREASED	1-Oct-07		Ongoing	Mild	None	Unlikely
12	307	39	Male	Comb	15-Jun-07	RAISED SERUM CREATININE	BLOOD CREATININE INCREASED	26-Dec-07		Ongoing	Mild	None	Unlikely
12	308	31	Male	PM	15-Jun-07	GASTRITIS	GASTRITIS	18-Jun-07	22-Jun-07	Resolved	Mild	None	Unlikely
12	308	31	Male	PM	15-Jun-07	PNEUMONIA	PNEUMONIA	10-Sep-07	20-Oct-07	Resolved	Mild	None	Unlikely
12	308	31	Male	PM	15-Jun-07	CHRONIC SINUSITIS	CHRONIC SINUSITIS	26-Nov-07	31-Dec-07	Resolved	Mild	None	Not related
12	310	13	Female	SSG	16-Jun-07	INJECTION SITE PAIN	INJECTION SITE PAIN	6-Jul-07	16-Jul-07	Resolved	Mild	None	Possible
12	312	20	Female	Comb	12-Jul-07	RAISED SERUM AMYLASE	BLOOD AMYLASE INCREASED	18-Jul-07		Ongoing	Mild	None	Possible

Cent No	Pat No	Age	Sex	Treatment	Treatment start date	Adverse Event	Preferred term	AE start date	AE end date	Outcome	Intensity	Action taken	Related
12	312	20	Female	Comb	12-Jul-07	RAISED SGOT	ASPARTATE AMINOTRANSFERASE INCREASED	18-Jul-07	25-Jul-07	Resolved	Mild	None	Unlikely
12	313	25	Male	PM	2-Aug-07	RAISED SGOT AND SGPT	TRANSAMINASES INCREASED	15-Aug-07	23-Aug-07	Resolved	Mild	None	Possible
12	313	25	Male	PM	2-Aug-07	RAISED LIVER FUNCTION TEST(SGOT)	ASPARTATE AMINOTRANSFERASE INCREASED	22-Nov-07		Ongoing	Mild	None	Not related
12	313	25	Male	PM	2-Aug-07	RAISED SERUM LEVEL OF SGPT	ALANINE AMINOTRANSFERASE INCREASED	22-Nov-07		Ongoing	Mild	None	Unlikely
12	313	25	Male	PM	2-Aug-07	AUDIOMETRIC SHIFT	AUDIOGRAM ABNORMAL	21-Feb-08		Ongoing	Mild	None	Unlikely
12	314	18	Male	SSG	7-Aug-07	RAISED SERUM AMYLASE	BLOOD AMYLASE INCREASED	13-Aug-07		Ongoing	Moderate	None	Possible
12	315	10	Male	Comb	29-Aug-07	RAISED SGPT	ALANINE AMINOTRANSFERASE INCREASED	11-Aug-08		Ongoing	Mild	None	Not related
12	316	36	Male	SSG	29-Aug-07	RAISED SERUM CREATININE	BLOOD CREATININE INCREASED	28-Dec-07		Ongoing	Mild	None	Unlikely
12	317	4	Male	SSG	29-Aug-07	RAISED SERUM LEVEL OF SG0T	ASPARTATE AMINOTRANSFERASE INCREASED	4-Sep-07	22-Apr-08	Resolved	Mild	None	Possible
12	317	4	Male	SSG	29-Aug-07	RAISED SERUM LEVEL OF SGPT	ALANINE AMINOTRANSFERASE INCREASED	4-Sep-07		Ongoing	Mild	None	Possible
12	319	8	Male	Comb	28-Aug-07	RAISED LIVER FUNCTION TEST (SGOT)	ASPARTATE AMINOTRANSFERASE INCREASED	11-Sep-07		Ongoing	Mild	None	Unlikely
12	320	8	Male	Comb	6-Sep-07	RAISED SERUM SGOT	ASPARTATE AMINOTRANSFERASE INCREASED	19-Mar-08		Ongoing	Mild	None	Not related
12	321	8	Male	Comb	6-Sep-07	INJECTION SITE PAIN	INJECTION SITE PAIN	12-Sep-07	21-Sep-07	Resolved	Mild	None	Possible
12	321	8	Male	Comb	6-Sep-07	GIARDIASIS	GIARDIASIS	19-Sep-07	24-Sep-07	Resolved	Mild	None	Not related
12	322	16	Female	PM	13-Sep-07	GIARDIASIS	GIARDIASIS	25-Sep-07	1-Oct-07	Resolved	Mild	None	Unlikely
12	322	16	Female	PM	13-Sep-07	AMOEBIASIS	AMOEBIASIS	25-Sep-07	1-Oct-07	Resolved	Mild	None	Unlikely
12	322	16	Female	PM	13-Sep-07	RAISED SGOT	ASPARTATE AMINOTRANSFERASE INCREASED	26-Sep-07	4-Oct-07	Resolved	Mild	None	Unlikely

Appendix 44: Listing of adverse events per patient

Cent No	Pat No	Age	Sex	Treatment	Treatment start date	Adverse Event	Preferred term	AE start date	AE end date	Outcome	Intensity	Action taken	Related
12	322	16	Female	PM	13-Sep-07	RAISED LIVER FUNCTION TEST (SGOT)	ASPARTATE AMINOTRANSFERASE INCREASED	16-Apr-08		Ongoing	Mild	None	Not related
12	322	16	Female	PM	13-Sep-07	RAISED SGPT	ALANINE AMINOTRANSFERASE INCREASED	16-Apr-08		Ongoing	Mild	None	Possible
12	323	32	Male	SSG	20-Sep-07	INJECTION SITE PAIN	INJECTION SITE PAIN	3-Oct-07	5-Oct-07	Resolved	Mild	None	Probable
12	323	32	Male	SSG	20-Sep-07	RAISED SGPT	ALANINE AMINOTRANSFERASE INCREASED	26-Sep-07		Ongoing	Mild	None	Possible
12	323	32	Male	SSG	20-Sep-07	RAISED SGOT	ASPARTATE AMINOTRANSFERASE INCREASED	3-Oct-07	20-Oct-07	Resolved	Mild	None	Possible
12	323	32	Male	SSG	20-Sep-07	RAISED LIVER FUNCTION TEST (SGPT)	ALANINE AMINOTRANSFERASE INCREASED	16-Apr-08		Ongoing	Mild	None	Not related
12	323	32	Male	SSG	20-Sep-07	RAISED SGOT	ASPARTATE AMINOTRANSFERASE INCREASED	16-Apr-08		Ongoing	Mild	None	Possible
12	324	30	Male	SSG	20-Sep-07	RAISED SGOT LEVEL	ASPARTATE AMINOTRANSFERASE INCREASED	26-Sep-07	20-Oct-07	Resolved	Mild	None	Possible
12	324	30	Male	SSG	20-Sep-07	RAISED SERUM AMYLASE	BLOOD AMYLASE INCREASED	26-Sep-07		Ongoing	Mild	None	Possible
12	324	30	Male	SSG	20-Sep-07	AMOEBIASIS	AMOEBIASIS	4-Oct-07	11-Oct-07	Resolved	Mild	None	Not related
12	324	30	Male	SSG	20-Sep-07	RAISED SERUM AMYLASE	BLOOD AMYLASE INCREASED	16-Apr-08		Ongoing	Mild	None	Not related
12	325	22	Male	PM	27-Sep-07	RAISED LIVER FUNCTION TEST (SG0T)	ASPARTATE AMINOTRANSFERASE INCREASED	3-Oct-07		Ongoing	Mild	None	Possible
12	325	22	Male	PM	27-Sep-07	RAISED LIVER FUNCTION TEST (SGPT)	ALANINE AMINOTRANSFERASE INCREASED	3-Oct-07		Ongoing	Mild	None	Possible
12	325	22	Male	PM	27-Sep-07	RAISED LIVER FUNCTION TEST (SGPT)	ALANINE AMINOTRANSFERASE INCREASED	16-Apr-08		Ongoing	Mild	None	Not related
12	325	22	Male	PM	27-Sep-07	RAISED SGOT	ASPARTATE AMINOTRANSFERASE INCREASED	16-Apr-08		Ongoing	Mild	None	Possible

Cent No	Pat No	Age	Sex	Treatment	Treatment start date	Adverse Event	Preferred term	AE start date	AE end date	Outcome	Intensity	Action taken	Related
12	326	25	Male	PM	3-Oct-07	ASCARIASIS	ASCARIASIS	9-Oct-07	11-Oct-07	Resolved	Mild	None	Not related
12	326	25	Male	PM	3-Oct-07	OTITIS MEDIA	OTITIS MEDIA	13-Oct-07	17-Oct-07	Resolved	Moderate	None	Unlikely
12	326	25	Male	PM	3-Oct-07	RAISED SERUM CREATININ	BLOOD CREATININE INCREASED	24-Oct-07	29-Oct-07	Resolved	Mild	None	Possible
12	326	25	Male	PM	3-Oct-07	AUDIOMETRIC SHIFT OF THE RIGHT EAR	AUDIOGRAM ABNORMAL	16-Oct-07		Ongoing	Mild	None	Possible
12	327	14	Male	Comb	3-Oct-07	RAISED SGOT LEVEL	ASPARTATE AMINOTRANSFERASE INCREASED	9-Oct-07		Ongoing	Mild	None	Possible
12	328	14	Male	PM	5-Oct-07	INCREASED LIVER FUNTION TEST (SGOT)	ASPARTATE AMINOTRANSFERASE INCREASED	11-Oct-07		Ongoing	Mild	None	Possible
12	328	14	Male	PM	5-Oct-07	INCREASED LIVER FUNTION TEST (SGPT)	ALANINE AMINOTRANSFERASE INCREASED	11-Oct-07		Ongoing	Mild	None	Possible
12	329	6	Male	SSG	10-Oct-07	RAISED SGOT	ASPARTATE AMINOTRANSFERASE INCREASED	16-Oct-07		Ongoing	Mild	None	Possible
12	329	6	Male	SSG	10-Oct-07	OTITIS MEDIA	OTITIS MEDIA	18-Oct-07	23-Oct-07	Resolved	Mild	None	Not related
12	330	11	Male	PM	10-Oct-07	RAISED LIVER FUNCTION TES (SGOT)	ASPARTATE AMINOTRANSFERASE INCREASED	16-Oct-07		Ongoing	Mild	None	Unlikely
12	330	11	Male	PM	10-Oct-07	RAISED LIVER FUNCTION TES (SGPT)	ALANINE AMINOTRANSFERASE INCREASED	23-Oct-07	31-Oct-07	Resolved	Mild	None	Unlikely
23	361	8	Male	Comb	20-Jan-05	MICROSCOPIC HAEMATURIA	HAEMATURIA	27-Jan-05	2-Feb-05	Resolved	Mild	None	Not related
23	361	8	Male	Comb	20-Jan-05	INCREASED URINE WBC	WHITE BLOOD CELLS URINE POSITIVE	27-Jan-05	2-Feb-05	Resolved	Mild	None	Not related
23	362	14	Male	PM	20-Jan-05	PNEUMONIA	PNEUMONIA	26-Jan-05	1-Feb-05	Resolved	Mild	None	Not related
23	363	5	Female	PM	20-Jan-05	FEVER	PYREXIA	22-Jan-05	25-Jan-05	Resolved	Mild	None	Not related
23	363	5	Female	PM	20-Jan-05	VOMITING	VOMITING	23-Jan-05	23-Jan-05	Resolved	Mild	None	Not related
23	363	5	Female	PM	20-Jan-05	FEVER	PYREXIA	27-Jan-05	27-Jan-05	Resolved	Mild	None	Unlikely
23	363	5	Female	PM	20-Jan-05	ELEVATED ALKALINE PHOSPHATASE	BLOOD ALKALINE PHOSPHATASE INCREASED	26-Jan-05		Ongoing	Mild	None	Probable
23	363	5	Female	PM	20-Jan-05	VOMITING	VOMITING	8-Feb-05	8-Feb-05	Resolved	Mild	None	Possible
23	364	12	Female	Comb	20-Jan-05	EPISTAXIS (NOSE BLEEDING)	EPISTAXIS	4-Feb-05	4-Feb-05	Resolved	Mild	None	Probable
23	365	15	Male	SSG	20-Jan-05	RAISED CREATININE	BLOOD CREATININE INCREASED	2-Feb-05	20-May-05	Resolved	Moderate	None	Probable

Cent No	Pat No	Age	Sex	Treatment	Treatment start date	Adverse Event	Preferred term	AE start date	AE end date	Outcome	Intensity	Action taken	Related
23	366	12	Male	Comb	1-Feb-05	RAISED SGOT/AST	ASPARTATE AMINOTRANSFERASE INCREASED	7-Feb-05	14-Feb-05	Resolved	Mild	None	Probable
23	366	12	Male	Comb	1-Feb-05	BILATERAL CONJUNCTIVITIS	CONJUNCTIVITIS	4-Aug-05		Ongoing	Mild	None	Not related
23	367	20	Male	SSG	1-Feb-05	EPISTAXIS	EPISTAXIS	3-Feb-05	3-Feb-05	Resolved	Mild	None	Not related
23	367	20	Male	SSG	1-Feb-05	PNEUMONIA	PNEUMONIA	26-Feb-05		Ongoing	Mild	None	Not related
23	367	20	Male	SSG	1-Feb-05	EPISTAXIS	EPISTAXIS	1-Mar-05	1-Mar-05	Resolved	Mild	None	Not related
23	367	20	Male	SSG	1-Feb-05	HERPETIC MOUTH ULCER	MOUTH ULCERATION	2-Mar-05	4-Mar-05	Resolved	Mild	None	Not related
23	367	20	Male	SSG	1-Feb-05	PNEUMONIA	PNEUMONIA	9-Feb-05	14-Feb-05	Resolved	Mild	None	Not related
23	367	20	Male	SSG	1-Feb-05	EPISTAXIS	EPISTAXIS	10-Feb-05	10-Feb-05	Resolved	Mild	None	Not related
23	367	20	Male	SSG	1-Feb-05	OTITIS MEDIA	OTITIS MEDIA	8-Jun-05	13-Jun-05	Resolved	Mild	None	Not related
23	367	20	Male	SSG	1-Feb-05	ALLERGIC CONJUCTIVITIS	CONJUNCTIVITIS ALLERGIC	9-Mar-05	14-Mar-05	Resolved	Mild	None	Not related
23	368	16	Female	PM	1-Feb-05	RAISED CREATININE	BLOOD CREATININE INCREASED	22-Feb-05	26-May-05	Resolved	Mild	None	Probable
23	368	16	Female	PM	1-Feb-05	URINARY TRACT INFECTION	URINARY TRACT INFECTION	26-May-05		Ongoing	Mild	None	Probable
23	368	16	Female	PM	1-Feb-05	RIGHT OTITIS MEDIA	OTITIS MEDIA	24-May-05	31-May-05	Resolved	Moderate	None	Not related
23	370	8	Male	SSG	4-Feb-05	A FALL WITH BRUISES ON HEAD CHEST AND LIPS	CONTUSION	1-Jun-05	23-Sep-05	Resolved	Mild	None	Not related
23	370	8	Male	SSG	4-Feb-05	BRADY CARDIA (SINUS)	BRADYCARDIA	8-Jun-05	23-Sep-05	Resolved	Mild	None	Possible
23	371	14	Female	PM	4-Feb-05	ABDOMINAL PAIN	ABDOMINAL PAIN	4-Feb-05	9-Feb-05	Resolved	Moderate	None	Not related
23	371	14	Female	PM	4-Feb-05	PERITONITIS	PERITONITIS	6-Feb-05	13-Feb-05	Resolved	Moderate	None	Not related
23	371	14	Female	PM	4-Feb-05	ASCITES	ASCITES	5-Feb-05	19-Feb-05	Resolved	Mild	None	Not related
23	371	14	Female	PM	4-Feb-05	ASCARIS	ASCARIASIS	4-Feb-05	10-Feb-05	Resolved	Mild	None	Not related
23	371	14	Female	PM	4-Feb-05	UPPER RESPIRATORY TRACT INFECTION	UPPER RESPIRATORY TRACT INFECTION	20-Feb-05	25-Feb-05	Resolved	Mild	None	Not related
23	371	14	Female	PM	4-Feb-05	URINARY TRACT INFECTION	URINARY TRACT INFECTION	21-Feb-05	30-Jan-06	Resolved	Moderate	None	Not related
23	371	14	Female	PM	4-Feb-05	MICROSCOPIC HAEMATURIA	HAEMATURIA	26-May-05		Ongoing	Mild	None	Not related
23	371	14	Female	PM	4-Feb-05	URTI	UPPER RESPIRATORY TRACT INFECTION	5-Sep-05	14-Sep-05	Resolved	Mild	None	Not related
23	372	25	Male	PM	11-Feb-05	RAISED CREATININE	BLOOD CREATININE INCREASED	24-Feb-05	4-Mar-05	Resolved	Mild	None	Possible
23	372	25	Male	PM	11-Feb-05	BRADYCARDIA	BRADYCARDIA	6-Jun-05		Ongoing	Moderate	None	Probable
23	373	21	Male	SSG	18-Feb-05	FACIAL RASH (ACNE)	ACNE	9-Mar-05	6-Jul-05	Resolved	Mild	None	Unlikely
23	373	21	Male	SSG	18-Feb-05	BRADY CARDIA	BRADYCARDIA	6-Jul-05		Ongoing	Moderate	None	Probable
23	374	24	Female	SSG	24-Feb-05	URINARY TRACT INFECTION	URINARY TRACT INFECTION	7-Mar-05	13-Mar-05	Resolved	Mild	None	Unlikely
23	374	24	Female	SSG	24-Feb-05	BLEEDING GUMS	GINGIVAL BLEEDING	12-Mar-05	15-Mar-05	Resolved	Mild	None	Unlikely
23	374	24	Female	SSG	24-Feb-05	NECK PAINS	NECK PAIN	17-Mar-05	21-Mar-05	Resolved	Mild	None	Not related
23	375	12	Male	Comb	25-Feb-05	RIGHT EYE CONJUNCTIVITIS	CONJUNCTIVITIS	11-Mar-05	14-Mar-05	Resolved	Mild	None	Unlikely
23	376	16	Female	SSG	1-Mar-05	FEVER	PYREXIA	7-Mar-05	8-Mar-05	Resolved	Mild	None	Probable
23	376	16	Female	SSG	1-Mar-05	HEADACHE	HEADACHE	7-Mar-05	8-Mar-05	Resolved	Mild	None	Probable

Cent No	Pat No	Age	Sex	Treatment	Treatment start date	Adverse Event	Preferred term	AE start date	AE end date	Outcome	Intensity	Action taken	Related
23	376	16	Female	SSG	1-Mar-05	JOINT ACHE	ARTHRALGIA	7-Mar-05	8-Mar-05	Resolved	Mild	None	Probable
23	376	16	Female	SSG	1-Mar-05	CONJUCTIVITIS RIGHT EYE	CONJUNCTIVITIS	28-Mar-05	31-Mar-05	Resolved	Mild	None	Not related
23	377	40	Female	Comb	9-Mar-05	ELEVATED AST	ASPARTATE AMINOTRANSFERASE INCREASED	15-Mar-05	30-Mar-05	Resolved	Severe	drug stopped	Probable
23	378	7	Male	Comb	9-Mar-05	VOMITING	VOMITING	10-Mar-05	10-Mar-05	Resolved	Mild	None	Unlikely
23	378	7	Male	Comb	9-Mar-05	URTI	UPPER RESPIRATORY TRACT INFECTION	26-Mar-05	30-Mar-05	Resolved	Mild	None	Not related
23	378	7	Male	Comb	9-Mar-05	BIL PNEUMONIA	PNEUMONIA	6-Jul-05	11-Jul-05	Resolved	Moderate	None	Unlikely
23	379	5	Male	PM	10-Mar-05	COUGH	COUGH	28-Mar-05	28-Mar-05	Resolved	Mild	None	Not related
23	379	5	Male	PM	10-Mar-05	BRUISE LEFT THIGH	CONTUSION	30-Mar-05	30-Mar-05	Resolved	Mild	None	Not related
23	379	5	Male	PM	10-Mar-05	SEPTIC WOUND LT FOOT	WOUND SEPSIS	30-Sep-05		Ongoing	Mild	None	Not related
23	381	13	Male	PM	15-Mar-05	RASH ON ARMS AND TRUNK	RASH	28-Mar-05	12-Jul-05	Resolved	Mild	None	Possible
23	381	13	Male	PM	15-Mar-05	PNEUMONIA	PNEUMONIA	12-Jul-05	21-Jul-05	Resolved	Moderate	None	Not related
23	381	13	Male	PM	15-Mar-05	MICROSCOPIC HAEMATURIA	HAEMATURIA	12-Jul-05	16-Nov-05	Resolved	Mild	None	Unlikely
23	382	15	Male	Comb	18-Mar-05	UPPER RESPIRATORY TRACT INFECTION	UPPER RESPIRATORY TRACT INFECTION	21-Mar-05	31-Mar-05	Resolved	Mild	None	Not related
23	382	15	Male	Comb	18-Mar-05	ELEVATED SERUM ALKALINE PHOSPHATASE	BLOOD ALKALINE PHOSPHATASE INCREASED	31-Mar-05	12-Jul-05	Resolved	Moderate	None	Possible
23	382	15	Male	Comb	18-Mar-05	CELLULITIS RT BUTTOCK	CELLULITIS	2-Apr-05		Ongoing	Mild	None	Probable
23	383	30	Male	PM	5-Apr-05	GLUTEAL TENDERNESS	TENDERNESS	18-Apr-05	25-Apr-05	Resolved	Moderate	None	Possible
23	383	30	Male	PM	5-Apr-05	HAEMATURIA	HAEMATURIA	29-Sep-05	24-Oct-05	Resolved	Mild	None	Probable
23	384	13	Male	SSG	5-Apr-05	MACULO-PAPULA RASH - FACE - UPPER	RASH MACULO-PAPULAR	11-Apr-05	16-Apr-05	Resolved	Mild	None	Possible
23	384	13	Male	SSG	5-Apr-05	FRACTURE OF LEFT RADIUS	RADIUS FRACTURE	27-Apr-05	28-Sep-05	Resolved	Moderate	None	Not related
23	384	13	Male	SSG	5-Apr-05	PNEUMONIA	PNEUMONIA	17-Nov-05	24-Nov-05	Resolved	Mild	None	Not related
23	385	14	Male	SSG	7-Apr-05	PNEUMONIA	PNEUMONIA	15-Apr-05	21-Apr-05	Resolved	Mild	None	Not related
23	385	14	Male	SSG	7-Apr-05	ITCHY SKIN OVER FOREARMS	PRURITUS	13-Apr-05	13-Apr-05	Resolved	Mild	None	Probable
23	386	13	Female	Comb	19-Apr-05	RAISED LIVER ENZYMES	HEPATIC ENZYME INCREASED	25-Apr-05	4-May-05	Resolved	Moderate	Drug	Possible
23	386	13	Female	Comb	19-Apr-05	RAISED ALKALINE PHOSPHATASE	BLOOD ALKALINE PHOSPHATASE INCREASED	19-Aug-05	11-Nov-05	Resolved	Moderate	None	Probable
23	387	15	Female	Comb	19-Apr-05	ELEVATED ALKALINE PHOSPHATASE	BLOOD ALKALINE PHOSPHATASE INCREASED	10-Aug-05		Ongoing	Moderate	None	Probable
23	388	15	Male	PM	19-Apr-05	RAISED ALKALINE PHOSPHATASE	BLOOD ALKALINE PHOSPHATASE INCREASED	28-Sep-05	2-Dec-05	Resolved	Mild	None	Not related
23	391	12	Female	Comb	22-Apr-05	ELEVATED SGOT	ASPARTATE AMINOTRANSFERASE INCREASED	28-Apr-05	5-May-05	Resolved	Mild	None	Probable
23	392	7	Male	Comb	22-Apr-05	PNEUMONIA	PNEUMONIA	24-Apr-05	29-Apr-05	Resolved	Mild	None	Unlikely

Cent No	Pat No	Age	Sex	Treatment	Treatment start date	Adverse Event	Preferred term	AE start date	AE end date	Outcome	Intensity	Action taken	Related
23	392	7	Male	Comb	22-Apr-05	RAISED LIVER ENZYMES (SGOT)	ASPARTATE AMINOTRANSFERASE INCREASED	28-Apr-05	4-May-05	Resolved	Moderate	Drug interupt	Probable
23	392	7	Male	Comb	22-Apr-05	BRUISES ON BOTH LEGS	CONTUSION	22-Jul-05	29-Sep-05	Resolved	Mild	None	Not related
23	393	12	Male	SSG	22-Apr-05	FACIAL AND UPPER LIMBS RASH	RASH	3-May-05	19-May-05	Resolved	Mild	None	Possible
23	394	9	Male	PM	26-Apr-05	ABDOMINAL PAIN	ABDOMINAL PAIN	30-Apr-05	2-May-05	Resolved	Mild	None	Probable
23	394	9	Male	PM	26-Apr-05	LUMBAR PAIN	BACK PAIN	1-May-05	1-May-05	Resolved	Mild	None	Not related
23	394	9	Male	PM	26-Apr-05	EOSINOPHILIA	EOSINOPHILIA	19-Aug-05		Ongoing	Moderate	None	Not related
23	394	9	Male	PM	26-Apr-05	ENTAMEBA HISTOLYTICA	AMOEBIC DYSENTERY	16-Nov-05		Ongoing	Mild	None	Not related
23	394	9	Male	PM	26-Apr-05	MICROSCOPIC HAEMATURIA	HAEMATURIA	16-Nov-05		Ongoing	Mild	None	Unlikely
23	394	9	Male	PM	26-Apr-05	LEUKOCYTOSIS	LEUKOCYTOSIS	16-Nov-05		Ongoing	Moderate	None	Unlikely
23	395	42	Male	PM	29-Apr-05	HEADACHE	HEADACHE	15-May-05	18-May-05	Resolved	Moderate	None	Unlikely
23	395	42	Male	PM	29-Apr-05	FEVER	PYREXIA	15-May-05	18-May-05	Resolved	Moderate	None	Unlikely
23	396	16	Male	SSG	13-May-05	ACUTE OTITIS MEDIA - LEFT EAR	OTITIS MEDIA ACUTE	20-May-05	25-May-05	Resolved	Mild	None	Not related
23	399	6	Male	PM	18-May-05	CONJUCTIVITIS LT EYE	CONJUNCTIVITIS	14-Oct-05	14-Dec-05	Resolved	Mild	None	Not related
23	399	6	Male	PM	18-May-05	WOUND RT BIG TOE	LIMB INJURY	14-Oct-05	14-Dec-05	Resolved	Mild	None	Not related
23	400	6	Female	PM	3-Jun-05	FEVER	PYREXIA	20-Jun-05	20-Jun-05	Resolved	Mild	None	Not related
23	401	40	Male	Comb	3-Jun-05	MALAISE	MALAISE	20-Dec-05		Ongoing	Moderate	None	Unlikely
23	403	22	Male	SSG	4-Jun-05	HEADACHE	HEADACHE	5-Jun-05	5-Jun-05	Resolved	Mild	None	Not related
23	403	22	Male	SSG	4-Jun-05	ABDOMINAL PAINS	ABDOMINAL PAIN	7-Jun-05	7-Jun-05	Resolved	Mild	None	Not related
23	403	22	Male	SSG	4-Jun-05	GINGIVITIS	GINGIVITIS	8-Jun-05	12-Jul-05	Resolved	Moderate	None	Not related
23	403	22	Male	SSG	4-Jun-05	EPISTAXIS	EPISTAXIS	21-Jun-05	2-Jul-05	Resolved	Moderate	None	Possible
23	403	22	Male	SSG	4-Jun-05	PAPULAR SKIN RASH (GENERALIZED)	RASH PAPULAR	18-Jun-05	5-Jul-05	Resolved	Moderate	None	Possible
23	403	22	Male	SSG	4-Jun-05	THROMBOCYTOPEANIA	THROMBOCYTOPENIA	12-Jan-06		Ongoing	Moderate	None	Not related
23	404	16	Male	SSG	4-Jun-05	FACIAL RASH	RASH	15-Jun-05	26-Jun-05	Resolved	Mild	None	Possible
23	404	16	Male	SSG	4-Jun-05	ELEVATED ALT	ALANINE AMINOTRANSFERASE INCREASED	17-Jun-05	24-Jun-05	Resolved	Mild	None	Possible
23	405	16	Male	PM	10-Jun-05	RAISED ALKALINE PHOSPHATASE	BLOOD ALKALINE PHOSPHATASE INCREASED	29-Sep-05		Ongoing	Moderate	None	Probable
34	453	4	Male	PM	18-Nov-04	MALARIA	MALARIA	21-Nov-04	27-Nov-04	Resolved	Moderate	None	Not related
34	454	5	Female	Comb	21-Nov-04	PKDL I	VISCERAL LEISHMANIASIS	8-Apr-05		Ongoing	Mild	None	Probable
34	455	11	Male	PM	19-Nov-04	MALARIA	MALARIA	25-Nov-04	4-Dec-04	Resolved	Moderate	None	Not related
34	456	8	Female	SSG	19-Nov-04	PKDL GRAD I	VISCERAL LEISHMANIASIS	30-Nov-04	19-Dec-04	Resolved	Mild	None	Probable
34	458	12	Female	PM	20-Nov-04	MALARIA	MALARIA	26-Nov-04	28-Nov-04	Resolved	Moderate	None	Not related
34	459	9	Female	Comb	20-Nov-04	PKDL GRAD I WITH MUCOSAL (LIP) INVOLVEMENT	VISCERAL LEISHMANIASIS	1-May-05		Ongoing	Moderate	None	Probable

Cent No	Pat No	Age	Sex	Treatment	Treatment start date	Adverse Event	Preferred term	AE start date	AE end date	Outcome	Intensity	Action taken	Related
34	462	4	Female	SSG	25-Nov-04	PKDL II	VISCERAL LEISHMANIASIS	7-Apr-05		Ongoing	Mild	None	Probable
34	462	4	Female	SSG	25-Nov-04	COUGH (URTI?)	UPPER RESPIRATORY TRACT	7-May-05		Ongoing	Mild	None	Not related
34	462	4	Female	SSG	25-Nov-04	FEVER	PYREXIA	25-May-05	7-Jun-05	Resolved	Mild	None	Not related
34	462	4	Female	SSG	25-Nov-04	APPETITE LOSS	ANOREXIA	25-May-05	7-Jun-05	Resolved	Mild		Not related
34	463	4	Male	Comb	27-Nov-04	CONJUNCTIVITIS	CONJUNCTIVITIS	27-Nov-04	29-Nov-04	Resolved	Mild	None	Not related
34	463	4	Male	Comb	27-Nov-04	PKDL III (MILD)	VISCERAL LEISHMANIASIS	16-Mar-05		Ongoing	Mild	None	Probable
34	464	22	Male	SSG	27-Nov-04	COMMON COLD	NASOPHARYNGITIS	13-Dec-04	19-Dec-04	Resolved	Mild	None	Not related
34	465	25	Female	SSG	29-Nov-04	COMMON COLD	NASOPHARYNGITIS	20-Dec-04	21-Dec-04	Resolved	Mild	None	Not related
34	465	25	Female	SSG	29-Nov-04	CONJUNCTIVITIS	CONJUNCTIVITIS	25-Dec-04	28-Dec-04	Resolved	Mild	None	Not related
34	467	14	Male	PM	29-Nov-04	MALARIA	MALARIA	8-Dec-04	10-Dec-04	Resolved	Moderate	None	Not related
34	467	14	Male	PM	29-Nov-04	PKDL I	VISCERAL LEISHMANIASIS	12-Jan-05		Ongoing	Mild	None	Probable
34	469	11	Female	SSG	29-Nov-04	MALARIA	MALARIA	9-Dec-04	11-Dec-04	Resolved	Moderate	None	Not related
34	469	11	Female	SSG	29-Nov-04	COMMON COLD	NASOPHARYNGITIS	13-Dec-04	14-Dec-04	Resolved	Mild	None	Not related
34	469	11	Female	SSG	29-Nov-04	PKDL I	VISCERAL LEISHMANIASIS	1-Feb-05		Resolved	Mild	None	Probable
34	469	11	Female	SSG	29-Nov-04	URTI	UPPER RESPIRATORY TRACT INFECTION	25-Feb-05		Resolved	Mild	None	Not related
34	470	4	Female	Comb	29-Nov-04	URTI	UPPER RESPIRATORY TRACT INFECTION	5-Jun-05		Ongoing	Mild	None	Not related
34	471	13	Male	PM	29-Nov-04	MALARIA	MALARIA	5-Dec-04	7-Dec-04	Resolved	Moderate	None	Not related
34	477	8	Female	SSG	6-Dec-04	MALARIA	MALARIA	26-Dec-04	28-Dec-04	Resolved	Moderate	None	Not related
34	477	8	Female	SSG	6-Dec-04	MALARIA	MALARIA	30-May-05		Ongoing	Mild	None	Not related
34	478	5	Female	Comb	7-Dec-04	REPEATED EPISODES OF FEVER AT NIGHT	PYREXIA	15-Mar-05		Resolved	Mild	None	Unlikely
34	482	18	Male	Comb	10-Dec-04	MALARIA	MALARIA	14-Mar-05	16-Mar-05	Resolved	Mild	None	Not related
34	482	18	Male	Comb	10-Dec-04	ACNE	ACNE	17-Mar-05		Ongoing	Mild	None	Not related
34	483	24	Male	PM	10-Dec-04	PNEUMONIA	PNEUMONIA	13-Dec-04	19-Dec-04	Resolved	Moderate	None	Not related
34	483	24	Male	PM	10-Dec-04	JOINT PAIN	ARTHRALGIA	27-Dec-04	29-Dec-04	Resolved	Mild	None	Unlikely
34	485	5	Female	Comb	12-Dec-04	MALARIA	MALARIA	1-May-05	3-May-05	Resolved	Mild	None	Not related
34	487	12	Male	SSG	13-Dec-04	MALARIA	MALARIA	2-Jan-05	4-Jan-05	Resolved	Moderate	None	Not related
34	487	12	Male	SSG	13-Dec-04	HEADACHE	HEADACHE	1-Apr-05	2-Apr-05	Resolved	Mild	None	Not related
34	489	11	Male	SSG	16-Dec-04	RIGHT PERICULOS OEDEMALOUS PAINFUL SWELLING	SWELLING	27-Dec-04	28-Dec-04	Resolved	Mild	None	Not related
34	492	39	Male	PM	19-Dec-04	BLOODY DIARHEA PROBABLY DYSENTRY	DYSENTRY	30-Mar-05	11-Apr-06	Resolved	Moderate	None	Not related
34	493	12	Female	SSG	17-Dec-04	HYPOGLYCEMIA	HYPOGLYCAEMIA	17-Dec-04	17-Dec-04	Resolved	Moderate	None	Not related
34	493	12	Female	SSG	17-Dec-04	MALARIA	MALARIA	1-Jan-05	3-Jan-05	Resolved	Moderate	None	Not related
34	493	12	Female	SSG	17-Dec-04	PKDL I WITH OCULAR + ORAL INVOLVEMENT	VISCERAL LEISHMANIASIS	12-Mar-05	18-May-05	Resolved	Moderate	None	Probable
34	494	30	Male	Comb	17-Dec-04	CONJUNCTIVITIS	CONJUNCTIVITIS	3-Jun-05		Ongoing	Mild	None	Not related

Cent No	Pat No	Age	Sex	Treatment	Treatment start date	Adverse Event	Preferred term	AE start date	AE end date	Outcome	Intensity	Action taken	Related
34	495	6	Female	SSG	18-Dec-04	ALLERGIC REACTION (INSECT BITE?)	ALLERGY TO ARTHROPOD BITE	24-Dec-04	25-Dec-04	Resolved	Mild	None	Not related
34	495	6	Female	SSG	18-Dec-04	COMMON COLD	NASOPHARYNGITIS	1-Mar-05	2-Mar-05	Resolved	Mild	None	Not related
34	495	6	Female	SSG	18-Dec-04	PKDL I	VISCERAL LEISHMANIASIS	17-Feb-05	1-Mar-05	Resolved	Mild	None	Probable
34	496	35	Male	SSG	19-Dec-04	BURNING FEET	BURNING SENSATION	18-Jan-05	1-Apr-05	Resolved	Mild	None	Unlikely
34	498	10	Female	SSG	21-Dec-04	PNEUMONIA	PNEUMONIA	18-Jan-05	20-Jan-05	Resolved	Moderate	None	Not related
34	498	10	Female	SSG	21-Dec-04	PKDL II	VISCERAL LEISHMANIASIS	6-Feb-05		Ongoing	Mild	None	Probable
34	498	10	Female	SSG	21-Dec-04	COMMON COLD	NASOPHARYNGITIS	4-Feb-05	6-Feb-05	Resolved	Mild	None	Not related
34	499	12	Male	Comb	21-Dec-04	MUCOSAL LESIONS IN THE MOUTH	ORAL SOFT TISSUE DISORDER	30-Dec-04		Ongoing	Mild	None	Unlikely
34	499	12	Male	Comb	21-Dec-04	CUT WOUND RIGHT FOOT	SKIN LACERATION	5-Jun-05	6-Jun-05	Resolved	Mild	None	Not related
34	501	9	Male	Comb	22-Dec-04	TRAUMA LEFT KNEE WITH TWO SUPERFICIAL WOUNDS	SKIN INJURY	7-Jun-05		Ongoing	Mild	None	
34	505	40	Male	SSG	28-Dec-04	URINARY TRACT INFECTION	URINARY TRACT INFECTION	3-Jan-05	10-Jan-05	Resolved	Mild	None	Not related
34	507	6	Male	SSG	31-Dec-04	ABDOMINAL DISTENSION OF UNKNOWN ORIGIN	ABDOMINAL DISTENSION	13-Jan-05	15-Jan-05	Resolved	Mild	None	Unlikely
34	507	6	Male	SSG	31-Dec-04	MALARIA	MALARIA	15-Jan-05	17-Jan-05	Resolved	Moderate	None	Not related
34	507	6	Male	SSG	31-Dec-04	PKDL II FACE, ARM, CHEST, BACK	VISCERAL LEISHMANIASIS	1-Mar-05		Ongoing	Mild	None	Probable
34	508	4	Male	PM	31-Dec-04	SCRATCH WOUND RIGHT FOOT	SCRATCH	2-Jan-05	3-Jan-05	Resolved	Mild	None	Not related
34	510	8	Male	Comb	1-Jan-05	MALARIA	MALARIA	3-Jan-05	5-Jan-05	Resolved	Moderate	None	Not related
34	511	6	Male	Comb	1-Jan-05	MALARIA	MALARIA	11-Jan-05	13-Jan-05	Resolved	Moderate	None	Not related
34	511	6	Male	Comb	1-Jan-05	COMMON COLD	NASOPHARYNGITIS	3-Jun-05		Ongoing	Mild	None	Not related
34	512	6	Female	PM	2-Jan-05	MALARIA	MALARIA	8-Jan-05	10-Jan-05	Resolved	Moderate	None	Not related
34	512	6	Female	PM	2-Jan-05	SWELLING AT THE FACE AFTER TRAUMA	SWELLING FACE	21-Jan-05	22-Jan-05	Resolved	Mild	None	Not related
34	512	6	Female	PM	2-Jan-05	NASAL BLEEDING	EPISTAXIS	20-Jan-05	21-Jan-05	Resolved		None	Unlikely
34	513	10	Male	Comb	3-Jan-05	ACUTE WATERY DIARRHOEA	DIARRHOEA	7-Jan-05	13-Jan-05	Resolved	Moderate	None	Not related
34	513	10	Male	Comb	3-Jan-05	SUPERFICIAL SKIN LESION (NASAL)	SKIN LESION	15-Jan-05	19-Jan-05	Resolved	Mild	None	Not related
34	514	7	Male	SSG	4-Jan-05	PNEUMONIA	PNEUMONIA	29-Jan-05	2-Feb-05	Resolved	Moderate	None	Not related
34	515	10	Male	SSG	5-Jan-05	MALARIA	MALARIA	13-May-05	15-May-05	Resolved	Mild	None	Not related
34	515	10	Male	SSG	5-Jan-05	PKDL II WITH MUCOSAL INVOLVEMENT	VISCERAL LEISHMANIASIS	13-Apr-05		Ongoing	Moderate	None	Probable
34	516	15	Male	PM	6-Jan-05	COMMON COLD	NASOPHARYNGITIS	10-Jan-05	11-Jan-05	Resolved	Mild	None	Not related
34	516	15	Male	PM	6-Jan-05	SUPERFICIAL FUNGAL SKIN INFECTION (PROBABLY SINCE ADMISSION)	FUNGAL SKIN INFECTION	19-Jan-05	26-Jan-05	Resolved	Mild	None	Unlikely
34	516	15	Male	PM	6-Jan-05	HEART BURN	DYSPEPSIA	24-Jan-05	25-Jan-05	Resolved	Mild	None	Unlikely

Cent No	Pat No	Age	Sex	Treatment	Treatment start date	Adverse Event	Preferred term	AE start date	AE end date	Outcome	Intensity	Action taken	Related
34	518	4	Male	SSG	9-Jan-05	ARTESUNATE RESISTANT MALARIA	MALARIA	11-Jan-05	13-Jan-05	Resolved	Moderate	None	Not related
34	518	4	Male	SSG	9-Jan-05	MALARIA (TREATED WITH QUININE)	MALARIA	18-Jan-05	24-Jan-05	Resolved	Moderate	Drug interupt	Not related
34	518	4	Male	SSG	9-Jan-05	PNEUMONIA	PNEUMONIA	18-Jan-05	22-Jan-05	Resolved	Moderate	None	Not related
34	518	4	Male	SSG	9-Jan-05	COMMON COLD	NASOPHARYNGITIS	15-Jan-05	16-Jan-05	Resolved	Mild	None	Not related
34	518	4	Male	SSG	9-Jan-05	ECG CHANGES WITH SSG + QUININE => SSG STOPPED	ELECTROCARDIOGRAM CHANGE	24-Jan-05	26-Jan-05	Resolved	Moderate	Drug interupt	Probable
34	519	8	Female	PM	7-Jan-05	MALARIA	MALARIA	13-Jan-05	15-Jan-05	Resolved	Moderate	None	Not related
34	519	8	Female	PM	7-Jan-05	HEADACHE	HEADACHE	22-Jan-05	23-Jan-05	Resolved	Mild	None	Unlikely
34	519	8	Female	PM	7-Jan-05	ABDOMINAL PAIN PARAUMBLICAL	ABDOMINAL PAIN	24-Jan-05	25-Jan-05	Resolved	Mild	None	Unlikely
34	519	8	Female	PM	7-Jan-05	MALARIA	MALARIA	22-Mar-05	25-Mar-05	Resolved	Moderate	None	Not related
34	520	19	Male	SSG	11-Jan-05	ACUTE WATERY DIARRHOEA	DIARRHOEA	17-Jan-05	18-Jan-05	Resolved	Moderate	None	Not related
34	520	19	Male	SSG	11-Jan-05	MUCOID STOOL	MUCOUS STOOLS	23-Jan-05	29-Jan-05	Resolved	Mild	None	Not related
34	520	19	Male	SSG	11-Jan-05	COMMON COLD	UPPER RESPIRATORY TRACT INFECTION	28-Jan-05	30-Jan-05	Resolved	Mild	None	Not related
34	520	19	Male	SSG	11-Jan-05	BACKACHE	BACK PAIN	2-Feb-05	3-Feb-05	Resolved	Mild	None	Unlikely
34	520	19	Male	SSG	11-Jan-05	HEADACHE	HEADACHE	28-Jan-05	30-Jan-05	Resolved	Mild	None	Not related
34	520	19	Male	SSG	11-Jan-05	PKDL I WITH ORAL INVOLVEMENT	VISCERAL LEISHMANIASIS	5-May-05		Ongoing	Mild	None	Probable
34	520	19	Male	SSG	11-Jan-05	HEADACHE AND VOMITING DIAGNOSED AS MALARIA IN RURAL HEALTH CENTER	MALARIA	15-May-05	15-May-05	Resolved	Mild	None	Not related
34	521	5	Male	PM	11-Jan-05	MALARIA	MALARIA	20-Jan-05	22-Jan-05	Resolved	Moderate	None	Not related
34	522	36	Male	Comb	11-Jan-05	ASTHMA	ASTHMA	19-Jan-05	20-Jan-05	Resolved	Moderate	None	Possible
34	522	36	Male	Comb	11-Jan-05	ASTHMA	ASTHMA	24-Jan-05	25-Jan-05	Resolved	Moderate	None	Possible
34	525	8	Male	Comb	13-Jan-05	PKDL III	VISCERAL LEISHMANIASIS	10-May-05		Ongoing	Mild	None	Probable
34	526	5	Male	SSG	14-Jan-05	MALARIA	MALARIA	16-Jan-05	18-Jan-05	Resolved	Moderate	None	Not related
34	526	5	Male	SSG	14-Jan-05	SUSPECTED TB	TUBERCULOSIS	15-May-05		Ongoing	Moderate	None	Not related
34	527	19	Female	Comb	15-Jan-05	VOMITING	VOMITING	16-Jan-05	17-Jan-05	Resolved	Mild	None	Unlikely
34	527	19	Female	Comb	15-Jan-05	PKDL I	VISCERAL LEISHMANIASIS	1-Apr-05	8-Apr-05	Resolved	Mild	None	Probable
34	528	6	Male	PM	17-Jan-05	ACUTE WATERY DIARRHOEA	DIARRHOEA	25-Jan-05	26-Jan-05	Resolved	Moderate	None	Not related
34	529	5	Female	Comb	18-Jan-05	COMMON COLD	NASOPHARYNGITIS	10-May-05	13-May-05	Resolved	Mild	None	Not related
34	530	22	Male	Comb	20-Jan-05	MINIMAL NASAL BLEEDING	EPISTAXIS	5-Feb-05	6-Feb-05	Resolved	Mild	None	Unlikely
34	530	22	Male	Comb	20-Jan-05	EAR INFECTION	EAR INFECTION	14-Apr-05	21-Apr-05	Resolved	Mild	None	Not related
34	532	4	Male	Comb	21-Jan-05	PKDL II	VISCERAL LEISHMANIASIS	10-May-05		Ongoing	Mild	None	Probable
34	533	10	Male	SSG	23-Jan-05	BACK PAIN	BACK PAIN	17-Feb-05	17-Feb-05	Resolved	Mild	None	Not related
34	533	10	Male	SSG	23-Jan-05	PKDL I	VISCERAL LEISHMANIASIS	16-Apr-05		Ongoing	Mild	None	Probable
34	534	7	Male	PM	24-Jan-05	PNEUMONIA	PNEUMONIA	30-Jan-05	3-Feb-05	Resolved	Moderate	None	Not related
34	534	7	Male	PM	24-Jan-05	WEIGHT LOSS (W/H <70%)	WEIGHT DECREASED	30-Jan-05	14-Feb-05	Resolved	Moderate	None	Probable
34	535	11	Female	PM	24-Jan-05	ACUTE WATERY DIARRHOE	DIARRHOEA	6-Feb-05	7-Feb-05	Resolved	Moderate	None	Not related

Cent No	Pat No	Age	Sex	Treatment	Treatment start date	Adverse Event	Preferred term	AE start date	AE end date	Outcome	Intensity	Action taken	Related
34	536	12	Male	PM	25-Jan-05	HEADACHE	HEADACHE	5-May-05	6-May-05	Resolved	Mild	None	Not related
34	537	4	Female	SSG	25-Jan-05	LARYNGITIS	LARYNGITIS	12-Feb-05	14-Feb-05	Resolved	Mild	None	Not related
34	538	6	Male	PM	26-Jan-05	NASAL BLEEDING	EPISTAXIS	30-Jan-05	31-Jan-05	Resolved	Moderate	None	Unlikely
34	539	6	Male	Comb	26-Jan-05	COMMON COLD	NASOPHARYNGITIS	10-Feb-05	11-Feb-05	Resolved	Mild	None	Not related
34	539	6	Male	Comb	26-Jan-05	MALARIA	MALARIA	31-May-05		Ongoing	Mild	None	Not related
34	540	11	Female	SSG	27-Jan-05	EPIGASTRIC PAIN	ABDOMINAL PAIN UPPER	29-Jan-05	31-Jan-05	Resolved	Mild	None	Possible
34	540	11	Female	SSG	27-Jan-05	PNEUMONIA	PNEUMONIA	9-Feb-05	19-Feb-05	Resolved	Moderate	None	Not related
35	646	16	Male	PM	28-Jan-05	SUPPURATIVE OTITIS MEDIA	OTITIS MEDIA	3-Feb-05	12-Feb-05	Resolved	Moderate	None	Not related
35	646	16	Male	PM	28-Jan-05	PAIN AT THE OF INJECTION	INJECTION SITE PAIN	1-Feb-05	1-Feb-05	Resolved	Mild	None	Possible
35	647	23	Male	PM	30-Jan-05	PAIN AT THE SITE OF INJECTION	INJECTION SITE PAIN	4-Feb-05	4-Feb-05	Resolved	Mild	None	Possible
35	647	23	Male	PM	30-Jan-05	UTI	URINARY TRACT INFECTION	20-Mar-05	24-Mar-05	Resolved	Mild	None	Unlikely
35	648	5	Male	Comb	30-Jan-05	PAIN AT THE SITE OF INJECTION	INJECTION SITE PAIN	30-Jan-05	30-Jan-05	Resolved	Mild	None	Possible
35	648	5	Male	Comb	30-Jan-05	ASTHMATIC ATTACK	ASTHMA	8-Feb-05	16-Feb-05	Resolved	Moderate	None	Unlikely
35	649	13	Female	Comb	30-Jan-05	PAIN AT THE SITE OF INJECTION	INJECTION SITE PAIN	30-Jan-05	30-Jan-05	Resolved	Mild	None	Possible
35	650	6	Male	SSG	30-Jan-05	PAIN AT THE INJECTION SITE	INJECTION SITE PAIN	3-Feb-05	3-Feb-05	Resolved	Mild	None	Possible
35	651	5	Male	SSG	30-Jan-05	CROUP	CROUP INFECTIOUS	10-Feb-05	12-Feb-05	Resolved	Mild	None	Not related
35	651	5	Male	SSG	30-Jan-05	MALARIA	MALARIA	18-Feb-05	21-Feb-05	Resolved	Mild	None	Not related
35	651	5	Male	SSG	30-Jan-05	PAIN AT THE SITE OF INJECTION	INJECTION SITE PAIN	3-Feb-05	3-Feb-05	Resolved	Mild	None	Possible
35	651	5	Male	SSG	30-Jan-05	PKDL, G2:1	VISCERAL LEISHMANIASIS	15-Jul-05		Ongoing	Moderate	None	Unlikely
35	652	8	Female	PM	1-Feb-05	EARACHE RT	EAR PAIN	6-Feb-05	11-Feb-05	Resolved	Moderate	None	Not related
35	652	8	Female	PM	1-Feb-05	ACUTE OTITIS MEDIA	OTITIS MEDIA ACUTE	7-Feb-05	16-Feb-05	Resolved	Moderate	None	Not related
35	653	6	Female	Comb	3-Feb-05	PAIN AT THE INJECTION SITE	INJECTION SITE PAIN	6-Feb-05	6-Feb-05	Resolved	Mild	None	Probable
35	654	5	Female	SSG	3-Feb-05	PURULENT CONJUNCTIVITIS	CONJUNCTIVITIS INFECTIVE	21-Feb-05	25-Feb-05	Resolved	Mild	None	Not related
35	654	5	Female	SSG	3-Feb-05	PKDL GI	VISCERAL LEISHMANIASIS	20-Jul-05		Ongoing	Mild	None	Unlikely
35	655	11	Male	Comb	3-Feb-05	PAIN AT THE INJECTION SITE	INJECTION SITE PAIN	7-Feb-05	7-Feb-05	Resolved	Mild	None	Probable
35	657	10	Female	PM	8-Feb-05	PAIN AT SITE OF INJECTION	INJECTION SITE PAIN	12-Feb-05	12-Feb-05	Resolved	Mild	None	Possible
35	658	15	Male	Comb	8-Feb-05	PAIN AT THE INJECTION SITE	INJECTION SITE PAIN	11-Feb-05	11-Feb-05	Resolved	Mild	None	Probable
35	658	15	Male	Comb	8-Feb-05	PAIN AT THE INJECTION SITE	INJECTION SITE PAIN	18-Feb-05	20-Feb-05	Resolved	Moderate	None	Probable
35	658	15	Male	Comb	8-Feb-05	AMOEBIC DYSENTRY	AMOEBIC DYSENTERY	17-Feb-05	21-Feb-05	Resolved	Mild	None	Not related
35	659	6	Male	SSG	8-Feb-05	EPISTAXIS	EPISTAXIS	13-Feb-05	13-Feb-05	Resolved	Mild	None	Possible
35	659	6	Male	SSG	8-Feb-05	PKDL	VISCERAL LEISHMANIASIS	4-Apr-05	2-Jul-05	Resolved	Moderate	None	Unlikely
35	662	25	Male	PM	20-Feb-05	MALARIA	MALARIA	21-Feb-05	24-Feb-05	Resolved	Mild	None	Not related
35	663	48	Male	SSG	20-Feb-05	PNEUMONIA	PNEUMONIA	23-Feb-05	26-Feb-05	Resolved	Moderate	None	Not related
35	663	48	Male	SSG	20-Feb-05	PROLONGED Q-T INTERVAL	ELECTROCARDIOGRAM QT PROLONGED	26-Feb-05	5-Mar-05	Resolved	Moderate	None	Probable
35	663	48	Male	SSG	20-Feb-05	PROLONGED Q.T INTERVAL	ELECTROCARDIOGRAM QT PROLONGED	12-Mar-05		Ongoing	Moderate	None	Probable

Cent No	Pat No	Age	Sex	Treatment	Treatment start date	Adverse Event	Preferred term	AE start date	AE end date	Outcome	Intensity	Action taken	Related
35	663	48	Male	SSG	20-Feb-05	ABD. PAIN LT HYPOCHONDRIUM	ABDOMINAL PAIN UPPER	15-Jun-05	23-Jun-05	Resolved	Mild	None	Not related
35	664	37	Female	Comb	20-Feb-05	HEART BURN	DYSPEPSIA	21-Feb-05	26-Feb-05	Resolved	Mild	None	Not related
35	664	37	Female	Comb	20-Feb-05	VOMITING	VOMITING	21-Feb-05	26-Feb-05	Resolved	Mild	None	Not related
35	666	12	Female	SSG	26-Feb-05	PKDL	VISCERAL LEISHMANIASIS	15-Jun-05		Ongoing	Moderate	None	Unlikely
35	670	10	Male	SSG	3-Mar-05	PKDL	VISCERAL LEISHMANIASIS	7-Apr-05	10-Jun-05	Resolved	Mild	None	Unlikely
35	673	11	Female	SSG	11-Mar-05	PKDL	VISCERAL LEISHMANIASIS	15-Aug-05	1-Oct-05	Resolved	Mild	None	Unlikely
35	677	37	Male	SSG	20-Mar-05	EPISTAXIS	EPISTAXIS	23-Mar-05	23-Mar-05	Resolved	Mild	None	Unlikely
35	678	15	Male	Comb	22-Mar-05	PKDL	VISCERAL LEISHMANIASIS	31-Mar-05	28-Apr-05	Resolved	Mild	None	Unlikely
35	679	45	Female	SSG	22-Mar-05	PKDL G1	VISCERAL LEISHMANIASIS	29-Sep-05		Ongoing	Mild	None	Unlikely
35	681	16	Male	Comb	22-Mar-05	PNEUMONIA	PNEUMONIA	27-Mar-05	1-Apr-05	Resolved	Mild	None	Not related
35	683	12	Female	Comb	22-Mar-05	MULLOSCUM CONTAGIOSUM	MOLLUSCUM CONTAGIOSUM	23-Mar-05	26-Mar-05	Resolved	Mild	None	Not related
35	684	15	Female	SSG	9-Apr-05	UN-COMPLICATED MALARIA	MALARIA	5-May-05	8-May-05	Resolved	Moderate	None	Not related
35	687	21	Female	SSG	13-Apr-05	ALLERGIC BRONCHITIS	ALLERGIC BRONCHITIS	16-Apr-05	19-Apr-05	Resolved	Mild	None	Unlikely
35	687	21	Female	SSG	13-Apr-05	URINARY TRACT INFECTION	URINARY TRACT INFECTION	5-May-05	8-May-05	Resolved	Mild	None	Unlikely
35	687	21	Female	SSG	13-Apr-05	LT HYPOCHONDRIAL PAIN	ABDOMINAL PAIN UPPER	20-Sep-05	30-Sep-05	Resolved	Mild	None	Not related
35	688	17	Male	PM	16-Apr-05	MALARIA	MALARIA	15-Sep-05	18-Sep-05	Resolved	Mild	None	Not related
35	690	10	Male	SSG	16-Apr-05	EPISTAXIS	EPISTAXIS	19-Apr-05	19-Apr-05	Resolved	Mild	None	Possible
35	690	10	Male	SSG	16-Apr-05	EPISTAXIS	EPISTAXIS	10-May-05	10-May-05	Resolved	Mild	None	Possible

Centre Number	Patient Number	Treatment	ECG Date	Visit	ECG Findings	Comment
11	1	PM	17-Jun-05	day 0	Normal	
11	1	PM	24-Jun-05	day 7	Normal	
11	1	PM	1-Jul-05	day 14	Normal	
11	1	PM	9-Jul-05	end day	Normal	
11	1	PM	5-Oct-05	month 3	Normal	
11	1	PM	5-Jan-06	month 6	Normal	
11	2	Combination	17-Jun-05	day 0	Normal	
11	2	Combination	24-Jun-05	day 7	Normal	
11	2	Combination	1-Jul-05	day 14	Normal	
11	2	Combination	5-Jul-05	end day	Normal	
11	2	Combination	29-Sep-05	month 3	Normal	
11	2	Combination	6-Jan-06	month 6	Normal	
11	3	Combination	17-Jun-05	day 0	Normal	
11	3	Combination	25-Jun-05	day 7	Normal	
11	3	Combination	2-Jul-05	day 14	Normal	
11	3	Combination	6-Jul-05	end day	Normal	
11	3	Combination	30-Sep-05	month 3	Normal	
11	3	Combination	29-Dec-05	month 6	Normal	
11	4	PM	27-Jun-05	day 0	Normal	
11	4	PM	6-Jul-05	day 7	Normal	
11	4	PM	13-Jul-05	day 14	Normal	
11	4	PM	21-Jul-05	end day	Normal	
11	4	PM	31-Dec-05	month 3	Normal	
11	4	PM	27-Jan-06	month 6	Normal	
11	5	PM	27-Jun-05	day 0	Normal	
11	5	PM	6-Jul-05	day 7	Normal	
11	5	PM	13-Jul-05	day 14	Normal	
11	5	PM	21-Jul-05	end day	Normal	
11	5	PM	18-Oct-05	month 3	Normal	
11	5	PM	15-Feb-06	month 6	Normal	
11	6	Combination	27-Jun-05	day 0	Normal	
11	6	Combination	8-Jul-05	day 7	Normal	
11	6	Combination	15-Jul-05	day 14	Normal	
11	6	Combination	19-Jul-05	end day	Normal	
11	6	Combination	13-Oct-05	month 3	Normal	
11	6	Combination	11-Jan-06	month 6	Normal	
11	7	PM	1-Jul-05	day 0	Normal	
11	7	PM	8-Jul-05	day 7	Normal	
11	7	PM	15-Jul-05	day 14	Normal	
11	7	PM	23-Jul-05	end day	Normal	
11	7	PM	21-Oct-05	month 3	Normal	
11	7	PM	20-Jan-06	month 6	Normal	
11	8	SSG	1-Jul-05	day 0	Normal	
11	8	SSG	8-Jul-05	day 7	Normal	
11	8	SSG	15-Jul-05	day 14	Abnormal (CS)	BRADYCARDIA
11	8	SSG	24-Jul-05	day 21	Normal	
11	8	SSG	1-Aug-05	end day	Normal	
11	8	SSG	31-Oct-05	month 3	Normal	
11	8	SSG	30-Jan-06	month 6	Normal	
11	9	SSG	1-Jul-05	day 0	Normal	
11	9	SSG	9-Jul-05	day 7	Normal	
11	9	SSG	16-Jul-05	day 14	Normal	

Centre Number	Patient Number	Treatment	ECG Date	Visit	ECG Findings	Comment
11	9	SSG	24-Jul-05	day 21	Normal	
11	9	SSG	2-Aug-05	end day	Normal	
11	9	SSG	1-Nov-05	month 3	Normal	
11	9	SSG	30-Jan-06	month 6	Normal	
11	10	SSG	1-Jul-05	day 0	Normal	
11	10	SSG	9-Jul-05	day 7	Normal	
11	10	SSG	16-Jul-05	day 14	Normal	
11	10	SSG	24-Jul-05	day 21	Normal	
11	10	SSG	2-Aug-05	end day	Normal	
11	10	SSG	1-Nov-05	month 3	Normal	
11	10	SSG	30-Jan-06	month 6	Normal	
11	11	Combination	1-Jul-05	day 0	Normal	
11	11	Combination	11-Jul-05	day 7	Normal	
11	11	Combination	18-Jul-05	day 14	Normal	
11	11	Combination	22-Jul-05	end day	Normal	
11	11	Combination	21-Oct-05	month 3	Normal	
11	11	Combination	20-Jan-06	month 6	Normal	
11	12	Combination	3-Jul-05	day 0	Normal	
11	12	Combination	14-Jul-05	day 7	Normal	
11	12	Combination	21-Jul-05	day 14	Normal	
11	12	Combination	25-Jul-05	end day	Normal	
11	12	Combination	25-Oct-05	month 3	Normal	
11	13	SSG	11-Jul-05	day 0	Normal	
11	13	SSG	18-Jul-05	day 7	Normal	
11	13	SSG	25-Jul-05	day 14	Normal	
11	13	SSG	1-Aug-05	day 21	Normal	
11	13	SSG	11-Aug-05	end day	Normal	
11	13	SSG	6-Feb-06	month 6	Normal	
11	14	SSG	7-Jul-05	day 0	Normal	
11	14	SSG	18-Jul-05	day 7	Normal	
11	14	SSG	25-Jul-05	day 14	Normal	
11	14	SSG	1-Aug-05	day 21	Normal	
11	14	SSG	11-Aug-05	end day	Normal	
11	14	SSG	11-Nov-05	month 3	Normal	
11	14	SSG	16-Apr-06	month 6	Normal	
11	15	PM	11-Jul-05	day 0	Normal	NON SPECIFIC ABNORMALITY NON SPECIFIC ABNORMALITY (SINUS TACHYCARDIA)
11	15	PM	20-Jul-05	day 7	Abnormal (CS)	TACHYCARDIA OF 140 BPM
11	15	PM	7-Nov-05	month 3	Normal	
11	15	PM	5-Apr-06	month 6	Normal	
11	16	Combination	11-Jul-05	day 0	Normal	
11	16	Combination	21-Jul-05	day 7	Normal	
11	16	Combination	28-Jul-05	day 14	Normal	
11	16	Combination	1-Aug-05	end day	Normal	
11	16	Combination	31-Oct-05	month 3	Normal	
11	16	Combination	6-Feb-06	month 6	Normal	
11	17	Combination	11-Jul-05	day 0	Normal	
11	17	Combination	25-Jul-05	day 7	Normal	
11	17	Combination	1-Aug-05	day 14	Normal	
11	17	Combination	5-Aug-05	end day	Normal	
11	17	Combination	7-Nov-05	month 3	Normal	

Centre Number	Patient Number	Treatment	ECG Date	Visit	ECG Findings	Comment
11	17	Combination	6-Feb-06	month 6	Normal	
11	18	SSG	13-Aug-05	day 0	Normal	
11	18	SSG	22-Aug-05	day 7	Normal	
11	18	SSG	29-Aug-05	day 14	Normal	
11	18	SSG	5-Sep-05	day 21	Normal	
11	18	SSG	15-Sep-05	end day	Normal	
11	18	SSG	22-Dec-05	month 3	Normal	
11	18	SSG	15-Mar-06	month 6	Abnormal (CS)	SINUS BRADYCARDIA
11	19	Combination	15-Aug-05	day 0	Normal	
11	19	Combination	22-Aug-05	day 7	Normal	
11	19	Combination	29-Aug-05	day 14	Normal	
11	19	Combination	2-Sep-05	end day	Normal	
11	19	Combination	29-Nov-05	month 3	Normal	
11	19	Combination	27-Feb-06	month 6	Normal	
11	20	SSG	15-Aug-05	day 0	Normal	
11	20	SSG	22-Aug-05	day 7	Normal	
11	20	SSG	29-Aug-05	day 14	Normal	
11	20	SSG	5-Sep-05	day 21	Normal	
11	20	SSG	15-Sep-05	end day	Normal	
11	20	SSG	14-Dec-05	month 3	Normal	
11	20	SSG	14-Mar-06	month 6	Normal	
11	21	SSG	15-Aug-05	day 0	Normal	
11	21	SSG	22-Aug-05	day 7	Normal	
11	21	SSG	29-Aug-05	day 14	Normal	
11	21	SSG	5-Sep-05	day 21	Normal	
11	21	SSG	15-Sep-05	end day	Normal	
11	21	SSG	22-Dec-05	month 3	Normal	
11	21	SSG	15-Mar-06	month 6	Normal	
11	22	SSG	16-Aug-05	day 0	Normal	
11	22	SSG	23-Aug-05	day 7	Normal	
11	22	SSG	30-Aug-05	day 14	Normal	
11	22	SSG	6-Sep-05	day 21	Normal	
11	22	SSG	16-Sep-05	end day	Normal	
11	22	SSG	15-Dec-05	month 3	Normal	
11	22	SSG	14-Apr-06	month 6	Normal	
11	23	PM	18-Aug-05	day 0	Normal	
11	23	PM	26-Aug-05	day 7	Normal	
11	23	PM	2-Sep-05	day 14	Normal	
11	23	PM	10-Sep-05	end day	Normal	
11	23	PM	11-Jan-06	month 3	Normal	
11	23	PM	31-May-06	month 6	Normal	
11	24	PM	18-Aug-05	day 0	Normal	
11	24	PM	26-Aug-05	day 7	Normal	
11	24	PM	2-Sep-05	day 14	Normal	
11	24	PM	10-Sep-05	end day	Normal	
11	24	PM	28-Nov-05	month 3	Normal	
11	24	PM	10-Feb-06	month 6	Normal	
11	25	SSG	19-Aug-05	day 0	Normal	
11	25	SSG	26-Aug-05	day 7	Normal	
11	25	SSG	2-Sep-05	day 14	Normal	
11	25	SSG	9-Sep-05	day 21	Normal	
11	25	SSG	19-Sep-05	end day	Normal	

Centre Number	Patient Number	Treatment	ECG Date	Visit	ECG Findings	Comment
11	25	SSG	3-Jan-06	month 3	Normal	
11	25	SSG	14-Apr-06	month 6	Normal	
11	26	Combination	24-Aug-05	day 0	Normal	
11	26	Combination	31-Aug-05	day 7	Normal	
11	26	Combination	7-Sep-05	day 14	Normal	
11	26	Combination	11-Sep-05	end day	Normal	
11	26	Combination	10-Nov-05	month 3	Normal	
11	26	Combination	16-Apr-06	month 6	Normal	
11	27	PM	16-Sep-05	day 0	Normal	
11	27	PM	25-Sep-05	day 7	Normal	
11	27	PM	2-Oct-05	day 14	Normal	
11	27	PM	10-Oct-05	end day	Normal	
11	27	PM	30-Jan-06	month 3	Normal	
11	28	Combination	16-Sep-05	day 0	Normal	
11	28	Combination	27-Sep-05	day 7	Normal	
11	28	Combination	4-Oct-05	day 14	Normal	
11	28	Combination	8-Oct-05	end day	Normal	
11	28	Combination	5-Jan-06	month 3	Normal	
11	28	Combination	4-Apr-06	month 6	Normal	
11	29	PM	21-Sep-05	day 0	Normal	
11	29	PM	28-Sep-05	day 7	Normal	
11	29	PM	5-Oct-05	day 14	Normal	
11	29	PM	13-Oct-05	end day	Normal	
11	29	PM	9-Jan-06	month 3	Normal	
11	29	PM	18-Apr-06	month 6	Normal	
11	30	PM	21-Sep-05	day 0	Normal	
11	30	PM	28-Sep-05	day 7	Normal	
11	30	PM	5-Oct-05	day 14	Normal	
11	30	PM	13-Oct-05	end day	Normal	
11	30	PM	3-Jan-06	month 3	Normal	
11	30	PM	14-Apr-06	month 6	Normal	
11	31	SSG	21-Sep-05	day 0	Normal	
11	31	SSG	29-Sep-05	day 7	Normal	SINUS TACHYCARDIA (NOT SIGNIFICANT)
11	31	SSG	6-Oct-05	day 14	Normal	
11	31	SSG	13-Oct-05	day 21	Normal	
11	31	SSG	23-Oct-05	end day	Normal	
11	31	SSG	2-Feb-06	month 3	Normal	
11	31	SSG	5-Apr-06	month 6	Normal	
11	32	Combination	22-Sep-05	day 0	Normal	
11	32	Combination	30-Sep-05	day 7	Normal	
11	32	Combination	7-Oct-05	day 14	Normal	
11	32	Combination	11-Oct-05	end day	Normal	
11	32	Combination	9-Jan-06	month 3	Normal	
11	32	Combination	3-Apr-06	month 6	Normal	
11	33	SSG	24-Sep-05	day 0	Normal	
11	33	SSG	1-Oct-05	day 7	Normal	
11	33	SSG	8-Oct-05	day 14	Normal	
11	33	SSG	15-Oct-05	day 21	Normal	
11	33	SSG	25-Oct-05	end day	Normal	
11	33	SSG	23-Jan-06	month 3	Normal	
11	33	SSG	16-Apr-06	month 6	Normal	
11	34	PM	24-Sep-05	day 0	Normal	

Centre Number	Patient Number	Treatment	ECG Date	Visit	ECG Findings	Comment
11	34	PM	3-Oct-05	day 7	Normal	
11	34	PM	10-Oct-05	day 14	Normal	
11	34	PM	18-Oct-05	end day	Normal	
11	34	PM	17-Jan-06	month 3	Normal	
11	34	PM	16-Apr-06	month 6	Normal	
11	35	SSG	24-Sep-05	day 0	Normal	
11	35	SSG	3-Oct-05	day 7	Normal	
11	35	SSG	10-Oct-05	day 14	Normal	
11	35	SSG	17-Oct-05	day 21	Normal	
11	35	SSG	27-Oct-05	end day	Normal	
11	35	SSG	11-Jan-06	month 3	Normal	
11	35	SSG	16-Apr-06	month 6	Normal	
11	36	SSG	27-Sep-05	day 0	Abnormal (CI)	NON SPECIFIC T.WAVE CHANGES (INVERSION) ON CHEST LEADS -SINUS TACHYCARDIA
11	36	SSG	4-Oct-05	day 7	Normal	
11	36	SSG	11-Oct-05	day 14	Normal	
11	36	SSG	18-Oct-05	day 21	Normal	
11	36	SSG	28-Oct-05	end day	Normal	
11	36	SSG	24-Jan-06	month 3	Normal	
11	36	SSG	10-Jun-06	month 6	Normal	
11	37	Combination	27-Sep-05	day 0	Normal	SINUS TACYCARDIA
11	37	Combination	4-Oct-05	day 7	Normal	HRZ 100BPM N- AXIS ST DEPRESSION V1-V3 ASS:-ST DEPRESSION V1-V3
11	37	Combination	11-Oct-05	day 14	Normal	
11	37	Combination	15-Oct-05	end day	Normal	
11	37	Combination	17-Jan-06	month 3	Normal	
11	37	Combination	14-Apr-06	month 6	Normal	
11	38	PM	17-Oct-05	day 0	Normal	
11	38	PM	25-Oct-05	day 7	Abnormal (CI)	AR. ATRIAL PREMATURE CONTRUCTION
11	38	PM	1-Nov-05	day 14	Normal	
11	38	PM	9-Nov-05	end day	Normal	
11	38	PM	15-Feb-06	month 3	Normal	
11	38	PM	1-Jun-06	month 6	Normal	
11	39	PM	17-Oct-05	day 0	Normal	
11	39	PM	25-Oct-05	day 7	Normal	
11	39	PM	1-Nov-05	day 14	Normal	
11	39	PM	9-Nov-05	end day	Normal	
11	39	PM	6-Mar-06	month 3	Abnormal (CI)	SINUS ARRYTHMIA
11	39	PM	23-May-06	month 6	Normal	
11	40	PM	19-Oct-05	day 0	Normal	
11	40	PM	26-Oct-05	day 7	Normal	
11	40	PM	2-Nov-05	day 14	Normal	
11	40	PM	14-Apr-06	month 3	Normal	
11	40	PM	24-May-06	month 6	Normal	
11	41	Combination	19-Oct-05	day 0	Normal	
11	41	Combination	26-Oct-05	day 7	Normal	
11	41	Combination	2-Nov-05	day 14	Normal	
11	41	Combination	6-Nov-05	end day	Normal	
11	41	Combination	13-Feb-06	month 3	Normal	
11	41	Combination	31-May-06	month 6	Normal	
11	42	Combination	19-Oct-05	day 0	Normal	

Centre Number	Patient Number	Treatment	ECG Date	Visit	ECG Findings	Comment
11	42	Combination	27-Oct-05	day 7	Normal	
11	42	Combination	3-Nov-05	day 14	Normal	
11	42	Combination	7-Nov-05	end day	Normal	PAC - CLINICALLY INSIGNIFICANT PACS
11	42	Combination	6-Feb-06	month 3	Normal	
11	42	Combination	7-Jun-06	month 6	Normal	
11	43	SSG	27-Oct-05	day 0	Normal	
11	43	SSG	3-Nov-05	day 7	Normal	
11	43	SSG	10-Nov-05	day 14	Normal	
11	43	SSG	17-Nov-05	day 21	Normal	
11	43	SSG	27-Nov-05	end day	Normal	
11	43	SSG	22-Feb-06	month 3	Normal	
11	43	SSG	24-May-06	month 6	Normal	
11	44	PM	27-Oct-05	day 0	Normal	
11	44	PM	3-Nov-05	day 7	Normal	
11	44	PM	10-Nov-05	day 14	Normal	
11	44	PM	18-Nov-05	end day	Normal	
11	44	PM	15-Feb-06	month 3	Normal	
11	44	PM	19-May-06	month 6	Normal	
11	45	Combination	27-Oct-05	day 0	Normal	
11	45	Combination	3-Nov-05	day 7	Normal	
11	45	Combination	10-Nov-05	day 14	Normal	
11	45	Combination	14-Nov-05	end day	Normal	
11	45	Combination	15-Feb-06	month 3	Normal	
11	45	Combination	19-May-06	month 6	Normal	
11	46	SSG	4-May-06	day 0	Normal	
11	46	SSG	11-May-06	day 7	Normal	HAS REGURLARY IRREGULAR RYTHM. QRS COMES EVERY 3-4 BUT CLINICALLY STABLE AND THE PATIENT IS PLAYFUL, NO DYSPREA, OR EARLY FATIGIABILITY
11	46	SSG	18-May-06	day 14	Normal	HAS STILL SINUS ARRHYTHMIA BUT NO CLINICAL SYMPTOMS.
11	46	SSG	25-May-06	day 21	Abnormal (CI)	SINUS ARRHYTHMIA
11	46	SSG	4-Jun-06	end day	Normal	
11	46	SSG	24-Oct-06	month 3	Normal	
11	46	SSG	5-Dec-06	month 6	Normal	
11	47	SSG	4-May-06	day 0	Normal	
11	47	SSG	11-May-06	day 7	Normal	
11	47	SSG	18-May-06	day 14	Normal	
11	47	SSG	25-May-06	day 21	Normal	
11	47	SSG	4-Jun-06	end day	Normal	
11	48	Combination	4-May-06	day 0	Normal	
11	48	Combination	11-May-06	day 7	Normal	
11	48	Combination	18-May-06	day 14	Normal	
11	48	Combination	22-May-06	end day	Normal	
11	48	Combination	2-Jan-07	month 6	Normal	
11	49	SSG	8-May-06	day 0	Normal	
11	49	SSG	15-May-06	day 7	Normal	
11	49	SSG	22-May-06	day 14	Normal	
11	49	SSG	29-May-06	day 21	Normal	
11	49	SSG	8-Jun-06	end day	Normal	
11	49	SSG	9-Oct-06	month 3	Normal	
11	49	SSG	27-Feb-07	month 6	Normal	
11	50	PM	17-May-06	day 0	Normal	

Centre Number	Patient Number	Treatment	ECG Date	Visit	ECG Findings	Comment
11	50	PM	24-May-06	day 7	Normal	
11	50	PM	31-May-06	day 14	Normal	
11	50	PM	8-Jun-06	end day	Normal	
11	50	PM	6-Sep-06	month 3	Normal	
11	50	PM	11-Dec-06	month 6	Normal	
11	51	Combination	19-May-06	day 0	Normal	
11	51	Combination	26-May-06	day 7	Normal	
11	51	Combination	2-Jun-06	day 14	Normal	
11	51	Combination	6-Jun-06	end day	Normal	
11	51	Combination	7-Sep-06	month 3	Normal	
11	51	Combination	2-Jan-07	month 6	Normal	
11	52	PM	19-May-06	day 0	Normal	
11	52	PM	26-May-06	day 7	Normal	
11	52	PM	2-Jun-06	day 14	Normal	
11	52	PM	10-Jun-06	end day	Normal	
11	52	PM	26-Sep-06	month 3	Normal	
11	52	PM	11-Jan-07	month 6	Normal	
11	53	SSG	25-May-06	day 0	Normal	
11	53	SSG	1-Jun-06	day 7	Normal	
11	53	SSG	8-Jun-06	day 14	Normal	
11	53	SSG	15-Jun-06	day 21	Normal	
11	53	SSG	25-Jun-06	end day	Normal	
11	53	SSG	26-Sep-06	month 3	Normal	
11	53	SSG	11-Dec-06	month 6	Normal	
11	54	PM	26-May-06	day 0	Normal	
11	54	PM	2-Jun-06	day 7	Normal	
11	54	PM	9-Jun-06	day 14	Normal	
11	54	PM	17-Jun-06	end day	Normal	
11	54	PM	28-Aug-06	month 3	Normal	
11	54	PM	8-Jan-07	month 6	Normal	
11	55	PM	29-May-06	day 0	Normal	
11	55	PM	5-Jun-06	day 7	Normal	
11	55	PM	12-Jun-06	day 14	Normal	
11	55	PM	20-Jun-06	end day	Normal	
11	55	PM	26-Sep-06	month 3	Normal	
11	55	PM	11-Dec-06	month 6	Normal	
11	56	SSG	29-May-06	day 0	Normal	
11	56	SSG	6-Jun-06	day 7	Normal	
11	56	SSG	13-Jun-06	day 14	Normal	
11	56	SSG	20-Jun-06	day 21	Normal	
11	56	SSG	30-Jun-06	end day	Normal	
11	56	SSG	28-Sep-06	month 3	Normal	
11	56	SSG	14-Dec-06	month 6	Normal	
11	57	Combination	3-Jun-06	day 0	Normal	
11	57	Combination	10-Jun-06	day 7	Normal	
11	57	Combination	17-Jun-06	day 14	Normal	
11	57	Combination	21-Jun-06	end day	Normal	
11	57	Combination	12-Sep-06	month 3	Normal	
11	57	Combination	12-Dec-06	month 6	Normal	
11	58	Combination	5-Jun-06	day 0	Normal	
11	58	Combination	12-Jun-06	day 7	Normal	
11	58	Combination	19-Jun-06	day 14	Normal	
11	58	Combination	23-Jun-06	end day	Normal	

Centre Number	Patient Number	Treatment	ECG Date	Visit	ECG Findings	Comment
11	58	Combination	26-Sep-06	month 3	Normal	
11	58	Combination	13-Dec-06	month 6	Normal	
11	59	PM	12-Jun-06	day 0	Normal	
11	59	PM	19-Jun-06	day 7	Normal	
11	59	PM	26-Jun-06	day 14	Normal	
11	59	PM	4-Jul-06	end day	Normal	
11	59	PM	4-Oct-06	month 3	Normal	
11	59	PM	2-Jan-07	month 6	Normal	
11	60	Combination	12-Jun-06	day 0	Normal	
11	60	Combination	19-Jun-06	day 7	Normal	
11	60	Combination	26-Jun-06	day 14	Normal	
11	60	Combination	30-Jun-06	end day	Normal	
11	60	Combination	28-Sep-06	month 3	Normal	
11	60	Combination	18-Dec-06	month 6	Normal	
11	61	PM	12-Jun-06	day 0	Normal	
11	61	PM	19-Jun-06	day 7	Normal	
11	61	PM	26-Jun-06	day 14	Normal	
11	61	PM	4-Jul-06	end day	Normal	
11	61	PM	2-Oct-06	month 3	Normal	
11	61	PM	2-Jan-07	month 6	Normal	
11	62	SSG	15-Jun-06	day 0	Normal	
11	62	SSG	22-Jun-06	day 7	Normal	
11	62	SSG	29-Jun-06	day 14	Normal	
11	62	SSG	6-Jul-06	day 21	Normal	
11	62	SSG	16-Jul-06	end day	Normal	
11	62	SSG	25-Oct-06	month 3	Normal	
11	62	SSG	10-Jan-07	month 6	Normal	
11	63	SSG	19-Jun-06	day 0	Normal	
11	63	SSG	26-Jun-06	day 7	Normal	
11	63	SSG	3-Jul-06	day 14	Normal	
11	63	SSG	10-Jul-06	day 21	Normal	
11	63	SSG	20-Jul-06	end day	Normal	
11	63	SSG	3-Oct-06	month 3	Normal	
11	63	SSG	28-Dec-06	month 6	Normal	
11	64	SSG	21-Jun-06	day 0	Normal	
11	64	SSG	28-Jun-06	day 7	Normal	
11	64	SSG	5-Jul-06	day 14	Normal	
11	64	SSG	12-Jul-06	day 21	Normal	
11	64	SSG	22-Jul-06	end day	Normal	
11	64	SSG	29-Sep-06	month 3	Normal	
11	64	SSG	14-Dec-06	month 6	Normal	
11	65	PM	21-Jun-06	day 0	Normal	
11	65	PM	29-Jun-06	day 7	Normal	
11	65	PM	6-Jul-06	day 14	Normal	
11	65	PM	14-Jul-06	end day	Normal	
11	66	Combination	22-Jun-06	day 0	Normal	
11	66	Combination	29-Jun-06	day 7	Normal	
11	66	Combination	6-Jul-06	day 14	Normal	
11	66	Combination	10-Jul-06	end day	Normal	
11	66	Combination	26-Sep-06	month 3	Normal	
11	66	Combination	11-Dec-06	month 6	Normal	
11	67	PM	30-Jun-06	day 0	Normal	
11	67	PM	7-Jul-06	day 7	Normal	

Centre Number	Patient Number	Treatment	ECG Date	Visit	ECG Findings	Comment
11	67	PM	14-Jul-06	day 14	Normal	
11	67	PM	22-Jul-06	end day	Normal	
11	67	PM	2-Oct-06	month 3	Normal	
11	67	PM	2-Jan-07	month 6	Normal	
11	68	SSG	7-Jul-06	day 0	Normal	
11	68	SSG	14-Jul-06	day 7	Normal	
11	68	SSG	21-Jul-06	day 14	Normal	
11	68	SSG	28-Jul-06	day 21	Normal	
11	68	SSG	7-Aug-06	end day	Normal	
11	68	SSG	11-Oct-06	month 3	Normal	
11	68	SSG	8-Jan-07	month 6	Normal	
11	69	Combination	14-Jul-06	day 0	Normal	
11	69	Combination	21-Jul-06	day 7	Normal	
11	69	Combination	28-Jul-06	day 14	Normal	
11	69	Combination	1-Aug-06	end day	Normal	
11	69	Combination	9-Oct-06	month 3	Normal	
11	69	Combination	13-Dec-06	month 6	Normal	
11	70	Combination	14-Jul-06	day 0	Normal	
11	70	Combination	21-Jul-06	day 7	Normal	
11	70	Combination	28-Jul-06	day 14	Normal	
11	70	Combination	1-Aug-06	end day	Normal	
11	70	Combination	9-Oct-06	month 3	Normal	
11	70	Combination	2-Jan-07	month 6	Normal	
11	71	Combination	21-Jul-06	day 0	Normal	
11	71	Combination	28-Jul-06	day 7	Normal	
11	71	Combination	4-Aug-06	day 14	Normal	
11	71	Combination	8-Aug-06	end day	Normal	
11	71	Combination	8-Jan-07	month 6	Normal	
11	72	SSG	21-Jul-06	day 0	Normal	
11	72	SSG	28-Jul-06	day 7	Normal	
11	72	SSG	4-Aug-06	day 14	Normal	
11	72	SSG	9-Nov-06	month 3	Normal	
11	72	SSG	7-Feb-07	month 6	Normal	
11	73	PM	24-Jul-06	day 0	Normal	
11	73	PM	31-Jul-06	day 7	Normal	
11	73	PM	7-Aug-06	day 14	Normal	
11	73	PM	15-Aug-06	end day	Normal	
11	73	PM	23-Oct-06	month 3	Normal	
11	73	PM	2-Apr-07	month 6	Normal	
11	74	PM	27-Jul-06	day 0	Normal	
11	74	PM	3-Aug-06	day 7	Normal	
11	74	PM	10-Aug-06	day 14	Normal	
11	74	PM	18-Aug-06	end day	Normal	
11	74	PM	25-Oct-06	month 3	Normal	
11	74	PM	23-Jan-07	month 6	Normal	
11	75	Combination	27-Jul-06	day 0	Normal	
11	75	Combination	3-Aug-06	day 7	Normal	
11	75	Combination	10-Aug-06	day 14	Normal	
11	75	Combination	14-Aug-06	end day	Normal	
11	75	Combination	31-Oct-06	month 3	Normal	
11	75	Combination	8-Jan-07	month 6	Normal	
11	76	PM	27-Jul-06	day 0	Normal	
11	76	PM	6-Aug-06	day 7	Normal	

Centre Number	Patient Number	Treatment	ECG Date	Visit	ECG Findings	Comment
11	76	PM	13-Aug-06	day 14	Normal	
11	76	PM	21-Aug-06	end day	Normal	
11	76	PM	30-Oct-06	month 3	Normal	
11	76	PM	29-Jan-07	month 6	Normal	
11	77	PM	29-Jul-06	day 0	Normal	
11	77	PM	6-Aug-06	day 7	Normal	
11	77	PM	13-Aug-06	day 14	Normal	
11	77	PM	21-Aug-06	end day	Normal	
11	77	PM	30-Oct-06	month 3	Normal	
11	77	PM	29-Jan-07	month 6	Normal	
11	78	SSG	29-Jul-06	day 0	Normal	
11	78	SSG	6-Aug-06	day 7	Normal	
11	78	SSG	13-Aug-06	day 14	Normal	
11	78	SSG	20-Aug-06	day 21	Normal	
11	78	SSG	30-Aug-06	end day	Normal	
11	78	SSG	30-Oct-06	month 3	Normal	
11	78	SSG	29-Jan-07	month 6	Normal	
11	79	PM	29-Jul-06	day 0	Normal	
11	79	PM	6-Aug-06	day 7	Normal	
11	79	PM	13-Aug-06	day 14	Normal	
11	79	PM	21-Aug-06	end day	Normal	
11	79	PM	30-Oct-06	month 3	Normal	
11	79	PM	30-Jan-07	month 6	Normal	
11	80	PM	2-Aug-06	day 0	Normal	
11	80	PM	9-Aug-06	day 7	Normal	
11	80	PM	16-Aug-06	day 14	Normal	
11	80	PM	24-Aug-06	end day	Normal	
11	80	PM	23-Nov-06	month 3	Normal	
11	80	PM	16-Mar-07	month 6	Normal	
11	81	Combination	5-Aug-06	day 0	Normal	
11	81	Combination	12-Aug-06	day 7	Normal	
11	81	Combination	19-Aug-06	day 14	Normal	
11	81	Combination	23-Aug-06	end day	Normal	
11	81	Combination	7-Nov-06	month 3	Normal	
11	81	Combination	6-Feb-07	month 6	Normal	
11	82	SSG	13-Aug-06	day 0	Normal	
11	82	SSG	21-Aug-06	day 7	Normal	
11	82	SSG	28-Aug-06	day 14	Normal	
11	82	SSG	4-Sep-06	day 21	Normal	
11	82	SSG	14-Sep-06	end day	Normal	
11	82	SSG	13-Nov-06	month 3	Normal	
11	82	SSG	5-Feb-07	month 6	Normal	
11	83	SSG	15-Aug-06	day 0	Normal	
11	83	SSG	22-Aug-06	day 7	Normal	
11	83	SSG	29-Aug-06	day 14	Normal	
11	83	SSG	5-Sep-06	day 21	Normal	
11	83	SSG	15-Sep-06	end day	Normal	
11	83	SSG	13-Nov-06	month 3	Normal	
11	83	SSG	13-Feb-07	month 6	Normal	
11	84	SSG	13-Aug-06	day 0	Normal	
11	84	SSG	24-Aug-06	day 7	Normal	
11	84	SSG	31-Aug-06	day 14	Normal	
11	84	SSG	7-Sep-06	day 21	Normal	

Centre Number	Patient Number	Treatment	ECG Date	Visit	ECG Findings	Comment
11	84	SSG	17-Sep-06	end day	Normal	
11	84	SSG	15-Nov-06	month 3	Normal	
11	84	SSG	14-Feb-07	month 6	Normal	
11	85	SSG	24-Aug-06	day 0	Normal	
11	85	SSG	31-Aug-06	day 7	Normal	
11	85	SSG	7-Sep-06	day 14	Normal	
11	85	SSG	14-Sep-06	day 21	Normal	
11	85	SSG	24-Sep-06	end day	Normal	
11	85	SSG	21-Nov-06	month 3	Normal	
11	86	Combination	28-Aug-06	day 0	Normal	
11	86	Combination	4-Sep-06	day 7	Normal	
11	86	Combination	11-Sep-06	day 14	Normal	
11	86	Combination	15-Sep-06	end day	Normal	
11	86	Combination	13-Nov-06	month 3	Normal	
11	86	Combination	5-Feb-07	month 6	Normal	
11	87	Combination	28-Aug-06	day 0	Normal	
11	87	Combination	4-Sep-06	day 7	Normal	
11	87	Combination	11-Sep-06	day 14	Normal	
11	87	Combination	15-Sep-06	end day	Normal	
11	87	Combination	14-Nov-06	month 3	Normal	
11	87	Combination	5-Feb-07	month 6	Normal	
11	88	PM	28-Aug-06	day 0	Normal	
11	88	PM	4-Sep-06	day 7	Normal	
11	88	PM	11-Sep-06	day 14	Normal	
11	88	PM	19-Sep-06	end day	Normal	
11	88	PM	21-Nov-06	month 3	Normal	
11	88	PM	27-Feb-07	month 6	Normal	
11	89	Combination	28-Aug-06	day 0	Normal	
11	89	Combination	8-Sep-06	day 7	Normal	
11	89	Combination	15-Sep-06	day 14	Normal	
11	89	Combination	19-Sep-06	end day	Normal	
11	89	Combination	17-Nov-06	month 3	Normal	
11	89	Combination	15-Mar-07	month 6	Normal	
11	90	Combination	1-Sep-06	day 0	Normal	
11	90	Combination	8-Sep-06	day 7	Normal	
11	90	Combination	15-Sep-06	day 14	Normal	
11	90	Combination	19-Sep-06	end day	Normal	
11	91	PM	17-Dec-06	day 0	Normal	
11	91	PM	31-Dec-06	day 14	Normal	
11	91	PM	8-Jan-07	end day	Normal	
11	91	PM	18-Jun-07	month 6	Normal	
11	92	Combination	30-Dec-06	day 0	Normal	
11	92	Combination	13-Jan-07	day 14	Normal	
11	92	Combination	17-Jan-07	end day	Normal	
11	92	Combination	26-Jun-07	month 6	Normal	
11	93	SSG	1-Jan-07	day 0	Normal	
11	93	SSG	8-Jan-07	day 7	Normal	
11	93	SSG	15-Jan-07	day 14	Normal	
11	93	SSG	22-Jan-07	day 21	Normal	
11	93	SSG	1-Feb-07	end day	Normal	
11	93	SSG	7-Aug-07	month 6	Normal	
11	94	SSG	3-Jan-07	day 0	Normal	
11	94	SSG	17-Jan-07	day 14	Normal	

Centre Number	Patient Number	Treatment	ECG Date	Visit	ECG Findings	Comment
11	94	SSG	3-Feb-07	end day	Normal	
11	94	SSG	25-Jun-07	month 6	Normal	
11	95	SSG	3-Jan-07	day 0	Normal	
11	95	SSG	17-Jan-07	day 14	Normal	
11	95	SSG	3-Feb-07	end day	Normal	
11	95	SSG	23-Jun-07	month 6	Normal	
11	96	Combination	19-Jan-07	day 0	Normal	
11	96	Combination	2-Feb-07	day 14	Normal	
11	96	Combination	6-Feb-07	end day	Normal	
11	96	Combination	6-Sep-07	month 6	Normal	
11	97	Combination	19-Jan-07	day 0	Normal	
11	97	Combination	2-Feb-07	day 14	Normal	
11	97	Combination	6-Feb-07	end day	Normal	
11	97	Combination	24-Jul-07	month 6	Normal	
11	98	PM	23-Jan-07	day 0	Normal	
11	98	PM	6-Feb-07	day 14	Normal	
11	98	PM	14-Feb-07	end day	Normal	
11	98	PM	26-Jul-07	month 6	Normal	
11	99	SSG	25-Jan-07	day 0	Normal	
11	99	SSG	8-Feb-07	day 14	Normal	
11	99	SSG	25-Feb-07	end day	Normal	
11	99	SSG	30-Jul-07	month 6	Normal	
11	100	PM	25-Jan-07	day 0	Normal	
11	100	PM	8-Feb-07	day 14	Normal	
11	100	PM	16-Feb-07	end day	Normal	
11	101	PM	2-Feb-07	day 0	Normal	
11	101	PM	16-Feb-07	day 14	Normal	
11	101	PM	24-Feb-07	end day	Normal	
11	101	PM	6-Nov-07	month 6	Normal	
11	102	Combination	2-Feb-07	day 0	Normal	
11	102	Combination	16-Feb-07	day 14	Normal	
11	102	Combination	20-Feb-07	end day	Normal	
11	102	Combination	30-Jul-07	month 6	Normal	
11	103	Combination	6-Feb-07	day 0	Normal	
11	103	Combination	20-Feb-07	day 14	Normal	
11	103	Combination	24-Feb-07	end day	Normal	
11	103	Combination	10-Aug-07	month 6	Normal	
11	104	PM	9-Feb-07	day 0	Normal	
11	104	PM	23-Feb-07	day 14	Normal	
11	104	PM	3-Mar-07	end day	Normal	
11	104	PM	27-Aug-07	month 6	Normal	
11	105	SSG	23-Feb-07	day 0	Normal	
11	105	SSG	9-Mar-07	day 14	Normal	
11	105	SSG	26-Mar-07	end day	Normal	
11	105	SSG	26-Aug-07	month 6	Normal	
11	106	Combination	24-Feb-07	day 0	Normal	
11	106	Combination	10-Mar-07	day 14	Normal	
11	106	Combination	14-Mar-07	end day	Normal	
11	106	Combination	19-Aug-07	month 6	Normal	
11	107	PM	24-Feb-07	day 0	Normal	
11	107	PM	10-Mar-07	day 14	Normal	
11	107	PM	18-Mar-07	end day	Normal	
11	107	PM	28-Aug-07	month 6	Normal	

Centre Number	Patient Number	Treatment	ECG Date	Visit	ECG Findings	Comment
11	108	PM	28-Feb-07	day 0	Normal	
11	108	PM	15-Mar-07	day 14	Normal	
11	108	PM	23-Mar-07	end day	Normal	
11	108	PM	27-Aug-07	month 6	Normal	
11	109	SSG	27-Feb-07	day 0	Normal	
11	109	SSG	16-Mar-07	day 14	Normal	
11	109	SSG	2-Apr-07	end day	Normal	
11	109	SSG	5-Aug-07	month 6	Normal	
11	110	PM	2-Mar-07	day 0	Normal	
11	110	PM	16-Mar-07	day 14	Normal	
11	110	PM	24-Mar-07	end day	Normal	
11	110	PM	27-Aug-07	month 6	Normal	
11	111	Combination	23-Mar-07	day 0	Normal	
11	111	Combination	6-Apr-07	day 14	Normal	
11	111	Combination	10-Apr-07	end day	Normal	
11	111	Combination	24-Sep-07	month 6	Normal	
11	112	Combination	23-Mar-07	day 0	Normal	
11	112	Combination	6-Apr-07	day 14	Normal	
11	112	Combination	10-Apr-07	end day	Normal	
11	112	Combination	24-Sep-07	month 6	Normal	
11	113	SSG	27-Mar-07	day 0	Normal	
11	113	SSG	10-Apr-07	day 14	Normal	
11	113	SSG	27-Apr-07	end day	Normal	
11	114	SSG	28-Mar-07	day 0	Normal	
11	114	SSG	12-Apr-07	day 14	Normal	
11	114	SSG	29-Apr-07	end day	Normal	
11	114	SSG	5-Oct-07	month 6	Normal	
11	115	Combination	30-Mar-07	day 0	Normal	
11	115	Combination	13-Apr-07	day 14	Normal	
11	115	Combination	17-Apr-07	end day	Normal	
11	115	Combination	21-Sep-07	month 6	Normal	
11	116	SSG	30-Mar-07	day 0	Normal	
11	116	SSG	13-Apr-07	day 14	Normal	
11	116	SSG	30-Apr-07	end day	Normal	
11	116	SSG	1-Nov-07	month 6	Normal	
11	117	PM	3-Apr-07	day 0	Normal	
11	117	PM	17-Apr-07	day 14	Normal	
11	117	PM	25-Apr-07	end day	Normal	
11	118	PM	18-Apr-07	day 0	Normal	
11	118	PM	2-May-07	day 14	Normal	
11	118	PM	10-May-07	end day	Normal	
11	118	PM	19-Sep-07	month 6	Normal	
11	119	SSG	20-Apr-07	day 0	Normal	
11	119	SSG	4-May-07	day 14	Normal	
11	119	SSG	21-May-07	end day	Normal	
11	119	SSG	29-Oct-07	month 6	Normal	
11	120	Combination	20-Apr-07	day 0	Normal	
11	120	Combination	4-May-07	day 14	Normal	
11	120	Combination	8-May-07	end day	Normal	
11	120	Combination	29-Oct-07	month 6	Normal	
11	121	Combination	20-Apr-07	day 0	Normal	
11	121	Combination	4-May-07	day 14	Normal	
11	121	Combination	8-May-07	end day	Normal	

Centre Number	Patient Number	Treatment	ECG Date	Visit	ECG Findings	Comment
11	121	Combination	29-Oct-07	month 6	Normal	
11	122	SSG	20-Apr-07	day 0	Normal	
11	122	SSG	4-May-07	day 14	Normal	
11	122	SSG	21-May-07	end day	Normal	
11	123	SSG	27-Apr-07	day 0	Normal	
11	123	SSG	11-May-07	day 14	Normal	
11	123	SSG	28-May-07	end day	Normal	
11	123	SSG	19-Oct-07	month 6	Normal	
11	124	SSG	20-May-07	day 0	Normal	
11	124	SSG	3-Jun-07	day 14	Normal	
11	124	SSG	20-Jun-07	end day	Normal	
11	124	SSG	7-Apr-08	month 6	Normal	
11	125	SSG	23-May-07	day 0	Normal	
11	125	SSG	6-Jun-07	day 14	Normal	
11	125	SSG	23-Jun-07	end day	Normal	
11	125	SSG	19-Nov-07	month 6	Normal	
11	126	Combination	26-May-07	day 0	Normal	
11	126	Combination	10-Jun-07	day 14	Normal	
11	126	Combination	14-Jun-07	end day	Normal	
11	126	Combination	10-Dec-07	month 6	Normal	
11	127	PM	26-May-07	day 0	Normal	
11	127	PM	10-Jun-07	day 14	Normal	
11	127	PM	18-Jun-07	end day	Normal	
11	127	PM	30-Oct-07	month 6	Normal	
11	128	Combination	1-Jun-07	day 0	Normal	
11	128	Combination	15-Jun-07	day 14	Normal	
11	128	Combination	19-Jun-07	end day	Normal	
11	128	Combination	16-Nov-07	month 6	Normal	
11	129	PM	6-Jun-07	day 0	Normal	
11	129	PM	20-Jun-07	day 14	Normal	
11	129	PM	28-Jun-07	end day	Normal	
11	129	PM	21-Nov-07	month 6	Normal	
11	130	Combination	7-Jun-07	day 0	Normal	
11	130	Combination	22-Jun-07	day 14	Normal	
11	130	Combination	26-Jun-07	end day	Normal	
11	130	Combination	19-Nov-07	month 6	Normal	
11	131	PM	10-Jun-07	day 0	Normal	
11	131	PM	24-Jun-07	day 14	Normal	
11	131	PM	2-Jul-07	end day	Normal	
11	131	PM	12-Dec-07	month 6	Normal	
11	132	SSG	15-Jun-07	day 0	Normal	
11	132	SSG	29-Jun-07	day 14	Normal	
11	132	SSG	16-Jul-07	end day	Normal	
11	132	SSG	19-Dec-07	month 6	Normal	
11	133	Combination	3-Jul-07	day 0	Normal	
11	133	Combination	17-Jul-07	day 14	Normal	
11	133	Combination	21-Jul-07	end day	Normal	
11	133	Combination	2-Jan-08	month 6	Normal	
11	134	PM	10-Jul-07	day 0	Normal	
11	134	PM	24-Jul-07	day 14	Normal	
11	134	PM	1-Aug-07	end day	Normal	
11	134	PM	8-Jan-08	month 6	Normal	
11	135	PM	12-Jul-07	day 0	Normal	

Centre Number	Patient Number	Treatment	ECG Date	Visit	ECG Findings	Comment
11	135	PM	26-Jul-07	day 14	Normal	
11	135	PM	3-Aug-07	end day	Normal	
11	135	PM	31-Dec-07	month 6	Normal	
12	241	Combination	28-Jun-05	day 0	Normal	CLINICALLY NO CARDIAC ABNORMALITY DETECTED
12	241	Combination	5-Jul-05	day 7	Normal	
12	241	Combination	12-Jul-05	day 14	Normal	
12	241	Combination	16-Jul-05	end day	Normal	
12	241	Combination	9-Feb-06	month 6	Normal	
12	242	SSG	28-Jun-05	day 0	Normal	
12	242	SSG	5-Jul-05	day 7	Normal	
12	242	SSG	12-Jul-05	day 14	Normal	
12	242	SSG	19-Jul-05	day 21	Normal	
12	242	SSG	29-Jul-05	end day	Normal	
12	242	SSG	24-Oct-05	month 3	Normal	
12	242	SSG	9-Feb-06	month 6	Normal	
12	243	PM	28-Jun-05	day 0	Normal	
12	243	PM	5-Jul-05	day 7	Normal	
12	243	PM	12-Jul-05	day 14	Normal	
12	243	PM	20-Jul-05	end day	Normal	
12	243	PM	4-Nov-05	month 3	Normal	
12	243	PM	6-Aug-06	month 6	Normal	
12	244	SSG	28-Jun-05	day 0	Normal	
12	244	SSG	5-Jul-05	day 7	Normal	
12	244	SSG	12-Jul-05	day 14	Normal	
12	244	SSG	19-Jul-05	day 21	Normal	
12	244	SSG	29-Jul-05	end day	Normal	
12	244	SSG	29-Oct-05	month 3	Normal	
12	244	SSG	6-Apr-06	month 6	Normal	
12	245	PM	15-Jul-05	day 0	Normal	
12	245	PM	25-Jul-05	day 7	Normal	
12	245	PM	1-Aug-05	day 14	Normal	
12	245	PM	9-Aug-05	end day	Normal	
12	245	PM	17-Nov-05	month 3	Normal	
12	246	PM	3-Aug-05	day 0	Normal	
12	246	PM	10-Aug-05	day 7	Normal	
12	246	PM	17-Aug-05	day 14	Normal	
12	246	PM	25-Aug-05	end day	Normal	
12	246	PM	25-Nov-05	month 3	Normal	
12	247	PM	12-Sep-05	day 0	Normal	
12	247	PM	4-Oct-05	day 7	Normal	
12	247	PM	11-Oct-05	day 14	Normal	
12	247	PM	19-Oct-05	end day	Normal	
12	247	PM	18-Jan-06	month 3	Normal	
12	247	PM	19-Apr-06	month 6	Normal	
12	248	PM	23-Sep-05	day 0	Normal	
12	248	PM	30-Sep-05	day 7	Normal	
12	248	PM	6-Oct-05	day 14	Normal	
12	248	PM	14-Oct-05	end day	Normal	
12	248	PM	23-Jan-06	month 3	Normal	
12	248	PM	19-Apr-06	month 6	Normal	
12	249	Combination	24-Oct-05	day 0	Normal	
12	249	Combination	31-Oct-05	day 7	Normal	

Centre Number	Patient Number	Treatment	ECG Date	Visit	ECG Findings	Comment
12	249	Combination	7-Nov-05	day 14	Normal	
12	249	Combination	11-Nov-05	end day	Normal	
12	249	Combination	4-Mar-06	month 3	Normal	
12	249	Combination	13-May-06	month 6	Normal	
12	250	SSG	24-Oct-05	day 0	Normal	
12	250	SSG	31-Oct-05	day 7	Normal	
12	250	SSG	7-Nov-05	day 14	Normal	
12	250	SSG	14-Nov-05	day 21	Normal	
12	250	SSG	24-Nov-05	end day	Normal	
12	250	SSG	22-Feb-06	month 3	Normal	
12	250	SSG	8-Jul-06	month 6	Normal	
12	251	Combination	27-Oct-05	day 0	Normal	
12	251	Combination	4-Nov-05	day 7	Normal	
12	251	Combination	11-Nov-05	day 14	Normal	
12	251	Combination	15-Nov-05	end day	Normal	
12	251	Combination	12-Feb-06	month 3	Normal	
12	251	Combination	14-May-06	month 6	Normal	
12	252	SSG	27-Oct-05	day 0	Normal	
12	252	SSG	11-Nov-05	day 14	Normal	
12	252	SSG	18-Nov-05	day 21	Normal	
12	252	SSG	28-Nov-05	end day	Normal	
12	252	SSG	6-Apr-06	month 3	Normal	
12	252	SSG	22-Jun-06	month 6	Normal	
12	253	SSG	27-Oct-05	day 0	Normal	
12	253	SSG	4-Nov-05	day 7	Normal	
12	253	SSG	11-Nov-05	day 14	Normal	
12	253	SSG	18-Nov-05	day 21	Normal	
12	253	SSG	27-Nov-05	end day	Normal	
12	253	SSG	5-Apr-06	month 3	Normal	
12	253	SSG	22-Jun-06	month 6	Normal	
12	254	Combination	27-Oct-05	day 0	Normal	
12	254	Combination	4-Nov-05	day 7	Normal	
12	254	Combination	11-Nov-05	day 14	Normal	
12	254	Combination	15-Nov-05	end day	Normal	
12	254	Combination	12-Feb-06	month 3	Normal	
12	254	Combination	24-May-06	month 6	Normal	
12	255	Combination	29-Oct-05	day 0	Normal	
12	255	Combination	7-Nov-05	day 7	Normal	
12	255	Combination	14-Nov-05	day 14	Normal	
12	255	Combination	18-Nov-05	end day	Normal	
12	255	Combination	9-Mar-06	month 3	Normal	
12	255	Combination	17-May-06	month 6	Normal	
12	256	Combination	17-Nov-05	day 0	Normal	
12	256	Combination	24-Nov-05	day 7	Normal	SHORT PR INTERVAL
12	256	Combination	30-Nov-05	day 14	Normal	SHORT PR INTERVAL
12	256	Combination	5-Dec-05	end day	Normal	SHORT PR INTERVAL
12	256	Combination	4-Mar-06	month 3	Normal	
12	256	Combination	6-Jun-06	month 6	Normal	
12	257	SSG	14-Nov-05	day 0	Normal	
12	257	SSG	24-Nov-05	day 7	Normal	
12	257	SSG	30-Nov-05	day 14	Normal	
12	257	SSG	7-Dec-05	day 21	Normal	
12	257	SSG	17-Dec-05	end day	Normal	

Centre Number	Patient Number	Treatment	ECG Date	Visit	ECG Findings	Comment
12	257	SSG	19-Apr-06	month 3	Normal	
12	257	SSG	14-Jun-06	month 6	Normal	
12	258	SSG	30-Nov-05	day 0	Normal	
12	258	SSG	12-Dec-05	day 7	Normal	
12	258	SSG	19-Dec-05	day 14	Normal	
12	258	SSG	26-Dec-05	day 21	Normal	
12	258	SSG	5-Jan-06	end day	Normal	
12	258	SSG	14-Jul-06	month 6	Normal	
12	259	PM	26-Dec-05	day 0	Normal	
12	259	PM	6-Jan-06	day 7	Normal	
12	259	PM	13-Jan-06	day 14	Normal	
12	259	PM	20-Jan-06	end day	Normal	
12	259	PM	8-May-06	month 3	Normal	
12	259	PM	27-Jul-06	month 6	Normal	
12	260	PM	25-Dec-05	day 0	Normal	
12	260	PM	9-Jan-06	day 7	Normal	
12	260	PM	16-Jan-06	day 14	Normal	
12	260	PM	24-Jan-06	end day	Normal	
12	260	PM	11-Jul-06	month 6	Normal	
12	261	Combination	26-Dec-05	day 0	Normal	
12	261	Combination	9-Jan-06	day 7	Normal	
12	261	Combination	16-Jan-06	day 14	Normal	
12	261	Combination	20-Jan-06	end day	Normal	
12	261	Combination	19-Apr-06	month 3	Normal	
12	261	Combination	21-Jul-06	month 6	Normal	
12	262	PM	26-Dec-05	day 0	Normal	
12	262	PM	9-Jan-06	day 7	Normal	
12	262	PM	16-Jan-06	day 14	Normal	
12	262	PM	23-Jan-06	end day	Normal	
12	262	PM	22-Jun-06	month 3	Normal	
12	262	PM	27-Jul-06	month 6	Normal	
12	263	SSG	23-Jan-06	day 0	Normal	
12	263	SSG	1-Feb-06	day 7	Normal	
12	263	SSG	9-Feb-06	day 14	Normal	
12	263	SSG	15-Feb-06	day 21	Normal	
12	263	SSG	25-Feb-06	end day	Normal	
12	263	SSG	29-May-06	month 3	Normal	
12	263	SSG	25-Aug-06	month 6	Normal	
12	264	Combination	11-Feb-06	day 0	Normal	
12	264	Combination	21-Feb-06	day 7	Normal	
12	264	Combination	28-Feb-06	day 14	Normal	
12	264	Combination	4-Mar-06	end day	Normal	
12	264	Combination	16-Jun-06	month 3	Normal	
12	264	Combination	1-Sep-06	month 6	Normal	
12	265	SSG	11-Feb-06	day 0	Normal	
12	265	SSG	21-Feb-06	day 7	Normal	
12	265	SSG	28-Feb-06	day 14	Normal	
12	265	SSG	7-Mar-06	day 21	Normal	
12	265	SSG	15-Mar-06	end day	Normal	
12	266	PM	11-Feb-06	day 0	Normal	
12	266	PM	21-Feb-06	day 7	Normal	
12	266	PM	28-Feb-06	day 14	Normal	
12	266	PM	8-Mar-06	end day	Normal	

Centre Number	Patient Number	Treatment	ECG Date	Visit	ECG Findings	Comment
12	266	PM	3-Jun-06	month 3	Normal	
12	266	PM	3-Oct-06	month 6	Normal	
12	267	SSG	11-Feb-06	day 0	Normal	
12	267	SSG	21-Feb-06	day 7	Normal	
12	267	SSG	28-Feb-06	day 14	Normal	
12	267	SSG	7-Mar-06	day 21	Normal	
12	267	SSG	17-Mar-06	end day	Normal	
12	267	SSG	14-Jun-06	month 3	Normal	
12	267	SSG	15-Sep-06	month 6	Normal	
12	268	PM	7-Mar-06	day 0	Normal	
12	268	PM	17-Mar-06	day 7	Normal	
12	268	PM	23-Mar-06	day 14	Normal	
12	268	PM	3-Apr-06	end day	Normal	
12	268	PM	29-Jun-06	month 3	Normal	
12	268	PM	26-Sep-06	month 6	Normal	
12	269	Combination	15-Mar-06	day 0	Abnormal (CI)	SINUS TACHY CARDIA ON ECG BUT IT HAS NO CLINICALLY SIGNIFICANT ABNORMALITY DETECTED
12	269	Combination	22-Mar-06	day 7	Normal	
12	269	Combination	29-Mar-06	day 14	Abnormal (CI)	NON SPECIFIC T-WAVE ABNORMALITY
12	269	Combination	3-Apr-06	end day	Normal	SINUS TACHY CARDIA ABNORMAL RHYTHM ECG
12	269	Combination	8-Jul-06	month 3	Normal	
12	269	Combination	14-Nov-06	month 6	Normal	
12	270	Combination	15-Mar-06	day 0	Normal	
12	270	Combination	22-Mar-06	day 7	Normal	
12	270	Combination	29-Mar-06	day 14	Normal	
12	270	Combination	3-Apr-06	end day	Normal	
12	270	Combination	10-Oct-06	month 6	Normal	
12	271	SSG	27-Mar-06	day 0	Normal	
12	271	SSG	6-Apr-06	day 7	Normal	
12	271	SSG	14-Apr-06	day 14	Normal	
12	271	SSG	21-Apr-06	day 21	Normal	
12	271	SSG	30-Apr-06	end day	Abnormal (CS)	LONG QTC INTERNAL
12	271	SSG	1-Aug-06	month 3	Normal	
12	271	SSG	31-Oct-06	month 6	Normal	
12	272	PM	6-Apr-06	day 0	Normal	
12	272	PM	14-Apr-06	day 7	Abnormal (CI)	SHORT QTC INTERVAL
12	272	PM	21-Apr-06	day 14	Abnormal (CI)	SHORT QT WAVE BUT CLINICALLY NOT SIGNIFICANT
12	272	PM	30-Apr-06	end day	Normal	
12	272	PM	26-Jun-06	month 3	Normal	
12	272	PM	11-Nov-06	month 6	Normal	
12	273	SSG	27-Mar-06	day 0	Normal	
12	273	SSG	14-Apr-06	day 7	Normal	
12	273	SSG	21-Apr-06	day 14	Normal	
12	273	SSG	28-Apr-06	day 21	Normal	
12	273	SSG	8-May-06	end day	Normal	
12	273	SSG	8-Aug-06	month 3	Normal	
12	273	SSG	11-Nov-06	month 6	Normal	
12	274	SSG	6-Apr-06	day 0	Abnormal (CI)	MARKED SINUS ARRHYTHMIA
12	274	SSG	14-Apr-06	day 7	Normal	

Centre Number	Patient Number	Treatment	ECG Date	Visit	ECG Findings	Comment
12	274	SSG	21-Apr-06	day 14	Normal	
12	274	SSG	28-Apr-06	day 21	Abnormal (CI)	NON SPECIFIC T-WAVE ABNORMALITY
12	274	SSG	8-May-06	end day	Normal	
12	274	SSG	8-Aug-06	month 3	Normal	1102 SINUS ARRHYTHMIA LONG QTCINTERVAL ABNORMAL ECG
12	274	SSG	11-Nov-06	month 6	Normal	
12	275	Combination	6-Apr-06	day 0	Normal	
12	275	Combination	14-Apr-06	day 7	Normal	
12	275	Combination	21-Apr-06	day 14	Normal	
12	275	Combination	25-Apr-06	end day	Normal	
12	275	Combination	25-Jul-06	month 3	Normal	
12	275	Combination	25-Oct-06	month 6	Normal	
12	276	PM	6-Apr-06	day 0	Normal	NONSPECIFIC T-WAVE ABNORMALITY BORDERLINE ECG
12	276	PM	14-Apr-06	day 7	Normal	
12	276	PM	21-Apr-06	day 14	Normal	
12	276	PM	30-Apr-06	end day	Normal	
12	276	PM	19-Jul-06	month 3	Normal	IT WAS DONE AT TIME OF VISIT BUT SOURCE DOCUMENT MISPLACED
12	276	PM	31-Oct-06	month 6	Normal	
12	277	Combination	19-Apr-06	day 0	Normal	
12	277	Combination	27-Apr-06	day 7	Normal	
12	277	Combination	5-May-06	day 14	Normal	
12	277	Combination	8-May-06	end day	Normal	
12	277	Combination	7-Aug-06	month 3	Normal	
12	277	Combination	11-Nov-06	month 6	Normal	
12	278	SSG	19-Apr-06	day 0	Normal	
12	278	SSG	27-Apr-06	day 7	Normal	
12	278	SSG	5-May-06	day 14	Normal	
12	278	SSG	11-May-06	day 21	Normal	
12	278	SSG	21-May-06	end day	Normal	
12	278	SSG	18-Aug-06	month 3	Normal	
12	279	PM	19-Apr-06	day 0	Normal	
12	279	PM	27-Apr-06	day 7	Normal	
12	279	PM	4-May-06	day 14	Normal	
12	279	PM	12-May-06	end day	Normal	
12	279	PM	12-Aug-06	month 3	Normal	
12	279	PM	11-Nov-06	month 6	Normal	
12	280	SSG		day 0	Not Done	
12	280	SSG	28-Apr-06	day 7	Normal	
12	280	SSG	4-May-06	day 14	Normal	
12	280	SSG	12-May-06	day 21	Normal	
12	280	SSG	21-May-06	end day	Normal	
12	280	SSG	25-Aug-06	month 3	Normal	
12	280	SSG	22-Nov-06	month 6	Normal	
12	281	Combination	19-Apr-06	day 0	Normal	
12	281	Combination	27-Apr-06	day 7	Normal	
12	281	Combination	5-May-06	day 14	Normal	
12	281	Combination	8-May-06	end day	Normal	TWAVE ABNORMALITY POSSIBLE ANTERIOR ISCHEMIA
12	281	Combination	8-Aug-06	month 3	Normal	
12	281	Combination	22-Dec-06	month 6	Normal	
12	282	PM	28-Apr-06	day 0	Normal	

Centre Number	Patient Number	Treatment	ECG Date	Visit	ECG Findings	Comment
12	282	PM	4-May-06	day 7	Normal	
12	282	PM	11-May-06	day 14	Normal	
12	282	PM	19-May-06	end day	Normal	
12	282	PM	25-Aug-06	month 3	Normal	
12	282	PM	20-Dec-06	month 6	Normal	
12	283	PM	11-May-06	day 0	Normal	
12	283	PM	17-May-06	day 7	Normal	
12	283	PM	24-May-06	day 14	Normal	
12	283	PM	1-Jun-06	end day	Normal	
12	283	PM	25-Aug-06	month 3	Normal	
12	283	PM	29-Dec-06	month 6	Normal	
12	284	Combination	19-May-06	day 0	Normal	
12	284	Combination	27-May-06	day 7	Normal	
12	284	Combination	3-Jun-06	day 14	Normal	
12	284	Combination	6-Jun-06	end day	Normal	
12	284	Combination	5-Sep-06	month 3	Normal	
12	284	Combination	6-Dec-06	month 6	Normal	
12	285	Combination	19-May-06	day 0	Normal	
12	285	Combination	27-May-06	day 7	Normal	
12	285	Combination	2-Jun-06	day 14	Normal	
12	285	Combination	6-Jun-06	end day	Normal	
12	285	Combination	5-Sep-06	month 3	Normal	
12	285	Combination	6-Dec-06	month 6	Normal	
12	286	PM	26-Dec-06	day 0	Normal	
12	286	PM	16-Jan-02	day 14	Normal	
12	286	PM	23-Jan-07	end day	Normal	
12	286	PM	1-May-07	month 3	Normal	
12	286	PM	4-Sep-07	month 6	Normal	
12	287	SSG	26-Dec-06	day 0	Normal	
12	287	SSG	16-Jan-07	day 14	Normal	
12	287	SSG	1-Feb-07	end day	Normal	
12	287	SSG	16-Jun-07	month 3	Normal	
12	287	SSG	7-Aug-07	month 6	Normal	
12	288	Combination	30-Dec-06	day 0	Normal	
12	288	Combination	8-Jan-07	day 7	Normal	MINIMAL VOLTAGE ENTERIA FOR LVH MAYBE NORMAL VARIANT BORDER LINE ECG
12	288	Combination	16-Jan-07	day 14	Normal	SINUS ARRHYTHMIA BORDERLINE ECG
12	288	Combination	19-Jan-07	end day	Normal	
12	288	Combination	21-Apr-07	month 3	Normal	
12	288	Combination	24-Jul-07	month 6	Normal	
12	289	Combination	30-Dec-06	day 0	Normal	
12	289	Combination	8-Jan-07	day 7	Normal	
12	289	Combination	16-Jan-07	day 14	Normal	
12	289	Combination	19-Jan-07	end day	Normal	
12	289	Combination	2-Sep-07	month 6	Normal	
12	290	SSG	5-Jan-07	day 0	Normal	
12	290	SSG	19-Jan-07	day 14	Normal	
12	290	SSG	27-Jan-07	day 21	Normal	ECG WAS DONE FOR CLINICAL REASON
12	290	SSG	6-Feb-07	end day	Normal	
12	290	SSG	6-Sep-07	month 6	Normal	
12	291	SSG	8-Jan-07	day 0	Normal	
12	291	SSG	25-Jan-07	day 14	Normal	

Centre Number	Patient Number	Treatment	ECG Date	Visit	ECG Findings	Comment
12	291	SSG	10-Feb-07	end day	Normal	
12	291	SSG	4-Sep-07	month 6	Normal	
12	292	PM	14-Jan-07	day 0	Normal	
12	292	PM	1-Feb-07	day 14	Normal	SINUS RHYTHM ABNORMAL ECG
12	292	PM	7-Feb-07	end day	Normal	
12	292	PM	21-Aug-07	month 6	Normal	
12	293	SSG	21-Jan-07	day 0	Normal	
12	293	SSG	6-Feb-07	day 14	Normal	NON SPECIFIC T-WAVE ABNORMALITY BORDER LINE ECG
12	293	SSG	23-Feb-07	end day	Normal	NON SPECIFIC T WAVE ABNORMALI SINUS ARRHYTHMIA
12	293	SSG	6-Sep-07	month 6	Normal	
12	294	Combination	20-Feb-07	day 0	Normal	
12	294	Combination	7-Mar-07	day 14	Normal	
12	294	Combination	12-Mar-07	end day	Normal	
12	294	Combination	11-Jun-07	month 3	Normal	
12	294	Combination	11-Sep-07	month 6	Normal	
12	295	Combination	12-Mar-07	day 0	Normal	
12	295	Combination	30-Mar-07	day 14	Normal	
12	295	Combination	2-Apr-07	end day	Normal	
12	295	Combination	20-Sep-07	month 6	Normal	
12	296	PM	10-Apr-07	day 0	Normal	
12	296	PM	25-Apr-07	day 14	Normal	
12	296	PM	3-May-07	end day	Normal	
12	296	PM	31-Jul-07	month 3	Normal	
12	296	PM	30-Oct-07	month 6	Normal	
12	297	SSG	11-Apr-07	day 0	Normal	
12	297	SSG	25-Apr-07	day 14	Normal	
12	298	PM	18-Apr-07	day 0	Normal	
12	298	PM	4-May-07	day 14	Normal	
12	298	PM	12-May-07	end day	Normal	
12	298	PM	9-Nov-07	month 6	Normal	
12	299	Combination	3-Jun-07	day 0	Normal	
12	299	Combination	18-Jun-07	day 14	Normal	
12	299	Combination	22-Jun-07	end day	Normal	
12	299	Combination	1-Jan-08	month 6	Normal	
12	300	PM	3-Jun-07	day 0	Normal	
12	300	PM	18-Jun-07	day 14	Normal	
12	300	PM	26-Jun-07	end day	Normal	
12	300	PM	24-Dec-07	month 6	Normal	
12	301	SSG	3-Jun-07	day 0	Normal	
12	301	SSG	18-Jun-07	day 14	Normal	
12	301	SSG	5-Jul-07	end day	Normal	
12	301	SSG	4-Feb-08	month 6	Normal	
12	302	SSG	3-Jun-07	day 0	Normal	
12	302	SSG	18-Jun-07	day 14	Not Done	
12	302	SSG	5-Jul-07	end day	Normal	
12	302	SSG	26-Dec-07	month 6	Normal	
12	303	PM	3-Jun-07	day 0	Normal	
12	303	PM	18-May-07	day 14	Normal	
12	303	PM	26-Jun-07	end day	Normal	
12	303	PM	26-Dec-07	month 6	Normal	
12	304	Combination	3-Jun-07	day 0	Normal	

Centre Number	Patient Number	Treatment	ECG Date	Visit	ECG Findings	Comment
12	304	Combination	18-Jun-07	day 14	Normal	
12	304	Combination	22-Jun-07	end day	Normal	
12	304	Combination	26-Dec-07	month 6	Normal	
12	305	PM	3-Jun-07	day 0	Normal	
12	305	PM	18-Jun-07	day 14	Normal	
12	305	PM	26-Jun-07	end day	Normal	
12	305	PM	26-Dec-07	month 6	Normal	
12	306	Combination	3-Jun-07	day 0	Normal	
12	306	Combination	18-Jun-07	day 14	Normal	
12	306	Combination	22-Jun-07	end day	Normal	
12	306	Combination	26-Dec-07	month 6	Normal	
12	307	Combination	13-Jun-07	day 0	Normal	
12	307	Combination	28-Jun-07	day 14	Normal	
12	307	Combination	2-Jul-07	end day	Normal	
12	307	Combination	26-Dec-07	month 6	Normal	
12	308	PM	13-Jun-07	day 0	Normal	
12	308	PM	28-Jun-07	day 14	Normal	
12	308	PM	6-Jul-07	end day	Normal	
12	308	PM	26-Dec-07	month 6	Normal	
12	309	PM	16-Jun-07	day 0	Normal	
12	309	PM	29-Jun-07	day 14	Normal	
12	309	PM	7-Jul-07	end day	Normal	
12	309	PM	26-Dec-07	month 6	Normal	
12	310	SSG	16-Jun-07	day 0	Normal	
12	310	SSG	29-Jun-07	day 14	Normal	
12	310	SSG	17-Jul-07	end day	Normal	
12	310	SSG	4-Feb-08	month 6	Normal	
12	311	SSG	4-Jul-07	day 0	Normal	
12	311	SSG	19-Jul-07	day 14	Normal	
12	311	SSG	4-Aug-07	end day	Normal	
12	311	SSG	11-Mar-08	month 6	Normal	
12	312	Combination	10-Jul-07	day 0	Normal	
12	312	Combination	25-Jul-07	day 14	Normal	
12	312	Combination	29-Jul-07	end day	Normal	
12	312	Combination	19-Mar-08	month 6	Normal	
12	313	PM	31-Jul-07	day 0	Normal	
12	313	PM	15-Aug-07	day 14	Normal	
12	313	PM	23-Aug-07	end day	Normal	
12	313	PM	21-Feb-08	month 6	Normal	
12	314	SSG	31-Jul-07	day 0	Normal	
12	314	SSG	21-Aug-07	day 14	Normal	
12	314	SSG	6-Sep-07	end day	Normal	
12	314	SSG	4-Mar-08	month 6	Normal	
12	315	Combination	21-Aug-07	day 0	Normal	
12	315	Combination	11-Sep-07	day 14	Normal	
12	315	Combination	15-Sep-07	end day	Normal	
12	315	Combination	11-Aug-08	month 6	Normal	
12	316	SSG	21-Aug-07	day 0	Normal	
12	316	SSG	11-Sep-07	day 14	Normal	
12	316	SSG	2-Apr-08	month 6	Normal	
12	317	SSG	21-Aug-07	day 0	Normal	
12	317	SSG	11-Sep-07	day 14	Normal	
12	317	SSG	29-Sep-07	end day	Normal	

Centre Number	Patient Number	Treatment	ECG Date	Visit	ECG Findings	Comment
12	317	SSG	22-Apr-08	month 6	Normal	
12	318	Combination	28-Aug-07	day 0	Normal	
12	318	Combination	11-Sep-07	day 14	Normal	
12	318	Combination	15-Sep-07	end day	Normal	
12	318	Combination	15-Mar-08	month 6	Normal	
12	319	Combination		day 0	Normal	
12	319	Combination	11-Sep-07	day 14	Normal	
12	319	Combination	15-Sep-07	end day	Normal	
12	319	Combination	15-Mar-08	month 6	Normal	
12	320	Combination	4-Sep-07	day 0	Normal	
12	320	Combination	19-Sep-07	day 14	Normal	
12	320	Combination	23-Sep-07	end day	Normal	
12	320	Combination	19-Mar-08	month 6	Normal	
12	321	Combination	4-Sep-07	day 0	Normal	
12	321	Combination	19-Sep-07	day 14	Normal	
12	321	Combination	23-Sep-07	end day	Normal	
12	321	Combination	24-Mar-08	month 6	Normal	
12	322	PM	11-Sep-07	day 0	Normal	
12	322	PM	26-Sep-07	day 14	Normal	
12	322	PM	4-Oct-07	end day	Normal	
12	322	PM	20-Apr-08	month 6	Normal	
12	323	SSG	19-Sep-07	day 0	Normal	
12	323	SSG	3-Oct-07	day 14	Normal	
12	323	SSG	20-Oct-07	end day	Normal	
12	323	SSG	17-Apr-08	month 6	Normal	
12	324	SSG	19-Sep-07	day 0	Normal	
12	324	SSG	3-Oct-07	day 14	Normal	
12	324	SSG	20-Oct-07	end day	Normal	
12	324	SSG	17-Apr-08	month 6	Normal	
12	325	PM	19-Sep-07	day 0	Normal	
12	325	PM	10-Oct-07	day 14	Normal	
12	325	PM	18-Oct-07	end day	Normal	
12	325	PM	17-Apr-08	month 6	Normal	
12	326	PM	2-Oct-07	day 0	Normal	
12	326	PM	16-Oct-07	day 14	Normal	
12	326	PM	24-Oct-07	end day	Normal	
12	326	PM	20-Apr-08	month 6	Normal	
12	327	Combination	2-Oct-07	day 0	Normal	
12	327	Combination	16-Oct-07	day 14	Normal	
12	327	Combination	20-Oct-07	end day	Normal	
12	327	Combination	17-Apr-08	month 6	Normal	
12	328	PM	3-Oct-07	day 0	Normal	
12	328	PM		day 14	Not Done	
12	328	PM	26-Oct-07	end day	Normal	
12	328	PM	14-Apr-08	month 6	Normal	
12	329	SSG	10-Oct-07	day 0	Normal	
12	329	SSG	23-Oct-07	day 14	Normal	
12	329	SSG	9-Nov-07	end day	Normal	
12	329	SSG	5-May-08	month 6	Normal	
12	330	PM	10-Oct-07	day 0	Normal	
12	330	PM	23-Oct-07	day 14	Normal	
12	330	PM	31-Oct-07	end day	Normal	
12	330	PM	5-May-08	month 6	Normal	

Centre Number	Patient Number	Treatment	ECG Date	Visit	ECG Findings	Comment
23	361	Combination	18-Jan-05	day 0	Normal	
23	361	Combination	26-Jan-05	day 7	Normal	
23	361	Combination	2-Feb-05	day 14	Normal	
23	361	Combination	6-Feb-05	end day	Normal	
23	361	Combination	5-May-05	month 3	Normal	
23	361	Combination	10-Aug-05	month 6	Normal	
23	362	PM	18-Jan-05	day 0	Normal	
23	362	PM	26-Jan-05	day 7	Normal	
23	362	PM	2-Feb-05	day 14	Normal	
23	362	PM	10-Feb-05	end day	Normal	
23	362	PM	10-May-05	month 3	Normal	
23	362	PM	16-Aug-05	month 6	Normal	
23	363	PM	18-Jan-05	day 0	Normal	
23	363	PM	26-Jan-05	day 7	Normal	
23	363	PM	2-Feb-05	day 14	Normal	
23	363	PM	10-Feb-05	end day	Normal	
23	363	PM	5-May-05	month 3	Normal	
23	363	PM	10-Aug-05	month 6	Normal	
23	364	Combination	18-Jan-05	day 0	Normal	
23	364	Combination	26-Jan-05	day 7	Normal	
23	364	Combination	2-Feb-05	day 14	Normal	
23	364	Combination	6-Feb-05	end day	Normal	
23	364	Combination	11-May-05	month 3	Normal	
23	365	SSG	18-Jan-05	day 0	Normal	
23	365	SSG	26-Jan-05	day 7	Normal	
23	365	SSG	2-Feb-05	day 14	Normal	
23	365	SSG	10-Feb-05	day 21	Normal	
23	365	SSG	19-Feb-05	end day	Normal	
23	365	SSG	20-May-05	month 3	Normal	
23	365	SSG	30-Aug-05	month 6	Normal	
23	366	Combination	28-Jan-05	day 0	Normal	
23	366	Combination	7-Feb-05	day 7	Normal	
23	366	Combination	14-Feb-05	day 14	Normal	
23	366	Combination	18-Feb-05	end day	Normal	
23	366	Combination	20-May-05	month 3	Normal	
23	366	Combination	19-Aug-05	month 6	Normal	
23	367	SSG	28-Jan-05	day 0	Normal	
23	367	SSG	7-Feb-05	day 7	Normal	
23	367	SSG	14-Feb-05	day 14	Normal	
23	367	SSG	21-Feb-05	day 21	Normal	
23	367	SSG	3-Mar-05	end day	Normal	
23	367	SSG	8-Jun-05	month 3	Normal	
23	367	SSG	23-Sep-05	month 6	Normal	
23	368	PM	28-Jan-05	day 0	Normal	
23	368	PM	7-Feb-05	day 7	Normal	
23	368	PM	14-Feb-05	day 14	Normal	
23	368	PM	22-Feb-05	end day	Normal	
23	368	PM	26-May-05	month 3	Normal	
23	368	PM	5-Sep-05	month 6	Normal	
23	369	Combination	28-Jan-05	day 0	Normal	
23	369	Combination	10-Feb-05	day 7	Normal	
23	369	Combination		day 14	Normal	
23	369	Combination	21-Feb-05	end day	Normal	

Centre Number	Patient Number	Treatment	ECG Date	Visit	ECG Findings	Comment
23	369	Combination	26-May-05	month 3	Normal	
23	369	Combination	30-Aug-05	month 6	Normal	
23	370	SSG	28-Jan-05	day 0	Normal	
23	370	SSG	10-Feb-05	day 7	Normal	
23	370	SSG	17-Feb-05	day 14	Normal	
23	370	SSG	24-Feb-05	day 21	Normal	
23	370	SSG	6-Mar-05	end day	Normal	
23	370	SSG	8-Jun-05	month 3	Normal	
23	370	SSG	23-Sep-05	month 6	Normal	
23	371	PM	4-Feb-05	day 0	Normal	
23	371	PM	10-Feb-05	day 7	Normal	
23	371	PM	17-Feb-05	day 14	Normal	
23	371	PM	25-Feb-05	end day	Normal	
23	371	PM	26-May-05	month 3	Normal	
23	371	PM	5-Sep-05	month 6	Normal	
23	372	PM	10-Feb-05	day 0	Normal	
23	372	PM	17-Feb-05	day 7	Normal	
23	372	PM	24-Feb-05	day 14	Normal	
23	372	PM	4-Mar-05	end day	Normal	
23	372	PM	6-Jun-05	month 3	Abnormal (CS)	SINUS BRADYCARDIA OF 49BPM REGULAR
23	372	PM	10-Sep-05	month 6	Abnormal (CS)	BRADYCARDIA
23	373	SSG	17-Feb-05	day 0	Normal	
23	373	SSG	24-Feb-05	day 7	Normal	
23	373	SSG	3-Mar-05	day 14	Normal	
23	373	SSG	10-Mar-05	day 21	Normal	
23	373	SSG	20-Mar-05	end day	Normal	
23	373	SSG	6-Jul-05	month 3	Abnormal (CS)	SINUS BRADY CARDIA OF 45 BPM
23	373	SSG	24-Oct-05	month 6	Abnormal (CS)	BRADY CARDIA 52BPM
23	374	SSG	18-Feb-05	day 0	Normal	
23	374	SSG	2-Mar-05	day 7	Normal	
23	374	SSG	9-Mar-05	day 14	Normal	
23	374	SSG	16-Mar-05	day 21	Normal	
23	374	SSG	28-Mar-05	end day	Normal	
23	374	SSG	27-Jun-05	month 3	Normal	
23	374	SSG	30-Sep-05	month 6	Normal	
23	375	Combination	25-Feb-05	day 0	Normal	
23	375	Combination	3-Mar-05	day 7	Normal	
23	375	Combination	10-Mar-05	day 14	Normal	
23	375	Combination	14-Mar-05	end day	Normal	
23	375	Combination	15-Jun-05	month 3	Normal	
23	375	Combination	23-Sep-05	month 6	Normal	
23	376	SSG	1-Mar-05	day 0	Normal	
23	376	SSG	7-Mar-05	day 7	Normal	
23	376	SSG	14-Mar-05	day 14	Normal	
23	376	SSG	21-Mar-05	day 21	Normal	
23	376	SSG	31-Mar-05	end day	Normal	
23	376	SSG	29-Jun-05	month 3	Normal	
23	376	SSG	28-Sep-05	month 6	Normal	
23	377	Combination	9-Mar-05	day 0	Normal	

Centre Number	Patient Number	Treatment	ECG Date	Visit	ECG Findings	Comment
23	377	Combination	15-Mar-05	day 7	Normal	
23	377	Combination	23-Sep-05	month 6	Normal	
23	378	Combination	9-Mar-05	day 0	Normal	
23	378	Combination	15-Mar-05	day 7	Normal	
23	378	Combination	22-Mar-05	day 14	Normal	
23	378	Combination	26-Mar-05	end day	Normal	
23	378	Combination	6-Jul-05	month 3	Normal	
23	378	Combination	23-Sep-05	month 6	Normal	
23	379	PM	10-Mar-05	day 0	Normal	
23	379	PM	16-Mar-05	day 7	Normal	
23	379	PM	23-Mar-05	day 14	Normal	
23	379	PM	31-Mar-05	end day	Normal	
23	379	PM	29-Jun-05	month 3	Normal	
23	380	SSG	15-Mar-05	day 0	Normal	
23	380	SSG	21-Mar-05	day 7	Normal	
23	380	SSG	28-Mar-05	day 14	Normal	
23	380	SSG	4-Apr-05	day 21	Normal	
23	380	SSG	14-Apr-05	end day	Normal	
23	380	SSG	19-Jul-05	month 3	Normal	
23	380	SSG	12-Oct-05	month 6	Normal	
23	381	PM	15-Mar-05	day 0	Normal	
23	381	PM	21-Mar-05	day 7	Normal	
23	381	PM	29-Mar-05	day 14	Normal	
23	381	PM	5-Apr-05	end day	Normal	
23	381	PM	12-Jul-05	month 3	Normal	
23	381	PM	17-Nov-05	month 6	Normal	
23	382	Combination	18-Mar-05	day 0	Normal	
23	382	Combination	24-Mar-05	day 7	Normal	
23	382	Combination	31-Mar-05	day 14	Normal	
23	382	Combination	4-Apr-05	end day	Normal	
23	382	Combination	12-Jul-05	month 3	Normal	
23	382	Combination	30-Sep-05	month 6	Normal	
23	383	PM	30-Mar-05	day 0	Normal	
23	383	PM	11-Apr-05	day 7	Normal	
23	383	PM	18-Apr-05	day 14	Normal	
23	383	PM	26-Apr-05	end day	Normal	
23	383	PM	30-Sep-05	month 3	Normal	
23	383	PM	24-Oct-05	month 6	Normal	
23	384	SSG	30-Mar-05	day 0	Normal	
23	384	SSG	11-Apr-05	day 7	Normal	
23	384	SSG	18-Apr-05	day 14	Normal	
23	384	SSG	25-Apr-05	day 21	Normal	
23	384	SSG	5-May-05	end day	Normal	
23	384	SSG	28-Sep-05	month 3	Normal	
23	384	SSG	17-Nov-05	month 6	Normal	
23	385	SSG	30-Mar-05	day 0	Normal	
23	385	SSG	13-Apr-05	day 7	Normal	
23	385	SSG	20-Apr-05	day 14	Normal	
23	385	SSG	27-Apr-05	day 21	Normal	
23	385	SSG	7-May-05	end day	Normal	
23	385	SSG	10-Aug-05	month 3	Normal	
23	385	SSG	11-Nov-05	month 6	Normal	
23	386	Combination	18-Apr-05	day 0	Normal	

Centre Number	Patient Number	Treatment	ECG Date	Visit	ECG Findings	Comment
23	386	Combination	25-Apr-05	day 7	Normal	
23	386	Combination	11-May-05	day 14	Normal	
23	386	Combination	14-May-05	end day	Normal	
23	386	Combination	19-Aug-05	month 3	Normal	
23	386	Combination	11-Nov-05	month 6	Normal	
23	387	Combination	18-Apr-05	day 0	Normal	
23	387	Combination	25-Apr-05	day 7	Normal	
23	387	Combination	2-May-05	day 14	Normal	
23	387	Combination	6-May-05	end day	Normal	
23	387	Combination	10-Aug-05	month 3	Normal	
23	387	Combination	11-Nov-05	month 6	Normal	
23	388	PM	18-Apr-05	day 0	Normal	
23	388	PM	25-Apr-05	day 7	Normal	
23	388	PM	2-May-05	day 14	Normal	
23	388	PM	10-May-05	end day	Normal	
23	388	PM	28-Sep-05	month 3	Normal	
23	388	PM	2-Dec-05	month 6	Normal	
23	389	SSG	18-Apr-05	day 0	Normal	
23	389	SSG	26-Apr-05	day 7	Normal	
23	389	SSG	3-May-05	day 14	Normal	
23	389	SSG	10-May-05	day 21	Normal	
23	389	SSG	20-May-05	end day	Normal	
23	389	SSG	5-Sep-05	month 3	Normal	
23	389	SSG	9-Dec-05	month 6	Normal	
23	390	PM	18-Apr-05	day 0	Normal	
23	390	PM	26-Apr-05	day 7	Normal	
23	390	PM	3-May-05	day 14	Normal	
23	390	PM	11-May-05	end day	Normal	
23	390	PM	10-Aug-05	month 3	Normal	
23	390	PM	2-Dec-05	month 6	Normal	
23	391	Combination	21-Apr-05	day 0	Normal	
23	391	Combination	28-Apr-05	day 7	Normal	
23	391	Combination	5-May-05	day 14	Normal	
23	391	Combination	9-May-05	end day	Normal	
23	391	Combination	19-Aug-05	month 3	Normal	
23	391	Combination	17-Nov-05	month 6	Normal	
23	392	Combination	21-Apr-05	day 0	Normal	
23	392	Combination	28-Apr-05	day 7	Normal	
23	392	Combination	5-May-05	day 14	Normal	
23	392	Combination	22-Jul-05	month 3	Normal	
23	392	Combination	1-Oct-05	month 6	Normal	
23	393	SSG	21-Apr-05	day 0	Normal	
23	393	SSG	28-Apr-05	day 7	Normal	
23	393	SSG	5-May-05	day 14	Normal	
23	393	SSG	12-May-05	day 21	Normal	
23	393	SSG	22-May-05	end day	Normal	
23	393	SSG	19-Aug-05	month 3	Normal	
23	393	SSG	17-Nov-05	month 6	Normal	
23	394	PM	18-Apr-05	day 0	Normal	
23	394	PM	2-May-05	day 7	Normal	
23	394	PM	9-May-05	day 14	Normal	
23	394	PM	17-May-05	end day	Normal	
23	394	PM	19-Aug-05	month 3	Normal	

Centre Number	Patient Number	Treatment	ECG Date	Visit	ECG Findings	Comment
23	394	PM	17-Nov-05	month 6	Normal	
23	395	PM	28-Apr-05	day 0	Normal	
23	395	PM	5-May-05	day 7	Normal	
23	395	PM	12-May-05	day 14	Normal	
23	395	PM	20-May-05	end day	Normal	
23	395	PM	14-Dec-05	month 6	Normal	
23	396	SSG	12-May-05	day 0	Normal	
23	396	SSG	19-May-05	day 7	Normal	
23	396	SSG	26-May-05	day 14	Normal	
23	396	SSG	2-Jun-05	day 21	Normal	
23	396	SSG	12-Jun-05	end day	Normal	
23	396	SSG	15-Sep-05	month 3	Normal	
23	396	SSG	14-Dec-05	month 6	Normal	
23	397	SSG	12-May-05	day 0	Normal	
23	397	SSG	19-May-05	day 7	Normal	
23	397	SSG	26-May-05	day 14	Normal	
23	397	SSG	2-Jun-05	day 21	Normal	
23	397	SSG	12-Jun-05	end day	Normal	
23	397	SSG	15-Sep-05	month 3	Normal	
23	397	SSG	14-Dec-05	month 6	Normal	
23	398	Combination	18-May-05	day 0	Normal	
23	398	Combination	24-May-05	day 7	Normal	
23	398	Combination	31-May-05	day 14	Normal	
23	398	Combination	4-Jun-05	end day	Normal	
23	398	Combination	1-Oct-05	month 3	Normal	
23	398	Combination	15-Dec-05	month 6	Normal	
23	399	PM	16-May-05	day 0	Normal	
23	399	PM	24-May-05	day 7	Normal	
23	399	PM	31-May-05	day 14	Normal	
23	399	PM	8-Jun-05	end day	Normal	
23	399	PM	14-Oct-05	month 3	Normal	
23	399	PM	14-Dec-05	month 6	Normal	
23	400	PM	3-Jun-05	day 0	Normal	
23	400	PM	9-Jun-05	day 7	Normal	
23	400	PM	16-Jun-05	day 14	Normal	
23	400	PM	24-Jun-05	end day	Normal	
23	400	PM	14-Oct-05	month 3	Normal	
23	400	PM	12-Jan-06	month 6	Normal	
23	401	Combination	2-Jun-05	day 0	Normal	
23	401	Combination	9-Jun-05	day 7	Normal	
23	401	Combination	16-Jun-05	day 14	Normal	
23	401	Combination	20-Jun-05	end day	Normal	
23	401	Combination	19-Sep-05	month 3	Normal	
23	401	Combination	20-Dec-05	month 6	Normal	
23	402	Combination	2-Jun-05	day 0	Normal	
23	402	Combination	9-Jun-05	day 7	Normal	
23	402	Combination	16-Jun-05	day 14	Normal	
23	402	Combination	20-Jun-05	end day	Normal	
23	402	Combination	23-Sep-05	month 3	Normal	
23	402	Combination	20-Dec-05	month 6	Normal	
23	403	SSG	2-Jun-05	day 0	Normal	
23	403	SSG	10-Jun-05	day 7	Normal	
23	403	SSG	17-Jun-05	day 14	Normal	

Centre Number	Patient Number	Treatment	ECG Date	Visit	ECG Findings	Comment
23	403	SSG	24-Jun-05	day 21	Normal	
23	403	SSG	4-Jul-05	end day	Normal	
23	403	SSG	30-Sep-05	month 3	Normal	
23	403	SSG	12-Jan-06	month 6	Normal	
23	404	SSG	2-Jun-05	day 0	Normal	
23	404	SSG	10-Jun-05	day 7	Normal	
23	404	SSG	17-Jun-05	day 14	Normal	
23	404	SSG	24-Jun-05	day 21	Normal	
23	404	SSG	4-Jul-05	end day	Normal	
23	404	SSG	12-Jan-06	month 6	Normal	
23	405	PM	2-Jun-05	day 0	Normal	
23	405	PM	16-Jun-05	day 7	Normal	
23	405	PM	23-Jun-05	day 14	Normal	
23	405	PM	1-Jul-05	end day	Normal	
23	405	PM	30-Sep-05	month 3	Normal	
23	405	PM	5-Jan-06	month 6	Normal	
34	451	Combination	16-Nov-04	day 0	Normal	
34	451	Combination	30-Nov-04	day 14	Normal	
34	451	Combination	4-Dec-04	end day	Normal	
34	451	Combination	3-Jun-05	month 6	Abnormal (CI)	SINUS ARRHYTHMIA
34	452	PM	16-Nov-04	day 0	Normal	
34	452	PM	30-Nov-04	day 14	Normal	
34	452	PM	8-Dec-04	end day	Normal	
34	453	PM	17-Nov-04	day 0	Normal	
34	453	PM	1-Dec-04	day 14	Normal	
34	453	PM	9-Dec-04	end day	Normal	
34	454	Combination	18-Nov-04	day 0	Normal	
34	454	Combination	4-Dec-04	day 14	Normal	
34	454	Combination	8-Dec-04	end day	Normal	
34	454	Combination	7-Jun-05	month 6	Abnormal (CI)	NEGATIVE TWAVE V1-V4 CLINICALLY WELL
34	455	PM	18-Nov-04	day 0	Normal	
34	455	PM	2-Dec-04	day 14	Normal	
34	455	PM	11-Dec-04	end day	Normal	
34	456	SSG	18-Nov-04	day 0	Normal	
34	456	SSG	2-Dec-04	day 14	Normal	
34	456	SSG	19-Dec-04	end day	Normal	
34	456	SSG	25-Jan-05	month 3	Normal	
34	457	Combination	18-Nov-04	day 0	Normal	
34	457	Combination	2-Dec-04	day 14	Normal	
34	457	Combination	6-Dec-04	end day	Normal	
34	458	PM	19-Nov-04	day 0	Normal	
34	458	PM	3-Dec-04	day 14	Normal	
34	458	PM	11-Dec-04	end day	Normal	
34	459	Combination	20-Nov-04	day 0	Normal	
34	459	Combination	3-Dec-04	day 14	Abnormal (CI)	SINUS ARRHYTHMIA
34	459	Combination	7-Dec-04	end day	Normal	
34	459	Combination	7-Jan-05	month 3	Abnormal (CI)	SINUS ARRHYTHMIA
34	459	Combination	3-Jun-05	month 6	Abnormal (CI)	SINUS ARRHYTHMIA
34	460	PM	22-Nov-04	day 0	Normal	
34	460	PM	7-Dec-04	day 14	Normal	
34	460	PM	15-Dec-04	end day	Normal	
34	461	SSG	24-Nov-04	day 0	Normal	

Centre Number	Patient Number	Treatment	ECG Date	Visit	ECG Findings	Comment
34	461	SSG	10-Dec-04	day 14	Normal	
34	462	SSG	24-Nov-04	day 0	Normal	
34	462	SSG	8-Dec-04	day 14	Normal	
34	462	SSG		end day	Normal	
34	462	SSG	7-Jun-05	month 6	Abnormal (CS)	SINUS TACHYCARDIA (BPM. 140/MIN) CLINICALLY WELL
34	463	Combination	27-Nov-04	day 0	Normal	
34	463	Combination	3-Dec-04	day 7	Abnormal (CI)	NEW SINUS ARRHYTHMIA
34	463	Combination	11-Dec-04	day 14	Abnormal (CI)	SINUS ARRHYTHMIA (SINCE DAY 7)
34	463	Combination	14-Dec-04	end day	Abnormal (CI)	SINUS ARRHYTHMIA SINCE DAY 7
34	464	SSG	27-Nov-04	day 0	Normal	
34	464	SSG	11-Dec-04	day 14	Normal	
34	464	SSG	27-Dec-04	end day	Normal	
34	464	SSG	2-Jun-05	month 6	Abnormal (CI)	RIGHT VENTRICULAR CONDUCTION DELAY
34	465	SSG	27-Nov-04	day 0	Normal	
34	465	SSG	12-Dec-04	day 14	Normal	
34	465	SSG	29-Dec-04	end day	Normal	
34	465	SSG	3-Jun-05	month 6	Abnormal (CI)	NEGATIVE TWAVE V1-V4 CLINICAL WELL
34	466	Combination	27-Nov-04	day 0	Normal	
34	466	Combination	13-Dec-04	day 14	Abnormal (CI)	NEW SINUS ARRHYTHMIA
34	466	Combination	17-Dec-04	end day	Abnormal (CI)	SINUS ARRHYTHMIA
34	467	PM	28-Nov-04	day 0	Normal	
34	467	PM	12-Dec-04	day 14	Normal	
34	467	PM	20-Dec-04	end day	Normal	
34	467	PM	5-Feb-05	month 3	Abnormal (CS)	SINUS TACHYCARDIA (139 BPM) FIRST DEGREE AV BLOCK RIGHT VENTRICULAR CONDUCTION DELAY NEG. TWAVE V1-V4 SHORT QT INTERVAL
34	468	Combination	28-Nov-04	day 0	Normal	
34	468	Combination	12-Dec-04	day 14	Normal	
34	468	Combination	16-Dec-04	end day	Normal	
34	468	Combination	4-Jun-05	month 6	Normal	
34	469	SSG	28-Nov-04	day 0	Normal	
34	469	SSG	12-Dec-04	day 14	Normal	
34	469	SSG	29-Dec-04	end day	Abnormal (CI)	SINUS ARRHYTHMIA (NEW)
34	469	SSG	10-Jun-05	month 6	Normal	
34	470	Combination	29-Nov-04	day 0	Normal	
34	470	Combination	12-Dec-04	day 14	Normal	
34	470	Combination	16-Dec-04	end day	Normal	
34	470	Combination	7-Jun-05	month 6	Normal	
34	471	PM	29-Nov-04	day 0	Normal	
34	471	PM	12-Dec-04	day 14	Normal	
34	471	PM	20-Dec-04	end day	Normal	
34	472	SSG	1-Dec-04	day 0	Normal	
34	472	SSG	14-Dec-04	day 14	Normal	
34	472	SSG	31-Dec-04	end day	Abnormal (CI)	SINUS ARRHYTHMIA
34	472	SSG	9-Jun-05	month 6	Abnormal (CI)	SINUS ARRHYTHMIA INCOMPLETE RIGHT BUNDLE BRANCH BLOCK CLINICALLY WELL
34	473	PM	1-Dec-04	day 0	Normal	
34	473	PM	16-Dec-04	day 14	Normal	

Centre Number	Patient Number	Treatment	ECG Date	Visit	ECG Findings	Comment
34	473	PM	24-Dec-04	end day	Normal	
34	474	Combination	3-Dec-04	day 0	Abnormal (CI)	ECG: FIRST DEGREE AV-BLOCK, CLINICALLY WELL
34	474	Combination	17-Dec-04	day 14	Normal	
34	474	Combination	21-Dec-04	end day	Normal	
34	474	Combination	1-Jun-05	month 6	Normal	
34	475	PM	4-Dec-04	day 0	Normal	
34	475	PM	18-Dec-04	day 14	Normal	
34	475	PM	26-Dec-04	end day	Normal	
34	476	PM	4-Dec-04	day 0	Normal	
34	476	PM	18-Dec-04	day 14	Normal	
34	476	PM	26-Dec-04	end day	Normal	
34	476	PM	10-Jan-05	month 3	Normal	
34	477	SSG	6-Dec-04	day 0	Normal	
34	477	SSG	19-Dec-04	day 14	Normal	
34	477	SSG	5-Jan-05	end day	Normal	
34	477	SSG	5-Jun-05	month 6	Abnormal (CI)	SINUS ARRHYTHMIA RIGHT VENTRICULAR CONDUCTION DELAY CLINICALLY WELL
34	478	Combination	6-Dec-04	day 0	Normal	
34	478	Combination	20-Dec-04	day 14	Normal	
34	478	Combination	24-Dec-04	end day	Abnormal (CI)	SINUS ARRHYTHMIA
34	478	Combination	5-Jun-05	month 6	Abnormal (CI)	SINUS ARRHYTHMIA RIGHT VENTRICULAR CONDUCTION DELAY CLINICALLY WELL
34	479	SSG	8-Dec-04	day 0	Normal	
34	479	SSG	21-Dec-04	day 14	Normal	
34	479	SSG	7-Jan-05	end day	Abnormal (CI)	SINUS ARRHYTHMIA
34	479	SSG	1-Jun-05	month 6	Abnormal (CI)	SINUS ARRHYTHMIA
34	480	SSG	8-Dec-04	day 0	Normal	
34	480	SSG	22-Dec-04	day 14	Normal	
34	480	SSG	8-Jan-05	end day	Abnormal (CI)	SINUS ARRHYTHMIA
34	480	SSG	13-Jun-05	month 6	Normal	
34	481	Combination	9-Dec-04	day 0	Normal	
34	481	Combination	22-Dec-04	day 14	Abnormal (CI)	SINUS ARRHYTHMIA (NEW)
34	481	Combination	26-Dec-04	end day	Abnormal (CI)	SINUS ARRHYTHMIA
34	481	Combination	3-Jun-05	month 6	Abnormal (CI)	SINUS ARRHYTHMIA
34	482	Combination	9-Dec-04	day 0	Normal	
34	482	Combination	23-Dec-04	day 14	Normal	
34	482	Combination	27-Dec-04	end day	Normal	
34	482	Combination	5-Jun-05	month 6	Abnormal (CI)	EARLY REPOLARIZATION CLINICALLY WELL
34	483	PM	9-Dec-04	day 0	Normal	
34	483	PM	23-Dec-04	day 14	Normal	
34	483	PM	31-Dec-04	end day	Normal	
34	483	PM	31-May-05	month 6	Normal	
34	484	PM	11-Dec-04	day 0	Normal	
34	485	Combination	11-Dec-04	day 0	Normal	
34	485	Combination	25-Dec-04	day 14	Normal	
34	485	Combination	29-Dec-04	end day	Normal	
34	485	Combination	6-Jun-05	month 6	Abnormal (CI)	SINUS ARRHYTHMIA RIGHT VENTRICULAR CONDUCTION DELAY NEG. T IN V1-V4 CLINICALLY WELL

Centre Number	Patient Number	Treatment	ECG Date	Visit	ECG Findings	Comment
34	486	Combination	11-Dec-04	day 0	Normal	
34	486	Combination	26-Dec-04	day 14	Normal	
34	486	Combination	30-Dec-04	end day	Normal	
34	486	Combination	6-Jun-05	month 6	Normal	
34	487	SSG	12-Dec-04	day 0	Normal	
34	487	SSG	26-Dec-04	day 14	Normal	
34	487	SSG	12-Jan-05	end day	Abnormal (CI)	NEW SINUS ARRHYTHMIA
34	487	SSG	2-Jun-05	month 6	Abnormal (CI)	RIGHT VENTRICULAR CONDUCTION DELAY
34	488	SSG	13-Dec-04	day 0	Normal	
34	488	SSG	27-Dec-04	day 14	Abnormal (CI)	SINUS ARRHYTHMIA (NEW)
34	488	SSG	13-Jan-05	end day	Abnormal (CI)	SINUS ARRHYTHMIA
34	488	SSG	17-Feb-05	month 3	Abnormal (CI)	SINUS ARRYTHMIA
34	488	SSG	7-Jun-05	month 6	Abnormal (CI)	NEGATIVE TWAVE V1-V4 CLINICALLY WELL
34	489	SSG	15-Dec-04	day 0	Normal	
34	489	SSG	29-Dec-04	day 14	Normal	
34	489	SSG	15-Jan-05	end day	Abnormal (CI)	SINUS ARRHYTHMIA (NEW)
34	489	SSG	3-Jun-05	month 6	Abnormal (CI)	SINUS ARRHYTHMIA
34	490	PM	16-Dec-04	day 0	Abnormal (CI)	ECG: FIRST DEGREE AV BLOCK, CLINICALLY WELL
34	490	PM	29-Dec-04	day 14	Abnormal (CI)	ECG: OCCASIONAL VENTRICULAR PREMATURE COMPLEXES, CLINICALLY WELL
34	490	PM	6-Jan-05	end day	Normal	ECG: OCCASIONAL PREMATUR VENTRICULAR COMPLEXES, CLINICALLY WELL
34	490	PM	15-Jun-05	month 6	Normal	
34	491	PM	16-Dec-04	day 0	Normal	ECG: OCCASIONAL VENTRICULAR PREMATURE COMPLEXES, CLINICALLY WELL
34	491	PM	30-Dec-04	day 14	Normal	
34	491	PM	7-Jan-05	end day	Normal	
34	492	PM	16-Dec-04	day 0	Normal	
34	492	PM	1-Jan-05	day 14	Normal	
34	492	PM	9-Jan-05	end day	Normal	
34	492	PM	2-Jun-05	month 6	Abnormal (CI)	RIGHT VENTRICULAR CONDUCTION DELAY
34	493	SSG	17-Dec-04	day 0	Normal	
34	493	SSG	30-Dec-04	day 14	Normal	
34	493	SSG	16-Jan-05	end day	Normal	
34	493	SSG	12-Apr-05	month 3	Abnormal (CI)	NEGATIVE TWAVE V1-V4 CLINICALLY WELL
34	494	Combination	17-Dec-04	day 0	Normal	
34	494	Combination	30-Dec-04	day 14	Normal	
34	494	Combination	3-Jan-05	end day	Normal	
34	494	Combination	3-Jun-05	month 6	Normal	
34	495	SSG	17-Dec-04	day 0	Normal	
34	495	SSG	31-Dec-04	day 14	Normal	
34	495	SSG	17-Jan-05	end day	Abnormal (CI)	SINUS ARRHYTHMIA
34	495	SSG	2-Jun-05	month 6	Abnormal (CI)	SINUS ARRHYTHMIA

Centre Number	Patient Number	Treatment	ECG Date	Visit	ECG Findings	Comment
34	496	SSG	18-Dec-04	day 0	Abnormal (CI)	ECG: RSR IN LEAD V1 V2, RIGHT VENTRICULAR CONDUCTION DELAY, CLINICALLY WELL
34	496	SSG	1-Jan-05	day 14	Normal	
34	496	SSG	18-Jan-05	end day	Abnormal (CI)	ECG: RIGHT VENTRICULAR CONDUCTION DELAY CLINICALLY WELL
34	496	SSG	2-Jun-05	month 6	Abnormal (CI)	RIGHT VENTRICULAR CONDUCTION DELAY
34	497	PM	20-Dec-04	day 0	Normal	
34	497	PM	3-Jan-05	day 14	Normal	
34	497	PM	11-Jan-05	end day	Abnormal (CI)	SINUS ARRHYTHMIA (NEW)
34	497	PM	12-Feb-05	month 3	Abnormal (CI)	RIGHT VENTRICULAR CONDUCTION DELAY
34	498	SSG	20-Dec-04	day 0	Normal	
34	498	SSG	3-Jan-05	day 14	Abnormal (CI)	1-3 PREMATURE VENTRICULAR COMPLEXES (NEW) (MINUTE)
34	498	SSG	5-Jun-05	month 6	Abnormal (CI)	RIGHT VENTRICULAR CONDUCTION DELAY
34	499	Combination	20-Dec-04	day 0	Normal	
34	499	Combination	3-Jan-05	day 14	Normal	
34	499	Combination	7-Jan-05	end day	Normal	
34	499	Combination	6-Jun-05	month 6	Normal	
34	500	PM	20-Dec-04	day 0	Normal	
34	500	PM	4-Jan-05	day 14	Normal	
34	500	PM	12-Jan-05	end day	Normal	
34	500	PM	7-Jun-05	month 6	Abnormal (CI)	SINUS ARRHYTHMIA RIGHT VENTRICULAR CONDUCTION DELAY CLINICALLY WELL
34	501	Combination	21-Dec-04	day 0	Normal	
34	501	Combination	4-Jan-05	day 14	Abnormal (CI)	NEW SINUS ARRHYTHMIA
34	501	Combination	8-Jan-05	end day	Abnormal (CI)	SINUS ARRHYTHMIA
34	501	Combination	8-Jun-05	month 6	Abnormal (CI)	SINUS ARRHYTHMIA RIGHT VENTRICULAR CONDUCTION DELAY
34	502	Combination	22-Dec-04	day 0	Normal	
34	502	Combination	5-Jan-05	day 14	Normal	
34	502	Combination	9-Jan-05	end day	Abnormal (CI)	SINUS ARRHYTHMIA
34	502	Combination	8-Jun-05	month 6	Abnormal (CI)	SINUS ARRHYTHMIA
34	503	PM	24-Dec-04	day 0	Normal	
34	503	PM	7-Jan-05	day 14	Abnormal (CI)	ECG: FIRST DEGREE AV-BLOCK CLINICALLY WELL
34	503	PM	15-Jan-05	end day	Abnormal (CI)	ECG: RIGHT BUNDLE BRANCH CONDUCTION DELAY CLINICALLY WELL
34	504	Combination	26-Dec-04	day 0	Normal	
34	504	Combination	9-Jan-05	day 14	Abnormal (CI)	SINUS ARRHYTHMIA
34	504	Combination	13-Jan-05	end day	Abnormal (CI)	SINUS ARRHYTHMIA
34	505	SSG	27-Dec-04	day 0	Normal	
34	505	SSG	10-Jan-05	day 14	Normal	
34	505	SSG	27-Jan-05	end day	Normal	
34	505	SSG	1-Jun-05	month 6	Normal	
34	506	SSG	30-Dec-04	day 0	Normal	
34	506	SSG	13-Jan-05	day 14	Normal	
34	506	SSG	30-Jan-05	end day	Abnormal (CI)	ECG: SINUS ARRHYTHMIA CLINICALLY WELL

Centre Number	Patient Number	Treatment	ECG Date	Visit	ECG Findings	Comment
34	506	SSG	10-Jun-05	month 6	Normal	
34	507	SSG	30-Dec-04	day 0	Normal	
34	507	SSG	13-Jan-05	day 14	Normal	
34	507	SSG	30-Jan-05	end day	Abnormal (CI)	ECG: SINUS ARRHYTHMIA CLINICALLY WELL
34	507	SSG	31-May-05	month 6	Abnormal (CI)	SINUS ARRHYTHMIA
34	508	PM	30-Dec-04	day 0	Normal	
34	508	PM	13-Jan-05	day 14	Normal	
34	508	PM	21-Jan-05	end day	Normal	
34	509	PM	31-Dec-04	day 0	Abnormal (CI)	SINUS TACHYCARDIA
34	509	PM	15-Jan-05	day 14	Abnormal (CI)	SINUS TACHYCARDIA REDUCING (117 BPM)
34	509	PM	23-Jan-05	end day	Normal	
34	510	Combination	31-Dec-04	day 0	Normal	
34	510	Combination	18-Jan-05	end day	Abnormal (CI)	ECG: RIGHT VENTRICULAR CONDUCTION DELAY CLINICALLY WELL
34	510	Combination	13-Jun-05	month 6	Abnormal (CI)	RIGHT VENTRICULAR CONDUCTION DELAY
34	511	Combination	1-Jan-05	day 0	Normal	
34	511	Combination	14-Jan-05	day 14	Abnormal (CI)	ECG: OCCASIONAL PREMATURE VENTRICULAR COMPLEXES, CLINICALLY WELL
34	511	Combination	18-Jan-05	end day	Normal	
34	511	Combination	10-Jun-05	month 6	Abnormal (CI)	SINUS ARRHYTHMIA
34	512	PM	1-Jan-05	day 0	Normal	
34	512	PM	15-Jan-05	day 14	Abnormal (CS)	SINUS TACHYCARDIA (FREQUENCE 145)
34	512	PM	23-Jan-05	end day	Normal	
34	513	Combination	2-Jan-05	day 0	Normal	
34	513	Combination	16-Jan-05	day 14	Normal	
34	513	Combination	20-Jan-05	end day	Normal	
34	513	Combination	28-Feb-05	month 3	Normal	
34	513	Combination	31-May-05	month 6	Normal	
34	514	SSG	3-Jan-05	day 0	Normal	
34	514	SSG	17-Jan-05	day 14	Normal	
34	514	SSG	3-Feb-05	end day	Abnormal (CI)	ECG: SINUS ARRHYTHMIA, RIGHT VENTRICULAR CONDUCTION DELAY CLINICALLY WELL
34	514	SSG	7-Jun-05	month 6	Abnormal (CI)	RIGHT VENTRICULAR CONDUCTION DELAY SINUS ARRHYTHMIA CLINICALLY WELL
34	515	SSG	5-Jan-05	day 0	Normal	
34	515	SSG	18-Jan-05	day 14	Normal	
34	515	SSG		end day	Abnormal (CI)	ECG: SINUS ARRHYTHMIA CLINICALLY WELL
34	515	SSG	14-May-05	month 3	Abnormal (CI)	SINUS ARRHYTHMIA
34	516	PM	6-Jan-05	day 0	Abnormal (CI)	SINUS ARRHYTHMIA
34	516	PM	19-Jan-05	day 14	Normal	
34	516	PM	27-Jan-05	end day	Normal	
34	516	PM	6-Jun-05	month 6	Abnormal (CI)	SINUS ARRHYTHMIA
34	517	PM	6-Jan-05	day 0	Normal	
34	517	PM	19-Jan-05	day 14	Normal	

Centre Number	Patient Number	Treatment	ECG Date	Visit	ECG Findings	Comment
34	517	PM	27-Jan-05	end day	Abnormal (CI)	ECG: SINUS ARRHYTHMIA CLINICALLY WELL
34	518	SSG	6-Jan-05	day 0	Normal	
34	518	SSG	21-Jan-05	day 14	Normal	
34	518	SSG	8-Feb-05	end day	Abnormal (CI)	ECG: SINUS ARRHYTHMIA
34	518	SSG	4-Jun-05	month 6	Abnormal (CI)	SINUS ARRHYTHMIA RIGHT VENTRICULAR CONDUCTION DELAY
34	519	PM	6-Jan-05	day 0	Normal	
34	519	PM	20-Jan-05	day 14	Normal	
34	519	PM	28-Jan-05	end day	Normal	
34	519	PM	3-Apr-05	month 3	Normal	
34	520	SSG	10-Jan-05	day 0	Normal	
34	520	SSG	24-Jan-05	day 14	Normal	
34	520	SSG	10-Feb-05	end day	Abnormal (CI)	ECG: SINUS ARRHYTHMIA CLINICALLY WELL
34	520	SSG	5-Jun-05	month 6	Abnormal (CI)	SINUS ARRHYTHMIA
34	521	PM	10-Jan-05	day 0	Normal	
34	521	PM	24-Jan-05	day 14	Normal	
34	521	PM	1-Feb-05	end day	Normal	
34	522	Combination	11-Jan-05	day 0	Abnormal (CI)	ECG: LOW QRS VOLTAGE IN LIMB LEADS CLINICALLY WELL
34	522	Combination	24-Jan-05	day 14	Abnormal (CI)	ECG: LOW QRS VOLTAGE IN LIMB LEADS, PR INTERVAL DROPPED CLINICALLY WELL
34	522	Combination	28-Jan-05	end day	Abnormal (CI)	ECG: PR INTERVAL DROPPED, LOW QRS VOLTAGE IN LIMB LEADS CLINICALLY WELL
34	523	SSG	12-Jan-05	day 0	Normal	
34	523	SSG	27-Jan-05	day 14	Abnormal (CI)	ECG: FIRST DEGREE AV BLOCK CLINICALLY WELL
34	523	SSG	13-Feb-05	end day	Normal	
34	523	SSG	1-Jun-05	month 6	Normal	
34	524	Combination	12-Jan-05	day 0	Normal	
34	524	Combination	26-Jan-05	day 14	Normal	
34	524	Combination	30-Jan-05	end day	Abnormal (CI)	ECG: SINUS ARRHYTHMIA CLINICALLY WELL
34	524	Combination	5-Jun-05	month 6	Normal	
34	525	Combination	12-Jan-05	day 0	Normal	
34	525	Combination	26-Jan-05	day 14	Normal	
34	525	Combination	30-Jan-05	end day	Abnormal (CI)	ECG: SINUS ARRHYTHMIA CLINICALLY WELL
34	525	Combination	9-Jun-05	month 6	Normal	
34	526	SSG	13-Jan-05	day 0	Normal	
34	526	SSG	27-Jan-05	day 14	Abnormal (CI)	ECG: RIGHT VENTRICULAR CONDUCTION DELAY CLINICALLY WELL
34	526	SSG	13-Feb-05	end day	Abnormal (CI)	ECG: SINUS ARRHYTHMIA, RIGHT VENTRICULAR CONDUCTION DELAY CLINICALLY WELL
34	526	SSG	4-Jun-05	month 6	Abnormal (CI)	RIGHT VENTRICULAR CONDUCTION DELAY CLINICALLY WELL

Centre Number	Patient Number	Treatment	ECG Date	Visit	ECG Findings	Comment
34	527	Combination	14-Jan-05	day 0	Abnormal (CS)	ECG: REENTRY TACHYCARDIA (FREQUENCE: 150BPM)DD:SINUS TACHYCARDIA POSSIBLE NEGATIVE T WAVE IN III, AV+-, V4-V6 CONSISTENT WITH LATERAL/INFERIOR ISCHEMIA CLINICALLY WELL
34	527	Combination	28-Jan-05	day 14	Abnormal (CI)	ECG: RIGHT VENTRICULAR CONDUCTION DELAY, POSSIBLE SINUS RHYTHMIA FREQUENCE - 114BPM CLINICALLY WELL
34	527	Combination	1-Feb-05	end day	Abnormal (CS)	ECG: RIGHT VENTRICULAR CONDUCTION DELAY POSSIBLE SINUS TACHYCARDIA (F:120BPM)
34	527	Combination	31-May-05	month 6	Abnormal (CI)	RIGHT VENTRICULAR CONDUCTION DELAY
34	528	PM	16-Jan-05	day 0	Normal	
34	528	PM	30-Jan-05	day 14	Normal	
34	528	PM	7-Feb-05	end day	Abnormal (CI)	ECG: SINUS ARRHYTHMIA, RIGHT VENTRICULAR CONDUCTION DELAY POSSIBLE CLINICALLY WELL
34	529	Combination	17-Jan-05	day 0	Normal	
34	529	Combination	31-Jan-05	day 14	Abnormal (CI)	ECG: SINUS ARRHYTHMIA, RIGHT VENTRICULAR CONDUCTION DELAY CLINICALLY WELL
34	529	Combination	4-Feb-05	end day	Abnormal (CI)	ECG: SINUS ARRHYTHMIA, RIGHT VENTRICULAR CONDUCTION DELAY CLINICALLY WELL
34	529	Combination	14-Jun-05	month 6	Abnormal (CI)	SINUS ARRHYTHMIA INCOMPLETE RIGHT BUNDLE BRANCH BLOCK
34	530	Combination	19-Jan-05	day 0	Normal	
34	530	Combination	2-Feb-05	day 14	Normal	
34	530	Combination	6-Feb-05	end day	Normal	
34	530	Combination	14-Jun-05	month 6	Abnormal (CI)	SINUS ARRHYTHMIA
34	531	SSG	20-Jan-05	day 0	Normal	
34	531	SSG	3-Feb-05	day 14	Normal	
34	531	SSG	20-Feb-05	end day	Normal	
34	532	Combination	20-Jan-05	day 0	Normal	
34	532	Combination	3-Feb-05	day 14	Normal	
34	532	Combination	7-Feb-05	end day	Normal	
34	532	Combination	3-Jun-05	month 6	Normal	
34	533	SSG	22-Jan-05	day 0	Normal	
34	533	SSG	5-Feb-05	day 14	Normal	
34	533	SSG	22-Feb-05	end day	Normal	
34	533	SSG	9-Jun-05	month 6	Abnormal (CI)	SINUS ARRHYTHMIA
34	534	PM	23-Jan-05	day 0	Normal	
34	534	PM	6-Feb-05	day 14	Normal	
34	534	PM	14-Feb-05	end day	Abnormal (CS)	ECG: SINUS TACHYCARDIA (BPM:150/MIN)
34	535	PM	23-Jan-05	day 0	Normal	
34	535	PM	6-Feb-05	day 14	Normal	
34	535	PM	14-Feb-05	end day	Normal	
34	535	PM	25-Mar-05	month 3	Abnormal (CS)	SINUS TACHYCARDIA (FREQUENCE 146 BPM)

Centre Number	Patient Number	Treatment	ECG Date	Visit	ECG Findings	Comment
34	536	PM	24-Jan-05	day 0	Abnormal (CI)	ECG: INCOMPLETE RIGHT BUNDLE BRANCH BLOCK CLINICALLY WELL
34	536	PM	7-Feb-04	day 14	Abnormal (CI)	ECG: RIGHT VENTRICULAR CONDUCTION DELAY, SINUS ARRHYTHMIA CLINICALLY WELL
34	536	PM	15-Feb-05	end day	Abnormal (CI)	ECG: RIGHT VENTRICULAR CONDUCTION DELAY CLINICALLY WELL
34	536	PM	6-Jun-05	month 6	Abnormal (CI)	INCOMPLETE RIGHT BUNDLE BRANCH BLOCK POSSIBLE CLINICALLY WELL
34	537	SSG	25-Jan-05	day 0	Abnormal (CI)	ECG: RIGHT VENTRICULAR CONDUCTION DELAY, CLINICALLY WELL
34	537	SSG	7-Feb-05	day 14	Abnormal (CS)	ECG: SINUS TACHYCARDIA (F-150BPM) CLINICALLY WELL
34	537	SSG	24-Feb-05	end day	Normal	
34	537	SSG	31-May-05	month 6	Abnormal (CI)	#NAME?
34	538	PM	24-Jan-05	day 0	Normal	
34	538	PM	8-Feb-05	day 14	Normal	
34	538	PM	16-Feb-05	end day	Normal	
34	539	Combination	25-Jan-05	day 0	Abnormal (CI)	SINUS ARRHYTHMIA
34	539	Combination	8-Feb-05	day 14	Normal	
34	539	Combination	12-Feb-05	end day	Normal	
34	539	Combination	5-Jun-05	month 6	Normal	
34	540	SSG	26-Jan-05	day 0	Normal	
34	540	SSG	9-Feb-05	day 14	Normal	
34	540	SSG	26-Feb-05	end day	Normal	
34	540	SSG	31-May-05	month 6	Normal	
35	646	PM	28-Jan-05	day 0	Normal	
35	646	PM	3-Feb-05	day 7	Normal	
35	646	PM	10-Feb-05	day 14	Normal	
35	646	PM	18-Feb-05	end day	Normal	
35	647	PM	29-Jan-05	day 0	Normal	
35	647	PM	5-Feb-05	day 7	Normal	
35	647	PM	12-Feb-05	day 14	Normal	
35	647	PM	20-Feb-05	end day	Normal	
35	648	Combination	29-Jan-05	day 0	Normal	
35	648	Combination	5-Feb-05	day 7	Normal	
35	648	Combination		day 14	Not Done	
35	648	Combination	16-Feb-05	end day	Normal	
35	648	Combination	25-Aug-05	month 6	Normal	
35	649	Combination	29-Jan-05	day 0	Normal	
35	649	Combination	5-Feb-05	day 7	Normal	
35	649	Combination		day 14	Not Done	
35	649	Combination	16-Feb-05	end day	Normal	
35	649	Combination	15-Sep-05	month 6	Normal	
35	650	SSG	29-Jan-05	day 0	Normal	
35	650	SSG	5-Feb-05	day 7	Normal	
35	650	SSG	12-Feb-05	day 14	Normal	
35	650	SSG	19-Feb-05	day 21	Normal	
35	650	SSG	1-Mar-05	end day	Normal	
35	650	SSG	15-Sep-05	month 6	Normal	
35	651	SSG	29-Jan-05	day 0	Normal	
35	651	SSG	5-Feb-05	day 7	Normal	
35	651	SSG	12-Feb-05	day 14	Normal	

Centre Number	Patient Number	Treatment	ECG Date	Visit	ECG Findings	Comment
35	651	SSG	19-Feb-05	day 21	Normal	
35	651	SSG	1-Mar-05	end day	Normal	
35	651	SSG	15-Sep-05	month 6	Normal	
35	652	PM	31-Jan-05	day 0	Normal	
35	652	PM	7-Feb-05	day 7	Normal	
35	652	PM	14-Feb-05	day 14	Normal	
35	652	PM	22-Feb-05	end day	Normal	
35	653	Combination	1-Feb-05	day 0	Normal	
35	653	Combination	9-Feb-05	day 7	Normal	
35	653	Combination	16-Feb-05	day 14	Normal	
35	653	Combination	20-Feb-05	end day	Normal	
35	653	Combination	25-Aug-05	month 6	Normal	
35	654	SSG	1-Feb-05	day 0	Normal	
35	654	SSG	9-Feb-05	day 7	Normal	
35	654	SSG	16-Feb-05	day 14	Normal	
35	654	SSG	23-Feb-05	day 21	Normal	
35	654	SSG	5-Mar-05	end day	Normal	
35	654	SSG	19-Sep-05	month 6	Normal	
35	655	Combination	1-Feb-05	day 0	Normal	
35	655	Combination	9-Feb-05	day 7	Normal	
35	655	Combination	16-Feb-05	day 14	Normal	
35	655	Combination	20-Feb-05	end day	Normal	
35	655	Combination	25-Aug-05	month 6	Normal	
35	656	SSG	3-Feb-05	day 0	Normal	
35	656	SSG	9-Feb-05	day 7	Normal	
35	656	SSG	16-Feb-05	day 14	Normal	
35	656	SSG	23-Feb-05	day 21	Normal	
35	656	SSG	5-Mar-05	end day	Normal	
35	656	SSG	17-Oct-05	month 6	Normal	
35	657	PM	7-Feb-05	day 0	Normal	
35	657	PM	14-Feb-05	day 7	Normal	
35	657	PM	21-Feb-05	day 14	Normal	
35	657	PM	1-Mar-05	end day	Normal	
35	657	PM	15-Sep-05	month 6	Normal	
35	658	Combination	6-Feb-05	day 0	Normal	
35	658	Combination	14-Feb-05	day 7	Normal	
35	658	Combination	21-Feb-05	day 14	Normal	
35	658	Combination	25-Feb-05	end day	Normal	
35	658	Combination	25-Aug-05	month 6	Normal	
35	659	SSG	7-Feb-05	day 0	Normal	
35	659	SSG	14-Feb-05	day 7	Normal	
35	659	SSG	21-Feb-05	day 14	Normal	
35	659	SSG	28-Feb-05	day 21	Normal	
35	659	SSG	10-Mar-05	end day	Normal	
35	659	SSG	29-Oct-05	month 6	Normal	
35	660	PM	10-Feb-05	day 0	Normal	
35	660	PM	16-Feb-05	day 7	Normal	
35	660	PM	23-Feb-05	day 14	Normal	
35	660	PM	3-Mar-05	end day	Normal	
35	660	PM	15-Sep-05	month 6	Normal	
35	661	Combination	16-Feb-05	day 0	Normal	LOW VOLTAGE ECG IN THE LIMB LEADS
35	661	Combination	22-Feb-05	day 7	Normal	LOW VOLTAGE QRS IN THE LIMP LEADS
35	661	Combination	1-Mar-05	day 14	Normal	

Centre Number	Patient Number	Treatment	ECG Date	Visit	ECG Findings	Comment
35	661	Combination	5-Mar-05	end day	Normal	
35	661	Combination	24-Sep-05	month 6	Normal	
35	662	PM	19-Feb-05	day 0	Normal	
35	662	PM	26-Feb-05	day 7	Normal	
35	662	PM	5-Mar-05	day 14	Normal	
35	662	PM	13-Mar-05	end day	Normal	
35	663	SSG	19-Feb-05	day 0	Normal	
35	663	SSG	26-Feb-05	day 7	Abnormal (CS)	QT LONG THE PATIENT WAS WELL
35	663	SSG	5-Mar-05	day 14	Normal	
35	663	SSG	12-Mar-05	day 21	Abnormal (CS)	PROLONGED QT INTERVAL. THE WAS WELL
35	663	SSG	22-Mar-05	end day	Normal	
35	663	SSG	22-Sep-05	month 6	Normal	
35	664	Combination	19-Feb-05	day 0	Normal	
35	664	Combination	26-Feb-05	day 7	Abnormal (CS)	QT PROLONGATION
35	665	SSG	22-Feb-05	day 0	Normal	
35	665	SSG	1-Mar-05	day 7	Normal	
35	665	SSG	8-Mar-05	day 14	Normal	
35	665	SSG	15-Mar-05	day 21	Normal	
35	665	SSG	25-Mar-05	end day	Normal	
35	665	SSG	23-Oct-05	month 6	Normal	
35	666	SSG	25-Feb-05	day 0	Normal	
35	666	SSG	4-Mar-05	day 7	Normal	
35	666	SSG	11-Mar-05	day 14	Normal	
35	666	SSG	18-Mar-05	day 21	Normal	
35	666	SSG	28-Mar-05	end day	Normal	
35	666	SSG	25-Sep-05	month 6	Normal	
35	667	Combination	28-Feb-05	day 0	Normal	
35	667	Combination	6-Mar-05	day 7	Normal	
35	667	Combination	13-Mar-05	day 14	Normal	
35	667	Combination	17-Mar-05	end day	Normal	
35	667	Combination	27-Oct-05	month 6	Normal	
35	668	PM	28-Feb-05	day 0	Normal	
35	668	PM	6-Mar-05	day 7	Normal	
35	668	PM	13-Mar-05	day 14	Normal	
35	668	PM	21-Mar-05	end day	Normal	
35	668	PM	25-Sep-05	month 6	Normal	
35	669	PM	28-Feb-05	day 0	Normal	
35	669	PM	6-Mar-05	day 7	Normal	
35	669	PM	13-Mar-05	day 14	Normal	
35	669	PM	21-Mar-05	end day	Normal	
35	670	SSG	3-Mar-05	day 0	Normal	
35	670	SSG	9-Mar-05	day 7	Normal	
35	670	SSG	16-Mar-05	day 14	Normal	
35	670	SSG	23-Mar-05	day 21	Normal	
35	670	SSG	2-Apr-05	end day	Normal	
35	670	SSG	17-Oct-05	month 6	Normal	
35	671	PM	10-Mar-05	day 0	Normal	
35	671	PM	17-Mar-05	day 7	Normal	
35	671	PM	24-Mar-05	day 14	Normal	
35	671	PM	1-Apr-05	end day	Normal	

Centre Number	Patient Number	Treatment	ECG Date	Visit	ECG Findings	Comment
35	672	Combination	10-Mar-05	day 0	Normal	
35	672	Combination	17-Mar-05	day 7	Normal	
35	672	Combination	24-Mar-05	day 14	Normal	
35	672	Combination	28-Mar-05	end day	Normal	
35	672	Combination	29-Oct-05	month 6	Normal	
35	673	SSG	10-Mar-05	day 0	Normal	
35	673	SSG	17-Mar-05	day 7	Normal	
35	673	SSG	24-Mar-05	day 14	Normal	
35	673	SSG	31-Mar-05	day 21	Normal	
35	673	SSG	10-Apr-05	end day	Normal	
35	673	SSG	17-Oct-05	month 6	Normal	
35	674	Combination	11-Mar-05	day 0	Normal	
35	674	Combination	19-Mar-05	day 7	Normal	
35	674	Combination	26-Mar-05	day 14	Normal	
35	674	Combination	30-Mar-05	end day	Normal	
35	674	Combination	25-Oct-05	month 6	Normal	
35	675	PM	13-Mar-05	day 0	Normal	
35	675	PM	20-Mar-05	day 7	Normal	
35	675	PM	27-Mar-05	day 14	Normal	
35	675	PM	4-Apr-05	end day	Normal	
35	676	PM	19-Mar-05	day 0	Normal	
35	676	PM	26-Mar-05	day 7	Normal	
35	676	PM	2-Apr-05	day 14	Normal	
35	676	PM	10-Apr-05	end day	Normal	
35	676	PM	25-Oct-05	month 6	Normal	
35	677	SSG	19-Mar-05	day 0	Normal	
35	677	SSG	26-Mar-05	day 7	Normal	
35	678	Combination	20-Mar-05	day 0	Normal	
35	678	Combination	28-Mar-05	day 7	Normal	
35	678	Combination	4-Apr-05	day 14	Normal	
35	678	Combination	8-Apr-05	end day	Normal	
35	678	Combination	25-Oct-05	month 6	Normal	
35	679	SSG	20-Mar-05	day 0	Normal	
35	679	SSG	28-Mar-05	day 7	Normal	
35	679	SSG	4-Apr-05	day 14	Normal	
35	679	SSG	11-Apr-05	day 21	Normal	
35	679	SSG	20-Apr-05	end day	Normal	
35	679	SSG	25-Oct-05	month 6	Normal	
35	680	Combination	21-Mar-05	day 0	Normal	
35	680	Combination	28-Mar-05	day 7	Normal	
35	680	Combination	4-Apr-05	day 14	Normal	
35	680	Combination	8-Apr-05	end day	Normal	
35	680	Combination	17-Oct-05	month 6	Normal	
35	681	Combination	21-Mar-05	day 0	Normal	
35	681	Combination	28-Mar-05	day 7	Normal	
35	681	Combination	4-Apr-05	day 14	Normal	
35	681	Combination	8-Apr-05	end day	Normal	
35	681	Combination	23-Oct-05	month 6	Normal	
35	682	PM	21-Mar-05	day 0	Normal	
35	682	PM	28-Mar-05	day 7	Normal	FIRST DEGRE AV BLOCK
35	682	PM	4-Apr-05	day 14	Normal	
35	682	PM	12-Apr-05	end day	Normal	FIRST DEGREE AV BLOCK
35	683	Combination	21-Mar-05	day 0	Normal	

Centre Number	Patient Number	Treatment	ECG Date	Visit	ECG Findings	Comment
35	683	Combination	28-Mar-05	day 7	Normal	
35	683	Combination	4-Apr-05	day 14	Normal	
35	683	Combination	8-Apr-05	end day	Normal	
35	683	Combination	29-Oct-05	month 6	Normal	
35	684	SSG	8-Apr-05	day 0	Normal	
35	684	SSG	15-Apr-05	day 7	Normal	
35	684	SSG	22-Apr-05	day 14	Normal	
35	684	SSG	29-Apr-05	day 21	Normal	
35	684	SSG	9-May-05	end day	Normal	
35	684	SSG	13-Nov-05	month 6	Normal	
35	685	PM	8-Apr-05	day 0	Normal	
35	685	PM	15-Apr-05	day 7	Normal	
35	685	PM	22-Apr-05	day 14	Normal	
35	685	PM	30-Apr-05	end day	Normal	
35	685	PM	29-Oct-05	month 6	Normal	
35	686	PM	13-Apr-05	day 0	Normal	
35	686	PM	19-Apr-05	day 7	Normal	
35	686	PM	26-Apr-05	day 14	Normal	
35	686	PM	4-May-05	end day	Normal	
35	687	SSG	13-Apr-05	day 0	Normal	
35	687	SSG	19-Apr-05	day 7	Normal	
35	687	SSG	26-Apr-05	day 14	Normal	
35	687	SSG	3-May-05	day 21	Normal	
35	687	SSG	13-May-05	end day	Normal	
35	687	SSG	29-Oct-05	month 6	Normal	
35	688	PM	15-Apr-05	day 0	Normal	
35	688	PM	22-Apr-05	day 7	Normal	
35	688	PM	29-Apr-05	day 14	Normal	
35	688	PM	7-May-05	end day	Normal	
35	688	PM	25-Oct-05	month 6	Normal	
35	689	Combination	16-Apr-05	day 0	Normal	
35	689	Combination	22-Apr-05	day 7	Normal	
35	689	Combination	29-Apr-05	day 14	Normal	
35	689	Combination	3-May-05	end day	Normal	
35	690	SSG	16-Apr-05	day 0	Normal	
35	690	SSG	22-Apr-05	day 7	Normal	
35	690	SSG	29-Apr-05	day 14	Normal	
35	690	SSG	6-May-05	day 21	Normal	
35	690	SSG	16-May-05	end day	Normal	
35	690	SSG	29-Oct-05	month 6	Normal	

Centre Number	Patient Number	Treatment	Chest X-ray Date	Chest X-ray Findings	Comment
11	1	PM	17-Jun-05	Normal	
11	3	Combination	17-Jun-05	Normal	
11	6	Combination	27-Jun-05	Normal	
11	11	Combination	4-Jul-05	Normal	
11	14	SSG	8-Jul-05	Normal	
11	16	Combination	12-Jul-05	Normal	
11	17	Combination	14-Jul-05	Normal	
11	31	SSG	21-Sep-05	Normal	
11	57	Combination	29-May-06	Normal	
11	83	SSG	15-Aug-06	Normal	
11	93	SSG	4-Jan-07	Normal	
11	105	SSG	16-Feb-07	Normal	
12	241	Combination	7-Jun-05	Normal	
12	242	SSG	7-Jun-05	Normal	
12	243	PM	7-Jun-05	Normal	
12	244	SSG	7-Jun-05	Normal	
12	245	PM	18-Jul-05	Normal	
12	246	PM	2-Aug-05	Normal	
12	247	PM	12-Sep-05	Normal	
12	248	PM	23-Sep-05	Normal	
12	249	Combination	24-Oct-05	Normal	
12	250	SSG	24-Oct-05	Normal	
12	251	Combination	27-Oct-05	Normal	
12	252	SSG	27-Oct-05	Normal	
12	253	SSG	27-Oct-05	Normal	
12	254	Combination	27-Oct-05	Normal	
12	255	Combination	29-Oct-05	Normal	
12	256	Combination	14-Nov-05	Normal	
12	257	SSG	14-Nov-05	Normal	
12	258	SSG	30-Nov-05	Normal	
12	259	PM	26-Dec-05	Normal	
12	260	PM	26-Dec-05	Normal	
12	261	Combination	26-Dec-05	Normal	
12	262	PM	26-Dec-05	Normal	
12	263	SSG	20-Jan-06	Normal	
12	264	Combination	14-Feb-06	Normal	
12	265	SSG	8-Feb-06	Normal	
12	266	PM	15-Feb-06	Normal	
12	267	SSG	11-Feb-06	Normal	
12	268	PM	6-Mar-06	Normal	
12	269	Combination	13-Mar-06	Normal	
12	270	Combination	13-Mar-06	Normal	
12	271	SSG	27-Mar-06	Normal	
12	272	PM	4-Apr-06	Normal	
12	273	SSG	4-Apr-06	Normal	
12	274	SSG	4-Apr-06	Normal	
12	275	Combination	4-Apr-06	Normal	
12	276	PM	4-Apr-06	Normal	THERE IS CARDIOMEGALLY BUT NO ACTIVE PARACHYMAL LESION ON THE LUNG
12	277	Combination	18-Apr-06	Normal	
12	278	SSG	17-Apr-06	Normal	
12	279	PM	18-Apr-06	Normal	
12	280	SSG	18-Apr-06	Normal	
12	281	Combination	18-Apr-06	Normal	

Centre Number	Patient Number	Treatment	Chest X-ray Date	Chest X-ray Findings	Comment
12	282	PM	27-Apr-06	Normal	
12	283	PM	10-May-06	Normal	
12	284	Combination	18-Jun-06	Normal	
12	285	Combination	18-May-06	Normal	
12	286	PM	22-Dec-06	Normal	
12	287	SSG	21-Dec-06	Normal	
12	288	Combination	26-Dec-06	Normal	
12	289	Combination	26-Dec-06	Normal	
12	290	SSG	4-Jan-07	Normal	
12	291	SSG	8-Jan-07	Normal	
12	292	PM	15-Jan-07	Normal	
12	293	SSG	21-Jan-07	Normal	
12	294	Combination	19-Feb-07	Normal	CARDIOMEGALY
12	295	Combination	11-Mar-07	Normal	
12	303	PM	4-Jun-07	Normal	
12	304	Combination	5-Jun-07	Normal	
12	309	PM	18-Jun-07	Normal	
12	314	SSG	30-Aug-07	Normal	
12	321	Combination	4-Sep-07	Normal	
23	361	Combination	18-Jan-05	Normal	
23	362	PM	18-Jan-05	Normal	
23	363	PM	18-Jan-05	Normal	
23	364	Combination	18-Jan-05	Normal	
23	365	SSG	18-Jan-05	Normal	
23	366	Combination	28-Feb-05	Normal	
23	367	SSG	28-Jan-05	Normal	
23	368	PM	28-Jan-05	Normal	
23	369	Combination	28-Jan-05	Abnormal	BASAL BRONCHO PNEUMONIA
23	370	SSG	28-Jan-05	Abnormal	BILATERAL BASAL BRONCHO PNEUMONIA
23	371	PM	4-Feb-05	Normal	
23	372	PM	10-Feb-05	Normal	
23	373	SSG	17-Feb-05	Normal	
23	374	SSG	18-Feb-05	Normal	
23	375	Combination	24-Feb-05	Normal	
23	376	SSG	24-Feb-05	Normal	
23	377	Combination	8-Mar-05	Normal	
23	378	Combination	8-Mar-05	Normal	
23	379	PM	24-Feb-05	Abnormal	FEATURES SUGGESTIVE OF PULMONARY TUBERCULOSIS (PATIENT TREATED AS A CASE OF PNEUMONIA)
23	380	SSG	8-Mar-05	Normal	
23	381	PM	8-Mar-05	Normal	
23	382	Combination	18-Mar-05	Normal	
23	383	PM	29-Mar-05	Abnormal	PERI-HILAR BRONCHOVASCULAR MARKINGS ARE PROMINENT INDICATING AN INFLAMMATORY PROCESS.
23	384	SSG	29-Mar-05	Abnormal	BILATERAL BRONCHO-PNEUMONIA
23	385	SSG	1-Apr-05	Abnormal	BILATERAL PEVICARDIAC OPACIFICATION. PATIENT TREATED AS A CASE OF PNEUMONIA
23	386	Combination	14-Apr-05	Normal	
23	387	Combination	14-Apr-05	Normal	
23	388	PM	15-Apr-05	Normal	
23	389	SSG	18-Apr-05	Normal	
23	390	PM	15-Apr-05	Normal	
23	391	Combination	20-Apr-05	Normal	
23	392	Combination	21-Apr-05	Normal	

Centre Number	Patient Number	Treatment	Chest X-ray Date	Chest X-ray Findings	Comment
23	393	SSG	21-Apr-05	Normal	
23	394	PM	18-Apr-05	Abnormal	BOTH PRERIHILAN AND RIGHT LOWER ZONE OPACIFIED BUT CLEAR COSTOPHENIC ANGLES TAKEN TO BE PNEUMONIA.
23	395	PM	23-Apr-05	Normal	
23	396	SSG	12-May-05	Normal	
23	397	SSG	12-May-05	Abnormal	BASAL PNEUMOTITIS WAS REPORTED
23	398	Combination	16-May-05	Normal	
23	399	PM	18-May-05	Normal	
23	400	PM	18-May-05	Abnormal	INCREASED LUNG MARKINGS OVER THE RIGHT LUNG BASE. FEATURES CONSISTENT WITH RIGHT BASAL PNEUMONIA
23	401	Combination	30-May-05	Normal	
23	402	Combination	30-May-05	Normal	
23	403	SSG	2-Jun-05	Normal	
23	404	SSG	1-Jun-05	Normal	
23	405	PM	30-May-05	Abnormal	ATYPICAL PNEUMONIA
35	660	PM	8-Feb-05	Normal	
35	686	PM	11-Apr-05	Normal	

Centre Number	Patient Number	Treatment	Audiometry Date	Visit	Audiometry Findings	Comment
11	1	PM	17-Jun-05	day 0	Normal	
11	1	PM	24-Jun-05	day 7	Normal	
11	1	PM	1-Jul-05	day 14	Normal	
11	1	PM	9-Jul-05	end day	Normal	
11	1	PM	6-Oct-05	month 3	Normal	
11	1	PM	5-Jan-06	month 6	Normal	
11	2	Combination	17-Jun-05	day 0	Normal	
11	2	Combination	24-Jun-05	day 7	Normal	
11	2	Combination	1-Jul-05	day 14	Normal	
11	2	Combination	5-Jul-05	end day	Normal	
11	2	Combination	30-Sep-05	month 3	Normal	
11	2	Combination	6-Jan-06	month 6	Normal	
11	3	Combination	18-Jun-05	day 0	Normal	
11	3	Combination	25-Jun-05	day 7	Normal	
11	3	Combination	2-Jul-05	day 14	Normal	
11	3	Combination	6-Jul-05	end day	Normal	
11	3	Combination	1-Oct-05	month 3	Normal	
11	3	Combination	29-Dec-05	month 6	Normal	
11	4	PM	27-Jun-05	day 0	Normal	
11	4	PM	6-Jul-05	day 7	Normal	
11	4	PM	13-Jul-05	day 14	Normal	
11	4	PM	21-Jul-05	end day	Normal	
11	4	PM	31-Dec-05	month 3	Normal	
11	4	PM	28-Jan-06	month 6	Normal	
11	5	PM	28-Jun-05	day 0	Normal	
11	5	PM	6-Jul-05	day 7	Normal	
11	5	PM	13-Jul-05	day 14	Normal	
11	5	PM	21-Jul-05	end day	Abnormal (CI)	MILD HEARING LOSS AT 250HZ & 500HZ ON THE RIGHT EAR
11	5	PM	18-Oct-05	month 3	Normal	
11	5	PM	14-Feb-06	month 6	Normal	
11	6	Combination	29-Jun-05	day 0	Normal	
11	6	Combination	8-Jul-05	day 7	Normal	
11	6	Combination	15-Jul-05	day 14	Normal	
11	6	Combination	19-Jul-05	end day	Normal	
11	6	Combination	17-Oct-05	month 3	Normal	
11	6	Combination	10-Jan-06	month 6	Normal	
11	7	PM	30-Jun-05	day 0	Normal	
11	7	PM	8-Jul-05	day 7	Abnormal (CI)	MILD HEARING LOSS RT EAR - AT 500HZ - 30DBL 8000HZ - 40DBL LT EAR - AT 500HZ - 30DBC 8000HZ - 35DBL
11	7	PM	15-Jul-05	day 14	Normal	
11	7	PM	23-Jul-05	end day	Abnormal (CI)	MODERATE DEGREE OF HEARING LOSS AT 8000HZ
11	7	PM	21-Oct-05	month 3	Normal	
11	7	PM	24-Jan-06	month 6	Normal	
11	8	SSG	30-Jun-05	day 0	Normal	
11	8	SSG	8-Jul-05	day 7	Abnormal (CI)	MILD HEARING LOSS LEFT EAR AT 250HZ - 30DB 500HZ - 35DB 1000HZ - 30DB
11	8	SSG	15-Jul-05	day 14	Normal	
11	8	SSG	24-Jul-05	day 21	Normal	
11	8	SSG	1-Aug-05	end day	Normal	
11	8	SSG	31-Oct-05	month 3	Normal	
11	8	SSG	30-Jan-06	month 6	Normal	

Centre Number	Patient Number	Treatment	Audiometry Date	Visit	Audiometry Findings	Comment
11	9	SSG	30-Jun-05	day 0	Normal	
11	9	SSG	9-Jul-05	day 7	Normal	
11	9	SSG	16-Jul-05	day 14	Normal	
11	9	SSG	24-Jul-05	day 21	Normal	
11	9	SSG	2-Aug-05	end day	Normal	
11	9	SSG	1-Nov-05	month 3	Normal	
11	9	SSG	30-Jan-06	month 6	Normal	
11	10	SSG	1-Jul-05	day 0	Normal	
11	10	SSG	9-Jul-05	day 7	Normal	
11	10	SSG	16-Jul-05	day 14	Normal	
11	10	SSG	24-Jul-05	day 21	Normal	
11	10	SSG	2-Aug-05	end day	Normal	
11	10	SSG	1-Nov-05	month 3	Normal	
11	10	SSG	30-Jan-06	month 6	Normal	
11	11	Combination	2-Jul-05	day 0	Normal	
11	11	Combination	11-Jul-05	day 7	Normal	
11	11	Combination	18-Jul-05	day 14	Normal	MILD HEARING LOSS AT LEFT EAR BY 500HZ - 35DB RIGHT EAR NORMAL.
11	11	Combination	22-Jul-05	end day	Normal	
11	11	Combination	21-Oct-05	month 3	Normal	
11	11	Combination	21-Jan-06	month 6	Normal	
11	12	Combination	7-Jul-05	day 0	Normal	
11	12	Combination	14-Jul-05	day 7	Abnormal (CI)	MODERATE HEARING LOSS ON LEFT EAR 8000HZ - 45DB(C)
11	12	Combination	21-Jul-05	day 14	Normal	ON HIS LEFT EAR - BY 8000HZ FREQUENCY HE HEARD ON 40DB. HIS HEARING LEVEL IS ON MILD
11	12	Combination	25-Jul-05	end day	Normal	ON HIS LEFT EAR - BY 8000HZ FREQUENCY ON 40DB. HIS HEARING LEVEL IS ON MILD
11	12	Combination	25-Oct-05	month 3	Normal	
11	12	Combination	6-Feb-06	month 6	Normal	
11	13	SSG	10-Jul-05	day 0	Normal	NORMAL EXCEPT - 8000HZ SHE HEARS ON 40DBHL IN BOTH LEFT AND RIGHT EAR - MILD
11	13	SSG	18-Jul-05	day 7	Normal	
11	13	SSG	25-Jul-05	day 14	Normal	MODERATE HEARING LOSS AT 8000HZ RT BOTH EARS
11	13	SSG	1-Aug-05	day 21	Normal	MODERATE HEARING LOSS AT 8000HZ
11	13	SSG	11-Aug-05	end day	Normal	HEARING LOSS BOTH EAR. ON RIGHT EAR 250HZ - 50DB, 500HZ - 50DB, 1000HZ - 40DB 8000HZ - 55DB. ON LEFT EAR 250HZ - 40DB 500HZ - 35DB 8000HZ - 45DB
11	13	SSG	10-Nov-05	month 3	Normal	
11	13	SSG	7-Feb-06	month 6	Normal	
11	14	SSG	7-Jul-05	day 0	Normal	
11	14	SSG	18-Jul-05	day 7	Normal	
11	14	SSG	25-Jul-05	day 14	Normal	
11	14	SSG	1-Aug-05	day 21	Normal	
11	14	SSG	11-Aug-05	end day	Abnormal (CI)	HEARING LOSS ON LEFT EAR BY 250HZ - 30DB ON RIGHT EAR NORMAL
11	14	SSG	11-Nov-05	month 3	Normal	
11	14	SSG	16-Apr-06	month 6	Normal	

Centre Number	Patient Number	Treatment	Audiometry Date	Visit	Audiometry Findings	Comment
11	15	PM	13-Jul-05	day 0	Normal	
11	15	PM	20-Jul-05	day 7	Normal	
11	15	PM	7-Nov-05	month 3	Normal	
11	15	PM	6-Apr-06	month 6	Normal	
11	16	Combination	14-Jul-05	day 0	Normal	
11	16	Combination	21-Jul-05	day 7	Normal	
11	16	Combination	28-Jul-05	day 14	Normal	
11	16	Combination	1-Aug-05	end day	Normal	
11	16	Combination	31-Oct-05	month 3	Abnormal (CI)	MODERATE HEARING LOSS ON LEFT EAR 8000HZ - 55 DBHL(AC)
11	16	Combination	6-Feb-06	month 6	Normal	
11	17	Combination	16-Jul-05	day 0	Normal	
11	17	Combination	25-Jul-05	day 7	Abnormal (CI)	LEFT EAR HAS DROPPED TO MODERATE LOSS FROM NORMAL AT BASELINE
11	17	Combination	1-Aug-05	day 14	Abnormal (CI)	LEFT EAR HAVE MODERATE LOSS ON 8000 HZ - 45 DB(C)
11	17	Combination	5-Aug-05	end day	Normal	MILD HEARING LOSS BOTH SIDE OF EAR BY 8000 HZ LEFT EAR - 40 DB RIGHT EAR - 35 DB
11	17	Combination	7-Nov-05	month 3	Normal	
11	17	Combination	7-Feb-06	month 6	Normal	
11	18	SSG	13-Aug-05	day 0	Normal	
11	18	SSG	22-Aug-05	day 7	Normal	
11	18	SSG	29-Aug-05	day 14	Normal	
11	18	SSG	5-Sep-05	day 21	Normal	MILD LOSS ON THE LEFT EAR BY 250HZ - 30DB , 500HZ - 30DB. RIGHT EAR NORMAL
11	18	SSG	15-Sep-05	end day	Normal	MILD HEARING LOSS ON THE RIGHT EAR BY 250HZ - 30DB, 500HZ - 30DB & 8000HZ - 30DB. LEFT EAR NORMAL
11	18	SSG	22-Dec-05	month 3	Normal	
11	18	SSG	15-Mar-06	month 6	Normal	
11	19	Combination	15-Aug-05	day 0	Normal	
11	19	Combination	22-Aug-05	day 7	Normal	
11	19	Combination	29-Aug-05	day 14	Normal	MILD HEARING LOSS BOTH EAR BY ON RIGHT EAR 8000HZ - 35DB, LEFT EAR 4000HZ - 30DB 8000HZ - 35DB.
11	19	Combination	2-Sep-05	end day	Normal	MILD HEARING LOSS BOTH EARS ON RIGHT EAR 4000HZ - 30DB 8000HZ - 35DB ON LEFT EAR 4000HZ - 30DB 8000HZ - 40DB
11	19	Combination	29-Nov-05	month 3	Normal	
11	19	Combination	28-Feb-06	month 6	Normal	
11	20	SSG	14-Aug-05	day 0	Normal	
11	20	SSG	22-Aug-05	day 7	Normal	
11	20	SSG	29-Aug-05	day 14	Normal	
11	20	SSG	5-Sep-05	day 21	Normal	
11	20	SSG	15-Sep-05	end day	Normal	
11	20	SSG	14-Dec-05	month 3	Normal	
11	20	SSG	14-Mar-06	month 6	Normal	
11	21	SSG	14-Aug-05	day 0	Normal	MILD HEARING LOSS ON BOTH EAR ON RIGHT EAR 500 HZ - 30 DB HL ON LEFT EAR 8000 HZ - 40 DB HL

Centre Number	Patient Number	Treatment	Audiometry Date	Visit	Audiometry Findings	Comment
11	21	SSG	22-Aug-05	day 7	Normal	MILD HEARING LOSS ON LEFT EAR 8000 HZ - 35 DBHL (AC)
11	21	SSG	29-Aug-05	day 14	Normal	MILD HEARING LOSS ON LEFT EAR 8000 HZ 30 DB HL(AC)
11	21	SSG	5-Sep-05	day 21	Normal	MILD HEARING LOSS ON BOTH EAR 8000 HZ - 30 DB(C)
11	21	SSG	15-Sep-05	end day	Normal	MILD HEARING LOSS BOTH EAR. ON THE RIGHT EAR BY. 250HZ - 35DB, 500HZ - 40DB, 1000HZ - 30DB. ON THE LEFT BY. 8000HZ - 35DB.
11	21	SSG	22-Dec-05	month 3	Normal	
11	21	SSG	15-Mar-06	month 6	Normal	
11	22	SSG	16-Aug-05	day 0	Normal	
11	22	SSG	23-Aug-05	day 7	Normal	
11	22	SSG	30-Aug-05	day 14	Normal	
11	22	SSG	6-Sep-05	day 21	Normal	MILD HEARING LOSS ON THE LEFT EAR BY 250HZ - 35DB. RIGHT EAR NORMAL
11	22	SSG	16-Sep-05	end day	Normal	
11	22	SSG	15-Dec-05	month 3	Normal	
11	22	SSG	14-Apr-06	month 6	Normal	
11	23	PM	18-Aug-05	day 0	Normal	
11	23	PM	26-Aug-05	day 7	Normal	
11	23	PM	2-Sep-05	day 14	Normal	
11	23	PM	10-Sep-05	end day	Normal	
11	23	PM	11-Jan-06	month 3	Normal	
11	23	PM	31-May-06	month 6	Normal	
11	24	PM	18-Aug-05	day 0	Normal	
11	24	PM	26-Aug-05	day 7	Normal	MILD HEARING LOSS BOTH EAR BY - 500HZ - 30 ON RIGHT EAR - 250HZ - 30 ON LEFT EAR
11	24	PM	2-Sep-05	day 14	Normal	
11	24	PM	10-Sep-05	end day	Normal	MILD HEARING LOSS ON THE LEFT EAR BY 250HZ - 30DB, 500HZ - 30DB. RIGHT EAR NORMAL
11	24	PM	28-Nov-05	month 3	Normal	
11	24	PM	10-Feb-06	month 6	Normal	
11	25	SSG	19-Aug-05	day 0	Normal	
11	25	SSG	26-Aug-05	day 7	Normal	HEARING LOSS ON THE LEFT EAR BY 500HZ - 30, 250HZ - 40. MILD
11	25	SSG	2-Sep-05	day 14	Normal	
11	25	SSG	9-Sep-05	day 21	Normal	
11	25	SSG	19-Sep-05	end day	Normal	
11	25	SSG	3-Jan-06	month 3	Normal	
11	25	SSG	14-Apr-06	month 6	Normal	
11	26	Combination	24-Aug-05	day 0	Normal	
11	26	Combination	31-Aug-05	day 7	Normal	
11	26	Combination	7-Sep-05	day 14	Normal	MILD HEARING LOSS ON THE LEFT EAR BY 8000HZ - 40DB. RIGHT EAR NORMAL
11	26	Combination	11-Sep-05	end day	Normal	
11	26	Combination	10-Nov-05	month 3	Normal	
11	26	Combination	16-Apr-06	month 6	Normal	
11	27	PM	17-Sep-05	day 0	Normal	MILD HEARING IMPAIRMENT AT RIGHT EAR BY 8000HZ - 40DB

Centre Number	Patient Number	Treatment	Audiometry Date	Visit	Audiometry Findings	Comment
11	27	PM	25-Sep-05	day 7	Normal	MILD HEARING LOSS ON LEFT EAR AT 250HZ - 30DBHL
11	27	PM	2-Oct-05	day 14	Normal	MILD HEARING LOSS ON LEFT EAR 500 AND 8000HZ - 30 DB(C) AND 250HZ 35DB(C)
11	27	PM	10-Oct-05	end day	Normal	MILD HEARING LOSS ON LEFT EAR 8000HZ 40DB(A)
11	27	PM	30-Jan-06	month 3	Abnormal (CI)	MILD HEARING LOSS (26 TO 40) FOR THE RIGHT EAR AND MODERATE HEARING LOSS FOR THE LEFT EAR
11	28	Combination	17-Sep-05	day 0	Normal	
11	28	Combination	27-Sep-05	day 7	Normal	
11	28	Combination	4-Oct-05	day 14	Normal	
11	28	Combination	8-Oct-05	end day	Normal	
11	28	Combination	5-Jan-06	month 3	Normal	
11	28	Combination	4-Apr-06	month 6	Normal	
11	29	PM	20-Sep-05	day 0	Normal	MILD HEARING LOSS ON LEFT EAR BY 250HZ - 30DB. RIGHT EAR NORMAL
11	29	PM	28-Sep-05	day 7	Normal	MILD HEARING LOSS ON LEFT EAR 500 AND 1000HZ - 30DBHL (AC)
11	29	PM	5-Oct-05	day 14	Normal	MODERATE HEARING LOSS ON LEFT EAR ON 2000 AND 8000HZ - 35DBA, ON 500 AND 4000HZ - 40DBA AND ON 250HZ - 45 DBA
11	29	PM	13-Oct-05	end day	Normal	
11	29	PM	11-Jan-06	month 3	Normal	
11	29	PM	18-Apr-06	month 6	Normal	
11	30	PM	22-Sep-05	day 0	Normal	MILD HEARING LOSS ON RIGHT EAR BY 500HZ - 30DB ON LEFT BY 8000HZ - 40DB
11	30	PM	28-Sep-05	day 7	Normal	MILD HEARING LOSS ON BOTH EAR ON RIGHT EAR 4000 AND 8000HZ - 30 DBA ON LEFT EAR 500, 1000, 2000 - 30DBA AND 250 AND 8000HZ - 35DBA
11	30	PM	5-Oct-05	day 14	Normal	MILD HEARING LOSS ON BOTH EAR, LEFT EAR 8000HZ - 40DBA AND ON RIGHT EAR 500HZ - 30DBA
11	30	PM	13-Oct-05	end day	Normal	MODERATE HEARING LOSS ON LEFT EAR 8000HZ - 45DB(A)
11	30	PM	3-Jan-06	month 3	Normal	
11	30	PM	14-Apr-06	month 6	Normal	
11	31	SSG	21-Sep-05	day 0	Normal	MILD HEARING LOSS ON BOTH RIGHT AND LEFT EAR
11	31	SSG	29-Sep-05	day 7	Normal	MILD HEARING LOSS ON LEFT EAR 500 AND 1000HZ - 30DBHL(AC) AND 250HZ - 35DBHL(C)
11	31	SSG	6-Oct-05	day 14	Normal	MODERATE HEARING LOSS ON LEFT EAR 4000HZ - 45DBA, 8000HZ - 50DBA AND 250HZ - 55DBA AND 500HZ - 50DBA
11	31	SSG	13-Oct-05	day 21	Normal	MODERATE HEARING LOSS ON LEFT EAR 250HZ - 55DB(A) AND 500HZ - 50DB(A) AND MILD HEARING LOSS ON RIGHT EAR

Centre Number	Patient Number	Treatment	Audiometry Date	Visit	Audiometry Findings	Comment
11	31	SSG	23-Oct-05	end day	Normal	MILD HEARING LOSS ON LEFT EAR 250 AND 500HZ - 40DB(A) AND 1000HZ AND 8000HZ - 35DB(A)
11	31	SSG	3-Feb-06	month 3	Normal	
11	31	SSG	7-Apr-06	month 6	Normal	
11	32	Combination	22-Sep-05	day 0	Abnormal (CI)	MODERATE HEARING LOSS ON RIGHT EAR 8000HZ - 50DBHL
11	32	Combination	30-Sep-05	day 7	Abnormal (CI)	MODERATELY SEVERE ON RIGHT EAR ON 8000HZ - DBA
11	32	Combination	7-Oct-05	day 14	Normal	MILD HEARING LOSS ON BOTH EAR ON RIGHT EAR - 250, 500 AND 8000HZ - 30DBA AND ON LEFT EAR 250 AND 500HZ - 35DBA
11	32	Combination	11-Oct-05	end day	Normal	MILD HEARING LOSS ON BOTH EAR ON RIGHT EAR 8000HZ - 35DB(A) ON LEFT EAR 500HZ - 30DB(A) AND 250HZ 35DB(A)
11	32	Combination	10-Jan-06	month 3	Normal	
11	32	Combination	3-Apr-06	month 6	Normal	
11	33	SSG	24-Sep-05	day 0	Abnormal (CI)	MILD HEARING LOSS ON RIGHT EAR 4000HZ - 35DBHL
11	33	SSG	1-Oct-05	day 7	Abnormal (CI)	MILD HEARING LOSS ON RIGHT EAR 8000HZ - 30DB(C) AND MODERATE HEARING LOSS ON LEFT EAR ON 4000HZ 45DB(C)
11	33	SSG	8-Oct-05	day 14	Normal	MILD HEARING LOSS ON 8000HZ - 35DB(A) ON RIGHT EAR
11	33	SSG	15-Oct-05	day 21	Normal	MILD HEARING LOSS ON RIGHT EAR 4000HZ AND 8000HZ - 30DB(A)
11	33	SSG	25-Oct-05	end day	Normal	
11	33	SSG	24-Jan-06	month 3	Normal	
11	33	SSG	16-Apr-06	month 6	Normal	
11	34	PM	26-Sep-05	day 0	Abnormal (CI)	MILD HEARING LOSS ON LEFT EAR ON 500 AND 250HZ - 30DBHL(AC)
11	34	PM	3-Oct-05	day 7	Normal	
11	34	PM	10-Oct-05	day 14	Normal	
11	34	PM	18-Oct-05	end day	Normal	
11	34	PM	17-Jan-06	month 3	Normal	
11	34	PM	16-Apr-06	month 6	Normal	
11	35	SSG	25-Sep-05	day 0	Normal	MILD HEARING LOSS ON RIGHT EAR 500HZ - 30DBHL(AC)
11	35	SSG	3-Oct-05	day 7	Abnormal (CI)	MODERATE HEARING LOSS ON BOTH EAR RIGHT EAR - 8000HZ - 55DB(C) LEFT EAR 8000HZ - 55DB(C)
11	35	SSG	10-Oct-05	day 14	Normal	MILD HEARING LOSS ON BOTH EAR ON LEFT EAR 8000HZ - 30DB(A) ON RIGHT EAR 500HZ - 40DB (A) 250HZ - 35DB(A)
11	35	SSG	17-Oct-05	day 21	Normal	MILD HEARING LOSS ON LEFT EAR 8000HZ 30DBA AND ON RIGHT EAR 250HZ AND 500HZ - 30DBA
11	35	SSG	27-Oct-05	end day	Normal	
11	35	SSG	11-Jan-06	month 3	Normal	
11	35	SSG	16-Apr-06	month 6	Normal	
11	36	SSG	27-Sep-05	day 0	Abnormal (CI)	MODERATE HEARING LOSS ON LEFT EAR 8000HZ - 50DBA

Centre Number	Patient Number	Treatment	Audiometry Date	Visit	Audiometry Findings	Comment
11	36	SSG	4-Oct-05	day 7	Normal	MODERATE HEARING LOSS AT LEFT EAR BY 8000HZ - 45DB. NORMAL AT RIGHT EAR
11	36	SSG	11-Oct-05	day 14	Normal	MILD HEARING LOSS ON LEFT EAR 8000HZ - 35DB(A)
11	36	SSG	18-Oct-05	day 21	Normal	MILD HEARING LOSS OF LEFT EAR BY 8000HZ - 35DB
11	36	SSG	28-Oct-05	end day	Normal	MILD HEARING LOSS AT LEFT EAR BY 250HZ - 30DB(A) & 500HZ - 30DB(A). RIGHT EAR NORMAL.
11	36	SSG	25-Jan-06	month 3	Normal	
11	36	SSG	10-Jun-06	month 6	Normal	
11	37	Combination	27-Sep-05	day 0	Normal	MILD HEARING LOSS FOR RT EAR BY 8000HZ - 35DBA AND MODERATE FOR LT EAR
11	37	Combination	4-Oct-05	day 7	Normal	MILD HEARING LOSS AT RIGHT EAR 8000HZ - 30DB BY LEFT EAR 2000HZ - 30DB, 4000HZ - 45DB 8000HZ - 45DB - MODERATE FOR THE RT EAR
11	37	Combination	11-Oct-05	day 14	Normal	ON LEFT EAR MODERATE HEARING LOSS 4000HZ - 50DB(A). 8000HZ - 40DB(A) ON RIGHT EAR MILD HEARING LOSS 4000HZ - 40DB(A)
11	37	Combination	15-Oct-05	end day	Normal	MODERATE HEARING LOSS ON LEFT EAR 4000HZ - 50DB(A)
11	37	Combination	17-Jan-06	month 3	Normal	
11	37	Combination	14-Apr-06	month 6	Normal	
11	38	PM	17-Oct-05	day 0	Normal	MODERATE HEARING LOSS ON RIGHT EAR 8000HZ - 45DB(A) MILD HEARING LOSS ON LEFT EAR 4000 AND 8000HZ - 40DB(A)
11	38	PM	25-Oct-05	day 7	Normal	MODERATE HEARING LOSS ON BOTH EAR RIGHT EAR 8000HZ - 50DBA AND ON LEFT EAR 4000HZ - 40DB(A) 8000HZ - 45DB(A)
11	38	PM	1-Nov-05	day 14	Normal	MODERATE HEARING LOSS ON BOTH EAR ON LEFT EAR 8000HZ - 50 AND ON RIGHT EAR 8000HZ - 50DBA
11	38	PM	9-Nov-05	end day	Normal	MODERATE HEARING LOSS BOTH EAR. AT LEFT EAR ON 4000HZ - 40DB(A) & 8000HZ - 50DB(A). AT RIGHT EAR BY 8000HZ - 50DB(A).
11	38	PM	15-Feb-06	month 3	Normal	
11	38	PM	1-Jun-06	month 6	Normal	
11	39	PM	18-Oct-05	day 0	Normal	MILD HEARING LOSS ON BOTH EAR 250HZ AND 2000HZ - 30DBA ON RIGHT EAR 500 AND 8000HZ - 35DB(A) - RIGHT EAR ON LEFT EAR. 250HZ AND 8000HZ - 35DB(A)
11	39	PM	25-Oct-05	day 7	Normal	MILD HEARING LOSS ON BOTH EAR ON RIGHT EAR 8000HZ - 40DB(A) AND ON LEFT EAR 250 AND 500HZ - 35DB(A) AND 8000HZ - 40DB(A)

Centre Number	Patient Number	Treatment	Audiometry Date	Visit	Audiometry Findings	Comment
11	39	PM	1-Nov-05	day 14	Normal	MILD HEARING LOSS ON BOTH EAR ON RIGHT EAR 8000, 500 AND 1000HZ - 40DBA ON LEFT EAR 250, 500 AND 8000HZ - 40DBA
11	39	PM	9-Nov-05	end day	Normal	MILD HEARING LOSS BOTH EAR. AT RIGHT EAR BY 250HZ - 30DB(A), 500HZ, 1000HZ - 30DB(A) & 8000HZ - 35DB(A). AT LEFT EAR BY 250HZ - 35DB(A)
11	39	PM	7-Mar-06	month 3	Abnormal (CI)	MODERATLY - SEVERE ON BOTH EAR ON RIGHT EAR 8000HZ - 70 DBHL AND ON THE LEFT EAR 4000HZ - 60 DBHL
11	39	PM	22-May-06	month 6	Normal	
11	40	PM	19-Oct-05	day 0	Normal	
11	40	PM	26-Oct-05	day 7	Normal	
11	40	PM	2-Nov-05	day 14	Normal	
11	40	PM	14-Apr-06	month 3	Normal	
11	40	PM	24-May-06	month 6	Normal	
11	41	Combination	19-Oct-05	day 0	Normal	MILD HEARING LOSS ON RIGHT EAR 250HZ - 35DB(A) AND 1000 AND 2000HZ - 30DB(A)
11	41	Combination	26-Oct-05	day 7	Normal	
11	41	Combination	2-Nov-05	day 14	Normal	
11	41	Combination	6-Nov-05	end day	Normal	MILD HEARING LOSS BOTH EAR AT LEFT EAR BY 500HZ - 35DB(A) 250HZ - 35DB(A). RIGHT EAR BY 500HZ - 30DB(A) 250HZ - 35DB(A)
11	41	Combination	13-Feb-06	month 3	Normal	
11	41	Combination	31-May-06	month 6	Normal	
11	42	Combination	19-Oct-05	day 0	Normal	MODERATE HEARING LOSS ON LEFT EAR 250 AND 500HZ - 45DB(A)
11	42	Combination	27-Oct-05	day 7	Normal	MODERATE HEARING LOSS AT LEFT EAR BY 250HZ - 45DBA, 500HZ - 40DBA & 1000HZ - 30DBA. RIGHT EAR NORMAL
11	42	Combination	3-Nov-05	day 14	Normal	MILD HEARING LOSS ON LEFT EAR 250 AND 500HZ - 30DBA
11	42	Combination	7-Nov-05	end day	Normal	
11	42	Combination	7-Feb-06	month 3	Normal	
11	42	Combination	8-Jun-06	month 6	Normal	
11	43	SSG	27-Oct-05	day 0	Normal	
11	43	SSG	3-Nov-05	day 7	Normal	
11	43	SSG	10-Nov-05	day 14	Normal	MILD HEARING LOSS ON LEFT EAR 250HZ 40DBA AND 500HZ - 35DBA
11	43	SSG	17-Nov-05	day 21	Normal	MILD HEARING LOSS AT LEFT EAR BY 250HZ - DB(A) AND 500HZ - 30DB(A). RIGHT EAR NORMAL.
11	43	SSG	27-Nov-05	end day	Normal	MILD HEARING LOSS ON LEFT EAR ON 250 AND 500HZ - 40DBA AND 1000HZ - 35DBA
11	43	SSG	23-Feb-06	month 3	Normal	
11	43	SSG	25-May-06	month 6	Normal	
11	44	PM	27-Oct-05	day 0	Normal	
11	44	PM	3-Nov-05	day 7	Normal	

Centre Number	Patient Number	Treatment	Audiometry Date	Visit	Audiometry Findings	Comment
11	44	PM	10-Nov-05	day 14	Normal	MILD HEARING LOSS ON BOTH EAR RIGHT EAR 500HZ - 30DBA LEFT EAR 250HZ - 30DBA, 500HZ. 35DBA
11	44	PM	18-Nov-05	end day	Normal	MILD HEARING LOSS AT LEFT EAR BY 250HZ - 30DB(A) AND 500HZ - 35DB(A). RIGHT EAR NORMAL
11	44	PM	14-Feb-06	month 3	Normal	
11	44	PM	19-May-06	month 6	Normal	
11	45	Combination	27-Oct-05	day 0	Normal	
11	45	Combination	3-Nov-05	day 7	Normal	MILD HEARING LOSS ON LEFT EAR ON 250HZ - 35DBA AND 500HZ - 30DBA
11	45	Combination	10-Nov-05	day 14	Normal	MILD HEARING LOSS ON LEFT EAR 250 AND 500HZ - 40DBA
11	45	Combination	14-Nov-05	end day	Normal	
11	45	Combination	14-Feb-06	month 3	Normal	
11	45	Combination	19-May-06	month 6	Normal	
11	46	SSG	4-May-06	day 0	Normal	
11	46	SSG	11-May-06	day 7	Normal	
11	46	SSG	18-May-06	day 14	Normal	
11	46	SSG	25-May-06	day 21	Normal	
11	46	SSG	4-Jun-06	end day	Normal	
11	46	SSG	24-Oct-06	month 3	Normal	
11	46	SSG	5-Dec-06	month 6	Normal	
11	47	SSG	4-May-06	day 0	Normal	
11	47	SSG	11-May-06	day 7	Normal	
11	47	SSG	18-May-06	day 14	Normal	
11	47	SSG	25-May-06	day 21	Normal	
11	47	SSG	4-Jun-06	end day	Normal	
11	48	Combination	4-May-06	day 0	Normal	
11	48	Combination	11-May-06	day 7	Normal	
11	48	Combination	18-May-06	day 14	Normal	
11	48	Combination	22-May-06	end day	Normal	
11	48	Combination	2-Jan-07	month 6	Normal	
11	49	SSG	8-May-06	day 0	Normal	
11	49	SSG	15-May-06	day 7	Normal	
11	49	SSG	22-May-06	day 14	Normal	
11	49	SSG	29-May-06	day 21	Normal	
11	49	SSG	8-Jun-06	end day	Normal	
11	49	SSG	9-Oct-06	month 3	Normal	
11	49	SSG	27-Feb-07	month 6	Normal	
11	50	PM	17-May-06	day 0	Normal	
11	50	PM	24-May-06	day 7	Normal	
11	50	PM	31-May-06	day 14	Normal	
11	50	PM	8-Jun-06	end day	Normal	
11	50	PM	6-Sep-06	month 3	Normal	
11	50	PM	11-Dec-06	month 6	Normal	
11	51	Combination	19-May-06	day 0	Normal	
11	51	Combination	26-May-06	day 7	Normal	
11	51	Combination	2-Jun-06	day 14	Normal	
11	51	Combination	6-Jun-06	end day	Normal	
11	51	Combination	7-Sep-06	month 3	Normal	
11	51	Combination	2-Jan-07	month 6	Normal	
11	52	PM	19-May-06	day 0	Normal	
11	52	PM	26-May-06	day 7	Normal	

Centre Number	Patient Number	Treatment	Audiometry Date	Visit	Audiometry Findings	Comment
11	52	PM	2-Jun-06	day 14	Normal	
11	52	PM	10-Jun-06	end day	Normal	
11	52	PM	26-Sep-06	month 3	Normal	
11	52	PM	11-Jan-07	month 6	Normal	
11	53	SSG	25-May-06	day 0	Normal	
11	53	SSG	1-Jun-06	day 7	Normal	
11	53	SSG	8-Jun-06	day 14	Normal	
11	53	SSG	15-Jun-06	day 21	Normal	
11	53	SSG	25-Jun-06	end day	Normal	
11	53	SSG	26-Sep-06	month 3	Normal	
11	53	SSG	11-Dec-06	month 6	Normal	
11	54	PM	26-May-06	day 0	Normal	
11	54	PM	2-Jun-06	day 7	Normal	
11	54	PM	9-Jun-06	day 14	Normal	
11	54	PM	17-Jun-06	end day	Normal	
11	54	PM	28-Aug-06	month 3	Normal	
11	54	PM	9-Jan-07	month 6	Normal	
11	55	PM	29-May-06	day 0	Normal	
11	55	PM	5-Jun-06	day 7	Normal	
11	55	PM	12-Jun-06	day 14	Normal	
11	55	PM	20-Jun-06	end day	Normal	
11	55	PM	26-Sep-06	month 3	Normal	
11	55	PM	11-Dec-06	month 6	Normal	
11	56	SSG	29-May-06	day 0	Normal	
11	56	SSG	6-Jun-06	day 7	Normal	
11	56	SSG	13-Jun-06	day 14	Normal	
11	56	SSG	20-Jun-06	day 21	Normal	
11	56	SSG	30-Jun-06	end day	Normal	
11	56	SSG	28-Sep-06	month 3	Normal	
11	56	SSG	14-Dec-06	month 6	Normal	
11	57	Combination	3-Jun-06	day 0	Normal	
11	57	Combination	10-Jun-06	day 7	Normal	
11	57	Combination	17-Jun-06	day 14	Normal	
11	57	Combination	21-Jun-06	end day	Normal	
11	57	Combination	12-Sep-06	month 3	Normal	
11	57	Combination	12-Dec-06	month 6	Normal	
11	58	Combination	5-Jun-06	day 0	Normal	
11	58	Combination	12-Jun-06	day 7	Normal	
11	58	Combination	19-Jun-06	day 14	Normal	
11	58	Combination	23-Jun-06	end day	Normal	
11	58	Combination	26-Sep-06	month 3	Normal	
11	58	Combination	13-Dec-06	month 6	Normal	
11	59	PM	12-Jun-06	day 0	Normal	
11	59	PM	19-Jun-06	day 7	Normal	
11	59	PM	26-Jun-06	day 14	Normal	
11	59	PM	4-Jul-06	end day	Normal	
11	59	PM	4-Oct-06	month 3	Normal	
11	59	PM	2-Jan-07	month 6	Normal	
11	60	Combination	12-Jun-06	day 0	Normal	
11	60	Combination	19-Jun-06	day 7	Normal	
11	60	Combination	26-Jun-06	day 14	Normal	
11	60	Combination	30-Jun-06	end day	Normal	
11	60	Combination	28-Sep-06	month 3	Normal	
11	60	Combination	18-Dec-06	month 6	Normal	

Centre Number	Patient Number	Treatment	Audiometry Date	Visit	Audiometry Findings	Comment
11	61	PM	12-Jun-06	day 0	Normal	
11	61	PM	19-Jun-06	day 7	Normal	
11	61	PM	26-Jun-06	day 14	Normal	
11	61	PM	4-Jul-06	end day	Normal	
11	61	PM	3-Oct-06	month 3	Normal	
11	61	PM	2-Jan-07	month 6	Normal	
11	62	SSG	15-Jun-06	day 0	Normal	
11	62	SSG	22-Jun-06	day 7	Normal	
11	62	SSG	29-Jun-06	day 14	Normal	
11	62	SSG	6-Jul-06	day 21	Normal	
11	62	SSG	16-Jul-06	end day	Normal	
11	62	SSG	25-Oct-06	month 3	Normal	
11	62	SSG	11-Jan-07	month 6	Normal	
11	63	SSG	19-Jun-06	day 0	Normal	
11	63	SSG	26-Jun-06	day 7	Normal	
11	63	SSG	3-Jul-06	day 14	Normal	
11	63	SSG	10-Jul-06	day 21	Normal	
11	63	SSG	20-Jul-06	end day	Normal	
11	63	SSG	3-Oct-06	month 3	Normal	
11	63	SSG	28-Dec-06	month 6	Normal	
11	64	SSG	21-Jun-06	day 0	Normal	
11	64	SSG	28-Jun-06	day 7	Normal	
11	64	SSG	5-Jul-06	day 14	Normal	
11	64	SSG	12-Jul-06	day 21	Normal	
11	64	SSG	22-Jul-06	end day	Normal	
11	64	SSG	29-Sep-06	month 3	Normal	
11	64	SSG	14-Dec-06	month 6	Normal	
11	65	PM	21-Jun-06	day 0	Normal	
11	65	PM	29-Jun-06	day 7	Normal	
11	65	PM	6-Jul-06	day 14	Normal	
11	65	PM	14-Jul-06	end day	Normal	
11	66	Combination	22-Jun-06	day 0	Normal	
11	66	Combination	29-Jun-06	day 7	Normal	
11	66	Combination	6-Jul-06	day 14	Abnormal (CI)	MODERATE HEARING LOSS ON BOTH EAR ON 8000HZ - 55DBHL(AC)
11	66	Combination	10-Jul-06	end day	Normal	
11	66	Combination	26-Sep-06	month 3	Normal	
11	66	Combination	11-Dec-06	month 6	Normal	
11	67	PM	30-Jun-06	day 0	Normal	
11	67	PM	7-Jul-06	day 7	Normal	
11	67	PM	14-Jul-06	day 14	Normal	
11	67	PM	22-Jul-06	end day	Normal	
11	67	PM	3-Oct-06	month 3	Normal	
11	67	PM	2-Jan-07	month 6	Normal	
11	68	SSG	7-Jul-06	day 0	Normal	
11	68	SSG	14-Jul-06	day 7	Normal	
11	68	SSG	21-Jul-06	day 14	Normal	
11	68	SSG	28-Jul-06	day 21	Normal	
11	68	SSG	7-Aug-06	end day	Normal	
11	68	SSG	11-Oct-06	month 3	Normal	
11	68	SSG	9-Jan-07	month 6	Normal	
11	69	Combination	14-Jul-06	day 0	Normal	
11	69	Combination	21-Jul-06	day 7	Normal	
11	69	Combination	28-Jul-06	day 14	Normal	

Centre Number	Patient Number	Treatment	Audiometry Date	Visit	Audiometry Findings	Comment
11	69	Combination	1-Aug-06	end day	Normal	
11	69	Combination	9-Oct-06	month 3	Normal	
11	69	Combination	13-Dec-06	month 6	Normal	
11	70	Combination	14-Jul-06	day 0	Normal	
11	70	Combination	21-Jul-06	day 7	Normal	
11	70	Combination	28-Jul-06	day 14	Normal	
11	70	Combination	1-Aug-06	end day	Normal	
11	70	Combination	9-Oct-06	month 3	Normal	
11	70	Combination	2-Jan-07	month 6	Normal	
11	71	Combination	21-Jul-06	day 0	Normal	
11	71	Combination	28-Jul-06	day 7	Normal	
11	71	Combination	4-Aug-06	day 14	Normal	
11	71	Combination	8-Aug-06	end day	Normal	
11	71	Combination	20-Oct-06	month 3	Normal	
11	71	Combination	8-Jan-07	month 6	Normal	
11	72	SSG	21-Jul-06	day 0	Normal	
11	72	SSG	28-Jul-06	day 7	Normal	
11	72	SSG	4-Aug-06	day 14	Abnormal (CI)	MODERATELY SEVERE HEARING LOSS IN LEFT EAR AT 8000HZ 65DBHL(AC)
11	72	SSG	9-Nov-06	month 3	Normal	
11	72	SSG	7-Feb-07	month 6	Normal	
11	73	PM	24-Jul-06	day 0	Normal	
11	73	PM	31-Jul-06	day 7	Normal	
11	73	PM	7-Aug-06	day 14	Normal	
11	73	PM	15-Aug-06	end day	Normal	
11	73	PM	23-Oct-06	month 3	Normal	
11	73	PM	2-Apr-07	month 6	Normal	
11	74	PM	27-Jul-06	day 0	Normal	
11	74	PM	3-Aug-06	day 7	Normal	
11	74	PM	10-Aug-06	day 14	Normal	
11	74	PM	18-Aug-06	end day	Normal	
11	74	PM	25-Oct-06	month 3	Normal	
11	74	PM	23-Jan-07	month 6	Normal	
11	75	Combination	27-Jul-06	day 0	Normal	
11	75	Combination	3-Aug-06	day 7	Normal	
11	75	Combination	10-Aug-06	day 14	Normal	
11	75	Combination	14-Aug-06	end day	Normal	
11	75	Combination	31-Oct-06	month 3	Normal	
11	75	Combination	9-Jan-07	month 6	Normal	
11	76	PM	27-Jul-06	day 0	Normal	
11	76	PM	6-Aug-06	day 7	Normal	
11	76	PM	13-Aug-06	day 14	Normal	
11	76	PM	21-Aug-06	end day	Normal	
11	76	PM	30-Oct-06	month 3	Normal	
11	76	PM	29-Jan-07	month 6	Normal	
11	77	PM	30-Jul-06	day 0	Normal	
11	77	PM	6-Aug-06	day 7	Normal	
11	77	PM	13-Aug-06	day 14	Normal	
11	77	PM	21-Aug-06	end day	Normal	
11	77	PM	30-Oct-06	month 3	Normal	
11	77	PM	29-Jan-07	month 6	Normal	
11	78	SSG	30-Jul-06	day 0	Normal	
11	78	SSG	6-Aug-06	day 7	Normal	
11	78	SSG	13-Aug-06	day 14	Normal	

Centre Number	Patient Number	Treatment	Audiometry Date	Visit	Audiometry Findings	Comment
11	78	SSG	20-Aug-06	day 21	Normal	
11	78	SSG	30-Aug-06	end day	Normal	
11	78	SSG	30-Oct-06	month 3	Normal	
11	78	SSG	29-Jan-07	month 6	Normal	
11	79	PM	29-Jul-06	day 0	Normal	
11	79	PM	6-Aug-06	day 7	Normal	
11	79	PM	13-Aug-06	day 14	Normal	
11	79	PM	21-Aug-06	end day	Normal	
11	79	PM	30-Oct-06	month 3	Normal	
11	79	PM	30-Jan-07	month 6	Normal	
11	80	PM	2-Aug-06	day 0	Normal	
11	80	PM	9-Aug-06	day 7	Normal	
11	80	PM	16-Aug-06	day 14	Normal	
11	80	PM	24-Aug-06	end day	Normal	
11	80	PM	23-Nov-06	month 3	Normal	
11	81	Combination	5-Aug-06	day 0	Normal	
11	81	Combination	12-Aug-06	day 7	Normal	
11	81	Combination	19-Aug-06	day 14	Normal	
11	81	Combination	23-Aug-06	end day	Normal	
11	81	Combination	7-Nov-06	month 3	Normal	
11	81	Combination	6-Feb-07	month 6	Normal	
11	82	SSG	13-Aug-06	day 0	Normal	
11	82	SSG	21-Aug-06	day 7	Normal	
11	82	SSG	28-Aug-06	day 14	Normal	
11	82	SSG	4-Sep-06	day 21	Normal	
11	82	SSG	14-Sep-06	end day	Normal	
11	82	SSG	13-Nov-06	month 3	Normal	
11	82	SSG	5-Feb-07	month 6	Normal	
11	83	SSG	15-Aug-06	day 0	Normal	
11	83	SSG	22-Aug-06	day 7	Normal	
11	83	SSG	29-Aug-06	day 14	Normal	
11	83	SSG	5-Sep-06	day 21	Normal	
11	83	SSG	15-Sep-06	end day	Normal	
11	83	SSG	13-Nov-06	month 3	Normal	
11	83	SSG	15-Feb-07	month 6	Normal	
11	84	SSG	13-Aug-06	day 0	Normal	
11	84	SSG	24-Aug-06	day 7	Normal	
11	84	SSG	31-Aug-06	day 14	Normal	
11	84	SSG	7-Sep-06	day 21	Normal	
11	84	SSG	17-Sep-06	end day	Normal	
11	84	SSG	15-Nov-06	month 3	Normal	
11	84	SSG	15-Feb-07	month 6	Normal	
11	85	SSG	24-Aug-06	day 0	Normal	
11	85	SSG	31-Aug-06	day 7	Normal	
11	85	SSG	7-Sep-06	day 14	Normal	
11	85	SSG	14-Sep-06	day 21	Normal	
11	85	SSG	24-Sep-06	end day	Normal	
11	85	SSG	21-Nov-06	month 3	Normal	
11	86	Combination	28-Aug-06	day 0	Normal	
11	86	Combination	4-Sep-06	day 7	Normal	
11	86	Combination	11-Sep-06	day 14	Normal	
11	86	Combination	15-Sep-06	end day	Normal	
11	86	Combination	13-Nov-06	month 3	Normal	
11	86	Combination	5-Feb-07	month 6	Normal	

Centre Number	Patient Number	Treatment	Audiometry Date	Visit	Audiometry Findings	Comment
11	87	Combination	28-Aug-06	day 0	Normal	
11	87	Combination	4-Sep-06	day 7	Normal	
11	87	Combination	11-Sep-06	day 14	Normal	
11	87	Combination	15-Sep-06	end day	Normal	
11	87	Combination	14-Nov-06	month 3	Normal	
11	87	Combination	5-Feb-07	month 6	Normal	
11	88	PM	28-Aug-06	day 0	Normal	
11	88	PM	4-Sep-06	day 7	Normal	
11	88	PM	11-Sep-06	day 14	Normal	
11	88	PM	19-Sep-06	end day	Normal	
11	88	PM	21-Nov-06	month 3	Normal	
11	88	PM	27-Feb-07	month 6	Normal	
11	89	Combination	28-Aug-06	day 0	Normal	
11	89	Combination	8-Sep-06	day 7	Normal	
11	89	Combination	15-Sep-06	day 14	Normal	
11	89	Combination	19-Sep-06	end day	Normal	
11	89	Combination	17-Nov-06	month 3	Normal	
11	89	Combination	15-Mar-07	month 6	Normal	
11	90	Combination	1-Sep-06	day 0	Normal	
11	90	Combination	8-Sep-06	day 7	Normal	
11	90	Combination	15-Sep-06	day 14	Normal	
11	90	Combination	19-Sep-06	end day	Normal	
11	91	PM	17-Dec-06	day 0	Normal	
11	91	PM	31-Dec-06	day 14	Normal	
11	91	PM	8-Jan-07	end day	Normal	
11	91	PM	18-Jun-07	month 6	Normal	
11	92	Combination	30-Dec-06	day 0	Normal	
11	92	Combination	13-Jan-07	day 14	Normal	
11	92	Combination	17-Jan-07	end day	Normal	
11	92	Combination	26-Jun-07	month 6	Normal	
11	93	SSG	1-Jan-07	day 0	Normal	
11	93	SSG	8-Jan-07	day 7	Normal	
11	93	SSG	15-Jan-07	day 14	Normal	
11	93	SSG	22-Jan-07	day 21	Normal	
11	93	SSG	1-Feb-07	end day	Normal	
11	93	SSG	7-Aug-07	month 6	Normal	
11	94	SSG	3-Jan-07	day 0	Normal	
11	94	SSG	17-Jan-07	day 14	Normal	
11	94	SSG	3-Feb-07	end day	Normal	
11	94	SSG	25-Jun-07	month 6	Normal	
11	95	SSG	3-Jan-07	day 0	Normal	
11	95	SSG	17-Jan-07	day 14	Normal	
11	95	SSG	3-Feb-07	end day	Normal	
11	95	SSG	23-Jun-07	month 6	Normal	
11	96	Combination	19-Jan-07	day 0	Normal	
11	96	Combination	2-Feb-07	day 14	Normal	
11	96	Combination	6-Feb-07	end day	Normal	
11	96	Combination	6-Aug-07	month 6	Normal	
11	97	Combination	19-Jan-07	day 0	Normal	
11	97	Combination	2-Feb-07	day 14	Normal	
11	97	Combination	6-Feb-07	end day	Normal	
11	97	Combination	24-Jul-07	month 6	Normal	
11	98	PM	23-Jan-07	day 0	Normal	
11	98	PM	6-Feb-07	day 14	Normal	

Centre Number	Patient Number	Treatment	Audiometry Date	Visit	Audiometry Findings	Comment
11	98	PM	14-Feb-07	end day	Normal	
11	98	PM	26-Jul-07	month 6	Normal	
11	99	SSG	25-Jan-07	day 0	Normal	
11	99	SSG	8-Feb-07	day 14	Normal	
11	99	SSG	25-Feb-07	end day	Normal	
11	99	SSG	30-Jul-07	month 6	Normal	
11	100	PM	25-Jan-07	day 0	Normal	
11	100	PM	8-Feb-07	day 14	Normal	
11	100	PM	16-Feb-07	end day	Normal	
11	101	PM	2-Feb-07	day 0	Normal	
11	101	PM	16-Feb-07	day 14	Normal	
11	101	PM	24-Feb-07	end day	Normal	
11	101	PM	6-Nov-07	month 6	Normal	
11	102	Combination	2-Feb-07	day 0	Normal	
11	102	Combination	16-Feb-07	day 14	Normal	
11	102	Combination	20-Feb-07	end day	Normal	
11	102	Combination	30-Jul-07	month 6	Normal	
11	103	Combination	6-Feb-07	day 0	Normal	
11	103	Combination	20-Feb-07	day 14	Normal	
11	103	Combination	24-Feb-07	end day	Normal	
11	103	Combination	10-Aug-07	month 6	Normal	
11	104	PM	9-Feb-07	day 0	Normal	
11	104	PM	23-Feb-07	day 14	Normal	
11	104	PM	3-Mar-07	end day	Normal	
11	104	PM	27-Aug-07	month 6	Normal	
11	105	SSG	23-Feb-07	day 0	Normal	
11	105	SSG	9-Mar-07	day 14	Normal	
11	105	SSG	26-Mar-07	end day	Normal	
11	105	SSG	27-Aug-07	month 6	Normal	
11	106	Combination	24-Feb-07	day 0	Normal	
11	106	Combination	10-Mar-07	day 14	Normal	
11	106	Combination	14-Mar-07	end day	Normal	
11	106	Combination	19-Aug-07	month 6	Normal	
11	107	PM	24-Feb-07	day 0	Normal	
11	107	PM	10-Mar-07	day 14	Normal	
11	107	PM	18-Mar-07	end day	Normal	
11	107	PM	28-Aug-07	month 6	Normal	
11	108	PM	28-Feb-07	day 0	Normal	
11	108	PM	15-Mar-07	day 14	Normal	
11	108	PM	23-Mar-07	end day	Normal	
11	108	PM	27-Aug-07	month 6	Normal	
11	109	SSG	27-Feb-07	day 0	Normal	
11	109	SSG	16-Mar-07	day 14	Normal	
11	109	SSG	2-Apr-07	end day	Normal	
11	109	SSG	5-Aug-07	month 6	Normal	
11	110	PM	2-Mar-07	day 0	Normal	
11	110	PM	16-Mar-07	day 14	Normal	
11	110	PM	24-Mar-07	end day	Normal	
11	110	PM	27-Aug-07	month 6	Normal	
11	111	Combination	23-Mar-07	day 0	Normal	
11	111	Combination	6-Apr-07	day 14	Normal	
11	111	Combination	10-Apr-07	end day	Normal	
11	111	Combination	24-Sep-07	month 6	Normal	
11	112	Combination	23-Mar-07	day 0	Normal	

Centre Number	Patient Number	Treatment	Audiometry Date	Visit	Audiometry Findings	Comment
11	112	Combination	6-Apr-07	day 14	Normal	
11	112	Combination	10-Apr-07	end day	Normal	
11	112	Combination	24-Sep-07	month 6	Normal	
11	113	SSG	23-Mar-07	day 0	Normal	
11	113	SSG	10-Apr-07	day 14	Normal	
11	113	SSG	27-Apr-07	end day	Normal	
11	114	SSG	28-Mar-07	day 0	Normal	
11	114	SSG	12-Apr-07	day 14	Normal	
11	114	SSG	29-Apr-07	end day	Normal	
11	114	SSG	5-Oct-07	month 6	Normal	
11	115	Combination	30-Mar-07	day 0	Normal	
11	115	Combination	13-Apr-07	day 14	Normal	
11	115	Combination	14-Apr-07	end day	Normal	
11	115	Combination	21-Sep-07	month 6	Normal	
11	116	SSG	30-Mar-07	day 0	Normal	
11	116	SSG	13-Apr-07	day 14	Normal	
11	116	SSG	30-Apr-07	end day	Normal	
11	116	SSG	1-Nov-07	month 6	Normal	
11	117	PM	3-Apr-07	day 0	Normal	
11	117	PM	17-Apr-07	day 14	Normal	
11	117	PM	25-Apr-07	end day	Normal	
11	118	PM	18-Apr-07	day 0	Normal	
11	118	PM	2-May-07	day 14	Normal	
11	118	PM	10-May-07	end day	Normal	
11	118	PM	19-Sep-07	month 6	Normal	
11	119	SSG	20-Apr-07	day 0	Normal	
11	119	SSG	4-May-07	day 14	Normal	
11	119	SSG	21-May-07	end day	Normal	
11	119	SSG	29-Oct-07	month 6	Normal	
11	120	Combination	20-Apr-07	day 0	Normal	
11	120	Combination	4-May-07	day 14	Normal	
11	120	Combination	8-May-07	end day	Normal	
11	120	Combination	29-Oct-07	month 6	Normal	
11	121	Combination	20-Apr-07	day 0	Normal	
11	121	Combination	4-May-07	day 14	Normal	
11	121	Combination	8-May-07	end day	Normal	
11	121	Combination	29-Oct-07	month 6	Normal	
11	122	SSG	20-Apr-07	day 0	Normal	
11	122	SSG	4-May-07	day 14	Normal	
11	122	SSG	21-May-07	end day	Normal	
11	123	SSG	27-Apr-07	day 0	Normal	
11	123	SSG	11-May-07	day 14	Normal	
11	123	SSG	28-May-07	end day	Normal	
11	123	SSG	19-Oct-07	month 6	Normal	
11	124	SSG	20-May-07	day 0	Normal	
11	124	SSG	3-Jun-07	day 14	Normal	
11	124	SSG	20-Jun-07	end day	Normal	
11	124	SSG	7-Apr-08	month 6	Normal	
11	125	SSG	23-May-07	day 0	Normal	
11	125	SSG	6-Jun-07	day 14	Normal	
11	125	SSG	23-Jun-07	end day	Normal	
11	125	SSG	19-Nov-07	month 6	Normal	
11	126	Combination	26-May-07	day 0	Normal	
11	126	Combination	10-Jun-07	day 14	Normal	

Centre Number	Patient Number	Treatment	Audiometry Date	Visit	Audiometry Findings	Comment
11	126	Combination	14-Jun-07	end day	Normal	
11	126	Combination	10-Dec-07	month 6	Normal	
11	127	PM	26-May-07	day 0	Normal	
11	127	PM	10-Jun-07	day 14	Normal	
11	127	PM	18-Jun-07	end day	Normal	
11	127	PM	30-Oct-07	month 6	Normal	
11	128	Combination	1-Jun-07	day 0	Normal	
11	128	Combination	15-Jun-07	day 14	Normal	
11	128	Combination	19-Jun-07	end day	Normal	
11	128	Combination	16-Nov-07	month 6	Normal	
11	129	PM	6-Jun-07	day 0	Normal	
11	129	PM	20-Jun-07	day 14	Normal	
11	129	PM	28-Jun-07	end day	Normal	
11	129	PM	21-Nov-07	month 6	Normal	
11	130	Combination	7-Jun-07	day 0	Normal	
11	130	Combination	22-Jun-07	day 14	Normal	
11	130	Combination	26-Jun-07	end day	Normal	
11	130	Combination	19-Nov-07	month 6	Normal	
11	131	PM	10-Jun-07	day 0	Normal	
11	131	PM	24-Jun-07	day 14	Normal	
11	131	PM	2-Jul-07	end day	Normal	
11	131	PM	12-Dec-07	month 6	Normal	
11	132	SSG	15-Jun-07	day 0	Normal	
11	132	SSG	29-Jun-07	day 14	Normal	
11	132	SSG	16-Jul-07	end day	Normal	
11	132	SSG	19-Dec-07	month 6	Normal	
11	133	Combination	3-Jul-07	day 0	Normal	
11	133	Combination	17-Jul-07	day 14	Normal	
11	133	Combination	21-Jul-07	end day	Normal	
11	133	Combination	2-Jan-08	month 6	Normal	
11	134	PM	10-Jul-07	day 0	Normal	
11	134	PM	24-Jul-07	day 14	Normal	
11	134	PM	1-Aug-07	end day	Normal	
11	134	PM	8-Jan-08	month 6	Normal	
11	135	PM	12-Jul-07	day 0	Normal	
11	135	PM	26-Jul-07	day 14	Normal	
11	135	PM	3-Aug-07	end day	Normal	
11	135	PM	31-Dec-07	month 6	Normal	
12	241	Combination	26-Jun-05	day 0	Normal	48DB (MODERATE HEARING LOSS)
12	241	Combination	5-Jul-05	day 7	Normal	AVERAGE 52 DBHL
12	241	Combination	12-Jul-05	day 14	Normal	
12	241	Combination	16-Jul-05	end day	Normal	LT EAR - 55DB RT EAR - 56
12	241	Combination	9-Feb-06	month 6	Normal	
12	242	SSG	26-Jun-05	day 0	Not Done	
12	242	SSG	12-Jul-05	day 14	Not Done	
12	242	SSG	29-Jul-05	end day	Not Done	
12	242	SSG	24-Oct-05	month 3	Normal	
12	242	SSG	12-Feb-06	month 6	Normal	
12	243	PM	26-Jun-05	day 0	Normal	51DB (MODERATE HEARING LOSS)
12	243	PM	5-Jul-05	day 7	Normal	RIGHT EAR - 31DB LEFT EAR - 37.5DB (MILD HEARING LOSS)
12	243	PM	12-Jul-05	day 14	Normal	
12	243	PM	20-Jul-05	end day	Normal	
12	243	PM	6-Aug-06	month 6	Normal	

Centre Number	Patient Number	Treatment	Audiometry Date	Visit	Audiometry Findings	Comment
12	244	SSG	26-Jun-05	day 0	Normal	45DB (MODERATE HEARING LOSS)
12	244	SSG	5-Jul-05	day 7	Abnormal (CI)	HIS AVERAGE OF 1000, 2000, 4000 & 500HZ HAS DETERIORATED FROM MODERATE TO MODERATELY SEVERE (ONE STEP). BUT THE PATIENT DID NOT COMPLAIN OF HEARING CAPACITY. AVERAGE 58DBHL
12	244	SSG	12-Jul-05	day 14	Normal	
12	244	SSG	19-Jul-05	day 21	Normal	
12	244	SSG	29-Jul-05	end day	Normal	
12	244	SSG	29-Oct-05	month 3	Normal	
12	244	SSG	6-Apr-06	month 6	Normal	
12	245	PM	16-Jul-05	day 0	Normal	
12	245	PM	25-Jul-05	day 7	Normal	
12	245	PM	1-Aug-05	day 14	Normal	
12	245	PM	9-Aug-05	end day	Not Done	
12	245	PM	18-Nov-05	month 3	Normal	
12	246	PM		day 0	Not Done	
12	246	PM		day 14	Not Done	
12	246	PM		end day	Not Done	
12	246	PM	25-Nov-05	month 3	Normal	
12	247	PM	23-Sep-05	day 0	Normal	RT EAR 8.75 DBHL LT EAR 8.75 DBHL
12	247	PM	4-Oct-05	day 7	Normal	RT EAR 6.5 DBHL LT EAR 2.5 DBHL
12	247	PM	11-Oct-05	day 14	Normal	RT EAR 13.75 DBHL LT EAR 28(27.75) DBHL
12	247	PM	19-Oct-05	end day	Normal	AVERAGE RT EAR 15 DBHL LT EAR 10 DBHL
12	247	PM	18-Jan-06	month 3	Normal	R EAR HAS MILD TO MODERATE HEARING PROBLEM
12	247	PM	19-Apr-06	month 6	Normal	
12	248	PM	22-Sep-05	day 0	Normal	RT EAR 19 DBHL LT EAR 36 DBHL
12	248	PM	29-Sep-05	day 7	Normal	RT EAR 20 DBHL LT EAR 38.8 DBHL
12	248	PM	6-Oct-05	day 14	Normal	RT EAR 24 DBHL LT EAR 37.5 DBHL
12	248	PM	14-Oct-05	end day	Normal	RT EAR 16 DBHL LT EAR 41 DBHL
12	248	PM	23-Jan-06	month 3	Normal	LT EAR HAS MILD HEARING LOSS
12	248	PM	19-Apr-06	month 6	Normal	
12	249	Combination	24-Oct-05	day 0	Normal	RT EAR 4DBHL LT EAR 14DBHL
12	249	Combination	31-Oct-05	day 7	Normal	RT EAR 8.75 DBHL LT EAR 16.25 DBHL
12	249	Combination	7-Nov-05	day 14	Normal	RT EAR 10 DBHL LT EAR 17.5 DBHL
12	249	Combination		day 21	Normal	
12	249	Combination	11-Nov-05	end day	Normal	RT EAR 2.5 DBHL LT EAR 23.33DBHL
12	249	Combination	4-Mar-06	month 3	Normal	
12	249	Combination	13-May-06	month 6	Normal	
12	250	SSG	24-Oct-05	day 0	Normal	RT EAR 16.25DBHZ LT EAR 18.75 DBHZ
12	250	SSG	31-Oct-05	day 7	Normal	LT EAR 13.75 DBHZ LT EAR 13.75 DBHZ
12	250	SSG	7-Nov-05	day 14	Normal	RT EAR 18.75 DBHZ LT EAR 15. DBHZ
12	250	SSG	14-Nov-05	day 21	Normal	RT EAR 16.25 DBHL LT EAR 18.75 DBHL
12	250	SSG	24-Nov-05	end day	Normal	RT EAR 12.5 DBHL (NORM) LT EAR 36.25 DBHZ (MODERATE)
12	250	SSG	22-Feb-06	month 3	Normal	
12	250	SSG	8-Jul-06	month 6	Normal	
12	251	Combination	27-Oct-05	day 0	Normal	RT EAR - 250 -45DB 8000 - 50DB LEFT EAR - 250 - 30 8000 - 65DB
12	251	Combination	11-Nov-05	day 14	Not Done	

Centre Number	Patient Number	Treatment	Audiometry Date	Visit	Audiometry Findings	Comment
12	251	Combination	15-Nov-05	end day	Normal	MILD HEARING LOSS ON BOTH RIGHT AND LEFT EAR AT 8000HZ, WHICH IS 35DB
12	251	Combination	12-Feb-06	month 3	Normal	
12	251	Combination	14-May-06	month 6	Normal	
12	252	SSG	27-Oct-05	day 0	Normal	AVERAGE RT 13.33 DBHZ LT 15 DBHZ
12	252	SSG	11-Nov-05	day 14	Normal	RT EAR 16.66 DBHZ LT EAR 9.16 DBHZ
12	252	SSG	18-Nov-05	day 21	Normal	RT EAR 8.33DBHL LT EAR 10 DBHL
12	252	SSG	28-Nov-05	end day	Normal	RT EAR 11.66 DBHL LT EAR 0 DBHL
12	252	SSG	6-Apr-06	month 3	Normal	
12	252	SSG	22-Jun-06	month 6	Normal	
12	253	SSG	27-Oct-05	day 0	Normal	
12	253	SSG	11-Nov-05	day 14	Normal	RT EAR 19.16 DBHL LT EAR 10.83 DBHL
12	253	SSG	18-Nov-05	day 21	Normal	RT EAR 25DBHL LT EAR 21.66 DBHL
12	253	SSG	28-Nov-05	end day	Normal	RT EAR 16.66 DBHL LT EAR 10 DBHL
12	253	SSG	6-Apr-06	month 3	Normal	
12	253	SSG	22-Jun-06	month 6	Normal	
12	254	Combination		day 0	Not Done	
12	254	Combination	11-Nov-05	day 14	Normal	RT EAR 14.16 DBHL LT EAR 11.66 DBHL
12	254	Combination	15-Nov-05	end day	Normal	RT EAR 10 DBHL LT EAR 15.83 DBHL
12	254	Combination	12-Feb-06	month 3	Normal	
12	254	Combination	22-May-06	month 6	Normal	
12	255	Combination	30-Oct-05	day 0	Normal	RT EAR 13.75 DBHL. LT EAR 11.25 DBHL
12	255	Combination	8-Nov-05	day 7	Normal	RT EAR 7.5 DBHL LT EAR 4DBHL
12	255	Combination	14-Nov-05	day 14	Normal	RT EAR 6.25 DBHL LT EAR 7.5DBHL
12	255	Combination	18-Nov-05	end day	Normal	RT EAR 6.66 DBHL LT EAR 10 DBHL
12	255	Combination	9-Mar-06	month 3	Normal	
12	255	Combination	19-May-06	month 6	Normal	
12	256	Combination	14-Nov-05	day 0	Normal	LT MILD *RT MODERATELY SEVERE
12	256	Combination	23-Nov-05	day 7	Normal	LT EAR 30 DBHL (MILD) RT EAR 61.25 DBHL (MODERATELY SEVERE)
12	256	Combination	30-Nov-05	day 14	Normal	LT EAR 30 DBHL RT EAR 58.75 DBHL
12	256	Combination	5-Dec-05	end day	Normal	RT EAR 61.25 DBHL (MODERATELY SEVER) LT EAR 32.5 DBHL (MILD)
12	256	Combination	4-Mar-06	month 3	Normal	
12	256	Combination	6-Jun-06	month 6	Normal	
12	257	SSG	14-Nov-05	day 0	Normal	
12	257	SSG	23-Nov-05	day 7	Normal	
12	257	SSG	30-Nov-05	day 14	Normal	
12	257	SSG	7-Dec-05	day 21	Normal	
12	257	SSG	17-Dec-05	end day	Normal	
12	257	SSG	19-Apr-06	month 3	Normal	
12	257	SSG	14-Jun-06	month 6	Normal	
12	258	SSG	30-Nov-05	day 0	Normal	
12	258	SSG	12-Dec-05	day 7	Normal	
12	258	SSG	19-Dec-05	day 14	Normal	
12	258	SSG	26-Dec-05	day 21	Normal	
12	258	SSG	5-Jan-06	end day	Normal	
12	258	SSG	12-Jul-06	month 6	Normal	
12	259	PM	25-Dec-05	day 0	Normal	
12	259	PM	6-Jan-06	day 7	Normal	
12	259	PM	13-Jan-06	day 14	Normal	
12	259	PM	20-Jan-06	end day	Normal	
12	259	PM	8-May-06	month 3	Normal	

Centre Number	Patient Number	Treatment	Audiometry Date	Visit	Audiometry Findings	Comment
12	259	PM	28-Jul-06	month 6	Normal	
12	260	PM	25-Dec-05	day 0	Normal	
12	260	PM	9-Jan-06	day 7	Normal	
12	260	PM	16-Jan-06	day 14	Normal	
12	260	PM	24-Jan-06	end day	Normal	
12	260	PM	11-Jul-06	month 6	Normal	
12	261	Combination	25-Dec-05	day 0	Normal	LT EAR NORMAL R HAS MODERATE & MODERATELY - SEVERE HEARING LOSS
12	261	Combination	9-Jan-06	day 7	Normal	LT EAR NORM R EAR MODERATE & MODERATELY SEVER HEARING LOSS
12	261	Combination	16-Jan-06	day 14	Normal	LT EAR NORMAL HEARING R EAR MODERATE & MODERATELY SEVERE HEARING LOSS
12	261	Combination	20-Jan-06	end day	Normal	LT EAR NORMAL RT EAR HAS MODERATE & MODERATELY SEVERE HEARING LOSS
12	261	Combination	19-Apr-06	month 3	Normal	THE RIGHT SIDE HEARING ABNORMALITY WHICH INITIALLY WAS MODERATE HEARING LOSS NOW HAS BECOME IN THE CATEGORY OF SEVERE HEARING IN ALL FREQUENCIES EXCEPT 250 HZ
12	261	Combination	22-Jul-06	month 6	Abnormal (CI)	HAS SEVERE HEARING LOSS ON THE RT EAR EXCEPT FOR 250 HZ WHICH IS MODERATELY SEVERE - LEFT EAR IS NORMAL
12	262	PM	25-Dec-05	day 0	Normal	
12	262	PM	9-Jan-06	day 7	Normal	
12	262	PM	16-Jan-06	day 14	Normal	
12	262	PM	23-Jan-06	end day	Normal	
12	262	PM	22-Jun-06	month 3	Normal	
12	262	PM	28-Jul-06	month 6	Normal	
12	263	SSG	22-Jan-06	day 0	Normal	
12	263	SSG	1-Feb-06	day 7	Normal	
12	263	SSG	9-Feb-06	day 14	Normal	
12	263	SSG	16-Feb-06	day 21	Normal	
12	263	SSG	25-Feb-06	end day	Normal	
12	263	SSG	29-May-06	month 3	Normal	
12	263	SSG	26-Aug-06	month 6	Normal	
12	264	Combination	12-Feb-06	day 0	Normal	
12	264	Combination	21-Feb-06	day 7	Normal	
12	264	Combination	28-Feb-06	day 14	Normal	
12	264	Combination	4-Mar-06	end day	Normal	
12	264	Combination	16-Jun-06	month 3	Normal	R EAR NORMAL LT EAR MODERATES HEARING LOSS BUT NO CLINICAL
12	264	Combination	1-Sep-06	month 6	Normal	
12	265	SSG	12-Feb-06	day 0	Normal	
12	265	SSG	21-Feb-06	day 7	Normal	
12	265	SSG	28-Feb-06	day 14	Normal	
12	266	PM	12-Feb-06	day 0	Normal	
12	266	PM	21-Feb-06	day 7	Normal	
12	266	PM	28-Feb-06	day 14	Normal	
12	266	PM	8-Mar-06	end day	Normal	
12	266	PM	5-Jun-06	month 3	Normal	

Centre Number	Patient Number	Treatment	Audiometry Date	Visit	Audiometry Findings	Comment
12	266	PM	3-Oct-06	month 6	Normal	
12	267	SSG	8-Feb-06	day 0	Normal	
12	267	SSG	21-Feb-06	day 7	Normal	
12	267	SSG	28-Feb-06	day 14	Normal	
12	267	SSG	7-Mar-06	day 21	Normal	
12	267	SSG	17-Mar-06	end day	Normal	
12	267	SSG	14-Jun-06	month 3	Normal	
12	267	SSG	15-Sep-06	month 6	Normal	
12	268	PM	7-Mar-06	day 0	Normal	
12	268	PM	17-Mar-06	day 7	Normal	
12	268	PM	23-Mar-06	day 14	Normal	
12	268	PM	31-Mar-06	end day	Normal	
12	268	PM	29-Jun-06	month 3	Normal	
12	268	PM	26-Sep-06	month 6	Normal	
12	269	Combination	13-Mar-06	day 0	Normal	
12	269	Combination	22-Mar-06	day 7	Normal	
12	269	Combination	29-Mar-06	day 14	Normal	
12	269	Combination	3-Apr-06	end day	Normal	
12	269	Combination	8-Jul-06	month 3	Normal	
12	269	Combination	14-Nov-06	month 6	Normal	
12	270	Combination		day 0	Not Done	
12	270	Combination		day 14	Not Done	
12	270	Combination		end day	Not Done	
12	270	Combination	10-Oct-06	month 6	Normal	
12	271	SSG	30-Mar-06	day 0	Normal	
12	271	SSG	5-Apr-06	day 7	Normal	
12	271	SSG	13-Apr-06	day 14	Normal	
12	271	SSG	21-Apr-06	day 21	Normal	
12	271	SSG	30-Apr-06	end day	Normal	
12	271	SSG	1-Aug-06	month 3	Normal	
12	271	SSG	31-Oct-06	month 6	Normal	
12	272	PM		day 0	Not Done	
12	272	PM	21-Apr-06	day 14	Not Done	
12	272	PM		end day	Not Done	
12	273	SSG	8-Apr-06	day 0	Normal	
12	273	SSG	14-Apr-06	day 7	Normal	
12	273	SSG	21-Apr-06	day 14	Normal	
12	273	SSG	28-Apr-06	day 21	Normal	
12	273	SSG	8-May-06	end day	Normal	
12	273	SSG	8-Aug-06	month 3	Normal	
12	273	SSG	11-Nov-06	month 6	Normal	
12	274	SSG	8-Apr-06	day 0	Normal	
12	274	SSG	14-Apr-06	day 7	Normal	
12	274	SSG	21-Apr-06	day 14	Normal	
12	274	SSG	28-Apr-06	day 21	Normal	
12	274	SSG	8-May-06	end day	Normal	
12	274	SSG	8-Aug-06	month 3	Normal	
12	274	SSG	11-Nov-06	month 6	Normal	
12	275	Combination	8-Apr-06	day 0	Normal	
12	275	Combination	14-Apr-06	day 7	Normal	
12	275	Combination	21-Apr-06	day 14	Normal	
12	275	Combination	25-Apr-06	end day	Normal	
12	275	Combination	25-Jul-06	month 3	Normal	
12	275	Combination	25-Oct-06	month 6	Normal	

Centre Number	Patient Number	Treatment	Audiometry Date	Visit	Audiometry Findings	Comment
12	276	PM		day 0	Not Done	
12	276	PM		day 14	Not Done	
12	276	PM		end day	Not Done	
12	277	Combination	20-Apr-06	day 0	Normal	
12	277	Combination	27-Apr-06	day 7	Normal	
12	277	Combination	4-May-06	day 14	Normal	
12	277	Combination	8-May-06	end day	Normal	
12	277	Combination	7-Aug-06	month 3	Normal	
12	277	Combination	11-Nov-06	month 6	Normal	
12	278	SSG	21-Apr-06	day 0	Normal	
12	278	SSG	27-Apr-06	day 7	Normal	
12	278	SSG	4-May-06	day 14	Normal	
12	278	SSG	11-May-06	day 21	Normal	
12	278	SSG	21-May-06	end day	Normal	
12	278	SSG	18-Aug-06	month 3	Normal	
12	279	PM	21-Apr-06	day 0	Normal	
12	279	PM	27-Apr-06	day 7	Normal	
12	279	PM	4-May-06	day 14	Normal	
12	279	PM	12-May-06	end day	Normal	
12	279	PM	12-Aug-06	month 3	Normal	
12	279	PM	11-Nov-06	month 6	Normal	
12	280	SSG		day 0	Not Done	
12	280	SSG		day 14	Not Done	
12	280	SSG		end day	Not Done	
12	281	Combination	20-Apr-06	day 0	Normal	
12	281	Combination	27-Apr-06	day 7	Normal	
12	281	Combination	4-May-06	day 14	Normal	
12	281	Combination	8-May-06	end day	Normal	
12	281	Combination	8-Aug-06	month 3	Normal	
12	281	Combination	22-Dec-06	month 6	Normal	
12	282	PM	28-Apr-06	day 0	Normal	
12	282	PM	4-May-06	day 7	Normal	
12	282	PM	11-May-06	day 14	Normal	
12	282	PM	19-May-06	end day	Normal	
12	282	PM	26-Aug-06	month 3	Normal	
12	282	PM	20-Dec-06	month 6	Normal	
12	283	PM	11-May-06	day 0	Normal	
12	283	PM	17-May-06	day 7	Normal	
12	283	PM	24-May-06	day 14	Normal	
12	283	PM	1-Jun-06	end day	Normal	
12	283	PM	26-Aug-06	month 3	Normal	
12	283	PM	29-Dec-06	month 6	Normal	
12	284	Combination	19-May-06	day 0	Normal	
12	284	Combination	27-May-06	day 7	Normal	
12	284	Combination	2-Jun-06	day 14	Normal	
12	284	Combination	6-Jun-06	end day	Normal	
12	284	Combination	5-Sep-06	month 3	Normal	
12	284	Combination	9-Dec-06	month 6	Normal	
12	285	Combination	19-May-06	day 0	Normal	
12	285	Combination	26-May-06	day 7	Normal	
12	285	Combination	2-Jun-06	day 14	Normal	
12	285	Combination	6-Jun-06	end day	Normal	
12	285	Combination	5-Sep-06	month 3	Normal	
12	285	Combination	6-Dec-06	month 6	Normal	

Centre Number	Patient Number	Treatment	Audiometry Date	Visit	Audiometry Findings	Comment
12	286	PM	28-Dec-06	day 0	Normal	
12	286	PM	15-Jan-07	day 14	Normal	
12	286	PM	24-Jan-07	end day	Normal	
12	286	PM	4-Sep-07	month 6	Normal	
12	287	SSG	28-Dec-06	day 0	Normal	
12	287	SSG	15-Jan-07	day 14	Normal	
12	287	SSG	1-Feb-07	end day	Normal	
12	287	SSG	7-Aug-07	month 6	Normal	
12	288	Combination	30-Dec-06	day 0	Normal	
12	288	Combination	15-Jan-07	day 14	Normal	
12	288	Combination	19-Jan-07	end day	Normal	
12	288	Combination	24-Jul-07	month 6	Normal	
12	289	Combination	30-Dec-06	day 0	Normal	
12	289	Combination	15-Jan-07	day 14	Normal	
12	289	Combination	19-Jan-07	end day	Normal	
12	289	Combination	2-Sep-07	month 6	Normal	
12	290	SSG	5-Jan-07	day 0	Normal	
12	290	SSG	19-Jan-07	day 14	Normal	
12	290	SSG	5-Feb-07	end day	Normal	
12	290	SSG	6-Sep-07	month 6	Normal	
12	291	SSG	8-Jan-07	day 0	Normal	-LEFT EAR AT 8KHZ IS 30DB
12	291	SSG	24-Jan-07	day 14	Normal	
12	291	SSG	10-Feb-07	end day	Normal	35 DB - AT 8K DB
12	291	SSG	4-Sep-07	month 6	Normal	
12	292	PM	14-Jan-07	day 0	Normal	
12	292	PM	31-Jan-07	day 14	Normal	
12	292	PM	7-Feb-07	end day	Normal	
12	292	PM	22-Aug-07	month 6	Normal	
12	293	SSG	21-Jan-07	day 0	Normal	
12	293	SSG	6-Feb-07	day 14	Normal	
12	293	SSG	23-Feb-07	end day	Normal	
12	293	SSG	6-Sep-07	month 6	Normal	
12	294	Combination	21-Feb-07	day 0	Normal	
12	294	Combination	7-Mar-07	day 14	Normal	
12	294	Combination	12-Mar-07	end day	Normal	
12	294	Combination	11-Jun-07	month 3	Normal	
12	294	Combination	11-Sep-07	month 6	Normal	
12	295	Combination	12-Mar-07	day 0	Normal	
12	295	Combination	29-Mar-07	day 14	Normal	
12	295	Combination	2-Apr-07	end day	Normal	
12	295	Combination	20-Sep-07	month 6	Normal	
12	296	PM	10-Apr-07	day 0	Normal	
12	296	PM	25-Apr-07	day 14	Normal	
12	296	PM	3-May-07	end day	Normal	
12	296	PM	1-Aug-07	month 3	Normal	
12	296	PM	30-Oct-07	month 6	Normal	
12	297	SSG	10-Apr-07	day 0	Normal	
12	297	SSG	25-Apr-07	day 14	Normal	
12	298	PM	18-Apr-07	day 0	Normal	
12	298	PM	4-May-07	day 14	Normal	
12	298	PM	12-May-07	end day	Normal	
12	298	PM	9-Nov-07	month 6	Normal	
12	299	Combination	3-Jun-07	day 0	Normal	
12	299	Combination	18-Jun-07	day 14	Normal	

Centre Number	Patient Number	Treatment	Audiometry Date	Visit	Audiometry Findings	Comment
12	299	Combination	22-Jun-07	end day	Normal	
12	299	Combination	1-Jan-08	month 6	Normal	
12	300	PM	3-Jun-07	day 0	Normal	
12	300	PM	18-Jun-07	day 14	Normal	
12	300	PM	26-Jun-07	end day	Normal	
12	300	PM	24-Dec-07	month 6	Normal	
12	301	SSG	3-Jun-07	day 0	Normal	
12	301	SSG	18-Jun-07	day 14	Normal	
12	301	SSG	5-Jul-07	end day	Normal	
12	301	SSG	4-Feb-08	month 6	Normal	
12	302	SSG		day 0	Not Done	
12	302	SSG		day 14	Not Done	
12	302	SSG	5-Jul-07	end day	Not Done	
12	303	PM	3-Jun-07	day 0	Normal	
12	303	PM	18-Jun-07	day 14	Normal	
12	303	PM	26-Jun-07	end day	Normal	
12	303	PM	26-Dec-07	month 6	Normal	
12	304	Combination	3-Jun-07	day 0	Normal	
12	304	Combination	18-Jun-07	day 14	Normal	
12	304	Combination	22-Jun-07	end day	Normal	
12	304	Combination	26-Dec-07	month 6	Normal	
12	305	PM		day 0	Not Done	
12	305	PM		day 14	Not Done	
12	305	PM		end day	Not Done	
12	306	Combination		day 0	Not Done	
12	306	Combination		day 14	Not Done	
12	306	Combination		end day	Not Done	
12	307	Combination	14-Jun-07	day 0	Normal	
12	307	Combination	28-Jun-07	day 14	Normal	
12	307	Combination	2-Jul-07	end day	Normal	
12	307	Combination	26-Dec-07	month 6	Normal	
12	308	PM	14-Jun-07	day 0	Normal	
12	308	PM	28-Jun-07	day 14	Normal	
12	308	PM	6-Jul-07	end day	Normal	
12	308	PM	26-Dec-07	month 6	Normal	
12	309	PM	16-Jun-07	day 0	Normal	
12	309	PM	29-Jun-07	day 14	Normal	
12	309	PM	7-Jul-07	end day	Normal	
12	309	PM	26-Dec-07	month 6	Normal	
12	310	SSG	16-Jun-07	day 0	Normal	
12	310	SSG	29-Jun-07	day 14	Normal	
12	310	SSG	17-Jul-07	end day	Normal	
12	310	SSG	4-Feb-08	month 6	Normal	
12	311	SSG	4-Jul-07	day 0	Normal	
12	311	SSG	18-Jul-07	day 14	Normal	
12	311	SSG	4-Aug-07	end day	Normal	
12	311	SSG	11-Mar-08	month 6	Normal	
12	312	Combination	11-Jul-07	day 0	Normal	
12	312	Combination	25-Jul-07	day 14	Normal	
12	312	Combination	29-Jul-07	end day	Normal	
12	312	Combination	19-Mar-08	month 6	Normal	
12	313	PM	1-Aug-07	day 0	Normal	
12	313	PM	15-Aug-07	day 14	Normal	
12	313	PM	23-Aug-07	end day	Normal	

Centre Number	Patient Number	Treatment	Audiometry Date	Visit	Audiometry Findings	Comment
12	313	PM	21-Feb-08	month 6	Normal	
12	314	SSG	1-Aug-07	day 0	Normal	
12	314	SSG	21-Aug-07	day 14	Normal	
12	314	SSG	6-Sep-07	end day	Normal	
12	314	SSG	4-Mar-08	month 6	Normal	
12	315	Combination	21-Aug-07	day 0	Normal	
12	315	Combination	11-Sep-07	day 14	Normal	
12	315	Combination	15-Sep-07	end day	Normal	
12	315	Combination	11-Aug-08	month 6	Normal	
12	316	SSG	21-Aug-07	day 0	Normal	
12	316	SSG	11-Sep-07	day 14	Normal	
12	316	SSG	2-Apr-08	month 6	Normal	
12	317	SSG	21-Aug-09	day 0	Not Done	PATIENT IS NOT COOPERATIVE -HAS NO CLINICALLY EVIDENT HEARING PROBLEM
12	317	SSG		day 14	Not Done	
12	317	SSG	29-Sep-07	end day	Normal	
12	318	Combination	28-Aug-07	day 0	Normal	
12	318	Combination	11-Sep-07	day 14	Normal	
12	318	Combination	15-Sep-07	end day	Normal	
12	318	Combination	15-Mar-08	month 6	Normal	
12	319	Combination		day 0	Normal	
12	319	Combination	11-Sep-07	day 14	Normal	
12	319	Combination	15-Sep-07	end day	Normal	
12	319	Combination	15-Mar-08	month 6	Normal	
12	320	Combination	5-Sep-07	day 0	Normal	
12	320	Combination	19-Sep-07	day 14	Normal	
12	320	Combination	23-Sep-07	end day	Normal	
12	320	Combination	19-Mar-08	month 6	Normal	
12	321	Combination	5-Sep-07	day 0	Normal	
12	321	Combination	19-Sep-07	day 14	Normal	
12	321	Combination	23-Sep-07	end day	Normal	
12	321	Combination	24-Mar-08	month 6	Normal	
12	322	PM	11-Sep-07	day 0	Normal	
12	322	PM	26-Sep-07	day 14	Normal	
12	322	PM	4-Oct-07	end day	Normal	
12	322	PM	20-Apr-08	month 6	Normal	
12	323	SSG	19-Sep-07	day 0	Normal	
12	323	SSG	3-Oct-07	day 14	Normal	
12	323	SSG	20-Oct-07	end day	Normal	
12	323	SSG	17-Apr-08	month 6	Normal	
12	324	SSG	19-Sep-07	day 0	Normal	
12	324	SSG	3-Oct-07	day 14	Normal	
12	324	SSG	20-Oct-07	end day	Normal	
12	324	SSG	17-Apr-08	month 6	Normal	
12	325	PM	19-Sep-07	day 0	Normal	
12	325	PM	10-Oct-07	day 14	Normal	
12	325	PM	18-Oct-07	end day	Normal	
12	325	PM	17-Apr-08	month 6	Normal	
12	326	PM	2-Oct-07	day 0	Normal	
12	326	PM	16-Oct-07	day 14	Normal	-HAS MILD HEARING LOSS LEVEL ON AUDIOMETRY FOR HE DEVELOPED OTITIS MEDIA 03 DAYS AGO.

Centre Number	Patient Number	Treatment	Audiometry Date	Visit	Audiometry Findings	Comment
12	326	PM	24-Oct-07	end day	Normal	
12	326	PM	20-Apr-08	month 6	Normal	
12	327	Combination	2-Oct-07	day 0	Normal	
12	327	Combination	16-Oct-07	day 14	Normal	
12	327	Combination	20-Oct-07	end day	Normal	
12	327	Combination	17-Apr-08	month 6	Normal	
12	328	PM	3-Oct-07	day 0	Normal	
12	328	PM		day 14	Not Done	
12	328	PM	26-Oct-07	end day	Normal	
12	328	PM	14-Apr-08	month 6	Normal	
12	329	SSG	9-Oct-07	day 0	Normal	
12	329	SSG	23-Oct-07	day 14	Normal	-HAS AUDIOMETRIC SHIFT TO MILD HEARING LEVEL FOR HE DEVELOPED OTITIS MEDIA 04 DAYS AGO
12	329	SSG	30-Oct-07	day 21	Normal	
12	329	SSG	9-Nov-07	end day	Normal	
12	329	SSG	5-May-08	month 6	Normal	
12	330	PM	9-Oct-07	day 0	Normal	
12	330	PM	23-Oct-07	day 14	Normal	
12	330	PM	31-Oct-07	end day	Normal	
12	330	PM	5-May-08	month 6	Normal	
23	361	Combination	18-Jan-05	day 0	Abnormal (CI)	LEFT EAR MILD HEARING LOSS FOR LOW FREQUENCIES
23	361	Combination	26-Jan-05	day 7	Normal	
23	361	Combination	2-Feb-05	day 14	Normal	
23	361	Combination	6-Feb-05	end day	Normal	
23	361	Combination	10-Aug-05	month 6	Normal	
23	362	PM	19-Jan-05	day 0	Normal	
23	362	PM	26-Jan-05	day 7	Normal	
23	362	PM	2-Feb-05	day 14	Normal	
23	362	PM	10-Feb-05	end day	Normal	
23	362	PM	10-May-05	month 3	Normal	
23	362	PM	16-Aug-05	month 6	Normal	
23	363	PM	18-Jan-05	day 0	Normal	
23	363	PM	26-Jan-05	day 7	Normal	
23	363	PM	2-Feb-05	day 14	Normal	
23	363	PM	10-Feb-05	end day	Normal	
23	363	PM	10-Aug-05	month 6	Normal	
23	364	Combination	18-Jan-05	day 0	Normal	
23	364	Combination	26-Jan-05	day 7	Normal	
23	364	Combination	2-Feb-05	day 14	Normal	
23	364	Combination	6-Feb-05	end day	Normal	
23	364	Combination	11-May-05	month 3	Normal	
23	364	Combination	19-Aug-05	month 6	Normal	
23	365	SSG	18-Jan-05	day 0	Normal	
23	365	SSG	26-Jan-05	day 7	Normal	
23	365	SSG	2-Feb-05	day 14	Normal	
23	365	SSG	10-Feb-05	day 21	Normal	
23	365	SSG	20-Feb-05	end day	Normal	
23	365	SSG	20-May-05	month 3	Abnormal (CI)	MODERATE HEARING LOSS RT EAR AT 4000HZ & 8000HZ MILD HEARING LOSS LT EAR AT 8000HZ RT - TYMPANIC MEMBRANE - MILD INFLAMMATION
23	365	SSG	30-Aug-05	month 6	Normal	

Centre Number	Patient Number	Treatment	Audiometry Date	Visit	Audiometry Findings	Comment
23	366	Combination	28-Jan-05	day 0	Normal	
23	366	Combination	7-Feb-05	day 7	Normal	
23	366	Combination	14-Feb-05	day 14	Normal	
23	366	Combination	18-Feb-05	end day	Normal	
23	366	Combination	20-May-05	month 3	Normal	
23	366	Combination	20-Aug-05	month 6	Normal	
23	367	SSG	28-Jan-05	day 0	Normal	
23	367	SSG	7-Feb-05	day 7	Normal	
23	367	SSG	14-Feb-05	day 14	Normal	
23	367	SSG	21-Feb-05	day 21	Normal	
23	367	SSG	3-Mar-05	end day	Normal	
23	367	SSG	8-Jun-05	month 3	Normal	
23	367	SSG	23-Sep-05	month 6	Normal	
23	368	PM	28-Jan-05	day 0	Normal	
23	368	PM	7-Feb-05	day 7	Normal	
23	368	PM	17-Feb-05	day 14	Abnormal (CI)	MILD HEARING LOSS - RT EAR CAUSED BY A PERFORATED RT TYMPANIC MEMBRANE
23	368	PM	23-Feb-05	end day	Abnormal (CI)	MILD HEARING LOSS RT EAR HAS A RUPTURED TYMPANIC MEMBRANE
23	368	PM	26-May-05	month 3	Abnormal (CI)	MILD HEARING LOSS LT EAR AT 250HZ, 500HZ 1000HZ, 2000HZ AND 4000HZ MILD HEARING LOSS RT EAR - HAS PERFORATED EARDRUM (RT) 20 TO CHRONIC OTITIS MEDIA RT EAR, MILD HEARING LOSS AT ALL FREQUENCIES. (250HZ, 500HZ, 1000HZ, 2000HZ, 4000HZ AND 8000HZ)
23	368	PM	6-Sep-05	month 6	Abnormal (CI)	MILD TO MODERATE HEARING LOSS RT EAR AT 250HZ, 500HZ, AND 1000HZ DUE TO RUPTURED RT TYMPANIC MEMBRANE
23	369	Combination	28-Jan-05	day 0	Normal	
23	369	Combination	10-Feb-05	day 7	Normal	
23	369	Combination	17-Feb-05	day 14	Normal	
23	369	Combination	21-Feb-05	end day	Normal	
23	369	Combination	26-May-05	month 3	Normal	
23	369	Combination	30-Aug-05	month 6	Normal	
23	370	SSG	28-Jan-05	day 0	Normal	
23	370	SSG	10-Feb-05	day 7	Normal	
23	370	SSG	17-Feb-05	day 14	Normal	
23	370	SSG	24-Feb-05	day 21	Normal	
23	370	SSG	6-Mar-05	end day	Normal	
23	370	SSG	8-Jun-05	month 3	Normal	
23	370	SSG	23-Sep-05	month 6	Normal	
23	371	PM	4-Feb-05	day 0	Normal	
23	371	PM	10-Feb-05	day 7	Normal	MILD HEARING LOSS (30DB)
23	371	PM	17-Feb-05	day 14	Normal	
23	371	PM	25-Feb-05	end day	Normal	
23	371	PM	26-May-05	month 3	Normal	
23	371	PM	6-Sep-05	month 6	Normal	
23	372	PM	10-Feb-05	day 0	Normal	
23	372	PM	17-Feb-05	day 7	Normal	
23	372	PM	24-Feb-05	day 14	Normal	

Centre Number	Patient Number	Treatment	Audiometry Date	Visit	Audiometry Findings	Comment
23	372	PM	4-Mar-05	end day	Normal	
23	372	PM	5-Jun-05	month 3	Normal	
23	372	PM	8-Sep-05	month 6	Normal	
23	373	SSG	17-Feb-05	day 0	Normal	
23	373	SSG	24-Feb-05	day 7	Normal	
23	373	SSG	3-Mar-05	day 14	Normal	
23	373	SSG	10-Mar-05	day 21	Normal	
23	373	SSG	20-Mar-05	end day	Normal	
23	373	SSG	6-Jul-05	month 3	Normal	
23	373	SSG	24-Oct-05	month 6	Normal	
23	374	SSG	18-Feb-05	day 0	Normal	
23	374	SSG	2-Mar-05	day 7	Normal	
23	374	SSG	9-Mar-05	day 14	Normal	
23	374	SSG	16-Mar-05	day 21	Normal	
23	374	SSG	28-Mar-05	end day	Normal	
23	374	SSG	27-Jun-05	month 3	Normal	
23	374	SSG	30-Sep-05	month 6	Normal	
23	375	Combination	25-Feb-05	day 0	Normal	
23	375	Combination	3-Mar-05	day 7	Normal	
23	375	Combination	10-Mar-05	day 14	Normal	
23	375	Combination	14-Mar-05	end day	Normal	
23	375	Combination	15-Jun-05	month 3	Normal	
23	375	Combination	23-Sep-05	month 6	Normal	
23	376	SSG	1-Mar-05	day 0	Normal	
23	376	SSG	7-Mar-05	day 7	Normal	
23	376	SSG	14-Mar-05	day 14	Normal	
23	376	SSG	21-Mar-05	day 21	Normal	
23	376	SSG	31-Mar-05	end day	Normal	
23	376	SSG	29-Jun-05	month 3	Normal	
23	376	SSG	28-Sep-05	month 6	Normal	
23	377	Combination	9-Mar-05	day 0	Normal	
23	377	Combination	15-Mar-05	day 7	Normal	
23	377	Combination	23-Sep-05	month 6	Normal	
23	378	Combination	9-Mar-05	day 0	Normal	
23	378	Combination	15-Mar-05	day 7	Normal	
23	378	Combination	22-Mar-05	day 14	Normal	
23	378	Combination	26-Mar-05	end day	Normal	
23	378	Combination	6-Jul-05	month 3	Normal	
23	378	Combination	23-Sep-05	month 6	Normal	
23	379	PM	10-Mar-05	day 0	Normal	
23	379	PM	16-Mar-05	day 7	Normal	
23	379	PM	23-Mar-05	day 14	Normal	
23	379	PM	31-Mar-05	end day	Normal	
23	379	PM	29-Jun-05	month 3	Normal	
23	380	SSG	15-Mar-05	day 0	Normal	
23	380	SSG	21-Mar-05	day 7	Normal	
23	380	SSG	28-Mar-05	day 14	Abnormal (CI)	MILD HEARING LOSS - BIL RT EAR 250 AND 500HZ LT EAR 500HZ
23	380	SSG	4-Apr-05	day 21	Normal	MILD LT EAR HEARING LOSS AT 500HZ
23	380	SSG	14-Apr-05	end day	Normal	
23	380	SSG	19-Jul-05	month 3	Normal	
23	380	SSG	12-Oct-05	month 6	Normal	
23	381	PM	15-Mar-05	day 0	Normal	
23	381	PM	21-Mar-05	day 7	Normal	

Centre Number	Patient Number	Treatment	Audiometry Date	Visit	Audiometry Findings	Comment
23	381	PM		day 14	Not Done	
23	381	PM	5-Apr-05	end day	Normal	
23	381	PM	12-Jul-05	month 3	Normal	
23	381	PM	17-Nov-05	month 6	Normal	
23	382	Combination	18-Mar-05	day 0	Normal	
23	382	Combination	24-Mar-05	day 7	Normal	
23	382	Combination	31-Mar-05	day 14	Normal	
23	382	Combination	4-Apr-05	end day	Normal	
23	382	Combination	12-Jul-05	month 3	Normal	
23	382	Combination	30-Sep-05	month 6	Normal	
23	383	PM	4-Apr-05	day 0	Normal	
23	383	PM	11-Apr-05	day 7	Normal	
23	383	PM	18-Apr-05	day 14	Abnormal (CI)	MILD RT HEARING LOSS AT 250HZ, 500HZ AND 4000HZ
23	383	PM	26-Apr-05	end day	Abnormal (CI)	MILD RT HEARING LOSS AT 4000HZ AND 8000HZ
23	383	PM	30-Sep-05	month 3	Normal	
23	383	PM	24-Oct-05	month 6	Normal	
23	384	SSG	30-Mar-05	day 0	Normal	
23	384	SSG	11-Apr-05	day 7	Normal	
23	384	SSG	18-Apr-05	day 14	Normal	
23	384	SSG	25-Apr-05	day 21	Normal	
23	384	SSG	5-May-05	end day	Normal	
23	384	SSG	28-Sep-05	month 3	Normal	
23	384	SSG	17-Nov-05	month 6	Normal	
23	385	SSG	30-Mar-05	day 0	Normal	
23	385	SSG	13-Apr-05	day 7	Abnormal (CI)	MILD HEARING AT 250HZ AND 500HZ IN THE RIGHT EAR IN LEFT EAR MILD HEARING LOSS AT 1000HZ
23	385	SSG	20-Apr-05	day 14	Normal	
23	385	SSG	27-Apr-05	day 21	Normal	
23	385	SSG	7-May-05	end day	Normal	
23	385	SSG	10-Aug-05	month 3	Normal	
23	385	SSG	11-Nov-05	month 6	Normal	
23	386	Combination	19-Apr-05	day 0	Normal	
23	386	Combination	25-Apr-05	day 7	Abnormal (CI)	MILD HEARING LOSS LT EAR AT 1000 AND 2000 HZ, AND 8000 HZ.
23	386	Combination	11-May-05	day 14	Normal	
23	386	Combination	14-May-05	end day	Normal	
23	386	Combination	19-Aug-05	month 3	Normal	
23	386	Combination	11-Nov-05	month 6	Normal	
23	387	Combination	19-Apr-05	day 0	Normal	
23	387	Combination	25-Apr-05	day 7	Normal	
23	387	Combination	2-May-05	day 14	Normal	
23	387	Combination	6-May-05	end day	Normal	
23	387	Combination	10-Aug-05	month 3	Normal	
23	387	Combination	11-Nov-05	month 6	Normal	
23	388	PM	19-Apr-05	day 0	Normal	
23	388	PM	25-Apr-05	day 7	Normal	
23	388	PM	2-May-05	day 14	Normal	
23	388	PM	10-May-05	end day	Normal	
23	388	PM	28-Sep-05	month 3	Normal	
23	388	PM	2-Dec-05	month 6	Normal	
23	389	SSG	19-Apr-05	day 0	Normal	

Centre Number	Patient Number	Treatment	Audiometry Date	Visit	Audiometry Findings	Comment
23	389	SSG	26-Apr-05	day 7	Normal	
23	389	SSG	3-May-05	day 14	Normal	
23	389	SSG	10-May-05	day 21	Normal	
23	389	SSG	20-May-05	end day	Normal	
23	389	SSG	6-Sep-05	month 3	Normal	
23	389	SSG	9-Dec-05	month 6	Normal	
23	390	PM	19-Apr-05	day 0	Normal	
23	390	PM	26-Apr-05	day 7	Normal	
23	390	PM	3-May-05	day 14	Normal	
23	390	PM	11-May-05	end day	Normal	
23	390	PM	10-Aug-05	month 3	Normal	
23	390	PM	2-Dec-05	month 6	Normal	
23	391	Combination	22-Apr-05	day 0	Normal	
23	391	Combination	28-Apr-05	day 7	Normal	
23	391	Combination	5-May-05	day 14	Normal	
23	391	Combination	9-May-05	end day	Normal	
23	391	Combination	19-Aug-05	month 3	Normal	
23	391	Combination	17-Nov-05	month 6	Normal	
23	392	Combination	20-Apr-05	day 0	Normal	
23	392	Combination	28-Apr-05	day 7	Normal	
23	392	Combination	5-May-05	day 14	Normal	
23	392	Combination	22-Jul-05	month 3	Normal	
23	392	Combination	30-Sep-05	month 6	Normal	
23	393	SSG	21-Apr-05	day 0	Normal	
23	393	SSG	28-Apr-05	day 7	Normal	
23	393	SSG	5-May-05	day 14	Normal	
23	393	SSG	12-May-05	day 21	Normal	
23	393	SSG	22-May-05	end day	Normal	
23	393	SSG	19-Aug-05	month 3	Normal	
23	393	SSG	17-Nov-05	month 6	Normal	
23	394	PM	25-Apr-05	day 0	Normal	
23	394	PM	2-May-05	day 7	Normal	
23	394	PM	9-May-05	day 14	Normal	
23	394	PM	17-May-05	end day	Normal	
23	394	PM	19-Aug-05	month 3	Normal	
23	394	PM	17-Nov-05	month 6	Normal	
23	395	PM	28-Apr-05	day 0	Normal	
23	395	PM	5-May-05	day 7	Normal	
23	395	PM	12-May-05	day 14	Abnormal (CI)	MILD HEARING LOSS RT EAR AT 250 HZ
23	395	PM	20-May-05	end day	Normal	
23	395	PM	15-Dec-05	month 6	Normal	
23	396	SSG	13-May-05	day 0	Normal	
23	396	SSG	19-May-05	day 7	Normal	
23	396	SSG	26-May-05	day 14	Normal	
23	396	SSG	2-Jun-05	day 21	Normal	
23	396	SSG	12-Jun-05	end day	Normal	
23	396	SSG	15-Sep-05	month 3	Normal	
23	396	SSG	15-Dec-05	month 6	Normal	
23	397	SSG	13-May-05	day 0	Normal	
23	397	SSG	19-May-05	day 7	Normal	
23	397	SSG	26-May-05	day 14	Normal	
23	397	SSG	2-Jun-05	day 21	Normal	
23	397	SSG	12-Jun-05	end day	Normal	
23	397	SSG	15-Sep-05	month 3	Normal	

Centre Number	Patient Number	Treatment	Audiometry Date	Visit	Audiometry Findings	Comment
23	397	SSG	15-Dec-05	month 6	Normal	
23	398	Combination	18-May-05	day 0	Normal	
23	398	Combination	24-May-05	day 7	Normal	
23	398	Combination	31-May-05	day 14	Normal	
23	398	Combination	4-Jun-05	end day	Normal	
23	398	Combination	30-Sep-05	month 3	Normal	
23	398	Combination	14-Dec-05	month 6	Normal	
23	399	PM	17-May-05	day 0	Normal	
23	399	PM	24-May-05	day 7	Normal	
23	399	PM	31-May-05	day 14	Normal	
23	399	PM	8-Jun-05	end day	Normal	
23	399	PM	14-Oct-05	month 3	Normal	
23	399	PM	15-Dec-05	month 6	Normal	
23	400	PM	3-Jun-05	day 0	Normal	
23	400	PM	9-Jun-05	day 7	Normal	
23	400	PM	16-Jun-05	day 14	Normal	
23	400	PM	24-Jun-05	end day	Normal	
23	400	PM	14-Oct-05	month 3	Normal	
23	400	PM	12-Jan-06	month 6	Normal	
23	401	Combination	2-Jun-05	day 0	Normal	
23	401	Combination	9-Jun-05	day 7	Normal	
23	401	Combination	16-Jun-05	day 14	Normal	
23	401	Combination	20-Jun-05	end day	Normal	
23	401	Combination	21-Sep-05	month 3	Normal	
23	401	Combination	20-Dec-05	month 6	Normal	
23	402	Combination	2-Jun-05	day 0	Normal	
23	402	Combination	9-Jun-05	day 7	Normal	
23	402	Combination	16-Jun-05	day 14	Normal	
23	402	Combination	20-Jun-05	end day	Normal	
23	402	Combination	23-Sep-05	month 3	Normal	
23	402	Combination	20-Dec-05	month 6	Normal	
23	403	SSG	2-Jun-05	day 0	Normal	
23	403	SSG	10-Jun-05	day 7	Normal	
23	403	SSG	17-Jun-05	day 14	Normal	
23	403	SSG	24-Jun-05	day 21	Normal	
23	403	SSG	4-Jul-05	end day	Normal	
23	403	SSG	30-Sep-05	month 3	Normal	
23	403	SSG	12-Jan-06	month 6	Normal	
23	404	SSG	2-Jun-05	day 0	Normal	
23	404	SSG	10-Jun-05	day 7	Normal	
23	404	SSG	17-Jun-05	day 14	Normal	
23	404	SSG	24-Jun-05	day 21	Normal	
23	404	SSG	4-Jul-05	end day	Normal	
23	404	SSG	12-Jan-06	month 6	Normal	
23	405	PM	2-Jun-05	day 0	Normal	
23	405	PM	16-Jun-05	day 7	Normal	
23	405	PM	23-Jun-05	day 14	Normal	
23	405	PM	1-Jul-05	end day	Normal	
23	405	PM	30-Sep-05	month 3	Normal	
23	405	PM	5-Jan-06	month 6	Normal	
34			Not done			
35	646	PM	28-Jan-05	day 0	Normal	
35	646	PM	3-Feb-05	day 7	Normal	
35	646	PM	10-Feb-05	day 14	Normal	

Centre Number	Patient Number	Treatment	Audiometry Date	Visit	Audiometry Findings	Comment
35	646	PM	18-Feb-05	end day	Normal	
35	647	PM	29-Jan-05	day 0	Normal	
35	647	PM	5-Feb-05	day 7	Normal	
35	647	PM	12-Feb-05	day 14	Normal	
35	647	PM	20-Feb-05	end day	Normal	
35	648	Combination	29-Jan-05	day 0	Normal	
35	648	Combination	5-Feb-05	day 7	Normal	
35	648	Combination		day 14	Not Done	
35	648	Combination	16-Feb-05	end day	Normal	
35	648	Combination	25-Aug-05	month 6	Normal	
35	649	Combination	29-Jan-05	day 0	Normal	
35	649	Combination	5-Feb-05	day 7	Normal	
35	649	Combination		day 14	Not Done	
35	649	Combination	16-Feb-05	end day	Normal	
35	649	Combination	15-Sep-05	month 6	Normal	
35	650	SSG	29-Jan-05	day 0	Normal	
35	650	SSG	5-Feb-05	day 7	Normal	
35	650	SSG	12-Feb-05	day 14	Normal	
35	650	SSG	19-Feb-05	day 21	Normal	
35	650	SSG	1-Mar-05	end day	Normal	
35	650	SSG	15-Sep-05	month 6	Normal	
35	651	SSG	29-Jan-05	day 0	Normal	
35	651	SSG	5-Feb-05	day 7	Normal	
35	651	SSG	12-Feb-05	day 14	Normal	
35	651	SSG	19-Feb-05	day 21	Normal	
35	651	SSG	1-Mar-05	end day	Normal	
35	651	SSG	15-Sep-05	month 6	Normal	
35	652	PM	31-Jan-05	day 0	Normal	
35	652	PM	7-Feb-05	day 7	Normal	
35	652	PM	14-Feb-05	day 14	Normal	
35	652	PM	22-Feb-05	end day	Normal	
35	653	Combination	1-Feb-05	day 0	Normal	
35	653	Combination	9-Feb-05	day 7	Normal	
35	653	Combination	16-Feb-05	day 14	Normal	
35	653	Combination	20-Feb-05	end day	Normal	
35	653	Combination	25-Aug-05	month 6	Normal	
35	654	SSG	1-Feb-05	day 0	Normal	
35	654	SSG	9-Feb-05	day 7	Normal	
35	654	SSG	16-Feb-05	day 14	Normal	
35	654	SSG	23-Feb-05	day 21	Normal	
35	654	SSG	5-Mar-05	end day	Normal	
35	654	SSG	19-Sep-05	month 6	Normal	
35	655	Combination	1-Feb-05	day 0	Normal	
35	655	Combination	9-Feb-05	day 7	Normal	
35	655	Combination	16-Feb-05	day 14	Normal	
35	655	Combination	20-Feb-05	end day	Normal	
35	655	Combination	25-Aug-05	month 6	Normal	MILD HEARING LOSS ON RT EAR 30 DBHL
35	656	SSG	3-Feb-05	day 0	Normal	
35	656	SSG	9-Feb-05	day 7	Normal	
35	656	SSG	16-Feb-05	day 14	Normal	
35	656	SSG	23-Feb-05	day 21	Normal	
35	656	SSG	5-Mar-05	end day	Normal	
35	656	SSG	17-Oct-05	month 6	Normal	

Centre Number	Patient Number	Treatment	Audiometry Date	Visit	Audiometry Findings	Comment
35	657	PM	7-Feb-05	day 0	Normal	
35	657	PM	14-Feb-05	day 7	Normal	
35	657	PM	21-Feb-05	day 14	Normal	
35	657	PM	1-Mar-05	end day	Normal	
35	657	PM	15-Sep-05	month 6	Normal	
35	658	Combination	6-Feb-05	day 0	Normal	
35	658	Combination	14-Feb-05	day 7	Normal	
35	658	Combination	21-Feb-05	day 14	Normal	
35	658	Combination	25-Feb-05	end day	Normal	
35	658	Combination	25-Aug-05	month 6	Normal	
35	659	SSG	7-Feb-05	day 0	Normal	
35	659	SSG	14-Feb-05	day 7	Normal	
35	659	SSG	21-Feb-05	day 14	Normal	
35	659	SSG	28-Feb-05	day 21	Normal	
35	659	SSG	10-Mar-05	end day	Normal	
35	659	SSG	29-Oct-05	month 6	Normal	
35	660	PM	10-Feb-05	day 0	Normal	MILD HEARING IMPAIRMENT
35	660	PM	16-Feb-05	day 7	Normal	MILD HEARING IMPAIRMENT
35	660	PM	23-Feb-05	day 14	Normal	MODERATE R MILD L
35	660	PM	3-Mar-05	end day	Normal	
35	660	PM	15-Sep-05	month 6	Normal	
35	661	Combination	16-Feb-05	day 0	Normal	
35	661	Combination	22-Feb-05	day 7	Normal	
35	661	Combination	1-Mar-05	day 14	Normal	
35	661	Combination	5-Mar-05	end day	Normal	
35	661	Combination	24-Sep-05	month 6	Normal	
35	662	PM	19-Feb-05	day 0	Normal	
35	662	PM	26-Feb-05	day 7	Normal	
35	662	PM	5-Mar-05	day 14	Normal	
35	662	PM	13-Mar-05	end day	Normal	
35	663	SSG	19-Feb-05	day 0	Normal	
35	663	SSG	26-Feb-05	day 7	Normal	
35	663	SSG	5-Mar-05	day 14	Normal	
35	663	SSG	12-Mar-05	day 21	Normal	
35	663	SSG	22-Mar-05	end day	Normal	
35	663	SSG	22-Sep-05	month 6	Normal	
35	664	Combination	19-Feb-05	day 0	Normal	
35	664	Combination	26-Feb-05	day 7	Normal	
35	665	SSG	22-Feb-05	day 0	Normal	
35	665	SSG	1-Mar-05	day 7	Normal	
35	665	SSG	8-Mar-05	day 14	Normal	
35	665	SSG	15-Mar-05	day 21	Normal	
35	665	SSG	25-Mar-05	end day	Normal	
35	665	SSG	23-Oct-05	month 6	Normal	
35	666	SSG	25-Feb-05	day 0	Normal	
35	666	SSG	4-Mar-05	day 7	Normal	
35	666	SSG	11-Mar-05	day 14	Normal	
35	666	SSG	18-Mar-05	day 21	Normal	
35	666	SSG	28-Mar-05	end day	Normal	
35	666	SSG	25-Sep-05	month 6	Normal	
35	667	Combination	28-Feb-05	day 0	Normal	
35	667	Combination	6-Mar-05	day 7	Normal	
35	667	Combination	13-Mar-05	day 14	Normal	
35	667	Combination	17-Mar-05	end day	Normal	

Centre Number	Patient Number	Treatment	Audiometry Date	Visit	Audiometry Findings	Comment
35	667	Combination	27-Oct-05	month 6	Normal	
35	668	PM	28-Feb-05	day 0	Normal	
35	668	PM	6-Mar-05	day 7	Normal	
35	668	PM	13-Mar-05	day 14	Normal	
35	668	PM	21-Mar-05	end day	Normal	
35	668	PM	25-Sep-05	month 6	Normal	
35	669	PM	28-Feb-05	day 0	Normal	
35	669	PM	6-Mar-05	day 7	Normal	
35	669	PM	13-Mar-05	day 14	Normal	
35	669	PM	21-Mar-05	end day	Normal	
35	670	SSG	3-Mar-05	day 0	Normal	
35	670	SSG	9-Mar-05	day 7	Normal	
35	670	SSG	16-Mar-05	day 14	Normal	
35	670	SSG	23-Mar-05	day 21	Normal	
35	670	SSG	2-Apr-05	end day	Normal	
35	670	SSG	17-Oct-05	month 6	Normal	
35	671	PM	10-Mar-05	day 0	Normal	
35	671	PM	17-Mar-05	day 7	Normal	
35	671	PM	24-Mar-05	day 14	Normal	
35	671	PM	1-Apr-05	end day	Normal	
35	672	Combination	10-Mar-05	day 0	Normal	
35	672	Combination	17-Mar-05	day 7	Normal	
35	672	Combination	24-Mar-05	day 14	Normal	
35	672	Combination	28-Mar-05	end day	Normal	
35	672	Combination	29-Oct-05	month 6	Normal	
35	673	SSG	10-Mar-05	day 0	Normal	
35	673	SSG	17-Mar-05	day 7	Normal	
35	673	SSG	24-Mar-05	day 14	Normal	
35	673	SSG	31-Mar-05	day 21	Normal	
35	673	SSG	10-Apr-05	end day	Normal	
35	673	SSG	17-Oct-05	month 6	Normal	
35	674	Combination	11-Mar-05	day 0	Normal	
35	674	Combination	19-Mar-05	day 7	Normal	
35	674	Combination	26-Mar-05	day 14	Normal	
35	674	Combination	30-Mar-05	end day	Normal	
35	674	Combination	25-Oct-05	month 6	Normal	
35	675	PM	13-Mar-05	day 0	Normal	
35	675	PM	20-Mar-05	day 7	Normal	
35	675	PM	27-Mar-05	day 14	Normal	
35	675	PM	4-Apr-05	end day	Normal	
35	676	PM	19-Mar-05	day 0	Normal	
35	676	PM	26-Mar-05	day 7	Normal	
35	676	PM	2-Apr-05	day 14	Normal	
35	676	PM	10-Apr-05	end day	Normal	
35	676	PM	25-Oct-05	month 6	Normal	
35	677	SSG	19-Mar-05	day 0	Normal	
35	677	SSG	26-Mar-05	day 7	Normal	
35	678	Combination	20-Mar-05	day 0	Normal	
35	678	Combination	28-Mar-05	day 7	Normal	
35	678	Combination	4-Apr-05	day 14	Normal	
35	678	Combination	8-Apr-05	end day	Normal	
35	678	Combination	25-Oct-05	month 6	Normal	
35	679	SSG	20-Mar-05	day 0	Normal	
35	679	SSG	4-Apr-05	day 7	Normal	

Centre Number	Patient Number	Treatment	Audiometry Date	Visit	Audiometry Findings	Comment
35	679	SSG	4-Apr-05	day 14	Normal	
35	679	SSG	11-Apr-05	day 21	Normal	
35	679	SSG	20-Apr-05	end day	Normal	
35	679	SSG	25-Oct-05	month 6	Normal	
35	680	Combination	21-Mar-05	day 0	Normal	
35	680	Combination	28-Mar-05	day 7	Normal	
35	680	Combination	4-Apr-05	day 14	Normal	
35	680	Combination	8-Apr-05	end day	Normal	
35	680	Combination	17-Oct-05	month 6	Normal	
35	681	Combination	21-Mar-05	day 0	Normal	
35	681	Combination	28-Mar-05	day 7	Normal	
35	681	Combination	4-Apr-05	day 14	Normal	
35	681	Combination	8-Apr-05	end day	Normal	
35	681	Combination	23-Oct-05	month 6	Normal	
35	682	PM	21-Mar-05	day 0	Normal	
35	682	PM	28-Mar-05	day 7	Normal	
35	682	PM	4-Apr-05	day 14	Normal	
35	682	PM	12-Apr-05	end day	Normal	
35	683	Combination	21-Mar-05	day 0	Normal	
35	683	Combination	28-Mar-05	day 7	Normal	
35	683	Combination	4-Apr-05	day 14	Normal	
35	683	Combination	8-Apr-05	end day	Normal	
35	683	Combination	29-Oct-05	month 6	Normal	
35	684	SSG	8-Apr-05	day 0	Normal	
35	684	SSG	15-Apr-05	day 7	Normal	
35	684	SSG	22-Apr-05	day 14	Normal	
35	684	SSG	29-Apr-05	day 21	Normal	
35	684	SSG	9-May-05	end day	Normal	
35	684	SSG	13-Nov-05	month 6	Normal	
35	685	PM	8-Apr-05	day 0	Normal	
35	685	PM	15-Apr-05	day 7	Normal	
35	685	PM	22-Apr-05	day 14	Normal	
35	685	PM	30-Apr-05	end day	Normal	
35	685	PM	29-Oct-05	month 6	Normal	
35	686	PM	13-Apr-05	day 0	Normal	
35	686	PM	19-Apr-05	day 7	Normal	
35	686	PM	26-Apr-05	day 14	Normal	
35	686	PM	4-May-05	end day	Normal	
35	687	SSG	13-Apr-05	day 0	Normal	MILD BILATERAL
35	687	SSG	19-Apr-05	day 7	Normal	MILD BILATERAL LT 30DB AT 500HZ, AND 35 DB AT 8000HZ RT 30DB AT 500HZ, 30DB AT 8000HZ
35	687	SSG	26-Apr-05	day 14	Normal	MILD RT EAR 30DB AT BOTH 4000 & 8000HZ
35	687	SSG	3-May-05	day 21	Normal	MILD BOTH LT 30 DB AT 500HZ & 1000HZ, 35 DB AT 250 & 2000HZ, RT 30DB AT 250HZ
35	687	SSG	13-May-05	end day	Normal	MILD BOTH EARS LT 30DB AT 250HZ, 35DB AT 500HZ RT 30DB AT 250HZ,500 & 1000HZ
35	687	SSG	29-Oct-05	month 6	Normal	
35	688	PM	15-Apr-05	day 0	Normal	
35	688	PM	22-Apr-05	day 7	Normal	MILD IMPAIRMENT IN THE LEFT YEAR I.E 35 DBHL(AC) 500 HZ

Centre Number	Patient Number	Treatment	Audiometry Date	Visit	Audiometry Findings	Comment
35	688	PM	29-Apr-05	day 14	Normal	
35	688	PM	7-May-05	end day	Normal	
35	688	PM	25-Oct-05	month 6	Normal	
35	689	Combination	16-Apr-05	day 0	Normal	
35	689	Combination	22-Apr-05	day 7	Normal	
35	689	Combination	29-Apr-05	day 14	Normal	
35	689	Combination	3-May-05	end day	Normal	
35	690	SSG	16-Apr-05	day 0	Normal	
35	690	SSG	22-Apr-05	day 7	Normal	
35	690	SSG	29-Apr-05	day 14	Normal	
35	690	SSG	6-May-05	day 21	Normal	
35	690	SSG	16-May-05	end day	Normal	
35	690	SSG	29-Oct-05	month 6	Normal	

Appendix 48 SAE reports

SERIOUS ADVERSE EXPERIENCE

PATIENT NUMBER: 34/522
SUBJECT INITIAL: AAY

Serious Adverse Experience: **Suspected TB Pericarditis**

Demography: Age (years): 36 Sex: M Race: African
Height (cm) 164.0 Weight (kg): 48.00

**Country:**	Sudan (Um El Kher)
**Protocol:**	A Multicentre comparative trial of efficacy and safety of Sodium stibogluconate (SSG) versus Paramomycin (PM) versus combination of SSG and PM as first line treatment for Visceral Leishmaniasis in Ethiopia, Kenya and Sudan
**Study Drug:**	SSG +PM
**Study Drug Start Date:**	11 January 2005
**Study Drug Stop Date:**	Study drug not stopped
**SAE Onset Date:**	28 January 2005
**Investigator Attribution:**	Not related

AAY, a 36 year old male on SSG + PM combination treatment which was started on 11.01.2005. He developed dyspnea on 19.01.2005 and on 24.01.2005 he was thought to be asthmatic and was started on hydrocortisone and salbutamol. Baseline investigations revealed that his chest was clear, Hb 10.5g/dl, spleen 7 cm, weight 48kg. He had bilateral leg oedema. HIV test was not done.

On 29.01.2005 his sputum was tested for Acid Fast Bacilli and was reported as negative. The patient was transferred to Gedaref Teaching Hospital for further investigations where chest X-ray done revealed pericarditis and signs of TB. The patient was put on anti TB drugs.

SAE Resolution: At the time of follow up report, 17.02.2005 patient was clinically well

SERIOUS ADVERSE EXPERIENCE

PATIENT NUMBER: 34/534

SUBJECT INITIALS: AMA

Serious Adverse Experience: Threatened Anaemic Heart failure

Demography: Age (years): 7 Sex: M Race: African

Height (cm) 1.09 Weight (kg): 14.0

Country: Sudan (Um El Kher)

Protocol: A Multicentre comparative trial of efficacy and safety of Sodium stibogluconate (SSG) versus Paramomycin (PM) versus combination of SSG and PM as first line treatment for Visceral Leishmaniasis in Ethiopia, Kenya and Sudan

Study Drug: PM

Study Drug Start Date: 23 January 2005

Study Drug Stop Date: Study drug not stopped

SAE Onset Date: 14 February 2005

Investigator Attribution: Probably Related

AMA, a 7 year old male who was put on PM arm on 23.01.2005 and received a full 21 day course. On day 7 of treatment with PM he was diagnosed to have pneumonia and treated with Ceftriaxone. Patient had poor appetite. Study drug was not stopped. On 14.02.2005 (i.e. day 22) he was found to have a low Hb 4.2g/dl with signs of heart failure. He was transfused 250mls of blood and his condition stabilized after he was transfused. This patient was not tested for HIV.

Parasitology at TOC on 14.02.05 negative, repeated on 15.02.05 due to patients poor condition. Parasitology positive 1+ i.e. treatment failure and started on Ambisome 5mg/kg x 6 doses.

SAE Resolution: Patient lost to follow up and was neither seen on the 3rd or 6th month follow up visit.

SERIOUS ADVERSE EXPERIENCE

PATIENT NUMBER: 34/461

SUBJECT INITIALS: IMI

Serious Adverse Experience: **Extensive Epistaxis**

Demography: Age (years): 50 Sex: M Race: African

Height (cm) 174.0 Weight (kg): 55.00

Country: Sudan (Um El Kher)

Protocol: A multicentre comparative trial of efficacy and safety of Sodium stibogluconate (SSG) versus Paramomycin (PM) versus combination of SSG and PM as first line treatment for Visceral Leishmaniasis in Ethiopia, Kenya and Sudan

Study Drug: SSG

Study Drug Start Date: 26 November 2004

Study Drug Stop Date: 10 December 2004

SAE Onset Date: 28 November 2004

Investigator Attribution: Not Related

IMI, a 50 year old male was admitted on 24.11.2004 complaining of episodes of epistaxis. A diagnosis of visceral leishmaniasis was made and he was randomized to receive SSG. On day 3 of treatment in the evening of 28.11.2004, the patient suddenly had extensive nasal bleeding. His Hb dropped from 8.3g/dl on day of admission to 5.8 g/dl on day 3 (28.11.04). BP, pulse and respiratory rate were normal. He was put on plasma expander (500ml) and vitamin K. He was also given folic acid (5mg), Ferrous sulphate (200mg), Ceftriaxone 2g and paracetamol 3g. He was transfused 450mls of blood. His condition stabilized after the transfusion. Hb rose to 7.2g/dl. The patient was not tested for HIV.

SAE Resolution: SAE resolved on 01.12.2004. However, on 09.12.04 patient diagnosed to have

TB and was withdrawn from study and referred for TB treatment.

SERIOUS ADVERSE EXPERIENCE

PATIENT NUMBER: 11/040

SUBJECT INITIALS: ATK

Serious Adverse Experience: Petecheal Haemorrhage

**Demography:**	Age (years):	8	Sex:	F	Race:	African
	Height (cm)	118.0	Weight (kg):	16.00		

**Country:**	Ethiopia (Gondar)
**Protocol:**	A multicentre comparative trial of efficacy and safety of Sodium stibogluconate (SSG) versus Paramomycin (PM) versus combination of SSG and PM as first line treatment for Visceral Leishmaniasis in Ethiopia, Kenya and Sudan
**Study Drug:**	PM
**Study Drug Start Date:**	20 October 2005
**Study Drug Stop Date:**	04 November 2005
**SAE Onset Date:**	26 October 2005
**Investigator Attribution:**	Possibly related

ATK, 8 year old HIV negative female patient was put on PM arm on 20.10.2005. On day 7 of treatment patient was found to have thrombocytopenia of 1,000/ml compared to 129,000/ml on day 0. The condition was thought to be life threatening and patient was put on Coartem, Dipyrone 1 cc stat (500mg) and Paracetamol 500mg stat. The SAE was possibly related to the study drug and the drug was discontinued, the patient remained stable and was given Ambisome. Patient was followed up at 3 and 6 months and is cured of VL.

SAE Resolution: SAE resolved on 09.11.2005

SERIOUS ADVERSE EXPERIENCE

PATIENT NUMBER: 11/027

STUDY INITIALS: TSHM

Serious Adverse Experience: Death

Demography: Age (years): 31 Sex: M Race: African

Height (cm) 164.0 Weight (kg): 46.00

Country: Ethiopia (Gondar)

Protocol: A Multicentre comparative trial of efficacy and safety of Sodium stibogluconate (SSG) versus Paramomycin (PM) versus combination of SSG and PM as first line treatment for visceral leishmaniasis in Ethiopia, Kenya and Sudan

Study Drug: PM

Study Drug Start Date: 19 September 2005

Study Drug Stop Date: Drug not stopped

SAE Onset Date: 19 February 2006

Investigator Attribution: Not Related

TSHM is a 31 year old male patient on PM arm of the study. He was HIV positive. At the end of treatment with PM, he still had VL, however the parasite load had reduced from 5+ before treatment to 1+ at the end of treatment (day 22). Clinically well, sent home for review at follow up.

At 3 months follow up the patient relapsed and the parasite load was 6+. He presented to the trial site with features consistent with sepsis secondary to gastroenteritis. He was treated with Ceftriaxone and rescue medication for VL (Ambisome). The patient was also started on anti retroviral medication. He suddenly died on 19.02.06, 4 months after initial treatment.

The SAE was not related to the trial medication.

SERIOUS ADVERSE EXPERIENCE

PATIENT NUMBER: 11/015

STUDY INITIALS: HDA

Serious Adverse Experience: Severe Anaemia

Demography: Age (years): 20 Sex: M Race: African

Height (cm) 165.0 Weight (kg): 47.00

Country: Ethiopia (Gondar)

Protocol: A Multicentre comparative trial of efficacy and safety of Sodium stibogluconate (SSG) versus Paramomycin (PM) versus combination of SSG and PM as first line treatment for visceral leishmaniasis in Ethiopia, Kenya and Sudan

Study Drug: PM

Study Drug Start Date: 14 July 2005

Study Drug Stop Date: 20 July 2005

SAE Onset Date: 20 July 2005

Investigator Attribution: Possibly related

HAD is a 20 year old HIV negative male on PM arm. Treatment was started on 14.07.2005. Pre-treatment Hb on day 0 was 5.2g/dl and this dropped to 4.0g/dl on day 7. This was recorded as a life threatening SAE and patient was transfused 2 units of blood and Hb measured three days later was 6.5g/dl. The patient was withdrawn from the trial and treated with Ambisome 4mg/kg for 10 days. Patient was seen at 3 and 6 months follow up and is cured of their VL. At 6 months follow up Hb was 9.2g/dl and patient was parasite negative.

SAE resolution: SAE resolved on 27.07.05

SERIOUS ADVERSE EXPERIENCE

PATIENT NUMBER: 11/072

STUDY INITIALS: ATT

Serious Adverse Experience: Severe sepsis GI focus (shock)

**Demography:**	Age (years):	23	Sex:	M		Race:	African
	Height (cm)	172.0	Weight (kg):	50.00			

**Country:**	Ethiopia (Gondar)
**Protocol:**	A Multicentre comparative trial of efficacy and safety of Sodium stibogluconate (SSG) versus Paramomycin (PM) versus combination of SSG and PM as first line treatment for visceral leishmaniasis in Ethiopia, Kenya and Sudan
**Study Drug:**	SSG
**Study Drug Start Date:**	22 July 2006
**Study Drug Stop Date:**	6 August 2006
**SAE Onset Date:**	5 August 2006
**Investigator Attribution:**	Unlikely Related

ATT is a VL and HIV positive patient on SSG arm of treatment. SSG was started on 22.07.06. On day 15 of treatment he developed diarrhea and after 12 hours his vital signs became abnormal. BP was 90/30 mmHg, pulse rate was weak at 120/min. Patient was given normal saline 1000mls and there was no significant improvement. He was subsequently put on hydrocortisone 50mg, Intravenous Ceftriaxone 1gm bid, Gentamycin 80mg tid, Lasix 20mg and blood transfusion (450mls).

SAE Resolution: Patient became better and was discharged home on 14.08.2006 on anti-retroviral drugs and is being followed up.

SERIOUS ADVERSE EXPERIENCE

PATIENT NUMBER: 35/664

SUBJECT INITIALS: KMA

Serious Adverse Experience: **Bilateral active parenchymal kidney disease (Interstitial Nephritis)**

Demography: Age (years): 37 Sex: F Race: African

Height (cm) 171.0 Weight (kg): 73.00

Country: Sudan (Kassab)

Protocol: A Multicentre comparative trial of efficacy and safety of Sodium Stibogluconate (SSG) versus Paramomycin (PM) versus combination of SSG and PM as first line treatment for Visceral Leishmaniasis in Ethiopia, Kenya and Sudan

Study Drug: SSG + PM

Study Drug Start Date: 20 February 2005

Study Drug Stop Date: 26 February 2005

SAE Onset Date: 26 February 2005

Investigator Attribution: Probably Related

KMA is a 37 year old female on combination arm (SSG + PM). Following evaluation on day 7 of treatment (26.02.05) she was found to have high BUN and serum creatinine levels. ECG showed prolonged QT interval. BUN day 0 i.e. 19.2.05 was 33g/dl (N = 10-50g/dl)

Creatinine day 0 was 1.0mg/dl (normal 0.7 – 1.4 mg/dl). On day 4 of treatment she complained of heartburn and vomiting which was not frequent. She was normotensive. She was put on antacid which was stopped within 72 hours.

Day 7 BUN = 102 mg/dl Creatinine = 3.5 mg/dl. Treatment was stopped on 26.02.2005.

On day 10 (01.03.05) ECG returned to normal. BUN and creatinine were still elevated at 113.0mg/dl and 6.0 mg/dl respectively. The patient remained generally well and was not oliguric. Abdominal ultrasound done on day 10 (01.03.05) revealed bilateral active parenchymal disease consistent with interstitial nephritis probably drug induced. Rescue therapy with Ambisome was started on 15/03/2005. Patient was parasite negative at six month follow up.

SAE Resolution: Patient recovered completely within 3 weeks (23 March 2005) and followed up thereafter several times.

SERIOUS ADVERSE EXPERIENCE

PATIENT NUMBER: 23/377

SUBJECT INITIALS: CMK

Serious Adverse Experience: Elevated Liver Enzymes

Demography: Age (years): 40 Sex: F Race: African

Height (cm) 158.0 Weight (kg): 46.00

Country: Kenya

Protocol: A Multicentre comparative trial of efficacy and safety of Sodium stibogluconate (SSG) versus Paramomycin (PM) versus combination of SSG and PM as first line treatment for visceral leishmaniasis in Ethiopia, Kenya and Sudan

Study Drug: SSG + PM

Study Drug Start Date: 9 March 2005

Study Drug Stop Date: 17 March 2005

SAE Onset Date: 16 March 2005

Investigator Attribution: Possibly related

CMK, a 40 year old HIV negative female on combination arm (SSG + PM) which was started on 9.03.2005. On day 8 of treatment she was noted to have elevated liver enzymes. AST 176 U/L (normal upto 38 U/L) ALT 98 U/L (normal up to 41 U/L). A repeat blood sample taken on day 9 (i.e. on 17.3.05) AST 311 U/L ALT 284 U/L. The study medication was stopped. Parasitology performed (splenic aspirate) on day 9, negative. Patient clinically well, therefore Ambisome not given.

At SAE follow up (29.3.05) both tests were normal: AST was 55 U/L and ALT 58 U/L. Patient was reviewed at six months follow up and was parasite negative.

SAE Resolution: The SAE was considered resolved on 29.03.05.

SERIOUS ADVERSE EXPERIENCE

PATIENT NUMBER: 23/397

SUBJECT INITIALS: CJL

Serious Adverse Experience: Intraperitoneal Bleed

Demography: Age (years): 14 Sex: F Race: African

Height (cm) 156.0 Weight (kg): 30.00

Country: Kenya

Protocol: A Multicentre comparative trial of efficacy and safety of Sodium Stibogluconate (SSG) versus Paramomycin (PM) versus combination of SSG and PM as first line treatment for visceral leishmaniasis in Ethiopia, Kenya and Sudan

Study Drug: SSG

Study Drug Start Date: 13 May 2005

Study Drug Stop Date: Drug not stopped

SAE Onset Date: 12 June 2005

Investigator Attribution: Not related

14 year old girl was randomized to receive SSG. Splenic Aspirate was done on day 31 after completing treatment. Patient developed pain and discomfort at the splenic aspirate site. Clinically intraperitoneal bleeding was suspected and this was confirmed by abdominal ultrasound. Patient was managed on intraveneous fluids and antibiotics. SAE resolved without blood transfusion. She was not tested for HIV. Patient was parasite negative at six month follow up.

SAE Resolution: SAE resolved on 15.06.05 and patient was discharged from hospital.

SERIOUS ADVERSE EXPERIENCE

PATIENT NUMBER: 35/677

SUBJECT INITIALS: AAI

**Serious Adverse Experience:**	Death following Acute renal failure					
**Demography:**	Age (years):	37	Sex:	M	Race:	African
	Height (cm)	180.0	Weight (kg):	61.00		

**Country:**	Sudan (Kassab)
**Protocol:**	A Multicentre comparative trial of efficacy and safety of Sodium Stibogluconate (SSG) versus Paramomycin (PM) versus combination of SSG and PM as first line treatment for visceral leishmaniasis in Ethiopia, Kenya and Sudan
**Study Drug:**	SSG
**Study Drug Start Date:**	20 March 2005
**Study Drug Stop Date:**	26 March 2005
**SAE Onset Date:**	26 March 2005
**Investigator Attribution:**	Probably related

AAI is a 37 year old HIV negative male on SSG arm. On day 7 of treatment (i.e. 26.03.05) his laboratory values of BUN and Serum creatinine were abnormal, 192 mg/dl and 3.3 mg/dl respectively. SSG was stopped on 26.03.05 and patient was withdrawn from the trial. Patient was started on Ambisome. After receiving 2 doses of Ambisome, his blood pressure became very low and he was transferred from Kassab to Gedarif Hospital Renal Unit on 31.03.05 for further management.

Abdominal ultrasound done revealed features consistent with interstitial nephritis probably related to SSG.

Patient died on 31.03.05 before any other renal profile investigations could be done and before blood transfusion was given.

SERIOUS ADVERSE EXPERIENCE

PATIENT NUMBER: 12/265

SUBJECT INITIALS: MMK

Serious Adverse Experience: Acute renal failure, death

Demography: Age (years): 5 Sex: F Race: African

Height (cm) 102.0 Weight (kg): 15.00

Country: Ethiopia (Arba Minch)

Protocol: A Multicentre comparative trial of efficacy and safety of Sodium Stibogluconate (SSG) versus Paramomycin (PM) versus combination of SSG and PM as first line treatment for visceral leishmaniasis in Ethiopia, Kenya and Sudan

Study Drug: SSG

Study Drug Start Date: 15 February 2006

Study Drug Stop Date: Study drug stopped (day 28)

SAE Onset Date: 14 March 2006

Investigator Attribution: Possibly related

MMK, a 5 year old HIV negative female patient was on SSG treatment arm. SSG treatment was started on 15.02.2006. On 14.03.06 (day 28 of treatment) her renal function tests were raised 5 fold above normal: BUN = 247 mg/dl (NR 15 – 45), Creatinine = 341 mmol/l (NR 53 – 97), Amylase was elevated (1140 u/l) (NR <125), Platelet count was low (53 x 10^3). Hb = 4.9gm/dl WBC = 1.4 x 10^3. Day 31 end of treatment parasitology not performed due to low platelets.

Treatment for VL was stopped and she was transferred to the General Hospital for intensive care. Patient was catheterized, input-output chart was maintained, ultrasound was done, antibiotics were given, furosamide and blood transfusion given.

SAE Resolution: Despite the above management, the patient died on 20.03.06 possibly due to acute respiratory failure and renal failure secondary to hypoxia and uraemia.

SERIOUS ADVERSE EXPERIENCE

PATIENT NUMBER: 12/246

SUBJECT INITIALS: LLI

Serious Adverse Experience: Death due to Sepsis of Gastrointestinal Tract

Demography: Age (years): 40 Sex: M Race: African

Height (cm) 187.0 Weight (kg): 47.00

Country: Ethiopia (Arba Minch)

Protocol: A Multicentre comparative trial of efficacy and safety of Sodium Stibogluconate (SSG) versus Paramomycin (PM) versus combination of SSG and PM as first line treatment for visceral leishmaniasis in Ethiopia, Kenya and Sudan

Study Drug: PM

Study Drug Start Date: 4 August 2005

Study Drug Stop Date: Study drug not stopped

SAE Onset Date: 12 January 2006

Investigator Attribution: Not related

LLI, a 40 year old male HIV positive patient who was on PM arm. He completed 21 doses of PM and at test of cure on day 22 was parasite negative. At 3 months follow up, parasitology negative and patient well. He presented with intractable diarrhoea and sepsis on 12.01.06 (5 months after treatment). He was started on Ceftriaxone, Chloramphenicol, Dexamethasone and Metronidazole, also on antiretroviral treatment. He developed penile ulcer on 05.01.06. Despite treatment the patient died on 02.02.06.

SERIOUS ADVERSE EXPERIENCE

PATIENT NUMBER: 11/047

SUBJECT INITIALS: TAF

Serious Adverse Experience: Death

Demography: Age (years): 20 Sex: M Race: African

Height (cm) 167.0 Weight (kg): 44.00

Country: Ethiopia (Gondar)

Protocol: A Multicentre comparative trial of efficacy and safety of Sodium Stibogluconate (SSG) versus Paramomycin (PM) versus combination of SSG and PM as first line treatment for visceral leishmaniasis in Ethiopia, Kenya and Sudan

Study Drug: SSG

Study Drug Start Date: 2 May 2005

Study Drug Stop Date: Drug not stopped

SAE Onset Date: 30 July 2006

Investigator Attribution: Not related

TAF, a 20 year old HIV negative male patient was on SSG treatment. He completed his study medication and at the test of cure he was parasite negative.

2 months after discharge, i.e. on 29.07.2006, while at home doing his routine activities, he began to complain of fatigue, fever, bloody diarrhea and occasional vomiting. Patient had no cough, trauma or abdominal cramps. He was conscious.

Sixteen hours after the onset of the above symptoms, while on the way to the health centre, he died possibly due to the dysentery and hypovolemic shock.

SAE Resolution: Patient died on 30.07.2006

PATIENT NUMBER: 12/297 **SUBJECT INITIALS:** ZKL

Serious Adverse Experience: Pancreatitis

Demography: **Age (years):** 29 **Sex:** M **Race:** African

Height (cm): 156cm **Weight (kg):** 47kg

Country: Ethiopia

Protocol: A Multicentre comparative trial of efficacy and safety of Sodium Stibogluconate (SSG) versus Paramomycin (PM) versus combination of SSG and PM as first line treatment for Visceral Leishmaniasis in Ethiopia, Kenya and Sudan

Study Drug: SSG

Study Drug start Date: 12 April 2007

Study Drug stop Date: 26 April 2007

SAE Onset date: 25 April 2007

Investigator Attribution: Possibly related

ZKL is a 29 year old HIV negative male admitted on 03.04.2007. His baseline clinical and laboratory evaluation results were within normal limits. The splenic aspirate was positive (5+) for VL. He was recruited into the LEAP 0104 study and randomized to SSG 850mg on 12.04.2007. On day 7 of treatment all clinical and laboratory values were normal except serum amylase levels which had increased from 121u/l to 460u/l. He was strictly followed up.

On day 14 of treatment, serum amylase levels had increased to 638u/l and the patient complained of epigastric discomfort and back pain. All other organ function tests done (i.e. CBC, U/A, ECG) were within normal limits. Patient was diagnosed with pancreatitis and was withdrawn from treatment and put on conservative management.

His clinical condition and laboratory results (Serum Amylase) improved with conservative management as follows:

Day 14<br>25.04.07	Day 19<br>30.04.07	Day 23<br>04.05.07
638 u/l	211 u/l	180 u/l

SAE Resolution: Patient was started on Ambisome on 04.05.07 after serum amylase levels dropped to 180u/l.

SAE has resolved.

PATIENT NUMBER: 12/316 **SUBJECT INITIALS:** KAG

Serious Adverse Experience: Elevated Serum Amylase

Demography: **Age (years):** 36 **Sex:** M **Race:** African

Height (cm): 173cm **Weight (kg):** 54kg

Country: Ethiopia (Arba Minch)

Protocol: A Multicentre comparative trial of efficacy and safety of Sodium Stibogluconate (SSG) versus Paramomycin (PM) versus combination of SSG and PM as first line treatment for Visceral Leishmaniasis in Ethiopia, Kenya and Sudan

Study Drug: SSG

Study Drug start Date: 29 August 2007

Study Drug stop Date: 12 September 2007

SAE Onset date: 11 September 2007

Investigator Attribution: Probably related

KAG a 36 year old male was admitted on 19.08.2007 with an eight month history of fever, headache and fatigue. He also complained of abdominal pains for four months prior to admission.

On examination spleen size was 18cm. ECG, urine analysis and audiometry were normal. KAG fulfilled inclusion /exclusion criteria and was enrolled into the study to receive SSG on 29.08.2007. He was HIV negative.

On day 7 of treatment (04.09.07) the serum amylase level was 492U/L and one week later the levels were 990U/L. Treatment was stopped on 12.09.07. Stool microscopy showed ascariasis and patient was started on mebendazole 100mg twice daily for three days.

Splenic aspirate done was negative for LD bodies and serum amylase dropped to 710U/L on 13.09.2007, and down to 252U/L on 25.09.2007.

The patient was discharged to come back for review after one month.

SAE Resolution: Resolved on 25.09.07.

Cent No	Pat No	Age	Sex	Treatment	Treatment start date	Adverse Event	Preferred term	AE start date	AE end date	Outcome	Intensity	Action taken	Related
11	15	20	Male	PM	14-Jul-05	SEVERE ANEAMIA	ANAEMIA	20-Jul-05	27-Jul-05	Severe	drug stopped	Resolved	Possible
11	27	31	Male	PM	19-Sep-05	DEATH	DEATH	19-Feb-06	19-Feb-06	Severe	None	Resolved	Not related
11	40	8	Female	PM	20-Oct-05	DROP OF PLETELATE (=1000/MM^3) PETECHIEL HEMGE	PLATELET COUNT DECREASED	26-Oct-05	9-Nov-05	Severe	drug stopped	Resolved	Possible
11	47	20	Male	SSG	5-May-06	DEATH	DEATH	30-Jul-06	30-Jul-06	Severe	None		Not related
11	72	23	Male	SSG	22-Jul-06	SEVERE SEPSIS GI FOCUS(SHOCK)	ABDOMINAL SEPSIS	5-Aug-06	14-Aug-06	Severe	drug stopped	Resolved	Unlikely
12	246	40	Male	PM	4-Aug-05	SEPSIS OF GI FOCUS	ABDOMINAL SEPSIS	12-Jan-06	2-Feb-06	Severe	None	Resolved	Not related
12	265	5	Female	SSG	15-Feb-06	ACUTE RENAL FAILURE	RENAL FAILURE ACUTE	14-Mar-06	20-Mar-06	Severe	drug stopped	Resolved	Possible
12	297	29	Male	SSG	12-Apr-07	PANCREATITIS	PANCREATITIS	25-Apr-07	4-May-07	Mild	drug stopped	Resolved	Possible
12	316	36	Male	SSG	29-Aug-07	RAISED SERUM AMYLASE	BLOOD AMYLASE INCREASED	11-Sep-07	25-Sep-07	Severe	drug stopped	Resolved	Probable
23	377	40	Female	Combination	9-Mar-05	ELEVATED LIVER ENZYMES	HEPATIC ENZYME INCREASED	16-Mar-05	29-Mar-05	Moderate	drug stopped	Resolved	Possible
23	397	14	Female	SSG	18-May-05	INTRAPERITONEAL BLEED	PERITONEAL HAEMORRHAGE	12-Jun-05	15-Jun-05	Moderate	None	Resolved	Not related
34	461	50	Male	SSG	26-Nov-04	EXTENSIVE EPISTAXIS	EPISTAXIS	28-Nov-04	1-Dec-04	Severe	None	Resolved	Not related
34	522	36	Male	Combination	11-Jan-05	TB PERICARDITIS	PERICARDITIS MYCOPLASMAL	28-Jan-05		Severe	None	Ongoing	Not related
34	534	7	Male	PM	24-Jan-05	THREATENED ANAEMIC HEART FAILURE	CARDIAC FAILURE HIGH OUTPUT	14-Feb-05		Severe	None	Ongoing	Probable
35	664	37	Female	Combination	20-Feb-05	BILATERAL ACTIVE PARENCHYMAL KIDNEY DISEASE (INTERSTITIAL NEPHRITIS)	RENAL FAILURE	26-Feb-05	23-Mar-05	Moderate	drug stopped	Resolved	Probable
35	677	37	Male	SSG	20-Mar-05	ACUTE RENAL FAILURE	RENAL FAILURE ACUTE	26-Mar-05	31-Mar-05	Severe	drug stopped	Resolved	Probable

16.2.9 Listing of individual laboratory measurements by patient

Appendix 49: Listing of Haematology measurements per patient – Haemaglobin

Appendix 50: Listing of Haematology measurements per patient – White blood cells

Appendix 51: Listing of Haematology measurements per patient – Platelets

Appendix 52: Listing of Haematology measurements per patient – Prothrombin time

Appendix 53: Listing of clinical chemistry measurements per patient – ALT and AST

Appendix 54: Listing of clinical chemistry measurements per patient – Alkaline Phosphatase

Appendix 55: Listing of clinical chemistry measurements per patient – Amylase

Appendix 56: Listing of clinical chemistry measurements per patient – Bilirubin

Appendix 57: Listing of clinical chemistry measurements per patient – Creatinine and BUN

Appendix 58: Listing of clinical chemistry measurements per patient – Globulin

Appendix 59: Listing of clinical chemistry measurements per patient - Albumin

Appendix 60: Listing of clinical chemistry measurements per patient – Total Protein

Appendix 61: Listing of urinalysis measurements per patient – Urinary Protein

Appendix 62: Listing of urinalysis measurements per patient – Urinary blood

Appendix 63: Listing of urinalysis measurements per patient – pH and Specific Gravity

Centre Number	Patient Number	Treatment	Haemoglobin (g/dl) Baseline	Day 7	Day 14	Day 21	EOT	3 Mon FU	6 Mon FU
11	1	PM	10	10.7	10.4		10.8	10.6	11.3
11	2	Combination	10.7	12.1	13.2		13.1	17.4	8.7
11	3	Combination	8.7	9.2	11.6			13.8	12.3
11	4	PM	6.5	8.3	10.1		10.7	12.9	12.6
11	5	PM	12.3	12.2	10.2		10.9	12.3	12.5
11	6	Combination	5.3	6.1	5		6.8	10.9	11.6
11	7	PM	8.7	8.3	7.6		8.1	10.8	10
11	8	SSG	8.3	9.2	9.7	9.6	9.8	12.7	12.7
11	9	SSG	11.7	12.2	12.3	11.8	11.3	12.7	12
11	10	SSG	12.3	13.5	12.7	13	12.1	14.3	12.8
11	11	Combination	11.8	11.7	13.1		11.6	12.2	13.5
11	12	Combination	10.4	8.2	9.9		10.1	12.7	13.8
11	13	SSG	9.2	8.1	6.7	6.8	6.1	11.8	10.5
11	14	SSG	10.4	11.3	11.3	10.4	11.1	14.2	12.9
11	15	PM	5.2	4				12.5	9.2
11	16	Combination	13.1	11.7	12.4		11.4	12.9	13
11	17	Combination	9.8	10.6	10.6		11.1	10.7	10.4
11	18	SSG	8.5	9.2	9.7	10.8	9.3	8.6	11.8
11	19	Combination	8.1	9.2	8.7		9	10.9	12.2
11	20	SSG	6.3	7.6	7.2	7.4	10.7	8.6	10.5
11	21	SSG	9.4	10.2	9.2	10.2	11	13.2	12.7
11	22	SSG	6.9	8	9.4	10.8	9.8	12.1	12.6
11	23	PM	11.3	9	15.9		14.5	15.5	13.1
11	24	PM	8.9	8	9.6		9.8	10.3	9.3
11	25	SSG	9	6.8	9.1	10.7	12.1	12.3	12.7
11	26	Combination	9.5	10.6	10.3		10.3	11.8	10.3
11	27	PM	8.4	7.4	8.7		8.2	5	
11	28	Combination	8.9	9.8	10.4		10.5	13.4	12.9
11	29	PM	7.4	8.4	8.8		9.4	11.7	15.8
11	30	PM	8.6	10.5	10.4		12.5	13.3	13.9
11	31	SSG	7.7	9.2	10.4	9.8	10.8	12.5	13
11	32	Combination	8.7	9.1	11.2		10.9	12.2	13.4
11	33	SSG	8.2	9.7	11.1	12	12.7	13.7	15
11	34	PM	6.3	6	5.4		6.9	9.5	12
11	35	SSG	11.6	13.5	13.4	13.4	12.9	9.2	11.1
11	36	SSG	6.2	4.6	6.5	8	11	13.7	14.2
11	37	Combination	12	11.1	12		11.6	15.1	16.4
11	38	PM	12	13.3	13.4		12.6	12.5	12.5
11	39	PM	8.5	9.4	10.5		11.1	11.3	11.2
11	40	PM	10.2	7.8	8			15.5	13.5
11	41	Combination	11.5	11.7	12.3		12.8	12.9	13.7
11	42	Combination	13.3	13.8	13.7		13.7	16.3	15.8
11	43	SSG	11.4	10.5	10	10.4	10.8	12.2	13.6
11	44	PM	10.1	11.5	11		12	12.2	13.5
11	45	Combination	13.3	13.7	12.8		12.7	13.9	13.8
11	46	SSG	9.6	10.6	11.7	12.1	11.4	12.6	11.8
11	47	SSG	6.2	6.7	8.1	8.7	9.1		
11	48	Combination	8.8	8.5	9.4		10.2		13.4
11	49	SSG	7.4	8.3	10.3	11.7	11.7	12.7	12.8
11	50	PM	6.6	6.7	7.5		9.6	12.6	14.4
11	51	Combination	7	8.9	10.4		9.4	12.1	12.9

11	52	PM	8.4	8.6	8.9		10.3	11.5	12.8
11	53	SSG	6.9	7.3	8.6	10.3	10.2	15	14.1
11	54	PM	9.6	9.1	8.6		9.6	10.4	11
11	55	PM	8	9	9.2		10.7	13	12.9
11	56	SSG	5	4.8	7.9	8.8	9	11.1	11.2
11	57	Combination	6.5	5.4	7.1		8.3	13.5	14.1
11	58	Combination	7.3	7.2	9.8		11.1	14	13
11	59	PM	5.9	6.4	8.9		11.6	11.2	12
11	60	Combination	7.2	6.9	8.4		9.5	15.3	8
11	61	PM	8.8	7.7	9.9		10.5	11.3	13.1
11	62	SSG	6.3	10.1	10.1	10.3	11.3	12.9	14.5
11	63	SSG	9.5	9.8	10.2	10.7	11.1	12.1	14
11	64	SSG	7.3	7.7	7.9	7.7	8.6	14.1	14.9
11	65	PM	7.5	7.1	7.9		9		
11	66	Combination	8	7.8	7.9		9.5	14.7	13.6
11	67	PM	9	8.3	10.8		12.4	13.5	13
11	68	SSG	7.6	7.4	10.4	11.3	11.4	13.5	13.6
11	69	Combination	5.1	6.9	8.8		8.5	11.2	11.8
11	70	Combination	6.4	7.4	8		9.3	12.6	14.6
11	71	Combination	8.9	9	10.2		9.8	11.5	10.9
11	72	SSG	10.2	9	9.2			14.3	15.7
11	73	PM	7.4	8.6	11.4		10.9	13.4	15
11	74	PM	11.9	11.6	9.8		12.7	12.9	13.6
11	75	Combination	6	6.3	7.9		8.5	10.9	9.7
11	76	PM	7.8	7.6	8.8		10.7	12.6	14
11	77	PM	10	9.5	9.5		11.1	12.3	14.2
11	78	SSG	6.5	7.3	9.3	9.7	9.4	11.7	14.1
11	79	PM	7.2	6.6	6.6		9.2	9	12.2
11	80	PM	9	9.4	12.5		12.5	13.8	15.3
11	81	Combination	6.9	7.8	9.8		10.2	9.8	14.6
11	82	SSG	6.7	7.2	10.8	10.8	12	12.3	13.8
11	83	SSG	5.5	5.8	6.5	7.7	9.7	12.7	14.1
11	84	SSG	7.2	9.3	10	10.4	10.4	12.2	12.9
11	85	SSG	7.4	6.7	7.3	7	9.2	10.8	
11	86	Combination	5.8	5.2	7.3		7.8	9.8	12.1
11	87	Combination	8.8	9.1	9.8		10.7	10.9	14.4
11	88	PM	11.5	11.1	11.6		12.3	14.7	16.6
11	89	Combination	9.2	7.6	10		9.8	12	16.4
11	90	Combination	8.1	7.6	10		9.7		
11	91	PM	5.6	7	10		10.8	6.9	7.9
11	92	Combination	7	6.7	10.1		10.8	12.2	12.9
11	93	SSG	8	8.4	9	9.8	11.3	17.6	16.3
11	94	SSG	8.9	11.1	12.7	13.1	13.4	12.3	13.7
11	95	SSG	9.5	11.8	11.6	11.1	11.3	12.3	12.4
11	96	Combination	7.4	8.4	10.7		10.8	12.1	14.1
11	97	Combination	10.1	9.8	10.2		11.9	13.8	13.2
11	98	PM	8.5	7.8	8.6		8.8	12.5	13.6
11	99	SSG	7.4	6.2	8.8	10.3	11	13.2	13.8
11	100	PM	6	7.9	8.5		9.6		
11	101	PM	8.7	9	9.3		10.6	15.7	16.5
11	102	Combination	8.3	8.9	10.4		10.2	13.9	14
11	103	Combination	9.2	9.9	11.7		11.7	15.3	15.4
11	104	PM	5.4	6.4	8.6		9.6	11.6	13.2

11	105	SSG	5.8	6.4	8.2	9.1	10.2	13.4	13.6
11	106	Combination	7.5	8.5	9.1		8.3	14.2	13.8
11	107	PM	9.4	10.5	11.6		13.5	14	14.5
11	108	PM	10.3	8.7	10.5		11.5	13.1	13.2
11	109	SSG	7.8	9.7	10.8	11.8	10.1	14.5	16
11	110	PM	8.2	6.1	7.6		9.4	13	13.3
11	111	Combination	5.4	10.1	10.7		10.9	12.8	15.1
11	112	Combination	9.7	9.2	8.5		9.4	13.2	14.3
11	113	SSG	11.2	9.6	11.2	10.8	9.5	10.7	
11	114	SSG	11.2	10	11.4	12.9	11.8	14.9	14.4
11	115	Combination	6	7.2	10.7		10.4	13.4	13.6
11	116	SSG	9.1	8.8	10.4	10.6	11.1	15.6	15.5
11	117	PM	8.7	9.4	12.6		13.4	16.6	
11	118	PM	9	10.2	12.6		12.1	12.7	14
11	119	SSG	8.9	8.6	10.3	11.6	12.3	15.3	16.4
11	120	Combination	7.6	9.5	10.2		11.4	13.8	13.9
11	121	Combination	8.6	9.1	10.7		11.4	13.5	13
11	122	SSG	8.1	8.2	9.5	9.5	10.1		
11	123	SSG	6.8	8.4	9.6	9.8	10.3	12.8	14.1
11	124	SSG	7.3	6	7.9	8.6	8.8		13.3
11	125	SSG	8.2	9.9	11.4	13.2	13.3	14.2	13.8
11	126	Combination	10.8	10.5	8.6		9.7		7.8
11	127	PM	8.5	8.5	10.3		11.2	13	12.5
11	128	Combination	9.7	9.7	9.3		9	9.3	9.3
11	129	PM	8.7	9.1	9.2		11.6	10.7	12.2
11	130	Combination	11.7	10.8	13.5		13.4	16.2	16.1
11	131	PM	9.1	8.9	10.5		11.4	12.1	12.3
11	132	SSG	7.3	9.6	11.2	10.9	11.5	13.3	7.7
11	133	Combination	6.5	5.4	9.6		8.7	12.7	12.8
11	134	PM	7.7	7	8.6		9.5	10.1	11.2
11	135	PM	7.3	7.6	9.2		8.7	11.8	14.1
12	241	Combination	7.8	6.3	8.3		8.3		17.9
12	242	SSG	7	6.8	7.7	9.5	6	10.9	11.7
12	243	PM	7	7.1	8.5		8.9	10.4	13.4
12	244	SSG	10.3	8.7	9.2	9.3	8.4	9.5	10.7
12	245	PM	5.4	6.8	7.6		8.3	6.5	
12	246	PM	6.7	6.6	7.7		7.8	6.8	
12	247	PM	5.6	5.8	7.3		8.2	11.6	11.6
12	248	PM	7.2	7.3	9		3.9	15.7	13.7
12	249	Combination	6.8	6.4	4.7		4.8	9.4	10.6
12	250	SSG	5.4	3.8	6.1	7.3	8	10.1	11.8
12	251	Combination	6.7	8.1	8.6		8.8	14	15.3
12	252	SSG	6.3	8.3	9.3	9.4	10.7	11.7	11.6
12	253	SSG	6.3	6.5	7.9	7.9	8.9	9.9	10.7
12	254	Combination	6.2	7	7		8.4	10.4	11.3
12	255	Combination	6	6.8	8.1		8.2	10.5	11.9
12	256	Combination	6.9	7.9	8.2		8.6	13.6	10.8
12	257	SSG	5.4	6.7	8	7.5	8.3	11.2	12.2
12	258	SSG	6.1	6.6	7.4	7.5	9		11.4
12	259	PM	8.1	9.4	9.3		11.4	13.9	10.9
12	260	PM	8.1	9.4	10		10.4		9.1
12	261	Combination	5.4	6.9	8.3		7.7	11	10.8
12	262	PM	8.1	9.1	9.1		11.6	10.7	11.9

12	263	SSG	9.9	9	10.5	3.8	9.9	13.4	14.8
12	264	Combination	3.9	4.8	7.5		8.8	11.9	13.2
12	265	SSG	5	6.2	6.7	7.5	4.9		
12	266	PM	8.9	8.6	8.7		8.7	9.9	14.4
12	267	SSG	13.4	11.7	11.8	12.6	10.5	11.9	13.8
12	268	PM	8.5	8.7	8.1		8.6	12.7	14.2
12	269	Combination	6.5	7.7	8.4		8	11.7	13.6
12	270	Combination	6.1	6.4	6.3		6.8		12.9
12	271	SSG	5.1	5.6	7.7	8.1	6.1	12.4	13.2
12	272	PM	3.9	9.6	7.6		7.7	9.9	10.6
12	273	SSG	7	8.4	8.8	8.8	10.1	12.2	10.6
12	274	SSG	7.9	8.9	9.7	9.6	11.2	13.2	11.8
12	275	Combination	5.9	7.2	7.8		8.5	10.8	13.8
12	276	PM	6.7	10	8.3		9	11.7	10.9
12	277	Combination	4.6	5.3	7.4		7.7	12.3	11.5
12	278	SSG	8.3	7.3	8.8	8.7	10	14.7	
12	279	PM	7	6.4	8.2		8.4	8.1	11.2
12	280	SSG	6	7.6	8.8	8.6	9.5	11.6	11.7
12	281	Combination	6.9	8.5	11.5		11.2	13.5	15.2
12	282	PM	5.5	6.5	7.3		6.1	11.5	11.1
12	283	PM	10.6	9.5	16		11.1	15.1	14.1
12	284	Combination	5.6	6.4	6.8		5.7	11.2	7.2
12	285	Combination	4.9	5.8	7.2		6.5	13.6	13
12	286	PM	5.3	5.4	7.3		7.9	11.7	12.8
12	287	SSG	8.7	10.7	9.9	9.6	10.6	13.5	14.2
12	288	Combination	7.9	7.1	8.3		10.6	12	13.9
12	289	Combination	8.6	8.9	9.8		8.8	13.9	14.9
12	290	SSG	7.4	8.5	9.8	10	10.2		13.9
12	291	SSG	6.9	6.6	8.1	9.5	11.1	14.2	15.3
12	292	PM	5.1	6.1	6.9		5.8		14.8
12	293	SSG	6.9	7.3	8	9.9	10.3		14
12	294	Combination	6.1	7.8	9.1		9	11.7	12.2
12	295	Combination	7.8	7.4	8.2		7.6		11.8
12	296	PM	9.7	9.6	11.1		12.4	13.5	13.9
12	297	SSG	10.5	10.4	11.7				
12	298	PM	8.2	8.2	9.7		10.5	13.7	14.2
12	299	Combination	6.6	7.2	7.6		10.1		14.5
12	300	PM	8.8	9.2	10.8		11.5	10.6	13.3
12	301	SSG	8	9.3	9.2	7.6	10	14.5	14.4
12	302	SSG	5.3	6	6.5	8.1	8.6	19.9	11.8
12	303	PM	9.1	7.5	9.8		9.7	12.6	14.2
12	304	Combination	7.8	10.7	10.8		10.7	14.1	13.8
12	305	PM	7.2	7.8	8.7		9.8	11.4	12.2
12	306	Combination	5.4	6.1	6.4		7.2	10.8	11.9
12	307	Combination	7.5	7.1	8.1		7.7	12.8	14
12	308	PM	6.4	8.5	9.2		10.2	9.6	15
12	309	PM	8	7.8	9.7		9.8	14.4	15
12	310	SSG	7.1	6.9	7.7	7.6	7.4	13.7	12
12	311	SSG	8.1	9.4	7.8	9.3	9.4	12	12.9
12	312	Combination	6.1	8.3	13.4		9.7	13.5	13.8
12	313	PM	6.7	6.8	9		10.9	15.09	16
12	314	SSG	7.6	8.5	9.6	9.8	10.3	13.2	14.1
12	315	Combination	6	6.7	8.2		8.8	11.6	13.3

12	316	SSG	9.3	9.5	11.9			14.7	15.2
12	317	SSG	11.5	8.4	8.3	8.2	9.3		13.1
12	318	Combination	8	8.8	11.7		10.9	13.1	13.1
12	319	Combination	6.7	7.8	9.6		9.6	13.1	13.5
12	320	Combination	7.1	8.4	9.1		9.4	14.6	13.9
12	321	Combination	5	7.1	6.7		6.9	11.6	11.4
12	322	PM	8.2	8.1	9.8		11	12.7	12.1
12	323	SSG	9.3	10.2	9.9	10.1	12.4	14.7	13.3
12	324	SSG	8.5	7.8	7.6	8.6	10.5	13.4	14.4
12	325	PM	8.1	8.9	10.4		12.1	14.4	14.7
12	326	PM	6.6	6.1	7.7		7.8	10.6	14.6
12	327	Combination	8.8	9.3	11.1		11.2	13.8	13.8
12	328	PM	5.9	6.1	9.1		10.8	13.6	13.3
12	329	SSG	6.6	7.8	8.3	8.7	8.7	11.1	11.2
12	330	PM	12.9	8.5	9.2		9.6	11.3	10.7
23	361	Combination	6	7	7.9		8.6	11.6	11.4
23	362	PM	7.3	7.8	10.5		10.4	13.5	13
23	363	PM	7.3	8	10.7		11	12.1	12.9
23	364	Combination	7.1	7.8	9.2		10.5	6.6	12.6
23	365	SSG	7.3	8	9.2	10.2	10.6	12.6	12.8
23	366	Combination	10.2	8	9.1		8	11.5	12.3
23	367	SSG	7.9	7.2	8	8.5	7.7	14.5	16
23	368	PM	6.5	6.7	9.5		10.1	12.8	10.6
23	369	Combination	7.4	7.2	8.8		8.7	11.8	11
23	370	SSG	6.3	7.1	7.8	7.9	8.7	12.3	10
23	371	PM	7.2	6.6	8.3		8.3	11.9	12.2
23	372	PM	9.3	10.6	10.5		11.5	16.2	15
23	373	SSG	6.8	5.9	8.8	9.7	11.6	15.2	12.7
23	374	SSG	6.6	5.7	7.2	9	10.2	11.5	12.1
23	375	Combination	7.1	8.1	7.2		9.4	11.7	11.1
23	376	SSG	8.4	7.4	8.6	9.5	10.2	13.1	13
23	377	Combination	5.9	6.6					12.6
23	378	Combination	5.8	5.8	7.3		8.4	7.7	10.7
23	379	PM	7.7	6.1	7.1		11	5.6	12.4
23	380	SSG	5	5.5	8.1	9	9.9	12.2	11.1
23	381	PM	5.9	6.6	7.9		10.4	11	14
23	382	Combination	5.8	6	8.7		8.5	13.8	11.6
23	383	PM	6.7	5.1	6.9		8.7	11.8	13.9
23	384	SSG	9.5	9.3	9.4	10.2	10	12.5	15.2
23	385	SSG	7.6	7.7	7.7	6.9	8.6	12	13
23	386	Combination	7.8	7.5	9.8		11.2	12.7	13.9
23	387	Combination	7	5.7	6.8		6.9	10.5	12.5
23	388	PM	8.4	9.8	11.3		12.1	11.3	13.9
23	389	SSG	6.3	7.9	7.6	9.3	10	11.6	13.4
23	390	PM	7.3	8	9.2		9.5	12.5	15.3
23	391	Combination	7.5	7.9	8.5		8.8	10.4	12
23	392	Combination	6.8	6.5	8.3			10.9	12.4
23	393	SSG	7.1	7.2	8.9	10.1	9.6	10.6	11.6
23	394	PM	5.5	6.5	7.9		9	10.1	10.1
23	395	PM	7.8	8.3	10.1		10.8	12.9	14.2
23	396	SSG	8	7.9	9.1	10.3	11.4	14.9	16.3
23	397	SSG	6.3	7.4	8.4	8.7	9.1	11.8	12
23	398	Combination	5.1	6.5	7.7		9.1	10.9	6.7

23	399	PM	5.4	4.8	6.8		7.9	7.8	10.4
23	400	PM	6.1	8.2	8.7		10	10.7	13.2
23	401	Combination	9.2	8.5	9.7		10.7	5.3	4.9
23	402	Combination	5.7	7.2	7.5		8.7	12	13.6
23	403	SSG	6.9	6.4	6.9	5.4	5.8	12.5	16.3
23	404	SSG	9.4	9.3	11.2	12.2	12.6		16.1
23	405	PM	7.5	7.9	9.3		8.7	12.1	15.4
34	451	Combination	5.7	6.2	7.7		9.1		12.4
34	452	PM	6.1	6.2	8		10.6		
34	453	PM	5.3	6.6	7.1		10		
34	454	Combination	7.7	6.7	9.5		10.3		12.4
34	455	PM	7.8	7.1	7.3		8		
34	456	SSG	8.1	6	7.6	9	11.1	12.5	
34	457	Combination	9.2	9.2	10.1		10.8		
34	458	PM	5.2	5.2	6.6		9.5	10.8	
34	459	Combination	8.1	8.5	9.4		9.4	12	13.7
34	460	PM	10.4	10.3	11		12.7		
34	461	SSG	8.3	7.2	6.1				
34	462	SSG	9.8	9.8	11.4	12.1	11.8		12.5
34	463	Combination	7.1	8.1	11.5		10		11
34	464	SSG	6	5.1	6.6	7.5	9.6		15
34	465	SSG	8.5	7.2	8.2	10	10.5		13.5
34	466	Combination	10.5	10.8	11.3		11.3		
34	467	PM	7.9	7.1	6.7		9.8	9.6	
34	468	Combination	13	12.1	11.9		12.9		12.3
34	469	SSG	8.4	7.3	6.3	7.8	10.5		14.6
34	470	Combination	8.1	9	9.5		11.5		14.1
34	471	PM	8.9	8.2	8.4		10		
34	472	SSG	12.3	11.8	11.7	12.4	11.2		12.6
34	473	PM	10.3	9.5	9.5		10.5		
34	474	Combination	11.4	10.9	12.2		11.5		14.6
34	475	PM	6.6	7.4	8.5		8.8		
34	476	PM	9.5	8.5	10.2		10.6	9.8	
34	477	SSG	7.7	8.3	8.6	7.7	8		9.8
34	478	Combination	7.9	8.7	11		11.5		12.3
34	479	SSG	10.9	9.1	10.5	11.2	11.6		10.6
34	480	SSG	9.4	9.7	11.1	12.5	12.5		15.2
34	481	Combination	9	9.7	11.5		13.1		13.1
34	482	Combination	12.2	11.8	12.7		12.1		15.3
34	483	PM	14	13.7	14.9		14.5		14.6
34	484	PM	9.5	6.9					
34	485	Combination	6.1	4.5	5.9		6.9		8.6
34	486	Combination	8.3	9.9	11.1		11.6		14.1
34	487	SSG	8.2	8	9.8	10.4	10.9		13.3
34	488	SSG	8	8.5	10.1	10.7	10.1	11.5	11.4
34	489	SSG	8.4	8.3	7.7	9	10		13.1
34	490	PM	7.8	6.3	6.5		8		11.7
34	491	PM	8.4	8.3	8.4		9.6		
34	492	PM	12.9	12.4	12.6		12.6		13.5
34	493	SSG	6.8	7.2	8.2	8.3	10.3	11	
34	494	Combination	8.7	7.5	9.5		9.8		15.3
34	495	SSG	8	9.2	11.5	12	12.5		11.8
34	496	SSG	11	10	12.1	12.3	12.8		15.3

34	497	PM	9.1	8.6	9.5		11.3	9.4	
34	498	SSG	7.7	8	9.6	12.4	12.6		15.8
34	499	Combination	6.5	8.1	10.1		10.5		13.9
34	500	PM	10.8	11.1	11.6		12.5		13.7
34	501	Combination	8.4	6.7	7.9		9.9		13.2
34	502	Combination	7.4	8.3	9.6		10		14.4
34	503	PM	9.1	9.2	9.3		10.8		
34	504	Combination	9.6	10	11		11.2		
34	505	SSG	9.1	9	8.9	10.4	11.4		14.5
34	506	SSG	10.5	9.5	10.5	11.1	12.1		13.2
34	507	SSG	7.7	6.3	6.5	7.7	10.6		12.7
34	508	PM	7.5	6.9	8		10.5		
34	509	PM	7.5	8	9.7		10.3		
34	510	Combination	8.6	8.9	11		10.7		12.7
34	511	Combination	8.2	8.9	9.7		10		12.7
34	512	PM	9.8	8	7.8		10.8		
34	513	Combination	6.5	6.9	9.4		9.9	11.4	10.5
34	514	SSG	8.5	9.9	10.2	11.6	11.6		14.1
34	515	SSG	8.9	10	12.9	12.4	12.5	10.5	
34	516	PM	9.5	10.2	11		13.4		13.6
34	517	PM	10.2	9.3	10.4		11.7		
34	518	SSG	6.4	6.2	7.5	8.1	9.5		12.1
34	519	PM	12.7	10.5	9.4		11.3	9.5	
34	520	SSG	12.7	11	11.9	12.9	14.4		16.3
34	521	PM	5.9	5.8	6.6		8.7		
34	522	Combination	10.5	11.4	11		11.8		
34	523	SSG	6.8	6.4	8.1	9.7	9.7		11.9
34	524	Combination	8.4	9.4	11.3		10.7		13.2
34	525	Combination	9.4	9.7	10.9		11.3		13.8
34	526	SSG	7.8	7.4	8.9	10.9	12.6		10
34	527	Combination	7.3	8.2	9.5		10.5		10.2
34	528	PM	7.2	6.6	7.3		8.4		
34	529	Combination	6.3	6.3	9		10.2		12.1
34	530	Combination	7.6	5.8	7.4		8.5		14.5
34	531	SSG	7	6.5	8.6	10.1	9.8		
34	532	Combination	8.7	7.4	8.1		8.6		11.2
34	533	SSG	8.4	9.3	10.8	12.8	11.7		14.2
34	534	PM	9	6.2	6.7		4.2		
34	535	PM	9.9	10	12.5		11.6	9.1	
34	536	PM	10.2	10.7	11.8		11.7		11.5
34	537	SSG	9.7	9.8	12.2	11.9	12.5		13.8
34	538	PM	7.8	6.9	7.9		8.7		
34	539	Combination	9.6	10.8	11.5		12.5		9.5
34	540	SSG	7.6	6.9	9	9.3	10.8		12.7
35	646	PM	7.1	6.3	7.9		9.9		11.4
35	647	PM	6.7	5.8	7.4		8.8	9.1	10.5
35	648	Combination	6.7	8.6			9.3	10.4	9.8
35	649	Combination	7.1	7.5			9.9	11.5	10.8
35	650	SSG	9.8	10.9	11.7	12	11.8	10.6	10
35	651	SSG	10	8.7	10.3	9.6	12.3	10.5	8.7
35	652	PM	7.1	7.2	7.1		9.4		11.1
35	653	Combination	6	4.7	7.4		7.8	7.5	8.1
35	654	SSG	5.9	6.7	8	7.8	6.5	8.9	9.1

35	655	Combination	6.9	8.2	10.7		11	10.8	9.6
35	656	SSG	6.7	4.3	6.9	9.9	8.1	9.2	9.8
35	657	PM	6.7	8.4	10.1		10.4	10.8	10.1
35	658	Combination	11.8	12.5	12.6		13	8.7	9
35	659	SSG	6.9	5.6	9.1	9.1	9.9	9	10.2
35	660	PM	10.6	11.9	11		10.6	10	9.5
35	661	Combination	8.5	8.3	8.8		8.8	8.6	15.9
35	662	PM	7.2	6.5	7.1		11.4	10.8	10.1
35	663	SSG	8.4	6.8	7	8.9	10.7	10.5	11.5
35	664	Combination	6.3	5.9					9.8
35	665	SSG	7.4	8.3	9.2	11	12	11.8	11.5
35	666	SSG	7.3	7.5	8.1	10.6	10.6	7.4	13.3
35	667	Combination	7.2	7.9	8.4		9.2	8.8	9.7
35	668	PM	5.4	6.7	9.3		10.5		11.8
35	669	PM	7.8	8.1	9.6		8.5		
35	670	SSG	5.7	5.9	8.5	9.9	10.5	9.8	10.1
35	671	PM	6.2	5.4	7.5		8.1		11
35	672	Combination	5.8	7.2	9		9.1		9.8
35	673	SSG	6.5	7	9.5	8.1	9	8.7	9.6
35	674	Combination	5.8	5.8	7.9		8.4	9	10.2
35	675	PM	8.8	7.3	8.6		10.4		11.4
35	676	PM	7	5.9	7.6		11.4	10.8	10.7
35	677	SSG	5.2	5					
35	678	Combination	5.8	6.8	8.2		10.1	9.8	10.2
35	679	SSG	6	6	6.7	9.5	8.8	9	9.8
35	680	Combination	7.1	8.1	8.5		9.1	8.7	9.1
35	681	Combination	7.6	9.7	11.1		11.3	9.2	10.4
35	682	PM	5.6	5.3	6.7		10.8		10.8
35	683	Combination	8	8.4	9.3		9.5		10.1
35	684	SSG	7.9	6.5	6.5	9.1	8.9	9.2	9.8
35	685	PM	7.8	8.4	8.8		11	10.5	10.8
35	686	PM	7.4	8.3	9.8		9.2	8.7	9.6
35	687	SSG	8.5	7.7	8.3	9	6.7	8.3	9.5
35	688	PM	9	8.4	10.7		9.7	9	10.5
35	689	Combination	5.2	5.3	6.9		7.9	8	8.6
35	690	SSG	5.4	5.4	7.8	7.8	8.6	9.1	10.4

Centre Number	Patient Number	Treatment	White Blood Cell Count (x 10^3/µL)						
			Baseline	Day 7	Day 14	Day 21	EOT	3 Mon FU	6 Mon FU
11	1	PM	2.9	3.8	3.7		4.4	5.4	5.1
11	2	Combination	1.5	1.9	3		2.9	3.1	3.4
11	3	Combination	2.5	2.5	3.5		99	5.3	5.4
11	4	PM	4.7	33	4.1		4.8	5.1	4.6
11	5	PM	5.9	9.9	9		6.2	7	8.1
11	6	Combination	5.36	4	3.1		3.5	7.2	7.3
11	7	PM	3.4	5.1	6.3		6.1	4.4	4
11	8	SSG	2.5	2.4	3.2	2.7	2.9	3.8	3.8
11	9	SSG	4.4	4.3	4.2	3.6	5.7	5.2	4.4
11	10	SSG	9.9	7.7	4.7	4.4	6.7	9.1	8.1
11	11	Combination	9.9	8	8.8		6.4	5.6	5.3
11	12	Combination	4.6	1.7	2.7		3.2	3.5	4.2
11	13	SSG	4.8	6	4.7	4.2	4	5.5	6.7
11	14	SSG	8.5	5.9	5.5	4.9	4.8	7.1	8.2
11	15	PM	3.3	2.8				4.6	5.1
11	16	Combination	4.8	3.2	4.8		5	6	4.9
11	17	Combination	5	4.7	4.5		5.5	3.3	5.3
11	18	SSG	3.1	4.1	3.6	2.9	2.5	2.7	4.9
11	19	Combination	1.9	1.8	2.6		2.4	2.3	3.9
11	20	SSG	5.4	4.5	4.3	4	3.4	6.9	8.4
11	21	SSG	4.7	4.3	4.6	4	5.3	5.5	4.5
11	22	SSG	2	4.5	3.2	4.5	6.4	8.6	7.4
11	23	PM	1.4	1.1	2		4.4	4.6	3.1
11	24	PM	5.6	3.3	4.9		4.2	5.9	4.4
11	25	SSG	2.1	1.4	2.4	3	3.7	6.9	5.5
11	26	Combination	5.2	3.5	4.1		4.4	5.1	6.3
11	27	PM	4.2	5.2	5.5		5.7	2.4	
11	28	Combination	4	3.3	3.6		3.7	7.5	7
11	29	PM	3.1	2.9	4.3		3.7	5.3	4
11	30	PM	2.4	4.2	3.1		4	6.9	6.3
11	31	SSG	6.3	5.1	5.8	5.3	5.7	7.9	6
11	32	Combination	1.6	2.9	2.8		2.7	5.8	6.8
11	33	SSG	3.2	2.7	3.3	3.8	3.9	4.1	5.2
11	34	PM	2.1	1.8	2.4		3.1	2.9	3.3
11	35	SSG	5.6	4.9	5.6	6	5.9	3.44	5.5
11	36	SSG	1.1	1	1.3	1.3	3.1	3.6	2.8
11	37	Combination	2.5	2	2.9		3.6	6.4	6.5
11	38	PM	9.6	13	11.5		10	7.5	14
11	39	PM	1.8	1.7	2.6		3.8	4	4.5
11	40	PM	3.7	1.1	2			6.3	7.3
11	41	Combination	6.7	5.7	7.7		6.2	7.8	9.2
11	42	Combination	2.9	3.3	4.9		6	7	4.5
11	43	SSG	4.2	2.8	3.2	2.8	3.4	4.9	5.9
11	44	PM	4.3	4.5	4.8		4.9	6.7	3.8
11	45	Combination	7.3	5	5.4		5.6	7.8	6.2
11	46	SSG	3.8	4.9	5.2	5.3	6.5	7.1	8.1
11	47	SSG	1.2	1.1	2.9	3.4	5.3		
11	48	Combination	3.3	2.2	2.9		3.9		6.5
11	49	SSG	2.3	2.6	3.1	4	4.3	5.6	8.7
11	50	PM	1.2	1.1	1.7		3.7	7.1	5.6
11	51	Combination	1.1	3.3	5.3		4	4.4	5.8
11	52	PM	1.8	1.3	1.9		3.6	2.9	3.4
11	53	SSG	1.4	1.78	3	4.7	12.9	3.5	3.2
11	54	PM	3.2	2.7	3.5		3.7	4.6	2.4
11	55	PM	1.7	1.39	1.7		2.5	4.9	6.2

Centre Number	Patient Number	Treatment	White Blood Cell Count (x 10^3/µL) Baseline	Day 7	Day 14	Day 21	EOT	3 Mon FU	6 Mon FU
11	56	SSG	1.1	1.5	3	4.9	6.9	6.8	5.7
11	57	Combination	1.13	1.1	1.7		2.2	4.4	3.9
11	58	Combination	1.9	3.1	5.5		5.14	7.2	5.2
11	59	PM	1.6	2.4	3.2		5.4	3.9	5.6
11	60	Combination	2	2	3.3		2.4	6.6	4.6
11	61	PM	2.1	1.5	3.2		3.4	3.5	6.5
11	62	SSG	1.9	4.4	3.9	6.58	8.4	8.7	7.6
11	63	SSG	2.3	1.7	1.8	1.9	2.1	4.7	4.3
11	64	SSG	1.1	2.9	2.3	3.5	4.2	4.2	5.3
11	65	PM	4.4	3.9	6.6		8.7		
11	66	Combination	1.1	1.5	2.09		2.2	4	5.2
11	67	PM	1.9	2.6	5		7.7	6.7	8.5
11	68	SSG	2.5	1.8	3.3	3.4	4.9	7.5	5.1
11	69	Combination	2.4	3	3.2		3.4	4.7	5
11	70	Combination	1.1	1.3	1.9		2.5	8.3	5.9
11	71	Combination	1.6	1.1	1.2		1.3	2.9	2
11	72	SSG	2.6	2.2	2.7			6.9	7.7
11	73	PM	1.6	1.7	3.5		3.7	4.8	5.4
11	74	PM	3.7	2.42	2		3.4	4.3	6.5
11	75	Combination	1.3	1.78	2.7		2.9	4.4	4.6
11	76	PM	1	1.7	2.8		3.1	3.9	5
11	77	PM	2.6	2.2	4.5		4.9	5.3	7.4
11	78	SSG	1.5	1.9	2.2	2.3	2.3	3.9	4.2
11	79	PM	1.1	1.6	2.9		3.4	3.8	5.5
11	80	PM	1.8	2.42	4.5		7.8	10.1	10.5
11	81	Combination	1.7	1.8	2.8		3.1	6.4	6
11	82	SSG	1.4	1.5	2.3	3.4	4.3	6.8	4.7
11	83	SSG	1.6	1.6	1.8	3.4	6.9	7.4	6.3
11	84	SSG	1.5	3.4	4.1	4.8	4.4	10.6	10.7
11	85	SSG	1	1.6	3.6	3.6	6.1	6.8	
11	86	Combination	1.1	2.1	2.6		2.1	3.8	3.8
11	87	Combination	1.3	2.7	1.7		3.4	3.6	5
11	88	PM	2.8	2.8	4.5		5.5	9.4	7.4
11	89	Combination	1.5	1.6	2.8		3.7	9	8.6
11	90	Combination	1.8	3.3	3.8		3.4		
11	91	PM	1.1	1.6	3.2		5.1	1.5	2.3
11	92	Combination	3.7	2.9	3.6		5.1	10.4	10.3
11	93	SSG	1.9	4.2	4.9	5.1	7.1	7.4	6.5
11	94	SSG	2.6	3.3	4.6	6.5	7.5	8	6.6
11	95	SSG	2.8	3.6	5.4	6.2	8.4	6.5	5.8
11	96	Combination	2.7	6.2	4.4		5.4	7.8	4.9
11	97	Combination	1	2.3	2.9		4	3.9	3.3
11	98	PM	2	1.9	2.3		2.6	7.9	4.6
11	99	SSG	1.3	1.4	2.8	3.3	4	4.6	3.5
11	100	PM	2.1	2.5	3.4		5		
11	101	PM	2.4	2.5	3.4		3.7	5.1	7.7
11	102	Combination	1.7	3.5	2.9		3.4	4.9	4.8
11	103	Combination	1.9	2.1	3.2		3.3	3.6	6.3
11	104	PM	1.8	2.9	4.3		6.1	7	6.5
11	105	SSG	1.3	1.6	2.4	2.2	3.3	6.8	4.3
11	106	Combination	1.9	3.8	7.3		5.9	8	5.1
11	107	PM	1.7	2.1	2.8		5	6.4	4.2
11	108	PM	1.7	1.3	2		2.8	2.5	2.9
11	109	SSG	1	2.1	4.2	5.9	5.7	7.4	6.9
11	110	PM	1.6	0.8	1.4		2.4	3.1	3.4

Centre Number	Patient Number	Treatment	White Blood Cell Count (x $10^3/\mu L$) Baseline	Day 7	Day 14	Day 21	EOT	3 Mon FU	6 Mon FU
11	111	Combination	1	2.7	3.1		3.8	7.9	9
11	112	Combination	1	2.3	2		3.2	2.8	3
11	113	SSG	1.9	2.6	2.9	4.7	8	4.4	
11	114	SSG	3.5	2.5	2.8	3.4	4.3	4.9	3.6
11	115	Combination	1.1	1.4	2.2		2	6.3	5
11	116	SSG	2.6	1.8	2.7	3	4.2	5	6.3
11	117	PM	1.2	1.3	3.1		4.8	5.3	
11	118	PM	1.8	1.9	2.6		3	3.5	3.1
11	119	SSG	1.6	1.9	2.2	2.8	3.3	4.3	4.6
11	120	Combination	2.3	2.8	3		4.1	4.4	5.6
11	121	Combination	1.6	3.5	2.7		3.4	5.3	6.4
11	122	SSG	1.7	1.9	3.4	4.5	6.1		
11	123	SSG	1.7	2.4	2.6	2.7	3.8	4.6	4.8
11	124	SSG	1	2	1.9	1.4	2.5		4.3
11	125	SSG	1.5	2.8	2.3	5.2	4.7	5.9	6.9
11	126	Combination	2.1	1.5	2		2.5		2.9
11	127	PM	1.4	1.4	3.1		4.6	4.1	5.4
11	128	Combination	4.2	3.1	5.8		4.1	5.5	2.6
11	129	PM	1.8	1.6	2.9		3.3	3.2	3.1
11	130	Combination	1.7	1.7	2.9		2.9	6.5	7.1
11	131	PM	3.3	2	4.5		4.2	7.3	6.7
11	132	SSG	1.4	1.4	2.5	4.2	7.4	7.2	2.4
11	133	Combination	1	1	1.9		3	7.6	6.4
11	134	PM	1.3	1.1	1.5		1.5	1.5	1.9
11	135	PM	2.2	2.8	4.2		5	7.3	7
12	241	Combination	7.4	2.34	3		3.6		4.4
12	242	SSG	6.3	2.8	3.5	4.8	5	3.6	8.4
12	243	PM	5.1	2.4	2.9		4.6	4.8	7
12	244	SSG	7.6	2.3	2.8	2.9	3	3.8	5.3
12	245	PM	1.6	2	2.6		3.6	1.6	
12	246	PM	3	1.6	2.7		3.3	2.3	
12	247	PM	1.9	3.2	2.5		3.8	4.1	4.6
12	248	PM	1.9	1.8	2.8		6.7	5.1	5.3
12	249	Combination	2.8	2.8	3.7		3.6	15.3	4
12	250	SSG	1.5	1.3	1.3	2.1	2.9	3.6	4.8
12	251	Combination	2.1	2.7	3.7		4.4	5.9	8.5
12	252	SSG	1.2	2.3	2.7	2.8	5.5	4.7	3.8
12	253	SSG	1.2	1.2	1.3	1.5	2	3.8	3.3
12	254	Combination	2	2.3	2		3.3	4.7	5.6
12	255	Combination	2	2.6	4.4		5.2	5.2	4.3
12	256	Combination	1	2.2	3.9		4.4	5.4	5.2
12	257	SSG	1.3	2	2.4	2.7	3.3	4.5	5.9
12	258	SSG	2	2.1	2.4	3.3	4.8		7.8
12	259	PM	1.7	1.9	2.4		3.7	3.9	4.8
12	260	PM	2.7	4.1	4.8		4.2		3.3
12	261	Combination	1.4	2.7	4		4.4	6.4	5.8
12	262	PM	2.2	2.6	4.1		4.1	3.8	5.6
12	263	SSG	3	2.6	3.1	9.1	4.6	6.4	7.1
12	264	Combination	3.8	2.7	1.8		3	6.4	6.4
12	265	SSG	1.3	2.1	2.6	1.7	1.4		
12	266	PM	4	3.4	4		3.3	3.8	4.7
12	267	SSG	8	4.3	4.8	4.7	5.4	7.3	9.6
12	268	PM	1	1.6	1.4		1.8	3.1	4
12	269	Combination	1.1	1.2	1.9		3.1	4.7	4.2
12	270	Combination	1.1	1.9	2.6		4		4.4

Appendix 50: Listing of White blood cell count per patient

Centre Number	Patient Number	Treatment	White Blood Cell Count (x 10^3/μL) Baseline	Day 7	Day 14	Day 21	EOT	3 Mon FU	6 Mon FU
12	271	SSG	1.3	2.9	4.8	7.9	4.8	9.6	9.6
12	272	PM	1.4	4.5	4.4		5.1	7.4	5.2
12	273	SSG	3.8	4.9	5.9	5.7	6.7	6.3	5.1
12	274	SSG	2.6	2	3.9	2.8	3.4	5.1	3.1
12	275	Combination	1.6	2	2.4		3.3	5.2	5.4
12	276	PM	2.9	3.2	4.6		3.6	5	4.3
12	277	Combination	1.1	1.5	2.8		3.6	5.5	4.6
12	278	SSG	1.7	1.4	2.2	2.8	3.2	5.2	
12	279	PM	1.1	1.2	2		2.4	3	3.8
12	280	SSG	1.3	2.9	4.7	4.4	7.3	6.4	8.2
12	281	Combination	1.6	3	5.1		6	6.6	8.6
12	282	PM	1.6	2.4	3.5		3.1	3.3	3.6
12	283	PM	4.1	3.2	2.4		3.7	4	3.6
12	284	Combination	2.5	2.8	3.3		3.7	6.2	2.2
12	285	Combination	2.2	2.4	3.6		3.8	8.6	5.2
12	286	PM	1.7	1.8	2.6		3.1	4.2	4.4
12	287	SSG	3.5	6.1	5.9	5	5.1	6.8	6.8
12	288	Combination	3.6	2.6	3.6		1.9	4.7	5.1
12	289	Combination	1.5	1.4	1.7		4	5	3.6
12	290	SSG	2.3	3.9	3.6	4.2	4.6		6.8
12	291	SSG	1	1.2	1.3	1.4	2.3	3.2	4.7
12	292	PM	1.4	1.4	3.4		2.6		4.4
12	293	SSG	1.7	2.4	3.9	3.8	4.2		5.2
12	294	Combination	1.1	2.8	3.9		3.1	7.3	6.2
12	295	Combination	1.9	1.7	1.9		3		3.2
12	296	PM	2.2	1.8	2.8		3.6	3.3	3.4
12	297	SSG	1.7	3	3.4				
12	298	PM	2.3	3.3	5		4.1	7.1	6
12	299	Combination	2	1.9	2.7		2.9		4.3
12	300	PM	2.4	3.5	3.8		5.1	4	5.6
12	301	SSG	1.6	2.8	3.2	6.9	4.1	5.3	6.4
12	302	SSG	2.7	2.4	2.4	3.7	2.7	5.1	5.6
12	303	PM	2.3	1.1	2		2.9	4.1	5.3
12	304	Combination	1	3.5	4.7		5	7.2	6.4
12	305	PM	2.7	3.6	5		6.6	8.8	8
12	306	Combination	1.8	2.7	2.6		3.5	7.4	3.4
12	307	Combination	1.2	1.7	3.5		3.4	4.9	6.2
12	308	PM	1.3	1.7	2.9		3	2	6.5
12	309	PM	1.9	2	4.4		5.6	5.4	6.4
12	310	SSG	2.3	2.7	2.7	2.9	3.2	6.5	5.2
12	311	SSG	1.1	1.6	1.4	3.8	4.4	7.7	9.1
12	312	Combination	1.3	1.6	4.6		3.3	3.9	4.5
12	313	PM	1.2	1.5	3		5	6.2	6.3
12	314	SSG	1.3	1.6	2.1	2.5	2.8	8.1	5.1
12	315	Combination	1.1	1.4	2.3		5.9	4.5	5.3
12	316	SSG	1.7	1.6	3.2			3.9	3.2
12	317	SSG	3.6	2.2	2.6	3.4	5.9		5.9
12	318	Combination	2.8	3.9	5.2		4.2	5.6	5.7
12	319	Combination	1.7	1.7	2.4		3.9	3.7	3.1
12	320	Combination	1.9	3	6.3		6.4	6.9	10.3
12	321	Combination	1.4	1.8	1.9		1.6	4.9	4.7
12	322	PM	1.3	1.3	1.8		3.2	2.6	2.5
12	323	SSG	2.4	2.8	3.2	4	5.4	4.8	5.9
12	324	SSG	2.4	1.4	1.9	2	2.6	3.1	4.4
12	325	PM	1.7	2.1	4		5.4	3.9	5.5

Centre Number	Patient Number	Treatment	White Blood Cell Count (x 10^3/µL)						
			Baseline	Day 7	Day 14	Day 21	EOT	3 Mon FU	6 Mon FU
12	326	PM	1.5	1.5	1.9		2.6	5	8.2
12	327	Combination	2.2	3.2	5.5		4.2	5.2	6.5
12	328	PM	1.2	1	2.1		2.1	2.9	5.2
12	329	SSG	2.7	3.5	3	4.7	4.9	9.7	5.5
12	330	PM	7	2.2	3.2		4.5	5.3	6.2
23	361	Combination	2.33	1.3	4.3		2.6	5	6.6
23	362	PM	2.3	3.2	3.4		3.8	4.3	4.7
23	363	PM	4.7	2.8	6		7	8.8	8.1
23	364	Combination	2.3	2.5	2.9		4	2.1	5.9
23	365	SSG	1.4	2.3	2.8	4.5	3.8	2.8	4.3
23	366	Combination	3	1.9	3.2		3.8	6.6	6.5
23	367	SSG	2.3	2.1	2.4	2.6	3.2	5.2	5.2
23	368	PM	2.8	4.3	5.2		5.5	8.9	8.7
23	369	Combination	2	3.4	4.3		4.9	5.7	4.9
23	370	SSG	2.9	3.9	4.2	5	6.4	9.8	6.7
23	371	PM	1.7	1.7	2.1		3.2	5.7	7.3
23	372	PM	1.8	2.8	3.7		3.6	5.6	4.7
23	373	SSG	1.5	2	2.6	3.6	4.9	5.4	5.2
23	374	SSG	1.8	2.1	2.9	2.5	4.5	4.6	5.4
23	375	Combination	3.5	4.1	4		4.5	6.8	5.9
23	376	SSG	4.7	9	4.2	4.9	6.6	8.1	9.5
23	377	Combination	2.2	2					4.9
23	378	Combination	1.7	2.1	6.2		3.7	3.3	5.1
23	379	PM	3.6	2.9	3.3		4.3	2.7	7.5
23	380	SSG	2.4	2.6	3.3	3.5	4.4	8.7	7.8
23	381	PM	2.4	2.5	2.8		4.6	5.3	5.2
23	382	Combination	3.2	4.5	4		4.7	5.6	6.8
23	383	PM	3.7	2.3	4.2		4	4.5	6.4
23	384	SSG	4.3	3.8	5.5	5	5.4	6.2	15.6
23	385	SSG	3.4	2.6	4.8	3.5	4.1	5.5	6.1
23	386	Combination	2.8	2	3.2		5.1	4.8	4.6
23	387	Combination	1.4	1.3	1.5		1.8	3.4	6
23	388	PM	2	2.7	3		3.6	6.2	4.7
23	389	SSG	2.1	2.8	3.5	4.6	5.3	4.7	5.2
23	390	PM	2.1	2.8	3.9		4.7	5.7	6.7
23	391	Combination	2.6	2.4	2.7		3.4	5.4	4.7
23	392	Combination	3.5	1.4	3			5.3	3.8
23	393	SSG	1.8	2.2	3	3.5	5.1	4.8	4.1
23	394	PM	4	4.4	6.6		6.5	12.6	15.9
23	395	PM	2	2.2	3.7		4.1	5.5	6.1
23	396	SSG	2	2.1	2	3.5	5.4	4.4	5.2
23	397	SSG	1.7	2.2	5	3.8	5.6	6.7	6.7
23	398	Combination	1.7	2.5	2.9		3.2	5.3	3.3
23	399	PM	3.2	3.8	3.9		4.7	5.8	6.4
23	400	PM	1.8	3	3.7		3.6	6.2	8.5
23	401	Combination	5.4	2.6	3.7		3.8	1.9	2.2
23	402	Combination	1.3	2.5	1.7		2.7	3.8	6.4
23	403	SSG	3	2.5	3.6	3.2	2.8	3.9	3.1
23	404	SSG	3.5	3.3	3.4	3.7	4.3		5.6
23	405	PM	3.4	4.7	5.6		5.7	4.8	5.1
34		Not done							
35	646	PM	1.2	1.9	3.3		7.5		4.2
35	647	PM	1.5	1.8	3.8		3.3	6.8	4.2
35	648	Combination	2.4	3.8			7.9	6.2	5
35	649	Combination	2.3	2.9			5.8	5.6	4.2

Centre Number	Patient Number	Treatment	White Blood Cell Count (x 10^3/μL) Baseline	Day 7	Day 14	Day 21	EOT	3 Mon FU	6 Mon FU
35	650	SSG	2.8	4.3	4.1	6.8	2.8	3.1	3
35	651	SSG	5	5.3	5.1	3.7	13.4	8	4.2
35	652	PM	2.6	4.9	4.8		6.8		3.8
35	653	Combination	2.8	4	3.9		4	3.8	4.3
35	654	SSG	2.5	3.7	5.5	5.4	9.1	5.9	5.2
35	655	Combination	2.4	3	3.9		4.1	4	4.2
35	656	SSG	2.2	4.3	4.1	3.5	6.9	5.1	4.5
35	657	PM	3.4	3.6	7.5		4.7	4.8	4.4
35	658	Combination	2.8	2.7	10.7		10	5.2	4.6
35	659	SSG	2.5	3.4	7.9	8.2	3.6	4.9	3.1
35	660	PM	8.8	9	7.7		8.3	3.8	4
35	661	Combination	2.2	2.3	6.2		6.4	5.2	3.2
35	662	PM	2.8	1.9	2.2		7	5.2	4.5
35	663	SSG	4	2.2	2.8	2.2	2.8	3.2	3.5
35	664	Combination	2.8	2.7					4
35	665	SSG	1.3	6.1	5.6	12.3	4.9	3.6	3.8
35	666	SSG	2.3	2.8	3.2	4.1	5.4	4.4	5
35	667	Combination	2.2	2.8	4.2		5.6	4.6	4
35	668	PM	2.1	2.6	3.5		3.8		4.8
35	669	PM	3.4	3.7	3.1		5.8		
35	670	SSG	5.3	5	3.4	3.7	5.5	4.7	3.6
35	671	PM	3.4	3.3	3.9		3.8		5.6
35	672	Combination	2.4	3.2	5.4		5.6		4.3
35	673	SSG	2.6	2.4	3.2	2.3	2.8	3.1	4.1
35	674	Combination	2	2.8	2.4		2.8	3.4	3.8
35	675	PM	8.4	4.7	5.3		7.6		5.2
35	676	PM	2.6	4.1	4.8		9.6	4.5	4.2
35	677	SSG	2.1	4					
35	678	Combination	2.6	3.6	3.4		5.6	4.2	4.5
35	679	SSG	2.2	4.5	3.9	4.2	5.8	4.1	4.3
35	680	Combination	3.2	7.1	3.4		4.9	3.6	3
35	681	Combination	1.9	5.2	4.9		5.1	5.3	4.8
35	682	PM	1.9	2.6	3		6.2		4.4
35	683	Combination	4.1	7.7	5		5.4		5
35	684	SSG	3.4	2.9	3.3	4.7	5.2	4.6	4.1
35	685	PM	3	4.2	6		8.4	5.2	5
35	686	PM	1.1	2.6	3.2		4.2	3.6	3.9
35	687	SSG	7.1	2.4	2.6	3.5	6	4	4.4
35	688	PM	2.4	3.7	3.9		5.5	4.8	5
35	689	Combination	2.1	1.7	2.4		2.9	3.6	3
35	690	SSG	2.5	4.1	5.9	5.3	7.4	4.5	5

Centre Number	Patient Number	Treatment	Platelets (x 10^3/µL) Baseline	Day 7	Day 14	Day 21	EOT	3 Mon FU	6 Mon FU
11	1	PM	175	168	234		265	216	145
11	2	Combination	105	110	137		193	156	56
11	3	Combination	59	51	84			228	159
11	4	PM	108	91	101		181	176	190
11	5	PM	249	157	225		200	249	303
11	6	Combination	78	70	84		101	165	151
11	7	PM	45	66	102		108	100	98
11	8	SSG	121	85	125	143	133	134	134
11	9	SSG	81	84	104	64	83	123	118
11	10	SSG	101	124	126	102	83	144	103
11	11	Combination	263	188	264		241	236	215
11	12	Combination	174	75	121		166	204	222
11	13	SSG	55	54	77	89	101	190	206
11	14	SSG	252	267	284	277	348	290	276
11	15	PM	44	56				134	86
11	16	Combination	204	187	280		193	212	184
11	17	Combination	207	232	279		294	85	333
11	18	SSG	79		114	125	43	150	110
11	19	Combination	143	33	38		56	57	65
11	20	SSG	120	107	155	166	106	167	203
11	21	SSG	106	94	118	152	138	109	122
11	22	SSG	191	174	209	241	246	138	170
11	23	PM	125	97	167		334	292	183
11	24	PM	111	98	151		126	135	151
11	25	SSG	101	88	141	201	118	295	201
11	26	Combination	161	229	296		288	333	227
11	27	PM	93	80	165		204	52	
11	28	Combination	47	67	73		88	102	113
11	29	PM	90	91	101		143	150	126
11	30	PM	110	105	202		174	153	214
11	31	SSG	138	273	211	113	162	240	168
11	32	Combination	119	171	179		194	244	226
11	33	SSG	104	122	56	54	135	179	157
11	34	PM	110	95	123		128	152	118
11	35	SSG	139	115	145	152	133	90	150
11	36	SSG	50	86	70	79	134	155	131
11	37	Combination	59	82	143		197	176	142
11	38	PM	881	725	933		874	888	531
11	39	PM	84	95	104		127	163	133
11	40	PM	129	1	1			195	208
11	41	Combination	190	216	270		257	226	155
11	42	Combination	104	161	192		240	172	178
11	43	SSG	84	67	122	96	84	114	110
11	44	PM	125	135	152		138	151	156
11	45	Combination	131	108	157		167	136	128
11	46	SSG	137	183	264	206	350	552	323

Centre Number	Patient Number	Treatment	Platelets (x 10^3/µL) Baseline	Day 7	Day 14	Day 21	EOT	3 Mon FU	6 Mon FU
11	47	SSG	79	92	107	89	169		
11	48	Combination	84	102	115		157		180
11	49	SSG	51	141	224	177	188	129	113
11	50	PM	46	43	86		124	137	121
11	51	Combination	52	150	173		178	120	182
11	52	PM	56	53	62		67	82	97
11	53	SSG	92	74	102	187	209	210	165
11	54	PM	120	113	165		204	188	192
11	55	PM	108	94	152		162	131	95
11	56	SSG	84	86	220	181	220	181	218
11	57	Combination	106	94	226		242	168	104
11	58	Combination	69	101	163		244	270	197
11	59	PM	43	49	210		307	253	161
11	60	Combination	85	98	170		126	190	96
11	61	PM	83	62	125		141	115	150
11	62	SSG	45	33	147	241	219	207	173
11	63	SSG	89	99	127	144	144	146	125
11	64	SSG	46	108	133	200	297	186	150
11	65	PM	173	162	172		214		
11	66	Combination	66	103	253		422	217	243
11	67	PM	91	86	166		188	234	308
11	68	SSG	58	141	152	243	218	255	225
11	69	Combination	75	61	79		108	155	184
11	70	Combination	54	77	106		148	134	123
11	71	Combination	76	53	58		75	74	86
11	72	SSG	168	100	132			324	366
11	73	PM	40	49	68		102	97	133
11	74	PM	142	182	205		267	230	216
11	75	Combination	86	120	192		269	158	199
11	76	PM	40	93	99		184	176	191
11	77	PM	111	95	239		196	188	221
11	78	SSG	41	63	59	88	87	131	140
11	79	PM	86	56	94		232	211	270
11	80	PM	62	55	233		203	279	230
11	81	Combination	63	64	165		167	339	207
11	82	SSG	40	82	184	102	288	234	256
11	83	SSG	90	85	101	131	172	122	125
11	84	SSG	46	135	292	336	320	518	303
11	85	SSG	82	86	135	181	200	103	
11	86	Combination	41	65	184		266	262	237
11	87	Combination	83	204	102		147	231	255
11	88	PM	88	79	110		144	191	152
11	89	Combination	109	121	176		226	382	252
11	90	Combination	40	61	110		105		
11	91	PM	41	158	237		289	135	238
11	92	Combination	99	118	179		207	273	264

Centre Number	Patient Number	Treatment	Platelets (x 10^3/μL) Baseline	Day 7	Day 14	Day 21	EOT	3 Mon FU	6 Mon FU
11	93	SSG	79	137	138	166	86	169	151
11	94	SSG	93	226	325	317	423	594	381
11	95	SSG	114	172	211	194	222	201	249
11	96	Combination	125	244	324		401	334	270
11	97	Combination	89	183	192		199	218	199
11	98	PM	75	103	290		202	266	197
11	99	SSG	43	100	208	214	201	176	167
11	100	PM	152	188	212		246		
11	101	PM	67	65	74		85	94	132
11	102	Combination	66	152	269		260	239	207
11	103	Combination	97	134	225		246	142	156
11	104	PM	47	114	256		403	209	192
11	105	SSG	43	55	167	142	109	182	194
11	106	Combination	112	338	267		222	155	149
11	107	PM	40	74	164		286	206	219
11	108	PM	96	69	119		181	102	130
11	109	SSG	43	149	197	209	234	201	185
11	110	PM	58	38	55		85	137	147
11	111	Combination	51	190	221		219	152	196
11	112	Combination	127	95	241		238	130	193
11	113	SSG	84	168	227	182	268	181	
11	114	SSG	215	154	189	169	172	218	152
11	115	Combination	42	66	92		113	195	158
11	116	SSG	99	122	119	198	245	189	224
11	117	PM	55	60	147		221	240	
11	118	PM	66	102	161		202	168	144
11	119	SSG	94	197	184	176	177	136	170
11	120	Combination	63	175	200		179	175	244
11	121	Combination	69	195	283		288	327	433
11	122	SSG	41	47	161	235	241		
11	123	SSG	70	113	144	129	170	194	189
11	124	SSG	44	65	192	171	177		260
11	125	SSG	167	152	283	280	238	195	187
11	126	Combination	163	104	140		201		109
11	127	PM	62	58	136		185	227	203
11	128	Combination	174	73	288		300	193	132
11	129	PM	130	62	134		213	173	187
11	130	Combination	73	172	227		214	197	230
11	131	PM	117	91	156		163	198	134
11	132	SSG	42	150	131	149	169	196	88
11	133	Combination	56	50	138		182	238	225
11	134	PM	45	22	57		58	110	84
11	135	PM	111	144	227		217	285	246
12	241	Combination		93	158		96		258
12	242	SSG		162	362	341	332	66	357
12	243	PM		105	167		248	206	276

Centre Number	Patient Number	Treatment	Baseline	Platelets (x 10^3/µL) Day 7	Day 14	Day 21	EOT	3 Mon FU	6 Mon FU
12	244	SSG		117	121	177	165	186	209
12	245	PM	60	105	119		143	93	
12	246	PM	210	140	268		222	191	
12	247	PM	44	71	119		110	206	255
12	248	PM	48	59	117		236	188	168
12	249	Combination	67	108	103		103	174	115
12	250	SSG	52	46	53	104	150	135	143
12	251	Combination	91	143	171		228	211	279
12	252	SSG	85	188	181	219	170	183	172
12	253	SSG	75	104	113	177	150	142	166
12	254	Combination	68	164	195		239	228	182
12	255	Combination	53	98	190		238	207	198
12	256	Combination	66	131	150		234	186	138
12	257	SSG	76	155	195	188	303	263	104
12	258	SSG	107	145	241	226	175		202
12	259	PM	68	107	169		223	233	349
12	260	PM	85	138	168		198		128
12	261	Combination	69	238	283		283	260	189
12	262	PM	164	102	194		292	222	225
12	263	SSG	121	172	229	173	213	272	253
12	264	Combination	59	200	217		128	255	164
12	265	SSG	55	102	134	97	53		
12	266	PM	104	128	127		115	130	148
12	267	SSG	189	88	165	136	179	56	133
12	268	PM	57	54	60		86	136	126
12	269	Combination	95	113	208		153	135	148
12	270	Combination	67	132	120		289		237
12	271	SSG	71	110	144	137	214	239	229
12	272	PM	130	106	228		307	205	224
12	273	SSG	118	159	239	217	267	290	252
12	274	SSG	152	157	225	226	221	200	169
12	275	Combination	53	96	98		98	186	207
12	276	PM	101	94	109		162	219	177
12	277	Combination	96	128	300		406	342	273
12	278	SSG	93	129	195	202	234	176	
12	279	PM	104	123	217		253	172	199
12	280	SSG	41	66	126	216	179	138	191
12	281	Combination	66	175	289		295	249	258
12	282	PM	108	180	240		274	203	197
12	283	PM	72	60	75		127	144	146
12	284	Combination	111	117	253		309	97	137
12	285	Combination	109	165	293		240	167	323
12	286	PM	87	53	107		140	172	190
12	287	SSG	184	276	288	228	221	239	205
12	288	Combination	189	182	361		236	229	200
12	289	Combination	95	149	210		305	259	239

Centre Number	Patient Number	Treatment	Platelets (x 10³/µL) Baseline	Day 7	Day 14	Day 21	EOT	3 Mon FU	6 Mon FU
12	290	SSG	88	151	245	285	191		262
12	291	SSG	103	124	149	155	181	172	219
12	292	PM	76	101	308		180		183
12	293	SSG	85	100	265	229	250		207
12	294	Combination	120	297	138		162	192	204
12	295	Combination	123	252	286		255		240
12	296	PM	122	139	245		242	218	237
12	297	SSG	108	262	245				
12	298	PM	71	141	198		236	224	276
12	299	Combination	131	136	179		174		170
12	300	PM	85	87	143		168	160	178
12	301	SSG	155	296	291	323	300	270	250
12	302	SSG	180	180	228	258	252	347	359
12	303	PM	84	101	112		77	92	350
12	304	Combination	85	122	224		228	252	221
12	305	PM	144	181	226		228	300	278
12	306	Combination	78	210	254		312	331	218
12	307	Combination	96	144	158		223	194	214
12	308	PM	136	140	151		185	151	257
12	309	PM	113	93	153		175	165	181
12	310	SSG	205	261	228	136	140	211	280
12	311	SSG	132	202	115	202	191	266	267
12	312	Combination	166	100	189		218	226	252
12	313	PM	144	142	231		317	242	261
12	314	SSG	81	116	110	122	136	213	149
12	315	Combination	91	70	177		254	118	133
12	316	SSG	127	156	207			201	157
12	317	SSG	125	182	225	193	242		243
12	318	Combination	110	220	451		407	218	355
12	319	Combination	94	114	215		214	251	260
12	320	Combination	80	198	284		320	280	317
12	321	Combination	123	108	104		151	138	165
12	322	PM	95	103	158		180	178	107
12	323	SSG	71	112	120	144	163	180	187
12	324	SSG	122	108	163	165	191	190	168
12	325	PM	89	98	143		147	159	176
12	326	PM	120	111	197		225	125	178
12	327	Combination	100	341	372		286	261	291
12	328	PM	63	72	100		143	209	220
12	329	SSG	81	159	200	194	283	270	201
12	330	PM	209	181	202		220	316	167

Centre Number	Patient Number	Treatment	Platelets (x $10^3/\mu L$) Baseline	Day 7	Day 14	Day 21	EOT	3 Mon FU	6 Mon FU
23	361	Combination	70	137	100		195	223	195
23	362	PM	104	130	217		188	213	298
23	363	PM	128	157	305		433	288	306
23	364	Combination	106	110	187		179	92	233
23	365	SSG	138	203	265	297	267	143	211
23	366	Combination	129	144	244		243	280	255
23	367	SSG	97	92	101	109	104	111	132
23	368	PM	85	101	177		156	140	128
23	369	Combination	65	83	170		163	202	173
23	370	SSG	99	222	225	233	272	273	159
23	371	PM	86	115	167		155	118	157
23	372	PM	129	116	134		174	198	214
23	373	SSG	103	201	219	204	189	188	152
23	374	SSG	84	133	129	128	172	59	156
23	375	Combination	154	302	297		308	273	265
23	376	SSG	250	212	273	307	292	290	342
23	377	Combination	107	106					235
23	378	Combination	113	115	135		165	164	173
23	379	PM	201	132	165		213	153	232
23	380	SSG	83	80	141	153	141	160	194
23	381	PM	114	119	213		207	190	235
23	382	Combination	87	142	226		210	189	161
23	383	PM	153	186	321		281	253	226
23	384	SSG	118	168	165	159	169	169	207
23	385	SSG	148	141	139	152	175	166	232
23	386	Combination	150	75	213		320	279	273
23	387	Combination	115	82	102		110	122	190
23	388	PM	121	162	220		228	255	202
23	389	SSG	90	218	159	154	267	170	425
23	390	PM	81	126	201		220	152	237
23	391	Combination	159	223	204		215	194	195
23	392	Combination	119	72	139			246	77
23	393	SSG	149	184	254	305	334	283	231
23	394	PM	160	209	229		387	2.54999995	418
23	395	PM	109	76	110		101	138	202
23	396	SSG	95	242	193	152	181	132	181
23	397	SSG	267	414	368	216	195	166	283
23	398	Combination	91	162	187		219	248	186
23	399	PM	86	110	147		249	237	282
23	400	PM	159	84	91		89	131	93
23	401	Combination	114	54	93		103	96	159
23	402	Combination	81	88	165		123	179	243
23	403	SSG	89	77	127	108	181	76	54
23	404	SSG	113	136	226	274	256		276
23	405	PM	153	160	194		189	165	200
34			Not Done						

Centre Number	Patient Number	Treatment	Platelets (x 10^3/µL) Baseline	Day 7	Day 14	Day 21	EOT	3 Mon FU	6 Mon FU
35	646	PM	48	57	150		143		236
35	647	PM	5	338	129		151	144	296
35	648	Combination	61	122			215	331	250
35	649	Combination	108	167			197	155	210
35	650	SSG	188	246	275	279	463	278	280
35	651	SSG	247	251	204	108	261	334	312
35	652	PM	110	194	204		251		282
35	653	Combination	139	146	217		230	262	270
35	654	SSG	210	343	470	373	461	448	503
35	655	Combination	110	165	188		210	179	250
35	656	SSG	53	55	79	175	314	219	210
35	657	PM	125	127	174		148	234	303
35	658	Combination	163	197	233		254	284	263
35	659	SSG	149	138	197	173	216	198	202
35	660	PM	123	146	156		121	122	213
35	661	Combination	124	131	280		273	210	296
35	662	PM	58	85	97		126	132	278
35	663	SSG	172	142	153	110	145	210	663
35	664	Combination	105	233					275
35	665	SSG	104	123	452	391	284	210	230
35	666	SSG	64	83	206	191	174	264	576
35	667	Combination	90	85	230		208	301	290
35	668	PM	58	67	154		119		401
35	669	PM	153	164	147		105		
35	670	SSG	82	85	166	106	204	301	247
35	671	PM	74	8.2	123		98		286
35	672	Combination	88	83	202		200		210
35	673	SSG	293	551	156	160	164	204	276
35	674	Combination	60	55	200		201	268	302
35	675	PM	199	147	178		278		294
35	676	PM	59	56	69		156	192	205
35	677	SSG	141	95					
35	678	Combination	152	160	165		426	305	280
35	679	SSG	91	260	481	123	565	204	236
35	680	Combination	177	255	260		437	310	293
35	681	Combination	61	168	225		227	220	261
35	682	PM	65	132	135		160		297
35	683	Combination	376	567	510		518		410
35	684	SSG	84	111	120	427	173	210	312
35	685	PM	176	125	166		427	317	281
35	686	PM	88	150	117		380	240	205
35	687	SSG	147	202	329	274	535	387	394
35	688	PM	99	183	212		184	210	360
35	689	Combination	164	96	201		219	230	210
35	690	SSG	77	431	210	331	263	303	360

Centre Number	Patient Number	Treatment	Prothrombin time (s) Baseline	Day 7	Day 14	Day 21	EOT	3 Mon FU	6 Mon FU
11	20	SSG			14.9				
11	23	PM		38.9					
11	24	PM		57.5					
11	25	SSG		51.2					
12			Not Done						
23	361	Combination	15.4						
23	362	PM	16.8						
23	363	PM	16.2						
23	364	Combination	15.6						
23	365	SSG	16.3						
23	367	SSG	19.2						
23	368	PM	16.8						
23	370	SSG	15.5						
23	371	PM	21						
23	372	PM	15.9						
23	373	SSG	16.2						
23	374	SSG	16.5						
23	375	Combination	15.5						
23	376	SSG	17.5						
23	377	Combination	15.3						
23	378	Combination	15.5						
23	379	PM	15.7						
23	380	SSG	14.4						
23	381	PM	15.2						
23	382	Combination	16.3						
23	383	PM	13.7						
23	384	SSG	16						
23	385	SSG	17.4						
23	386	Combination	16						
23	387	Combination	16.3						
23	388	PM	14.8						
23	389	SSG	15.9						
23	390	PM	15						
23	391	Combination	13.21						
23	392	Combination	13.54						
23	393	SSG	15.1						
23	394	PM	14.9						
23	395	PM	14.9						
23	396	SSG	15.1						
23	397	SSG	12.49						
23	398	Combination	15.09						
23	399	PM	14.1						
23	400	PM	16						

Centre Number	Patient Number	Treatment	Prothrombin time (s) Baseline	Day 7	Day 14	Day 21	EOT	3 Mon FU	6 Mon FU
23	401	Combination	14.22						
23	402	Combination	14.3						
23	403	SSG	15.6						
23	404	SSG	14.6						
23	405	PM	15.3						
34			Not Done						
35	646	PM	13	12	12		10		
35	647	PM	11	13	13		12	10	
35	648	Combination	13	12			12	10	11
35	649	Combination	12	13			12	11	11
35	650	SSG	12	11	12	10	12	11	11
35	651	SSG	13	12	12	11	11	10	10
35	652	PM	11	11	12		11		
35	653	Combination	12	13	11		12	11	11
35	654	SSG	11	11	13	12	12	11	11
35	655	Combination	12	13	12		11	10	11
35	656	SSG	12	12	13	11	12	10	11
35	657	PM	11	12	13		11	11	10
35	658	Combination	13	12	11		12	10	10
35	659	SSG	12	13	12	11	12	11	11
35	660	PM	11	12	12		12	11	11
35	661	Combination	13	12	11		11	11	11
35	662	PM	10	11	12		11	10	
35	663	SSG	12	12	11	12	11	11	11
35	664	Combination	13	13					
35	665	SSG		12	14	12	12	11	11
35	666	SSG	12	10	12	11	10	10	11
35	667	Combination	13	13	12		11	10	11
35	668	PM	13	10	11		9		11
35	669	PM	11	11	10		11		
35	670	SSG	12	10	11	11	11	10	11
35	671	PM	11	10	14		13		
35	672	Combination	12	11	12		11		11
35	673	SSG	11	10	13	12	11	12	11
35	674	Combination	13	12	12		11	11	12
35	675	PM	13	12	12		11		
35	676	PM	12	11	10		12	11	12
35	677	SSG	13	12					
35	678	Combination	11	11	12		13	12	12
35	679	SSG	10	10	10	10	11	12	12
35	680	Combination	10	10	13		13	12	11
35	681	Combination	13	11	11		12	11	12
35	682	PM	12	11	11		10		

Centre Number	Patient Number	Treatment	Prothrombin time (s) Baseline	Day 7	Day 14	Day 21	EOT	3 Mon FU	6 Mon FU
35	683	Combination	11	11	13		12		12
35	684	SSG	12	10	10	12	11	11	12
35	685	PM	13	13	10		12	12	12
35	686	PM	11	12	11		12	11	
35	687	SSG	11	10	12	11	11	11	11
35	688	PM	10	11	11		10	11	12
35	689	Combination	12	11	11		10	11	
35	690	SSG	12	10	12	11	12	11	12

Centre	Patient		Aspartate Aminotransferase (U/L)						
Number	Number	Treatment	Baseline	Day 7	Day 14	Day 21	EOT	3 Mon FU	6 Mon FU
11	1	PM	104	120	63		61	39	32
11	2	Combination	57	88	49		54	22	38
11	3	Combination	96	207	98		98	36	51
11	4	PM	44	31	44		63	38	26
11	5	PM	44	34	34		48	36	28
11	6	Combination	62	57	50		63	35	31
11	7	PM	64	35	36		40	23	16
11	8	SSG	51	42	69	37	42	20	28
11	9	SSG	107	72	70	59	68	104	81
11	10	SSG	60	39	44	129	47	29	19
11	11	Combination	50	55	58		58	51	152
11	12	Combination	55	65	57		62	28	28
11	13	SSG	25	46	39	41	33	18	17
11	14	SSG	25	91	25	56	60	46	41
11	15	PM	52	57				29	27
11	16	Combination	26	50	70		56	8	19
11	17	Combination	21	54	45		48	21	19
11	18	SSG	35	68	71	40	55	106	26
11	19	Combination	26	56	59		55	32	27
11	20	SSG	22	37	31	39	37	37	26
11	21	SSG	32	62	103	82	50	27	29
11	22	SSG	43	72	48	43	34	26	16
11	23	PM	76	54	65		51	34	25
11	24	PM	41	38	48		42	32	26
11	25	SSG	49	311	63	61	37	21	18
11	26	Combination	39	95	73		68	48	41
11	27	PM	17	30	29		31	23	
11	28	Combination	29	76	71		63	40	25
11	29	PM	25	51	40		42	22	22
11	30	PM	53	94	69		70	50	40
11	31	SSG	16	14	19	104	67	22	16
11	32	Combination	37	106	85		69	30	26
11	33	SSG	25	80	168	55	61	23	25
11	34	PM	25	36	38		42	31	34
11	35	SSG	25	41	45	42	43	30	36
11	36	SSG	44	17	37	36	52	22	28
11	37	Combination	92	114	66		128	33	38
11	38	PM	52	86	73		47	30	45
11	39	PM	70	71	81		51	30	38
11	40	PM	13	53	46			25	22
11	41	Combination	43	60	48		66	49	51
11	42	Combination	65	166	92		70	26	32
11	43	SSG	31	83	52	48	75	38	28
11	44	PM	29	23	30		27	23	23
11	45	Combination	25	69	58		61	26	26
11	46	SSG	39	55	65	48	49	26	68
11	47	SSG	45	110	67	43	42		
11	48	Combination	23	38	76		74		22
11	49	SSG	87	321	50	62	90	30	35
11	50	PM	33	59	38		52	35	42
11	51	Combination	29	26	38		31	24	31
11	52	PM	43	43	70		41	77	629
11	53	SSG	27	46	78	67	29	83	95
11	54	PM	59	58	57		67	36	39

Centre Number	Patient Number	Treatment	Aspartate Aminotransferase (U/L) Baseline	Day 7	Day 14	Day 21	EOT	3 Mon FU	6 Mon FU
11	55	PM	32	41	53		50	35	28
11	56	SSG	15	35	34	38	36	54	29
11	57	Combination	16	106	71		41	19	28
11	58	Combination	24	42	119		98	67	39
11	59	PM	38	30	52		55	466	224
11	60	Combination	68	145	69		103	41	57
11	61	PM	56	93	80		62	62	57
11	62	SSG	75	71	56	37	39	46	36
11	63	SSG	31	73	62	61	39	25	41
11	64	SSG	98	96	62	63	45	51	90
11	65	PM	31	37	21		40		
11	66	Combination	53	89	44		40	26	47
11	67	PM	42	50	50		74	39	38
11	68	SSG	51	48	40	56	40	38	38
11	69	Combination	40	63	81		48	34	59
11	70	Combination	44	123	113		76	40	46
11	71	Combination	39	91	71		98	70	83
11	72	SSG	82	157	178			45	30
11	73	PM	91	45	52		70	43	40
11	74	PM	55	153	79		31	34	23
11	75	Combination	52	82	77		97	45	34
11	76	PM	104	199	99		45	43	28
11	77	PM	43	53	81		78	48	35
11	78	SSG	52	37	62	55	53	30	29
11	79	PM	102	113	66		67	152	54
11	80	PM	21	26	55		52	37	35
11	81	Combination	13	34	74		82	31	39
11	82	SSG	44	158	46	121	107	32	37
11	83	SSG	62	39	38	33	49	47	24
11	84	SSG	61	53	53	49	56	33	48
11	85	SSG	37	136	68	57	93	39	
11	86	Combination	98	40	31		58	39	33
11	87	Combination	97	144	131		59	43	35
11	88	PM	84	85	98		59	26	37
11	89	Combination	104	132	71		63	32	35
11	90	Combination	104	42	36		82		
11	91	PM	85	39	58		74	18	49
11	92	Combination	81	60	78		57	53	44
11	93	SSG	74	98	46	36	49	47	71
11	94	SSG	76	110	55	56	53	42	50
11	95	SSG	49	120	79	55	34	23	34
11	96	Combination	76	164	74		72	29	30
11	97	Combination	36	75	33		65	52	61
11	98	PM	104	112	125		149	224	55
11	99	SSG	46	87	77	50	49	41	32
11	100	PM	26	33	50		55		
11	101	PM	95	86	70		75	61	58
11	102	Combination	51	68	46		52	31	36
11	103	Combination	110	173	85		80	36	39
11	104	PM	44	171	31		39	82	69
11	105	SSG	32	43	47	51	46	31	25
11	106	Combination	107	65	90		54	27	39
11	107	PM	76	43	42		40	35	32
11	108	PM	29	41	54		39	37	35

Centre Number	Patient Number	Treatment	Aspartate Aminotransferase (U/L) Baseline	Day 7	Day 14	Day 21	EOT	3 Mon FU	6 Mon FU
11	109	SSG	49	82	53	48	51	26	23
11	110	PM	73	98	93		114	212	39
11	111	Combination	75	130	81		57	24	26
11	112	Combination	41	55	38		30	46	44
11	113	SSG	109	188	107	102	117	34	
11	114	SSG	62	138	104	130	119	46	28
11	115	Combination	94	76	60		80	51	71
11	116	SSG	67	56	44	56	59	38	37
11	117	PM	76	70	57		56	27	
11	118	PM	37	54	127		78	43	48
11	119	SSG	74	65	47	38	46	44	52
11	120	Combination	57	86	43		42	36	32
11	121	Combination	71	55	37		41	50	38
11	122	SSG	109	54	35	34	38		
11	123	SSG	48	76	70	67	51	57	42
11	124	SSG	92	160	50	35	33		26
11	125	SSG	100	94	51	35	45	36	30
11	126	Combination	45	113	144		169		51
11	127	PM	58	85	147		68	28	34
11	128	Combination	48	130	120		152	50	46
11	129	PM	26	69	32		39	32	42
11	130	Combination	61	121	60		53	74	47
11	131	PM	63	72	48		71	41	76
11	132	SSG	97	72	39	49	47	40	38
11	133	Combination	48	68	54		48	30	26
11	134	PM	67	96	74		54	95	83
11	135	PM	26	47	60		63	65	42
12	241	Combination	37	54	151		186		37
12	242	SSG	16	19	58	44	32	37	54
12	243	PM	24	24	53		45	38	25.5
12	244	SSG	27	52	216	154	72	23.5	41
12	245	PM	40	21	41.1		39.5	30	
12	246	PM	68	74	49		63	128	
12	247	PM	42	29	24		31	37	37
12	248	PM	32	37	45.9		36.3	20	20
12	249	Combination	28	100	77		62	46	16.4
12	250	SSG	32	32.5	30	30	29	22.1	19.6
12	251	Combination	74	34	27		59	45	28.5
12	252	SSG	41	33	112	51	28	32	41
12	253	SSG	48	40	47	45	32	27	34.1
12	254	Combination	60	34	218		71	41	33
12	255	Combination	44.8	253	108		103	19	26
12	256	Combination	36	68.8	80		75	9.8	36
12	257	SSG	48	74.1	51	51	38	41	37.9
12	258	SSG	32	38	142	70	31		29
12	259	PM	20	32	43		40	43	26.6
12	260	PM	35	40	54		65		15
12	261	Combination	49	49	64		50.4	34	5
12	262	PM	32	39	60		49	26.7	30
12	263	SSG	28	122	81	48.2	77	30	17.2
12	264	Combination	11	33	88.2		122	54	10
12	265	SSG	14	38	50.4	36	326		
12	266	PM	48	56	44.1		35	52	20.2
12	267	SSG	42	85	75.3	61	51	39	31

Centre	Patient		Aspartate Aminotransferase (U/L)						
Number	Number	Treatment	Baseline	Day 7	Day 14	Day 21	EOT	3 Mon FU	6 Mon FU
12	268	PM	168	136	145		134	51.8	30.3
12	269	Combination	34.5	85	69		56	33	24
12	270	Combination	22	80.2	58		40		38.2
12	271	SSG	73	71	70	48	42	15.8	20
12	272	PM	144	182	188		415	28.4	10
12	273	SSG	14.8	18	108	68.8	113	36.6	15
12	274	SSG	39	74	76	79.3	86	68.1	49
12	275	Combination	78	63	57		67	15.2	20.6
12	276	PM	35	28	44		57	30.9	12
12	277	Combination	59	98	91		95	13.2	16
12	278	SSG	143	139	68	50	53.3	29.4	
12	279	PM	20	19	25		39	50.3	28
12	280	SSG	87	297	142	111	120	37.8	33
12	281	Combination	28	69.2	63		46	52.5	33.9
12	282	PM	38.3	43	44		74	18.1	23.6
12	283	PM	26.8	45.4	37.8		52.5	51	34.1
12	284	Combination	29.01	32	42		44	22	23.8
12	285	Combination	45.7	99.6	86		38.7	16.3	20.4
12	286	PM	15.2	18.8	21		26	24.3	20.1
12	287	SSG	81	126	136	118	68	45	40
12	288	Combination	33	71	58		67.4	38.8	32.3
12	289	Combination	19	28.9	51		78	47	31
12	290	SSG	20	67	54	70.8	77.3		48.2
12	291	SSG	27.2	41	49	48.5	39.5	31.4	19.6
12	292	PM	289	379	95.8		34		26.8
12	293	SSG	17.5	21	54.3	46.2	47.1		32.4
12	294	Combination	22.9	27.4	49.5		23	41.1	20.1
12	295	Combination	53.8	102	46.4		49.4		34
12	296	PM	36.5	58	57.8		62.2	30.8	41
12	297	SSG	71.5	70.4	56.8				
12	298	PM	37.8	41.6	37.1		32.1	28	39
12	299	Combination	68.4	83.9	94.6		90.6		38
12	300	PM	26.3	27.4	31.2		33.2	86	36
12	301	SSG	26.1	82.8	46.2	30.9	72.4	23	19
12	302	SSG	36.4	47	31.8	26.4	24.1	44	39
12	303	PM	24.6	78.5	74.4		55.4	61	28
12	304	Combination	247	444	132		75.9	33	31
12	305	PM	21.3	47.1	37.2		71.4	33	35
12	306	Combination	21.4	26.3	29.3		26.9	37	35
12	307	Combination	56.3	21	27		40.7	95	42
12	308	PM	16.4	31.2	39.7		40.1	20	31
12	309	PM	47.3	30.7	26.7		52.1	34	29
12	310	SSG	33.4	30.3	36.4	32.1	35.5	47	29
12	311	SSG	55.5	85.8	131	53.5	63	41.5	34.7
12	312	Combination	12.8	103	42		49.7	46	12.8
12	313	PM	42.5	63.6	78.9		56.1	96.2	76
12	314	SSG	65.1	120	91.5	91.9	89.2	25	37
12	315	Combination	42.3	43.1	46.4		47.2	60	23
12	316	SSG	22.5	103	79.1			21	28
12	317	SSG	43.6	90.9	74.5	94	112		43
12	318	Combination	25.1	86.2	42.8		66.9	50	68
12	319	Combination	31.2	36.2	40.2		60	54	46.6
12	320	Combination	40.5	62.4	50		46	41	90
12	321	Combination	32.2	38.9	29		34	42	38

Centre Number	Patient Number	Treatment	Aspartate Aminotransferase (U/L) Baseline	Day 7	Day 14	Day 21	EOT	3 Mon FU	6 Mon FU
12	322	PM	37.2	55	144		62.5	35	80
12	323	SSG	65	59	96	90	45	31	78
12	324	SSG	33	103	56	73	32	43	70
12	325	PM	37	54	110		69	20	98
12	326	PM	57	93	36		56	91.4	94
12	327	Combination	45	81	50		90	30	91
12	328	PM	28	40	62		84	38	43
12	329	SSG	44	65	59	81	72	34	33
12	330	PM	86	110	98		91	52	45
23	361	Combination	36	60	103		67	40	37
23	362	PM	69	89	162		146	63	76
23	363	PM	46	126	113		78	48	37
23	364	Combination	72	124	93		88	36	50
23	365	SSG	49	94	90	70	35	67	24
23	366	Combination	66	190	68		40	43	21
23	367	SSG	73	90	43	108	67	56	49
23	368	PM	78	19	110		36	16	18
23	369	Combination	60	78	50		46	37	36
23	370	SSG	83	92	46	60	92	47	46
23	371	PM	42	15	49		30	27	11
23	372	PM	31	61	82		118	42	31
23	373	SSG	65	48	40	48	41	26	34
23	374	SSG	42	92	59	49	35	76	50
23	375	Combination	15	85	53		49	18	32
23	376	SSG	32	61	107	57	74	52	41
23	377	Combination	44	311					22
23	378	Combination	59	63	61		55	29	49
23	379	PM		45	64		65	33	35
23	380	SSG	94	40	97	73	44	57	34
23	381	PM	89	51	70		87	32	30
23	382	Combination	50	74	127		64	26	33
23	383	PM	58	46	68		46	39	42
23	384	SSG	62	76	67	47	53	54	52
23	385	SSG	89	88	95	42	88	39	41
23	386	Combination	83	283	42		58	43	36
23	387	Combination	50	32	99		75	31	35
23	388	PM	76	38	67		53	53	39
23	389	SSG	50	74	77	71	47	37	29
23	390	PM	55	40	47		51	30	17
23	391	Combination	108	136	115		78	48	37
23	392	Combination	62	122	57			70	49
23	393	SSG	20	36	41	41	39	65	69
23	394	PM	100	97	40		56	27	27
23	395	PM	53	43	57		54	38	16
23	396	SSG	77	69	86	58	73	61	42
23	397	SSG	64	68	86	67	79	32	36
23	398	Combination	34	29	46		36	29	22
23	399	PM	32	13	31		45	35	20
23	400	PM	49	31	42		56	60	59
23	401	Combination	34	41	54		56	36	20
23	402	Combination	37	25	42		53	32	23
23	403	SSG	113	57	93	85	71	52	67
23	404	SSG	62	99	82	52	37		38
23	405	PM	40	15	37		39	30	34

Centre Number	Patient Number	Treatment	Aspartate Aminotransferase (U/L) Baseline	Day 7	Day 14	Day 21	EOT	3 Mon FU	6 Mon FU
34	451	Combination	55	58.6	42.3		47.8		29.3
34	452	PM	92.6	66.6	77.2		54.2		
34	453	PM	76.7	97.9	67		104		
34	454	Combination	48.1	57.8	52.4		76.4		34.3
34	455	PM	27.7	36.8	44.9		57.2		
34	456	SSG	114	101	50.5	47.7	37.7	49.9	
34	457	Combination	39.3	81.4	44		51.8		
34	458	PM	42.1	47.2	29.1		56.5	161	
34	459	Combination	47.7	91	86.7		76.2	26.8	42
34	460	PM	54.3	74.4	60		71.8		
34	461	SSG	41.2	66.7	105				
34	462	SSG	73.6	48.5	64.8	54.3	50.5		36.3
34	463	Combination	72.6	85.2	58.1		77.1		29.4
34	464	SSG	52.1	57.2	62.9	37.7	34.7		23.1
34	465	SSG	63.9	79	37.8	39.3	43.9		18.9
34	466	Combination	20.2	65.4	61.2		38.3		
34	467	PM	63.4	158	209		122	302	
34	468	Combination	86.9	90.1	39.4		38.4		24.9
34	469	SSG	23.6	56.3	95.6	55	39.3		28.6
34	470	Combination	25.3	37.4	60.6		59.3		29.5
34	471	PM	85.3	88.4	92.8		164		
34	472	SSG	72.9	116	61.9	72.4	59.7		32.5
34	473	PM	115	87.7	108		157		
34	474	Combination	28.2	48.2	53.6		59.4		24.7
34	475	PM	26.5	28	36.8		29.7		
34	476	PM	43.4	64.9	315		84.4	90.9	
34	477	SSG	89.5	116	52.6	49.4	45.3		29.2
34	478	Combination	39.6	58.1	42		54.6		24.9
34	479	SSG	63.8	72.8	56.1	47.4	94.5		35.7
34	480	SSG	106	190	99.8	83.8	61		27.8
34	481	Combination	77.6	109	54.7		62.9		20.5
34	482	Combination	118	424	71.7		67.3		22.5
34	483	PM	12.7	31.7	38.9		31.8		15.7
34	484	PM	28.6	84.5					
34	485	Combination	69.4	104	91.9		75.6		46.4
34	486	Combination	13.2	52.2	39		41.7		54.1
34	487	SSG	58.4	188	62.7	55.4	49.8		26.3
34	488	SSG	19.6	46.6	40.5	51.1	50.9	24.9	85.1
34	489	SSG	22.3	49.5	52.6	26.8	41.1		32.7
34	490	PM	82.4	139	93.1		65.9		27
34	491	PM	26.9	57.3	49.6		70.1		
34	492	PM	29.3	72.3	92.7		147		73.2
34	493	SSG	18.6	41.9	37.5	39.1	31.8		
34	494	Combination	124	101	44.6		41.6		30.8
34	495	SSG	57.6	60.8	54.7	106	68.2		36.8
34	496	SSG	37.5	181	58.2	47.9	46		21.3
34	497	PM	70.8	37.2	33.5		35.9	56.2	
34	498	SSG	19.2	71.9	57.6	100	86.8		22.9
34	499	Combination	57.2	58.8	50.2		79.9		32.7
34	500	PM	52.5	82.3	77.4		71.1		26.1
34	501	Combination	31.2	102	56.1		37.7		79.2
34	502	Combination	101	102	33.2		26.1		11.9
34	503	PM	67.1	117	75.6		85.6		
34	504	Combination	30.7	44.6	58.9		79.6		

Centre Number	Patient Number	Treatment	Aspartate Aminotransferase (U/L) Baseline	Day 7	Day 14	Day 21	EOT	3 Mon FU	6 Mon FU
34	505	SSG	72.9	63.1	49.5	36.3	32.8		44.5
34	506	SSG	98.2	54.8	55.3	43	34.4		51.7
34	507	SSG	81.7	114	54.4	56.7	80.9		52.7
34	508	PM	66.9	121	48.2		75.1		
34	509	PM	33.9	22.4	22.3		19.6		
34	510	Combination	72.4	78.7	45.3		45		55.9
34	511	Combination	28	57.3	59.1		56.1		33.6
34	512	PM	103	115	47.1		49.3		
34	513	Combination	15.8	22.5	29		25	42.3	29.8
34	514	SSG	63.7	104	93.8	77.4	77.1		40.4
34	515	SSG	56.2	43.2	54.3	32.2	29.9	25.9	
34	516	PM	25.1	34.6	36.9		39.1		18.6
34	517	PM	47.8	223	191		93.4		
34	518	SSG	52.3	48.5	52.8	45.3	40.9		46.4
34	519	PM	59.9	65.7	60.3		55.7	130	
34	520	SSG	27.7	133	40.1	69.2	49.3		18.8
34	521	PM	63.7	45.1	74.1		86.8		
34	522	Combination	24.1	66.4	68.2		71.3		
34	523	SSG	54.5	79.1	74	56.3	78.1		40.3
34	524	Combination	45.8	58.3	64.6		84.1		50
34	525	Combination	103	191	73.3		71.2		31
34	526	SSG	54.7	105	42.9	76.3	110		52.4
34	527	Combination	51.1	104	36.3		48		18.7
34	528	PM	23	39.8	41.6		43.2		
34	529	Combination	95.8	51.2	34.8		39.1		29.9
34	530	Combination	116	70.6	61.2		53.5		31.6
34	531	SSG	41.2	94.4	57.9	81.7	88.2		
34	532	Combination	96	65.9	36.6		37.6		42.7
34	533	SSG	38.5	54.5	52.7	64.2	52.1		23.6
34	534	PM	122	129	128		78.1		
34	535	PM	58.5	66.4	43.3		40.2	34.2	
34	536	PM	48.3	34.6	37.7		47.4		31
34	537	SSG	77.8	76.9	47.1	68.8	65.6		37.2
34	538	PM	74	33	39.2		70.6		
34	539	Combination	26.7	82.8	77.1		70.8		30.7
34	540	SSG	108	131	52.1	71.5	118		31.7
35	646	PM	12	12	14		12		20
35	647	PM	8	12	15		14	38	18
35	648	Combination	18	18			16	27	18
35	649	Combination	9	20			14	32	19
35	650	SSG	20	18	16	12	16	24	22
35	651	SSG	19	19	16	14	12	30	24
35	652	PM	10	10	16		14		14
35	653	Combination	20	18	17		12	29	20
35	654	SSG	20	19	20	16	14	27	22
35	655	Combination	12	12	15		16	27	20
35	656	SSG	16	9	16	18	16	27	28
35	657	PM	12	14	12		16	41	28
35	658	Combination	6	10	14		16	34	22
35	659	SSG	12	14	17	14	18	20	18
35	660	PM	9	14	12		16	32	24
35	661	Combination	12	10	16		14	24	20
35	662	PM	16	14	18		16	24	12.2
35	663	SSG	17	16	17	18	16	32	30

Centre Number	Patient Number	Treatment	Aspartate Aminotransferase (U/L) Baseline	Day 7	Day 14	Day 21	EOT	3 Mon FU	6 Mon FU
35	664	Combination	16	18					14
35	665	SSG	18	20	18	16	12	26	20
35	666	SSG	16	14	18	12	18	38	30
35	667	Combination	10	18	12		16	24	22
35	668	PM	12	18	18		16		30
35	669	PM	16	14	10		14		
35	670	SSG	14	14	16	18	17	24	26
35	671	PM	14	18	12		15		13
35	672	Combination	18	16	16		18		24
35	673	SSG	16	12	14	15	18	26	20
35	674	Combination	18	16	18		15	19	20
35	675	PM	18	14	16		15		11
35	676	PM	16	14	15		16	28	24
35	677	SSG	18	16					
35	678	Combination	20	17	18		16	20	20
35	679	SSG	18	16	17	15	16	20	18
35	680	Combination	20	20	14		16	24	26
35	681	Combination	28	21	19		19	26	18
35	682	PM	18	14	15		16		12
35	683	Combination	18	18	18		17		20
35	684	SSG	18	16	22	24	23	34	26
35	685	PM	17	16	20		18	26	21
35	686	PM	18	19	26		24	29	20
35	687	SSG	20	19	24	22	39.4	37.2	22
35	688	PM	26	24	22		23	34	22
35	689	Combination	26	22	23		24	30	24
35	690	SSG	27	26	24	25	40.1	36	24

Centre Number	Patient Number	Treatment	Alanine Aminotransferase (U/L) Baseline	Day 7	Day 14	Day 21	EOT	3 Mon FU	6 Mon FU
11	1	PM	28	36	28		31	29	21
11	2	Combination	9	52	31		40	18	13
11	3	Combination	13	85	56		56	38	44
11	4	PM	25	35	72		92	38	28
11	5	PM	27	27	26		37	20	17
11	6	Combination	28	27	39		43	38	34
11	7	PM	28	21	23		24	25	11
11	8	SSG	19	37	58	36	45	16	24
11	9	SSG	81	56	52	37	34	69	81
11	10	SSG	29	34	49	113	48	6	12
11	11	Combination	47	95	55		41	45	108
11	12	Combination	24	52	33		35	12	18
11	13	SSG	12	27	23	20	18	14	10
11	14	SSG	22	92	22	40	40	30	32
11	15	PM	15	17				22	18
11	16	Combination	38	82	111		61	7	13
11	17	Combination	22	82	63		56	14	18
11	18	SSG	34	61	82	52	46	115	27
11	19	Combination	17	28	37		39	19	20
11	20	SSG	15	35	28	26	23	22	17
11	21	SSG	21	36	72	71	34	17	17
11	22	SSG	29	53	46	29	25	16	12
11	23	PM	71	48	67		58	30	20
11	24	PM	18	23	41		31	17	13
11	25	SSG	26	143	68	62	33	17	30
11	26	Combination	30	66	49		39	60	38
11	27	PM	6	11	23		17	8	
11	28	Combination	17	37	67		61	20	17
11	29	PM	18	39	36		41	12	13
11	30	PM	43	58	60		70	37	20
11	31	SSG	12	11	11	77	45	15	26
11	32	Combination	38	66	82		66	47	32
11	33	SSG	28	97	199	31	94	23	21
11	34	PM	18	20	24		28	23	30
11	35	SSG	22	36	24	23	16	20	35
11	36	SSG	18	11	16	22	44	17	23
11	37	Combination	44	59	35		187	28	35
11	38	PM	40	114	86		38	19	30
11	39	PM	73	72	57		43	25	22
11	40	PM	17	22	23			23	19
11	41	Combination	35	42	37		39	42	34
11	42	Combination	47	143	107		66	17	24
11	43	SSG	35	74	73	41	42	31	20
11	44	PM	15	14	34		19	17	24
11	45	Combination	26	54	42		36	21	26
11	46	SSG	33	50	47	36	31	19	36
11	47	SSG	26	80	43	28	26		
11	48	Combination	24	19	51		43		16
11	49	SSG	38	174	83	51	72	37	27
11	50	PM	12	23	14		27	29	33
11	51	Combination	9	15	22		15	15	22
11	52	PM	28	18	38		30	59	477
11	53	SSG	14	19	43	41	21	82	56
11	54	PM	27	22	30		44	35	36

Centre Number	Patient Number	Treatment	Alanine Aminotransferase (U/L) Baseline	Day 7	Day 14	Day 21	EOT	3 Mon FU	6 Mon FU
11	55	PM	15	20	30		39	31	19
11	56	SSG	10	9	13	17	17	54	45
11	57	Combination	7	40	48		27	15	31
11	58	Combination	12	20	104		96	110	32
11	59	PM	10	12	42		57	505	258
11	60	Combination	49	123	66		66	41	39
11	61	PM	37	53	56		64	35	51
11	62	SSG	42	40	26	19	19	46	29
11	63	SSG	28	60	65	59	32	19	27
11	64	SSG	28	37	29	28	18	34	52
11	65	PM	21	11	17		31		
11	66	Combination	8	33	20		21	29	28
11	67	PM	10	23	25		49	32	24
11	68	SSG	49	32	27	47	39	21	42
11	69	Combination	16	43	77		45	37	28
11	70	Combination	13	50	39		31	18	23
11	71	Combination	14	24	20		29	29	32
11	72	SSG	44	50	42			30	22
11	73	PM	33	26	30		52	38	21
11	74	PM	54	121	158		53	36	32
11	75	Combination	19	40	42		54	29	30
11	76	PM	71	122	115		43	37	31
11	77	PM	15	19	88		79	35	26
11	78	SSG	23	30	56	54	47	27	34
11	79	PM	31	31	40		49	91	38
11	80	PM	13	11	49		46	47	41
11	81	Combination	10	15	48		60	23	26
11	82	SSG	16	71	35	83	77	26	29
11	83	SSG	68	59	36	25	34	84	32
11	84	SSG	38	37	49	56	39	48	62
11	85	SSG	23	82	37	29	24	27	
11	86	Combination	25	23	21		25	22	22
11	87	Combination	28	76	78		73	34	32
11	88	PM	76	91	112		74	35	44
11	89	Combination	69	80	43		35	33	25
11	90	Combination	18	19	28		51		
11	91	PM	27	43	58		96	15	33
11	92	Combination	31	41	88		46	65	33
11	93	SSG	57	66	36	25	31	33	64
11	94	SSG	31	64	37	38	15	34	26
11	95	SSG	38	350	124	58	27	21	23
11	96	Combination	51	109	87		65	34	19
11	97	Combination	40	37	25		35	29	35
11	98	PM	31	32	39		31	176	24
11	99	SSG	19	69	60	52	35	34	28
11	100	PM	21	18	21		28		
11	101	PM	19	24	31		34	27	22
11	102	Combination	28	42	29		45	20	19
11	103	Combination	111	135	75		73	34	21
11	104	PM	21	90	24		28	69	61
11	105	SSG	12	17	19	24	20	19	16
11	106	Combination	51	36	38		21	17	16
11	107	PM	27	27	27		30	21	17
11	108	PM	19	24	36		26	35	22

Centre Number	Patient Number	Treatment	Alanine Aminotransferase (U/L) Baseline	Day 7	Day 14	Day 21	EOT	3 Mon FU	6 Mon FU
11	109	SSG	38	79	35	42	31	24	21
11	110	PM	15	19	38		68	245	31
11	111	Combination	30	119	83		54	18	20
11	112	Combination	33	32	29		21	30	31
11	113	SSG	96	117	64	55	60	29	
11	114	SSG	83	147	86	103	77	38	27
11	115	Combination	24	33	37		47	24	26
11	116	SSG	33	43	43	30	31	31	37
11	117	PM	27	54	39		31	14	
11	118	PM	28	43	142		91	32	21
11	119	SSG	59	52	48	42	50	71	94
11	120	Combination	40	24	43		38	25	30
11	121	Combination	23	34	31		30	29	31
11	122	SSG	28	40	24	20	23		
11	123	SSG	26	50	66	58	38	28	30
11	124	SSG	27	44	22	20	16		18
11	125	SSG	31	56	35	27	30	22	18
11	126	Combination	29	48	74		113		25
11	127	PM	31	47	160		72	20	21
11	128	Combination	34	59	47		61	70	25
11	129	PM	13	16	24		29	15	25
11	130	Combination	17	47	31		30	34	21
11	131	PM	24	28	36		55	29	69
11	132	SSG	21	32	19	21	29	19	14
11	133	Combination	15	29	37		31	24	24
11	134	PM	18	28	33		28	40	54
11	135	PM	13	17	34		47	50	19
12	241	Combination	39	50	217		264		36
12	242	SSG	12	12	51	39	57	46	52
12	243	PM	33	15	33		31	32	26
12	244	SSG	20	53	210	144	73	16	19
12	245	PM	14.8	18	39		63	19	
12	246	PM	74	81	68		70	50	
12	247	PM	38	31	22		37	15	26
12	248	PM	11	30	54.7		61.4	19	21
12	249	Combination	39	38	27		51	31	7.9
12	250	SSG	50	17	15.3	12	28.5	15	18.3
12	251	Combination	70	46	74		53	39	27.2
12	252	SSG	33	41	142	37	36	33	34
12	253	SSG	40	36	38	22	27	29	24
12	254	Combination	33	28	93		51	21	30.8
12	255	Combination	17	114	65		81	14	22
12	256	Combination	24	53	74		43	8.8	28
12	257	SSG	14	54.7	29	27	21	31	34.4
12	258	SSG	14	24	154	83	49		33
12	259	PM	16	41	50.5		35	47	19
12	260	PM	35	45	60		78		9.8
12	261	Combination	20	51	71		47.4	22	18.5
12	262	PM	31	65	64		62	19.3	21
12	263	SSG	41	124	97	54	66	19.5	21.2
12	264	Combination	11	25	66		52	44	66.4
12	265	SSG	10	16	27	39	18.5		
12	266	PM	43	78	71		32	42.9	29.8
12	267	SSG	40	61	66	58	53	30	40

Centre Number	Patient Number	Treatment	Alanine Aminotransferase (U/L) Baseline	Day 7	Day 14	Day 21	EOT	3 Mon FU	6 Mon FU
12	268	PM	74	70	101		85	47.5	25.6
12	269	Combination	11	35	47.7		31	12	19
12	270	Combination	10	38.2	36.9		23		34.5
12	271	SSG	43.9	31.9	47	41	89	16.1	15
12	272	PM	26	64	135		270	18.5	8
12	273	SSG	13.8	20	61	30.9	89	36.6	13
12	274	SSG	41	51	53	54.2	63	80.6	46
12	275	Combination	65	27	27		31	9.96	20.3
12	276	PM	15	10	23		33	17.8	9
12	277	Combination	33	64	67		58	11.3	14
12	278	SSG	54	74	41	28	25	18.8	
12	279	PM	12	12	21		23	31.8	24
12	280	SSG	57	140	76	67	84.2	32.8	34
12	281	Combination	14	47	51		22	44	25.2
12	282	PM	12	16	21		49.7	9.93	10.3
12	283	PM	20.5	39	38.8		53	42.5	38.2
12	284	Combination	8.08	20	33		25	16.9	6.3
12	285	Combination	31.5	56	64.2		48	17.3	16.3
12	286	PM	10.1	8.84	12		18.8	17.4	18.2
12	287	SSG	25.6	80	106	59	37.8	37	36
12	288	Combination	17.7	42	39		88.5	47.9	45.8
12	289	Combination	13.7	29.3	64.3		46	34	29
12	290	SSG	14.3	35	41	37	39.4		31.1
12	291	SSG	16.1	28	31	48.4	34.2	39.2	22.1
12	292	PM	134	201	108		26		15.1
12	293	SSG	5.45	18	48	41	31.6		28.4
12	294	Combination	5.13	14.1	22.2		7	24.2	23.4
12	295	Combination	23.7	42.2	22.1		30.7		29.2
12	296	PM	11.2	12.5	28.2		26.4	10.3	29
12	297	SSG	41.4	57.8	57.8				
12	298	PM	14.7	19.4	26.7		24.2	32	41
12	299	Combination	40.6	41.4	58.1		45.1		28
12	300	PM	20.9	22.3	26.4		65.1	72	41
12	301	SSG	34.2	39.4	29.4	36.1	40.3	20	21
12	302	SSG	28.4	18.2	39.8	15.7	19.3	34	19
12	303	PM	28.9	45.7	39.8		37.3	72	25
12	304	Combination	87.2	176	49.3		26.9	16	12
12	305	PM	18.4	20.3	19.4		24.3	18	17
12	306	Combination	23.1	24.2	20.2		21.4	24	9
12	307	Combination	33.4	29	31		37.4	94	37
12	308	PM	8	27.2	30.3		31.5	15	19
12	309	PM	21.6	26.2	30.1		45.2	26	56
12	310	SSG	23.6	24.5	43.2	34	30	27	24
12	311	SSG	38.2	51.7	54	26	24	29	29.3
12	312	Combination	7.8	62	52		47	31	7.8
12	313	PM	23	43.1	84.9		53.6	84.2	94
12	314	SSG	38.3	163	5	68.2	67	16	20
12	315	Combination	16.3	13.4	20.3		58	21	41
12	316	SSG	5	36	60.3			25	24
12	317	SSG	7.78	91.2	36.5	69	74		49
12	318	Combination	23.2	34	36		39	47	48
12	319	Combination	29.4	27	32		36	26	60
12	320	Combination	25	31.6	38		34	24	36
12	321	Combination	11.9	12.4	11		16	22	26

Centre Number	Patient Number	Treatment	Alanine Aminotransferase (U/L) Baseline	Day 7	Day 14	Day 21	EOT	3 Mon FU	6 Mon FU
12	322	PM	29.6	29	42		47.1	19	52
12	323	SSG	19	120	84	65	51	39	57
12	324	SSG	19	44	34	31	19	27	56
12	325	PM	38	55	95		83	26	66
12	326	PM	28	50	34		42	60.2	63
12	327	Combination	9	52	44		38	34	34
12	328	PM	8	68	29		60	36	29
12	329	SSG	14	20	26	12	22	21	24
12	330	PM	72	70	99		66	46	32
23	361	Combination	26	28	44		39	15	16
23	362	PM	101	54	164		176	18	87
23	363	PM	38	41	101		62	33	22
23	364	Combination	59	82	68		35	26	70
23	365	SSG	44	39	94	20	15	64	31
23	366	Combination	22	68	66		42	21	42
23	367	SSG	21	55	49	36	34	26	22
23	368	PM	45	35	75		34	22	10
23	369	Combination	32	36	27		21	14	28
23	370	SSG	24	56	30	26	49	21	41
23	371	PM	15	13	17		25	10	27
23	372	PM	35	50	72		124	21	34
23	373	SSG	65	61	64	52	32	14	32
23	374	SSG	23	56	32	29	18	61	21
23	375	Combination	49	68	32		27	12	12
23	376	SSG	20	36	107	48	48	15	31
23	377	Combination	11	284					23
23	378	Combination	14	25	57		53	14	125
23	379	PM	37	17	27		23	14	55
23	380	SSG	25	22	34	30	17	41	44
23	381	PM	42	33	46		94	20	43
23	382	Combination	37	38	93		47	24	24
23	383	PM	22	36	47		36	12	27
23	384	SSG	28	30	19	25	17	28	43
23	385	SSG	28	46	50	37	50	18	33
23	386	Combination	23	166	37		19	53	35
23	387	Combination	14	27	54		58	46	35
23	388	PM	29	34	35		45	25	30
23	389	SSG	23	61	61	45	35	4	20
23	390	PM	22	27	35		4	43	27
23	391	Combination	57	108	83		63	36	26
23	392	Combination	40	64	47			79	81
23	393	SSG	19	26	33	9	46	69	62
23	394	PM	13	21	11		31	34	21
23	395	PM	31	31	26		35	29	20
23	396	SSG	54	35	52	52	53	33	37
23	397	SSG	28	36	29	44	34	25	14
23	398	Combination	10	10	29		30	38	16
23	399	PM	19	39	17		24	45	25
23	400	PM	50	9	25		48	53	55
23	401	Combination	22	37	34		36	18	5
23	402	Combination	18	55	23		27	16	15
23	403	SSG	84	33	48	37	35	34	55
23	404	SSG	55	121	150	90	31		37
23	405	PM	15	35	15		25	15	28

Centre Number	Patient Number	Treatment	Alanine Aminotransferase (U/L)					
			Baseline	Day 7	Day 14	Day 21	EOT	3 Mon FU	6 Mon FU
34	451	Combination	8.48	35.9	38.1		37.7		21.2
34	452	PM	27.1	26.2	41.8		56.1		
34	453	PM	10.2	17.7	31.5		47.4		
34	454	Combination	9.55	31.1	39.9		31.5		22.8
34	455	PM	8.37	15.5	23.1		32		
34	456	SSG	59.3	59.9	42	35.7	29.8	39.6	
34	457	Combination	16.6	70.3	41.3		43.3		
34	458	PM	9.92	16.4	17.1		36.3	89.9	
34	459	Combination	21.6	80.8	111		81.1	20.8	52.7
34	460	PM	9.85	29.2	47.2		50.3		
34	461	SSG	15.6	30	31.4				
34	462	SSG	21.2	33.2	34.9	29.4	28.4		26.9
34	463	Combination	26.4	47.4	47.4		44.9		22.9
34	464	SSG	20.9	25.1	29.4	25.5	22		16.3
34	465	SSG	23.9	39.7	25.4	27.6	19.1		14.7
34	466	Combination	13.8	48.3	38.9		31.1		
34	467	PM	11.6	39.3	78.7		69.4	66.4	
34	468	Combination	59.9	77.2	36.1		25.7		19.4
34	469	SSG	12.8	29.2	32.8	19.7	22.2		27.1
34	470	Combination	12.8	15.4	30.4		34.6		14.9
34	471	PM	50.2	67.9	101		166		
34	472	SSG	32.6	78.3	41.3	40.5	33.3		22.8
34	473	PM	36.3	43.1	82.4		81.1		
34	474	Combination	15.5	23.6	27.1		31.3		18.5
34	475	PM	8.16	10.2	9.5		12.2		
34	476	PM	30.3	22.5	168		86.9	70	
34	477	SSG	21.8	54	28.7	21	15.7		19.2
34	478	Combination	8.3	28.1	25.3		28.1		12.2
34	479	SSG	15.7	51.2	32.7	27.7	37		17.8
34	480	SSG	97.6	155	105	87.5	65.9		23.1
34	481	Combination	19.9	43.7	31.4		33.8		18.2
34	482	Combination	120	442	74.5		49.3		21.8
34	483	PM	7.81	17.6	38.4		32.4		8.01
34	484	PM	13.9	21.1					
34	485	Combination	18.7	38.8	27.4		23.5		16.5
34	486	Combination	10.1	26	28		27.9		67.7
34	487	SSG	18	114	57.4	38.3	31.4		19.1
34	488	SSG	5	10.8	10.3	17.2	17.5	8.03	33
34	489	SSG	10.5	22	22.3	19.9	22.1		35.7
34	490	PM	18	37	41.3		40.3		17.5
34	491	PM	18.7	40.4	42.7		70.6		
34	492	PM	18.4	47	56.7		95.8		43.9
34	493	SSG	8.08	18.3	19.4	22.2	18.2		
34	494	Combination	58.1	62.9	30.2		26.9		13.4
34	495	SSG	17.9	38.1	33.7	69.6	45.1		28.7
34	496	SSG	27.3	130	61.9	39.4	43.3		24
34	497	PM	15.5	26.1	28.3		28.4	28.1	
34	498	SSG	21.5	49.3	31	95.5	99.6		19.5
34	499	Combination	22.9	27.3	32.7		41.7		37.3
34	500	PM	20.3	136	89		87.5		24
34	501	Combination	11.5	42.2	46.1		34.6		110
34	502	Combination	34	60	29.7		20.3		10.9
34	503	PM	27.6	41.3	38		50.6		
34	504	Combination	13.3	25	47.2		59		

Centre	Patient		Alanine Aminotransferase (U/L)						
Number	Number	Treatment	Baseline	Day 7	Day 14	Day 21	EOT	3 Mon FU	6 Mon FU
34	505	SSG	23.6	41.8	34.3	24.9	24.2		33.7
34	506	SSG	21.3	27.3	22.3	19.9	19.4		31.3
34	507	SSG	15.5	45.4	22.6	35.7	46.5		57
34	508	PM	7.8	16.7	16.9		33.6		
34	509	PM	15	14	12.3		15.4		
34	510	Combination	37.2	67.8	40.6		30.6		57.4
34	511	Combination	16.1	55.3	39.2		44.1		24
34	512	PM	37.4	43.9	34.5		30.3		
34	513	Combination	9.34	20.4	33		28.8	31.8	31
34	514	SSG	24.9	43.1	58.6	43.6	54.1		23.8
34	515	SSG	22.2	41.4	34.5	15	25.8	29.8	
34	516	PM	19.4	21.1	22.5		23.2		13.4
34	517	PM	87.4	112	184		104		
34	518	SSG	11	16.4	19.9	14	16.5		22.2
34	519	PM	25.1	41.5	46.6		58.6	21.4	
34	520	SSG	12.1	116	34.4	44.4	28.1		15.6
34	521	PM	11.6	16	39.3		60.7		
34	522	Combination	18.5	33.4	27.2		31		
34	523	SSG	23.3	28.2	26.6	24.5	31.2		25.3
34	524	Combination	19.8	13.7	41.3		58		46.3
34	525	Combination	88.3	134	58.8		51.4		22.9
34	526	SSG	20.2	64.2	40.1	55.2	61.5		16
34	527	Combination	16.4	50.4	25.4		26.1		13.7
34	528	PM	12.6	20.8	23.6		23.9		
34	529	Combination	22.9	31.6	33		34.5		20.1
34	530	Combination	75.3	50.5	59.7		56.3		39.1
34	531	SSG	27.5	57.8	46.8	53.9	79.7		
34	532	Combination	29.7	35.2	19.3		31.4		17.6
34	533	SSG	16.9	39.6	46.7	39.3	34.7		16.6
34	534	PM	27.3	27.9	54.7		64.1		
34	535	PM	27.1	93.5	46.2		41.1	14.8	
34	536	PM	54.1	33.4	30.7		37		22.9
34	537	SSG	16.9	49.2	51.9	38.2	29.5		27.8
34	538	PM	29.1	15.4	19		34.3		
34	539	Combination	27.4	158	73.2		46.4		24.3
34	540	SSG	25.8	77.5	47.1	45.4	75.2		21.1
35	646	PM	15	15	11		10		18
35	647	PM	19	15	12		12	30	16
35	648	Combination	12	12			12	24	20
35	649	Combination	10	18			10	28	18
35	650	SSG	18	9	16	10	11	20	17
35	651	SSG	19	19	16	10	10	26	20
35	652	PM	16	16	15		13		12
35	653	Combination	30	24	20		10	21	18
35	654	SSG	19	19	18	12	10	21	18
35	655	Combination	20	18	17		12	22	18
35	656	SSG	16	10	10	14	12	21	24
35	657	PM	15	12	10		14	34	22
35	658	Combination	20	18	12		10	22	20
35	659	SSG	10	12	15	12	14	18	16
35	660	PM	10	11	10		14	28	20
35	661	Combination	16	8	12		10	18	18
35	662	PM	14	10	16		14	18	11.2
35	663	SSG	15	12	15	14	12	24	22

Centre Number	Patient Number	Treatment	Alanine Aminotransferase (U/L) Baseline	Day 7	Day 14	Day 21	EOT	3 Mon FU	6 Mon FU
35	664	Combination	14	16					16
35	665	SSG	16	14	14	12	10	22	18
35	666	SSG	14	12	10	10	16	27	28
35	667	Combination	14	14	10		12	18	19
35	668	PM	16	14	10		14		26
35	669	PM	12	10	8		12		
35	670	SSG	10	10	12	16	15	20	18
35	671	PM	12	14	16		12		12
35	672	Combination	14	10	12		14		18
35	673	SSG	10	8	12	13	14	19	18
35	674	Combination	12	14	18		13	16	18
35	675	PM	12	8	10		12		10
35	676	PM	10	12	13		12	22	20
35	677	SSG	20	18					
35	678	Combination	16	15	15		14	18	18
35	679	SSG	14	12	15	14	15	18	16
35	680	Combination	16	16	10		13	16	20
35	681	Combination	20	24	17		17	22	22
35	682	PM	14	16	13		14		11
35	683	Combination	16	20	16		14		16
35	684	SSG	14	18	20	22	21	27	24
35	685	PM	15	14	20		16	22	19
35	686	PM	16	16	24		22	24	26
35	687	SSG	24	17	20	19	26	28	20
35	688	PM	24	22	20		21	31	20
35	689	Combination	24	20	21		22	26	22
35	690	SSG	26	24	20	23	22.3	28	20

Centre Number	Patient Number	Treatment	Alkaline Phosphatase (U/L)						
			Baseline	Day 7	Day 14	Day 21	EOT	3 Mon FU	6 Mon FU
11	1	PM	314	292	595		621	171	183
11	2	Combination	96	108	182		235	198	208
11	3	Combination	261	839	769		769	367	467
11	4	PM	181	375	465		492	860	859
11	5	PM	246	433	299		348	581	557
11	6	Combination	106	230	249		323	412	410
11	7	PM	63	125	146		162	169	173
11	8	SSG	87	184	231	260	248	210	242
11	9	SSG	192	368	390	392	443	731	654
11	10	SSG	172	371	369	347	427	573	377
11	11	Combination	903	839	1013		882	604	460
11	12	Combination	169	257	240		225	383	343
11	13	SSG	142	178	179	198	201	265	204
11	14	SSG	364	453	364	528	550	722	678
11	15	PM	97	104				210	154
11	16	Combination	131	176	328		311	167	129
11	17	Combination	248	449	408		375	230	217
11	18	SSG	144	207	257	247	225	277	183
11	19	Combination	108	131	145		141	155	146
11	20	SSG	238	289	365	369	349	404	494
11	21	SSG	182	242	288	334	328	258	261
11	22	SSG	145	383	179	368	385	481	404
11	23	PM	380	423	425		403	90	437
11	24	PM	169	227	242		219	277	256
11	25	SSG	186	310	294	373	81	554	628
11	26	Combination	549	765	746		610	752	566
11	27	PM	227	322	393		423	422	
11	28	Combination	132	295	293		367	534	640
11	29	PM	162	198	205		218	290	185
11	30	PM	382	314	440		691	422	820
11	31	SSG	228	307	338	647	533	197	158
11	32	Combination	359	474	458		426	348	289
11	33	SSG	160	219	357	197	323	317	291
11	34	PM	280	269	329		364	437	376
11	35	SSG	364	379	347	392	521	520	453
11	36	SSG	98	141	188	311	425	498	395
11	37	Combination	171	324	227		179	205	193
11	38	PM	202	359	330		383	230	271
11	39	PM	319	365	360		352	506	437
11	40	PM	302	266	245			737	561
11	41	Combination	540	512	563		552	863	852
11	42	Combination	267	396	398		364	320	311
11	43	SSG	344	297	303	293	286	328	405
11	44	PM	294	369	355		368	370	385
11	45	Combination	166	188	198		91	174	158
11	46	SSG	399	393	451	618	597	503	653
11	47	SSG	189	739	532	290	244		

Centre Number	Patient Number	Treatment	Alkaline Phosphatase (U/L)						
			Baseline	Day 7	Day 14	Day 21	EOT	3 Mon FU	6 Mon FU
11	48	Combination	246	282	540		495		557
11	49	SSG	313	806	836	666	621	569	510
11	50	PM	529	671	455		384	204	366
11	51	Combination	111	168	207		185	508	548
11	52	PM	192	154	164		161	260	434
11	53	SSG	534	701	876	705	1058	463	360
11	54	PM	1344	823	878		756	755	615
11	55	PM	236	285	429		351	282	262
11	56	SSG	92	122	178	228	270	812	601
11	57	Combination	130	953	739		466	236	238
11	58	Combination	233	262	446		513	688	517
11	59	PM	100	132	279		1849	593	564
11	60	Combination	971	1923	1144		1248	408	343
11	61	PM	329	395	438		440	427	327
11	62	SSG	398	327	293	287	294	533	597
11	63	SSG	205	284	426	473	340	196	146
11	64	SSG	434	592	483	496	378	294	332
11	65	PM	110	149	204		244		
11	66	Combination	211	451	333		337	208	172
11	67	PM	102	145	210		263	323	710
11	68	SSG	641	590	584	496	672	468	576
11	69	Combination	163	246	365		366	216	450
11	70	Combination	203	479	225		273	265	346
11	71	Combination	381	498	464		521	609	502
11	72	SSG	1551	2289	1823			725	607
11	73	PM	147	195	232		272	305	326
11	74	PM	158	200	267		212	157	176
11	75	Combination	128	77	239		302	238	139
11	76	PM	270	563	782		487	512	508
11	77	PM	186	173	269		382	592	608
11	78	SSG	146	184	210	254	265	200	257
11	79	PM	183	206	246		363	777	592
11	80	PM	137	91	174		293	568	621
11	81	Combination	311	496	717		702	880	248
11	82	SSG	162	288	416	479	544	587	674
11	83	SSG	1548	4304	2620	1303	771	325	282
11	84	SSG	658	637	641	418	581	627	662
11	85	SSG	486	1619	802	1026	945	287	
11	86	Combination	116	192	222		279	546	606
11	87	Combination	181	324	273		510	419	512
11	88	PM	132	101	222		548	184	186
11	89	Combination	1644	1854	780		1527	382	250
11	90	Combination	162	130	230		605		
11	91	PM	180	125	249		261	156	346
11	92	Combination	156	160	308		460	590	467
11	93	SSG	1681	1628	943	638	303	364	320
11	94	SSG	439	673	623	508	521	347	443

Centre Number	Patient Number	Treatment	Alkaline Phosphatase (U/L)						
			Baseline	Day 7	Day 14	Day 21	EOT	3 Mon FU	6 Mon FU
11	95	SSG	231	1050	884	517	454	397	320
11	96	Combination	110	356	163		467	488	552
11	97	Combination	996	1398	707		534	1063	401
11	98	PM	852	717	686		564	602	453
11	99	SSG	152	455	477	428	428	678	786
11	100	PM	265	223	287		324		
11	101	PM	846	1022	923		747	482	254
11	102	Combination	87	309	299		254	336	283
11	103	Combination	681	787	482		407	201	252
11	104	PM	615	1294	680		477	317	366
11	105	SSG	122	348	255	198	196	232	275
11	106	Combination	163	200	152		178	259	227
11	107	PM	205	311	331		367	351	476
11	108	PM	458	609	499		373	361	252
11	109	SSG	432	905	405	351	286	290	336
11	110	PM	312	370	400		946	615	517
11	111	Combination	970	1553	959		637	278	322
11	112	Combination	603	1279	806		629	364	382
11	113	SSG	900	3977	1844	1211	1624	499	
11	114	SSG	1334	2010	1727	1253	891	418	250
11	115	Combination	117	145	296		346	509	455
11	116	SSG	242	399	386	424	362	500	366
11	117	PM	101	131	172		163	189	
11	118	PM	403	430	810		875	391	381
11	119	SSG	1259	653	464	416	358	249	427
11	120	Combination	208	969	480		427	309	308
11	121	Combination	198	276	300		377	511	298
11	122	SSG	908	1439	945	761	546		
11	123	SSG	333	446	505	596	625	833	897
11	124	SSG	657	700	471	394	514		175
11	125	SSG	255	686	596	425	322	374	385
11	126	Combination	152	495	451		465		282
11	127	PM	172	211	561		463	606	607
11	128	Combination	291	573	702		637	435	283
11	129	PM	191	186	223		242	142	199
11	130	Combination	113	254	265		304	312	290
11	131	PM	230	181	318		379	430	467
11	132	SSG	304	338	286	265	279	238	142
11	133	Combination	253	696	473		240	296	292
11	134	PM	342	440	318		235	277	287
11	135	PM	171	168	224		231	283	313
12	241	Combination	124	95	268		274		212
12	242	SSG	160	217	118	285	131	189	252
12	243	PM	139	216	204		91	356	184
12	244	SSG	169	106	264	178	181	221	76.7
12	245	PM	394	185	298.34		229	263	
12	246	PM	179	284	439		302	320	

Centre Number	Patient Number	Treatment	Alkaline Phosphatase (U/L) Baseline	Day 7	Day 14	Day 21	EOT	3 Mon FU	6 Mon FU
12	247	PM	93	127	236		107	92	217
12	248	PM	170	134	78.4		82.9	115	98
12	249	Combination	143	273	339		575	179	135
12	250	SSG	140	154	204	134	152	201	182
12	251	Combination	340	700	348		340	567	185
12	252	SSG	668	260	309	292	120	113	567
12	253	SSG	271	498	228	281	182	150	183
12	254	Combination	168	224	1147		909	571	185
12	255	Combination	115	254	305		347	825	572
12	256	Combination	125	117	203		200	135	87.5
12	257	SSG	153	332	314	254	264	163	152
12	258	SSG	149	204	242	217	180		726
12	259	PM	126	240	314.94		150	217	209
12	260	PM	135	178	176		182		202
12	261	Combination	92	211	214		207	231	154
12	262	PM	186	167	159		334	198	224
12	263	SSG	182	128	249	190.7	246	448	97
12	264	Combination	130	165	108		133	121	119
12	265	SSG	148	179	93.9	142	74		
12	266	PM	153	154	149		295	157	171.2
12	267	SSG	242	115	114	172	112	287	186
12	268	PM	180	372	420		346	215	448
12	269	Combination	173	324	154		158	257	241
12	270	Combination	101	177	140		142		120
12	271	SSG	104	108	218	185	193	71.5	276
12	272	PM	431	224	240		193	203	168
12	273	SSG	580	34.5	367	156	186	132	196
12	274	SSG	149	125	150	129	168	329	286
12	275	Combination	139	81	100		132	262	346
12	276	PM	62	83	109		91	125	346
12	277	Combination	314	249	249		228	109	244
12	278	SSG	203	354	237	170	113	147	
12	279	PM	104	130	129		131	189	394
12	280	SSG	78	855	424	273	256	200	197
12	281	Combination	148	182	177		180	127	139
12	282	PM	80.7	62	79		93.7	101	164
12	283	PM	56.5	102	65.1		62.7	73.2	72.3
12	284	Combination	62.8	107	253		104	229	
12	285	Combination	239	312	506		186	239	207
12	286	PM	130	92	104		90.6	73.2	68.1
12	287	SSG	140	180	155	159	145	176	181
12	288	Combination	94	185	145		95	139	194
12	289	Combination	140	83.6	82		173	144	64.4
12	290	SSG	80	203	135	146	131		120
12	291	SSG	187	208	222	170	145	113	119
12	292	PM	351	414	363		163		154
12	293	SSG	88.9	88.6	101	156	163		151

Centre Number	Patient Number	Treatment	Alkaline Phosphatase (U/L)						
			Baseline	Day 7	Day 14	Day 21	EOT	3 Mon FU	6 Mon FU
12	294	Combination	119	129	177		115	168	140
12	295	Combination	204	142	195		154		125
12	296	PM	103	82.9	107		113	131	125
12	297	SSG	66.6	90.2	87.5				
12	298	PM	84.9	73.1	78.1		82.5	130	166
12	299	Combination	434	542	539		452		293
12	300	PM	96	147	165		121	135	191
12	301	SSG	126	134	133	122	136	81.7	79
12	302	SSG	216	219	216	174	106	320	310
12	303	PM	210	245	297		110	210	252
12	304	Combination	146	249	239		136	230	305
12	305	PM	176	87.8	93.4		85.4	410	563
12	306	Combination	168	178	182		188	347	400
12	307	Combination	256	84.5	322		250	325	269
12	308	PM	112	126	133		144	171	205
12	309	PM	210	208	195		91.8	121	243
12	310	SSG	195	184	169.3	73.3	96.7	364	84
12	311	SSG	133	73.1	76	139	132	144	302
12	312	Combination	137	244	182		109	210	137
12	313	PM	124	113	158		151	212	250
12	314	SSG	632	94.7	49.6	72.7	76.2	78.4	211
12	315	Combination	39.4	46.2	87		96	226	105
12	316	SSG	150	172	184			185	176
12	317	SSG	73	19	39	49	51.4		310
12	318	Combination	69.4	72	84		99	210	34
12	319	Combination	101	108	116		120	300	116
12	320	Combination	199	207	162		136	194	136
12	321	Combination	52	69	125		82.5	185	86
12	322	PM	129	149	160		142	200	239
12	323	SSG	220	144	310	357	218	109	168
12	324	SSG	136	163	210	252	250	145	105
12	325	PM	281	135	280		140	110	158
12	326	PM	420	390	534		296	182	196
12	327	Combination	130	136	280		302	182	167
12	328	PM	121	160	212		210	120	180
12	329	SSG	153	265	302	250	232	210	296
12	330	PM	565	190	420		122	131	242
23	361	Combination	92.4	228	336		231	495	504
23	362	PM	77	341	370		409	654	738
23	363	PM	108	403	550		551	661	1073
23	364	Combination	208	329	315		376	264	598
23	365	SSG	62	363	372	387	396	569	353
23	366	Combination	221	570	272		162	478	574
23	367	SSG	588	644	360	578	427	379	230
23	368	PM	279	491	725		474	252	156
23	369	Combination	188	213	224		196	528	408
23	370	SSG	367	688	293	403	569	688	550

Centre Number	Patient Number	Treatment	Alkaline Phosphatase (U/L) Baseline	Day 7	Day 14	Day 21	EOT	3 Mon FU	6 Mon FU
23	371	PM	114	89	96		138	233	149
23	372	PM	283	256	260		153	246	250
23	373	SSG	172	175	404	312	284	124	231
23	374	SSG	324	695	210	276	245	221	239
23	375	Combination	369	541	342		364	612	185
23	376	SSG	252	203	375	372	460	752	434
23	377	Combination	152	643					430
23	378	Combination	189	364	428		401	269	581
23	379	PM	225	348	382		525	193	558
23	380	SSG	185	318	367	470	697	733	650
23	381	PM	328	217	294		373	189	413
23	382	Combination	351	716	1040		747	474	433
23	383	PM	482	420	590		420	182	191
23	384	SSG	390	562	340	421	476	665	777
23	385	SSG	288	399	374	198	592	722	662
23	386	Combination	246	697	554		533	943	796
23	387	Combination	223	266	275		271	1118	888
23	388	PM	315	365	466		486	754	552
23	389	SSG	246	583	454	498	653	764	533
23	390	PM	223	320	235		225	342	256
23	391	Combination	593	576	520		496	572	764
23	392	Combination	808	913	675			671	608
23	393	SSG	261	312	338	568	527	459	564
23	394	PM	200	283	412		479	485	520
23	395	PM	537	385	305		293	231	126
23	396	SSG	471	300	292	367	294	440	395
23	397	SSG	247	585	282	310	403	651	458
23	398	Combination	203	196	249		251	289	113
23	399	PM	355	269	293		372	431	521
23	400	PM	603	270	265		249	429	396
23	401	Combination	218	209	263		267	170	116
23	402	Combination	175	264	215		306	656	738
23	403	SSG	418	277	366	287	666	412	396
23	404	SSG	414	476	487	302	319		362
23	405	PM	393	381	369		369	1156	1053
34	451	Combination	118	410	389		320		378
34	452	PM	231	320	466		365		
34	453	PM	289	290	375		412		
34	454	Combination	162	337	347		360		456
34	455	PM	187	212	222		226		
34	456	SSG	320	402	566	583	676	817	
34	457	Combination	146	509	361		295		
34	458	PM	173	278	283		403	488	
34	459	Combination	204	227	319		346	503	589
34	460	PM	230	456	594		478		
34	461	SSG	366	1060	636				
34	462	SSG	273	357	448	450	472		357

Centre Number	Patient Number	Treatment	Alkaline Phosphatase (U/L)						
			Baseline	Day 7	Day 14	Day 21	EOT	3 Mon FU	6 Mon FU
34	463	Combination	497	1220	615		712		547
34	464	SSG	333	338	403	333	278		160
34	465	SSG	271	691	344	344	336		219
34	466	Combination	388	346	408		403		
34	467	PM	328	1320	2020		1100	1210	
34	468	Combination	300	502	305		247		167
34	469	SSG	246	336	556	379	381		508
34	470	Combination	235	409	522		402		318
34	471	PM	238	357	317		292		
34	472	SSG	344	421	451	420	402		362
34	473	PM	286	303	373		342		
34	474	Combination	381	357	274		276		351
34	475	PM	217	91.3	210		237		
34	476	PM	297	356	392		525	632	
34	477	SSG	163	241	253	294	292		282
34	478	Combination	229	240	195		198		243
34	479	SSG	248	321	338	424	523		356
34	480	SSG	435	523	606	622	513		636
34	481	Combination	444	854	556		509		442
34	482	Combination	297	832	455		394		337
34	483	PM	176	196	250		189		179
34	484	PM	382	205					
34	485	Combination	311	355	516		450		596
34	486	Combination	229	369	389		376		433
34	487	SSG	180	299	336	468	549		655
34	488	SSG	303	419	389	521	655	514	550
34	489	SSG	349	370	420	386	413		467
34	490	PM	258	564	938		507		474
34	491	PM	588	1170	1230		1270		
34	492	PM	209	313	268		338		440
34	493	SSG	145	214	256	332	347		
34	494	Combination	277	566	348		306		222
34	495	SSG	247	384	409	491	411		470
34	496	SSG	179	347	234	284	251		217
34	497	PM	146	192	241		209	214	
34	498	SSG	301	473	582	704	569		593
34	499	Combination	353	407	458		526		629
34	500	PM	361	467	604		609		588
34	501	Combination	292	478	511		485		671
34	502	Combination	264	334	348		307		451
34	503	PM	293	357	376		379		
34	504	Combination	275	365	410		475		
34	505	SSG	142	215	212	184	182		225
34	506	SSG	242	287	426	494	637		561
34	507	SSG	192	965	673	650	654		413
34	508	PM	177	205	243		373		
34	509	PM	328	496	402		290		

Centre Number	Patient Number	Treatment	Alkaline Phosphatase (U/L)						
			Baseline	Day 7	Day 14	Day 21	EOT	3 Mon FU	6 Mon FU
34	510	Combination	253	367	438		471		551
34	511	Combination	447	628	532		575		608
34	512	PM	253	307	296		379		
34	513	Combination	337	447	373		309	583	565
34	514	SSG	447	1020	853	526	591		681
34	515	SSG	184	260	270	153	253	258	
34	516	PM	532	478	427		412		466
34	517	PM	375	594	674		461		
34	518	SSG	164	286	353	383	344		607
34	519	PM	305	408	576		460	302	
34	520	SSG	172	665	369	269	244		197
34	521	PM	223	222	453		524		
34	522	Combination	278	330	280		313		
34	523	SSG	472	479	325	355	398		545
34	524	Combination	270	288	388		409		625
34	525	Combination	643	751	599		582		479
34	526	SSG	288	379	444	483	620		307
34	527	Combination	332	1400	627		442		179
34	528	PM	275	263	299		255		
34	529	Combination	346	575	621		749		619
34	530	Combination	159	240	236		248		164
34	531	SSG	300	759	617	504	494		
34	532	Combination	260	321	277		451		775
34	533	SSG	357	430	422	432	515		505
34	534	PM	176	229	241		340		
34	535	PM	178	426	419		323	185	
34	536	PM	469	359	331		309		351
34	537	SSG	285	930	644	486	355		352
34	538	PM	390	489	486		610		
34	539	Combination	498	491	552		548		360
34	540	SSG	340	1930	1420	856	1220		975
35			Not Done						

Centre Number	Patient Number	Treatment	Amylase (U/L) Baseline	Day 7	Day 14	Day 21	EOT	3 Mon FU	6 Mon FU
11			Not done						
12	241	Combination		114	91.5		19.2		29
12	242	SSG		64	95.7	14.8	95.7	30.4	59.43
12	243	PM		14	14			14	
12	244	SSG		61.1	76.4	33.9	78.5	40.7	134
12	245	PM	16.2	16.3	36.9		33.5		
12	246	PM	50.3	50.9	51.7		50.5	49	
12	247	PM	41	14	55		37.4		
12	248	PM	26.5	30	42		39.8	29	
12	249	Combination	30.4	68.8	61.4		67.2	111	87.1
12	250	SSG	30	20.2	57.3	125	81	85.7	
12	251	Combination	37.4	96.3	4.6		22.5	140	121
12	252	SSG	30.6	106	103	137	203	130	
12	253	SSG	27.8	15.6	17.6	109	268	291	
12	254	Combination	14	71.8	72.4		85	221	227
12	255	Combination	14	28.2	28.8		23.6	114	88.3
12	256	Combination	31	70	14		66	101	
12	257	SSG	29.8	109	317	289	20		
12	258	SSG	111	305	242	195	160		
12	259	PM	71.3	65.7	80.9		79	44	
12	260	PM	86.3	103			79.2		
12	261	Combination	67.4	237	129		161		
12	262	PM	149	196	140		125		
12	263	SSG	129	195	143	174	142		
12	264	Combination		235	234		128		
12	265	SSG	108	100	203	138	1140		
12	266	PM	237	94.7	105		86.5		90
12	267	SSG	138	155	151	130	117		
12	268	PM	153	166	210		153		218
12	269	Combination	147	29.8	272		292		45
12	270	Combination	67.3	190	123		128		124
12	271	SSG							
12	272	PM							78
12	273	SSG					28.9		28
12	274	SSG					30.5		54
12	275	Combination							
12	276	PM	77.7						
12	277	Combination			41.2		89.4		114
12	278	SSG			141	71.5	157		
12	279	PM			79.6		108		64
12	280	SSG			14	31.7	53.3		
12	281	Combination			40.9		34.4		
12	282	PM		14	14		59.4		

Centre Number	Patient Number	Treatment	Amylase (U/L) Baseline	Day 7	Day 14	Day 21	EOT	3 Mon FU	6 Mon FU
12	283	PM	53.5	210	139				
12	284	Combination	62.8						
12	285	Combination	131	384					
12	286	PM		45	44.2		42.8	55.8	
12	287	SSG		291	216	255	221		96
12	288	Combination	49.6	56.1	58		116	65.6	68.3
12	289	Combination	52.1	90	12		61.7	92	114
12	290	SSG	74	104	152	128	143		94
12	291	SSG	76	107	96	82	94	138	88.6
12	292	PM	93	81.5	96		87		144
12	293	SSG	86	151	1.55	148	108		173
12	294	Combination	32.4	101	94		142	86	
12	295	Combination	139	170	125		254		105
12	296	PM	101	72.6	94.3		91.3	113	83
12	297	SSG	121	460	638				
12	298	PM	62.9	61.9	92.5		87.6	73	57
12	299	Combination	113	116					111
12	300	PM	104	106			153	169	83
12	301	SSG	115	120			303	97	66
12	302	SSG	69.3	71.2			107	86	88
12	303	PM	67.4	105			135	113	117
12	304	Combination	90.8	94.2				105	104
12	305	PM	82.1	83.4			108	119	89
12	306	Combination	67.4	70.3				61	79
12	307	Combination			245		380	141	103
12	308	PM			173		178	80	94
12	309	PM			87.4		94.8	66	72
12	310	SSG			143	159	160	102	81
12	311	SSG	65.7	117	122	108	121	94	70
12	312	Combination	54	542	438		312	98	54
12	313	PM	162	131	160		152	102	107
12	314	SSG	99.8	388	587	615	540	182	112
12	315	Combination	66	107	118		64	89	105
12	316	SSG	108	492	990		255	230	234
12	317	SSG	69	76.9	132	90	112		203
12	318	Combination	77	134	106		80	40	56
12	319	Combination	66	142	115		97	105	76
12	320	Combination	123	212	144		162	110	101
12	321	Combination	74.5	136	113		99	103	99
12	322	PM	89	74	59		94	75	104
12	323	SSG	74	136	140	168	84	73	111
12	324	SSG	119	190	181	210	189	125	203
12	325	PM	110	175	143		103	96	138

Centre	Patient		Amylase (U/L)						
Number	Number	Treatment	Baseline	Day 7	Day 14	Day 21	EOT	3 Mon FU	6 Mon FU
12	326	PM	93	101	110		194	114	143
12	327	Combination	63	114	179		87	83	169
12	328	PM	45	68	66		75	55	37
12	329	SSG	44	68	57	74	86	74	104
12	330	PM	49	104	104		102.1	87	110
23			Not done						
34			Not done						
35			Not done						

Centre Number	Patient Number	Treatment	Bilirubin (µmol/L) Baseline	Day 7	Day 14	Day 21	EOT	3 Mon FU	6 Mon FU
11	1	PM	5.1		13.7		6.8	10.3	10.3
11	2	Combination	10.3		18.8		13.7	13.7	13.7
11	3	Combination	12.0		20.5		20.5	5.1	10.3
11	4	PM		18.8	15.4		30.8	20.5	17.1
11	5	PM		10.3	5.1		13.7	6.8	5.1
11	6	Combination		22.2	20.5		18.8	10.3	8.6
11	7	PM		17.1	17.1		22.2	8.6	10.3
11	8	SSG		15.4	18.8	13.7	10.3	8.6	12.0
11	9	SSG		10.3	1.7	15.4	6.8	15.4	10.3
11	10	SSG		6.8	1.7	5.1	3.4	8.6	10.3
11	11	Combination	6.8	3.4	10.3		6.8	3.4	5.1
11	12	Combination		17.1	10.3		10.3	8.6	10.3
11	13	SSG	25.7	23.9	29.1	18.8	29.1	12.0	12.0
11	14	SSG	6.8	6.8	6.8	5.1	6.8	8.6	5.1
11	15	PM	15.4	25.7				13.7	20.5
11	16	Combination	5.1	5.1	8.6		10.3	8.6	6.8
11	17	Combination	13.7	12.0	8.6		8.6	10.3	6.8
11	18	SSG	5.1	5.1	8.6	15.4	15.4	8.6	12.0
11	19	Combination	15.4	6.8	17.1		15.4	15.4	10.3
11	20	SSG	20.5	8.6	12.0	15.4	18.8	17.1	12.0
11	21	SSG	10.3	3.4	13.7	13.7	17.1	12.0	15.4
11	22	SSG	3.4	6.8	5.1	6.8	12.0	5.1	5.1
11	23	PM	5.1	5.1	5.1		6.8	10.3	8.6
11	24	PM	6.8	13.7	13.7		12.0	12.0	13.7
11	25	SSG	3.4	6.8	6.8	5.1	8.6	5.1	6.8
11	26	Combination	6.8	6.8	6.8		6.8	3.4	6.8
11	27	PM	12.0	18.8	12.0		12.0	8.6	
11	28	Combination	10.3	8.6	15.4		8.6	8.6	8.6
11	29	PM	15.4	18.8	17.1		15.4	15.4	10.3
11	30	PM	8.6	10.3	1.7		5.1	1.7	3.4
11	31	SSG	6.8	3.4	6.8	5.1	5.1	8.6	12.0
11	32	Combination	8.6	3.4	10.3		8.6	10.3	10.3
11	33	SSG	12.0	6.8	6.8	10.3	10.3	10.3	12.0
11	34	PM	20.5	20.5	22.2		22.2	12.0	12.0
11	35	SSG	17.1	12.0	12.0	13.7	13.7	17.1	22.2
11	36	SSG	12.0	8.6	10.3	6.8	10.3	6.8	8.6
11	37	Combination	18.8	10.3	10.3		6.8	12.0	17.1
11	38	PM	5.1	6.8	8.6		8.6	8.6	1.7
11	39	PM	3.4	5.1	5.1		5.1	5.1	5.1
11	40	PM	5.1	8.6	10.3			10.3	10.3
11	41	Combination	5.1	5.1	3.4		5.1	3.4	5.1
11	42	Combination	3.4	6.8	13.7		12.0	12.0	8.6
11	43	SSG	15.4	18.8	17.1	15.4	15.4	10.3	17.1

Centre Number	Patient Number	Treatment	Bilirubin (µmol/L) Baseline	Day 7	Day 14	Day 21	EOT	3 Mon FU	6 Mon FU
11	44	PM	13.7	15.4	13.7		15.4	10.3	10.3
11	45	Combination	10.3	8.6	10.3		10.3	12.0	10.3
11	46	SSG	6.8	8.6	3.4	6.8	3.4	5.1	6.8
11	47	SSG	1.7	8.6	10.3	10.3	10.3		
11	48	Combination	10.3	3.4	10.3		12.0		15.4
11	49	SSG	6.8	12.0	5.1	6.8	6.8	3.4	6.8
11	50	PM	8.6	8.6	12.0		13.7	13.7	8.6
11	51	Combination	10.3	6.8	8.6		6.8	6.8	6.8
11	52	PM	5.1	10.3	12.0		6.8	10.3	8.6
11	53	SSG	18.8	13.7	17.1	15.4	18.8	15.4	25.7
11	54	PM	13.7	8.6	8.6		8.6	6.8	8.6
11	55	PM	13.7	17.1	12.0		12.0	27.4	22.2
11	56	SSG	8.6	5.1	6.8	5.1	5.1	6.8	8.6
11	57	Combination	6.8	6.8	8.6		5.1	6.8	13.7
11	58	Combination	8.6	6.8	6.8		10.3	5.1	6.8
11	59	PM	5.1	6.8	6.8		8.6	6.8	6.8
11	60	Combination	15.4	10.3	13.7		6.8	13.7	18.8
11	61	PM	8.6	8.6	5.1		6.8	6.8	12.0
11	62	SSG	12.0	3.4	5.1	8.6	3.4	5.1	6.8
11	63	SSG	6.8	5.1	5.1	5.1	5.1	8.6	12.0
11	64	SSG	12.0	17.1	15.4	15.4	12.0	12.0	6.8
11	65	PM	5.1	6.8	5.1		5.1		
11	66	Combination	5.1	6.8	8.6		8.6	15.4	5.1
11	67	PM	6.8	8.6	12.0		8.6	8.6	6.8
11	68	SSG	8.6	5.1	5.1	6.8	8.6	6.8	5.1
11	69	Combination	23.9	15.4	22.2		23.9	18.8	12.0
11	70	Combination	10.3	10.3	25.7		13.7	18.8	27.4
11	71	Combination	6.8	5.1	5.1		6.8	12.0	15.4
11	72	SSG	8.6	3.4	15.4			5.1	10.3
11	73	PM	8.6	8.6	12.0		13.7	6.8	8.6
11	74	PM	8.6	13.7	5.1		10.3	12.0	3.4
11	75	Combination	8.6	12.0	8.6		8.6	12.0	12.0
11	76	PM	8.6	12.0	13.7		15.4	18.8	12.0
11	77	PM	3.4	5.1	5.1		5.1	5.1	3.4
11	78	SSG	10.3	8.6	10.3	12.0	10.3	13.7	15.4
11	79	PM	5.1	6.8	3.4		5.1	6.8	6.8
11	80	PM	6.8	6.8	5.1		6.8	5.1	3.4
11	81	Combination	10.3	8.6	8.6		8.6	8.6	8.6
11	82	SSG	6.8	3.4	6.8	8.6	1.7	5.1	1.7
11	83	SSG	13.7	18.8	12.0	10.3	1.7	8.6	3.4
11	84	SSG	18.8	8.6	5.1	10.3	6.8	5.1	3.4
11	85	SSG	27.4	12.0	12.0	8.6	10.3	15.4	
11	86	Combination	3.4	5.1	5.1		6.8	3.4	3.4

Centre Number	Patient Number	Treatment	Bilirubin (µmol/L)						
			Baseline	Day 7	Day 14	Day 21	EOT	3 Mon FU	6 Mon FU
11	87	Combination	3.4	3.4	5.1		1.7	6.8	3.4
11	88	PM	12.0	12.0	12.0		12.0	10.3	12.0
11	89	Combination	17.1	6.8	8.6		10.3	10.3	13.7
11	90	Combination	15.4	10.3	10.3		10.3		
11	91	PM	17.1	8.6	12.0		13.7	22.2	10.3
11	92	Combination	8.6	5.1	8.6		1.7	3.4	5.1
11	93	SSG	13.7	12.0	8.6	8.6	8.6	13.7	15.4
11	94	SSG	5.1	1.7	3.4	3.4	3.4	3.4	6.8
11	95	SSG	5.1	1.7	3.4	6.8	1.7	3.4	5.1
11	96	Combination	6.8	6.8	5.1		5.1	3.4	
11	97	Combination	15.4	6.8	5.1		6.8	8.6	5.1
11	98	PM	39.3	25.7	17.1		13.7	8.6	12.0
11	99	SSG	6.8	5.1	12.0	8.6	10.3	10.3	6.8
11	100	PM	8.6	6.8	6.8		5.1		
11	101	PM	15.4	20.5	13.7		13.7	17.1	18.8
11	102	Combination	25.7	10.3	8.6		8.6	6.8	6.8
11	103	Combination	15.4	12.0	13.7		10.3	20.5	18.8
11	104	PM	8.6	8.6	5.1		5.1	5.1	5.1
11	105	SSG	12.0	8.6	12.0	13.7	10.3	10.3	8.6
11	106	Combination	17.1	10.3	3.4		15.4	17.1	23.9
11	107	PM	6.8	5.1	3.4		6.8	8.6	6.8
11	108	PM	10.3	8.6	8.6		6.8	8.6	10.3
11	109	SSG	6.8	5.1	8.6	13.7	5.1	5.1	6.8
11	110	PM	17.1	13.7	13.7		13.7	13.7	12.0
11	111	Combination	8.6	12.0	15.4		15.4	25.7	23.9
11	112	Combination	10.3	13.7	6.8		8.6	10.3	15.4
11	113	SSG	10.3	13.7	10.3	8.6	8.6	6.8	
11	114	SSG	5.1	6.8	6.8	8.6	10.3	10.3	8.6
11	115	Combination	13.7	8.6	1.7		8.6	8.6	6.8
11	116	SSG	10.3	8.6	6.8	8.6	8.6	8.6	12.0
11	117	PM	6.8	12.0	13.7		15.4	13.7	
11	118	PM	12.0	10.3	10.3		13.7	10.3	8.6
11	119	SSG	18.8	13.7	13.7	12.0	10.3	10.3	13.7
11	120	Combination	8.6	6.8	6.8		8.6	8.6	8.6
11	121	Combination	3.4	8.6	5.1		5.1	1.7	17.1
11	122	SSG	15.4	8.6	6.8	6.8	6.8		
11	123	SSG	13.7	8.6	12.0	5.1	12.0	8.6	8.6
11	124	SSG	13.7	13.7	10.3	8.6	6.8		13.7
11	125	SSG	10.3	10.3	8.6	10.3	10.3	10.3	10.3
11	126	Combination	6.8	6.8	5.1		8.6		8.6
11	127	PM	8.6	10.3	13.7		12.0	6.8	6.8
11	128	Combination	10.3	6.8	6.8		6.8	3.4	6.8
11	129	PM	10.3	15.4	12.0		10.3	13.7	18.8

Centre Number	Patient Number	Treatment	Bilirubin (µmol/L)						
			Baseline	Day 7	Day 14	Day 21	EOT	3 Mon FU	6 Mon FU
11	130	Combination	1.7	6.8	10.3		10.3	8.6	12.0
11	131	PM	6.8	5.1	6.8		8.6	6.8	3.4
11	132	SSG	6.8	10.3	10.3	12.0	10.3	6.8	17.1
11	133	Combination	13.7	10.3	10.3		3.4	10.3	5.1
11	134	PM	20.5	20.5	18.8		13.7	15.4	15.4
11	135	PM	10.3	8.6	8.6		12.0	12.0	8.6
12	241	Combination	5.1	5.1	8.6		6.8		17.1
12	242	SSG	3.4	3.4	3.4	5.1	5.1	12.0	13.7
12	243	PM	5.1	3.4	5.1		8.6	5.1	10.4
12	244	SSG	15.4	8.6	10.3	10.3	6.8	13.7	22.2
12	245	PM	13.7	15.4	17.1		10.3	17.1	
12	246	PM	8.6	5.1	5.1		10.3	6.8	
12	247	PM	5.1	5.1	13.7		14.5	6.8	7.9
12	248	PM	5.1	6.8	17.1		16.2	15.7	17.1
12	249	Combination	13.7	17.3	3.4		8.6	23.9	8.6
12	250	SSG	10.3	12.3	5.1	6.8	5.1	20.0	14.0
12	251	Combination	23.9	18.8	3.4		6.8	18.8	13.0
12	252	SSG	20.5	20.5	6.8	16.1	8.6	8.6	10.3
12	253	SSG	3.4	5.1	5.1	15.7	10.3	12.0	18.1
12	254	Combination	5.1	6.8	5.1		8.6	19.2	17.4
12	255	Combination	22.7	5.1	13.7		16.4	13.7	14.7
12	256	Combination	8.6	6.8	8.6		6.8	17.4	15.4
12	257	SSG	8.9	7.0	10.3	8.6	18.5	17.4	21.6
12	258	SSG	5.1	10.3	6.8	13.3	15.9		9.7
12	259	PM	11.1	19.7	3.4		6.8	13.7	15.9
12	260	PM	14.4	5.1	10.3		10.3		16.2
12	261	Combination	17.4	6.8	6.8		15.4	18.1	12.0
12	262	PM	27.7	10.3	3.4		10.3	14.0	14.0
12	263	SSG	10.3	13.7	10.3	8.6	20.5	13.7	3.4
12	264	Combination	12.0	8.6	14.2		19.0	10.3	13.7
12	265	SSG	10.3	10.3	18.6	15.1	14.0		
12	266	PM	13.7	5.1	27.4		15.4	16.8	28.6
12	267	SSG	20.5	12.0	20.0	15.4	17.4	10.3	10.3
12	268	PM	5.1	20.5	6.8		8.6	20.5	19.5
12	269	Combination	6.8	8.9	13.0		6.8	13.7	13.7
12	270	Combination	8.6	10.9	12.0		12.0		13.0
12	271	SSG	13.7	16.8	6.8	14.7	13.3	17.6	13.7
12	272	PM	8.6	3.4	14.0		11.6	13.0	10.3
12	273	SSG	17.8	6.8	22.2	18.1	17.1	13.0	6.8
12	274	SSG	10.3	5.1	10.3	12.0	12.0	9.9	8.6
12	275	Combination	17.4	6.8	8.6		10.1	10.6	15.4
12	276	PM	17.4	13.7	20.5		13.7	12.0	13.7
12	277	Combination	14.7	12.1	20.0		19.8	12.5	12.0

Centre Number	Patient Number	Treatment	Bilirubin (µmol/L) Baseline	Day 7	Day 14	Day 21	EOT	3 Mon FU	6 Mon FU
12	278	SSG	8.2	20.4	25.7	20.5	16.9	27.4	
12	279	PM	12.3	8.6	15.7		15.4	15.4	13.7
12	280	SSG	11.5	29.8	24.6	20.5	14.9	15.4	20.7
12	281	Combination	8.6	12.8	24.6		17.4	13.5	12.8
12	282	PM	20.5	16.6			22.9	25.7	20.2
12	283	PM	14.4	39.0	15.1		14.2	9.4	13.7
12	284	Combination	9.7	9.9	8.2		10.6	18.1	17.1
12	285	Combination	18.1	15.7	10.3		13.3	15.6	17.4
12	286	PM	24.3	16.4	21.4		19.5	15.4	13.7
12	287	SSG	16.4	23.9	20.2	15.9	16.8	15.7	13.7
12	288	Combination	12.3	12.0	12.8		15.9	15.9	9.6
12	289	Combination	24.1	19.8	18.6		14.5	15.4	13.9
12	290	SSG	15.4	14.0	17.1	20.0	16.4		10.9
12	291	SSG	18.8	13.7	13.7	17.1	16.8	11.3	9.6
12	292	PM	18.8	23.9	25.7		27.4		17.1
12	293	SSG	20.9	19.2	16.9	15.4	16.6		9.4
12	294	Combination	18.0	17.1	14.7		16.4	11.3	9.9
12	295	Combination	13.7	12.5	10.3		13.5		11.3
12	296	PM	17.6	13.5	16.2		15.4	10.1	12.0
12	297	SSG	19.8	15.7	20.0				
12	298	PM	17.1	15.4	14.5		15.4	15.2	10.6
12	299	Combination	13.0	24.1	23.3		17.4		17.4
12	300	PM	9.2	10.8	10.6			11.8	12.3
12	301	SSG	10.1	10.6	12.7	14.4	15.4	14.0	11.8
12	302	SSG	16.8	13.0	13.7	14.7	14.5	12.1	8.6
12	303	PM	14.0	13.3	14.0		12.1	9.9	8.6
12	304	Combination	12.5	13.3	13.7		13.0	12.7	15.4
12	305	PM	9.9	10.3	11.6		12.0	14.0	10.3
12	306	Combination	18.3	17.4	17.4		17.1	15.4	12.0
12	307	Combination	13.0	11.5	13.0		13.3	11.5	10.3
12	308	PM	20.2	15.7	8.2		16.4	13.5	8.6
12	309	PM	17.6	16.9	17.1		15.2	13.7	13.7
12	310	SSG	12.5	13.7	14.7	15.7	8.6	15.4	12.3
12	311	SSG	14.9	8.6	10.6	16.4	16.8	9.6	20.5
12	312	Combination	17.1	12.1	12.3		12.7	12.1	17.1
12	313	PM	8.6	8.9	8.6		13.7	12.3	11.5
12	314	SSG	8.7	16.4	11.6	9.6	9.6	9.2	12.0
12	315	Combination	11.5	10.6	12.8		11.8	12.1	15.7
12	316	SSG	8.6	11.8	16.9			8.6	11.8
12	317	SSG	20.0	16.9	16.4	13.7	13.9		11.5
12	318	Combination	12.3	11.6	9.9		10.6	10.6	22.2
12	319	Combination	15.6	8.7	8.9		9.1	10.3	18.8
12	320	Combination	15.6	16.4	11.5		11.3	12.1	22.2

Centre Number	Patient Number	Treatment	Bilirubin (µmol/L)						
			Baseline	Day 7	Day 14	Day 21	EOT	3 Mon FU	6 Mon FU
12	321	Combination	8.7	9.6	10.4		10.6	14.7	10.6
12	322	PM	16.8	12.3	11.8		12.0	13.9	5.1
12	323	SSG	14.7	12.0	15.4	15.2	18.8	10.3	6.8
12	324	SSG	14.0	14.0	14.4	14.0	13.7	8.7	13.7
12	325	PM	11.3	10.6	12.3		15.4	15.1	13.7
12	326	PM	11.8	12.3	13.7		15.4	13.3	6.8
12	327	Combination	15.1	15.7	8.6		17.1	10.6	12.0
12	328	PM	15.4	14.7	10.1		13.2	10.4	11.3
12	329	SSG	11.6	13.7	12.0	12.3	13.0	11.3	12.7
12	330	PM	12.3	8.6	11.6		10.4	10.6	8.9
23	361	Combination	11.4	11.0	1.0		5.0	11.0	12.0
23	362	PM	12.4	11.0	1.0		17.0	12.0	14.0
23	363	PM	11.4	41.0	0.0		13.0	11.0	14.0
23	364	Combination	10.9	8.0	0.0		9.0	12.0	3.0
23	365	SSG	6.2	7.0	0.0	10.0	8.0	13.0	9.0
23	366	Combination	5.0	1.0	14.0		13.0	9.0	5.0
23	367	SSG	10.0	13.0	10.0	9.0	6.0	11.0	9.0
23	368	PM	8.0	10.0	12.0		9.0	5.0	10.0
23	369	Combination	6.0	6.0	5.0		6.0	6.0	11.0
23	370	SSG	5.0	6.0	5.0	8.0	2.0	7.0	8.0
23	371	PM	2.0	16.0	10.0		19.0	13.0	18.0
23	372	PM	6.0	10.0	8.0		14.0	12.0	18.0
23	373	SSG	9.0	8.0	9.0	13.0	13.0	14.0	30.0
23	374	SSG	11.0	8.0	8.0	1.0	6.0	2.0	9.0
23	375	Combination	8.0	6.0	10.0		2.0	11.0	11.0
23	376	SSG	9.0	7.0	9.0	6.0	8.0	8.0	6.0
23	377	Combination	10.0	6.0					9.0
23	378	Combination	6.0	4.0	7.0		1.0	5.0	5.0
23	379	PM	8.0	1.0	9.0		10.0	5.0	5.0
23	380	SSG	8.0	8.0	2.0	6.0	8.0	4.0	6.0
23	381	PM	3.0	6.0	8.0		10.0	6.0	9.0
23	382	Combination	1.0	13.0	12.0		9.0	8.0	37.0
23	383	PM	8.0	6.0	6.0		6.0	13.0	11.0
23	384	SSG	12.0	10.0	9.0	10.0	9.0	9.0	5.0
23	385	SSG	7.0	7.0	5.0	14.0	7.0	9.0	8.0
23	386	Combination	9.0	11.0	15.0		19.0	29.0	12.0
23	387	Combination	12.0	10.0	10.0		10.0	9.0	9.0
23	388	PM	4.0	8.0	7.0		9.0	6.0	6.0
23	389	SSG	4.0	5.0	6.0	5.0	5.0	6.0	8.0
23	390	PM	11.0	16.0	12.0		12.0	17.0	11.0
23	391	Combination	11.0	12.0	12.0		12.0	4.0	17.0
23	392	Combination	6.0	4.0	4.0			4.0	5.0
23	393	SSG	8.0	8.0	7.0	8.0	8.0	3.0	17.0

Centre Number	Patient Number	Treatment	Bilirubin (µmol/L)						
			Baseline	Day 7	Day 14	Day 21	EOT	3 Mon FU	6 Mon FU
23	394	PM	5.0	6.0	6.0		2.0	2.0	5.0
23	395	PM	9.0	7.0	6.0		7.0	8.0	6.0
23	396	SSG	10.0	8.0	6.0	12.0	13.0	16.0	16.0
23	397	SSG	9.0	8.0	14.0	13.0	13.0	4.0	16.0
23	398	Combination	5.0	7.0	3.0		7.0	6.0	6.0
23	399	PM	8.0	11.0	8.0		8.0	11.0	7.0
23	400	PM	8.0	11.0	16.0		20.0	13.0	13.0
23	401	Combination	16.0	15.0	13.0		10.0	16.0	18.0
23	402	Combination	23.0	7.0	17.0		16.0	30.0	24.0
23	403	SSG	64.0	34.0	26.0	20.0	17.0	21.0	39.0
23	404	SSG	6.0	7.0	8.0	10.0	9.0		8.0
23	405	PM	21.0	19.0	19.0		15.0	39.0	37.0
34	451	Combination	11.0	8.6	8.6		8.6		8.6
34	452	PM	10.0	9.8	8.6		9.5		
34	453	PM	8.6	11.8	8.6		9.0		
34	454	Combination	10.4	8.6	8.6		10.2		8.6
34	455	PM	8.6	8.6	8.6		8.6		
34	456	SSG	8.6	8.6	8.6	8.6	8.6	8.6	
34	457	Combination	14.1	8.6	8.6		8.6		
34	458	PM	8.6	8.6	8.6		8.6	8.6	
34	459	Combination	8.6	8.6	8.6		8.6	8.6	8.6
34	460	PM	13.8	8.9	9.0		8.6		
34	461	SSG	8.6	11.9	10.2				
34	462	SSG	8.9	8.6	10.2	8.6	8.6		8.6
34	463	Combination	10.5	16.8	8.6		8.6		8.6
34	464	SSG	17.4	11.8	21.4	11.7	9.5		29.4
34	465	SSG	8.6	8.6	8.6	8.6	8.6		8.6
34	466	Combination	8.6	8.6	8.6		8.6		
34	467	PM	10.0	9.8	10.5		8.6	20.2	
34	468	Combination	8.6	8.6	9.5		8.6		8.6
34	469	SSG	8.6	8.6	8.6	8.6	8.6		9.4
34	470	Combination	8.6	8.6	8.6		8.6		8.6
34	471	PM	8.6	8.7	8.6		8.6		
34	472	SSG	8.6	8.6	8.6	8.6	8.6		8.6
34	473	PM	24.5	10.1	8.6		8.6		
34	474	Combination	12.5	8.6	8.6		8.6		8.6
34	475	PM	8.6	8.6	8.6		8.6		
34	476	PM	16.6	16.9	8.6		10.7	8.7	
34	477	SSG	24.3	13.9	18.0	27.4	12.9		28.7
34	478	Combination	17.6	8.6	8.6		8.6		8.6
34	479	SSG	8.6	8.6	8.6	8.6	8.6		8.6
34	480	SSG	9.6	8.6	8.6	9.0	8.6		8.6
34	481	Combination	8.6	15.8	8.6		8.6		8.6

Centre Number	Patient Number	Treatment	Bilirubin (µmol/L) Baseline	Day 7	Day 14	Day 21	EOT	3 Mon FU	6 Mon FU
34	482	Combination	8.6	8.6	8.6		8.6		8.6
34	483	PM	10.4	8.6	8.6		8.6		10.2
34	484	PM	12.9	15.2					
34	485	Combination	23.6	27.5	30.8		8.6		40.0
34	486	Combination	8.6	8.6	8.6		8.6		8.6
34	487	SSG	8.6	8.6	8.6	8.6	8.6		8.6
34	488	SSG	8.6	8.6	8.6	8.6	8.6	8.6	8.6
34	489	SSG	8.6	8.6	8.6	8.6	8.6		12.1
34	490	PM	8.6	8.6	8.6		8.7		10.0
34	491	PM	12.3	16.8	11.5		11.9		
34	492	PM	11.3	8.6	10.5				14.3
34	493	SSG	10.7	8.6	8.6	8.6	8.6		
34	494	Combination	11.9	8.6	8.6		8.6		30.8
34	495	SSG	8.6	8.6	8.6	8.6	8.6		8.6
34	496	SSG	8.6	8.6	8.6	8.6	8.6		8.6
34	497	PM	22.9	8.6	14.2		16.0	14.6	
34	498	SSG	8.6	8.6	8.6	8.6	8.6		10.9
34	499	Combination	8.6	8.6	8.6		8.6		8.6
34	500	PM	8.6	8.6	8.6		8.6		8.6
34	501	Combination	8.6	8.6	8.6		8.6		8.6
34	502	Combination	8.6	8.6	8.6				8.6
34	503	PM	8.6	8.6	9.9		9.7		
34	504	Combination	8.6	8.6	8.6		8.6		
34	505	SSG	10.2	8.6	8.6	8.9	8.6		9.9
34	506	SSG	12.7	8.6	8.6	8.6			8.6
34	507	SSG	8.6	8.6	8.6	8.6	8.6		8.6
34	508	PM	14.9	8.6	8.6		11.4		
34	509	PM	19.7	10.4	9.2		8.6		
34	510	Combination	11.9	8.6	8.6		8.6		8.6
34	511	Combination	8.6	8.6	8.6		8.6		8.6
34	512	PM	11.4	8.6	8.6				
34	513	Combination	8.6	8.6	8.6		8.6	8.6	8.6
34	514	SSG	8.6	8.6	8.6	526.0	8.6		8.6
34	515	SSG	9.5	8.6	8.6	8.6	8.6	8.6	
34	516	PM	8.6	8.6	8.6		8.6		8.6
34	517	PM	9.8	8.6	8.6		8.6		
34	518	SSG	8.6	8.6	12.9	11.2	9.4		10.6
34	519	PM	8.6	8.6	8.6		8.6	8.6	
34	520	SSG	8.6	8.6	8.6	8.6	9.0		8.6
34	521	PM	8.6	8.6	8.6		8.6		
34	522	Combination	35.1	21.6	22.6		20.9		
34	523	SSG	8.6	8.6	8.6	8.6	8.6		8.6
34	524	Combination	8.6	8.6	8.6		8.6		8.6

Centre Number	Patient Number	Treatment	Bilirubin (µmol/L) Baseline	Day 7	Day 14	Day 21	EOT	3 Mon FU	6 Mon FU
34	525	Combination	8.6	8.6	8.6				8.6
34	526	SSG	8.6	8.6	8.6	8.6	9.9		8.6
34	527	Combination	8.6	8.6	8.6		8.6		8.8
34	528	PM	11.9	9.2	10.9		8.6		
34	529	Combination	10.7	8.6	8.6		8.6		8.6
34	530	Combination	16.8	12.5	8.6		8.6		14.6
34	531	SSG	8.6	8.6	8.6	8.6	8.6		
34	532	Combination	8.6	8.6	8.6		8.6		8.6
34	533	SSG	8.6	8.6	8.6	8.6	8.6		8.6
34	534	PM	8.6	8.6	10.8		9.4		
34	535	PM	8.6	10.2	8.6		8.6	8.9	
34	536	PM	8.6	8.6	11.2		9.6		10.8
34	537	SSG	8.6	12.6	8.6	8.6	8.6		13.2
34	538	PM	8.6	8.6	8.6		8.6		
34	539	Combination	8.6	8.6	8.6		8.6		15.2
34	540	SSG	9.4	16.0	9.7	8.6	11.6		8.6
35	646	PM	15.4	10.3	6.8		5.1		6.8
35	647	PM	12.0	17.1	13.7		5.1	12.0	5.1
35	648	Combination	15.4	13.7			6.8	10.3	10.3
35	649	Combination	17.1	13.7			13.7	12.0	8.6
35	650	SSG	13.7	12.0	8.6	13.7	10.3	8.6	8.6
35	651	SSG	17.1	17.1	6.8	5.1	6.8	12.0	8.6
35	652	PM	10.3	6.8	6.8		13.7		3.4
35	653	Combination	15.4	15.4	13.7		13.7	12.0	8.6
35	654	SSG	6.8	8.6	12.0	17.1	17.1	6.8	6.8
35	655	Combination	12.0	13.7	12.0		10.3	12.0	8.6
35	656	SSG	13.7	12.0	15.4	17.1	8.6	6.8	8.6
35	657	PM	8.6	12.0	13.7		17.1	13.7	10.3
35	658	Combination	17.1	13.7	17.1		15.4	13.7	10.3
35	659	SSG	13.7	12.0	13.7	15.4	15.4	12.0	10.3
35	660	PM	15.4	12.0	10.3		13.7	10.3	8.6
35	661	Combination	3.4	13.7	10.3		10.3	8.6	10.3
35	662	PM	5.1	6.8	8.6		6.8	8.6	5.1
35	663	SSG	5.1	5.1	8.6	5.1	17.1	10.3	6.8
35	664	Combination	6.8	8.6					6.8
35	665	SSG	15.4	17.1	12.0	12.0	8.6	6.8	8.6
35	666	SSG	10.3	10.3	5.1	6.8	10.3	6.8	10.3
35	667	Combination	8.6	10.3	8.6		3.4	6.8	8.6
35	668	PM	17.1	13.7	10.3		8.6		6.8
35	669	PM	13.7	12.0	8.6		12.0		
35	670	SSG	12.0	12.0	8.6	8.6	13.7	8.6	6.8
35	671	PM	12.0	5.1	8.6		10.3		6.8
35	672	Combination	15.4	5.1	13.7		15.4		10.3

Centre Number	Patient Number	Treatment	Bilirubin (µmol/L)						
			Baseline	Day 7	Day 14	Day 21	EOT	3 Mon FU	6 Mon FU
35	673	SSG	8.6	8.6	13.7	15.4	12.0	10.3	8.6
35	674	Combination	12.0	8.6	12.0		10.3	12.0	12.0
35	675	PM	8.6	8.6	5.1		10.3		6.8
35	676	PM	12.0	8.6	8.6		10.3	8.6	8.6
35	677	SSG	5.1	13.7					
35	678	Combination	6.8	15.4	12.0		13.7	12.0	10.3
35	679	SSG	6.8	10.3	6.8	10.3	6.8	8.6	6.8
35	680	Combination	15.4	10.3	8.6		6.8	8.6	6.8
35	681	Combination	5.1	10.3	6.8		6.8	8.6	10.3
35	682	PM	5.1	10.3	8.6		12.0		3.4
35	683	Combination	5.1	10.3	10.3		8.6		8.6
35	684	SSG	5.1	13.7	10.3	6.8	8.6	8.6	10.3
35	685	PM	6.8	17.1	15.4		6.8	8.6	8.6
35	686	PM	13.7	13.7	12.0		8.6	6.8	8.6
35	687	SSG	12.0	15.4	10.3	13.7	10.3	6.8	8.6
35	688	PM	12.0	6.8	5.1		10.3	8.6	6.8
35	689	Combination	17.1	15.4	12.0		5.1	10.3	8.6
35	690	SSG	12.0	10.3	6.8	10.3	12.0	8.6	8.6

Centre Number	Patient Number	Treatment	Creatinine (µmol/L) Baseline	Day 7	Day 14	Day 21	EOT	3 Mon FU	6 Mon FU
11	1	PM	88.4	97.2	106.1		106.1	106.1	88.4
11	2	Combination	88.4	106.1	88.4		97.2	97.2	97.2
11	3	Combination	88.4	79.6	79.6		79.6	79.6	70.7
11	4	PM	88.4	88.4	79.6		79.6	79.6	70.7
11	5	PM	53.0	70.7	70.7		70.7	61.9	70.7
11	6	Combination	61.9	61.9	70.7		79.6	61.9	70.7
11	7	PM	70.7	88.4	88.4		88.4	70.7	88.4
11	8	SSG	106.1	106.1	88.4	88.4	97.2	97.2	88.4
11	9	SSG	97.2	97.2	97.2	97.2	97.2	79.6	88.4
11	10	SSG	61.9	79.6	88.4	79.6	88.4	79.6	70.7
11	11	Combination	70.7	79.6	70.7		70.7	61.9	61.9
11	12	Combination	88.4	97.2	114.9		88.4	97.2	97.2
11	13	SSG	97.2	79.6	79.6	88.4	79.6	70.7	79.6
11	14	SSG	70.7	61.9	70.7	61.9	70.7	70.7	70.7
11	15	PM	106.1	106.1				88.4	88.4
11	16	Combination	88.4	79.6	88.4		88.4	88.4	79.6
11	17	Combination	106.1	88.4	79.6		106.1	88.4	88.4
11	18	SSG	97.2	97.2	79.6	97.2	88.4	97.2	88.4
11	19	Combination	97.2	97.2	88.4		114.9	97.2	106.1
11	20	SSG	88.4	70.7	61.9	70.7	70.7	70.7	79.6
11	21	SSG	88.4	88.4	88.4	88.4	106.1	79.6	79.6
11	22	SSG	70.7	70.7	70.7	70.7	61.9	61.9	79.6
11	23	PM	79.6	70.7	70.7		70.7	70.7	88.4
11	24	PM	61.9	70.7	70.7		53.0	70.7	70.7
11	25	SSG	70.7	70.7	61.9	79.6	61.9	61.9	88.4
11	26	Combination	79.6	79.6	79.6		79.6	61.9	79.6
11	27	PM	97.2	88.4	88.4		79.6	79.6	
11	28	Combination	70.7	79.6	79.6		70.7	70.7	79.6
11	29	PM	88.4	97.2	88.4		88.4	97.2	97.2
11	30	PM	70.7	70.7	61.9		61.9	61.9	70.7
11	31	SSG	97.2	79.6	79.6	70.7	79.6	79.6	114.9
11	32	Combination	79.6	79.6	88.4		79.6	79.6	88.4
11	33	SSG	88.4	88.4	88.4	114.9	70.7	88.4	97.2
11	34	PM	97.2	97.2	97.2		97.2	97.2	106.1
11	35	SSG	70.7	70.7	70.7	61.9	61.9	79.6	79.6
11	36	SSG	88.4	79.6	79.6	79.6	79.6	79.6	97.2
11	37	Combination	106.1	114.9	106.1		88.4	106.1	114.9
11	38	PM	79.6	70.7	79.6		88.4	106.1	141.4
11	39	PM	70.7	70.7	61.9		70.7	88.4	70.7
11	40	PM	79.6	79.6	70.7			70.7	79.6
11	41	Combination	61.9	61.9	61.9		61.9	61.9	70.7
11	42	Combination	88.4	79.6	88.4		79.6	79.6	88.4
11	43	SSG	70.7	70.7	70.7	88.4	79.6	79.6	97.2
11	44	PM	70.7	79.6	70.7		88.4	88.4	88.4
11	45	Combination	79.6	88.4	70.7		79.6	79.6	88.4
11	46	SSG	79.6	79.6	79.6	79.6	70.7	72.5	76.0
11	47	SSG	106.1	106.1	79.6	97.2	88.4		
11	48	Combination	79.6	79.6	97.2		88.4		86.6
11	49	SSG	88.4	79.6	88.4	79.6	70.7	70.7	70.7
11	50	PM	132.6	114.9	106.1		97.2	94.6	66.3
11	51	Combination	88.4	79.6	70.7		79.6	89.3	84.9
11	52	PM	97.2	79.6	79.6		79.6	88.4	79.6
11	53	SSG	132.6	106.1	97.2	97.2	123.8	97.2	97.2
11	54	PM	106.1	106.1	114.9		97.2	106.1	106.1

Centre Number	Patient Number	Treatment	Creatinine (µmol/L) Baseline	Day 7	Day 14	Day 21	EOT	3 Mon FU	6 Mon FU
11	55	PM	88.4	88.4	88.4		97.2	97.2	88.4
11	56	SSG	97.2	97.2	88.4	88.4	88.4	106.1	88.4
11	57	Combination	97.2	106.1	97.2		106.1	103.4	89.7
11	58	Combination	79.6	79.6	79.6		79.6	79.6	78.7
11	59	PM	97.2	88.4	88.4		106.1	85.7	69.0
11	60	Combination	53.0	106.1	97.2		106.1	106.1	88.4
11	61	PM	44.2	132.6	132.6		132.6	114.9	106.1
11	62	SSG	70.7	70.7	88.4	79.6	70.7	61.9	64.5
11	63	SSG	114.9	114.9	123.8	123.8	114.9	106.1	97.2
11	64	SSG	123.8	97.2	106.1	106.1	106.1	114.9	97.2
11	65	PM	88.4	88.4	88.4		79.6		
11	66	Combination	97.2	106.1	106.1		106.1	79.6	65.4
11	67	PM	114.9	88.4	88.4		79.6	79.6	83.1
11	68	SSG	97.2	70.7	70.7	79.6	76.0	70.7	78.7
11	69	Combination	97.2	88.4	79.6		79.6	79.6	85.7
11	70	Combination	123.8	106.1	97.2		97.2	94.6	85.7
11	71	Combination	106.1	97.2	97.2		92.8	97.2	127.3
11	72	SSG	106.1	123.8	106.1			97.2	96.4
11	73	PM	106.1	79.6	101.7		106.1	85.7	79.6
11	74	PM	123.8	123.8	114.9		106.1	97.2	87.5
11	75	Combination	114.9	97.2	106.1		97.2	106.1	155.6
11	76	PM	97.2	93.7	88.4		88.4	70.7	89.3
11	77	PM	106.1	95.5	97.2		85.7	70.7	80.4
11	78	SSG	114.9	96.4	106.1	97.2	97.2	88.4	88.4
11	79	PM	88.4	91.1	88.4		94.6	70.7	70.7
11	80	PM	88.4	94.6	79.6		87.5	79.6	72.5
11	81	Combination	114.9	114.9	114.9		114.9	141.4	98.1
11	82	SSG	88.4	78.7	88.4	80.4	89.3	70.7	66.3
11	83	SSG	88.4	88.4	84.9	75.1	97.2	97.2	84.9
11	84	SSG	106.1	84.9	88.4	87.5	70.7	79.6	72.5
11	85	SSG	114.9	114.9	108.7	97.2	97.2	97.2	
11	86	Combination	88.4	88.4	77.8		70.7	79.6	75.1
11	87	Combination	123.8	96.4	112.3		91.1	88.4	87.5
11	88	PM	106.1	112.3	102.5		85.7	88.4	91.1
11	89	Combination	106.1	106.1	106.1		114.9	97.2	93.7
11	90	Combination	79.6	79.6	79.6		79.6		
11	91	PM	106.1	97.2	106.1		102.5	102.5	137.9
11	92	Combination	84.9	61.9	70.7		69.0	61.9	73.4
11	93	SSG	88.4	78.7	83.1	87.5	91.9	86.6	95.5
11	94	SSG	61.9	70.7	53.9	64.5	70.7	64.5	60.1
11	95	SSG	70.7	44.2	61.9	63.6	61.9	64.5	65.4
11	96	Combination	61.9	83.1	67.2		71.6	72.5	70.7
11	97	Combination	70.7	93.7	84.0		82.2	86.6	77.8
11	98	PM	63.6	81.3	81.3		87.5	81.3	79.6
11	99	SSG	79.6	97.2	93.7	94.6	90.2	77.8	82.2
11	100	PM	88.4	103.4	105.2		109.6		
11	101	PM	97.2	78.7	94.6		91.1	91.9	96.4
11	102	Combination	88.4	73.4	89.3		71.6	91.9	79.6
11	103	Combination	104.3	90.2	79.6		91.9	93.7	79.6
11	104	PM	68.1	88.4	82.2		83.1	70.7	71.6
11	105	SSG	83.1	79.6	85.7	67.2	77.8	85.7	70.7
11	106	Combination	116.7	107.0	61.9		89.3	88.4	98.1
11	107	PM	87.5	87.5	61.9		96.4	89.3	95.5
11	108	PM	97.2	86.6	86.6		92.8	95.5	87.5

Centre Number	Patient Number	Treatment	Creatinine (µmol/L) Baseline	Day 7	Day 14	Day 21	EOT	3 Mon FU	6 Mon FU
11	109	SSG	88.4	80.4	75.1	83.1	90.2	77.8	88.4
11	110	PM	67.2	63.6	65.4		64.5	73.4	57.5
11	111	Combination	87.5	79.6	89.3		75.1	80.4	83.1
11	112	Combination	100.8	106.1	99.9		110.5	96.4	79.6
11	113	SSG	99.9	84.9	88.4	87.5	90.2	78.7	
11	114	SSG	70.7	79.6	87.5	70.7	79.6	90.2	80.4
11	115	Combination	79.6	82.2	74.3		76.9	77.8	72.5
11	116	SSG	88.4	104.3	107.0	102.5	116.7	94.6	94.6
11	117	PM	93.7	86.6	88.4		84.0	82.2	
11	118	PM	88.4	109.6	88.4		88.4	85.7	72.5
11	119	SSG	91.9	77.8	82.2	81.3	91.1	89.3	79.6
11	120	Combination	99.0	87.5	86.6		84.9	92.8	88.4
11	121	Combination	70.7	61.9	71.6		68.1	71.6	70.7
11	122	SSG	97.2	79.6	93.7	84.9	92.8		
11	123	SSG	103.4	89.3	87.5	88.4	107.0	85.7	79.6
11	124	SSG	107.0	92.8	91.9	88.4	94.6		83.1
11	125	SSG	88.4	93.7	97.2	99.9	81.3	76.0	79.6
11	126	Combination	112.3	118.5	114.9		108.7		95.5
11	127	PM	99.0	96.4	101.7		95.5	69.0	61.9
11	128	Combination	132.6	97.2	147.6		173.3	70.7	95.5
11	129	PM	106.1	114.9	101.7		98.1	98.1	88.4
11	130	Combination	79.6	84.9	83.1		84.9	76.9	76.0
11	131	PM	68.1	84.9	49.5		70.7	73.4	69.0
11	132	SSG	110.5	97.2	105.2	101.7	87.5	81.3	83.1
11	133	Combination	112.3	92.8	90.2		61.9	89.3	76.0
11	134	PM	88.4	91.1	82.2		91.1	88.4	93.7
11	135	PM	88.4	123.8	106.1		105.2	107.9	98.1
12	241	Combination	75.8	70.0	61.6		72.0		70.7
12	242	SSG	81.8	47.0	34.2	34.3	63.0	43.6	54.8
12	243	PM	78.9	69.7	47.9		51.5	55.9	44.0
12	244	SSG	51.3	51.2	58.6	38.6	76.0	56.2	60.7
12	245	PM	65.0	62.0	96.3		69.6	110.2	
12	246	PM	6.7	96.0	105.0		101.0	88.0	
12	247	PM	38.5	62.0	68.6		58.4	55.7	36.2
12	248	PM	58.4	95.0	69.0		72.3	70.7	41.5
12	249	Combination	86.1	56.0	87.3		90.7	52.2	47.7
12	250	SSG	75.3	50.5	61.1	44.0	84.0	46.9	52.8
12	251	Combination	72.2	80.0	72.9		47.9	61.0	59.2
12	252	SSG	56.9	62.6	91.0	50.1	51.6	78.3	52.8
12	253	SSG	47.7	41.5	46.2	63.1	44.2	87.1	44.0
12	254	Combination	46.4	47.4	40.8		58.4	56.6	44.2
12	255	Combination	55.4	65.5	44.0		58.5	81.3	53.0
12	256	Combination	48.5	73.0	49.6		89.0	50.4	44.2
12	257	SSG	46.8	85.0	62.0	60.1	74.3	53.9	50.2
12	258	SSG	49.0	40.7	44.2	44.0	44.2		44.0
12	259	PM	73.4	70.7	61.2		69.8	53.0	74.8
12	260	PM	61.2	88.4	106.1		44.2		44.9
12	261	Combination	68.7	114.9	90.2		50.4	141.4	50.4
12	262	PM	51.6	97.2	84.0		41.5	44.0	50.2
12	263	SSG	61.9	53.0	60.1	44.2	52.2	52.2	60.0
12	264	Combination	70.7	43.3	60.1		53.0	45.7	58.0
12	265	SSG	76.9	60.1	37.1	64.5	341.0		
12	266	PM	46.0	69.0	60.1		105.2	53.0	67.0
12	267	SSG	60.1	61.9	58.3	82.2	77.8	65.1	69.0

Centre Number	Patient Number	Treatment	Creatinine (µmol/L) Baseline	Day 7	Day 14	Day 21	EOT	3 Mon FU	6 Mon FU
12	268	PM	106.1	71.6	72.5		55.7	64.4	79.3
12	269	Combination	90.2	76.0	78.7		79.6	57.0	63.0
12	270	Combination	81.3	61.0	171.5		44.2		63.2
12	271	SSG	64.5	61.9	56.6	67.2	89.3	48.4	64.0
12	272	PM	44.2	42.4	44.2		137.0	44.2	56.0
12	273	SSG	44.2	87.5	46.0	44.2	53.0	44.2	54.0
12	274	SSG	74.3	38.9	40.7	44.2	49.3	44.2	52.0
12	275	Combination	83.1	56.6	44.2		85.7	53.7	62.0
12	276	PM	46.9	21.2	44.2		66.3	44.2	64.0
12	277	Combination	90.2	68.1	51.3		61.0	51.9	60.0
12	278	SSG	104.3	90.2	65.4	76.0	76.9	62.0	
12	279	PM	82.2	61.0	57.5		55.7	88.0	62.0
12	280	SSG	38.9	44.2	44.2	44.2	44.2	52.0	
12	281	Combination	99.9	57.5	44.2		58.3	44.2	
12	282	PM	44.2	49.5	46.0		44.2	58.0	
12	283	PM	70.7	77.8	70.7		67.2	72.0	78.3
12	284	Combination	44.2	44.2	104.0		44.2	97.1	
12	285	Combination	65.4	61.0	61.9		51.3	83.6	
12	286	PM	66.3	47.7	52.0		50.0	85.0	57.0
12	287	SSG	63.5	45.8	52.0	53.4	59.4	63.4	58.2
12	288	Combination	42.0	44.2	45.0		58.8	44.2	44.2
12	289	Combination	62.0	55.7	60.0		48.0	52.0	62.6
12	290	SSG	52.5	51.5	46.2	52.0	61.2		50.2
12	291	SSG	74.3	68.0	55.0	72.0	76.0	51.2	50.0
12	292	PM	103.0	96.0	92.1		61.2		68.0
12	293	SSG	52.7	51.7	54.1	44.2	56.0		44.2
12	294	Combination	50.0	44.2	50.0		88.4	49.0	46.2
12	295	Combination	44.2	45.1	53.0		48.2		40.4
12	296	PM	52.3	47.3	51.8		49.4	56.0	81.6
12	297	SSG	70.3	76.4	81.9				
12	298	PM	56.3	61.2	54.5		56.3	54.0	76.2
12	299	Combination	58.8	62.4	64.9		63.4		101.0
12	300	PM	63.5	64.2	66.3		74.2	54.6	74.2
12	301	SSG	74.2	67.3	69.4	51.3	56.2	66.0	61.2
12	302	SSG	50.2	56.3	53.4	61.3	66.6	56.9	93.0
12	303	PM	63.4	72.3	81.4		69.4	54.2	91.4
12	304	Combination	77.6	79.4	80.4		77.4	59.2	74.2
12	305	PM	63.1	66.4	69.3		67.3	47.1	79.4
12	306	Combination	44.2	63.4	69.8		70.4	53.0	68.0
12	307	Combination	64.3	104.3	94.6		83.4	72.1	135.0
12	308	PM	80.9	76.4	64.3		66.1	71.4	118.0
12	309	PM	95.0	87.4	74.3		76.6	60.4	74.0
12	310	SSG	67.4	70.3	64.6	74.2	59.4	75.7	69.2
12	311	SSG	44.2	54.0	61.0	44.2	46.1	44.0	62.0
12	312	Combination	68.0	54.8	44.8		45.2	74.2	68.0
12	313	PM	61.2	85.8	92.8		104.0	92.3	74.2
12	314	SSG	71.0	69.8	80.0	70.2	68.4	66.4	70.4
12	315	Combination	44.2	48.4	34.9		60.4	61.0	88.4
12	316	SSG	44.2	52.4	56.9			114.5	44.2
12	317	SSG	58.0	54.2	62.4	66.2	64.1		53.9
12	318	Combination	46.4	51.2	52.4		54.3	64.1	78.0
12	319	Combination	46.2	50.3	52.1		60.4	48.0	79.3
12	320	Combination	44.2	48.4	52.0		64.0	81.2	77.0
12	321	Combination	46.0	50.4	51.2		56.4	69.1	56.4

Centre Number	Patient Number	Treatment	Creatinine (µmol/L) Baseline	Day 7	Day 14	Day 21	EOT	3 Mon FU	6 Mon FU
12	322	PM	54.2	61.8	63.4		61.8	53.4	53.0
12	323	SSG	57.8	54.2	60.9	42.9	94.9	41.0	44.2
12	324	SSG	61.4	53.4	59.4	89.2	95.0	46.0	61.9
12	325	PM	61.7	66.9	54.2		50.4	49.1	70.7
12	326	PM	60.1	62.2	52.9		115.0	92.0	70.7
12	327	Combination	62.4	64.1	41.8		63.2	52.0	44.2
12	328	PM	54.1	88.4	56.3		74.6	61.2	53.9
12	329	SSG	81.3	71.7	72.4	37.1	81.1	72.1	60.1
12	330	PM	80.0	58.5	83.0		61.4	54.2	46.0
23	361	Combination	85.0	92.0	128.0		85.0	67.0	61.0
23	362	PM	95.0	128.0	80.0		120.0	72.0	71.0
23	363	PM	96.0	86.0	92.0		102.0	61.0	55.0
23	364	Combination	94.0	92.0	81.0		80.0	81.0	58.0
23	365	SSG	101.0	123.0	170.0	166.0	138.0	114.0	91.0
23	366	Combination	105.0	86.0	86.0		101.0	87.0	58.0
23	367	SSG	112.0	102.0	123.0	117.0	104.0	97.0	83.0
23	368	PM	114.0	106.0	88.0		142.0	102.0	95.0
23	369	Combination	102.0	93.0	92.0		109.0	90.0	80.0
23	370	SSG	156.0	102.0	125.0	88.0	88.0	88.0	68.0
23	371	PM	115.0	108.0	114.0		115.0	122.0	100.0
23	372	PM	130.0	130.0	141.0		127.0	100.0	95.0
23	373	SSG	113.0	110.0	93.0	100.0	68.0	89.0	83.0
23	374	SSG	91.0	123.0	115.0	97.0	98.0	86.0	69.0
23	375	Combination	77.0	105.0	93.0		119.0	77.0	83.0
23	376	SSG	112.0	104.0	101.0	136.0	96.0	88.0	65.0
23	377	Combination	108.0	107.0					66.0
23	378	Combination	133.0	69.0	87.0		81.0	78.0	60.0
23	379	PM	117.0	70.0	88.0		79.0	104.0	71.0
23	380	SSG	87.0	102.0	72.0	68.0	80.0	73.0	51.0
23	381	PM	83.0	86.0	92.0		83.0	89.0	73.0
23	382	Combination	96.0	92.0	98.0		84.0	94.0	96.0
23	383	PM	85.0	96.0	92.0		96.0	93.0	105.0
23	384	SSG	84.0	105.0	77.0	90.0	76.0	65.0	69.0
23	385	SSG	103.0	94.0	95.0	92.0	57.0	61.0	84.0
23	386	Combination	91.0	71.0	78.0		81.0	58.0	75.0
23	387	Combination	90.0	95.0	81.0		77.0	70.0	65.0
23	388	PM	82.0	71.0	61.0		58.0	65.0	99.0
23	389	SSG	85.0	85.0	72.0	68.0	95.0	75.0	2.9
23	390	PM	95.0	83.0	73.0		83.0	61.0	64.0
23	391	Combination	97.0	84.0	82.0		101.0	56.0	66.0
23	392	Combination	66.0	67.0	71.0			73.0	60.0
23	393	SSG	75.0	72.0	71.0	77.0	100.0	64.0	73.0
23	394	PM	86.0	79.0	74.0		68.0	60.0	66.0
23	395	PM	95.0	81.0	86.0		107.0	109.0	96.0
23	396	SSG	89.0	72.0	94.0	117.0	119.0	71.0	86.0
23	397	SSG	70.0	85.0	98.0	96.0	97.0	71.0	81.0
23	398	Combination	78.0	82.0	89.0		90.0	80.0	88.0
23	399	PM	68.0	79.0	83.0		84.0	70.0	75.0
23	400	PM	68.0	71.0	64.0		62.0	68.0	67.0
23	401	Combination	114.0	103.0	106.0		100.0	113.0	83.0
23	402	Combination	110.0	96.0	94.0		100.0	75.0	72.0
23	403	SSG	113.0	99.0	116.0	87.0	93.0	72.0	84.0
23	404	SSG	123.0	121.0	115.0	98.0	112.0		113.0
23	405	PM	94.0	90.0	92.0		111.0	66.0	82.0

Centre Number	Patient Number	Treatment	Creatinine (µmol/L) Baseline	Day 7	Day 14	Day 21	EOT	3 Mon FU	6 Mon FU
34	451	Combination	44.2	44.2	44.2		44.2		44.2
34	452	PM	44.2	44.2	44.2		44.2		
34	453	PM	44.2	44.2	44.2		44.2		
34	454	Combination	44.2	44.2	44.2		44.2		44.2
34	455	PM	44.2	44.2	44.2		44.2		
34	456	SSG	44.2	44.2	44.2	44.2	44.2	44.2	
34	457	Combination	73.7	46.9	44.2		44.2		
34	458	PM	44.2	44.2	44.2		44.2	44.2	
34	459	Combination	46.9	44.2	44.2		44.2	44.2	44.2
34	460	PM	44.2	44.2	44.2		44.2		
34	461	SSG	74.3	82.5	82.6				
34	462	SSG	44.2	44.2	44.2	44.2	44.2		44.2
34	463	Combination	44.2	44.2	44.2		44.2		44.2
34	464	SSG	56.6	65.9	54.0	46.3	50.3		78.5
34	465	SSG	57.2	44.3	44.2	44.2	44.2		52.2
34	466	Combination	44.2	44.2	44.2		44.2		
34	467	PM	45.5	44.2	44.2		44.2	51.2	
34	468	Combination	44.6	44.2	44.2		44.2		44.2
34	469	SSG	44.2	44.2	44.2	44.2	44.2		44.2
34	470	Combination	44.2	44.2	44.2		44.2		44.2
34	471	PM	46.7	44.2	45.8		44.2		
34	472	SSG	44.2	44.2	44.2	44.2	44.2		44.2
34	473	PM	44.2	44.2	44.2		44.2		
34	474	Combination	44.2	44.2	44.2		44.2		44.2
34	475	PM	44.2	44.2	44.2		44.2		
34	476	PM	44.2	44.2	44.2		44.2	44.2	
34	477	SSG	44.2	44.2	44.2	44.2	44.2		44.2
34	478	Combination	44.2	44.2	44.2		44.2		44.2
34	479	SSG	44.2	44.2	44.2	44.2	44.2		44.2
34	480	SSG	44.2	44.2	44.2	44.2	44.2		44.2
34	481	Combination	44.2	44.2	44.2		44.2		44.2
34	482	Combination	71.9	65.6	49.3		48.5		45.5
34	483	PM	53.7	48.7	50.3		44.2		44.2
34	484	PM	44.2	44.2					
34	485	Combination	44.2	44.2	44.2		44.2		44.2
34	486	Combination	44.2	44.2	44.2		44.2		44.2
34	487	SSG	69.7	44.2	44.2	44.2	44.2		44.2
34	488	SSG	44.2	44.2	44.2	44.2	44.2	44.2	44.2
34	489	SSG	44.2	44.2	44.2	44.2	44.2		44.2
34	490	PM	44.2	44.2	44.2		44.2		44.2
34	491	PM	109.6	85.1	86.9		81.8		
34	492	PM	69.8	78.7	76.6		72.0		85.6
34	493	SSG	44.2	44.2	44.2	44.2	44.2		
34	494	Combination	70.9	49.7	47.4		48.7		73.8
34	495	SSG	44.2	44.2	44.2	44.2	44.2		44.2
34	496	SSG	78.9	53.0	49.8	44.2	53.6		57.4
34	497	PM	63.9	50.5	50.2		52.6	64.4	
34	498	SSG	45.7	44.2	44.2	44.2	44.2		44.2
34	499	Combination	44.3	44.2	44.2		44.2		44.2
34	500	PM	44.2	44.2	44.2		44.2		44.2
34	501	Combination	47.1	44.2	44.2		44.2		44.2
34	502	Combination	44.2	44.2	44.2		44.2		44.2
34	503	PM	44.2	45.3	44.2		44.2		
34	504	Combination	44.2	44.2	44.2		44.2		

Centre Number	Patient Number	Treatment	Creatinine (µmol/L)						
			Baseline	Day 7	Day 14	Day 21	EOT	3 Mon FU	6 Mon FU
34	505	SSG	67.4	49.0	45.2	44.2	44.2		65.9
34	506	SSG	44.2	44.2	44.2	44.2	44.2		44.2
34	507	SSG	44.2	44.2	44.2	44.2	44.2		44.2
34	508	PM	44.2	44.2	44.2		44.2		
34	509	PM	64.1	44.4	44.2		44.2		
34	510	Combination	44.2	44.2	44.2		44.2		44.2
34	511	Combination	44.2	44.2	44.2		44.2		44.2
34	512	PM	44.2	44.2	44.2		44.2		
34	513	Combination	44.2	44.2	44.2		44.2	44.2	44.2
34	514	SSG	44.2	44.2	44.2	44.2	44.2		44.2
34	515	SSG	44.2	44.2	44.2	44.2	44.2	44.2	
34	516	PM	44.2	44.2	44.2		44.2		44.2
34	517	PM	53.4	44.2	44.2		44.2		
34	518	SSG	44.2	44.2	44.2	44.2	44.2		44.2
34	519	PM	44.2	44.2	44.2		44.2	44.2	
34	520	SSG	97.2	71.4	44.6	47.6	44.2		79.9
34	521	PM	44.2	44.2	44.2		44.2		
34	522	Combination	61.9	56.9	62.6		56.0		
34	523	SSG	44.2	44.2	44.2	44.2	44.2		44.2
34	524	Combination	44.2	44.2	44.2		44.2		44.2
34	525	Combination	44.2	44.2	44.2		44.2		44.2
34	526	SSG	44.2	44.2	44.2	44.2	44.2		44.2
34	527	Combination	57.2	44.2	44.2		44.2		44.2
34	528	PM	44.2	44.2	44.2		44.2		
34	529	Combination	44.2	44.2	44.2		44.2		44.2
34	530	Combination	84.2	51.4	46.4		44.2		44.2
34	531	SSG	44.2	44.2	44.2	44.2	44.2		
34	532	Combination	44.2	44.2	44.2		44.2		44.2
34	533	SSG	44.2	44.2	44.2	44.2	44.2		44.2
34	534	PM	44.2	44.2	44.2		45.3		
34	535	PM	44.2	44.2	44.2		44.2	44.2	
34	536	PM	44.2	44.2	44.2		44.2		44.2
34	537	SSG	44.2	44.2	44.2	44.2	44.2		44.2
34	538	PM	44.2	44.2	44.2		44.2		
34	539	Combination	44.2	44.2	44.2		44.2		44.2
34	540	SSG	45.2	44.2	44.2	44.2	44.2		44.2
35	646	PM	132.6	88.4	88.4		61.9		53.0
35	647	PM	114.9	132.6	88.4		88.4	70.7	44.2
35	648	Combination	114.9	88.4			70.7	44.2	44.2
35	649	Combination	132.6	88.4			88.4	88.4	61.9
35	650	SSG	70.7	132.6	88.4	88.4	70.7	53.0	44.2
35	651	SSG	114.9	44.2	44.2	44.2	70.7	44.2	35.4
35	652	PM	114.9	88.4	70.7		70.7		35.4
35	653	Combination	114.9	88.4	70.7		61.9	53.0	44.2
35	654	SSG	88.4	88.4	70.7	88.4	106.1	70.7	53.0
35	655	Combination	88.4	44.2	44.2		61.9	44.2	35.4
35	656	SSG	114.9	44.2	61.9	44.2	61.9	61.9	53.0
35	657	PM	88.4	61.9	70.7		70.7	88.4	44.2
35	658	Combination	106.1	70.7	70.7		61.9	44.2	44.2
35	659	SSG	88.4	70.7	70.7	44.2	70.7	53.0	44.2
35	660	PM	88.4	70.7	88.4		70.7	53.0	44.2
35	661	Combination	70.7	70.7	88.4		88.4	70.7	53.0
35	662	PM	88.4	88.4	79.6		97.2	70.7	35.4
35	663	SSG	106.1	88.4	88.4	123.8	106.1	79.6	88.4

Centre Number	Patient Number	Treatment	Creatinine (µmol/L) Baseline	Day 7	Day 14	Day 21	EOT	3 Mon FU	6 Mon FU
35	664	Combination	88.4	309.4					61.9
35	665	SSG	106.1	70.7	106.1	44.2	44.2	61.9	53.0
35	666	SSG	88.4	70.7	26.5	61.9	88.4	79.6	53.0
35	667	Combination	70.7	70.7	88.4		106.1	70.7	61.9
35	668	PM	106.1	106.1	88.4		88.4		53.0
35	669	PM	61.9	53.0	53.0		88.4		
35	670	SSG	61.9	53.0	53.0	44.2	88.4	70.7	53.0
35	671	PM	70.7	70.7	88.4		88.4		53.0
35	672	Combination	106.1	35.4	44.2		53.0		61.9
35	673	SSG	70.7	70.7	53.0	88.4	79.6	70.7	53.0
35	674	Combination	53.0	88.4	70.7		79.6	44.2	53.0
35	675	PM	88.4	70.7	88.4		70.7		44.2
35	676	PM	88.4	53.0	79.6		70.7	61.9	44.2
35	677	SSG	88.4	291.7					
35	678	Combination	70.7	88.4	70.7		70.7	53.0	53.0
35	679	SSG	70.7	53.0	79.6	70.7	88.4	79.6	61.9
35	680	Combination	70.7	88.4	79.6		70.7	53.0	44.2
35	681	Combination	35.4	53.0	61.9		70.7	53.0	44.2
35	682	PM	26.5	53.0	70.7		70.7		35.4
35	683	Combination	26.5	53.0	61.9		70.7		53.0
35	684	SSG	70.7	53.0	70.7	88.4	88.4	53.0	35.4
35	685	PM	70.7	53.0	61.9		88.4	70.7	53.0
35	686	PM	70.7	53.0	70.7		88.4	70.7	53.0
35	687	SSG	35.4	88.4	70.7	61.9	53.0	44.2	53.0
35	688	PM	61.9	70.7	106.1		88.4	61.9	53.0
35	689	Combination	61.9	70.7	53.0		61.9	44.2	44.2
35	690	SSG	61.9	70.7	88.4	88.4	88.4	70.7	61.9

Centre Number	Patient Number	Treatment	Blood Urea Nitrogen (mmol/L) Baseline	Day 7	Day 14	Day 21	EOT	3 Mon FU	6 Mon FU
11	1	PM	12.05		6.13		5.48	5.88	6.21
11	2	Combination	8.76		4.71		4.24	4.31	3.25
11	3	Combination	9.49		5.29		5.29	4.34	2.99
11	4	PM		4.64	4.78		4.75	5.48	4.16
11	5	PM		4.89	3.58		4.42	4.60	4.24
11	6	Combination		4.31	4.49		4.27	4.56	4.89
11	7	PM		5.40	7.12		6.43	6.32	1.86
11	8	SSG		5.11	5.59	5.33	5.22	5.55	4.34
11	9	SSG		5.18	5.40	5.70	5.11	5.22	4.64
11	10	SSG		4.02	5.00	4.24	4.60	4.64	3.87
11	11	Combination	4.09	4.02	3.98		3.72	3.61	4.86
11	12	Combination		5.51	6.32		5.40	6.10	5.37
11	13	SSG	4.86	4.89	4.49	3.87	4.16	3.98	4.02
11	14	SSG	4.42	4.31	4.42	4.34	4.78	3.43	3.80
11	15	PM	5.07	4.71				4.53	4.24
11	16	Combination	4.93	4.71	4.64		4.27	5.18	4.24
11	17	Combination	5.51	5.04	5.33		5.95	5.99	4.05
11	18	SSG	5.29	5.22	5.62	5.51	4.49	2.96	3.36
11	19	Combination	4.82	5.84	6.43		5.95	2.63	4.05
11	20	SSG	4.78	4.93	5.22	4.20	4.16	3.14	3.76
11	21	SSG	4.60	5.07	5.29	4.75	5.73	2.81	3.54
11	22	SSG	4.20	4.27	5.11	4.24	4.08	2.34	3.94
11	23	PM	4.42	4.75	5.15		4.16	3.94	5.77
11	24	PM	4.75	5.00	5.40		4.07	2.45	4.02
11	25	SSG	3.43	3.58	3.87	2.99	3.75	2.77	3.07
11	26	Combination	5.18	5.00	4.53		4.67	4.78	4.49
11	27	PM	5.32	4.34	4.09		5.29	2.56	
11	28	Combination	5.79	4.60	5.18		4.24	5.48	5.11
11	29	PM	4.97	5.91	5.66		5.59	5.70	2.70
11	30	PM	4.86	5.11	4.24		5.15	1.72	3.14
11	31	SSG	5.18	5.04	4.75	5.26	4.27	4.42	3.69
11	32	Combination	6.94	4.02	5.07		4.97	5.33	3.98
11	33	SSG	4.64	3.43	4.16	5.18	5.00	2.74	3.98
11	34	PM	5.51	5.33	5.29		5.26	3.10	4.16
11	35	SSG	3.54	3.83	4.13	4.75	3.98	5.15	3.43
11	36	SSG	6.21	4.16	3.72	4.02	5.15	4.53	5.77
11	37	Combination	4.93	7.12	5.07		5.48	1.68	3.25
11	38	PM	5.66	4.78	5.81		6.13	4.67	6.48
11	39	PM	5.95	4.97	5.29		4.05	6.54	4.71
11	40	PM	5.26	4.34	4.53			3.91	3.72
11	41	Combination	5.40	4.27	0.26		3.10	5.04	5.15
11	42	Combination	5.40	4.31	0.37		3.36	3.94	4.27
11	43	SSG	4.82	3.80	3.50	3.03	1.52	3.94	4.24
11	44	PM	6.39	5.18	5.22		2.15	6.75	6.39
11	45	Combination	5.33	4.16	3.87		4.49	4.49	5.81
11	46	SSG	4.89	3.14	3.72	4.71	4.64	4.16	6.28
11	47	SSG	4.42	3.40	3.40	5.22	4.60		
11	48	Combination	4.67	4.53	12.30		4.75		4.56
11	49	SSG	5.07	4.56	7.27	4.93	4.09	4.75	6.81
11	50	PM	6.61	6.06	4.86		5.62	3.91	5.04
11	51	Combination	4.49	5.51	4.27		3.94	4.84	4.82
11	52	PM	6.46	6.83	5.51		6.17	6.57	6.35
11	53	SSG	5.33	6.17	4.45	5.15	4.89	4.56	4.42
11	54	PM	6.39	4.93	2.89		5.55	5.59	5.48

Centre Number	Patient Number	Treatment	Blood Urea Nitrogen (mmol/L)						
			Baseline	Day 7	Day 14	Day 21	EOT	3 Mon FU	6 Mon FU
11	55	PM	4.31	2.24	2.80		5.04	5.15	4.60
11	56	SSG	5.29	3.32	4.38	5.18	4.78	4.56	3.51
11	57	Combination	3.16	6.43	5.00		6.17	6.11	5.16
11	58	Combination	2.46	2.36	4.82		4.99	4.71	4.05
11	59	PM	2.64	5.26	5.77		5.22	4.34	4.78
11	60	Combination	3.10	6.79	5.81		5.51	4.78	5.36
11	61	PM	2.70	6.39	6.39		5.95	4.09	5.07
11	62	SSG	4.75	3.87	5.04	4.60	3.80	6.43	5.37
11	63	SSG	6.06	5.44	5.55	4.89	4.20	5.15	4.76
11	64	SSG	5.73	5.45	4.60	5.55	4.42	5.88	5.50
11	65	PM	5.07	6.75	6.02		4.86		
11	66	Combination	5.51	4.78	5.59		5.99	5.33	3.69
11	67	PM	5.00	6.41	5.37		4.56	5.40	5.18
11	68	SSG	6.03	2.99	4.16	3.90	4.05	6.65	4.38
11	69	Combination	5.40	4.53	4.46		5.18	4.34	4.78
11	70	Combination	5.88	5.48	3.98		4.75	6.64	4.97
11	71	Combination	5.07	3.67	4.53		3.91	4.49	4.45
11	72	SSG	4.64		3.91			4.02	4.20
11	73	PM	4.78	3.66	4.06		4.38	4.05	3.82
11	74	PM	6.50	5.16	3.21		4.64	6.68	6.94
11	75	Combination	5.00	3.90	3.50		5.99	5.44	5.40
11	76	PM	4.64	5.48	6.79		6.06	5.91	6.24
11	77	PM	5.22	5.81	7.96		5.99	5.55	5.53
11	78	SSG	6.75	4.89	5.91	3.54	3.80	3.76	4.48
11	79	PM	4.24	4.89	6.43		6.90	4.16	5.16
11	80	PM	5.15	4.80	4.89		4.82	6.63	5.30
11	81	Combination	5.48	5.88	4.16		4.60	5.28	4.21
11	82	SSG	6.28	4.34	4.38	4.94	1.27	3.91	5.58
11	83	SSG	4.31	4.13	4.09	3.72	4.49	4.31	5.13
11	84	SSG	6.72	4.09	3.72	5.45	3.29	5.44	5.26
11	85	SSG	4.42	2.99	3.35	3.25	4.72	3.88	
11	86	Combination	6.28	8.35	3.79		3.37	5.00	4.60
11	87	Combination	7.23	7.17	4.20		4.79	5.00	5.06
11	88	PM	4.27	5.75	4.43		3.90	4.46	4.03
11	89	Combination	5.88	3.68	4.88		3.13	4.01	4.95
11	90	Combination	6.64	3.99	5.81		2.57		
11	91	PM	6.28	5.74	5.88		6.21	5.36	6.39
11	92	Combination	4.45	2.97	4.56		5.55	3.68	5.36
11	93	SSG	4.53	4.80	4.11	4.38	4.38	5.41	5.62
11	94	SSG	4.64	6.54	4.44	4.49	4.53	3.60	3.91
11	95	SSG	6.82	3.80	6.00	6.24	5.50	6.83	7.12
11	96	Combination	4.24	4.83	5.02		5.43	4.57	6.55
11	97	Combination	4.22	3.55	5.57		4.54	4.26	5.77
11	98	PM	5.22	4.44	5.11		6.36	4.40	4.16
11	99	SSG	4.70	3.98	5.17	4.48	4.82	5.78	5.15
11	100	PM	4.40	4.75	5.88		6.90		
11	101	PM	4.79	3.44	5.04		4.41	3.97	4.82
11	102	Combination	4.49	4.77	5.26		4.28	6.88	6.94
11	103	Combination	5.54	4.50	3.90		4.62	7.09	6.64
11	104	PM	5.32	5.77	5.41		5.45	6.05	6.10
11	105	SSG	5.07	4.54	4.50	4.56	5.02	3.75	4.86
11	106	Combination	6.50	5.16	4.89		0.81	5.33	7.19
11	107	PM	6.13	5.68	5.13		5.31	4.59	5.84
11	108	PM	2.72	5.51	5.79		4.67	5.75	6.02

Centre Number	Patient Number	Treatment	Blood Urea Nitrogen (mmol/L) Baseline	Day 7	Day 14	Day 21	EOT	3 Mon FU	6 Mon FU
11	109	SSG	5.76	4.40	4.82	4.56	4.44	4.89	5.07
11	110	PM	5.29	4.42	4.74		4.47	4.48	6.64
11	111	Combination	4.44	2.75	4.27		3.81	6.00	6.75
11	112	Combination	4.25	4.33	3.41		3.73	5.77	7.03
11	113	SSG	4.30	4.01	3.57	3.92	3.65	5.99	
11	114	SSG	4.22	4.19	4.47	4.23	4.17	5.30	5.46
11	115	Combination	7.96	4.29	4.15		4.18	5.47	6.83
11	116	SSG	4.56	3.84	4.21	4.53	4.71	5.51	5.15
11	117	PM	4.87	3.95	4.26		4.41	4.14	
11	118	PM	4.49	4.71	5.98		5.69	5.08	5.11
11	119	SSG	5.52	4.71	4.49	4.99	5.07	5.07	5.55
11	120	Combination	4.61	3.84	4.80		5.03	5.22	4.89
11	121	Combination	3.88	4.45	5.02		5.46	4.89	5.66
11	122	SSG	5.73	4.45	4.47	5.37	4.71		
11	123	SSG	5.84	4.85	5.24	5.16	5.07	5.55	5.27
11	124	SSG	4.66	3.76	3.65	3.68	4.52		14.10
11	125	SSG	4.31	4.13	4.49	5.35	4.88	4.96	5.33
11	126	Combination	4.98	4.85	5.21		5.31		5.95
11	127	PM	5.36	4.64	6.60		6.36	4.86	4.02
11	128	Combination	5.24	4.44	8.00		9.82	4.86	4.68
11	129	PM	5.36	6.13	5.40		5.87	7.41	6.28
11	130	Combination	5.36	4.42	4.65		4.99	5.73	7.78
11	131	PM	5.72	4.66	6.31		5.58	6.50	4.86
11	132	SSG	6.37	5.25	4.66	4.29	4.86	6.86	5.99
11	133	Combination	5.94	5.00	4.93		5.18	5.44	4.97
11	134	PM	4.60	5.77	5.48		5.22	6.47	4.74
11	135	PM	3.80	5.07	5.15		5.48	4.60	4.82
12	241	Combination	11.68	5.48	6.57		4.75		10.95
12	242	SSG	7.67	4.02	5.84	4.38	4.38	12.78	10.22
12	243	PM	11.32	7.30	6.57		10.22	5.11	6.94
12	244	SSG	5.84	3.29	6.94	5.11	5.84	13.03	9.93
12	245	PM	5.48	8.03	6.21		5.48	5.84	
12	246	PM	10.95	11.68	15.33		16.06	12.05	
12	247	PM	6.21	9.49	7.30		13.84	14.60	10.22
12	248	PM	6.21	9.13	12.78		11.94	5.48	9.49
12	249	Combination	12.78	12.92	13.14		9.49	9.49	10.70
12	250	SSG	10.59	9.68	8.65	5.48	10.22	10.37	11.68
12	251	Combination	12.05	5.84	10.22		8.03	8.40	11.83
12	252	SSG	6.94	5.11	4.38	8.76	9.49	11.46	12.41
12	253	SSG	9.60	6.57	4.38	5.84	8.03	11.25	10.22
12	254	Combination	9.75	5.84	6.94		5.11	9.49	7.30
12	255	Combination	11.98	10.81	4.02		5.48	12.05	8.03
12	256	Combination	5.84	9.49	10.95		12.05	12.05	6.94
12	257	SSG	4.38	8.76	7.30	12.41	10.22	11.32	10.95
12	258	SSG	6.21	9.86	6.94	11.68	9.60		10.22
12	259	PM	11.68	11.68	9.86		5.84	9.49	13.14
12	260	PM	11.68	11.68	16.06		10.95		9.49
12	261	Combination	14.97	9.86	9.49		12.05	9.49	6.21
12	262	PM	11.68	11.68	13.36		7.67	9.49	9.49
12	263	SSG	6.94	9.13	10.22	12.96	20.23	11.68	5.84
12	264	Combination	10.95	6.57	12.19		7.30	11.10	10.59
12	265	SSG	9.86	9.13	13.11	15.26	90.18		
12	266	PM	7.67	5.11	10.70		16.03	7.67	6.57
12	267	SSG	10.95	6.57	10.73	11.68	9.64	13.51	5.11

Centre Number	Patient Number	Treatment	Blood Urea Nitrogen (mmol/L)						
			Baseline	Day 7	Day 14	Day 21	EOT	3 Mon FU	6 Mon FU
12	268	PM	8.25	10.04	9.64		8.91	16.43	6.57
12	269	Combination	11.54	7.30	11.03		11.57	8.40	10.95
12	270	Combination	13.73	9.78	9.49		8.76		7.67
12	271	SSG	9.49	10.37	9.78	7.30	10.22	10.22	10.95
12	272	PM	8.25	8.65	10.92		17.52	10.22	8.76
12	273	SSG	10.59	8.84	10.04	8.76	9.49	5.11	9.49
12	274	SSG	11.50	7.30	9.35	9.49	0.20	6.21	9.49
12	275	Combination	10.66	7.67	9.38		14.17	11.68	11.32
12	276	PM	7.48	6.94	8.07		11.68	6.57	11.68
12	277	Combination	12.89	12.05	11.21		10.95	11.68	10.95
12	278	SSG	13.87	15.33	14.93	10.95	10.11	3.65	
12	279	PM	11.83	10.95	17.20		18.99	6.94	8.76
12	280	SSG	7.56	8.11	10.37	12.23	13.03	8.40	
12	281	Combination	10.51	9.86	13.44		13.14	5.84	
12	282	PM	8.76	14.82	10.95		9.93	5.11	
12	283	PM	11.79	13.14	14.60		9.13	6.21	9.02
12	284	Combination	13.18	13.87	7.67		6.21	13.87	
12	285	Combination	12.41	15.33	7.30		8.40	6.21	
12	286	PM	12.27	10.11	12.78		11.87	7.48	12.45
12	287	SSG	8.77	11.57	12.05	8.73	9.20	10.70	11.68
12	288	Combination	10.44	10.00	8.40		8.76	8.29	7.34
12	289	Combination	9.49	10.08	9.86		11.32	7.74	7.34
12	290	SSG	8.54	10.22	10.22	9.38	9.86		9.49
12	291	SSG	9.64	10.59	9.13	10.04	10.59	10.62	8.54
12	292	PM	17.89	12.67	13.69		11.68		10.95
12	293	SSG	8.03	8.76	9.49	10.59	11.32		10.59
12	294	Combination	12.01	8.73	8.76		12.92	8.18	9.57
12	295	Combination	8.32	8.54	8.84		7.30		7.08
12	296	PM	8.58	9.31	10.99		14.02	7.67	8.76
12	297	SSG	10.70	8.84	8.25				
12	298	PM	10.48	9.53	8.98		9.64	9.86	7.74
12	299	Combination	14.86	15.12	13.47		11.46		10.22
12	300	PM	12.34	10.73	9.64		10.70	9.86	8.03
12	301	SSG	11.83	11.39	10.08	11.39	10.66	7.30	8.40
12	302	SSG	14.28	14.68	11.43	11.10	11.94	8.84	8.76
12	303	PM	13.14	11.83	10.70		10.00	8.80	9.86
12	304	Combination	8.18	8.62	9.64		8.87	7.38	21.54
12	305	PM	9.57	7.30	8.43		10.62	9.16	6.94
12	306	Combination	12.49	12.27	11.46		11.06	8.76	11.32
12	307	Combination	12.45	13.80	14.38		12.89	13.40	11.68
12	308	PM	9.82	12.85	13.22		10.66	8.03	21.54
12	309	PM	15.30	15.12	16.87		14.09	11.03	13.51
12	310	SSG	9.97	9.13	10.62	11.06	11.10	9.86	8.03
12	311	SSG	9.75	7.30	8.76	73.39	10.59	7.74	6.57
12	312	Combination	11.03	10.59	9.86		9.60	12.41	11.03
12	313	PM	11.94	11.68	11.83		13.07	10.15	8.76
12	314	SSG	11.32	12.41	10.95	11.32	11.32	9.49	9.86
12	315	Combination	10.88	10.81	11.72		14.38	11.68	8.76
12	316	SSG	12.49	13.91	14.68			8.76	11.35
12	317	SSG	12.27	13.22	11.83	13.22	13.98		10.22
12	318	Combination	9.89	10.66	11.46		12.49	11.32	7.67
12	319	Combination	8.76	10.22	10.95		12.41	9.86	8.40
12	320	Combination	10.66	11.10	12.41		9.86	11.68	13.14
12	321	Combination	14.90	15.12	10.59		10.22	12.41	10.22

Centre Number	Patient Number	Treatment	Blood Urea Nitrogen (mmol/L)						
			Baseline	Day 7	Day 14	Day 21	EOT	3 Mon FU	6 Mon FU
12	322	PM	8.18	9.49	9.86		10.33	8.76	9.13
12	323	SSG	11.32	8.76	13.14	8.76	10.22	10.59	7.67
12	324	SSG	11.68	12.41	13.14	12.41	8.76	12.05	7.30
12	325	PM	7.30	9.49	9.86		10.92	9.89	6.57
12	326	PM	15.33	16.06	11.83		10.22	9.86	10.59
12	327	Combination	12.23	14.31	9.93		6.94	7.67	8.03
12	328	PM	13.51	8.76	9.38		7.30	9.49	8.76
12	329	SSG	8.03	12.38	7.67	11.68	10.73	7.81	13.51
12	330	PM	7.30	10.33	8.76		9.89	8.76	10.22
23	361	Combination	2.80	4.12	5.30		5.04	5.92	3.52
23	362	PM	3.30	4.38	5.00		3.60	4.55	4.43
23	363	PM	4.10	5.72	1.99		6.31	5.29	2.44
23	364	Combination	3.50	3.50	4.40		1.90	3.95	3.06
23	365	SSG	4.60	3.69	2.99	3.50	2.65	3.43	3.21
23	366	Combination	7.40	3.01	2.13		2.34	3.69	2.46
23	367	SSG	4.34	1.90	6.63	4.96	3.92	5.54	3.73
23	368	PM	5.10	5.90	5.60		5.80	7.02	4.06
23	369	Combination	3.65	4.72	2.97		2.50	3.28	1.88
23	370	SSG	3.80	8.25	5.80	2.60	2.00	4.82	3.43
23	371	PM	4.21	7.43	7.16		2.17	4.16	2.49
23	372	PM	3.50	5.00	2.30		7.60	3.51	3.66
23	373	SSG	6.36	1.03	2.76	3.20	2.98	4.58	4.98
23	374	SSG	2.99	2.68	5.20	4.74	2.06	2.91	3.55
23	375	Combination	4.45	4.83	4.63		1.89	5.12	5.00
23	376	SSG	5.57	1.04	2.60	4.27	6.75	2.76	2.72
23	377	Combination	4.37	2.77					4.38
23	378	Combination	3.79	1.75	3.73		2.52	5.79	4.82
23	379	PM	3.46	2.72	2.20		4.46	4.19	4.01
23	380	SSG	2.45	2.80	2.50	3.66	4.72	2.31	5.42
23	381	PM	2.03	2.27	4.91		4.41	1.98	2.19
23	382	Combination	3.66	2.20	4.19		3.49	3.76	3.66
23	383	PM	5.95	5.16	6.15		5.16	4.41	2.26
23	384	SSG	3.87	4.54	3.96	3.36	5.08	2.88	2.35
23	385	SSG	5.45	4.32	4.85	5.85	5.27	4.87	2.76
23	386	Combination	2.89	2.60	3.73		3.14	5.51	3.16
23	387	Combination	3.39	3.38	2.39		4.48	3.27	2.33
23	388	PM	1.99	2.37	1.66		4.67	3.52	9.87
23	389	SSG	2.60	4.60	2.86	5.44	5.34	3.43	6.03
23	390	PM	3.05	3.89	3.38		5.09	1.84	2.45
23	391	Combination	3.33	5.68	4.55		2.36	3.57	2.25
23	392	Combination	2.32	3.59	3.59			1.90	5.25
23	393	SSG	3.99	2.81	5.27	2.88	1.61	3.06	2.63
23	394	PM	2.98	2.72	2.76		5.47	5.63	3.02
23	395	PM	3.61	3.20	1.99		3.29	4.57	2.93
23	396	SSG	2.23	4.87	2.68	4.96	3.52	3.41	2.05
23	397	SSG	2.66	3.50	3.71	4.73	4.17	3.81	3.48
23	398	Combination	2.40	3.67	4.65		4.65	2.97	2.80
23	399	PM	2.79	3.25	5.45		4.27	5.25	3.70
23	400	PM	3.69	2.58	4.87		6.00	4.55	3.64
23	401	Combination	7.40	3.84	6.22		1.88	5.49	3.53
23	402	Combination	7.68	3.50	4.28		2.22	4.49	4.20
23	403	SSG	4.87	3.92	3.28	4.94	3.20	3.80	2.90
23	404	SSG	6.66	5.19	7.47	6.14	3.00		4.23
23	405	PM	4.65	6.19	5.11		4.66	3.28	3.96

Centre Number	Patient Number	Treatment	Blood Urea Nitrogen (mmol/L) Baseline	Day 7	Day 14	Day 21	EOT	3 Mon FU	6 Mon FU
34	451	Combination							
34	452	PM	9.53	10.00	9.78		7.34		
34	453	PM	8.29	9.86	8.21				
34	454	Combination		7.38					12.30
34	455	PM	8.73		10.62				
34	456	SSG							
34	457	Combination							
34	458	PM							
34	459	Combination	7.45						
34	460	PM	7.89	7.48					
34	461	SSG	14.17	15.37	20.26				
34	462	SSG	12.27	7.41					8.29
34	463	Combination		7.89					
34	464	SSG							8.00
34	465	SSG	9.16						
34	466	Combination	9.27						
34	467	PM	7.63						
34	468	Combination							
34	469	SSG							
34	470	Combination							
34	471	PM	8.14	9.89					
34	472	SSG	8.32			7.89			
34	473	PM	9.97						
34	474	Combination			8.84				
34	475	PM		7.48					
34	476	PM			7.45				
34	477	SSG							
34	478	Combination	9.53						
34	479	SSG		9.78	8.80				
34	480	SSG							
34	481	Combination							7.34
34	482	Combination							
34	483	PM							
34	484	PM		14.06					
34	485	Combination							
34	486	Combination							
34	487	SSG	12.71	7.96	7.41				
34	488	SSG							
34	489	SSG							8.25
34	490	PM							
34	491	PM	12.82	8.94			8.25		
34	492	PM	8.11						
34	493	SSG	8.07						
34	494	Combination							
34	495	SSG	7.96						
34	496	SSG							
34	497	PM	13.07		7.38				
34	498	SSG							7.45
34	499	Combination							8.32
34	500	PM							8.69
34	501	Combination	7.74						
34	502	Combination							
34	503	PM	8.43	8.40					
34	504	Combination							

Centre Number	Patient Number	Treatment	Blood Urea Nitrogen (mmol/L)						
			Baseline	Day 7	Day 14	Day 21	EOT	3 Mon FU	6 Mon FU
34	505	SSG	12.27						
34	506	SSG							7.56
34	507	SSG							
34	508	PM							
34	509	PM							
34	510	Combination							
34	511	Combination			7.56				
34	512	PM							
34	513	Combination							
34	514	SSG							
34	515	SSG							
34	516	PM							
34	517	PM							
34	518	SSG							8.87
34	519	PM							
34	520	SSG	7.81						8.69
34	521	PM					9.71		
34	522	Combination	7.38		8.40		8.18		
34	523	SSG	8.40						
34	524	Combination							9.02
34	525	Combination							7.41
34	526	SSG	8.07						
34	527	Combination							
34	528	PM							
34	529	Combination							
34	530	Combination	17.60						
34	531	SSG							
34	532	Combination							
34	533	SSG							7.67
34	534	PM		8.94	12.23				
34	535	PM							
34	536	PM							10.19
34	537	SSG							
34	538	PM							
34	539	Combination							9.78
34	540	SSG							
35	646	PM	6.10	3.83	9.38		4.86		8.76
35	647	PM	10.30	9.13	6.68		12.52	9.24	6.57
35	648	Combination	7.19	8.00			4.97	8.76	7.30
35	649	Combination	4.67	5.07			5.48	10.84	8.07
35	650	SSG	3.83	6.86	5.48	8.76	9.24	7.52	7.30
35	651	SSG	4.27	3.43	3.65	6.83	7.78	10.00	8.76
35	652	PM	5.11	5.59	6.46		13.40		7.30
35	653	Combination	4.53	4.56	4.67		5.33	7.34	6.57
35	654	SSG	4.02	3.83	6.64	7.78	9.86	9.24	10.15
35	655	Combination	5.04	3.98	6.13		7.48	8.84	6.57
35	656	SSG	14.09	8.58	6.21	7.27	9.35	7.67	6.94
35	657	PM	3.72	7.30	10.33		8.29	10.84	7.67
35	658	Combination	6.21	5.48	9.13		9.86	11.68	8.76
35	659	SSG	4.34	4.38	18.26	11.68	11.65	9.20	7.38
35	660	PM	7.41	3.69	8.76		11.39	9.64	7.30
35	661	Combination	5.18	10.95	10.22		10.37	9.13	13.36
35	662	PM	10.22	8.76	9.13		10.22	9.13	6.75
35	663	SSG	13.14	8.76	7.08	7.48	10.37	9.20	6.90

Centre Number	Patient Number	Treatment	Blood Urea Nitrogen (mmol/L) Baseline	Day 7	Day 14	Day 21	EOT	3 Mon FU	6 Mon FU
35	664	Combination	12.05	37.24					6.43
35	665	SSG	10.95	9.24	11.87	10.70	9.75	8.14	7.67
35	666	SSG	10.30	9.75	6.06	8.29	6.79	7.30	9.57
35	667	Combination	8.76	9.86	7.63		7.05	7.30	8.03
35	668	PM	9.75	10.00	9.78		10.66		7.96
35	669	PM	10.66	10.59	11.46		6.10		
35	670	SSG	7.27	10.59	6.10	5.44	6.54	7.59	7.30
35	671	PM	9.89	9.68	12.45		10.62		7.59
35	672	Combination	15.12	9.24	10.48		10.59		7.45
35	673	SSG	13.58	7.05	6.72	7.63	7.23	7.30	6.79
35	674	Combination	6.54	5.70	7.05		8.11	7.52	8.47
35	675	PM	12.74	7.12	11.10		8.29		6.64
35	676	PM	9.35	7.30	7.48		8.32	7.01	8.76
35	677	SSG	14.97	70.10					
35	678	Combination	10.66	5.07	6.21		5.59	6.64	7.30
35	679	SSG	13.22	8.47	7.27	9.46	11.68	8.03	6.94
35	680	Combination	7.78	7.63	7.45		7.12	6.64	7.45
35	681	Combination	13.87	6.79	5.99		6.10	6.79	7.30
35	682	PM	14.68	14.86	14.53		10.55		6.79
35	683	Combination	17.34	6.79	6.57		6.61		7.30
35	684	SSG	6.61	8.76	5.07	5.59	6.06	7.01	7.30
35	685	PM	9.13	11.10	13.69		12.67	9.64	8.76
35	686	PM	8.14	8.84	10.15		9.53	7.67	8.76
35	687	SSG	14.46	11.43	11.68	8.94	11.32	9.13	7.67
35	688	PM	8.18	9.13	9.13		8.58	7.30	9.20
35	689	Combination	12.85	12.16	11.10		11.90	7.30	6.75
35	690	SSG	12.27	11.17	9.64	10.48	11.68	9.49	8.03

Appendix 58: Listing of globulin per patient

Centre Number	Patient Number	Treatment	Globulin (mg/dL) Baseline	Day 7	Day 14	Day 21	EOT	3 Mon FU	6 Mon FU
11	N/A		not done						
12	N/A	Combination	not done						
23	361	Combination	51.6	82	67		43	33	20
23	362	PM	68.3	80	71		80	47	40
23	363	PM	56.5	61	76		68	36	25
23	364	Combination	19.9	59	53		55	54	29
23	365	SSG	67.1	113	91	87	68	31	33
23	366	Combination	88	71	77		79	31	30
23	367	SSG	69	92	56	104	36	31	25
23	368	PM	87	49	94		41	39	22
23	369	Combination	75	75	125		78	35	31
23	370	SSG	68	72	49	21	71	49	34
23	371	PM	85	74	48		44	92	73
23	372	PM	83	81	46		96	35	48
23	373	SSG	36	36	41	25	65	46	52
23	374	SSG	60	50	93	57	40	21	33
23	375	Combination	78	42	52		66	48	21
23	376	SSG	103	26	53	78	66	39	80
23	377	Combination	65	91					26
23	378	Combination	104	59	56		31	52	34
23	379	PM	82	88	68		62	81	51
23	380	SSG	68	53	62	60	66	50	26
23	381	PM	67	61	40		60	46	37
23	382	Combination	71	40	69		67	51	22
23	383	PM	48	86	85		86	35	51
23	384	SSG	84	92	84	90	78	71	71
23	385	SSG	85	93	101	42	84	40	51
23	386	Combination	71	80	61		77	32	59
23	387	Combination	88	78	78		87	57	26
23	388	PM	58	27	40		34	49	43
23	389	SSG	107	139	91	74	63	31	26
23	390	PM	87	121	95		86	41	33
23	391	Combination	89	78	85		78	47	62
23	392	Combination	43	55	78			51	22
23	393	SSG	55	44	63	65	43	40	73
23	394	PM	64	70	73		66	23	82
23	395	PM	76	65	77		75	69	13
23	396	SSG	77	80	70	66	65	39	26
23	397	SSG	49	73	65	47	42	42	22
23	398	Combination	76	78	91		62	65	67
23	399	PM	81	74	94		63	51	32
23	400	PM	39	54	40		67	42	40
23	401	Combination	46	58	68		56	71	63
23	402	Combination	63	82	58		77	27	50
23	403	SSG	77	77	102	90	20	53	51
23	404	SSG	62	75	72	100	40		49
23	405	PM	87	83	105		87	48	44
34	N/A		Not done						
35			Not done						
35	679	SSG					3.4		

Centre Number	Patient Number	Treatment	Albumin (mg/dl) Baseline	Day 7	Day 14	Day 21	EOT	3 Mon FU	6 Mon FU
11			Not done						
12			Not done						
23	361	Combination	40.8	27	26		41	37	40
23	362	PM	33.7	19	29		28	40	36
23	363	PM	40.3	28	28		31	34	37
23	364	Combination	44.6	26	39		33	15	36
23	365	SSG	38.7	30	29	30	30	41	46
23	366	Combination	14	16	18		33	35	34
23	367	SSG	15	22	26	20	24	39	35
23	368	PM	19	24	26		25	41	37
23	369	Combination	18	18	25		23	45	35
23	370	SSG	13	24	19	35	29	27	29
23	371	PM	32	28	48		39	21	34
23	372	PM	33	25	31		30	47	46
23	373	SSG	19	19	37	32	25	21	26
23	374	SSG	14	24	42	30	42	39	34
23	375	Combination	19	33	16		25	43	31
23	376	SSG	31	16	32	32	34	34	31
23	377	Combination	13	12					42
23	378	Combination	27	15	25		34	24	39
23	379	PM	20	20	27		31	34	40
23	380	SSG	20	24	29	30	30	27	37
23	381	PM	32	19	26		29	22	32
23	382	Combination	21	24	24		23	30	37
23	383	PM	25	29	13		29	35	39
23	384	SSG	20	21	19	27	29	34	34
23	385	SSG	19	23	20	19	25	37	37
23	386	Combination	21	24	35		18	36	27
23	387	Combination	19	35	27		23	20	38
23	388	PM	21	43	31		39	43	36
23	389	SSG	15	20	25	38	35	44	43
23	390	PM	14	19	24		28	32	78
23	391	Combination	20	34	25		28	29	28
23	392	Combination	19	20	29			24	41
23	393	SSG	23	32	40	33	41	35	32
23	394	PM	21	23	23		35	31	27
23	395	PM	24	25	23		31	28	44
23	396	SSG	17	26	24	30	31	38	45
23	397	SSG	17	24	34	36	29	33	41
23	398	Combination	18	21	29		31	28	22
23	399	PM	22	26	27		35	28	28
23	400	PM	24	18	42		28	34	34
23	401	Combination	29	23	39		36	21	21
23	402	Combination	24	26	33		36	39	32
23	403	SSG	20	11	22	16	73	27	34
23	404	SSG	22	22	38	33	40		31
23	405	PM	17	30	18		28	32	33
34	N/A		Not done						
35	646	PM	22	29	30		43		34
35	647	PM	27	25	29		36	43	30
35	648	Combination	29	33			30	41	34

35	649	Combination	23	23			29	33	30
35	650	SSG	35	37	36	50	42	38	30
35	651	SSG	33	35	36	41	43	37	30
35	652	PM	33	30	30		42		41
35	653	Combination	25	27	30		30	28	30
35	654	SSG	25	28	31	54	43	41	36
35	655	Combination	24	28	29		29	36	27
35	656	SSG	26	23	28	33	38	31	29
35	657	PM	33	30	41		38	42	30
35	658	Combination	26	28	40		40	39	30
35	659	SSG	30	28	45	42	39	28	30
35	660	PM	32	30	42		42	36	29
35	661	Combination	30	24			37	41	33
35	662	PM	32		31		36	41	32
35	663	SSG	29	24	22	38	32	28	32
35	664	Combination	36	35					32
35	665	SSG	37	42	29	42	33	31	29
35	666	SSG	27	25	37	42	48	32	33
35	667	Combination	36	38	41		43	40	34
35	668	PM	29	36	41		40		31
35	669	PM	32	31	38		33		
35	670	SSG	34	31	39	40	46	35	32
35	671	PM	22	32	31		34		32
35	672	Combination	22	30	34		36		35
35	673	SSG	38	36	41	42	38	34	32
35	674	Combination	32	30	35		37	32	38
35	675	PM	34	30	33		41		36
35	676	PM	34	31	34		35	34	34
35	677	SSG	29	31					
35	678	Combination	24	27	32		39	30	33
35	679	SSG	30	31	33	42		30	28
35	680	Combination	23	28	34		42	36	32
35	681	Combination	33	37	38		39	30	33
35	682	PM	34	35	37		41		38
35	683	Combination	35	32	38		37		34
35	684	SSG	38	40	35	34	36	32	34
35	685	PM	39	39	32		43	36	32
35	686	PM	37	33	43		37	32	28
35	687	SSG	36	35	37	39	32	29	31
35	688	PM	41	41	43		42	36	32
35	689	Combination	38	27	30		38	30	32
35	690	SSG	3.8	32	39	41	37	30	26

Appendix 60: Listing of Total protein per patient

Centre Number	Patient Number	Treatment	Total Protein (g/L)						
			Baseline	Day 7	Day 14	Day 21	EOT	3 Mon FU	6 Mon FU
11			Not done						
12	241	Combination	68	76	83		86		72
12	242	SSG	65	96	76	76	92	82	84
12	243	PM	60	80	85		87	70	70
12	244	SSG	74	56	67	62	78	70	70
12	245	PM	82	60	95		100	90	
12	246	PM	100	74			70	80	
12	247	PM	80		72		70	86	70
12	248	PM	86					80	60
12	249	Combination	69	70	72		80	70	80
12	250	SSG	79	70	70	80	70	70	80
12	251	Combination	100	90	80		80	100	60
12	252	SSG	59	70	90	100	70	70	80
12	253	SSG	68	80	110	120	70	70	70
12	254	Combination	58	80	90		70	100	80
12	255	Combination	80	65	80		110	70	90
12	256	Combination	90	70	90		80	70	60
12	257	SSG	90	80	100	80	110	70	70
12	258	SSG	80	64	62	80	72		80
12	259	PM	84	68	90			70	80
12	260	PM	110	100	74		80		70
12	261	Combination	80	90	85		82	70	70
12	262	PM		70	70		60	70	70
12	263	SSG	60	70	88		78	80	70
12	264	Combination	70		80		70		60
12	265	SSG	80		100	100			
12	266	PM	92		75		68	60	70
12	267	SSG	84		80	82	78	70	
12	268	PM	60	60	90		80	70	60
12	269	Combination	60	70	90		90	70	70
12	270	Combination		80	88		86		70
12	271	SSG	70		60	80	70	80	
12	272	PM	80	70	80			70	60
12	273	SSG	80	80	80	80	90	80	90
12	274	SSG	70	70	70	80	70	70	60
12	275	Combination	90	90	60		90	80	80
12	276	PM	70	70	80		70	70	70
12	277	Combination	80	80	80		90	70	70
12	278	SSG	70	70	70	80	80	73	
12	279	PM	70	75			80	80	80
12	280	SSG	50	70	80	80	70	68	
12	281	Combination	70	80	80		90	90	
12	282	PM	90	80	110		90	70	
12	283	PM	70	80	80		80		
12	284	Combination	70	80	60		70	70	
12	285	Combination	90	90	70		80	70	

Centre Number	Patient Number	Treatment	Total Protein (g/L) Baseline	Day 7	Day 14	Day 21	EOT	3 Mon FU	6 Mon FU
12									
12	287	SSG							
12	288	Combination							
12	289	Combination							
12	290	SSG							
12	291	SSG							
12	292	PM							
12	293	SSG							
12	294	Combination							
12	295	Combination							
12	296	PM							
12	297	SSG							
12	298	PM							
12	299	Combination							
12	300	PM							
12	301	SSG							
12	302	SSG							
12	303	PM							
12	304	Combination							
12	305	PM							
12	306	Combination							
12	307	Combination							
12	308	PM							
12	309	PM							
12	310	SSG							
12	311	SSG							
12	312	Combination							
12	313	PM							
12	314	SSG							
12	315	Combination							80
12	316	SSG							
12	317	SSG							
12	318	Combination							
12	319	Combination							
12	320	Combination							
12	321	Combination							
12	322	PM							
12	323	SSG							
12	324	SSG							
12	325	PM							
12	326	PM							
12	327	Combination							
12	328	PM							
12	329	SSG							
12	330	PM							
23			Not done						
34			Not done						

Centre Number	Patient Number	Treatment	Total Protein (g/L)					
			Baseline	Day 7	Day 14	Day 21	EOT	3 Mon FU	6 Mon FU
35	646	PM	65	57	63		76		62
35	647	PM	71	69	76		95	72	66
35	648	Combination	75	76			76	80	62
35	649	Combination	68	87			90	77	62
35	650	SSG	77	76	69	75	80	72	58
35	651	SSG	80	62	69	70	77	72	59
35	652	PM	70	77	73		80		68
35	653	Combination	77	73	80		76	67	60
35	654	SSG	61		76	76	78	66	80
35	655	Combination	61	63	80		78	81	54
35	656	SSG	63	63	62	73	80	68	62
35	657	PM	80	76	69		77	74	62
35	658	Combination	72	90	76		72	61	54
35	659	SSG	75	69	73	77	74	61	58
35	660	PM	59	69	67		74	72	60
35	661	Combination	84	80	86		84	76	67
35	662	PM	75	73	70		75	76	66
35	663	SSG	75	73	70	72	70	57	48
35	664	Combination	77	75					58
35	665	SSG	59	80	74	82	72	68	58
35	666	SSG	75	72	75	69	71	64	47
35	667	Combination	75	77	77		76	71	68
35	668	PM	75	78	77		72		47
35	669	PM	77	72	77		68		
35	670	SSG	77	72	75	68	73	68	64
35	671	PM	72	80	75		72		58
35	672	Combination	74	66	68		70		62
35	673	SSG	74	68	77	75	68	62	59
35	674	Combination	77	71	70		69	61	66
35	675	PM	75	51	64		73		66
35	676	PM	76	64	77		73	68	70
35	677	SSG	68	72					
35	678	Combination	49	51	64		67	65	68
35	679	SSG	51	66	76		73	64	60
35	680	Combination	63	61	68		67	64	58
35	681	Combination	70	66	67		68	72	64
35	682	PM	76	74	65		77		66
35	683	Combination	78	77	70		72		68
35	684	SSG	68		71	76	80	70	60
35	685	PM	74	68	71		80	72	66
35	686	PM	71	76	80		79	68	60
35	687	SSG	51	76	78	69	64	58	64
35	688	PM	66	74	69		77	67	60
35	689	Combination	69	68	64		73	58	61
35	690	SSG	69	67	73	77	66	62	60

Centre Number	Patient Number	Treatment	Dipstick Urinalysis - Urinary Protein					
			Baseline	Day 7	Day 14	Day 21	EOT	3Mon FU	6Mon FU
11	1	PM	positive	positive	negative		negative	negative	negative
11	2	Combination	negative	negative	negative		negative	negative	negative
11	3	Combination	negative	negative	negative		negative	negative	negative
11	4	PM	negative	negative	negative		negative	negative	negative
11	5	PM	negative	negative	negative		negative	negative	negative
11	6	Combination	negative	negative	negative		negative	negative	negative
11	7	PM	negative	negative	negative		negative	negative	negative
11	8	SSG	negative	negative	negative	negative	negative	negative	negative
11	9	SSG	negative	negative	negative	negative	positive	negative	negative
11	10	SSG	negative	negative	negative	negative	negative	negative	negative
11	11	Combination	negative	negative	negative		negative	negative	negative
11	12	Combination	negative	negative	negative		negative	negative	negative
11	13	SSG	negative	positive	negative	positive	positive	negative	negative
11	14	SSG	negative	negative	negative	negative	negative	negative	negative
11	15	PM	negative	negative				negative	negative
11	16	Combination	negative	negative	negative		negative	negative	negative
11	17	Combination	negative	negative	negative		negative	negative	negative
11	18	SSG	negative	negative	negative	positive	negative	negative	negative
11	19	Combination	negative	negative	negative		negative	negative	negative
11	20	SSG	negative	negative	negative	negative	negative	negative	negative
11	21	SSG	negative	negative	positive	negative	negative	negative	negative
11	22	SSG	negative	negative	negative	negative	negative	negative	negative
11	23	PM	negative	negative	negative		negative	negative	negative
11	24	PM	negative	negative	negative		negative	negative	negative
11	25	SSG	negative	positive	negative	negative	positive	negative	negative
11	26	Combination	negative	negative	negative		negative	negative	negative
11	27	PM	negative	negative	negative		negative	negative	
11	28	Combination	negative	negative	negative		negative	negative	negative
11	29	PM	negative	negative	negative		negative	negative	negative
11	30	PM	negative	negative	negative		negative	negative	negative
11	31	SSG	negative	negative	negative	negative	negative	negative	negative
11	32	Combination	negative	negative	negative		negative	negative	negative
11	33	SSG	negative	negative	negative	negative	negative	negative	negative
11	34	PM	not done	negative	negative		negative	negative	negative
11	35	SSG	negative	negative	negative	negative	negative	negative	negative
11	36	SSG	negative	negative	negative	negative	negative	negative	negative
11	37	Combination	negative	negative	negative		negative	negative	negative
11	38	PM	negative	negative	negative		negative	negative	negative
11	39	PM	negative	negative	negative		negative	negative	negative
11	40	PM	negative	negative	negative			negative	negative
11	41	Combination	negative	negative	negative		negative	negative	negative
11	42	Combination	negative	negative	negative		negative	negative	negative
11	43	SSG	negative	negative	negative	negative	negative	negative	negative
11	44	PM	negative	negative	negative		negative	negative	negative
11	45	Combination	negative	negative	negative		negative	negative	negative

Centre Number	Patient Number	Treatment	Dipstick Urinalysis - Urinary Protein						
			Baseline	Day 7	Day 14	Day 21	EOT	3Mon FU	6Mon FU
11	46	SSG	negative	negative	negative	negative	negative	negative	negative
11	47	SSG	negative	negative	negative	negative	negative		
11	48	Combination	negative	negative	negative		negative		negative
11	49	SSG	negative	negative	negative	negative	negative	negative	negative
11	50	PM	negative	negative	negative		negative	negative	negative
11	51	Combination	negative	negative	negative		negative	negative	negative
11	52	PM	negative	negative	negative		negative	negative	negative
11	53	SSG	negative	negative	negative	negative	negative	negative	negative
11	54	PM	negative	negative	negative		negative	negative	negative
11	55	PM	negative	negative	negative		negative	negative	negative
11	56	SSG	negative	negative	negative	negative	negative	positive	negative
11	57	Combination	negative	negative	negative		negative	negative	negative
11	58	Combination	negative	negative	negative		negative	negative	negative
11	59	PM	negative	negative	negative		negative	negative	negative
11	60	Combination	negative	negative	negative		negative	negative	negative
11	61	PM	negative	negative	negative		negative	negative	positive
11	62	SSG	negative	negative	negative	negative	negative	negative	negative
11	63	SSG	negative	negative	negative	negative	negative	negative	negative
11	64	SSG	negative	negative	negative	negative	negative	negative	negative
11	65	PM	negative	negative	negative		negative		
11	66	Combination	negative	negative	negative		negative	negative	negative
11	67	PM	negative	negative	negative		negative	negative	negative
11	68	SSG	negative	negative	negative	negative	negative	negative	negative
11	69	Combination	negative	negative	negative		negative	negative	negative
11	70	Combination	negative	negative	negative		negative	negative	negative
11	71	Combination	negative	negative	negative		negative	negative	positive
11	72	SSG	negative	negative	negative			negative	negative
11	73	PM	negative	negative	negative		negative	negative	negative
11	74	PM	negative	negative	negative		negative	negative	positive
11	75	Combination	negative	negative	negative		negative	negative	negative
11	76	PM	negative	negative	negative		negative	negative	negative
11	77	PM	negative	negative	negative		negative	negative	negative
11	78	SSG	negative	negative	negative	negative	negative	negative	negative
11	79	PM	negative	negative	negative		negative	negative	negative
11	80	PM	negative	negative	negative		negative	negative	negative
11	81	Combination	negative	negative	negative		negative	negative	negative
11	82	SSG	negative	negative	negative	negative	negative	negative	negative
11	83	SSG	negative	negative	negative	negative	negative	negative	negative
11	84	SSG	positive	negative	negative	negative	positive	negative	negative
11	85	SSG	positive	positive	negative	negative	negative	negative	
11	86	Combination	positive	negative	negative		negative	negative	negative
11	87	Combination	positive	negative	negative		negative	negative	negative
11	88	PM	negative	negative	negative		negative	negative	negative
11	89	Combination	negative	negative	negative		negative	negative	negative
11	90	Combination	negative	negative	negative		negative		

Centre Number	Patient Number	Treatment	Dipstick Urinalysis - Urinary Protein Baseline	Day 7	Day 14	Day 21	EOT	3Mon FU	6Mon FU
11	91	PM	positive	negative	positive		negative	positive	positive
11	92	Combination	negative	negative	negative		negative	negative	negative
11	93	SSG	negative	negative	negative	negative	negative	negative	negative
11	94	SSG	negative	negative	negative	negative	negative	negative	negative
11	95	SSG	negative	negative	negative	negative	negative	negative	negative
11	96	Combination	positive	negative	negative		negative	negative	negative
11	97	Combination	negative	positive	negative		negative	negative	negative
11	98	PM	negative	negative	positive		positive	negative	negative
11	99	SSG	negative	negative	positive	negative	negative	negative	negative
11	100	PM	negative	negative	negative		positive		
11	101	PM	positive	negative	negative		negative	negative	negative
11	102	Combination	positive	negative	negative		negative	negative	negative
11	103	Combination	positive	negative	negative		negative	negative	negative
11	104	PM	negative	negative	negative		negative	negative	negative
11	105	SSG	positive	negative	negative	negative	negative	negative	negative
11	106	Combination	positive	negative	negative		negative	negative	negative
11	107	PM	positive	negative	negative		negative	negative	negative
11	108	PM	negative	negative	negative		negative	negative	negative
11	109	SSG	negative	negative	negative	negative	negative	negative	negative
11	110	PM	positive	negative	negative		negative	negative	negative
11	111	Combination	negative	negative	negative		negative	negative	negative
11	112	Combination	negative	positive	negative		negative	negative	negative
11	113	SSG	negative	negative	negative	negative	negative	negative	
11	114	SSG	negative	negative	negative	negative	negative	negative	negative
11	115	Combination	positive	negative	negative		negative	negative	negative
11	116	SSG	positive	negative	negative	negative	negative	negative	negative
11	117	PM	negative	positive	negative		negative	negative	
11	118	PM	negative	negative	negative		negative	negative	negative
11	119	SSG	positive	negative	negative	negative	negative	negative	negative
11	120	Combination	positive	negative	negative		negative	negative	negative
11	121	Combination	negative	negative	negative		negative	negative	negative
11	122	SSG	positive	negative	negative	negative	negative		
11	123	SSG	negative	negative	negative	negative	negative	negative	negative
11	124	SSG	positive	negative	negative	negative	negative		negative
11	125	SSG	positive	positive	negative	negative	negative	negative	negative
11	126	Combination	negative	negative	negative		negative		negative
11	127	PM	positive	positive	negative		negative	negative	negative
11	128	Combination	negative	positive	positive		positive	negative	negative
11	129	PM	positive	positive	negative		negative	negative	negative
11	130	Combination	negative	negative	negative		negative	negative	negative
11	131	PM	positive	negative	negative		negative	negative	negative
11	132	SSG	positive	negative	negative	negative	negative	negative	negative
11	133	Combination	negative	negative	negative		negative	negative	negative
11	134	PM	positive	positive	negative		negative	negative	negative
11	135	PM	negative	negative	negative		negative	negative	negative

Appendix 61: Listing of urinary protein per patient

Centre Number	Patient Number	Treatment	Dipstick Urinalysis - Urinary Protein						
			Baseline	Day 7	Day 14	Day 21	EOT	3Mon FU	6Mon FU
12	241	Combination	negative	positive	positive		positive		positive
12	242	SSG	negative	negative	negative	not done	negative	negative	negative
12	243	PM	positive	negative	negative		negative	negative	negative
12	244	SSG	negative	negative	negative	negative	negative	negative	negative
12	245	PM	negative	negative	positive		negative	positive	
12	246	PM	negative	negative	negative		negative	negative	
12	247	PM	negative	negative	negative		negative	negative	positive
12	248	PM	negative	negative	negative		negative	negative	negative
12	249	Combination	negative	negative	negative		positive	negative	negative
12	250	SSG	negative	negative	negative	negative	negative	positive	negative
12	251	Combination	positive	positive	positive		positive	negative	negative
12	252	SSG	negative	negative	negative	negative	negative	positive	negative
12	253	SSG	positive	negative	positive	negative	negative	negative	negative
12	254	Combination	positive	positive	negative		negative	negative	negative
12	255	Combination	positive	negative	negative		negative	negative	positive
12	256	Combination	negative	negative	positive		positive	negative	negative
12	257	SSG	positive	negative	negative	negative	negative	negative	negative
12	258	SSG	negative	negative	not done	negative	not done		negative
12	259	PM	not done	not done	negative		not done	negative	negative
12	260	PM	not done	positive	positive		positive		positive
12	261	Combination	negative	negative	negative		negative	negative	negative
12	262	PM	negative	negative	negative		negative	positive	negative
12	263	SSG	positive	negative	negative	positive	positive	negative	positive
12	264	Combination	negative	negative	negative		positive	negative	negative
12	265	SSG	negative	negative	positive	positive			
12	266	PM	negative	negative	negative		negative	negative	negative
12	267	SSG	negative	negative	negative	negative	negative	positive	negative
12	268	PM	negative	negative	negative		negative	negative	negative
12	269	Combination	negative	negative	negative		negative	negative	negative
12	270	Combination	positive	negative	negative		negative		negative
12	271	SSG	positive	negative	negative	negative	negative	negative	negative
12	272	PM	positive	negative	negative		positive	negative	negative
12	273	SSG	negative	negative	negative	negative	negative	negative	negative
12	274	SSG	negative	negative	negative	negative	negative	negative	negative
12	275	Combination	negative	negative	positive		positive	positive	negative
12	276	PM	negative	negative	negative		negative	negative	negative
12	277	Combination	positive	positive	negative		positive	negative	negative
12	278	SSG	positive	positive	negative	positive	positive	negative	
12	279	PM	negative	positive	negative		positive	negative	negative
12	280	SSG	negative	positive	negative	positive	negative	negative	negative
12	281	Combination	positive	negative	negative		positive	negative	negative
12	282	PM	negative	negative	positive		positive	negative	negative
12	283	PM	positive	positive	negative		negative	negative	negative
12	284	Combination	not done	negative	negative		negative	negative	negative
12	285	Combination	positive	positive	positive		negative	negative	negative

16.4 CASE REPORT FORMS

16.4.1 CRFs for deaths, serious adverse events and withdrawals for AE

16.5 INDIVIDUAL PATIENT DATA LISTINGS (US ARCHIVAL LISTINGS)

Available upon request.§
